# Supplementary material for: HIVprotI: an integrated web based platform for prediction and design of HIV proteins inhibitors
Source: J Cheminform. 2018 Mar 9;10:12. doi: 10.1186/s13321-018-0266-y (PMC5845081; doi:10.1186/s13321-018-0266-y)
Supplement: Supplementary file 2 — Additional file 2. Source code of HIVProtI web server. [file 13321_2018_266_MOESM2_ESM.zip › HIVprotI_Source-code/hivprotI/weka-3-6-6/doc/index-all.html]

 
 
 
 
 
 
Index
 

 

 

 
function windowTitle()
{
    if (location.href.indexOf('is-external=true') == -1) {
        parent.document.title="Index";
    }
}
 
 
 

 

 
 


 
   
  
 
 
 
   
 
   
          Overview     
        Package   
        Class   
          Tree     
          Deprecated     
       Index    
          Help           Weka's home     
   
 
 
  
 
 
 

 
  
 PREV 
 NEXT  
  
    FRAMES     
   NO FRAMES     
  
    All Classes  ');
  }
  //-->
 
 
    All Classes  
 


  
 
 
  
 

 A   B   C   D   E   F   G   H   I   J   K   L   M   N   O   P   Q   R   S   T   U   V   W   X   Y   Z   
    
 A  
 
   abortExperiment()   - 
Method in class weka.experiment. RemoteExperiment 
 Set the abort flag
   ABS   - 
Static variable in interface weka.core.mathematicalexpression. sym 
  
   ABS   - 
Static variable in interface weka.filters.unsupervised.instance.subsetbyexpression. sym 
  
   AbstractAssociator   - Class in  weka.associations  Abstract scheme for learning associations.   AbstractAssociator()   - 
Constructor for class weka.associations. AbstractAssociator 
  
   AbstractClusterer   - Class in  weka.clusterers  Abstract clusterer.   AbstractClusterer()   - 
Constructor for class weka.clusterers. AbstractClusterer 
  
   AbstractDataSink   - Class in  weka.gui.beans  Abstract class for objects that store instances to some destination.   AbstractDataSink()   - 
Constructor for class weka.gui.beans. AbstractDataSink 
  
   AbstractDataSinkBeanInfo   - Class in  weka.gui.beans  Bean info class for the AbstractDataSink   AbstractDataSinkBeanInfo()   - 
Constructor for class weka.gui.beans. AbstractDataSinkBeanInfo 
  
   AbstractDataSource   - Class in  weka.gui.beans  Abstract class for objects that can provide instances from some source   AbstractDataSource()   - 
Constructor for class weka.gui.beans. AbstractDataSource 
 Creates a new  AbstractDataSource  instance.
   AbstractDataSourceBeanInfo   - Class in  weka.gui.beans  Bean info class for AbstractDataSource.   AbstractDataSourceBeanInfo()   - 
Constructor for class weka.gui.beans. AbstractDataSourceBeanInfo 
  
   AbstractDensityBasedClusterer   - Class in  weka.clusterers  Abstract clustering model that produces (for each test instance)
 an estimate of the membership in each cluster 
 (ie.   AbstractDensityBasedClusterer()   - 
Constructor for class weka.clusterers. AbstractDensityBasedClusterer 
  
   AbstractEvaluator   - Class in  weka.gui.beans  Abstract class for objects that can provide some kind of evaluation for
 classifier, clusterers etc.   AbstractEvaluator()   - 
Constructor for class weka.gui.beans. AbstractEvaluator 
 Constructor
   AbstractFileLoader   - Class in  weka.core.converters  Abstract superclass for all file loaders.   AbstractFileLoader()   - 
Constructor for class weka.core.converters. AbstractFileLoader 
  
   AbstractFileSaver   - Class in  weka.core.converters  Abstract class for Savers that save to a file

 Valid options are:

 -i input arff file  
 The input filw in arff format.   AbstractFileSaver()   - 
Constructor for class weka.core.converters. AbstractFileSaver 
  
   AbstractLoader   - Class in  weka.core.converters  Abstract class gives default implementation of setSource 
 methods.   AbstractLoader()   - 
Constructor for class weka.core.converters. AbstractLoader 
  
   AbstractSaver   - Class in  weka.core.converters  Abstract class for Saver   AbstractSaver()   - 
Constructor for class weka.core.converters. AbstractSaver 
  
   AbstractStringDistanceFunction   - Class in  weka.core  Represents the abstract ancestor for string-based distance functions, like
 EditDistance.   AbstractStringDistanceFunction()   - 
Constructor for class weka.core. AbstractStringDistanceFunction 
 Constructor that doesn't set the data
   AbstractStringDistanceFunction(Instances)   - 
Constructor for class weka.core. AbstractStringDistanceFunction 
 Constructor that sets the data
   AbstractTestSetProducer   - Class in  weka.gui.beans  Abstract class for TestSetProducers that contains default
 implementations of add/remove listener methods and defualt
 visual representation.   AbstractTestSetProducer()   - 
Constructor for class weka.gui.beans. AbstractTestSetProducer 
 Creates a new  AbstractTestSetProducer  instance.
   AbstractTestSetProducerBeanInfo   - Class in  weka.gui.beans  BeanInfo class for AbstractTestSetProducer   AbstractTestSetProducerBeanInfo()   - 
Constructor for class weka.gui.beans. AbstractTestSetProducerBeanInfo 
  
   AbstractTimeSeries   - Class in  weka.filters.unsupervised.attribute  An abstract instance filter that assumes instances form time-series data and
 performs some merging of attribute values in the current instance with 
 attribute attribute values of some previous (or future) instance.   AbstractTimeSeries()   - 
Constructor for class weka.filters.unsupervised.attribute. AbstractTimeSeries 
  
   AbstractTrainAndTestSetProducer   - Class in  weka.gui.beans  Abstract base class for TrainAndTestSetProducers that contains default
 implementations of add/remove listener methods and defualt
 visual representation.   AbstractTrainAndTestSetProducer()   - 
Constructor for class weka.gui.beans. AbstractTrainAndTestSetProducer 
 Creates a new  AbstractTrainAndTestSetProducer  instance.
   AbstractTrainAndTestSetProducerBeanInfo   - Class in  weka.gui.beans  Bean info class for AbstractTrainAndTestSetProducers   AbstractTrainAndTestSetProducerBeanInfo()   - 
Constructor for class weka.gui.beans. AbstractTrainAndTestSetProducerBeanInfo 
  
   AbstractTrainingSetProducer   - Class in  weka.gui.beans  Abstract class for TrainingSetProducers that contains default
 implementations of add/remove listener methods and default
 visual representation   AbstractTrainingSetProducer()   - 
Constructor for class weka.gui.beans. AbstractTrainingSetProducer 
 Creates a new  AbstractTrainingSetProducer  instance.
   AbstractTrainingSetProducerBeanInfo   - Class in  weka.gui.beans  BeanInfo class for AbstractTrainingSetProducer   AbstractTrainingSetProducerBeanInfo()   - 
Constructor for class weka.gui.beans. AbstractTrainingSetProducerBeanInfo 
  
   accept(File)   - 
Method in class weka.clusterers.forOPTICSAndDBScan.OPTICS_GUI. SERFileFilter 
 Whether the given file is accepted by this filter.
   accept(File)   - 
Method in class weka.gui. ExtensionFileFilter 
 Returns true if the supplied file should be accepted (i.e.: if it
 has the required extension or is a directory).
   accept(File, String)   - 
Method in class weka.gui. ExtensionFileFilter 
 Returns true if the file in the given directory with the given name
 should be accepted.
   ACCEPT   - 
Static variable in class weka.gui.treevisualizer. TreeDisplayEvent 
 States that the user has accepted the tree.
   acceptClassifier(BatchClassifierEvent)   - 
Method in interface weka.gui.beans. BatchClassifierListener 
 Accept a BatchClassifierEvent
   acceptClassifier(BatchClassifierEvent)   - 
Method in class weka.gui.beans. ClassifierPerformanceEvaluator 
 Accept a classifier to be evaluated
   acceptClassifier(IncrementalClassifierEvent)   - 
Method in class weka.gui.beans. IncrementalClassifierEvaluator 
 Accepts and processes a classifier encapsulated in an incremental
 classifier event
   acceptClassifier(IncrementalClassifierEvent)   - 
Method in interface weka.gui.beans. IncrementalClassifierListener 
 Accept the event
   acceptClassifier(IncrementalClassifierEvent)   - 
Method in class weka.gui.beans. PredictionAppender 
 Accept and process an incremental classifier event
   acceptClassifier(BatchClassifierEvent)   - 
Method in class weka.gui.beans. PredictionAppender 
 Accept and process a batch classifier event
   acceptClassifier(IncrementalClassifierEvent)   - 
Method in class weka.gui.beans. SerializedModelSaver 
 Accept and save an incrementally trained classifier.
   acceptClassifier(BatchClassifierEvent)   - 
Method in class weka.gui.beans. SerializedModelSaver 
 Accept and save a batch trained classifier.
   acceptClusterer(BatchClustererEvent)   - 
Method in interface weka.gui.beans. BatchClustererListener 
 Accept a BatchClustererEvent
   acceptClusterer(BatchClustererEvent)   - 
Method in class weka.gui.beans. ClustererPerformanceEvaluator 
 Accept a clusterer to be evaluated
   acceptClusterer(BatchClustererEvent)   - 
Method in class weka.gui.beans. PredictionAppender 
 Accept and process a batch clusterer event
   acceptClusterer(BatchClustererEvent)   - 
Method in class weka.gui.beans. SerializedModelSaver 
 Accept and save a batch trained clusterer.
   acceptDataPoint(ChartEvent)   - 
Method in interface weka.gui.beans. ChartListener 
  
   acceptDataPoint(ChartEvent)   - 
Method in class weka.gui.beans. StripChart 
 Accept a data point (encapsulated in a chart event) to plot
   acceptDataPoint(double[])   - 
Method in class weka.gui.beans. StripChart 
 Accept a data point to plot
   acceptDataSet(DataSetEvent)   - 
Method in class weka.gui.beans. AbstractDataSink 
 Accept a data set
   acceptDataSet(ThresholdDataEvent)   - 
Method in class weka.gui.beans. AbstractDataSink 
 Accept a threshold data set
   acceptDataSet(DataSetEvent)   - 
Method in class weka.gui.beans. AbstractTrainAndTestSetProducer 
 Subclass must implement
   acceptDataSet(DataSetEvent)   - 
Method in class weka.gui.beans. Associator 
  
   acceptDataSet(DataSetEvent)   - 
Method in class weka.gui.beans. ClassAssigner 
  
   acceptDataSet(DataSetEvent)   - 
Method in class weka.gui.beans. ClassValuePicker 
  
   acceptDataSet(ThresholdDataEvent)   - 
Method in class weka.gui.beans. CostBenefitAnalysis 
 Accept a threshold data event and set up the visualization.
   acceptDataSet(DataSetEvent)   - 
Method in class weka.gui.beans. CrossValidationFoldMaker 
 Accept a data set
   acceptDataSet(DataSetEvent)   - 
Method in interface weka.gui.beans. DataSourceListener 
  
   acceptDataSet(DataSetEvent)   - 
Method in class weka.gui.beans. DataVisualizer 
 Accept a data set
   acceptDataSet(DataSetEvent)   - 
Method in class weka.gui.beans. Filter 
 Accept a data set
   acceptDataSet(ThresholdDataEvent)   - 
Method in class weka.gui.beans. ModelPerformanceChart 
 Display a threshold curve.
   acceptDataSet(VisualizableErrorEvent)   - 
Method in class weka.gui.beans. ModelPerformanceChart 
 Display a scheme error plot.
   acceptDataSet(DataSetEvent)   - 
Method in class weka.gui.beans. Saver 
 Method reacts to a dataset event and starts the writing process in batch mode
   acceptDataSet(ThresholdDataEvent)   - 
Method in class weka.gui.beans. Saver 
 Method reacts to a threshold data event ans starts the writing process
 in batch mode.
   acceptDataSet(DataSetEvent)   - 
Method in class weka.gui.beans. TestSetMaker 
 Accepts and processes a data set event
   acceptDataSet(DataSetEvent)   - 
Method in class weka.gui.beans. TextViewer 
 Accept a data set for displaying as text
   acceptDataSet(ThresholdDataEvent)   - 
Method in interface weka.gui.beans. ThresholdDataListener 
  
   acceptDataSet(DataSetEvent)   - 
Method in class weka.gui.beans. TrainingSetMaker 
 Accept a data set
   acceptDataSet(DataSetEvent)   - 
Method in class weka.gui.beans. TrainTestSplitMaker 
 Accept a data set
   acceptDataSet(VisualizableErrorEvent)   - 
Method in interface weka.gui.beans. VisualizableErrorListener 
  
   acceptGraph(GraphEvent)   - 
Method in interface weka.gui.beans. GraphListener 
 Describe  acceptGraph  method here.
   acceptGraph(GraphEvent)   - 
Method in class weka.gui.beans. GraphViewer 
 Accept a graph
   acceptInstance(InstanceEvent)   - 
Method in class weka.gui.beans. AbstractDataSink 
 Accept an instance
   acceptInstance(InstanceEvent)   - 
Method in class weka.gui.beans. ClassAssigner 
  
   acceptInstance(InstanceEvent)   - 
Method in class weka.gui.beans. Classifier 
 Accepts an instance for incremental processing.
   acceptInstance(InstanceEvent)   - 
Method in class weka.gui.beans. Filter 
 Accept an instance for processing by StreamableFilters only
   acceptInstance(InstanceEvent)   - 
Method in interface weka.gui.beans. InstanceListener 
 Accept and process an instance event
   acceptInstance(InstanceEvent)   - 
Method in class weka.gui.beans. InstanceStreamToBatchMaker 
 Accept an instance to add to the batch.
   acceptInstance(InstanceEvent)   - 
Method in class weka.gui.beans. Saver 
 Methods reacts to instance events and saves instances incrementally.
   acceptInstance(InstanceEvent)   - 
Method in class weka.gui.beans. StripChart 
  
   acceptResult(ResultProducer, Object[], Object[])   - 
Method in class weka.experiment. AveragingResultProducer 
 Accepts results from a ResultProducer.
   acceptResult(ResultProducer, Object[], Object[])   - 
Method in class weka.experiment. CSVResultListener 
 Just prints out each result as it is received.
   acceptResult(ResultProducer, Object[], Object[])   - 
Method in class weka.experiment. DatabaseResultListener 
 Submit the result to the appropriate table of the database
   acceptResult(ResultProducer, Object[], Object[])   - 
Method in class weka.experiment. DatabaseResultProducer 
 Accepts results from a ResultProducer.
   acceptResult(ResultProducer, Object[], Object[])   - 
Method in class weka.experiment. InstancesResultListener 
 Collects each instance and adjusts the header information.
   acceptResult(ResultProducer, Object[], Object[])   - 
Method in class weka.experiment. LearningRateResultProducer 
 Accepts results from a ResultProducer.
   acceptResult(ResultProducer, Object[], Object[])   - 
Method in interface weka.experiment. ResultListener 
 Accepts results from a ResultProducer.
   acceptTestSet(TestSetEvent)   - 
Method in class weka.gui.beans. AbstractDataSink 
 Accept a test set
   acceptTestSet(TestSetEvent)   - 
Method in class weka.gui.beans. ClassAssigner 
  
   acceptTestSet(TestSetEvent)   - 
Method in class weka.gui.beans. Classifier 
 Accepts a test set for a batch trained classifier
   acceptTestSet(TestSetEvent)   - 
Method in class weka.gui.beans. Clusterer 
 Accepts a test set for a batch trained clusterer
   acceptTestSet(TestSetEvent)   - 
Method in class weka.gui.beans. CrossValidationFoldMaker 
 Accept a test set
   acceptTestSet(TestSetEvent)   - 
Method in class weka.gui.beans. DataVisualizer 
 Accept a test set
   acceptTestSet(TestSetEvent)   - 
Method in class weka.gui.beans. Filter 
 Accept a test set
   acceptTestSet(TestSetEvent)   - 
Method in class weka.gui.beans. Saver 
 Method reacts to a test set event and starts the writing process in batch mode
   acceptTestSet(TestSetEvent)   - 
Method in interface weka.gui.beans. TestSetListener 
 Accept and process a test set event
   acceptTestSet(TestSetEvent)   - 
Method in class weka.gui.beans. TextViewer 
 Accept a test set for displaying as text
   acceptTestSet(TestSetEvent)   - 
Method in class weka.gui.beans. TrainTestSplitMaker 
 Accept a test set
   acceptText(TextEvent)   - 
Method in interface weka.gui.beans. TextListener 
 Accept and process a text event
   acceptText(TextEvent)   - 
Method in class weka.gui.beans. TextViewer 
 Accept some text
   acceptTrainingSet(TrainingSetEvent)   - 
Method in class weka.gui.beans. AbstractDataSink 
 Accept a training set
   acceptTrainingSet(TrainingSetEvent)   - 
Method in class weka.gui.beans. Associator 
 Accept a training set
   acceptTrainingSet(TrainingSetEvent)   - 
Method in class weka.gui.beans. ClassAssigner 
  
   acceptTrainingSet(TrainingSetEvent)   - 
Method in class weka.gui.beans. Classifier 
 Accepts a training set and builds batch classifier
   acceptTrainingSet(TrainingSetEvent)   - 
Method in class weka.gui.beans. Clusterer 
 Accepts a training set and builds batch clusterer
   acceptTrainingSet(TrainingSetEvent)   - 
Method in class weka.gui.beans. CrossValidationFoldMaker 
 Accept a training set
   acceptTrainingSet(TrainingSetEvent)   - 
Method in class weka.gui.beans. DataVisualizer 
 Accept a training set
   acceptTrainingSet(TrainingSetEvent)   - 
Method in class weka.gui.beans. Filter 
 Accept a training set
   acceptTrainingSet(TrainingSetEvent)   - 
Method in class weka.gui.beans. Saver 
 Method reacts to a training set event and starts the writing process in batch
 mode
   acceptTrainingSet(TrainingSetEvent)   - 
Method in class weka.gui.beans. TextViewer 
 Accept a training set for displaying as text
   acceptTrainingSet(TrainingSetEvent)   - 
Method in interface weka.gui.beans. TrainingSetListener 
 Accept and process a training set
   acceptTrainingSet(TrainingSetEvent)   - 
Method in class weka.gui.beans. TrainTestSplitMaker 
 Accept a training set
   accuracy()   - 
Method in class weka.associations. RuleItem 
 Gets the expected predictive accuracy of a rule
   ACCURACY   - 
Static variable in class weka.classifiers.meta. ThresholdSelector 
 accuracy
   actEntropy   - 
Variable in class weka.classifiers.lazy.kstar. KStarWrapper 
 used/reused to hold the actual entropy
   action_table()   - 
Method in class weka.core.mathematicalexpression. Parser 
 Access to parse-action table.
   action_table()   - 
Method in class weka.filters.unsupervised.instance.subsetbyexpression. Parser 
 Access to parse-action table.
   actionPerformed(ActionEvent)   - 
Method in class weka.gui.arffviewer. ArffPanel 
 invoked when an action occurs
   actionPerformed(ActionEvent)   - 
Method in class weka.gui.arffviewer. ArffViewerMainPanel 
 invoked when an action occurs
   actionPerformed(ActionEvent)   - 
Method in class weka.gui.experiment. AlgorithmListPanel 
 Handle actions when buttons get pressed.
   actionPerformed(ActionEvent)   - 
Method in class weka.gui.experiment. DatasetListPanel 
 Handle actions when buttons get pressed.
   actionPerformed(ActionEvent)   - 
Method in class weka.gui.experiment. GeneratorPropertyIteratorPanel 
 Handles the various button clicking type activities.
   actionPerformed(ActionEvent)   - 
Method in class weka.gui.experiment. HostListPanel 
 Handle actions when text is entered into the host field or the
 delete button is pressed.
   actionPerformed(ActionEvent)   - 
Method in class weka.gui.experiment. RunPanel 
 Controls starting and stopping the experiment.
   actionPerformed(ActionEvent)   - 
Method in class weka.gui. SimpleCLIPanel 
 Only gets called when return is pressed in the input area, which
 starts the command running.
   actionPerformed(ActionEvent)   - 
Method in class weka.gui.streams. InstanceLoader 
  
   actionPerformed(ActionEvent)   - 
Method in class weka.gui.treevisualizer. TreeVisualizer 
 Performs the action associated with the ActionEvent.
   actual()   - 
Method in class weka.classifiers.evaluation. NominalPrediction 
 Gets the actual class value.
   actual()   - 
Method in class weka.classifiers.evaluation. NumericPrediction 
 Gets the actual class value.
   actual()   - 
Method in interface weka.classifiers.evaluation. Prediction 
 Gets the actual class value.
   actualNumBags()   - 
Method in class weka.classifiers.trees.j48. Distribution 
 Returns number of non-empty bags of distribution.
   actualNumClasses()   - 
Method in class weka.classifiers.trees.j48. Distribution 
 Returns number of classes actually occuring in distribution.
   actualNumClasses(int)   - 
Method in class weka.classifiers.trees.j48. Distribution 
 Returns number of classes actually occuring in given bag.
   acuityTipText()   - 
Method in class weka.clusterers. Cobweb 
 Returns the tip text for this property
   AdaBoostM1   - Class in  weka.classifiers.meta  Class for boosting a nominal class classifier using the Adaboost M1 method.   AdaBoostM1()   - 
Constructor for class weka.classifiers.meta. AdaBoostM1 
 Constructor.
   add(Object)   - 
Method in class weka.associations.tertius. SimpleLinkedList 
  
   add(int, Instance)   - 
Method in class weka.classifiers.trees.j48. Distribution 
 Adds given instance to given bag.
   add(int, double[])   - 
Method in class weka.classifiers.trees.j48. Distribution 
 Adds counts to given bag.
   add(double, Object)   - 
Method in class weka.clusterers.forOPTICSAndDBScan.Utils. PriorityQueue 
 Adds a new Object to the queue
   add(double, Object, String)   - 
Method in class weka.clusterers.forOPTICSAndDBScan.Utils. UpdateQueue 
 Adds a new Object to the queue
   add(AlgVector)   - 
Method in class weka.core. AlgVector 
 Returns the sum of this vector with another.
   add(Instance)   - 
Method in class weka.core. Instances 
 Adds one instance to the end of the set.
   add(Matrix)   - 
Method in class weka.core. Matrix 
  Deprecated.  Returns the sum of this matrix with another.
   add(String)   - 
Method in class weka.core. Stopwords 
 adds the given word to the stopword list (is automatically converted to
 lower case and trimmed)
   add(TechnicalInformation)   - 
Method in class weka.core. TechnicalInformation 
 adds the given information to the list of additional technical 
 informations
   add(TechnicalInformation.Type)   - 
Method in class weka.core. TechnicalInformation 
 Adds an empty technical information with the given type to the list
 of additional informations and returns the instance.
   add(PrintStream)   - 
Method in class weka.core. Tee 
 adds the given PrintStream to the list of streams, with NO timestamp and
 NO prefix.
   add(PrintStream, boolean)   - 
Method in class weka.core. Tee 
 adds the given PrintStream to the list of streams, with NO prefix.
   add(PrintStream, boolean, String)   - 
Method in class weka.core. Tee 
 adds the given PrintStream to the list of streams.
   add(String)   - 
Method in class weka.core. Trie 
 Ensures that this collection contains the specified element.
   add(String)   - 
Method in class weka.core. Trie.TrieNode 
 adds the given string to its children (creates children if necessary)
   add(String, Method)   - 
Method in class weka.core.xml. MethodHandler 
 adds the specified method for the property with the given displayname
 to its internal list.
   add(Class, Method)   - 
Method in class weka.core.xml. MethodHandler 
 adds the specified method for the given class to its internal list.
   add(double, double)   - 
Method in class weka.experiment. PairedStats 
 Add an observed pair of values.
   add(double[], double[])   - 
Method in class weka.experiment. PairedStats 
 Adds an array of observed pair of values.
   add(double)   - 
Method in class weka.experiment. Stats 
 Adds a value to the observed values
   add(double, double)   - 
Method in class weka.experiment. Stats 
 Adds a value that has been seen n times to the observed values
   Add   - Class in  weka.filters.unsupervised.attribute  An instance filter that adds a new attribute to the dataset.   Add()   - 
Constructor for class weka.filters.unsupervised.attribute. Add 
  
   add(int, Object)   - 
Method in class weka.gui. CheckBoxList.CheckBoxListModel 
 Inserts the specified element at the specified position in this list.
   add(String)   - 
Method in class weka.gui. HierarchyPropertyParser 
 Add the given item of property to the tree
   ADD_CHILDREN   - 
Static variable in class weka.gui.treevisualizer. TreeDisplayEvent 
  
   addActionListener(ActionListener)   - 
Method in class weka.gui.boundaryvisualizer. BoundaryPanel 
 Register a listener to be notified when plotting completes
   addActionListener(ActionListener)   - 
Method in class weka.gui.experiment. GeneratorPropertyIteratorPanel 
 Add a listener interested in kowing about editor status changes
   addActionListener(ActionListener)   - 
Method in class weka.gui.visualize. ClassPanel 
 Add an action listener that will be notified if the user changes the
 colour of a label
   addActionListener(ActionListener)   - 
Method in class weka.gui.visualize. VisualizePanel 
 Add a listener for this visualize panel
   addAll(SimpleLinkedList)   - 
Method in class weka.associations.tertius. SimpleLinkedList 
  
   addAll(Collection)   - 
Method in class weka.core.neighboursearch.covertrees. Stack 
 Adds all the given elements in the stack.
   addAll(Collection<? extends String>)   - 
Method in class weka.core. Trie 
 Adds all of the elements in the specified collection to this collection
   addAllBeansToContainer(JComponent)   - 
Static method in class weka.gui.beans. BeanInstance 
 Adds all beans to the supplied component
   addAllowed(Class, String)   - 
Method in class weka.core.xml. PropertyHandler 
 adds the given property (display name) to the list of allowed properties 
 for the specified class.
   addAndUpdate(Rule)   - 
Method in class weka.classifiers.rules. RuleStats 
 Add a rule to the ruleset and update the stats
   addArc(String, String)   - 
Method in class weka.classifiers.bayes.net. EditableBayesNet 
 Add arc between two nodes Distributions are updated by duplication for
 every value of the parent node.
   addArc(int, int)   - 
Method in class weka.classifiers.bayes.net. EditableBayesNet 
 Add arc between two nodes Distributions are updated by duplication for
 every value of the parent node.
   addArc(String, FastVector)   - 
Method in class weka.classifiers.bayes.net. EditableBayesNet 
 Add arc between parent node and each of the nodes in a given list.
   addAttributePanelListener(AttributePanelListener)   - 
Method in class weka.gui.visualize. AttributePanel 
 Add a listener to the list of things listening to this panel
   addBatchClassifierListener(BatchClassifierListener)   - 
Method in class weka.gui.beans. Classifier 
 Add a batch classifier listener
   addBatchClustererListener(BatchClustererListener)   - 
Method in class weka.gui.beans. Clusterer 
 Add a batch clusterer listener
   addBean(JComponent)   - 
Method in class weka.gui.beans. BeanInstance 
 Adds this bean to the global list of beans and
 to the supplied container.
   addBefore(Object)   - 
Method in class weka.associations.tertius. SimpleLinkedList.LinkedListIterator 
  
   addCancelListener(ActionListener)   - 
Method in class weka.gui. GenericObjectEditor.GOEPanel 
 This is used to hook an action listener to the cancel button.
   addCapabilitiesFilterListener(Explorer.CapabilitiesFilterChangeListener)   - 
Method in class weka.gui.explorer. Explorer 
 adds the listener to the list of objects that listen for changes of the
 CapabilitiesFilter
   addChangeListener(ChangeListener)   - 
Method in class weka.gui.arffviewer. ArffPanel 
 Adds a ChangeListener to the panel
   addChangeListener(ChangeListener)   - 
Method in class weka.gui.arffviewer. ArffTable 
 Adds a ChangeListener to the panel
   addChartListener(ChartListener)   - 
Method in class weka.gui.beans. IncrementalClassifierEvaluator 
 Add a chart listener
   addCheckBoxActionListener(ActionListener)   - 
Method in class weka.gui.experiment. DistributeExperimentPanel 
 Enable objects to listen for changes to the check box
   addChild(Splitter, ADTree)   - 
Method in class weka.classifiers.trees.adtree. PredictionNode 
 Adds a child to this node.
   addChild(Edge)   - 
Method in class weka.gui.treevisualizer. Node 
 Set the value of children.
   addChildClique(MarginCalculator.JunctionTreeNode)   - 
Method in class weka.classifiers.bayes.net. MarginCalculator.JunctionTreeNode 
  
   addChildFrame(Container)   - 
Method in class weka.gui. GUIChooser 
 adds the given child frame to the list of frames.
   addChildFrame(Container)   - 
Method in class weka.gui. Main 
 adds the given child frame to the list of frames.
   AddClassification   - Class in  weka.filters.supervised.attribute  A filter for adding the classification, the class distribution and an error flag to a dataset with a classifier.   AddClassification()   - 
Constructor for class weka.filters.supervised.attribute. AddClassification 
  
   AddCluster   - Class in  weka.filters.unsupervised.attribute  A filter that adds a new nominal attribute representing the cluster assigned to each instance by the specified clustering algorithm.   AddCluster()   - 
Constructor for class weka.filters.unsupervised.attribute. AddCluster 
  
   addConnectionListener(ConnectionListener)   - 
Method in class weka.gui.sql. ConnectionPanel 
 adds the given listener to the list of listeners.
   addConnectionListener(ConnectionListener)   - 
Method in class weka.gui.sql. SqlViewer 
 adds the given listener to the list of listeners.
   addCons(int[])   - 
Method in class weka.associations. PriorEstimation 
 generates a class association rule out of a given premise.
   addCVParameter(String)   - 
Method in class weka.classifiers.meta. CVParameterSelection 
 Adds a scheme parameter to the list of parameters to be set
 by cross-validation
   addDataFormatListener(DataFormatListener)   - 
Method in class weka.gui.beans. ClassAssigner 
  
   addDataFormatListener(DataFormatListener)   - 
Method in class weka.gui.beans. ClassValuePicker 
  
   addDataSourceListener(DataSourceListener)   - 
Method in class weka.gui.beans. AbstractDataSource 
 Add a listener
   addDataSourceListener(DataSourceListener)   - 
Method in class weka.gui.beans. ClassAssigner 
  
   addDataSourceListener(DataSourceListener)   - 
Method in class weka.gui.beans. ClassValuePicker 
  
   addDataSourceListener(DataSourceListener)   - 
Method in interface weka.gui.beans. DataSource 
 Add a data source listener
   addDataSourceListener(DataSourceListener)   - 
Method in class weka.gui.beans. DataVisualizer 
 Add a listener
   addDataSourceListener(DataSourceListener)   - 
Method in class weka.gui.beans. Filter 
 Add a data source listener
   addDataSourceListener(DataSourceListener)   - 
Method in class weka.gui.beans. InstanceStreamToBatchMaker 
  
   addDataSourceListener(DataSourceListener)   - 
Method in class weka.gui.beans. Loader 
 Add a listener
   addDataSourceListener(DataSourceListener)   - 
Method in class weka.gui.beans. PredictionAppender 
 Add a datasource listener
   addElement(Literal)   - 
Method in class weka.associations.tertius. LiteralSet 
 Add a Literal to this set.
   addElement(Object)   - 
Method in class weka.core. FastVector 
 Adds an element to this vector.
   addElement(int, int, double)   - 
Method in class weka.core. Matrix 
  Deprecated.  Add a value to an element.
   addElement(double)   - 
Method in class weka.core.matrix. DoubleVector 
 Adds an element into the vector
   addElement(Object)   - 
Method in class weka.gui. CheckBoxList.CheckBoxListModel 
 Adds the specified component to the end of this list.
   addErrs(double, double, float)   - 
Static method in class weka.classifiers.trees.j48. Stats 
 Computes estimated extra error for given total number of instances
 and error using normal approximation to binomial distribution
 (and continuity correction).
   AddExpression   - Class in  weka.filters.unsupervised.attribute  An instance filter that creates a new attribute by applying a mathematical expression to existing attributes.   AddExpression()   - 
Constructor for class weka.filters.unsupervised.attribute. AddExpression 
  
   addFile(String)   - 
Static method in class weka.core. ClassloaderUtil 
 Add file to CLASSPATH
   addFile(File)   - 
Static method in class weka.core. ClassloaderUtil 
 Add file to CLASSPATH
   addFirst(Object)   - 
Method in class weka.associations.tertius. SimpleLinkedList 
  
   addGraphListener(GraphListener)   - 
Method in class weka.gui.beans. Associator 
 Add a graph listener
   addGraphListener(GraphListener)   - 
Method in class weka.gui.beans. Classifier 
 Add a graph listener
   addGraphListener(GraphListener)   - 
Method in class weka.gui.beans. Clusterer 
 Add a graph listener
   addHeader(String, String)   - 
Method in class weka.experiment. ResultMatrix 
 adds the key-value pair to the header
   addHistoryChangedListener(HistoryChangedListener)   - 
Method in class weka.gui.sql. ConnectionPanel 
 adds the given listener to the list of listeners.
   addHistoryChangedListener(HistoryChangedListener)   - 
Method in class weka.gui.sql. QueryPanel 
 adds the given listener to the list of listeners.
   addHistoryChangedListener(HistoryChangedListener)   - 
Method in class weka.gui.sql. SqlViewer 
 adds the given listener to the list of listeners.
   AddID   - Class in  weka.filters.unsupervised.attribute  An instance filter that adds an ID attribute to the dataset.   AddID()   - 
Constructor for class weka.filters.unsupervised.attribute. AddID 
  
   addIgnored(String)   - 
Method in class weka.core.xml. PropertyHandler 
 adds the given display name of a property to the ignore list.
   addIgnored(Class, String)   - 
Method in class weka.core.xml. PropertyHandler 
 adds the given class with the display name of a property to the ignore list.
   addIncrementalClassifierListener(IncrementalClassifierListener)   - 
Method in class weka.gui.beans. Classifier 
 Add an incremental classifier listener
   addInstance(Instance)   - 
Method in class weka.clusterers. Cobweb 
  Deprecated.   updateClusterer(Instance) should be used instead 
   addInstance(BallNode, Instance)   - 
Method in class weka.core.neighboursearch.balltrees. BallTreeConstructor 
 Adds an instance to the ball tree.
   addInstance(BallNode, Instance)   - 
Method in class weka.core.neighboursearch.balltrees. BottomUpConstructor 
 Adds an instance to the ball tree.
   addInstance(BallNode, Instance)   - 
Method in class weka.core.neighboursearch.balltrees. MiddleOutConstructor 
 Adds an instance to the tree.
   addInstance(BallNode, Instance)   - 
Method in class weka.core.neighboursearch.balltrees. TopDownConstructor 
 Adds an instance to the ball tree.
   addInstanceInfo(Instance)   - 
Method in class weka.core.neighboursearch. BallTree 
 Adds the given instance's info.
   addInstanceInfo(Instance)   - 
Method in class weka.core.neighboursearch. CoverTree 
 Adds the given instance info.
   addInstanceInfo(Instance)   - 
Method in class weka.core.neighboursearch. KDTree 
 Adds one instance to KDTree loosly.
   addInstanceInfo(Instance)   - 
Method in class weka.core.neighboursearch. LinearNNSearch 
 Adds the given instance info.
   addInstanceInfo(Instance)   - 
Method in class weka.core.neighboursearch. NearestNeighbourSearch 
 Adds information from the given instance without modifying the 
 datastructure a lot.
   addInstanceListener(InstanceListener)   - 
Method in class weka.gui.beans. AbstractDataSource 
 Add an instance listener
   addInstanceListener(InstanceListener)   - 
Method in class weka.gui.beans. ClassAssigner 
  
   addInstanceListener(InstanceListener)   - 
Method in interface weka.gui.beans. DataSource 
 Add an instance listener
   addInstanceListener(InstanceListener)   - 
Method in class weka.gui.beans. Filter 
 Add an instance listener
   addInstanceListener(InstanceListener)   - 
Method in class weka.gui.beans. InstanceStreamToBatchMaker 
  
   addInstanceListener(InstanceListener)   - 
Method in class weka.gui.beans. Loader 
 Add an instance listener
   addInstanceListener(InstanceListener)   - 
Method in class weka.gui.beans. PredictionAppender 
 Add an instance listener
   addInstanceListener(InstanceListener)   - 
Method in class weka.gui.streams. InstanceJoiner 
  
   addInstanceListener(InstanceListener)   - 
Method in class weka.gui.streams. InstanceLoader 
  
   addInstanceListener(InstanceListener)   - 
Method in interface weka.gui.streams. InstanceProducer 
  
   addInstanceNumberAttribute()   - 
Method in class weka.gui.visualize. PlotData2D 
 Adds an instance number attribute to the plottable instances,
   AddInstanceToBestCluster(Instance)   - 
Method in class weka.clusterers. CLOPE 
 Add instance to best cluster
   addInstWithUnknown(Instances, int)   - 
Method in class weka.classifiers.trees.j48. Distribution 
 Adds all instances with unknown values for given attribute, weighted
 according to frequency of instances in each bag.
   additional()   - 
Method in class weka.core. TechnicalInformation 
 returns an enumeration of all the additional technical informations (if
 there are any)
   AdditionalMeasureProducer   - Interface in  weka.core  Interface to something that can produce measures other than those
 calculated by evaluation modules.   AdditiveRegression   - Class in  weka.classifiers.meta  Meta classifier that enhances the performance of a regression base classifier.   AdditiveRegression()   - 
Constructor for class weka.classifiers.meta. AdditiveRegression 
 Default constructor specifying DecisionStump as the classifier
   AdditiveRegression(Classifier)   - 
Constructor for class weka.classifiers.meta. AdditiveRegression 
 Constructor which takes base classifier as argument.
   addLayoutCompleteEventListener(LayoutCompleteEventListener)   - 
Method in class weka.gui.graphvisualizer. HierarchicalBCEngine 
 Method to add a LayoutCompleteEventListener
   addLayoutCompleteEventListener(LayoutCompleteEventListener)   - 
Method in interface weka.gui.graphvisualizer. LayoutEngine 
 This method adds a LayoutCompleteEventListener to the
 LayoutEngine.
   addLiteral(Literal)   - 
Method in class weka.associations.tertius. Predicate 
  
   addMouseListener(MouseListener)   - 
Method in class weka.gui.boundaryvisualizer. BoundaryPanel 
 Adds a mouse listener.
   addMouseListenerToHeader(JTable)   - 
Method in class weka.gui. SortedTableModel 
 Adds a mouselistener to the header: left-click on the header sorts in
 ascending manner, using shift-left-click in descending manner.
   addNode(String, int)   - 
Method in class weka.classifiers.bayes.net. EditableBayesNet 
 Add new node to the network, initializing instances, parentsets,
 distributions.
   addNode(String, int, int, int)   - 
Method in class weka.classifiers.bayes.net. EditableBayesNet 
 Add node to network at a given position, initializing instances, parentsets,
 distributions.
   addNodeValue(int, String)   - 
Method in class weka.classifiers.bayes.net. EditableBayesNet 
 Add node value to a node.
   AddNoise   - Class in  weka.filters.unsupervised.attribute  An instance filter that changes a percentage of a given attributes values.   AddNoise()   - 
Constructor for class weka.filters.unsupervised.attribute. AddNoise 
  
   addNoise(Instances, int, int, int, boolean)   - 
Method in class weka.filters.unsupervised.attribute. AddNoise 
 add noise to the dataset
 
 a given percentage of the instances are changed in the  way, that 
 a set of instances are randomly selected using seed.
   addObject(String, Object)   - 
Method in class weka.gui. ResultHistoryPanel 
 Adds an object to the results list
   addOkListener(ActionListener)   - 
Method in class weka.gui. GenericObjectEditor.GOEPanel 
 This is used to hook an action listener to the ok button.
   addParent(int, Instances)   - 
Method in class weka.classifiers.bayes.net. ParentSet 
 Add parent to parent set and update internals (specifically the cardinality of the parent set)
   addParent(int, int, Instances)   - 
Method in class weka.classifiers.bayes.net. ParentSet 
 Add parent to parent set at specific location 
 and update internals (specifically the cardinality of the parent set)
   addPlot(PlotData2D)   - 
Method in class weka.gui.visualize. Plot2D 
 Add a plot to the list of plots to display
   addPlot(PlotData2D)   - 
Method in class weka.gui.visualize. VisualizePanel 
 Set a new plot to the visualize panel
   addPrediction(NominalPrediction)   - 
Method in class weka.classifiers.evaluation. ConfusionMatrix 
 Includes a prediction in the confusion matrix.
   addPredictions(FastVector)   - 
Method in class weka.classifiers.evaluation. ConfusionMatrix 
 Includes a whole bunch of predictions in the confusion matrix.
   addPropertyChangeListener(String, PropertyChangeListener)   - 
Method in class weka.gui.beans. AbstractDataSource 
 Add a property change listener to this bean
   addPropertyChangeListener(PropertyChangeListener)   - 
Method in class weka.gui.beans. AssociatorCustomizer 
 Add a property change listener
   addPropertyChangeListener(PropertyChangeListener)   - 
Method in class weka.gui.beans. BeanVisual 
 Add a listener for property change events
   addPropertyChangeListener(PropertyChangeListener)   - 
Method in class weka.gui.beans. ClassAssignerCustomizer 
 Add a property change listener
   addPropertyChangeListener(PropertyChangeListener)   - 
Method in class weka.gui.beans. ClassifierCustomizer 
 Add a property change listener
   addPropertyChangeListener(PropertyChangeListener)   - 
Method in class weka.gui.beans. ClassValuePickerCustomizer 
 Add a property change listener
   addPropertyChangeListener(PropertyChangeListener)   - 
Method in class weka.gui.beans. ClustererCustomizer 
 Add a property change listener
   addPropertyChangeListener(PropertyChangeListener)   - 
Method in class weka.gui.beans. CrossValidationFoldMakerCustomizer 
 Add a property change listener
   addPropertyChangeListener(String, PropertyChangeListener)   - 
Method in class weka.gui.beans. DataVisualizer 
 Add a property change listener to this bean
   addPropertyChangeListener(PropertyChangeListener)   - 
Method in class weka.gui.beans. FilterCustomizer 
 Add a property change listener
   addPropertyChangeListener(PropertyChangeListener)   - 
Method in class weka.gui.beans. IncrementalClassifierEvaluatorCustomizer 
 Add a property change listener
   addPropertyChangeListener(PropertyChangeListener)   - 
Method in class weka.gui.beans. LoaderCustomizer 
 Add a property change listener
   addPropertyChangeListener(String, PropertyChangeListener)   - 
Method in class weka.gui.beans. ModelPerformanceChart 
 Add a property change listener to this bean
   addPropertyChangeListener(PropertyChangeListener)   - 
Method in class weka.gui.beans. PredictionAppenderCustomizer 
 Add a property change listener
   addPropertyChangeListener(PropertyChangeListener)   - 
Method in class weka.gui.beans. SaverCustomizer 
 Add a property change listener
   addPropertyChangeListener(PropertyChangeListener)   - 
Method in class weka.gui.beans. SerializedModelSaverCustomizer 
 Add a property change listener
   addPropertyChangeListener(PropertyChangeListener)   - 
Method in class weka.gui.beans. StripChartCustomizer 
 Add a property change listener
   addPropertyChangeListener(String, PropertyChangeListener)   - 
Method in class weka.gui.beans. TextViewer 
 Add a property change listener to this bean
   addPropertyChangeListener(PropertyChangeListener)   - 
Method in class weka.gui.beans. TrainTestSplitMakerCustomizer 
 Add a property change listener
   addPropertyChangeListener(PropertyChangeListener)   - 
Method in class weka.gui. CostMatrixEditor 
 Adds an object to the list of those that wish to be informed when the
 cost matrix changes.
   addPropertyChangeListener(PropertyChangeListener)   - 
Method in class weka.gui.experiment. SetupModePanel 
 Adds a PropertyChangeListener who will be notified of value changes.
   addPropertyChangeListener(PropertyChangeListener)   - 
Method in class weka.gui.experiment. SetupPanel 
 Adds a PropertyChangeListener who will be notified of value changes.
   addPropertyChangeListener(PropertyChangeListener)   - 
Method in class weka.gui.experiment. SimpleSetupPanel 
 Adds a PropertyChangeListener who will be notified of value changes.
   addPropertyChangeListener(PropertyChangeListener)   - 
Method in class weka.gui.explorer. PreprocessPanel 
 Adds a PropertyChangeListener who will be notified of value changes.
   addPropertyChangeListener(PropertyChangeListener)   - 
Method in class weka.gui. GenericArrayEditor 
 Adds a PropertyChangeListener who will be notified of value changes.
   addPropertyChangeListener(PropertyChangeListener)   - 
Method in class weka.gui. GenericObjectEditor 
 Adds a PropertyChangeListener who will be notified of value changes.
   addPropertyChangeListener(PropertyChangeListener)   - 
Method in class weka.gui. PropertySheetPanel 
 Adds a PropertyChangeListener.
   addPropertyChangeListener(PropertyChangeListener)   - 
Method in class weka.gui. SetInstancesPanel 
 Adds a PropertyChangeListener who will be notified of value changes.
   addPropertyChangeListener(PropertyChangeListener)   - 
Method in class weka.gui. SimpleDateFormatEditor 
 Adds an object to the list of those that wish to be informed when the
 date format changes.
   addPropertyChangeListenersSubFlow(PropertyChangeListener)   - 
Method in class weka.gui.beans. MetaBean 
  
   addPSFontReplacement(String, String)   - 
Static method in class weka.gui.visualize. PostscriptGraphics 
 adds the PS font name to replace and its replacement in the replacement
 hashtable
   addQueryExecuteListener(QueryExecuteListener)   - 
Method in class weka.gui.sql. QueryPanel 
 adds the given listener to the list of listeners.
   addQueryExecuteListener(QueryExecuteListener)   - 
Method in class weka.gui.sql. SqlViewer 
 adds the given listener to the list of listeners.
   addRange(int, Instances, int, int)   - 
Method in class weka.classifiers.trees.j48. Distribution 
 Adds all instances in given range to given bag.
   addReference(Instance)   - 
Method in class weka.classifiers.trees.adtree. ReferenceInstances 
 Adds one instance reference to the end of the set.
   addRelation(Instances)   - 
Method in class weka.core. Attribute 
 Adds a relation to a relation-valued attribute.
   addRemoteExperimentListener(RemoteExperimentListener)   - 
Method in class weka.experiment. RemoteExperiment 
 Add an object to the list of those interested in recieving update
 information from the RemoteExperiment
   addRemoteExperimentListener(RemoteExperimentListener)   - 
Method in class weka.gui.boundaryvisualizer. BoundaryPanelDistributed 
 Add an object to the list of those interested in recieving update
 information from the RemoteExperiment
   addRemoteHost(String)   - 
Method in class weka.experiment. RemoteExperiment 
 Add a host name to the list of remote hosts
   addRenderingHints(Map)   - 
Method in class weka.gui.visualize. PostscriptGraphics 
  
   addRepaintNotify(Component)   - 
Method in class weka.gui.visualize. ClassPanel 
 Adds a component that will need to be repainted if the user
 changes the colour of a label.
   addRepaintNotify(Component)   - 
Method in class weka.gui.visualize. LegendPanel 
 Adds a component that will need to be repainted if the user
 changes the colour of a label.
   addResult(String, StringBuffer)   - 
Method in class weka.gui. ResultHistoryPanel 
 Adds a new result to the result list.
   addResultChangedListener(ResultChangedListener)   - 
Method in class weka.gui.sql. ResultPanel 
 adds the given listener to the list of listeners
   addResultChangedListener(ResultChangedListener)   - 
Method in class weka.gui.sql. SqlViewer 
 adds the given listener to the list of listeners.
   addStartupListener(StartUpListener)   - 
Static method in class weka.gui.beans. KnowledgeFlowApp 
 Add a listener to be notified when startup is complete
   addStartupListener(StartUpListener)   - 
Static method in class weka.gui. Main 
 Add a listener to be notified when startup is complete.
   addStringValue(String)   - 
Method in class weka.core. Attribute 
 Adds a string value to the list of valid strings for attributes
 of type STRING and returns the index of the string.
   addStringValue(Attribute, int)   - 
Method in class weka.core. Attribute 
 Adds a string value to the list of valid strings for attributes
 of type STRING and returns the index of the string.
   addTableModelListener(TableModelListener)   - 
Method in class weka.gui.arffviewer. ArffSortedTableModel 
 adds a listener to the list that is notified each time a change to data 
 model occurs
   addTableModelListener(TableModelListener)   - 
Method in class weka.gui.arffviewer. ArffTableModel 
 adds a listener to the list that is notified each time a change to data
 model occurs
   addTableModelListener(TableModelListener)   - 
Method in class weka.gui.sql. ResultSetTableModel 
 adds a listener to the list that is notified each time a change to data 
 model occurs.
   addTestSetListener(TestSetListener)   - 
Method in class weka.gui.beans. AbstractTestSetProducer 
 Add a listener for test sets
   addTestSetListener(TestSetListener)   - 
Method in class weka.gui.beans. AbstractTrainAndTestSetProducer 
 Add a test set listener
   addTestSetListener(TestSetListener)   - 
Method in class weka.gui.beans. ClassAssigner 
  
   addTestSetListener(TestSetListener)   - 
Method in class weka.gui.beans. Filter 
 Add a test set listener
   addTestSetListener(TestSetListener)   - 
Method in class weka.gui.beans. PredictionAppender 
 Add a test set listener
   addTestSetListener(TestSetListener)   - 
Method in interface weka.gui.beans. TestSetProducer 
 Add a listener for test set events
   addTextListener(TextListener)   - 
Method in class weka.gui.beans. Associator 
 Add a text listener
   addTextListener(TextListener)   - 
Method in class weka.gui.beans. Classifier 
 Add a text listener
   addTextListener(TextListener)   - 
Method in class weka.gui.beans. ClassifierPerformanceEvaluator 
 Add a text listener
   addTextListener(TextListener)   - 
Method in class weka.gui.beans. Clusterer 
 Add a text listener
   addTextListener(TextListener)   - 
Method in class weka.gui.beans. ClustererPerformanceEvaluator 
 Add a text listener
   addTextListener(TextListener)   - 
Method in class weka.gui.beans. IncrementalClassifierEvaluator 
 Add a text listener
   addTextListener(TextListener)   - 
Method in class weka.gui.beans. TextViewer 
 Add a text listener
   addThresholdDataListener(ThresholdDataListener)   - 
Method in class weka.gui.beans. ClassifierPerformanceEvaluator 
 Add a threshold data listener
   addToList(Object[], double)   - 
Method in class weka.attributeSelection. BestFirst.LinkedList2 
 adds an element (Link) to the list.
   addToList(Object[], double)   - 
Method in class weka.attributeSelection. LFSMethods.LinkedList2 
 adds an element (Link) to the list.
   addTrainingInstance(Instance)   - 
Method in class weka.gui.boundaryvisualizer. BoundaryPanel 
 Adds a training instance to the visualization dataset.
   addTrainingInstanceFromMouseLocation(int, int, int, double)   - 
Method in class weka.gui.boundaryvisualizer. BoundaryPanel 
 Adds a training instance to our dataset, based on the coordinates of the mouse on the panel.
   addTrainingSetListener(TrainingSetListener)   - 
Method in class weka.gui.beans. AbstractTrainAndTestSetProducer 
 Add a training set listener
   addTrainingSetListener(TrainingSetListener)   - 
Method in class weka.gui.beans. AbstractTrainingSetProducer 
 Add a training set listener
   addTrainingSetListener(TrainingSetListener)   - 
Method in class weka.gui.beans. ClassAssigner 
  
   addTrainingSetListener(TrainingSetListener)   - 
Method in class weka.gui.beans. Filter 
 Add a training set listener
   addTrainingSetListener(TrainingSetListener)   - 
Method in class weka.gui.beans. PredictionAppender 
 Add a training set listener
   addTrainingSetListener(TrainingSetListener)   - 
Method in interface weka.gui.beans. TrainingSetProducer 
 Add a training set listener
   addUndoPoint()   - 
Method in interface weka.core. Undoable 
 adds an undo point to the undo history
   addUndoPoint()   - 
Method in class weka.gui.arffviewer. ArffPanel 
 adds the current state of the instances to the undolist
   addUndoPoint()   - 
Method in class weka.gui.arffviewer. ArffSortedTableModel 
 adds an undo point to the undo history
   addUndoPoint()   - 
Method in class weka.gui.arffviewer. ArffTableModel 
 adds an undo point to the undo history, if the undo support is enabled
   addUndoPoint()   - 
Method in class weka.gui.explorer. PreprocessPanel 
 Backs up the current state of the dataset, so the changes can be undone.
   addURL(URL)   - 
Static method in class weka.core. ClassloaderUtil 
 Add URL to CLASSPATH
   addValue(double, double)   - 
Method in class weka.classifiers.bayes.net.estimate. DiscreteEstimatorBayes 
 Add a new data value to the current estimator.
   addValue(double, double, double)   - 
Method in interface weka.estimators. ConditionalEstimator 
 Add a new data value to the current estimator.
   addValue(double, double, double)   - 
Method in class weka.estimators. DDConditionalEstimator 
 Add a new data value to the current estimator.
   addValue(double, double)   - 
Method in class weka.estimators. DiscreteEstimator 
 Add a new data value to the current estimator.
   addValue(double, double, double)   - 
Method in class weka.estimators. DKConditionalEstimator 
 Add a new data value to the current estimator.
   addValue(double, double, double)   - 
Method in class weka.estimators. DNConditionalEstimator 
 Add a new data value to the current estimator.
   addValue(double, double)   - 
Method in class weka.estimators. Estimator 
 Add a new data value to the current estimator.
   addValue(double, double)   - 
Method in interface weka.estimators. IncrementalEstimator 
 Add one value to the current estimator.
   addValue(double, double, double)   - 
Method in class weka.estimators. KDConditionalEstimator 
 Add a new data value to the current estimator.
   addValue(double, double)   - 
Method in class weka.estimators. KernelEstimator 
 Add a new data value to the current estimator.
   addValue(double, double, double)   - 
Method in class weka.estimators. KKConditionalEstimator 
 Add a new data value to the current estimator.
   addValue(double, double)   - 
Method in class weka.estimators. MahalanobisEstimator 
 Add a new data value to the current estimator.
   addValue(double, double, double)   - 
Method in class weka.estimators. NDConditionalEstimator 
 Add a new data value to the current estimator.
   addValue(double, double, double)   - 
Method in class weka.estimators. NNConditionalEstimator 
 Add a new data value to the current estimator.
   addValue(double, double)   - 
Method in class weka.estimators. NormalEstimator 
 Add a new data value to the current estimator.
   addValue(double, double)   - 
Method in class weka.estimators. PoissonEstimator 
 Add a new data value to the current estimator.
   addValues(Instances, int)   - 
Method in class weka.estimators. Estimator 
 Initialize the estimator with a new dataset.
   addValues(Instances, int, double, double, double)   - 
Method in class weka.estimators. Estimator 
 Initialize the estimator with all values of one attribute of a dataset.
   addValues(Instances, int, int, int)   - 
Method in class weka.estimators. Estimator 
 Initialize the estimator using only the instance of one class.
   addValues(Instances, int, int, int, double, double)   - 
Method in class weka.estimators. Estimator 
 Initialize the estimator using only the instance of one class.
   AddValues   - Class in  weka.filters.unsupervised.attribute  Adds the labels from the given list to an attribute if they are missing.   AddValues()   - 
Constructor for class weka.filters.unsupervised.attribute. AddValues 
  
   addVariable(String, String)   - 
Method in class weka.core. Environment 
 Add a variable to the internal map.
   addVetoableChangeListener(String, VetoableChangeListener)   - 
Method in class weka.gui.beans. AbstractDataSource 
 Add a vetoable change listener to this bean
   addVetoableChangeListener(String, VetoableChangeListener)   - 
Method in class weka.gui.beans. CostBenefitAnalysis 
  
   addVetoableChangeListener(String, VetoableChangeListener)   - 
Method in class weka.gui.beans. DataVisualizer 
 Add a vetoable change listener to this bean
   addVetoableChangeListener(String, VetoableChangeListener)   - 
Method in class weka.gui.beans. GraphViewer 
 Add a vetoable change listener to this bean
   addVetoableChangeListener(String, VetoableChangeListener)   - 
Method in class weka.gui.beans. ModelPerformanceChart 
 Add a vetoable change listener to this bean
   addVetoableChangeListener(String, VetoableChangeListener)   - 
Method in class weka.gui.beans. TextViewer 
 Add a vetoable change listener to this bean
   addVisualizableErrorListener(VisualizableErrorListener)   - 
Method in class weka.gui.beans. ClassifierPerformanceEvaluator 
 Add a visualizable error listener
   addWeights(Instance, double[])   - 
Method in class weka.classifiers.trees.j48. Distribution 
 Adds given instance to all bags weighting it according to given weights.
   adjustCenter(double)   - 
Method in class weka.gui.treevisualizer. Node 
 Will increase or decrease the postion of center.
   adjustSize(SERObject)   - 
Method in class weka.clusterers.forOPTICSAndDBScan.OPTICS_GUI. GraphPanel 
 Adjusts the size of this panel in respect of the shown content
   adjustWeightsTipText()   - 
Method in class weka.filters.supervised.instance. SpreadSubsample 
 Returns the tip text for this property
   ADNode   - Class in  weka.classifiers.bayes.net  The ADNode class implements the ADTree datastructure which increases
 the speed with which sub-contingency tables can be constructed from
 a data set in an Instances object.   ADNode()   - 
Constructor for class weka.classifiers.bayes.net. ADNode 
 Creates new ADNode
   ADTree   - Class in  weka.classifiers.trees  Class for generating an alternating decision tree.   ADTree()   - 
Constructor for class weka.classifiers.trees. ADTree 
  
   advanceCounters()   - 
Method in class weka.experiment. Experiment 
 Increments iteration counters appropriately.
   advanceCounters()   - 
Method in class weka.experiment. RemoteExperiment 
 overides the one in Experiment
   Agrawal   - Class in  weka.datagenerators.classifiers.classification  Generates a people database and is based on the paper by Agrawal et al.: 
 R.   Agrawal()   - 
Constructor for class weka.datagenerators.classifiers.classification. Agrawal 
 initializes the generator with default values
   AIC   - 
Static variable in interface weka.classifiers.bayes.net.search.local. Scoreable 
  
   ALGORITHM_HAAR   - 
Static variable in class weka.filters.unsupervised.attribute. Wavelet 
 the type of algorithm: Haar wavelet
   ALGORITHM_PLS1   - 
Static variable in class weka.filters.supervised.attribute. PLSFilter 
 the type of algorithm: PLS1
   ALGORITHM_SIMPLS   - 
Static variable in class weka.filters.supervised.attribute. PLSFilter 
 the type of algorithm: SIMPLS
   AlgorithmListPanel   - Class in  weka.gui.experiment  This panel controls setting a list of algorithms for an experiment to
 iterate over.   AlgorithmListPanel(Experiment)   - 
Constructor for class weka.gui.experiment. AlgorithmListPanel 
 Creates the algorithm list panel with the given experiment.
   AlgorithmListPanel()   - 
Constructor for class weka.gui.experiment. AlgorithmListPanel 
 Create the algorithm list panel initially disabled.
   AlgorithmListPanel.ObjectCellRenderer   - Class in  weka.gui.experiment  Class required to show the Classifiers nicely in the list   AlgorithmListPanel.ObjectCellRenderer()   - 
Constructor for class weka.gui.experiment. AlgorithmListPanel.ObjectCellRenderer 
  
   algorithmTipText()   - 
Method in class weka.filters.supervised.attribute. PLSFilter 
 Returns the tip text for this property
   algorithmTipText()   - 
Method in class weka.filters.unsupervised.attribute. Wavelet 
 Returns the tip text for this property
   ALGORITHMTYPE_ARITHMETIC   - 
Static variable in class weka.classifiers.mi. MILR 
 collective MI assumption, arithmetic mean for posteriors
   ALGORITHMTYPE_DEFAULT   - 
Static variable in class weka.classifiers.mi. MILR 
 standard MI assumption
   ALGORITHMTYPE_GEOMETRIC   - 
Static variable in class weka.classifiers.mi. MILR 
 collective MI assumption, geometric mean for posteriors
   algorithmTypeTipText()   - 
Method in class weka.classifiers.mi. MILR 
 Returns the tip text for this property
   AlgVector   - Class in  weka.core  Class for performing operations on an algebraic vector
 of floating-point values.   AlgVector(int)   - 
Constructor for class weka.core. AlgVector 
 Constructs a vector and initializes it with default values.
   AlgVector(double[])   - 
Constructor for class weka.core. AlgVector 
 Constructs a vector using a given array.
   AlgVector(Instances, Random)   - 
Constructor for class weka.core. AlgVector 
 Constructs a vector using a given data format.
   AlgVector(Instance)   - 
Constructor for class weka.core. AlgVector 
 Constructs a vector using an instance.
   alignBottom(FastVector)   - 
Method in class weka.classifiers.bayes.net. EditableBayesNet 
 align set of nodes with the bottom most node in the list
   alignLeft(FastVector)   - 
Method in class weka.classifiers.bayes.net. EditableBayesNet 
 align set of nodes with the left most node in the list
   alignRight(FastVector)   - 
Method in class weka.classifiers.bayes.net. EditableBayesNet 
 align set of nodes with the right most node in the list
   alignTop(FastVector)   - 
Method in class weka.classifiers.bayes.net. EditableBayesNet 
 align set of nodes with the top most node in the list
   ALL   - 
Static variable in class weka.core. Debug 
 the log level All
   AllFilter   - Class in  weka.filters  A simple instance filter that passes all instances directly
 through.   AllFilter()   - 
Constructor for class weka.filters. AllFilter 
  
   AllJavadoc   - Class in  weka.core  Applies all known Javadoc-derived classes to a source file.   AllJavadoc()   - 
Constructor for class weka.core. AllJavadoc 
  
   allowed()   - 
Method in class weka.core.xml. PropertyHandler 
 returns an enumeration of the classnames for which only certain properties
 (display names) are allowed
   allowUnclassifiedInstancesTipText()   - 
Method in class weka.classifiers.trees. RandomTree 
 Returns the tip text for this property
   AlphabeticTokenizer   - Class in  weka.core.tokenizers  Alphabetic string tokenizer, tokens are to be formed only from contiguous alphabetic sequences.   AlphabeticTokenizer()   - 
Constructor for class weka.core.tokenizers. AlphabeticTokenizer 
  
   alphaTipText()   - 
Method in class weka.classifiers.bayes.net.estimate. BayesNetEstimator 
  
   alphaTipText()   - 
Method in class weka.classifiers.functions. Winnow 
 Returns the tip text for this property
   amplitudeTipText()   - 
Method in class weka.datagenerators.classifiers.regression. Expression 
 Returns the tip text for this property
   amplitudeTipText()   - 
Method in class weka.datagenerators.classifiers.regression. MexicanHat 
 Returns the tip text for this property
   and(Capabilities)   - 
Method in class weka.core. Capabilities 
 performs an AND conjunction with the capabilities of the given 
 Capabilities object and updates itself
   AND   - 
Static variable in interface weka.core.mathematicalexpression. sym 
  
   AND   - 
Static variable in interface weka.filters.unsupervised.instance.subsetbyexpression. sym 
  
   AODE   - Class in  weka.classifiers.bayes  AODE achieves highly accurate classification by averaging over all of a small space of alternative naive-Bayes-like models that have weaker (and hence less detrimental) independence assumptions than naive Bayes.   AODE()   - 
Constructor for class weka.classifiers.bayes. AODE 
  
   AODEsr   - Class in  weka.classifiers.bayes  AODEsr augments AODE with Subsumption Resolution.AODEsr detects specializations between two attribute values at classification time and deletes the generalization attribute value. 
 For more information, see: 
 Fei Zheng, Geoffrey I.   AODEsr()   - 
Constructor for class weka.classifiers.bayes. AODEsr 
  
   append(String, String)   - 
Method in class weka.gui.sql. InfoPanel 
 adds the given message to the end of the list (with the associated icon
 at the beginning)
   append(Object)   - 
Method in class weka.gui.sql. InfoPanel 
 adds the given message to the end of the list
   appendElements(FastVector)   - 
Method in class weka.core. FastVector 
 Appends all elements of the supplied vector to this vector.
   appendPredictedProbabilitiesTipText()   - 
Method in class weka.gui.beans. PredictionAppender 
 Return a tip text suitable for displaying in a GUI
   applyClassifier(PMMLModel, Instances)   - 
Static method in class weka.core.pmml. PMMLFactory 
  
   applyCostMatrix(Instances, Random)   - 
Method in class weka.classifiers. CostMatrix 
 Applies the cost matrix to a set of instances.
   applyMinMaxRescaleCast(double)   - 
Method in class weka.core.pmml. TargetMetaInfo 
 Apply min and max, rescaleFactor, rescaleConstant and castInteger - in
 that order (where defined).
   applyMissingAndOutlierTreatments(double[])   - 
Method in class weka.core.pmml. MiningSchema 
 Apply both missing and outlier treatments to an incoming instance.
   applyMissingValuesTreatment(double[])   - 
Method in class weka.core.pmml. MiningSchema 
 Apply the missing value treatments (if any) to an incoming instance.
   applyMissingValueTreatment(double)   - 
Method in class weka.core.pmml. MiningFieldMetaInfo 
 Apply the missing value treatment method for this field.
   applyOutlierTreatment(double)   - 
Method in class weka.core.pmml. MiningFieldMetaInfo 
 Apply the outlier treatment method for this field.
   applyOutlierTreatment(double[])   - 
Method in class weka.core.pmml. MiningSchema 
 Apply the outlier treatment methods (if any) to an incoming instance.
   APPROVE_OPTION   - 
Static variable in class weka.gui.experiment. OutputFormatDialog 
 Signifies an OK property selection.
   APPROVE_OPTION   - 
Static variable in class weka.gui. ListSelectorDialog 
 Signifies an OK property selection
   APPROVE_OPTION   - 
Static variable in class weka.gui. PropertySelectorDialog 
 Signifies an OK property selection
   APPROVE_OPTION   - 
Static variable in class weka.gui. ViewerDialog 
 Signifies an OK property selection
   Apriori   - Class in  weka.associations  Class implementing an Apriori-type algorithm.   Apriori()   - 
Constructor for class weka.associations. Apriori 
 Constructor that allows to sets default values for the 
 minimum confidence and the maximum number of rules
 the minimum confidence.
   aprioriGen(FastVector)   - 
Static method in class weka.associations.gsp. Sequence 
 Generates all possible candidate k-Sequences and prunes the ones that 
 contain an infrequent (k-1)-Sequence.
   AprioriItemSet   - Class in  weka.associations  Class for storing a set of items.   AprioriItemSet(int)   - 
Constructor for class weka.associations. AprioriItemSet 
 Constructor
   areaUnderROC(int)   - 
Method in class weka.classifiers. Evaluation 
 Returns the area under ROC for those predictions that have been collected
 in the evaluateClassifier(Classifier, Instances) method.
   ARFF_ATTRIBUTE   - 
Static variable in class weka.core. Attribute 
 The keyword used to denote the start of an arff attribute declaration
   ARFF_ATTRIBUTE_DATE   - 
Static variable in class weka.core. Attribute 
 The keyword used to denote a date attribute
   ARFF_ATTRIBUTE_INTEGER   - 
Static variable in class weka.core. Attribute 
 A keyword used to denote a numeric attribute
   ARFF_ATTRIBUTE_NUMERIC   - 
Static variable in class weka.core. Attribute 
 A keyword used to denote a numeric attribute
   ARFF_ATTRIBUTE_REAL   - 
Static variable in class weka.core. Attribute 
 A keyword used to denote a numeric attribute
   ARFF_ATTRIBUTE_RELATIONAL   - 
Static variable in class weka.core. Attribute 
 The keyword used to denote a relation-valued attribute
   ARFF_ATTRIBUTE_STRING   - 
Static variable in class weka.core. Attribute 
 The keyword used to denote a string attribute
   ARFF_DATA   - 
Static variable in class weka.core. Instances 
 The keyword used to denote the start of the arff data section
   ARFF_END_SUBRELATION   - 
Static variable in class weka.core. Attribute 
 The keyword used to denote the end of the declaration of a subrelation
   ARFF_RELATION   - 
Static variable in class weka.core. Instances 
 The keyword used to denote the start of an arff header
   ArffLoader   - Class in  weka.core.converters  Reads a source that is in arff (attribute relation file format) format.   ArffLoader()   - 
Constructor for class weka.core.converters. ArffLoader 
  
   ArffLoader.ArffReader   - Class in  weka.core.converters  Reads data from an ARFF file, either in incremental or batch mode.   ArffLoader.ArffReader(Reader)   - 
Constructor for class weka.core.converters. ArffLoader.ArffReader 
 Reads the data completely from the reader.
   ArffLoader.ArffReader(Reader, int)   - 
Constructor for class weka.core.converters. ArffLoader.ArffReader 
 Reads only the header and reserves the specified space for instances.
   ArffLoader.ArffReader(Reader, Instances, int)   - 
Constructor for class weka.core.converters. ArffLoader.ArffReader 
 Reads the data without header according to the specified template.
   ArffLoader.ArffReader(Reader, Instances, int, int)   - 
Constructor for class weka.core.converters. ArffLoader.ArffReader 
 Initializes the reader without reading the header according to the
 specified template.
   ArffPanel   - Class in  weka.gui.arffviewer  A Panel representing an ARFF-Table and the associated filename.   ArffPanel()   - 
Constructor for class weka.gui.arffviewer. ArffPanel 
 initializes the panel with no data
   ArffPanel(String)   - 
Constructor for class weka.gui.arffviewer. ArffPanel 
 initializes the panel and loads the specified file
   ArffPanel(Instances)   - 
Constructor for class weka.gui.arffviewer. ArffPanel 
 initializes the panel with the given data
   ArffSaver   - Class in  weka.core.converters  Writes to a destination in arff text format.   ArffSaver()   - 
Constructor for class weka.core.converters. ArffSaver 
 Constructor
   ArffSortedTableModel   - Class in  weka.gui.arffviewer  A sorter for the ARFF-Viewer - necessary because of the custom CellRenderer.   ArffSortedTableModel(String)   - 
Constructor for class weka.gui.arffviewer. ArffSortedTableModel 
 initializes the sorter w/o a model, but loads the given file and creates
 from that a model
   ArffSortedTableModel(Instances)   - 
Constructor for class weka.gui.arffviewer. ArffSortedTableModel 
 initializes the sorter w/o a model, but uses the given data to create
 a model from that
   ArffSortedTableModel(TableModel)   - 
Constructor for class weka.gui.arffviewer. ArffSortedTableModel 
 initializes the sorter with the given model
   ArffTable   - Class in  weka.gui.arffviewer  A specialized JTable for the Arff-Viewer.   ArffTable()   - 
Constructor for class weka.gui.arffviewer. ArffTable 
 initializes with no model
   ArffTable(TableModel)   - 
Constructor for class weka.gui.arffviewer. ArffTable 
 initializes with the given model
   ArffTableCellRenderer   - Class in  weka.gui.arffviewer  Handles the background colors for missing values differently than the
 DefaultTableCellRenderer.   ArffTableCellRenderer()   - 
Constructor for class weka.gui.arffviewer. ArffTableCellRenderer 
 initializes the Renderer with a standard color
   ArffTableCellRenderer(Color, Color)   - 
Constructor for class weka.gui.arffviewer. ArffTableCellRenderer 
 initializes the Renderer with the given colors
   ArffTableCellRenderer(Color, Color, Color, Color)   - 
Constructor for class weka.gui.arffviewer. ArffTableCellRenderer 
 initializes the Renderer with the given colors
   ArffTableModel   - Class in  weka.gui.arffviewer  The model for the Arff-Viewer.   ArffTableModel(String)   - 
Constructor for class weka.gui.arffviewer. ArffTableModel 
 initializes the object and loads the given file
   ArffTableModel(Instances)   - 
Constructor for class weka.gui.arffviewer. ArffTableModel 
 initializes the model with the given data
   ArffViewer   - Class in  weka.gui.arffviewer  A little tool for viewing ARFF files.   ArffViewer()   - 
Constructor for class weka.gui.arffviewer. ArffViewer 
 initializes the object
   ArffViewerMainPanel   - Class in  weka.gui.arffviewer  The main panel of the ArffViewer.   ArffViewerMainPanel(Container)   - 
Constructor for class weka.gui.arffviewer. ArffViewerMainPanel 
 initializes the object
   arrayLeftDivide(Matrix)   - 
Method in class weka.core.matrix. Matrix 
 Element-by-element left division, C = A.\B
   arrayLeftDivideEquals(Matrix)   - 
Method in class weka.core.matrix. Matrix 
 Element-by-element left division in place, A = A.\B
   arrayRightDivide(Matrix)   - 
Method in class weka.core.matrix. Matrix 
 Element-by-element right division, C = A./B
   arrayRightDivideEquals(Matrix)   - 
Method in class weka.core.matrix. Matrix 
 Element-by-element right division in place, A = A./B
   arrayTimes(Matrix)   - 
Method in class weka.core.matrix. Matrix 
 Element-by-element multiplication, C = A.*B
   arrayTimesEquals(Matrix)   - 
Method in class weka.core.matrix. Matrix 
 Element-by-element multiplication in place, A = A.*B
   arrayToString(Object)   - 
Static method in class weka.core. Utils 
 Returns the given Array in a string representation.
   arrayToString(Object[])   - 
Static method in class weka.experiment. DatabaseUtils 
 Converts an array of objects to a string by inserting a space
 between each element.
   artificialSizeTipText()   - 
Method in class weka.classifiers.meta. Decorate 
 Returns the tip text for this property
   ASEvaluation   - Class in  weka.attributeSelection  Abstract attribute selection evaluation class   ASEvaluation()   - 
Constructor for class weka.attributeSelection. ASEvaluation 
  
   ASSearch   - Class in  weka.attributeSelection  Abstract attribute selection search class.   ASSearch()   - 
Constructor for class weka.attributeSelection. ASSearch 
  
   assign(Capabilities)   - 
Method in class weka.core. Capabilities 
 retrieves the data from the given Capabilities object
   assign(TestInstances)   - 
Method in class weka.core. TestInstances 
 updates itself with all the settings from the given TestInstances
 object
   assign(Tester)   - 
Method in class weka.experiment. PairedTTester 
 retrieves all the settings from the given Tester
   assign(ResultMatrix)   - 
Method in class weka.experiment. ResultMatrix 
 acquires the data from the given matrix
   assign(Tester)   - 
Method in interface weka.experiment. Tester 
 retrieves all the settings from the given Tester
   assignIDs(int)   - 
Method in class weka.classifiers.trees.ft. FTtree 
 Assigns unique IDs to all nodes in the tree
   assignIDs(int)   - 
Method in class weka.classifiers.trees.j48. ClassifierTree 
 Assigns a uniqe id to every node in the tree.
   assignIDs(int)   - 
Method in class weka.classifiers.trees.lmt. LMTNode 
 Assigns unique IDs to all nodes in the tree
   assignLeafModelNumbers(int)   - 
Method in class weka.classifiers.trees.ft. FTtree 
 Assigns numbers to the logistic regression models at the leaves of the tree
   assignLeafModelNumbers(int)   - 
Method in class weka.classifiers.trees.lmt. LMTNode 
 Assigns numbers to the logistic regression models at the leaves of the tree
   assignSubToCenters(KDTreeNode, Instances, int[], int[])   - 
Method in class weka.core.neighboursearch. KDTree 
 Assigns instances of this node to center.
   associatedConnections(Vector)   - 
Static method in class weka.gui.beans. BeanConnection 
 Returns a vector of BeanConnections associated with
 the supplied vector of BeanInstances, i.e.
   AssociationsPanel   - Class in  weka.gui.explorer  This panel allows the user to select, configure, and run a scheme
 that learns associations.   AssociationsPanel()   - 
Constructor for class weka.gui.explorer. AssociationsPanel 
 Creates the associator panel
   Associator   - Interface in  weka.associations      Associator   - Class in  weka.gui.beans  Bean that wraps around weka.associations   Associator()   - 
Constructor for class weka.gui.beans. Associator 
 Creates a new  Associator  instance.
   AssociatorBeanInfo   - Class in  weka.gui.beans  BeanInfo class for the Associator wrapper bean   AssociatorBeanInfo()   - 
Constructor for class weka.gui.beans. AssociatorBeanInfo 
  
   AssociatorCustomizer   - Class in  weka.gui.beans  GUI customizer for the associator wrapper bean   AssociatorCustomizer()   - 
Constructor for class weka.gui.beans. AssociatorCustomizer 
  
   AssociatorEvaluation   - Class in  weka.associations  Class for evaluating Associaters.   AssociatorEvaluation()   - 
Constructor for class weka.associations. AssociatorEvaluation 
 default constructor
   associatorTipText()   - 
Method in class weka.associations. SingleAssociatorEnhancer 
 Returns the tip text for this property
   aSubsumesB(RuleItem, RuleItem)   - 
Static method in class weka.associations. CaRuleGeneration 
 Methods that decides whether or not rule a subsumes rule b.
   aSubsumesB(RuleItem, RuleItem)   - 
Static method in class weka.associations. RuleGeneration 
 Methods that decides whether or not rule a subsumes rule b.
   ATT_ARRAY   - 
Static variable in class weka.core.xml. XMLSerialization 
 the tag whether array or not (yes/no)
   ATT_ARRAY_DEFAULT   - 
Static variable in class weka.core.xml. XMLSerialization 
 default value for attribute ATT_ARRAY
   ATT_CLASS   - 
Static variable in class weka.core.xml. XMLInstances 
 the class attribute
   ATT_CLASS   - 
Static variable in class weka.core.xml. XMLSerialization 
 the tag for the class
   ATT_FORMAT   - 
Static variable in class weka.core.xml. XMLInstances 
 the format attribute (for date attributes)
   ATT_INDEX   - 
Static variable in class weka.core.xml. XMLInstances 
 the index attribute
   ATT_MISSING   - 
Static variable in class weka.core.xml. XMLInstances 
 the missing attribute
   ATT_NAME   - 
Static variable in class weka.core.xml. XMLDocument 
 the "name" attribute.
   ATT_NAME   - 
Static variable in class weka.core.xml. XMLOptions 
 the name attribute.
   ATT_NAME   - 
Static variable in class weka.core.xml. XMLSerialization 
 the tag for the name
   ATT_NULL   - 
Static variable in class weka.core.xml. XMLSerialization 
 the tag whether null or not (yes/no)
   ATT_NULL_DEFAULT   - 
Static variable in class weka.core.xml. XMLSerialization 
 default value for attribute ATT_NULL
   ATT_PRIMITIVE   - 
Static variable in class weka.core.xml. XMLSerialization 
 the tag whether primitive or not (yes/no)
   ATT_PRIMITIVE_DEFAULT   - 
Static variable in class weka.core.xml. XMLSerialization 
 default value for attribute ATT_PRIMITIVE
   ATT_TYPE   - 
Static variable in class weka.core.xml. XMLInstances 
 the type attribute
   ATT_TYPE   - 
Static variable in class weka.core.xml. XMLOptions 
 the type attribute.
   ATT_VALUE   - 
Static variable in class weka.core.xml. XMLOptions 
 the value attribute.
   ATT_VERSION   - 
Static variable in class weka.core.xml. XMLDocument 
 the "version" attribute.
   ATT_VERSION   - 
Static variable in class weka.core.xml. XMLInstances 
 the version attribute
   ATT_VERSION   - 
Static variable in class weka.core.xml. XMLSerialization 
 the version attribute
   ATT_WEIGHT   - 
Static variable in class weka.core.xml. XMLInstances 
 the weight attribute
   attIndex()   - 
Method in class weka.classifiers.trees.j48. BinC45Split 
 Returns index of attribute for which split was generated.
   attIndex()   - 
Method in class weka.classifiers.trees.j48. C45Split 
 Returns index of attribute for which split was generated.
   attIndex()   - 
Method in class weka.classifiers.trees.j48. NBTreeSplit 
 Returns index of attribute for which split was generated.
   attList_IrrTipText()   - 
Method in class weka.datagenerators.classifiers.classification. RDG1 
 Returns the tip text for this property
   attribute()   - 
Method in class weka.classifiers.trees.j48. GraftSplit 
  
   Attribute   - Class in  weka.core  Class for handling an attribute.   Attribute(String)   - 
Constructor for class weka.core. Attribute 
 Constructor for a numeric attribute.
   Attribute(String, ProtectedProperties)   - 
Constructor for class weka.core. Attribute 
 Constructor for a numeric attribute, where metadata is supplied.
   Attribute(String, String)   - 
Constructor for class weka.core. Attribute 
 Constructor for a date attribute.
   Attribute(String, String, ProtectedProperties)   - 
Constructor for class weka.core. Attribute 
 Constructor for a date attribute, where metadata is supplied.
   Attribute(String, FastVector)   - 
Constructor for class weka.core. Attribute 
 Constructor for nominal attributes and string attributes.
   Attribute(String, FastVector, ProtectedProperties)   - 
Constructor for class weka.core. Attribute 
 Constructor for nominal attributes and string attributes, where
 metadata is supplied.
   Attribute(String, Instances)   - 
Constructor for class weka.core. Attribute 
 Constructor for relation-valued attributes.
   Attribute(String, Instances, ProtectedProperties)   - 
Constructor for class weka.core. Attribute 
 Constructor for relation-valued attributes.
   Attribute(String, int)   - 
Constructor for class weka.core. Attribute 
 Constructor for a numeric attribute with a particular index.
   Attribute(String, String, int)   - 
Constructor for class weka.core. Attribute 
 Constructor for date attributes with a particular index.
   Attribute(String, FastVector, int)   - 
Constructor for class weka.core. Attribute 
 Constructor for nominal attributes and string attributes with
 a particular index.
   Attribute(String, Instances, int)   - 
Constructor for class weka.core. Attribute 
 Constructor for a relation-valued attribute with a particular index.
   attribute(int)   - 
Method in class weka.core. Instance 
 Returns the attribute with the given index.
   attribute(int)   - 
Method in class weka.core. Instances 
 Returns an attribute.
   attribute(String)   - 
Method in class weka.core. Instances 
 Returns an attribute given its name.
   ATTRIBUTE   - 
Static variable in interface weka.filters.unsupervised.instance.subsetbyexpression. sym 
  
   attributeAsClass()   - 
Method in class weka.gui.arffviewer. ArffPanel 
 sets the current attribute as class attribute, i.e.
   attributeAsClass()   - 
Method in class weka.gui.arffviewer. ArffViewerMainPanel 
 sets the current selected Attribute as class attribute, i.e.
   attributeAsClassAt(int)   - 
Method in class weka.gui.arffviewer. ArffSortedTableModel 
 sets the attribute at the given col index as the new class attribute
   attributeAsClassAt(int)   - 
Method in class weka.gui.arffviewer. ArffTableModel 
 sets the attribute at the given col index as the new class attribute, i.e.
   AttributeEvaluator   - Interface in  weka.attributeSelection  Interface for classes that evaluate attributes individually.   attributeEvaluatorTipText()   - 
Method in class weka.attributeSelection. FilteredAttributeEval 
 Returns the tip text for this property
   attributeEvaluatorTipText()   - 
Method in class weka.attributeSelection. RaceSearch 
 Returns the tip text for this property
   attributeEvaluatorTipText()   - 
Method in class weka.attributeSelection. RankSearch 
 Returns the tip text for this property
   AttributeExpression   - Class in  weka.core  A general purpose class for parsing mathematical expressions
 involving attribute values.   AttributeExpression()   - 
Constructor for class weka.core. AttributeExpression 
  
   attributeIndexesTipText()   - 
Method in class weka.filters.unsupervised.attribute. NominalToString 
 Returns the tip text for this property
   attributeIndexTipText()   - 
Method in class weka.filters.unsupervised.attribute. Add 
 Returns the tip text for this property.
   attributeIndexTipText()   - 
Method in class weka.filters.unsupervised.attribute. AddNoise 
 Returns the tip text for this property
   attributeIndexTipText()   - 
Method in class weka.filters.unsupervised.attribute. AddValues 
 Returns the tip text for this property
   attributeIndexTipText()   - 
Method in class weka.filters.unsupervised.attribute. ChangeDateFormat 
  
   attributeIndexTipText()   - 
Method in class weka.filters.unsupervised.attribute. MakeIndicator 
  
   attributeIndexTipText()   - 
Method in class weka.filters.unsupervised.attribute. MergeTwoValues 
  
   attributeIndexTipText()   - 
Method in class weka.filters.unsupervised.attribute. SwapValues 
  
   attributeIndexTipText()   - 
Method in class weka.filters.unsupervised.instance. RemoveFrequentValues 
 Returns the tip text for this property
   attributeIndexTipText()   - 
Method in class weka.filters.unsupervised.instance. RemoveWithValues 
 Returns the tip text for this property
   attributeIndicesTipText()   - 
Method in class weka.core. NormalizableDistance 
 Returns the tip text for this property.
   attributeIndicesTipText()   - 
Method in class weka.filters.supervised.attribute. Discretize 
 Returns the tip text for this property
   attributeIndicesTipText()   - 
Method in class weka.filters.unsupervised.attribute. AbstractTimeSeries 
 Returns the tip text for this property
   attributeIndicesTipText()   - 
Method in class weka.filters.unsupervised.attribute. Copy 
 Returns the tip text for this property
   attributeIndicesTipText()   - 
Method in class weka.filters.unsupervised.attribute. Discretize 
 Returns the tip text for this property
   attributeIndicesTipText()   - 
Method in class weka.filters.unsupervised.attribute. FirstOrder 
 Returns the tip text for this property
   attributeIndicesTipText()   - 
Method in class weka.filters.unsupervised.attribute. InterquartileRange 
 Returns the tip text for this property
   attributeIndicesTipText()   - 
Method in class weka.filters.unsupervised.attribute. NominalToBinary 
 Returns the tip text for this property
   attributeIndicesTipText()   - 
Method in class weka.filters.unsupervised.attribute. NumericCleaner 
 Returns the tip text for this property
   attributeIndicesTipText()   - 
Method in class weka.filters.unsupervised.attribute. NumericToNominal 
 Returns the tip text for this property
   attributeIndicesTipText()   - 
Method in class weka.filters.unsupervised.attribute. NumericTransform 
 Returns the tip text for this property
   attributeIndicesTipText()   - 
Method in class weka.filters.unsupervised.attribute. RELAGGS 
 Returns the tip text for this property
   attributeIndicesTipText()   - 
Method in class weka.filters.unsupervised.attribute. Remove 
 Returns the tip text for this property
   attributeIndicesTipText()   - 
Method in class weka.filters.unsupervised.attribute. Reorder 
 Returns the tip text for this property
   attributeIndicesTipText()   - 
Method in class weka.filters.unsupervised.attribute. StringToWordVector 
 Returns the tip text for this property.
   attributeList(BitSet)   - 
Method in class weka.attributeSelection. ScatterSearchV1 
 converts a BitSet into a list of attribute indexes
   AttributeListPanel   - Class in  weka.gui  Creates a panel that displays the attributes contained in a set of
 instances, letting the user select a single attribute for inspection.   AttributeListPanel()   - 
Constructor for class weka.gui. AttributeListPanel 
 Creates the attribute selection panel with no initial instances.
   AttributeLocator   - Class in  weka.core  This class locates and records the indices of a certain type of attributes, 
 recursively in case of Relational attributes.   AttributeLocator(Instances, int)   - 
Constructor for class weka.core. AttributeLocator 
 Initializes the AttributeLocator with the given data for the specified
 type of attribute.
   AttributeLocator(Instances, int, int, int)   - 
Constructor for class weka.core. AttributeLocator 
 Initializes the AttributeLocator with the given data for the specified
 type of attribute.
   AttributeLocator(Instances, int, int[])   - 
Constructor for class weka.core. AttributeLocator 
 initializes the AttributeLocator with the given data for the specified
 type of attribute.
   attributeNamePrefixTipText()   - 
Method in class weka.filters.unsupervised.attribute. StringToWordVector 
 Returns the tip text for this property.
   attributeNames()   - 
Method in class weka.classifiers.functions. SMO 
 Returns the attribute names.
   attributeNames()   - 
Method in class weka.classifiers.mi. MISMO 
 Returns the attribute names.
   attributeNameTipText()   - 
Method in class weka.filters.unsupervised.attribute. Add 
 Returns the tip text for this property.
   attributeNameTipText()   - 
Method in class weka.filters.unsupervised.attribute. AddID 
 Returns the tip text for this property
   AttributePanel   - Class in  weka.gui.visualize  This panel displays one dimensional views of the attributes in a
 dataset.   AttributePanel()   - 
Constructor for class weka.gui.visualize. AttributePanel 
  
   AttributePanel(Color)   - 
Constructor for class weka.gui.visualize. AttributePanel 
 This constructs an attributePanel.
   AttributePanelEvent   - Class in  weka.gui.visualize  Class encapsulating a change in the AttributePanel's selected x and y
 attributes.   AttributePanelEvent(boolean, boolean, int)   - 
Constructor for class weka.gui.visualize. AttributePanelEvent 
 Constructor
   AttributePanelListener   - Interface in  weka.gui.visualize  Interface for classes that want to listen for Attribute selection
 changes in the attribute panel   attributeRangeTipText()   - 
Method in class weka.filters.unsupervised.attribute. StringToNominal 
  
   AttributeSelectedClassifier   - Class in  weka.classifiers.meta  Dimensionality of training and test data is reduced by attribute selection before being passed on to a classifier.   AttributeSelectedClassifier()   - 
Constructor for class weka.classifiers.meta. AttributeSelectedClassifier 
 Default constructor.
   AttributeSelection   - Class in  weka.attributeSelection  Attribute selection class.   AttributeSelection()   - 
Constructor for class weka.attributeSelection. AttributeSelection 
 constructor.
   AttributeSelection   - Class in  weka.filters.supervised.attribute  A supervised attribute filter that can be used to select attributes.   AttributeSelection()   - 
Constructor for class weka.filters.supervised.attribute. AttributeSelection 
 Constructor
   attributeSelectionChange(AttributePanelEvent)   - 
Method in interface weka.gui.visualize. AttributePanelListener 
 Called when the user clicks on an attribute bar
   attributeSelectionMethodTipText()   - 
Method in class weka.classifiers.functions. LinearRegression 
 Returns the tip text for this property
   AttributeSelectionPanel   - Class in  weka.gui  Creates a panel that displays the attributes contained in a set of
 instances, letting the user toggle whether each attribute is selected
 or not (eg: so that unselected attributes can be removed before
 classification).   AttributeSelectionPanel()   - 
Constructor for class weka.gui. AttributeSelectionPanel 
 Creates the attribute selection panel with no initial instances.
   AttributeSelectionPanel(boolean, boolean, boolean, boolean)   - 
Constructor for class weka.gui. AttributeSelectionPanel 
 Creates the attribute selection panel with no initial instances.
   AttributeSelectionPanel   - Class in  weka.gui.explorer  This panel allows the user to select and configure an attribute
 evaluator and a search method, set the
 attribute of the current dataset to be used as the class, and perform
 attribute selection using one of two  selection modes (select using all the
 training data or perform a n-fold cross validation---on each trial
 selecting features using n-1 folds of the data).   AttributeSelectionPanel()   - 
Constructor for class weka.gui.explorer. AttributeSelectionPanel 
 Creates the classifier panel
   AttributeSetEvaluator   - Class in  weka.attributeSelection  Abstract attribute set evaluator.   AttributeSetEvaluator()   - 
Constructor for class weka.attributeSelection. AttributeSetEvaluator 
  
   attributeSparse(int)   - 
Method in class weka.core. Instance 
 Returns the attribute with the given index.
   attributeSparse(int)   - 
Method in class weka.core. SparseInstance 
 Returns the attribute associated with the internal index.
   AttributeStats   - Class in  weka.core  A Utility class that contains summary information on an
 the values that appear in a dataset for a particular attribute.   AttributeStats()   - 
Constructor for class weka.core. AttributeStats 
  
   attributeStats(int)   - 
Method in class weka.core. Instances 
 Calculates summary statistics on the values that appear in this
 set of instances for a specified attribute.
   attributesToString()   - 
Method in class weka.datagenerators.clusterers. SubspaceClusterDefinition 
 Make a string from the attribues list.
   attributeString(Instances)   - 
Method in class weka.classifiers.trees.adtree. Splitter 
 Gets the string describing the attributes the split depends on.
   attributeString(Instances)   - 
Method in class weka.classifiers.trees.adtree. TwoWayNominalSplit 
 Gets the string describing the attributes the split depends on.
   attributeString(Instances)   - 
Method in class weka.classifiers.trees.adtree. TwoWayNumericSplit 
 Gets the string describing the attributes the split depends on.
   AttributeSummarizer   - Class in  weka.gui.beans  Bean that encapsulates displays bar graph summaries for attributes in
 a data set.   AttributeSummarizer()   - 
Constructor for class weka.gui.beans. AttributeSummarizer 
 Creates a new  AttributeSummarizer  instance.
   AttributeSummarizerBeanInfo   - Class in  weka.gui.beans  Bean info class for the attribute summarizer bean   AttributeSummarizerBeanInfo()   - 
Constructor for class weka.gui.beans. AttributeSummarizerBeanInfo 
  
   AttributeSummaryPanel   - Class in  weka.gui  This panel displays summary statistics about an attribute: name, type
 number/% of missing/unique values, number of distinct values.   AttributeSummaryPanel()   - 
Constructor for class weka.gui. AttributeSummaryPanel 
 Creates the instances panel with no initial instances.
   attributeToDoubleArray(int)   - 
Method in class weka.core. Instances 
 Gets the value of all instances in this dataset for a particular
 attribute.
   AttributeTransformer   - Interface in  weka.attributeSelection  Abstract attribute transformer.   attributeTypeTipText()   - 
Method in class weka.filters.unsupervised.attribute. Add 
 Returns the tip text for this property
   attributeTypeTipText()   - 
Method in class weka.filters.unsupervised.attribute. RemoveType 
 Returns the tip text for this property
   attributeTypeToString(int)   - 
Static method in class weka.core. CheckScheme 
 returns a string representation of the attribute type
   AttributeValueLiteral   - Class in  weka.associations.tertius      AttributeValueLiteral(Predicate, String, int, int, int)   - 
Constructor for class weka.associations.tertius. AttributeValueLiteral 
  
   AttributeVisualizationPanel   - Class in  weka.gui  Creates a panel that shows a visualization of an
 attribute in a dataset.   AttributeVisualizationPanel()   - 
Constructor for class weka.gui. AttributeVisualizationPanel 
 Constructor - If used then the class will not show the class selection
 combo box.
   AttributeVisualizationPanel(boolean)   - 
Constructor for class weka.gui. AttributeVisualizationPanel 
 Constructor.
   attrIndexRangeTipText()   - 
Method in class weka.datagenerators.clusterers. SubspaceClusterDefinition 
 Returns the tip text for this property
   attrSplit(int, Instances)   - 
Method in class weka.classifiers.trees.m5. CorrelationSplitInfo 
 Finds the best splitting point for an attribute in the instances
   attrSplit(int, Instances)   - 
Method in interface weka.classifiers.trees.m5. SplitEvaluate 
 Finds the best splitting point for an attribute in the instances
   attrSplit(int, Instances)   - 
Method in class weka.classifiers.trees.m5. YongSplitInfo 
 Finds the best splitting point for an attribute in the instances
   attsToEliminatePerIterationTipText()   - 
Method in class weka.attributeSelection. SVMAttributeEval 
 Returns a tip text for this property suitable for display in the
 GUI
   autoBuildTipText()   - 
Method in class weka.classifiers.functions. MultilayerPerceptron 
  
   autoKeyGenerationTipText()   - 
Method in class weka.core.converters. DatabaseSaver 
 Returns the tip text for this property.
   AVERAGE_RULE   - 
Static variable in class weka.classifiers.meta. Vote 
 combination rule: Average of Probabilities
   AveragingResultProducer   - Class in  weka.experiment  Takes the results from a ResultProducer and submits the average to the result listener.   AveragingResultProducer()   - 
Constructor for class weka.experiment. AveragingResultProducer 
  
   avgCost()   - 
Method in class weka.classifiers. Evaluation 
 Gets the average cost, that is, total cost of misclassifications
 (incorrect plus unclassified) over the total number of instances.
   avgProb   - 
Variable in class weka.classifiers.lazy.kstar. KStarWrapper 
 used/reused to hold the average transformation probability
 
 
    
 B  
 
   B_ENTROPY   - 
Static variable in interface weka.classifiers.lazy.kstar. KStarConstants 
  
   B_SPHERE   - 
Static variable in interface weka.classifiers.lazy.kstar. KStarConstants 
 Blend setting modes
   backfitData(Instances)   - 
Method in class weka.classifiers.trees. RandomTree 
 Backfits the given data into the tree.
   backQuoteChars(String)   - 
Static method in class weka.core. Utils 
 Converts carriage returns and new lines in a string into \r and \n.
   backward(PaceMatrix, IntVector, int, int)   - 
Method in class weka.classifiers.functions.pace. PaceMatrix 
 Backward ordering of columns in terms of response explanation.
   Bagging   - Class in  weka.classifiers.meta  Class for bagging a classifier to reduce variance.   Bagging()   - 
Constructor for class weka.classifiers.meta. Bagging 
 Constructor.
   bagSizePercentTipText()   - 
Method in class weka.classifiers.meta. Bagging 
 Returns the tip text for this property
   bagSizePercentTipText()   - 
Method in class weka.classifiers.meta. MetaCost 
 Returns the tip text for this property
   balanceClassTipText()   - 
Method in class weka.datagenerators.classifiers.classification. Agrawal 
 Returns the tip text for this property
   balancedTipText()   - 
Method in class weka.classifiers.functions. Winnow 
 Returns the tip text for this property
   BallNode   - Class in  weka.core.neighboursearch.balltrees  Class representing a node of a BallTree.   BallNode(int)   - 
Constructor for class weka.core.neighboursearch.balltrees. BallNode 
 Constructor.
   BallNode(int, int, int)   - 
Constructor for class weka.core.neighboursearch.balltrees. BallNode 
 Creates a new instance of BallNode.
   BallNode(int, int, int, Instance, double)   - 
Constructor for class weka.core.neighboursearch.balltrees. BallNode 
 Creates a new instance of BallNode.
   BallSplitter   - Class in  weka.core.neighboursearch.balltrees  Abstract class for splitting a ball tree's BallNode.   BallSplitter()   - 
Constructor for class weka.core.neighboursearch.balltrees. BallSplitter 
 default constructor.
   BallSplitter(int[], Instances, EuclideanDistance)   - 
Constructor for class weka.core.neighboursearch.balltrees. BallSplitter 
 Creates a new instance of BallSplitter.
   ballSplitterTipText()   - 
Method in class weka.core.neighboursearch.balltrees. TopDownConstructor 
 Returns the tip text for this property.
   BallTree   - Class in  weka.core.neighboursearch  Class implementing the BallTree/Metric Tree algorithm for nearest neighbour search. 
 The connection to dataset is only a reference.   BallTree()   - 
Constructor for class weka.core.neighboursearch. BallTree 
 Creates a new instance of BallTree.
   BallTree(Instances)   - 
Constructor for class weka.core.neighboursearch. BallTree 
 Creates a new instance of BallTree.
   BallTreeConstructor   - Class in  weka.core.neighboursearch.balltrees  Abstract class for constructing a BallTree .   BallTreeConstructor()   - 
Constructor for class weka.core.neighboursearch.balltrees. BallTreeConstructor 
 Creates a new instance of BallTreeConstructor.
   ballTreeConstructorTipText()   - 
Method in class weka.core.neighboursearch. BallTree 
 Returns the tip text for this property.
   baseTipText()   - 
Method in class weka.core.neighboursearch. CoverTree 
 Returns the tip text for this property.
   BATCH   - 
Static variable in interface weka.core.converters. Loader 
  
   BATCH   - 
Static variable in interface weka.core.converters. Saver 
  
   BATCH_FINISHED   - 
Static variable in class weka.gui.beans. IncrementalClassifierEvent 
  
   BATCH_FINISHED   - 
Static variable in class weka.gui.beans. InstanceEvent 
  
   BATCH_FINISHED   - 
Static variable in class weka.gui.streams. InstanceEvent 
 Specifies that the batch of instances is finished
   BatchClassifierEvent   - Class in  weka.gui.beans  Class encapsulating a built classifier and a batch of instances to
 test on.   BatchClassifierEvent(Object, Classifier, DataSetEvent, DataSetEvent, int, int)   - 
Constructor for class weka.gui.beans. BatchClassifierEvent 
 Creates a new  BatchClassifierEvent  instance.
   BatchClassifierEvent(Object, Classifier, DataSetEvent, DataSetEvent, int, int, int, int)   - 
Constructor for class weka.gui.beans. BatchClassifierEvent 
 Creates a new  BatchClassifierEvent  instance.
   BatchClassifierListener   - Interface in  weka.gui.beans  Interface to something that can process a BatchClassifierEvent   BatchClustererEvent   - Class in  weka.gui.beans  Class encapsulating a built clusterer and a batch of instances to
 test on.   BatchClustererEvent(Object, Clusterer, DataSetEvent, int, int, int)   - 
Constructor for class weka.gui.beans. BatchClustererEvent 
 Creates a new  BatchClustererEvent  instance.
   BatchClustererListener   - Interface in  weka.gui.beans  Interface to something that can process a BatchClustererEvent   BatchConverter   - Interface in  weka.core.converters  Marker interface for a loader/saver that can retrieve instances in batch mode   batchFilterFile(Filter, String[])   - 
Static method in class weka.filters. Filter 
 Method for testing filters ability to process multiple batches.
   batchFinished()   - 
Method in class weka.filters. Filter 
 Signify that this batch of input to the filter is finished.
   batchFinished()   - 
Method in class weka.filters. MultiFilter 
 Signify that this batch of input to the filter is finished.
   batchFinished()   - 
Method in class weka.filters. SimpleBatchFilter 
 Signify that this batch of input to the filter is finished.
   batchFinished()   - 
Method in class weka.filters. SimpleStreamFilter 
 Signify that this batch of input to the filter is finished.
   batchFinished()   - 
Method in class weka.filters.supervised.attribute. AttributeSelection 
 Signify that this batch of input to the filter is finished.
   batchFinished()   - 
Method in class weka.filters.supervised.attribute. ClassOrder 
 Signify that this batch of input to the filter is finished.
   batchFinished()   - 
Method in class weka.filters.supervised.attribute. Discretize 
 Signifies that this batch of input to the filter is finished.
   batchFinished()   - 
Method in class weka.filters.supervised.attribute. NominalToBinary 
 Signify that this batch of input to the filter is finished.
   batchFinished()   - 
Method in class weka.filters.supervised.instance. Resample 
 Signify that this batch of input to the filter is finished.
   batchFinished()   - 
Method in class weka.filters.supervised.instance. SMOTE 
 Signify that this batch of input to the filter is finished.
   batchFinished()   - 
Method in class weka.filters.supervised.instance. SpreadSubsample 
 Signify that this batch of input to the filter is finished.
   batchFinished()   - 
Method in class weka.filters.supervised.instance. StratifiedRemoveFolds 
 Signify that this batch of input to the filter is
 finished.
   batchFinished()   - 
Method in class weka.filters.unsupervised.attribute. AbstractTimeSeries 
 Signifies that this batch of input to the filter is finished.
   batchFinished()   - 
Method in class weka.filters.unsupervised.attribute. AddCluster 
 Signify that this batch of input to the filter is finished.
   batchFinished()   - 
Method in class weka.filters.unsupervised.attribute. AddID 
 Signify that this batch of input to the filter is finished.
   batchFinished()   - 
Method in class weka.filters.unsupervised.attribute. AddNoise 
 Signify that this batch of input to the filter is finished.
   batchFinished()   - 
Method in class weka.filters.unsupervised.attribute. Center 
 Signify that this batch of input to the filter is finished.
   batchFinished()   - 
Method in class weka.filters.unsupervised.attribute. ClusterMembership 
 Signify that this batch of input to the filter is finished.
   batchFinished()   - 
Method in class weka.filters.unsupervised.attribute. Discretize 
 Signifies that this batch of input to the filter is finished.
   batchFinished()   - 
Method in class weka.filters.unsupervised.attribute. MathExpression 
 Signify that this batch of input to the filter is finished.
   batchFinished()   - 
Method in class weka.filters.unsupervised.attribute. MultiInstanceToPropositional 
 Signify that this batch of input to the filter is finished.
   batchFinished()   - 
Method in class weka.filters.unsupervised.attribute. NominalToString 
 Signifies that this batch of input to the filter is finished.
   batchFinished()   - 
Method in class weka.filters.unsupervised.attribute. Normalize 
 Signify that this batch of input to the filter is finished.
   batchFinished()   - 
Method in class weka.filters.unsupervised.attribute. PrincipalComponents 
 Signify that this batch of input to the filter is finished.
   batchFinished()   - 
Method in class weka.filters.unsupervised.attribute. PropositionalToMultiInstance 
 Signify that this batch of input to the filter is finished.
   batchFinished()   - 
Method in class weka.filters.unsupervised.attribute. RandomProjection 
 Signify that this batch of input to the filter is finished.
   batchFinished()   - 
Method in class weka.filters.unsupervised.attribute. RemoveType 
 Signify that this batch of input to the filter is finished.
   batchFinished()   - 
Method in class weka.filters.unsupervised.attribute. RemoveUseless 
 Signify that this batch of input to the filter is finished.
   batchFinished()   - 
Method in class weka.filters.unsupervised.attribute. ReplaceMissingValues 
 Signify that this batch of input to the filter is finished.
   batchFinished()   - 
Method in class weka.filters.unsupervised.attribute. Standardize 
 Signify that this batch of input to the filter is finished.
   batchFinished()   - 
Method in class weka.filters.unsupervised.attribute. StringToNominal 
 Signifies that this batch of input to the filter is finished.
   batchFinished()   - 
Method in class weka.filters.unsupervised.attribute. StringToWordVector 
 Signify that this batch of input to the filter is finished.
   batchFinished()   - 
Method in class weka.filters.unsupervised.instance. Randomize 
 Signify that this batch of input to the filter is finished.
   batchFinished()   - 
Method in class weka.filters.unsupervised.instance. RemoveFolds 
 Signify that this batch of input to the filter is
 finished.
   batchFinished()   - 
Method in class weka.filters.unsupervised.instance. RemoveFrequentValues 
 Signifies that this batch of input to the filter is finished.
   batchFinished()   - 
Method in class weka.filters.unsupervised.instance. RemoveMisclassified 
 Signify that this batch of input to the filter is finished.
   batchFinished()   - 
Method in class weka.filters.unsupervised.instance. RemovePercentage 
 Signify that this batch of input to the filter is
 finished.
   batchFinished()   - 
Method in class weka.filters.unsupervised.instance. RemoveRange 
 Signify that this batch of input to the filter is
 finished.
   batchFinished()   - 
Method in class weka.filters.unsupervised.instance. Resample 
 Signify that this batch of input to the filter is finished.
   batchFinished()   - 
Method in class weka.filters.unsupervised.instance. ReservoirSample 
 Signify that this batch of input to the filter is finished.
   batchFinished()   - 
Method in class weka.gui.streams. InstanceJoiner 
 Signify that this batch of input to the filter is finished.
   batchFinished()   - 
Method in class weka.gui.streams. InstanceSavePanel 
  
   batchFinished()   - 
Method in class weka.gui.streams. InstanceTable 
  
   batchFinished()   - 
Method in class weka.gui.streams. InstanceViewer 
  
   BAYES   - 
Static variable in interface weka.classifiers.bayes.net.search.local. Scoreable 
 score types
   BayesianLogisticRegression   - Class in  weka.classifiers.bayes  Implements Bayesian Logistic Regression for both Gaussian and Laplace Priors. 
  
 For more information, see 
  
 Alexander Genkin, David D.   BayesianLogisticRegression()   - 
Constructor for class weka.classifiers.bayes. BayesianLogisticRegression 
  
   BayesNet   - Class in  weka.classifiers.bayes  Bayes Network learning using various search algorithms and quality measures. 
 Base class for a Bayes Network classifier.   BayesNet()   - 
Constructor for class weka.classifiers.bayes. BayesNet 
  
   BayesNet   - 
Static variable in interface weka.core. Drawable 
  
   BayesNet   - Class in  weka.datagenerators.classifiers.classification  Generates random instances based on a Bayes network.   BayesNet()   - 
Constructor for class weka.datagenerators.classifiers.classification. BayesNet 
 initializes the generator
   BayesNetEstimator   - Class in  weka.classifiers.bayes.net.estimate  BayesNetEstimator is the base class for estimating the conditional probability tables of a Bayes network once the structure has been learned.   BayesNetEstimator()   - 
Constructor for class weka.classifiers.bayes.net.estimate. BayesNetEstimator 
  
   BayesNetGenerator   - Class in  weka.classifiers.bayes.net  Bayes Network learning using various search algorithms and quality measures. 
 Base class for a Bayes Network classifier.   BayesNetGenerator()   - 
Constructor for class weka.classifiers.bayes.net. BayesNetGenerator 
 Constructor for BayesNetGenerator.
   BDeu   - 
Static variable in interface weka.classifiers.bayes.net.search.local. Scoreable 
  
   BEAN_EXECUTING   - 
Static variable in class weka.gui.beans. BeanInstance 
  
   BeanCommon   - Interface in  weka.gui.beans  Interface specifying routines that all weka beans should implement
 in order to allow the bean environment to exercise some control over the
 bean and also to allow the bean to exercise some control over connections.   BeanConnection   - Class in  weka.gui.beans  Class for encapsulating a connection between two beans.   BeanConnection(BeanInstance, BeanInstance, EventSetDescriptor)   - 
Constructor for class weka.gui.beans. BeanConnection 
 Creates a new  BeanConnection  instance.
   BeanInstance   - Class in  weka.gui.beans  Class that manages a set of beans.   BeanInstance(JComponent, Object, int, int)   - 
Constructor for class weka.gui.beans. BeanInstance 
 Creates a new  BeanInstance  instance.
   BeanInstance(JComponent, String, int, int)   - 
Constructor for class weka.gui.beans. BeanInstance 
 Creates a new  BeanInstance  instance given the fully
 qualified name of the bean
   BeanVisual   - Class in  weka.gui.beans  BeanVisual encapsulates icons and label for a given bean.   BeanVisual(String, String, String)   - 
Constructor for class weka.gui.beans. BeanVisual 
 Constructor
   BestFirst   - Class in  weka.attributeSelection  BestFirst: 
  
 Searches the space of attribute subsets by greedy hillclimbing augmented with a backtracking facility.   BestFirst()   - 
Constructor for class weka.attributeSelection. BestFirst 
 Constructor
   BestFirst.Link2   - Class in  weka.attributeSelection  Class for a node in a linked list.   BestFirst.Link2(Object[], double)   - 
Constructor for class weka.attributeSelection. BestFirst.Link2 
 Constructor
   BestFirst.LinkedList2   - Class in  weka.attributeSelection  Class for handling a linked list.   BestFirst.LinkedList2(int)   - 
Constructor for class weka.attributeSelection. BestFirst.LinkedList2 
  
   betaTipText()   - 
Method in class weka.classifiers.functions. Winnow 
 Returns the tip text for this property
   BetaVector   - 
Variable in class weka.classifiers.bayes. BayesianLogisticRegression 
 Array for storing coefficients of Bayesian regression model.
   BFTree   - Class in  weka.classifiers.trees  Class for building a best-first decision tree classifier.   BFTree()   - 
Constructor for class weka.classifiers.trees. BFTree 
  
   bias()   - 
Method in class weka.classifiers.functions. SMO 
 Returns the bias of each binary SMO.
   bias()   - 
Method in class weka.classifiers.mi. MISMO 
 Returns the bias of each binary SMO.
   biasTipText()   - 
Method in class weka.classifiers.functions. LibLINEAR 
 Returns the tip text for this property
   biasTipText()   - 
Method in class weka.classifiers.misc. VFI 
 Returns the tip text for this property
   biasToUniformClassTipText()   - 
Method in class weka.filters.supervised.instance. Resample 
 Returns the tip text for this property.
   BIBTEX_ENDTAG   - 
Static variable in class weka.core. TechnicalInformationHandlerJavadoc 
 the end comment tag for inserting the generated BibTex
   BIBTEX_STARTTAG   - 
Static variable in class weka.core. TechnicalInformationHandlerJavadoc 
 the start comment tag for inserting the generated BibTex
   BIFFileTipText()   - 
Method in class weka.classifiers.bayes. BayesNet 
  
   BIFFormatException   - Exception in  weka.gui.graphvisualizer  This is the Exception thrown by BIFParser, if there
 was an error in parsing the XMLBIF string or input
 stream.   BIFFormatException(String)   - 
Constructor for exception weka.gui.graphvisualizer. BIFFormatException 
  
   BIFParser   - Class in  weka.gui.graphvisualizer  This class parses an inputstream or a string in
 XMLBIF ver.   BIFParser(String, FastVector, FastVector)   - 
Constructor for class weka.gui.graphvisualizer. BIFParser 
 Constructor (if our input is a String)
   BIFParser(InputStream, FastVector, FastVector)   - 
Constructor for class weka.gui.graphvisualizer. BIFParser 
 Constructor (if our input is an InputStream)
   BIFReader   - Class in  weka.classifiers.bayes.net  Builds a description of a Bayes Net classifier stored in XML BIF 0.3 format. 
  
 For more details on XML BIF see: 
  
 Fabio Cozman, Marek Druzdzel, Daniel Garcia (1998).   BIFReader()   - 
Constructor for class weka.classifiers.bayes.net. BIFReader 
 the default constructor
   bigF(double, double)   - 
Static method in class weka.classifiers.bayes. BayesianLogisticRegression 
 This is a convient function that defines and upper bound
 (Delta>0) for values of r(i) reachable by updates in the
 trust region.
   binarizeNumericAttributesTipText()   - 
Method in class weka.attributeSelection. ChiSquaredAttributeEval 
 Returns the tip text for this property
   binarizeNumericAttributesTipText()   - 
Method in class weka.attributeSelection. InfoGainAttributeEval 
 Returns the tip text for this property
   BINARY   - 
Static variable in class weka.gui.beans. SerializedModelSaver 
  
   binaryAttributesNominalTipText()   - 
Method in class weka.filters.supervised.attribute. NominalToBinary 
 Returns the tip text for this property
   binaryAttributesNominalTipText()   - 
Method in class weka.filters.unsupervised.attribute. NominalToBinary 
 Returns the tip text for this property
   BinarySparseInstance   - Class in  weka.core  Class for storing a binary-data-only instance as a sparse vector.   BinarySparseInstance(Instance)   - 
Constructor for class weka.core. BinarySparseInstance 
 Constructor that generates a sparse instance from the given
 instance.
   BinarySparseInstance(SparseInstance)   - 
Constructor for class weka.core. BinarySparseInstance 
 Constructor that copies the info from the given instance.
   BinarySparseInstance(double, double[])   - 
Constructor for class weka.core. BinarySparseInstance 
 Constructor that generates a sparse instance from the given
 parameters.
   BinarySparseInstance(double, int[], int)   - 
Constructor for class weka.core. BinarySparseInstance 
 Constructor that inititalizes instance variable with given
 values.
   BinarySparseInstance(int)   - 
Constructor for class weka.core. BinarySparseInstance 
 Constructor of an instance that sets weight to one, all values to
 1, and the reference to the dataset to null.
   binarySplitsTipText()   - 
Method in class weka.classifiers.rules. PART 
 Returns the tip text for this property
   binarySplitsTipText()   - 
Method in class weka.classifiers.trees. J48 
 Returns the tip text for this property
   binarySplitsTipText()   - 
Method in class weka.classifiers.trees. J48graft 
 Returns the tip text for this property
   binaryToKOML(String, String)   - 
Static method in class weka.core.xml. SerialUIDChanger 
 converts a binary file into a KOML XML file
   BinC45ModelSelection   - Class in  weka.classifiers.trees.j48  Class for selecting a C4.5-like binary (!) split for a given dataset.   BinC45ModelSelection(int, Instances)   - 
Constructor for class weka.classifiers.trees.j48. BinC45ModelSelection 
 Initializes the split selection method with the given parameters.
   BinC45Split   - Class in  weka.classifiers.trees.j48  Class implementing a binary C4.5-like split on an attribute.   BinC45Split(int, int, double)   - 
Constructor for class weka.classifiers.trees.j48. BinC45Split 
 Initializes the split model.
   binomialDistribution(double, double, double)   - 
Static method in class weka.associations. RuleGeneration 
 calculates the probability using a binomial distribution.
   binomialStandardError(double, int)   - 
Static method in class weka.core. Statistics 
 Computes standard error for observed values of a binomial
 random variable.
   binomP(double, double, double)   - 
Method in class weka.classifiers.lazy. LBR 
 Significance test
 binomp:
   binSplitTipText()   - 
Method in class weka.classifiers.trees. FT 
 Returns the tip text for this property
   binsTipText()   - 
Method in class weka.filters.unsupervised.attribute. Discretize 
 Returns the tip text for this property
   binsTipText()   - 
Method in class weka.filters.unsupervised.attribute. PKIDiscretize 
 Returns the tip text for this property
   binValueTipText()   - 
Method in class weka.clusterers. XMeans 
 Returns the tip text for this property.
   biprob(double, double, double)   - 
Method in class weka.classifiers.trees.j48. C45PruneableClassifierTreeG 
 Significance test
   BIRCHCluster   - Class in  weka.datagenerators.clusterers  Cluster data generator designed for the BIRCH System 
  
 Dataset is generated with instances in K clusters. 
 Instances are 2-d data points. 
 Each cluster is characterized by the number of data points in itits radius and its center.   BIRCHCluster()   - 
Constructor for class weka.datagenerators.clusterers. BIRCHCluster 
 initializes the generator with default values
   blocker(boolean)   - 
Method in class weka.classifiers.functions. MultilayerPerceptron 
 A function used to stop the code that called buildclassifier
 from continuing on before the user has finished the decision tree.
   BMAEstimator   - Class in  weka.classifiers.bayes.net.estimate  BMAEstimator estimates conditional probability tables of a Bayes network using Bayes Model Averaging (BMA).   BMAEstimator()   - 
Constructor for class weka.classifiers.bayes.net.estimate. BMAEstimator 
  
   BMPWriter   - Class in  weka.gui.visualize  This class takes any JComponent and outputs it to a BMP-file.   BMPWriter()   - 
Constructor for class weka.gui.visualize. BMPWriter 
 initializes the object
   BMPWriter(JComponent)   - 
Constructor for class weka.gui.visualize. BMPWriter 
 initializes the object with the given Component
   BMPWriter(JComponent, File)   - 
Constructor for class weka.gui.visualize. BMPWriter 
 initializes the object with the given Component and filename
   Body   - Class in  weka.associations.tertius  Class representing the body of a rule.   Body()   - 
Constructor for class weka.associations.tertius. Body 
 Constructor without storing the counter-instances.
   Body(Instances)   - 
Constructor for class weka.associations.tertius. Body 
 Constructor storing the counter-instances.
   bodyContains(Literal)   - 
Method in class weka.associations.tertius. Rule 
 Test if the body of the rule contains a literal.
   BOOL   - 
Static variable in class weka.experiment. DatabaseUtils 
 Type mapping for BOOL used for reading experiment results.
   BOOLEAN   - 
Static variable in interface weka.core.mathematicalexpression. sym 
  
   BOOLEAN   - 
Static variable in interface weka.filters.unsupervised.instance.subsetbyexpression. sym 
  
   booleanColsTipText()   - 
Method in class weka.datagenerators. ClusterGenerator 
 Returns the tip text for this property
   boost()   - 
Method in class weka.classifiers.trees. ADTree 
 Performs a single boosting iteration, using two-class optimized method.
   BottomUpConstructor   - Class in  weka.core.neighboursearch.balltrees  The class that constructs a ball tree bottom up.   BottomUpConstructor()   - 
Constructor for class weka.core.neighboursearch.balltrees. BottomUpConstructor 
 Creates a new instance of BottomUpConstructor.
   BoundaryPanel   - Class in  weka.gui.boundaryvisualizer  BoundaryPanel.   BoundaryPanel(int, int)   - 
Constructor for class weka.gui.boundaryvisualizer. BoundaryPanel 
 Creates a new  BoundaryPanel  instance.
   BoundaryPanelDistributed   - Class in  weka.gui.boundaryvisualizer  This class extends BoundaryPanel with code for distributing the
 processing necessary to create a visualization among a list of
 remote machines.   BoundaryPanelDistributed(int, int)   - 
Constructor for class weka.gui.boundaryvisualizer. BoundaryPanelDistributed 
 Creates a new  BoundaryPanelDistributed  instance.
   BoundaryVisualizer   - Class in  weka.gui.boundaryvisualizer  BoundaryVisualizer.   BoundaryVisualizer()   - 
Constructor for class weka.gui.boundaryvisualizer. BoundaryVisualizer 
 Creates a new  BoundaryVisualizer  instance.
   branchInstanceGoesDown(Instance)   - 
Method in class weka.classifiers.trees.adtree. Splitter 
 Gets the index of the branch that an instance applies to.
   branchInstanceGoesDown(Instance)   - 
Method in class weka.classifiers.trees.adtree. TwoWayNominalSplit 
 Gets the index of the branch that an instance applies to.
   branchInstanceGoesDown(Instance)   - 
Method in class weka.classifiers.trees.adtree. TwoWayNumericSplit 
 Gets the index of the branch that an instance applies to.
   BrowserHelper   - Class in  weka.gui  A little helper class for browser related stuff.   BrowserHelper()   - 
Constructor for class weka.gui. BrowserHelper 
  
   bubbleSubsetSort(List<ScatterSearchV1.Subset>)   - 
Method in class weka.attributeSelection. ScatterSearchV1 
 Sort a List of subsets according to their merits
   build(String, String)   - 
Method in class weka.gui. HierarchyPropertyParser 
 Build a tree from the given property with the given delimitor
   buildAssociations(Instances)   - 
Method in class weka.associations. Apriori 
 Method that generates all large itemsets with a minimum support, and from
 these all association rules with a minimum confidence.
   buildAssociations(Instances)   - 
Method in interface weka.associations. Associator 
 Generates an associator.
   buildAssociations(Instances)   - 
Method in class weka.associations. FilteredAssociator 
 Build the associator on the filtered data.
   buildAssociations(Instances)   - 
Method in class weka.associations. FPGrowth 
 Method that generates all large item sets with a minimum support, and from
 these all association rules with a minimum metric (i.e.
   buildAssociations(Instances)   - 
Method in class weka.associations. GeneralizedSequentialPatterns 
 Extracts all sequential patterns out of a given sequential data set and 
 prints out the results.
   buildAssociations(Instances)   - 
Method in class weka.associations. PredictiveApriori 
 Method that generates all large itemsets with a minimum support, and from
 these all association rules.
   buildAssociations(Instances)   - 
Method in class weka.associations. Tertius 
 Method that launches the search to find the rules with the highest 
 confirmation.
   buildClassifier(Instances)   - 
Method in class weka.classifiers.bayes. AODE 
 Generates the classifier.
   buildClassifier(Instances)   - 
Method in class weka.classifiers.bayes. AODEsr 
 Generates the classifier.
   buildClassifier(Instances)   - 
Method in class weka.classifiers.bayes. BayesianLogisticRegression 
 
         (1) Set the data to the class attribute m_Instances.
  (2)Call the method initialize() to initialize the values.
 
   buildClassifier(Instances)   - 
Method in class weka.classifiers.bayes. BayesNet 
 Generates the classifier.
   buildClassifier(Instances)   - 
Method in class weka.classifiers.bayes. ComplementNaiveBayes 
 Generates the classifier.
   buildClassifier(Instances)   - 
Method in class weka.classifiers.bayes. DMNBtext 
 Generates the classifier.
   buildClassifier(Instances)   - 
Method in class weka.classifiers.bayes. HNB 
 Generates the classifier.
   buildClassifier(Instances)   - 
Method in class weka.classifiers.bayes. NaiveBayes 
 Generates the classifier.
   buildClassifier(Instances)   - 
Method in class weka.classifiers.bayes. NaiveBayesMultinomial 
 Generates the classifier.
   buildClassifier(Instances)   - 
Method in class weka.classifiers.bayes. NaiveBayesMultinomialUpdateable 
 Generates the classifier.
   buildClassifier(Instances)   - 
Method in class weka.classifiers.bayes. NaiveBayesSimple 
 Generates the classifier.
   buildClassifier(Instances)   - 
Method in class weka.classifiers.bayes. WAODE 
 Generates the classifier.
   buildClassifier(Instances)   - 
Method in class weka.classifiers. Classifier 
 Generates a classifier.
   buildClassifier(Instances)   - 
Method in class weka.classifiers.functions. GaussianProcesses 
 Method for building the classifier.
   buildClassifier(Instances)   - 
Method in class weka.classifiers.functions. IsotonicRegression 
 Builds an isotonic regression model given the supplied training data.
   buildClassifier(Instances)   - 
Method in class weka.classifiers.functions. LeastMedSq 
 Build lms regression
   buildClassifier(Instances)   - 
Method in class weka.classifiers.functions. LibLINEAR 
 builds the classifier
   buildClassifier(Instances)   - 
Method in class weka.classifiers.functions. LibSVM 
 builds the classifier
   buildClassifier(Instances)   - 
Method in class weka.classifiers.functions. LinearRegression 
 Builds a regression model for the given data.
   buildClassifier(Instances)   - 
Method in class weka.classifiers.functions. Logistic 
 Builds the classifier
   buildClassifier(Instances)   - 
Method in class weka.classifiers.functions. MultilayerPerceptron 
 Call this function to build and train a neural network for the training
 data provided.
   buildClassifier(Instances)   - 
Method in class weka.classifiers.functions. PaceRegression 
 Builds a pace regression model for the given data.
   buildClassifier(Instances)   - 
Method in class weka.classifiers.functions. PLSClassifier 
 builds the classifier
   buildClassifier(Instances)   - 
Method in class weka.classifiers.functions. RBFNetwork 
 Builds the classifier
   buildClassifier(Instances)   - 
Method in class weka.classifiers.functions. SimpleLinearRegression 
 Builds a simple linear regression model given the supplied training data.
   buildClassifier(Instances)   - 
Method in class weka.classifiers.functions. SimpleLogistic 
 Builds the logistic regression using LogitBoost.
   buildClassifier(Instances)   - 
Method in class weka.classifiers.functions. SMO 
 Method for building the classifier.
   buildClassifier(Instances)   - 
Method in class weka.classifiers.functions. SMOreg 
 Method for building the classifier.
   buildClassifier(Instances)   - 
Method in class weka.classifiers.functions. SPegasos 
 Method for building the classifier.
   buildClassifier(Instances)   - 
Method in class weka.classifiers.functions.supportVector. RegOptimizer 
 learn SVM parameters from data.
   buildClassifier(Instances)   - 
Method in class weka.classifiers.functions.supportVector. RegSMO 
 learn SVM parameters from data using Smola's SMO algorithm.
   buildClassifier(Instances)   - 
Method in class weka.classifiers.functions.supportVector. RegSMOImproved 
 learn SVM parameters from data using Keerthi's SMO algorithm.
   buildClassifier(Instances)   - 
Method in class weka.classifiers.functions. VotedPerceptron 
 Builds the ensemble of perceptrons.
   buildClassifier(Instances)   - 
Method in class weka.classifiers.functions. Winnow 
 Builds the classifier
   buildClassifier(Instances)   - 
Method in class weka.classifiers. IteratedSingleClassifierEnhancer 
 Stump method for building the classifiers.
   buildClassifier(Instances)   - 
Method in class weka.classifiers.lazy. IB1 
 Generates the classifier.
   buildClassifier(Instances)   - 
Method in class weka.classifiers.lazy. IBk 
 Generates the classifier.
   buildClassifier(Instances)   - 
Method in class weka.classifiers.lazy. KStar 
 Generates the classifier.
   buildClassifier(Instances)   - 
Method in class weka.classifiers.lazy. LBR 
 For lazy learning, building classifier is only to prepare their inputs
 until classification time.
   buildClassifier(Instances)   - 
Method in class weka.classifiers.lazy. LWL 
 Generates the classifier.
   buildClassifier(Instances)   - 
Method in class weka.classifiers.meta. AdaBoostM1 
 Boosting method.
   buildClassifier(Instances)   - 
Method in class weka.classifiers.meta. AdditiveRegression 
 Build the classifier on the supplied data
   buildClassifier(Instances)   - 
Method in class weka.classifiers.meta. AttributeSelectedClassifier 
 Build the classifier on the dimensionally reduced data.
   buildClassifier(Instances)   - 
Method in class weka.classifiers.meta. Bagging 
 Bagging method.
   buildClassifier(Instances)   - 
Method in class weka.classifiers.meta. ClassificationViaClustering 
 builds the classifier
   buildClassifier(Instances)   - 
Method in class weka.classifiers.meta. ClassificationViaRegression 
 Builds the classifiers.
   buildClassifier(Instances)   - 
Method in class weka.classifiers.meta. CostSensitiveClassifier 
 Builds the model of the base learner.
   buildClassifier(Instances)   - 
Method in class weka.classifiers.meta. CVParameterSelection 
 Generates the classifier.
   buildClassifier(Instances)   - 
Method in class weka.classifiers.meta. Dagging 
 Bagging method.
   buildClassifier(Instances)   - 
Method in class weka.classifiers.meta. Decorate 
 Build Decorate classifier
   buildClassifier(Instances)   - 
Method in class weka.classifiers.meta. END 
 Builds the committee of randomizable classifiers.
   buildClassifier(Instances)   - 
Method in class weka.classifiers.meta. FilteredClassifier 
 Build the classifier on the filtered data.
   buildClassifier(Instances)   - 
Method in class weka.classifiers.meta. GridSearch 
 builds the classifier
   buildClassifier(Instances)   - 
Method in class weka.classifiers.meta. LogitBoost 
 Builds the boosted classifier
   buildClassifier(Instances)   - 
Method in class weka.classifiers.meta. MetaCost 
 Builds the model of the base learner.
   buildClassifier(Instances)   - 
Method in class weka.classifiers.meta. MultiBoostAB 
 Method for building this classifier.
   buildClassifier(Instances)   - 
Method in class weka.classifiers.meta. MultiClassClassifier 
 Builds the classifiers.
   buildClassifier(Instances)   - 
Method in class weka.classifiers.meta. MultiScheme 
 Buildclassifier selects a classifier from the set of classifiers
 by minimising error on the training data.
   buildClassifier(Instances)   - 
Method in class weka.classifiers.meta.nestedDichotomies. ClassBalancedND 
 Builds tree recursively.
   buildClassifier(Instances)   - 
Method in class weka.classifiers.meta.nestedDichotomies. DataNearBalancedND 
 Builds tree recursively.
   buildClassifier(Instances)   - 
Method in class weka.classifiers.meta.nestedDichotomies. ND 
 Builds the classifier.
   buildClassifier(Instances)   - 
Method in class weka.classifiers.meta. OrdinalClassClassifier 
 Builds the classifiers.
   buildClassifier(Instances)   - 
Method in class weka.classifiers.meta. RacedIncrementalLogitBoost 
 Builds the classifier.
   buildClassifier(Instances)   - 
Method in class weka.classifiers.meta. RandomCommittee 
 Builds the committee of randomizable classifiers.
   buildClassifier(Instances)   - 
Method in class weka.classifiers.meta. RandomSubSpace 
 builds the classifier.
   buildClassifier(Instances)   - 
Method in class weka.classifiers.meta. RegressionByDiscretization 
 Generates the classifier.
   buildClassifier(Instances)   - 
Method in class weka.classifiers.meta. RotationForest 
 builds the classifier.
   buildClassifier(Instances)   - 
Method in class weka.classifiers.meta. Stacking 
 Buildclassifier selects a classifier from the set of classifiers
 by minimising error on the training data.
   buildClassifier(Instances)   - 
Method in class weka.classifiers.meta. ThresholdSelector 
 Generates the classifier.
   buildClassifier(Instances)   - 
Method in class weka.classifiers.meta. Vote 
 Buildclassifier selects a classifier from the set of classifiers
 by minimising error on the training data.
   buildClassifier(Instances)   - 
Method in class weka.classifiers.mi. CitationKNN 
 Builds the classifier
   buildClassifier(Instances)   - 
Method in class weka.classifiers.mi. MDD 
 Builds the classifier
   buildClassifier(Instances)   - 
Method in class weka.classifiers.mi. MIBoost 
 Builds the classifier
   buildClassifier(Instances)   - 
Method in class weka.classifiers.mi. MIDD 
 Builds the classifier
   buildClassifier(Instances)   - 
Method in class weka.classifiers.mi. MIEMDD 
 Builds the classifier
   buildClassifier(Instances)   - 
Method in class weka.classifiers.mi. MILR 
 Builds the classifier
   buildClassifier(Instances)   - 
Method in class weka.classifiers.mi. MINND 
 As normal Nearest Neighbour algorithm does, it's lazy and simply
 records the exemplar information (i.e.
   buildClassifier(Instances)   - 
Method in class weka.classifiers.mi. MIOptimalBall 
 Builds the classifier
   buildClassifier(Instances)   - 
Method in class weka.classifiers.mi. MISMO 
 Method for building the classifier.
   buildClassifier(Instances)   - 
Method in class weka.classifiers.mi. MISVM 
 Builds the classifier
   buildClassifier(Instances)   - 
Method in class weka.classifiers.mi. MIWrapper 
 Builds the classifier
   buildClassifier(Instances)   - 
Method in class weka.classifiers.mi. SimpleMI 
 Builds the classifier
   buildClassifier(Instances)   - 
Method in class weka.classifiers.misc. HyperPipes 
 Generates the classifier.
   buildClassifier(Instances)   - 
Method in class weka.classifiers.misc. SerializedClassifier 
 loads only the serialized classifier
   buildClassifier(Instances)   - 
Method in class weka.classifiers.misc. VFI 
 Generates the classifier.
   buildClassifier(Instances)   - 
Method in class weka.classifiers.pmml.consumer. PMMLClassifier 
 Throw an exception - PMML models are pre-built.
   buildClassifier(Instances)   - 
Method in class weka.classifiers.rules. ConjunctiveRule 
 Builds a single rule learner with REP dealing with nominal classes or
 numeric classes.
   buildClassifier(Instances)   - 
Method in class weka.classifiers.rules. DecisionTable 
 Generates the classifier.
   buildClassifier(Instances)   - 
Method in class weka.classifiers.rules. DTNB 
 Generates the classifier.
   buildClassifier(Instances)   - 
Method in class weka.classifiers.rules. JRip 
 Builds Ripper in the order of class frequencies.
   buildClassifier(Instances)   - 
Method in class weka.classifiers.rules. NNge 
 Generates a classifier.
   buildClassifier(Instances)   - 
Method in class weka.classifiers.rules. OneR 
 Generates the classifier.
   buildClassifier(Instances)   - 
Method in class weka.classifiers.rules. PART 
 Generates the classifier.
   buildClassifier(Instances)   - 
Method in class weka.classifiers.rules.part. MakeDecList 
 Builds dec list.
   buildClassifier(Instances)   - 
Method in class weka.classifiers.rules. Prism 
 Generates the classifier.
   buildClassifier(Instances)   - 
Method in class weka.classifiers.rules. Ridor 
 Builds a ripple-down manner rule learner.
   buildClassifier(Instances)   - 
Method in class weka.classifiers.rules. ZeroR 
 Generates the classifier.
   buildClassifier(Instances)   - 
Method in class weka.classifiers.trees. ADTree 
 Builds a classifier for a set of instances.
   buildClassifier(Instances)   - 
Method in class weka.classifiers.trees. BFTree 
 Method for building a BestFirst decision tree classifier.
   buildClassifier(Instances)   - 
Method in class weka.classifiers.trees. DecisionStump 
 Generates the classifier.
   buildClassifier(Instances)   - 
Method in class weka.classifiers.trees. FT 
 Builds the classifier.
   buildClassifier(Instances)   - 
Method in class weka.classifiers.trees.ft. FTInnerNode 
 Method for building a Functional Inner tree (only called for the root node).
   buildClassifier(Instances)   - 
Method in class weka.classifiers.trees.ft. FTLeavesNode 
 Method for building a Functional Leaves tree (only called for the root node).
   buildClassifier(Instances)   - 
Method in class weka.classifiers.trees.ft. FTNode 
 Method for building a Functional tree (only called for the root node).
   buildClassifier(Instances)   - 
Method in class weka.classifiers.trees.ft. FTtree 
 Method for building a Functional Tree (only called for the root node).
   buildClassifier(Instances)   - 
Method in class weka.classifiers.trees. Id3 
 Builds Id3 decision tree classifier.
   buildClassifier(Instances)   - 
Method in class weka.classifiers.trees.j48. BinC45Split 
 Creates a C4.5-type split on the given data.
   buildClassifier(Instances)   - 
Method in class weka.classifiers.trees. J48 
 Generates the classifier.
   buildClassifier(Instances)   - 
Method in class weka.classifiers.trees.j48. C45PruneableClassifierTree 
 Method for building a pruneable classifier tree.
   buildClassifier(Instances)   - 
Method in class weka.classifiers.trees.j48. C45PruneableClassifierTreeG 
 Method for building a pruneable classifier tree.
   buildClassifier(Instances)   - 
Method in class weka.classifiers.trees.j48. C45Split 
 Creates a C4.5-type split on the given data.
   buildClassifier(Instances)   - 
Method in class weka.classifiers.trees.j48. ClassifierSplitModel 
 Builds the classifier split model for the given set of instances.
   buildClassifier(Instances)   - 
Method in class weka.classifiers.trees.j48. ClassifierTree 
 Method for building a classifier tree.
   buildClassifier(Instances)   - 
Method in class weka.classifiers.trees.j48. GraftSplit 
 builds m_graftdistro using the passed data
   buildClassifier(Instances)   - 
Method in class weka.classifiers.trees.j48. NBTreeClassifierTree 
 Method for building a naive bayes classifier tree
   buildClassifier(Instances)   - 
Method in class weka.classifiers.trees.j48. NBTreeNoSplit 
 Build the no-split node
   buildClassifier(Instances)   - 
Method in class weka.classifiers.trees.j48. NBTreeSplit 
 Creates a NBTree-type split on the given data.
   buildClassifier(Instances)   - 
Method in class weka.classifiers.trees.j48. NoSplit 
 Creates a "no-split"-split for a given set of instances.
   buildClassifier(Instances)   - 
Method in class weka.classifiers.trees.j48. PruneableClassifierTree 
 Method for building a pruneable classifier tree.
   buildClassifier(Instances)   - 
Method in class weka.classifiers.trees. J48graft 
 Generates the classifier.
   buildClassifier(Instances)   - 
Method in class weka.classifiers.trees. LADTree 
 Builds a classifier for a set of instances.
   buildClassifier(Instances)   - 
Method in class weka.classifiers.trees. LMT 
 Builds the classifier.
   buildClassifier(Instances)   - 
Method in class weka.classifiers.trees.lmt. LMTNode 
 Method for building a logistic model tree (only called for the root node).
   buildClassifier(Instances)   - 
Method in class weka.classifiers.trees.lmt. LogisticBase 
 Builds the logistic regression model usiing LogitBoost.
   buildClassifier(Instances, double[][], double[][])   - 
Method in class weka.classifiers.trees.lmt. ResidualSplit 
 Builds the split.
   buildClassifier(Instances)   - 
Method in class weka.classifiers.trees.lmt. ResidualSplit 
 Method not in use
   buildClassifier(Instances)   - 
Method in class weka.classifiers.trees.m5. M5Base 
 Generates the classifier.
   buildClassifier(Instances)   - 
Method in class weka.classifiers.trees.m5. PreConstructedLinearModel 
 Builds the classifier.
   buildClassifier(Instances)   - 
Method in class weka.classifiers.trees.m5. Rule 
 Generates a single rule or m5 model tree.
   buildClassifier(Instances)   - 
Method in class weka.classifiers.trees.m5. RuleNode 
 Build this node (find an attribute and split point)
   buildClassifier(Instances)   - 
Method in class weka.classifiers.trees. NBTree 
 Generates the classifier.
   buildClassifier(Instances)   - 
Method in class weka.classifiers.trees. RandomForest 
 Builds a classifier for a set of instances.
   buildClassifier(Instances)   - 
Method in class weka.classifiers.trees. RandomTree 
 Builds classifier.
   buildClassifier(Instances)   - 
Method in class weka.classifiers.trees. REPTree 
 Builds classifier.
   buildClassifier(Instances)   - 
Method in class weka.classifiers.trees. SimpleCart 
 Build the classifier.
   buildClassifier(Instances)   - 
Method in class weka.classifiers.trees. UserClassifier 
 Call this function to build a decision tree for the training
 data provided.
   buildClassifierForNode(ND.NDTree, Instances)   - 
Method in class weka.classifiers.meta.nestedDichotomies. ND 
 Builds the classifier for one node.
   buildClusterer(Instances)   - 
Method in class weka.clusterers. AbstractClusterer 
 Generates a clusterer.
   buildClusterer(Instances)   - 
Method in class weka.clusterers. CLOPE 
 Generate Clustering via CLOPE
   buildClusterer(Instances)   - 
Method in interface weka.clusterers. Clusterer 
 Generates a clusterer.
   buildClusterer(Instances)   - 
Method in class weka.clusterers. Cobweb 
 Builds the clusterer.
   buildClusterer(Instances)   - 
Method in class weka.clusterers. DBScan 
 Generate Clustering via DBScan
   buildClusterer(Instances)   - 
Method in class weka.clusterers. EM 
 Generates a clusterer.
   buildClusterer(Instances)   - 
Method in class weka.clusterers. FarthestFirst 
 Generates a clusterer.
   buildClusterer(Instances)   - 
Method in class weka.clusterers. FilteredClusterer 
 Build the clusterer on the filtered data.
   buildClusterer(Instances)   - 
Method in class weka.clusterers. HierarchicalClusterer 
  
   buildClusterer(Instances)   - 
Method in class weka.clusterers. MakeDensityBasedClusterer 
 Builds a clusterer for a set of instances.
   buildClusterer(Instances)   - 
Method in class weka.clusterers. OPTICS 
 Generate Clustering via OPTICS
   buildClusterer(Instances)   - 
Method in class weka.clusterers. sIB 
 Generates a clusterer.
   buildClusterer(Instances)   - 
Method in class weka.clusterers. SimpleKMeans 
 Generates a clusterer.
   buildClusterer(Instances)   - 
Method in class weka.clusterers. XMeans 
 Generates the X-Means clusterer.
   buildCNN()   - 
Method in class weka.classifiers.mi. CitationKNN 
 generates all the variables associated to the citation
 classifier
   buildDecList(Instances, boolean)   - 
Method in class weka.classifiers.rules.part. C45PruneableDecList 
 Builds the partial tree without hold out set.
   buildDecList(Instances, boolean)   - 
Method in class weka.classifiers.rules.part. ClassifierDecList 
 Builds the partial tree without hold out set.
   buildDecList(Instances, Instances, boolean)   - 
Method in class weka.classifiers.rules.part. PruneableDecList 
 Builds the partial tree with hold out set
   buildDistribution(double, double)   - 
Method in class weka.associations. PriorEstimation 
 updates the distribution of the confidence values.
   buildEstimator(Estimator, String[], boolean)   - 
Static method in class weka.estimators. Estimator 
 Build an estimator using the options.
   buildEstimator(Estimator, Instances, int, int, int, boolean)   - 
Static method in class weka.estimators. Estimator 
  
   buildEvaluator(Instances)   - 
Method in class weka.attributeSelection. ASEvaluation 
 Generates a attribute evaluator.
   buildEvaluator(Instances)   - 
Method in class weka.attributeSelection. CfsSubsetEval 
 Generates a attribute evaluator.
   buildEvaluator(Instances)   - 
Method in class weka.attributeSelection. ChiSquaredAttributeEval 
 Initializes a chi-squared attribute evaluator.
   buildEvaluator(Instances)   - 
Method in class weka.attributeSelection. ClassifierSubsetEval 
 Generates a attribute evaluator.
   buildEvaluator(Instances)   - 
Method in class weka.attributeSelection. ConsistencySubsetEval 
 Generates a attribute evaluator.
   buildEvaluator(Instances)   - 
Method in class weka.attributeSelection. CostSensitiveASEvaluation 
 Generates a attribute evaluator.
   buildEvaluator(Instances)   - 
Method in class weka.attributeSelection. FilteredAttributeEval 
 Initializes a filtered attribute evaluator.
   buildEvaluator(Instances)   - 
Method in class weka.attributeSelection. FilteredSubsetEval 
 Initializes a filtered attribute evaluator.
   buildEvaluator(Instances)   - 
Method in class weka.attributeSelection. GainRatioAttributeEval 
 Initializes a gain ratio attribute evaluator.
   buildEvaluator(Instances)   - 
Method in class weka.attributeSelection. InfoGainAttributeEval 
 Initializes an information gain attribute evaluator.
   buildEvaluator(Instances)   - 
Method in class weka.attributeSelection. LatentSemanticAnalysis 
 Initializes the singular values/vectors and performs the analysis
   buildEvaluator(Instances)   - 
Method in class weka.attributeSelection. OneRAttributeEval 
 Initializes a OneRAttribute attribute evaluator.
   buildEvaluator(Instances)   - 
Method in class weka.attributeSelection. PrincipalComponents 
 Initializes principal components and performs the analysis
   buildEvaluator(Instances)   - 
Method in class weka.attributeSelection. ReliefFAttributeEval 
 Initializes a ReliefF attribute evaluator.
   buildEvaluator(Instances)   - 
Method in class weka.attributeSelection. SVMAttributeEval 
 Initializes the evaluator.
   buildEvaluator(Instances)   - 
Method in class weka.attributeSelection. SymmetricalUncertAttributeEval 
 Initializes a symmetrical uncertainty attribute evaluator.
   buildEvaluator(Instances)   - 
Method in class weka.attributeSelection. WrapperSubsetEval 
 Generates a attribute evaluator.
   buildGenerator(Instances)   - 
Method in interface weka.gui.boundaryvisualizer. DataGenerator 
 Build the data generator
   buildGenerator(Instances)   - 
Method in class weka.gui.boundaryvisualizer. KDDataGenerator 
 Initialize the generator using the supplied instances
   buildKernel(Instances)   - 
Method in class weka.classifiers.functions.supportVector. CachedKernel 
 builds the kernel with the given data.
   buildKernel(Instances)   - 
Method in class weka.classifiers.functions.supportVector. Kernel 
 builds the kernel with the given data
   buildKernel(Instances)   - 
Method in class weka.classifiers.functions.supportVector. Puk 
 builds the kernel with the given data.
   buildKernel(Instances)   - 
Method in class weka.classifiers.functions.supportVector. RBFKernel 
 builds the kernel with the given data.
   buildKernel(Instances)   - 
Method in class weka.classifiers.functions.supportVector. StringKernel 
 builds the kernel with the given data.
   buildKernel(Instances)   - 
Method in class weka.classifiers.mi.supportVector. MIRBFKernel 
 builds the kernel with the given data.
   buildLogisticModelsTipText()   - 
Method in class weka.classifiers.functions. SMO 
 Returns the tip text for this property
   buildLogisticModelsTipText()   - 
Method in class weka.classifiers.mi. MISMO 
 Returns the tip text for this property
   buildRegressionTreeTipText()   - 
Method in class weka.classifiers.trees.m5. M5Base 
 Returns the tip text for this property
   buildRule(Instances)   - 
Method in class weka.classifiers.rules.part. ClassifierDecList 
 Method for building a pruned partial tree.
   buildRule(Instances, Instances)   - 
Method in class weka.classifiers.rules.part. PruneableDecList 
 Method for building a pruned partial tree.
   buildStructure()   - 
Method in class weka.classifiers.bayes. BayesNet 
 buildStructure determines the network structure/graph of the network.
   buildStructure(BayesNet, Instances)   - 
Method in class weka.classifiers.bayes.net.search.fixed. FromFile 
  
   buildStructure(BayesNet, Instances)   - 
Method in class weka.classifiers.bayes.net.search.fixed. NaiveBayes 
  
   buildStructure(BayesNet, Instances)   - 
Method in class weka.classifiers.bayes.net.search.global. TAN 
 buildStructure determines the network structure/graph of the network
 using the maximimum weight spanning tree algorithm of Chow and Liu
   buildStructure(BayesNet, Instances)   - 
Method in class weka.classifiers.bayes.net.search.local. LocalScoreSearchAlgorithm 
 buildStructure determines the network structure/graph of the network
 with the K2 algorithm, restricted by its initial structure (which can
 be an empty graph, or a Naive Bayes graph.
   buildStructure(BayesNet, Instances)   - 
Method in class weka.classifiers.bayes.net.search.local. TAN 
 buildStructure determines the network structure/graph of the network
 using the maximimum weight spanning tree algorithm of Chow and Liu
   buildStructure(BayesNet, Instances)   - 
Method in class weka.classifiers.bayes.net.search. SearchAlgorithm 
 buildStructure determines the network structure/graph of the network.
   buildTree(Instances, SimpleLinearRegression[][], double, double)   - 
Method in class weka.classifiers.trees.ft. FTInnerNode 
 Method for building the tree structure.
   buildTree(Instances, SimpleLinearRegression[][], double, double)   - 
Method in class weka.classifiers.trees.ft. FTLeavesNode 
 Method for building the tree structure.
   buildTree(Instances, SimpleLinearRegression[][], double, double)   - 
Method in class weka.classifiers.trees.ft. FTNode 
 Method for building the tree structure.
   buildTree(Instances, SimpleLinearRegression[][], double, double)   - 
Method in class weka.classifiers.trees.ft. FTtree 
 Abstract method for building the tree structure.
   buildTree(Instances, boolean)   - 
Method in class weka.classifiers.trees.j48. ClassifierTree 
 Builds the tree structure.
   buildTree(Instances, Instances, boolean)   - 
Method in class weka.classifiers.trees.j48. ClassifierTree 
 Builds the tree structure with hold out set
   buildTree(Instances, SimpleLinearRegression[][], double, double)   - 
Method in class weka.classifiers.trees.lmt. LMTNode 
 Method for building the tree structure.
   buildTree()   - 
Method in class weka.core.neighboursearch.balltrees. BallTreeConstructor 
 Builds the ball tree.
   buildTree()   - 
Method in class weka.core.neighboursearch.balltrees. BottomUpConstructor 
 Builds the ball tree bottom up.
   buildTree()   - 
Method in class weka.core.neighboursearch.balltrees. MiddleOutConstructor 
 Builds a ball tree middle out.
   buildTree()   - 
Method in class weka.core.neighboursearch.balltrees. TopDownConstructor 
 Builds the ball tree top down.
   BuiltInArithmetic   - Class in  weka.core.pmml  Built-in function for +, -, *, /.   BuiltInArithmetic(BuiltInArithmetic.Operator)   - 
Constructor for class weka.core.pmml. BuiltInArithmetic 
 Construct a new Arithmetic built-in pmml function.
   BuiltInMath   - Class in  weka.core.pmml  Built-in function for min, max, sum, avg, log10,
 ln, sqrt, abs, exp, pow, threshold, floor, ceil and round.   BuiltInMath(BuiltInMath.MathFunc)   - 
Constructor for class weka.core.pmml. BuiltInMath 
 Construct a new built-in pmml Math function.
   BuiltInString   - Class in  weka.core.pmml  Built-in function for uppercase, substring and trimblanks.   BVDecompose   - Class in  weka.classifiers  Class for performing a Bias-Variance decomposition on any classifier using the method specified in: 
  
 Ron Kohavi, David H.   BVDecompose()   - 
Constructor for class weka.classifiers. BVDecompose 
  
   BVDecomposeSegCVSub   - Class in  weka.classifiers  This class performs Bias-Variance decomposion on any classifier using the sub-sampled cross-validation procedure as specified in (1). 
 The Kohavi and Wolpert definition of bias and variance is specified in (2). 
 The Webb definition of bias and variance is specified in (3). 
  
 Geoffrey I.   BVDecomposeSegCVSub()   - 
Constructor for class weka.classifiers. BVDecomposeSegCVSub 
  
   BYTE   - 
Static variable in class weka.experiment. DatabaseUtils 
 Type mapping for BYTE used for reading experiment results.
 
 
    
 C  
 
   C45Loader   - Class in  weka.core.converters  Reads a file that is C45 format.   C45Loader()   - 
Constructor for class weka.core.converters. C45Loader 
  
   C45ModelSelection   - Class in  weka.classifiers.trees.j48  Class for selecting a C4.5-type split for a given dataset.   C45ModelSelection(int, Instances)   - 
Constructor for class weka.classifiers.trees.j48. C45ModelSelection 
 Initializes the split selection method with the given parameters.
   C45PruneableClassifierTree   - Class in  weka.classifiers.trees.j48  Class for handling a tree structure that can
 be pruned using C4.5 procedures.   C45PruneableClassifierTree(ModelSelection, boolean, float, boolean, boolean)   - 
Constructor for class weka.classifiers.trees.j48. C45PruneableClassifierTree 
 Constructor for pruneable tree structure.
   C45PruneableClassifierTreeG   - Class in  weka.classifiers.trees.j48  Class for handling a tree structure that can
 be pruned using C4.5 procedures and have nodes grafted on.   C45PruneableClassifierTreeG(ModelSelection, boolean, float, boolean, boolean, boolean)   - 
Constructor for class weka.classifiers.trees.j48. C45PruneableClassifierTreeG 
 Constructor for pruneable tree structure.
   C45PruneableClassifierTreeG(ModelSelection, Instances, ClassifierSplitModel, boolean, float, boolean, boolean, boolean, boolean)   - 
Constructor for class weka.classifiers.trees.j48. C45PruneableClassifierTreeG 
 Constructor for pruneable tree structure.
   C45PruneableDecList   - Class in  weka.classifiers.rules.part  Class for handling a partial tree structure pruned using C4.5's
 pruning heuristic.   C45PruneableDecList(ModelSelection, double, int)   - 
Constructor for class weka.classifiers.rules.part. C45PruneableDecList 
 Constructor for pruneable tree structure.
   C45Saver   - Class in  weka.core.converters  Writes to a destination that is in the format used by the C4.5 algorithm. 
 Therefore it outputs a names and a data file.   C45Saver()   - 
Constructor for class weka.core.converters. C45Saver 
 Constructor
   C45Split   - Class in  weka.classifiers.trees.j48  Class implementing a C4.5-type split on an attribute.   C45Split(int, int, double)   - 
Constructor for class weka.classifiers.trees.j48. C45Split 
 Initializes the split model.
   CachedKernel   - Class in  weka.classifiers.functions.supportVector  Base class for RBFKernel and PolyKernel that implements a simple LRU.   CachedKernel()   - 
Constructor for class weka.classifiers.functions.supportVector. CachedKernel 
 default constructor - does nothing.
   cacheKeyNameTipText()   - 
Method in class weka.experiment. DatabaseResultListener 
 Returns the tip text for this property
   cacheSizeTipText()   - 
Method in class weka.classifiers.functions. LibSVM 
 Returns the tip text for this property
   cacheSizeTipText()   - 
Method in class weka.classifiers.functions.supportVector. CachedKernel 
 Returns the tip text for this property
   cacheSizeTipText()   - 
Method in class weka.classifiers.functions.supportVector. StringKernel 
 Returns the tip text for this property
   calcCentroidPivot(int[], Instances)   - 
Static method in class weka.core.neighboursearch.balltrees. BallNode 
 Calculates the centroid pivot of a node.
   calcCentroidPivot(int, int, int[], Instances)   - 
Static method in class weka.core.neighboursearch.balltrees. BallNode 
 Calculates the centroid pivot of a node.
   calcColumnWidth(int)   - 
Method in class weka.gui. JTableHelper 
 calcs the optimal column width of the given column
   calcColumnWidth(JTable, int)   - 
Static method in class weka.gui. JTableHelper 
 Calculates the optimal width for the column of the given table.
   calcFullMargins(BayesNet)   - 
Method in class weka.classifiers.bayes.net. MarginCalculator 
  
   calcGraph(int, int)   - 
Method in class weka.gui. AttributeVisualizationPanel 
 Recalculates the barplot or histogram to display, required usually when the
 attribute is changed or the component is resized.
   calcHeaderWidth(int)   - 
Method in class weka.gui. JTableHelper 
 calcs the optimal header width of the given column
   calcHeaderWidth(JTable, int)   - 
Static method in class weka.gui. JTableHelper 
 Calculates the optimal width for the header of the given table.
   calcMargins(BayesNet)   - 
Method in class weka.classifiers.bayes.net. MarginCalculator 
 Calc marginal distributions of nodes in Bayesian network
         Note that a connected network is assumed.
   calcNodeScore(int)   - 
Method in class weka.classifiers.bayes.net.search.local. LocalScoreSearchAlgorithm 
 Calc Node Score for given parent set
   calcOutOfBagTipText()   - 
Method in class weka.classifiers.meta. Bagging 
 Returns the tip text for this property
   calcPivot(BallNode, BallNode, Instances)   - 
Static method in class weka.core.neighboursearch.balltrees. BallNode 
 Calculates the centroid pivot of a node based on its
 two child nodes (if merging two nodes).
   calcPivot(BottomUpConstructor.TempNode, BottomUpConstructor.TempNode, Instances)   - 
Method in class weka.core.neighboursearch.balltrees. BottomUpConstructor 
 Calculates the centroid pivot of a node based on its
 two child nodes.
   calcPivot(MiddleOutConstructor.TempNode, MiddleOutConstructor.TempNode, Instances)   - 
Method in class weka.core.neighboursearch.balltrees. MiddleOutConstructor 
 /**
 Calculates the centroid pivot of a node based on its
 two child nodes (if merging two nodes).
   calcPivot(MiddleOutConstructor.MyIdxList, MiddleOutConstructor.MyIdxList, Instances)   - 
Method in class weka.core.neighboursearch.balltrees. MiddleOutConstructor 
 Calculates the centroid pivot of a node based on 
 the list of points that it  contains (tbe two 
 lists of its children are provided).
   calcRadius(int[], Instances, Instance, DistanceFunction)   - 
Static method in class weka.core.neighboursearch.balltrees. BallNode 
 Calculates the radius of node.
   calcRadius(int, int, int[], Instances, Instance, DistanceFunction)   - 
Static method in class weka.core.neighboursearch.balltrees. BallNode 
 Calculates the radius of a node.
   calcRadius(BallNode, BallNode, Instance, DistanceFunction)   - 
Static method in class weka.core.neighboursearch.balltrees. BallNode 
 Calculates the radius of a node based on its two 
 child nodes (if merging two nodes).
   calcRadius(BottomUpConstructor.TempNode, BottomUpConstructor.TempNode)   - 
Method in class weka.core.neighboursearch.balltrees. BottomUpConstructor 
 Calculates the radius of a node based on its two
 child nodes.
   calcRadius(MiddleOutConstructor.TempNode, MiddleOutConstructor.TempNode)   - 
Method in class weka.core.neighboursearch.balltrees. MiddleOutConstructor 
 Calculates the radius of a node based on its two 
 child nodes (if merging two nodes).
   calcRadius(MiddleOutConstructor.MyIdxList, MiddleOutConstructor.MyIdxList, Instance, Instances)   - 
Method in class weka.core.neighboursearch.balltrees. MiddleOutConstructor 
 Calculates the radius of a node based on the
 list of points that it contains (the two lists of 
 its children are provided).
   calcScore(BayesNet)   - 
Method in class weka.classifiers.bayes.net.search.global. GlobalScoreSearchAlgorithm 
 performCV returns the accuracy calculated using cross validation.
   calcScoreWithExtraParent(int, int)   - 
Method in class weka.classifiers.bayes.net.search.global. GlobalScoreSearchAlgorithm 
 Calc Node Score With Added Parent
   calcScoreWithExtraParent(int, int)   - 
Method in class weka.classifiers.bayes.net.search.local. LocalScoreSearchAlgorithm 
 Calc Node Score With AddedParent
   calcScoreWithMissingParent(int, int)   - 
Method in class weka.classifiers.bayes.net.search.global. GlobalScoreSearchAlgorithm 
 Calc Node Score With Parent Deleted
   calcScoreWithMissingParent(int, int)   - 
Method in class weka.classifiers.bayes.net.search.local. LocalScoreSearchAlgorithm 
 Calc Node Score With Parent Deleted
   calcScoreWithReversedParent(int, int)   - 
Method in class weka.classifiers.bayes.net.search.global. GlobalScoreSearchAlgorithm 
 Calc Node Score With Arrow reversed
   calculateAlphas()   - 
Method in class weka.classifiers.trees.lmt. LMTNode 
 Updates the alpha field for all nodes.
   calculateAlphas()   - 
Method in class weka.classifiers.trees. SimpleCart 
 Updates the alpha field for all nodes.
   calculateConfirmation()   - 
Method in class weka.associations.tertius. Rule 
 Calculate the confirmation of this rule.
   calculateDerived()   - 
Method in class weka.experiment. PairedStats 
 Calculates the derived statistics (significance etc).
   calculateDerived()   - 
Method in class weka.experiment. PairedStatsCorrected 
 Calculates the derived statistics (significance etc).
   calculateDerived()   - 
Method in class weka.experiment. Stats 
 Tells the object to calculate any statistics that don't have their
 values automatically updated during add.
   calculateDistance(Instances)   - 
Method in class weka.classifiers.mi. MIOptimalBall 
 calculate the distances from each instance in a positive bag to each bag.
   calculateOptimistic()   - 
Method in class weka.associations.tertius. Rule 
 Calculate the optimistic estimate of this rule.
   calculatePriorSum(boolean, double)   - 
Method in class weka.associations. PriorEstimation 
 calculates the numerator and the denominator of the prior equation
   calculateStatistics(Instance, int, int, int)   - 
Method in class weka.experiment. PairedCorrectedTTester 
 Computes a paired t-test comparison for a specified dataset between
 two resultsets.
   calculateStatistics(Instance, int, int, int)   - 
Method in class weka.experiment. PairedTTester 
 Computes a paired t-test comparison for a specified dataset between
 two resultsets.
   calculateStatistics(Instance, int, int, int)   - 
Method in interface weka.experiment. Tester 
 Computes a paired t-test comparison for a specified dataset between
 two resultsets.
   calculateStdDevsTipText()   - 
Method in class weka.experiment. AveragingResultProducer 
 Returns the tip text for this property
   calculateTreshhold()   - 
Method in class weka.attributeSelection. ScatterSearchV1 
 Calculate the treshold of a dataSet given an evaluator
   canAcceptConnection(Class)   - 
Method in class weka.gui.beans. MetaBean 
 Checks to see if any of the inputs to this group implements
 the supplied listener class
   cancel()   - 
Method in class weka.core.converters. AbstractFileSaver 
 Cancels the incremental saving process.
   cancel()   - 
Method in class weka.core.converters. AbstractSaver 
 Cancels the incremental saving process if the write mode is CANCEL.
   cancel()   - 
Method in class weka.core.converters. DatabaseSaver 
 Cancels the incremental saving process and tries to drop the table if 
 the write mode is CANCEL.
   CANCEL_OPTION   - 
Static variable in class weka.gui.experiment. OutputFormatDialog 
 Signifies a cancelled property selection.
   CANCEL_OPTION   - 
Static variable in class weka.gui. ListSelectorDialog 
 Signifies a cancelled property selection
   CANCEL_OPTION   - 
Static variable in class weka.gui. PropertySelectorDialog 
 Signifies a cancelled property selection
   CANCEL_OPTION   - 
Static variable in class weka.gui. ViewerDialog 
 Signifies a cancelled property selection
   canKeep(Instance, Literal)   - 
Method in class weka.associations.tertius. Body 
 Test if an instance can be kept as a counter-instance,
 if a new literal is added to this body.
   canKeep(Instance, Literal)   - 
Method in class weka.associations.tertius. Head 
 Test if an instance can be kept as a counter-instance,
 if a new literal is added to this head.
   canKeep(Instance, Literal)   - 
Method in class weka.associations.tertius. LiteralSet 
 Test if an instance can be kept as a counter-instance,
 given a new literal.
   canMoveDown(JList)   - 
Static method in class weka.gui. JListHelper 
 checks whether the selected items can be moved down
   canMoveUp(JList)   - 
Static method in class weka.gui. JListHelper 
 checks whether the selected items can be moved up
   canRedo()   - 
Method in class weka.classifiers.bayes.net. EditableBayesNet 
 return whether there is something on the undo stack that can be performed
   canUndo()   - 
Method in class weka.classifiers.bayes.net. EditableBayesNet 
 return whether there is something on the undo stack that can be performed
   canUndo()   - 
Method in interface weka.core. Undoable 
 returns whether an undo is possible, i.e.
   canUndo()   - 
Method in class weka.gui.arffviewer. ArffPanel 
 returns whether an undo is possible
   canUndo()   - 
Method in class weka.gui.arffviewer. ArffSortedTableModel 
 returns whether an undo is possible, i.e.
   canUndo()   - 
Method in class weka.gui.arffviewer. ArffTableModel 
 returns whether an undo is possible, i.e.
   Capabilities   - Class in  weka.core  A class that describes the capabilites (e.g., handling certain types of
 attributes, missing values, types of classes, etc.) of a specific
 classifier.   Capabilities(CapabilitiesHandler)   - 
Constructor for class weka.core. Capabilities 
 initializes the capabilities for the given owner
   capabilities()   - 
Method in class weka.core. Capabilities 
 Returns an Iterator over the stored capabilities
   Capabilities.Capability   - Enum in  weka.core  enumeration of all capabilities   capabilitiesFilterChanged(Explorer.CapabilitiesFilterChangeEvent)   - 
Method in class weka.gui.explorer. AssociationsPanel 
 method gets called in case of a change event
   capabilitiesFilterChanged(Explorer.CapabilitiesFilterChangeEvent)   - 
Method in class weka.gui.explorer. AttributeSelectionPanel 
 method gets called in case of a change event
   capabilitiesFilterChanged(Explorer.CapabilitiesFilterChangeEvent)   - 
Method in class weka.gui.explorer. ClassifierPanel 
 method gets called in case of a change event
   capabilitiesFilterChanged(Explorer.CapabilitiesFilterChangeEvent)   - 
Method in class weka.gui.explorer. ClustererPanel 
 method gets called in case of a change event
   capabilitiesFilterChanged(Explorer.CapabilitiesFilterChangeEvent)   - 
Method in interface weka.gui.explorer. Explorer.CapabilitiesFilterChangeListener 
 method gets called in case of a change event
   capabilitiesFilterChanged(Explorer.CapabilitiesFilterChangeEvent)   - 
Method in class weka.gui.explorer. PreprocessPanel 
 method gets called in case of a change event
   CapabilitiesHandler   - Interface in  weka.core  Classes implementing this interface return their capabilities in regards
 to datasets.   capacity()   - 
Method in class weka.core. FastVector 
 Returns the capacity of the vector.
   capacity()   - 
Method in class weka.core.matrix. DoubleVector 
 Gets the capacity of the vector.
   capacity()   - 
Method in class weka.core.matrix. IntVector 
 Returns the capacity of the vector
   cardinalityTipText()   - 
Method in class weka.datagenerators.classifiers.classification. BayesNet 
 Returns the tip text for this property
   caretUpdate(CaretEvent)   - 
Method in class weka.gui. LogWindow 
 Called when the caret position is updated.
   caretUpdate(CaretEvent)   - 
Method in class weka.gui.sql. ConnectionPanel 
 Called when the caret position is updated.
   caretUpdate(CaretEvent)   - 
Method in class weka.gui.sql. QueryPanel 
 Called when the caret position is updated.
   carTipText()   - 
Method in class weka.associations. Apriori 
 Returns the tip text for this property
   carTipText()   - 
Method in class weka.associations. PredictiveApriori 
 Returns the tip text for this property
   CaRuleGeneration   - Class in  weka.associations  Class implementing the rule generation procedure of the predictive apriori algorithm for class association rules.   CaRuleGeneration(ItemSet)   - 
Constructor for class weka.associations. CaRuleGeneration 
 Constructor
   CARuleMiner   - Interface in  weka.associations  Interface for learning class association rules.   cat(DoubleVector)   - 
Method in class weka.core.matrix. DoubleVector 
 Combine two vectors together
   cbind(PaceMatrix)   - 
Method in class weka.classifiers.functions.pace. PaceMatrix 
 Returns a new matrix which binds two matrices with columns.
   CEIL   - 
Static variable in interface weka.core.mathematicalexpression. sym 
  
   CEIL   - 
Static variable in interface weka.filters.unsupervised.instance.subsetbyexpression. sym 
  
   Center   - Class in  weka.filters.unsupervised.attribute  Centers all numeric attributes in the given dataset to have zero mean (apart from the class attribute, if set).   Center()   - 
Constructor for class weka.filters.unsupervised.attribute. Center 
  
   centerDataTipText()   - 
Method in class weka.attributeSelection. PrincipalComponents 
 Returns the tip text for this property
   centerDataTipText()   - 
Method in class weka.filters.unsupervised.attribute. PrincipalComponents 
 Returns the tip text for this property
   centerHorizontal(FastVector)   - 
Method in class weka.classifiers.bayes.net. EditableBayesNet 
 center set of nodes half way between left and right most node in the list
   centerInstances(Instances, int[], double)   - 
Method in class weka.core.neighboursearch. KDTree 
 Assigns instances to centers using KDTree.
   centerVertical(FastVector)   - 
Method in class weka.classifiers.bayes.net. EditableBayesNet 
 center set of nodes half way between top and bottom most node in the list
   CfsSubsetEval   - Class in  weka.attributeSelection  CfsSubsetEval : 
  
 Evaluates the worth of a subset of attributes by considering the individual predictive ability of each feature along with the degree of redundancy between them. 
  
 Subsets of features that are highly correlated with the class while having low intercorrelation are preferred. 
  
 For more information see: 
  
 M.   CfsSubsetEval()   - 
Constructor for class weka.attributeSelection. CfsSubsetEval 
 Constructor
   change()   - 
Method in class weka.associations. RuleGeneration 
 Gets if the list fo the best rules has been changed
   Change   - 
Variable in class weka.classifiers.bayes. BayesianLogisticRegression 
 This variable is used to keep track of change in
 the value of delta summation of r(i).
   ChangeDateFormat   - Class in  weka.filters.unsupervised.attribute  Changes the date format used by a date attribute.   ChangeDateFormat()   - 
Constructor for class weka.filters.unsupervised.attribute. ChangeDateFormat 
  
   changeLength(double)   - 
Method in class weka.core. AlgVector 
 Changes the length of a vector.
   changeUID(long, long, String, String)   - 
Static method in class weka.core.xml. SerialUIDChanger 
 changes the oldUID into newUID from the given file (binary/KOML) into the
 other one (binary/KOML).
   CharacterDelimitedTokenizer   - Class in  weka.core.tokenizers  Abstract superclass for tokenizers that take characters as delimiters.   CharacterDelimitedTokenizer()   - 
Constructor for class weka.core.tokenizers. CharacterDelimitedTokenizer 
  
   charSetTipText()   - 
Method in class weka.core.converters. TextDirectoryLoader 
 the tip text for this property
   ChartEvent   - Class in  weka.gui.beans  Event encapsulating info for plotting a data point on the StripChart   ChartEvent(Object, Vector, double, double, double[], boolean)   - 
Constructor for class weka.gui.beans. ChartEvent 
 Creates a new  ChartEvent  instance.
   ChartEvent(Object)   - 
Constructor for class weka.gui.beans. ChartEvent 
 Creates a new  ChartEvent  instance.
   ChartListener   - Interface in  weka.gui.beans  Interface to something that can process a ChartEvent   ChebyshevDistance   - Class in  weka.core  Implements the Chebyshev distance.   ChebyshevDistance()   - 
Constructor for class weka.core. ChebyshevDistance 
 Constructs an Chebyshev Distance object, Instances must be still set.
   ChebyshevDistance(Instances)   - 
Constructor for class weka.core. ChebyshevDistance 
 Constructs an Chebyshev Distance object and automatically initializes the
 ranges.
   check(double)   - 
Method in class weka.classifiers.trees.j48. Distribution 
 Checks if at least two bags contain a minimum number of instances.
   Check   - Class in  weka.core  Abstract general class for testing in Weka.   Check()   - 
Constructor for class weka.core. Check 
  
   CheckAssociator   - Class in  weka.associations  Class for examining the capabilities and finding problems with 
 associators.   CheckAssociator()   - 
Constructor for class weka.associations. CheckAssociator 
  
   CheckAttributeSelection   - Class in  weka.attributeSelection  Class for examining the capabilities and finding problems with 
 attribute selection schemes.   CheckAttributeSelection()   - 
Constructor for class weka.attributeSelection. CheckAttributeSelection 
  
   CheckBoxList   - Class in  weka.gui  An extended JList that contains CheckBoxes.   CheckBoxList()   - 
Constructor for class weka.gui. CheckBoxList 
 initializes the list with an empty CheckBoxListModel
   CheckBoxList(CheckBoxList.CheckBoxListModel)   - 
Constructor for class weka.gui. CheckBoxList 
 initializes the list with the given CheckBoxListModel
   CheckBoxList.CheckBoxListModel   - Class in  weka.gui  A specialized model.   CheckBoxList.CheckBoxListModel()   - 
Constructor for class weka.gui. CheckBoxList.CheckBoxListModel 
 initializes the model with no data.
   CheckBoxList.CheckBoxListModel(Object[])   - 
Constructor for class weka.gui. CheckBoxList.CheckBoxListModel 
 Constructs a CheckBoxListModel from an array of objects and then applies 
 setModel to it.
   CheckBoxList.CheckBoxListModel(Vector)   - 
Constructor for class weka.gui. CheckBoxList.CheckBoxListModel 
 Constructs a CheckBoxListModel from a Vector and then applies setModel 
 to it.
   CheckBoxList.CheckBoxListRenderer   - Class in  weka.gui  A specialized CellRenderer for the CheckBoxList   CheckBoxList.CheckBoxListRenderer()   - 
Constructor for class weka.gui. CheckBoxList.CheckBoxListRenderer 
  
   checkCanonicalUserOptions()   - 
Method in class weka.core. CheckOptionHandler 
 checks whether the user-supplied options stay the same after settting,
 getting and re-setting again
   CheckClassifier   - Class in  weka.classifiers  Class for examining the capabilities and finding problems with 
 classifiers.   CheckClassifier()   - 
Constructor for class weka.classifiers. CheckClassifier 
  
   CheckClusterer   - Class in  weka.clusterers  Class for examining the capabilities and finding problems with 
 clusterers.   CheckClusterer()   - 
Constructor for class weka.clusterers. CheckClusterer 
 default constructor
   checkDefaultOptions()   - 
Method in class weka.core. CheckOptionHandler 
 checks whether the default options can be processed completely
 or some invalid options are returned by the getOptions() method.
   checkErrorRateTipText()   - 
Method in class weka.classifiers.rules. JRip 
 Returns the tip text for this property
   CheckEstimator   - Class in  weka.estimators  Class for examining the capabilities and finding problems with 
 estimators.   CheckEstimator()   - 
Constructor for class weka.estimators. CheckEstimator 
  
   CheckEstimator.AttrTypes   - Class in  weka.estimators  class that contains info about the attribute types the estimator can estimate
 estimator work on one attribute only   CheckEstimator.EstTypes   - Class in  weka.estimators  public class that contains info about the chosen attribute type
 estimator work on one attribute only   CheckEstimator.EstTypes()   - 
Constructor for class weka.estimators. CheckEstimator.EstTypes 
 Constructor
   CheckEstimator.EstTypes(boolean, boolean, boolean)   - 
Constructor for class weka.estimators. CheckEstimator.EstTypes 
 Constructor
   CheckEstimator.PostProcessor   - Class in  weka.estimators  a class for postprocessing the test-data   CheckEstimator.PostProcessor()   - 
Constructor for class weka.estimators. CheckEstimator.PostProcessor 
  
   checkForAttributeType(int)   - 
Method in class weka.core. Instances 
 Checks for attributes of the given type in the dataset
   checkForMissing(Instance, Instances)   - 
Method in class weka.classifiers.functions. PaceRegression 
 Checks if an instance has a missing value.
   checkForNominalAttributes(Instances)   - 
Method in class weka.clusterers. XMeans 
 Checks for nominal attributes in the dataset.
   checkForRemainingOptions(String[])   - 
Static method in class weka.core. Utils 
 Checks if the given array contains any non-empty options.
   checkForStringAttributes()   - 
Method in class weka.core. Instances 
 Checks for string attributes in the dataset
   checkGlobalInfo()   - 
Method in class weka.core. CheckGOE 
 checks whether the object declares a globalInfo method.
   CheckGOE   - Class in  weka.core  Simple command line checking of classes that are editable in the GOE. 

 Usage:  
  
     CheckGOE -W classname -- test options
    

  
 Valid options are:     CheckGOE()   - 
Constructor for class weka.core. CheckGOE 
 default constructor
   checkIndicesList(MiddleOutConstructor.MyIdxList, int, int)   - 
Method in class weka.core.neighboursearch.balltrees. MiddleOutConstructor 
 Checks whether if the points in an index list
 are in some specified of the master index array.
   checkInstance(Instance)   - 
Method in class weka.core. Instances 
 Checks if the given instance is compatible
 with this dataset.
   CheckKernel   - Class in  weka.classifiers.functions.supportVector  Class for examining the capabilities and finding problems with 
 kernels.   CheckKernel()   - 
Constructor for class weka.classifiers.functions.supportVector. CheckKernel 
  
   checkListOptions()   - 
Method in class weka.core. CheckOptionHandler 
 checks whether the listOptions method works
   checkModel()   - 
Method in class weka.classifiers.trees.j48. ClassifierSplitModel 
 Checks if generated model is valid.
   checkModel(int)   - 
Method in class weka.classifiers.trees.lmt. ResidualSplit 
 Checks if there are at least 2 subsets that contain >= minNumInstances.
   CheckOptionHandler   - Class in  weka.core  Simple command line checking of classes that implement OptionHandler. 

 Usage:  
  
     CheckOptionHandler -W optionHandlerClassName -- test options
    

  
 Valid options are:     CheckOptionHandler()   - 
Constructor for class weka.core. CheckOptionHandler 
  
   checkRemainingOptions()   - 
Method in class weka.core. CheckOptionHandler 
 checks whether the user-supplied options can be processed completely
 or some "left-over" options remain
   checkResettingOptions()   - 
Method in class weka.core. CheckOptionHandler 
 checks whether the optionhandler can be re-setted again to default
 options after the user-supplied options have been set.
   CheckScheme   - Class in  weka.core  Abstract general class for testing schemes in Weka.   CheckScheme()   - 
Constructor for class weka.core. CheckScheme 
  
   CheckScheme.PostProcessor   - Class in  weka.core  a class for postprocessing the test-data   CheckScheme.PostProcessor()   - 
Constructor for class weka.core. CheckScheme.PostProcessor 
  
   checkSetOptions()   - 
Method in class weka.core. CheckOptionHandler 
 checks whether the user-supplied options can be processed at all
   CheckSource   - Class in  weka.classifiers  A simple class for checking the source generated from Classifiers
 implementing the  weka.classifiers.Sourcable  interface.   CheckSource()   - 
Constructor for class weka.classifiers. CheckSource 
  
   CheckSource   - Class in  weka.filters  A simple class for checking the source generated from Filters
 implementing the  weka.filters.Sourcable  interface.   CheckSource()   - 
Constructor for class weka.filters. CheckSource 
  
   checkStatus(Object)   - 
Method in interface weka.experiment. Compute 
 Check on the status of a  Task 
   checkStatus(Object)   - 
Method in class weka.experiment. RemoteEngine 
 Returns status information on a particular task
   checksTurnedOffTipText()   - 
Method in class weka.classifiers.functions. SMO 
 Returns the tip text for this property
   checksTurnedOffTipText()   - 
Method in class weka.classifiers.functions.supportVector. Kernel 
 Returns the tip text for this property
   checksTurnedOffTipText()   - 
Method in class weka.classifiers.mi. MISMO 
 Returns the tip text for this property
   checksTurnedOffTipText()   - 
Method in class weka.filters.unsupervised.attribute. KernelFilter 
 Returns the tip text for this property
   checkToolTips()   - 
Method in class weka.core. CheckGOE 
 checks whether the object declares tip text method for all its
 properties.
   children()   - 
Method in class weka.classifiers.trees.adtree. PredictionNode 
 Enumerates the children of this node.
   childrenValues()   - 
Method in class weka.gui. HierarchyPropertyParser 
 The value in the children nodes.
   chisqDistribution   - 
Static variable in class weka.core.matrix. Maths 
 Distribution type: chi-squared
   ChisqMixture   - Class in  weka.classifiers.functions.pace  Class for manipulating chi-square mixture distributions.   ChisqMixture()   - 
Constructor for class weka.classifiers.functions.pace. ChisqMixture 
 Contructs an empty ChisqMixture
   chiSquared(double[][], boolean)   - 
Static method in class weka.core. ContingencyTables 
 Returns chi-squared probability for a given matrix.
   ChiSquaredAttributeEval   - Class in  weka.attributeSelection  ChiSquaredAttributeEval : 
  
 Evaluates the worth of an attribute by computing the value of the chi-squared statistic with respect to the class. 
  
  

  
 Valid options are:     ChiSquaredAttributeEval()   - 
Constructor for class weka.attributeSelection. ChiSquaredAttributeEval 
 Constructor
   chiSquaredProbability(double, double)   - 
Static method in class weka.core. Statistics 
 Returns chi-squared probability for given value and degrees
 of freedom.
   chiVal(double[][], boolean)   - 
Static method in class weka.core. ContingencyTables 
 Computes chi-squared statistic for a contingency table.
   chol()   - 
Method in class weka.core.matrix. Matrix 
 Cholesky Decomposition
   CholeskyDecomposition   - Class in  weka.core.matrix  Cholesky Decomposition.   CholeskyDecomposition(Matrix)   - 
Constructor for class weka.core.matrix. CholeskyDecomposition 
 Cholesky algorithm for symmetric and positive definite matrix.
   chooseIndex()   - 
Method in class weka.classifiers.rules.part. ClassifierDecList 
 Method for choosing a subset to expand.
   chooseLastIndex()   - 
Method in class weka.classifiers.rules.part. ClassifierDecList 
 Choose last index (ie.
   CISearchAlgorithm   - Class in  weka.classifiers.bayes.net.search.ci  The CISearchAlgorithm class supports Bayes net structure search algorithms that are based on conditional independence test (as opposed to for example score based of cross validation based search algorithms).   CISearchAlgorithm()   - 
Constructor for class weka.classifiers.bayes.net.search.ci. CISearchAlgorithm 
  
   CitationKNN   - Class in  weka.classifiers.mi  Modified version of the Citation kNN multi instance classifier. 
  
 For more information see: 
  
 Jun Wang, Zucker, Jean-Daniel: Solving Multiple-Instance Problem: A Lazy Learning Approach.   CitationKNN()   - 
Constructor for class weka.classifiers.mi. CitationKNN 
  
   CLASS_IS_LAST   - 
Static variable in class weka.core. TestInstances 
 can be used for settting the class attribute index to last
   CLASS_PYTHONINERPRETER   - 
Static variable in class weka.core. Jython 
 the classname of the Python interpreter
   CLASS_PYTHONOBJECTINPUTSTREAM   - 
Static variable in class weka.core. Jython 
 the classname of the Python ObjectInputStream
   ClassAssigner   - Class in  weka.filters.unsupervised.attribute  Filter that can set and unset the class index.   ClassAssigner()   - 
Constructor for class weka.filters.unsupervised.attribute. ClassAssigner 
  
   ClassAssigner   - Class in  weka.gui.beans  Bean that assigns a class attribute to a data set.   ClassAssigner()   - 
Constructor for class weka.gui.beans. ClassAssigner 
  
   ClassAssignerBeanInfo   - Class in  weka.gui.beans  BeanInfo class for the class assigner bean   ClassAssignerBeanInfo()   - 
Constructor for class weka.gui.beans. ClassAssignerBeanInfo 
  
   ClassAssignerCustomizer   - Class in  weka.gui.beans  GUI customizer for the class assigner bean   ClassAssignerCustomizer()   - 
Constructor for class weka.gui.beans. ClassAssignerCustomizer 
  
   classAttribute()   - 
Method in class weka.core. Instance 
 Returns class attribute.
   classAttribute()   - 
Method in class weka.core. Instances 
 Returns the class attribute.
   classAttributeNames()   - 
Method in class weka.classifiers.functions. SMO 
  
   classAttributeNames()   - 
Method in class weka.classifiers.mi. MISMO 
 Returns the names of the class attributes.
   ClassBalancedND   - Class in  weka.classifiers.meta.nestedDichotomies  A meta classifier for handling multi-class datasets with 2-class classifiers by building a random class-balanced tree structure. 
  
 For more info, check 
  
 Lin Dong, Eibe Frank, Stefan Kramer: Ensembles of Balanced Nested Dichotomies for Multi-class Problems.   ClassBalancedND()   - 
Constructor for class weka.classifiers.meta.nestedDichotomies. ClassBalancedND 
 Constructor.
   classColumnTipText()   - 
Method in class weka.gui.beans. ClassAssigner 
 Tool tip text for this property
   ClassDiscovery   - Class in  weka.core  This class is used for discovering classes that implement a certain
 interface or a derived from a certain class.   ClassDiscovery()   - 
Constructor for class weka.core. ClassDiscovery 
  
   ClassDiscovery.StringCompare   - Class in  weka.core  compares two strings.   ClassDiscovery.StringCompare()   - 
Constructor for class weka.core. ClassDiscovery.StringCompare 
  
   classFirst(boolean)   - 
Method in class weka.experiment. Experiment 
 Sets whether the first attribute is treated as the class
 for all datasets involved in the experiment.
   classFlagTipText()   - 
Method in class weka.datagenerators. ClusterGenerator 
 Returns the tip text for this property
   ClassificationGenerator   - Class in  weka.datagenerators  Abstract class for data generators for classifiers.   ClassificationGenerator()   - 
Constructor for class weka.datagenerators. ClassificationGenerator 
 initializes with default values
   classificationTipText()   - 
Method in class weka.associations. Tertius 
 Returns the tip text for this property.
   ClassificationViaClustering   - Class in  weka.classifiers.meta  A simple meta-classifier that uses a clusterer for classification.   ClassificationViaClustering()   - 
Constructor for class weka.classifiers.meta. ClassificationViaClustering 
 default constructor
   ClassificationViaRegression   - Class in  weka.classifiers.meta  Class for doing classification using regression methods.   ClassificationViaRegression()   - 
Constructor for class weka.classifiers.meta. ClassificationViaRegression 
 Default constructor.
   Classifier   - Class in  weka.classifiers  Abstract classifier.   Classifier()   - 
Constructor for class weka.classifiers. Classifier 
  
   Classifier   - Class in  weka.gui.beans  Bean that wraps around weka.classifiers   Classifier()   - 
Constructor for class weka.gui.beans. Classifier 
 Creates a new  Classifier  instance.
   ClassifierBeanInfo   - Class in  weka.gui.beans  BeanInfo class for the Classifier wrapper bean   ClassifierBeanInfo()   - 
Constructor for class weka.gui.beans. ClassifierBeanInfo 
  
   ClassifierCustomizer   - Class in  weka.gui.beans  GUI customizer for the classifier wrapper bean   ClassifierCustomizer()   - 
Constructor for class weka.gui.beans. ClassifierCustomizer 
  
   ClassifierDecList   - Class in  weka.classifiers.rules.part  Class for handling a rule (partial tree) for a decision list.   ClassifierDecList(ModelSelection, int)   - 
Constructor for class weka.classifiers.rules.part. ClassifierDecList 
 Constructor - just calls constructor of class DecList.
   ClassifierPanel   - Class in  weka.gui.explorer  This panel allows the user to select and configure a classifier, set the
 attribute of the current dataset to be used as the class, and evaluate
 the classifier using a number of testing modes (test on the training data,
 train/test on a percentage split, n-fold cross-validation, test on a
 separate split).   ClassifierPanel()   - 
Constructor for class weka.gui.explorer. ClassifierPanel 
 Creates the classifier panel
   ClassifierPerformanceEvaluator   - Class in  weka.gui.beans  A bean that evaluates the performance of batch trained classifiers   ClassifierPerformanceEvaluator()   - 
Constructor for class weka.gui.beans. ClassifierPerformanceEvaluator 
  
   ClassifierPerformanceEvaluatorBeanInfo   - Class in  weka.gui.beans  Bean info class for the classifier performance evaluator   ClassifierPerformanceEvaluatorBeanInfo()   - 
Constructor for class weka.gui.beans. ClassifierPerformanceEvaluatorBeanInfo 
  
   classifiers()   - 
Method in class weka.classifiers.meta. LogitBoost 
 Returns the array of classifiers that have been built.
   ClassifierSplitEvaluator   - Class in  weka.experiment  A SplitEvaluator that produces results for a classification scheme on a nominal class attribute.   ClassifierSplitEvaluator()   - 
Constructor for class weka.experiment. ClassifierSplitEvaluator 
 No args constructor.
   ClassifierSplitModel   - Class in  weka.classifiers.trees.j48  Abstract class for classification models that can be used 
 recursively to split the data.   ClassifierSplitModel()   - 
Constructor for class weka.classifiers.trees.j48. ClassifierSplitModel 
  
   classifiersTipText()   - 
Method in class weka.classifiers.meta. MultiScheme 
 Returns the tip text for this property
   classifiersTipText()   - 
Method in class weka.classifiers. MultipleClassifiersCombiner 
 Returns the tip text for this property
   ClassifierSubsetEval   - Class in  weka.attributeSelection  Classifier subset evaluator: 
  
 Evaluates attribute subsets on training data or a seperate hold out testing set.   ClassifierSubsetEval()   - 
Constructor for class weka.attributeSelection. ClassifierSubsetEval 
  
   classifierTipText()   - 
Method in class weka.attributeSelection. ClassifierSubsetEval 
 Returns the tip text for this property
   classifierTipText()   - 
Method in class weka.attributeSelection. WrapperSubsetEval 
 Returns the tip text for this property
   classifierTipText()   - 
Method in class weka.classifiers. SingleClassifierEnhancer 
 Returns the tip text for this property
   classifierTipText()   - 
Method in class weka.experiment. ClassifierSplitEvaluator 
 Returns the tip text for this property
   classifierTipText()   - 
Method in class weka.experiment. RegressionSplitEvaluator 
 Returns the tip text for this property
   classifierTipText()   - 
Method in class weka.filters.supervised.attribute. AddClassification 
 Returns the tip text for this property.
   classifierTipText()   - 
Method in class weka.filters.unsupervised.instance. RemoveMisclassified 
 Returns the tip text for this property
   ClassifierTree   - Class in  weka.classifiers.trees.j48  Class for handling a tree structure used for
 classification.   ClassifierTree(ModelSelection)   - 
Constructor for class weka.classifiers.trees.j48. ClassifierTree 
 Constructor.
   CLASSIFY_CHILD   - 
Static variable in class weka.gui.treevisualizer. TreeDisplayEvent 
 Asks for another learning scheme to classify this node.
   classifyInstance(Instance)   - 
Method in class weka.classifiers.bayes. BayesianLogisticRegression 
 Classifies the given instance using the Bayesian Logistic Regression function.
   classifyInstance(Instance)   - 
Method in class weka.classifiers.bayes. ComplementNaiveBayes 
 Classifies a given instance.
   classifyInstance(Instance)   - 
Method in class weka.classifiers. Classifier 
 Classifies the given test instance.
   classifyInstance(Instance)   - 
Method in class weka.classifiers.functions. GaussianProcesses 
 Classifies a given instance.
   classifyInstance(Instance)   - 
Method in class weka.classifiers.functions. IsotonicRegression 
 Generate a prediction for the supplied instance.
   classifyInstance(Instance)   - 
Method in class weka.classifiers.functions. LeastMedSq 
 Classify a given instance using the best generated
 LinearRegression Classifier.
   classifyInstance(Instance)   - 
Method in class weka.classifiers.functions. LinearRegression 
 Classifies the given instance using the linear regression function.
   classifyInstance(Instance)   - 
Method in class weka.classifiers.functions. PaceRegression 
 Classifies the given instance using the linear regression function.
   classifyInstance(Instance)   - 
Method in class weka.classifiers.functions. PLSClassifier 
 Classifies the given test instance.
   classifyInstance(Instance)   - 
Method in class weka.classifiers.functions. SimpleLinearRegression 
 Generate a prediction for the supplied instance.
   classifyInstance(Instance)   - 
Method in class weka.classifiers.functions. SMOreg 
 Classifies the given instance using the linear regression function.
   classifyInstance(Instance)   - 
Method in class weka.classifiers.functions. Winnow 
 Outputs the prediction for the given instance.
   classifyInstance(Instance)   - 
Method in class weka.classifiers.lazy. IB1 
 Classifies the given test instance.
   classifyInstance(Instance)   - 
Method in class weka.classifiers.meta. AdditiveRegression 
 Classify an instance.
   classifyInstance(Instance)   - 
Method in class weka.classifiers.meta. ClassificationViaClustering 
 Classifies the given test instance.
   classifyInstance(Instance)   - 
Method in class weka.classifiers.meta. GridSearch 
 Classifies the given instance.
   classifyInstance(Instance)   - 
Method in class weka.classifiers.meta. RegressionByDiscretization 
 Returns a predicted class for the test instance.
   classifyInstance(Instance)   - 
Method in class weka.classifiers.meta. Vote 
 Classifies the given test instance.
   classifyInstance(Instance)   - 
Method in class weka.classifiers.mi. MINND 
 Use Kullback Leibler distance to find the nearest neighbours of
 the given exemplar.
   classifyInstance(Instance)   - 
Method in class weka.classifiers.rules. NNge 
 Classifies a given instance.
   classifyInstance(Instance)   - 
Method in class weka.classifiers.rules. OneR 
 Classifies a given instance.
   classifyInstance(Instance)   - 
Method in class weka.classifiers.rules.part. ClassifierDecList 
 Classifies an instance.
   classifyInstance(Instance)   - 
Method in class weka.classifiers.rules. PART 
 Classifies an instance.
   classifyInstance(Instance)   - 
Method in class weka.classifiers.rules.part. MakeDecList 
 Classifies an instance.
   classifyInstance(Instance)   - 
Method in class weka.classifiers.rules. Prism 
 Classifies a given instance.
   classifyInstance(Instance)   - 
Method in class weka.classifiers.rules. Ridor 
 Classify the test instance with the rule learner
   classifyInstance(Instance)   - 
Method in class weka.classifiers.rules. ZeroR 
 Classifies a given instance.
   classifyInstance(Instance)   - 
Method in class weka.classifiers.trees. FT 
 Classifies an instance.
   classifyInstance(Instance)   - 
Method in class weka.classifiers.trees. Id3 
 Classifies a given test instance using the decision tree.
   classifyInstance(Instance)   - 
Method in class weka.classifiers.trees.j48. ClassifierSplitModel 
 Classifies a given instance.
   classifyInstance(Instance)   - 
Method in class weka.classifiers.trees.j48. ClassifierTree 
 Classifies an instance.
   classifyInstance(Instance)   - 
Method in class weka.classifiers.trees. J48 
 Classifies an instance.
   classifyInstance(Instance)   - 
Method in class weka.classifiers.trees. J48graft 
 Classifies an instance.
   classifyInstance(Instance)   - 
Method in class weka.classifiers.trees. LMT 
 Classifies an instance.
   classifyInstance(Instance)   - 
Method in class weka.classifiers.trees.m5. M5Base 
 Calculates a prediction for an instance using a set of rules
 or an M5 model tree
   classifyInstance(Instance)   - 
Method in class weka.classifiers.trees.m5. PreConstructedLinearModel 
 Predicts the class of the supplied instance using the linear model.
   classifyInstance(Instance)   - 
Method in class weka.classifiers.trees.m5. Rule 
 Calculates a prediction for an instance using this rule
 or M5 model tree
   classifyInstance(Instance)   - 
Method in class weka.classifiers.trees.m5. RuleNode 
 Classify an instance using this node.
   classifyInstance(Instance)   - 
Method in class weka.classifiers.trees. NBTree 
 Classifies an instance.
   ClassIndex   - 
Variable in class weka.classifiers.bayes. BayesianLogisticRegression 
 The class index from the training data
   classIndex()   - 
Method in class weka.core. Instance 
 Returns the class attribute's index.
   classIndex()   - 
Method in class weka.core. Instances 
 Returns the class attribute's index.
   classIndexTipText()   - 
Method in class weka.associations. Apriori 
 Returns the tip text for this property
   classIndexTipText()   - 
Method in class weka.associations. FilteredAssociator 
 Returns the tip text for this property
   classIndexTipText()   - 
Method in class weka.associations. PredictiveApriori 
 Returns the tip text for this property
   classIndexTipText()   - 
Method in class weka.associations. Tertius 
 Returns the tip text for this property.
   classIndexTipText()   - 
Method in class weka.core.converters. LibSVMSaver 
 Returns the tip text for this property
   classIndexTipText()   - 
Method in class weka.core.converters. SVMLightSaver 
 Returns the tip text for this property.
   classIndexTipText()   - 
Method in class weka.core.converters. XRFFSaver 
 Returns the tip text for this property
   classIndexTipText()   - 
Method in class weka.filters.unsupervised.attribute. ClassAssigner 
 Returns the tip text for this property.
   classIndexTipText()   - 
Method in class weka.filters.unsupervised.instance. RemoveMisclassified 
 Returns the tip text for this property
   classIsMissing()   - 
Method in class weka.core. Instance 
 Tests if an instance's class is missing.
   ClassloaderUtil   - Class in  weka.core  Utility class that can add jar files to the classpath dynamically.   ClassloaderUtil()   - 
Constructor for class weka.core. ClassloaderUtil 
  
   className(int)   - 
Method in class weka.classifiers.evaluation. ConfusionMatrix 
 Gets the name of one of the classes.
   classNameTipText()   - 
Method in class weka.filters.unsupervised.attribute. NumericTransform 
 Returns the tip text for this property
   ClassOrder   - Class in  weka.filters.supervised.attribute  Changes the order of the classes so that the class values are no longer of in the order specified in the header.   ClassOrder()   - 
Constructor for class weka.filters.supervised.attribute. ClassOrder 
  
   classOrderTipText()   - 
Method in class weka.filters.supervised.attribute. ClassOrder 
 Returns the tip text for this property
   ClassPanel   - Class in  weka.gui.visualize  This panel displays coloured labels for nominal attributes and a spectrum
 for numeric attributes.   ClassPanel()   - 
Constructor for class weka.gui.visualize. ClassPanel 
  
   ClassPanel(Color)   - 
Constructor for class weka.gui.visualize. ClassPanel 
  
   classProb(int, Instance, int)   - 
Method in class weka.classifiers.trees.j48. BinC45Split 
 Gets class probability for instance.
   classProb(int, Instance, int)   - 
Method in class weka.classifiers.trees.j48. C45Split 
 Gets class probability for instance.
   classProb(int, Instance, int)   - 
Method in class weka.classifiers.trees.j48. ClassifierSplitModel 
 Gets class probability for instance.
   classProb(int, Instance, int)   - 
Method in class weka.classifiers.trees.j48. GraftSplit 
 returns the probability for instance for the specified class
   classProb(int, Instance, int)   - 
Method in class weka.classifiers.trees.j48. NBTreeNoSplit 
 Return the probability for a class value
   classProb(int, Instance, int)   - 
Method in class weka.classifiers.trees.j48. NBTreeSplit 
 Return the probability for a class value
   classProbLaplace(int, Instance, int)   - 
Method in class weka.classifiers.trees.j48. ClassifierSplitModel 
 Gets class probability for instance.
   classSgn(double)   - 
Static method in class weka.classifiers.bayes. BayesianLogisticRegression 
 This class is used to mask the internal class labels.
   classValue()   - 
Method in class weka.core. Instance 
 Returns an instance's class value in internal format.
   ClassValuePicker   - Class in  weka.gui.beans      ClassValuePicker()   - 
Constructor for class weka.gui.beans. ClassValuePicker 
  
   ClassValuePickerBeanInfo   - Class in  weka.gui.beans  BeanInfo class for the class value picker bean   ClassValuePickerBeanInfo()   - 
Constructor for class weka.gui.beans. ClassValuePickerBeanInfo 
  
   ClassValuePickerCustomizer   - Class in  weka.gui.beans      ClassValuePickerCustomizer()   - 
Constructor for class weka.gui.beans. ClassValuePickerCustomizer 
  
   classValueTipText()   - 
Method in class weka.filters.supervised.instance. SMOTE 
 Returns the tip text for this property.
   clean()   - 
Method in class weka.classifiers.functions.supportVector. CachedKernel 
 Frees the cache used by the kernel.
   clean()   - 
Method in class weka.classifiers.functions.supportVector. Kernel 
 Frees the memory used by the kernel.
   clean()   - 
Method in class weka.classifiers.functions.supportVector. PrecomputedKernelMatrixKernel 
 Frees the memory used by the kernel.
   clean()   - 
Method in class weka.classifiers.functions.supportVector. StringKernel 
 Frees the memory used by the kernel.
   cleanse(Instance)   - 
Method in class weka.classifiers.mi. MINND 
 Cleanse the given exemplar according to the valid and noise data
 statistics
   cleanup(Instances)   - 
Method in class weka.classifiers.rules.part. ClassifierDecList 
 Cleanup in order to save memory.
   cleanUp()   - 
Method in class weka.classifiers.rules. RuleStats 
 Frees up memory after classifier has been built.
   cleanup()   - 
Method in class weka.classifiers.trees.ft. FTtree 
 Cleanup in order to save memory.
   cleanup()   - 
Method in class weka.classifiers.trees.j48. BinC45ModelSelection 
 Sets reference to training data to null.
   cleanup()   - 
Method in class weka.classifiers.trees.j48. C45ModelSelection 
 Sets reference to training data to null.
   cleanup(Instances)   - 
Method in class weka.classifiers.trees.j48. ClassifierTree 
 Cleanup in order to save memory.
   cleanup()   - 
Method in class weka.classifiers.trees.j48. NBTreeModelSelection 
 Sets reference to training data to null.
   cleanup()   - 
Method in class weka.classifiers.trees.lmt. LMTNode 
 Cleanup in order to save memory.
   cleanup()   - 
Method in class weka.classifiers.trees.lmt. LogisticBase 
 Cleanup in order to save memory.
   cleanup()   - 
Method in class weka.classifiers.trees.lmt. ResidualModelSelection 
 Method not in use
   clear()   - 
Method in class weka.associations.tertius. SimpleLinkedList 
  
   clear()   - 
Method in class weka.classifiers.lazy.kstar. KStarCache.CacheTable 
 Clears this hashtable so that it contains no keys.
   clear()   - 
Method in class weka.classifiers.xml. XMLClassifier 
 generates internally a new XML document and clears also the IgnoreList and
 the mappings for the Read/Write-Methods
   clear()   - 
Method in class weka.core.neighboursearch.covertrees. Stack 
 Removes all the elements from the stack.
   clear()   - 
Method in class weka.core. ProtectedProperties 
 Overrides a method to prevent the properties from being modified.
   clear()   - 
Method in class weka.core. Stopwords 
 removes all stopwords
   clear()   - 
Method in class weka.core. Tee 
 removes all streams and places the default printstream, if any, again in
 the list.
   clear()   - 
Method in class weka.core. Trie 
 Removes all of the elements from this collection
   clear()   - 
Method in class weka.core.xml. MethodHandler 
 removes all mappings
   clear()   - 
Method in class weka.core.xml. XMLBasicSerialization 
 generates internally a new XML document and clears also the IgnoreList
 and the mappings for the Read/Write-Methods
   clear()   - 
Method in class weka.core.xml. XMLDocument 
 sets up an empty DOM document, with the current DOCTYPE and root node.
   clear()   - 
Method in class weka.core.xml. XMLSerialization 
 generates internally a new XML document and clears also the IgnoreList and
 the mappings for the Read/Write-Methods
   clear()   - 
Method in class weka.core.xml. XMLSerializationMethodHandler 
 removes all current methods and adds the methods according to the
   clear()   - 
Method in class weka.experiment. ResultMatrix 
 removes the stored data and the ordering, but retains the dimensions of
 the matrix
   clear()   - 
Method in class weka.experiment. ResultMatrixCSV 
 removes the stored data but retains the dimensions of the matrix
   clear()   - 
Method in class weka.experiment. ResultMatrixGnuPlot 
 removes the stored data but retains the dimensions of the matrix
   clear()   - 
Method in class weka.experiment. ResultMatrixHTML 
 removes the stored data but retains the dimensions of the matrix
   clear()   - 
Method in class weka.experiment. ResultMatrixLatex 
 removes the stored data but retains the dimensions of the matrix
   clear()   - 
Method in class weka.experiment. ResultMatrixPlainText 
 removes the stored data but retains the dimensions of the matrix
   clear()   - 
Method in class weka.experiment. ResultMatrixSignificance 
 removes the stored data but retains the dimensions of the matrix
   clear()   - 
Method in class weka.experiment.xml. XMLExperiment 
 generates internally a new XML document and clears also the IgnoreList and
 the mappings for the Read/Write-Methods
   clear()   - 
Method in class weka.gui.beans.xml. XMLBeans 
 generates internally a new XML document and clears also the IgnoreList and
 the mappings for the Read/Write-Methods
   clear()   - 
Method in class weka.gui. LogWindow 
 clears the output
   clear()   - 
Method in class weka.gui.sql. ConnectionPanel 
 sets the parameters back to standard.
   clear()   - 
Method in class weka.gui.sql. InfoPanel 
 clears the content of the panel
   clear()   - 
Method in class weka.gui.sql. QueryPanel 
 clears the textarea.
   clear()   - 
Method in class weka.gui.sql. ResultPanel 
 sets the parameters back to standard
   clear()   - 
Method in class weka.gui.sql. SqlViewer 
 calls the clear method of all sub-panels to set back to default values
 and free up memory.
   clearCache()   - 
Static method in class weka.core. ClassDiscovery 
 clears the cache for class/classnames relation.
   clearHeader()   - 
Method in class weka.experiment. ResultMatrix 
 removes all the header information
   clearLayout()   - 
Method in class weka.gui.beans. KnowledgeFlowApp 
  
   clearRanking()   - 
Method in class weka.experiment. ResultMatrix 
 clears the currently stored ranking data
   clearRect(int, int, int, int)   - 
Method in class weka.gui.visualize. PostscriptGraphics 
 Draw a filled rectangle with the background color.
   clearResults()   - 
Method in class weka.gui. ResultHistoryPanel 
 Removes all of the result buffers from the history.
   clearSearch()   - 
Method in class weka.gui.arffviewer. ArffPanel 
 clears the search, i.e.
   clearSearch()   - 
Method in class weka.gui.arffviewer. ArffViewerMainPanel 
 clears the search, i.e.
   clearStatus()   - 
Method in class weka.gui.beans. LogPanel 
 Clear the status area.
   clearSummary()   - 
Method in class weka.experiment. ResultMatrix 
 clears the current summary data
   clearUndo()   - 
Method in interface weka.core. Undoable 
 removes the undo history
   clearUndo()   - 
Method in class weka.gui.arffviewer. ArffPanel 
 removes the undo history
   clearUndo()   - 
Method in class weka.gui.arffviewer. ArffSortedTableModel 
 removes the undo history
   clearUndo()   - 
Method in class weka.gui.arffviewer. ArffTableModel 
 removes the undo history
   clearUndoStack()   - 
Method in class weka.classifiers.bayes.net. EditableBayesNet 
 remove all actions from the undo stack
   clip(Shape)   - 
Method in class weka.gui.visualize. PostscriptGraphics 
  
   clipRect(int, int, int, int)   - 
Method in class weka.gui.visualize. PostscriptGraphics 
 Not implemented
   clone()   - 
Method in class weka.associations.gsp. Element 
 Returns a deep clone of an Element.
   clone()   - 
Method in class weka.associations.gsp. Sequence 
 Returns a deep clone of a Sequence.
   clone()   - 
Method in class weka.associations.tertius. LiteralSet 
 Returns a shallow copy of this set.
   clone()   - 
Method in class weka.associations.tertius. Rule 
 Returns a shallow copy of this rule.
   clone()   - 
Method in class weka.attributeSelection. ScatterSearchV1.Subset 
  
   clone()   - 
Method in class weka.classifiers.evaluation. ConfusionMatrix 
 Creates and returns a clone of this object.
   clone()   - 
Method in class weka.classifiers.functions.pace. DiscreteFunction 
 Clones the discrete function
   clone()   - 
Method in class weka.classifiers.functions.pace. PaceMatrix 
 Clone the PaceMatrix object.
   clone()   - 
Method in interface weka.classifiers. IterativeClassifier 
 Performs a deep copy of the classifier, and a reference copy
 of the training instances (or a deep copy if required).
   clone()   - 
Method in class weka.classifiers.trees. ADTree 
 Creates a clone that is identical to the current tree, but is independent.
   clone()   - 
Method in class weka.classifiers.trees.adtree. PredictionNode 
 Clones this node.
   clone()   - 
Method in class weka.classifiers.trees.adtree. Splitter 
 Clones this node.
   clone()   - 
Method in class weka.classifiers.trees.adtree. TwoWayNominalSplit 
 Clones this node.
   clone()   - 
Method in class weka.classifiers.trees.adtree. TwoWayNumericSplit 
 Clones this node.
   clone()   - 
Method in class weka.classifiers.trees.j48. ClassifierSplitModel 
 Allows to clone a model (shallow copy).
   clone()   - 
Method in class weka.classifiers.trees.j48. Distribution 
 Clones distribution (Deep copy of distribution).
   clone()   - 
Method in class weka.core. AlgVector 
 Creates and returns a clone of this object.
   clone()   - 
Method in class weka.core. Capabilities 
 Creates and returns a copy of this object.
   clone()   - 
Method in class weka.core. Matrix 
  Deprecated.  Creates and returns a clone of this object.
   clone()   - 
Method in class weka.core.matrix. DoubleVector 
 Clones the DoubleVector object.
   clone()   - 
Method in class weka.core.matrix. IntVector 
 Clones the IntVector object.
   clone()   - 
Method in class weka.core.matrix. Matrix 
 Clone the Matrix object.
   clone()   - 
Method in class weka.core. PropertyPath.PathElement 
 returns a clone of the current object
   clone()   - 
Method in class weka.core. TestInstances 
 creates a clone of the current object
   clone()   - 
Method in class weka.core. Trie 
 returns a deep copy of itself
   clone()   - 
Method in class weka.core. Trie.TrieNode 
 creates a deep copy of itself
   clone(Estimator)   - 
Static method in class weka.estimators. Estimator 
 Creates a deep copy of the given estimator using serialization.
   CLOPE   - Class in  weka.clusterers  Yiling Yang, Xudong Guan, Jinyuan You: CLOPE: a fast and effective clustering algorithm for transactional data.   CLOPE()   - 
Constructor for class weka.clusterers. CLOPE 
 the default constructor
   close(ResultSet)   - 
Method in class weka.experiment. DatabaseUtils 
 closes the ResultSet and the statement that generated the ResultSet to
 avoid memory leaks in JDBC drivers - in contrast to the JDBC specs, a lot
 of JDBC drives don't clean up correctly.
   close()   - 
Method in class weka.experiment. DatabaseUtils 
 closes the m_PreparedStatement to avoid memory leaks.
   close()   - 
Method in class weka.gui.arffviewer. ArffViewerMainPanel 
 closes the window, i.e., if the parent is not null and implements
 the WindowListener interface it calls the windowClosing method
   close()   - 
Method in class weka.gui. LogWindow 
 closes the frame
   closeAllFiles()   - 
Method in class weka.gui.arffviewer. ArffViewerMainPanel 
 closes all open files
   closeFile()   - 
Method in class weka.gui.arffviewer. ArffViewerMainPanel 
 closes the current tab
   closeFile(boolean)   - 
Method in class weka.gui.arffviewer. ArffViewerMainPanel 
 closes the current tab
   closeFrame()   - 
Method in class weka.gui. SetInstancesPanel 
 closes the frame, i.e., the visibility is set to false
   closestPoint(Instance, Instances, int[])   - 
Method in class weka.core. EuclideanDistance 
 Returns the index of the closest point to the current instance.
   closeToDefaultTipText()   - 
Method in class weka.filters.unsupervised.attribute. NumericCleaner 
 Returns the tip text for this property
   closeToTipText()   - 
Method in class weka.filters.unsupervised.attribute. NumericCleaner 
 Returns the tip text for this property
   closeToToleranceTipText()   - 
Method in class weka.filters.unsupervised.attribute. NumericCleaner 
 Returns the tip text for this property
   ClusterDefinition   - Class in  weka.datagenerators  Ancestor to all ClusterDefinitions, i.e., subclasses that handle their
 own parameters that the cluster generator only passes on.   ClusterDefinition()   - 
Constructor for class weka.datagenerators. ClusterDefinition 
 initializes the cluster, without a parent cluster (necessary for GOE)
   ClusterDefinition(ClusterGenerator)   - 
Constructor for class weka.datagenerators. ClusterDefinition 
 initializes the cluster
   clusterDefinitionsTipText()   - 
Method in class weka.datagenerators.clusterers. SubspaceCluster 
 Returns the tip text for this property
   Clusterer   - Interface in  weka.clusterers  Interface for clusterers.   Clusterer   - Class in  weka.gui.beans  Bean that wraps around weka.clusterers   Clusterer()   - 
Constructor for class weka.gui.beans. Clusterer 
 Creates a new  Clusterer  instance.
   ClustererBeanInfo   - Class in  weka.gui.beans  BeanInfo class for the Clusterer wrapper bean   ClustererBeanInfo()   - 
Constructor for class weka.gui.beans. ClustererBeanInfo 
  
   ClustererCustomizer   - Class in  weka.gui.beans  GUI customizer for the Clusterer wrapper bean   ClustererCustomizer()   - 
Constructor for class weka.gui.beans. ClustererCustomizer 
  
   ClustererPanel   - Class in  weka.gui.explorer  This panel allows the user to select and configure a clusterer, and evaluate
 the clusterer using a number of testing modes (test on the training data,
 train/test on a percentage split, test on a
 separate split).   ClustererPanel()   - 
Constructor for class weka.gui.explorer. ClustererPanel 
 Creates the clusterer panel
   ClustererPerformanceEvaluator   - Class in  weka.gui.beans  A bean that evaluates the performance of batch trained clusterers   ClustererPerformanceEvaluator()   - 
Constructor for class weka.gui.beans. ClustererPerformanceEvaluator 
  
   ClustererPerformanceEvaluatorBeanInfo   - Class in  weka.gui.beans  Bean info class for the clusterer performance evaluator   ClustererPerformanceEvaluatorBeanInfo()   - 
Constructor for class weka.gui.beans. ClustererPerformanceEvaluatorBeanInfo 
  
   clustererTipText()   - 
Method in class weka.classifiers.meta. ClassificationViaClustering 
 Returns the tip text for this property
   clustererTipText()   - 
Method in class weka.clusterers. MakeDensityBasedClusterer 
 Returns the tip text for this property
   clustererTipText()   - 
Method in class weka.clusterers. SingleClustererEnhancer 
 Returns the tip text for this property
   clustererTipText()   - 
Method in class weka.experiment. DensityBasedClustererSplitEvaluator 
 Returns the tip text for this property
   clustererTipText()   - 
Method in class weka.filters.unsupervised.attribute. AddCluster 
 Returns the tip text for this property
   ClusterEvaluation   - Class in  weka.clusterers  Class for evaluating clustering models. 

 Valid options are:  

 -t name of the training file  
 Specify the training file.   ClusterEvaluation()   - 
Constructor for class weka.clusterers. ClusterEvaluation 
 Constructor.
   ClusterGenerator   - Class in  weka.datagenerators  Abstract class for cluster data generators.   ClusterGenerator()   - 
Constructor for class weka.datagenerators. ClusterGenerator 
 initializes the generator
   clusteringSeedTipText()   - 
Method in class weka.classifiers.functions. RBFNetwork 
 Returns the tip text for this property
   clusterInstance(Instance)   - 
Method in class weka.clusterers. AbstractClusterer 
 Classifies a given instance.
   clusterInstance(Instance)   - 
Method in class weka.clusterers. CLOPE 
 Classifies a given instance.
   clusterInstance(Instance)   - 
Method in interface weka.clusterers. Clusterer 
 Classifies a given instance.
   clusterInstance(Instance)   - 
Method in class weka.clusterers. Cobweb 
 Classifies a given instance.
   clusterInstance(Instance)   - 
Method in class weka.clusterers. DBScan 
 Classifies a given instance.
   clusterInstance(Instance)   - 
Method in class weka.clusterers. FarthestFirst 
 Classifies a given instance.
   clusterInstance(Instance)   - 
Method in class weka.clusterers. HierarchicalClusterer 
  
   clusterInstance(Instance)   - 
Method in class weka.clusterers. OPTICS 
 Classifies a given instance.
   clusterInstance(Instance)   - 
Method in class weka.clusterers. sIB 
 Cluster a given instance, this is the method defined in Clusterer
 interface do nothing but just return the cluster assigned to it
   clusterInstance(Instance)   - 
Method in class weka.clusterers. SimpleKMeans 
 Classifies a given instance.
   clusterInstance(Instance)   - 
Method in class weka.clusterers. XMeans 
 Classifies a given instance.
   ClusterMembership   - Class in  weka.filters.unsupervised.attribute  A filter that uses a density-based clusterer to generate cluster membership values; filtered instances are composed of these values plus the class attribute (if set in the input data).   ClusterMembership()   - 
Constructor for class weka.filters.unsupervised.attribute. ClusterMembership 
  
   clusterPriors()   - 
Method in class weka.clusterers. AbstractDensityBasedClusterer 
 Returns the prior probability of each cluster.
   clusterPriors()   - 
Method in interface weka.clusterers. DensityBasedClusterer 
 Returns the prior probability of each cluster.
   clusterPriors()   - 
Method in class weka.clusterers. EM 
 Returns the cluster priors.
   clusterPriors()   - 
Method in class weka.clusterers. MakeDensityBasedClusterer 
 Returns the cluster priors.
   clusterResultsToString()   - 
Method in class weka.clusterers. ClusterEvaluation 
 return the results of clustering.
   clusters   - 
Variable in class weka.clusterers. CLOPE 
 Array of clusters
   clusterSubTypeTipText()   - 
Method in class weka.datagenerators.clusterers. SubspaceClusterDefinition 
 Returns the tip text for this property
   clusterTypeTipText()   - 
Method in class weka.datagenerators.clusterers. SubspaceClusterDefinition 
 Returns the tip text for this property
   Cobweb   - Class in  weka.clusterers  Class implementing the Cobweb and Classit clustering algorithms. 
  
 Note: the application of node operators (merging, splitting etc.) in terms of ordering and priority differs (and is somewhat ambiguous) between the original Cobweb and Classit papers.   Cobweb()   - 
Constructor for class weka.clusterers. Cobweb 
 default constructor
   cochransCriterion(double[][])   - 
Static method in class weka.core. ContingencyTables 
 Tests if Cochran's criterion is fullfilled for the given
 contingency table.
   codingCost()   - 
Method in class weka.classifiers.trees.j48. C45Split 
 Returns coding cost for split (used in rule learner).
   codingCost()   - 
Method in class weka.classifiers.trees.j48. ClassifierSplitModel 
 Returns coding costs of model.
   coef0TipText()   - 
Method in class weka.classifiers.functions. LibSVM 
 Returns the tip text for this property
   coefficients()   - 
Method in class weka.classifiers.functions. LinearRegression 
 Returns the coefficients for this linear model.
   coefficients()   - 
Method in class weka.classifiers.functions. Logistic 
 Returns the coefficients for this logistic model.
   coefficients()   - 
Method in class weka.classifiers.functions. PaceRegression 
 Returns the coefficients for this linear model.
   coefficients()   - 
Method in class weka.classifiers.trees.m5. PreConstructedLinearModel 
 Return the array of coefficients
   collapse()   - 
Method in class weka.classifiers.trees.j48. C45PruneableClassifierTree 
 Collapses a tree to a node if training error doesn't increase.
   collapse()   - 
Method in class weka.classifiers.trees.j48. C45PruneableClassifierTreeG 
 Collapses a tree to a node if training error doesn't increase.
   COLOR_STDERR   - 
Static variable in class weka.gui. LogWindow 
 the Color of the style for stderr
   COLOR_STDOUT   - 
Static variable in class weka.gui. LogWindow 
 the color of the style for stdout
   Colors   - Class in  weka.gui.treevisualizer  This class maintains a list that contains all the colornames from the 
 dotty standard and what color (in RGB) they represent   Colors()   - 
Constructor for class weka.gui.treevisualizer. Colors 
  
   columnResponseExplanation(PaceMatrix, IntVector, int, int)   - 
Method in class weka.classifiers.functions.pace. PaceMatrix 
 Returns the squared ks-th response value if the j-th column becomes
  the ks-th after orthogonal transformation.
   combinationRuleTipText()   - 
Method in class weka.classifiers.meta. Vote 
 Returns the tip text for this property
   combinations(int, int)   - 
Static method in class weka.classifiers.functions. LeastMedSq 
 Produces the combination nCr
   combinationTipText()   - 
Method in class weka.attributeSelection. ScatterSearchV1 
 Returns the tip text for this property
   combinedDL(double, double)   - 
Method in class weka.classifiers.rules. RuleStats 
 Compute the combined DL of the ruleset in this class, i.e.
   CombineParents()   - 
Method in class weka.attributeSelection. ScatterSearchV1 
 Combine all the posible pair solutions existing in the Population
   combSort11(double[], int[])   - 
Static method in class weka.core.neighboursearch. NearestNeighbourSearch 
 sorts the two given arrays.
   COMMA   - 
Static variable in interface weka.core.mathematicalexpression. sym 
  
   COMMA   - 
Static variable in interface weka.filters.unsupervised.instance.subsetbyexpression. sym 
  
   compactify()   - 
Method in class weka.core. Instances 
 Compactifies the set of instances.
   compare(Object, Object)   - 
Method in class weka.core. ClassDiscovery.StringCompare 
 Compares its two arguments for order.
   compare(Object, Object)   - 
Method in class weka.core. InstanceComparator 
 compares the two instances, returns -1 if o1 is smaller than o2, 0
 if equal and +1 if greater.
   compareTo(FPGrowth.AssociationRule)   - 
Method in class weka.associations. FPGrowth.AssociationRule 
 Compare this rule to the supplied rule.
   compareTo(FPGrowth.BinaryItem)   - 
Method in class weka.associations. FPGrowth.BinaryItem 
 Ensures that items will be sorted in descending order of frequency.
   compareTo(Object)   - 
Method in class weka.associations. RuleItem 
 compares two RuleItems and allows an ordering concerning
 expected predictive accuracy and time of generation
 Note: this class has a natural ordering that is inconsistent with equals
   compareTo(Object)   - 
Method in class weka.classifiers.trees.j48. GraftSplit 
 method needed for sorting a collection of GraftSplits by laplace value
   compareTo(AttributeLocator)   - 
Method in class weka.core. AttributeLocator 
 Compares this object with the specified object for order.
   compareTo(Object)   - 
Method in class weka.core. Version 
 checks the version of this class against the given version-string
   compareTo(SortedTableModel.SortContainer)   - 
Method in class weka.gui. SortedTableModel.SortContainer 
 Compares this object with the specified object for order.
   comparisonString(int, Instances)   - 
Method in class weka.classifiers.trees.adtree. Splitter 
 Gets the string describing the comparision the split depends on for a particular
 branch.
   comparisonString(int, Instances)   - 
Method in class weka.classifiers.trees.adtree. TwoWayNominalSplit 
 Gets the string describing the comparision the split depends on for a particular
 branch.
   comparisonString(int, Instances)   - 
Method in class weka.classifiers.trees.adtree. TwoWayNumericSplit 
 Gets the string describing the comparision the split depends on for a particular
 branch.
   ComplementNaiveBayes   - Class in  weka.classifiers.bayes  Class for building and using a Complement class Naive Bayes classifier. 
  
 For more information see,  
  
 Jason D.   ComplementNaiveBayes()   - 
Constructor for class weka.classifiers.bayes. ComplementNaiveBayes 
  
   complexityParameterTipText()   - 
Method in class weka.attributeSelection. SVMAttributeEval 
 Returns a tip text for this property suitable for display in the
 GUI
   ComponentHelper   - Class in  weka.gui  A helper class for some common tasks with Dialogs, Icons, etc.   ComponentHelper()   - 
Constructor for class weka.gui. ComponentHelper 
  
   componentHidden(ComponentEvent)   - 
Method in class weka.gui.hierarchyvisualizer. HierarchyVisualizer 
  
   componentMoved(ComponentEvent)   - 
Method in class weka.gui.hierarchyvisualizer. HierarchyVisualizer 
  
   componentResized(ComponentEvent)   - 
Method in class weka.gui.hierarchyvisualizer. HierarchyVisualizer 
  
   componentShown(ComponentEvent)   - 
Method in class weka.gui.hierarchyvisualizer. HierarchyVisualizer 
  
   compressOutputTipText()   - 
Method in class weka.core.converters. ArffSaver 
 Returns the tip text for this property
   compressOutputTipText()   - 
Method in class weka.core.converters. XRFFSaver 
 Returns the tip text for this property
   Compute   - Interface in  weka.experiment  Interface to something that can accept remote connections and execute
 a task.   computeLoglikelihood(double[], Instances)   - 
Method in class weka.classifiers.bayes.blr. GaussianPriorImpl 
 This method calls the log-likelihood implemented in the Prior
 abstract class.
   computeLogLikelihood(double[], Instances)   - 
Method in class weka.classifiers.bayes.blr. LaplacePriorImpl 
 Computes the log-likelihood values using the implementation in the Prior class.
   computelogLikelihood(double[], Instances)   - 
Method in class weka.classifiers.bayes.blr. Prior 
 Function computes the log-likelihood value:
 -sum{1 to n}{ln(1+exp(-Beta*x(i)*y(i))}
   computeMinMaxAtts()   - 
Method in class weka.gui.boundaryvisualizer. BoundaryPanel 
 Set up the bounds of our graphic based by finding the smallest reasonable
      area in the instance space to surround our data points.
   computePenalty(double[], double[])   - 
Method in class weka.classifiers.bayes.blr. GaussianPriorImpl 
 This function computes the penalty term specific to Gaussian distribution.
   computePenalty(double[], double[])   - 
Method in class weka.classifiers.bayes.blr. LaplacePriorImpl 
 This function computes the penalty term specific to Laplacian distribution.
   computePenalty(double[], double[])   - 
Method in class weka.classifiers.bayes.blr. Prior 
 Skeleton function to compute penalty terms.
   cond()   - 
Method in class weka.core.matrix. Matrix 
 Matrix condition (2 norm)
   cond()   - 
Method in class weka.core.matrix. SingularValueDecomposition 
 Two norm condition number
   ConditionalEstimator   - Interface in  weka.estimators  Interface for conditional probability estimators.   confidenceFactorTipText()   - 
Method in class weka.classifiers.rules. PART 
 Returns the tip text for this property
   confidenceFactorTipText()   - 
Method in class weka.classifiers.trees. J48 
 Returns the tip text for this property
   confidenceFactorTipText()   - 
Method in class weka.classifiers.trees. J48graft 
 Returns the tip text for this property
   confidenceForRule(AprioriItemSet, AprioriItemSet)   - 
Static method in class weka.associations. AprioriItemSet 
 Outputs the confidence for a rule.
   CONFIG   - 
Static variable in class weka.core. Debug 
 the log level Vonfig
   confirmationComparator   - 
Static variable in class weka.associations.tertius. Rule 
 Comparator used to compare two rules according to their confirmation value.
   confirmationThenObservedComparator   - 
Static variable in class weka.associations.tertius. Rule 
 Comparator used to compare two rules according to their confirmation and 
 then their observed number of counter-instances.
   confirmationThresholdTipText()   - 
Method in class weka.associations. Tertius 
 Returns the tip text for this property.
   confirmationValuesTipText()   - 
Method in class weka.associations. Tertius 
 Returns the tip text for this property.
   ConfusionMatrix   - Class in  weka.classifiers.evaluation  Cells of this matrix correspond to counts of the number (or weight)
 of predictions for each actual value / predicted value combination.   confusionMatrix()   - 
Method in class weka.classifiers. Evaluation 
 Returns a copy of the confusion matrix.
   ConfusionMatrix(String[])   - 
Constructor for class weka.classifiers.evaluation. ConfusionMatrix 
 Creates the confusion matrix with the given class names.
   ConjunctiveRule   - Class in  weka.classifiers.rules  This class implements a single conjunctive rule learner that can predict for numeric and nominal class labels. 
  
 A rule consists of antecedents "AND"ed together and the consequent (class value) for the classification/regression.   ConjunctiveRule()   - 
Constructor for class weka.classifiers.rules. ConjunctiveRule 
  
   connect(NeuralConnection, NeuralConnection)   - 
Static method in class weka.classifiers.functions.neural. NeuralConnection 
 Connects two units together.
   CONNECT   - 
Static variable in class weka.gui.sql.event. ConnectionEvent 
 it was a connect try
   CONNECTED   - 
Static variable in class weka.classifiers.functions.neural. NeuralConnection 
 This flag is set once the unit has a connection.
   connectionAllowed(EventSetDescriptor)   - 
Method in class weka.gui.beans. AbstractDataSink 
 Returns true if, at this time, 
 the object will accept a connection according to the supplied
 EventSetDescriptor
   connectionAllowed(String)   - 
Method in class weka.gui.beans. AbstractDataSink 
 Returns true if, at this time, 
 the object will accept a connection according to the supplied
 event name
   connectionAllowed(String)   - 
Method in class weka.gui.beans. AbstractEvaluator 
 Returns true if, at this time, 
 the object will accept a connection according to the supplied
 event name
   connectionAllowed(EventSetDescriptor)   - 
Method in class weka.gui.beans. AbstractEvaluator 
 Returns true if, at this time, 
 the object will accept a connection according to the supplied
 EventSetDescriptor
   connectionAllowed(String)   - 
Method in class weka.gui.beans. AbstractTestSetProducer 
 Returns true if, at this time, 
 the object will accept a connection according to the supplied
 event name
   connectionAllowed(EventSetDescriptor)   - 
Method in class weka.gui.beans. AbstractTestSetProducer 
 Returns true if, at this time, 
 the object will accept a connection according to the supplied
 EventSetDescriptor
   connectionAllowed(String)   - 
Method in class weka.gui.beans. AbstractTrainAndTestSetProducer 
 Returns true if, at this time, 
 the object will accept a connection according to the supplied
 event name
   connectionAllowed(EventSetDescriptor)   - 
Method in class weka.gui.beans. AbstractTrainAndTestSetProducer 
 Returns true if, at this time, 
 the object will accept a connection according to the supplied
 EventSetDescriptor
   connectionAllowed(String)   - 
Method in class weka.gui.beans. AbstractTrainingSetProducer 
 Returns true if, at this time, 
 the object will accept a connection according to the supplied
 event name
   connectionAllowed(EventSetDescriptor)   - 
Method in class weka.gui.beans. AbstractTrainingSetProducer 
 Returns true if, at this time, 
 the object will accept a connection according to the supplied
 EventSetDescriptor
   connectionAllowed(String)   - 
Method in class weka.gui.beans. Associator 
 Returns true if, at this time, 
 the object will accept a connection with respect to the named event
   connectionAllowed(EventSetDescriptor)   - 
Method in class weka.gui.beans. Associator 
 Returns true if, at this time, 
 the object will accept a connection according to the supplied
 EventSetDescriptor
   connectionAllowed(EventSetDescriptor)   - 
Method in interface weka.gui.beans. BeanCommon 
 Returns true if, at this time, 
 the object will accept a connection via the supplied
 EventSetDescriptor
   connectionAllowed(String)   - 
Method in interface weka.gui.beans. BeanCommon 
 Returns true if, at this time, 
 the object will accept a connection via the named event
   connectionAllowed(String)   - 
Method in class weka.gui.beans. ClassAssigner 
 Returns true if, at this time, 
 the object will accept a connection according to the supplied
 event name
   connectionAllowed(EventSetDescriptor)   - 
Method in class weka.gui.beans. ClassAssigner 
 Returns true if, at this time, 
 the object will accept a connection according to the supplied
 EventSetDescriptor
   connectionAllowed(String)   - 
Method in class weka.gui.beans. Classifier 
 Returns true if, at this time, 
 the object will accept a connection with respect to the named event
   connectionAllowed(EventSetDescriptor)   - 
Method in class weka.gui.beans. Classifier 
 Returns true if, at this time, 
 the object will accept a connection according to the supplied
 EventSetDescriptor
   connectionAllowed(String)   - 
Method in class weka.gui.beans. ClassValuePicker 
 Returns true if, at this time, 
 the object will accept a connection according to the supplied
 event name
   connectionAllowed(EventSetDescriptor)   - 
Method in class weka.gui.beans. ClassValuePicker 
 Returns true if, at this time, 
 the object will accept a connection according to the supplied
 EventSetDescriptor
   connectionAllowed(String)   - 
Method in class weka.gui.beans. Clusterer 
 Returns true if, at this time, 
 the object will accept a connection with respect to the named event
   connectionAllowed(EventSetDescriptor)   - 
Method in class weka.gui.beans. Clusterer 
 Returns true if, at this time, 
 the object will accept a connection according to the supplied
 EventSetDescriptor
   connectionAllowed(String)   - 
Method in class weka.gui.beans. CostBenefitAnalysis 
 Returns true if, at this time, 
 the object will accept a connection via the named event
   connectionAllowed(EventSetDescriptor)   - 
Method in class weka.gui.beans. CostBenefitAnalysis 
 Returns true if, at this time, 
 the object will accept a connection according to the supplied
 EventSetDescriptor
   connectionAllowed(String)   - 
Method in class weka.gui.beans. Filter 
 Returns true if, at this time, 
 the object will accept a connection with respect to the supplied
 event name
   connectionAllowed(EventSetDescriptor)   - 
Method in class weka.gui.beans. Filter 
 Returns true if, at this time, 
 the object will accept a connection according to the supplied
 EventSetDescriptor
   connectionAllowed(EventSetDescriptor)   - 
Method in class weka.gui.beans. InstanceStreamToBatchMaker 
 Returns true if, at this time, 
 the object will accept a connection according to the supplied
 EventSetDescriptor
   connectionAllowed(String)   - 
Method in class weka.gui.beans. InstanceStreamToBatchMaker 
 Returns true if, at this time, 
 the object will accept a connection with respect to the named event
   connectionAllowed(EventSetDescriptor)   - 
Method in class weka.gui.beans. Loader 
 Returns true if, at this time, 
 the object will accept a connection via the supplied
 EventSetDescriptor.
   connectionAllowed(String)   - 
Method in class weka.gui.beans. Loader 
 Returns true if, at this time, 
 the object will accept a connection via the named event
   connectionAllowed(EventSetDescriptor)   - 
Method in class weka.gui.beans. MetaBean 
 Returns true if, at this time, 
 the object will accept a connection with respect to the
 supplied EventSetDescriptor
   connectionAllowed(String)   - 
Method in class weka.gui.beans. MetaBean 
  
   connectionAllowed(String)   - 
Method in class weka.gui.beans. PredictionAppender 
 Returns true if, at this time, 
 the object will accept a connection according to the supplied
 event name
   connectionAllowed(EventSetDescriptor)   - 
Method in class weka.gui.beans. PredictionAppender 
 Returns true if, at this time, 
 the object will accept a connection according to the supplied
 EventSetDescriptor
   connectionAllowed(EventSetDescriptor)   - 
Method in class weka.gui.beans. SerializedModelSaver 
 Returns true if, at this time, 
 the object will accept a connection according to the supplied
 EventSetDescriptor.
   connectionAllowed(String)   - 
Method in class weka.gui.beans. SerializedModelSaver 
 Returns true if, at this time, 
 the object will accept a connection according to the supplied
 event name.
   connectionAllowed(String)   - 
Method in class weka.gui.beans. StripChart 
 Returns true if, at this time,
 the object will accept a connection via the named event
   connectionAllowed(EventSetDescriptor)   - 
Method in class weka.gui.beans. StripChart 
 Returns true if, at this time,
 the object will accept a connection according to the supplied
 EventSetDescriptor
   connectionAllowed(EventSetDescriptor)   - 
Method in class weka.gui.beans. TextViewer 
 Returns true if, at this time, 
 the object will accept a connection via the supplied
 EventSetDescriptor
   connectionAllowed(String)   - 
Method in class weka.gui.beans. TextViewer 
 Returns true if, at this time, 
 the object will accept a connection via the named event
   connectionChange(ConnectionEvent)   - 
Method in interface weka.gui.sql.event. ConnectionListener 
 This method gets called when the connection is either established
 or disconnected.
   connectionChange(ConnectionEvent)   - 
Method in class weka.gui.sql. QueryPanel 
 This method gets called when the connection is either established
 or disconnected.
   connectionChange(ConnectionEvent)   - 
Method in class weka.gui.sql. SqlViewer 
 This method gets called when the connection is either established
 or disconnected.
   ConnectionEvent   - Class in  weka.gui.sql.event  An event that is generated when a connection is established or dropped.   ConnectionEvent(Object, int, DbUtils)   - 
Constructor for class weka.gui.sql.event. ConnectionEvent 
 constructs the event
   ConnectionEvent(Object, int, DbUtils, Exception)   - 
Constructor for class weka.gui.sql.event. ConnectionEvent 
 constructs the event
   ConnectionListener   - Interface in  weka.gui.sql.event  A listener for connect/disconnect events.   connectionNotification(String, Object)   - 
Method in class weka.gui.beans. AbstractDataSink 
 Notify this object that it has been registered as a listener with
 a source with respect to the supplied event name
   connectionNotification(String, Object)   - 
Method in class weka.gui.beans. AbstractEvaluator 
 Notify this object that it has been registered as a listener with
 a source with respect to the supplied event name
   connectionNotification(String, Object)   - 
Method in class weka.gui.beans. AbstractTestSetProducer 
 Notify this object that it has been registered as a listener with
 a source with respect to the supplied event name
   connectionNotification(String, Object)   - 
Method in class weka.gui.beans. AbstractTrainAndTestSetProducer 
 Notify this object that it has been registered as a listener with
 a source with respect to the supplied event name
   connectionNotification(String, Object)   - 
Method in class weka.gui.beans. AbstractTrainingSetProducer 
 Notify this object that it has been registered as a listener with
 a source with respect to the supplied event name
   connectionNotification(String, Object)   - 
Method in class weka.gui.beans. Associator 
 Notify this object that it has been registered as a listener with
 a source with respect to the named event
   connectionNotification(String, Object)   - 
Method in interface weka.gui.beans. BeanCommon 
 Notify this object that it has been registered as a listener with
 a source for recieving events described by the named event
 This object is responsible for recording this fact.
   connectionNotification(String, Object)   - 
Method in class weka.gui.beans. ClassAssigner 
 Notify this object that it has been registered as a listener with
 a source with respect to the supplied event name
   connectionNotification(String, Object)   - 
Method in class weka.gui.beans. Classifier 
 Notify this object that it has been registered as a listener with
 a source with respect to the named event
   connectionNotification(String, Object)   - 
Method in class weka.gui.beans. ClassValuePicker 
 Notify this object that it has been registered as a listener with
 a source with respect to the supplied event name
   connectionNotification(String, Object)   - 
Method in class weka.gui.beans. Clusterer 
 Notify this object that it has been registered as a listener with
 a source with respect to the named event
   connectionNotification(String, Object)   - 
Method in interface weka.gui.beans. ConnectionNotificationConsumer 
 Notify this object that it has been registered as a listener with a source
 with respect to the supplied event name.
   connectionNotification(String, Object)   - 
Method in class weka.gui.beans. CostBenefitAnalysis 
 Notify this object that it has been registered as a listener with
 a source for recieving events described by the named event
 This object is responsible for recording this fact.
   connectionNotification(String, Object)   - 
Method in class weka.gui.beans. Filter 
 Notify this object that it has been registered as a listener with
 a source with respect to the supplied event name
   connectionNotification(String, Object)   - 
Method in class weka.gui.beans. InstanceStreamToBatchMaker 
 Notify this object that it has been registered as a listener with
 a source with respect to the named event
   connectionNotification(String, Object)   - 
Method in class weka.gui.beans. Loader 
 Notify this object that it has been registered as a listener with
 a source for receiving events described by the named event
 This object is responsible for recording this fact.
   connectionNotification(String, Object)   - 
Method in class weka.gui.beans. MetaBean 
 Notify this object that it has been registered as a listener with
 a source with respect to the named event.
   connectionNotification(String, Object)   - 
Method in class weka.gui.beans. PredictionAppender 
 Notify this object that it has been registered as a listener with
 a source with respect to the supplied event name
   connectionNotification(String, Object)   - 
Method in class weka.gui.beans. SerializedModelSaver 
 Notify this object that it has been registered as a listener with
 a source with respect to the supplied event name.
   connectionNotification(String, Object)   - 
Method in class weka.gui.beans. StripChart 
 Notify this object that it has been registered as a listener with
 a source for recieving events described by the named event
 This object is responsible for recording this fact.
   connectionNotification(String, Object)   - 
Method in class weka.gui.beans. TextViewer 
 Notify this object that it has been registered as a listener with
 a source for recieving events described by the named event
 This object is responsible for recording this fact.
   ConnectionNotificationConsumer   - Interface in  weka.gui.beans  Interface for Beans that can receive (dis-)connection events generated when
 (dis-)connecting data processing nodes in the Weka KnowledgeFlow.   ConnectionPanel   - Class in  weka.gui.sql  Enables the user to insert a database URL, plus user/password to connect
 to this database.   ConnectionPanel(JFrame)   - 
Constructor for class weka.gui.sql. ConnectionPanel 
 initializes the panel.
   CONNECTIONS   - 
Static variable in class weka.gui.beans. BeanConnection 
 The list of connections
   connectToDatabase()   - 
Method in class weka.core.converters. DatabaseLoader 
 Opens a connection to the database
   connectToDatabase()   - 
Method in class weka.core.converters. DatabaseSaver 
 Opens a connection to the database.
   connectToDatabase()   - 
Method in class weka.experiment. DatabaseUtils 
 Opens a connection to the database.
   consequence()   - 
Method in class weka.associations. RuleItem 
 Gets the consequence of a rule
   conservativeForwardSelectionTipText()   - 
Method in class weka.attributeSelection. GreedyStepwise 
 Returns the tip text for this property
   ConsistencySubsetEval   - Class in  weka.attributeSelection  ConsistencySubsetEval : 
  
 Evaluates the worth of a subset of attributes by the level of consistency in the class values when the training instances are projected onto the subset of attributes.   ConsistencySubsetEval()   - 
Constructor for class weka.attributeSelection. ConsistencySubsetEval 
 Constructor.
   ConsistencySubsetEval.hashKey   - Class in  weka.attributeSelection  Class providing keys to the hash table.   ConsistencySubsetEval.hashKey(Instance, int)   - 
Constructor for class weka.attributeSelection. ConsistencySubsetEval.hashKey 
 Constructor for a hashKey
   ConsistencySubsetEval.hashKey(double[])   - 
Constructor for class weka.attributeSelection. ConsistencySubsetEval.hashKey 
 Constructor for a hashKey
   ConsoleLogger   - Class in  weka.core.logging  A simple logger that outputs the logging information in the console.   ConsoleLogger()   - 
Constructor for class weka.core.logging. ConsoleLogger 
  
   CONST_AUTOMATIC_SHAPE   - 
Static variable in class weka.gui.visualize. Plot2D 
  
   Constant   - Class in  weka.core.pmml  Class encapsulating a Constant Expression.   Constant(Element, FieldMetaInfo.Optype, ArrayList<Attribute>)   - 
Constructor for class weka.core.pmml. Constant 
 Construct an new Constant Expression.
   constructWithCopy(double[][])   - 
Static method in class weka.core.matrix. Matrix 
 Construct a matrix from a copy of a 2-D array.
   containChildBallsTipText()   - 
Method in class weka.core.neighboursearch.balltrees. BallTreeConstructor 
 Returns the tip text for this property.
   containedBy(Instance)   - 
Method in class weka.associations. ItemSet 
 Checks if an instance contains an item set.
   contains(Literal)   - 
Method in class weka.associations.tertius. LiteralSet 
 Test if this LiteralSet contains a given Literal.
   contains(int)   - 
Method in class weka.classifiers.bayes.net. ParentSet 
 test if node is contained in parent set
   contains(int)   - 
Method in class weka.classifiers.functions.supportVector. SMOset 
 Checks whether an element is in the set.
   contains(DataObject)   - 
Method in interface weka.clusterers.forOPTICSAndDBScan.Databases. Database 
 Tests if the database contains the dataObject_Query
   contains(DataObject)   - 
Method in class weka.clusterers.forOPTICSAndDBScan.Databases. SequentialDatabase 
 Tests if the database contains the dataObject_Query
   contains(Object)   - 
Method in class weka.core. FastVector 
 added by akibriya
   contains(PrintStream)   - 
Method in class weka.core. Tee 
 checks whether the given PrintStream is already in the list.
   contains(Object)   - 
Method in class weka.core. Trie 
 Returns true if this collection contains the specified element.
   contains(String)   - 
Method in class weka.core. Trie.TrieNode 
 checks whether a suffix can be found in its children
   contains(String)   - 
Method in class weka.core.xml. MethodHandler 
 checks whether a method is stored for the given property
   contains(Class)   - 
Method in class weka.core.xml. MethodHandler 
 checks whether a method is stored for the given class
   contains(Object)   - 
Method in class weka.gui. CheckBoxList.CheckBoxListModel 
 Tests whether the specified object is a component in this list.
   contains(String)   - 
Method in class weka.gui. HierarchyPropertyParser 
 Whether the HierarchyPropertyParser contains the given
 string
   containsAll(Collection<?>)   - 
Method in class weka.core. Trie 
 Returns true if this collection contains all of the elements in the 
 specified collection.
   containsEnvVariables(String)   - 
Static method in class weka.core. Environment 
 Tests for the presence of environment variables.
   containsItems(ArrayList<Attribute>, boolean)   - 
Method in class weka.associations. FPGrowth.AssociationRule 
  
   containsKey(double)   - 
Method in class weka.classifiers.lazy.kstar. KStarCache.CacheTable 
 Tests if the specified double is a key in this hashtable.
   containsKey(double)   - 
Method in class weka.classifiers.lazy.kstar. KStarCache 
 Checks if the specified key maps with an entry in the cache table
   containsOverOneEvent()   - 
Method in class weka.associations.gsp. Element 
 Checks if an Element contains over one event.
   containsPrefix(String)   - 
Method in class weka.core. Trie 
 checks whether the given prefix is stored in the trie
   containsValue(double)   - 
Method in class weka.core.pmml. FieldMetaInfo.Interval 
 Returns true if this interval contains the supplied value.
   containsWindow(Class)   - 
Method in class weka.gui. Main 
 checks, whether an instance of the given window class is already in
 the Window list.
   containsWindow(String)   - 
Method in class weka.gui. Main 
 checks, whether a window with the given title is already in
 the Window list.
   context()   - 
Method in class weka.gui. HierarchyPropertyParser 
 The context of the current node, i.e.
   ContingencyTables   - Class in  weka.core  Class implementing some statistical routines for contingency tables.   ContingencyTables()   - 
Constructor for class weka.core. ContingencyTables 
  
   CONTINUOUS   - 
Static variable in class weka.datagenerators.clusterers. SubspaceCluster 
 cluster subtype: continuous
   ConverterFileChooser   - Class in  weka.gui  A specialized JFileChooser that lists all available file Loaders and Savers.   ConverterFileChooser()   - 
Constructor for class weka.gui. ConverterFileChooser 
 onstructs a FileChooser pointing to the user's default directory.
   ConverterFileChooser(File)   - 
Constructor for class weka.gui. ConverterFileChooser 
 Constructs a FileChooser using the given File as the path.
   ConverterFileChooser(String)   - 
Constructor for class weka.gui. ConverterFileChooser 
 Constructs a FileChooser using the given path.
   ConverterUtils   - Class in  weka.core.converters  Utility routines for the converter package.   ConverterUtils()   - 
Constructor for class weka.core.converters. ConverterUtils 
  
   ConverterUtils.DataSink   - Class in  weka.core.converters  Helper class for saving data to files.   ConverterUtils.DataSink(String)   - 
Constructor for class weka.core.converters. ConverterUtils.DataSink 
 initializes the sink to save the data to the given file.
   ConverterUtils.DataSink(Saver)   - 
Constructor for class weka.core.converters. ConverterUtils.DataSink 
 initializes the sink to save the data to the given Saver (expected to be 
 fully configured).
   ConverterUtils.DataSink(OutputStream)   - 
Constructor for class weka.core.converters. ConverterUtils.DataSink 
 initializes the sink to save the data in the stream (always in ARFF 
 format).
   ConverterUtils.DataSource   - Class in  weka.core.converters  Helper class for loading data from files and URLs.   ConverterUtils.DataSource(String)   - 
Constructor for class weka.core.converters. ConverterUtils.DataSource 
 Tries to load the data from the file.
   ConverterUtils.DataSource(Instances)   - 
Constructor for class weka.core.converters. ConverterUtils.DataSource 
 Initializes the datasource with the given dataset.
   ConverterUtils.DataSource(Loader)   - 
Constructor for class weka.core.converters. ConverterUtils.DataSource 
 Initializes the datasource with the given Loader.
   ConverterUtils.DataSource(InputStream)   - 
Constructor for class weka.core.converters. ConverterUtils.DataSource 
 Initializes the datasource with the given input stream.
   convertInfixToPostfix(String)   - 
Method in class weka.core. AttributeExpression 
 Converts a string containing a mathematical expression in infix form
 to postfix form.
   convertInstance(Instance)   - 
Method in interface weka.attributeSelection. AttributeTransformer 
 Transforms an instance in the format of the original data to the
 transformed space
   convertInstance(Instance)   - 
Method in class weka.attributeSelection. LatentSemanticAnalysis 
 Transform an instance in original (unnormalized) format
   convertInstance(Instance)   - 
Method in class weka.attributeSelection. PrincipalComponents 
 Transform an instance in original (unormalized) format.
   convertNewLines(String)   - 
Static method in class weka.core. Utils 
 Converts carriage returns and new lines in a string into \r and \n.
   convertNominalTipText()   - 
Method in class weka.classifiers.trees. LMT 
 Returns the tip text for this property
   convertNominalToBinaryTipText()   - 
Method in class weka.classifiers.functions. LibLINEAR 
 Returns the tip text for this property
   convertNumericAttToNominal(int, ArrayList<String>)   - 
Method in class weka.core.pmml. MiningSchema 
 Convert a numeric attribute in the mining schema to nominal.
   convertStringAttsToNominal()   - 
Method in class weka.core.pmml. MiningSchema 
 Method to convert any string attributes in the mining schema
 Instances to nominal attributes.
   convertToAttribX(double)   - 
Method in class weka.gui.visualize. Plot2D 
 convert a Panel x coordinate to a raw x value.
   convertToAttribY(double)   - 
Method in class weka.gui.visualize. Plot2D 
 convert a Panel y coordinate to a raw y value.
   convertToPanelX(double)   - 
Method in class weka.gui.visualize. Plot2D 
 Convert an raw x value to Panel x coordinate.
   convertToPanelY(double)   - 
Method in class weka.gui.visualize. Plot2D 
 Convert an raw y value to Panel y coordinate.
   convertToRelativePath(File)   - 
Static method in class weka.core. Utils 
 Converts a File's absolute path to a path relative to the user
 (ie start) directory.
   convictionForRule(AprioriItemSet, AprioriItemSet, int, int)   - 
Method in class weka.associations. AprioriItemSet 
 Outputs the conviction for a rule.
   copy()   - 
Method in class weka.associations.tertius. IndividualInstance 
  
   copy(ParentSet)   - 
Method in class weka.classifiers.bayes.net. ParentSet 
 Copy makes current parents set equal to other parent set
   copy()   - 
Method in class weka.classifiers.rules. Rule 
 Get a shallow copy of this rule
   copy()   - 
Method in class weka.classifiers.trees.m5. CorrelationSplitInfo 
 Makes a copy of this CorrelationSplitInfo object
   copy()   - 
Method in interface weka.classifiers.trees.m5. SplitEvaluate 
 makes a copy of the SplitEvaluate object
   copy()   - 
Method in class weka.classifiers.trees.m5. YongSplitInfo 
 Makes a copy of this SplitInfo object
   copy()   - 
Method in class weka.core. Attribute 
 Produces a shallow copy of this attribute.
   copy(String)   - 
Method in class weka.core. Attribute 
 Produces a shallow copy of this attribute with a new name.
   copy()   - 
Method in class weka.core. BinarySparseInstance 
 Produces a shallow copy of this instance.
   copy()   - 
Method in interface weka.core. Copyable 
 This method produces a shallow copy of an object.
   copy()   - 
Method in class weka.core. FastVector 
 Produces a shallow copy of this vector.
   copy()   - 
Method in class weka.core. Instance 
 Produces a shallow copy of this instance.
   copy()   - 
Method in class weka.core.matrix. DoubleVector 
 Makes a deep copy of the vector
   copy()   - 
Method in class weka.core.matrix. IntVector 
 Makes a deep copy of the vector
   copy()   - 
Method in class weka.core.matrix. Matrix 
 Make a deep copy of a matrix
   copy()   - 
Method in class weka.core. SparseInstance 
 Produces a shallow copy of this instance.
   Copy   - Class in  weka.filters.unsupervised.attribute  An instance filter that copies a range of attributes in the dataset.   Copy()   - 
Constructor for class weka.filters.unsupervised.attribute. Copy 
  
   Copyable   - Interface in  weka.core  Interface implemented by classes that can produce "shallow" copies
 of their objects.   copyArea(int, int, int, int, int, int)   - 
Method in class weka.gui.visualize. PostscriptGraphics 
 Not implemented
   copyContent()   - 
Method in class weka.gui.arffviewer. ArffPanel 
 copies the content of the selection to the clipboard
   copyContent()   - 
Method in class weka.gui.arffviewer. ArffViewerMainPanel 
 copies the content of the selection to the clipboard
   copyElements()   - 
Method in class weka.core. FastVector 
 Clones the vector and shallow copies all its elements.
   copyInto(Object[])   - 
Method in class weka.gui. CheckBoxList.CheckBoxListModel 
 Copies the components of this list into the specified array.
   copyRelationalValues(Instance, Instances, AttributeLocator)   - 
Static method in class weka.core. RelationalLocator 
 Copies relational values contained in the instance copied to a new
 dataset.
   copyRelationalValues(Instance, boolean, Instances, AttributeLocator, Instances, AttributeLocator)   - 
Static method in class weka.core. RelationalLocator 
 Takes relational values referenced by an Instance and copies them from a
 source dataset to a destination dataset.
   Copyright   - Class in  weka.core  A class for providing centralized Copyright information.   Copyright()   - 
Constructor for class weka.core. Copyright 
  
   copyStringValues(Instance, Instances, AttributeLocator)   - 
Static method in class weka.core. StringLocator 
 Copies string values contained in the instance copied to a new
 dataset.
   copyStringValues(Instance, boolean, Instances, AttributeLocator, Instances, AttributeLocator)   - 
Static method in class weka.core. StringLocator 
 Takes string values referenced by an Instance and copies them from a
 source dataset to a destination dataset.
   copyToClipboard()   - 
Method in class weka.gui.sql. InfoPanel 
 copies the currently selected error message to the clipboard
   CORE_FILE_LOADERS   - 
Static variable in class weka.core.converters. ConverterUtils 
 the core loaders - hardcoded list necessary for RMI/Remote Experiments 
 (comma-separated list).
   CORE_FILE_SAVERS   - 
Static variable in class weka.core.converters. ConverterUtils 
 the core savers - hardcoded list necessary for RMI/Remote Experiments 
 (comma-separated list).
   coreDistance(int, double, DataObject)   - 
Method in interface weka.clusterers.forOPTICSAndDBScan.Databases. Database 
 Calculates the coreDistance for the specified DataObject.
   coreDistance(int, double, DataObject)   - 
Method in class weka.clusterers.forOPTICSAndDBScan.Databases. SequentialDatabase 
 Calculates the coreDistance for the specified DataObject.
   correct()   - 
Method in class weka.classifiers.evaluation. ConfusionMatrix 
 Gets the number of correct classifications (that is, for which a
 correct prediction was made).
   correct()   - 
Method in class weka.classifiers. Evaluation 
 Gets the number of instances correctly classified (that is, for
 which a correct prediction was made).
   correlation(double[], double[], int)   - 
Static method in class weka.core. Utils 
 Returns the correlation coefficient of two double vectors.
   correlation   - 
Variable in class weka.experiment. PairedStats 
 The correlation coefficient
   correlationCoefficient()   - 
Method in class weka.classifiers. Evaluation 
 Returns the correlation coefficient if the class is numeric.
   CorrelationSplitInfo   - Class in  weka.classifiers.trees.m5  Finds split points using correlation.   CorrelationSplitInfo(int, int, int)   - 
Constructor for class weka.classifiers.trees.m5. CorrelationSplitInfo 
 Constructs an object which contains the split information
   COS   - 
Static variable in interface weka.core.mathematicalexpression. sym 
  
   COS   - 
Static variable in interface weka.filters.unsupervised.instance.subsetbyexpression. sym 
  
   CostBenefitAnalysis   - Class in  weka.gui.beans  Bean that aids in analyzing cost/benefit tradeoffs.   CostBenefitAnalysis()   - 
Constructor for class weka.gui.beans. CostBenefitAnalysis 
 Constructor.
   CostBenefitAnalysisBeanInfo   - Class in  weka.gui.beans  Bean info class for the cost/benefit analysis   CostBenefitAnalysisBeanInfo()   - 
Constructor for class weka.gui.beans. CostBenefitAnalysisBeanInfo 
  
   CostCurve   - Class in  weka.classifiers.evaluation  Generates points illustrating probablity cost tradeoffs that can be 
 obtained by varying the threshold value between classes.   CostCurve()   - 
Constructor for class weka.classifiers.evaluation. CostCurve 
  
   CostMatrix   - Class in  weka.classifiers  Class for storing and manipulating a misclassification cost matrix.   CostMatrix(int)   - 
Constructor for class weka.classifiers. CostMatrix 
 Creates a default cost matrix of a particular size.
   CostMatrix(CostMatrix)   - 
Constructor for class weka.classifiers. CostMatrix 
 Creates a cost matrix that is a copy of another.
   CostMatrix(Reader)   - 
Constructor for class weka.classifiers. CostMatrix 
 Reads a matrix from a reader.
   CostMatrixEditor   - Class in  weka.gui  Class for editing CostMatrix objects.   CostMatrixEditor()   - 
Constructor for class weka.gui. CostMatrixEditor 
 Constructs a new CostMatrixEditor.
   costMatrixSourceTipText()   - 
Method in class weka.attributeSelection. CostSensitiveASEvaluation 
  
   costMatrixSourceTipText()   - 
Method in class weka.classifiers.meta. CostSensitiveClassifier 
  
   costMatrixSourceTipText()   - 
Method in class weka.classifiers.meta. MetaCost 
 Returns the tip text for this property
   costMatrixTipText()   - 
Method in class weka.attributeSelection. CostSensitiveASEvaluation 
  
   costMatrixTipText()   - 
Method in class weka.classifiers.meta. CostSensitiveClassifier 
  
   costMatrixTipText()   - 
Method in class weka.classifiers.meta. MetaCost 
 Returns the tip text for this property
   CostSensitiveASEvaluation   - Class in  weka.attributeSelection  Abstract base class for cost-sensitive subset and attribute evaluators.   CostSensitiveASEvaluation()   - 
Constructor for class weka.attributeSelection. CostSensitiveASEvaluation 
  
   CostSensitiveAttributeEval   - Class in  weka.attributeSelection  A meta subset evaluator that makes its base subset evaluator cost-sensitive.   CostSensitiveAttributeEval()   - 
Constructor for class weka.attributeSelection. CostSensitiveAttributeEval 
 Default constructor.
   CostSensitiveClassifier   - Class in  weka.classifiers.meta  A metaclassifier that makes its base classifier cost-sensitive.   CostSensitiveClassifier()   - 
Constructor for class weka.classifiers.meta. CostSensitiveClassifier 
 Default constructor.
   CostSensitiveClassifierSplitEvaluator   - Class in  weka.experiment  SplitEvaluator that produces results for a classification scheme on a nominal class attribute, including weighted misclassification costs.   CostSensitiveClassifierSplitEvaluator()   - 
Constructor for class weka.experiment. CostSensitiveClassifierSplitEvaluator 
  
   CostSensitiveSubsetEval   - Class in  weka.attributeSelection  A meta subset evaluator that makes its base subset evaluator cost-sensitive.   CostSensitiveSubsetEval()   - 
Constructor for class weka.attributeSelection. CostSensitiveSubsetEval 
 Default constructor.
   costTipText()   - 
Method in class weka.classifiers.functions. LibLINEAR 
 Returns the tip text for this property
   costTipText()   - 
Method in class weka.classifiers.functions. LibSVM 
 Returns the tip text for this property
   count()   - 
Method in class weka.associations. RuleGeneration 
 Gets the actual maximum value of the generation time
   count   - 
Variable in class weka.experiment. PairedStats 
 The number of data points seen
   count   - 
Variable in class weka.experiment. Stats 
 The number of values seen
   countBagCiters(Instance)   - 
Method in class weka.classifiers.mi. CitationKNN 
 calculates the citers associated to a bag
   countBagReferences(Instance)   - 
Method in class weka.classifiers.mi. CitationKNN 
 Calculates the references of the exemplar bag
   countData()   - 
Method in class weka.classifiers.rules. RuleStats 
 Filter the data according to the ruleset and compute the basic
 stats: coverage/uncoverage, true/false positive/negatives of 
 each rule
   countData(int, Instances, double[][])   - 
Method in class weka.classifiers.rules. RuleStats 
 Count data from the position index in the ruleset
 assuming that given data are not covered by the rules
 in position 0...(index-1), and the statistics of these
 rules are provided. 
 This procedure is typically useful when a temporary 
 object of RuleStats is constructed in order to efficiently
 calculate the relative DL of rule in position index, 
 thus all other stuff is not needed.
   counter()   - 
Method in class weka.associations. ItemSet 
 Gets the counter
   counterInstance(Instance, Instance)   - 
Method in class weka.associations.tertius. LiteralSet 
 Test if an individual instance, given a part instance of this individual,
 is a counter-instance of this LiteralSet.
   counterInstance(Instance)   - 
Method in class weka.associations.tertius. LiteralSet 
 Test if an instance is a counter-instance of this LiteralSet.
   counterInstance(Instance)   - 
Method in class weka.associations.tertius. Rule 
 Test if an instance is a counter-instance of this rule.
   countsForInstance(Instance)   - 
Method in class weka.classifiers.bayes. BayesNet 
 Calculates the counts for Dirichlet distribution for the 
 class membership probabilities for the given test instance.
   covers(Instance)   - 
Method in class weka.classifiers.rules. Rule 
 Whether the instance covered by this rule
   CoverTree   - Class in  weka.core.neighboursearch  Class implementing the CoverTree datastructure. 
 The class is very much a translation of the c source code made available by the authors. 
  
 For more information and original source code see: 
  
 Alina Beygelzimer, Sham Kakade, John Langford: Cover trees for nearest neighbor.   CoverTree()   - 
Constructor for class weka.core.neighboursearch. CoverTree 
 default constructor.
   CoverTree.CoverTreeNode   - Class in  weka.core.neighboursearch  class representing a node of the cover tree.   CoverTree.CoverTreeNode()   - 
Constructor for class weka.core.neighboursearch. CoverTree.CoverTreeNode 
 Constructor for the class.
   CoverTree.CoverTreeNode(Integer, double, double, Stack<CoverTree.CoverTreeNode>, int, int)   - 
Constructor for class weka.core.neighboursearch. CoverTree.CoverTreeNode 
 Constructor.
   CramersV(double[][])   - 
Static method in class weka.core. ContingencyTables 
 Computes Cramer's V for a contingency table.
   create(Reader)   - 
Method in class weka.gui.treevisualizer. TreeBuild 
 This will build A node structure from the dotty format passed.
   create()   - 
Method in class weka.gui.visualize. PostscriptGraphics 
 Clone a PostscriptGraphics object
   createExperimentIndex()   - 
Method in class weka.experiment. DatabaseUtils 
 Attempts to create the experiment index table.
   createExperimentIndexEntry(ResultProducer)   - 
Method in class weka.experiment. DatabaseUtils 
 Attempts to insert a results entry for the table into the
 experiment index.
   createNewVisualizerWindow(Classifier, Instances)   - 
Static method in class weka.gui.boundaryvisualizer. BoundaryVisualizer 
 Creates a new GUI window with all of the BoundaryVisualizer trappings,
   CreatePopulation(int)   - 
Method in class weka.attributeSelection. ScatterSearchV1 
 Create the initial Population
   createResultsTable(ResultProducer, String)   - 
Method in class weka.experiment. DatabaseUtils 
 Creates a results table for the supplied result producer.
   createSingleton(String[])   - 
Static method in class weka.gui.beans. KnowledgeFlowApp 
 Create the singleton instance of the KnowledgeFlow
   createSingleton()   - 
Static method in class weka.gui. GUIChooser 
 Create a singleton instance of the GUIChooser
   createSingleton(String[])   - 
Static method in class weka.gui. Main 
 Create the singleton instance of the Main GUI.
   createSubsampleWithoutReplacement(Random, int, int, int, int[])   - 
Method in class weka.filters.supervised.instance. Resample 
 creates the subsample without replacement.
   createSubsampleWithoutReplacement(Random, int, int)   - 
Method in class weka.filters.unsupervised.instance. Resample 
 creates the subsample without replacement
   createSubsampleWithReplacement(Random, int, int, int, int[])   - 
Method in class weka.filters.supervised.instance. Resample 
 creates the subsample with replacement.
   createSubsampleWithReplacement(Random, int, int)   - 
Method in class weka.filters.unsupervised.instance. Resample 
 creates the subsample with replacement
   criticalValueTipText()   - 
Method in class weka.classifiers.bayes. AODEsr 
 Returns the tip text for this property
   crossoverProbTipText()   - 
Method in class weka.attributeSelection. GeneticSearch 
 Returns the tip text for this property
   crossValidate(NaiveBayesUpdateable, Instances, Random)   - 
Static method in class weka.classifiers.trees.j48. NBTreeNoSplit 
 Utility method for fast 5-fold cross validation of a naive bayes
 model
   CrossValidateAttributes()   - 
Method in class weka.attributeSelection. AttributeSelection 
 Perform a cross validation for attribute selection.
   crossValidateModel(Classifier, Instances, int, Random, Object...)   - 
Method in class weka.classifiers. Evaluation 
 Performs a (stratified if class is nominal) cross-validation 
 for a classifier on a set of instances.
   crossValidateModel(String, Instances, int, String[], Random)   - 
Method in class weka.classifiers. Evaluation 
 Performs a (stratified if class is nominal) cross-validation 
 for a classifier on a set of instances.
   crossValidateModel(DensityBasedClusterer, Instances, int, Random)   - 
Static method in class weka.clusterers. ClusterEvaluation 
 Perform a cross-validation for DensityBasedClusterer on a set of instances.
   crossValidateModel(String, Instances, int, String[], Random)   - 
Static method in class weka.clusterers. ClusterEvaluation 
 Performs a cross-validation 
 for a DensityBasedClusterer clusterer on a set of instances.
   crossValidateTipText()   - 
Method in class weka.classifiers.lazy. IBk 
 Returns the tip text for this property.
   CrossValidationFoldMaker   - Class in  weka.gui.beans  Bean for splitting instances into training ant test sets according to
 a cross validation   CrossValidationFoldMaker()   - 
Constructor for class weka.gui.beans. CrossValidationFoldMaker 
  
   CrossValidationFoldMakerBeanInfo   - Class in  weka.gui.beans  BeanInfo class for the cross validation fold maker bean   CrossValidationFoldMakerBeanInfo()   - 
Constructor for class weka.gui.beans. CrossValidationFoldMakerBeanInfo 
  
   CrossValidationFoldMakerCustomizer   - Class in  weka.gui.beans  GUI Customizer for the cross validation fold maker bean   CrossValidationFoldMakerCustomizer()   - 
Constructor for class weka.gui.beans. CrossValidationFoldMakerCustomizer 
  
   CrossValidationResultProducer   - Class in  weka.experiment  Generates for each run, carries out an n-fold cross-validation, using the set SplitEvaluator to generate some results.   CrossValidationResultProducer()   - 
Constructor for class weka.experiment. CrossValidationResultProducer 
  
   crossValTipText()   - 
Method in class weka.classifiers.rules. DecisionTable 
 Returns the tip text for this property
   CSVLoader   - Class in  weka.core.converters  Reads a source that is in comma separated or tab separated format.   CSVLoader()   - 
Constructor for class weka.core.converters. CSVLoader 
 default constructor.
   CSVResultListener   - Class in  weka.experiment  Takes results from a result producer and assembles them into comma separated value form.   CSVResultListener()   - 
Constructor for class weka.experiment. CSVResultListener 
 Sets temporary file.
   CSVSaver   - Class in  weka.core.converters  Writes to a destination that is in csv format
  
  

  
 Valid options are:     CSVSaver()   - 
Constructor for class weka.core.converters. CSVSaver 
 Constructor
   cTipText()   - 
Method in class weka.classifiers.functions. SMO 
 Returns the tip text for this property
   cTipText()   - 
Method in class weka.classifiers.functions. SMOreg 
 Returns the tip text for this property
   cTipText()   - 
Method in class weka.classifiers.mi. MISMO 
 Returns the tip text for this property
   cTipText()   - 
Method in class weka.classifiers.mi. MISVM 
 Returns the tip text for this property
   cumulate()   - 
Method in class weka.core.matrix. DoubleVector 
 Returns a vector that stores the cumulated values of the original
 vector
   cumulateInPlace()   - 
Method in class weka.core.matrix. DoubleVector 
 Cumulates the original vector in place
   cumulativeCV(BayesNet)   - 
Method in class weka.classifiers.bayes.net.search.global. GlobalScoreSearchAlgorithm 
 CumulativeCV returns the accuracy calculated using cumulative
 cross validation.
   CustomizerCloseRequester   - Interface in  weka.gui.beans  Customizers who want to be able to close the customizer window
 themselves can implement this window.   customizerClosing()   - 
Method in class weka.gui.beans. ClassAssignerCustomizer 
  
   customizerClosing()   - 
Method in class weka.gui.beans. ClassifierCustomizer 
  
   customizerClosing()   - 
Method in class weka.gui.beans. ClassValuePickerCustomizer 
  
   customizerClosing()   - 
Method in interface weka.gui.beans. CustomizerClosingListener 
 Customizer classes that want to know when they are being
 disposed of can implement this method.
   CustomizerClosingListener   - Interface in  weka.gui.beans      CustomPanelSupplier   - Interface in  weka.gui  An interface for objects that are capable of supplying their own
 custom GUI components.   cutOffFactorTipText()   - 
Method in class weka.clusterers. XMeans 
 Returns the tip text for this property.
   cutoffTipText()   - 
Method in class weka.clusterers. Cobweb 
 Returns the tip text for this property
   cutpointsToString(double[], boolean[])   - 
Static method in class weka.estimators. EstimatorUtils 
 Returns a string representing the cutpoints
   CV_BASED   - 
Static variable in class weka.classifiers.bayes. BayesianLogisticRegression 
  
   CVBasedHyperparameter()   - 
Method in class weka.classifiers.bayes. BayesianLogisticRegression 
 Method computes the best hyperparameter value by doing cross
 -validation on the training data and compute the likelihood.
   CVParameterSelection   - Class in  weka.classifiers.meta  Class for performing parameter selection by cross-validation for any classifier. 
  
 For more information, see: 
  
 R.   CVParameterSelection()   - 
Constructor for class weka.classifiers.meta. CVParameterSelection 
  
   CVParametersTipText()   - 
Method in class weka.classifiers.meta. CVParameterSelection 
 Returns the tip text for this property
   CVResultsString()   - 
Method in class weka.attributeSelection. AttributeSelection 
 returns a string summarizing the results of repeated attribute
 selection runs on splits of a dataset.
   CVTypeTipText()   - 
Method in class weka.classifiers.bayes.net.search.global. GlobalScoreSearchAlgorithm 
  
 
 
    
 D  
 
   D_CONVCHCLOSER   - 
Static variable in class weka.clusterers. XMeans 
 have a closer look at converge children.
   D_CURR   - 
Static variable in class weka.clusterers. XMeans 
 for current debug.
   D_FOLLOWSPLIT   - 
Static variable in class weka.clusterers. XMeans 
 follows the splitting of the centers.
   D_GENERAL   - 
Static variable in class weka.clusterers. XMeans 
 general debugging.
   D_ITERCOUNT   - 
Static variable in class weka.clusterers. XMeans 
 follow iterations.
   D_KDTREE   - 
Static variable in class weka.clusterers. XMeans 
 check on kdtree.
   D_METH_MISUSE   - 
Static variable in class weka.clusterers. XMeans 
 functions were maybe misused.
   D_PRINTCENTERS   - 
Static variable in class weka.clusterers. XMeans 
 print the centers.
   D_RANDOMVECTOR   - 
Static variable in class weka.clusterers. XMeans 
 check on random vectors.
   Dagging   - Class in  weka.classifiers.meta  This meta classifier creates a number of disjoint, stratified folds out of the data and feeds each chunk of data to a copy of the supplied base classifier.   Dagging()   - 
Constructor for class weka.classifiers.meta. Dagging 
 Constructor.
   Database   - Interface in  weka.clusterers.forOPTICSAndDBScan.Databases  
 Database.java  
 Authors: Rainer Holzmann, Zhanna Melnikova-Albrecht, Matthias Schubert  
 Date: Aug 20, 2004  
 Time: 1:03:43 PM  
 $ Revision 1.4 $     database_distanceTypeTipText()   - 
Method in class weka.clusterers. DBScan 
 Returns the tip text for this property
   database_distanceTypeTipText()   - 
Method in class weka.clusterers. OPTICS 
 Returns the tip text for this property
   database_TypeTipText()   - 
Method in class weka.clusterers. DBScan 
 Returns the tip text for this property
   database_TypeTipText()   - 
Method in class weka.clusterers. OPTICS 
 Returns the tip text for this property
   DatabaseConnection   - Class in  weka.core.converters  Connects to a database.   DatabaseConnection()   - 
Constructor for class weka.core.converters. DatabaseConnection 
 Sets up the database drivers
   DatabaseConnectionDialog   - Class in  weka.gui  A dialog to enter URL, username and password for a database connection.   DatabaseConnectionDialog(Frame)   - 
Constructor for class weka.gui. DatabaseConnectionDialog 
 Create database connection dialog.
   DatabaseConnectionDialog(Frame, String, String)   - 
Constructor for class weka.gui. DatabaseConnectionDialog 
 Create database connection dialog.
   DatabaseConnectionDialog(Frame, String, String, boolean)   - 
Constructor for class weka.gui. DatabaseConnectionDialog 
 Create database connection dialog.
   DatabaseConverter   - Interface in  weka.core.converters  Marker interface for a loader/saver that uses a database   databaseForName(String, Instances)   - 
Method in class weka.clusterers. DBScan 
 Returns a new Class-Instance of the specified database
   databaseForName(String, Instances)   - 
Method in class weka.clusterers. OPTICS 
 Returns a new Class-Instance of the specified database
   DatabaseLoader   - Class in  weka.core.converters  Reads Instances from a Database.   DatabaseLoader()   - 
Constructor for class weka.core.converters. DatabaseLoader 
 Constructor
   databaseOutputTipText()   - 
Method in class weka.clusterers. OPTICS 
 Returns the tip text for this property.
   DatabaseResultListener   - Class in  weka.experiment  Takes results from a result producer and sends them to a database.   DatabaseResultListener()   - 
Constructor for class weka.experiment. DatabaseResultListener 
 Sets up the database drivers
   DatabaseResultProducer   - Class in  weka.experiment  Examines a database and extracts out the results produced by the specified ResultProducer and submits them to the specified ResultListener.   DatabaseResultProducer()   - 
Constructor for class weka.experiment. DatabaseResultProducer 
 Creates the DatabaseResultProducer, letting the parent constructor do
 it's thing.
   DatabaseSaver   - Class in  weka.core.converters  Writes to a database (tested with MySQL, InstantDB, HSQLDB).   DatabaseSaver()   - 
Constructor for class weka.core.converters. DatabaseSaver 
 Constructor.
   databaseURLTipText()   - 
Method in class weka.experiment. DatabaseUtils 
 Returns the tip text for this property.
   DatabaseUtils   - Class in  weka.experiment  DatabaseUtils provides utility functions for accessing the experiment
 database.   DatabaseUtils()   - 
Constructor for class weka.experiment. DatabaseUtils 
 Reads properties and sets up the database drivers.
   dataDL(double, double, double, double, double)   - 
Static method in class weka.classifiers.rules. RuleStats 
 The description length of data given the parameters of the data
 based on the ruleset.
   DataFormatListener   - Interface in  weka.gui.beans  Listener interface that customizer classes that are interested
 in data format changes can implement.   DataGenerator   - Class in  weka.datagenerators  Abstract superclass for data generators that generate data for 
 classifiers and clusterers.   DataGenerator()   - 
Constructor for class weka.datagenerators. DataGenerator 
 initializes with default settings.
   DataGenerator   - Interface in  weka.gui.boundaryvisualizer  Interface to something that can generate new instances based on
 a set of input instances   DataGeneratorPanel   - Class in  weka.gui.explorer  A panel for generating artificial data via DataGenerators.   DataGeneratorPanel()   - 
Constructor for class weka.gui.explorer. DataGeneratorPanel 
 creates the panel
   DataNearBalancedND   - Class in  weka.classifiers.meta.nestedDichotomies  A meta classifier for handling multi-class datasets with 2-class classifiers by building a random data-balanced tree structure. 
  
 For more info, check 
  
 Lin Dong, Eibe Frank, Stefan Kramer: Ensembles of Balanced Nested Dichotomies for Multi-class Problems.   DataNearBalancedND()   - 
Constructor for class weka.classifiers.meta.nestedDichotomies. DataNearBalancedND 
 Constructor.
   DataObject   - Interface in  weka.clusterers.forOPTICSAndDBScan.DataObjects  
 DataObject.java  
 Authors: Rainer Holzmann, Zhanna Melnikova-Albrecht, Matthias Schubert  
 Date: Aug 19, 2004  
 Time: 5:48:59 PM  
 $ Revision 1.4 $     dataObjectForName(String, Instance, String, Database)   - 
Method in class weka.clusterers. DBScan 
 Returns a new Class-Instance of the specified database
   dataObjectForName(String, Instance, String, Database)   - 
Method in class weka.clusterers. OPTICS 
 Returns a new Class-Instance of the specified database
   dataObjectIterator()   - 
Method in interface weka.clusterers.forOPTICSAndDBScan.Databases. Database 
 Returns an iterator over all the dataObjects in the database
   dataObjectIterator()   - 
Method in class weka.clusterers.forOPTICSAndDBScan.Databases. SequentialDatabase 
 Returns an iterator over all the dataObjects in the database
   dataSeqIDTipText()   - 
Method in class weka.associations. GeneralizedSequentialPatterns 
 Returns the dataSeqID option tip text for the Weka GUI.
   dataset()   - 
Method in class weka.core. Instance 
 Returns the dataset this instance has access to.
   DATASET_FIELD_NAME   - 
Static variable in class weka.experiment. CrossValidationResultProducer 
 The name of the key field containing the dataset name
   DATASET_FIELD_NAME   - 
Static variable in class weka.experiment. RandomSplitResultProducer 
 The name of the key field containing the dataset name
   DataSetEvent   - Class in  weka.gui.beans  Event encapsulating a data set   DataSetEvent(Object, Instances)   - 
Constructor for class weka.gui.beans. DataSetEvent 
  
   DatasetListPanel   - Class in  weka.gui.experiment  This panel controls setting a list of datasets for an experiment to
 iterate over.   DatasetListPanel(Experiment)   - 
Constructor for class weka.gui.experiment. DatasetListPanel 
 Creates the dataset list panel with the given experiment.
   DatasetListPanel()   - 
Constructor for class weka.gui.experiment. DatasetListPanel 
 Create the dataset list panel initially disabled.
   DataSink   - Interface in  weka.gui.beans  Indicator interface to something that can store instances to some
 destination   DataSource   - Interface in  weka.gui.beans  Interface to something that is capable of being a source for data - 
 either batch or incremental data   DataSourceListener   - Interface in  weka.gui.beans  Interface to something that can accept DataSetEvents   DATATYPE_LAYOUT   - 
Static variable in class weka.gui.beans.xml. XMLBeans 
 the data that is about to be read/written contains a complete layout
   DATATYPE_USERCOMPONENTS   - 
Static variable in class weka.gui.beans.xml. XMLBeans 
 the data that is about to be read/written contains user-components, i.e., 
 Metabeans
   DataVisualizer   - Class in  weka.gui.beans  Bean that encapsulates weka.gui.visualize.VisualizePanel   DataVisualizer()   - 
Constructor for class weka.gui.beans. DataVisualizer 
  
   DataVisualizerBeanInfo   - Class in  weka.gui.beans  Bean info class for the data visualizer   DataVisualizerBeanInfo()   - 
Constructor for class weka.gui.beans. DataVisualizerBeanInfo 
  
   DATE   - 
Static variable in class weka.core. Attribute 
 Constant set for attributes with date values.
   DATE   - 
Static variable in class weka.experiment. DatabaseUtils 
 Type mapping for DATE used for reading experiment results.
   dateAttributesTipText()   - 
Method in class weka.core.converters. CSVLoader 
 Returns the tip text for this property.
   dateFormatTipText()   - 
Method in class weka.core.converters. CSVLoader 
 Returns the tip text for this property.
   dateFormatTipText()   - 
Method in class weka.filters.unsupervised.attribute. Add 
 Returns the tip text for this property.
   dateFormatTipText()   - 
Method in class weka.filters.unsupervised.attribute. ChangeDateFormat 
  
   DbConnectionDialog(String, String)   - 
Method in class weka.gui. DatabaseConnectionDialog 
 Display the database connection dialog
   DbConnectionDialog(String, String, boolean)   - 
Method in class weka.gui. DatabaseConnectionDialog 
 Display the database connection dialog
   DBScan   - Class in  weka.clusterers  Martin Ester, Hans-Peter Kriegel, Joerg Sander, Xiaowei Xu: A Density-Based Algorithm for Discovering Clusters in Large Spatial Databases with Noise.   DBScan()   - 
Constructor for class weka.clusterers. DBScan 
  
   DbUtils   - Class in  weka.gui.sql  A little bit extended DatabaseUtils class.   DbUtils()   - 
Constructor for class weka.gui.sql. DbUtils 
 initializes the object.
   dchisq(double)   - 
Static method in class weka.core.matrix. Maths 
 Returns the density of the Chi-squared distribution.
   dchisq(double, double)   - 
Static method in class weka.core.matrix. Maths 
 Returns the density of the noncentral Chi-squared distribution.
   dchisq(double, DoubleVector)   - 
Static method in class weka.core.matrix. Maths 
 Returns the density of the noncentral Chi-squared distribution.
   dchisqLog(double)   - 
Static method in class weka.core.matrix. Maths 
 Returns the log-density of the noncentral Chi-square distribution.
   dchisqLog(double, double)   - 
Static method in class weka.core.matrix. Maths 
 Returns the log-density value of a noncentral Chi-square distribution.
   dchisqLog(double, DoubleVector)   - 
Static method in class weka.core.matrix. Maths 
 Returns the log-density of a set of noncentral Chi-squared
  distributions.
   DDConditionalEstimator   - Class in  weka.estimators  Conditional probability estimator for a discrete domain conditional upon
 a discrete domain.   DDConditionalEstimator(int, int, boolean)   - 
Constructor for class weka.estimators. DDConditionalEstimator 
 Constructor
   Debug   - Class in  weka.core  A helper class for debug output, logging, clocking, etc.   Debug()   - 
Constructor for class weka.core. Debug 
 default constructor, prints only to stdout
   Debug(String)   - 
Constructor for class weka.core. Debug 
 logs the output to the specified file (and stdout).
   Debug(String, int, int)   - 
Constructor for class weka.core. Debug 
 logs the output
   DEBUG   - 
Static variable in class weka.gui. LogWindow 
 whether we're debugging - enables output on stdout
   Debug.Clock   - Class in  weka.core  A little helper class for clocking and outputting times.   Debug.Clock()   - 
Constructor for class weka.core. Debug.Clock 
 automatically starts the clock with FORMAT_SECONDS format and CPU
 time if available
   Debug.Clock(int)   - 
Constructor for class weka.core. Debug.Clock 
 automatically starts the clock with the given output format and CPU
 time if available
   Debug.Clock(boolean)   - 
Constructor for class weka.core. Debug.Clock 
 starts the clock depending on  start  immediately with the
 FORMAT_SECONDS output format and CPU time if available
   Debug.Clock(boolean, int)   - 
Constructor for class weka.core. Debug.Clock 
 starts the clock depending on  start  immediately, using
 CPU time if available
   Debug.DBO   - Class in  weka.core  contains debug methods   Debug.DBO()   - 
Constructor for class weka.core. Debug.DBO 
  
   Debug.Log   - Class in  weka.core  A helper class for logging stuff.   Debug.Log()   - 
Constructor for class weka.core. Debug.Log 
 default constructor, uses only stdout
   Debug.Log(String)   - 
Constructor for class weka.core. Debug.Log 
 creates a logger that logs into the specified file, if null then only
 stdout is used.
   Debug.Log(String, int, int)   - 
Constructor for class weka.core. Debug.Log 
 creates a logger that logs into the specified file, if null then only
 stdout is used.
   Debug.Random   - Class in  weka.core  This extended Random class enables one to print the generated random
 numbers etc., before they are returned.   Debug.Random()   - 
Constructor for class weka.core. Debug.Random 
 Creates a new random number generator.
   Debug.Random(long)   - 
Constructor for class weka.core. Debug.Random 
 Creates a new random number generator using a single long seed.
   Debug.Random(boolean)   - 
Constructor for class weka.core. Debug.Random 
 Creates a new random number generator.
   Debug.Random(long, boolean)   - 
Constructor for class weka.core. Debug.Random 
 Creates a new random number generator using a single long seed.
   Debug.SimpleLog   - Class in  weka.core  A little, simple helper class for logging stuff.   Debug.SimpleLog()   - 
Constructor for class weka.core. Debug.SimpleLog 
 default constructor, uses only stdout
   Debug.SimpleLog(String)   - 
Constructor for class weka.core. Debug.SimpleLog 
 Creates a logger that writes into the specified file.
   Debug.SimpleLog(String, boolean)   - 
Constructor for class weka.core. Debug.SimpleLog 
 Creates a logger that writes into the specified file.
   Debug.Timestamp   - Class in  weka.core  A class that can be used for timestamps in files, The toString() method
 simply returns the associated Date object in a timestamp format.   Debug.Timestamp()   - 
Constructor for class weka.core. Debug.Timestamp 
 creates a timestamp with the current date and time and the default
 format.
   Debug.Timestamp(String)   - 
Constructor for class weka.core. Debug.Timestamp 
 creates a timestamp with the current date and time and the specified
 format.
   Debug.Timestamp(Date)   - 
Constructor for class weka.core. Debug.Timestamp 
 creates a timestamp with the given date and the default format.
   Debug.Timestamp(Date, String)   - 
Constructor for class weka.core. Debug.Timestamp 
 creates a timestamp with the given date and format.
   debugLevelTipText()   - 
Method in class weka.clusterers. XMeans 
 Returns the tip text for this property.
   debugTipText()   - 
Method in class weka.associations. GeneralizedSequentialPatterns 
 Returns the tip text for this property
   debugTipText()   - 
Method in class weka.attributeSelection. RaceSearch 
 Returns the tip text for this property
   debugTipText()   - 
Method in class weka.attributeSelection. ScatterSearchV1 
 Returns the tip text for this property
   debugTipText()   - 
Method in class weka.classifiers.bayes. BayesianLogisticRegression 
 Returns the tip text for this property
   debugTipText()   - 
Method in class weka.classifiers. Classifier 
 Returns the tip text for this property
   debugTipText()   - 
Method in class weka.classifiers.functions. LinearRegression 
 Returns the tip text for this property
   debugTipText()   - 
Method in class weka.classifiers.functions. Logistic 
 Returns the tip text for this property
   debugTipText()   - 
Method in class weka.classifiers.functions. PaceRegression 
 Returns the tip text for this property
   debugTipText()   - 
Method in class weka.classifiers.functions.supportVector. Kernel 
 Returns the tip text for this property
   debugTipText()   - 
Method in class weka.classifiers.meta. MultiScheme 
 Returns the tip text for this property
   debugTipText()   - 
Method in class weka.classifiers.rules. JRip 
 Returns the tip text for this property
   debugTipText()   - 
Method in class weka.clusterers. EM 
 Returns the tip text for this property
   debugTipText()   - 
Method in class weka.clusterers. HierarchicalClusterer 
 Returns the tip text for this property
   debugTipText()   - 
Method in class weka.clusterers. sIB 
 Returns the tip text for this property
   debugTipText()   - 
Method in class weka.core.converters. TextDirectoryLoader 
 the tip text for this property
   debugTipText()   - 
Method in class weka.datagenerators. DataGenerator 
 Returns the tip text for this property
   debugTipText()   - 
Method in class weka.estimators. Estimator 
 Returns the tip text for this property
   debugTipText()   - 
Method in class weka.experiment. DatabaseUtils 
 Returns the tip text for this property.
   debugTipText()   - 
Method in class weka.filters. SimpleFilter 
 Returns the tip text for this property
   debugTipText()   - 
Method in class weka.filters.unsupervised.attribute. AddExpression 
 Returns the tip text for this property
   debugVectorsFileTipText()   - 
Method in class weka.clusterers. XMeans 
 Returns the tip text for this property.
   decayTipText()   - 
Method in class weka.classifiers.functions. MultilayerPerceptron 
  
   decimalsTipText()   - 
Method in class weka.filters.unsupervised.attribute. NumericCleaner 
 Returns the tip text for this property
   DecisionStump   - Class in  weka.classifiers.trees  Class for building and using a decision stump.   DecisionStump()   - 
Constructor for class weka.classifiers.trees. DecisionStump 
  
   DecisionTable   - Class in  weka.classifiers.rules  Class for building and using a simple decision table majority classifier. 
  
 For more information see:  
  
 Ron Kohavi: The Power of Decision Tables.   DecisionTable()   - 
Constructor for class weka.classifiers.rules. DecisionTable 
 Constructor for a DecisionTable
   DecisionTableHashKey   - Class in  weka.classifiers.rules  Class providing hash table keys for DecisionTable   DecisionTableHashKey(Instance, int, boolean)   - 
Constructor for class weka.classifiers.rules. DecisionTableHashKey 
 Constructor for a hashKey
   DecisionTableHashKey(double[])   - 
Constructor for class weka.classifiers.rules. DecisionTableHashKey 
 Constructor for a hashKey
   decompose()   - 
Method in class weka.classifiers. BVDecompose 
 Carry out the bias-variance decomposition
   decompose()   - 
Method in class weka.classifiers. BVDecomposeSegCVSub 
 Carry out the bias-variance decomposition using the sub-sampled cross-validation method.
   Decorate   - Class in  weka.classifiers.meta  DECORATE is a meta-learner for building diverse ensembles of classifiers by using specially constructed artificial training examples.   Decorate()   - 
Constructor for class weka.classifiers.meta. Decorate 
 Constructor.
   decreaseFrequency(int)   - 
Method in class weka.associations. FPGrowth.BinaryItem 
 Decrease the frequency of this item.
   decreaseFrequency()   - 
Method in class weka.associations. FPGrowth.BinaryItem 
 Decrement the frequency of this item.
   DEFAULT_COLORS   - 
Static variable in class weka.gui.boundaryvisualizer. BoundaryPanel 
 default colours for classes
   DEFAULT_FORMAT   - 
Static variable in class weka.core. Debug.Timestamp 
 the default format
   DEFAULT_FORMAT   - 
Static variable in class weka.gui. SimpleDateFormatEditor 
 the default format
   DEFAULT_HEIGHT   - 
Static variable in class weka.gui.arffviewer. ArffViewerMainPanel 
 the default for height
   DEFAULT_LEFT   - 
Static variable in class weka.gui.arffviewer. ArffViewerMainPanel 
 the default for left
   DEFAULT_SEPARATORS   - 
Static variable in class weka.core. TestInstances 
 the default word separators used in strings
   DEFAULT_SHAPE_SIZE   - 
Static variable in class weka.gui.visualize. Plot2D 
  
   DEFAULT_TOP   - 
Static variable in class weka.gui.arffviewer. ArffViewerMainPanel 
 the default for top
   DEFAULT_WIDTH   - 
Static variable in class weka.gui.arffviewer. ArffViewerMainPanel 
 the default for width
   DEFAULT_WORDS   - 
Static variable in class weka.core. TestInstances 
 the default list of words used in strings
   defaultEvaluatorString()   - 
Method in class weka.attributeSelection. CostSensitiveASEvaluation 
 Return the name of the default evaluator.
   defaultEvaluatorString()   - 
Method in class weka.attributeSelection. CostSensitiveAttributeEval 
 Return the name of the default evaluator.
   defaultOutput()   - 
Method in class weka.datagenerators. DataGenerator 
 Gets the string writer, which is used for outputting to stdout.
   defaultWeightTipText()   - 
Method in class weka.classifiers.functions. Winnow 
 Returns the tip text for this property
   defineDataFormat()   - 
Method in class weka.datagenerators.classifiers.classification. Agrawal 
 Initializes the format for the dataset produced.
   defineDataFormat()   - 
Method in class weka.datagenerators.classifiers.classification. BayesNet 
 Initializes the format for the dataset produced.
   defineDataFormat()   - 
Method in class weka.datagenerators.classifiers.classification. LED24 
 Initializes the format for the dataset produced.
   defineDataFormat()   - 
Method in class weka.datagenerators.classifiers.classification. RandomRBF 
 Initializes the format for the dataset produced.
   defineDataFormat()   - 
Method in class weka.datagenerators.classifiers.classification. RDG1 
 Initializes the format for the dataset produced.
   defineDataFormat()   - 
Method in class weka.datagenerators.classifiers.regression. Expression 
 Initializes the format for the dataset produced.
   defineDataFormat()   - 
Method in class weka.datagenerators.classifiers.regression. MexicanHat 
 Initializes the format for the dataset produced.
   defineDataFormat()   - 
Method in class weka.datagenerators.clusterers. BIRCHCluster 
 Initializes the format for the dataset produced.
   defineDataFormat()   - 
Method in class weka.datagenerators.clusterers. SubspaceCluster 
 Initializes the format for the dataset produced.
   defineDataFormat()   - 
Method in class weka.datagenerators. DataGenerator 
 Initializes the format for the dataset produced.
   DefineFunction   - Class in  weka.core.pmml  Class encapsulating DefineFunction (used in TransformationDictionary).   DefineFunction(Element, TransformationDictionary)   - 
Constructor for class weka.core.pmml. DefineFunction 
  
   degreeTipText()   - 
Method in class weka.classifiers.functions. LibSVM 
 Returns the tip text for this property
   del(int, Instance)   - 
Method in class weka.classifiers.trees.j48. Distribution 
 Deletes given instance from given bag.
   delete(int)   - 
Method in class weka.classifiers.functions.supportVector. SMOset 
 Deletes an element from the set.
   delete()   - 
Method in class weka.core. Instances 
 Removes all instances from the set.
   delete(int)   - 
Method in class weka.core. Instances 
 Removes an instance at the given position from the set.
   deleteArc(String, String)   - 
Method in class weka.classifiers.bayes.net. EditableBayesNet 
 Delete arc between two nodes.
   deleteArc(int, int)   - 
Method in class weka.classifiers.bayes.net. EditableBayesNet 
 Delete arc between two nodes.
   deleteAttribute()   - 
Method in class weka.gui.arffviewer. ArffPanel 
 deletes the currently selected attribute
   deleteAttribute(boolean)   - 
Method in class weka.gui.arffviewer. ArffViewerMainPanel 
 deletes the current selected Attribute or several chosen ones
   deleteAttributeAt(int)   - 
Method in class weka.core. Instance 
 Deletes an attribute at the given position (0 to 
 numAttributes() - 1).
   deleteAttributeAt(int)   - 
Method in class weka.core. Instances 
 Deletes an attribute at the given position
 (0 to numAttributes() - 1).
   deleteAttributeAt(int)   - 
Method in class weka.gui.arffviewer. ArffSortedTableModel 
 deletes the attribute at the given col index
   deleteAttributeAt(int)   - 
Method in class weka.gui.arffviewer. ArffTableModel 
 deletes the attribute at the given col index.
   deleteAttributeAt(int, boolean)   - 
Method in class weka.gui.arffviewer. ArffTableModel 
 deletes the attribute at the given col index
   deleteAttributes()   - 
Method in class weka.gui.arffviewer. ArffPanel 
 deletes the chosen attributes
   deleteAttributes(int[])   - 
Method in class weka.gui.arffviewer. ArffSortedTableModel 
 deletes the attributes at the given indices
   deleteAttributes(int[])   - 
Method in class weka.gui.arffviewer. ArffTableModel 
 deletes the attributes at the given indices
   deleteAttributeType(int)   - 
Method in class weka.core. Instances 
 Deletes all attributes of the given type in the dataset.
   deleteEmptyBinsTipText()   - 
Method in class weka.classifiers.meta. RegressionByDiscretization 
 Returns the tip text for this property
   deleteEvent(String)   - 
Method in class weka.associations.gsp. Element 
 Deletes the first or last event of an Element.
   deleteGraftedCases(Instances)   - 
Method in class weka.classifiers.trees.j48. GraftSplit 
 deletes the cases in data that belong to leaf pointed to by
 the test (i.e.
   deleteInfrequentSequences(FastVector, long)   - 
Static method in class weka.associations.gsp. Sequence 
 Deletes Sequences of a given set which don't meet the minimum support 
 count threshold.
   deleteInstance()   - 
Method in class weka.gui.arffviewer. ArffPanel 
 deletes the currently selected instance
   deleteInstance(boolean)   - 
Method in class weka.gui.arffviewer. ArffViewerMainPanel 
 deletes the current selected Instance or several chosen ones
   deleteInstanceAt(int)   - 
Method in class weka.gui.arffviewer. ArffSortedTableModel 
 deletes the instance at the given index
   deleteInstanceAt(int)   - 
Method in class weka.gui.arffviewer. ArffTableModel 
 deletes the instance at the given index
   deleteInstanceAt(int, boolean)   - 
Method in class weka.gui.arffviewer. ArffTableModel 
 deletes the instance at the given index
   deleteInstances()   - 
Method in class weka.gui.arffviewer. ArffPanel 
 deletes all the currently selected instances
   deleteInstances(int[])   - 
Method in class weka.gui.arffviewer. ArffSortedTableModel 
 deletes the instances at the given positions
   deleteInstances(int[])   - 
Method in class weka.gui.arffviewer. ArffTableModel 
 deletes the instances at the given positions
   deleteItemSets(FastVector, int, int)   - 
Static method in class weka.associations. ItemSet 
 Deletes all item sets that don't have minimum support.
   deleteItemSets(FastVector, int, int)   - 
Static method in class weka.associations. LabeledItemSet 
 Deletes all item sets that don't have minimum support and have more than maximum support
   deleteLastParent(Instances)   - 
Method in class weka.classifiers.bayes.net. ParentSet 
 Delete last added parent from parent set and update internals (specifically the cardinality of the parent set)
   deleteNode(String)   - 
Method in class weka.classifiers.bayes.net. EditableBayesNet 
 Delete node from the network, updating instances, parentsets,
 distributions Conditional distributions are condensed by taking the
 values for the target node to be its first value.
   deleteNode(int)   - 
Method in class weka.classifiers.bayes.net. EditableBayesNet 
 Delete node from the network, updating instances, parentsets,
 distributions Conditional distributions are condensed by taking the
 values for the target node to be its first value.
   deleteParent(int, Instances)   - 
Method in class weka.classifiers.bayes.net. ParentSet 
 delete node from parent set
   deleteSelection(FastVector)   - 
Method in class weka.classifiers.bayes.net. EditableBayesNet 
 Delete nodes with indexes in selection from the network, updating instances, parentsets,
 distributions Conditional distributions are condensed by taking the
 values for the target node to be its first value.
   deleteStringAttributes()   - 
Method in class weka.core. Instances 
 Deletes all string attributes in the dataset.
   deleteWithMissing(int)   - 
Method in class weka.core. Instances 
 Removes all instances with missing values for a particular
 attribute from the dataset.
   deleteWithMissing(Attribute)   - 
Method in class weka.core. Instances 
 Removes all instances with missing values for a particular
 attribute from the dataset.
   deleteWithMissingClass()   - 
Method in class weka.core. Instances 
 Removes all instances with a missing class value
 from the dataset.
   delimitersTipText()   - 
Method in class weka.core.tokenizers. CharacterDelimitedTokenizer 
 Returns the tip text for this property
   delNodeValue(int, String)   - 
Method in class weka.classifiers.bayes.net. EditableBayesNet 
 Delete node value from a node.
   delRange(int, Instances, int, int)   - 
Method in class weka.classifiers.trees.j48. Distribution 
 Deletes all instances in given range from given bag.
   Delta   - 
Variable in class weka.classifiers.bayes. BayesianLogisticRegression 
 Trust Region Radius
   DeltaBeta   - 
Variable in class weka.classifiers.bayes. BayesianLogisticRegression 
 Array to store Regression Coefficient updates.
   DeltaR   - 
Variable in class weka.classifiers.bayes. BayesianLogisticRegression 
 This vector is used to store the increments on the R(i).
   deltaTipText()   - 
Method in class weka.associations. Apriori 
 Returns the tip text for this property
   deltaTipText()   - 
Method in class weka.associations. FPGrowth 
 Returns the tip text for this property
   deltaTipText()   - 
Method in class weka.classifiers.bayes.net.search.global. SimulatedAnnealing 
  
   deltaTipText()   - 
Method in class weka.classifiers.bayes.net.search.local. SimulatedAnnealing 
  
   DeltaUpdate   - 
Variable in class weka.classifiers.bayes. BayesianLogisticRegression 
 Trust Region Radius Update
   DensityBasedClusterer   - Interface in  weka.clusterers  Interface for clusterers that can estimate the density for a given instance.   DensityBasedClustererSplitEvaluator   - Class in  weka.experiment  A SplitEvaluator that produces results for a density based clusterer.   DensityBasedClustererSplitEvaluator()   - 
Constructor for class weka.experiment. DensityBasedClustererSplitEvaluator 
  
   densityBasedClustererTipText()   - 
Method in class weka.filters.unsupervised.attribute. ClusterMembership 
 Returns a description of this option suitable for display
 as a tip text in the gui.
   dependencies()   - 
Method in class weka.core. Capabilities 
 Returns an Iterator over the stored dependencies
   depth()   - 
Method in class weka.gui. HierarchyPropertyParser 
 Get the depth of the tree, i.e.
   DerivedFieldMetaInfo   - Class in  weka.core.pmml      DerivedFieldMetaInfo(Element, ArrayList<Attribute>, TransformationDictionary)   - 
Constructor for class weka.core.pmml. DerivedFieldMetaInfo 
  
   descendantPopulationSizeTipText()   - 
Method in class weka.classifiers.bayes.net.search.global. GeneticSearch 
  
   descendantPopulationSizeTipText()   - 
Method in class weka.classifiers.bayes.net.search.local. GeneticSearch 
  
   description()   - 
Method in class weka.associations.tertius. Predicate 
  
   description()   - 
Method in class weka.core. Option 
 Returns the option's description.
   deserialize(InputStream)   - 
Static method in class weka.core. Jython 
 deserializes the Python Object from the stream
   deSerialize(String)   - 
Static method in class weka.core.xml. XStream 
 Deserializes an object from the supplied XML string
   designatedClassTipText()   - 
Method in class weka.classifiers.meta. ThresholdSelector 
  
   desiredSizeTipText()   - 
Method in class weka.classifiers.meta. Decorate 
 Returns the tip text for this property
   desiredWeightOfInstancesPerIntervalTipText()   - 
Method in class weka.filters.unsupervised.attribute. Discretize 
 Returns the tip text for this property
   det()   - 
Method in class weka.core.matrix. LUDecomposition 
 Determinant
   det()   - 
Method in class weka.core.matrix. Matrix 
 Matrix determinant
   detectionPerAttributeTipText()   - 
Method in class weka.filters.unsupervised.attribute. InterquartileRange 
 Returns the tip text for this property
   determineBounds()   - 
Method in class weka.gui.visualize. Plot2D 
 Determine the min and max values for axis and colouring attributes
   determineColumnConstraints(ResultProducer)   - 
Method in class weka.experiment. AveragingResultProducer 
 Determines if there are any constraints (imposed by the
 destination) on the result columns to be produced by
 resultProducers.
   determineColumnConstraints(ResultProducer)   - 
Method in class weka.experiment. CSVResultListener 
 Determines if there are any constraints (imposed by the
 destination) on the result columns to be produced by
 resultProducers.
   determineColumnConstraints(ResultProducer)   - 
Method in class weka.experiment. DatabaseResultListener 
 Determines if there are any constraints (imposed by the
 destination) on any additional measures produced by
 resultProducers.
   determineColumnConstraints(ResultProducer)   - 
Method in class weka.experiment. LearningRateResultProducer 
 Determines if there are any constraints (imposed by the
 destination) on the result columns to be produced by
 resultProducers.
   determineColumnConstraints(ResultProducer)   - 
Method in interface weka.experiment. ResultListener 
 Determines if there are any constraints (imposed by the
 destination) on additional result columns to be produced by
 resultProducers.
   determineValues(Instances)   - 
Method in class weka.filters.unsupervised.instance. RemoveFrequentValues 
 determines the values to retain, it is always at least 1
 and up to the maximum number of distinct values
   DIAMOND_SHAPE   - 
Static variable in class weka.gui.visualize. Plot2D 
  
   differencesProbability   - 
Variable in class weka.experiment. PairedStats 
 The probability of obtaining the observed differences
   differencesSignificance   - 
Variable in class weka.experiment. PairedStats 
 A significance indicator:
 0 if the differences are not significant
 > 0 if x significantly greater than y
    differencesStats   - 
Variable in class weka.experiment. PairedStats 
 The stats associated with the paired differences
   DIRECTED   - 
Static variable in interface weka.gui.graphvisualizer. GraphConstants 
 Types of Edges
   directionTipText()   - 
Method in class weka.attributeSelection. BestFirst 
 Returns the tip text for this property
   disable(Capabilities.Capability)   - 
Method in class weka.core. Capabilities 
 disables the given capability
 Disabling NOMINAL_ATTRIBUTES also disables BINARY_ATTRIBUTES, 
 UNARY_ATTRIBUTES and EMPTY_NOMINAL_ATTRIBUTES.
   disable(Capabilities.Capability)   - 
Method in class weka.core. FindWithCapabilities 
 disables the given capability.
   disableAll()   - 
Method in class weka.core. Capabilities 
 disables all attribute and class types (including dependencies)
   disableAllAttributeDependencies()   - 
Method in class weka.core. Capabilities 
 disables all attribute type dependencies
   disableAllAttributes()   - 
Method in class weka.core. Capabilities 
 disables all attribute types
   disableAllClassDependencies()   - 
Method in class weka.core. Capabilities 
 disables all class type dependencies
   disableAllClasses()   - 
Method in class weka.core. Capabilities 
 disables all class types
   disabled_getEquivalent()   - 
Method in class weka.associations. Tertius 
 Get the value of equivalent.
   disabled_getPartFile()   - 
Method in class weka.associations. Tertius 
 Get the value of partFile.
   disabled_getSameClause()   - 
Method in class weka.associations. Tertius 
 Get the value of sameClause.
   disabled_getSubsumption()   - 
Method in class weka.associations. Tertius 
 Get the value of subsumption.
   disabled_setEquivalent(boolean)   - 
Method in class weka.associations. Tertius 
 Set the value of equivalent.
   disabled_setPartFile(File)   - 
Method in class weka.associations. Tertius 
 Set the value of partFile.
   disabled_setSameClause(boolean)   - 
Method in class weka.associations. Tertius 
 Set the value of sameClause.
   disabled_setSubsumption(boolean)   - 
Method in class weka.associations. Tertius 
 Set the value of subsumption.
   disableDependency(Capabilities.Capability)   - 
Method in class weka.core. Capabilities 
 disables the dependency of the given capability
 Disabling NOMINAL_ATTRIBUTES also disables BINARY_ATTRIBUTES, 
 UNARY_ATTRIBUTES and EMPTY_NOMINAL_ATTRIBUTES.
   disableNot(Capabilities.Capability)   - 
Method in class weka.core. FindWithCapabilities 
 disables the given "not to have" capability.
   disconnect(NeuralConnection, NeuralConnection)   - 
Static method in class weka.classifiers.functions.neural. NeuralConnection 
 Disconnects two units.
   DISCONNECT   - 
Static variable in class weka.gui.sql.event. ConnectionEvent 
 it was a disconnect
   disconnectFromDatabase()   - 
Method in class weka.experiment. DatabaseUtils 
 Closes the connection to the database.
   disconnectionNotification(String, Object)   - 
Method in class weka.gui.beans. AbstractDataSink 
 Notify this object that it has been deregistered as a listener with
 a source with respect to the supplied event name
   disconnectionNotification(String, Object)   - 
Method in class weka.gui.beans. AbstractEvaluator 
 Notify this object that it has been deregistered as a listener with
 a source with respect to the supplied event named
   disconnectionNotification(String, Object)   - 
Method in class weka.gui.beans. AbstractTestSetProducer 
 Notify this object that it has been deregistered as a listener with
 a source with respect to the supplied event name
   disconnectionNotification(String, Object)   - 
Method in class weka.gui.beans. AbstractTrainAndTestSetProducer 
 Notify this object that it has been deregistered as a listener with
 a source with respect to the supplied event name
   disconnectionNotification(String, Object)   - 
Method in class weka.gui.beans. AbstractTrainingSetProducer 
 Notify this object that it has been deregistered as a listener with
 a source with respect to the supplied event name
   disconnectionNotification(String, Object)   - 
Method in class weka.gui.beans. Associator 
 Notify this object that it has been deregistered as a listener with
 a source with respect to the supplied event name
   disconnectionNotification(String, Object)   - 
Method in interface weka.gui.beans. BeanCommon 
 Notify this object that it has been deregistered as a listener with
 a source for named event.
   disconnectionNotification(String, Object)   - 
Method in class weka.gui.beans. ClassAssigner 
 Notify this object that it has been deregistered as a listener with
 a source with respect to the supplied event name
   disconnectionNotification(String, Object)   - 
Method in class weka.gui.beans. Classifier 
 Notify this object that it has been deregistered as a listener with
 a source with respect to the supplied event name
   disconnectionNotification(String, Object)   - 
Method in class weka.gui.beans. ClassValuePicker 
 Notify this object that it has been deregistered as a listener with
 a source with respect to the supplied event name
   disconnectionNotification(String, Object)   - 
Method in class weka.gui.beans. Clusterer 
 Notify this object that it has been deregistered as a listener with
 a source with respect to the supplied event name
   disconnectionNotification(String, Object)   - 
Method in interface weka.gui.beans. ConnectionNotificationConsumer 
 Notify this object that it has been deregistered as a listener with a
 source with respect to the supplied event name
 
 This method should be implemented  synchronized .
   disconnectionNotification(String, Object)   - 
Method in class weka.gui.beans. CostBenefitAnalysis 
 Notify this object that it has been deregistered as a listener with
 a source for named event.
   disconnectionNotification(String, Object)   - 
Method in class weka.gui.beans. Filter 
 Notify this object that it has been deregistered as a listener with
 a source with respect to the supplied event name
   disconnectionNotification(String, Object)   - 
Method in class weka.gui.beans. InstanceStreamToBatchMaker 
 Notify this object that it has been deregistered as a listener with
 a source with respect to the supplied event name
   disconnectionNotification(String, Object)   - 
Method in class weka.gui.beans. Loader 
 Notify this object that it has been deregistered as a listener with
 a source for named event.
   disconnectionNotification(String, Object)   - 
Method in class weka.gui.beans. MetaBean 
 Notify this object that it has been deregistered as a listener with
 a source with respect to the supplied event name.
   disconnectionNotification(String, Object)   - 
Method in class weka.gui.beans. PredictionAppender 
 Notify this object that it has been deregistered as a listener with
 a source with respect to the supplied event name
   disconnectionNotification(String, Object)   - 
Method in class weka.gui.beans. SerializedModelSaver 
 Notify this object that it has been deregistered as a listener with
 a source with respect to the supplied event name.
   disconnectionNotification(String, Object)   - 
Method in class weka.gui.beans. StripChart 
 Notify this object that it has been deregistered as a listener with
 a source for named event.
   disconnectionNotification(String, Object)   - 
Method in class weka.gui.beans. TextViewer 
 Notify this object that it has been deregistered as a listener with
 a source for named event.
   DiscreteEstimator   - Class in  weka.estimators  Simple symbolic probability estimator based on symbol counts.   DiscreteEstimator(int, boolean)   - 
Constructor for class weka.estimators. DiscreteEstimator 
 Constructor
   DiscreteEstimator(int, double)   - 
Constructor for class weka.estimators. DiscreteEstimator 
 Constructor
   DiscreteEstimatorBayes   - Class in  weka.classifiers.bayes.net.estimate  Symbolic probability estimator based on symbol counts and a prior.   DiscreteEstimatorBayes(int, double)   - 
Constructor for class weka.classifiers.bayes.net.estimate. DiscreteEstimatorBayes 
 Constructor
   DiscreteEstimatorFullBayes   - Class in  weka.classifiers.bayes.net.estimate  Symbolic probability estimator based on symbol counts and a prior.   DiscreteEstimatorFullBayes(int, double, double, DiscreteEstimatorBayes, DiscreteEstimatorBayes, double)   - 
Constructor for class weka.classifiers.bayes.net.estimate. DiscreteEstimatorFullBayes 
 Constructor
   DiscreteFunction   - Class in  weka.classifiers.functions.pace  Class for handling discrete functions.   DiscreteFunction()   - 
Constructor for class weka.classifiers.functions.pace. DiscreteFunction 
 Constructs an empty discrete function
   DiscreteFunction(DoubleVector)   - 
Constructor for class weka.classifiers.functions.pace. DiscreteFunction 
 Constructs a discrete function with the point values provides and the
  function values are all 1/n.
   DiscreteFunction(DoubleVector, DoubleVector)   - 
Constructor for class weka.classifiers.functions.pace. DiscreteFunction 
 Constructs a discrete function with both the point values and
  function values provided.
   Discretize   - Class in  weka.core.pmml  Class encapsulating a Discretize Expression.   Discretize(Element, FieldMetaInfo.Optype, ArrayList<Attribute>)   - 
Constructor for class weka.core.pmml. Discretize 
 Constructs a Discretize Expression
   Discretize   - Class in  weka.filters.supervised.attribute  An instance filter that discretizes a range of numeric attributes in the dataset into nominal attributes.   Discretize()   - 
Constructor for class weka.filters.supervised.attribute. Discretize 
 Constructor - initialises the filter
   Discretize   - Class in  weka.filters.unsupervised.attribute  An instance filter that discretizes a range of numeric attributes in the dataset into nominal attributes.   Discretize()   - 
Constructor for class weka.filters.unsupervised.attribute. Discretize 
 Constructor - initialises the filter
   Discretize(String)   - 
Constructor for class weka.filters.unsupervised.attribute. Discretize 
 Another constructor, sets the attribute indices immediately
   discretizeBinTipText()   - 
Method in class weka.classifiers.mi. MIBoost 
 Returns the tip text for this property
   displayModelInOldFormatTipText()   - 
Method in class weka.classifiers.bayes. NaiveBayes 
 Returns the tip text for this property
   displayModelInOldFormatTipText()   - 
Method in class weka.clusterers. EM 
 Returns the tip text for this property
   displayResultset(int)   - 
Method in class weka.experiment. PairedTTester 
 Checks whether the resultset with the given index shall be displayed.
   displayResultset(int)   - 
Method in interface weka.experiment. Tester 
 Checks whether the resultset with the given index shall be displayed.
   displayRulesTipText()   - 
Method in class weka.classifiers.rules. DecisionTable 
 Returns the tip text for this property
   displayStdDevsTipText()   - 
Method in class weka.clusterers. SimpleKMeans 
 Returns the tip text for this property
   dispose()   - 
Method in class weka.gui. GUIChooser.ChildFrameSDI 
 de-registers the child frame with the parent first.
   dispose()   - 
Method in class weka.gui. Main.ChildFrameMDI 
 de-registers the child frame with the parent first.
   dispose()   - 
Method in class weka.gui. Main.ChildFrameSDI 
 de-registers the child frame with the parent first.
   dispose()   - 
Method in class weka.gui.visualize. PostscriptGraphics 
 Not implemented
   disposeSplash()   - 
Static method in class weka.gui. SplashWindow 
 Closes the splash window.
   distance(Instance, Instance)   - 
Method in class weka.classifiers.mi. CitationKNN 
 distance between two instances
   distance(DataObject)   - 
Method in interface weka.clusterers.forOPTICSAndDBScan.DataObjects. DataObject 
 Calculates the distance between dataObject and this.dataObject
   distance(DataObject)   - 
Method in class weka.clusterers.forOPTICSAndDBScan.DataObjects. EuclidianDataObject 
 Calculates the euclidian-distance between dataObject and this.dataObject
   distance(DataObject)   - 
Method in class weka.clusterers.forOPTICSAndDBScan.DataObjects. ManhattanDataObject 
 Calculates the manhattan-distance between dataObject and this.dataObject
   distance(Instance, Instance, double, PerformanceStats)   - 
Method in class weka.core. AbstractStringDistanceFunction 
 Calculates the distance between two instances.
   distance(Instance, Instance)   - 
Method in interface weka.core. DistanceFunction 
 Calculates the distance between two instances.
   distance(Instance, Instance, PerformanceStats)   - 
Method in interface weka.core. DistanceFunction 
 Calculates the distance between two instances.
   distance(Instance, Instance, double)   - 
Method in interface weka.core. DistanceFunction 
 Calculates the distance between two instances.
   distance(Instance, Instance, double, PerformanceStats)   - 
Method in interface weka.core. DistanceFunction 
 Calculates the distance between two instances.
   distance(Instance, Instance)   - 
Method in class weka.core. EuclideanDistance 
 Calculates the distance between two instances.
   distance(Instance, Instance, PerformanceStats)   - 
Method in class weka.core. EuclideanDistance 
 Calculates the distance (or similarity) between two instances.
   distance(Instance, Instance)   - 
Method in class weka.core. NormalizableDistance 
 Calculates the distance between two instances.
   distance(Instance, Instance, PerformanceStats)   - 
Method in class weka.core. NormalizableDistance 
 Calculates the distance between two instances.
   distance(Instance, Instance, double)   - 
Method in class weka.core. NormalizableDistance 
 Calculates the distance between two instances.
   distance(Instance, Instance, double, PerformanceStats)   - 
Method in class weka.core. NormalizableDistance 
 Calculates the distance between two instances.
   distanceFTipText()   - 
Method in class weka.clusterers. XMeans 
 Returns the tip text for this property.
   DistanceFunction   - Interface in  weka.core  Interface for any class that can compute and return distances between two
 instances.   distanceFunctionTipText()   - 
Method in class weka.clusterers. HierarchicalClusterer 
  
   distanceFunctionTipText()   - 
Method in class weka.clusterers. SimpleKMeans 
 Returns the tip text for this property.
   distanceFunctionTipText()   - 
Method in class weka.core.neighboursearch. NearestNeighbourSearch 
 Returns the tip text for this property.
   distanceIsBranchLengthTipText()   - 
Method in class weka.clusterers. HierarchicalClusterer 
  
   distanceSet(Instance, Instance)   - 
Method in class weka.classifiers.mi. CitationKNN 
 Calculates the distance between two instances
   distanceWeightingTipText()   - 
Method in class weka.classifiers.lazy. IBk 
 Returns the tip text for this property.
   distinctCount   - 
Variable in class weka.core. AttributeStats 
 The number of distinct values
   distMultTipText()   - 
Method in class weka.datagenerators.clusterers. BIRCHCluster 
 Returns the tip text for this property
   distributedExperimentSelected()   - 
Method in class weka.gui.experiment. DistributeExperimentPanel 
 Returns true if the distribute experiment checkbox is selected
   DistributeExperimentPanel   - Class in  weka.gui.experiment  This panel enables an experiment to be distributed to multiple hosts;
 it also allows remote host names to be specified.   DistributeExperimentPanel()   - 
Constructor for class weka.gui.experiment. DistributeExperimentPanel 
 Constructor
   DistributeExperimentPanel(Experiment)   - 
Constructor for class weka.gui.experiment. DistributeExperimentPanel 
 Creates the panel with the supplied initial experiment.
   distribution()   - 
Method in class weka.classifiers.evaluation. NominalPrediction 
 Gets the predicted probabilities
   distribution()   - 
Method in class weka.classifiers.trees.j48. ClassifierSplitModel 
 Returns the distribution of class values induced by the model.
   Distribution   - Class in  weka.classifiers.trees.j48  Class for handling a distribution of class values.   Distribution(int, int)   - 
Constructor for class weka.classifiers.trees.j48. Distribution 
 Creates and initializes a new distribution.
   Distribution(double[][])   - 
Constructor for class weka.classifiers.trees.j48. Distribution 
 Creates and initializes a new distribution using the given
 array.
   Distribution(Instances)   - 
Constructor for class weka.classifiers.trees.j48. Distribution 
 Creates a distribution with only one bag according
 to instances in source.
   Distribution(Instances, ClassifierSplitModel)   - 
Constructor for class weka.classifiers.trees.j48. Distribution 
 Creates a distribution according to given instances and
 split model.
   Distribution(Distribution)   - 
Constructor for class weka.classifiers.trees.j48. Distribution 
 Creates distribution with only one bag by merging all
 bags of given distribution.
   Distribution(Distribution, int)   - 
Constructor for class weka.classifiers.trees.j48. Distribution 
 Creates distribution with two bags by merging all bags apart of
 the indicated one.
   distributionForInstance(Instance)   - 
Method in class weka.classifiers.bayes. AODE 
 Calculates the class membership probabilities for the given test
 instance.
   distributionForInstance(Instance)   - 
Method in class weka.classifiers.bayes. AODEsr 
 Calculates the class membership probabilities for the given test
 instance.
   distributionForInstance(Instance)   - 
Method in class weka.classifiers.bayes. BayesNet 
 Calculates the class membership probabilities for the given test
 instance.
   distributionForInstance(Instance)   - 
Method in class weka.classifiers.bayes. DMNBtext 
 Calculates the class membership probabilities for the given test
 instance.
   distributionForInstance(Instance)   - 
Method in class weka.classifiers.bayes. DMNBtext.DNBBinary 
 Calculates the class membership probabilities for the given test
 instance.
   distributionForInstance(Instance)   - 
Method in class weka.classifiers.bayes. HNB 
 Calculates the class membership probabilities for the given test instance
   distributionForInstance(Instance)   - 
Method in class weka.classifiers.bayes. NaiveBayes 
 Calculates the class membership probabilities for the given test 
 instance.
   distributionForInstance(Instance)   - 
Method in class weka.classifiers.bayes. NaiveBayesMultinomial 
 Calculates the class membership probabilities for the given test 
 instance.
   distributionForInstance(Instance)   - 
Method in class weka.classifiers.bayes. NaiveBayesMultinomialUpdateable 
 Calculates the class membership probabilities for the given test
 instance.
   distributionForInstance(Instance)   - 
Method in class weka.classifiers.bayes. NaiveBayesSimple 
 Calculates the class membership probabilities for the given test instance.
   distributionForInstance(BayesNet, Instance)   - 
Method in class weka.classifiers.bayes.net.estimate. BayesNetEstimator 
 Calculates the class membership probabilities for the given test
 instance.
   distributionForInstance(BayesNet, Instance)   - 
Method in class weka.classifiers.bayes.net.estimate. MultiNomialBMAEstimator 
 Calculates the class membership probabilities for the given test
 instance.
   distributionForInstance(BayesNet, Instance)   - 
Method in class weka.classifiers.bayes.net.estimate. SimpleEstimator 
 Calculates the class membership probabilities for the given test
 instance.
   distributionForInstance(Instance)   - 
Method in class weka.classifiers.bayes. WAODE 
 Calculates the class membership probabilities for the given test instance
   distributionForInstance(Instance)   - 
Method in class weka.classifiers. Classifier 
 Predicts the class memberships for a given instance.
   distributionForInstance(Instance)   - 
Method in class weka.classifiers.functions. LibLINEAR 
 Computes the distribution for a given instance.
   distributionForInstance(Instance)   - 
Method in class weka.classifiers.functions. LibSVM 
 Computes the distribution for a given instance.
   distributionForInstance(Instance)   - 
Method in class weka.classifiers.functions. Logistic 
 Computes the distribution for a given instance
   distributionForInstance(Instance)   - 
Method in class weka.classifiers.functions. MultilayerPerceptron 
 Call this function to predict the class of an instance once a 
 classification model has been built with the buildClassifier call.
   distributionForInstance(Instance)   - 
Method in class weka.classifiers.functions. RBFNetwork 
 Computes the distribution for a given instance
   distributionForInstance(Instance)   - 
Method in class weka.classifiers.functions. SimpleLogistic 
 Returns class probabilities for an instance.
   distributionForInstance(Instance)   - 
Method in class weka.classifiers.functions. SMO 
 Estimates class probabilities for given instance.
   distributionForInstance(Instance)   - 
Method in class weka.classifiers.functions. SPegasos 
 Computes the distribution for a given instance
   distributionForInstance(Instance)   - 
Method in class weka.classifiers.functions. VotedPerceptron 
 Outputs the distribution for the given output.
   distributionForInstance(Instance)   - 
Method in class weka.classifiers.lazy. IBk 
 Calculates the class membership probabilities for the given test instance.
   distributionForInstance(Instance)   - 
Method in class weka.classifiers.lazy. KStar 
 Calculates the class membership probabilities for the given test instance.
   distributionForInstance(Instance)   - 
Method in class weka.classifiers.lazy. LBR 
 Calculates the class membership probabilities
 for the given test instance.
   distributionForInstance(Instance)   - 
Method in class weka.classifiers.lazy. LWL 
 Calculates the class membership probabilities for the given test instance.
   distributionForInstance(Instance)   - 
Method in class weka.classifiers.meta. AdaBoostM1 
 Calculates the class membership probabilities for the given test instance.
   distributionForInstance(Instance)   - 
Method in class weka.classifiers.meta. AttributeSelectedClassifier 
 Classifies a given instance after attribute selection
   distributionForInstance(Instance)   - 
Method in class weka.classifiers.meta. Bagging 
 Calculates the class membership probabilities for the given test
 instance.
   distributionForInstance(Instance)   - 
Method in class weka.classifiers.meta. ClassificationViaRegression 
 Returns the distribution for an instance.
   distributionForInstance(Instance)   - 
Method in class weka.classifiers.meta. CostSensitiveClassifier 
 Returns class probabilities.
   distributionForInstance(Instance)   - 
Method in class weka.classifiers.meta. CVParameterSelection 
 Predicts the class distribution for the given test instance.
   distributionForInstance(Instance)   - 
Method in class weka.classifiers.meta. Dagging 
 Calculates the class membership probabilities for the given test
 instance.
   distributionForInstance(Instance)   - 
Method in class weka.classifiers.meta. Decorate 
 Calculates the class membership probabilities for the given test instance.
   distributionForInstance(Instance)   - 
Method in class weka.classifiers.meta. END 
 Calculates the class membership probabilities for the given test
 instance.
   distributionForInstance(Instance)   - 
Method in class weka.classifiers.meta. FilteredClassifier 
 Classifies a given instance after filtering.
   distributionForInstance(Instance)   - 
Method in class weka.classifiers.meta. Grading 
 Returns class probabilities for a given instance using the stacked classifier.
   distributionForInstance(Instance)   - 
Method in class weka.classifiers.meta. LogitBoost 
 Calculates the class membership probabilities for the given test instance.
   distributionForInstance(Instance)   - 
Method in class weka.classifiers.meta. MetaCost 
 Classifies a given instance after filtering.
   distributionForInstance(Instance)   - 
Method in class weka.classifiers.meta. MultiClassClassifier 
 Returns the distribution for an instance.
   distributionForInstance(Instance)   - 
Method in class weka.classifiers.meta. MultiScheme 
 Returns class probabilities.
   distributionForInstance(Instance)   - 
Method in class weka.classifiers.meta.nestedDichotomies. ClassBalancedND 
 Predicts the class distribution for a given instance
   distributionForInstance(Instance)   - 
Method in class weka.classifiers.meta.nestedDichotomies. DataNearBalancedND 
 Predicts the class distribution for a given instance
   distributionForInstance(Instance)   - 
Method in class weka.classifiers.meta.nestedDichotomies. ND 
 Predicts the class distribution for a given instance
   distributionForInstance(Instance)   - 
Method in class weka.classifiers.meta. OrdinalClassClassifier 
 Returns the distribution for an instance.
   distributionForInstance(Instance)   - 
Method in class weka.classifiers.meta. RacedIncrementalLogitBoost 
 Computes class distribution of an instance using the best committee.
   distributionForInstance(Instance)   - 
Method in class weka.classifiers.meta. RandomCommittee 
 Calculates the class membership probabilities for the given test
 instance.
   distributionForInstance(Instance)   - 
Method in class weka.classifiers.meta. RandomSubSpace 
 Calculates the class membership probabilities for the given test
 instance.
   distributionForInstance(Instance)   - 
Method in class weka.classifiers.meta. RotationForest 
 Calculates the class membership probabilities for the given test
 instance.
   distributionForInstance(Instance)   - 
Method in class weka.classifiers.meta. Stacking 
 Returns class probabilities.
   distributionForInstance(Instance)   - 
Method in class weka.classifiers.meta. StackingC 
 Classifies a given instance using the stacked classifier.
   distributionForInstance(Instance)   - 
Method in class weka.classifiers.meta. ThresholdSelector 
 Calculates the class membership probabilities for the given test instance.
   distributionForInstance(Instance)   - 
Method in class weka.classifiers.meta. Vote 
 Classifies a given instance using the selected combination rule.
   distributionForInstance(Instance)   - 
Method in class weka.classifiers.mi. CitationKNN 
 Computes the distribution for a given exemplar
   distributionForInstance(Instance)   - 
Method in class weka.classifiers.mi. MDD 
 Computes the distribution for a given exemplar
   distributionForInstance(Instance)   - 
Method in class weka.classifiers.mi. MIBoost 
 Computes the distribution for a given exemplar
   distributionForInstance(Instance)   - 
Method in class weka.classifiers.mi. MIDD 
 Computes the distribution for a given exemplar
   distributionForInstance(Instance)   - 
Method in class weka.classifiers.mi. MIEMDD 
 Computes the distribution for a given exemplar
   distributionForInstance(Instance)   - 
Method in class weka.classifiers.mi. MILR 
 Computes the distribution for a given exemplar
   distributionForInstance(Instance)   - 
Method in class weka.classifiers.mi. MIOptimalBall 
 Computes the distribution for a given multiple instance
   distributionForInstance(Instance)   - 
Method in class weka.classifiers.mi. MISMO 
 Estimates class probabilities for given instance.
   distributionForInstance(Instance)   - 
Method in class weka.classifiers.mi. MISVM 
 Computes the distribution for a given exemplar
   distributionForInstance(Instance)   - 
Method in class weka.classifiers.mi. MIWrapper 
 Computes the distribution for a given exemplar
   distributionForInstance(Instance)   - 
Method in class weka.classifiers.mi. SimpleMI 
 Computes the distribution for a given exemplar
   distributionForInstance(Instance)   - 
Method in class weka.classifiers.misc. HyperPipes 
 Classifies the given test instance.
   distributionForInstance(Instance)   - 
Method in class weka.classifiers.misc. SerializedClassifier 
 Calculates the class membership probabilities for the given test
 instance.
   distributionForInstance(Instance)   - 
Method in class weka.classifiers.misc. VFI 
 Classifies the given test instance.
   distributionForInstance(Instance)   - 
Method in class weka.classifiers.pmml.consumer. GeneralRegression 
 Classifies the given test instance.
   distributionForInstance(Instance)   - 
Method in class weka.classifiers.pmml.consumer. NeuralNetwork 
 Classifies the given test instance.
   distributionForInstance(Instance)   - 
Method in class weka.classifiers.pmml.consumer. Regression 
 Classifies the given test instance.
   distributionForInstance(Instance)   - 
Method in class weka.classifiers.rules. ConjunctiveRule 
 Computes class distribution for the given instance.
   distributionForInstance(Instance)   - 
Method in class weka.classifiers.rules. DecisionTable 
 Calculates the class membership probabilities for the given 
 test instance.
   distributionForInstance(Instance)   - 
Method in class weka.classifiers.rules. DTNB 
 Calculates the class membership probabilities for the given 
 test instance.
   distributionForInstance(Instance)   - 
Method in class weka.classifiers.rules. JRip 
 Classify the test instance with the rule learner and provide
 the class distributions
   distributionForInstance(Instance)   - 
Method in class weka.classifiers.rules.part. ClassifierDecList 
 Returns class probabilities for a weighted instance.
   distributionForInstance(Instance)   - 
Method in class weka.classifiers.rules. PART 
 Returns class probabilities for an instance.
   distributionForInstance(Instance)   - 
Method in class weka.classifiers.rules.part. MakeDecList 
 Returns the class distribution for an instance.
   distributionForInstance(Instance)   - 
Method in class weka.classifiers.rules. ZeroR 
 Calculates the class membership probabilities for the given test instance.
   distributionForInstance(Instance)   - 
Method in class weka.classifiers.trees. ADTree 
 Returns the class probability distribution for an instance.
   distributionForInstance(Instance)   - 
Method in class weka.classifiers.trees. BFTree 
 Computes class probabilities for instance using the decision tree.
   distributionForInstance(Instance)   - 
Method in class weka.classifiers.trees. DecisionStump 
 Calculates the class membership probabilities for the given test instance.
   distributionForInstance(Instance)   - 
Method in class weka.classifiers.trees. FT 
 Returns class probabilities for an instance.
   distributionForInstance(Instance)   - 
Method in class weka.classifiers.trees.ft. FTInnerNode 
 Returns the class probabilities for an instance given by the Functional tree.
   distributionForInstance(Instance)   - 
Method in class weka.classifiers.trees.ft. FTLeavesNode 
 Returns the class probabilities for an instance given by the Functional Leaves tree.
   distributionForInstance(Instance)   - 
Method in class weka.classifiers.trees.ft. FTNode 
 Returns the class probabilities for an instance given by the Functional Tree.
   distributionForInstance(Instance)   - 
Method in class weka.classifiers.trees.ft. FTtree 
 Returns the class probabilities for an instance given by the Functional tree.
   distributionForInstance(Instance)   - 
Method in class weka.classifiers.trees. Id3 
 Computes class distribution for instance using decision tree.
   distributionForInstance(Instance, boolean)   - 
Method in class weka.classifiers.trees.j48. ClassifierTree 
 Returns class probabilities for a weighted instance.
   distributionForInstance(Instance)   - 
Method in class weka.classifiers.trees. J48 
 Returns class probabilities for an instance.
   distributionForInstance(Instance)   - 
Method in class weka.classifiers.trees. J48graft 
 Returns class probabilities for an instance.
   distributionForInstance(Instance)   - 
Method in class weka.classifiers.trees. LADTree 
 Returns the class probability distribution for an instance.
   distributionForInstance(Instance)   - 
Method in class weka.classifiers.trees. LMT 
 Returns class probabilities for an instance.
   distributionForInstance(Instance)   - 
Method in class weka.classifiers.trees.lmt. LMTNode 
 Returns the class probabilities for an instance given by the logistic model tree.
   distributionForInstance(Instance)   - 
Method in class weka.classifiers.trees.lmt. LogisticBase 
 Returns class probabilities for an instance.
   distributionForInstance(Instance)   - 
Method in class weka.classifiers.trees. NBTree 
 Returns class probabilities for an instance.
   distributionForInstance(Instance)   - 
Method in class weka.classifiers.trees. RandomForest 
 Returns the class probability distribution for an instance.
   distributionForInstance(Instance)   - 
Method in class weka.classifiers.trees. RandomTree 
 Computes class distribution of an instance using the decision tree.
   distributionForInstance(Instance)   - 
Method in class weka.classifiers.trees. REPTree 
 Computes class distribution of an instance using the tree.
   distributionForInstance(Instance)   - 
Method in class weka.classifiers.trees. SimpleCart 
 Computes class probabilities for instance using the decision tree.
   distributionForInstance(Instance)   - 
Method in class weka.classifiers.trees. UserClassifier 
 Call this function to get a double array filled with the probability
 of how likely each class type is the class of the instance.
   distributionForInstance(Instance)   - 
Method in class weka.clusterers. AbstractClusterer 
 Predicts the cluster memberships for a given instance.
   distributionForInstance(Instance)   - 
Method in class weka.clusterers. AbstractDensityBasedClusterer 
 Returns the cluster probability distribution for an instance.
   distributionForInstance(Instance)   - 
Method in interface weka.clusterers. Clusterer 
 Predicts the cluster memberships for a given instance.
   distributionForInstance(Instance)   - 
Method in class weka.clusterers. FilteredClusterer 
 Classifies a given instance after filtering.
   distributionForInstance(Instance)   - 
Method in class weka.clusterers. HierarchicalClusterer 
  
   distributionsByOriginalIndex(double[])   - 
Method in class weka.filters.supervised.attribute. ClassOrder 
 Convert the given class distribution back to the distributions
 with the original internal class index
   distributionSpreadTipText()   - 
Method in class weka.filters.supervised.instance. SpreadSubsample 
 Returns the tip text for this property
   distributionTipText()   - 
Method in class weka.filters.unsupervised.attribute. RandomProjection 
 Returns the tip text for this property
   divergence(BayesNet)   - 
Method in class weka.classifiers.bayes.net. BIFReader 
 calculates the divergence between the probability distribution
 represented by this network and that of another, that is,
 \sum_{x\in X} P(x)log P(x)/Q(x)
 where X is the set of values the nodes in the network can take,
 P(x) the probability of this network for configuration x
 Q(x) the probability of the other network for configuration x
   divide(Instances, boolean)   - 
Static method in class weka.associations. LabeledItemSet 
 Splits the class attribute away.
   dividedBy(DoubleVector)   - 
Method in class weka.core.matrix. DoubleVector 
 Divided by another DoubleVector element by element
   dividedByEquals(DoubleVector)   - 
Method in class weka.core.matrix. DoubleVector 
 Divided by another DoubleVector element by element in place
   DIVISION   - 
Static variable in interface weka.core.mathematicalexpression. sym 
  
   DIVISION   - 
Static variable in interface weka.filters.unsupervised.instance.subsetbyexpression. sym 
  
   DKConditionalEstimator   - Class in  weka.estimators  Conditional probability estimator for a discrete domain conditional upon
 a numeric domain.   DKConditionalEstimator(int, double)   - 
Constructor for class weka.estimators. DKConditionalEstimator 
 Constructor
   dl(int)   - 
Method in class weka.core. Debug.DBO 
 Return true if the debug level is set
 same method as outpuTypeSet but better name
   DMNBtext   - Class in  weka.classifiers.bayes  Class for building and using a Discriminative Multinomial Naive Bayes classifier.   DMNBtext()   - 
Constructor for class weka.classifiers.bayes. DMNBtext 
  
   DMNBtext.DNBBinary   - Class in  weka.classifiers.bayes      DMNBtext.DNBBinary()   - 
Constructor for class weka.classifiers.bayes. DMNBtext.DNBBinary 
  
   DNConditionalEstimator   - Class in  weka.estimators  Conditional probability estimator for a discrete domain conditional upon
 a numeric domain.   DNConditionalEstimator(int, double)   - 
Constructor for class weka.estimators. DNConditionalEstimator 
 Constructor
   dnorm(double)   - 
Static method in class weka.core.matrix. Maths 
 Returns the density of the standard normal.
   dnorm(double, double, double)   - 
Static method in class weka.core.matrix. Maths 
 Returns the density value of a standard normal.
   dnorm(double, DoubleVector, double)   - 
Static method in class weka.core.matrix. Maths 
 Returns the density values of a set of normal distributions with
  different means.
   dnormLog(double)   - 
Static method in class weka.core.matrix. Maths 
 Returns the log-density of the standard normal.
   dnormLog(double, double, double)   - 
Static method in class weka.core.matrix. Maths 
 Returns the log-density value of a standard normal.
   dnormLog(double, DoubleVector, double)   - 
Static method in class weka.core.matrix. Maths 
 Returns the log-density values of a set of normal distributions with
  different means.
   do_action(int, lr_parser, Stack, int)   - 
Method in class weka.core.mathematicalexpression. Parser 
 Invoke a user supplied parse action.
   do_action(int, lr_parser, Stack, int)   - 
Method in class weka.filters.unsupervised.instance.subsetbyexpression. Parser 
 Invoke a user supplied parse action.
   doCommandlineCompletion(KeyEvent)   - 
Method in class weka.gui. SimpleCLIPanel 
 performs commandline completion on packages and classnames.
   DOCTYPE   - 
Static variable in class weka.core.xml. XMLInstances 
 the DTD
   DOCTYPE   - 
Static variable in class weka.core.xml. XMLOptions 
 the DTD for the XML file.
   DOCTYPE   - 
Static variable in class weka.core.xml. XMLSerialization 
 the DOCTYPE for the serialization
   doGrafting(Instances)   - 
Method in class weka.classifiers.trees.j48. C45PruneableClassifierTreeG 
 Initializes variables for grafting.
   doHistory(KeyEvent)   - 
Method in class weka.gui. SimpleCLIPanel 
 Changes the currently displayed command line when certain keys
 are pressed.
   doMetaConnection(BeanInstance, BeanInstance, EventSetDescriptor, JComponent)   - 
Static method in class weka.gui.beans. BeanConnection 
  
   done()   - 
Method in interface weka.classifiers. IterativeClassifier 
 Signal end of iterating, useful for any house-keeping/cleanup
   done()   - 
Method in class weka.classifiers.pmml.consumer. PMMLClassifier 
 Signal that a scoring run has been completed.
   done()   - 
Method in class weka.classifiers.trees. ADTree 
 Frees memory that is no longer needed for a final model - will no longer be able
 to increment the classifier after calling this.
   done()   - 
Method in class weka.classifiers.trees. LADTree 
  
   doNotOperateOnPerClassBasisTipText()   - 
Method in class weka.filters.unsupervised.attribute. StringToWordVector 
 Returns the tip text for this property.
   doNotReplaceMissingValuesTipText()   - 
Method in class weka.classifiers.functions. LibLINEAR 
 Returns the tip text for this property
   doNotReplaceMissingValuesTipText()   - 
Method in class weka.classifiers.functions. LibSVM 
 Returns the tip text for this property
   dontFilterAfterFirstBatchTipText()   - 
Method in class weka.filters.unsupervised.instance. RemoveWithValues 
 Returns the tip text for this property.
   dontNormalizeTipText()   - 
Method in class weka.classifiers.functions. SPegasos 
 Returns the tip text for this property
   dontNormalizeTipText()   - 
Method in class weka.core. NormalizableDistance 
 Returns the tip text for this property.
   dontReplaceMissingTipText()   - 
Method in class weka.classifiers.functions. SPegasos 
 Returns the tip text for this property
   dontReplaceMissingValuesTipText()   - 
Method in class weka.clusterers. SimpleKMeans 
 Returns the tip text for this property
   doRun(int)   - 
Method in class weka.experiment. AveragingResultProducer 
 Gets the results for a specified run number.
   doRun(int)   - 
Method in class weka.experiment. CrossValidationResultProducer 
 Gets the results for a specified run number.
   doRun(int)   - 
Method in class weka.experiment. DatabaseResultProducer 
 Gets the results for a specified run number.
   doRun(int)   - 
Method in class weka.experiment. LearningRateResultProducer 
 Gets the results for a specified run number.
   doRun(int)   - 
Method in class weka.experiment. RandomSplitResultProducer 
 Gets the results for a specified run number.
   doRun(int)   - 
Method in interface weka.experiment. ResultProducer 
 Gets the results for a specified run number.
   doRunKeys(int)   - 
Method in class weka.experiment. AveragingResultProducer 
 Gets the keys for a specified run number.
   doRunKeys(int)   - 
Method in class weka.experiment. CrossValidationResultProducer 
 Gets the keys for a specified run number.
   doRunKeys(int)   - 
Method in class weka.experiment. DatabaseResultProducer 
 Gets the keys for a specified run number.
   doRunKeys(int)   - 
Method in class weka.experiment. LearningRateResultProducer 
 Gets the keys for a specified run number.
   doRunKeys(int)   - 
Method in class weka.experiment. RandomSplitResultProducer 
 Gets the keys for a specified run number.
   doRunKeys(int)   - 
Method in interface weka.experiment. ResultProducer 
 Gets the keys for a specified run number.
   doTests()   - 
Method in class weka.associations. CheckAssociator 
 Begin the tests, reporting results to System.out
   doTests()   - 
Method in class weka.attributeSelection. CheckAttributeSelection 
 Begin the tests, reporting results to System.out
   doTests()   - 
Method in class weka.classifiers. CheckClassifier 
 Begin the tests, reporting results to System.out
   doTests()   - 
Method in class weka.classifiers.functions.supportVector. CheckKernel 
 Begin the tests, reporting results to System.out
   doTests()   - 
Method in class weka.clusterers. CheckClusterer 
 Begin the tests, reporting results to System.out
   doTests()   - 
Method in class weka.core. Check 
 Begin the tests, reporting results to System.out
   doTests()   - 
Method in class weka.core. CheckGOE 
 Runs some diagnostic tests on the object.
   doTests()   - 
Method in class weka.core. CheckOptionHandler 
 Runs some diagnostic tests on an optionhandler object.
   doTests()   - 
Method in class weka.core. CheckScheme 
 Begin the tests, reporting results to System.out
   doTests()   - 
Method in class weka.estimators. CheckEstimator 
 Begin the tests, reporting results to System.out
   dotMultiply(AlgVector)   - 
Method in class weka.core. AlgVector 
 Returns the inner (or dot) product of two vectors
   DotParser   - Class in  weka.gui.graphvisualizer  This class parses input in DOT format, and
 builds the datastructures that are passed to it.   DotParser(Reader, FastVector, FastVector)   - 
Constructor for class weka.gui.graphvisualizer. DotParser 
 Dot parser Constructor
   DOUBLE   - 
Static variable in class weka.experiment. DatabaseUtils 
 Type mapping for DOUBLE used for reading experiment results.
   DOUBLE   - 
Static variable in interface weka.gui.graphvisualizer. GraphConstants 
 Types of Edges
   doubleToString(double, int)   - 
Static method in class weka.core. Utils 
 Rounds a double and converts it into String.
   doubleToString(double, int, int)   - 
Static method in class weka.core. Utils 
 Rounds a double and converts it into a formatted decimal-justified String.
   DoubleVector   - Class in  weka.core.matrix  A vector specialized on doubles.   DoubleVector()   - 
Constructor for class weka.core.matrix. DoubleVector 
 Constructs a null vector.
   DoubleVector(int)   - 
Constructor for class weka.core.matrix. DoubleVector 
 Constructs an n-vector of zeros.
   DoubleVector(int, double)   - 
Constructor for class weka.core.matrix. DoubleVector 
 Constructs a constant n-vector.
   DoubleVector(double[])   - 
Constructor for class weka.core.matrix. DoubleVector 
 Constructs a vector directly from a double array
   dp(String)   - 
Method in class weka.core. Debug.DBO 
 prints out text  if verbose is on.
   dp(int, String)   - 
Method in class weka.core. Debug.DBO 
 prints out text but only if debug level is set.
   dpln(String)   - 
Method in class weka.core. Debug.DBO 
 prints out text + endofline if verbose is on.
   dpln(int, String)   - 
Method in class weka.core. Debug.DBO 
 prints out text + endofline but only if parameter debug type is set.
   draw(Shape)   - 
Method in class weka.gui.visualize. PostscriptGraphics 
  
   draw3DRect(int, int, int, int, boolean)   - 
Method in class weka.gui.visualize. PostscriptGraphics 
 Draw an outlined rectangle with 3D effect in current pen color.
   Drawable   - Interface in  weka.core  Interface to something that can be drawn as a graph.   drawArc(int, int, int, int, int, int)   - 
Method in class weka.gui.visualize. PostscriptGraphics 
 Not implemented
   drawBytes(byte[], int, int, int, int)   - 
Method in class weka.gui.visualize. PostscriptGraphics 
 simply calls drawString(String,int,int)
   drawChars(char[], int, int, int, int)   - 
Method in class weka.gui.visualize. PostscriptGraphics 
 simply calls drawString(String,int,int)
   drawGlyphVector(GlyphVector, float, float)   - 
Method in class weka.gui.visualize. PostscriptGraphics 
  
   drawHighlight(Graphics, int, int)   - 
Method in class weka.classifiers.functions.neural. NeuralConnection 
 Call this function to draw the node highlighted.
   drawImage(Image, int, int, Color, ImageObserver)   - 
Method in class weka.gui.visualize. PostscriptGraphics 
 calls drawImage(Image,int,int,int,int,Color,ImageObserver)
   drawImage(Image, int, int, ImageObserver)   - 
Method in class weka.gui.visualize. PostscriptGraphics 
 calls drawImage(Image,int,int,Color,ImageObserver) with Color.WHITE as 
 background color
   drawImage(Image, int, int, int, int, Color, ImageObserver)   - 
Method in class weka.gui.visualize. PostscriptGraphics 
 PS see http://astronomy.swin.edu.au/~pbourke/geomformats/postscript/
 Java http://show.docjava.com:8086/book/cgij/doc/ip/graphics/SimpleImageFrame.java.html
   drawImage(Image, int, int, int, int, ImageObserver)   - 
Method in class weka.gui.visualize. PostscriptGraphics 
 calls drawImage(Image,int,int,int,int,Color,ImageObserver) with the color 
 WHITE as background
   drawImage(Image, int, int, int, int, int, int, int, int, Color, ImageObserver)   - 
Method in class weka.gui.visualize. PostscriptGraphics 
 Not implemented
   drawImage(Image, int, int, int, int, int, int, int, int, ImageObserver)   - 
Method in class weka.gui.visualize. PostscriptGraphics 
 calls drawImage(Image,int,int,int,int,int,int,int,int,Color,ImageObserver)
 with Color.WHITE as background color
   drawImage(BufferedImage, BufferedImageOp, int, int)   - 
Method in class weka.gui.visualize. PostscriptGraphics 
  
   drawImage(Image, AffineTransform, ImageObserver)   - 
Method in class weka.gui.visualize. PostscriptGraphics 
  
   drawInputLines(Graphics, int, int)   - 
Method in class weka.classifiers.functions.neural. NeuralConnection 
 Call this function to draw the nodes input connections.
   drawLine(int, int, int, int)   - 
Method in class weka.gui.visualize. PostscriptGraphics 
 Draw a line in current pen color.
   drawNode(Graphics, int, int)   - 
Method in class weka.classifiers.functions.neural. NeuralConnection 
 Call this function to draw the node.
   drawOutputLines(Graphics, int, int)   - 
Method in class weka.classifiers.functions.neural. NeuralConnection 
 Call this function to draw the nodes output connections.
   drawOval(int, int, int, int)   - 
Method in class weka.gui.visualize. PostscriptGraphics 
 Draw an Oval outline in current pen color.
   drawPolygon(int[], int[], int)   - 
Method in class weka.gui.visualize. PostscriptGraphics 
 Not implemented
   drawPolyline(int[], int[], int)   - 
Method in class weka.gui.visualize. PostscriptGraphics 
 Not implemented
   drawRect(int, int, int, int)   - 
Method in class weka.gui.visualize. PostscriptGraphics 
 Draw an outlined rectangle in current pen color.
   drawRenderableImage(RenderableImage, AffineTransform)   - 
Method in class weka.gui.visualize. PostscriptGraphics 
  
   drawRenderedImage(RenderedImage, AffineTransform)   - 
Method in class weka.gui.visualize. PostscriptGraphics 
  
   drawRoundRect(int, int, int, int, int, int)   - 
Method in class weka.gui.visualize. PostscriptGraphics 
 Not implemented
   drawString(AttributedCharacterIterator, int, int)   - 
Method in class weka.gui.visualize. PostscriptGraphics 
 Not implemented
   drawString(String, int, int)   - 
Method in class weka.gui.visualize. PostscriptGraphics 
 Draw text in current pen color.
   drawString(AttributedCharacterIterator, float, float)   - 
Method in class weka.gui.visualize. PostscriptGraphics 
  
   drawString(String, float, float)   - 
Method in class weka.gui.visualize. PostscriptGraphics 
  
   DTD_ANY   - 
Static variable in class weka.core.xml. XMLDocument 
 the ANY placeholder.
   DTD_AT_LEAST_ONE   - 
Static variable in class weka.core.xml. XMLDocument 
 the at least one marker.
   DTD_ATTLIST   - 
Static variable in class weka.core.xml. XMLDocument 
 the AttList definition.
   DTD_CDATA   - 
Static variable in class weka.core.xml. XMLDocument 
 the CDATA placeholder.
   DTD_DOCTYPE   - 
Static variable in class weka.core.xml. XMLDocument 
 the DocType definition.
   DTD_ELEMENT   - 
Static variable in class weka.core.xml. XMLDocument 
 the Element definition.
   DTD_IMPLIED   - 
Static variable in class weka.core.xml. XMLDocument 
 the #IMPLIED placeholder.
   DTD_OPTIONAL   - 
Static variable in class weka.core.xml. XMLDocument 
 the optional marker.
   DTD_PCDATA   - 
Static variable in class weka.core.xml. XMLDocument 
 the #PCDATA placeholder.
   DTD_REQUIRED   - 
Static variable in class weka.core.xml. XMLDocument 
 the #REQUIRED placeholder.
   DTD_SEPARATOR   - 
Static variable in class weka.core.xml. XMLDocument 
 the option separator.
   DTD_ZERO_OR_MORE   - 
Static variable in class weka.core.xml. XMLDocument 
 the zero or more marker.
   DTNB   - Class in  weka.classifiers.rules  Class for building and using a decision table/naive bayes hybrid classifier.   DTNB()   - 
Constructor for class weka.classifiers.rules. DTNB 
  
   dumpDistribution()   - 
Method in class weka.classifiers.trees.j48. Distribution 
 Prints distribution.
   dumpLabel(int, Instances)   - 
Method in class weka.classifiers.trees.j48. ClassifierSplitModel 
 Prints label for subset index of instances (eg class).
   dumpLabelG(int, Instances)   - 
Method in class weka.classifiers.trees.j48. GraftSplit 
 Prints label for subset index of instances (eg class).
   dumpModel(Instances)   - 
Method in class weka.classifiers.trees.j48. ClassifierSplitModel 
 Prints the split model.
 
 
    
 E  
 
   EAST_CONNECTOR   - 
Static variable in class weka.gui.beans. BeanVisual 
  
   Edge   - Class in  weka.gui.treevisualizer  This class is used in conjunction with the Node class to form a tree 
 structure.   Edge(String, String, String)   - 
Constructor for class weka.gui.treevisualizer. Edge 
 This constructs an Edge with the specified label 
 and parent , child serial tags.
   edit()   - 
Method in class weka.gui.explorer. PreprocessPanel 
 edits the current instances object in the viewer
   EditableBayesNet   - Class in  weka.classifiers.bayes.net  Bayes Network learning using various search algorithms and quality measures. 
 Base class for a Bayes Network classifier.   EditableBayesNet()   - 
Constructor for class weka.classifiers.bayes.net. EditableBayesNet 
 standard constructor *
   EditableBayesNet(Instances)   - 
Constructor for class weka.classifiers.bayes.net. EditableBayesNet 
 constructor, creates empty network with nodes based on the attributes in a data set
   EditableBayesNet(BIFReader)   - 
Constructor for class weka.classifiers.bayes.net. EditableBayesNet 
 constructor, copies Bayesian network structure from a Bayesian network
 encapsulated in a BIFReader
   EditableBayesNet(boolean)   - 
Constructor for class weka.classifiers.bayes.net. EditableBayesNet 
 constructor that potentially initializes instances as well
   editableProperties()   - 
Method in class weka.gui. PropertySheetPanel 
 Gets the number of editable properties for the current target.
   EditDistance   - Class in  weka.core  Computes the Levenshtein edit distance between two strings.   EditDistance()   - 
Constructor for class weka.core. EditDistance 
  
   EditDistance(Instances)   - 
Constructor for class weka.core. EditDistance 
  
   eig()   - 
Method in class weka.core.matrix. Matrix 
 Eigenvalue Decomposition
   EigenvalueDecomposition   - Class in  weka.core.matrix  Eigenvalues and eigenvectors of a real matrix.   eigenvalueDecomposition(double[][], double[])   - 
Method in class weka.core. Matrix 
  Deprecated.  Performs Eigenvalue Decomposition using Householder QR Factorization

 Matrix must be symmetrical.
   EigenvalueDecomposition(Matrix)   - 
Constructor for class weka.core.matrix. EigenvalueDecomposition 
 Check for symmetry, then construct the eigenvalue decomposition
   Element   - Class in  weka.associations.gsp  Class representing an Element, i.e., a set of events/items.   Element()   - 
Constructor for class weka.associations.gsp. Element 
 Constructor
   Element(int)   - 
Constructor for class weka.associations.gsp. Element 
 Constructor accepting an initial size of the events Array as parameter.
   element(int)   - 
Method in class weka.core.neighboursearch.covertrees. Stack 
 Returns the ith element in the stack.
   elementAt(int)   - 
Method in class weka.core. FastVector 
 Returns the element at the given position.
   elementAt(int)   - 
Method in class weka.gui. CheckBoxList.CheckBoxListModel 
 Returns the component at the specified index.
   elements()   - 
Method in class weka.core. FastVector 
 Returns an enumeration of this vector.
   elements(int)   - 
Method in class weka.core. FastVector 
 Returns an enumeration of this vector, skipping the
 element with the given index.
   elements   - 
Variable in class weka.core.neighboursearch.covertrees. Stack 
 The elements inside the stack.
   elements()   - 
Method in class weka.core. Stopwords 
 Returns a sorted enumeration over all stored stopwords
   eliminateColinearAttributesTipText()   - 
Method in class weka.classifiers.functions. LinearRegression 
 Returns the tip text for this property
   EM   - Class in  weka.clusterers  Simple EM (expectation maximisation) class. 
  
 EM assigns a probability distribution to each instance which indicates the probability of it belonging to each of the clusters.   EM()   - 
Constructor for class weka.clusterers. EM 
 Constructor.
   empiricalBayesEstimate(double)   - 
Method in class weka.classifiers.functions.pace. NormalMixture 
 Returns the empirical Bayes estimate of a single value.
   empiricalBayesEstimate(DoubleVector)   - 
Method in class weka.classifiers.functions.pace. NormalMixture 
 Returns the empirical Bayes estimate of a vector.
   empiricalProbability(DoubleVector, PaceMatrix)   - 
Method in class weka.classifiers.functions.pace. MixtureDistribution 
 Computes the empirical probabilities of the data over a set of
  intervals.
   empty()   - 
Method in class weka.core. Queue 
 Checks if queue is empty.
   enable(Capabilities.Capability)   - 
Method in class weka.core. Capabilities 
 enables the given capability.
   enable(Capabilities.Capability)   - 
Method in class weka.core. FindWithCapabilities 
 enables the given capability.
   enableAll()   - 
Method in class weka.core. Capabilities 
 enables all attribute and class types (including dependencies)
   enableAllAttributeDependencies()   - 
Method in class weka.core. Capabilities 
 enables all attribute type dependencies
   enableAllAttributes()   - 
Method in class weka.core. Capabilities 
 enables all attribute types
   enableAllClassDependencies()   - 
Method in class weka.core. Capabilities 
 enables all class type dependencies
   enableAllClasses()   - 
Method in class weka.core. Capabilities 
 enables all class types
   enableDependency(Capabilities.Capability)   - 
Method in class weka.core. Capabilities 
 enables the dependency flag for the given capability
 Enabling NOMINAL_ATTRIBUTES also enables BINARY_ATTRIBUTES, 
 UNARY_ATTRIBUTES and EMPTY_NOMINAL_ATTRIBUTES.
   enableNot(Capabilities.Capability)   - 
Method in class weka.core. FindWithCapabilities 
 enables the given "not to have" capability.
   END   - Class in  weka.classifiers.meta  A meta classifier for handling multi-class datasets with 2-class classifiers by building an ensemble of nested dichotomies. 
  
 For more info, check 
  
 Lin Dong, Eibe Frank, Stefan Kramer: Ensembles of Balanced Nested Dichotomies for Multi-class Problems.   END()   - 
Constructor for class weka.classifiers.meta. END 
 Constructor.
   entropicAutoBlendTipText()   - 
Method in class weka.classifiers.lazy. KStar 
 Returns the tip text for this property
   ENTROPY   - 
Static variable in interface weka.classifiers.bayes.net.search.local. Scoreable 
  
   entropy(double[])   - 
Static method in class weka.core. ContingencyTables 
 Computes the entropy of the given array.
   EntropyBasedSplitCrit   - Class in  weka.classifiers.trees.j48  "Abstract" class for computing splitting criteria
 based on the entropy of a class distribution.   EntropyBasedSplitCrit()   - 
Constructor for class weka.classifiers.trees.j48. EntropyBasedSplitCrit 
  
   entropyConditionedOnColumns(double[][])   - 
Static method in class weka.core. ContingencyTables 
 Computes conditional entropy of the rows given
 the columns.
   entropyConditionedOnRows(double[][])   - 
Static method in class weka.core. ContingencyTables 
 Computes conditional entropy of the columns given
 the rows.
   entropyConditionedOnRows(double[][], double[][], double)   - 
Static method in class weka.core. ContingencyTables 
 Computes conditional entropy of the columns given the rows
 of the test matrix with respect to the train matrix.
   entropyGain()   - 
Method in class weka.classifiers.trees.lmt. ResidualSplit 
 Computes entropy gain for current split.
   entropyOverColumns(double[][])   - 
Static method in class weka.core. ContingencyTables 
 Computes the columns' entropy for the given contingency table.
   entropyOverRows(double[][])   - 
Static method in class weka.core. ContingencyTables 
 Computes the rows' entropy for the given contingency table.
   EntropySplitCrit   - Class in  weka.classifiers.trees.j48  Class for computing the entropy for a given distribution.   EntropySplitCrit()   - 
Constructor for class weka.classifiers.trees.j48. EntropySplitCrit 
  
   enumerateAttributes()   - 
Method in class weka.core. Instance 
 Returns an enumeration of all the attributes.
   enumerateAttributes()   - 
Method in class weka.core. Instances 
 Returns an enumeration of all the attributes.
   enumerateInstances()   - 
Method in class weka.core. Instances 
 Returns an enumeration of all instances in the dataset.
   enumerateLiterals()   - 
Method in class weka.associations.tertius. LiteralSet 
 Enumerate the literals contained in this set.
   enumerateMeasures()   - 
Method in class weka.classifiers.bayes. BayesNet 
 Returns an enumeration of the measure names.
   enumerateMeasures()   - 
Method in class weka.classifiers.functions. SimpleLogistic 
 Returns an enumeration of the additional measure names
   enumerateMeasures()   - 
Method in class weka.classifiers.functions. SMOreg 
 Returns an enumeration of the measure names.
   enumerateMeasures()   - 
Method in class weka.classifiers.lazy. IBk 
 Returns an enumeration of the additional measure names 
 produced by the neighbour search algorithm, plus the chosen K in case
 cross-validation is enabled.
   enumerateMeasures()   - 
Method in class weka.classifiers.lazy. LWL 
 Returns an enumeration of the additional measure names 
 produced by the neighbour search algorithm.
   enumerateMeasures()   - 
Method in class weka.classifiers.meta. AdditiveRegression 
 Returns an enumeration of the additional measure names
   enumerateMeasures()   - 
Method in class weka.classifiers.meta. AttributeSelectedClassifier 
 Returns an enumeration of the additional measure names
   enumerateMeasures()   - 
Method in class weka.classifiers.meta. Bagging 
 Returns an enumeration of the additional measure names.
   enumerateMeasures()   - 
Method in class weka.classifiers.meta. GridSearch 
 Returns an enumeration of the measure names.
   enumerateMeasures()   - 
Method in class weka.classifiers.rules. DecisionTable 
 Returns an enumeration of the additional measure names
   enumerateMeasures()   - 
Method in class weka.classifiers.rules. DTNB 
 Returns an enumeration of the additional measure names
   enumerateMeasures()   - 
Method in class weka.classifiers.rules. JRip 
 Returns an enumeration of the additional measure names
   enumerateMeasures()   - 
Method in class weka.classifiers.rules. PART 
 Returns an enumeration of the additional measure names
   enumerateMeasures()   - 
Method in class weka.classifiers.rules. Ridor 
 Returns an enumeration of the additional measure names
   enumerateMeasures()   - 
Method in class weka.classifiers.trees. ADTree 
 Returns an enumeration of the additional measure names.
   enumerateMeasures()   - 
Method in class weka.classifiers.trees. BFTree 
 Return an enumeration of the measure names.
   enumerateMeasures()   - 
Method in class weka.classifiers.trees. FT 
 Returns an enumeration of the additional measure names
   enumerateMeasures()   - 
Method in class weka.classifiers.trees. J48 
 Returns an enumeration of the additional measure names
   enumerateMeasures()   - 
Method in class weka.classifiers.trees. J48graft 
 Returns an enumeration of the additional measure names
   enumerateMeasures()   - 
Method in class weka.classifiers.trees. LADTree 
 Returns an enumeration of the additional measure names.
   enumerateMeasures()   - 
Method in class weka.classifiers.trees. LMT 
 Returns an enumeration of the additional measure names
   enumerateMeasures()   - 
Method in class weka.classifiers.trees.m5. M5Base 
 Returns an enumeration of the additional measure names
   enumerateMeasures()   - 
Method in class weka.classifiers.trees. NBTree 
 Returns an enumeration of the additional measure names
   enumerateMeasures()   - 
Method in class weka.classifiers.trees. RandomForest 
 Returns an enumeration of the additional measure names.
   enumerateMeasures()   - 
Method in class weka.classifiers.trees. REPTree 
 Returns an enumeration of the additional measure names.
   enumerateMeasures()   - 
Method in class weka.classifiers.trees. SimpleCart 
 Return an enumeration of the measure names.
   enumerateMeasures()   - 
Method in interface weka.core. AdditionalMeasureProducer 
 Returns an enumeration of the measure names.
   enumerateMeasures()   - 
Method in class weka.core.neighboursearch. BallTree 
 Returns an enumeration of the additional measure names.
   enumerateMeasures()   - 
Method in class weka.core.neighboursearch. CoverTree 
 Returns an enumeration of the additional measure names.
   enumerateMeasures()   - 
Method in class weka.core.neighboursearch. KDTree 
 Returns an enumeration of the additional measure names.
   enumerateMeasures()   - 
Method in class weka.core.neighboursearch. NearestNeighbourSearch 
 Returns an enumeration of the additional measure names.
   enumerateMeasures()   - 
Method in class weka.core.neighboursearch. PerformanceStats 
 Returns an enumeration of the additional measure names.
   enumerateMeasures()   - 
Method in class weka.core.neighboursearch. TreePerformanceStats 
 Returns an enumeration of the additional measure names.
   enumerateMeasures()   - 
Method in class weka.experiment. AveragingResultProducer 
 Returns an enumeration of any additional measure names that might be
 in the result producer
   enumerateMeasures()   - 
Method in class weka.experiment. ClassifierSplitEvaluator 
 Returns an enumeration of any additional measure names that might be
 in the classifier
   enumerateMeasures()   - 
Method in class weka.experiment. CrossValidationResultProducer 
 Returns an enumeration of any additional measure names that might be
 in the SplitEvaluator
   enumerateMeasures()   - 
Method in class weka.experiment. DatabaseResultProducer 
 Returns an enumeration of any additional measure names that might be
 in the result producer
   enumerateMeasures()   - 
Method in class weka.experiment. DensityBasedClustererSplitEvaluator 
 Returns an enumeration of any additional measure names that might be
 in the classifier
   enumerateMeasures()   - 
Method in class weka.experiment. LearningRateResultProducer 
 Returns an enumeration of any additional measure names that might be
 in the result producer
   enumerateMeasures()   - 
Method in class weka.experiment. RandomSplitResultProducer 
 Returns an enumeration of any additional measure names that might be
 in the SplitEvaluator
   enumerateMeasures()   - 
Method in class weka.experiment. RegressionSplitEvaluator 
 Returns an enumeration of any additional measure names that might be
 in the classifier
   enumerateRequests()   - 
Method in class weka.gui.beans. Associator 
 Return an enumeration of requests that can be made by the user
   enumerateRequests()   - 
Method in class weka.gui.beans. AttributeSummarizer 
 Return an enumeration of actions that the user can ask this bean to
 perform
   enumerateRequests()   - 
Method in class weka.gui.beans. Classifier 
 Return an enumeration of requests that can be made by the user
   enumerateRequests()   - 
Method in class weka.gui.beans. ClassifierPerformanceEvaluator 
 Return an enumeration of user activated requests for this bean
   enumerateRequests()   - 
Method in class weka.gui.beans. Clusterer 
 Return an enumeration of requests that can be made by the user
   enumerateRequests()   - 
Method in class weka.gui.beans. ClustererPerformanceEvaluator 
 Return an enumeration of user activated requests for this bean
   enumerateRequests()   - 
Method in class weka.gui.beans. CostBenefitAnalysis 
  
   enumerateRequests()   - 
Method in class weka.gui.beans. CrossValidationFoldMaker 
 Return an enumeration of user requests
   enumerateRequests()   - 
Method in class weka.gui.beans. DataVisualizer 
 Describe  enumerateRequests  method here.
   enumerateRequests()   - 
Method in class weka.gui.beans. Filter 
 Return an enumeration of user requests
   enumerateRequests()   - 
Method in class weka.gui.beans. GraphViewer 
 Return an enumeration of user requests
   enumerateRequests()   - 
Method in class weka.gui.beans. MetaBean 
 Return an enumeration of requests that can be made by the user
   enumerateRequests()   - 
Method in class weka.gui.beans. ModelPerformanceChart 
 Describe  enumerateRequests  method here.
   enumerateRequests()   - 
Method in class weka.gui.beans. StripChart 
 Describe  enumerateRequests  method here.
   enumerateRequests()   - 
Method in class weka.gui.beans. TextViewer 
 Get a list of user requests
   enumerateRequests()   - 
Method in class weka.gui.beans. TrainTestSplitMaker 
 Get list of user requests
   enumerateRequests()   - 
Method in interface weka.gui.beans. UserRequestAcceptor 
 Get a list of performable requests
   enumerateValues()   - 
Method in class weka.core. Attribute 
 Returns an enumeration of all the attribute's values if the
 attribute is nominal, string, or relation-valued, null otherwise.
   Environment   - Class in  weka.core  This class encapsulates a map of all environment and java system properties.   Environment()   - 
Constructor for class weka.core. Environment 
  
   EnvironmentHandler   - Interface in  weka.core  Interface for something that can utilize environment
 variables.   EOF   - 
Static variable in interface weka.core.mathematicalexpression. sym 
  
   EOF   - 
Static variable in interface weka.filters.unsupervised.instance.subsetbyexpression. sym 
  
   EOF_sym()   - 
Method in class weka.core.mathematicalexpression. Parser 
  EOF  Symbol index.
   EOF_sym()   - 
Method in class weka.filters.unsupervised.instance.subsetbyexpression. Parser 
  EOF  Symbol index.
   epochsTipText()   - 
Method in class weka.classifiers.functions. SPegasos 
 Returns the tip text for this property
   EPSILON   - 
Static variable in interface weka.classifiers.lazy.kstar. KStarConstants 
  
   epsilonParameterTipText()   - 
Method in class weka.attributeSelection. SVMAttributeEval 
 Returns a tip text for this property suitable for display in the
 GUI
   epsilonParameterTipText()   - 
Method in class weka.classifiers.functions.supportVector. RegOptimizer 
 Returns the tip text for this property
   EpsilonRange_ListElement   - Class in  weka.clusterers.forOPTICSAndDBScan.Utils  
 EpsilonRange_ListElement.java  
 Authors: Rainer Holzmann, Zhanna Melnikova-Albrecht, Matthias Schubert  
 Date: Sep 7, 2004  
 Time: 2:12:34 PM  
 $ Revision 1.4 $     EpsilonRange_ListElement(double, DataObject)   - 
Constructor for class weka.clusterers.forOPTICSAndDBScan.Utils. EpsilonRange_ListElement 
 Constructs a new Element that is stored in the ArrayList which is
 built in the k_nextNeighbourQuery-method from a specified database.
   epsilonRangeQuery(double, DataObject)   - 
Method in interface weka.clusterers.forOPTICSAndDBScan.Databases. Database 
 Performs an epsilon range query for this dataObject
   epsilonRangeQuery(double, DataObject)   - 
Method in class weka.clusterers.forOPTICSAndDBScan.Databases. SequentialDatabase 
 Performs an epsilon range query for this dataObject
   epsilonTipText()   - 
Method in class weka.classifiers.functions. SMO 
 Returns the tip text for this property
   epsilonTipText()   - 
Method in class weka.classifiers.functions.supportVector. RegSMO 
 Returns the tip text for this property
   epsilonTipText()   - 
Method in class weka.classifiers.mi. MISMO 
 Returns the tip text for this property
   epsilonTipText()   - 
Method in class weka.clusterers. DBScan 
 Returns the tip text for this property
   epsilonTipText()   - 
Method in class weka.clusterers. OPTICS 
 Returns the tip text for this property
   epsTipText()   - 
Method in class weka.classifiers.functions. LibLINEAR 
 Returns the tip text for this property
   epsTipText()   - 
Method in class weka.classifiers.functions. LibSVM 
 Returns the tip text for this property
   EQ   - 
Static variable in interface weka.core.mathematicalexpression. sym 
  
   eq(double, double)   - 
Static method in class weka.core. Utils 
 Tests if a is equal to b.
   EQ   - 
Static variable in interface weka.filters.unsupervised.instance.subsetbyexpression. sym 
  
   equalCondset(Object)   - 
Method in class weka.associations. LabeledItemSet 
 Compares two item sets
   equalExemplars(Instance, Instance)   - 
Method in class weka.classifiers.mi. CitationKNN 
 Wether the instances of two exemplars are or  are not equal
   equalHeaders(Instance)   - 
Method in class weka.core. Instance 
 Tests if the headers of two instances are equivalent.
   equalHeaders(Instances)   - 
Method in class weka.core. Instances 
 Checks if two headers are equivalent.
   equals(Object)   - 
Method in class weka.associations. AssociatorEvaluation 
 Tests whether the current evaluation object is equal to another
 evaluation object
   equals(Object)   - 
Method in class weka.associations. FPGrowth.AssociationRule 
 Return true if this rule is equal to the supplied one.
   equals(Object)   - 
Method in class weka.associations. FPGrowth.BinaryItem 
  
   equals(Object)   - 
Method in class weka.associations.gsp. Element 
 Checks if two Elements are equal.
   equals(Object)   - 
Method in class weka.associations.gsp. Sequence 
 Checks if two Sequences are equal.
   equals(Object)   - 
Method in class weka.associations. ItemSet 
 Tests if two item sets are equal.
   equals(Object)   - 
Method in class weka.associations. LabeledItemSet 
 Tests if two item sets are equal.
   equals(Object)   - 
Method in class weka.associations. RuleItem 
 returns whether two RuleItems are equal
   equals(Object)   - 
Method in class weka.attributeSelection. ConsistencySubsetEval.hashKey 
 Tests if two instances are equal
   equals(Object)   - 
Method in class weka.classifiers. Evaluation 
 Tests whether the current evaluation object is equal to another
 evaluation object
   equals(Object)   - 
Method in class weka.classifiers.functions.supportVector. KernelEvaluation 
 Tests whether the current evaluation object is equal to another
 evaluation object
   equals(Object)   - 
Method in class weka.classifiers.rules. DecisionTableHashKey 
 Tests if two instances are equal
   equals(Object)   - 
Method in class weka.clusterers. ClusterEvaluation 
 Tests whether the current evaluation object is equal to another
 evaluation object
   equals(DataObject)   - 
Method in interface weka.clusterers.forOPTICSAndDBScan.DataObjects. DataObject 
 Compares two DataObjects in respect to their attribute-values
   equals(DataObject)   - 
Method in class weka.clusterers.forOPTICSAndDBScan.DataObjects. EuclidianDataObject 
 Compares two DataObjects in respect to their attribute-values
   equals(DataObject)   - 
Method in class weka.clusterers.forOPTICSAndDBScan.DataObjects. ManhattanDataObject 
 Compares two DataObjects in respect to their attribute-values
   equals(Object)   - 
Method in class weka.core. Attribute 
 Tests if given attribute is equal to this attribute.
   equals(Object)   - 
Method in class weka.core. AttributeLocator 
 Indicates whether some other object is "equal to" this one.
   equals(Object)   - 
Method in class weka.core. ClassDiscovery.StringCompare 
 Indicates whether some other object is "equal to" this Comparator.
   equals(Object)   - 
Method in class weka.core. SelectedTag 
 Returns true if this SelectedTag equals another object
   equals(Object)   - 
Method in class weka.core. SerializedObject 
  
   equals(Object)   - 
Method in class weka.core. Trie 
 Compares the specified object with this collection for equality.
   equals(Object)   - 
Method in class weka.core. Trie.TrieNode 
 Indicates whether some other object is "equal to" this one.
   equals(Object)   - 
Method in class weka.core. Version 
 whether the given version string is equal to this version
   equals(Object)   - 
Method in class weka.estimators. Estimator 
 Tests whether the current estimation object is equal to another
 estimation object
   equals(Object)   - 
Method in class weka.gui.graphvisualizer. GraphEdge 
  
   equals(Object)   - 
Method in class weka.gui.graphvisualizer. GraphNode 
 Returns true if passed in argument is an instance
  of GraphNode and is equal to this node.
   equals(Object)   - 
Method in class weka.gui. SortedTableModel.SortContainer 
 Indicates whether some other object is "equal to" this one.
   equalTo(Splitter)   - 
Method in class weka.classifiers.trees.adtree. Splitter 
 Tests whether two splitters are equivalent.
   equalTo(Splitter)   - 
Method in class weka.classifiers.trees.adtree. TwoWayNominalSplit 
 Tests whether two splitters are equivalent.
   equalTo(Splitter)   - 
Method in class weka.classifiers.trees.adtree. TwoWayNumericSplit 
 Tests whether two splitters are equivalent.
   equalTo(Test)   - 
Method in class weka.datagenerators. Test 
 Compares the test with the test that is given as parameter.
   equivalentTipText()   - 
Method in class weka.associations. Tertius 
 Returns the tip text for this property.
   equivalentTo(Rule)   - 
Method in class weka.associations.tertius. Rule 
 Test if this rule is equivalent to another rule.
   errms(StreamTokenizer, String)   - 
Static method in class weka.core.converters. ConverterUtils 
 Throws error message with line number and last token read.
   error()   - 
Method in class weka.classifiers.evaluation. NumericPrediction 
 Calculates the prediction error.
   error   - 
Static variable in interface weka.core.mathematicalexpression. sym 
  
   error   - 
Static variable in interface weka.filters.unsupervised.instance.subsetbyexpression. sym 
  
   ERROR_SHAPE   - 
Static variable in class weka.gui.visualize. Plot2D 
  
   error_sym()   - 
Method in class weka.core.mathematicalexpression. Parser 
  error  Symbol index.
   error_sym()   - 
Method in class weka.filters.unsupervised.instance.subsetbyexpression. Parser 
  error  Symbol index.
   ErrorBasedMeritEvaluator   - Interface in  weka.attributeSelection  Interface for evaluators that calculate the "merit" of attributes/subsets
 as the error of a learning scheme   errorFunction(double)   - 
Static method in class weka.core. Statistics 
 Returns the error function of the normal distribution.
   errorFunctionComplemented(double)   - 
Static method in class weka.core. Statistics 
 Returns the complementary Error function of the normal distribution.
   errorOnProbabilitiesTipText()   - 
Method in class weka.classifiers.functions. SimpleLogistic 
 Returns the tip text for this property
   errorOnProbabilitiesTipText()   - 
Method in class weka.classifiers.trees. FT 
 Returns the tip text for this property
   errorOnProbabilitiesTipText()   - 
Method in class weka.classifiers.trees. LMT 
 Returns the tip text for this property
   errorRate()   - 
Method in class weka.classifiers.evaluation. ConfusionMatrix 
 Returns the estimated error rate.
   errorRate()   - 
Method in class weka.classifiers. Evaluation 
 Returns the estimated error rate or the root mean squared error
 (if the class is numeric).
   errorValue(NeuralNode)   - 
Method in class weka.classifiers.functions.neural. LinearUnit 
 This function calculates what the error value should be.
   errorValue(boolean)   - 
Method in class weka.classifiers.functions.neural. NeuralConnection 
 Call this to get the error value of this unit.
   errorValue(NeuralNode)   - 
Method in interface weka.classifiers.functions.neural. NeuralMethod 
 This function calculates what the error value should be.
   errorValue(boolean)   - 
Method in class weka.classifiers.functions.neural. NeuralNode 
 Call this to get the error value of this unit.
   errorValue(NeuralNode)   - 
Method in class weka.classifiers.functions.neural. SigmoidUnit 
 This function calculates what the error value should be.
   estimateCPTs()   - 
Method in class weka.classifiers.bayes. BayesNet 
 estimateCPTs estimates the conditional probability tables for the Bayes
 Net using the network structure.
   estimateCPTs(BayesNet)   - 
Method in class weka.classifiers.bayes.net.estimate. BayesNetEstimator 
 estimateCPTs estimates the conditional probability tables for the Bayes
 Net using the network structure.
   estimateCPTs(BayesNet)   - 
Method in class weka.classifiers.bayes.net.estimate. BMAEstimator 
 estimateCPTs estimates the conditional probability tables for the Bayes
 Net using the network structure.
   estimateCPTs(BayesNet)   - 
Method in class weka.classifiers.bayes.net.estimate. MultiNomialBMAEstimator 
 estimateCPTs estimates the conditional probability tables for the Bayes
 Net using the network structure.
   estimateCPTs(BayesNet)   - 
Method in class weka.classifiers.bayes.net.estimate. SimpleEstimator 
 estimateCPTs estimates the conditional probability tables for the Bayes
 Net using the network structure.
   estimatePrior()   - 
Method in class weka.associations. PriorEstimation 
 Method to estimate the prior probabilities
   Estimator   - Class in  weka.estimators  Abstract class for all estimators.   Estimator()   - 
Constructor for class weka.estimators. Estimator 
  
   estimatorTipText()   - 
Method in class weka.classifiers.bayes. BayesNet 
 This will return a string describing the BayesNetEstimator.
   estimatorTipText()   - 
Method in class weka.classifiers.functions. PaceRegression 
 Returns the tip text for this property
   EstimatorUtils   - Class in  weka.estimators  Contains static utility functions for Estimators.   EstimatorUtils()   - 
Constructor for class weka.estimators. EstimatorUtils 
  
   EuclideanDistance   - Class in  weka.core  Implementing Euclidean distance (or similarity) function. 
  
 One object defines not one distance but the data model in which the distances between objects of that data model can be computed. 
  
 Attention: For efficiency reasons the use of consistency checks (like are the data models of the two instances exactly the same), is low. 
  
 For more information, see: 
  
 Wikipedia.   EuclideanDistance()   - 
Constructor for class weka.core. EuclideanDistance 
 Constructs an Euclidean Distance object, Instances must be still set.
   EuclideanDistance(Instances)   - 
Constructor for class weka.core. EuclideanDistance 
 Constructs an Euclidean Distance object and automatically initializes the
 ranges.
   EuclidianDataObject   - Class in  weka.clusterers.forOPTICSAndDBScan.DataObjects  
 EuclidianDataObject.java  
 Authors: Rainer Holzmann, Zhanna Melnikova-Albrecht, Matthias Schubert  
 Date: Aug 19, 2004  
 Time: 5:50:22 PM  
 $ Revision 1.4 $     EuclidianDataObject(Instance, String, Database)   - 
Constructor for class weka.clusterers.forOPTICSAndDBScan.DataObjects. EuclidianDataObject 
 Constructs a new DataObject.
   eval(int, int, Instance)   - 
Method in class weka.classifiers.functions.supportVector. CachedKernel 
 Implements the abstract function of Kernel using the cache.
   eval(int, int, Instance)   - 
Method in class weka.classifiers.functions.supportVector. Kernel 
 Computes the result of the kernel function for two instances.
   eval(int, int, Instance)   - 
Method in class weka.classifiers.functions.supportVector. NormalizedPolyKernel 
 Computes the result of the kernel function for two instances.
   eval(int, int, Instance)   - 
Method in class weka.classifiers.functions.supportVector. PrecomputedKernelMatrixKernel 
  
   eval(int, int, Instance)   - 
Method in class weka.classifiers.functions.supportVector. StringKernel 
 Computes the result of the kernel function for two instances.
   EVAL_ACCURACY   - 
Static variable in class weka.classifiers.rules. DecisionTable 
  
   EVAL_AUC   - 
Static variable in class weka.classifiers.rules. DecisionTable 
  
   EVAL_CROSS_VALIDATION   - 
Static variable in class weka.classifiers.meta. ThresholdSelector 
 n-fold cross-validation
   EVAL_DEFAULT   - 
Static variable in class weka.classifiers.rules. DecisionTable 
 default is accuracy for discrete class and RMSE for numeric class
   EVAL_MAE   - 
Static variable in class weka.classifiers.rules. DecisionTable 
  
   EVAL_RMSE   - 
Static variable in class weka.classifiers.rules. DecisionTable 
  
   EVAL_TRAINING_SET   - 
Static variable in class weka.classifiers.meta. ThresholdSelector 
 entire training set
   EVAL_TUNED_SPLIT   - 
Static variable in class weka.classifiers.meta. ThresholdSelector 
 single tuned fold
   evalBoolean(String)   - 
Method in class weka.core.xml. XMLDocument 
 Evaluates and returns the boolean result of the XPath expression.
   evalDouble(String)   - 
Method in class weka.core.xml. XMLDocument 
 Evaluates and returns the double result of the XPath expression.
   evalString(String)   - 
Method in class weka.core.xml. XMLDocument 
 Evaluates and returns the boolean result of the XPath expression.
   evaluate(String, String[])   - 
Static method in class weka.associations. AssociatorEvaluation 
 Evaluates an associator with the options given in an array of strings.
   evaluate(Associator, String[])   - 
Static method in class weka.associations. AssociatorEvaluation 
 Evaluates the associator with the given commandline options and returns
 the evaluation string.
   evaluate(Associator, Instances)   - 
Method in class weka.associations. AssociatorEvaluation 
 Evaluates the associator with the given commandline options and returns
 the evaluation string.
   evaluate(Kernel, String[])   - 
Static method in class weka.classifiers.functions.supportVector. KernelEvaluation 
 Evaluates the Kernel with the given commandline options and returns
 the evaluation string.
   evaluate(String, String[])   - 
Static method in class weka.classifiers.functions.supportVector. KernelEvaluation 
 Evaluates a kernel with the options given in an array of strings.
   evaluate(Kernel, Instances)   - 
Method in class weka.classifiers.functions.supportVector. KernelEvaluation 
 Evaluates the Kernel with the given commandline options and returns
 the evaluation string.
   evaluate(String, HashMap)   - 
Static method in class weka.core. MathematicalExpression 
 Parses and evaluates the given expression.
   evaluateAttribute(int)   - 
Method in interface weka.attributeSelection. AttributeEvaluator 
 evaluates an individual attribute
   evaluateAttribute(int)   - 
Method in class weka.attributeSelection. AttributeSetEvaluator 
 evaluates an individual attribute
   evaluateAttribute(int[], int[])   - 
Method in class weka.attributeSelection. AttributeSetEvaluator 
 Evaluates a set of attributes
   evaluateAttribute(int)   - 
Method in class weka.attributeSelection. ChiSquaredAttributeEval 
 evaluates an individual attribute by measuring its
 chi-squared value.
   evaluateAttribute(int)   - 
Method in class weka.attributeSelection. CostSensitiveAttributeEval 
 Evaluates an individual attribute.
   evaluateAttribute(int)   - 
Method in class weka.attributeSelection. FilteredAttributeEval 
 Evaluates an individual attribute by delegating to the base
 evaluator.
   evaluateAttribute(int)   - 
Method in class weka.attributeSelection. GainRatioAttributeEval 
 evaluates an individual attribute by measuring the gain ratio
 of the class given the attribute.
   evaluateAttribute(int)   - 
Method in class weka.attributeSelection. InfoGainAttributeEval 
 evaluates an individual attribute by measuring the amount
 of information gained about the class given the attribute.
   evaluateAttribute(int)   - 
Method in class weka.attributeSelection. LatentSemanticAnalysis 
 Evaluates the merit of a transformed attribute.
   evaluateAttribute(int)   - 
Method in class weka.attributeSelection. OneRAttributeEval 
 evaluates an individual attribute by measuring the amount
 of information gained about the class given the attribute.
   evaluateAttribute(int)   - 
Method in class weka.attributeSelection. PrincipalComponents 
 Evaluates the merit of a transformed attribute.
   evaluateAttribute(int)   - 
Method in class weka.attributeSelection. ReliefFAttributeEval 
 Evaluates an individual attribute using ReliefF's instance based approach.
   evaluateAttribute(int)   - 
Method in class weka.attributeSelection. SVMAttributeEval 
 Evaluates an attribute by returning the rank of the square of its coefficient in a
 linear support vector machine.
   evaluateAttribute(int)   - 
Method in class weka.attributeSelection. SymmetricalUncertAttributeEval 
 evaluates an individual attribute by measuring the symmetrical
 uncertainty between it and the class.
   evaluateClusterer(Instances)   - 
Method in class weka.clusterers. ClusterEvaluation 
 Evaluate the clusterer on a set of instances.
   evaluateClusterer(Instances, String)   - 
Method in class weka.clusterers. ClusterEvaluation 
 Evaluate the clusterer on a set of instances.
   evaluateClusterer(Instances, String, boolean)   - 
Method in class weka.clusterers. ClusterEvaluation 
 Evaluate the clusterer on a set of instances.
   evaluateClusterer(Clusterer, String[])   - 
Static method in class weka.clusterers. ClusterEvaluation 
 Evaluates a clusterer with the options given in an array of
 strings.
   evaluateExpression(Instance)   - 
Method in class weka.core. AttributeExpression 
 Evaluate the expression using the supplied Instance.
   evaluateExpression(double[])   - 
Method in class weka.core. AttributeExpression 
 Evaluate the expression using the supplied array of attribute values.
   evaluateModel(String, String[])   - 
Static method in class weka.classifiers. Evaluation 
 Evaluates a classifier with the options given in an array of
 strings.
   evaluateModel(Classifier, String[])   - 
Static method in class weka.classifiers. Evaluation 
 Evaluates a classifier with the options given in an array of
 strings.
   evaluateModel(Classifier, Instances, Object...)   - 
Method in class weka.classifiers. Evaluation 
 Evaluates the classifier on a given set of instances.
   evaluateModelOnce(Classifier, Instance)   - 
Method in class weka.classifiers. Evaluation 
 Evaluates the classifier on a single instance.
   evaluateModelOnce(double[], Instance)   - 
Method in class weka.classifiers. Evaluation 
 Evaluates the supplied distribution on a single instance.
   evaluateModelOnce(double, Instance)   - 
Method in class weka.classifiers. Evaluation 
 Evaluates the supplied prediction on a single instance.
   evaluateModelOnceAndRecordPrediction(Classifier, Instance)   - 
Method in class weka.classifiers. Evaluation 
 Evaluates the classifier on a single instance and records the
 prediction (if the class is nominal).
   evaluateModelOnceAndRecordPrediction(double[], Instance)   - 
Method in class weka.classifiers. Evaluation 
 Evaluates the supplied distribution on a single instance.
   evaluateSubset(BitSet)   - 
Method in class weka.attributeSelection. CfsSubsetEval 
 evaluates a subset of attributes
   evaluateSubset(BitSet)   - 
Method in class weka.attributeSelection. ClassifierSubsetEval 
 Evaluates a subset of attributes
   evaluateSubset(BitSet, Instances)   - 
Method in class weka.attributeSelection. ClassifierSubsetEval 
 Evaluates a subset of attributes with respect to a set of instances.
   evaluateSubset(BitSet, Instance, boolean)   - 
Method in class weka.attributeSelection. ClassifierSubsetEval 
 Evaluates a subset of attributes with respect to a single instance.
   evaluateSubset(BitSet)   - 
Method in class weka.attributeSelection. ConsistencySubsetEval 
 Evaluates a subset of attributes
   evaluateSubset(BitSet)   - 
Method in class weka.attributeSelection. CostSensitiveSubsetEval 
 Evaluates a subset of attributes.
   evaluateSubset(BitSet)   - 
Method in class weka.attributeSelection. FilteredSubsetEval 
 evaluates a subset of attributes
   evaluateSubset(BitSet, Instances)   - 
Method in class weka.attributeSelection. HoldOutSubsetEvaluator 
 Evaluates a subset of attributes with respect to a set of instances.
   evaluateSubset(BitSet, Instance, boolean)   - 
Method in class weka.attributeSelection. HoldOutSubsetEvaluator 
 Evaluates a subset of attributes with respect to a single instance.
   evaluateSubset(BitSet)   - 
Method in interface weka.attributeSelection. SubsetEvaluator 
 evaluates a subset of attributes
   evaluateSubset(BitSet)   - 
Method in class weka.attributeSelection. WrapperSubsetEval 
 Evaluates a subset of attributes
   Evaluation   - Class in  weka.classifiers  Class for evaluating machine learning models.   Evaluation(Instances)   - 
Constructor for class weka.classifiers. Evaluation 
 Initializes all the counters for the evaluation.
   Evaluation(Instances, CostMatrix)   - 
Constructor for class weka.classifiers. Evaluation 
 Initializes all the counters for the evaluation and also takes a
 cost matrix as parameter.
   EVALUATION_ACC   - 
Static variable in class weka.classifiers.meta. GridSearch 
 evaluation via: Accuracy
   EVALUATION_CC   - 
Static variable in class weka.classifiers.meta. GridSearch 
 evaluation via: Correlation coefficient
   EVALUATION_COMBINED   - 
Static variable in class weka.classifiers.meta. GridSearch 
 evaluation via: Combined = (1-CC) + RRSE + RAE
   EVALUATION_KAPPA   - 
Static variable in class weka.classifiers.meta. GridSearch 
 evaluation via: kappa statistic
   EVALUATION_MAE   - 
Static variable in class weka.classifiers.meta. GridSearch 
 evaluation via: Mean absolute error
   EVALUATION_RAE   - 
Static variable in class weka.classifiers.meta. GridSearch 
 evaluation via: Relative absolute error
   EVALUATION_RMSE   - 
Static variable in class weka.classifiers.meta. GridSearch 
 evaluation via: Root mean squared error
   EVALUATION_RRSE   - 
Static variable in class weka.classifiers.meta. GridSearch 
 evaluation via: Root relative squared error
   evaluationMeasureTipText()   - 
Method in class weka.classifiers.rules. DecisionTable 
 Returns the tip text for this property
   evaluationModeTipText()   - 
Method in class weka.classifiers.meta. ThresholdSelector 
  
   evaluationTipText()   - 
Method in class weka.classifiers.meta. GridSearch 
 Returns the tip text for this property
   EvaluationUtils   - Class in  weka.classifiers.evaluation  Contains utility functions for generating lists of predictions in 
 various manners.   EvaluationUtils()   - 
Constructor for class weka.classifiers.evaluation. EvaluationUtils 
  
   evaluatorTipText()   - 
Method in class weka.attributeSelection. CostSensitiveASEvaluation 
 Returns the tip text for this property
   evaluatorTipText()   - 
Method in class weka.classifiers.meta. AttributeSelectedClassifier 
 Returns the tip text for this property
   evaluatorTipText()   - 
Method in class weka.filters.supervised.attribute. AttributeSelection 
 Returns the tip text for this property
   evalUsingTrainingDataTipText()   - 
Method in class weka.attributeSelection. OneRAttributeEval 
 Returns a string for this option suitable for display in the gui
 as a tip text
   EventConstraints   - Interface in  weka.gui.beans  Interface for objects that want to be able to specify at any given
 time whether their current configuration allows a particular event
 to be generated.   eventGeneratable(EventSetDescriptor)   - 
Method in class weka.gui.beans. Associator 
 Returns true, if at the current time, the event described by the
 supplied event descriptor could be generated.
   eventGeneratable(String)   - 
Method in class weka.gui.beans. Associator 
 Returns true, if at the current time, the named event could
 be generated.
   eventGeneratable(String)   - 
Method in class weka.gui.beans. ClassAssigner 
 Returns true, if at the current time, the named event could
 be generated.
   eventGeneratable(EventSetDescriptor)   - 
Method in class weka.gui.beans. Classifier 
 Returns true, if at the current time, the event described by the
 supplied event descriptor could be generated.
   eventGeneratable(String)   - 
Method in class weka.gui.beans. Classifier 
 Returns true, if at the current time, the named event could
 be generated.
   eventGeneratable(String)   - 
Method in class weka.gui.beans. ClassifierPerformanceEvaluator 
 Returns true, if at the current time, the named event could
 be generated.
   eventGeneratable(String)   - 
Method in class weka.gui.beans. ClassValuePicker 
 Returns true, if at the current time, the named event could
 be generated.
   eventGeneratable(EventSetDescriptor)   - 
Method in class weka.gui.beans. Clusterer 
 Returns true, if at the current time, the event described by the
 supplied event descriptor could be generated.
   eventGeneratable(String)   - 
Method in class weka.gui.beans. Clusterer 
 Returns true, if at the current time, the named event could
 be generated.
   eventGeneratable(String)   - 
Method in class weka.gui.beans. ClustererPerformanceEvaluator 
 Returns true, if at the current time, the named event could
 be generated.
   eventGeneratable(String)   - 
Method in class weka.gui.beans. CrossValidationFoldMaker 
 Returns true, if at the current time, the named event could
 be generated.
   eventGeneratable(String)   - 
Method in interface weka.gui.beans. EventConstraints 
 Returns true if, at the current time, the named event could be
 generated.
   eventGeneratable(String)   - 
Method in class weka.gui.beans. Filter 
 Returns true, if at the current time, the named event could
 be generated.
   eventGeneratable(String)   - 
Method in class weka.gui.beans. IncrementalClassifierEvaluator 
 Returns true, if at the current time, the named event could
 be generated.
   eventGeneratable(String)   - 
Method in class weka.gui.beans. InstanceStreamToBatchMaker 
 Returns true if, at the current time, the named event could be
 generated.
   eventGeneratable(String)   - 
Method in class weka.gui.beans. Loader 
 Returns true if the named event can be generated at this time
   eventGeneratable(EventSetDescriptor)   - 
Method in class weka.gui.beans. MetaBean 
 Returns true, if at the current time, the event described by the
 supplied event descriptor could be generated.
   eventGeneratable(String)   - 
Method in class weka.gui.beans. MetaBean 
 Returns true, if at the current time, the named event could
 be generated.
   eventGeneratable(String)   - 
Method in class weka.gui.beans. PredictionAppender 
 Returns true, if at the current time, the named event could
 be generated.
   eventGeneratable(String)   - 
Method in class weka.gui.beans. TestSetMaker 
 Returns true, if at the current time, the named event could
 be generated.
   eventGeneratable(String)   - 
Method in class weka.gui.beans. TextViewer 
 Returns true, if at the current time, the named event could
 be generated.
   eventGeneratable(String)   - 
Method in class weka.gui.beans. TrainingSetMaker 
 Returns true, if at the current time, the named event could
 be generated.
   eventGeneratable(String)   - 
Method in class weka.gui.beans. TrainTestSplitMaker 
 Returns true, if at the current time, the named event could
 be generated.
   exclusiveTipText()   - 
Method in class weka.classifiers.rules. ConjunctiveRule 
 Returns the tip text for this property
   execute()   - 
Method in class weka.classifiers. CheckSource 
 performs the comparison test
   execute(String)   - 
Method in class weka.experiment. DatabaseUtils 
 Executes a SQL query.
   execute()   - 
Method in class weka.experiment. RemoteExperimentSubTask 
 Run the experiment
   execute()   - 
Method in interface weka.experiment. Task 
 Execute this task.
   execute()   - 
Method in class weka.filters. CheckSource 
 performs the comparison test
   execute()   - 
Method in class weka.gui.boundaryvisualizer. RemoteBoundaryVisualizerSubTask 
 Perform the sub task
   execute()   - 
Method in class weka.gui.explorer. DataGeneratorPanel 
 generates the instances, returns TRUE if successful
   execute()   - 
Method in class weka.gui. GenericPropertiesCreator 
 generates the props-file for the GenericObjectEditor and stores it
   execute(boolean)   - 
Method in class weka.gui. GenericPropertiesCreator 
 generates the props-file for the GenericObjectEditor and stores it only
 if the the param  store  is TRUE.
   execute()   - 
Method in class weka.gui.sql. QueryPanel 
 executes the current query.
   executeTask(Task)   - 
Method in interface weka.experiment. Compute 
 Execute a task
   executeTask(Task)   - 
Method in class weka.experiment. RemoteEngine 
 Takes a task object and queues it for execution
   ExhaustiveSearch   - Class in  weka.attributeSelection  ExhaustiveSearch :  
  
 Performs an exhaustive search through the space of attribute subsets starting from the empty set of attrubutes.   ExhaustiveSearch()   - 
Constructor for class weka.attributeSelection. ExhaustiveSearch 
 Constructor
   exists(TechnicalInformation.Field)   - 
Method in class weka.core. TechnicalInformation 
 returns TRUE if the field is stored and has a value different from the
 empty string.
   EXP   - 
Static variable in interface weka.core.mathematicalexpression. sym 
  
   EXP   - 
Static variable in interface weka.filters.unsupervised.instance.subsetbyexpression. sym 
  
   EXP_INDEX_TABLE   - 
Static variable in class weka.experiment. DatabaseUtils 
 The name of the table containing the index to experiments.
   EXP_RESULT_COL   - 
Static variable in class weka.experiment. DatabaseUtils 
 The name of the column containing the results table name.
   EXP_RESULT_PREFIX   - 
Static variable in class weka.experiment. DatabaseUtils 
 The prefix for result table names.
   EXP_SETUP_COL   - 
Static variable in class weka.experiment. DatabaseUtils 
 The name of the column containing the experiment setup (parameters).
   EXP_TYPE_COL   - 
Static variable in class weka.experiment. DatabaseUtils 
 The name of the column containing the experiment type (ResultProducer).
   expectation(double, int, double[], Hashtable)   - 
Static method in class weka.associations. RuleGeneration 
 calculates the expected predctive accuracy of a rule
   expectedCosts(double[])   - 
Method in class weka.classifiers. CostMatrix 
 Calculates the expected misclassification cost for each possible class value,
 given class probability estimates.
   expectedCosts(double[], Instance)   - 
Method in class weka.classifiers. CostMatrix 
 Calculates the expected misclassification cost for each possible class value,
 given class probability estimates.
   expectedResultsPerAverageTipText()   - 
Method in class weka.experiment. AveragingResultProducer 
 Returns the tip text for this property
   Experiment   - Class in  weka.experiment  Holds all the necessary configuration information for a standard
 type experiment.   Experiment()   - 
Constructor for class weka.experiment. Experiment 
  
   Experimenter   - Class in  weka.gui.experiment  The main class for the experiment environment.   Experimenter(boolean)   - 
Constructor for class weka.gui.experiment. Experimenter 
 Creates the experiment environment gui with no initial experiment
   ExperimenterDefaults   - Class in  weka.gui.experiment  This class offers get methods for the default Experimenter settings in 
 the props file  weka/gui/experiment/Experimenter.props .   ExperimenterDefaults()   - 
Constructor for class weka.gui.experiment. ExperimenterDefaults 
  
   experimentIndexExists()   - 
Method in class weka.experiment. DatabaseUtils 
 Returns true if the experiment index exists.
   EXPLICIT   - 
Static variable in class weka.associations. Tertius 
 Way of handling missing values: min counterinstances
   Explorer   - Class in  weka.gui.explorer  The main class for the Weka explorer.   Explorer()   - 
Constructor for class weka.gui.explorer. Explorer 
 Creates the experiment environment gui with no initial experiment
   Explorer.CapabilitiesFilterChangeEvent   - Class in  weka.gui.explorer  This event can be fired in case the capabilities filter got changed   Explorer.CapabilitiesFilterChangeEvent(Object, Capabilities)   - 
Constructor for class weka.gui.explorer. Explorer.CapabilitiesFilterChangeEvent 
 Constructs a GOECapabilitiesFilterChangeEvent object.
   Explorer.CapabilitiesFilterChangeListener   - Interface in  weka.gui.explorer  Interface for classes that listen for filter changes.   Explorer.ExplorerPanel   - Interface in  weka.gui.explorer  A common interface for panels to be displayed in the Explorer   Explorer.LogHandler   - Interface in  weka.gui.explorer  A common interface for panels in the explorer that can handle logs   ExplorerDefaults   - Class in  weka.gui.explorer  This class offers get methods for the default Explorer settings in 
 the props file  weka/gui/explorer/Explorer.props .   ExplorerDefaults()   - 
Constructor for class weka.gui.explorer. ExplorerDefaults 
  
   ExponentialFormat   - Class in  weka.core.matrix      ExponentialFormat()   - 
Constructor for class weka.core.matrix. ExponentialFormat 
  
   ExponentialFormat(int)   - 
Constructor for class weka.core.matrix. ExponentialFormat 
  
   ExponentialFormat(int, boolean)   - 
Constructor for class weka.core.matrix. ExponentialFormat 
  
   ExponentialFormat(int, int, boolean, boolean)   - 
Constructor for class weka.core.matrix. ExponentialFormat 
  
   exponentTipText()   - 
Method in class weka.classifiers.functions.supportVector. PolyKernel 
 Returns the tip text for this property
   exponentTipText()   - 
Method in class weka.classifiers.functions. VotedPerceptron 
 Returns the tip text for this property
   Expression   - Class in  weka.core.pmml      Expression(FieldMetaInfo.Optype, ArrayList<Attribute>)   - 
Constructor for class weka.core.pmml. Expression 
  
   Expression   - Class in  weka.datagenerators.classifiers.regression  A data generator for generating y according to a given expression out of randomly generated x. 
 E.g., the mexican hat can be generated like this: 
    sin(abs(a1)) / abs(a1) 
 In addition to this function, the amplitude can be changed and gaussian noise can be added.   Expression()   - 
Constructor for class weka.datagenerators.classifiers.regression. Expression 
 initializes the generator
   expressionTipText()   - 
Method in class weka.datagenerators.classifiers.regression. Expression 
 Returns the tip text for this property
   expressionTipText()   - 
Method in class weka.filters.unsupervised.attribute. AddExpression 
 Returns the tip text for this property
   expressionTipText()   - 
Method in class weka.filters.unsupervised.attribute. MathExpression 
 Returns the tip text for this property
   expressionTipText()   - 
Method in class weka.filters.unsupervised.instance. SubsetByExpression 
 Returns the tip text for this property.
   ExtensionFileFilter   - Class in  weka.gui  Provides a file filter for FileChoosers that accepts or rejects files
 based on their extension.   ExtensionFileFilter(String, String)   - 
Constructor for class weka.gui. ExtensionFileFilter 
 Creates the ExtensionFileFilter
   ExtensionFileFilter(String[], String)   - 
Constructor for class weka.gui. ExtensionFileFilter 
 Creates an ExtensionFileFilter that accepts files that have any of
 the extensions contained in the supplied array.
   extraArcs(BayesNet)   - 
Method in class weka.classifiers.bayes.net. BIFReader 
 Count nr of exta arcs  from other network compared to current network
 Note that an arc is not 'extra' if it is reversed.
   extract(RevisionHandler)   - 
Static method in class weka.core. RevisionUtils 
 Extracts the revision string returned by the RevisionHandler.
   extract(String)   - 
Static method in class weka.core. RevisionUtils 
 Extracts the revision string.
   extractFilterAttributes(String)   - 
Method in class weka.associations. GeneralizedSequentialPatterns 
 Parses a given String containing attribute numbers which are used for 
 result filtering.
   extremeValuesAsOutliersTipText()   - 
Method in class weka.filters.unsupervised.attribute. InterquartileRange 
 Returns the tip text for this property
   extremeValuesFactorTipText()   - 
Method in class weka.filters.unsupervised.attribute. InterquartileRange 
 Returns the tip text for this property
 
 
    
 F  
 
   f(double)   - 
Method in class weka.classifiers.functions.pace. ChisqMixture 
 Computes the value of f(x) given the mixture.
   f(DoubleVector)   - 
Method in class weka.classifiers.functions.pace. ChisqMixture 
 Computes the value of f(x) given the mixture, where x is a vector.
   f(double)   - 
Method in class weka.classifiers.functions.pace. NormalMixture 
 Computes the value of f(x) given the mixture.
   f(DoubleVector)   - 
Method in class weka.classifiers.functions.pace. NormalMixture 
 Computes the value of f(x) given the mixture, where x is a vector.
   FAILED   - 
Static variable in class weka.experiment. TaskStatusInfo 
  
   failed()   - 
Method in class weka.gui.sql.event. ConnectionEvent 
 whether an exception happened and is stored
   failed()   - 
Method in class weka.gui.sql.event. QueryExecuteEvent 
 is TRUE in case the exception is not NULL, i.e.
   FALLOUT_NAME   - 
Static variable in class weka.classifiers.evaluation. ThresholdCurve 
 attribute name: Fallout
   FALSE   - 
Static variable in interface weka.core.mathematicalexpression. sym 
  
   FALSE   - 
Static variable in interface weka.filters.unsupervised.instance.subsetbyexpression. sym 
  
   FALSE_NEG_NAME   - 
Static variable in class weka.classifiers.evaluation. ThresholdCurve 
 attribute name: False Negatives
   FALSE_POS_NAME   - 
Static variable in class weka.classifiers.evaluation. ThresholdCurve 
 attribute name: False Positives
   falseNegativeRate(int)   - 
Method in class weka.classifiers. Evaluation 
 Calculate the false negative rate with respect to a particular class.
   falsePositiveRate(int)   - 
Method in class weka.classifiers. Evaluation 
 Calculate the false positive rate with respect to a particular class.
   FarthestFirst   - Class in  weka.clusterers  Cluster data using the FarthestFirst algorithm. 
  
 For more information see: 
  
 Hochbaum, Shmoys (1985).   FarthestFirst()   - 
Constructor for class weka.clusterers. FarthestFirst 
  
   fastRegressionTipText()   - 
Method in class weka.classifiers.trees. LMT 
 Returns the tip text for this property
   FastVector   - Class in  weka.core  Implements a fast vector class without synchronized
 methods.   FastVector()   - 
Constructor for class weka.core. FastVector 
 Constructs an empty vector with initial
 capacity zero.
   FastVector(int)   - 
Constructor for class weka.core. FastVector 
 Constructs a vector with the given capacity.
   FastVector.FastVectorEnumeration   - Class in  weka.core  Class for enumerating the vector's elements.   FastVector.FastVectorEnumeration(FastVector)   - 
Constructor for class weka.core. FastVector.FastVectorEnumeration 
 Constructs an enumeration.
   FastVector.FastVectorEnumeration(FastVector, int)   - 
Constructor for class weka.core. FastVector.FastVectorEnumeration 
 Constructs an enumeration with a special element.
   FieldMetaInfo   - Class in  weka.core.pmml  Abstract superclass for various types of field meta
 data.   FieldMetaInfo(Element)   - 
Constructor for class weka.core.pmml. FieldMetaInfo 
 Construct a new FieldMetaInfo.
   FieldMetaInfo.Interval   - Class in  weka.core.pmml  Inner class for an Interval.   FieldMetaInfo.Interval.Closure   - Enum in  weka.core.pmml  Enumerated type for the closure.   FieldMetaInfo.Optype   - Enum in  weka.core.pmml  Enumerated type for the Optype   FieldMetaInfo.Value   - Class in  weka.core.pmml  Inner class for Values   FieldMetaInfo.Value.Property   - Enum in  weka.core.pmml  Enumerated type for the property.   FieldRef   - Class in  weka.core.pmml  Class encapsulating a FieldRef Expression.   FieldRef(Element, FieldMetaInfo.Optype, ArrayList<Attribute>)   - 
Constructor for class weka.core.pmml. FieldRef 
  
   fields()   - 
Method in class weka.core. TechnicalInformation 
 returns an enumeration over all the stored fields
   FILE_EXTENSION   - 
Static variable in class weka.classifiers. CostMatrix 
 The deafult file extension for cost matrix files
   FILE_EXTENSION   - 
Static variable in class weka.core.converters. ArffLoader 
 the file extension
   FILE_EXTENSION   - 
Static variable in class weka.core.converters. C45Loader 
 the file extension
   FILE_EXTENSION   - 
Static variable in class weka.core.converters. CSVLoader 
 the file extension.
   FILE_EXTENSION   - 
Static variable in class weka.core.converters. LibSVMLoader 
 the file extension.
   FILE_EXTENSION   - 
Static variable in class weka.core.converters. LibSVMSaver 
 the file extension
   FILE_EXTENSION   - 
Static variable in class weka.core.converters. SerializedInstancesLoader 
 the file extension
   FILE_EXTENSION   - 
Static variable in class weka.core.converters. SVMLightLoader 
 the file extension.
   FILE_EXTENSION   - 
Static variable in class weka.core.converters. SVMLightSaver 
 the file extension.
   FILE_EXTENSION   - 
Static variable in class weka.core.converters. XRFFLoader 
 the file extension
   FILE_EXTENSION   - 
Static variable in class weka.core. Instances 
 The filename extension that should be used for arff files
   FILE_EXTENSION   - 
Static variable in class weka.core.xml. KOML 
 the extension for KOML files (including '.')
   FILE_EXTENSION   - 
Static variable in class weka.core.xml. XMLInstances 
 The filename extension that should be used for xrff files
   FILE_EXTENSION   - 
Static variable in class weka.core.xml. XStream 
 the extension for XStream files (including '.')
   FILE_EXTENSION   - 
Static variable in class weka.experiment. Experiment 
 The filename extension that should be used for experiment files
   FILE_EXTENSION   - 
Static variable in class weka.gui.beans. Classifier 
 the extension for serialized models (binary Java serialization)
   FILE_EXTENSION   - 
Static variable in class weka.gui.beans. KnowledgeFlowApp 
 the extension for the serialized setups (Java serialization)
   FILE_EXTENSION   - 
Static variable in class weka.gui.beans. SerializedModelSaver 
 the extension for serialized models (binary Java serialization)
   FILE_EXTENSION_COMPRESSED   - 
Static variable in class weka.core.converters. AbstractFileLoader 
 the extension for compressed files
   FILE_EXTENSION_COMPRESSED   - 
Static variable in class weka.core.converters. ArffLoader 
  
   FILE_EXTENSION_COMPRESSED   - 
Static variable in class weka.core.converters. XRFFLoader 
 the extension for compressed files
   FILE_EXTENSION_XML   - 
Static variable in class weka.gui.beans. KnowledgeFlowApp 
 the extension for the serialized setups (Java serialization)
   FileEditor   - Class in  weka.gui  A PropertyEditor for File objects that lets the user select a file.   FileEditor()   - 
Constructor for class weka.gui. FileEditor 
  
   FileLogger   - Class in  weka.core.logging  A simple file logger, that just logs to a single file.   FileLogger()   - 
Constructor for class weka.core.logging. FileLogger 
  
   filePrefix()   - 
Method in class weka.core.converters. AbstractFileSaver 
 Gets the file name prefix
   filePrefix()   - 
Method in class weka.core.converters. AbstractSaver 
 Default implementation throws an IOException.
   filePrefix()   - 
Method in interface weka.core.converters. Saver 
 Gets the file prefix
 This method is used in the KnowledgeFlow GUI.
   FileSourcedConverter   - Interface in  weka.core.converters  Interface to a loader/saver that loads/saves from a file source.   fill(Shape)   - 
Method in class weka.gui.visualize. PostscriptGraphics 
  
   fill3DRect(int, int, int, int, boolean)   - 
Method in class weka.gui.visualize. PostscriptGraphics 
 Draw a filled rectangle with 3D effect in current pen color.
   fillArc(int, int, int, int, int, int)   - 
Method in class weka.gui.visualize. PostscriptGraphics 
 Not implemented
   fillFrame(Component)   - 
Method in interface weka.gui. MainMenuExtension 
 Fills the frame with life, like adding components, window listeners,
 setting size, location, etc.
   fillIn(int[], boolean[][])   - 
Method in class weka.classifiers.bayes.net. MarginCalculator 
 Apply Tarjan and Yannakakis (1984) fill in algorithm for graph
 triangulation.
   fillOval(int, int, int, int)   - 
Method in class weka.gui.visualize. PostscriptGraphics 
 Draw a filled Oval in current pen color.
   fillPolygon(int[], int[], int)   - 
Method in class weka.gui.visualize. PostscriptGraphics 
 Not implemented
   fillPolygon(Polygon)   - 
Method in class weka.gui.visualize. PostscriptGraphics 
 Not implemented
   fillRect(int, int, int, int)   - 
Method in class weka.gui.visualize. PostscriptGraphics 
 Draw a filled rectangle in current pen color.
   fillRoundRect(int, int, int, int, int, int)   - 
Method in class weka.gui.visualize. PostscriptGraphics 
 Not implemented
   fillWithMissingTipText()   - 
Method in class weka.filters.unsupervised.attribute. AbstractTimeSeries 
 Returns the tip text for this property
   Filter   - Class in  weka.filters  An abstract class for instance filters: objects that take instances
 as input, carry out some transformation on the instance and then
 output the instance.   Filter()   - 
Constructor for class weka.filters. Filter 
  
   filter(String, Instances)   - 
Static method in class weka.filters.unsupervised.instance.subsetbyexpression. Parser 
 Filters the input dataset against the provided expression.
   Filter   - Class in  weka.gui.beans  A wrapper bean for Weka filters   Filter()   - 
Constructor for class weka.gui.beans. Filter 
  
   FILTER_NONE   - 
Static variable in class weka.classifiers.functions. GaussianProcesses 
 no filter
   FILTER_NONE   - 
Static variable in class weka.classifiers.functions. SMO 
 filter: No normalization/standardization
   FILTER_NONE   - 
Static variable in class weka.classifiers.functions. SMOreg 
 The filter to apply to the training data: None
   FILTER_NONE   - 
Static variable in class weka.classifiers.mi. MDD 
 No normalization/standardization
   FILTER_NONE   - 
Static variable in class weka.classifiers.mi. MIDD 
 No normalization/standardization
   FILTER_NONE   - 
Static variable in class weka.classifiers.mi. MIEMDD 
 No normalization/standardization
   FILTER_NONE   - 
Static variable in class weka.classifiers.mi. MIOptimalBall 
 No normalization/standardization
   FILTER_NONE   - 
Static variable in class weka.classifiers.mi. MISMO 
 No normalization/standardization
   FILTER_NONE   - 
Static variable in class weka.classifiers.mi. MISVM 
 No normalization/standardization
   FILTER_NONE   - 
Static variable in class weka.filters.unsupervised.attribute. StringToWordVector 
 normalization: No normalization.
   FILTER_NORMALIZE   - 
Static variable in class weka.classifiers.functions. GaussianProcesses 
 normalizes the data
   FILTER_NORMALIZE   - 
Static variable in class weka.classifiers.functions. SMO 
 filter: Normalize training data
   FILTER_NORMALIZE   - 
Static variable in class weka.classifiers.functions. SMOreg 
 The filter to apply to the training data: Normalzie
   FILTER_NORMALIZE   - 
Static variable in class weka.classifiers.mi. MDD 
 Normalize training data
   FILTER_NORMALIZE   - 
Static variable in class weka.classifiers.mi. MIDD 
 Normalize training data
   FILTER_NORMALIZE   - 
Static variable in class weka.classifiers.mi. MIEMDD 
 Normalize training data
   FILTER_NORMALIZE   - 
Static variable in class weka.classifiers.mi. MIOptimalBall 
 Normalize training data
   FILTER_NORMALIZE   - 
Static variable in class weka.classifiers.mi. MISMO 
 Normalize training data
   FILTER_NORMALIZE   - 
Static variable in class weka.classifiers.mi. MISVM 
 Normalize training data
   FILTER_NORMALIZE_ALL   - 
Static variable in class weka.filters.unsupervised.attribute. StringToWordVector 
 normalization: Normalize all data.
   FILTER_NORMALIZE_TEST_ONLY   - 
Static variable in class weka.filters.unsupervised.attribute. StringToWordVector 
 normalization: Normalize test data only.
   FILTER_STANDARDIZE   - 
Static variable in class weka.classifiers.functions. GaussianProcesses 
 standardizes the data
   FILTER_STANDARDIZE   - 
Static variable in class weka.classifiers.functions. SMO 
 filter: Standardize training data
   FILTER_STANDARDIZE   - 
Static variable in class weka.classifiers.functions. SMOreg 
 The filter to apply to the training data: Standardize
   FILTER_STANDARDIZE   - 
Static variable in class weka.classifiers.mi. MDD 
 Standardize training data
   FILTER_STANDARDIZE   - 
Static variable in class weka.classifiers.mi. MIDD 
 Standardize training data
   FILTER_STANDARDIZE   - 
Static variable in class weka.classifiers.mi. MIEMDD 
 Standardize training data
   FILTER_STANDARDIZE   - 
Static variable in class weka.classifiers.mi. MIOptimalBall 
 Standardize training data
   FILTER_STANDARDIZE   - 
Static variable in class weka.classifiers.mi. MISMO 
 Standardize training data
   FILTER_STANDARDIZE   - 
Static variable in class weka.classifiers.mi. MISVM 
 Standardize training data
   filterAfterFirstBatchTipText()   - 
Method in class weka.filters.unsupervised.instance. SubsetByExpression 
 Returns the tip text for this property.
   filterAttributesTipText()   - 
Method in class weka.associations. GeneralizedSequentialPatterns 
 Returns the filterAttributes option tip text for the Weka GUI.
   FilterBeanInfo   - Class in  weka.gui.beans  Bean info class for the Filter bean   FilterBeanInfo()   - 
Constructor for class weka.gui.beans. FilterBeanInfo 
  
   FilterCustomizer   - Class in  weka.gui.beans  GUI customizer for the filter bean   FilterCustomizer()   - 
Constructor for class weka.gui.beans. FilterCustomizer 
  
   FilteredAssociator   - Class in  weka.associations  Class for running an arbitrary associator on data that has been passed through an arbitrary filter.   FilteredAssociator()   - 
Constructor for class weka.associations. FilteredAssociator 
 Default constructor.
   FilteredAttributeEval   - Class in  weka.attributeSelection  Class for running an arbitrary attribute evaluator on data that has been passed through an 
 arbitrary filter (note: filters that alter the order or number of attributes are not allowed).   FilteredAttributeEval()   - 
Constructor for class weka.attributeSelection. FilteredAttributeEval 
  
   FilteredClassifier   - Class in  weka.classifiers.meta  Class for running an arbitrary classifier on data that has been passed through an arbitrary filter.   FilteredClassifier()   - 
Constructor for class weka.classifiers.meta. FilteredClassifier 
 Default constructor.
   FilteredClusterer   - Class in  weka.clusterers  Class for running an arbitrary clusterer on data that has been passed through an arbitrary filter.   FilteredClusterer()   - 
Constructor for class weka.clusterers. FilteredClusterer 
 Default constructor.
   FilteredSubsetEval   - Class in  weka.attributeSelection  Class for running an arbitrary subset evaluator on data that has been passed through an arbitrary 
 filter (note: filters that alter the order or number of attributes are not allowed).   FilteredSubsetEval()   - 
Constructor for class weka.attributeSelection. FilteredSubsetEval 
  
   filterFile(Filter, String[])   - 
Static method in class weka.filters. Filter 
 Method for testing filters.
   filtersTipText()   - 
Method in class weka.filters. MultiFilter 
 Returns the tip text for this property
   filtersTipText()   - 
Method in class weka.filters.unsupervised.attribute. PartitionedMultiFilter 
 Returns the tip text for this property.
   filterSubset(List<ScatterSearchV1.Subset>, int)   - 
Method in class weka.attributeSelection. ScatterSearchV1 
 Filter a given Lis of Subsets removing the equals subsets
   filterTipText()   - 
Method in class weka.associations. FilteredAssociator 
 Returns the tip text for this property
   filterTipText()   - 
Method in class weka.attributeSelection. FilteredAttributeEval 
 Returns the tip text for this property
   filterTipText()   - 
Method in class weka.attributeSelection. FilteredSubsetEval 
 Returns the tip text for this property
   filterTipText()   - 
Method in class weka.classifiers.functions. PLSClassifier 
 Returns the tip text for this property
   filterTipText()   - 
Method in class weka.classifiers.meta. FilteredClassifier 
 Returns the tip text for this property
   filterTipText()   - 
Method in class weka.classifiers.meta. GridSearch 
 Returns the tip text for this property
   filterTipText()   - 
Method in class weka.clusterers. FilteredClusterer 
 Returns the tip text for this property.
   filterTipText()   - 
Method in class weka.filters.unsupervised.attribute. Wavelet 
 Returns the tip text for this property
   filterTypeTipText()   - 
Method in class weka.attributeSelection. SVMAttributeEval 
 Returns a tip text for this property suitable for display in the
 GUI
   filterTypeTipText()   - 
Method in class weka.classifiers.functions. GaussianProcesses 
 Returns the tip text for this property
   filterTypeTipText()   - 
Method in class weka.classifiers.functions. SMO 
 Returns the tip text for this property
   filterTypeTipText()   - 
Method in class weka.classifiers.functions. SMOreg 
 Returns the tip text for this property
   filterTypeTipText()   - 
Method in class weka.classifiers.mi. MDD 
 Returns the tip text for this property
   filterTypeTipText()   - 
Method in class weka.classifiers.mi. MIDD 
 Returns the tip text for this property
   filterTypeTipText()   - 
Method in class weka.classifiers.mi. MIEMDD 
 Returns the tip text for this property
   filterTypeTipText()   - 
Method in class weka.classifiers.mi. MIOptimalBall 
 Returns the tip text for this property
   filterTypeTipText()   - 
Method in class weka.classifiers.mi. MISMO 
 Returns the tip text for this property
   filterTypeTipText()   - 
Method in class weka.classifiers.mi. MISVM 
 Returns the tip text for this property
   finalize()   - 
Method in class weka.gui.sql. ResultSetTable 
 frees up the memory
   finalize()   - 
Method in class weka.gui.sql. ResultSetTableModel 
 frees up the memory.
   finalize()   - 
Method in class weka.gui.visualize. PostscriptGraphics 
 Not implemented
   find(String, String[])   - 
Static method in class weka.core. ClassDiscovery 
 Checks the given packages for classes that inherited from the given class,
 in case it's a class, or implement this class, in case it's an interface.
   find(String, String)   - 
Static method in class weka.core. ClassDiscovery 
 Checks the given package for classes that inherited from the given class,
 in case it's a class, or implement this class, in case it's an interface.
   find(Class, String[])   - 
Static method in class weka.core. ClassDiscovery 
 Checks the given packages for classes that inherited from the given class,
 in case it's a class, or implement this class, in case it's an interface.
   find(Class, String)   - 
Static method in class weka.core. ClassDiscovery 
 Checks the given package for classes that inherited from the given class,
 in case it's a class, or implement this class, in case it's an interface.
   find()   - 
Method in class weka.core. FindWithCapabilities 
 returns a list with all the classnames that fit the criteria.
   find(Object, PropertyPath.Path)   - 
Static method in class weka.core. PropertyPath 
 returns the property and object associated with the given path, null if 
 a problem occurred.
   find(String)   - 
Method in class weka.core. Trie.TrieNode 
 returns the node with the given suffix
   findAllRulesForSupportLevelTipText()   - 
Method in class weka.associations. FPGrowth 
 Tip text for this property suitable for displaying
 in the GUI.
   findArgmin(double[], double[][])   - 
Method in class weka.core. Optimization 
 Main algorithm.
   findBestLeaf(double[], RuleNode[])   - 
Method in class weka.classifiers.trees.m5. RuleNode 
 Find the leaf with greatest coverage
   findCentralTendencies(double[])   - 
Method in class weka.classifiers. BVDecomposeSegCVSub 
 Finds the central tendency, given the classifications for an instance.
   findInstance(Point)   - 
Static method in class weka.gui.beans. BeanInstance 
 Looks for a bean (if any) whose bounds contain the supplied point
   findInstances(Rectangle)   - 
Static method in class weka.gui.beans. BeanInstance 
 Looks for all beans (if any) located within
 the supplied bounding box.
   findIntervall(double)   - 
Method in class weka.associations. PriorEstimation 
 searches the mid point of the interval a given confidence value falls into
   findMinDistance(Instances, int)   - 
Static method in class weka.estimators. EstimatorUtils 
 Find the minimum distance between values
   findNodes(String)   - 
Method in class weka.core.xml. XMLDocument 
 Returns the nodes that the given xpath expression will find in the 
 document.
   findNumBinsTipText()   - 
Method in class weka.filters.unsupervised.attribute. Discretize 
 Returns the tip text for this property
   findNumBinsTipText()   - 
Method in class weka.filters.unsupervised.attribute. PKIDiscretize 
 Returns the tip text for this property
   findPackages()   - 
Static method in class weka.core. ClassDiscovery 
 Lists all packages it can find in the classpath.
   findRadius(Instances)   - 
Method in class weka.classifiers.mi. MIOptimalBall 
 Find the maximum radius for the optimal ball.
   findReadMethod(Object, String)   - 
Static method in class weka.core.xml. XMLSerializationMethodHandler 
 returns the method with the given name that has the same signature as
  readFromXML()  of the  XMLSerialiation  class.
   findWeights(int, double[][])   - 
Method in class weka.classifiers.mi. MINND 
 Use gradient descent to distort the MU parameter for
 the exemplar.
   FindWithCapabilities   - Class in  weka.core  Locates all classes with certain capabilities.   FindWithCapabilities()   - 
Constructor for class weka.core. FindWithCapabilities 
  
   findWriteMethod(Object, String)   - 
Static method in class weka.core.xml. XMLSerializationMethodHandler 
 returns the method with the given name that has the same signature as
  writeToXML()  of the  XMLSerialiation  class.
   FINE   - 
Static variable in class weka.core. Debug 
 the log level Fine
   FINER   - 
Static variable in class weka.core. Debug 
 the log level Finer
   FINEST   - 
Static variable in class weka.core. Debug 
 the log level Finest
   finished()   - 
Method in class weka.experiment. OutputZipper 
 Closes the zip file.
   FINISHED   - 
Static variable in class weka.experiment. TaskStatusInfo 
  
   finished()   - 
Method in class weka.gui.visualize. PostscriptGraphics 
 Finalizes output file.
   fireLayoutCompleteEvent(LayoutCompleteEvent)   - 
Method in class weka.gui.graphvisualizer. HierarchicalBCEngine 
 Fires a LayoutCompleteEvent.
   fireLayoutCompleteEvent(LayoutCompleteEvent)   - 
Method in interface weka.gui.graphvisualizer. LayoutEngine 
 This fires a LayoutCompleteEvent once a layout has been completed.
   FIRST   - 
Static variable in class weka.filters.unsupervised.attribute. ClassAssigner 
 use the first attribute as class.
   firstElement()   - 
Method in class weka.core. FastVector 
 Returns the first element of the vector.
   firstElement()   - 
Method in class weka.gui. CheckBoxList.CheckBoxListModel 
 Returns the first component of this list.
   firstInstance()   - 
Method in class weka.core. Instances 
 Returns the first instance in the set.
   FirstOrder   - Class in  weka.filters.unsupervised.attribute  This instance filter takes a range of N numeric attributes and replaces them with N-1 numeric attributes, the values of which are the difference between consecutive attribute values from the original instance.   FirstOrder()   - 
Constructor for class weka.filters.unsupervised.attribute. FirstOrder 
  
   firstValueIndexTipText()   - 
Method in class weka.filters.unsupervised.attribute. MergeTwoValues 
  
   firstValueIndexTipText()   - 
Method in class weka.filters.unsupervised.attribute. SwapValues 
  
   fit(DoubleVector)   - 
Method in class weka.classifiers.functions.pace. MixtureDistribution 
 Fits the mixture (or mixing) distribution to the data.
   fit(DoubleVector, int)   - 
Method in class weka.classifiers.functions.pace. MixtureDistribution 
 Fits the mixture (or mixing) distribution to the data.
   fitForSingleCluster(DoubleVector, int)   - 
Method in class weka.classifiers.functions.pace. MixtureDistribution 
 Fits the mixture (or mixing) distribution to the data.
   fittingIntervals(DoubleVector)   - 
Method in class weka.classifiers.functions.pace. ChisqMixture 
 Contructs the set of fitting intervals for mixture estimation.
   fittingIntervals(DoubleVector)   - 
Method in class weka.classifiers.functions.pace. MixtureDistribution 
 Contructs the set of fitting intervals for mixture estimation.
   fittingIntervals(DoubleVector)   - 
Method in class weka.classifiers.functions.pace. NormalMixture 
 Contructs the set of fitting intervals for mixture estimation.
   fitToScreen()   - 
Method in class weka.gui.hierarchyvisualizer. HierarchyVisualizer 
 Fits the tree to the current screen size.
   fitToScreen()   - 
Method in class weka.gui.treevisualizer. TreeVisualizer 
 Fits the tree to the current screen size.
   FlexibleDecimalFormat   - Class in  weka.core.matrix      FlexibleDecimalFormat()   - 
Constructor for class weka.core.matrix. FlexibleDecimalFormat 
  
   FlexibleDecimalFormat(int)   - 
Constructor for class weka.core.matrix. FlexibleDecimalFormat 
  
   FlexibleDecimalFormat(int, boolean)   - 
Constructor for class weka.core.matrix. FlexibleDecimalFormat 
  
   FlexibleDecimalFormat(int, boolean, boolean, boolean)   - 
Constructor for class weka.core.matrix. FlexibleDecimalFormat 
  
   FlexibleDecimalFormat(double)   - 
Constructor for class weka.core.matrix. FlexibleDecimalFormat 
  
   FLOAT   - 
Static variable in class weka.experiment. DatabaseUtils 
 Type mapping for FLOAT used for reading experiment results.
   floatingForwardSearch(int, BitSet, int[], int, boolean, int, Instances, SubsetEvaluator, boolean)   - 
Method in class weka.attributeSelection. LFSMethods 
 Performs linear floating forward selection
 ( the stopping criteria cannot be changed to a specific size value )
   FloatingPointFormat   - Class in  weka.core.matrix  Class for the format of floating point numbers   FloatingPointFormat()   - 
Constructor for class weka.core.matrix. FloatingPointFormat 
 Default constructor
   FloatingPointFormat(int)   - 
Constructor for class weka.core.matrix. FloatingPointFormat 
  
   FloatingPointFormat(int, int)   - 
Constructor for class weka.core.matrix. FloatingPointFormat 
  
   FloatingPointFormat(int, int, boolean)   - 
Constructor for class weka.core.matrix. FloatingPointFormat 
  
   FLOOR   - 
Static variable in interface weka.classifiers.lazy.kstar. KStarConstants 
  
   FLOOR   - 
Static variable in interface weka.core.mathematicalexpression. sym 
  
   FLOOR   - 
Static variable in interface weka.filters.unsupervised.instance.subsetbyexpression. sym 
  
   FLOOR1   - 
Static variable in interface weka.classifiers.lazy.kstar. KStarConstants 
  
   FlowRunner   - Class in  weka.gui.beans  Small utility class for executing KnowledgeFlow
 flows outside of the KnowledgeFlow application   FlowRunner()   - 
Constructor for class weka.gui.beans. FlowRunner 
 Constructor
   FlowRunner.SimpleLogger   - Class in  weka.gui.beans      FlowRunner.SimpleLogger()   - 
Constructor for class weka.gui.beans. FlowRunner.SimpleLogger 
  
   flush()   - 
Method in class weka.core.logging. OutputLogger.OutputPrintStream 
 ignored.
   flush()   - 
Method in class weka.core. Tee 
 flushes all the printstreams.
   fMeasure(int)   - 
Method in class weka.classifiers. Evaluation 
 Calculate the F-Measure with respect to a particular class.
   FMEASURE   - 
Static variable in class weka.classifiers.meta. ThresholdSelector 
 F-measure
   FMEASURE_NAME   - 
Static variable in class weka.classifiers.evaluation. ThresholdCurve 
 attribute name: FMeasure
   FOLD_FIELD_NAME   - 
Static variable in class weka.experiment. CrossValidationResultProducer 
 The name of the key field containing the fold number
   foldsTipText()   - 
Method in class weka.attributeSelection. OneRAttributeEval 
 Returns a string for this option suitable for display in the gui
 as a tip text
   foldsTipText()   - 
Method in class weka.attributeSelection. WrapperSubsetEval 
 Returns the tip text for this property
   foldsTipText()   - 
Method in class weka.classifiers.rules. ConjunctiveRule 
 Returns the tip text for this property
   foldsTipText()   - 
Method in class weka.classifiers.rules. JRip 
 Returns the tip text for this property
   foldsTipText()   - 
Method in class weka.classifiers.rules. Ridor 
 Returns the tip text for this property
   foldsTipText()   - 
Method in class weka.gui.beans. CrossValidationFoldMaker 
 Tip text for this property
   foldsTypeTipText()   - 
Method in class weka.attributeSelection. RaceSearch 
 Returns the tip text for this property
   foldTipText()   - 
Method in class weka.filters.supervised.instance. StratifiedRemoveFolds 
 Returns the tip text for this property
   foldTipText()   - 
Method in class weka.filters.unsupervised.instance. RemoveFolds 
 Returns the tip text for this property
   forCapabilities(Capabilities)   - 
Static method in class weka.core. TestInstances 
 returns a TestInstances instance setup already for the the given
 capabilities.
   forInstances(Instances)   - 
Static method in class weka.core. Capabilities 
 returns a Capabilities object specific for this data.
   forInstances(Instances, boolean)   - 
Static method in class weka.core. Capabilities 
 returns a Capabilities object specific for this data.
   format(double, StringBuffer, FieldPosition)   - 
Method in class weka.core.matrix. ExponentialFormat 
  
   format(double, StringBuffer, FieldPosition)   - 
Method in class weka.core.matrix. FlexibleDecimalFormat 
  
   format(double, StringBuffer, FieldPosition)   - 
Method in class weka.core.matrix. FloatingPointFormat 
  
   FORMAT_AVAILABLE   - 
Static variable in class weka.gui.beans. InstanceEvent 
  
   FORMAT_AVAILABLE   - 
Static variable in class weka.gui.streams. InstanceEvent 
 Specifies that the instance format is available
   FORMAT_HHMMSS   - 
Static variable in class weka.core. Debug.Clock 
 the output format in hours:minutes:seconds, with fraction of msecs
   FORMAT_MILLISECONDS   - 
Static variable in class weka.core. Debug.Clock 
 the output format in milli-seconds
   FORMAT_SECONDS   - 
Static variable in class weka.core. Debug.Clock 
 the output format in seconds, with fraction of msecs
   formatDate(double)   - 
Method in class weka.core. Attribute 
 Returns the given amount of milliseconds formatted according to the
 current Date format.
   formatString(String)   - 
Method in class weka.core.matrix. FlexibleDecimalFormat 
  
   formatTipText()   - 
Method in class weka.datagenerators. DataGenerator 
 Returns the tip text for this property
   forName(String, String[])   - 
Static method in class weka.associations. AbstractAssociator 
 Creates a new instance of a associator given it's class name and
 (optional) arguments to pass to it's setOptions method.
   forName(String, String[])   - 
Static method in class weka.attributeSelection. ASEvaluation 
 Creates a new instance of an attribute/subset evaluator 
 given it's class name and
 (optional) arguments to pass to it's setOptions method.
   forName(String, String[])   - 
Static method in class weka.attributeSelection. ASSearch 
 Creates a new instance of a search class given it's class name and
 (optional) arguments to pass to it's setOptions method.
   forName(String, String[])   - 
Static method in class weka.classifiers. Classifier 
 Creates a new instance of a classifier given it's class name and
 (optional) arguments to pass to it's setOptions method.
   forName(String, String[])   - 
Static method in class weka.classifiers.functions.supportVector. Kernel 
 Creates a new instance of a kernel given it's class name and
 (optional) arguments to pass to it's setOptions method.
   forName(String, String[])   - 
Static method in class weka.clusterers. AbstractClusterer 
 Creates a new instance of a clusterer given it's class name and
 (optional) arguments to pass to it's setOptions method.
   forName(Class, String, String[])   - 
Static method in class weka.core. Utils 
 Creates a new instance of an object given it's class name and
 (optional) arguments to pass to it's setOptions method.
   forName(String, String[])   - 
Static method in class weka.estimators. Estimator 
 Creates a new instance of a estimatorr given it's class name and
 (optional) arguments to pass to it's setOptions method.
   forward(PaceMatrix, IntVector, int)   - 
Method in class weka.classifiers.functions.pace. PaceMatrix 
 Forward ordering of columns in terms of response explanation.
   forwardSearch(int, BitSet, int[], int, boolean, int, int, Instances, SubsetEvaluator, boolean)   - 
Method in class weka.attributeSelection. LFSMethods 
 Performs linear forward selection
   forwardSelectionMethodTipText()   - 
Method in class weka.attributeSelection. LinearForwardSelection 
 Returns the tip text for this property
   foundUsefulAttribute()   - 
Method in class weka.classifiers.functions. SimpleLinearRegression 
 Returns true if a usable attribute was found.
   FP_RATE_NAME   - 
Static variable in class weka.classifiers.evaluation. ThresholdCurve 
 attribute name: False Positive Rate"
   FPGrowth   - Class in  weka.associations  Class implementing the FP-growth algorithm for finding large item sets without candidate generation.   FPGrowth()   - 
Constructor for class weka.associations. FPGrowth 
 Construct a new FPGrowth object.
   FPGrowth.AssociationRule   - Class in  weka.associations      FPGrowth.AssociationRule(Collection<FPGrowth.BinaryItem>, Collection<FPGrowth.BinaryItem>, FPGrowth.AssociationRule.METRIC_TYPE, int, int, int, int)   - 
Constructor for class weka.associations. FPGrowth.AssociationRule 
 Construct a new association rule.
   FPGrowth.AssociationRule.METRIC_TYPE   - Enum in  weka.associations  Enum for holding different metric types   FPGrowth.BinaryItem   - Class in  weka.associations  Inner class that handles a single binary item   FPGrowth.BinaryItem(Attribute, int)   - 
Constructor for class weka.associations. FPGrowth.BinaryItem 
  
   FProbability(double, int, int)   - 
Static method in class weka.core. Statistics 
 Computes probability of F-ratio.
   freeNotCoveredInstances()   - 
Method in class weka.classifiers.trees.m5. Rule 
 Free up memory consumed by the set of instances
 not covered by this rule.
   FREQ_ASCEND   - 
Static variable in class weka.filters.supervised.attribute. ClassOrder 
 The class values are sorted in ascending order based on their frequencies
   FREQ_DESCEND   - 
Static variable in class weka.filters.supervised.attribute. ClassOrder 
 The class values are sorted in descending order based on their frequencies
   frequencyLimitTipText()   - 
Method in class weka.classifiers.bayes. AODE 
 Returns the tip text for this property
   frequencyLimitTipText()   - 
Method in class weka.classifiers.bayes. AODEsr 
 Returns the tip text for this property
   frequencyThresholdTipText()   - 
Method in class weka.associations. Tertius 
 Returns the tip text for this property.
   FromFile   - Class in  weka.classifiers.bayes.net.search.fixed  The FromFile reads the structure of a Bayes net from a file in BIFF format.   FromFile()   - 
Constructor for class weka.classifiers.bayes.net.search.fixed. FromFile 
  
   fromXML(Document)   - 
Method in class weka.core.xml. XMLSerialization 
 returns the given DOM document as an instance of the specified class
   FT   - Class in  weka.classifiers.trees  Classifier for building 'Functional trees', which are classification trees  that could have logistic regression functions at the inner nodes and/or leaves.   FT()   - 
Constructor for class weka.classifiers.trees. FT 
 Creates an instance of FT with standard options
   FTInnerNode   - Class in  weka.classifiers.trees.ft  Class for Functional Inner tree structure.   FTInnerNode(boolean, int, int, double, boolean)   - 
Constructor for class weka.classifiers.trees.ft. FTInnerNode 
 Constructor for Functional Inner tree node.
   FTLeavesNode   - Class in  weka.classifiers.trees.ft  Class for Functional Leaves tree version.   FTLeavesNode(boolean, int, int, double, boolean)   - 
Constructor for class weka.classifiers.trees.ft. FTLeavesNode 
 Constructor for Functional Leaves tree node.
   FTNode   - Class in  weka.classifiers.trees.ft  Class for Functional tree structure.   FTNode(boolean, int, int, double, boolean)   - 
Constructor for class weka.classifiers.trees.ft. FTNode 
 Constructor for Functional tree node.
   FTtree   - Class in  weka.classifiers.trees.ft  Abstract class for Functional tree structure.   FTtree()   - 
Constructor for class weka.classifiers.trees.ft. FTtree 
  
   fullValue()   - 
Method in class weka.gui. HierarchyPropertyParser 
 The full value of the current node, i.e.
   Function   - Class in  weka.core.pmml  Abstract superclass for PMML built-in and DefineFunctions.   Function()   - 
Constructor for class weka.core.pmml. Function 
  
   FUNCTION_1   - 
Static variable in class weka.datagenerators.classifiers.classification. Agrawal 
 function 1
   FUNCTION_10   - 
Static variable in class weka.datagenerators.classifiers.classification. Agrawal 
 function 10
   FUNCTION_2   - 
Static variable in class weka.datagenerators.classifiers.classification. Agrawal 
 function 2
   FUNCTION_3   - 
Static variable in class weka.datagenerators.classifiers.classification. Agrawal 
 function 3
   FUNCTION_4   - 
Static variable in class weka.datagenerators.classifiers.classification. Agrawal 
 function 4
   FUNCTION_5   - 
Static variable in class weka.datagenerators.classifiers.classification. Agrawal 
 function 5
   FUNCTION_6   - 
Static variable in class weka.datagenerators.classifiers.classification. Agrawal 
 function 6
   FUNCTION_7   - 
Static variable in class weka.datagenerators.classifiers.classification. Agrawal 
 function 7
   FUNCTION_8   - 
Static variable in class weka.datagenerators.classifiers.classification. Agrawal 
 function 8
   FUNCTION_9   - 
Static variable in class weka.datagenerators.classifiers.classification. Agrawal 
 function 9
   FUNCTION_TAGS   - 
Static variable in class weka.datagenerators.classifiers.classification. Agrawal 
 the funtion tags
   functionTipText()   - 
Method in class weka.datagenerators.classifiers.classification. Agrawal 
 Returns the tip text for this property
 
 
    
 G  
 
   g1(double, double)   - 
Method in class weka.classifiers.functions.pace. PaceMatrix 
 Constructs the Givens rotation
   g2(double[], int, int, int)   - 
Method in class weka.classifiers.functions.pace. PaceMatrix 
 Performs the Givens rotation
   gainRatio()   - 
Method in class weka.classifiers.trees.j48. BinC45Split 
 Returns (C4.5-type) gain ratio for the generated split.
   gainRatio()   - 
Method in class weka.classifiers.trees.j48. C45Split 
 Returns (C4.5-type) gain ratio for the generated split.
   gainRatio(double[][])   - 
Static method in class weka.core. ContingencyTables 
 Computes gain ratio for contingency table (split on rows).
   GainRatioAttributeEval   - Class in  weka.attributeSelection  GainRatioAttributeEval : 
  
 Evaluates the worth of an attribute by measuring the gain ratio with respect to the class. 
  
 GainR(Class, Attribute) = (H(Class) - H(Class | Attribute)) / H(Attribute). 
  
  

  
 Valid options are:     GainRatioAttributeEval()   - 
Constructor for class weka.attributeSelection. GainRatioAttributeEval 
 Constructor
   GainRatioSplitCrit   - Class in  weka.classifiers.trees.j48  Class for computing the gain ratio for a given distribution.   GainRatioSplitCrit()   - 
Constructor for class weka.classifiers.trees.j48. GainRatioSplitCrit 
  
   gamma(double)   - 
Static method in class weka.core. Statistics 
 Returns the Gamma function of the argument.
   gammaTipText()   - 
Method in class weka.classifiers.functions. LibSVM 
 Returns the tip text for this property
   gammaTipText()   - 
Method in class weka.classifiers.functions.supportVector. RBFKernel 
 Returns the tip text for this property
   GAUSSIAN   - 
Static variable in class weka.classifiers.bayes. BayesianLogisticRegression 
 Distributions available
   GAUSSIAN   - 
Static variable in class weka.datagenerators.clusterers. SubspaceCluster 
 cluster type: gaussian
   GAUSSIAN   - 
Static variable in class weka.filters.unsupervised.attribute. RandomProjection 
 distribution type: gaussian
   GaussianPriorImpl   - Class in  weka.classifiers.bayes.blr  Implementation of the Gaussian Prior update function based on
 CLG Algorithm with a certain Trust Region Update.   GaussianPriorImpl()   - 
Constructor for class weka.classifiers.bayes.blr. GaussianPriorImpl 
  
   GaussianProcesses   - Class in  weka.classifiers.functions  Implements Gaussian Processes for regression without hyperparameter-tuning.   GaussianProcesses()   - 
Constructor for class weka.classifiers.functions. GaussianProcesses 
 the default constructor
   GE   - 
Static variable in interface weka.core.mathematicalexpression. sym 
  
   GE   - 
Static variable in interface weka.filters.unsupervised.instance.subsetbyexpression. sym 
  
   GeneralizedSequentialPatterns   - Class in  weka.associations  Class implementing a GSP algorithm for discovering sequential patterns in a sequential data set. 
 The attribute identifying the distinct data sequences contained in the set can be determined by the respective option.   GeneralizedSequentialPatterns()   - 
Constructor for class weka.associations. GeneralizedSequentialPatterns 
 Constructor.
   GeneralRegression   - Class in  weka.classifiers.pmml.consumer  Class implementing import of PMML General Regression model.   GeneralRegression(Element, Instances, MiningSchema)   - 
Constructor for class weka.classifiers.pmml.consumer. GeneralRegression 
 Constructs a GeneralRegression classifier.
   generate()   - 
Method in class weka.core. Javadoc 
 generates either the plain Javadoc (if no filename specified) or the
 updated file (if a filename is specified).
   generate()   - 
Method in class weka.core. ListOptions 
 generates the options string.
   generate()   - 
Method in class weka.core. TestInstances 
 Generates a new dataset
   generate(String)   - 
Method in class weka.core. TestInstances 
 generates a new dataset.
   generateDistribution()   - 
Method in class weka.associations. PriorEstimation 
 Calculates the prior distribution.
   generateExample()   - 
Method in class weka.datagenerators.classifiers.classification. Agrawal 
 Generates one example of the dataset.
   generateExample()   - 
Method in class weka.datagenerators.classifiers.classification. BayesNet 
 Generates one example of the dataset.
   generateExample()   - 
Method in class weka.datagenerators.classifiers.classification. LED24 
 Generates one example of the dataset.
   generateExample()   - 
Method in class weka.datagenerators.classifiers.classification. RandomRBF 
 Generates one example of the dataset.
   generateExample()   - 
Method in class weka.datagenerators.classifiers.classification. RDG1 
 Generate an example of the dataset dataset.
   generateExample()   - 
Method in class weka.datagenerators.classifiers.regression. Expression 
 Generates one example of the dataset.
   generateExample()   - 
Method in class weka.datagenerators.classifiers.regression. MexicanHat 
 Generates one example of the dataset.
   generateExample()   - 
Method in class weka.datagenerators.clusterers. BIRCHCluster 
 Generate an example of the dataset.
   generateExample()   - 
Method in class weka.datagenerators.clusterers. SubspaceCluster 
 Generate an example of the dataset.
   generateExample()   - 
Method in class weka.datagenerators. DataGenerator 
 Generates one example of the dataset.
   generateExamples()   - 
Method in class weka.datagenerators.classifiers.classification. Agrawal 
 Generates all examples of the dataset.
   generateExamples()   - 
Method in class weka.datagenerators.classifiers.classification. BayesNet 
 Generates all examples of the dataset.
   generateExamples()   - 
Method in class weka.datagenerators.classifiers.classification. LED24 
 Generates all examples of the dataset.
   generateExamples()   - 
Method in class weka.datagenerators.classifiers.classification. RandomRBF 
 Generates all examples of the dataset.
   generateExamples()   - 
Method in class weka.datagenerators.classifiers.classification. RDG1 
 Generate all examples of the dataset.
   generateExamples(int, Random, Instances)   - 
Method in class weka.datagenerators.classifiers.classification. RDG1 
 Generate all examples of the dataset.
   generateExamples()   - 
Method in class weka.datagenerators.classifiers.regression. Expression 
 Generates all examples of the dataset.
   generateExamples()   - 
Method in class weka.datagenerators.classifiers.regression. MexicanHat 
 Generates all examples of the dataset.
   generateExamples()   - 
Method in class weka.datagenerators.clusterers. BIRCHCluster 
 Generate all examples of the dataset.
   generateExamples(Random, Instances)   - 
Method in class weka.datagenerators.clusterers. BIRCHCluster 
 Generate all examples of the dataset.
   generateExamples()   - 
Method in class weka.datagenerators.clusterers. SubspaceCluster 
 Generate all examples of the dataset.
   generateExamples()   - 
Method in class weka.datagenerators. DataGenerator 
 Generates all examples of the dataset.
   generateFinished()   - 
Method in class weka.datagenerators.classifiers.classification. Agrawal 
 Generates a comment string that documentats the data generator.
   generateFinished()   - 
Method in class weka.datagenerators.classifiers.classification. BayesNet 
 Generates a comment string that documentats the data generator.
   generateFinished()   - 
Method in class weka.datagenerators.classifiers.classification. LED24 
 Generates a comment string that documentats the data generator.
   generateFinished()   - 
Method in class weka.datagenerators.classifiers.classification. RandomRBF 
 Generates a comment string that documentats the data generator.
   generateFinished()   - 
Method in class weka.datagenerators.classifiers.classification. RDG1 
 Compiles documentation about the data generation.
   generateFinished()   - 
Method in class weka.datagenerators.classifiers.regression. Expression 
 Generates a comment string that documentats the data generator.
   generateFinished()   - 
Method in class weka.datagenerators.classifiers.regression. MexicanHat 
 Generates a comment string that documentats the data generator.
   generateFinished()   - 
Method in class weka.datagenerators.clusterers. BIRCHCluster 
 Compiles documentation about the data generation after
 the generation process
   generateFinished()   - 
Method in class weka.datagenerators.clusterers. SubspaceCluster 
 Compiles documentation about the data generation after
 the generation process
   generateFinished()   - 
Method in class weka.datagenerators. DataGenerator 
 Generates a comment string that documentates the data generator.
   generateHelp()   - 
Method in class weka.core. Javadoc 
 generates a string to print as help on the console
   generateHelp()   - 
Method in class weka.core. ListOptions 
 generates a string to print as help on the console
   generateInstances()   - 
Method in class weka.classifiers.bayes.net. BayesNetGenerator 
 GenerateInstances generates random instances sampling from the
 distribution represented by the Bayes network structure.
   generateInstances(int[])   - 
Method in interface weka.gui.boundaryvisualizer. DataGenerator 
 Generate an instance.
   generateInstances(int[])   - 
Method in class weka.gui.boundaryvisualizer. KDDataGenerator 
 Generates a new instance using one kernel estimator.
   generateInstances()   - 
Method in class weka.gui.explorer. PreprocessPanel 
 sets Instances generated via DataGenerators (pops up a Dialog)
   generateOutput()   - 
Method in class weka.gui.visualize. BMPWriter 
 generates the actual output
   generateOutput()   - 
Method in class weka.gui.visualize. JPEGWriter 
 generates the actual output.
   generateOutput()   - 
Method in class weka.gui.visualize. PNGWriter 
 generates the actual output
   generateOutput()   - 
Method in class weka.gui.visualize. PostscriptWriter 
 generates the actual output
   generateRandomNetwork()   - 
Method in class weka.classifiers.bayes.net. BayesNetGenerator 
 Generate random connected Bayesian network with discrete nodes
 having all the same cardinality.
   generateRandomNetworkStructure(int, int)   - 
Method in class weka.classifiers.bayes.net. BayesNetGenerator 
 GenerateRandomNetworkStructure generate random connected Bayesian network
   generateRandomNumber(int)   - 
Method in class weka.attributeSelection. ScatterSearchV1 
  
   generateRankingTipText()   - 
Method in class weka.attributeSelection. GreedyStepwise 
 Returns the tip text for this property
   generateRankingTipText()   - 
Method in class weka.attributeSelection. RaceSearch 
 Returns the tip text for this property
   generateRankingTipText()   - 
Method in class weka.attributeSelection. Ranker 
 Returns the tip text for this property
   GenerateReferenceSet(List<ScatterSearchV1.Subset>, int, int)   - 
Method in class weka.attributeSelection. ScatterSearchV1 
 Generate the a ReferenceSet containing the n best solutions and the m most diverse solutions of
 the initial Population.
   generateRuleItem(ItemSet, ItemSet, Instances, int, int, double[], Hashtable)   - 
Method in class weka.associations. RuleItem 
 Constructs a new RuleItem if the support of the given rule is above the support threshold.
   generateRules(double, FastVector, int)   - 
Method in class weka.associations. AprioriItemSet 
 Generates all rules for an item set.
   generateRules(int, double[], Hashtable, double, Instances, TreeSet, int)   - 
Method in class weka.associations. CaRuleGeneration 
 Generates all rules for an item set.
   generateRules(double, boolean)   - 
Method in class weka.associations. LabeledItemSet 
 Generates rules out of item sets
   generateRules(int, double[], Hashtable, double, Instances, TreeSet, int)   - 
Method in class weka.associations. RuleGeneration 
 Generates all rules for an item set.
   generateRulesBruteForce(double, int, FastVector, int, int, double)   - 
Method in class weka.associations. AprioriItemSet 
 Generates all significant rules for an item set.
   generateRulesBruteForce(FPGrowth.FrequentItemSets, FPGrowth.AssociationRule.METRIC_TYPE, double, int, int, int)   - 
Static method in class weka.associations. FPGrowth.AssociationRule 
 Generate all association rules, from the supplied frequet item sets,
 that meet a given minimum metric threshold.
   generateRulesTipText()   - 
Method in class weka.classifiers.trees.m5. M5Base 
 Returns the tip text for this property
   generateStart()   - 
Method in class weka.datagenerators.classifiers.classification. Agrawal 
 Generates a comment string that documentates the data generator.
   generateStart()   - 
Method in class weka.datagenerators.classifiers.classification. BayesNet 
 Generates a comment string that documentates the data generator.
   generateStart()   - 
Method in class weka.datagenerators.classifiers.classification. LED24 
 Generates a comment string that documentates the data generator.
   generateStart()   - 
Method in class weka.datagenerators.classifiers.classification. RandomRBF 
 Generates a comment string that documentates the data generator.
   generateStart()   - 
Method in class weka.datagenerators.classifiers.classification. RDG1 
 Generates a comment string that documentates the data generator.
   generateStart()   - 
Method in class weka.datagenerators.classifiers.regression. Expression 
 Generates a comment string that documentates the data generator.
   generateStart()   - 
Method in class weka.datagenerators.classifiers.regression. MexicanHat 
 Generates a comment string that documentates the data generator.
   generateStart()   - 
Method in class weka.datagenerators.clusterers. BIRCHCluster 
 Compiles documentation about the data generation before
 the generation process
   generateStart()   - 
Method in class weka.datagenerators.clusterers. SubspaceCluster 
 Compiles documentation about the data generation before
 the generation process
   generateStart()   - 
Method in class weka.datagenerators. DataGenerator 
 Generates a comment string that documentates the data generator.
   GeneratorPropertyIteratorPanel   - Class in  weka.gui.experiment  This panel controls setting a list of values for an arbitrary
 resultgenerator property for an experiment to iterate over.   GeneratorPropertyIteratorPanel()   - 
Constructor for class weka.gui.experiment. GeneratorPropertyIteratorPanel 
 Creates the property iterator panel initially disabled.
   GeneratorPropertyIteratorPanel(Experiment)   - 
Constructor for class weka.gui.experiment. GeneratorPropertyIteratorPanel 
 Creates the property iterator panel and sets the experiment.
   GenericArrayEditor   - Class in  weka.gui  A PropertyEditor for arrays of objects that themselves have
 property editors.   GenericArrayEditor()   - 
Constructor for class weka.gui. GenericArrayEditor 
 Sets up the array editor.
   GenericObjectEditor   - Class in  weka.gui  A PropertyEditor for objects.   GenericObjectEditor()   - 
Constructor for class weka.gui. GenericObjectEditor 
 Default constructor.
   GenericObjectEditor(boolean)   - 
Constructor for class weka.gui. GenericObjectEditor 
 Constructor that allows specifying whether it is possible
 to change the class within the editor dialog.
   GenericObjectEditor.CapabilitiesFilterDialog   - Class in  weka.gui  A dialog for selecting Capabilities to look for in the GOE tree.   GenericObjectEditor.CapabilitiesFilterDialog()   - 
Constructor for class weka.gui. GenericObjectEditor.CapabilitiesFilterDialog 
 creates a dialog to choose Capabilities from.
   GenericObjectEditor.GOEPanel   - Class in  weka.gui  Handles the GUI side of editing values.   GenericObjectEditor.GOEPanel()   - 
Constructor for class weka.gui. GenericObjectEditor.GOEPanel 
 Creates the GUI editor component.
   GenericObjectEditor.GOETreeNode   - Class in  weka.gui  A specialized TreeNode for supporting filtering via Capabilities.   GenericObjectEditor.GOETreeNode()   - 
Constructor for class weka.gui. GenericObjectEditor.GOETreeNode 
 Creates a tree node that has no parent and no children, but which 
 allows children.
   GenericObjectEditor.GOETreeNode(Object)   - 
Constructor for class weka.gui. GenericObjectEditor.GOETreeNode 
 Creates a tree node with no parent, no children, but which allows 
 children, and initializes it with the specified user object.
   GenericObjectEditor.GOETreeNode(Object, boolean)   - 
Constructor for class weka.gui. GenericObjectEditor.GOETreeNode 
 Creates a tree node with no parent, no children, initialized with the 
 specified user object, and that allows children only if specified.
   GenericObjectEditor.JTreePopupMenu   - Class in  weka.gui  Creates a popup menu containing a tree that is aware
 of the screen dimensions.   GenericObjectEditor.JTreePopupMenu(JTree)   - 
Constructor for class weka.gui. GenericObjectEditor.JTreePopupMenu 
 Constructs a new popup menu.
   GenericPropertiesCreator   - Class in  weka.gui  This class can generate the properties object that is normally loaded from
 the  GenericObjectEditor.props  file (= PROPERTY_FILE).   GenericPropertiesCreator()   - 
Constructor for class weka.gui. GenericPropertiesCreator 
 initializes the creator, locates the props file with the Utils class.
   GenericPropertiesCreator(String)   - 
Constructor for class weka.gui. GenericPropertiesCreator 
 initializes the creator, the given file overrides the props-file search
 of the Utils class
   GeneticSearch   - Class in  weka.attributeSelection  GeneticSearch: 
  
 Performs a search using the simple genetic algorithm described in Goldberg (1989). 
  
 For more information see: 
  
 David E.   GeneticSearch()   - 
Constructor for class weka.attributeSelection. GeneticSearch 
 Constructor.
   GeneticSearch   - Class in  weka.classifiers.bayes.net.search.global  This Bayes Network learning algorithm uses genetic search for finding a well scoring Bayes network structure.   GeneticSearch()   - 
Constructor for class weka.classifiers.bayes.net.search.global. GeneticSearch 
  
   GeneticSearch   - Class in  weka.classifiers.bayes.net.search.local  This Bayes Network learning algorithm uses genetic search for finding a well scoring Bayes network structure.   GeneticSearch()   - 
Constructor for class weka.classifiers.bayes.net.search.local. GeneticSearch 
  
   get(int)   - 
Method in class weka.core.matrix. DoubleVector 
 Gets a single element.
   get(int)   - 
Method in class weka.core.matrix. IntVector 
 Gets the value of an element.
   get(int, int)   - 
Method in class weka.core.matrix. Matrix 
 Get a single element.
   get(int)   - 
Method in class weka.core. PropertyPath.Path 
 returns the element at the given index
   get(int)   - 
Method in class weka.core. Tee 
 returns the specified PrintStream from the list.
   get(String)   - 
Method in class weka.core.xml. MethodHandler 
 returns the stored method for the given property
   get(Class)   - 
Method in class weka.core.xml. MethodHandler 
 returns the stored method for the given class
   get(int)   - 
Method in class weka.gui. CheckBoxList.CheckBoxListModel 
 Returns the element at the specified position in this list.
   get(String, String)   - 
Static method in class weka.gui.experiment. ExperimenterDefaults 
 returns the value for the specified property, if non-existent then the
 default value.
   get(String, String)   - 
Static method in class weka.gui.explorer. ExplorerDefaults 
 returns the value for the specified property, if non-existent then the
 default value.
   getAboutPanel()   - 
Method in class weka.gui. PropertySheetPanel 
 Return the panel containing global info and help for
 the object being edited.
   getActionListener(JFrame)   - 
Method in interface weka.gui. MainMenuExtension 
 If the extension has a custom ActionListener for the menu item, then it
 must be returned here.
   getActualIndex(int)   - 
Method in class weka.core. AttributeLocator 
 returns actual index in the Instances object.
   getActualRow(int)   - 
Method in class weka.gui. SortedTableModel 
 Returns the actual underlying row the given visible one represents.
   getAcuity()   - 
Method in class weka.clusterers. Cobweb 
 get the acuity value
   getAddress()   - 
Static method in class weka.core. Copyright 
 returns the address of the owner
   getAdjustWeights()   - 
Method in class weka.filters.supervised.instance. SpreadSubsample 
 Returns true if instance  weights will be adjusted to maintain
 total weight per class.
   getADTree()   - 
Method in class weka.classifiers.bayes. BayesNet 
 get ADTree strucrture containing efficient representation of counts.
   getAdvanceDataSetFirst()   - 
Method in class weka.experiment. Experiment 
 Get the value of m_DataSetFirstFirst.
   getAlgorithm()   - 
Method in class weka.filters.supervised.attribute. PLSFilter 
 Gets the type of algorithm to use
   getAlgorithm()   - 
Method in class weka.filters.unsupervised.attribute. Wavelet 
 Gets the type of algorithm to use
   getAlgorithmStart()   - 
Method in class weka.associations. GeneralizedSequentialPatterns 
 Returns the time/date string the algorithm was started
   getAlgorithmType()   - 
Method in class weka.classifiers.mi. MILR 
 Gets the type of algorithm.
   getAllBits(List<ScatterSearchV1.Subset>)   - 
Method in class weka.attributeSelection. ScatterSearchV1 
 Save in Bitset all the gens that are in many others subsets.
   getAllowedIndices()   - 
Method in class weka.core. AttributeLocator 
 returns the indices that are allowed to check for the attribute type
   getAllowUnclassifiedInstances()   - 
Method in class weka.classifiers.trees. RandomTree 
 Get the value of NumFolds.
   getAllTheRules()   - 
Method in class weka.associations. Apriori 
 returns all the rules
   getAllTheRules()   - 
Method in class weka.associations. PredictiveApriori 
 returns all the rules
   getAlpha()   - 
Method in class weka.classifiers.bayes.net.estimate. BayesNetEstimator 
 Get prior used in probability table estimation
   getAlpha()   - 
Method in class weka.classifiers.functions. Winnow 
 Get the value of Alpha.
   getAmplitude()   - 
Method in class weka.datagenerators.classifiers.regression. MexicanHat 
 Gets the amplitude multiplier.
   getAnimatedIcon()   - 
Method in class weka.gui.beans. BeanVisual 
 Returns the animated icon
   getAnimatedIconPath()   - 
Method in class weka.gui.beans. BeanVisual 
 returns the path for the animated icon
   getAppendPredictedProbabilities()   - 
Method in class weka.gui.beans. PredictionAppender 
 Return true if predicted probabilities are to be appended rather
 than class value
   getArffFile()   - 
Method in class weka.gui.streams. InstanceLoader 
  
   getArffFile()   - 
Method in class weka.gui.streams. InstanceSavePanel 
  
   getArray()   - 
Method in class weka.core.matrix. DoubleVector 
 Access the internal one-dimensional array.
   getArray()   - 
Method in class weka.core.matrix. IntVector 
 Access the internal one-dimensional array.
   getArray()   - 
Method in class weka.core.matrix. Matrix 
 Access the internal two-dimensional array.
   getArrayClass(Class)   - 
Static method in class weka.core. Utils 
 Returns the basic class of an array class (handles multi-dimensional
 arrays).
   getArrayCopy()   - 
Method in class weka.core.matrix. DoubleVector 
 Returns a copy of the DoubleVector usng a double array.
   getArrayCopy()   - 
Method in class weka.core.matrix. IntVector 
 Returns a copy of the internal one-dimensional array.
   getArrayCopy()   - 
Method in class weka.core.matrix. Matrix 
 Copy the internal two-dimensional array.
   getArrayDimensions(Class)   - 
Static method in class weka.core. Utils 
 Returns the dimensions of the given array.
   getArrayDimensions(Object)   - 
Static method in class weka.core. Utils 
 Returns the dimensions of the given array.
   getArtificialSize()   - 
Method in class weka.classifiers.meta. Decorate 
 Factor that determines number of artificial examples to generate.
   getASCrossvalidationFolds()   - 
Static method in class weka.gui.explorer. ExplorerDefaults 
 returns the default number of folds of the CV in the attribute selection 
 panel.
   getASEvaluator()   - 
Static method in class weka.gui.explorer. ExplorerDefaults 
 returns the default attribute evalautor (fully configured) for the 
 attribute selection panel.
   getAsInstance(Instances, Random)   - 
Method in class weka.core. AlgVector 
 Gets the elements of the vector as an instance.
   getASRandomSeed()   - 
Static method in class weka.gui.explorer. ExplorerDefaults 
 returns the default random seed value in the attribute selection panel.
   getASSearch()   - 
Static method in class weka.gui.explorer. ExplorerDefaults 
 returns the default attribute selection search scheme (fully configured) 
 for the attribute selection panel.
   getAssignments()   - 
Method in class weka.clusterers. SimpleKMeans 
 Gets the assignments for each instance
   getAssociatedConnections()   - 
Method in class weka.gui.beans. MetaBean 
  
   getAssociationRules()   - 
Method in class weka.associations. FPGrowth 
 Gets the list of mined association rules.
   getAssociator()   - 
Method in class weka.associations. CheckAssociator 
 Get the associator being tested
   getAssociator()   - 
Method in class weka.associations. SingleAssociatorEnhancer 
 Get the associator used as the base associator.
   getAssociator()   - 
Method in class weka.gui.beans. Associator 
 Get the associator currently set for this wrapper
   getAssociator()   - 
Static method in class weka.gui.explorer. ExplorerDefaults 
 returns the default associator (fully configured) for the associations panel.
   getASTestMode()   - 
Static method in class weka.gui.explorer. ExplorerDefaults 
 returns the default attribute selection test mode for the attribute
 selection panel.
   getAsText()   - 
Method in class weka.gui. CostMatrixEditor 
 Some objects can be represented as text, but a cost matrix cannot.
   getAsText()   - 
Method in class weka.gui. GenericArrayEditor 
 Returns null as we don't support getting/setting values as text.
   getAsText()   - 
Method in class weka.gui. GenericObjectEditor 
 Returns null as we don't support getting/setting values as text.
   getAsText()   - 
Method in class weka.gui. SelectedTagEditor 
 Gets the current value as text.
   getAsText()   - 
Method in class weka.gui. SimpleDateFormatEditor 
 Returns the date format string.
   getAttIndex(int)   - 
Method in class weka.classifiers.lazy. LBR.Indexes 
 Returns the boolean value at the specified index in the Attribute Indexes array
   getAttList_Irr()   - 
Method in class weka.datagenerators.classifiers.classification. RDG1 
 Gets the array that defines which of the attributes
 are seen to be irrelevant.
   getAttribute()   - 
Method in class weka.associations. FPGrowth.BinaryItem 
 Get the attribute that this item corresponds to.
   getAttribute1()   - 
Method in class weka.gui.visualize. VisualizePanelEvent 
  
   getAttribute2()   - 
Method in class weka.gui.visualize. VisualizePanelEvent 
  
   getAttributeAt(int)   - 
Method in class weka.gui.arffviewer. ArffSortedTableModel 
 returns the attribute at the given index, can be NULL if not an attribute
 column
   getAttributeAt(int)   - 
Method in class weka.gui.arffviewer. ArffTableModel 
 returns the attribute at the given index, can be NULL if not an attribute
 column
   getAttributeCapabilities()   - 
Method in class weka.core. Capabilities 
 returns all attribute capabilities
   getAttributeColumn(String)   - 
Method in class weka.gui.arffviewer. ArffSortedTableModel 
 returns the column of the given attribute name, -1 if not found
   getAttributeColumn(String)   - 
Method in class weka.gui.arffviewer. ArffTableModel 
 returns the column of the given attribute name, -1 if not found
   getAttributeEvaluator()   - 
Method in class weka.attributeSelection. FilteredAttributeEval 
 Get the attribute evaluator to use
   getAttributeEvaluator()   - 
Method in class weka.attributeSelection. RaceSearch 
 Get the attribute evaluator used to generate the ranking.
   getAttributeEvaluator()   - 
Method in class weka.attributeSelection. RankSearch 
 Get the attribute evaluator used to generate the ranking.
   getAttributeID()   - 
Method in class weka.experiment. ClassifierSplitEvaluator 
 Get the index of Attibute Identifying the instances
   getAttributeIndex()   - 
Method in class weka.classifiers.functions. SimpleLinearRegression 
 Returns the index of the attribute used in the regression.
   getAttributeIndex()   - 
Method in class weka.filters.unsupervised.attribute. Add 
 Get the index of the attribute used.
   getAttributeIndex()   - 
Method in class weka.filters.unsupervised.attribute. AddNoise 
 Get the index of the attribute used.
   getAttributeIndex()   - 
Method in class weka.filters.unsupervised.attribute. AddValues 
 Get the index of the attribute used.
   getAttributeIndex()   - 
Method in class weka.filters.unsupervised.attribute. ChangeDateFormat 
 Gets the index of the attribute converted.
   getAttributeIndex()   - 
Method in class weka.filters.unsupervised.attribute. MakeIndicator 
 Get the index of the attribute used.
   getAttributeIndex()   - 
Method in class weka.filters.unsupervised.attribute. MergeTwoValues 
 Get the index of the attribute used.
   getAttributeIndex()   - 
Method in class weka.filters.unsupervised.attribute. SwapValues 
 Get the index of the attribute used.
   getAttributeIndex()   - 
Method in class weka.filters.unsupervised.instance. RemoveFrequentValues 
 Get the index of the attribute used.
   getAttributeIndex()   - 
Method in class weka.filters.unsupervised.instance. RemoveWithValues 
 Get the index of the attribute used.
   getAttributeIndexes()   - 
Method in class weka.filters.unsupervised.attribute. NominalToString 
 Get the index of the attribute used.
   getAttributeIndices()   - 
Method in class weka.core. AttributeLocator 
 Returns the indices of the attributes.
   getAttributeIndices()   - 
Method in interface weka.core. DistanceFunction 
 Gets the range of attributes used in the calculation of the distance.
   getAttributeIndices()   - 
Method in class weka.core. NormalizableDistance 
 Gets the range of attributes used in the calculation of the distance.
   getAttributeIndices()   - 
Method in class weka.filters.supervised.attribute. Discretize 
 Gets the current range selection
   getAttributeIndices()   - 
Method in class weka.filters.unsupervised.attribute. AbstractTimeSeries 
 Get the current range selection
   getAttributeIndices()   - 
Method in class weka.filters.unsupervised.attribute. Copy 
 Get the current range selection
   getAttributeIndices()   - 
Method in class weka.filters.unsupervised.attribute. Discretize 
 Gets the current range selection
   getAttributeIndices()   - 
Method in class weka.filters.unsupervised.attribute. FirstOrder 
 Get the current range selection
   getAttributeIndices()   - 
Method in class weka.filters.unsupervised.attribute. InterquartileRange 
 Gets the current range selection
   getAttributeIndices()   - 
Method in class weka.filters.unsupervised.attribute. NominalToBinary 
 Gets the current range selection
   getAttributeIndices()   - 
Method in class weka.filters.unsupervised.attribute. NumericCleaner 
 Gets the selection of the columns, e.g., first-last or first-3,5-last
   getAttributeIndices()   - 
Method in class weka.filters.unsupervised.attribute. NumericToNominal 
 Gets the current range selection
   getAttributeIndices()   - 
Method in class weka.filters.unsupervised.attribute. NumericTransform 
 Get the current range selection
   getAttributeIndices()   - 
Method in class weka.filters.unsupervised.attribute. Remove 
 Get the current range selection.
   getAttributeIndices()   - 
Method in class weka.filters.unsupervised.attribute. Reorder 
 Get the current range selection
   getAttributeIndices()   - 
Method in class weka.filters.unsupervised.attribute. StringToWordVector 
 Gets the current range selection.
   getAttributeMaxValues()   - 
Method in interface weka.clusterers.forOPTICSAndDBScan.Databases. Database 
 Returns the array of maximum-values for each attribute
   getAttributeMaxValues()   - 
Method in class weka.clusterers.forOPTICSAndDBScan.Databases. SequentialDatabase 
 Returns the array of maximum-values for each attribute
   getAttributeMinValues()   - 
Method in interface weka.clusterers.forOPTICSAndDBScan.Databases. Database 
 Returns the array of minimum-values for each attribute
   getAttributeMinValues()   - 
Method in class weka.clusterers.forOPTICSAndDBScan.Databases. SequentialDatabase 
 Returns the array of minimum-values for each attribute
   getAttributeName()   - 
Method in class weka.filters.unsupervised.attribute. Add 
 Get the name of the attribute to be created.
   getAttributeName()   - 
Method in class weka.filters.unsupervised.attribute. AddID 
 Get the name of the attribute to be created
   getAttributeNamePrefix()   - 
Method in class weka.filters.unsupervised.attribute. StringToWordVector 
 Get the attribute name prefix.
   getAttributeRange()   - 
Method in class weka.filters.unsupervised.attribute. StringToNominal 
 Get the range of indices of the attributes used.
   getAttributes()   - 
Method in class weka.datagenerators.clusterers. SubspaceClusterDefinition 
  
   getAttributes()   - 
Method in class weka.gui.arffviewer. ArffPanel 
 returns a list with the attributes
   getAttributeSelectionMethod()   - 
Method in class weka.classifiers.functions. LinearRegression 
 Gets the method used to select attributes for use in the
 linear regression.
   getAttributeType()   - 
Method in class weka.filters.unsupervised.attribute. Add 
 Gets the type of attribute to generate.
   getAttributeType()   - 
Method in class weka.filters.unsupervised.attribute. RemoveType 
 Gets the attribute type to be deleted by the filter.
   getAttributeTypes()   - 
Method in class weka.filters.unsupervised.instance.subsetbyexpression. Parser 
 Returns the current attribute - attribute-type relation in use.
   getAttrIndexRange()   - 
Method in class weka.datagenerators.clusterers. SubspaceClusterDefinition 
 returns the attribute range(s).
   getAttsToEliminatePerIteration()   - 
Method in class weka.attributeSelection. SVMAttributeEval 
 Get the constant rate of attribute elimination per iteration
   getAutoBuild()   - 
Method in class weka.classifiers.functions. MultilayerPerceptron 
  
   getAutoKeyGeneration()   - 
Method in class weka.core.converters. DatabaseSaver 
 Gets whether or not a primary key will be generated automatically.
   getAverage(int)   - 
Method in class weka.experiment. ResultMatrix 
 returns the average of the mean at the given position, if the position is
 valid, otherwise 0
   getBackground()   - 
Method in class weka.gui.visualize. BMPWriter 
 returns the current background color
   getBackground()   - 
Method in class weka.gui.visualize. JPEGWriter 
 returns the current background color.
   getBackground()   - 
Method in class weka.gui.visualize. PNGWriter 
 returns the current background color
   getBackground()   - 
Method in class weka.gui.visualize. PostscriptGraphics 
  
   getBackup()   - 
Method in class weka.gui. GenericObjectEditor 
 Returns the backup object (may be null if there is no
 backup.
   getBagSizePercent()   - 
Method in class weka.classifiers.meta. Bagging 
 Gets the size of each bag, as a percentage of the training set size.
   getBagSizePercent()   - 
Method in class weka.classifiers.meta. MetaCost 
 Gets the size of each bag, as a percentage of the training set size.
   getBalanceClass()   - 
Method in class weka.datagenerators.classifiers.classification. Agrawal 
 Gets whether the class is balanced.
   getBalanced()   - 
Method in class weka.classifiers.functions. Winnow 
 Get the value of Balanced.
   getBallSplitter()   - 
Method in class weka.core.neighboursearch.balltrees. TopDownConstructor 
 Returns the BallSplitter algorithm set that would be 
 used by the TopDown BallTree constructor.
   getBallTreeConstructor()   - 
Method in class weka.core.neighboursearch. BallTree 
 Returns the BallTreeConstructor currently in use.
   getBase()   - 
Method in class weka.core.neighboursearch. CoverTree 
 Returns the base in use for expansion constant.
   getBaseExperiment()   - 
Method in class weka.experiment. RemoteExperiment 
 Get the base experiment used by this remote experiment
   getBean()   - 
Method in class weka.gui.beans. BeanInstance 
 Gets the bean encapsulated in this instance
   getBeanContext()   - 
Method in class weka.gui.beans. AbstractDataSource 
 Return the bean context (if any) that this bean is embedded in
   getBeanContext()   - 
Method in class weka.gui.beans. CostBenefitAnalysis 
  
   getBeanContext()   - 
Method in class weka.gui.beans. DataVisualizer 
 Return the bean context (if any) that this bean is embedded in
   getBeanContext()   - 
Method in class weka.gui.beans. GraphViewer 
 Return the bean context (if any) that this bean is embedded in
   getBeanContext()   - 
Method in class weka.gui.beans. ModelPerformanceChart 
 Return the bean context (if any) that this bean is embedded in
   getBeanContext()   - 
Method in class weka.gui.beans. TextViewer 
 Return the bean context (if any) that this bean is embedded in
   getBeanDescriptor()   - 
Method in class weka.gui.beans. AssociatorBeanInfo 
 Get the bean descriptor for this bean
   getBeanDescriptor()   - 
Method in class weka.gui.beans. ClassAssignerBeanInfo 
  
   getBeanDescriptor()   - 
Method in class weka.gui.beans. ClassifierBeanInfo 
 Get the bean descriptor for this bean
   getBeanDescriptor()   - 
Method in class weka.gui.beans. ClassValuePickerBeanInfo 
  
   getBeanDescriptor()   - 
Method in class weka.gui.beans. ClustererBeanInfo 
 Get the bean descriptor for this bean
   getBeanDescriptor()   - 
Method in class weka.gui.beans. CrossValidationFoldMakerBeanInfo 
 Return the bean descriptor for this bean
   getBeanDescriptor()   - 
Method in class weka.gui.beans. FilterBeanInfo 
 Get the bean descriptor for this bean
   getBeanDescriptor()   - 
Method in class weka.gui.beans. IncrementalClassifierEvaluatorBeanInfo 
 Return the bean descriptor for this bean
   getBeanDescriptor()   - 
Method in class weka.gui.beans. LoaderBeanInfo 
 Get the bean descriptor for this bean
   getBeanDescriptor()   - 
Method in class weka.gui.beans. PredictionAppenderBeanInfo 
 Return the bean descriptor for this bean
   getBeanDescriptor()   - 
Method in class weka.gui.beans. SaverBeanInfo 
 Get the bean descriptor for this bean
   getBeanDescriptor()   - 
Method in class weka.gui.beans. SerializedModelSaverBeanInfo 
 Get the bean descriptor for this bean
   getBeanDescriptor()   - 
Method in class weka.gui.beans. StripChartBeanInfo 
 Get the bean descriptor for this bean
   getBeanDescriptor()   - 
Method in class weka.gui.beans. TrainTestSplitMakerBeanInfo 
 Get the bean descriptor for this bean
   getBeanInfoInputs()   - 
Method in class weka.gui.beans. MetaBean 
  
   getBeanInfoOutputs()   - 
Method in class weka.gui.beans. MetaBean 
  
   getBeanInfoSubFlow()   - 
Method in class weka.gui.beans. MetaBean 
  
   getBeanInstances()   - 
Static method in class weka.gui.beans. BeanInstance 
 Return the list of displayed beans
   getBeansInInputs()   - 
Method in class weka.gui.beans. MetaBean 
 Return all the beans in the inputs
   getBeansInOutputs()   - 
Method in class weka.gui.beans. MetaBean 
 Return all the beans in the outputs
   getBeansInSubFlow()   - 
Method in class weka.gui.beans. MetaBean 
 Return all the beans in the sub flow
   getBestClassifier()   - 
Method in class weka.classifiers.meta. GridSearch 
 returns the best Classifier setup
   getBestClassifierIndex()   - 
Method in class weka.classifiers.meta. MultiScheme 
 Get the index of the classifier that was determined as best during 
 cross-validation.
   getBestClassifierOptions()   - 
Method in class weka.classifiers.meta. CVParameterSelection 
 Returns (a copy of) the best options found for the classifier.
   getBestCommitteeChunkSize()   - 
Method in class weka.classifiers.meta. RacedIncrementalLogitBoost 
 Get the best committee chunk size
   getBestCommitteeErrorEstimate()   - 
Method in class weka.classifiers.meta. RacedIncrementalLogitBoost 
 Get the best committee's error on the validation data
   getBestCommitteeLLEstimate()   - 
Method in class weka.classifiers.meta. RacedIncrementalLogitBoost 
 Get the best committee's log likelihood on the validation data
   getBestCommitteeSize()   - 
Method in class weka.classifiers.meta. RacedIncrementalLogitBoost 
 Get the number of members in the best committee
   getBestFilter()   - 
Method in class weka.classifiers.meta. GridSearch 
 returns the best filter setup
   getBestgen(ScatterSearchV1.Subset, BitSet)   - 
Method in class weka.attributeSelection. ScatterSearchV1 
 Evaluate each gen of a BitSet inserted in a Subset and get the most significant for that Subset
   getBestGroup()   - 
Method in class weka.attributeSelection. LFSMethods 
  
   getBestGroupOfSize(int)   - 
Method in class weka.attributeSelection. LFSMethods 
  
   getBestMerit()   - 
Method in class weka.attributeSelection. LFSMethods 
  
   getBeta()   - 
Method in class weka.classifiers.functions. Winnow 
 Get the value of Beta.
   getBias()   - 
Method in class weka.classifiers. BVDecompose 
 Get the calculated bias squared
   getBias()   - 
Method in class weka.classifiers.functions. LibLINEAR 
 Returns bias term value (default 1)
 No bias term is added if value < 0
   getBias()   - 
Method in class weka.classifiers.misc. VFI 
 Get the value of the bias parameter
   getBiasToUniformClass()   - 
Method in class weka.filters.supervised.instance. Resample 
 Gets the bias towards a uniform class.
   getBIFFile()   - 
Method in class weka.classifiers.bayes. BayesNet 
 Get name of network in BIF file to compare with
   getBIFFile()   - 
Method in class weka.classifiers.bayes.net.search.fixed. FromFile 
 Get name of network in BIF file to read structure from
   getBIFHeader()   - 
Method in class weka.classifiers.bayes. BayesNet 
  
   getBinarizeNumericAttributes()   - 
Method in class weka.attributeSelection. ChiSquaredAttributeEval 
 get whether numeric attributes are just being binarized.
   getBinarizeNumericAttributes()   - 
Method in class weka.attributeSelection. InfoGainAttributeEval 
 get whether numeric attributes are just being binarized.
   getBinaryAttributesNominal()   - 
Method in class weka.filters.supervised.attribute. NominalToBinary 
 Gets if binary attributes are to be treated as nominal ones.
   getBinaryAttributesNominal()   - 
Method in class weka.filters.unsupervised.attribute. NominalToBinary 
 Gets if binary attributes are to be treated as nominal ones.
   getBinarySplits()   - 
Method in class weka.classifiers.rules. PART 
 Get the value of binarySplits.
   getBinarySplits()   - 
Method in class weka.classifiers.trees. J48 
 Get the value of binarySplits.
   getBinarySplits()   - 
Method in class weka.classifiers.trees. J48graft 
 Get the value of binarySplits.
   getBins()   - 
Method in class weka.filters.unsupervised.attribute. Discretize 
 Gets the number of bins numeric attributes will be divided into
   getBins()   - 
Method in class weka.filters.unsupervised.attribute. PKIDiscretize 
 Ignored
   getBinSplit()   - 
Method in class weka.classifiers.trees. FT 
 Get the value of binarySplits.
   getBinValue()   - 
Method in class weka.clusterers. XMeans 
 Gets value that represents true in a new numeric attribute.
   getBooleanCols()   - 
Method in class weka.datagenerators. ClusterGenerator 
 returns the range of boolean attributes.
   getBuilder()   - 
Method in class weka.core.xml. XMLDocument 
 returns the DocumentBuilder.
   getBuildLogisticModels()   - 
Method in class weka.classifiers.functions. SMO 
 Get the value of buildLogisticModels.
   getBuildLogisticModels()   - 
Method in class weka.classifiers.mi. MISMO 
 Get the value of buildLogisticModels.
   getBuildRegressionTree()   - 
Method in class weka.classifiers.trees.m5. M5Base 
 Get the value of regressionTree.
   getC()   - 
Method in class weka.classifiers.functions. SMO 
 Get the value of C.
   getC()   - 
Method in class weka.classifiers.functions. SMOreg 
 Get the value of C.
   getC()   - 
Method in class weka.classifiers.mi. MISMO 
 Get the value of C.
   getC()   - 
Method in class weka.classifiers.mi. MISVM 
 Get the value of C.
   getCacheHits()   - 
Method in class weka.classifiers.functions.supportVector. RegOptimizer 
 return the number of kernel cache hits
   getCacheKeyName()   - 
Method in class weka.experiment. DatabaseResultListener 
 Get the value of CacheKeyName.
   getCacheSize()   - 
Method in class weka.classifiers.functions. LibSVM 
 Gets cache memory size in MB
   getCacheSize()   - 
Method in class weka.classifiers.functions.supportVector. CachedKernel 
 Gets the size of the cache
   getCacheSize()   - 
Method in class weka.classifiers.functions.supportVector. StringKernel 
 Gets the size of the cache
   getCacheValues(double)   - 
Method in class weka.classifiers.lazy.kstar. KStarCache 
 Returns the values in the cache mapped by the specified key
   getCalcOutOfBag()   - 
Method in class weka.classifiers.meta. Bagging 
 Get whether the out of bag error is calculated.
   getCalculatedNumToSelect()   - 
Method in class weka.attributeSelection. GreedyStepwise 
 Gets the calculated number of attributes to retain.
   getCalculatedNumToSelect()   - 
Method in class weka.attributeSelection. RaceSearch 
 Gets the calculated number of attributes to retain.
   getCalculatedNumToSelect()   - 
Method in interface weka.attributeSelection. RankedOutputSearch 
 Gets the calculated number of attributes to retain.
   getCalculatedNumToSelect()   - 
Method in class weka.attributeSelection. Ranker 
 Gets the calculated number to select.
   getCalculateStdDevs()   - 
Method in class weka.experiment. AveragingResultProducer 
 Get the value of CalculateStdDevs.
   getCanChangeClassInDialog()   - 
Method in class weka.gui. GenericObjectEditor 
 Returns whether the user can change the class in the dialog.
   getCapabilities()   - 
Method in class weka.associations. AbstractAssociator 
 Returns the Capabilities of this associator.
   getCapabilities()   - 
Method in class weka.associations. Apriori 
 Returns default capabilities of the classifier.
   getCapabilities()   - 
Method in interface weka.associations. Associator 
 Returns the Capabilities of this associator.
   getCapabilities()   - 
Method in class weka.associations. FilteredAssociator 
 Returns default capabilities of the associator.
   getCapabilities()   - 
Method in class weka.associations. FPGrowth 
 Returns default capabilities of the classifier.
   getCapabilities()   - 
Method in class weka.associations. GeneralizedSequentialPatterns 
 Returns the Capabilities of the algorithm.
   getCapabilities()   - 
Method in class weka.associations. PredictiveApriori 
 Returns default capabilities of the classifier.
   getCapabilities()   - 
Method in class weka.associations. SingleAssociatorEnhancer 
 Returns default capabilities of the base associator.
   getCapabilities()   - 
Method in class weka.associations. Tertius 
 Returns default capabilities of the classifier.
   getCapabilities()   - 
Method in class weka.attributeSelection. ASEvaluation 
 Returns the capabilities of this evaluator.
   getCapabilities()   - 
Method in class weka.attributeSelection. CfsSubsetEval 
 Returns the capabilities of this evaluator.
   getCapabilities()   - 
Method in class weka.attributeSelection. ChiSquaredAttributeEval 
 Returns the capabilities of this evaluator.
   getCapabilities()   - 
Method in class weka.attributeSelection. ClassifierSubsetEval 
 Returns the capabilities of this evaluator.
   getCapabilities()   - 
Method in class weka.attributeSelection. ConsistencySubsetEval 
 Returns the capabilities of this evaluator.
   getCapabilities()   - 
Method in class weka.attributeSelection. CostSensitiveASEvaluation 
 Returns default capabilities of the classifier.
   getCapabilities()   - 
Method in class weka.attributeSelection. FilteredAttributeEval 
 Returns default capabilities of the evaluator.
   getCapabilities()   - 
Method in class weka.attributeSelection. FilteredSubsetEval 
 Returns default capabilities of the evaluator.
   getCapabilities()   - 
Method in class weka.attributeSelection. GainRatioAttributeEval 
 Returns the capabilities of this evaluator.
   getCapabilities()   - 
Method in class weka.attributeSelection. InfoGainAttributeEval 
 Returns the capabilities of this evaluator.
   getCapabilities()   - 
Method in class weka.attributeSelection. LatentSemanticAnalysis 
 Returns the capabilities of this evaluator.
   getCapabilities()   - 
Method in class weka.attributeSelection. OneRAttributeEval 
 Returns the capabilities of this evaluator.
   getCapabilities()   - 
Method in class weka.attributeSelection. PrincipalComponents 
 Returns the capabilities of this evaluator.
   getCapabilities()   - 
Method in class weka.attributeSelection. ReliefFAttributeEval 
 Returns the capabilities of this evaluator.
   getCapabilities()   - 
Method in class weka.attributeSelection. SVMAttributeEval 
 Returns the capabilities of this evaluator.
   getCapabilities()   - 
Method in class weka.attributeSelection. SymmetricalUncertAttributeEval 
 Returns the capabilities of this evaluator.
   getCapabilities()   - 
Method in class weka.attributeSelection. WrapperSubsetEval 
 Returns the capabilities of this evaluator.
   getCapabilities()   - 
Method in class weka.classifiers.bayes. AODE 
 Returns default capabilities of the classifier.
   getCapabilities()   - 
Method in class weka.classifiers.bayes. AODEsr 
 Returns default capabilities of the classifier.
   getCapabilities()   - 
Method in class weka.classifiers.bayes. BayesianLogisticRegression 
 This method tests what kind of data this classifier can handle.
   getCapabilities()   - 
Method in class weka.classifiers.bayes. BayesNet 
 Returns default capabilities of the classifier.
   getCapabilities()   - 
Method in class weka.classifiers.bayes. ComplementNaiveBayes 
 Returns default capabilities of the classifier.
   getCapabilities()   - 
Method in class weka.classifiers.bayes. DMNBtext 
 Returns default capabilities of the classifier.
   getCapabilities()   - 
Method in class weka.classifiers.bayes. HNB 
 Returns default capabilities of the classifier.
   getCapabilities()   - 
Method in class weka.classifiers.bayes. NaiveBayes 
 Returns default capabilities of the classifier.
   getCapabilities()   - 
Method in class weka.classifiers.bayes. NaiveBayesMultinomial 
 Returns default capabilities of the classifier.
   getCapabilities()   - 
Method in class weka.classifiers.bayes. NaiveBayesSimple 
 Returns default capabilities of the classifier.
   getCapabilities()   - 
Method in class weka.classifiers.bayes. WAODE 
 Returns default capabilities of the classifier.
   getCapabilities()   - 
Method in class weka.classifiers. Classifier 
 Returns the Capabilities of this classifier.
   getCapabilities()   - 
Method in class weka.classifiers.functions. GaussianProcesses 
 Returns default capabilities of the classifier.
   getCapabilities()   - 
Method in class weka.classifiers.functions. IsotonicRegression 
 Returns default capabilities of the classifier.
   getCapabilities()   - 
Method in class weka.classifiers.functions. LeastMedSq 
 Returns default capabilities of the classifier.
   getCapabilities()   - 
Method in class weka.classifiers.functions. LibLINEAR 
 Returns default capabilities of the classifier.
   getCapabilities()   - 
Method in class weka.classifiers.functions. LibSVM 
 Returns default capabilities of the classifier.
   getCapabilities()   - 
Method in class weka.classifiers.functions. LinearRegression 
 Returns default capabilities of the classifier.
   getCapabilities()   - 
Method in class weka.classifiers.functions. Logistic 
 Returns default capabilities of the classifier.
   getCapabilities()   - 
Method in class weka.classifiers.functions. MultilayerPerceptron 
 Returns default capabilities of the classifier.
   getCapabilities()   - 
Method in class weka.classifiers.functions. PaceRegression 
 Returns default capabilities of the classifier.
   getCapabilities()   - 
Method in class weka.classifiers.functions. PLSClassifier 
 Returns default capabilities of the classifier.
   getCapabilities()   - 
Method in class weka.classifiers.functions. RBFNetwork 
 Returns default capabilities of the classifier, i.e.,  and "or" of
 Logistic and LinearRegression.
   getCapabilities()   - 
Method in class weka.classifiers.functions. SimpleLinearRegression 
 Returns default capabilities of the classifier.
   getCapabilities()   - 
Method in class weka.classifiers.functions. SimpleLogistic 
 Returns default capabilities of the classifier.
   getCapabilities()   - 
Method in class weka.classifiers.functions. SMO 
 Returns default capabilities of the classifier.
   getCapabilities()   - 
Method in class weka.classifiers.functions. SMOreg 
 Returns default capabilities of the classifier.
   getCapabilities()   - 
Method in class weka.classifiers.functions. SPegasos 
 Returns default capabilities of the classifier.
   getCapabilities()   - 
Method in class weka.classifiers.functions.supportVector. Kernel 
 Returns the Capabilities of this kernel.
   getCapabilities()   - 
Method in class weka.classifiers.functions.supportVector. PolyKernel 
 Returns the Capabilities of this kernel.
   getCapabilities()   - 
Method in class weka.classifiers.functions.supportVector. PrecomputedKernelMatrixKernel 
 Returns the Capabilities of this kernel.
   getCapabilities()   - 
Method in class weka.classifiers.functions.supportVector. Puk 
 Returns the Capabilities of this kernel.
   getCapabilities()   - 
Method in class weka.classifiers.functions.supportVector. RBFKernel 
 Returns the Capabilities of this kernel.
   getCapabilities()   - 
Method in class weka.classifiers.functions.supportVector. StringKernel 
 Returns the Capabilities of this kernel.
   getCapabilities()   - 
Method in class weka.classifiers.functions. VotedPerceptron 
 Returns default capabilities of the classifier.
   getCapabilities()   - 
Method in class weka.classifiers.functions. Winnow 
 Returns default capabilities of the classifier.
   getCapabilities()   - 
Method in class weka.classifiers.lazy. IB1 
 Returns default capabilities of the classifier.
   getCapabilities()   - 
Method in class weka.classifiers.lazy. IBk 
 Returns default capabilities of the classifier.
   getCapabilities()   - 
Method in class weka.classifiers.lazy. KStar 
 Returns default capabilities of the classifier.
   getCapabilities()   - 
Method in class weka.classifiers.lazy. LBR 
 Returns default capabilities of the classifier.
   getCapabilities()   - 
Method in class weka.classifiers.lazy. LWL 
 Returns default capabilities of the classifier.
   getCapabilities()   - 
Method in class weka.classifiers.meta. AdaBoostM1 
 Returns default capabilities of the classifier.
   getCapabilities()   - 
Method in class weka.classifiers.meta. AdditiveRegression 
 Returns default capabilities of the classifier.
   getCapabilities()   - 
Method in class weka.classifiers.meta. AttributeSelectedClassifier 
 Returns default capabilities of the classifier.
   getCapabilities()   - 
Method in class weka.classifiers.meta. ClassificationViaClustering 
 Returns default capabilities of the classifier.
   getCapabilities()   - 
Method in class weka.classifiers.meta. ClassificationViaRegression 
 Returns default capabilities of the classifier.
   getCapabilities()   - 
Method in class weka.classifiers.meta. CostSensitiveClassifier 
 Returns default capabilities of the classifier.
   getCapabilities()   - 
Method in class weka.classifiers.meta. CVParameterSelection 
 Returns default capabilities of the classifier.
   getCapabilities()   - 
Method in class weka.classifiers.meta. Decorate 
 Returns default capabilities of the classifier.
   getCapabilities()   - 
Method in class weka.classifiers.meta. END 
 Returns default capabilities of the classifier.
   getCapabilities()   - 
Method in class weka.classifiers.meta. FilteredClassifier 
 Returns default capabilities of the classifier.
   getCapabilities()   - 
Method in class weka.classifiers.meta. GridSearch 
 Returns default capabilities of the classifier.
   getCapabilities()   - 
Method in class weka.classifiers.meta. LogitBoost 
 Returns default capabilities of the classifier.
   getCapabilities()   - 
Method in class weka.classifiers.meta. MetaCost 
 Returns default capabilities of the classifier.
   getCapabilities()   - 
Method in class weka.classifiers.meta. MultiClassClassifier 
 Returns default capabilities of the classifier.
   getCapabilities()   - 
Method in class weka.classifiers.meta.nestedDichotomies. ClassBalancedND 
 Returns default capabilities of the classifier.
   getCapabilities()   - 
Method in class weka.classifiers.meta.nestedDichotomies. DataNearBalancedND 
 Returns default capabilities of the classifier.
   getCapabilities()   - 
Method in class weka.classifiers.meta.nestedDichotomies. ND 
 Returns default capabilities of the classifier.
   getCapabilities()   - 
Method in class weka.classifiers.meta. OrdinalClassClassifier 
 Returns default capabilities of the classifier.
   getCapabilities()   - 
Method in class weka.classifiers.meta. RacedIncrementalLogitBoost 
 Returns default capabilities of the classifier.
   getCapabilities()   - 
Method in class weka.classifiers.meta. RegressionByDiscretization 
 Returns default capabilities of the classifier.
   getCapabilities()   - 
Method in class weka.classifiers.meta. Stacking 
 Returns combined capabilities of the base classifiers, i.e., the
 capabilities all of them have in common.
   getCapabilities()   - 
Method in class weka.classifiers.meta. ThresholdSelector 
 Returns default capabilities of the classifier.
   getCapabilities()   - 
Method in class weka.classifiers.meta. Vote 
 Returns default capabilities of the classifier.
   getCapabilities()   - 
Method in class weka.classifiers.mi. CitationKNN 
 Returns default capabilities of the classifier.
   getCapabilities()   - 
Method in class weka.classifiers.mi. MDD 
 Returns default capabilities of the classifier.
   getCapabilities()   - 
Method in class weka.classifiers.mi. MIBoost 
 Returns default capabilities of the classifier.
   getCapabilities()   - 
Method in class weka.classifiers.mi. MIDD 
 Returns default capabilities of the classifier.
   getCapabilities()   - 
Method in class weka.classifiers.mi. MIEMDD 
 Returns default capabilities of the classifier.
   getCapabilities()   - 
Method in class weka.classifiers.mi. MILR 
 Returns default capabilities of the classifier.
   getCapabilities()   - 
Method in class weka.classifiers.mi. MINND 
 Returns default capabilities of the classifier.
   getCapabilities()   - 
Method in class weka.classifiers.mi. MIOptimalBall 
 Returns default capabilities of the classifier.
   getCapabilities()   - 
Method in class weka.classifiers.mi. MISMO 
 Returns default capabilities of the classifier.
   getCapabilities()   - 
Method in class weka.classifiers.mi. MISVM 
 Returns default capabilities of the classifier.
   getCapabilities()   - 
Method in class weka.classifiers.mi. MIWrapper 
 Returns default capabilities of the classifier.
   getCapabilities()   - 
Method in class weka.classifiers.mi. SimpleMI 
 Returns default capabilities of the classifier.
   getCapabilities()   - 
Method in class weka.classifiers.mi.supportVector. MIPolyKernel 
 Returns the Capabilities of this kernel.
   getCapabilities()   - 
Method in class weka.classifiers.mi.supportVector. MIRBFKernel 
 Returns the Capabilities of this kernel.
   getCapabilities()   - 
Method in class weka.classifiers.misc. HyperPipes 
 Returns default capabilities of the classifier.
   getCapabilities()   - 
Method in class weka.classifiers.misc. SerializedClassifier 
 Returns default capabilities of the base classifier.
   getCapabilities()   - 
Method in class weka.classifiers.misc. VFI 
 Returns default capabilities of the classifier.
   getCapabilities()   - 
Method in class weka.classifiers. MultipleClassifiersCombiner 
 Returns combined capabilities of the base classifiers, i.e., the
 capabilities all of them have in common.
   getCapabilities()   - 
Method in class weka.classifiers.rules. ConjunctiveRule 
 Returns default capabilities of the classifier.
   getCapabilities()   - 
Method in class weka.classifiers.rules. DecisionTable 
 Returns default capabilities of the classifier.
   getCapabilities()   - 
Method in class weka.classifiers.rules. DTNB 
 Returns default capabilities of the classifier.
   getCapabilities()   - 
Method in class weka.classifiers.rules. JRip 
 Returns default capabilities of the classifier.
   getCapabilities()   - 
Method in class weka.classifiers.rules. NNge 
 Returns default capabilities of the classifier.
   getCapabilities()   - 
Method in class weka.classifiers.rules. OneR 
 Returns default capabilities of the classifier.
   getCapabilities()   - 
Method in class weka.classifiers.rules. PART 
 Returns default capabilities of the classifier.
   getCapabilities()   - 
Method in class weka.classifiers.rules.part. MakeDecList 
 Returns default capabilities of the classifier.
   getCapabilities()   - 
Method in class weka.classifiers.rules. Prism 
 Returns default capabilities of the classifier.
   getCapabilities()   - 
Method in class weka.classifiers.rules. Ridor 
 Returns default capabilities of the classifier.
   getCapabilities()   - 
Method in class weka.classifiers.rules. ZeroR 
 Returns default capabilities of the classifier.
   getCapabilities()   - 
Method in class weka.classifiers. SingleClassifierEnhancer 
 Returns default capabilities of the base classifier.
   getCapabilities()   - 
Method in class weka.classifiers.trees. ADTree 
 Returns default capabilities of the classifier.
   getCapabilities()   - 
Method in class weka.classifiers.trees. BFTree 
 Returns default capabilities of the classifier.
   getCapabilities()   - 
Method in class weka.classifiers.trees. DecisionStump 
 Returns default capabilities of the classifier.
   getCapabilities()   - 
Method in class weka.classifiers.trees. FT 
 Returns default capabilities of the classifier.
   getCapabilities()   - 
Method in class weka.classifiers.trees. Id3 
 Returns default capabilities of the classifier.
   getCapabilities()   - 
Method in class weka.classifiers.trees.j48. C45PruneableClassifierTree 
 Returns default capabilities of the classifier tree.
   getCapabilities()   - 
Method in class weka.classifiers.trees.j48. C45PruneableClassifierTreeG 
 Returns default capabilities of the classifier tree.
   getCapabilities()   - 
Method in class weka.classifiers.trees.j48. ClassifierTree 
 Returns default capabilities of the classifier tree.
   getCapabilities()   - 
Method in class weka.classifiers.trees. J48 
 Returns default capabilities of the classifier.
   getCapabilities()   - 
Method in class weka.classifiers.trees.j48. NBTreeClassifierTree 
 Returns default capabilities of the classifier tree.
   getCapabilities()   - 
Method in class weka.classifiers.trees.j48. PruneableClassifierTree 
 Returns default capabilities of the classifier tree.
   getCapabilities()   - 
Method in class weka.classifiers.trees. J48graft 
 Returns default capabilities of the classifier.
   getCapabilities()   - 
Method in class weka.classifiers.trees. LADTree 
 Returns default capabilities of the classifier.
   getCapabilities()   - 
Method in class weka.classifiers.trees. LMT 
 Returns default capabilities of the classifier.
   getCapabilities()   - 
Method in class weka.classifiers.trees.m5. M5Base 
 Returns default capabilities of the classifier, i.e., of LinearRegression.
   getCapabilities()   - 
Method in class weka.classifiers.trees. NBTree 
 Returns default capabilities of the classifier.
   getCapabilities()   - 
Method in class weka.classifiers.trees. RandomForest 
 Returns default capabilities of the classifier.
   getCapabilities()   - 
Method in class weka.classifiers.trees. RandomTree 
 Returns default capabilities of the classifier.
   getCapabilities()   - 
Method in class weka.classifiers.trees. REPTree 
 Returns default capabilities of the classifier.
   getCapabilities()   - 
Method in class weka.classifiers.trees. SimpleCart 
 Returns default capabilities of the classifier.
   getCapabilities()   - 
Method in class weka.classifiers.trees. UserClassifier 
 Returns default capabilities of the classifier.
   getCapabilities()   - 
Method in class weka.clusterers. AbstractClusterer 
 Returns the Capabilities of this clusterer.
   getCapabilities()   - 
Method in class weka.clusterers. CLOPE 
 Returns default capabilities of the clusterer.
   getCapabilities()   - 
Method in interface weka.clusterers. Clusterer 
 Returns the Capabilities of this clusterer.
   getCapabilities()   - 
Method in class weka.clusterers. Cobweb 
 Returns default capabilities of the clusterer.
   getCapabilities()   - 
Method in class weka.clusterers. DBScan 
 Returns default capabilities of the clusterer.
   getCapabilities()   - 
Method in class weka.clusterers. EM 
 Returns default capabilities of the clusterer (i.e., the ones of 
 SimpleKMeans).
   getCapabilities()   - 
Method in class weka.clusterers. FarthestFirst 
 Returns default capabilities of the clusterer.
   getCapabilities()   - 
Method in class weka.clusterers. FilteredClusterer 
 Returns default capabilities of the clusterer.
   getCapabilities()   - 
Method in class weka.clusterers. HierarchicalClusterer 
  
   getCapabilities()   - 
Method in class weka.clusterers. MakeDensityBasedClusterer 
 Returns default capabilities of the clusterer (i.e., of the wrapper
 clusterer).
   getCapabilities()   - 
Method in class weka.clusterers. OPTICS 
 Returns default capabilities of the clusterer.
   getCapabilities()   - 
Method in class weka.clusterers. sIB 
 Returns default capabilities of the clusterer.
   getCapabilities()   - 
Method in class weka.clusterers. SimpleKMeans 
 Returns default capabilities of the clusterer.
   getCapabilities()   - 
Method in class weka.clusterers. SingleClustererEnhancer 
 Returns default capabilities of the clusterer.
   getCapabilities()   - 
Method in class weka.clusterers. XMeans 
 Returns default capabilities of the clusterer.
   getCapabilities()   - 
Method in interface weka.core. CapabilitiesHandler 
 Returns the capabilities of this object.
   getCapabilities()   - 
Method in class weka.core.converters. AbstractSaver 
 Returns the Capabilities of this saver.
   getCapabilities()   - 
Method in class weka.core.converters. ArffSaver 
 Returns the Capabilities of this saver.
   getCapabilities()   - 
Method in class weka.core.converters. C45Saver 
 Returns the Capabilities of this saver.
   getCapabilities()   - 
Method in class weka.core.converters. CSVSaver 
 Returns the Capabilities of this saver.
   getCapabilities()   - 
Method in class weka.core.converters. DatabaseSaver 
 Returns the Capabilities of this saver.
   getCapabilities()   - 
Method in class weka.core.converters. LibSVMSaver 
 Returns the Capabilities of this saver.
   getCapabilities()   - 
Method in class weka.core.converters. SerializedInstancesSaver 
 Returns the Capabilities of this saver.
   getCapabilities()   - 
Method in class weka.core.converters. SVMLightSaver 
 Returns the Capabilities of this saver.
   getCapabilities()   - 
Method in class weka.core.converters. XRFFSaver 
 Returns the Capabilities of this saver.
   getCapabilities()   - 
Method in class weka.core. FindWithCapabilities 
 The capabilities to search for.
   getCapabilities()   - 
Method in class weka.estimators. DiscreteEstimator 
 Returns default capabilities of the classifier.
   getCapabilities()   - 
Method in class weka.estimators. Estimator 
 Returns the Capabilities of this Estimator.
   getCapabilities()   - 
Method in class weka.estimators. KernelEstimator 
 Returns default capabilities of the classifier.
   getCapabilities()   - 
Method in class weka.estimators. MahalanobisEstimator 
 Returns default capabilities of the classifier.
   getCapabilities()   - 
Method in class weka.estimators. NormalEstimator 
 Returns default capabilities of the classifier.
   getCapabilities()   - 
Method in class weka.estimators. PoissonEstimator 
 Returns default capabilities of the classifier.
   getCapabilities()   - 
Method in class weka.filters. AllFilter 
 Returns the Capabilities of this filter.
   getCapabilities()   - 
Method in class weka.filters. Filter 
 Returns the Capabilities of this filter.
   getCapabilities(Instances)   - 
Method in class weka.filters. Filter 
 Returns the Capabilities of this filter, customized based on the data.
   getCapabilities()   - 
Method in class weka.filters. MultiFilter 
 Returns the Capabilities of this filter.
   getCapabilities()   - 
Method in class weka.filters.supervised.attribute. AddClassification 
 Returns the Capabilities of this filter.
   getCapabilities()   - 
Method in class weka.filters.supervised.attribute. AttributeSelection 
 Returns the Capabilities of this filter.
   getCapabilities()   - 
Method in class weka.filters.supervised.attribute. ClassOrder 
 Returns the Capabilities of this filter.
   getCapabilities()   - 
Method in class weka.filters.supervised.attribute. Discretize 
 Returns the Capabilities of this filter.
   getCapabilities()   - 
Method in class weka.filters.supervised.attribute. NominalToBinary 
 Returns the Capabilities of this filter.
   getCapabilities()   - 
Method in class weka.filters.supervised.attribute. PLSFilter 
 Returns the Capabilities of this filter.
   getCapabilities()   - 
Method in class weka.filters.supervised.instance. Resample 
 Returns the Capabilities of this filter.
   getCapabilities()   - 
Method in class weka.filters.supervised.instance. SMOTE 
 Returns the Capabilities of this filter.
   getCapabilities()   - 
Method in class weka.filters.supervised.instance. SpreadSubsample 
 Returns the Capabilities of this filter.
   getCapabilities()   - 
Method in class weka.filters.supervised.instance. StratifiedRemoveFolds 
 Returns the Capabilities of this filter.
   getCapabilities()   - 
Method in class weka.filters.unsupervised.attribute. Add 
 Returns the Capabilities of this filter.
   getCapabilities(Instances)   - 
Method in class weka.filters.unsupervised.attribute. AddCluster 
 Returns the Capabilities of this filter, makes sure that the class is
 never set (for the clusterer).
   getCapabilities()   - 
Method in class weka.filters.unsupervised.attribute. AddCluster 
 Returns the Capabilities of this filter.
   getCapabilities()   - 
Method in class weka.filters.unsupervised.attribute. AddExpression 
 Returns the Capabilities of this filter.
   getCapabilities()   - 
Method in class weka.filters.unsupervised.attribute. AddID 
 Returns the Capabilities of this filter.
   getCapabilities()   - 
Method in class weka.filters.unsupervised.attribute. AddNoise 
 Returns the Capabilities of this filter.
   getCapabilities()   - 
Method in class weka.filters.unsupervised.attribute. AddValues 
 Returns the Capabilities of this filter.
   getCapabilities()   - 
Method in class weka.filters.unsupervised.attribute. Center 
 Returns the Capabilities of this filter.
   getCapabilities()   - 
Method in class weka.filters.unsupervised.attribute. ChangeDateFormat 
 Returns the Capabilities of this filter.
   getCapabilities()   - 
Method in class weka.filters.unsupervised.attribute. ClassAssigner 
 Returns the Capabilities of this filter.
   getCapabilities()   - 
Method in class weka.filters.unsupervised.attribute. ClusterMembership 
 Returns the Capabilities of this filter.
   getCapabilities(Instances)   - 
Method in class weka.filters.unsupervised.attribute. ClusterMembership 
 Returns the Capabilities of this filter, makes sure that the class is
 never set (for the clusterer).
   getCapabilities()   - 
Method in class weka.filters.unsupervised.attribute. Copy 
 Returns the Capabilities of this filter.
   getCapabilities()   - 
Method in class weka.filters.unsupervised.attribute. Discretize 
 Returns the Capabilities of this filter.
   getCapabilities()   - 
Method in class weka.filters.unsupervised.attribute. FirstOrder 
 Returns the Capabilities of this filter.
   getCapabilities()   - 
Method in class weka.filters.unsupervised.attribute. InterquartileRange 
 Returns the Capabilities of this filter.
   getCapabilities()   - 
Method in class weka.filters.unsupervised.attribute. KernelFilter 
 Returns the Capabilities of this filter.
   getCapabilities()   - 
Method in class weka.filters.unsupervised.attribute. MakeIndicator 
 Returns the Capabilities of this filter.
   getCapabilities()   - 
Method in class weka.filters.unsupervised.attribute. MathExpression 
 Returns the Capabilities of this filter.
   getCapabilities()   - 
Method in class weka.filters.unsupervised.attribute. MergeTwoValues 
 Returns the Capabilities of this filter.
   getCapabilities()   - 
Method in class weka.filters.unsupervised.attribute. MultiInstanceToPropositional 
 Returns the Capabilities of this filter.
   getCapabilities()   - 
Method in class weka.filters.unsupervised.attribute. NominalToBinary 
 Returns the Capabilities of this filter.
   getCapabilities()   - 
Method in class weka.filters.unsupervised.attribute. NominalToString 
 Returns the Capabilities of this filter.
   getCapabilities()   - 
Method in class weka.filters.unsupervised.attribute. Normalize 
 Returns the Capabilities of this filter.
   getCapabilities()   - 
Method in class weka.filters.unsupervised.attribute. NumericCleaner 
 Returns the Capabilities of this filter.
   getCapabilities()   - 
Method in class weka.filters.unsupervised.attribute. NumericToBinary 
 Returns the Capabilities of this filter.
   getCapabilities()   - 
Method in class weka.filters.unsupervised.attribute. NumericToNominal 
 Returns the Capabilities of this filter.
   getCapabilities()   - 
Method in class weka.filters.unsupervised.attribute. NumericTransform 
 Returns the Capabilities of this filter.
   getCapabilities()   - 
Method in class weka.filters.unsupervised.attribute. Obfuscate 
 Returns the Capabilities of this filter.
   getCapabilities()   - 
Method in class weka.filters.unsupervised.attribute. PrincipalComponents 
 Returns the capabilities of this evaluator.
   getCapabilities()   - 
Method in class weka.filters.unsupervised.attribute. PropositionalToMultiInstance 
 Returns the Capabilities of this filter.
   getCapabilities()   - 
Method in class weka.filters.unsupervised.attribute. RandomProjection 
 Returns the Capabilities of this filter.
   getCapabilities()   - 
Method in class weka.filters.unsupervised.attribute. RandomSubset 
 Returns the Capabilities of this filter.
   getCapabilities()   - 
Method in class weka.filters.unsupervised.attribute. RELAGGS 
 Returns the Capabilities of this filter.
   getCapabilities()   - 
Method in class weka.filters.unsupervised.attribute. Remove 
 Returns the Capabilities of this filter.
   getCapabilities()   - 
Method in class weka.filters.unsupervised.attribute. RemoveType 
 Returns the Capabilities of this filter.
   getCapabilities()   - 
Method in class weka.filters.unsupervised.attribute. RemoveUseless 
 Returns the Capabilities of this filter.
   getCapabilities()   - 
Method in class weka.filters.unsupervised.attribute. Reorder 
 Returns the Capabilities of this filter.
   getCapabilities()   - 
Method in class weka.filters.unsupervised.attribute. ReplaceMissingValues 
 Returns the Capabilities of this filter.
   getCapabilities()   - 
Method in class weka.filters.unsupervised.attribute. Standardize 
 Returns the Capabilities of this filter.
   getCapabilities()   - 
Method in class weka.filters.unsupervised.attribute. StringToNominal 
 Returns the Capabilities of this filter.
   getCapabilities()   - 
Method in class weka.filters.unsupervised.attribute. StringToWordVector 
 Returns the Capabilities of this filter.
   getCapabilities()   - 
Method in class weka.filters.unsupervised.attribute. SwapValues 
 Returns the Capabilities of this filter.
   getCapabilities()   - 
Method in class weka.filters.unsupervised.attribute. TimeSeriesDelta 
 Returns the Capabilities of this filter.
   getCapabilities()   - 
Method in class weka.filters.unsupervised.attribute. TimeSeriesTranslate 
 Returns the Capabilities of this filter.
   getCapabilities()   - 
Method in class weka.filters.unsupervised.attribute. Wavelet 
 Returns the Capabilities of this filter.
   getCapabilities()   - 
Method in class weka.filters.unsupervised.instance. NonSparseToSparse 
 Returns the Capabilities of this filter.
   getCapabilities()   - 
Method in class weka.filters.unsupervised.instance. Normalize 
 Returns the Capabilities of this filter.
   getCapabilities()   - 
Method in class weka.filters.unsupervised.instance. Randomize 
 Returns the Capabilities of this filter.
   getCapabilities()   - 
Method in class weka.filters.unsupervised.instance. RemoveFolds 
 Returns the Capabilities of this filter.
   getCapabilities()   - 
Method in class weka.filters.unsupervised.instance. RemoveFrequentValues 
 Returns the Capabilities of this filter.
   getCapabilities()   - 
Method in class weka.filters.unsupervised.instance. RemoveMisclassified 
 Returns the Capabilities of this filter.
   getCapabilities()   - 
Method in class weka.filters.unsupervised.instance. RemovePercentage 
 Returns the Capabilities of this filter.
   getCapabilities()   - 
Method in class weka.filters.unsupervised.instance. RemoveRange 
 Returns the Capabilities of this filter.
   getCapabilities()   - 
Method in class weka.filters.unsupervised.instance. RemoveWithValues 
 Returns the Capabilities of this filter.
   getCapabilities()   - 
Method in class weka.filters.unsupervised.instance. Resample 
 Returns the Capabilities of this filter.
   getCapabilities()   - 
Method in class weka.filters.unsupervised.instance. ReservoirSample 
 Returns the Capabilities of this filter.
   getCapabilities()   - 
Method in class weka.filters.unsupervised.instance. SparseToNonSparse 
 Returns the Capabilities of this filter.
   getCapabilities()   - 
Method in class weka.filters.unsupervised.instance. SubsetByExpression 
 Returns the Capabilities of this filter.
   getCapabilities()   - 
Method in class weka.gui. GenericObjectEditor.CapabilitiesFilterDialog 
 returns the currently selected capabilities.
   getCapabilitiesFilter()   - 
Method in class weka.gui. ConverterFileChooser 
 returns the capabilities filter for the savers, can be null if all are
 listed.
   getCapabilitiesFilter()   - 
Method in class weka.gui. GenericObjectEditor 
 Returns the current Capabilities filter, can be null.
   getCar()   - 
Method in class weka.associations. Apriori 
 Gets whether class association ruels are mined
   getCar()   - 
Method in class weka.associations. PredictiveApriori 
 Gets whether class association ruels are mined
   getCardinality(int)   - 
Method in class weka.classifiers.bayes. BayesNet 
 get number of values a node can take
   getCardinality()   - 
Method in class weka.datagenerators.classifiers.classification. BayesNet 
 Gets the cardinality of the attributes (incl class attribute)
   getCardinalityOfParents()   - 
Method in class weka.classifiers.bayes.net. ParentSet 
 returns cardinality of parents
   getCell(int, int)   - 
Method in class weka.classifiers. CostMatrix 
 Return the contents of a particular cell.
   getCellEditor(int, int)   - 
Method in class weka.gui.arffviewer. ArffTable 
 returns the cell editor for the given cell
   getCells()   - 
Method in class weka.gui.sql. ResultSetHelper 
 returns an 2-dimensional array with the content of the resultset, the first
 dimension is the row, the second the column (i.e., getCells()[y][x]).
   getCenter()   - 
Method in class weka.gui.treevisualizer. Node 
 Get the value of center.
   getCenterData()   - 
Method in class weka.attributeSelection. PrincipalComponents 
 Get whether to center (rather than standardize)
 the data.
   getCenterData()   - 
Method in class weka.filters.unsupervised.attribute. PrincipalComponents 
 Get whether to center (rather than standardize)
 the data.
   getChangeInWeights()   - 
Method in class weka.classifiers.functions.neural. NeuralNode 
 call this function to get the chnage in weights array.
   getChar()   - 
Method in class weka.core. Trie.TrieNode 
 returns the stored character
   getCharSet()   - 
Method in class weka.core.converters. TextDirectoryLoader 
 Get the character set to use when reading text files.
   getChecked(int)   - 
Method in class weka.gui. CheckBoxList.CheckBoxListModel 
 returns the checked state of the element at the given index
   getChecked(int)   - 
Method in class weka.gui. CheckBoxList 
 returns the checked state of the element at the given index
   getCheckedIndices()   - 
Method in class weka.gui. CheckBoxList 
 returns an array with the indices of all checked items
   getCheckErrorRate()   - 
Method in class weka.classifiers.rules. JRip 
 Gets whether to check for error rate is in stopping criterion
   getChecksTurnedOff()   - 
Method in class weka.classifiers.functions. SMO 
 Returns whether the checks are turned off or not.
   getChecksTurnedOff()   - 
Method in class weka.classifiers.functions.supportVector. Kernel 
 Returns whether the checks are turned off or not.
   getChecksTurnedOff()   - 
Method in class weka.classifiers.mi. MISMO 
 Returns whether the checks are turned off or not.
   getChecksTurnedOff()   - 
Method in class weka.filters.unsupervised.attribute. KernelFilter 
 Returns whether the checks are turned off or not.
   getChild(int)   - 
Method in class weka.gui.treevisualizer. Node 
 Get the Edge for the child number 'i'.
   getChildForBranch(int)   - 
Method in class weka.classifiers.trees.adtree. Splitter 
 Gets the child for a branch of the split.
   getChildForBranch(int)   - 
Method in class weka.classifiers.trees.adtree. TwoWayNominalSplit 
 Gets the child for a branch of the split.
   getChildForBranch(int)   - 
Method in class weka.classifiers.trees.adtree. TwoWayNumericSplit 
 Gets the child for a branch of the split.
   getChildren(int)   - 
Method in class weka.classifiers.bayes.net. EditableBayesNet 
 return list of children of a node
   getChildren()   - 
Method in class weka.classifiers.trees.adtree. PredictionNode 
 Gets the children of this node.
   getChildTags(Node)   - 
Static method in class weka.core.xml. XMLDocument 
 returns all non tag-children from the given node.
   getChildTags(Node, String)   - 
Static method in class weka.core.xml. XMLDocument 
 returns all non tag-children from the given node.
   getChooseClassPopupMenu()   - 
Method in class weka.gui. GenericObjectEditor 
 Returns a popup menu that allows the user to change
 the class of object.
   getCindex()   - 
Method in class weka.gui.visualize. PlotData2D 
 Get the currently set colouring index of the data
   getCIndex()   - 
Method in class weka.gui.visualize. VisualizePanel 
 Get the index of the attribute selected for coloring
   getClassAttribute()   - 
Method in class weka.gui.beans. ThresholdDataEvent 
 Return the class attribute for which the threshold data was generated
 for.
   getClassCapabilities()   - 
Method in class weka.core. Capabilities 
 returns all class capabilities
   getClassColumn()   - 
Method in class weka.gui.beans. ClassAssigner 
  
   getClassCounts()   - 
Method in class weka.filters.supervised.attribute. ClassOrder 
 Get the class distribution of the sorted class values.
   getClassesToClusters()   - 
Method in class weka.clusterers. ClusterEvaluation 
 Return the array (ordered by cluster number) of minimum error class to
 cluster mappings
   getClassFlag()   - 
Method in class weka.datagenerators. ClusterGenerator 
 Gets the class flag.
   getClassForIRStatistics()   - 
Method in class weka.experiment. ClassifierSplitEvaluator 
 Get the value of ClassForIRStatistics.
   getClassification()   - 
Method in class weka.associations. Tertius 
 Get the value of classification.
   getClassifier()   - 
Method in class weka.attributeSelection. ClassifierSubsetEval 
 Get the classifier used as the base learner.
   getClassifier()   - 
Method in class weka.attributeSelection. WrapperSubsetEval 
 Get the classifier used as the base learner.
   getClassifier()   - 
Method in class weka.classifiers. BVDecompose 
 Gets the name of the classifier being analysed
   getClassifier()   - 
Method in class weka.classifiers. BVDecomposeSegCVSub 
 Gets the name of the classifier being analysed
   getClassifier()   - 
Method in class weka.classifiers. CheckClassifier 
 Get the classifier used as the classifier
   getClassifier()   - 
Method in class weka.classifiers. CheckSource 
 Gets the classifier being used for the tests, can be null.
   getClassifier(int)   - 
Method in class weka.classifiers.meta. MultiScheme 
 Gets a single classifier from the set of available classifiers.
   getClassifier(int)   - 
Method in class weka.classifiers. MultipleClassifiersCombiner 
 Gets a single classifier from the set of available classifiers.
   getClassifier()   - 
Method in class weka.classifiers. SingleClassifierEnhancer 
 Get the classifier used as the base learner.
   getClassifier()   - 
Method in class weka.experiment. ClassifierSplitEvaluator 
 Get the value of Classifier.
   getClassifier()   - 
Method in class weka.experiment. RegressionSplitEvaluator 
 Get the value of Classifier.
   getClassifier()   - 
Method in class weka.filters.supervised.attribute. AddClassification 
 Gets the classifier used by the filter.
   getClassifier()   - 
Method in class weka.filters.unsupervised.instance. RemoveMisclassified 
 Gets the classifier used by the filter.
   getClassifier()   - 
Method in class weka.gui.beans. BatchClassifierEvent 
 Get the classifier
   getClassifier()   - 
Method in class weka.gui.beans. Classifier 
 Get the classifier currently set for this wrapper
   getClassifier()   - 
Method in class weka.gui.beans. IncrementalClassifierEvent 
 Get the classifier
   getClassifier()   - 
Static method in class weka.gui.explorer. ExplorerDefaults 
 returns the default classifier (fully configured) for the classify panel.
   getClassifierCostSensitiveEval()   - 
Static method in class weka.gui.explorer. ExplorerDefaults 
 returns whether the evaluation of the classifier is done cost-sensitively.
   getClassifierCrossvalidationFolds()   - 
Static method in class weka.gui.explorer. ExplorerDefaults 
 returns the default number of folds of the CV in the classify panel.
   getClassifierOutputAdditionalAttributes()   - 
Static method in class weka.gui.explorer. ExplorerDefaults 
 returns the string with the additional indices to output alongside the
 predictions.
   getClassifierOutputConfusionMatrix()   - 
Static method in class weka.gui.explorer. ExplorerDefaults 
 returns whether the confusion matrix for the classifier is output.
   getClassifierOutputEntropyEvalMeasures()   - 
Static method in class weka.gui.explorer. ExplorerDefaults 
 returns whether entropy-based evaluation meastures of the classifier 
 are output.
   getClassifierOutputModel()   - 
Static method in class weka.gui.explorer. ExplorerDefaults 
 returns whether the built model is output.
   getClassifierOutputPerClassStats()   - 
Static method in class weka.gui.explorer. ExplorerDefaults 
 returns whether additional per-class stats of the classifier are output.
   getClassifierOutputPredictions()   - 
Static method in class weka.gui.explorer. ExplorerDefaults 
 returns whether the predictions of the classifier are output as well.
   getClassifierOutputSourceCode()   - 
Static method in class weka.gui.explorer. ExplorerDefaults 
 returns whether the source of a sourcable Classifier is output
 in the classify tab.
   getClassifierPercentageSplit()   - 
Static method in class weka.gui.explorer. ExplorerDefaults 
 returns the default classifier test mode for the classify panel (0-99).
   getClassifierPreserveOrder()   - 
Static method in class weka.gui.explorer. ExplorerDefaults 
 returns whether the order is preserved in case of the percentage split 
 in the classify tab.
   getClassifierRandomSeed()   - 
Static method in class weka.gui.explorer. ExplorerDefaults 
 returns the default random seed value for the classifier for the 
 classify panel.
   getClassifiers()   - 
Method in class weka.classifiers.meta. MultiScheme 
 Gets the list of possible classifers to choose from.
   getClassifiers()   - 
Method in class weka.classifiers. MultipleClassifiersCombiner 
 Gets the list of possible classifers to choose from.
   getClassifierSourceCodeClass()   - 
Static method in class weka.gui.explorer. ExplorerDefaults 
 returns the default classname for a sourcable Classifier in the classify tab.
   getClassifierStorePredictionsForVis()   - 
Static method in class weka.gui.explorer. ExplorerDefaults 
 returns whether the predictions of the classifier are stored for 
 visualization.
   getClassifierTemplate()   - 
Method in class weka.gui.beans. Classifier 
 Return the classifier template currently in use.
   getClassifierTestMode()   - 
Static method in class weka.gui.explorer. ExplorerDefaults 
 returns the default classifier test mode for the classify panel.
   getClassifyIterations()   - 
Method in class weka.classifiers. BVDecomposeSegCVSub 
 Gets the number of times an instance is classified
   getClassIndex()   - 
Method in class weka.associations. Apriori 
 Gets the class index
   getClassIndex()   - 
Method in class weka.associations. FilteredAssociator 
 Gets the class index
   getClassIndex()   - 
Method in class weka.associations. PredictiveApriori 
 Gets the index of the class attribute
   getClassIndex()   - 
Method in class weka.associations. Tertius 
 Get the value of classIndex.
   getClassIndex()   - 
Method in class weka.classifiers. BVDecompose 
 Get the index (starting from 1) of the attribute used as the class.
   getClassIndex()   - 
Method in class weka.classifiers. BVDecomposeSegCVSub 
 Get the index (starting from 1) of the attribute used as the class.
   getClassIndex()   - 
Method in class weka.classifiers. CheckSource 
 Gets the class index of the dataset.
   getClassIndex()   - 
Method in class weka.core.converters. LibSVMSaver 
 Get the index of the class attribute.
   getClassIndex()   - 
Method in class weka.core.converters. SVMLightSaver 
 Get the index of the class attribute.
   getClassIndex()   - 
Method in class weka.core.converters. XRFFSaver 
 Get the index of the class attribute.
   getClassIndex()   - 
Method in class weka.core. FindWithCapabilities 
 returns the current current class index, -1 if no class attribute.
   getClassIndex()   - 
Method in class weka.core. TestInstances 
 returns the current class index (0-based), -1 is last attribute
   getClassIndex()   - 
Method in class weka.filters. CheckSource 
 Gets the class index of the dataset.
   getClassIndex()   - 
Method in class weka.filters.unsupervised.attribute. ClassAssigner 
 returns the class index.
   getClassIndex()   - 
Method in class weka.filters.unsupervised.instance. RemoveMisclassified 
 Gets the attribute on which misclassifications are based.
   getClassMatches(String)   - 
Method in class weka.gui. SimpleCLIPanel.CommandlineCompletion 
 returns all the class/package matches with the partial search string.
   getClassname()   - 
Method in class weka.core. Javadoc 
 returns the current classname
   getClassname()   - 
Method in class weka.core. ListOptions 
 returns the current classname
   getClassName()   - 
Method in class weka.filters.unsupervised.attribute. NumericTransform 
 Get the class containing the transformation method.
   getClassname(String)   - 
Method in class weka.gui. SimpleCLIPanel.CommandlineCompletion 
 returns the classname part of the partial classname.
   getClassnames(String)   - 
Static method in class weka.gui. GenericObjectEditor 
 Returns the available classnames for a certain property in the 
 props file.
   getClassOrder()   - 
Method in class weka.filters.supervised.attribute. ClassOrder 
 Get the wanted class order
   getClassPriors()   - 
Method in class weka.classifiers. Evaluation 
 Get the current weighted class counts
   getClassType()   - 
Method in class weka.core. TestInstances 
 returns the current class type
   getClassValue()   - 
Method in class weka.filters.supervised.instance. SMOTE 
 Gets the index of the class value to which SMOTE should be applied.
   getClassValue()   - 
Method in class weka.gui.beans. ClassValuePicker 
 Gets the class value considered to be the "positive"
 class value.
   getClearEachDataset()   - 
Method in class weka.gui.streams. InstanceViewer 
  
   getClip()   - 
Method in class weka.gui.visualize. PostscriptGraphics 
 Not implemented
   getClipBounds()   - 
Method in class weka.gui.visualize. PostscriptGraphics 
 This returns the full current drawing area
   getClipBounds(Rectangle)   - 
Method in class weka.gui.visualize. PostscriptGraphics 
 This returns the full current drawing area
   getClipRect()   - 
Method in class weka.gui.visualize. PostscriptGraphics 
 Not implemented
   getClock()   - 
Method in class weka.core. Debug 
 returns the instance of the Clock that is internally used
   getClosestConnections(Point, int)   - 
Static method in class weka.gui.beans. BeanConnection 
 Return a list of connections within some delta of a point
   getClosestConnectorPoint(Point)   - 
Method in class weka.gui.beans. BeanVisual 
 Returns the coordinates of the closest "connector" point to the
 supplied point.
   getCloseTo()   - 
Method in class weka.filters.unsupervised.attribute. NumericCleaner 
 Get the "close to" number.
   getCloseToDefault()   - 
Method in class weka.filters.unsupervised.attribute. NumericCleaner 
 Get the "close to" default.
   getCloseToTolerance()   - 
Method in class weka.filters.unsupervised.attribute. NumericCleaner 
 Get the "close to" Tolerance.
   getClusterAssignments()   - 
Method in class weka.clusterers. ClusterEvaluation 
 Return an array of cluster assignments corresponding to the most
 recent set of instances clustered.
   getClusterCenters()   - 
Method in class weka.clusterers. XMeans 
 Return the centers of the clusters as an Instances object
   getClusterCentroids()   - 
Method in class weka.clusterers. SimpleKMeans 
 Gets the the cluster centroids
   getClusterDefinitions()   - 
Method in class weka.datagenerators.clusterers. SubspaceCluster 
 returns the currently set clusters
   getClusterer()   - 
Method in class weka.attributeSelection. UnsupervisedSubsetEvaluator 
 Get the clusterer
   getClusterer()   - 
Method in class weka.classifiers.meta. ClassificationViaClustering 
 Get the clusterer used as the base learner.
   getClusterer()   - 
Method in class weka.clusterers. CheckClusterer 
 Get the clusterer used as the clusterer
   getClusterer()   - 
Method in class weka.clusterers. MakeDensityBasedClusterer 
 Gets the clusterer being wrapped.
   getClusterer()   - 
Method in class weka.clusterers. SingleClustererEnhancer 
 Get the clusterer used as the base clusterer.
   getClusterer()   - 
Method in class weka.experiment. DensityBasedClustererSplitEvaluator 
 Get the value of clusterer
   getClusterer()   - 
Method in class weka.filters.unsupervised.attribute. AddCluster 
 Gets the clusterer used by the filter.
   getClusterer()   - 
Method in class weka.gui.beans. BatchClustererEvent 
 Get the clusterer
   getClusterer()   - 
Method in class weka.gui.beans. Clusterer 
 Get the clusterer currently set for this wrapper
   getClusterer()   - 
Static method in class weka.gui.explorer. ExplorerDefaults 
 returns the default clusterer (fully configured) for the clusterer panel.
   getClustererStoreClustersForVis()   - 
Static method in class weka.gui.explorer. ExplorerDefaults 
 returns whether the clusters are storeed for visualization purposes
 in the cluster panel.
   getClustererTestMode()   - 
Static method in class weka.gui.explorer. ExplorerDefaults 
 returns the default cluster test mode for the cluster panel.
   getClusteringSeed()   - 
Method in class weka.classifiers.functions. RBFNetwork 
 Get the random seed used by K-means.
   getClusterLabel()   - 
Method in interface weka.clusterers.forOPTICSAndDBScan.DataObjects. DataObject 
 Returns the clusterID, to which this DataObject belongs to
   getClusterLabel()   - 
Method in class weka.clusterers.forOPTICSAndDBScan.DataObjects. EuclidianDataObject 
 Returns the clusterID, to which this DataObject belongs to
   getClusterLabel()   - 
Method in class weka.clusterers.forOPTICSAndDBScan.DataObjects. ManhattanDataObject 
 Returns the clusterID, to which this DataObject belongs to
   getClusterModelsNumericAtts()   - 
Method in class weka.clusterers. EM 
 Return the normal distributions for the cluster models
   getClusterNominalCounts()   - 
Method in class weka.clusterers. SimpleKMeans 
 Returns for each cluster the frequency counts for the values of each 
 nominal attribute
   getClusterPriors()   - 
Method in class weka.clusterers. EM 
 Return the priors for the clusters
   getClusterSizes()   - 
Method in class weka.clusterers. SimpleKMeans 
 Gets the number of instances in each cluster
   getClusterStandardDevs()   - 
Method in class weka.clusterers. SimpleKMeans 
 Gets the standard deviations of the numeric attributes in each cluster
   getClusterSubType()   - 
Method in class weka.datagenerators.clusterers. SubspaceClusterDefinition 
 Gets the cluster sub type.
   getClusterType()   - 
Method in class weka.datagenerators.clusterers. SubspaceClusterDefinition 
 Gets the cluster type.
   getCoef0()   - 
Method in class weka.classifiers.functions. LibSVM 
 Gets coef
   getCoefficients()   - 
Method in class weka.core.matrix. LinearRegression 
 returns the calculated coefficients
   getColCount()   - 
Method in class weka.experiment. ResultMatrix 
 returns the number of columns
   getColHidden(int)   - 
Method in class weka.experiment. ResultMatrix 
 returns the hidden status of the column, if the index is valid, otherwise
 false
   getColName(int)   - 
Method in class weka.experiment. ResultMatrix 
 returns the name of the row, if the index is valid, otherwise null.
   getColNameWidth()   - 
Method in class weka.experiment. ResultMatrix 
 returns the current width for the column names
   getColor()   - 
Method in class weka.gui.treevisualizer. Node 
 Get the value of color.
   getColor()   - 
Method in class weka.gui.visualize. PostscriptGraphics 
 Get current pen color.
   getColorBox()   - 
Method in class weka.gui. AttributeVisualizationPanel 
 Returns the class selection combo box if the parent component wants to
 place it in itself or in some component other than this component.
   getColOrder()   - 
Method in class weka.experiment. ResultMatrix 
 returns the current order of the columns, null means the default order
   getColoringIndex()   - 
Method in class weka.gui. AttributeVisualizationPanel 
 Get the coloring (class) index for the plot
   getColoringIndex()   - 
Method in class weka.gui.beans. AttributeSummarizer 
 Return the coloring index for the attribute summary plots
   getColors()   - 
Method in class weka.gui.boundaryvisualizer. BoundaryPanel 
 Get the current vector of Color objects used for the classes
   getColumn(int)   - 
Method in class weka.classifiers.functions.pace. PaceMatrix 
 Return a DoubleVector that stores a column of the matrix
   getColumn(int, int, int)   - 
Method in class weka.classifiers.functions.pace. PaceMatrix 
 Return a DoubleVector that stores some elements of a column of the
  matrix
   getColumn(int)   - 
Method in class weka.core. Matrix 
  Deprecated.  Gets a column of the matrix and returns it as a double array.
   getColumn()   - 
Static method in class weka.gui.experiment. ExperimenterDefaults 
 the comma-separated list of attribute names that identify a column
   getColumnClass(int)   - 
Method in class weka.gui.arffviewer. ArffTableModel 
 returns the most specific superclass for all the cell values in the
 column (always String)
   getColumnClass(int)   - 
Method in class weka.gui. SortedTableModel 
 Returns the most specific superclass for all the cell values in the
 column.
   getColumnClass(int)   - 
Method in class weka.gui.sql. ResultSetTableModel 
 returns the most specific superclass for all the cell values in the 
 column (always String).
   getColumnClasses()   - 
Method in class weka.gui.sql. ResultSetHelper 
 returns the classes for the columns.
   getColumnCount()   - 
Method in class weka.clusterers.forOPTICSAndDBScan.OPTICS_GUI. ResultVectorTableModel 
 Returns the number of columns of this model.
   getColumnCount()   - 
Method in class weka.gui.arffviewer. ArffTableModel 
 returns the number of columns in the model
   getColumnCount()   - 
Method in class weka.gui. SortedTableModel 
 Returns the number of columns in the model
   getColumnCount()   - 
Method in class weka.gui.sql. ResultSetHelper 
 returns the number of columns in the resultset.
   getColumnCount()   - 
Method in class weka.gui.sql. ResultSetTableModel 
 returns the number of columns in the model.
   getColumnDimension()   - 
Method in class weka.core.matrix. Matrix 
 Get column dimension.
   getColumnName(int)   - 
Method in class weka.gui.arffviewer. ArffTableModel 
 returns the name of the column at columnIndex
   getColumnName(int)   - 
Method in class weka.gui. SortedTableModel 
 Returns the name of the column at columnIndex
   getColumnName(int)   - 
Method in class weka.gui.sql. ResultSetTableModel 
 returns the name of the column at columnIndex.
   getColumnNames()   - 
Method in class weka.gui.sql. ResultSetHelper 
 returns an array with the names of the columns in the resultset.
   getColumnPackedCopy()   - 
Method in class weka.core.matrix. Matrix 
 Make a one-dimensional column packed copy of the internal array.
   getCombination()   - 
Method in class weka.attributeSelection. ScatterSearchV1 
 Get the combination
   getCombinationRule()   - 
Method in class weka.classifiers.meta. Vote 
 Gets the combination rule used
   getCommand()   - 
Method in class weka.gui.treevisualizer. TreeDisplayEvent 
  
   getComment()   - 
Method in enum weka.core. TechnicalInformation.Field 
 returns the comment string
   getComment()   - 
Method in enum weka.core. TechnicalInformation.Type 
 returns the comment string
   getCommonPrefix()   - 
Method in class weka.core. Trie 
 returns the common prefix for all the nodes
   getCommonPrefix()   - 
Method in class weka.core. Trie.TrieNode 
 returns the common prefix for all the nodes starting with this node.
   getCommonPrefix(String)   - 
Method in class weka.core. Trie.TrieNode 
 returns the common prefix for all the nodes starting with the node
 for the specified prefix.
   getCommonPrefix(Vector<String>)   - 
Method in class weka.gui. SimpleCLIPanel.CommandlineCompletion 
 returns the common prefix for all the items in the list.
   getComparisonField()   - 
Static method in class weka.gui.experiment. ExperimenterDefaults 
 returns the name of the field used for comparison
   getCompatibilityState()   - 
Method in class weka.experiment. AveragingResultProducer 
 Gets a description of the internal settings of the result
 producer, sufficient for distinguishing a ResultProducer
 instance from another with different settings (ignoring
 those settings set through this interface).
   getCompatibilityState()   - 
Method in class weka.experiment. CrossValidationResultProducer 
 Gets a description of the internal settings of the result
 producer, sufficient for distinguishing a ResultProducer
 instance from another with different settings (ignoring
 those settings set through this interface).
   getCompatibilityState()   - 
Method in class weka.experiment. DatabaseResultProducer 
 Gets a description of the internal settings of the result
 producer, sufficient for distinguishing a ResultProducer
 instance from another with different settings (ignoring
 those settings set through this interface).
   getCompatibilityState()   - 
Method in class weka.experiment. LearningRateResultProducer 
 Gets a description of the internal settings of the result
 producer, sufficient for distinguishing a ResultProducer
 instance from another with different settings (ignoring
 those settings set through this interface).
   getCompatibilityState()   - 
Method in class weka.experiment. RandomSplitResultProducer 
 Gets a description of the internal settings of the result
 producer, sufficient for distinguishing a ResultProducer
 instance from another with different settings (ignoring
 those settings set through this interface).
   getCompatibilityState()   - 
Method in interface weka.experiment. ResultProducer 
 Gets a description of the internal settings of the result
 producer, sufficient for distinguishing a ResultProducer
 instance from another with different settings (ignoring
 those settings set through this interface).
   getComplexityParameter()   - 
Method in class weka.attributeSelection. SVMAttributeEval 
 Get the value of C used with SMO
   getComponent()   - 
Method in class weka.gui.visualize. JComponentWriter 
 returns the component that is stored in the output format
   getComponent()   - 
Method in class weka.gui.visualize. PrintableComponent 
 returns the GUI component this print dialog is part of.
   getComposite()   - 
Method in class weka.gui.visualize. PostscriptGraphics 
  
   getCompressOutput()   - 
Method in class weka.core.converters. ArffSaver 
 Gets whether the output data is compressed.
   getCompressOutput()   - 
Method in class weka.core.converters. XRFFSaver 
 Gets whether the output data is compressed.
   getConfidenceFactor()   - 
Method in class weka.classifiers.rules. PART 
 Get the value of CF.
   getConfidenceFactor()   - 
Method in class weka.classifiers.trees. J48 
 Get the value of CF.
   getConfidenceFactor()   - 
Method in class weka.classifiers.trees. J48graft 
 Get the value of CF.
   getConfirmation()   - 
Method in class weka.associations.tertius. Rule 
 Get the confirmation value of this rule.
   getConfirmationThreshold()   - 
Method in class weka.associations. Tertius 
 Get the value of confirmationThreshold.
   getConfirmationValues()   - 
Method in class weka.associations. Tertius 
 Get the value of confirmationValues.
   getConfirmExit()   - 
Method in class weka.gui.arffviewer. ArffViewer 
 returns the setting of whether to display a confirm messagebox or not
 on exit
   getConfirmExit()   - 
Method in class weka.gui.arffviewer. ArffViewerMainPanel 
 returns the setting of whether to display a confirm messagebox or not
 on exit
   getConfusionMatrix()   - 
Method in class weka.classifiers.evaluation. TwoClassStats 
 Generates a  ConfusionMatrix  representing the current
 two-class statistics, using class names "negative" and "positive".
   getConnectedFormat()   - 
Method in class weka.gui.beans. ClassAssigner 
 Returns the structure of the incoming instances (if any)
   getConnectedFormat()   - 
Method in class weka.gui.beans. ClassValuePicker 
 Returns the structure of the incoming instances (if any)
   getConnection()   - 
Method in class weka.gui.sql. DbUtils 
 returns the current database connection.
   getConnections()   - 
Static method in class weka.gui.beans. BeanConnection 
 Returns the list of connections
   getConnectorPoint(int)   - 
Method in class weka.gui.beans. BeanVisual 
 Returns the coordinates of the connector point given a compass point
   getConsequence()   - 
Method in class weka.associations. FPGrowth.AssociationRule 
 Get the consequence of this rule.
   getConsequenceSupport()   - 
Method in class weka.associations. FPGrowth.AssociationRule 
 Get the support for the consequence.
   getConsequent()   - 
Method in class weka.classifiers.rules. Rule 
 Get the consequent of this rule, i.e.
   getConservativeForwardSelection()   - 
Method in class weka.attributeSelection. GreedyStepwise 
 Gets whether conservative selection has been enabled
   getConstError(double[])   - 
Method in class weka.classifiers.trees.ft. FTtree 
  
   getContainChildBalls()   - 
Method in class weka.core.neighboursearch.balltrees. BallTreeConstructor 
 Gets whether if a parent ball should completely enclose
 its two child balls.
   getContent(Element)   - 
Method in class weka.classifiers.bayes.net. BIFReader 
 Returns all TEXT children of the given node in one string.
   getContent(Element)   - 
Method in class weka.classifiers.bayes.net. EditableBayesNet 
 XML helper function.
   getContent(Element)   - 
Static method in class weka.core.xml. XMLDocument 
 returns the text between the opening and closing tag of a node
 (performs a  trim()  on the result).
   getControlPanel()   - 
Method in class weka.gui.graphvisualizer. HierarchicalBCEngine 
 This method returns a handle to the extra
 controls panel, so that the visualizing
 class can add it to some of it's own
 gui panel.
   getControlPanel()   - 
Method in interface weka.gui.graphvisualizer. LayoutEngine 
 This method returns the extra controls panel
 for the LayoutEngine, if there is any.
   getConvertNominal()   - 
Method in class weka.classifiers.trees. LMT 
 Get the value of convertNominal.
   getConvertNominalToBinary()   - 
Method in class weka.classifiers.functions. LibLINEAR 
 Gets whether conversion of nominal to binary is
 turned on.
   getCoreConvertersOnly()   - 
Method in class weka.gui. ConverterFileChooser 
 Returns whether only the hardcoded core converters are displayed.
   getCoreDistance()   - 
Method in interface weka.clusterers.forOPTICSAndDBScan.DataObjects. DataObject 
 Returns the coreDistance for this dataObject
   getCoreDistance()   - 
Method in class weka.clusterers.forOPTICSAndDBScan.DataObjects. EuclidianDataObject 
 Returns the coreDistance for this dataObject
   getCoreDistance()   - 
Method in class weka.clusterers.forOPTICSAndDBScan.DataObjects. ManhattanDataObject 
 Returns the coreDistance for this dataObject
   getCost()   - 
Method in class weka.classifiers.functions. LibLINEAR 
 Returns the cost parameter C
   getCost()   - 
Method in class weka.classifiers.functions. LibSVM 
 Sets the parameter C of C-SVC, epsilon-SVR, and nu-SVR
   getCostMatrix()   - 
Method in class weka.attributeSelection. CostSensitiveASEvaluation 
 Gets the misclassification cost matrix.
   getCostMatrix()   - 
Method in class weka.classifiers.meta. CostSensitiveClassifier 
 Gets the misclassification cost matrix.
   getCostMatrix()   - 
Method in class weka.classifiers.meta. MetaCost 
 Gets the misclassification cost matrix.
   getCostMatrixSource()   - 
Method in class weka.attributeSelection. CostSensitiveASEvaluation 
 Gets the source location method of the cost matrix.
   getCostMatrixSource()   - 
Method in class weka.classifiers.meta. CostSensitiveClassifier 
 Gets the source location method of the cost matrix.
   getCostMatrixSource()   - 
Method in class weka.classifiers.meta. MetaCost 
 Gets the source location method of the cost matrix.
   getCount(double)   - 
Method in class weka.classifiers.bayes.net.estimate. DiscreteEstimatorBayes 
 Get a counts for a value
   getCount(double)   - 
Method in class weka.estimators. DiscreteEstimator 
 Get the count for a value
   getCount(int)   - 
Method in class weka.experiment. ResultMatrix 
 returns the count for the row.
   getCount(Node, int)   - 
Static method in class weka.gui.treevisualizer. Node 
 Recursively finds the number of visible nodes there are (this may 
 accidentally count some of the invis nodes).
   getCounterInstancesFrequency()   - 
Method in class weka.associations.tertius. LiteralSet 
 Get the frequency of counter-instances of this LiteralSet in the data.
   getCounterInstancesNumber()   - 
Method in class weka.associations.tertius. LiteralSet 
 Get the number of counter-instances of this LiteralSet.
   getCounts(int[], int[], int[], int, int, boolean)   - 
Method in class weka.classifiers.bayes.net. ADNode 
 get counts for specific instantiation of a set of nodes
   getCounts(int[], int[], int[], int, int, ADNode, boolean)   - 
Method in class weka.classifiers.bayes.net. VaryNode 
 get counts for specific instantiation of a set of nodes
   getCountWidth()   - 
Method in class weka.experiment. ResultMatrix 
 returns the current width for the counts
   getCreatorApplication()   - 
Method in class weka.classifiers.pmml.consumer. PMMLClassifier 
 Get the name of the application that created this model
   getCreatorApplication()   - 
Method in interface weka.core.pmml. PMMLModel 
 Get the name of the application that created this model.
   getCriticalValue()   - 
Method in class weka.classifiers.bayes. AODEsr 
 Gets the critical value.
   getCrossoverProb()   - 
Method in class weka.attributeSelection. GeneticSearch 
 get the probability of crossover
   getCrossVal()   - 
Method in class weka.classifiers.rules. DecisionTable 
 Gets the number of folds for cross validation
   getCrossValidate()   - 
Method in class weka.classifiers.lazy. IBk 
 Gets whether hold-one-out cross-validation will be used
 to select the best k value.
   getCurrent()   - 
Method in class weka.core. Memory 
 returns the current memory consumption
   getCurrentDatasetNumber()   - 
Method in class weka.experiment. Experiment 
 When an experiment is running, this returns the current dataset number.
   getCurrentDir()   - 
Static method in class weka.core. Debug 
 returns the current working directory of the user
   getCurrentFilename()   - 
Method in class weka.gui.arffviewer. ArffViewerMainPanel 
 returns the filename of the current tab
   getCurrentIndex()   - 
Method in class weka.gui.arffviewer. ArffViewerMainPanel 
 returns the currently selected tab index
   getCurrentInstance()   - 
Method in class weka.gui.beans. IncrementalClassifierEvent 
 Get the current instance
   getCurrentModel()   - 
Method in class weka.classifiers.misc. SerializedClassifier 
 Gets the currently loaded model (can be null).
   getCurrentPanel()   - 
Method in class weka.gui.arffviewer. ArffViewerMainPanel 
 returns the currently selected panel
   getCurrentPropertyNumber()   - 
Method in class weka.experiment. Experiment 
 When an experiment is running, this returns the index of the
 current custom property value.
   getCurrentRunNumber()   - 
Method in class weka.experiment. Experiment 
 When an experiment is running, this returns the current run number.
   getCurve(FastVector)   - 
Method in class weka.classifiers.evaluation. CostCurve 
 Calculates the performance stats for the default class and return 
 results as a set of Instances.
   getCurve(FastVector, int)   - 
Method in class weka.classifiers.evaluation. CostCurve 
 Calculates the performance stats for the desired class and return 
 results as a set of Instances.
   getCurve(FastVector)   - 
Method in class weka.classifiers.evaluation. MarginCurve 
 Calculates the cumulative margin distribution for the set of
 predictions, returning the result as a set of Instances.
   getCurve(FastVector)   - 
Method in class weka.classifiers.evaluation. ThresholdCurve 
 Calculates the performance stats for the default class and return 
 results as a set of Instances.
   getCurve(FastVector, int)   - 
Method in class weka.classifiers.evaluation. ThresholdCurve 
 Calculates the performance stats for the desired class and return 
 results as a set of Instances.
   getCustomEditor()   - 
Method in class weka.gui. CostMatrixEditor 
 Gets a GUI component with which the user can edit the cost matrix.
   getCustomEditor()   - 
Method in class weka.gui. FileEditor 
 Gets the custom editor component.
   getCustomEditor()   - 
Method in class weka.gui. GenericArrayEditor 
 Returns the array editing component.
   getCustomEditor()   - 
Method in class weka.gui. GenericObjectEditor 
 Returns the array editing component.
   getCustomEditor()   - 
Method in class weka.gui. SimpleDateFormatEditor 
 Gets a GUI component with which the user can edit the date format.
   getCustomHeight()   - 
Method in class weka.gui.visualize. JComponentWriter 
 gets the custom height currently used
   getCustomName()   - 
Method in class weka.gui.beans. Associator 
 Get the custom (descriptive) name for this bean (if one has been set)
   getCustomName()   - 
Method in interface weka.gui.beans. BeanCommon 
 Get the custom (descriptive) name for this bean (if one has been set)
   getCustomName()   - 
Method in class weka.gui.beans. ClassAssigner 
 Get the custom (descriptive) name for this bean (if one has been set)
   getCustomName()   - 
Method in class weka.gui.beans. Classifier 
 Get the custom (descriptive) name for this bean (if one has been set)
   getCustomName()   - 
Method in class weka.gui.beans. ClassifierPerformanceEvaluator 
 Get the custom (descriptive) name for this bean (if one has been set)
   getCustomName()   - 
Method in class weka.gui.beans. ClassValuePicker 
 Get the custom (descriptive) name for this bean (if one has been set)
   getCustomName()   - 
Method in class weka.gui.beans. Clusterer 
 Get the custom (descriptive) name for this bean (if one has been set)
   getCustomName()   - 
Method in class weka.gui.beans. ClustererPerformanceEvaluator 
 Get the custom (descriptive) name for this bean (if one has been set)
   getCustomName()   - 
Method in class weka.gui.beans. CostBenefitAnalysis 
 Get the custom (descriptive) name for this bean (if one has been set)
   getCustomName()   - 
Method in class weka.gui.beans. CrossValidationFoldMaker 
 Get the custom (descriptive) name for this bean (if one has been set)
   getCustomName()   - 
Method in class weka.gui.beans. Filter 
 Get the custom (descriptive) name for this bean (if one has been set)
   getCustomName()   - 
Method in class weka.gui.beans. IncrementalClassifierEvaluator 
 Get the custom (descriptive) name for this bean (if one has been set)
   getCustomName()   - 
Method in class weka.gui.beans. InstanceStreamToBatchMaker 
 Get the custom (descriptive) name for this bean (if one has been set)
   getCustomName()   - 
Method in class weka.gui.beans. Loader 
 Get the custom (descriptive) name for this bean (if one has been set)
   getCustomName()   - 
Method in class weka.gui.beans. MetaBean 
 Get the custom (descriptive) name for this bean (if one has been set)
   getCustomName()   - 
Method in class weka.gui.beans. PredictionAppender 
 Get the custom (descriptive) name for this bean (if one has been set)
   getCustomName()   - 
Method in class weka.gui.beans. Saver 
 Get the custom (descriptive) name for this bean (if one has been set)
   getCustomName()   - 
Method in class weka.gui.beans. SerializedModelSaver 
 Get the custom (descriptive) name for this bean (if one has been set)
   getCustomName()   - 
Method in class weka.gui.beans. StripChart 
 Get the custom (descriptive) name for this bean (if one has been set)
   getCustomName()   - 
Method in class weka.gui.beans. TestSetMaker 
 Get the custom (descriptive) name for this bean (if one has been set)
   getCustomName()   - 
Method in class weka.gui.beans. TextViewer 
 Get the custom (descriptive) name for this bean (if one has been set)
   getCustomName()   - 
Method in class weka.gui.beans. TrainingSetMaker 
 Get the custom (descriptive) name for this bean (if one has been set)
   getCustomName()   - 
Method in class weka.gui.beans. TrainTestSplitMaker 
 Get the custom (descriptive) name for this bean (if one has been set)
   getCustomPanel()   - 
Method in interface weka.gui. CustomPanelSupplier 
 Gets the custom panel for the object.
   getCustomPanel()   - 
Method in class weka.gui. GenericObjectEditor 
 Gets the custom panel used for editing the object.
   getCustomWidth()   - 
Method in class weka.gui.visualize. JComponentWriter 
 gets the custom width currently used
   getCutoff()   - 
Method in class weka.clusterers. Cobweb 
 get the cutoff
   getCutOffFactor()   - 
Method in class weka.clusterers. XMeans 
 Gets the cutoff factor.
   getCutPoints(int)   - 
Method in class weka.filters.supervised.attribute. Discretize 
 Gets the cut points for an attribute
   getCutPoints(int)   - 
Method in class weka.filters.unsupervised.attribute. Discretize 
 Gets the cut points for an attribute
   getCVisible()   - 
Method in class weka.gui.treevisualizer. Node 
 Get If this node's childs are visible.
   getCVParameter(int)   - 
Method in class weka.classifiers.meta. CVParameterSelection 
 Gets the scheme paramter with the given index.
   getCVParameters()   - 
Method in class weka.classifiers.meta. CVParameterSelection 
 Get method for CVParameters.
   getCVPredictions(Classifier, Instances, int)   - 
Method in class weka.classifiers.evaluation. EvaluationUtils 
 Generate a bunch of predictions ready for processing, by performing a
 cross-validation on the supplied dataset.
   getCVType()   - 
Method in class weka.classifiers.bayes.net.search.global. GlobalScoreSearchAlgorithm 
 get cross validation strategy to be used in searching for networks.
   getCycleEnd()   - 
Method in class weka.associations. GeneralizedSequentialPatterns 
 Returns the time/date string the cycle ended
   getCycleStart()   - 
Method in class weka.associations. GeneralizedSequentialPatterns 
 Returns the time/date string the cycle was started
   getD()   - 
Method in class weka.core.matrix. EigenvalueDecomposition 
 Return the block diagonal eigenvalue matrix
   getData()   - 
Method in class weka.attributeSelection. BestFirst.Link2 
 Get a group
   getData()   - 
Method in class weka.attributeSelection. LFSMethods.Link2 
 Get a group
   getData()   - 
Method in class weka.classifiers.rules. RuleStats 
 Get the data of the stats
   getData()   - 
Method in class weka.core. AttributeLocator 
 returns the underlying data
   getData()   - 
Method in class weka.core.converters. ArffLoader.ArffReader 
 Returns the data that was read
   getData()   - 
Method in class weka.core. TestInstances 
 returns the current dataset, can be null
   getDatabase_distanceType()   - 
Method in class weka.clusterers. DBScan 
 Returns the distance-type
   getDatabase_distanceType()   - 
Method in class weka.clusterers.forOPTICSAndDBScan.OPTICS_GUI. SERObject 
 Returns the distance-type
   getDatabase_distanceType()   - 
Method in class weka.clusterers. OPTICS 
 Returns the distance-type
   getDatabase_Type()   - 
Method in class weka.clusterers. DBScan 
 Returns the type of the used index (database)
   getDatabase_Type()   - 
Method in class weka.clusterers.forOPTICSAndDBScan.OPTICS_GUI. SERObject 
 Returns the type of the used index (database)
   getDatabase_Type()   - 
Method in class weka.clusterers. OPTICS 
 Returns the type of the used index (database)
   getDatabaseOutput()   - 
Method in class weka.clusterers. OPTICS 
 Returns the file to save the database to - if directory, database is not
 saved.
   getDatabaseSize()   - 
Method in class weka.clusterers.forOPTICSAndDBScan.OPTICS_GUI. SERObject 
 Returns the database's size
   getDatabaseURL()   - 
Method in class weka.experiment. DatabaseUtils 
 Get the value of DatabaseURL.
   getDataDictionary()   - 
Method in class weka.classifiers.pmml.consumer. PMMLClassifier 
 Get the data dictionary.
   getDataFileName()   - 
Method in class weka.classifiers. BVDecompose 
 Get the name of the data file used for the decomposition
   getDataFileName()   - 
Method in class weka.classifiers. BVDecomposeSegCVSub 
 Get the name of the data file used for the decomposition
   getDataObject(String)   - 
Method in interface weka.clusterers.forOPTICSAndDBScan.Databases. Database 
 Select a dataObject from the database
   getDataObject(String)   - 
Method in class weka.clusterers.forOPTICSAndDBScan.Databases. SequentialDatabase 
 Select a dataObject from the database
   getDataObject()   - 
Method in class weka.clusterers.forOPTICSAndDBScan.Utils. EpsilonRange_ListElement 
 Returns this dataObject
   getDataPoint()   - 
Method in class weka.gui.beans. ChartEvent 
 Get the data point
   getDataSeqID()   - 
Method in class weka.associations. GeneralizedSequentialPatterns 
 Returns the attribute representing the data sequence ID.
   getDataset()   - 
Method in class weka.classifiers. CheckSource 
 Gets the dataset to use for testing, can be null.
   getDataSet()   - 
Method in class weka.core.converters. AbstractLoader 
  
   getDataSet()   - 
Method in class weka.core.converters. ArffLoader 
 Return the full data set.
   getDataSet()   - 
Method in class weka.core.converters. C45Loader 
 Return the full data set.
   getDataSet()   - 
Method in class weka.core.converters. ConverterUtils.DataSource 
 returns the full dataset, can be null in case of an error.
   getDataSet(int)   - 
Method in class weka.core.converters. ConverterUtils.DataSource 
 returns the full dataset with the specified class index set, 
 can be null in case of an error.
   getDataSet()   - 
Method in class weka.core.converters. CSVLoader 
 Return the full data set.
   getDataSet()   - 
Method in class weka.core.converters. DatabaseLoader 
 Return the full data set in batch mode (header and all intances at once).
   getDataSet()   - 
Method in class weka.core.converters. LibSVMLoader 
 Return the full data set.
   getDataSet()   - 
Method in interface weka.core.converters. Loader 
 Return the full data set.
   getDataSet()   - 
Method in class weka.core.converters. SerializedInstancesLoader 
 Return the full data set.
   getDataSet()   - 
Method in class weka.core.converters. SVMLightLoader 
 Return the full data set.
   getDataSet()   - 
Method in class weka.core.converters. TextDirectoryLoader 
 Return the full data set.
   getDataSet()   - 
Method in class weka.core.converters. XRFFLoader 
 Return the full data set.
   getDataset()   - 
Method in class weka.filters. CheckSource 
 Gets the dataset to use for testing, can be null.
   getDataSet()   - 
Method in class weka.gui.beans. DataSetEvent 
 Return the instances of the data set
   getDataSet()   - 
Method in class weka.gui.beans. ThresholdDataEvent 
 Return the instances of the data set
   getDataSet()   - 
Method in class weka.gui.beans. VisualizableErrorEvent 
 Return the instances of the data set
   getDatasetFormat()   - 
Method in class weka.datagenerators. DataGenerator 
 Gets the format of the dataset that is to be generated.
   getDatasetKeyColumns()   - 
Method in class weka.experiment. PairedTTester 
 Get the value of DatasetKeyColumns.
   getDatasetKeyColumns()   - 
Method in interface weka.experiment. Tester 
 Get the value of DatasetKeyColumns.
   getDatasets()   - 
Method in class weka.experiment. Experiment 
 Gets the datasets in the experiment.
   getDatasetsFirst()   - 
Static method in class weka.gui.experiment. ExperimenterDefaults 
 whether datasets or algorithms are iterated first
   getDataType()   - 
Method in class weka.gui.beans.xml. XMLBeans 
 returns the type of data that is to be read/written
   getDateAttributes()   - 
Method in class weka.core.converters. CSVLoader 
 Returns the current attribute range to be forced to type date.
   getDateFormat()   - 
Method in class weka.core. Attribute 
 Returns the Date format pattern in case this attribute is of type DATE,
 otherwise an empty string.
   getDateFormat()   - 
Method in class weka.core.converters. CSVLoader 
 Get the format to use for parsing date values.
   getDateFormat()   - 
Method in class weka.filters.unsupervised.attribute. Add 
 Get the date format, complying to ISO-8601.
   getDateFormat()   - 
Method in class weka.filters.unsupervised.attribute. ChangeDateFormat 
 Get the date format used in output.
   getDbUtils()   - 
Method in class weka.gui.sql.event. ConnectionEvent 
 returns the DbUtils instance that is responsible for the
 connect/disconnect.
   getDbUtils()   - 
Method in class weka.gui.sql.event. QueryExecuteEvent 
 returns the DbUtils instance that was executed the query
   getDebug()   - 
Method in class weka.associations. GeneralizedSequentialPatterns 
 Get whether debugging is turned on.
   getDebug()   - 
Method in class weka.attributeSelection. RaceSearch 
 Get whether output is to be verbose
   getDebug()   - 
Method in class weka.attributeSelection. ScatterSearchV1 
 Get whether output is to be verbose
   getDebug()   - 
Method in class weka.classifiers. BVDecompose 
 Gets whether debugging is turned on
   getDebug()   - 
Method in class weka.classifiers. BVDecomposeSegCVSub 
 Gets whether debugging is turned on
   getDebug()   - 
Method in class weka.classifiers. Classifier 
 Get whether debugging is turned on.
   getDebug()   - 
Method in class weka.classifiers.functions. LeastMedSq 
 Returns whether or not debugging output shouild be printed
   getDebug()   - 
Method in class weka.classifiers.functions. LinearRegression 
 Controls whether debugging output will be printed
   getDebug()   - 
Method in class weka.classifiers.functions. Logistic 
 Gets whether debugging output will be printed.
   getDebug()   - 
Method in class weka.classifiers.functions. PaceRegression 
 Controls whether debugging output will be printed
   getDebug()   - 
Method in class weka.classifiers.functions.supportVector. Kernel 
 Gets whether debugging output is turned on or not.
   getDebug()   - 
Method in class weka.classifiers.meta. MultiScheme 
 Get whether debugging is turned on
   getDebug()   - 
Method in class weka.classifiers.rules. JRip 
 Gets whether debug information is output to the console
   getDebug()   - 
Method in class weka.clusterers. EM 
 Get debug mode
   getDebug()   - 
Method in class weka.clusterers. HierarchicalClusterer 
 Get whether debugging is turned on.
   getDebug()   - 
Method in class weka.clusterers. sIB 
 Get debug mode
   getDebug()   - 
Method in class weka.core. Check 
 Get whether debugging is turned on
   getDebug()   - 
Method in class weka.core.converters. TextDirectoryLoader 
 Gets whether additional debug information is printed.
   getDebug()   - 
Method in class weka.core. Debug.Random 
 returns whether to print the generated random values or not
   getDebug()   - 
Method in class weka.datagenerators. DataGenerator 
 Gets the debug flag.
   getDebug()   - 
Method in class weka.estimators. CheckEstimator 
 Get whether debugging is turned on
   getDebug()   - 
Method in class weka.estimators. Estimator 
 Get whether debugging is turned on.
   getDebug()   - 
Method in class weka.experiment. DatabaseUtils 
 Gets whether there should be printed some debugging output to stderr or not.
   getDebug()   - 
Method in class weka.filters. SimpleFilter 
 Returns the current debugging mode state.
   getDebug()   - 
Method in class weka.filters.unsupervised.attribute. AddExpression 
 Gets whether debug is set
   getDebug()   - 
Method in class weka.gui. DatabaseConnectionDialog 
 Returns the debug flag
   getDebug()   - 
Method in class weka.gui. SimpleCLIPanel.CommandlineCompletion 
 returns whether debug mode is on.
   getDebug()   - 
Method in class weka.gui.streams. InstanceCounter 
  
   getDebug()   - 
Method in class weka.gui.streams. InstanceJoiner 
  
   getDebug()   - 
Method in class weka.gui.streams. InstanceLoader 
  
   getDebug()   - 
Method in class weka.gui.streams. InstanceSavePanel 
  
   getDebug()   - 
Method in class weka.gui.streams. InstanceTable 
  
   getDebug()   - 
Method in class weka.gui.streams. InstanceViewer 
  
   getDebugLevel()   - 
Method in class weka.clusterers. XMeans 
 Gets the debug level.
   getDebugVectorsFile()   - 
Method in class weka.clusterers. XMeans 
 Gets the file name for a file that has the random vectors stored.
   getDecay()   - 
Method in class weka.classifiers.functions. MultilayerPerceptron 
  
   getDecimals()   - 
Method in class weka.filters.unsupervised.attribute. NumericCleaner 
 Get the number of decimals to round to.
   getDefault()   - 
Method in class weka.core. Tee 
 returns the default printstrean, can be NULL.
   getDefaultValue()   - 
Method in class weka.core.pmml. TargetMetaInfo 
 Get the default value (numeric target)
   getDefaultWeight()   - 
Method in class weka.classifiers.functions. Winnow 
 Get the value of defaultWeight.
   getDegree()   - 
Method in class weka.classifiers.functions. LibSVM 
 Gets the degree of the kernel
   getDegreesOfFreedom()   - 
Method in class weka.experiment. PairedStats 
 Gets the degrees of freedom.
   getDeleteEmptyBins()   - 
Method in class weka.classifiers.meta. RegressionByDiscretization 
 Gets the number of bins numeric attributes will be divided into
   getDelimiters()   - 
Method in class weka.core.tokenizers. CharacterDelimitedTokenizer 
 Get the value of delimiters (not backquoted).
   getDelta()   - 
Method in class weka.associations. Apriori 
 Get the value of delta.
   getDelta()   - 
Method in class weka.associations. FPGrowth 
 Get the value of delta.
   getDelta()   - 
Method in class weka.classifiers.bayes.net.search.global. SimulatedAnnealing 
  
   getDelta()   - 
Method in class weka.classifiers.bayes.net.search.local. SimulatedAnnealing 
  
   getDensityBasedClusterer()   - 
Method in class weka.filters.unsupervised.attribute. ClusterMembership 
 Get the clusterer used by this filter
   getDerivedFields()   - 
Method in class weka.core.pmml. MiningSchema 
  
   getDerivedValue(double[])   - 
Method in class weka.core.pmml. DerivedFieldMetaInfo 
 Get the derived field value for the given incoming vector of
 values.
   getDescendantPopulationSize()   - 
Method in class weka.classifiers.bayes.net.search.global. GeneticSearch 
  
   getDescendantPopulationSize()   - 
Method in class weka.classifiers.bayes.net.search.local. GeneticSearch 
  
   getDescription()   - 
Method in class weka.clusterers.forOPTICSAndDBScan.OPTICS_GUI. SERFileFilter 
 The description of this filter.
   getDescription()   - 
Method in class weka.gui. ExtensionFileFilter 
 Gets the description of accepted files.
   getDescription()   - 
Method in class weka.gui.visualize. BMPWriter 
 returns the name of the writer, to display in the FileChooser.
   getDescription()   - 
Method in class weka.gui.visualize. JComponentWriter 
 returns the name of the writer, to display in the FileChooser.
   getDescription()   - 
Method in class weka.gui.visualize. JPEGWriter 
 returns the name of the writer, to display in the FileChooser.
   getDescription()   - 
Method in class weka.gui.visualize. PNGWriter 
 returns the name of the writer, to display in the FileChooser.
   getDescription()   - 
Method in class weka.gui.visualize. PostscriptWriter 
 returns the name of the writer, to display in the FileChooser.
   getDesignatedClass()   - 
Method in class weka.classifiers.meta. ThresholdSelector 
 Gets the method to determine which class value to optimize.
   getDesignVersion()   - 
Method in interface weka.gui.visualize.plugins. VisualizePlugin 
 Get the specific version of Weka the class is designed for.
   getDesiredSize()   - 
Method in class weka.classifiers.meta. Decorate 
 Gets the desired size of the committee.
   getDesiredWeightOfInstancesPerInterval()   - 
Method in class weka.filters.unsupervised.attribute. Discretize 
 Get the DesiredWeightOfInstancesPerInterval value.
   getDestination()   - 
Static method in class weka.gui.experiment. ExperimenterDefaults 
 returns the default destination
   getDetectionPerAttribute()   - 
Method in class weka.filters.unsupervised.attribute. InterquartileRange 
 Gets whether an Outlier/ExtremeValue attribute pair is generated for 
 each numeric attribute ("true") or just one pair for all numeric 
 attributes together ("false").
   getDeviceConfiguration()   - 
Method in class weka.gui.visualize. PostscriptGraphics 
  
   getDir()   - 
Method in class weka.core. Javadoc 
 returns the current dir containing the class to update.
   getDir()   - 
Method in class weka.gui. Loader 
 returns the dir prefix
   getDirection()   - 
Method in class weka.attributeSelection. BestFirst 
 Get the search direction
   getDirectory()   - 
Method in class weka.core.converters. TextDirectoryLoader 
 get the Dir specified as the source
   getDirectory()   - 
Method in class weka.gui.beans. SerializedModelSaver 
 Get the directory that the model(s) will be saved into
   getDiscretizeBin()   - 
Method in class weka.classifiers.mi. MIBoost 
 Get the number of bins in discretization
   getDiscretizer()   - 
Method in class weka.classifiers.trees.j48. NBTreeNoSplit 
 Return the discretizer used at this node
   getDisplay()   - 
Method in enum weka.core. TechnicalInformation.Field 
 returns the display string
   getDisplay()   - 
Method in enum weka.core. TechnicalInformation.Type 
 returns the display string
   getDisplayCol(int)   - 
Method in class weka.experiment. ResultMatrix 
 returns the displayed index of the given col, depending on the order of
 columns, returns -1 if index out of bounds
   getDisplayedResultsets()   - 
Method in class weka.experiment. PairedTTester 
 Gets the indices of the the datasets that are displayed (if  null 
 then all are displayed).
   getDisplayedResultsets()   - 
Method in interface weka.experiment. Tester 
 Gets the indices of the the datasets that are displayed (if  null 
 then all are displayed).
   getDisplayModelInOldFormat()   - 
Method in class weka.classifiers.bayes. NaiveBayes 
 Get whether to display model output in the old, original
 format.
   getDisplayModelInOldFormat()   - 
Method in class weka.clusterers. EM 
 Get whether to display model output in the old, original
 format.
   getDisplayName()   - 
Method in class weka.experiment. PairedCorrectedTTester 
 returns the name of the tester
   getDisplayName()   - 
Method in class weka.experiment. PairedTTester 
 returns the name of the tester
   getDisplayName()   - 
Method in class weka.experiment. ResultMatrix 
 returns the name of the output format
   getDisplayName()   - 
Method in class weka.experiment. ResultMatrixCSV 
 returns the name of the output format
   getDisplayName()   - 
Method in class weka.experiment. ResultMatrixGnuPlot 
 returns the name of the output format
   getDisplayName()   - 
Method in class weka.experiment. ResultMatrixHTML 
 returns the name of the output format
   getDisplayName()   - 
Method in class weka.experiment. ResultMatrixLatex 
 returns the name of the output format
   getDisplayName()   - 
Method in class weka.experiment. ResultMatrixPlainText 
 returns the name of the output format
   getDisplayName()   - 
Method in class weka.experiment. ResultMatrixSignificance 
 returns the name of the output format
   getDisplayName()   - 
Method in interface weka.experiment. Tester 
 returns the name of the testing algorithm
   getDisplayRow(int)   - 
Method in class weka.experiment. ResultMatrix 
 returns the displayed index of the given row, depending on the order of
 rows, returns -1 if index out of bounds
   getDisplayRules()   - 
Method in class weka.classifiers.rules. DecisionTable 
 Gets whether rules are being printed
   getDisplayStdDevs()   - 
Method in class weka.clusterers. SimpleKMeans 
 Gets whether standard deviations and nominal count
 Should be displayed in the clustering output
   getDisplayValue()   - 
Method in class weka.core.pmml. FieldMetaInfo.Value 
  
   getDistance()   - 
Method in class weka.clusterers.forOPTICSAndDBScan.Utils. EpsilonRange_ListElement 
 Returns the distance that was calulcated for this dataObject
 (The distance between this dataObject and the dataObject for which an epsilon-range-query
 was performed.)
   getDistanceF()   - 
Method in class weka.clusterers. XMeans 
 Gets the distance function.
   getDistanceFunction()   - 
Method in class weka.clusterers. HierarchicalClusterer 
  
   getDistanceFunction()   - 
Method in class weka.clusterers. SimpleKMeans 
 returns the distance function currently in use.
   getDistanceFunction()   - 
Method in class weka.core.neighboursearch. KDTree 
 returns the distance function currently in use.
   getDistanceFunction()   - 
Method in class weka.core.neighboursearch. NearestNeighbourSearch 
 returns the distance function currently in use.
   getDistanceIsBranchLength()   - 
Method in class weka.clusterers. HierarchicalClusterer 
  
   getDistances()   - 
Method in class weka.core.neighboursearch. BallTree 
 Returns the distances of the k nearest neighbours.
   getDistances()   - 
Method in class weka.core.neighboursearch. CoverTree 
 Returns the distances of the (k)-NN(s) found earlier
 by kNearestNeighbours()/nearestNeighbour().
   getDistances()   - 
Method in class weka.core.neighboursearch. KDTree 
 Returns the distances to the kNearest or 1 nearest neighbour currently
 found with either the kNearestNeighbours or the nearestNeighbour method.
   getDistances()   - 
Method in class weka.core.neighboursearch. LinearNNSearch 
 Returns the distances of the k nearest neighbours.
   getDistances()   - 
Method in class weka.core.neighboursearch. NearestNeighbourSearch 
 Returns the distances of the k nearest neighbours.
   getDistanceWeighting()   - 
Method in class weka.classifiers.lazy. IBk 
 Gets the distance weighting method used.
   getDistMult()   - 
Method in class weka.datagenerators.clusterers. BIRCHCluster 
 Gets the distance multiplier.
   getDistribution(String)   - 
Method in class weka.classifiers.bayes.net. EditableBayesNet 
 returns distribution of a node in matrix form with matrix representing distribution
 with P[i][j] = P(node = j | parent configuration = i)
   getDistribution(int)   - 
Method in class weka.classifiers.bayes.net. EditableBayesNet 
 returns distribution of a node in matrix form with matrix representing distribution
 with P[i][j] = P(node = j | parent configuration = i)
   getDistribution()   - 
Method in class weka.filters.unsupervised.attribute. RandomProjection 
 Returns the current distribution that'll be used for calculating the 
 random matrix
   getDistributions()   - 
Method in class weka.classifiers.bayes. BayesNet 
 Get full set of estimators.
   getDistributions(int)   - 
Method in class weka.classifiers.rules. RuleStats 
 Get the class distribution predicted by the rule in
 given position
   getDistributionSpread()   - 
Method in class weka.filters.supervised.instance. SpreadSubsample 
 Gets the value for the distribution spread
   getDocType()   - 
Method in class weka.core.xml. XMLDocument 
 returns the current DOCTYPE, can be  null .
   getDocument()   - 
Method in class weka.core.xml. XMLDocument 
 returns the parsed DOM document.
   getDocument()   - 
Method in class weka.core.xml. XMLOptions 
 returns the parsed DOM document.
   getDoNotOperateOnPerClassBasis()   - 
Method in class weka.filters.unsupervised.attribute. StringToWordVector 
 Get the DoNotOperateOnPerClassBasis value.
   getDoNotReplaceMissingValues()   - 
Method in class weka.classifiers.functions. LibLINEAR 
 Gets whether automatic replacement of missing values is
 disabled.
   getDoNotReplaceMissingValues()   - 
Method in class weka.classifiers.functions. LibSVM 
 Gets whether automatic replacement of missing values is
 disabled.
   getDontFilterAfterFirstBatch()   - 
Method in class weka.filters.unsupervised.instance. RemoveWithValues 
 Get whether to apply the filter to instances that arrive once
 the first (training) batch has been seen.
   getDontNormalize()   - 
Method in class weka.classifiers.functions. SPegasos 
 Get whether normalization has been turned off.
   getDontNormalize()   - 
Method in class weka.core. NormalizableDistance 
 Gets whether if the attribute values are to be normazlied in distance
 calculation.
   getDontReplaceMissing()   - 
Method in class weka.classifiers.functions. SPegasos 
 Get whether global replacement of missing values has been
 disabled.
   getDontReplaceMissingValues()   - 
Method in class weka.clusterers. SimpleKMeans 
 Gets whether missing values are to be replaced
   getDoublePivot()   - 
Method in class weka.core.matrix. LUDecomposition 
 Return pivot permutation vector as a one-dimensional double array
   getEditor()   - 
Method in class weka.gui. PropertyDialog 
 Gets the current property editor.
   getEditorActive()   - 
Method in class weka.gui.experiment. GeneratorPropertyIteratorPanel 
 Returns true if the editor is currently in an active status---that
 is the array is active and able to be edited.
   getElapsedTime()   - 
Method in class weka.clusterers.forOPTICSAndDBScan.OPTICS_GUI. SERObject 
 Returns the elapsed-time
   getElement(int, int)   - 
Method in class weka.classifiers. CostMatrix 
 Return the value of a cell as a double (for legacy code)
   getElement(int, int, Instance)   - 
Method in class weka.classifiers. CostMatrix 
 Return the value of a cell as a double.
   getElement(int)   - 
Method in class weka.core. AlgVector 
 Returns the value of a cell in the matrix.
   getElement(int, int)   - 
Method in class weka.core. Matrix 
  Deprecated.  Returns the value of a cell in the matrix.
   getElementAt(int)   - 
Method in class weka.gui. CheckBoxList.CheckBoxListModel 
 Returns the component at the specified index.
   getElements()   - 
Method in class weka.core. AlgVector 
 Gets the elements of the vector and returns them as double array.
   getEliminateColinearAttributes()   - 
Method in class weka.classifiers.functions. LinearRegression 
 Get the value of EliminateColinearAttributes.
   getEnabled()   - 
Method in class weka.core. Debug 
 returns whether the logging is enabled
   getEntropicAutoBlend()   - 
Method in class weka.classifiers.lazy. KStar 
 Get whether entropic blending being used
   getEntry(double)   - 
Method in class weka.classifiers.lazy.kstar. KStarCache.CacheTable 
 Returns the table entry to which the specified key is mapped in 
 this hashtable.
   getEnumerateColNames()   - 
Method in class weka.experiment. ResultMatrix 
 returns whether column names or numbers instead are enumerateed
   getEnumerateRowNames()   - 
Method in class weka.experiment. ResultMatrix 
 returns whether row names or numbers instead are enumerateed
   getEnvironment()   - 
Method in class weka.gui.beans. FlowRunner 
 Get the environment variables that are in use.
   getEpochs()   - 
Method in class weka.classifiers.functions. SPegasos 
 Get current number of epochs
   getEps()   - 
Method in class weka.classifiers.functions. LibLINEAR 
 Gets tolerance of termination criterion
   getEps()   - 
Method in class weka.classifiers.functions. LibSVM 
 Gets tolerance of termination criterion
   getEpsilon()   - 
Method in class weka.classifiers.functions. SMO 
 Get the value of epsilon.
   getEpsilon()   - 
Method in class weka.classifiers.functions.supportVector. RegSMO 
 Get the value of epsilon.
   getEpsilon()   - 
Method in class weka.classifiers.mi. MISMO 
 Get the value of epsilon.
   getEpsilon()   - 
Method in class weka.clusterers. DBScan 
 Returns the value of epsilon
   getEpsilon()   - 
Method in class weka.clusterers.forOPTICSAndDBScan.OPTICS_GUI. SERObject 
 Returns the value of epsilon
   getEpsilon()   - 
Method in class weka.clusterers. OPTICS 
 Returns the value of epsilon
   getEpsilonParameter()   - 
Method in class weka.attributeSelection. SVMAttributeEval 
 Get the value of P used with SMO
   getEpsilonParameter()   - 
Method in class weka.classifiers.functions.supportVector. RegOptimizer 
 Get the value of epsilon parameter of the epsilon insensitive loss function.
   getError()   - 
Method in class weka.classifiers. BVDecompose 
 Get the calculated error rate
   getError()   - 
Method in class weka.classifiers. BVDecomposeSegCVSub 
 Get the calculated error rate
   getErrorOnProbabilities()   - 
Method in class weka.classifiers.functions. SimpleLogistic 
 Get the value of errorOnProbabilities.
   getErrorOnProbabilities()   - 
Method in class weka.classifiers.trees. FT 
 Get the value of errorOnProbabilities.
   getErrorOnProbabilities()   - 
Method in class weka.classifiers.trees. LMT 
 Get the value of errorOnProbabilities.
   getErrors()   - 
Method in class weka.classifiers.trees.j48. NBTreeNoSplit 
 Return the errors made by the naive bayes model at this node
   getErrors()   - 
Method in class weka.classifiers.trees.j48. NBTreeSplit 
 Return the errors made by the naive bayes models arising
 from this split.
   getEstimatedErrorsForLeaf()   - 
Method in class weka.classifiers.rules.part. C45PruneableDecList 
 Computes estimated errors for leaf.
   getEstimator()   - 
Method in class weka.classifiers.bayes. BayesNet 
 Get the BayesNetEstimator used for calculating the CPTs
   getEstimator()   - 
Method in class weka.classifiers.functions. PaceRegression 
 Gets the estimator
   getEstimator()   - 
Method in class weka.estimators. CheckEstimator 
 Get the estimator used as the estimator
   getEstimator(double)   - 
Method in interface weka.estimators. ConditionalEstimator 
 Get a probability estimator for a value
   getEstimator(double)   - 
Method in class weka.estimators. DDConditionalEstimator 
 Get a probability estimator for a value
   getEstimator(double)   - 
Method in class weka.estimators. DKConditionalEstimator 
 Get a probability estimator for a value
   getEstimator(double)   - 
Method in class weka.estimators. DNConditionalEstimator 
 Get a probability estimator for a value
   getEstimator(double)   - 
Method in class weka.estimators. KDConditionalEstimator 
 Get a probability estimator for a value
   getEstimator(double)   - 
Method in class weka.estimators. KKConditionalEstimator 
 Get a probability estimator for a value
   getEstimator(double)   - 
Method in class weka.estimators. NDConditionalEstimator 
 Get a probability estimator for a value
   getEstimator(double)   - 
Method in class weka.estimators. NNConditionalEstimator 
 Get a probability estimator for a value
   getEvaluation()   - 
Method in class weka.classifiers.meta. GridSearch 
 Gets the criterion used for evaluating the classifier performance.
   getEvaluationMeasure()   - 
Method in class weka.classifiers.rules. DecisionTable 
 Gets the currently set performance evaluation measure used for selecting
 attributes for the decision table
   getEvaluationMode()   - 
Method in class weka.classifiers.meta. ThresholdSelector 
 Gets the evaluation mode used.
   getEvaluator()   - 
Method in class weka.attributeSelection. CheckAttributeSelection 
 Get the current evaluator
   getEvaluator()   - 
Method in class weka.attributeSelection. CostSensitiveASEvaluation 
 Get the evaluator used as the base evaluator.
   getEvaluator()   - 
Method in class weka.classifiers.meta. AttributeSelectedClassifier 
 Gets the attribute evaluator used
   getEvaluator()   - 
Method in class weka.filters.supervised.attribute. AttributeSelection 
 Get the name of the attribute/subset evaluator
   getEvalUsingTrainingData()   - 
Method in class weka.attributeSelection. OneRAttributeEval 
 Returns true if the training data is to be used for evaluation
   getEventName()   - 
Method in class weka.gui.beans. BeanConnection 
 Returns the name of the event for this conncetion
   getEvents()   - 
Method in class weka.associations.gsp. Element 
 Returns the events Array of an Element.
   getEventSetDescriptors()   - 
Method in class weka.gui.beans. AbstractDataSinkBeanInfo 
 Get the event set descriptors for this bean
   getEventSetDescriptors()   - 
Method in class weka.gui.beans. AbstractDataSourceBeanInfo 
 Get the event set descriptors pertinent to data sources
   getEventSetDescriptors()   - 
Method in class weka.gui.beans. AbstractTestSetProducerBeanInfo 
  
   getEventSetDescriptors()   - 
Method in class weka.gui.beans. AbstractTrainAndTestSetProducerBeanInfo 
  
   getEventSetDescriptors()   - 
Method in class weka.gui.beans. AbstractTrainingSetProducerBeanInfo 
 Returns event set descriptors for this type of bean
   getEventSetDescriptors()   - 
Method in class weka.gui.beans. AssociatorBeanInfo 
  
   getEventSetDescriptors()   - 
Method in class weka.gui.beans. AttributeSummarizerBeanInfo 
 Get the event set descriptors for this bean
   getEventSetDescriptors()   - 
Method in class weka.gui.beans. ClassAssignerBeanInfo 
 Returns the event set descriptors
   getEventSetDescriptors()   - 
Method in class weka.gui.beans. ClassifierBeanInfo 
  
   getEventSetDescriptors()   - 
Method in class weka.gui.beans. ClassifierPerformanceEvaluatorBeanInfo 
  
   getEventSetDescriptors()   - 
Method in class weka.gui.beans. ClassValuePickerBeanInfo 
 Returns the event set descriptors
   getEventSetDescriptors()   - 
Method in class weka.gui.beans. ClustererBeanInfo 
  
   getEventSetDescriptors()   - 
Method in class weka.gui.beans. ClustererPerformanceEvaluatorBeanInfo 
  
   getEventSetDescriptors()   - 
Method in class weka.gui.beans. CostBenefitAnalysisBeanInfo 
 Get the event set descriptors for this bean
   getEventSetDescriptors()   - 
Method in class weka.gui.beans. DataVisualizerBeanInfo 
 Get the event set descriptors for this bean
   getEventSetDescriptors()   - 
Method in class weka.gui.beans. FilterBeanInfo 
 Get the event set descriptors for this bean
   getEventSetDescriptors()   - 
Method in class weka.gui.beans. GraphViewerBeanInfo 
 Get the event set descriptors for this bean
   getEventSetDescriptors()   - 
Method in class weka.gui.beans. IncrementalClassifierEvaluatorBeanInfo 
 Get the event set descriptors for this bean
   getEventSetDescriptors()   - 
Method in class weka.gui.beans. InstanceStreamToBatchMakerBeanInfo 
 Returns the event set descriptors
   getEventSetDescriptors()   - 
Method in class weka.gui.beans. ModelPerformanceChartBeanInfo 
 Get the event set descriptors for this bean
   getEventSetDescriptors()   - 
Method in class weka.gui.beans. PredictionAppenderBeanInfo 
 Get the event set descriptors pertinent to data sources
   getEventSetDescriptors()   - 
Method in class weka.gui.beans. ScatterPlotMatrixBeanInfo 
 Get the event set descriptors for this bean
   getEventSetDescriptors()   - 
Method in class weka.gui.beans. SerializedModelSaverBeanInfo 
 Get the event set descriptors for this bean
   getEventSetDescriptors()   - 
Method in class weka.gui.beans. StripChartBeanInfo 
 Get the event set descriptors for this bean
   getEventSetDescriptors()   - 
Method in class weka.gui.beans. TextViewerBeanInfo 
 Get the event set descriptors for this bean
   getEvidence(int)   - 
Method in class weka.classifiers.bayes.net. EditableBayesNet 
 get evidence state of a node.
   getException()   - 
Method in class weka.gui.sql.event. ConnectionEvent 
 returns the stored exception, if any (can be NULL)
   getException()   - 
Method in class weka.gui.sql.event. QueryExecuteEvent 
 returns the exception, if one happened, otherwise NULL
   getExclusive()   - 
Method in class weka.classifiers.rules. ConjunctiveRule 
 Returns whether exclusive expressions for nominal attributes splits are 
 considered
   getExecutionSlots()   - 
Method in class weka.gui.beans. Classifier 
 Get the number of execution slots (threads) used
 to train models.
   getExecutionStatus()   - 
Method in class weka.experiment. TaskStatusInfo 
 Get the execution status of this Task.
   getExitIfNoWindowsOpen()   - 
Static method in class weka.gui.boundaryvisualizer. BoundaryVisualizer 
 Gets whether System.exit gets called after the last window gets closed
   getExitOnClose()   - 
Method in class weka.gui.arffviewer. ArffViewer 
 returns TRUE if a System.exit(0) is done on a close
   getExitOnClose()   - 
Method in class weka.gui.arffviewer. ArffViewerMainPanel 
 returns TRUE if a System.exit(0) is done on a close
   getExpectedFrequency()   - 
Method in class weka.associations.tertius. Rule 
 Get the expected frequency of counter-instances of this rule.
   getExpectedNumber()   - 
Method in class weka.associations.tertius. Rule 
  
   getExpectedResultsPerAverage()   - 
Method in class weka.experiment. AveragingResultProducer 
 Get the value of ExpectedResultsPerAverage.
   getExperiment()   - 
Method in class weka.experiment. RemoteExperimentSubTask 
 Get the experiment for this sub task
   getExperiment()   - 
Method in class weka.gui.experiment. SetupModePanel 
 Gets the currently configured experiment.
   getExperiment()   - 
Method in class weka.gui.experiment. SetupPanel 
 Gets the currently configured experiment.
   getExperiment()   - 
Method in class weka.gui.experiment. SimpleSetupPanel 
 Gets the currently configured experiment.
   getExperimentType()   - 
Static method in class weka.gui.experiment. ExperimenterDefaults 
 returns the default experiment type
   getExplicitPropsFile()   - 
Method in class weka.gui. GenericPropertiesCreator 
 returns TRUE, if a file is loaded and not the Utils class used for 
 locating the props file.
   getExplorer()   - 
Method in class weka.gui.explorer. AssociationsPanel 
 returns the parent Explorer frame
   getExplorer()   - 
Method in class weka.gui.explorer. AttributeSelectionPanel 
 returns the parent Explorer frame
   getExplorer()   - 
Method in class weka.gui.explorer. ClassifierPanel 
 returns the parent Explorer frame
   getExplorer()   - 
Method in class weka.gui.explorer. ClustererPanel 
 returns the parent Explorer frame
   getExplorer()   - 
Method in interface weka.gui.explorer. Explorer.ExplorerPanel 
 returns the parent Explorer frame
   getExplorer()   - 
Method in class weka.gui.explorer. PreprocessPanel 
 returns the parent Explorer frame
   getExplorer()   - 
Method in class weka.gui.explorer. VisualizePanel 
 returns the parent Explorer frame
   getExponent()   - 
Method in class weka.classifiers.functions.supportVector. PolyKernel 
 Gets the exponent value.
   getExponent()   - 
Method in class weka.classifiers.functions. VotedPerceptron 
 Get the value of exponent.
   getExpression(Node, FieldMetaInfo.Optype, ArrayList<Attribute>, TransformationDictionary)   - 
Static method in class weka.core.pmml. Expression 
 Static factory method that returns a subclass of Expression that
 encapsulates the type of expression contained in the Element
 supplied.
   getExpression(String, Node, FieldMetaInfo.Optype, ArrayList<Attribute>, TransformationDictionary)   - 
Static method in class weka.core.pmml. Expression 
 Static factory method that returns a subclass of Expression that
 encapsulates the type of expression supplied as an argument.
   getExpression()   - 
Method in class weka.datagenerators.classifiers.regression. Expression 
 Gets the mathematical expression for generating y out of x
   getExpression()   - 
Method in class weka.filters.unsupervised.attribute. AddExpression 
 Get the expression
   getExpression()   - 
Method in class weka.filters.unsupervised.attribute. MathExpression 
 Get the expression
   getExpression()   - 
Method in class weka.filters.unsupervised.instance. SubsetByExpression 
 Returns the expression used for filtering.
   getExtension()   - 
Static method in class weka.gui.experiment. ExperimenterDefaults 
 returns the default experiment extension
   getExtension()   - 
Method in class weka.gui.visualize. BMPWriter 
 returns the extension (incl.
   getExtension()   - 
Method in class weka.gui.visualize. JComponentWriter 
 returns the extension (incl.
   getExtension()   - 
Method in class weka.gui.visualize. JPEGWriter 
 returns the extension (incl.
   getExtension()   - 
Method in class weka.gui.visualize. PNGWriter 
 returns the extension (incl.
   getExtension()   - 
Method in class weka.gui.visualize. PostscriptWriter 
 returns the extension (incl.
   getExtensions()   - 
Method in class weka.gui. ExtensionFileFilter 
 Returns a copy of the acceptable extensions.
   getExtremeValuesAsOutliers()   - 
Method in class weka.filters.unsupervised.attribute. InterquartileRange 
 Get whether extreme values are also tagged as outliers.
   getExtremeValuesFactor()   - 
Method in class weka.filters.unsupervised.attribute. InterquartileRange 
 Gets the factor for determining the thresholds for extreme values.
   getFactory()   - 
Method in class weka.core.xml. XMLDocument 
 returns the DocumentBuilderFactory.
   getFailReason()   - 
Method in class weka.core. Capabilities 
 returns the reason why the tests failed, is null if tests succeeded
   getFallout()   - 
Method in class weka.classifiers.evaluation. TwoClassStats 
 Calculate the fallout.
   getFalseNegative()   - 
Method in class weka.classifiers.evaluation. TwoClassStats 
 Gets the number of positive instances predicted as negative
   getFalsePositive()   - 
Method in class weka.classifiers.evaluation. TwoClassStats 
 Gets the number of negative instances predicted as positive
   getFalsePositiveRate()   - 
Method in class weka.classifiers.evaluation. TwoClassStats 
 Calculate the false positive rate.
   getFastRegression()   - 
Method in class weka.classifiers.trees. LMT 
 Get the value of fastRegression.
   getFieldAsAttribute()   - 
Method in class weka.core.pmml. DerivedFieldMetaInfo 
 Get this derived field as an Attribute.
   getFieldAsAttribute()   - 
Method in class weka.core.pmml. FieldMetaInfo 
 Return this field as an Attribute.
   getFieldAsAttribute()   - 
Method in class weka.core.pmml. MiningFieldMetaInfo 
 Return this mining field as an Attribute.
   getFieldAsAttribute()   - 
Method in class weka.core.pmml. TargetMetaInfo 
 Return this field as an Attribute.
   getFieldDef(String)   - 
Method in class weka.core.pmml. Expression 
 Return the named attribute from the list of reference fields.
   getFieldDefIndex(String)   - 
Method in class weka.core.pmml. Expression 
  
   getFieldName()   - 
Method in class weka.core.pmml. FieldMetaInfo 
 Get the name of this field.
   getFieldsAsInstances()   - 
Method in class weka.core.pmml. MiningSchema 
 Get the all the fields (both mining schema and derived) as Instances.
   getFieldsMappingString()   - 
Method in class weka.classifiers.pmml.consumer. PMMLClassifier 
 Get a textual description of the mapping between mining schema
 fields and incoming data fields.
   getFieldsMappingString()   - 
Method in class weka.core.pmml. MappingInfo 
 Get a textual description of them mapping between mining schema
 fields and incoming data fields.
   getFile()   - 
Method in class weka.gui.visualize. JComponentWriter 
 returns the file being used for storing the output
   getFileDescription()   - 
Method in class weka.core.converters. AbstractFileSaver 
 to be pverridden
   getFileDescription()   - 
Method in class weka.core.converters. ArffLoader 
 Returns a description of the file type.
   getFileDescription()   - 
Method in class weka.core.converters. ArffSaver 
 Returns a description of the file type.
   getFileDescription()   - 
Method in class weka.core.converters. C45Loader 
 Returns a description of the file type.
   getFileDescription()   - 
Method in class weka.core.converters. C45Saver 
 Returns a description of the file type.
   getFileDescription()   - 
Method in class weka.core.converters. CSVLoader 
 Returns a description of the file type.
   getFileDescription()   - 
Method in class weka.core.converters. CSVSaver 
 Returns a description of the file type.
   getFileDescription()   - 
Method in interface weka.core.converters. FileSourcedConverter 
 Get a one line description of the type of file
   getFileDescription()   - 
Method in class weka.core.converters. LibSVMLoader 
 Returns a description of the file type.
   getFileDescription()   - 
Method in class weka.core.converters. LibSVMSaver 
 Returns a description of the file type.
   getFileDescription()   - 
Method in class weka.core.converters. SerializedInstancesLoader 
 Returns a description of the file type.
   getFileDescription()   - 
Method in class weka.core.converters. SerializedInstancesSaver 
 Returns a description of the file type.
   getFileDescription()   - 
Method in class weka.core.converters. SVMLightLoader 
 Returns a description of the file type.
   getFileDescription()   - 
Method in class weka.core.converters. SVMLightSaver 
 Returns a description of the file type.
   getFileDescription()   - 
Method in class weka.core.converters. TextDirectoryLoader 
 Returns a description of the file type, actually it's directories.
   getFileDescription()   - 
Method in class weka.core.converters. XRFFLoader 
 Returns a description of the file type.
   getFileDescription()   - 
Method in class weka.core.converters. XRFFSaver 
 Returns a description of the file type.
   getFileExtension()   - 
Method in class weka.core.converters. AbstractFileSaver 
 Gets ihe file extension.
   getFileExtension()   - 
Method in class weka.core.converters. AbstractSaver 
 Default implementation throws an IOException.
   getFileExtension()   - 
Method in class weka.core.converters. ArffLoader 
 Get the file extension used for arff files
   getFileExtension()   - 
Method in class weka.core.converters. C45Loader 
 Get the file extension used for arff files
   getFileExtension()   - 
Method in class weka.core.converters. CSVLoader 
 Get the file extension used for arff files.
   getFileExtension()   - 
Method in interface weka.core.converters. FileSourcedConverter 
 Get the file extension used for this type of file
   getFileExtension()   - 
Method in class weka.core.converters. LibSVMLoader 
 Get the file extension used for libsvm files.
   getFileExtension()   - 
Method in interface weka.core.converters. Saver 
 Gets the file extension
   getFileExtension()   - 
Method in class weka.core.converters. SerializedInstancesLoader 
 Get the file extension used for arff files
   getFileExtension()   - 
Method in class weka.core.converters. SVMLightLoader 
 Get the file extension used for svm light files.
   getFileExtension()   - 
Method in class weka.core.converters. XRFFLoader 
 Get the file extension used for libsvm files
   getFileExtensions()   - 
Method in class weka.core.converters. AbstractFileSaver 
 Gets all the file extensions used for this type of file
   getFileExtensions()   - 
Method in class weka.core.converters. ArffLoader 
 Gets all the file extensions used for this type of file
   getFileExtensions()   - 
Method in class weka.core.converters. ArffSaver 
 Gets all the file extensions used for this type of file
   getFileExtensions()   - 
Method in class weka.core.converters. C45Loader 
 Gets all the file extensions used for this type of file
   getFileExtensions()   - 
Method in class weka.core.converters. CSVLoader 
 Gets all the file extensions used for this type of file.
   getFileExtensions()   - 
Method in interface weka.core.converters. FileSourcedConverter 
 Gets all the file extensions used for this type of file
   getFileExtensions()   - 
Method in class weka.core.converters. LibSVMLoader 
 Gets all the file extensions used for this type of file.
   getFileExtensions()   - 
Method in class weka.core.converters. SerializedInstancesLoader 
 Gets all the file extensions used for this type of file
   getFileExtensions()   - 
Method in class weka.core.converters. SVMLightLoader 
 Gets all the file extensions used for this type of file.
   getFileExtensions()   - 
Method in class weka.core.converters. XRFFLoader 
 Gets all the file extensions used for this type of file
   getFileExtensions()   - 
Method in class weka.core.converters. XRFFSaver 
 Gets all the file extensions used for this type of file
   getFileFormat()   - 
Method in class weka.gui.beans. SerializedModelSaver 
 Get the file format to use for saving.
   getFileLoaders()   - 
Static method in class weka.core.converters. ConverterUtils 
 returns a vector with the classnames of all the file loaders.
   getFileMatches(String)   - 
Method in class weka.gui. SimpleCLIPanel.CommandlineCompletion 
 returns all the file/dir matches with the partial search string.
   getFileMustExist()   - 
Method in class weka.gui. ConverterFileChooser 
 Returns whether the selected file must exist (only open dialog).
   getFileName()   - 
Method in class weka.classifiers.bayes.net. BIFReader 
 returns the current filename
   getFilename()   - 
Method in class weka.core. Debug.Log 
 returns the filename of the log, can be null
   getFilename()   - 
Method in class weka.core. Debug.SimpleLog 
 returns the filename of the log, can be null
   getFilename()   - 
Method in class weka.core. FindWithCapabilities 
 returns the current filename for the dataset to base the capabilities on.
   getFilename()   - 
Method in class weka.gui.arffviewer. ArffPanel 
 returns the filename
   getFilename(int)   - 
Method in class weka.gui.arffviewer. ArffViewerMainPanel 
 returns the filename of the specified panel
   getFileSavers()   - 
Static method in class weka.core.converters. ConverterUtils 
 returns a vector with the classnames of all the file savers.
   getFillWithMissing()   - 
Method in class weka.filters.unsupervised.attribute. AbstractTimeSeries 
 Gets whether missing values should be used rather than removing instances
 where the translated value is not known (due to border effects).
   getFilter()   - 
Method in class weka.associations. FilteredAssociator 
 Gets the filter used.
   getFilter()   - 
Method in class weka.attributeSelection. FilteredAttributeEval 
 Get the filter to use
   getFilter()   - 
Method in class weka.attributeSelection. FilteredSubsetEval 
 Get the filter to use
   getFilter()   - 
Method in class weka.classifiers.functions. PLSClassifier 
 Get the PLS filter.
   getFilter()   - 
Method in class weka.classifiers.meta. FilteredClassifier 
 Gets the filter used.
   getFilter()   - 
Method in class weka.classifiers.meta. GridSearch 
 Get the kernel filter.
   getFilter()   - 
Method in class weka.clusterers. FilteredClusterer 
 Gets the filter used.
   getFilter()   - 
Method in class weka.filters. CheckSource 
 Gets the filter being used for the tests, can be null.
   getFilter(int)   - 
Method in class weka.filters. MultiFilter 
 Gets a single filter from the set of available filters.
   getFilter(int)   - 
Method in class weka.filters.unsupervised.attribute. PartitionedMultiFilter 
 Gets a single filter from the set of available filters.
   getFilter()   - 
Method in class weka.filters.unsupervised.attribute. Wavelet 
 Get the preprocessing filter.
   getFilter()   - 
Method in class weka.gui.beans. Filter 
  
   getFilter()   - 
Method in class weka.gui.explorer. Explorer.CapabilitiesFilterChangeEvent 
 returns the associated Capabilities filter
   getFilter()   - 
Static method in class weka.gui.explorer. ExplorerDefaults 
 returns the default filter (fully configured) for the preprocess panel.
   getFilterAfterFirstBatch()   - 
Method in class weka.filters.unsupervised.instance. SubsetByExpression 
 Get whether to apply the filter to instances that arrive once
 the first (training) batch has been seen.
   getFilterAttributes()   - 
Method in class weka.associations. GeneralizedSequentialPatterns 
 Returns the String containing the attributes which are used for output 
 filtering.
   getFiltered(int)   - 
Method in class weka.classifiers.rules. RuleStats 
 Get the data after filtering the given rule
   getFilters()   - 
Method in class weka.filters. MultiFilter 
 Gets the list of possible filters to choose from.
   getFilters()   - 
Method in class weka.filters.unsupervised.attribute. PartitionedMultiFilter 
 Gets the list of possible filters to choose from.
   getFilterType()   - 
Method in class weka.attributeSelection. SVMAttributeEval 
 Get the filtering mode passed to SMO
   getFilterType()   - 
Method in class weka.classifiers.functions. GaussianProcesses 
 Gets how the training data will be transformed.
   getFilterType()   - 
Method in class weka.classifiers.functions. SMO 
 Gets how the training data will be transformed.
   getFilterType()   - 
Method in class weka.classifiers.functions. SMOreg 
 Gets how the training data will be transformed.
   getFilterType()   - 
Method in class weka.classifiers.mi. MDD 
 Gets how the training data will be transformed.
   getFilterType()   - 
Method in class weka.classifiers.mi. MIDD 
 Gets how the training data will be transformed.
   getFilterType()   - 
Method in class weka.classifiers.mi. MIEMDD 
 Gets how the training data will be transformed.
   getFilterType()   - 
Method in class weka.classifiers.mi. MIOptimalBall 
 Gets how the training data will be transformed.
   getFilterType()   - 
Method in class weka.classifiers.mi. MISMO 
 Gets how the training data will be transformed.
   getFilterType()   - 
Method in class weka.classifiers.mi. MISVM 
 Gets how the training data will be transformed.
   getFindAllRulesForSupportLevel()   - 
Method in class weka.associations. FPGrowth 
 Get whether all rules meeting the lower bound on min support
 and the minimum metric threshold are to be found.
   getFindNumBins()   - 
Method in class weka.filters.unsupervised.attribute. Discretize 
 Get the value of FindNumBins.
   getFindNumBins()   - 
Method in class weka.filters.unsupervised.attribute. PKIDiscretize 
 Get the value of FindNumBins.
   getFirst()   - 
Method in class weka.associations.tertius. SimpleLinkedList 
  
   getFirstToken(StreamTokenizer)   - 
Static method in class weka.core.converters. ConverterUtils 
 Gets token, skipping empty lines.
   getFirstValueIndex()   - 
Method in class weka.filters.unsupervised.attribute. MergeTwoValues 
 Get the index of the first value used.
   getFirstValueIndex()   - 
Method in class weka.filters.unsupervised.attribute. SwapValues 
 Get the index of the first value used.
   getFlag(char, String[])   - 
Static method in class weka.core. Utils 
 Checks if the given array contains the flag "-Char".
   getFlag(String, String[])   - 
Static method in class weka.core. Utils 
 Checks if the given array contains the flag "-String".
   getFlow()   - 
Method in class weka.gui.beans. KnowledgeFlowApp 
 Gets the current flow being edited.
   getFlows()   - 
Method in class weka.gui.beans. FlowRunner 
 Get the vector holding the flow(s)
   getFMeasure()   - 
Method in class weka.classifiers.evaluation. TwoClassStats 
 Calculate the F-Measure.
   getFold()   - 
Method in class weka.filters.supervised.instance. StratifiedRemoveFolds 
 Gets the fold which is selected.
   getFold()   - 
Method in class weka.filters.unsupervised.instance. RemoveFolds 
 Gets the fold which is selected.
   getFoldColumn()   - 
Method in class weka.experiment. PairedTTester 
 Get the value of FoldColumn.
   getFoldColumn()   - 
Method in interface weka.experiment. Tester 
 Get the value of FoldColumn.
   getFolds()   - 
Method in class weka.attributeSelection. OneRAttributeEval 
 Get the number of folds used for cross validation
   getFolds()   - 
Method in class weka.attributeSelection. WrapperSubsetEval 
 Get the number of folds used for accuracy estimation
   getFolds()   - 
Method in class weka.classifiers.rules. ConjunctiveRule 
 returns the current number of folds
   getFolds()   - 
Method in class weka.classifiers.rules. JRip 
 Gets the number of folds
   getFolds()   - 
Method in class weka.classifiers.rules. Ridor 
  
   getFolds()   - 
Method in class weka.gui.beans. CrossValidationFoldMaker 
 Get the currently set number of folds
   getFolds()   - 
Static method in class weka.gui.experiment. ExperimenterDefaults 
 returns the number of folds used for cross-validation
   getFoldsType()   - 
Method in class weka.attributeSelection. RaceSearch 
 Get the xfold type
   getFont()   - 
Method in class weka.gui.visualize. PostscriptGraphics 
 Get current font.
   getFontMetrics(Font)   - 
Method in class weka.gui.visualize. PostscriptGraphics 
 Get Font metrics
   getFontRenderContext()   - 
Method in class weka.gui.visualize. PostscriptGraphics 
 START overridden Graphics2D methods
   getFormat()   - 
Method in class weka.core. Debug.Timestamp 
 returns the current timestamp format
   getForwardSelectionMethod()   - 
Method in class weka.attributeSelection. LinearForwardSelection 
 Get the search direction
   getFPRate()   - 
Method in class weka.associations.tertius. Rule 
 Get the rate of False Positive instances of this rule.
   getFrameLocation()   - 
Method in class weka.gui. MemoryUsagePanel 
 Returns the default position for the dialog.
   getFrameTitle()   - 
Method in class weka.gui.arffviewer. ArffViewerMainPanel 
 returns the title (incl.
   getFrequency()   - 
Method in class weka.associations. FPGrowth.BinaryItem 
 Get the frequency of this item.
   getFrequencyLimit()   - 
Method in class weka.classifiers.bayes. AODE 
 Gets the frequency limit.
   getFrequencyLimit()   - 
Method in class weka.classifiers.bayes. AODEsr 
 Gets the frequency limit.
   getFrequencyThreshold()   - 
Method in class weka.associations. Tertius 
 Get the value of frequencyThreshold.
   getFreshCardinalityOfParents(Instances)   - 
Method in class weka.classifiers.bayes.net. ParentSet 
 returns cardinality of parents after recalculation
   getFromYear()   - 
Static method in class weka.core. Copyright 
 returns the start year of the copyright
   getFunction(String)   - 
Static method in class weka.core.pmml. Function 
 Get a built-in PMML Function.
   getFunction(String, TransformationDictionary)   - 
Static method in class weka.core.pmml. Function 
 Get either a function.
   getFunction()   - 
Method in class weka.datagenerators.classifiers.classification. Agrawal 
 Gets the function for generating the data.
   getFunctionValue(int)   - 
Method in class weka.classifiers.functions.pace. DiscreteFunction 
 Gets a particular function value
   getGamma()   - 
Method in class weka.classifiers.functions. LibSVM 
 Gets gamma
   getGamma()   - 
Method in class weka.classifiers.functions.supportVector. RBFKernel 
 Gets the gamma value.
   getGCount(Node, int)   - 
Static method in class weka.gui.treevisualizer. Node 
 Recursively finds the number of visible groups of siblings there are.
   getGenerateRanking()   - 
Method in class weka.attributeSelection. GreedyStepwise 
 Gets whether ranking has been requested.
   getGenerateRanking()   - 
Method in class weka.attributeSelection. RaceSearch 
 Gets whether ranking has been requested.
   getGenerateRanking()   - 
Method in interface weka.attributeSelection. RankedOutputSearch 
 Gets whether the user has opted to see a ranked list of
 attributes rather than the normal result of the search
   getGenerateRanking()   - 
Method in class weka.attributeSelection. Ranker 
 This is a dummy method.
   getGenerator()   - 
Method in class weka.gui.explorer. DataGeneratorPanel 
 returns the currently selected DataGenerator
   getGeneratorSamplesBase()   - 
Method in class weka.gui.boundaryvisualizer. BoundaryPanel 
 Get the base used for computing the number of samples to obtain from
 each generator
   getGlobalBlend()   - 
Method in class weka.classifiers.lazy. KStar 
 Get the value of the global blend parameter
   getGlobalInfo(Object)   - 
Static method in class weka.gui.beans. KnowledgeFlowApp 
 Utility method for grabbing the global info help (if it exists) from
 an arbitrary object
   getGlobalModel()   - 
Method in class weka.classifiers.trees.j48. NBTreeSplit 
 Return the global naive bayes model for this node
   getGraphString()   - 
Method in class weka.gui.beans. GraphEvent 
 Return the dot string for the graph
   getGraphTitle()   - 
Method in class weka.gui.beans. GraphEvent 
 Return the graph title
   getGraphType()   - 
Method in class weka.gui.beans. GraphEvent 
 Return the graph type
   getGridExtensionsPerformed()   - 
Method in class weka.classifiers.meta. GridSearch 
 returns the number of grid extensions that took place during the search
 (only applicable if the grid was extendable).
   getGridIsExtendable()   - 
Method in class weka.classifiers.meta. GridSearch 
 Get whether the grid can be extended dynamically.
   getGridWidth()   - 
Method in class weka.gui.beans. AttributeSummarizer 
 Get the width of the grid of plots
   getGroupIdentifier()   - 
Method in class weka.gui.beans. BatchClassifierEvent 
  
   getGUI()   - 
Method in class weka.classifiers.functions. MultilayerPerceptron 
  
   getGUIType()   - 
Method in class weka.gui. Main 
 Gets the currently set type of GUI to display.
   getH()   - 
Method in class weka.core.matrix. QRDecomposition 
 Return the Householder vectors
   getHandler()   - 
Method in class weka.core. FindWithCapabilities 
 returns the current set CapabilitiesHandler to generate the dataset
 for, can be null.
   getHandler()   - 
Method in class weka.core. TestInstances 
 returns the current set CapabilitiesHandler to generate the dataset
 for, can be null
   getHashtable(FastVector, int)   - 
Static method in class weka.associations. ItemSet 
 Return a hashtable filled with the given item sets.
   getHashtable(FastVector, int)   - 
Static method in class weka.associations. LabeledItemSet 
 Return a hashtable filled with the given item sets.
   getHDRank()   - 
Method in class weka.classifiers.mi. CitationKNN 
 Returns the rank associated to the Hausdorff distance
   getHeader(String)   - 
Method in class weka.experiment. ResultMatrix 
 returns the value associated with the given key, null if if cannot be
 found
   getHeight()   - 
Method in class weka.gui.beans. BeanInstance 
 Gets the height of this bean
   getHeight(Node, int)   - 
Static method in class weka.gui.treevisualizer. Node 
 Recursively finds the number of visible levels there are.
   getHeuristic()   - 
Method in class weka.classifiers.trees. BFTree 
 Get if use heuristic search for nominal attributes in multi-class problems.
   getHeuristic()   - 
Method in class weka.classifiers.trees. SimpleCart 
 Get if use heuristic search for nominal attributes in multi-class problems.
   getHeuristicStop()   - 
Method in class weka.classifiers.functions. SimpleLogistic 
 Get the value of heuristicStop.
   getHiddenLayers()   - 
Method in class weka.classifiers.functions. MultilayerPerceptron 
  
   getHistory()   - 
Method in class weka.gui.sql. ConnectionPanel 
 returns the history.
   getHistory()   - 
Method in class weka.gui.sql.event. HistoryChangedEvent 
 returns the history model
   getHistory()   - 
Method in class weka.gui.sql. QueryPanel 
 returns the history.
   getHistoryName()   - 
Method in class weka.gui.sql.event. HistoryChangedEvent 
 returns the name of the history
   getHoldOutFile()   - 
Method in class weka.attributeSelection. ClassifierSubsetEval 
 Gets the file that holds hold out/test instances.
   getHomeDir()   - 
Static method in class weka.core. Debug 
 returns the home directory of the user
   getHornClauses()   - 
Method in class weka.associations. Tertius 
 Get the value of hornClauses.
   getHyperparameterRange()   - 
Method in class weka.classifiers.bayes. BayesianLogisticRegression 
 Get the range of hyperparameter values to consider
 during CV-based selection.
   getHyperparameterSelection()   - 
Method in class weka.classifiers.bayes. BayesianLogisticRegression 
 Get the method used to select the hyperparameter
   getHyperparameterValue()   - 
Method in class weka.classifiers.bayes. BayesianLogisticRegression 
 Get the hyperparameter value.
   getIconPath()   - 
Method in class weka.gui.beans. BeanVisual 
 returns the path for the icon
   getId()   - 
Method in class weka.classifiers.functions.neural. NeuralConnection 
  
   getID()   - 
Method in class weka.core. Debug.Random 
 returns the unique ID of this number generator
   getID()   - 
Method in class weka.core. Tag 
 Gets the numeric ID of the Tag.
   getID()   - 
Method in class weka.core. TechnicalInformation 
 returns the unique ID (either the one used in creating this instance
 or the automatically generated one)
   getID()   - 
Method in class weka.gui.streams. InstanceEvent 
 Get the event type
   getID()   - 
Method in class weka.gui.treevisualizer. TreeDisplayEvent 
  
   getIDFTransform()   - 
Method in class weka.filters.unsupervised.attribute. StringToWordVector 
 Sets whether if the word frequencies in a document should be transformed
 into:  
 fij*log(num of Docs/num of Docs with word i)  
      where fij is the frequency of word i in document(instance) j.
   getIDIndex()   - 
Method in class weka.filters.unsupervised.attribute. AddID 
 Get the index of the attribute used.
   getIDStr()   - 
Method in class weka.core. Tag 
 Gets the string ID of the Tag.
   getIgnoreClass()   - 
Method in class weka.filters.unsupervised.attribute. PotentialClassIgnorer 
 Gets the IgnoreClass value.
   getIgnoredAttributeIndices()   - 
Method in class weka.filters.unsupervised.attribute. AddCluster 
 Gets ranges of attributes to be ignored.
   getIgnoredAttributeIndices()   - 
Method in class weka.filters.unsupervised.attribute. ClusterMembership 
 Gets ranges of attributes to be ignored.
   getIgnoredProperties()   - 
Method in class weka.core. CheckGOE 
 Get the ignored properties used in checkToolTips() as comma-separated 
 list (sorted).
   getIgnoreRange()   - 
Method in class weka.filters.unsupervised.attribute. MathExpression 
 Get the current range selection.
   getImage(String, String)   - 
Static method in class weka.gui. ComponentHelper 
 returns the Image for a given directory and filename, NULL if not successful
   getImage(String)   - 
Static method in class weka.gui. ComponentHelper 
 returns the Image for a given filename, NULL if not successful
   getImageIcon(String, String)   - 
Static method in class weka.gui. ComponentHelper 
 returns the ImageIcon for a given filename and directory, NULL if not successful
   getImageIcon(String)   - 
Static method in class weka.gui. ComponentHelper 
 returns the ImageIcon for a given filename, NULL if not successful
   getImagEigenvalues()   - 
Method in class weka.core.matrix. EigenvalueDecomposition 
 Return the imaginary parts of the eigenvalues
   getIncludeClass()   - 
Method in class weka.core. InstanceComparator 
 returns TRUE if the class is included in the comparison
   getIncludeClass()   - 
Method in class weka.filters.unsupervised.attribute. NumericCleaner 
 Gets whether the class is included in the cleaning process or always 
 skipped.
   getIndex()   - 
Method in class weka.associations.tertius. Predicate 
  
   getIndex()   - 
Method in class weka.core. PropertyPath.PathElement 
 returns the index of the property, -1 if the property is not an
 index-based one
   getIndex()   - 
Method in class weka.core. SingleIndex 
 Gets the selected index
   getIndex()   - 
Method in class weka.gui. SortedTableModel.SortContainer 
 Returns the original index of the item.
   getIndexofBiggest(List<Integer>)   - 
Method in class weka.attributeSelection. ScatterSearchV1 
 get the index in a List where this have the biggest number
   getInitAsNaiveBayes()   - 
Method in class weka.classifiers.bayes.net.search.global. HillClimber 
 Gets whether to init as naive bayes
   getInitAsNaiveBayes()   - 
Method in class weka.classifiers.bayes.net.search.global. K2 
 Gets whether to init as naive bayes
   getInitAsNaiveBayes()   - 
Method in class weka.classifiers.bayes.net.search.local. HillClimber 
 Gets whether to init as naive bayes
   getInitAsNaiveBayes()   - 
Method in class weka.classifiers.bayes.net.search.local. K2 
 Gets whether to init as naive bayes
   getInitFile()   - 
Method in class weka.filters.unsupervised.attribute. KernelFilter 
 Gets the file to initialize the filter with, can be null.
   getInitFileClassIndex()   - 
Method in class weka.filters.unsupervised.attribute. KernelFilter 
 Gets the class index of the file to initialize the filter with.
   getInitGenericObjectEditorFilter()   - 
Static method in class weka.gui.explorer. ExplorerDefaults 
 returns if the GOEs in the Explorer will be initialized based on the
 data that is loaded into the Explorer.
   getInitial()   - 
Method in class weka.core. Memory 
 returns the initial size of the JVM
   getInitialDatasetsDirectory()   - 
Static method in class weka.gui.experiment. ExperimenterDefaults 
 returns the initial directory for the datasets (if empty, it returns
 the user's home directory)
   getInitialDirectory()   - 
Static method in class weka.gui.explorer. ExplorerDefaults 
 Returns the initial directory for the file chooser used for opening
 datasets.
   getInputCenterFile()   - 
Method in class weka.clusterers. XMeans 
 Gets the file to read the list of centers from.
   getInputFilename()   - 
Method in class weka.gui. GenericPropertiesCreator 
 returns the name of the input file
   getInputNums()   - 
Method in class weka.classifiers.functions.neural. NeuralConnection 
 Use this to get easy access to the input numbers.
   getInputOrder()   - 
Method in class weka.datagenerators.clusterers. BIRCHCluster 
 Gets the input order.
   getInputProperties()   - 
Method in class weka.gui. GenericPropertiesCreator 
 returns the input properties object (template containing the packages)
   getInputs()   - 
Method in class weka.classifiers.functions.neural. NeuralConnection 
 Use this to get easy access to the inputs.
   getInputs()   - 
Method in class weka.gui.beans. MetaBean 
  
   getInputStream(String, String)   - 
Static method in class weka.gui. Loader 
 returns an InputStream for the given dir and filename, can be NULL if it 
 fails
   getInputStream(String)   - 
Method in class weka.gui. Loader 
 returns an InputStream for the given filename, can be NULL if it fails
   getInstalledLookAndFeels()   - 
Static method in class weka.gui. LookAndFeel 
 returns an array with the classnames of all the installed LnFs
   getInstance()   - 
Static method in class weka.associations.gsp. Messages 
 getInstance.
   getInstance()   - 
Static method in class weka.associations. Messages 
 getInstance.
   getInstance()   - 
Method in interface weka.clusterers.forOPTICSAndDBScan.DataObjects. DataObject 
 Returns the original instance
   getInstance()   - 
Method in class weka.clusterers.forOPTICSAndDBScan.DataObjects. EuclidianDataObject 
 Returns the original instance
   getInstance()   - 
Method in class weka.clusterers.forOPTICSAndDBScan.DataObjects. ManhattanDataObject 
 Returns the original instance
   getInstance()   - 
Static method in class weka.gui.arffviewer. Messages 
 getInstance.
   getInstance()   - 
Method in class weka.gui.beans. InstanceEvent 
 Get the instance
   getInstance()   - 
Static method in class weka.gui.beans. Messages 
 getInstance.
   getInstance()   - 
Static method in class weka.gui.beans.xml. Messages 
 getInstance.
   getInstance()   - 
Static method in class weka.gui.boundaryvisualizer. Messages 
 getInstance.
   getInstance()   - 
Static method in class weka.gui.experiment. Messages 
 getInstance.
   getInstance()   - 
Static method in class weka.gui.explorer. Messages 
 getInstance.
   getInstance()   - 
Static method in class weka.gui.graphvisualizer. Messages 
 getInstance.
   getInstance()   - 
Static method in class weka.gui.hierarchyvisualizer. Messages 
 getInstance.
   getInstance()   - 
Static method in class weka.gui. Messages 
 getInstance.
   getInstance()   - 
Static method in class weka.gui.sql.event. Messages 
 getInstance.
   getInstance()   - 
Static method in class weka.gui.sql. Messages 
 getInstance.
   getInstance()   - 
Static method in class weka.gui.streams. Messages 
 getInstance.
   getInstance()   - 
Static method in class weka.gui.treevisualizer. Messages 
 getInstance.
   getInstance()   - 
Static method in class weka.gui.visualize. Messages 
 getInstance.
   getInstanceIndex(int)   - 
Method in class weka.classifiers.lazy. LBR.Indexes 
 Returns the boolean value at the specified index in the Instance Index array
   getInstanceRange()   - 
Method in class weka.filters.unsupervised.attribute. AbstractTimeSeries 
 Gets the number of instances forward to translate values between.
   getInstances()   - 
Method in interface weka.clusterers.forOPTICSAndDBScan.Databases. Database 
 Returns the original instances delivered from WEKA
   getInstances()   - 
Method in class weka.clusterers.forOPTICSAndDBScan.Databases. SequentialDatabase 
 Returns the original instances delivered from WEKA
   getInstances()   - 
Method in class weka.core.converters. AbstractSaver 
 Gets instances that should be stored.
   getInstances()   - 
Method in interface weka.core. DistanceFunction 
 returns the instances currently set.
   getInstances()   - 
Method in class weka.core.neighboursearch. NearestNeighbourSearch 
 returns the instances currently set.
   getInstances()   - 
Method in class weka.core. NormalizableDistance 
 returns the instances currently set.
   getInstances()   - 
Method in class weka.core.xml. XMLInstances 
 returns the current instances, either the ones that were set or the ones
 that were generated from the XML structure.
   getInstances()   - 
Method in class weka.experiment. PairedTTester 
 Get the value of Instances.
   getInstances()   - 
Method in interface weka.experiment. Tester 
 Get the value of Instances.
   getInstances()   - 
Method in class weka.gui.arffviewer. ArffPanel 
 returns the instances of the panel, if none then NULL
   getInstances()   - 
Method in class weka.gui.arffviewer. ArffSortedTableModel 
 returns the data
   getInstances()   - 
Method in class weka.gui.arffviewer. ArffTableModel 
 returns the data
   getInstances()   - 
Method in class weka.gui.boundaryvisualizer. BoundaryVisualizer 
 Get the training instances
   getInstances()   - 
Method in class weka.gui.explorer. DataGeneratorPanel 
 returns the generated instances, null if the process was cancelled.
   getInstances()   - 
Method in class weka.gui.explorer. PreprocessPanel 
 Gets the working set of instances.
   getInstances()   - 
Method in class weka.gui. SetInstancesPanel 
 Gets the set of instances currently held by the panel
   getInstances()   - 
Method in class weka.gui.treevisualizer. Node 
 This will return the Instances object related to this node.
   getInstances()   - 
Method in class weka.gui. ViewerDialog 
 returns the currently displayed instances
   getInstances()   - 
Method in class weka.gui.visualize. VisualizePanel 
 Get the master plot's instances
   getInstances1()   - 
Method in class weka.gui.visualize. VisualizePanelEvent 
  
   getInstances2()   - 
Method in class weka.gui.visualize. VisualizePanelEvent 
  
   getInstancesFromClass(Instances, int, int, double, Instances)   - 
Static method in class weka.estimators. EstimatorUtils 
 Returns a dataset that contains all instances of a certain class value.
   getInstancesFromClass(Instances, int, double)   - 
Static method in class weka.estimators. EstimatorUtils 
 Returns a dataset that contains of all instances of a certain class value.
   getInstancesFromValue(Instances, int, double)   - 
Static method in class weka.estimators. EstimatorUtils 
 Returns a dataset that contains of all instances of a certain value
 for the given attribute.
   getInstancesIndices()   - 
Method in class weka.filters.unsupervised.instance. RemoveRange 
 Gets ranges of instances selected.
   getInstancesNoClass()   - 
Method in class weka.associations. Apriori 
 Gets the instances without the class atrribute.
   getInstancesNoClass()   - 
Method in interface weka.associations. CARuleMiner 
 Gets the instances without the class attribute
   getInstancesNoClass()   - 
Method in class weka.associations. PredictiveApriori 
 Gets the instances without the class attribute
   getInstancesOnlyClass()   - 
Method in class weka.associations. Apriori 
 Gets only the class attribute of the instances.
   getInstancesOnlyClass()   - 
Method in interface weka.associations. CARuleMiner 
 Gets the class attribute and its values for all instances
   getInstancesOnlyClass()   - 
Method in class weka.associations. PredictiveApriori 
 Gets the class attribute of all instances
   getInstancesValueAt(int, int)   - 
Method in class weka.gui.arffviewer. ArffSortedTableModel 
 returns the double value of the underlying Instances object at the
 given position, -1 if out of bounds
   getInstancesValueAt(int, int)   - 
Method in class weka.gui.arffviewer. ArffTableModel 
 returns the double value of the underlying Instances object at the
 given position, -1 if out of bounds
   getIntercept()   - 
Method in class weka.classifiers.functions. SimpleLinearRegression 
 Returns the intercept of the function.
   getInternalCacheSize()   - 
Method in class weka.classifiers.functions.supportVector. StringKernel 
 Gets the size of the internal cache
   getInternals()   - 
Method in class weka.classifiers.bayes. WAODE 
 Gets whether more internals of the classifier are printed.
   getInterpreter()   - 
Method in class weka.core. Jython 
 returns the currently used Python Interpreter
   getInvert()   - 
Method in class weka.core. Range 
 Gets whether the range sense is inverted, i.e.
   getInvert()   - 
Method in class weka.filters.unsupervised.instance. RemoveMisclassified 
 Get whether selection is inverted.
   getInvertSelection()   - 
Method in interface weka.core. DistanceFunction 
 Gets whether the matching sense of attribute indices is inverted or not.
   getInvertSelection()   - 
Method in class weka.core. NormalizableDistance 
 Gets whether the matching sense of attribute indices is inverted or not.
   getInvertSelection()   - 
Method in class weka.filters.supervised.attribute. Discretize 
 Gets whether the supplied columns are to be removed or kept
   getInvertSelection()   - 
Method in class weka.filters.supervised.instance. Resample 
 Gets whether selection is inverted (only if instances are drawn WIHTOUT 
 replacement).
   getInvertSelection()   - 
Method in class weka.filters.supervised.instance. StratifiedRemoveFolds 
 Gets if selection is to be inverted.
   getInvertSelection()   - 
Method in class weka.filters.unsupervised.attribute. AbstractTimeSeries 
 Get whether the supplied columns are to be removed or kept
   getInvertSelection()   - 
Method in class weka.filters.unsupervised.attribute. Copy 
 Get whether the supplied columns are to be removed or kept
   getInvertSelection()   - 
Method in class weka.filters.unsupervised.attribute. Discretize 
 Gets whether the supplied columns are to be removed or kept
   getInvertSelection()   - 
Method in class weka.filters.unsupervised.attribute. MathExpression 
 Get whether the supplied columns are to be select or unselect
   getInvertSelection()   - 
Method in class weka.filters.unsupervised.attribute. NominalToBinary 
 Gets whether the supplied columns are to be removed or kept
   getInvertSelection()   - 
Method in class weka.filters.unsupervised.attribute. NumericCleaner 
 Gets whether the selection of the columns is inverted
   getInvertSelection()   - 
Method in class weka.filters.unsupervised.attribute. NumericToNominal 
 Gets whether the supplied columns are to be worked on or the others.
   getInvertSelection()   - 
Method in class weka.filters.unsupervised.attribute. NumericTransform 
 Get whether the supplied columns are to be transformed or not
   getInvertSelection()   - 
Method in class weka.filters.unsupervised.attribute. RELAGGS 
 Gets whether the supplied columns are to be processed or skipped
   getInvertSelection()   - 
Method in class weka.filters.unsupervised.attribute. Remove 
 Get whether the supplied columns are to be removed or kept
   getInvertSelection()   - 
Method in class weka.filters.unsupervised.attribute. RemoveType 
 Get whether the supplied columns are to be removed or kept
   getInvertSelection()   - 
Method in class weka.filters.unsupervised.attribute. StringToWordVector 
 Gets whether the supplied columns are to be processed or skipped.
   getInvertSelection()   - 
Method in class weka.filters.unsupervised.instance. RemoveFolds 
 Gets if selection is to be inverted.
   getInvertSelection()   - 
Method in class weka.filters.unsupervised.instance. RemoveFrequentValues 
 Get whether the supplied columns are to be removed or kept
   getInvertSelection()   - 
Method in class weka.filters.unsupervised.instance. RemovePercentage 
 Gets if selection is to be inverted.
   getInvertSelection()   - 
Method in class weka.filters.unsupervised.instance. RemoveRange 
 Gets if selection is to be inverted.
   getInvertSelection()   - 
Method in class weka.filters.unsupervised.instance. RemoveWithValues 
 Get whether the supplied columns are to be removed or kept
   getInvertSelection()   - 
Method in class weka.filters.unsupervised.instance. Resample 
 Gets whether selection is inverted (only if instances are drawn WIHTOUT 
 replacement).
   getJavaInitializationString()   - 
Method in class weka.gui. CostMatrixEditor 
 Returns the Java code that generates an object the same as the one being edited.
   getJavaInitializationString()   - 
Method in class weka.gui. FileEditor 
 Returns a representation of the current property value as java source.
   getJavaInitializationString()   - 
Method in class weka.gui. GenericArrayEditor 
 Supposedly returns an initialization string to create a classifier
 identical to the current one, including it's state, but this doesn't
 appear possible given that the initialization string isn't supposed to
 contain multiple statements.
   getJavaInitializationString()   - 
Method in class weka.gui. GenericObjectEditor 
 Supposedly returns an initialization string to create a Object
 identical to the current one, including it's state, but this doesn't
 appear possible given that the initialization string isn't supposed to
 contain multiple statements.
   getJavaInitializationString()   - 
Method in class weka.gui. SelectedTagEditor 
 Returns a description of the property value as java source.
   getJavaInitializationString()   - 
Method in class weka.gui. SimpleDateFormatEditor 
 Returns the Java code that generates an object the same as the one being edited.
   getJTable()   - 
Method in class weka.gui. JTableHelper 
 returns the JTable
   getKDTree()   - 
Method in class weka.clusterers. XMeans 
 Gets the KDTree class.
   getKernel()   - 
Method in class weka.classifiers.functions. GaussianProcesses 
 Gets the kernel to use.
   getKernel()   - 
Method in class weka.classifiers.functions. SMO.BinarySMO 
 Returns the kernel to use
   getKernel()   - 
Method in class weka.classifiers.functions. SMO 
 Returns the kernel to use
   getKernel()   - 
Method in class weka.classifiers.functions. SMOreg 
 Returns the kernel to use
   getKernel()   - 
Method in class weka.classifiers.functions.supportVector. CheckKernel 
 Get the kernel being tested
   getKernel()   - 
Method in class weka.classifiers.mi. MISMO 
 Gets the kernel to use.
   getKernel()   - 
Method in class weka.classifiers.mi. MISVM 
 Gets the kernel to use.
   getKernel()   - 
Method in class weka.filters.unsupervised.attribute. KernelFilter 
 Gets the kernel to use.
   getKernelBandwidth()   - 
Method in class weka.gui.boundaryvisualizer. KDDataGenerator 
 Get the kernel bandwidth
   getKernelEvaluations()   - 
Method in class weka.classifiers.functions.supportVector. RegOptimizer 
 returns the number of kernel evaluations
   getKernelFactorExpression()   - 
Method in class weka.filters.unsupervised.attribute. KernelFilter 
 Gets the expression for the kernel.
   getKernelMatrixFile()   - 
Method in class weka.classifiers.functions.supportVector. PrecomputedKernelMatrixKernel 
 Gets the file containing the kernel matrix.
   getKernelType()   - 
Method in class weka.classifiers.functions. LibSVM 
 Gets type of kernel function
   getKey()   - 
Method in interface weka.clusterers.forOPTICSAndDBScan.DataObjects. DataObject 
 Returns the key for this DataObject
   getKey()   - 
Method in class weka.clusterers.forOPTICSAndDBScan.DataObjects. EuclidianDataObject 
 Returns the key for this DataObject
   getKey()   - 
Method in class weka.clusterers.forOPTICSAndDBScan.DataObjects. ManhattanDataObject 
 Returns the key for this DataObject
   getKey()   - 
Method in class weka.experiment. ClassifierSplitEvaluator 
 Gets the key describing the current SplitEvaluator.
   getKey()   - 
Method in class weka.experiment. DensityBasedClustererSplitEvaluator 
 Gets the key describing the current SplitEvaluator.
   getKey()   - 
Method in class weka.experiment. RegressionSplitEvaluator 
 Gets the key describing the current SplitEvaluator.
   getKey()   - 
Method in interface weka.experiment. SplitEvaluator 
 Gets the key describing the current SplitEvaluator.
   getKeyFieldName()   - 
Method in class weka.experiment. AveragingResultProducer 
 Get the value of KeyFieldName.
   getKeyNames()   - 
Method in class weka.experiment. AveragingResultProducer 
 Gets the names of each of the columns produced for a single run.
   getKeyNames()   - 
Method in class weka.experiment. ClassifierSplitEvaluator 
 Gets the names of each of the key columns produced for a single run.
   getKeyNames()   - 
Method in class weka.experiment. CrossValidationResultProducer 
 Gets the names of each of the columns produced for a single run.
   getKeyNames()   - 
Method in class weka.experiment. DatabaseResultProducer 
 Gets the names of each of the columns produced for a single run.
   getKeyNames()   - 
Method in class weka.experiment. DensityBasedClustererSplitEvaluator 
 Gets the names of each of the key columns produced for a single run.
   getKeyNames()   - 
Method in class weka.experiment. LearningRateResultProducer 
 Gets the names of each of the columns produced for a single run.
   getKeyNames()   - 
Method in class weka.experiment. RandomSplitResultProducer 
 Gets the names of each of the columns produced for a single run.
   getKeyNames()   - 
Method in class weka.experiment. RegressionSplitEvaluator 
 Gets the names of each of the key columns produced for a single run.
   getKeyNames()   - 
Method in interface weka.experiment. ResultProducer 
 Gets the names of each of the key columns produced for a single run.
   getKeyNames()   - 
Method in interface weka.experiment. SplitEvaluator 
 Gets the names of each of the key columns produced for a single run.
   getKeys()   - 
Method in class weka.core.converters. DatabaseLoader 
 Gets the key columns' name
   getKeyTypes()   - 
Method in class weka.experiment. AveragingResultProducer 
 Gets the data types of each of the columns produced for a single run.
   getKeyTypes()   - 
Method in class weka.experiment. ClassifierSplitEvaluator 
 Gets the data types of each of the key columns produced for a single run.
   getKeyTypes()   - 
Method in class weka.experiment. CrossValidationResultProducer 
 Gets the data types of each of the columns produced for a single run.
   getKeyTypes()   - 
Method in class weka.experiment. DatabaseResultProducer 
 Gets the data types of each of the columns produced for a single run.
   getKeyTypes()   - 
Method in class weka.experiment. DensityBasedClustererSplitEvaluator 
 Gets the data types of each of the key columns produced for a single run.
   getKeyTypes()   - 
Method in class weka.experiment. LearningRateResultProducer 
 Gets the data types of each of the columns produced for a single run.
   getKeyTypes()   - 
Method in class weka.experiment. RandomSplitResultProducer 
 Gets the data types of each of the columns produced for a single run.
   getKeyTypes()   - 
Method in class weka.experiment. RegressionSplitEvaluator 
 Gets the data types of each of the key columns produced for a single run.
   getKeyTypes()   - 
Method in interface weka.experiment. ResultProducer 
 Gets the data types of each of the key columns produced for a single run.
   getKeyTypes()   - 
Method in interface weka.experiment. SplitEvaluator 
 Gets the data types of each of the key columns produced for a single run.
   getKeywords()   - 
Method in class weka.experiment. DatabaseUtils 
 Returns the currently stored keywords (as comma-separated list).
   getKeywordsMaskChar()   - 
Method in class weka.experiment. DatabaseUtils 
 Returns the currently set mask character.
   getKNN()   - 
Method in class weka.classifiers.lazy. IBk 
 Gets the number of neighbours the learner will use.
   getKNN()   - 
Method in class weka.classifiers.lazy. LWL 
 Gets the number of neighbours used for kernel bandwidth setting.
   getKValue()   - 
Method in class weka.classifiers.trees. RandomTree 
 Get the value of K.
   getKWBias()   - 
Method in class weka.classifiers. BVDecomposeSegCVSub 
 Get the calculated bias squared according to the Kohavi and Wolpert definition
   getKWSigma()   - 
Method in class weka.classifiers. BVDecomposeSegCVSub 
 Get the calculated sigma according to the Kohavi and Wolpert definition
   getKWVariance()   - 
Method in class weka.classifiers. BVDecomposeSegCVSub 
 Get the calculated variance according to the Kohavi and Wolpert definition
   getL()   - 
Method in class weka.core.matrix. CholeskyDecomposition 
 Return triangular factor.
   getL()   - 
Method in class weka.core. Matrix 
  Deprecated.  Returns the L part of the matrix.
   getL()   - 
Method in class weka.core.matrix. LUDecomposition 
 Return lower triangular factor
   getLabel()   - 
Method in class weka.gui.treevisualizer. Edge 
 Get the value of label.
   getLabel()   - 
Method in class weka.gui.treevisualizer. Node 
 Get the value of label.
   getLabels()   - 
Method in class weka.filters.unsupervised.attribute. AddValues 
 Get the comma-separated list of labels that are added.
   getLambda()   - 
Method in class weka.classifiers.functions. SPegasos 
 Get the current value of lambda
   getLambda()   - 
Method in class weka.classifiers.functions.supportVector. StringKernel 
 Gets the lambda constant used in the string kernel
   getLast()   - 
Method in class weka.associations.tertius. SimpleLinkedList 
  
   getLastLiteral()   - 
Method in class weka.associations.tertius. LiteralSet 
 Give the last literal added to this set.
   getLeaf()   - 
Method in class weka.classifiers.trees.j48. GraftSplit 
  
   getLearningRate()   - 
Method in class weka.classifiers.functions. MultilayerPerceptron 
  
   getLegendText()   - 
Method in class weka.gui.beans. ChartEvent 
 Get the legend text vector
   getLevel()   - 
Method in class weka.gui. HierarchyPropertyParser 
 Get the level of current node.
   getLikelihoodThreshold()   - 
Method in class weka.classifiers.meta. LogitBoost 
 Get the value of Precision.
   getLine(int)   - 
Method in class weka.gui.treevisualizer. Edge 
 Returns line number  n 
   getLine(int)   - 
Method in class weka.gui.treevisualizer. Node 
 Returns the text String for the specfied line.
   getLineNo()   - 
Method in class weka.core.converters. ArffLoader.ArffReader 
 returns the current line number
   getLinkAt(int)   - 
Method in class weka.attributeSelection. BestFirst.LinkedList2 
 returns the element (Link) at a specific index from the list.
   getLinkAt(int)   - 
Method in class weka.attributeSelection. LFSMethods.LinkedList2 
 returns the element (Link) at a specific index from the list.
   getLinkType()   - 
Method in class weka.clusterers. HierarchicalClusterer 
  
   getList()   - 
Method in class weka.gui. ResultHistoryPanel 
 Gets the JList used by the results list
   getListCellRendererComponent(JList, Object, int, boolean, boolean)   - 
Method in class weka.gui. CheckBoxList.CheckBoxListRenderer 
 Return a component that has been configured to display the specified 
 value.
   getListCellRendererComponent(JList, Object, int, boolean, boolean)   - 
Method in class weka.gui.experiment. AlgorithmListPanel.ObjectCellRenderer 
 Return a component that has been configured to display the specified 
 value.
   getListCellRendererComponent(JList, Object, int, boolean, boolean)   - 
Method in class weka.gui.sql. InfoPanelCellRenderer 
 Return a component that has been configured to display the specified value.
   getLiteral(int)   - 
Method in class weka.associations.tertius. Predicate 
  
   getLNorm()   - 
Method in class weka.filters.unsupervised.instance. Normalize 
 Get the L Norm used.
   getLoader()   - 
Method in class weka.core.converters. ConverterUtils.DataSource 
 returns the determined loader, null if the DataSource was initialized
 with data alone and not a file/URL.
   getLoader()   - 
Method in class weka.gui.beans. Loader 
 Get the loader
   getLoader()   - 
Method in class weka.gui. ConverterFileChooser 
 returns the loader that was chosen by the user, can be null in case the
 user aborted the dialog or the save dialog was shown
   getLoader()   - 
Method in class weka.gui. SetInstancesPanel 
 Gets the currently used Loader
   getLoaderForExtension(String)   - 
Static method in class weka.core.converters. ConverterUtils 
 tries to determine the loader to use for this kind of extension, returns
 null if none can be found.
   getLoaderForFile(String)   - 
Static method in class weka.core.converters. ConverterUtils 
 tries to determine the loader to use for this kind of file, returns
 null if none can be found.
   getLoaderForFile(File)   - 
Static method in class weka.core.converters. ConverterUtils 
 tries to determine the loader to use for this kind of file, returns
 null if none can be found.
   getLocallyPredictive()   - 
Method in class weka.attributeSelection. CfsSubsetEval 
 Return true if including locally predictive attributes
   getLocator(int)   - 
Method in class weka.core. AttributeLocator 
 Returns the AttributeLocator at the given index.
   getLocatorIndices()   - 
Method in class weka.core. AttributeLocator 
 Returns the indices of the AttributeLocator objects.
   getLog()   - 
Method in class weka.classifiers.pmml.consumer. PMMLClassifier 
 Get the logger.
   getLog()   - 
Method in class weka.core. Debug.Random 
 the currently used log, if null then stdout is used for outputting
 the debugging information
   getLog()   - 
Method in interface weka.core.pmml. PMMLModel 
 Get the logger.
   getLogFile()   - 
Method in class weka.classifiers.meta. GridSearch 
 Gets current log file.
   getLoglikeliHood(double[], Instances)   - 
Method in class weka.classifiers.bayes. BayesianLogisticRegression 
  
   getLoglikelihood()   - 
Method in class weka.classifiers.bayes.blr. Prior 
  
   getLogLikelihood()   - 
Method in class weka.clusterers. ClusterEvaluation 
 Return the log likelihood corresponding to the most recent
 set of instances clustered.
   getLogPosterior()   - 
Method in class weka.classifiers.bayes.blr. Prior 
  
   getLogProbForTargetClass(Instance)   - 
Method in class weka.classifiers.bayes. DMNBtext.DNBBinary 
 Calculates the class membership probabilities for the given test
 instance.
   getLookupCacheSize()   - 
Method in class weka.attributeSelection. BestFirst 
 Return the maximum size of the evaluated subset cache (expressed as a multiplier
 for the number of attributes in a data set.
   getLookupCacheSize()   - 
Method in class weka.attributeSelection. LinearForwardSelection 
 Return the maximum size of the evaluated subset cache (expressed as a
 multiplier for the number of attributes in a data set.
   getLookupCacheSize()   - 
Method in class weka.attributeSelection. SubsetSizeForwardSelection 
 Return the maximum size of the evaluated subset cache (expressed as a
 multiplier for the number of attributes in a data set.
   getLoss()   - 
Method in class weka.classifiers.functions. LibSVM 
 Gets the epsilon in loss function of epsilon-SVR
   getLossFunction()   - 
Method in class weka.classifiers.functions. SPegasos 
 Get the current loss function.
   getLower()   - 
Method in class weka.gui.experiment. RunNumberPanel 
 Gets the current lower run number.
   getLowerBoundMinSupport()   - 
Method in class weka.associations. Apriori 
 Get the value of lowerBoundMinSupport.
   getLowerBoundMinSupport()   - 
Method in class weka.associations. FPGrowth 
 Get the value of lowerBoundMinSupport.
   getLowerCaseTokens()   - 
Method in class weka.filters.unsupervised.attribute. StringToWordVector 
 Gets whether if the tokens are to be downcased or not.
   getLowerNumericBound()   - 
Method in class weka.core. Attribute 
 Returns the lower bound of a numeric attribute.
   getLowerSize()   - 
Method in class weka.experiment. LearningRateResultProducer 
 Get the value of LowerSize.
   getM5RootNode()   - 
Method in class weka.classifiers.trees.m5. M5Base 
  
   getM5RootNode()   - 
Method in class weka.classifiers.trees.m5. Rule 
  
   getMainPanel()   - 
Method in class weka.gui.arffviewer. ArffViewer 
 returns the main panel
   getMajorityClass()   - 
Method in class weka.classifiers.rules. Ridor 
  
   getMakeBinary()   - 
Method in class weka.filters.supervised.attribute. Discretize 
 Gets whether binary attributes should be made for discretized ones.
   getMakeBinary()   - 
Method in class weka.filters.unsupervised.attribute. Discretize 
 Gets whether binary attributes should be made for discretized ones.
   getManualThresholdValue()   - 
Method in class weka.classifiers.meta. ThresholdSelector 
 Returns the value of the manual threshold.
   getMargin(int)   - 
Method in class weka.classifiers.bayes.net. EditableBayesNet 
 return marginal distibution for a node
   getMargin(int)   - 
Method in class weka.classifiers.bayes.net. MarginCalculator 
  
   getMarkovBlanketClassifier()   - 
Method in class weka.classifiers.bayes.net.search.global. GlobalScoreSearchAlgorithm 
  
   getMarkovBlanketClassifier()   - 
Method in class weka.classifiers.bayes.net.search.local. LocalScoreSearchAlgorithm 
  
   getMasterPlot()   - 
Method in class weka.gui.visualize. Plot2D 
 Get the master plot
   getMatches()   - 
Method in class weka.core. FindWithCapabilities 
 returns the matches from the last find call.
   getMatches(String)   - 
Method in class weka.gui. SimpleCLIPanel.CommandlineCompletion 
 returns all the matches with the partial search string, files or
 classes.
   getMatchMissingValues()   - 
Method in class weka.filters.unsupervised.instance. RemoveWithValues 
 Gets whether missing values are counted as a match.
   getMatrix(int, int, int, int)   - 
Method in class weka.core.matrix. Matrix 
 Get a submatrix.
   getMatrix(int[], int[])   - 
Method in class weka.core.matrix. Matrix 
 Get a submatrix.
   getMatrix(int, int, int[])   - 
Method in class weka.core.matrix. Matrix 
 Get a submatrix.
   getMatrix(int[], int, int)   - 
Method in class weka.core.matrix. Matrix 
 Get a submatrix.
   getMax()   - 
Method in class weka.core. Memory 
 returns the maximum amount of memory that can be assigned
   getMax()   - 
Method in class weka.gui.beans. ChartEvent 
 Get the max y value
   getMaxArray()   - 
Method in class weka.filters.unsupervised.attribute. Normalize 
 Returns the calculated maximum values for the attributes in the data.
   getMaxBoostingIterations()   - 
Method in class weka.classifiers.functions. SimpleLogistic 
 Get the value of maxBoostingIterations.
   getMaxC()   - 
Method in class weka.gui.visualize. Plot2D 
 Return the current max value of the colouring attribute
   getMaxCardinality()   - 
Method in class weka.classifiers.bayes.net.search.ci. ICSSearchAlgorithm 
 returns the max cardinality
   getMaxCardinality()   - 
Method in class weka.filters.unsupervised.attribute. RELAGGS 
 Gets the maximum number of values allowed for nominal attributes, before
 they're skipped.
   getMaxChunkSize()   - 
Method in class weka.classifiers.meta. RacedIncrementalLogitBoost 
 Get the maximum chunk size
   getMaxCoordsPerPoint()   - 
Method in class weka.core.neighboursearch. PerformanceStats 
 Returns the maximum of coords per point.
   getMaxCost(int)   - 
Method in class weka.classifiers. CostMatrix 
 Gets the maximum cost for a particular class value.
   getMaxCost(int, Instance)   - 
Method in class weka.classifiers. CostMatrix 
 Gets the maximum cost for a particular class value.
   getMaxCount()   - 
Method in class weka.filters.supervised.instance. SpreadSubsample 
 Gets the value for the max count
   getMaxDefault()   - 
Method in class weka.filters.unsupervised.attribute. NumericCleaner 
 Get the maximum default.
   getMaxDepth()   - 
Method in class weka.classifiers.trees. RandomForest 
 Get the maximum depth of trh tree, 0 for unlimited.
   getMaxDepth()   - 
Method in class weka.classifiers.trees. RandomTree 
 Get the maximum depth of trh tree, 0 for unlimited.
   getMaxDepth()   - 
Method in class weka.classifiers.trees. REPTree 
 Get the value of MaxDepth.
   getMaxDepth()   - 
Method in class weka.core.neighboursearch.balltrees. BallTreeConstructor 
 Returns the depth of the built tree.
   getMaxGenerations()   - 
Method in class weka.attributeSelection. GeneticSearch 
 get the number of generations
   getMaxGridExtensions()   - 
Method in class weka.classifiers.meta. GridSearch 
 Gets the maximum number of grid extensions, -1 for unlimited.
   getMaxGroup()   - 
Method in class weka.classifiers.meta. RotationForest 
 Gets the maximum size of a group.
   getMaximumAttributeNames()   - 
Method in class weka.attributeSelection. LatentSemanticAnalysis 
 Gets maximum number of attributes to include in
 transformed attribute names.
   getMaximumAttributeNames()   - 
Method in class weka.attributeSelection. PrincipalComponents 
 Gets maximum number of attributes to include in
 transformed attribute names.
   getMaximumAttributeNames()   - 
Method in class weka.filters.unsupervised.attribute. PrincipalComponents 
 Gets maximum number of attributes to include in
 transformed attribute names.
   getMaximumAttributes()   - 
Method in class weka.filters.unsupervised.attribute. PrincipalComponents 
 Gets maximum number of PC attributes to retain.
   getMaximumVariancePercentageAllowed()   - 
Method in class weka.filters.unsupervised.attribute. RemoveUseless 
 Gets the maximum variance attributes are allowed to have before they are
 deleted by the filter.
   getMaxInstancesInLeaf()   - 
Method in class weka.core.neighboursearch.balltrees. BallTreeConstructor 
 Returns the maximum number of instances allowed in a leaf.
   getMaxInstInLeaf()   - 
Method in class weka.core.neighboursearch. KDTree 
 Get the maximum number of instances in a leaf.
   getMaxInstNum()   - 
Method in class weka.datagenerators.clusterers. BIRCHCluster 
 Gets the upper boundary for instances per cluster.
   getMaxInstNum()   - 
Method in class weka.datagenerators.clusterers. SubspaceClusterDefinition 
 Gets the upper boundary for instances per cluster.
   getMaxIntNodesVisited()   - 
Method in class weka.core.neighboursearch. TreePerformanceStats 
 returns the maximum of internal nodes visited.
   getMaxIterations()   - 
Method in class weka.classifiers.bayes. BayesianLogisticRegression 
 Get the maximum number of iterations to perform
   getMaxIterations()   - 
Method in class weka.classifiers.mi. MIBoost 
 Get the maximum number of boost iterations
   getMaxIterations()   - 
Method in class weka.classifiers.mi. MISVM 
 Gets the maximum number of iterations.
   getMaxIterations()   - 
Method in class weka.classifiers.trees.lmt. LogisticBase 
 Returns the maxIterations parameter.
   getMaxIterations()   - 
Method in class weka.clusterers. EM 
 Get the maximum number of iterations
   getMaxIterations()   - 
Method in class weka.clusterers. sIB 
 Get the max number of iterations
   getMaxIterations()   - 
Method in class weka.clusterers. SimpleKMeans 
 gets the number of maximum iterations to be executed
   getMaxIterations()   - 
Method in class weka.clusterers. XMeans 
 Gets the maximum number of iterations.
   getMaxIterations()   - 
Method in class weka.filters.unsupervised.instance. RemoveMisclassified 
 Gets the maximum number of cleansing iterations performed
   getMaxIts()   - 
Method in class weka.classifiers.functions. Logistic 
 Get the value of MaxIts.
   getMaxIts()   - 
Method in class weka.classifiers.functions. RBFNetwork 
 Get the value of MaxIts.
   getMaxK()   - 
Method in class weka.classifiers.functions. VotedPerceptron 
 Get the value of maxK.
   getMaxKMeans()   - 
Method in class weka.clusterers. XMeans 
 Gets the maximum number of iterations in KMeans.
   getMaxKMeansForChildren()   - 
Method in class weka.clusterers. XMeans 
 Gets the maximum number of iterations in KMeans.
   getMaxLeavesVisited()   - 
Method in class weka.core.neighboursearch. TreePerformanceStats 
 Returns the maximum number of leaves visited.
   getMaxNrOfParents()   - 
Method in class weka.classifiers.bayes.net.search.global. HillClimber 
 Gets the max number of parents.
   getMaxNrOfParents()   - 
Method in class weka.classifiers.bayes.net.search.global. K2 
 Gets the max number of parents.
   getMaxNrOfParents()   - 
Method in class weka.classifiers.bayes.net.search.local. HillClimber 
 Gets the max number of parents.
   getMaxNrOfParents()   - 
Method in class weka.classifiers.bayes.net.search.local. K2 
 Gets the max number of parents.
   getMaxNrOfParents()   - 
Method in class weka.classifiers.bayes.net.search.local. LAGDHillClimber 
 Gets the max number of parents.
   getMaxNumberOfItems()   - 
Method in class weka.associations. FPGrowth 
 Gets the maximum number of items to be included in large item sets.
   getMaxNumClusters()   - 
Method in class weka.clusterers. XMeans 
 Gets the maximum number of clusters to generate.
   getMaxPlots()   - 
Method in class weka.gui.beans. AttributeSummarizer 
 Get the number of plots to display
   getMaxPointsVisited()   - 
Method in class weka.core.neighboursearch. PerformanceStats 
 Returns the maximum of points visited.
   getMaxRadius()   - 
Method in class weka.datagenerators.clusterers. BIRCHCluster 
 Gets the upper boundary for the radiuses of the clusters.
   getMaxRange()   - 
Method in class weka.datagenerators.classifiers.regression. MexicanHat 
 Gets the upper boundary for the range of x
   getMaxRelativeLeafRadius()   - 
Method in class weka.core.neighboursearch.balltrees. BallTreeConstructor 
 Returns the maximum relative radius of a leaf node.
   getMaxRows()   - 
Method in class weka.gui.sql.event. QueryExecuteEvent 
 returns the maximum number of rows to retrieve.
   getMaxRows()   - 
Method in class weka.gui.sql. QueryPanel 
 returns the current value for the maximum number of rows.
   getMaxRows()   - 
Method in class weka.gui.sql. ResultSetHelper 
 the maximum number of rows to retrieve, less than 1 means unlimited.
   getMaxRuleSize()   - 
Method in class weka.datagenerators.classifiers.classification. RDG1 
 Gets the maximum number of tests in rules.
   getMaxRunNumber()   - 
Method in class weka.gui.beans. BatchClassifierEvent 
 Get the maximum run number
   getMaxRunNumber()   - 
Method in class weka.gui.beans. TestSetEvent 
 Get the maximum number of runs.
   getMaxRunNumber()   - 
Method in class weka.gui.beans. TrainingSetEvent 
 Get the maximum number of runs.
   getMaxSetNumber()   - 
Method in class weka.gui.beans. BatchClassifierEvent 
 Get the maximum set number (ie the total number of training
 and testing sets in the series).
   getMaxSetNumber()   - 
Method in class weka.gui.beans. BatchClustererEvent 
 Get the maximum set number (ie the total number of training
 and testing sets in the series).
   getMaxSetNumber()   - 
Method in class weka.gui.beans. TestSetEvent 
 Get the maximum set number
   getMaxSetNumber()   - 
Method in class weka.gui.beans. TrainingSetEvent 
 Get the maximum set number
   getMaxSubsequenceLength()   - 
Method in class weka.classifiers.functions.supportVector. StringKernel 
 Returns the maximum length of the subsequence
   getMaxThreshold()   - 
Method in class weka.filters.unsupervised.attribute. NumericCleaner 
 Get the maximum threshold.
   getMaxValue()   - 
Method in class weka.datagenerators.clusterers. SubspaceClusterDefinition 
  
   getMaxVersion()   - 
Method in interface weka.gui.visualize.plugins. VisualizePlugin 
 Get the maximum version of Weka, exclusive, the class
 is designed to work with.
   getMaxX()   - 
Method in class weka.gui.visualize. Plot2D 
 Return the current max value of the attribute plotted on the x axis
   getMaxXBound()   - 
Method in class weka.gui.boundaryvisualizer. BoundaryPanel 
 Gets the maximum x-coordinate bound, in training-instance units (not mouse coordinates).
   getMaxY()   - 
Method in class weka.gui.visualize. Plot2D 
 Return the current max value of the attribute plotted on the y axis
   getMaxYBound()   - 
Method in class weka.gui.boundaryvisualizer. BoundaryPanel 
 Gets the maximum x-coordinate bound, in training-instance units (not mouse coordinates).
   getMean()   - 
Method in class weka.estimators. NormalEstimator 
 Return the value of the mean of this normal estimator.
   getMean(int, int)   - 
Method in class weka.experiment. ResultMatrix 
 returns the mean at the given position, if the position is valid,
 otherwise 0
   getMeanCoordsPerPoint()   - 
Method in class weka.core.neighboursearch. PerformanceStats 
 Returns the mean of coords per point.
   getMeanIntNodesVisited()   - 
Method in class weka.core.neighboursearch. TreePerformanceStats 
 Returns the mean of internal nodes visited.
   getMeanLeavesVisited()   - 
Method in class weka.core.neighboursearch. TreePerformanceStats 
 Returns the mean of number of leaves visited.
   getMeanPointsVisited()   - 
Method in class weka.core.neighboursearch. PerformanceStats 
 Returns the mean of points visited.
   getMeanPrec()   - 
Method in class weka.experiment. ResultMatrix 
 returns the current precision for the means
   getMeanPrec()   - 
Method in class weka.gui.experiment. OutputFormatDialog 
 Gets the precision used for printing the mean.
   getMeanPrecision()   - 
Static method in class weka.gui.experiment. ExperimenterDefaults 
 returns the default precision for the mean
   getMeans()   - 
Method in class weka.estimators. KernelEstimator 
 Return the means of the kernels.
   getMeanSquared()   - 
Method in class weka.classifiers.lazy. IBk 
 Gets whether the mean squared error is used rather than mean
 absolute error when doing cross-validation.
   getMeanStddev()   - 
Method in class weka.datagenerators.clusterers. SubspaceClusterDefinition 
 returns the current mean/stddev setup
   getMeanValue()   - 
Method in class weka.datagenerators.clusterers. SubspaceClusterDefinition 
  
   getMeanWidth()   - 
Method in class weka.experiment. ResultMatrix 
 returns the current width for the mean
   getMeasure(String)   - 
Method in class weka.classifiers.bayes. BayesNet 
 Returns the value of the named measure
   getMeasure(String)   - 
Method in class weka.classifiers.functions. SimpleLogistic 
 Returns the value of the named measure
   getMeasure(String)   - 
Method in class weka.classifiers.functions. SMOreg 
 Returns the value of the named measure
   getMeasure(String)   - 
Method in class weka.classifiers.lazy. IBk 
 Returns the value of the named measure from the 
 neighbour search algorithm, plus the chosen K in case
 cross-validation is enabled.
   getMeasure(String)   - 
Method in class weka.classifiers.lazy. LWL 
 Returns the value of the named measure from the 
 neighbour search algorithm.
   getMeasure(String)   - 
Method in class weka.classifiers.meta. AdditiveRegression 
 Returns the value of the named measure
   getMeasure(String)   - 
Method in class weka.classifiers.meta. AttributeSelectedClassifier 
 Returns the value of the named measure
   getMeasure(String)   - 
Method in class weka.classifiers.meta. Bagging 
 Returns the value of the named measure.
   getMeasure(String)   - 
Method in class weka.classifiers.meta. GridSearch 
 Returns the value of the named measure
   getMeasure()   - 
Method in class weka.classifiers.meta. ThresholdSelector 
 get measure used for determining threshold
   getMeasure(String)   - 
Method in class weka.classifiers.rules. DecisionTable 
 Returns the value of the named measure
   getMeasure(String)   - 
Method in class weka.classifiers.rules. DTNB 
 Returns the value of the named measure
   getMeasure(String)   - 
Method in class weka.classifiers.rules. JRip 
 Returns the value of the named measure
   getMeasure(String)   - 
Method in class weka.classifiers.rules. PART 
 Returns the value of the named measure
   getMeasure(String)   - 
Method in class weka.classifiers.rules. Ridor 
 Returns the value of the named measure
   getMeasure(String)   - 
Method in class weka.classifiers.trees. ADTree 
 Returns the value of the named measure.
   getMeasure(String)   - 
Method in class weka.classifiers.trees. BFTree 
 Returns the value of the named measure
   getMeasure(String)   - 
Method in class weka.classifiers.trees. FT 
 Returns the value of the named measure
   getMeasure(String)   - 
Method in class weka.classifiers.trees. J48 
 Returns the value of the named measure
   getMeasure(String)   - 
Method in class weka.classifiers.trees. J48graft 
 Returns the value of the named measure
   getMeasure(String)   - 
Method in class weka.classifiers.trees. LADTree 
 Returns the value of the named measure.
   getMeasure(String)   - 
Method in class weka.classifiers.trees. LMT 
 Returns the value of the named measure
   getMeasure(String)   - 
Method in class weka.classifiers.trees.m5. M5Base 
 Returns the value of the named measure
   getMeasure(String)   - 
Method in class weka.classifiers.trees. NBTree 
 Returns the value of the named measure
   getMeasure(String)   - 
Method in class weka.classifiers.trees. RandomForest 
 Returns the value of the named measure.
   getMeasure(String)   - 
Method in class weka.classifiers.trees. REPTree 
 Returns the value of the named measure.
   getMeasure(String)   - 
Method in class weka.classifiers.trees. SimpleCart 
 Returns the value of the named measure.
   getMeasure(String)   - 
Method in interface weka.core. AdditionalMeasureProducer 
 Returns the value of the named measure
   getMeasure(String)   - 
Method in class weka.core.neighboursearch. BallTree 
 Returns the value of the named measure.
   getMeasure(String)   - 
Method in class weka.core.neighboursearch. CoverTree 
 Returns the value of the named measure.
   getMeasure(String)   - 
Method in class weka.core.neighboursearch. KDTree 
 Returns the value of the named measure.
   getMeasure(String)   - 
Method in class weka.core.neighboursearch. NearestNeighbourSearch 
 Returns the value of the named measure.
   getMeasure(String)   - 
Method in class weka.core.neighboursearch. PerformanceStats 
 Returns the value of the named measure.
   getMeasure(String)   - 
Method in class weka.core.neighboursearch. TreePerformanceStats 
 Returns the value of the named measure.
   getMeasure(String)   - 
Method in class weka.experiment. AveragingResultProducer 
 Returns the value of the named measure
   getMeasure(String)   - 
Method in class weka.experiment. ClassifierSplitEvaluator 
 Returns the value of the named measure
   getMeasure(String)   - 
Method in class weka.experiment. CrossValidationResultProducer 
 Returns the value of the named measure
   getMeasure(String)   - 
Method in class weka.experiment. DatabaseResultProducer 
 Returns the value of the named measure
   getMeasure(String)   - 
Method in class weka.experiment. DensityBasedClustererSplitEvaluator 
 Returns the value of the named measure
   getMeasure(String)   - 
Method in class weka.experiment. LearningRateResultProducer 
 Returns the value of the named measure
   getMeasure(String)   - 
Method in class weka.experiment. RandomSplitResultProducer 
 Returns the value of the named measure
   getMeasure(String)   - 
Method in class weka.experiment. RegressionSplitEvaluator 
 Returns the value of the named measure
   getMeasurePerformance()   - 
Method in class weka.core.neighboursearch. NearestNeighbourSearch 
 Gets whether performance statistics are being calculated or not.
   getMenu()   - 
Method in class weka.gui.arffviewer. ArffViewerMainPanel 
 returns the menu bar to be added in a frame
   getMenuBar()   - 
Method in class weka.classifiers.bayes.net. GUI 
 Get the menu bar for this application.
   getMenuTitle()   - 
Method in interface weka.gui. MainMenuExtension 
 Returns the name of the menu item.
   getMestWeight()   - 
Method in class weka.classifiers.bayes. AODEsr 
 Gets the weight used in m-estimate
   getMetaClassifier()   - 
Method in class weka.classifiers.meta. Stacking 
 Gets the meta classifier.
   getMetadata()   - 
Method in class weka.core. Attribute 
 Returns the properties supplied for this attribute.
   getMetaData()   - 
Method in class weka.core.converters. DatabaseConnection 
 Gets meta data for the database connection object.
   getMethod()   - 
Method in class weka.classifiers.functions.neural. NeuralNode 
  
   getMethod()   - 
Method in class weka.classifiers.meta. MultiClassClassifier 
 Gets the method used.
   getMethod()   - 
Method in class weka.classifiers.mi. MIWrapper 
 Get the method used in testing.
   getMethodName()   - 
Method in class weka.filters.unsupervised.attribute. NumericTransform 
 Get the transformation method.
   getMetricType()   - 
Method in class weka.associations. Apriori 
 Get the metric type
   getMetricType()   - 
Method in class weka.associations. FPGrowth.AssociationRule 
 Get the metric type of this rule (e.g.
   getMetricType()   - 
Method in class weka.associations. FPGrowth 
 Get the metric type to use.
   getMetricValue()   - 
Method in class weka.associations. FPGrowth.AssociationRule 
 Get the value of the metric for this rule.
   getMiddle(double[])   - 
Method in class weka.core. EuclideanDistance 
 Returns value in the middle of the two parameter values.
   getMidPoints()   - 
Method in class weka.associations. PriorEstimation 
 returns an ordered array of all mid points
   getMin()   - 
Method in class weka.gui.beans. ChartEvent 
 Get the min y value
   getMinArray()   - 
Method in class weka.filters.unsupervised.attribute. Normalize 
 Returns the calculated minimum values for the attributes in the data.
   getMinBoxRelWidth()   - 
Method in class weka.core.neighboursearch. KDTree 
 Gets the minimum relative box width.
   getMinBucketSize()   - 
Method in class weka.classifiers.rules. OneR 
 Get the value of minBucketSize.
   getMinC()   - 
Method in class weka.gui.visualize. Plot2D 
 Return the current min value of the colouring attribute
   getMinChange()   - 
Method in class weka.clusterers. sIB 
 get the minimum number of changes
   getMinChunkSize()   - 
Method in class weka.classifiers.meta. RacedIncrementalLogitBoost 
 Get the minimum chunk size
   getMinCoordsPerPoint()   - 
Method in class weka.core.neighboursearch. PerformanceStats 
 Returns the minimum of coords per point.
   getMinDefault()   - 
Method in class weka.filters.unsupervised.attribute. NumericCleaner 
 Get the minimum default.
   getMinFunction()   - 
Method in class weka.core. Optimization 
 Get the minimal function value
   getMinGroup()   - 
Method in class weka.classifiers.meta. RotationForest 
 Gets the minimum size of a group.
   getMinimax()   - 
Method in class weka.classifiers.mi. MISMO 
 Check if the MIMinimax feature space is to be used.
   getMinimizeExpectedCost()   - 
Method in class weka.classifiers.meta. CostSensitiveClassifier 
 Gets the value of MinimizeExpectedCost.
   getMinimumBucketSize()   - 
Method in class weka.attributeSelection. OneRAttributeEval 
 Get the minimum bucket size used by oneR
   getMinimumNumberInstances()   - 
Method in class weka.core. Capabilities 
 returns the minimum number of instances that have to be in the dataset
   getMiningFields()   - 
Method in class weka.core.pmml. MiningSchema 
  
   getMiningSchema()   - 
Method in class weka.classifiers.pmml.consumer. PMMLClassifier 
 Get the mining schema for this model.
   getMiningSchema()   - 
Method in interface weka.core.pmml. PMMLModel 
 Get the mining schema.
   getMiningSchemaAsInstances()   - 
Method in class weka.core.pmml. MiningSchema 
 Get the mining schema fields as an Instances object.
   getMinInstNum()   - 
Method in class weka.datagenerators.clusterers. BIRCHCluster 
 Gets the lower boundary for instances per cluster.
   getMinInstNum()   - 
Method in class weka.datagenerators.clusterers. SubspaceClusterDefinition 
 Gets the lower boundary for instances per cluster.
   getMinIntNodesVisited()   - 
Method in class weka.core.neighboursearch. TreePerformanceStats 
 Returns the minimum of internal nodes visited.
   getMinLeavesVisited()   - 
Method in class weka.core.neighboursearch. TreePerformanceStats 
 Returns the minimum number of leaves visited.
   getMinLevel()   - 
Method in class weka.core.logging. Logger 
 Returns the minimum level log messages must have in order to appear in
 the log.
   getMinMax(Instances, int, double[])   - 
Static method in class weka.estimators. CheckEstimator 
 Find the minimum and the maximum of the attribute and return it in 
 the last parameter..
   getMinMax(Instances, int, double[])   - 
Static method in class weka.estimators. EstimatorUtils 
 Find the minimum and the maximum of the attribute and return it in 
 the last parameter..
   getMinMetric()   - 
Method in class weka.associations. Apriori 
 Get the value of minConfidence.
   getMinMetric()   - 
Method in class weka.associations. FPGrowth 
 Get the value of minConfidence.
   getMinNo()   - 
Method in class weka.classifiers.rules. ConjunctiveRule 
 Gets the minimum total weight of the instances in a rule
   getMinNo()   - 
Method in class weka.classifiers.rules. JRip 
 Gets the minimum total weight of the instances in a rule
   getMinNo()   - 
Method in class weka.classifiers.rules. Ridor 
  
   getMinNum()   - 
Method in class weka.classifiers.trees. RandomTree 
 Get the value of MinNum.
   getMinNum()   - 
Method in class weka.classifiers.trees. REPTree 
 Get the value of MinNum.
   getMinNumClusters()   - 
Method in class weka.clusterers. XMeans 
 Gets the minimum number of clusters to generate.
   getMinNumInstances()   - 
Method in class weka.classifiers.trees. FT 
 Get the value of minNumInstances.
   getMinNumInstances()   - 
Method in class weka.classifiers.trees. LMT 
 Get the value of minNumInstances.
   getMinNumInstances()   - 
Method in class weka.classifiers.trees.m5. M5Base 
 Get the minimum number of instances to allow at a leaf node
   getMinNumInstances()   - 
Method in class weka.classifiers.trees.m5. Rule 
 Get the minimum number of instances to allow at a leaf node
   getMinNumInstances()   - 
Method in class weka.classifiers.trees.m5. RuleNode 
 Get the minimum number of instances to allow at a leaf node
   getMinNumObj()   - 
Method in class weka.classifiers.rules. PART 
 Get the value of minNumObj.
   getMinNumObj()   - 
Method in class weka.classifiers.trees. BFTree 
 Get minimal number of instances at the terminal nodes.
   getMinNumObj()   - 
Method in class weka.classifiers.trees. J48 
 Get the value of minNumObj.
   getMinNumObj()   - 
Method in class weka.classifiers.trees. J48graft 
 Get the value of minNumObj.
   getMinNumObj()   - 
Method in class weka.classifiers.trees. SimpleCart 
 Get minimal number of instances at the terminal nodes.
   getMinPoints()   - 
Method in class weka.clusterers. DBScan 
 Returns the value of minPoints
   getMinPoints()   - 
Method in class weka.clusterers.forOPTICSAndDBScan.OPTICS_GUI. SERObject 
 Returns the number of minPoints
   getMinPoints()   - 
Method in class weka.clusterers. OPTICS 
 Returns the value of minPoints
   getMinPointsVisited()   - 
Method in class weka.core.neighboursearch. PerformanceStats 
 Returns the minimum of points visited.
   getMinRadius()   - 
Method in class weka.datagenerators.clusterers. BIRCHCluster 
 Gets the lower boundary for the radiuses of the clusters.
   getMinRange()   - 
Method in class weka.datagenerators.classifiers.regression. MexicanHat 
 Gets the lower boundary for the range of x
   getMinRuleSize()   - 
Method in class weka.datagenerators.classifiers.classification. RDG1 
 Gets the minimum number of tests in rules.
   getMinStdDev()   - 
Method in class weka.classifiers.functions. RBFNetwork 
 Get the MinStdDev value.
   getMinStdDev()   - 
Method in class weka.clusterers. EM 
 Get the minimum allowable standard deviation.
   getMinStdDev()   - 
Method in class weka.clusterers. MakeDensityBasedClusterer 
 Get the minimum allowable standard deviation.
   getMinSupport()   - 
Method in class weka.associations. GeneralizedSequentialPatterns 
 Returns the minimum support threshold.
   getMinTermFreq()   - 
Method in class weka.filters.unsupervised.attribute. StringToWordVector 
 Get the MinTermFreq value.
   getMinThreshold()   - 
Method in class weka.filters.unsupervised.attribute. NumericCleaner 
 Get the minimum threshold.
   getMinValue()   - 
Method in class weka.datagenerators.clusterers. SubspaceClusterDefinition 
  
   getMinVarianceProp()   - 
Method in class weka.classifiers.trees. REPTree 
 Get the value of MinVarianceProp.
   getMinVersion()   - 
Method in interface weka.gui.visualize.plugins. VisualizePlugin 
 Get the minimum version of Weka, inclusive, the class
 is designed to work with.
   getMinX()   - 
Method in class weka.gui.visualize. Plot2D 
 Return the current min value of the attribute plotted on the x axis
   getMinXBound()   - 
Method in class weka.gui.boundaryvisualizer. BoundaryPanel 
 Gets the minimum x-coordinate bound, in training-instance units (not mouse coordinates).
   getMinY()   - 
Method in class weka.gui.visualize. Plot2D 
 Return the current min value of the attribute plotted on the y axis
   getMinYBound()   - 
Method in class weka.gui.boundaryvisualizer. BoundaryPanel 
 Gets the minimum y-coordinate bound, in training-instance units (not mouse coordinates).
   getMisses()   - 
Method in class weka.core. FindWithCapabilities 
 returns the misses from the last find call.
   getMissingMerge()   - 
Method in class weka.attributeSelection. ChiSquaredAttributeEval 
 get whether missing values are being distributed or not
   getMissingMerge()   - 
Method in class weka.attributeSelection. GainRatioAttributeEval 
 get whether missing values are being distributed or not
   getMissingMerge()   - 
Method in class weka.attributeSelection. InfoGainAttributeEval 
 get whether missing values are being distributed or not
   getMissingMerge()   - 
Method in class weka.attributeSelection. SymmetricalUncertAttributeEval 
 get whether missing values are being distributed or not
   getMissingMode()   - 
Method in class weka.classifiers.lazy. KStar 
 Gets the method to use for handling missing values.
   getMissingSeparate()   - 
Method in class weka.attributeSelection. CfsSubsetEval 
 Return true is missing is treated as a separate value
   getMissingValue()   - 
Method in class weka.core.converters. CSVLoader 
 Returns the current placeholder for missing values.
   getMissingValues()   - 
Method in class weka.associations. Tertius 
 Get the value of missingValues.
   getMissingValueTreatmentMethod()   - 
Method in class weka.core.pmml. MiningFieldMetaInfo 
 Get the missing value treatment method for this field.
   getMixingDistribution()   - 
Method in class weka.classifiers.functions.pace. MixtureDistribution 
 Gets the mixing distribution
   getModel()   - 
Method in class weka.classifiers.functions. LibLINEAR 
  
   getModel()   - 
Method in class weka.classifiers.trees.m5. RuleNode 
 Get the linear model at this node
   getModel()   - 
Method in class weka.gui. SortedTableModel 
 returns the current model, can be null
   getModelFile()   - 
Method in class weka.classifiers.misc. SerializedClassifier 
 Gets the file containing the serialized model.
   getModelParameters()   - 
Method in class weka.classifiers.trees.ft. FTtree 
 Returns a string describing the number of LogitBoost iterations performed at this node, the total number
 of LogitBoost iterations performed (including iterations at higher levels in the tree), and the number
 of training examples at this node.
   getModelParameters()   - 
Method in class weka.classifiers.trees.lmt. LMTNode 
 Returns a string describing the number of LogitBoost iterations performed at this node, the total number
 of LogitBoost iterations performed (including iterations at higher levels in the tree), and the number
 of training examples at this node.
   getModelType()   - 
Method in class weka.classifiers.trees. FT 
 Get the type of functional tree model being used.
   getModelValueAt(int, int)   - 
Method in class weka.gui.arffviewer. ArffSortedTableModel 
 returns the value at the given position
   getModifyHeader()   - 
Method in class weka.filters.unsupervised.instance. RemoveFrequentValues 
 Gets whether the header will be modified when selecting on nominal
 attributes.
   getModifyHeader()   - 
Method in class weka.filters.unsupervised.instance. RemoveWithValues 
 Gets whether the header will be modified when selecting on nominal
 attributes.
   getMomentum()   - 
Method in class weka.classifiers.functions. MultilayerPerceptron 
  
   getMultiInstance()   - 
Method in class weka.core. TestInstances 
 Gets whether multi-instance data (with a fixed structure) is generated
   getMultiInstanceCapabilities()   - 
Method in class weka.classifiers.mi. CitationKNN 
 Returns the capabilities of this multi-instance classifier for the
 relational data.
   getMultiInstanceCapabilities()   - 
Method in class weka.classifiers.mi. MDD 
 Returns the capabilities of this multi-instance classifier for the
 relational data.
   getMultiInstanceCapabilities()   - 
Method in class weka.classifiers.mi. MIBoost 
 Returns the capabilities of this multi-instance classifier for the
 relational data.
   getMultiInstanceCapabilities()   - 
Method in class weka.classifiers.mi. MIDD 
 Returns the capabilities of this multi-instance classifier for the
 relational data.
   getMultiInstanceCapabilities()   - 
Method in class weka.classifiers.mi. MIEMDD 
 Returns the capabilities of this multi-instance classifier for the
 relational data.
   getMultiInstanceCapabilities()   - 
Method in class weka.classifiers.mi. MILR 
 Returns the capabilities of this multi-instance classifier for the
 relational data.
   getMultiInstanceCapabilities()   - 
Method in class weka.classifiers.mi. MINND 
 Returns the capabilities of this multi-instance classifier for the
 relational data.
   getMultiInstanceCapabilities()   - 
Method in class weka.classifiers.mi. MIOptimalBall 
 Returns the capabilities of this multi-instance classifier for the
 relational data.
   getMultiInstanceCapabilities()   - 
Method in class weka.classifiers.mi. MISMO 
 Returns the capabilities of this multi-instance classifier for the
 relational data.
   getMultiInstanceCapabilities()   - 
Method in class weka.classifiers.mi. MISVM 
 Returns the capabilities of this multi-instance classifier for the
 relational data.
   getMultiInstanceCapabilities()   - 
Method in class weka.classifiers.mi. MIWrapper 
 Returns the capabilities of this multi-instance classifier for the
 relational data.
   getMultiInstanceCapabilities()   - 
Method in class weka.classifiers.mi. SimpleMI 
 Returns the capabilities of this multi-instance classifier for the
 relational data.
   getMultiInstanceCapabilities()   - 
Method in class weka.classifiers.mi.supportVector. MIPolyKernel 
 Returns the capabilities of this multi-instance kernel for the
 relational data.
   getMultiInstanceCapabilities()   - 
Method in class weka.classifiers.mi.supportVector. MIRBFKernel 
 Returns the capabilities of this multi-instance kernel for the
 relational data.
   getMultiInstanceCapabilities()   - 
Method in interface weka.core. MultiInstanceCapabilitiesHandler 
 Returns the capabilities of this multi-instance classifier for the
 relational data (i.e., the bags).
   getMultiInstanceCapabilities()   - 
Method in class weka.filters.unsupervised.attribute. MultiInstanceToPropositional 
 Returns the capabilities of this multi-instance filter for the
 relational data (i.e., the bags).
   getMultinomialWord()   - 
Method in class weka.classifiers.bayes. DMNBtext 
 Gets whether use binary text representation
   getMutationProb()   - 
Method in class weka.attributeSelection. GeneticSearch 
 get the probability of mutation
   getNaiveBayesModel()   - 
Method in class weka.classifiers.trees.j48. NBTreeNoSplit 
 Get the naive bayes model at this node
   getName()   - 
Method in class weka.classifiers.bayes. BayesNet 
 get name of the Bayes network
   getName()   - 
Method in class weka.core.pmml. Function 
  
   getName()   - 
Method in class weka.core.pmml. MiningFieldMetaInfo 
 Get the name of this field.
   getName()   - 
Method in class weka.core. PropertyPath.PathElement 
 returns the name of the property
   getName()   - 
Method in class weka.filters.unsupervised.attribute. AddExpression 
 Returns the name of the new attribute
   getName()   - 
Method in class weka.gui.visualize. VisualizePanel 
 Returns the name associated with this plot.
   getNameAtIndex(int)   - 
Method in class weka.gui. ResultHistoryPanel 
 Gets the name of theitem in the list at the specified index
   getNamedBuffer(String)   - 
Method in class weka.gui. ResultHistoryPanel 
 Gets the named buffer
   getNamedObject(String)   - 
Method in class weka.gui. ResultHistoryPanel 
 Get the named object from the list
   getNearestNeighbors()   - 
Method in class weka.filters.supervised.instance. SMOTE 
 Gets the number of nearest neighbors to use.
   getNearestNeighbourSearchAlgorithm()   - 
Method in class weka.classifiers.lazy. IBk 
 Returns the current nearestNeighbourSearch algorithm in use.
   getNearestNeighbourSearchAlgorithm()   - 
Method in class weka.classifiers.lazy. LWL 
 Returns the current nearestNeighbourSearch algorithm in use.
   getNegation()   - 
Method in class weka.associations. Tertius 
 Get the value of negation.
   getNegation()   - 
Method in class weka.associations.tertius. Literal 
  
   getNext(int)   - 
Method in class weka.classifiers.functions.supportVector. SMOset 
 Gets the next element in the set.
   getNextDebugVectorsInstance(Instances)   - 
Method in class weka.clusterers. XMeans 
 Read an instance from debug vectors file.
   getNextInstance(Instances)   - 
Method in class weka.core.converters. AbstractLoader 
  
   getNextInstance(Instances)   - 
Method in class weka.core.converters. ArffLoader 
 Read the data set incrementally---get the next instance in the data
 set or returns null if there are no
 more instances to get.
   getNextInstance(Instances)   - 
Method in class weka.core.converters. C45Loader 
 Read the data set incrementally---get the next instance in the data 
 set or returns null if there are no
 more instances to get.
   getNextInstance(Instances)   - 
Method in class weka.core.converters. CSVLoader 
 CSVLoader is unable to process a data set incrementally.
   getNextInstance(Instances)   - 
Method in class weka.core.converters. DatabaseLoader 
 Read the data set incrementally---get the next instance in the data 
 set or returns null if there are no
 more instances to get.
   getNextInstance(Instances)   - 
Method in class weka.core.converters. LibSVMLoader 
 LibSVmLoader is unable to process a data set incrementally.
   getNextInstance(Instances)   - 
Method in interface weka.core.converters. Loader 
 Read the data set incrementally---get the next instance in the data 
 set or returns null if there are no
 more instances to get.
   getNextInstance(Instances)   - 
Method in class weka.core.converters. SerializedInstancesLoader 
 Read the data set incrementally---get the next instance in the data 
 set or returns null if there are no
 more instances to get.
   getNextInstance(Instances)   - 
Method in class weka.core.converters. SVMLightLoader 
 SVMLightLoader is unable to process a data set incrementally.
   getNextInstance(Instances)   - 
Method in class weka.core.converters. TextDirectoryLoader 
 TextDirectoryLoader is unable to process a data set incrementally.
   getNextInstance(Instances)   - 
Method in class weka.core.converters. XRFFLoader 
 XRFFLoader is unable to process a data set incrementally.
   getNGramMaxSize()   - 
Method in class weka.core.tokenizers. NGramTokenizer 
 Gets the max N of the NGram.
   getNGramMinSize()   - 
Method in class weka.core.tokenizers. NGramTokenizer 
 Gets the min N of the NGram.
   getNoClass()   - 
Method in class weka.core. TestInstances 
 whether no class attribute is generated
   getNode(String)   - 
Method in class weka.classifiers.bayes.net. BIFReader 
 getNode finds the index of the node with name sNodeName
 and throws an exception if no such node can be found.
   getNode(String)   - 
Method in class weka.classifiers.bayes.net. EditableBayesNet 
 returns index of node with given name.
   getNode(String)   - 
Method in class weka.classifiers.bayes.net. MarginCalculator 
  
   getNode(String)   - 
Method in class weka.core.xml. XMLDocument 
 Returns the node represented by the XPath expression.
   getNode2(String)   - 
Method in class weka.classifiers.bayes.net. EditableBayesNet 
 returns index of node with given name, or -1 if no such node exists
   getNodeName(int)   - 
Method in class weka.classifiers.bayes. BayesNet 
 get name of a node in the Bayes network
   getNodes()   - 
Method in class weka.classifiers.trees.ft. FTtree 
 Return a list of all inner nodes in the tree
   getNodes(Vector)   - 
Method in class weka.classifiers.trees.ft. FTtree 
 Fills a list with all inner nodes in the tree
   getNodes()   - 
Method in class weka.classifiers.trees.lmt. LMTNode 
 Return a list of all inner nodes in the tree
   getNodes(Vector)   - 
Method in class weka.classifiers.trees.lmt. LMTNode 
 Fills a list with all inner nodes in the tree
   getNodes()   - 
Method in class weka.gui.graphvisualizer. HierarchicalBCEngine 
 give access to set of graph nodes
   getNodes()   - 
Method in interface weka.gui.graphvisualizer. LayoutEngine 
 give access to set of graph nodes
   getNodeSplitter()   - 
Method in class weka.core.neighboursearch. KDTree 
 Returns the splitting method currently in use to split the nodes of the
 KDTree.
   getNodeValue(int, int)   - 
Method in class weka.classifiers.bayes. BayesNet 
 get name of a particular value of a node
   getNoise()   - 
Method in class weka.classifiers.functions. GaussianProcesses 
 Get the value of noise.
   getNoisePercent()   - 
Method in class weka.datagenerators.classifiers.classification. LED24 
 Gets the noise percentage.
   getNoiseRate()   - 
Method in class weka.datagenerators.classifiers.regression. MexicanHat 
 Gets the gaussian noise rate.
   getNoiseRate()   - 
Method in class weka.datagenerators.clusterers. BIRCHCluster 
 Gets the percentage of noise set.
   getNoiseRate()   - 
Method in class weka.datagenerators.clusterers. SubspaceCluster 
 Gets the percentage of noise set.
   getNoiseThreshold()   - 
Method in class weka.associations. Tertius 
 Get the value of noiseThreshold.
   getNoiseVariance()   - 
Method in class weka.datagenerators.classifiers.regression. MexicanHat 
 Gets the noise variance
   getNominalAttributes()   - 
Method in class weka.core.converters. CSVLoader 
 Returns the current attribute range to be forced to type nominal.
   getNominalCols()   - 
Method in class weka.datagenerators. ClusterGenerator 
 returns the range of nominal attributes
   getNominalIndices()   - 
Method in class weka.filters.unsupervised.instance. RemoveWithValues 
 Get the set of nominal value indices that will be used for selection
   getNominalLabels()   - 
Method in class weka.filters.unsupervised.attribute. Add 
 Get the list of labels for nominal attribute creation.
   getNominalToBinaryFilter()   - 
Method in class weka.classifiers.functions. MultilayerPerceptron 
  
   getNoPruning()   - 
Method in class weka.classifiers.trees. REPTree 
 Get the value of NoPruning.
   getNoReplacement()   - 
Method in class weka.filters.supervised.instance. Resample 
 Gets whether instances are drawn with or without replacement.
   getNoReplacement()   - 
Method in class weka.filters.unsupervised.instance. Resample 
 Gets whether instances are drawn with or without replacement.
   getNorm()   - 
Method in class weka.filters.unsupervised.instance. Normalize 
 Get the instance's Norm.
   getNormalize()   - 
Method in class weka.attributeSelection. LatentSemanticAnalysis 
 Gets whether or not input data is to be normalized
   getNormalize()   - 
Method in class weka.classifiers.functions. LibLINEAR 
 whether to normalize input data
   getNormalize()   - 
Method in class weka.classifiers.functions. LibSVM 
 whether to normalize input data
   getNormalizeAttributes()   - 
Method in class weka.classifiers.functions. MultilayerPerceptron 
  
   getNormalizeDimWidths()   - 
Method in class weka.core.neighboursearch.balltrees. MedianOfWidestDimension 
 Whether we are normalizing the widths(ranges) of the dimensions (attributes) 
 or not.
   getNormalizeDocLength()   - 
Method in class weka.filters.unsupervised.attribute. StringToWordVector 
 Gets whether if the word frequencies for a document (instance) should
  be normalized or not.
   getNormalizeNodeWidth()   - 
Method in class weka.core.neighboursearch. KDTree 
 Gets the normalize flag.
   getNormalizeNumericClass()   - 
Method in class weka.classifiers.functions. MultilayerPerceptron 
  
   getNormalizeWordWeights()   - 
Method in class weka.classifiers.bayes. ComplementNaiveBayes 
 Returns true if the word weights for each class are to be normalized
   getNot()   - 
Method in class weka.datagenerators. Test 
 Negates the test.
   getNotCapabilities()   - 
Method in class weka.core. FindWithCapabilities 
 The "not to have" capabilities to search for.
   getNotes()   - 
Method in class weka.experiment. Experiment 
 Get the user notes.
   getNotUnifyNorm()   - 
Method in class weka.clusterers. sIB 
 Get whether to normalize instances to unify prior probability 
 before building the clusterer
   getNPointPrecision(Instances, int)   - 
Static method in class weka.classifiers.evaluation. ThresholdCurve 
 Calculates the n point precision result, which is the precision averaged
 over n evenly spaced (w.r.t recall) samples of the curve.
   getNrOfGoodOperations()   - 
Method in class weka.classifiers.bayes.net.search.local. LAGDHillClimber 
 Gets the number of "good operations"
   getNrOfLookAheadSteps()   - 
Method in class weka.classifiers.bayes.net.search.local. LAGDHillClimber 
 Gets the number of look-ahead steps
   getNrOfNodes()   - 
Method in class weka.classifiers.bayes. BayesNet 
 get number of nodes in the Bayes network
   getNrOfParents(int)   - 
Method in class weka.classifiers.bayes. BayesNet 
 get number of parents of a node in the network structure
   getNrOfParents()   - 
Method in class weka.classifiers.bayes.net. ParentSet 
 returns number of parents
   getNu()   - 
Method in class weka.classifiers.functions. LibSVM 
 Gets nu of nu-SVC, one-class SVM, and nu-SVR (default 0.5)
   getNumAntds()   - 
Method in class weka.classifiers.rules. ConjunctiveRule 
 Gets the number of antecedants
   getNumArcs()   - 
Method in class weka.datagenerators.classifiers.classification. BayesNet 
 Gets the number of arcs for the bayesian net
   getNumAttemptsOfGeneOption()   - 
Method in class weka.classifiers.rules. NNge 
 Gets the number of attempts for generalisation.
   getNumAttributes()   - 
Method in class weka.classifiers.lazy. LBR.Indexes 
 Returns the number of attributes in the dataset
   getNumAttributes()   - 
Method in class weka.core. TestInstances 
 returns the overall number of attributes (incl.
   getNumAttributes()   - 
Method in class weka.datagenerators.classifiers.classification. BayesNet 
 Gets the number of attributes that should be produced.
   getNumAttributes()   - 
Method in class weka.datagenerators.classifiers.classification. RandomRBF 
 Gets the number of attributes that should be produced.
   getNumAttributes()   - 
Method in class weka.datagenerators.classifiers.classification. RDG1 
 Gets the number of attributes that should be produced.
   getNumAttributes()   - 
Method in class weka.datagenerators. ClusterGenerator 
 Gets the number of attributes that should be produced.
   getNumAttributes()   - 
Method in class weka.filters.unsupervised.attribute. RandomSubset 
 Get the number of attributes (< 1 percentage, >= 1 absolute number).
   getNumAttributesSet()   - 
Method in class weka.classifiers.lazy. LBR.Indexes 
 Returns the number of attributes "in use"
   getNumberLiterals()   - 
Method in class weka.associations. Tertius 
 Get the value of numberLiterals.
   getNumberOfAttributes()   - 
Method in class weka.clusterers.forOPTICSAndDBScan.OPTICS_GUI. SERObject 
 Returns the number of Attributes of the specified database
   getNumberOfAttributes()   - 
Method in class weka.filters.unsupervised.attribute. RandomProjection 
 Gets the current number of attributes (dimensionality) to which the data 
 will be reduced to.
   getNumberOfGeneratedClusters()   - 
Method in class weka.clusterers.forOPTICSAndDBScan.OPTICS_GUI. SERObject 
 Returns the number of generated clusters
   getNumberOfGroups()   - 
Method in class weka.classifiers.meta. RotationForest 
 Get whether minGroup and maxGroup refer to the number of groups or their 
 size
   getNumBins()   - 
Method in class weka.classifiers.meta. RegressionByDiscretization 
 Gets the number of bins numeric attributes will be divided into
   getNumBoostingIterations()   - 
Method in class weka.classifiers.functions. SimpleLogistic 
 Get the value of numBoostingIterations.
   getNumBoostingIterations()   - 
Method in class weka.classifiers.trees. FT 
 Get the value of numBoostingIterations.
   getNumBoostingIterations()   - 
Method in class weka.classifiers.trees. LMT 
 Get the value of numBoostingIterations.
   getNumCentroids()   - 
Method in class weka.datagenerators.classifiers.classification. RandomRBF 
 Gets the number of centroids.
   getNumCiters()   - 
Method in class weka.classifiers.mi. CitationKNN 
 Returns the number of citers considered to estimate
 the class prediction of tests bags
   getNumClasses()   - 
Method in class weka.core. TestInstances 
 returns the current number of classes
   getNumClasses()   - 
Method in class weka.datagenerators.classifiers.classification. RandomRBF 
 Gets the number of classes the dataset should have.
   getNumClasses()   - 
Method in class weka.datagenerators.classifiers.classification. RDG1 
 Gets the number of classes the dataset should have.
   getNumClusters()   - 
Method in class weka.attributeSelection. UnsupervisedSubsetEvaluator 
 Return the number of clusters used by the subset evaluator
   getNumClusters()   - 
Method in class weka.classifiers.functions. RBFNetwork 
 Return the number of clusters to generate.
   getNumClusters()   - 
Method in class weka.clusterers. ClusterEvaluation 
 Return the number of clusters found for the most recent call to
 evaluateClusterer
   getNumClusters()   - 
Method in class weka.clusterers. EM 
 Get the number of clusters
   getNumClusters()   - 
Method in class weka.clusterers. FarthestFirst 
 gets the number of clusters to generate
   getNumClusters()   - 
Method in class weka.clusterers. HierarchicalClusterer 
  
   getNumClusters()   - 
Method in class weka.clusterers. sIB 
 Get the number of clusters
   getNumClusters()   - 
Method in class weka.clusterers. SimpleKMeans 
 gets the number of clusters to generate
   getNumClusters()   - 
Method in class weka.datagenerators.clusterers. BIRCHCluster 
 Gets the number of clusters the dataset should have.
   getNumComponents()   - 
Method in class weka.filters.supervised.attribute. PLSFilter 
 returns the maximum number of attributes to use.
   getNumCycles()   - 
Method in class weka.datagenerators.clusterers. BIRCHCluster 
 Gets the number of cycles.
   getNumDatasets()   - 
Method in class weka.experiment. PairedTTester 
 Gets the number of datasets in the resultsets
   getNumDatasets()   - 
Method in interface weka.experiment. Tester 
 Gets the number of datasets in the resultsets
   getNumDate()   - 
Method in class weka.core. CheckScheme 
 returns the current number of date attributes
   getNumDate()   - 
Method in class weka.core. TestInstances 
 returns the current number of date attributes
   getNumeric()   - 
Method in class weka.filters.unsupervised.attribute. MakeIndicator 
 Check if new attribute is to be numeric.
   getNumericColumns()   - 
Method in class weka.gui.sql. ResultSetHelper 
 returns an array that indicates whether a column is numeric or nor.
   getNumEvalsCached()   - 
Method in class weka.attributeSelection. LFSMethods 
  
   getNumEvalsTotal()   - 
Method in class weka.attributeSelection. LFSMethods 
  
   getNumExamples()   - 
Method in class weka.datagenerators. ClassificationGenerator 
 Gets the number of examples, given by option.
   getNumExamples()   - 
Method in class weka.datagenerators.classifiers.classification. BayesNet 
 Gets the number of examples, given by option.
   getNumExamples()   - 
Method in class weka.datagenerators. RegressionGenerator 
 Gets the number of examples, given by option.
   getNumExamplesAct()   - 
Method in class weka.datagenerators. DataGenerator 
 Gets the number of examples the dataset should have.
   getNumFeatures()   - 
Method in class weka.classifiers.trees. RandomForest 
 Get the number of features used in random selection.
   getNumFiles()   - 
Method in class weka.core. Debug.Log 
 returns the number of files being used
   getNumFoldersMIOption()   - 
Method in class weka.classifiers.rules. NNge 
 Gets the number of folder for mutual information.
   getNumFolds()   - 
Method in class weka.classifiers.bayes. BayesianLogisticRegression 
 Return the number of folds for CV-based hyperparameter selection
   getNumFolds()   - 
Method in class weka.classifiers.functions. SMO 
 Get the value of numFolds.
   getNumFolds()   - 
Method in class weka.classifiers.meta. CVParameterSelection 
 Gets the number of folds for the cross-validation.
   getNumFolds()   - 
Method in class weka.classifiers.meta. Dagging 
 Gets the number of folds to use for splitting the training set.
   getNumFolds()   - 
Method in class weka.classifiers.meta. LogitBoost 
 Get the value of NumFolds.
   getNumFolds()   - 
Method in class weka.classifiers.meta. MultiScheme 
 Gets the number of folds for cross-validation.
   getNumFolds()   - 
Method in class weka.classifiers.meta. Stacking 
 Gets the number of folds for the cross-validation.
   getNumFolds()   - 
Method in class weka.classifiers.mi. MISMO 
 Get the value of numFolds.
   getNumFolds()   - 
Method in class weka.classifiers.rules. PART 
 Get the value of numFolds.
   getNumFolds()   - 
Method in class weka.classifiers.trees. J48 
 Get the value of numFolds.
   getNumFolds()   - 
Method in class weka.classifiers.trees. RandomTree 
 Get the value of NumFolds.
   getNumFolds()   - 
Method in class weka.classifiers.trees. REPTree 
 Get the value of NumFolds.
   getNumFolds()   - 
Method in class weka.experiment. CrossValidationResultProducer 
 Get the value of NumFolds.
   getNumFolds()   - 
Method in class weka.filters.supervised.instance. StratifiedRemoveFolds 
 Gets the number of folds in which dataset is to be split into.
   getNumFolds()   - 
Method in class weka.filters.unsupervised.instance. RemoveFolds 
 Gets the number of folds in which dataset is to be split into.
   getNumFolds()   - 
Method in class weka.filters.unsupervised.instance. RemoveMisclassified 
 Gets the number of cross-validation folds used by the filter.
   getNumFoldsPruning()   - 
Method in class weka.classifiers.trees. BFTree 
 Set number of folds in internal cross-validation.
   getNumFoldsPruning()   - 
Method in class weka.classifiers.trees. SimpleCart 
 Set number of folds in internal cross-validation.
   getNumGeneratingModels()   - 
Method in interface weka.gui.boundaryvisualizer. DataGenerator 
 Returns the number of generating models used by this DataGenerator
   getNumGeneratingModels()   - 
Method in class weka.gui.boundaryvisualizer. KDDataGenerator 
 Return the number of kernels (there is one per training instance)
   getNumInnerNodes()   - 
Method in class weka.classifiers.trees.ft. FTtree 
 Method to count the number of inner nodes in the tree
   getNumInnerNodes()   - 
Method in class weka.classifiers.trees.lmt. LMTNode 
 Method to count the number of inner nodes in the tree
   getNumInputs()   - 
Method in class weka.classifiers.functions.neural. NeuralConnection 
  
   getNumInstances()   - 
Method in class weka.classifiers.lazy. LBR.Indexes 
 Returns the number of instances in the dataset
   getNumInstances()   - 
Method in class weka.classifiers.trees.m5. RuleNode 
 Return the number of instances that reach this node.
   getNumInstances()   - 
Method in class weka.core. CheckScheme 
 Gets the current number of instances to use for the datasets.
   getNumInstances()   - 
Method in class weka.core. TestInstances 
 returns the current number of instances to produce
   getNumInstances()   - 
Method in class weka.datagenerators.clusterers. SubspaceClusterDefinition 
  
   getNumInstances()   - 
Method in class weka.estimators. CheckEstimator 
 Gets the current number of instances to use for the datasets.
   getNumInstancesRelational()   - 
Method in class weka.core. CheckScheme 
 returns the current number of instances in relational/bag attributes to produce
   getNumInstancesRelational()   - 
Method in class weka.core. TestInstances 
 returns the current number of instances in relational/bag attributes to produce
   getNumInstancesSet()   - 
Method in class weka.classifiers.lazy. LBR.Indexes 
 Returns the number of instances "in use"
   getNumIrrelevant()   - 
Method in class weka.datagenerators.classifiers.classification. RDG1 
 Gets the number of irrelevant attributes.
   getNumIterations()   - 
Method in class weka.classifiers.bayes. DMNBtext 
 Gets the number of iterations to be performed
   getNumIterations()   - 
Method in class weka.classifiers.functions. VotedPerceptron 
 Get the value of NumIterations.
   getNumIterations()   - 
Method in class weka.classifiers.functions. Winnow 
 Get the value of numIterations.
   getNumIterations()   - 
Method in class weka.classifiers. IteratedSingleClassifierEnhancer 
 Gets the number of bagging iterations
   getNumIterations()   - 
Method in class weka.classifiers.meta. MetaCost 
 Gets the number of bagging iterations
   getNumKernels()   - 
Method in class weka.estimators. KernelEstimator 
 Return the number of kernels in this kernel estimator
   getNumLeaves()   - 
Method in class weka.classifiers.trees.ft. FTtree 
 Returns the number of leaves in the tree.
   getNumLeaves()   - 
Method in class weka.classifiers.trees.lmt. LMTNode 
 Returns the number of leaves in the tree.
   getNumLeaves()   - 
Method in class weka.core.neighboursearch.balltrees. BallTreeConstructor 
 Returns the number of leaves in the built tree.
   getNumNeighbours()   - 
Method in class weka.attributeSelection. ReliefFAttributeEval 
 Get the number of nearest neighbours
   getNumNeighbours()   - 
Method in class weka.classifiers.mi. MINND 
 Returns the number of nearest neighbours to estimate
 the class prediction of tests bags
   getNumNodes()   - 
Method in class weka.core.neighboursearch.balltrees. BallTreeConstructor 
 Returns the number of nodes (internal + leaf) 
 in the built tree.
   getNumNominal()   - 
Method in class weka.core. CheckScheme 
 returns the current number of nominal attributes
   getNumNominal()   - 
Method in class weka.core. TestInstances 
 returns the current number of nominal attributes
   getNumNominalValues()   - 
Method in class weka.core. TestInstances 
 returns the current number of values for nominal attributes
   getNumNumeric()   - 
Method in class weka.core. CheckScheme 
 returns the current number of numeric attributes
   getNumNumeric()   - 
Method in class weka.core. TestInstances 
 returns the current number of numeric attributes
   getNumNumeric()   - 
Method in class weka.datagenerators.classifiers.classification. RDG1 
 Gets the number of numerical attributes.
   getNumOfBoostingIterations()   - 
Method in class weka.classifiers.trees. ADTree 
 Gets the number of boosting iterations.
   getNumOfBoostingIterations()   - 
Method in class weka.classifiers.trees. LADTree 
 Gets the number of boosting iterations.
   getNumOfBranches()   - 
Method in class weka.classifiers.trees.adtree. Splitter 
 Gets the number of branches of the split.
   getNumOfBranches()   - 
Method in class weka.classifiers.trees.adtree. TwoWayNominalSplit 
 Gets the number of branches of the split.
   getNumOfBranches()   - 
Method in class weka.classifiers.trees.adtree. TwoWayNumericSplit 
 Gets the number of branches of the split.
   getNumOutputs()   - 
Method in class weka.classifiers.functions.neural. NeuralConnection 
  
   getNumQueries()   - 
Method in class weka.core.neighboursearch. PerformanceStats 
 Returns the number of queries.
   getNumReferences()   - 
Method in class weka.classifiers.mi. CitationKNN 
 Returns the number of references considered to estimate
 the class prediction of tests bags
   getNumRegressions()   - 
Method in class weka.classifiers.functions. SimpleLogistic 
 Get the number of LogitBoost iterations performed (= the number of 
 regression functions fit by LogitBoost).
   getNumRegressions()   - 
Method in class weka.classifiers.trees.lmt. LogisticBase 
 The number of LogitBoost iterations performed (= the number of simple 
 regression functions fit).
   getNumRelational()   - 
Method in class weka.core. CheckScheme 
 returns the current number of relational attributes
   getNumRelational()   - 
Method in class weka.core. TestInstances 
 returns the current number of relational attributes
   getNumRelationalDate()   - 
Method in class weka.core. TestInstances 
 returns the current number of date attributes in a relational attribute
   getNumRelationalNominal()   - 
Method in class weka.core. TestInstances 
 returns the current number of nominal attributes in a relational attribute
   getNumRelationalNominalValues()   - 
Method in class weka.core. TestInstances 
 returns the current number of values for nominal attributes in a relational attribute
   getNumRelationalNumeric()   - 
Method in class weka.core. TestInstances 
 returns the current number of numeric attributes in a relational attribute
   getNumRelationalString()   - 
Method in class weka.core. TestInstances 
 returns the current number of string attributes in a relational attribute
   getNumRestarts()   - 
Method in class weka.clusterers. sIB 
 Get the number of restarts
   getNumResultsets()   - 
Method in class weka.experiment. PairedTTester 
 Gets the number of resultsets in the data.
   getNumResultsets()   - 
Method in interface weka.experiment. Tester 
 Gets the number of resultsets in the data.
   getNumRules()   - 
Method in class weka.associations. Apriori 
 Get the value of numRules.
   getNumRules()   - 
Method in class weka.associations. PredictiveApriori 
 Get the value of the number of required rules.
   getNumRulesToFind()   - 
Method in class weka.associations. FPGrowth 
 Get the number of rules to find.
   getNumRuns()   - 
Method in class weka.classifiers.meta. LogitBoost 
 Get the value of NumRuns.
   getNumSamplesPerRegion()   - 
Method in class weka.gui.boundaryvisualizer. BoundaryPanel 
 Get the number of points to sample from a region (fixed dimensions).
   getNumString()   - 
Method in class weka.core. CheckScheme 
 returns the current number of string attributes
   getNumString()   - 
Method in class weka.core. TestInstances 
 returns the current number of string attributes
   getNumSubCmtys()   - 
Method in class weka.classifiers.meta. MultiBoostAB 
 Get the number of sub committees to use
   getNumSubsetSizeCVFolds()   - 
Method in class weka.attributeSelection. SubsetSizeForwardSelection 
 Get the number of cross validation folds for subset size determination
 (default = 5).
   getNumSymbols()   - 
Method in class weka.classifiers.bayes.net.estimate. DiscreteEstimatorBayes 
 Gets the number of symbols this estimator operates with
   getNumSymbols()   - 
Method in class weka.estimators. DiscreteEstimator 
 Gets the number of symbols this estimator operates with
   getNumTestingNoises()   - 
Method in class weka.classifiers.mi. MINND 
 Returns The number of nearest neighbour instances in the 
 selection of noises in the test data
   getNumToSelect()   - 
Method in class weka.attributeSelection. GreedyStepwise 
 Gets the number of attributes to be retained.
   getNumToSelect()   - 
Method in class weka.attributeSelection. RaceSearch 
 Gets the number of attributes to be retained.
   getNumToSelect()   - 
Method in interface weka.attributeSelection. RankedOutputSearch 
 Gets the user specified number of attributes to be retained.
   getNumToSelect()   - 
Method in class weka.attributeSelection. Ranker 
 Gets the number of attributes to be retained.
   getNumTraining()   - 
Method in class weka.classifiers.lazy. IBk 
 Get the number of training instances the classifier is currently using.
   getNumTrainingNoises()   - 
Method in class weka.classifiers.mi. MINND 
 Returns the number of nearest neighbour instances in the 
 selection of noises in the training data
   getNumTrees()   - 
Method in class weka.classifiers.trees. RandomForest 
 Get the value of numTrees.
   getNumUsedAttributes()   - 
Method in class weka.attributeSelection. LinearForwardSelection 
 Get the number of top-ranked attributes that taken into account by the
 search process.
   getNumUsedAttributes()   - 
Method in class weka.attributeSelection. SubsetSizeForwardSelection 
 Get the number of top-ranked attributes that taken into account by the
 search process.
   getNumValues()   - 
Method in class weka.datagenerators.clusterers. SubspaceCluster 
 returns array that stores the number of values for a nominal attribute.
   getNumValues()   - 
Method in class weka.filters.unsupervised.instance. RemoveFrequentValues 
 Gets how many values are retained
   getNumXValFolds()   - 
Method in class weka.classifiers.meta. ThresholdSelector 
 Get the number of folds used for cross-validation.
   getObject()   - 
Method in class weka.clusterers.forOPTICSAndDBScan.Utils. PriorityQueueElement 
 Returns the object
   getObject()   - 
Method in class weka.clusterers.forOPTICSAndDBScan.Utils. UpdateQueueElement 
 Returns the object
   getObject()   - 
Method in class weka.core. CheckGOE 
 Get the object used in the tests.
   getObject()   - 
Method in class weka.core. SerializedObject 
 Returns a serialized object.
   getObjectKey()   - 
Method in class weka.clusterers.forOPTICSAndDBScan.Utils. UpdateQueueElement 
 Returns the key
   getObservedFrequency()   - 
Method in class weka.associations.tertius. Rule 
 Get the observed frequency of counter-instances of this rule in the dataset.
   getObservedNumber()   - 
Method in class weka.associations.tertius. Rule 
 Get the observed number of counter-instances of this rule in the dataset.
   getOmega()   - 
Method in class weka.classifiers.functions.supportVector. Puk 
 Gets the omega value.
   getOnDemandDirectory()   - 
Method in class weka.attributeSelection. CostSensitiveASEvaluation 
 Returns the directory that will be searched for cost files when
 loading on demand.
   getOnDemandDirectory()   - 
Method in class weka.classifiers.meta. CostSensitiveClassifier 
 Returns the directory that will be searched for cost files when
 loading on demand.
   getOnDemandDirectory()   - 
Method in class weka.classifiers.meta. MetaCost 
 Returns the directory that will be searched for cost files when
 loading on demand.
   getOnDemandDirectory()   - 
Method in class weka.experiment. CostSensitiveClassifierSplitEvaluator 
 Returns the directory that will be searched for cost files when
 loading on demand.
   getOneElements(Instances)   - 
Static method in class weka.associations.gsp. Element 
 Returns all events of the given data set as Elements containing a single 
 event.
   getOptimistic()   - 
Method in class weka.associations.tertius. Rule 
 Get the optimistic estimate of the confirmation obtained by refining
 this rule.
   getOptimizations()   - 
Method in class weka.classifiers.rules. JRip 
 Gets the the number of optimization runs
   getOption(char, String[])   - 
Static method in class weka.core. Utils 
 Gets an option indicated by a flag "-Char" from the given array
 of strings.
   getOption(String, String[])   - 
Static method in class weka.core. Utils 
 Gets an option indicated by a flag "-String" from the given array
 of strings.
   getOptionHandler()   - 
Method in class weka.core. CheckOptionHandler 
 Get the OptionHandler used in the tests.
   getOptionPos(char, String[])   - 
Static method in class weka.core. Utils 
 Gets the index of an option or flag indicated by a flag "-Char" from 
 the given array of strings.
   getOptionPos(String, String[])   - 
Static method in class weka.core. Utils 
 Gets the index of an option or flag indicated by a flag "-String" from 
 the given array of strings.
   getOptions()   - 
Method in class weka.associations. Apriori 
 Gets the current settings of the Apriori object.
   getOptions()   - 
Method in class weka.associations. CheckAssociator 
 Gets the current settings of the CheckAssociator.
   getOptions()   - 
Method in class weka.associations. FilteredAssociator 
 Gets the current settings of the Associator.
   getOptions()   - 
Method in class weka.associations. FPGrowth 
 Gets the current settings of the classifier.
   getOptions()   - 
Method in class weka.associations. GeneralizedSequentialPatterns 
 Returns an Array containing the current options settings.
   getOptions()   - 
Method in class weka.associations. PredictiveApriori 
 Gets the current settings of the PredictiveApriori object.
   getOptions()   - 
Method in class weka.associations. SingleAssociatorEnhancer 
 Gets the current settings of the associator.
   getOptions()   - 
Method in class weka.associations. Tertius 
 Gets the current settings of the Tertius object.
   getOptions()   - 
Method in class weka.attributeSelection. BestFirst 
 Gets the current settings of BestFirst.
   getOptions()   - 
Method in class weka.attributeSelection. CfsSubsetEval 
 Gets the current settings of CfsSubsetEval
   getOptions()   - 
Method in class weka.attributeSelection. CheckAttributeSelection 
 Gets the current settings of the CheckAttributeSelection.
   getOptions()   - 
Method in class weka.attributeSelection. ChiSquaredAttributeEval 
 Gets the current settings.
   getOptions()   - 
Method in class weka.attributeSelection. ClassifierSubsetEval 
 Gets the current settings of ClassifierSubsetEval
   getOptions()   - 
Method in class weka.attributeSelection. CostSensitiveASEvaluation 
 Gets the current settings of the subset evaluator.
   getOptions()   - 
Method in class weka.attributeSelection. ExhaustiveSearch 
 Gets the current settings of RandomSearch.
   getOptions()   - 
Method in class weka.attributeSelection. FilteredAttributeEval 
 Gets the current settings of the subset evaluator.
   getOptions()   - 
Method in class weka.attributeSelection. FilteredSubsetEval 
 Gets the current settings of the subset evaluator.
   getOptions()   - 
Method in class weka.attributeSelection. GainRatioAttributeEval 
 Gets the current settings of WrapperSubsetEval.
   getOptions()   - 
Method in class weka.attributeSelection. GeneticSearch 
 Gets the current settings of ReliefFAttributeEval.
   getOptions()   - 
Method in class weka.attributeSelection. GreedyStepwise 
 Gets the current settings of ReliefFAttributeEval.
   getOptions()   - 
Method in class weka.attributeSelection. InfoGainAttributeEval 
 Gets the current settings of WrapperSubsetEval.
   getOptions()   - 
Method in class weka.attributeSelection. LatentSemanticAnalysis 
 Gets the current settings of LatentSemanticAnalysis
   getOptions()   - 
Method in class weka.attributeSelection. LinearForwardSelection 
 Gets the current settings of LinearForwardSelection.
   getOptions()   - 
Method in class weka.attributeSelection. OneRAttributeEval 
 returns the current setup.
   getOptions()   - 
Method in class weka.attributeSelection. PrincipalComponents 
 Gets the current settings of PrincipalComponents
   getOptions()   - 
Method in class weka.attributeSelection. RaceSearch 
 Gets the current settings of BestFirst.
   getOptions()   - 
Method in class weka.attributeSelection. RandomSearch 
 Gets the current settings of RandomSearch.
   getOptions()   - 
Method in class weka.attributeSelection. Ranker 
 Gets the current settings of ReliefFAttributeEval.
   getOptions()   - 
Method in class weka.attributeSelection. RankSearch 
 Gets the current settings of WrapperSubsetEval.
   getOptions()   - 
Method in class weka.attributeSelection. ReliefFAttributeEval 
 Gets the current settings of ReliefFAttributeEval.
   getOptions()   - 
Method in class weka.attributeSelection. ScatterSearchV1 
 Gets the current settings of ScatterSearchV1.
   getOptions()   - 
Method in class weka.attributeSelection. SubsetSizeForwardSelection 
 Gets the current settings of LinearForwardSelection.
   getOptions()   - 
Method in class weka.attributeSelection. SVMAttributeEval 
 Gets the current settings of SVMAttributeEval
   getOptions()   - 
Method in class weka.attributeSelection. SymmetricalUncertAttributeEval 
 Gets the current settings of WrapperSubsetEval.
   getOptions()   - 
Method in class weka.attributeSelection. WrapperSubsetEval 
 Gets the current settings of WrapperSubsetEval.
   getOptions()   - 
Method in class weka.classifiers.bayes. AODE 
 Gets the current settings of the classifier.
   getOptions()   - 
Method in class weka.classifiers.bayes. AODEsr 
 Gets the current settings of the classifier.
   getOptions()   - 
Method in class weka.classifiers.bayes. BayesianLogisticRegression 
  
   getOptions()   - 
Method in class weka.classifiers.bayes. BayesNet 
 Gets the current settings of the classifier.
   getOptions()   - 
Method in class weka.classifiers.bayes. ComplementNaiveBayes 
 Gets the current settings of the classifier.
   getOptions()   - 
Method in class weka.classifiers.bayes. DMNBtext 
 Gets the current settings of the classifier.
   getOptions()   - 
Method in class weka.classifiers.bayes. NaiveBayes 
 Gets the current settings of the classifier.
   getOptions()   - 
Method in class weka.classifiers.bayes.net. BayesNetGenerator 
 Gets the current settings of the classifier.
   getOptions()   - 
Method in class weka.classifiers.bayes.net.estimate. BayesNetEstimator 
 Gets the current settings of the classifier.
   getOptions()   - 
Method in class weka.classifiers.bayes.net.estimate. BMAEstimator 
 Gets the current settings of the classifier.
   getOptions()   - 
Method in class weka.classifiers.bayes.net.estimate. MultiNomialBMAEstimator 
 Gets the current settings of the classifier.
   getOptions()   - 
Method in class weka.classifiers.bayes.net.search.ci. ICSSearchAlgorithm 
 Gets the current settings of the Classifier.
   getOptions()   - 
Method in class weka.classifiers.bayes.net.search.fixed. FromFile 
 Gets the current settings of the search algorithm.
   getOptions()   - 
Method in class weka.classifiers.bayes.net.search.global. GeneticSearch 
 Gets the current settings of the search algorithm.
   getOptions()   - 
Method in class weka.classifiers.bayes.net.search.global. GlobalScoreSearchAlgorithm 
 Gets the current settings of the search algorithm.
   getOptions()   - 
Method in class weka.classifiers.bayes.net.search.global. HillClimber 
 Gets the current settings of the search algorithm.
   getOptions()   - 
Method in class weka.classifiers.bayes.net.search.global. K2 
 Gets the current settings of the search algorithm.
   getOptions()   - 
Method in class weka.classifiers.bayes.net.search.global. RepeatedHillClimber 
 Gets the current settings of the search algorithm.
   getOptions()   - 
Method in class weka.classifiers.bayes.net.search.global. SimulatedAnnealing 
 Gets the current settings of the search algorithm.
   getOptions()   - 
Method in class weka.classifiers.bayes.net.search.global. TabuSearch 
 Gets the current settings of the search algorithm.
   getOptions()   - 
Method in class weka.classifiers.bayes.net.search.global. TAN 
 Gets the current settings of the Classifier.
   getOptions()   - 
Method in class weka.classifiers.bayes.net.search.local. GeneticSearch 
 Gets the current settings of the search algorithm.
   getOptions()   - 
Method in class weka.classifiers.bayes.net.search.local. HillClimber 
 Gets the current settings of the search algorithm.
   getOptions()   - 
Method in class weka.classifiers.bayes.net.search.local. K2 
 Gets the current settings of the search algorithm.
   getOptions()   - 
Method in class weka.classifiers.bayes.net.search.local. LAGDHillClimber 
 Gets the current settings of the search algorithm.
   getOptions()   - 
Method in class weka.classifiers.bayes.net.search.local. LocalScoreSearchAlgorithm 
 Gets the current settings of the search algorithm.
   getOptions()   - 
Method in class weka.classifiers.bayes.net.search.local. RepeatedHillClimber 
 Gets the current settings of the search algorithm.
   getOptions()   - 
Method in class weka.classifiers.bayes.net.search.local. SimulatedAnnealing 
 Gets the current settings of the search algorithm.
   getOptions()   - 
Method in class weka.classifiers.bayes.net.search.local. TabuSearch 
 Gets the current settings of the search algorithm.
   getOptions()   - 
Method in class weka.classifiers.bayes.net.search.local. TAN 
 Gets the current settings of the Classifier.
   getOptions()   - 
Method in class weka.classifiers.bayes.net.search. SearchAlgorithm 
 Gets the current settings of the Classifier.
   getOptions()   - 
Method in class weka.classifiers.bayes. WAODE 
 Gets the current settings of the filter.
   getOptions()   - 
Method in class weka.classifiers. BVDecompose 
 Gets the current settings of the CheckClassifier.
   getOptions()   - 
Method in class weka.classifiers. BVDecomposeSegCVSub 
 Gets the current settings of the CheckClassifier.
   getOptions()   - 
Method in class weka.classifiers. CheckClassifier 
 Gets the current settings of the CheckClassifier.
   getOptions()   - 
Method in class weka.classifiers. CheckSource 
 Gets the current settings of the Classifier.
   getOptions()   - 
Method in class weka.classifiers. Classifier 
 Gets the current settings of the Classifier.
   getOptions()   - 
Method in class weka.classifiers.functions. GaussianProcesses 
 Gets the current settings of the classifier.
   getOptions()   - 
Method in class weka.classifiers.functions. LeastMedSq 
 Gets the current option settings for the OptionHandler.
   getOptions()   - 
Method in class weka.classifiers.functions. LibLINEAR 
 Returns the current options
   getOptions()   - 
Method in class weka.classifiers.functions. LibSVM 
 Returns the current options
   getOptions()   - 
Method in class weka.classifiers.functions. LinearRegression 
 Gets the current settings of the classifier.
   getOptions()   - 
Method in class weka.classifiers.functions. Logistic 
 Gets the current settings of the classifier.
   getOptions()   - 
Method in class weka.classifiers.functions. MultilayerPerceptron 
 Gets the current settings of NeuralNet.
   getOptions()   - 
Method in class weka.classifiers.functions. PaceRegression 
 Gets the current settings of the classifier.
   getOptions()   - 
Method in class weka.classifiers.functions. PLSClassifier 
 returns the options of the current setup
   getOptions()   - 
Method in class weka.classifiers.functions. RBFNetwork 
 Gets the current settings of the classifier.
   getOptions()   - 
Method in class weka.classifiers.functions. SimpleLogistic 
 Gets the current settings of the Classifier.
   getOptions()   - 
Method in class weka.classifiers.functions. SMO 
 Gets the current settings of the classifier.
   getOptions()   - 
Method in class weka.classifiers.functions. SMOreg 
 Gets the current settings of the classifier.
   getOptions()   - 
Method in class weka.classifiers.functions. SPegasos 
 Gets the current settings of the classifier.
   getOptions()   - 
Method in class weka.classifiers.functions.supportVector. CachedKernel 
 Gets the current settings of the Kernel.
   getOptions()   - 
Method in class weka.classifiers.functions.supportVector. CheckKernel 
 Gets the current settings of the CheckKernel.
   getOptions()   - 
Method in class weka.classifiers.functions.supportVector. Kernel 
 Gets the current settings of the Kernel.
   getOptions()   - 
Method in class weka.classifiers.functions.supportVector. PolyKernel 
 Gets the current settings of the Kernel.
   getOptions()   - 
Method in class weka.classifiers.functions.supportVector. PrecomputedKernelMatrixKernel 
 Gets the current settings of the Kernel.
   getOptions()   - 
Method in class weka.classifiers.functions.supportVector. Puk 
 Gets the current settings of the Kernel.
   getOptions()   - 
Method in class weka.classifiers.functions.supportVector. RBFKernel 
 Gets the current settings of the Kernel.
   getOptions()   - 
Method in class weka.classifiers.functions.supportVector. RegOptimizer 
 Gets the current settings of the classifier.
   getOptions()   - 
Method in class weka.classifiers.functions.supportVector. RegSMO 
 Gets the current settings of the classifier.
   getOptions()   - 
Method in class weka.classifiers.functions.supportVector. RegSMOImproved 
 Gets the current settings of the object.
   getOptions()   - 
Method in class weka.classifiers.functions.supportVector. StringKernel 
 Gets the current settings of the Kernel.
   getOptions()   - 
Method in class weka.classifiers.functions. VotedPerceptron 
 Gets the current settings of the classifier.
   getOptions()   - 
Method in class weka.classifiers.functions. Winnow 
 Gets the current settings of the classifier.
   getOptions()   - 
Method in class weka.classifiers. IteratedSingleClassifierEnhancer 
 Gets the current settings of the classifier.
   getOptions()   - 
Method in class weka.classifiers.lazy. IBk 
 Gets the current settings of IBk.
   getOptions()   - 
Method in class weka.classifiers.lazy. KStar 
 Gets the current settings of K*.
   getOptions()   - 
Method in class weka.classifiers.lazy. LWL 
 Gets the current settings of the classifier.
   getOptions()   - 
Method in class weka.classifiers.meta. AdaBoostM1 
 Gets the current settings of the Classifier.
   getOptions()   - 
Method in class weka.classifiers.meta. AdditiveRegression 
 Gets the current settings of the Classifier.
   getOptions()   - 
Method in class weka.classifiers.meta. AttributeSelectedClassifier 
 Gets the current settings of the Classifier.
   getOptions()   - 
Method in class weka.classifiers.meta. Bagging 
 Gets the current settings of the Classifier.
   getOptions()   - 
Method in class weka.classifiers.meta. ClassificationViaClustering 
 returns the options of the current setup
   getOptions()   - 
Method in class weka.classifiers.meta. CostSensitiveClassifier 
 Gets the current settings of the Classifier.
   getOptions()   - 
Method in class weka.classifiers.meta. CVParameterSelection 
 Gets the current settings of the Classifier.
   getOptions()   - 
Method in class weka.classifiers.meta. Dagging 
 Gets the current settings of the Classifier.
   getOptions()   - 
Method in class weka.classifiers.meta. Decorate 
 Gets the current settings of the Classifier.
   getOptions()   - 
Method in class weka.classifiers.meta. FilteredClassifier 
 Gets the current settings of the Classifier.
   getOptions()   - 
Method in class weka.classifiers.meta. GridSearch 
 returns the options of the current setup
   getOptions()   - 
Method in class weka.classifiers.meta. LogitBoost 
 Gets the current settings of the Classifier.
   getOptions()   - 
Method in class weka.classifiers.meta. MetaCost 
 Gets the current settings of the Classifier.
   getOptions()   - 
Method in class weka.classifiers.meta. MultiBoostAB 
 Gets the current settings of the Classifier.
   getOptions()   - 
Method in class weka.classifiers.meta. MultiClassClassifier 
 Gets the current settings of the Classifier.
   getOptions()   - 
Method in class weka.classifiers.meta. MultiScheme 
 Gets the current settings of the Classifier.
   getOptions()   - 
Method in class weka.classifiers.meta. OrdinalClassClassifier 
 Gets the current settings of the Classifier.
   getOptions()   - 
Method in class weka.classifiers.meta. RacedIncrementalLogitBoost 
 Gets the current settings of the Classifier.
   getOptions()   - 
Method in class weka.classifiers.meta. RandomSubSpace 
 Gets the current settings of the Classifier.
   getOptions()   - 
Method in class weka.classifiers.meta. RegressionByDiscretization 
 Gets the current settings of the Classifier.
   getOptions()   - 
Method in class weka.classifiers.meta. RotationForest 
 Gets the current settings of the Classifier.
   getOptions()   - 
Method in class weka.classifiers.meta. Stacking 
 Gets the current settings of the Classifier.
   getOptions()   - 
Method in class weka.classifiers.meta. ThresholdSelector 
 Gets the current settings of the Classifier.
   getOptions()   - 
Method in class weka.classifiers.meta. Vote 
 Gets the current settings of Vote.
   getOptions()   - 
Method in class weka.classifiers.mi. CitationKNN 
 Gets the current option settings for the OptionHandler.
   getOptions()   - 
Method in class weka.classifiers.mi. MDD 
 Gets the current settings of the classifier.
   getOptions()   - 
Method in class weka.classifiers.mi. MIBoost 
 Gets the current settings of the classifier.
   getOptions()   - 
Method in class weka.classifiers.mi. MIDD 
 Gets the current settings of the classifier.
   getOptions()   - 
Method in class weka.classifiers.mi. MIEMDD 
 Gets the current settings of the classifier.
   getOptions()   - 
Method in class weka.classifiers.mi. MILR 
 Gets the current settings of the classifier.
   getOptions()   - 
Method in class weka.classifiers.mi. MINND 
 Gets the current settings of the Classifier.
   getOptions()   - 
Method in class weka.classifiers.mi. MIOptimalBall 
 Gets the current settings of the classifier.
   getOptions()   - 
Method in class weka.classifiers.mi. MISMO 
 Gets the current settings of the classifier.
   getOptions()   - 
Method in class weka.classifiers.mi. MISVM 
 Gets the current settings of the classifier.
   getOptions()   - 
Method in class weka.classifiers.mi. MIWrapper 
 Gets the current settings of the Classifier.
   getOptions()   - 
Method in class weka.classifiers.mi. SimpleMI 
 Gets the current settings of the Classifier.
   getOptions()   - 
Method in class weka.classifiers.misc. SerializedClassifier 
 returns the options of the current setup
   getOptions()   - 
Method in class weka.classifiers.misc. VFI 
 Gets the current settings of VFI
   getOptions()   - 
Method in class weka.classifiers. MultipleClassifiersCombiner 
 Gets the current settings of the Classifier.
   getOptions()   - 
Method in class weka.classifiers. RandomizableClassifier 
 Gets the current settings of the classifier.
   getOptions()   - 
Method in class weka.classifiers. RandomizableIteratedSingleClassifierEnhancer 
 Gets the current settings of the classifier.
   getOptions()   - 
Method in class weka.classifiers. RandomizableMultipleClassifiersCombiner 
 Gets the current settings of the classifier.
   getOptions()   - 
Method in class weka.classifiers. RandomizableSingleClassifierEnhancer 
 Gets the current settings of the classifier.
   getOptions()   - 
Method in class weka.classifiers.rules. ConjunctiveRule 
 Gets the current settings of the Classifier.
   getOptions()   - 
Method in class weka.classifiers.rules. DecisionTable 
 Gets the current settings of the classifier.
   getOptions()   - 
Method in class weka.classifiers.rules. DTNB 
 Gets the current settings of the classifier.
   getOptions()   - 
Method in class weka.classifiers.rules. JRip 
 Gets the current settings of the Classifier.
   getOptions()   - 
Method in class weka.classifiers.rules. NNge 
 Gets the current option settings for the OptionHandler.
   getOptions()   - 
Method in class weka.classifiers.rules. OneR 
 Gets the current settings of the OneR classifier.
   getOptions()   - 
Method in class weka.classifiers.rules. PART 
 Gets the current settings of the Classifier.
   getOptions()   - 
Method in class weka.classifiers.rules. Ridor 
 Gets the current settings of the Classifier.
   getOptions()   - 
Method in class weka.classifiers. SingleClassifierEnhancer 
 Gets the current settings of the Classifier.
   getOptions()   - 
Method in class weka.classifiers.trees. ADTree 
 Gets the current settings of ADTree.
   getOptions()   - 
Method in class weka.classifiers.trees. BFTree 
 Gets the current settings of the Classifier.
   getOptions()   - 
Method in class weka.classifiers.trees. FT 
 Gets the current settings of the Classifier.
   getOptions()   - 
Method in class weka.classifiers.trees. J48 
 Gets the current settings of the Classifier.
   getOptions()   - 
Method in class weka.classifiers.trees. J48graft 
 Gets the current settings of the Classifier.
   getOptions()   - 
Method in class weka.classifiers.trees. LADTree 
 Gets the current settings of ADTree.
   getOptions()   - 
Method in class weka.classifiers.trees. LMT 
 Gets the current settings of the Classifier.
   getOptions()   - 
Method in class weka.classifiers.trees.m5. M5Base 
 Gets the current settings of the classifier.
   getOptions()   - 
Method in class weka.classifiers.trees. M5P 
 Gets the current settings of the classifier.
   getOptions()   - 
Method in class weka.classifiers.trees. RandomForest 
 Gets the current settings of the forest.
   getOptions()   - 
Method in class weka.classifiers.trees. RandomTree 
 Gets options from this classifier.
   getOptions()   - 
Method in class weka.classifiers.trees. REPTree 
 Gets options from this classifier.
   getOptions()   - 
Method in class weka.classifiers.trees. SimpleCart 
 Gets the current settings of the classifier.
   getOptions()   - 
Method in class weka.clusterers. CheckClusterer 
 Gets the current settings of the CheckClusterer.
   getOptions()   - 
Method in class weka.clusterers. CLOPE 
 Gets the current settings of CLOPE
   getOptions()   - 
Method in class weka.clusterers. Cobweb 
 Gets the current settings of Cobweb.
   getOptions()   - 
Method in class weka.clusterers. DBScan 
 Gets the current option settings for the OptionHandler.
   getOptions()   - 
Method in class weka.clusterers. EM 
 Gets the current settings of EM.
   getOptions()   - 
Method in class weka.clusterers. FarthestFirst 
 Gets the current settings of FarthestFirst
   getOptions()   - 
Method in class weka.clusterers. FilteredClusterer 
 Gets the current settings of the clusterer.
   getOptions()   - 
Method in class weka.clusterers. HierarchicalClusterer 
 Gets the current settings of the clusterer.
   getOptions()   - 
Method in class weka.clusterers. MakeDensityBasedClusterer 
 Gets the current settings of the clusterer.
   getOptions()   - 
Method in class weka.clusterers. OPTICS 
 Gets the current option settings for the OptionHandler.
   getOptions()   - 
Method in class weka.clusterers. RandomizableClusterer 
 Gets the current settings of the classifier.
   getOptions()   - 
Method in class weka.clusterers. RandomizableDensityBasedClusterer 
 Gets the current settings of the classifier.
   getOptions()   - 
Method in class weka.clusterers. RandomizableSingleClustererEnhancer 
 Gets the current settings of the classifier.
   getOptions()   - 
Method in class weka.clusterers. sIB 
 Gets the current settings.
   getOptions()   - 
Method in class weka.clusterers. SimpleKMeans 
 Gets the current settings of SimpleKMeans
   getOptions()   - 
Method in class weka.clusterers. SingleClustererEnhancer 
 Gets the current settings of the clusterer.
   getOptions()   - 
Method in class weka.clusterers. XMeans 
 Gets the current settings of SimpleKMeans.
   getOptions()   - 
Method in class weka.core. Check 
 Gets the current settings of the CheckClassifier.
   getOptions()   - 
Method in class weka.core. CheckGOE 
 Gets the current settings of the object.
   getOptions()   - 
Method in class weka.core. CheckOptionHandler 
 Gets the current settings of the CheckClassifier.
   getOptions()   - 
Method in class weka.core. CheckScheme 
 Gets the current settings of the CheckClassifier.
   getOptions()   - 
Method in class weka.core.converters. AbstractFileSaver 
 Gets the current settings of the Saver object.
   getOptions()   - 
Method in class weka.core.converters. ArffSaver 
 returns the options of the current setup
   getOptions()   - 
Method in class weka.core.converters. C45Saver 
 Gets the current settings of the C45Saver object.
   getOptions()   - 
Method in class weka.core.converters. CSVLoader 
 Gets the current settings of the Classifier.
   getOptions()   - 
Method in class weka.core.converters. DatabaseLoader 
 Gets the setting
   getOptions()   - 
Method in class weka.core.converters. DatabaseSaver 
 Gets the setting.
   getOptions()   - 
Method in class weka.core.converters. LibSVMSaver 
 returns the options of the current setup
   getOptions()   - 
Method in class weka.core.converters. SVMLightSaver 
 returns the options of the current setup.
   getOptions()   - 
Method in class weka.core.converters. TextDirectoryLoader 
 Gets the setting
   getOptions()   - 
Method in class weka.core.converters. XRFFSaver 
 returns the options of the current setup
   getOptions()   - 
Method in class weka.core. FindWithCapabilities 
 Gets the current settings of this object.
   getOptions()   - 
Method in class weka.core. Javadoc 
 Gets the current settings of this object.
   getOptions()   - 
Method in class weka.core. ListOptions 
 Gets the current settings of this object.
   getOptions()   - 
Method in class weka.core.neighboursearch. BallTree 
 Gets the current settings of KDtree.
   getOptions()   - 
Method in class weka.core.neighboursearch.balltrees. BallSplitter 
 Gets the current settings of the object.
   getOptions()   - 
Method in class weka.core.neighboursearch.balltrees. BallTreeConstructor 
 Gets the current settings.
   getOptions()   - 
Method in class weka.core.neighboursearch.balltrees. MedianDistanceFromArbitraryPoint 
 Gets the current settings of the object.
   getOptions()   - 
Method in class weka.core.neighboursearch.balltrees. MedianOfWidestDimension 
 Gets the current settings.
   getOptions()   - 
Method in class weka.core.neighboursearch.balltrees. MiddleOutConstructor 
 Gets the current settings of this BallTree MiddleOutConstructor.
   getOptions()   - 
Method in class weka.core.neighboursearch.balltrees. TopDownConstructor 
 Gets the current settings of KDtree.
   getOptions()   - 
Method in class weka.core.neighboursearch. CoverTree 
 Gets the current settings of KDtree.
   getOptions()   - 
Method in class weka.core.neighboursearch. KDTree 
 Gets the current settings of KDtree.
   getOptions()   - 
Method in class weka.core.neighboursearch.kdtrees. KDTreeNodeSplitter 
 Gets the current settings of the object.
   getOptions()   - 
Method in class weka.core.neighboursearch. LinearNNSearch 
 Gets the current settings.
   getOptions()   - 
Method in class weka.core.neighboursearch. NearestNeighbourSearch 
 Gets the current settings.
   getOptions()   - 
Method in class weka.core. NormalizableDistance 
 Gets the current settings.
   getOptions()   - 
Method in interface weka.core. OptionHandler 
 Gets the current option settings for the OptionHandler.
   getOptions()   - 
Method in class weka.core. OptionHandlerJavadoc 
 Gets the current settings of this object.
   getOptions()   - 
Method in class weka.core.stemmers. SnowballStemmer 
 Gets the current settings of the classifier.
   getOptions()   - 
Method in class weka.core. TechnicalInformationHandlerJavadoc 
 Gets the current settings of this object.
   getOptions()   - 
Method in class weka.core. TestInstances 
 Gets the current settings of this object.
   getOptions()   - 
Method in class weka.core.tokenizers. CharacterDelimitedTokenizer 
 Gets the current option settings for the OptionHandler.
   getOptions()   - 
Method in class weka.core.tokenizers. NGramTokenizer 
 Gets the current option settings for the OptionHandler.
   getOptions()   - 
Method in class weka.core.tokenizers. Tokenizer 
 Gets the current option settings for the OptionHandler.
   getOptions()   - 
Method in class weka.datagenerators. ClassificationGenerator 
 Gets the current settings of the classifier.
   getOptions()   - 
Method in class weka.datagenerators.classifiers.classification. Agrawal 
 Gets the current settings of the datagenerator.
   getOptions()   - 
Method in class weka.datagenerators.classifiers.classification. BayesNet 
 Gets the current settings of the datagenerator.
   getOptions()   - 
Method in class weka.datagenerators.classifiers.classification. LED24 
 Gets the current settings of the datagenerator.
   getOptions()   - 
Method in class weka.datagenerators.classifiers.classification. RandomRBF 
 Gets the current settings of the datagenerator.
   getOptions()   - 
Method in class weka.datagenerators.classifiers.classification. RDG1 
 Gets the current settings of the datagenerator RDG1.
   getOptions()   - 
Method in class weka.datagenerators.classifiers.regression. Expression 
 Gets the current settings of the datagenerator BIRCHCluster.
   getOptions()   - 
Method in class weka.datagenerators.classifiers.regression. MexicanHat 
 Gets the current settings of the datagenerator BIRCHCluster.
   getOptions()   - 
Method in class weka.datagenerators. ClusterDefinition 
 Gets the current settings of the datagenerator BIRCHCluster.
   getOptions()   - 
Method in class weka.datagenerators.clusterers. BIRCHCluster 
 Gets the current settings of the datagenerator BIRCHCluster.
   getOptions()   - 
Method in class weka.datagenerators.clusterers. SubspaceCluster 
 Gets the current settings of the datagenerator.
   getOptions()   - 
Method in class weka.datagenerators.clusterers. SubspaceClusterDefinition 
 Gets the current settings of the datagenerator BIRCHCluster.
   getOptions()   - 
Method in class weka.datagenerators. ClusterGenerator 
 Gets the current settings of the classifier.
   getOptions()   - 
Method in class weka.datagenerators. DataGenerator 
 Gets the current settings of the datagenerator RDG1.
   getOptions()   - 
Method in class weka.datagenerators. RegressionGenerator 
 Gets the current settings of the classifier.
   getOptions()   - 
Method in class weka.estimators. CheckEstimator 
 Gets the current settings of the CheckEstimator.
   getOptions()   - 
Method in class weka.estimators. Estimator 
 Gets the current settings of the Estimator.
   getOptions()   - 
Method in class weka.experiment. AveragingResultProducer 
 Gets the current settings of the result producer.
   getOptions()   - 
Method in class weka.experiment. ClassifierSplitEvaluator 
 Gets the current settings of the Classifier.
   getOptions()   - 
Method in class weka.experiment. CostSensitiveClassifierSplitEvaluator 
 Gets the current settings of the Classifier.
   getOptions()   - 
Method in class weka.experiment. CrossValidationResultProducer 
 Gets the current settings of the result producer.
   getOptions()   - 
Method in class weka.experiment. CSVResultListener 
 Gets the current settings of the Classifier.
   getOptions()   - 
Method in class weka.experiment. DatabaseResultProducer 
 Gets the current settings of the result producer.
   getOptions()   - 
Method in class weka.experiment. DensityBasedClustererSplitEvaluator 
 Gets the current settings of the Classifier.
   getOptions()   - 
Method in class weka.experiment. Experiment 
 Gets the current settings of the experiment iterator.
   getOptions()   - 
Method in class weka.experiment. InstanceQuery 
 Gets the current settings of InstanceQuery
   getOptions()   - 
Method in class weka.experiment. LearningRateResultProducer 
 Gets the current settings of the result producer.
   getOptions()   - 
Method in class weka.experiment. PairedTTester 
 Gets current settings of the PairedTTester.
   getOptions()   - 
Method in class weka.experiment. RandomSplitResultProducer 
 Gets the current settings of the result producer.
   getOptions()   - 
Method in class weka.experiment. RegressionSplitEvaluator 
 Gets the current settings of the Classifier.
   getOptions()   - 
Method in class weka.filters. CheckSource 
 Gets the current settings of the filter.
   getOptions()   - 
Method in class weka.filters. MultiFilter 
 Gets the current settings of the filter.
   getOptions()   - 
Method in class weka.filters. SimpleFilter 
 Gets the current settings of the filter.
   getOptions()   - 
Method in class weka.filters.supervised.attribute. AddClassification 
 Gets the current settings of the classifier.
   getOptions()   - 
Method in class weka.filters.supervised.attribute. AttributeSelection 
 Gets the current settings for the attribute selection (search, evaluator)
 etc.
   getOptions()   - 
Method in class weka.filters.supervised.attribute. ClassOrder 
 Gets the current settings of the filter.
   getOptions()   - 
Method in class weka.filters.supervised.attribute. Discretize 
 Gets the current settings of the filter.
   getOptions()   - 
Method in class weka.filters.supervised.attribute. NominalToBinary 
 Gets the current settings of the filter.
   getOptions()   - 
Method in class weka.filters.supervised.attribute. PLSFilter 
 returns the options of the current setup
   getOptions()   - 
Method in class weka.filters.supervised.instance. Resample 
 Gets the current settings of the filter.
   getOptions()   - 
Method in class weka.filters.supervised.instance. SMOTE 
 Gets the current settings of the filter.
   getOptions()   - 
Method in class weka.filters.supervised.instance. SpreadSubsample 
 Gets the current settings of the filter.
   getOptions()   - 
Method in class weka.filters.supervised.instance. StratifiedRemoveFolds 
 Gets the current settings of the filter.
   getOptions()   - 
Method in class weka.filters.unsupervised.attribute. AbstractTimeSeries 
 Gets the current settings of the filter.
   getOptions()   - 
Method in class weka.filters.unsupervised.attribute. Add 
 Gets the current settings of the filter.
   getOptions()   - 
Method in class weka.filters.unsupervised.attribute. AddCluster 
 Gets the current settings of the filter.
   getOptions()   - 
Method in class weka.filters.unsupervised.attribute. AddExpression 
 Gets the current settings of the filter.
   getOptions()   - 
Method in class weka.filters.unsupervised.attribute. AddID 
 Gets the current settings of the filter.
   getOptions()   - 
Method in class weka.filters.unsupervised.attribute. AddNoise 
 Gets the current settings of the filter.
   getOptions()   - 
Method in class weka.filters.unsupervised.attribute. AddValues 
 Gets the current settings of the filter.
   getOptions()   - 
Method in class weka.filters.unsupervised.attribute. ChangeDateFormat 
 Gets the current settings of the filter.
   getOptions()   - 
Method in class weka.filters.unsupervised.attribute. ClassAssigner 
 Gets the current settings of the filter.
   getOptions()   - 
Method in class weka.filters.unsupervised.attribute. ClusterMembership 
 Gets the current settings of the filter.
   getOptions()   - 
Method in class weka.filters.unsupervised.attribute. Copy 
 Gets the current settings of the filter.
   getOptions()   - 
Method in class weka.filters.unsupervised.attribute. Discretize 
 Gets the current settings of the filter.
   getOptions()   - 
Method in class weka.filters.unsupervised.attribute. FirstOrder 
 Gets the current settings of the filter.
   getOptions()   - 
Method in class weka.filters.unsupervised.attribute. InterquartileRange 
 Gets the current settings of the filter.
   getOptions()   - 
Method in class weka.filters.unsupervised.attribute. KernelFilter 
 Gets the current settings of the filter.
   getOptions()   - 
Method in class weka.filters.unsupervised.attribute. MakeIndicator 
 Gets the current settings of the filter.
   getOptions()   - 
Method in class weka.filters.unsupervised.attribute. MathExpression 
 Gets the current settings of the filter.
   getOptions()   - 
Method in class weka.filters.unsupervised.attribute. MergeTwoValues 
 Gets the current settings of the filter.
   getOptions()   - 
Method in class weka.filters.unsupervised.attribute. MultiInstanceToPropositional 
 Gets the current settings of the classifier.
   getOptions()   - 
Method in class weka.filters.unsupervised.attribute. NominalToBinary 
 Gets the current settings of the filter.
   getOptions()   - 
Method in class weka.filters.unsupervised.attribute. NominalToString 
 Gets the current settings of the filter.
   getOptions()   - 
Method in class weka.filters.unsupervised.attribute. Normalize 
 Gets the current settings of the filter.
   getOptions()   - 
Method in class weka.filters.unsupervised.attribute. NumericCleaner 
 Gets the current settings of the filter.
   getOptions()   - 
Method in class weka.filters.unsupervised.attribute. NumericToNominal 
 Gets the current settings of the filter.
   getOptions()   - 
Method in class weka.filters.unsupervised.attribute. NumericTransform 
 Gets the current settings of the filter.
   getOptions()   - 
Method in class weka.filters.unsupervised.attribute. PartitionedMultiFilter 
 Gets the current settings of the filter.
   getOptions()   - 
Method in class weka.filters.unsupervised.attribute. PKIDiscretize 
 Gets the current settings of the filter.
   getOptions()   - 
Method in class weka.filters.unsupervised.attribute. PotentialClassIgnorer 
 Gets the current settings of the filter.
   getOptions()   - 
Method in class weka.filters.unsupervised.attribute. PrincipalComponents 
 Gets the current settings of the filter.
   getOptions()   - 
Method in class weka.filters.unsupervised.attribute. PropositionalToMultiInstance 
 Gets the current settings of the classifier.
   getOptions()   - 
Method in class weka.filters.unsupervised.attribute. RandomProjection 
 Gets the current settings of the filter.
   getOptions()   - 
Method in class weka.filters.unsupervised.attribute. RandomSubset 
 Gets the current settings of the filter.
   getOptions()   - 
Method in class weka.filters.unsupervised.attribute. RELAGGS 
 Gets the current settings of the classifier.
   getOptions()   - 
Method in class weka.filters.unsupervised.attribute. Remove 
 Gets the current settings of the filter.
   getOptions()   - 
Method in class weka.filters.unsupervised.attribute. RemoveType 
 Gets the current settings of the filter.
   getOptions()   - 
Method in class weka.filters.unsupervised.attribute. RemoveUseless 
 Gets the current settings of the filter.
   getOptions()   - 
Method in class weka.filters.unsupervised.attribute. Reorder 
 Gets the current settings of the filter.
   getOptions()   - 
Method in class weka.filters.unsupervised.attribute. StringToNominal 
 Gets the current settings of the filter.
   getOptions()   - 
Method in class weka.filters.unsupervised.attribute. StringToWordVector 
 Gets the current settings of the filter.
   getOptions()   - 
Method in class weka.filters.unsupervised.attribute. SwapValues 
 Gets the current settings of the filter.
   getOptions()   - 
Method in class weka.filters.unsupervised.attribute. Wavelet 
 returns the options of the current setup
   getOptions()   - 
Method in class weka.filters.unsupervised.instance. Normalize 
 Gets the current settings of the filter.
   getOptions()   - 
Method in class weka.filters.unsupervised.instance. Randomize 
 Gets the current settings of the filter.
   getOptions()   - 
Method in class weka.filters.unsupervised.instance. RemoveFolds 
 Gets the current settings of the filter.
   getOptions()   - 
Method in class weka.filters.unsupervised.instance. RemoveFrequentValues 
 Gets the current settings of the filter.
   getOptions()   - 
Method in class weka.filters.unsupervised.instance. RemoveMisclassified 
 Gets the current settings of the filter.
   getOptions()   - 
Method in class weka.filters.unsupervised.instance. RemovePercentage 
 Gets the current settings of the filter.
   getOptions()   - 
Method in class weka.filters.unsupervised.instance. RemoveRange 
 Gets the current settings of the filter.
   getOptions()   - 
Method in class weka.filters.unsupervised.instance. RemoveWithValues 
 Gets the current settings of the filter.
   getOptions()   - 
Method in class weka.filters.unsupervised.instance. Resample 
 Gets the current settings of the filter.
   getOptions()   - 
Method in class weka.filters.unsupervised.instance. ReservoirSample 
 Gets the current settings of the filter.
   getOptions()   - 
Method in class weka.filters.unsupervised.instance. SubsetByExpression 
 Gets the current settings of the filter.
   getOptions()   - 
Method in class weka.gui. Main 
 returns the options of the current setup.
   getOptype()   - 
Method in class weka.core.pmml. Expression 
 Get the optype of the result of applying this Expression.
   getOptype()   - 
Method in class weka.core.pmml. FieldMetaInfo 
 Get the optype.
   getOrder()   - 
Method in enum weka.core.logging. Logger.Level 
 Returns the order of this level.
   getOrderedFlag()   - 
Method in class weka.datagenerators.clusterers. BIRCHCluster 
 Gets the ordered flag (option O).
   getOriginalCoords()   - 
Method in class weka.gui.beans. MetaBean 
 returns the vector containing the original coordinates (instances of class
 Point) for the inputs
   getOtherCapabilities()   - 
Method in class weka.core. Capabilities 
 returns all other capabilities, besides class and attribute related ones
   getOtherLeaf()   - 
Method in class weka.classifiers.trees.j48. GraftSplit 
  
   getOutlierFactor()   - 
Method in class weka.filters.unsupervised.attribute. InterquartileRange 
 Gets the factor for determining the thresholds for outliers.
   getOutlierTreatmentMethod()   - 
Method in class weka.core.pmml. MiningFieldMetaInfo 
 Get the outlier treatment method used for this field.
   getOutput()   - 
Method in class weka.datagenerators. DataGenerator 
 Gets the print writer.
   getOutput()   - 
Method in class weka.gui.explorer. DataGeneratorPanel 
 returns the generated output as text
   getOutputCenterFile()   - 
Method in class weka.clusterers. XMeans 
 Gets the file to write the list of centers to.
   getOutputClassification()   - 
Method in class weka.filters.supervised.attribute. AddClassification 
 Get whether the classifiction of the classifier is output.
   getOutputDef()   - 
Method in class weka.core.pmml. BuiltInArithmetic 
 Get the structure of the result produced by this function.
   getOutputDef()   - 
Method in class weka.core.pmml. BuiltInMath 
 Get the structure of the result produced by this function.
   getOutputDef()   - 
Method in class weka.core.pmml. BuiltInString 
 Get the structure of the result produced by this function.
   getOutputDef()   - 
Method in class weka.core.pmml. DefineFunction 
 Get the structure of the result produced by this function.
   getOutputDef()   - 
Method in class weka.core.pmml. FieldRef 
 Return the structure of the result of applying this Expression
 as an Attribute.
   getOutputDef()   - 
Method in class weka.core.pmml. Function 
 Get the structure of the result produced by this function.
   getOutputDistribution()   - 
Method in class weka.filters.supervised.attribute. AddClassification 
 Get whether the classifiction of the classifier is output.
   getOutputErrorFlag()   - 
Method in class weka.filters.supervised.attribute. AddClassification 
 Get whether the classifiction of the classifier is output.
   getOutputFile()   - 
Method in class weka.experiment. CrossValidationResultProducer 
 Get the value of OutputFile.
   getOutputFile()   - 
Method in class weka.experiment. CSVResultListener 
 Get the value of OutputFile.
   getOutputFile()   - 
Method in class weka.experiment. RandomSplitResultProducer 
 Get the value of OutputFile.
   getOutputFilename()   - 
Method in class weka.core.converters. TextDirectoryLoader 
 Gets whether the filename will be stored as an extra attribute.
   getOutputFilename()   - 
Method in class weka.gui. GenericPropertiesCreator 
 returns the name of the output file
   getOutputFormat()   - 
Method in class weka.core. Debug.Clock 
 returns the output format
   getOutputFormat()   - 
Method in class weka.filters. Filter 
 Gets the format of the output instances.
   getOutputFormat()   - 
Method in class weka.filters.unsupervised.attribute. PotentialClassIgnorer 
 Gets the format of the output instances.
   getOutputFormat()   - 
Static method in class weka.gui.experiment. ExperimenterDefaults 
 returns the classname of the ResultMatrix class, responsible for the
 output format
   getOutputItemSets()   - 
Method in class weka.associations. Apriori 
 Gets whether itemsets are output as well
   getOutputNums()   - 
Method in class weka.classifiers.functions.neural. NeuralConnection 
 Use this to get easy access to the output numbers.
   getOutputOffsetMultiplier()   - 
Method in class weka.filters.unsupervised.attribute. InterquartileRange 
 Gets whether an additional attribute "Offset" is generated per 
 Outlier/ExtremeValue attribute pair that lists the multiplier the value
 is off the median: value = median + 'multiplier' * IQR.
   getOutputPerClassInfoRetrievalStats()   - 
Method in class weka.gui.beans. IncrementalClassifierEvaluator 
 Get whether per-class information retrieval stats are to be output.
   getOutputProperties()   - 
Method in class weka.gui. GenericPropertiesCreator 
 returns the output properties object (structure like the template, but
 filled with classes instead of packages)
   getOutputs()   - 
Method in class weka.classifiers.functions.neural. NeuralConnection 
 Use this to get easy access to the outputs.
   getOutputs()   - 
Method in class weka.gui.beans. MetaBean 
  
   getOutputTypes()   - 
Method in class weka.core. Debug.DBO 
 Gets the current output type selection
   getOutputWordCounts()   - 
Method in class weka.filters.unsupervised.attribute. StringToWordVector 
 Gets whether output instances contain 0 or 1 indicating word
 presence, or word counts.
   getOverwriteWarning()   - 
Method in class weka.gui. ConverterFileChooser 
 Returns whether a popup appears with a warning that the file already 
 exists (only save dialog).
   getOwner()   - 
Method in class weka.core. Capabilities 
 returns the owner of this capabilities object
   getOwner()   - 
Static method in class weka.core. Copyright 
 returns the entity owning the copyright
   getP()   - 
Method in class weka.classifiers. BVDecomposeSegCVSub 
 Get the proportion of instances that are common between two training sets.
   getPackage(String)   - 
Method in class weka.gui. SimpleCLIPanel.CommandlineCompletion 
 returns the packages part of the partial classname.
   getPadding()   - 
Method in class weka.filters.unsupervised.attribute. Wavelet 
 Gets the type of Padding to use
   getPaint()   - 
Method in class weka.gui.visualize. PostscriptGraphics 
  
   getPanel(int)   - 
Method in class weka.gui.arffviewer. ArffViewerMainPanel 
 returns the specified panel,  null  if index is out of bounds
   getPanelCount()   - 
Method in class weka.gui.arffviewer. ArffViewerMainPanel 
 returns the number of panels currently open
   getPanels()   - 
Method in class weka.gui.explorer. Explorer 
 returns all the panels, apart from the PreprocessPanel
   getParameterNames()   - 
Method in class weka.core.pmml. BuiltInArithmetic 
 Returns an array of the names of the parameters expected
 as input by this function
   getParameterNames()   - 
Method in class weka.core.pmml. BuiltInMath 
 Returns an array of the names of the parameters expected
 as input by this function.
   getParameterNames()   - 
Method in class weka.core.pmml. BuiltInString 
 Returns an array of the names of the parameters expected
 as input by this function.
   getParameterNames()   - 
Method in class weka.core.pmml. DefineFunction 
 Returns an array of the names of the parameters expected
 as input by this function.
   getParameterNames()   - 
Method in class weka.core.pmml. Function 
 Returns an array of the names of the parameters expected
 as input by this function.
   getParent(int, int)   - 
Method in class weka.classifiers.bayes. BayesNet 
 get node index of a parent of a node in the network structure
   getParent(int)   - 
Method in class weka.classifiers.bayes.net. ParentSet 
 returns index parent of parent specified by index
   getParent()   - 
Method in class weka.datagenerators. ClusterDefinition 
 returns the parent datagenerator this cluster belongs to
   getParent(int)   - 
Method in class weka.gui.treevisualizer. Node 
 Get the parent edge.
   getParentCardinality(int)   - 
Method in class weka.classifiers.bayes. BayesNet 
 get number of values the collection of parents of a node can take
   getParentDialog(Container)   - 
Static method in class weka.gui. PropertyDialog 
 Tries to determine the dialog this panel is part of.
   getParentFrame()   - 
Method in class weka.gui.arffviewer. ArffViewerMainPanel 
 returns the parent frame, if it's a JFrame, otherwise null
   getParentFrame()   - 
Method in class weka.gui. GUIChooser.ChildFrameSDI 
 returns the parent frame, can be null.
   getParentFrame()   - 
Method in class weka.gui. Main.ChildFrameMDI 
 returns the parent frame, can be null.
   getParentFrame()   - 
Method in class weka.gui. Main.ChildFrameSDI 
 returns the parent frame, can be null.
   getParentFrame(Container)   - 
Static method in class weka.gui. PropertyDialog 
 Tries to determine the frame this panel is part of.
   getParentFrame()   - 
Method in class weka.gui. SetInstancesPanel 
 Returns the current frame the panel knows of, that it resides in.
   getParentInternalFrame()   - 
Method in class weka.gui.arffviewer. ArffViewerMainPanel 
 returns the parent frame, if it's a JInternalFrame, otherwise null
   getParents()   - 
Method in class weka.classifiers.bayes.net. ParentSet 
  
   getParentSet(int)   - 
Method in class weka.classifiers.bayes. BayesNet 
 get the parent set of a node
   getParentSets()   - 
Method in class weka.classifiers.bayes. BayesNet 
 Get full set of parent sets.
   getParts()   - 
Method in class weka.associations.tertius. IndividualInstance 
  
   getPassword()   - 
Method in class weka.core.converters. DatabaseLoader 
 Returns the database password
   getPassword()   - 
Method in class weka.core.converters. DatabaseSaver 
 Returns the database password.
   getPassword()   - 
Method in class weka.experiment. DatabaseUtils 
 Get the database password.
   getPassword()   - 
Method in class weka.gui. DatabaseConnectionDialog 
 Returns password from dialog
   getPassword()   - 
Method in class weka.gui.sql. ConnectionPanel 
 returns the current Password.
   getPassword()   - 
Method in class weka.gui.sql.event. ResultChangedEvent 
 returns the password that produced the table model
   getPassword()   - 
Method in class weka.gui.sql. ResultSetTable 
 returns the password that produced the table model
   getPassword()   - 
Method in class weka.gui.sql. SqlViewer 
 returns the password from the currently active tab in the ResultPanel,
 otherwise an empty string.
   getPassword()   - 
Method in class weka.gui.sql. SqlViewerDialog 
 returns the chosen password, if any
   getPath()   - 
Method in class weka.gui. PropertySelectorDialog 
 Gets the path of property nodes to the selected property.
   getPattern()   - 
Method in class weka.datagenerators.clusterers. BIRCHCluster 
 Gets the pattern type.
   getPenalty()   - 
Method in class weka.classifiers.bayes.blr. Prior 
  
   getPercent()   - 
Method in class weka.filters.unsupervised.attribute. AddNoise 
 Gets the size of noise data as a percentage of the original set.
   getPercent()   - 
Method in class weka.filters.unsupervised.attribute. RandomProjection 
 Gets the percent the attributes (dimensions) of the data will be reduced to
   getPercentage()   - 
Method in class weka.filters.supervised.instance. SMOTE 
 Gets the percentage of SMOTE instances to create.
   getPercentage()   - 
Method in class weka.filters.unsupervised.instance. RemovePercentage 
 Gets the percentage of instances to select.
   getPercentCompleted()   - 
Method in class weka.gui.boundaryvisualizer. RemoteResult 
 Return the progress for this row
   getPercentThreshold()   - 
Method in class weka.attributeSelection. SVMAttributeEval 
 Get the threshold below which percentage elimination reverts to 
 constant elimination.
   getPercentToEliminatePerIteration()   - 
Method in class weka.attributeSelection. SVMAttributeEval 
 Get the percentage rate of attribute elimination per iteration
   getPerformanceStats()   - 
Method in class weka.core.neighboursearch. NearestNeighbourSearch 
 Gets the class object that contains the performance statistics of
 the search method.
   getPerformPrediction()   - 
Method in class weka.filters.supervised.attribute. PLSFilter 
 Gets whether the class attribute is updated with the predicted value.
   getPerformRanking()   - 
Method in class weka.attributeSelection. LinearForwardSelection 
 Get boolean if initial ranking should be performed to select the
 top-ranked attributes
   getPerformRanking()   - 
Method in class weka.attributeSelection. SubsetSizeForwardSelection 
 Get boolean if initial ranking should be performed to select the
 top-ranked attributes
   getPeriodicPruning()   - 
Method in class weka.filters.unsupervised.attribute. StringToWordVector 
 Gets the rate at which the dictionary is periodically pruned, as a 
 percentage of the dataset size.
   getPerturbationFraction()   - 
Method in class weka.datagenerators.classifiers.classification. Agrawal 
 Gets the perturbation fraction.
   getPivot()   - 
Method in class weka.core.matrix. LUDecomposition 
 Return pivot permutation vector
   getPivot()   - 
Method in class weka.core.neighboursearch.balltrees. BallNode 
 Returns the pivot/centre of the
 node's ball.
   getPlainColumnName(int)   - 
Method in class weka.gui.arffviewer. ArffTable 
 returns the basically the attribute name of the column and not the
 HTML column name via getColumnName(int)
   getPlotInstances()   - 
Method in class weka.gui.visualize. PlotData2D 
 Returns the instances for this plot
   getPlotName()   - 
Method in class weka.gui.visualize. PlotData2D 
 Get the name of this plot
   getPlotNameHTML()   - 
Method in class weka.gui.visualize. PlotData2D 
 Get the name of the plot for use in a tool tip text.
   getPlotPanel()   - 
Method in class weka.gui.visualize. VisualizePanel 
 Returns the underlying plot panel.
   getPlots()   - 
Method in class weka.gui.visualize. Plot2D 
 Return the list of plots
   getPlotTrainingData()   - 
Method in class weka.gui.boundaryvisualizer. BoundaryPanel 
 Returns true if training data is to be superimposed
   getPMMLModel(String)   - 
Static method in class weka.core.pmml. PMMLFactory 
 Read and return a PMML model.
   getPMMLModel(File)   - 
Static method in class weka.core.pmml. PMMLFactory 
 Read and return a PMML model.
   getPMMLModel(InputStream)   - 
Static method in class weka.core.pmml. PMMLFactory 
 Read and return a PMML model.
   getPMMLModel(String, Logger)   - 
Static method in class weka.core.pmml. PMMLFactory 
 Read and return a PMML model.
   getPMMLModel(File, Logger)   - 
Static method in class weka.core.pmml. PMMLFactory 
 Read and return a PMML model.
   getPMMLModel(InputStream, Logger)   - 
Static method in class weka.core.pmml. PMMLFactory 
 Read and return a PMML model.
   getPMMLVersion()   - 
Method in class weka.classifiers.pmml.consumer. PMMLClassifier 
 Get the PMML version used for this model.
   getPMMLVersion()   - 
Method in interface weka.core.pmml. PMMLModel 
 Get the version of PMML used to encode this model.
   getPointValue(int)   - 
Method in class weka.classifiers.functions.pace. DiscreteFunction 
 Gets a particular point value
   getPopulationSize()   - 
Method in class weka.attributeSelection. GeneticSearch 
 get the size of the population
   getPopulationSize()   - 
Method in class weka.attributeSelection. ScatterSearchV1 
 Get the population size
   getPopulationSize()   - 
Method in class weka.classifiers.bayes.net.search.global. GeneticSearch 
  
   getPopulationSize()   - 
Method in class weka.classifiers.bayes.net.search.local. GeneticSearch 
  
   getPopup()   - 
Method in class weka.gui. GenericObjectEditor.CapabilitiesFilterDialog 
 returns the currently set JPopupMenu.
   getPositionX(int)   - 
Method in class weka.classifiers.bayes.net. EditableBayesNet 
 get x position of a node
   getPositionY(int)   - 
Method in class weka.classifiers.bayes.net. EditableBayesNet 
 get y position of a node
   getPositiveIndex()   - 
Method in class weka.associations. FPGrowth 
 Get the index of the attribute value to consider as positive
 for binary attributes in normal dense instances.
   getPostFixExpression()   - 
Method in class weka.core. AttributeExpression 
 Return the postfix expression
   getPostProcessor()   - 
Method in class weka.core. CheckScheme 
 returns the current PostProcessor, can be null
   getPostProcessor()   - 
Method in class weka.estimators. CheckEstimator 
 returns the current PostProcessor, can be null
   getPrecision()   - 
Method in class weka.classifiers.evaluation. TwoClassStats 
 Calculate the precision.
   getPrecision()   - 
Method in class weka.estimators. KernelEstimator 
 Return the precision of this kernel estimator.
   getPrecision()   - 
Method in class weka.estimators. NormalEstimator 
 Return the value of the precision of this normal estimator.
   getPredicate()   - 
Method in class weka.associations.tertius. Literal 
  
   getPrediction(Classifier, Instance)   - 
Method in class weka.classifiers.evaluation. EvaluationUtils 
 Generate a single prediction for a test instance given the pre-trained
 classifier.
   getPredTargetColumn()   - 
Method in class weka.experiment. ClassifierSplitEvaluator 
  
   getPreferredScrollableViewportSize()   - 
Method in class weka.gui. AttributeSelectionPanel 
  
   getPrefix()   - 
Method in class weka.gui.beans. SerializedModelSaver 
 Get the prefix to prepend to the model file names.
   getPremise()   - 
Method in class weka.associations. FPGrowth.AssociationRule 
 Get the premise of this rule.
   getPremiseSupport()   - 
Method in class weka.associations. FPGrowth.AssociationRule 
 Get the support for the premise.
   getPreprocessing()   - 
Method in class weka.filters.supervised.attribute. PLSFilter 
 Gets the type of preprocessing to use
   getPreprocessing()   - 
Method in class weka.filters.unsupervised.attribute. KernelFilter 
 Gets the filter used for preprocessing
   getPreprocessPanel()   - 
Method in class weka.gui.explorer. Explorer 
 returns the instance of the PreprocessPanel being used in this instance
 of the Explorer
   getPreserveInstancesOrder()   - 
Method in class weka.clusterers. SimpleKMeans 
 Gets whether order of instances must be preserved
   getPrintColNames()   - 
Method in class weka.experiment. ResultMatrix 
 returns whether column names or numbers instead are printed
   getPrintNewick()   - 
Method in class weka.clusterers. HierarchicalClusterer 
  
   getPrintRowNames()   - 
Method in class weka.experiment. ResultMatrix 
 returns whether row names or numbers instead are printed
   getPriorClass()   - 
Method in class weka.classifiers.bayes. BayesianLogisticRegression 
 Get the type of prior to use.
   getPriority(int)   - 
Method in class weka.clusterers.forOPTICSAndDBScan.Utils. PriorityQueue 
 Returns the priority for the object at the specified index
   getPriority()   - 
Method in class weka.clusterers.forOPTICSAndDBScan.Utils. PriorityQueueElement 
 Returns the priority for this object
   getPriority(int)   - 
Method in class weka.clusterers.forOPTICSAndDBScan.Utils. UpdateQueue 
 Returns the priority for the object at the specified index
   getPriority()   - 
Method in class weka.clusterers.forOPTICSAndDBScan.Utils. UpdateQueueElement 
 Returns the priority for this object
   getPriorProbability(String)   - 
Method in class weka.core.pmml. TargetMetaInfo 
 Get the prior probability for the supplied value.
   getProbabilities()   - 
Method in class weka.gui.boundaryvisualizer. RemoteResult 
 Return the probability distributions  for this row in the visualization
   getProbability(int, int, int)   - 
Method in class weka.classifiers.bayes. BayesNet 
 get particular probability of the conditional probability distribtion
 of a node given its parents.
   getProbability(double)   - 
Method in class weka.classifiers.bayes.net.estimate. DiscreteEstimatorBayes 
 Get a probability estimate for a value
   getProbability(double, double)   - 
Method in interface weka.estimators. ConditionalEstimator 
 Get a probability for a value conditional on another value
   getProbability(double, double)   - 
Method in class weka.estimators. DDConditionalEstimator 
 Get a probability estimate for a value
   getProbability(double)   - 
Method in class weka.estimators. DiscreteEstimator 
 Get a probability estimate for a value
   getProbability(double, double)   - 
Method in class weka.estimators. DKConditionalEstimator 
 Get a probability estimate for a value
   getProbability(double, double)   - 
Method in class weka.estimators. DNConditionalEstimator 
 Get a probability estimate for a value
   getProbability(double)   - 
Method in class weka.estimators. Estimator 
 Get a probability estimate for a value.
   getProbability(double, double)   - 
Method in class weka.estimators. KDConditionalEstimator 
 Get a probability estimate for a value
   getProbability(double)   - 
Method in class weka.estimators. KernelEstimator 
 Get a probability estimate for a value.
   getProbability(double, double)   - 
Method in class weka.estimators. KKConditionalEstimator 
 Get a probability estimate for a value
   getProbability(double)   - 
Method in class weka.estimators. MahalanobisEstimator 
 Get a probability estimate for a value
   getProbability(double, double)   - 
Method in class weka.estimators. NDConditionalEstimator 
 Get a probability estimate for a value
   getProbability(double, double)   - 
Method in class weka.estimators. NNConditionalEstimator 
 Get a probability estimate for a value
   getProbability(double)   - 
Method in class weka.estimators. NormalEstimator 
 Get a probability estimate for a value
   getProbability(double)   - 
Method in class weka.estimators. PoissonEstimator 
 Get a probability estimate for a value
   getProbabilityEstimates()   - 
Method in class weka.classifiers.functions. LibLINEAR 
 Sets whether to generate probability estimates instead of -1/+1 for
 classification problems.
   getProbabilityEstimates()   - 
Method in class weka.classifiers.functions. LibSVM 
 Sets whether to generate probability estimates instead of -1/+1 for 
 classification problems.
   getProgressBar()   - 
Method in class weka.gui.graphvisualizer. HierarchicalBCEngine 
 Returns a handle to the progressBar
 of this LayoutEngine.
   getProgressBar()   - 
Method in interface weka.gui.graphvisualizer. LayoutEngine 
 This method returns the progress bar
 for the LayoutEngine, which shows
 the progress of the layout process,
 if it takes a while to layout the
 graph
   getProjectionFilter()   - 
Method in class weka.classifiers.meta. RotationForest 
 Gets the filter used to project the data.
   getProlog()   - 
Method in class weka.core. OptionHandlerJavadoc 
 whether "Valid options are..." prolog is included in the Javadoc
   getProlog()   - 
Method in class weka.core. TechnicalInformationHandlerJavadoc 
 whether "Valid options are..." prolog is included in the Javadoc
   getProperties()   - 
Static method in class weka.gui.experiment. ExperimenterDefaults 
 returns the associated properties file
   getProperties()   - 
Static method in class weka.gui.explorer. ExplorerDefaults 
 returns the associated properties file.
   getProperty()   - 
Method in class weka.core.pmml. FieldMetaInfo.Value 
  
   getPropertyArray()   - 
Method in class weka.experiment. Experiment 
 Gets the array of values to set the custom property to.
   getPropertyArrayLength()   - 
Method in class weka.experiment. Experiment 
 Gets the number of custom iterator values that have been defined
 for the experiment.
   getPropertyArrayValue(int)   - 
Method in class weka.experiment. Experiment 
 Gets a specified value from the custom property iterator array.
   getPropertyDescriptor(Object, PropertyPath.Path)   - 
Static method in class weka.core. PropertyPath 
 returns the property associated with the given path, null if a problem
 occurred.
   getPropertyDescriptor(Object, String)   - 
Static method in class weka.core. PropertyPath 
 returns the property associated with the given path
   getPropertyDescriptors()   - 
Method in class weka.gui.beans. ClassAssignerBeanInfo 
 Returns the property descriptors
   getPropertyDescriptors()   - 
Method in class weka.gui.beans. ClassValuePickerBeanInfo 
 Returns the property descriptors
   getPropertyDescriptors()   - 
Method in class weka.gui.beans. CrossValidationFoldMakerBeanInfo 
 Return the property descriptors for this bean
   getPropertyDescriptors()   - 
Method in class weka.gui.beans. IncrementalClassifierEvaluatorBeanInfo 
 Return the property descriptors for this bean
   getPropertyDescriptors()   - 
Method in class weka.gui.beans. PredictionAppenderBeanInfo 
 Return the property descriptors for this bean
   getPropertyDescriptors()   - 
Method in class weka.gui.beans. StripChartBeanInfo 
 Get the property descriptors for this bean
   getPropertyDescriptors()   - 
Method in class weka.gui.beans. TrainTestSplitMakerBeanInfo 
 Get the property descriptors for this bean
   getPropertyPath()   - 
Method in class weka.experiment. Experiment 
 Gets the path of properties taken to get to the custom property
 to iterate over.
   getPruningMethod()   - 
Method in class weka.classifiers.functions.supportVector. StringKernel 
 Gets the method used for pruning.
   getPruningStrategy()   - 
Method in class weka.classifiers.trees. BFTree 
 Gets the pruning strategy.
   getPruningType()   - 
Method in class weka.classifiers.meta. RacedIncrementalLogitBoost 
 Get the pruning type
   getQ()   - 
Method in class weka.core.matrix. QRDecomposition 
 Generate and return the (economy-sized) orthogonal factor
   getQuality()   - 
Method in class weka.gui.visualize. JPEGWriter 
 returns the quality the JPEG will be stored in.
   getQuery()   - 
Method in class weka.core.converters. DatabaseLoader 
 Gets the query to execute against the database
   getQuery()   - 
Method in class weka.experiment. InstanceQuery 
 Get the query to execute against the database
   getQuery()   - 
Method in class weka.gui.sql.event. QueryExecuteEvent 
 returns the query that was executed
   getQuery()   - 
Method in class weka.gui.sql.event. ResultChangedEvent 
 returns the query that was executed
   getQuery()   - 
Method in class weka.gui.sql. QueryPanel 
 returns the currently displayed query.
   getQuery()   - 
Method in class weka.gui.sql. ResultSetTable 
 returns the query that produced the table model
   getQuery()   - 
Method in class weka.gui.sql. SqlViewer 
 returns the query from the currently active tab in the ResultPanel,
 otherwise an empty string.
   getQuery()   - 
Method in class weka.gui.sql. SqlViewerDialog 
 returns the chosen query, if any
   getQueryPanel()   - 
Method in class weka.gui.sql. ResultPanel 
 returns the currently set QueryPanel, can be NULL
   getR()   - 
Method in class weka.core.matrix. QRDecomposition 
 Return the upper triangular factor
   getRaceType()   - 
Method in class weka.attributeSelection. RaceSearch 
 Get the race type
   getRadius()   - 
Method in class weka.core.neighboursearch.balltrees. BallNode 
 Returns the radius of the node's ball.
   getRandom()   - 
Method in class weka.datagenerators. DataGenerator 
 Gets the random generator.
   getRandomize()   - 
Method in class weka.filters.unsupervised.attribute. PropositionalToMultiInstance 
 Gets whether the order of the generated is randomized
   getRandomizeData()   - 
Method in class weka.experiment. RandomSplitResultProducer 
 Get if dataset is to be randomized
   getRandomNumberGenerator(long)   - 
Method in class weka.core. Instances 
 Returns a random number generator.
   getRandomOrder()   - 
Method in class weka.classifiers.bayes.net.search.global. K2 
 Get random order flag
   getRandomOrder()   - 
Method in class weka.classifiers.bayes.net.search.local. K2 
 Get random order flag
   getRandomSeed()   - 
Method in class weka.classifiers.functions. LeastMedSq 
 get the seed for the random number generator
   getRandomSeed()   - 
Method in class weka.classifiers.functions. SMO 
 Get the value of randomSeed.
   getRandomSeed()   - 
Method in class weka.classifiers.mi. MISMO 
 Get the value of randomSeed.
   getRandomSeed()   - 
Method in class weka.classifiers.trees. ADTree 
 Gets random seed for a random walk.
   getRandomSeed()   - 
Method in class weka.core.neighboursearch.balltrees. MedianDistanceFromArbitraryPoint 
 Returns the seed value of random 
 number generator.
   getRandomSeed()   - 
Method in class weka.filters.supervised.instance. Resample 
 Gets the random number seed.
   getRandomSeed()   - 
Method in class weka.filters.supervised.instance. SMOTE 
 Gets the random number seed.
   getRandomSeed()   - 
Method in class weka.filters.supervised.instance. SpreadSubsample 
 Gets the random number seed.
   getRandomSeed()   - 
Method in class weka.filters.unsupervised.attribute. AddNoise 
 Gets the random number seed.
   getRandomSeed()   - 
Method in class weka.filters.unsupervised.attribute. RandomProjection 
 Gets the random seed of the random number generator
   getRandomSeed()   - 
Method in class weka.filters.unsupervised.instance. Randomize 
 Get the random number generator seed value.
   getRandomSeed()   - 
Method in class weka.filters.unsupervised.instance. Resample 
 Gets the random number seed.
   getRandomSeed()   - 
Method in class weka.filters.unsupervised.instance. ReservoirSample 
 Gets the random number seed.
   getRandomWidthFactor()   - 
Method in class weka.classifiers.meta. MultiClassClassifier 
 Gets the multiplier when generating random codes.
   getRange(int)   - 
Method in class weka.filters.unsupervised.attribute. PartitionedMultiFilter 
 Gets a single Range from the set of available Ranges.
   getRangeCorrection()   - 
Method in class weka.classifiers.meta. ThresholdSelector 
 Gets the confidence range correction mode used.
   getRanges()   - 
Method in class weka.core. NormalizableDistance 
 Method to get the ranges.
   getRanges()   - 
Method in class weka.core. Range 
 Gets the string representing the selected range of values
   getRanges()   - 
Method in class weka.filters.unsupervised.attribute. PartitionedMultiFilter 
 Gets the list of possible Ranges to choose from.
   getRank()   - 
Method in class weka.attributeSelection. LatentSemanticAnalysis 
 Gets the desired matrix rank (or coverage proportion) for feature-space reduction
   getRawOutput()   - 
Method in class weka.experiment. CrossValidationResultProducer 
 Get if raw split evaluator output is to be saved
   getRawOutput()   - 
Method in class weka.experiment. RandomSplitResultProducer 
 Get if raw split evaluator output is to be saved
   getRawResultOutput()   - 
Method in class weka.experiment. ClassifierSplitEvaluator 
 Gets the raw output from the classifier
   getRawResultOutput()   - 
Method in class weka.experiment. DensityBasedClustererSplitEvaluator 
 Gets the raw output from the classifier
   getRawResultOutput()   - 
Method in class weka.experiment. RegressionSplitEvaluator 
 Gets the raw output from the classifier
   getRawResultOutput()   - 
Method in interface weka.experiment. SplitEvaluator 
 Returns the raw output for the most recent call to getResult.
   getReachabilityDistance()   - 
Method in interface weka.clusterers.forOPTICSAndDBScan.DataObjects. DataObject 
 Returns the reachabilityDistance for this dataObject
   getReachabilityDistance()   - 
Method in class weka.clusterers.forOPTICSAndDBScan.DataObjects. EuclidianDataObject 
 Returns the reachabilityDistance for this dataObject
   getReachabilityDistance()   - 
Method in class weka.clusterers.forOPTICSAndDBScan.DataObjects. ManhattanDataObject 
 Returns the reachabilityDistance for this dataObject
   getReadable()   - 
Method in class weka.core. Tag 
 Gets the string description of the Tag.
   getReader(String, String)   - 
Static method in class weka.gui. Loader 
 returns a Reader for the given filename and dir, can be NULL if it fails
   getReader(String)   - 
Method in class weka.gui. Loader 
 returns a Reader for the given filename, can be NULL if it fails
   getReadIncrementally()   - 
Method in class weka.gui. SetInstancesPanel 
 Gets whether instances are to be read incrementally or not
   getRealEigenvalues()   - 
Method in class weka.core.matrix. EigenvalueDecomposition 
 Return the real parts of the eigenvalues
   getRecall()   - 
Method in class weka.classifiers.evaluation. TwoClassStats 
 Calculate the recall.
   getReducedErrorPruning()   - 
Method in class weka.classifiers.rules. PART 
 Get the value of reducedErrorPruning.
   getReducedErrorPruning()   - 
Method in class weka.classifiers.trees. J48 
 Get the value of reducedErrorPruning.
   getRefer()   - 
Method in class weka.gui.treevisualizer. Node 
 Get the value of refer.
   getRefreshFreq()   - 
Method in class weka.gui.beans. StripChart 
 Get the refresh frequency
   getRegOptimizer()   - 
Method in class weka.classifiers.functions. SMOreg 
 returns the learning algorithm
   getRegressionTree()   - 
Method in class weka.classifiers.trees.m5. Rule 
 Get the value of regressionTree.
   getRegressionTree()   - 
Method in class weka.classifiers.trees.m5. RuleNode 
 Get the value of regressionTree.
   getRelabel()   - 
Method in class weka.classifiers.trees. J48graft 
 Get the value of relabelling
   getRelation()   - 
Method in class weka.core. TestInstances 
 returns the current name of the relation
   getRelationalClassFormat()   - 
Method in class weka.core. TestInstances 
 returns the current strcuture of the relational class attribute, can
 be null
   getRelationalFormat(int)   - 
Method in class weka.core. TestInstances 
 returns the format for the specified relational attribute, can be null
   getRelationForTableName()   - 
Method in class weka.core.converters. DatabaseSaver 
 Gets whether or not the relation name is used as name of the table.
   getRelationName()   - 
Method in class weka.datagenerators. DataGenerator 
 Gets the relation name the dataset should have.
   getRelationNameForFilename()   - 
Method in class weka.gui.beans. Saver 
 Get whether the relation name is the primary part of the filename.
   getRemoteHosts()   - 
Method in class weka.experiment. RemoteExperiment 
 Get the list of remote host names
   getRemoveAllMissingCols()   - 
Method in class weka.associations. Apriori 
 Returns whether columns containing all missing values are to be removed
   getRemoveClassColumn()   - 
Method in class weka.experiment. DensityBasedClustererSplitEvaluator 
 Get whether the class column is to be removed.
   getRemovedPercentage()   - 
Method in class weka.classifiers.meta. RotationForest 
 Gets the percentage of instances to be removed
   getRemoveFilterClassnames()   - 
Static method in class weka.gui.experiment. ExperimenterDefaults 
 whether the filter classnames in the dataset names are removed by default
   getRemoveFilterName()   - 
Method in class weka.experiment. ResultMatrix 
 returns whether the filter classname is removed from the dataset name
   getRemoveFilterName()   - 
Method in class weka.gui.experiment. OutputFormatDialog 
 returns whether the filter classname is removed from the dataset name.
   getRemoveOldClass()   - 
Method in class weka.filters.supervised.attribute. AddClassification 
 Get whether the old class attribute is removed.
   getRemoveUnused()   - 
Method in class weka.filters.unsupervised.attribute. PartitionedMultiFilter 
 Gets whether unused attributes (ones that are not covered by any of the
 ranges) are removed from the output.
   getRenderingHint(RenderingHints.Key)   - 
Method in class weka.gui.visualize. PostscriptGraphics 
  
   getRenderingHints()   - 
Method in class weka.gui.visualize. PostscriptGraphics 
  
   getRepeatLiterals()   - 
Method in class weka.associations. Tertius 
 Get the value of repeatLiterals.
   getRepetitions()   - 
Static method in class weka.gui.experiment. ExperimenterDefaults 
 returns the number of repetitions to use
   getReplaceMissing()   - 
Method in class weka.filters.supervised.attribute. PLSFilter 
 Gets whether missing values are replace.
   getReplaceMissingValues()   - 
Method in class weka.filters.unsupervised.attribute. RandomProjection 
 Gets the current setting for using ReplaceMissingValues filter
   getReportFrequency()   - 
Method in class weka.attributeSelection. GeneticSearch 
 get how often repports are generated
   getRepulsion()   - 
Method in class weka.clusterers. CLOPE 
 gets the repulsion
   getReset()   - 
Method in class weka.classifiers.functions. MultilayerPerceptron 
  
   getReset()   - 
Method in class weka.gui.beans. ChartEvent 
 get the value of the reset flag
   getResult()   - 
Method in class weka.core.mathematicalexpression. Parser 
 Returns the result of the evaluation.
   getResult(double[])   - 
Method in class weka.core.pmml. BuiltInArithmetic 
 Get the result of applying this function.
   getResult(double[])   - 
Method in class weka.core.pmml. BuiltInMath 
 Get the result of applying this function.
   getResult(double[])   - 
Method in class weka.core.pmml. BuiltInString 
 Get the result of applying this function.
   getResult(double[])   - 
Method in class weka.core.pmml. Constant 
 Get the result of evaluating the expression.
   getResult(double[])   - 
Method in class weka.core.pmml. DefineFunction 
 Get the result of applying this function.
   getResult(double[])   - 
Method in class weka.core.pmml. Discretize 
 Get the result of evaluating the expression.
   getResult(double[])   - 
Method in class weka.core.pmml. Expression 
 Get the result of evaluating the expression.
   getResult(double[])   - 
Method in class weka.core.pmml. FieldRef 
  
   getResult(double[])   - 
Method in class weka.core.pmml. Function 
 Get the result of applying this function.
   getResult(double[])   - 
Method in class weka.core.pmml. NormContinuous 
 Get the result of evaluating the expression.
   getResult(double[])   - 
Method in class weka.core.pmml. NormDiscrete 
 Get the result of evaluating the expression.
   getResult(Instances, Instances)   - 
Method in class weka.experiment. ClassifierSplitEvaluator 
 Gets the results for the supplied train and test datasets.
   getResult(Instances, Instances)   - 
Method in class weka.experiment. CostSensitiveClassifierSplitEvaluator 
 Gets the results for the supplied train and test datasets.
   getResult(Instances, Instances)   - 
Method in class weka.experiment. DensityBasedClustererSplitEvaluator 
 Gets the results for the supplied train and test datasets.
   getResult(Instances, Instances)   - 
Method in class weka.experiment. RegressionSplitEvaluator 
 Gets the results for the supplied train and test datasets.
   getResult(Instances, Instances)   - 
Method in interface weka.experiment. SplitEvaluator 
 Gets the results for the supplied train and test datasets.
   getResult()   - 
Method in class weka.filters.unsupervised.instance.subsetbyexpression. Parser 
 Returns the result of the evaluation.
   getResult()   - 
Method in class weka.gui.experiment. OutputFormatDialog 
 the result from the last display of the dialog, the same is returned
 from  showDialog .
   getResultCategorical(double[])   - 
Method in class weka.core.pmml. Constant 
 Gets the result of evaluating the expression when the
 optype is categorical or ordinal as the actual String
 value.
   getResultCategorical(double[])   - 
Method in class weka.core.pmml. Discretize 
 Gets the result of evaluating the expression when the
 optype is categorical or ordinal as the actual String
 value.
   getResultCategorical(double[])   - 
Method in class weka.core.pmml. Expression 
 Gets the result of evaluating the expression when the
 optype is categorical or ordinal as the actual String
 value.
   getResultCategorical(double[])   - 
Method in class weka.core.pmml. FieldRef 
  
   getResultCategorical(double[])   - 
Method in class weka.core.pmml. NormContinuous 
 Always throws an Exception since the result of NormContinuous must
 be continuous.
   getResultCategorical(double[])   - 
Method in class weka.core.pmml. NormDiscrete 
 Always throws an Exception since the result of NormDiscrete must
 be continuous.
   getResultContinuous(double[])   - 
Method in class weka.core.pmml. Expression 
 Get the result of evaluating the expression for continuous
 optype.
   getResultFromTable(String, ResultProducer, Object[])   - 
Method in class weka.experiment. DatabaseUtils 
 Executes a database query to extract a result for the supplied key
 from the database.
   getResultInverse(double[])   - 
Method in class weka.core.pmml. NormContinuous 
 Compute the inverse of the normalization (i.e.
   getResultListener()   - 
Method in class weka.experiment. Experiment 
 Gets the result listener where results will be sent.
   getResultMatrix()   - 
Method in class weka.experiment. PairedTTester 
 Gets the instance that produces the output.
   getResultMatrix()   - 
Method in interface weka.experiment. Tester 
 Gets the instance that produces the output.
   getResultMatrix()   - 
Method in class weka.gui.experiment. OutputFormatDialog 
 Gets the currently selected output format result matrix.
   getResultNames()   - 
Method in class weka.experiment. AveragingResultProducer 
 Gets the names of each of the columns produced for a single run.
   getResultNames()   - 
Method in class weka.experiment. ClassifierSplitEvaluator 
 Gets the names of each of the result columns produced for a single run.
   getResultNames()   - 
Method in class weka.experiment. CostSensitiveClassifierSplitEvaluator 
 Gets the names of each of the result columns produced for a single run.
   getResultNames()   - 
Method in class weka.experiment. CrossValidationResultProducer 
 Gets the names of each of the columns produced for a single run.
   getResultNames()   - 
Method in class weka.experiment. DatabaseResultProducer 
 Gets the names of each of the columns produced for a single run.
   getResultNames()   - 
Method in class weka.experiment. DensityBasedClustererSplitEvaluator 
 Gets the names of each of the result columns produced for a single run.
   getResultNames()   - 
Method in class weka.experiment. LearningRateResultProducer 
 Gets the names of each of the columns produced for a single run.
   getResultNames()   - 
Method in class weka.experiment. RandomSplitResultProducer 
 Gets the names of each of the columns produced for a single run.
   getResultNames()   - 
Method in class weka.experiment. RegressionSplitEvaluator 
 Gets the names of each of the result columns produced for a single run.
   getResultNames()   - 
Method in interface weka.experiment. ResultProducer 
 Gets the names of each of the result columns produced for a single run.
   getResultNames()   - 
Method in interface weka.experiment. SplitEvaluator 
 Gets the names of each of the result columns produced for a single run.
   getResultProducer()   - 
Method in class weka.experiment. AveragingResultProducer 
 Get the ResultProducer.
   getResultProducer()   - 
Method in class weka.experiment. DatabaseResultProducer 
 Get the ResultProducer.
   getResultProducer()   - 
Method in class weka.experiment. Experiment 
 Get the result producer used for the current experiment.
   getResultProducer()   - 
Method in class weka.experiment. LearningRateResultProducer 
 Get the ResultProducer.
   getResults()   - 
Method in class weka.associations. Tertius 
 returns the results
   getResultSet()   - 
Method in class weka.experiment. DatabaseUtils 
 Gets the results generated by a previous query.
   getResultSet()   - 
Method in class weka.gui.sql.event. QueryExecuteEvent 
 returns the resultset that was produced, can be null in case the query
 failed
   getResultSet()   - 
Method in class weka.gui.sql. ResultSetHelper 
 the underlying resultset.
   getResultsetKeyColumns()   - 
Method in class weka.experiment. PairedTTester 
 Get the value of ResultsetKeyColumns.
   getResultsetKeyColumns()   - 
Method in interface weka.experiment. Tester 
 Get the value of ResultsetKeyColumns.
   getResultsetName(int)   - 
Method in class weka.experiment. PairedTTester 
 Gets a string descriptive of the specified resultset.
   getResultsetName(int)   - 
Method in interface weka.experiment. Tester 
 Gets a string descriptive of the specified resultset.
   getResultsTableName(ResultProducer)   - 
Method in class weka.experiment. DatabaseUtils 
 Gets the name of the experiment table that stores results from a
 particular ResultProducer.
   getResultTypes()   - 
Method in class weka.experiment. AveragingResultProducer 
 Gets the data types of each of the columns produced for a single run.
   getResultTypes()   - 
Method in class weka.experiment. ClassifierSplitEvaluator 
 Gets the data types of each of the result columns produced for a 
 single run.
   getResultTypes()   - 
Method in class weka.experiment. CostSensitiveClassifierSplitEvaluator 
 Gets the data types of each of the result columns produced for a 
 single run.
   getResultTypes()   - 
Method in class weka.experiment. CrossValidationResultProducer 
 Gets the data types of each of the columns produced for a single run.
   getResultTypes()   - 
Method in class weka.experiment. DatabaseResultProducer 
 Gets the data types of each of the columns produced for a single run.
   getResultTypes()   - 
Method in class weka.experiment. DensityBasedClustererSplitEvaluator 
 Gets the data types of each of the result columns produced for a 
 single run.
   getResultTypes()   - 
Method in class weka.experiment. LearningRateResultProducer 
 Gets the data types of each of the columns produced for a single run.
   getResultTypes()   - 
Method in class weka.experiment. RandomSplitResultProducer 
 Gets the data types of each of the columns produced for a single run.
   getResultTypes()   - 
Method in class weka.experiment. RegressionSplitEvaluator 
 Gets the data types of each of the result columns produced for a 
 single run.
   getResultTypes()   - 
Method in interface weka.experiment. ResultProducer 
 Gets the data types of each of the result columns produced for a 
 single run.
   getResultTypes()   - 
Method in interface weka.experiment. SplitEvaluator 
 Gets the data types of each of the result columns produced for a 
 single run.
   getResultVector()   - 
Method in class weka.clusterers.forOPTICSAndDBScan.OPTICS_GUI. SERObject 
 Returns the resultVector
   getResultVector()   - 
Method in class weka.clusterers. OPTICS 
 Returns the resultVector
   getReturnValue()   - 
Method in class weka.gui. DatabaseConnectionDialog 
 Returns which of OK or cancel was clicked from dialog
   getReturnValue()   - 
Method in class weka.gui.sql. SqlViewerDialog 
 returns whether the user clicked OK (JOptionPane.OK_OPTION) or whether he
 cancelled the dialog (JOptionPane.CANCEL_OPTION)
   getRevision()   - 
Method in class weka.associations. AbstractAssociator 
 Returns the revision string.
   getRevision()   - 
Method in class weka.associations. Apriori 
 Returns the revision string.
   getRevision()   - 
Method in class weka.associations. AprioriItemSet 
 Returns the revision string.
   getRevision()   - 
Method in class weka.associations. AssociatorEvaluation 
 Returns the revision string.
   getRevision()   - 
Method in class weka.associations. CaRuleGeneration 
 Returns the revision string.
   getRevision()   - 
Method in class weka.associations. CheckAssociator 
 Returns the revision string.
   getRevision()   - 
Method in class weka.associations. FilteredAssociator 
 Returns the revision string.
   getRevision()   - 
Method in class weka.associations. FPGrowth 
 Returns the revision string.
   getRevision()   - 
Method in class weka.associations. GeneralizedSequentialPatterns 
 Returns the revision string.
   getRevision()   - 
Method in class weka.associations.gsp. Element 
 Returns the revision string.
   getRevision()   - 
Method in class weka.associations.gsp. Sequence 
 Returns the revision string.
   getRevision()   - 
Method in class weka.associations. ItemSet 
 Returns the revision string.
   getRevision()   - 
Method in class weka.associations. LabeledItemSet 
 Returns the revision string.
   getRevision()   - 
Method in class weka.associations. PredictiveApriori 
 Returns the revision string.
   getRevision()   - 
Method in class weka.associations. PriorEstimation 
 Returns the revision string.
   getRevision()   - 
Method in class weka.associations. RuleGeneration 
 Returns the revision string.
   getRevision()   - 
Method in class weka.associations. RuleItem 
 Returns the revision string.
   getRevision()   - 
Method in class weka.associations.tertius. AttributeValueLiteral 
 Returns the revision string.
   getRevision()   - 
Method in class weka.associations.tertius. Body 
 Returns the revision string.
   getRevision()   - 
Method in class weka.associations. Tertius 
 Returns the revision string.
   getRevision()   - 
Method in class weka.associations.tertius. Head 
 Returns the revision string.
   getRevision()   - 
Method in class weka.associations.tertius. IndividualInstance 
 Returns the revision string.
   getRevision()   - 
Method in class weka.associations.tertius. IndividualInstances 
 Returns the revision string.
   getRevision()   - 
Method in class weka.associations.tertius. IndividualLiteral 
 Returns the revision string.
   getRevision()   - 
Method in class weka.associations.tertius. Predicate 
 Returns the revision string.
   getRevision()   - 
Method in class weka.associations.tertius. Rule 
 Returns the revision string.
   getRevision()   - 
Method in class weka.associations.tertius. SimpleLinkedList 
 Returns the revision string.
   getRevision()   - 
Method in class weka.associations.tertius. SimpleLinkedList.LinkedListInverseIterator 
 Returns the revision string.
   getRevision()   - 
Method in class weka.associations.tertius. SimpleLinkedList.LinkedListIterator 
 Returns the revision string.
   getRevision()   - 
Method in class weka.attributeSelection. ASEvaluation 
 Returns the revision string.
   getRevision()   - 
Method in class weka.attributeSelection. ASSearch 
 Returns the revision string.
   getRevision()   - 
Method in class weka.attributeSelection. AttributeSelection 
 Returns the revision string.
   getRevision()   - 
Method in class weka.attributeSelection. BestFirst 
 Returns the revision string.
   getRevision()   - 
Method in class weka.attributeSelection. BestFirst.Link2 
 Returns the revision string.
   getRevision()   - 
Method in class weka.attributeSelection. BestFirst.LinkedList2 
 Returns the revision string.
   getRevision()   - 
Method in class weka.attributeSelection. CfsSubsetEval 
 Returns the revision string.
   getRevision()   - 
Method in class weka.attributeSelection. CheckAttributeSelection 
 Returns the revision string.
   getRevision()   - 
Method in class weka.attributeSelection. ChiSquaredAttributeEval 
 Returns the revision string.
   getRevision()   - 
Method in class weka.attributeSelection. ClassifierSubsetEval 
 Returns the revision string.
   getRevision()   - 
Method in class weka.attributeSelection. ConsistencySubsetEval 
 Returns the revision string.
   getRevision()   - 
Method in class weka.attributeSelection. ConsistencySubsetEval.hashKey 
 Returns the revision string.
   getRevision()   - 
Method in class weka.attributeSelection. CostSensitiveASEvaluation 
 Returns the revision string.
   getRevision()   - 
Method in class weka.attributeSelection. CostSensitiveAttributeEval 
 Returns the revision string.
   getRevision()   - 
Method in class weka.attributeSelection. CostSensitiveSubsetEval 
 Returns the revision string.
   getRevision()   - 
Method in class weka.attributeSelection. ExhaustiveSearch 
 Returns the revision string.
   getRevision()   - 
Method in class weka.attributeSelection. FilteredAttributeEval 
 Returns the revision string.
   getRevision()   - 
Method in class weka.attributeSelection. FilteredSubsetEval 
 Returns the revision string.
   getRevision()   - 
Method in class weka.attributeSelection. GainRatioAttributeEval 
 Returns the revision string.
   getRevision()   - 
Method in class weka.attributeSelection. GeneticSearch 
 Returns the revision string.
   getRevision()   - 
Method in class weka.attributeSelection. GreedyStepwise 
 Returns the revision string.
   getRevision()   - 
Method in class weka.attributeSelection. InfoGainAttributeEval 
 Returns the revision string.
   getRevision()   - 
Method in class weka.attributeSelection. LatentSemanticAnalysis 
 Returns the revision string.
   getRevision()   - 
Method in class weka.attributeSelection. LFSMethods 
 Returns the revision string.
   getRevision()   - 
Method in class weka.attributeSelection. LFSMethods.Link2 
 Returns the revision string.
   getRevision()   - 
Method in class weka.attributeSelection. LFSMethods.LinkedList2 
 Returns the revision string.
   getRevision()   - 
Method in class weka.attributeSelection. LinearForwardSelection 
 Returns the revision string.
   getRevision()   - 
Method in class weka.attributeSelection. OneRAttributeEval 
 Returns the revision string.
   getRevision()   - 
Method in class weka.attributeSelection. PrincipalComponents 
 Returns the revision string.
   getRevision()   - 
Method in class weka.attributeSelection. RaceSearch 
 Returns the revision string.
   getRevision()   - 
Method in class weka.attributeSelection. RandomSearch 
 Returns the revision string.
   getRevision()   - 
Method in class weka.attributeSelection. Ranker 
 Returns the revision string.
   getRevision()   - 
Method in class weka.attributeSelection. RankSearch 
 Returns the revision string.
   getRevision()   - 
Method in class weka.attributeSelection. ReliefFAttributeEval 
 Returns the revision string.
   getRevision()   - 
Method in class weka.attributeSelection. ScatterSearchV1 
 Returns the revision string.
   getRevision()   - 
Method in class weka.attributeSelection. SubsetSizeForwardSelection 
 Returns the revision string.
   getRevision()   - 
Method in class weka.attributeSelection. SVMAttributeEval 
 Returns the revision string.
   getRevision()   - 
Method in class weka.attributeSelection. SymmetricalUncertAttributeEval 
 Returns the revision string.
   getRevision()   - 
Method in class weka.attributeSelection. WrapperSubsetEval 
 Returns the revision string.
   getRevision()   - 
Method in class weka.classifiers.bayes. AODE 
 Returns the revision string.
   getRevision()   - 
Method in class weka.classifiers.bayes. AODEsr 
 Returns the revision string.
   getRevision()   - 
Method in class weka.classifiers.bayes. BayesianLogisticRegression 
 Returns the revision string.
   getRevision()   - 
Method in class weka.classifiers.bayes. BayesNet 
 Returns the revision string.
   getRevision()   - 
Method in class weka.classifiers.bayes.blr. GaussianPriorImpl 
 Returns the revision string.
   getRevision()   - 
Method in class weka.classifiers.bayes.blr. LaplacePriorImpl 
 Returns the revision string.
   getRevision()   - 
Method in class weka.classifiers.bayes. ComplementNaiveBayes 
 Returns the revision string.
   getRevision()   - 
Method in class weka.classifiers.bayes. DMNBtext 
 Returns the revision string.
   getRevision()   - 
Method in class weka.classifiers.bayes. HNB 
 Returns the revision string.
   getRevision()   - 
Method in class weka.classifiers.bayes. NaiveBayes 
 Returns the revision string.
   getRevision()   - 
Method in class weka.classifiers.bayes. NaiveBayesMultinomial 
 Returns the revision string.
   getRevision()   - 
Method in class weka.classifiers.bayes. NaiveBayesMultinomialUpdateable 
 Returns the revision string.
   getRevision()   - 
Method in class weka.classifiers.bayes. NaiveBayesSimple 
 Returns the revision string.
   getRevision()   - 
Method in class weka.classifiers.bayes. NaiveBayesUpdateable 
 Returns the revision string.
   getRevision()   - 
Method in class weka.classifiers.bayes.net. ADNode 
 Returns the revision string.
   getRevision()   - 
Method in class weka.classifiers.bayes.net. BayesNetGenerator 
 Returns the revision string.
   getRevision()   - 
Method in class weka.classifiers.bayes.net. BIFReader 
 Returns the revision string.
   getRevision()   - 
Method in class weka.classifiers.bayes.net. EditableBayesNet 
 Returns the revision string.
   getRevision()   - 
Method in class weka.classifiers.bayes.net.estimate. BayesNetEstimator 
 Returns the revision string.
   getRevision()   - 
Method in class weka.classifiers.bayes.net.estimate. BMAEstimator 
 Returns the revision string.
   getRevision()   - 
Method in class weka.classifiers.bayes.net.estimate. DiscreteEstimatorBayes 
 Returns the revision string.
   getRevision()   - 
Method in class weka.classifiers.bayes.net.estimate. DiscreteEstimatorFullBayes 
 Returns the revision string.
   getRevision()   - 
Method in class weka.classifiers.bayes.net.estimate. MultiNomialBMAEstimator 
 Returns the revision string.
   getRevision()   - 
Method in class weka.classifiers.bayes.net.estimate. SimpleEstimator 
 Returns the revision string.
   getRevision()   - 
Method in class weka.classifiers.bayes.net. MarginCalculator 
 Returns the revision string.
   getRevision()   - 
Method in class weka.classifiers.bayes.net. MarginCalculator.JunctionTreeNode 
 Returns the revision string.
   getRevision()   - 
Method in class weka.classifiers.bayes.net. MarginCalculator.JunctionTreeSeparator 
 Returns the revision string.
   getRevision()   - 
Method in class weka.classifiers.bayes.net. ParentSet 
 Returns the revision string.
   getRevision()   - 
Method in class weka.classifiers.bayes.net.search.ci. CISearchAlgorithm 
 Returns the revision string.
   getRevision()   - 
Method in class weka.classifiers.bayes.net.search.ci. ICSSearchAlgorithm 
 Returns the revision string.
   getRevision()   - 
Method in class weka.classifiers.bayes.net.search.fixed. FromFile 
 Returns the revision string.
   getRevision()   - 
Method in class weka.classifiers.bayes.net.search.fixed. NaiveBayes 
 Returns the revision string.
   getRevision()   - 
Method in class weka.classifiers.bayes.net.search.global. GeneticSearch 
 Returns the revision string.
   getRevision()   - 
Method in class weka.classifiers.bayes.net.search.global. GlobalScoreSearchAlgorithm 
 Returns the revision string.
   getRevision()   - 
Method in class weka.classifiers.bayes.net.search.global. HillClimber 
 Returns the revision string.
   getRevision()   - 
Method in class weka.classifiers.bayes.net.search.global. K2 
 Returns the revision string.
   getRevision()   - 
Method in class weka.classifiers.bayes.net.search.global. RepeatedHillClimber 
 Returns the revision string.
   getRevision()   - 
Method in class weka.classifiers.bayes.net.search.global. SimulatedAnnealing 
 Returns the revision string.
   getRevision()   - 
Method in class weka.classifiers.bayes.net.search.global. TabuSearch 
 Returns the revision string.
   getRevision()   - 
Method in class weka.classifiers.bayes.net.search.global. TAN 
 Returns the revision string.
   getRevision()   - 
Method in class weka.classifiers.bayes.net.search.local. GeneticSearch 
 Returns the revision string.
   getRevision()   - 
Method in class weka.classifiers.bayes.net.search.local. HillClimber 
 Returns the revision string.
   getRevision()   - 
Method in class weka.classifiers.bayes.net.search.local. K2 
 Returns the revision string.
   getRevision()   - 
Method in class weka.classifiers.bayes.net.search.local. LAGDHillClimber 
 Returns the revision string.
   getRevision()   - 
Method in class weka.classifiers.bayes.net.search.local. LocalScoreSearchAlgorithm 
 Returns the revision string.
   getRevision()   - 
Method in class weka.classifiers.bayes.net.search.local. RepeatedHillClimber 
 Returns the revision string.
   getRevision()   - 
Method in class weka.classifiers.bayes.net.search.local. SimulatedAnnealing 
 Returns the revision string.
   getRevision()   - 
Method in class weka.classifiers.bayes.net.search.local. TabuSearch 
 Returns the revision string.
   getRevision()   - 
Method in class weka.classifiers.bayes.net.search.local. TAN 
 Returns the revision string.
   getRevision()   - 
Method in class weka.classifiers.bayes.net.search. SearchAlgorithm 
 Returns the revision string.
   getRevision()   - 
Method in class weka.classifiers.bayes.net. VaryNode 
 Returns the revision string.
   getRevision()   - 
Method in class weka.classifiers.bayes. WAODE 
 Returns the revision string.
   getRevision()   - 
Method in class weka.classifiers. BVDecompose 
 Returns the revision string.
   getRevision()   - 
Method in class weka.classifiers. BVDecomposeSegCVSub 
 Returns the revision string.
   getRevision()   - 
Method in class weka.classifiers. CheckClassifier 
 Returns the revision string.
   getRevision()   - 
Method in class weka.classifiers. CheckSource 
 Returns the revision string.
   getRevision()   - 
Method in class weka.classifiers. Classifier 
 Returns the revision string.
   getRevision()   - 
Method in class weka.classifiers. CostMatrix 
 Returns the revision string.
   getRevision()   - 
Method in class weka.classifiers.evaluation. ConfusionMatrix 
 Returns the revision string.
   getRevision()   - 
Method in class weka.classifiers.evaluation. CostCurve 
 Returns the revision string.
   getRevision()   - 
Method in class weka.classifiers.evaluation. EvaluationUtils 
 Returns the revision string.
   getRevision()   - 
Method in class weka.classifiers. Evaluation 
 Returns the revision string.
   getRevision()   - 
Method in class weka.classifiers.evaluation. MarginCurve 
 Returns the revision string.
   getRevision()   - 
Method in class weka.classifiers.evaluation. NominalPrediction 
 Returns the revision string.
   getRevision()   - 
Method in class weka.classifiers.evaluation. NumericPrediction 
 Returns the revision string.
   getRevision()   - 
Method in class weka.classifiers.evaluation. ThresholdCurve 
 Returns the revision string.
   getRevision()   - 
Method in class weka.classifiers.evaluation. TwoClassStats 
 Returns the revision string.
   getRevision()   - 
Method in class weka.classifiers.functions. GaussianProcesses 
 Returns the revision string.
   getRevision()   - 
Method in class weka.classifiers.functions. IsotonicRegression 
 Returns the revision string.
   getRevision()   - 
Method in class weka.classifiers.functions. LeastMedSq 
 Returns the revision string.
   getRevision()   - 
Method in class weka.classifiers.functions. LibLINEAR 
 Returns the revision string.
   getRevision()   - 
Method in class weka.classifiers.functions. LibSVM 
 Returns the revision string.
   getRevision()   - 
Method in class weka.classifiers.functions. LinearRegression 
 Returns the revision string.
   getRevision()   - 
Method in class weka.classifiers.functions. Logistic 
 Returns the revision string.
   getRevision()   - 
Method in class weka.classifiers.functions. MultilayerPerceptron 
 Returns the revision string.
   getRevision()   - 
Method in class weka.classifiers.functions.neural. LinearUnit 
 Returns the revision string.
   getRevision()   - 
Method in class weka.classifiers.functions.neural. NeuralNode 
 Returns the revision string.
   getRevision()   - 
Method in class weka.classifiers.functions.neural. SigmoidUnit 
 Returns the revision string.
   getRevision()   - 
Method in class weka.classifiers.functions.pace. ChisqMixture 
 Returns the revision string.
   getRevision()   - 
Method in class weka.classifiers.functions.pace. DiscreteFunction 
 Returns the revision string.
   getRevision()   - 
Method in class weka.classifiers.functions.pace. NormalMixture 
 Returns the revision string.
   getRevision()   - 
Method in class weka.classifiers.functions.pace. PaceMatrix 
 Returns the revision string.
   getRevision()   - 
Method in class weka.classifiers.functions. PaceRegression 
 Returns the revision string.
   getRevision()   - 
Method in class weka.classifiers.functions. PLSClassifier 
 Returns the revision string.
   getRevision()   - 
Method in class weka.classifiers.functions. RBFNetwork 
 Returns the revision string.
   getRevision()   - 
Method in class weka.classifiers.functions. SimpleLinearRegression 
 Returns the revision string.
   getRevision()   - 
Method in class weka.classifiers.functions. SimpleLogistic 
 Returns the revision string.
   getRevision()   - 
Method in class weka.classifiers.functions. SMO.BinarySMO 
 Returns the revision string.
   getRevision()   - 
Method in class weka.classifiers.functions. SMO 
 Returns the revision string.
   getRevision()   - 
Method in class weka.classifiers.functions. SMOreg 
 Returns the revision string.
   getRevision()   - 
Method in class weka.classifiers.functions. SPegasos 
 Returns the revision string.
   getRevision()   - 
Method in class weka.classifiers.functions.supportVector. CheckKernel 
 Returns the revision string.
   getRevision()   - 
Method in class weka.classifiers.functions.supportVector. Kernel 
 Returns the revision string.
   getRevision()   - 
Method in class weka.classifiers.functions.supportVector. KernelEvaluation 
 Returns the revision string.
   getRevision()   - 
Method in class weka.classifiers.functions.supportVector. NormalizedPolyKernel 
 Returns the revision string.
   getRevision()   - 
Method in class weka.classifiers.functions.supportVector. PolyKernel 
 Returns the revision string.
   getRevision()   - 
Method in class weka.classifiers.functions.supportVector. PrecomputedKernelMatrixKernel 
 Returns the revision string.
   getRevision()   - 
Method in class weka.classifiers.functions.supportVector. Puk 
 Returns the revision string.
   getRevision()   - 
Method in class weka.classifiers.functions.supportVector. RBFKernel 
 Returns the revision string.
   getRevision()   - 
Method in class weka.classifiers.functions.supportVector. RegOptimizer 
 Returns the revision string.
   getRevision()   - 
Method in class weka.classifiers.functions.supportVector. RegSMO 
 Returns the revision string.
   getRevision()   - 
Method in class weka.classifiers.functions.supportVector. RegSMOImproved 
 Returns the revision string.
   getRevision()   - 
Method in class weka.classifiers.functions.supportVector. SMOset 
 Returns the revision string.
   getRevision()   - 
Method in class weka.classifiers.functions.supportVector. StringKernel 
 Returns the revision string.
   getRevision()   - 
Method in class weka.classifiers.functions. VotedPerceptron 
 Returns the revision string.
   getRevision()   - 
Method in class weka.classifiers.functions. Winnow 
 Returns the revision string.
   getRevision()   - 
Method in class weka.classifiers.lazy. IB1 
 Returns the revision string.
   getRevision()   - 
Method in class weka.classifiers.lazy. IBk 
 Returns the revision string.
   getRevision()   - 
Method in class weka.classifiers.lazy. KStar 
 Returns the revision string.
   getRevision()   - 
Method in class weka.classifiers.lazy.kstar. KStarCache.CacheTable 
 Returns the revision string.
   getRevision()   - 
Method in class weka.classifiers.lazy.kstar. KStarCache 
 Returns the revision string.
   getRevision()   - 
Method in class weka.classifiers.lazy.kstar. KStarCache.TableEntry 
 Returns the revision string.
   getRevision()   - 
Method in class weka.classifiers.lazy.kstar. KStarNominalAttribute 
 Returns the revision string.
   getRevision()   - 
Method in class weka.classifiers.lazy.kstar. KStarNumericAttribute 
 Returns the revision string.
   getRevision()   - 
Method in class weka.classifiers.lazy.kstar. KStarWrapper 
 Returns the revision string.
   getRevision()   - 
Method in class weka.classifiers.lazy. LBR 
 Returns the revision string.
   getRevision()   - 
Method in class weka.classifiers.lazy. LBR.Indexes 
 Returns the revision string.
   getRevision()   - 
Method in class weka.classifiers.lazy. LWL 
 Returns the revision string.
   getRevision()   - 
Method in class weka.classifiers.meta. AdaBoostM1 
 Returns the revision string.
   getRevision()   - 
Method in class weka.classifiers.meta. AdditiveRegression 
 Returns the revision string.
   getRevision()   - 
Method in class weka.classifiers.meta. AttributeSelectedClassifier 
 Returns the revision string.
   getRevision()   - 
Method in class weka.classifiers.meta. Bagging 
 Returns the revision string.
   getRevision()   - 
Method in class weka.classifiers.meta. ClassificationViaClustering 
 Returns the revision string.
   getRevision()   - 
Method in class weka.classifiers.meta. ClassificationViaRegression 
 Returns the revision string.
   getRevision()   - 
Method in class weka.classifiers.meta. CostSensitiveClassifier 
 Returns the revision string.
   getRevision()   - 
Method in class weka.classifiers.meta. CVParameterSelection 
 Returns the revision string.
   getRevision()   - 
Method in class weka.classifiers.meta. Dagging 
 Returns the revision string.
   getRevision()   - 
Method in class weka.classifiers.meta. Decorate 
 Returns the revision string.
   getRevision()   - 
Method in class weka.classifiers.meta. END 
 Returns the revision string.
   getRevision()   - 
Method in class weka.classifiers.meta. FilteredClassifier 
 Returns the revision string.
   getRevision()   - 
Method in class weka.classifiers.meta. Grading 
 Returns the revision string.
   getRevision()   - 
Method in class weka.classifiers.meta. GridSearch 
 Returns the revision string.
   getRevision()   - 
Method in class weka.classifiers.meta. LogitBoost 
 Returns the revision string.
   getRevision()   - 
Method in class weka.classifiers.meta. MetaCost 
 Returns the revision string.
   getRevision()   - 
Method in class weka.classifiers.meta. MultiBoostAB 
 Returns the revision string.
   getRevision()   - 
Method in class weka.classifiers.meta. MultiClassClassifier 
 Returns the revision string.
   getRevision()   - 
Method in class weka.classifiers.meta. MultiScheme 
 Returns the revision string.
   getRevision()   - 
Method in class weka.classifiers.meta.nestedDichotomies. ClassBalancedND 
 Returns the revision string.
   getRevision()   - 
Method in class weka.classifiers.meta.nestedDichotomies. DataNearBalancedND 
 Returns the revision string.
   getRevision()   - 
Method in class weka.classifiers.meta.nestedDichotomies. ND 
 Returns the revision string.
   getRevision()   - 
Method in class weka.classifiers.meta. OrdinalClassClassifier 
 Returns the revision string.
   getRevision()   - 
Method in class weka.classifiers.meta. RacedIncrementalLogitBoost 
 Returns the revision string.
   getRevision()   - 
Method in class weka.classifiers.meta. RandomCommittee 
 Returns the revision string.
   getRevision()   - 
Method in class weka.classifiers.meta. RandomSubSpace 
 Returns the revision string.
   getRevision()   - 
Method in class weka.classifiers.meta. RegressionByDiscretization 
 Returns the revision string.
   getRevision()   - 
Method in class weka.classifiers.meta. RotationForest 
 Returns the revision string.
   getRevision()   - 
Method in class weka.classifiers.meta. Stacking 
 Returns the revision string.
   getRevision()   - 
Method in class weka.classifiers.meta. StackingC 
 Returns the revision string.
   getRevision()   - 
Method in class weka.classifiers.meta. ThresholdSelector 
 Returns the revision string.
   getRevision()   - 
Method in class weka.classifiers.meta. Vote 
 Returns the revision string.
   getRevision()   - 
Method in class weka.classifiers.mi. CitationKNN 
 Returns the revision string.
   getRevision()   - 
Method in class weka.classifiers.mi. MDD 
 Returns the revision string.
   getRevision()   - 
Method in class weka.classifiers.mi. MIBoost 
 Returns the revision string.
   getRevision()   - 
Method in class weka.classifiers.mi. MIDD 
 Returns the revision string.
   getRevision()   - 
Method in class weka.classifiers.mi. MIEMDD 
 Returns the revision string.
   getRevision()   - 
Method in class weka.classifiers.mi. MILR 
 Returns the revision string.
   getRevision()   - 
Method in class weka.classifiers.mi. MINND 
 Returns the revision string.
   getRevision()   - 
Method in class weka.classifiers.mi. MIOptimalBall 
 Returns the revision string.
   getRevision()   - 
Method in class weka.classifiers.mi. MISMO 
 Returns the revision string.
   getRevision()   - 
Method in class weka.classifiers.mi. MISVM 
 Returns the revision string.
   getRevision()   - 
Method in class weka.classifiers.mi. MIWrapper 
 Returns the revision string.
   getRevision()   - 
Method in class weka.classifiers.mi. SimpleMI 
 Returns the revision string.
   getRevision()   - 
Method in class weka.classifiers.mi.supportVector. MIPolyKernel 
 Returns the revision string.
   getRevision()   - 
Method in class weka.classifiers.mi.supportVector. MIRBFKernel 
 Returns the revision string.
   getRevision()   - 
Method in class weka.classifiers.misc. HyperPipes 
 Returns the revision string.
   getRevision()   - 
Method in class weka.classifiers.misc. SerializedClassifier 
 Returns the revision string.
   getRevision()   - 
Method in class weka.classifiers.misc. VFI 
 Returns the revision string.
   getRevision()   - 
Method in class weka.classifiers.pmml.consumer. GeneralRegression 
  
   getRevision()   - 
Method in class weka.classifiers.pmml.consumer. NeuralNetwork 
  
   getRevision()   - 
Method in class weka.classifiers.pmml.consumer. Regression 
  
   getRevision()   - 
Method in class weka.classifiers.rules. ConjunctiveRule 
 Returns the revision string.
   getRevision()   - 
Method in class weka.classifiers.rules. DecisionTable 
 Returns the revision string.
   getRevision()   - 
Method in class weka.classifiers.rules. DecisionTableHashKey 
 Returns the revision string.
   getRevision()   - 
Method in class weka.classifiers.rules. DTNB 
 Returns the revision string.
   getRevision()   - 
Method in class weka.classifiers.rules. JRip 
 Returns the revision string.
   getRevision()   - 
Method in class weka.classifiers.rules. M5Rules 
 Returns the revision string.
   getRevision()   - 
Method in class weka.classifiers.rules. NNge 
 Returns the revision string.
   getRevision()   - 
Method in class weka.classifiers.rules. OneR 
 Returns the revision string.
   getRevision()   - 
Method in class weka.classifiers.rules.part. C45PruneableDecList 
 Returns the revision string.
   getRevision()   - 
Method in class weka.classifiers.rules.part. ClassifierDecList 
 Returns the revision string.
   getRevision()   - 
Method in class weka.classifiers.rules. PART 
 Returns the revision string.
   getRevision()   - 
Method in class weka.classifiers.rules.part. MakeDecList 
 Returns the revision string.
   getRevision()   - 
Method in class weka.classifiers.rules.part. PruneableDecList 
 Returns the revision string.
   getRevision()   - 
Method in class weka.classifiers.rules. Prism 
 Returns the revision string.
   getRevision()   - 
Method in class weka.classifiers.rules. Ridor 
 Returns the revision string.
   getRevision()   - 
Method in class weka.classifiers.rules. RuleStats 
 Returns the revision string.
   getRevision()   - 
Method in class weka.classifiers.rules. ZeroR 
 Returns the revision string.
   getRevision()   - 
Method in class weka.classifiers.trees. ADTree 
 Returns the revision string.
   getRevision()   - 
Method in class weka.classifiers.trees.adtree. PredictionNode 
 Returns the revision string.
   getRevision()   - 
Method in class weka.classifiers.trees.adtree. ReferenceInstances 
 Returns the revision string.
   getRevision()   - 
Method in class weka.classifiers.trees.adtree. TwoWayNominalSplit 
 Returns the revision string.
   getRevision()   - 
Method in class weka.classifiers.trees.adtree. TwoWayNumericSplit 
 Returns the revision string.
   getRevision()   - 
Method in class weka.classifiers.trees. BFTree 
 Returns the revision string.
   getRevision()   - 
Method in class weka.classifiers.trees. DecisionStump 
 Returns the revision string.
   getRevision()   - 
Method in class weka.classifiers.trees.ft. FTInnerNode 
 Returns the revision string.
   getRevision()   - 
Method in class weka.classifiers.trees.ft. FTLeavesNode 
 Returns the revision string.
   getRevision()   - 
Method in class weka.classifiers.trees.ft. FTNode 
 Returns the revision string.
   getRevision()   - 
Method in class weka.classifiers.trees.ft. FTtree 
 Returns the revision string.
   getRevision()   - 
Method in class weka.classifiers.trees. FT 
 Returns the revision string.
   getRevision()   - 
Method in class weka.classifiers.trees. Id3 
 Returns the revision string.
   getRevision()   - 
Method in class weka.classifiers.trees.j48. BinC45ModelSelection 
 Returns the revision string.
   getRevision()   - 
Method in class weka.classifiers.trees.j48. BinC45Split 
 Returns the revision string.
   getRevision()   - 
Method in class weka.classifiers.trees.j48. C45ModelSelection 
 Returns the revision string.
   getRevision()   - 
Method in class weka.classifiers.trees.j48. C45PruneableClassifierTree 
 Returns the revision string.
   getRevision()   - 
Method in class weka.classifiers.trees.j48. C45PruneableClassifierTreeG 
 Returns the revision string.
   getRevision()   - 
Method in class weka.classifiers.trees.j48. C45Split 
 Returns the revision string.
   getRevision()   - 
Method in class weka.classifiers.trees.j48. ClassifierTree 
 Returns the revision string.
   getRevision()   - 
Method in class weka.classifiers.trees.j48. Distribution 
 Returns the revision string.
   getRevision()   - 
Method in class weka.classifiers.trees.j48. EntropySplitCrit 
 Returns the revision string.
   getRevision()   - 
Method in class weka.classifiers.trees.j48. GainRatioSplitCrit 
 Returns the revision string.
   getRevision()   - 
Method in class weka.classifiers.trees. J48 
 Returns the revision string.
   getRevision()   - 
Method in class weka.classifiers.trees.j48. GraftSplit 
 Returns the revision string.
   getRevision()   - 
Method in class weka.classifiers.trees.j48. InfoGainSplitCrit 
 Returns the revision string.
   getRevision()   - 
Method in class weka.classifiers.trees.j48. NBTreeClassifierTree 
 Returns the revision string.
   getRevision()   - 
Method in class weka.classifiers.trees.j48. NBTreeModelSelection 
 Returns the revision string.
   getRevision()   - 
Method in class weka.classifiers.trees.j48. NBTreeNoSplit 
 Returns the revision string.
   getRevision()   - 
Method in class weka.classifiers.trees.j48. NBTreeSplit 
 Returns the revision string.
   getRevision()   - 
Method in class weka.classifiers.trees.j48. NoSplit 
 Returns the revision string.
   getRevision()   - 
Method in class weka.classifiers.trees.j48. PruneableClassifierTree 
 Returns the revision string.
   getRevision()   - 
Method in class weka.classifiers.trees.j48. Stats 
 Returns the revision string.
   getRevision()   - 
Method in class weka.classifiers.trees. J48graft 
 Returns the revision string.
   getRevision()   - 
Method in class weka.classifiers.trees. LADTree 
 Returns the revision string.
   getRevision()   - 
Method in class weka.classifiers.trees. LMT 
 Returns the revision string.
   getRevision()   - 
Method in class weka.classifiers.trees.lmt. LMTNode 
 Returns the revision string.
   getRevision()   - 
Method in class weka.classifiers.trees.lmt. LogisticBase 
 Returns the revision string.
   getRevision()   - 
Method in class weka.classifiers.trees.lmt. ResidualModelSelection 
 Returns the revision string.
   getRevision()   - 
Method in class weka.classifiers.trees.lmt. ResidualSplit 
 Returns the revision string.
   getRevision()   - 
Method in class weka.classifiers.trees.m5. CorrelationSplitInfo 
 Returns the revision string.
   getRevision()   - 
Method in class weka.classifiers.trees.m5. Impurity 
 Returns the revision string.
   getRevision()   - 
Method in class weka.classifiers.trees.m5. PreConstructedLinearModel 
 Returns the revision string.
   getRevision()   - 
Method in class weka.classifiers.trees.m5. Rule 
 Returns the revision string.
   getRevision()   - 
Method in class weka.classifiers.trees.m5. RuleNode 
 Returns the revision string.
   getRevision()   - 
Method in class weka.classifiers.trees.m5. Values 
 Returns the revision string.
   getRevision()   - 
Method in class weka.classifiers.trees.m5. YongSplitInfo 
 Returns the revision string.
   getRevision()   - 
Method in class weka.classifiers.trees. M5P 
 Returns the revision string.
   getRevision()   - 
Method in class weka.classifiers.trees. NBTree 
 Returns the revision string.
   getRevision()   - 
Method in class weka.classifiers.trees. RandomForest 
 Returns the revision string.
   getRevision()   - 
Method in class weka.classifiers.trees. RandomTree 
 Returns the revision string.
   getRevision()   - 
Method in class weka.classifiers.trees. REPTree 
 Returns the revision string.
   getRevision()   - 
Method in class weka.classifiers.trees. SimpleCart 
 Returns the revision string.
   getRevision()   - 
Method in class weka.classifiers.trees. UserClassifier 
 Returns the revision string.
   getRevision()   - 
Method in class weka.classifiers.xml. XMLClassifier 
 Returns the revision string.
   getRevision()   - 
Method in class weka.clusterers. AbstractClusterer 
 Returns the revision string.
   getRevision()   - 
Method in class weka.clusterers. CheckClusterer 
 Returns the revision string.
   getRevision()   - 
Method in class weka.clusterers. CLOPE 
 Returns the revision string.
   getRevision()   - 
Method in class weka.clusterers. ClusterEvaluation 
 Returns the revision string.
   getRevision()   - 
Method in class weka.clusterers. Cobweb 
 Returns the revision string.
   getRevision()   - 
Method in class weka.clusterers. DBScan 
 Returns the revision string.
   getRevision()   - 
Method in class weka.clusterers. EM 
 Returns the revision string.
   getRevision()   - 
Method in class weka.clusterers. FarthestFirst 
 Returns the revision string.
   getRevision()   - 
Method in class weka.clusterers. FilteredClusterer 
 Returns the revision string.
   getRevision()   - 
Method in class weka.clusterers.forOPTICSAndDBScan.Databases. SequentialDatabase 
 Returns the revision string.
   getRevision()   - 
Method in class weka.clusterers.forOPTICSAndDBScan.DataObjects. EuclidianDataObject 
 Returns the revision string.
   getRevision()   - 
Method in class weka.clusterers.forOPTICSAndDBScan.DataObjects. ManhattanDataObject 
 Returns the revision string.
   getRevision()   - 
Method in class weka.clusterers.forOPTICSAndDBScan.OPTICS_GUI. GraphPanel 
 Returns the revision string.
   getRevision()   - 
Method in class weka.clusterers.forOPTICSAndDBScan.OPTICS_GUI. OPTICS_Visualizer 
 Returns the revision string.
   getRevision()   - 
Method in class weka.clusterers.forOPTICSAndDBScan.OPTICS_GUI. ResultVectorTableModel 
 Returns the revision string.
   getRevision()   - 
Method in class weka.clusterers.forOPTICSAndDBScan.OPTICS_GUI. SERFileFilter 
 Returns the revision string.
   getRevision()   - 
Method in class weka.clusterers.forOPTICSAndDBScan.OPTICS_GUI. SERObject 
 Returns the revision string.
   getRevision()   - 
Method in class weka.clusterers.forOPTICSAndDBScan.Utils. EpsilonRange_ListElement 
 Returns the revision string.
   getRevision()   - 
Method in class weka.clusterers.forOPTICSAndDBScan.Utils. PriorityQueue 
 Returns the revision string.
   getRevision()   - 
Method in class weka.clusterers.forOPTICSAndDBScan.Utils. PriorityQueueElement 
 Returns the revision string.
   getRevision()   - 
Method in class weka.clusterers.forOPTICSAndDBScan.Utils. UpdateQueue 
 Returns the revision string.
   getRevision()   - 
Method in class weka.clusterers.forOPTICSAndDBScan.Utils. UpdateQueueElement 
 Returns the revision string.
   getRevision()   - 
Method in class weka.clusterers. HierarchicalClusterer 
 Returns the revision string.
   getRevision()   - 
Method in class weka.clusterers. MakeDensityBasedClusterer 
 Returns the revision string.
   getRevision()   - 
Method in class weka.clusterers. OPTICS 
 Returns the revision string.
   getRevision()   - 
Method in class weka.clusterers. sIB 
 Returns the revision string.
   getRevision()   - 
Method in class weka.clusterers. SimpleKMeans 
 Returns the revision string.
   getRevision()   - 
Method in class weka.clusterers. XMeans 
 Returns the revision string.
   getRevision()   - 
Method in class weka.core. AlgVector 
 Returns the revision string.
   getRevision()   - 
Method in class weka.core. AllJavadoc 
 Returns the revision string.
   getRevision()   - 
Method in class weka.core. Attribute 
 Returns the revision string.
   getRevision()   - 
Method in class weka.core. AttributeExpression 
 Returns the revision string.
   getRevision()   - 
Method in class weka.core. AttributeLocator 
 Returns the revision string.
   getRevision()   - 
Method in class weka.core. AttributeStats 
 Returns the revision string.
   getRevision()   - 
Method in class weka.core. BinarySparseInstance 
 Returns the revision string.
   getRevision()   - 
Method in class weka.core. Capabilities 
 Returns the revision string.
   getRevision()   - 
Method in class weka.core. ChebyshevDistance 
 Returns the revision string.
   getRevision()   - 
Method in class weka.core. CheckGOE 
 Returns the revision string.
   getRevision()   - 
Method in class weka.core. CheckOptionHandler 
 Returns the revision string.
   getRevision()   - 
Method in class weka.core. CheckScheme.PostProcessor 
 Returns the revision string.
   getRevision()   - 
Method in class weka.core. ClassDiscovery 
 Returns the revision string.
   getRevision()   - 
Method in class weka.core. ClassDiscovery.StringCompare 
 Returns the revision string.
   getRevision()   - 
Method in class weka.core. ClassloaderUtil 
 Returns the revision string.
   getRevision()   - 
Method in class weka.core. ContingencyTables 
 Returns the revision string.
   getRevision()   - 
Method in class weka.core.converters. ArffLoader.ArffReader 
 Returns the revision string.
   getRevision()   - 
Method in class weka.core.converters. ArffLoader 
 Returns the revision string.
   getRevision()   - 
Method in class weka.core.converters. ArffSaver 
 Returns the revision string.
   getRevision()   - 
Method in class weka.core.converters. C45Loader 
 Returns the revision string.
   getRevision()   - 
Method in class weka.core.converters. C45Saver 
 Returns the revision string.
   getRevision()   - 
Method in class weka.core.converters. ConverterUtils.DataSink 
 Returns the revision string.
   getRevision()   - 
Method in class weka.core.converters. ConverterUtils.DataSource 
 Returns the revision string.
   getRevision()   - 
Method in class weka.core.converters. ConverterUtils 
 Returns the revision string.
   getRevision()   - 
Method in class weka.core.converters. CSVLoader 
 Returns the revision string.
   getRevision()   - 
Method in class weka.core.converters. CSVSaver 
 Returns the revision string.
   getRevision()   - 
Method in class weka.core.converters. DatabaseConnection 
 Returns the revision string.
   getRevision()   - 
Method in class weka.core.converters. DatabaseLoader 
 Returns the revision string.
   getRevision()   - 
Method in class weka.core.converters. DatabaseSaver 
 Returns the revision string.
   getRevision()   - 
Method in class weka.core.converters. LibSVMLoader 
 Returns the revision string.
   getRevision()   - 
Method in class weka.core.converters. LibSVMSaver 
 Returns the revision string.
   getRevision()   - 
Method in class weka.core.converters. SerializedInstancesLoader 
 Returns the revision string.
   getRevision()   - 
Method in class weka.core.converters. SerializedInstancesSaver 
 Returns the revision string.
   getRevision()   - 
Method in class weka.core.converters. SVMLightLoader 
 Returns the revision string.
   getRevision()   - 
Method in class weka.core.converters. SVMLightSaver 
 Returns the revision string.
   getRevision()   - 
Method in class weka.core.converters. TextDirectoryLoader 
 Returns the revision string.
   getRevision()   - 
Method in class weka.core.converters. XRFFLoader 
 Returns the revision string.
   getRevision()   - 
Method in class weka.core.converters. XRFFSaver 
 Returns the revision string.
   getRevision()   - 
Method in class weka.core. Debug.Clock 
 Returns the revision string.
   getRevision()   - 
Method in class weka.core. Debug.DBO 
 Returns the revision string.
   getRevision()   - 
Method in class weka.core. Debug 
 Returns the revision string.
   getRevision()   - 
Method in class weka.core. Debug.Log 
 Returns the revision string.
   getRevision()   - 
Method in class weka.core. Debug.Random 
 Returns the revision string.
   getRevision()   - 
Method in class weka.core. Debug.SimpleLog 
 Returns the revision string.
   getRevision()   - 
Method in class weka.core. Debug.Timestamp 
 Returns the revision string.
   getRevision()   - 
Method in class weka.core. EditDistance 
 Returns the revision string.
   getRevision()   - 
Method in class weka.core. Environment 
 Returns the revision string.
   getRevision()   - 
Method in class weka.core. EuclideanDistance 
 Returns the revision string.
   getRevision()   - 
Method in class weka.core. FastVector.FastVectorEnumeration 
 Returns the revision string.
   getRevision()   - 
Method in class weka.core. FastVector 
 Returns the revision string.
   getRevision()   - 
Method in class weka.core. FindWithCapabilities 
 Returns the revision string.
   getRevision()   - 
Method in class weka.core. GlobalInfoJavadoc 
 Returns the revision string.
   getRevision()   - 
Method in class weka.core. Instance 
 Returns the revision string.
   getRevision()   - 
Method in class weka.core. InstanceComparator 
 Returns the revision string.
   getRevision()   - 
Method in class weka.core. Instances 
 Returns the revision string.
   getRevision()   - 
Method in class weka.core. Jython 
 Returns the revision string.
   getRevision()   - 
Method in class weka.core. ListOptions 
 Returns the revision string.
   getRevision()   - 
Method in class weka.core.logging. ConsoleLogger 
 Returns the revision string.
   getRevision()   - 
Method in class weka.core.logging. FileLogger 
 Returns the revision string.
   getRevision()   - 
Method in class weka.core.logging. OutputLogger 
 Returns the revision string.
   getRevision()   - 
Method in class weka.core. ManhattanDistance 
 Returns the revision string.
   getRevision()   - 
Method in class weka.core. MathematicalExpression 
 Returns the revision string.
   getRevision()   - 
Method in class weka.core.matrix. CholeskyDecomposition 
 Returns the revision string.
   getRevision()   - 
Method in class weka.core.matrix. DoubleVector 
 Returns the revision string.
   getRevision()   - 
Method in class weka.core.matrix. EigenvalueDecomposition 
 Returns the revision string.
   getRevision()   - 
Method in class weka.core.matrix. ExponentialFormat 
 Returns the revision string.
   getRevision()   - 
Method in class weka.core.matrix. FlexibleDecimalFormat 
 Returns the revision string.
   getRevision()   - 
Method in class weka.core.matrix. FloatingPointFormat 
 Returns the revision string.
   getRevision()   - 
Method in class weka.core. Matrix 
  Deprecated.  Returns the revision string.
   getRevision()   - 
Method in class weka.core.matrix. IntVector 
 Returns the revision string.
   getRevision()   - 
Method in class weka.core.matrix. LinearRegression 
 Returns the revision string.
   getRevision()   - 
Method in class weka.core.matrix. LUDecomposition 
 Returns the revision string.
   getRevision()   - 
Method in class weka.core.matrix. Maths 
 Returns the revision string.
   getRevision()   - 
Method in class weka.core.matrix. Matrix 
 Returns the revision string.
   getRevision()   - 
Method in class weka.core.matrix. QRDecomposition 
 Returns the revision string.
   getRevision()   - 
Method in class weka.core.matrix. SingularValueDecomposition 
 Returns the revision string.
   getRevision()   - 
Method in class weka.core. Memory 
 Returns the revision string.
   getRevision()   - 
Method in class weka.core.neighboursearch. BallTree 
 Returns the revision string.
   getRevision()   - 
Method in class weka.core.neighboursearch.balltrees. BallNode 
 Returns the revision string.
   getRevision()   - 
Method in class weka.core.neighboursearch.balltrees. BallSplitter 
 Returns the revision string.
   getRevision()   - 
Method in class weka.core.neighboursearch.balltrees. BallTreeConstructor 
 Returns the revision string.
   getRevision()   - 
Method in class weka.core.neighboursearch.balltrees. BottomUpConstructor 
 Returns the revision string.
   getRevision()   - 
Method in class weka.core.neighboursearch.balltrees. MedianDistanceFromArbitraryPoint 
 Returns the revision string.
   getRevision()   - 
Method in class weka.core.neighboursearch.balltrees. MedianOfWidestDimension 
 Returns the revision string.
   getRevision()   - 
Method in class weka.core.neighboursearch.balltrees. MiddleOutConstructor 
 Returns the revision string.
   getRevision()   - 
Method in class weka.core.neighboursearch.balltrees. PointsClosestToFurthestChildren 
 Returns the revision string.
   getRevision()   - 
Method in class weka.core.neighboursearch.balltrees. TopDownConstructor 
 Returns the revision string.
   getRevision()   - 
Method in class weka.core.neighboursearch. CoverTree.CoverTreeNode 
 Returns the revision string.
   getRevision()   - 
Method in class weka.core.neighboursearch. CoverTree 
 Returns the revision string.
   getRevision()   - 
Method in class weka.core.neighboursearch.covertrees. Stack 
 Returns the revision string.
   getRevision()   - 
Method in class weka.core.neighboursearch. KDTree 
 Returns the revision string.
   getRevision()   - 
Method in class weka.core.neighboursearch.kdtrees. KDTreeNode 
 Returns the revision string.
   getRevision()   - 
Method in class weka.core.neighboursearch.kdtrees. KDTreeNodeSplitter 
 Returns the revision string.
   getRevision()   - 
Method in class weka.core.neighboursearch.kdtrees. KMeansInpiredMethod 
 Returns the revision string.
   getRevision()   - 
Method in class weka.core.neighboursearch.kdtrees. MedianOfWidestDimension 
 Returns the revision string.
   getRevision()   - 
Method in class weka.core.neighboursearch.kdtrees. MidPointOfWidestDimension 
 Returns the revision string.
   getRevision()   - 
Method in class weka.core.neighboursearch.kdtrees. SlidingMidPointOfWidestSide 
 Returns the revision string.
   getRevision()   - 
Method in class weka.core.neighboursearch. LinearNNSearch 
 Returns the revision string.
   getRevision()   - 
Method in class weka.core.neighboursearch. PerformanceStats 
 Returns the revision string.
   getRevision()   - 
Method in class weka.core.neighboursearch. TreePerformanceStats 
 Returns the revision string.
   getRevision()   - 
Method in class weka.core. Option 
 Returns the revision string.
   getRevision()   - 
Method in class weka.core. OptionHandlerJavadoc 
 Returns the revision string.
   getRevision()   - 
Method in class weka.core. PropertyPath 
 Returns the revision string.
   getRevision()   - 
Method in class weka.core. PropertyPath.Path 
 Returns the revision string.
   getRevision()   - 
Method in class weka.core. PropertyPath.PathElement 
 Returns the revision string.
   getRevision()   - 
Method in class weka.core. ProtectedProperties 
 Returns the revision string.
   getRevision()   - 
Method in class weka.core. Queue 
 Returns the revision string.
   getRevision()   - 
Method in class weka.core. RandomVariates 
 Returns the revision string.
   getRevision()   - 
Method in class weka.core. Range 
 Returns the revision string.
   getRevision()   - 
Method in class weka.core. RelationalLocator 
 Returns the revision string.
   getRevision()   - 
Method in interface weka.core. RevisionHandler 
 Returns the revision string.
   getRevision()   - 
Method in class weka.core. SelectedTag 
 Returns the revision string.
   getRevision()   - 
Method in class weka.core. SerializationHelper 
 Returns the revision string.
   getRevision()   - 
Method in class weka.core. SerializedObject 
 Returns the revision string.
   getRevision()   - 
Method in class weka.core. SingleIndex 
 Returns the revision string.
   getRevision()   - 
Method in class weka.core. SparseInstance 
 Returns the revision string.
   getRevision()   - 
Method in class weka.core. SpecialFunctions 
 Returns the revision string.
   getRevision()   - 
Method in class weka.core. Statistics 
 Returns the revision string.
   getRevision()   - 
Method in class weka.core.stemmers. IteratedLovinsStemmer 
 Returns the revision string.
   getRevision()   - 
Method in class weka.core.stemmers. LovinsStemmer 
 Returns the revision string.
   getRevision()   - 
Method in class weka.core.stemmers. NullStemmer 
 Returns the revision string.
   getRevision()   - 
Method in class weka.core.stemmers. SnowballStemmer 
 Returns the revision string.
   getRevision()   - 
Method in class weka.core.stemmers. Stemming 
 Returns the revision string.
   getRevision()   - 
Method in class weka.core. Stopwords 
 Returns the revision string.
   getRevision()   - 
Method in class weka.core. StringLocator 
 Returns the revision string.
   getRevision()   - 
Method in class weka.core. SystemInfo 
 Returns the revision string.
   getRevision()   - 
Method in class weka.core. Tag 
 Returns the revision string.
   getRevision()   - 
Method in class weka.core. TechnicalInformation 
 Returns the revision string.
   getRevision()   - 
Method in class weka.core. TechnicalInformationHandlerJavadoc 
 Returns the revision string.
   getRevision()   - 
Method in class weka.core. Tee 
 Returns the revision string.
   getRevision()   - 
Method in class weka.core. TestInstances 
 Returns the revision string.
   getRevision()   - 
Method in class weka.core.tokenizers. AlphabeticTokenizer 
 Returns the revision string.
   getRevision()   - 
Method in class weka.core.tokenizers. NGramTokenizer 
 Returns the revision string.
   getRevision()   - 
Method in class weka.core.tokenizers. WordTokenizer 
 Returns the revision string.
   getRevision()   - 
Method in class weka.core. Trie 
 Returns the revision string.
   getRevision()   - 
Method in class weka.core. Trie.TrieIterator 
 Returns the revision string.
   getRevision()   - 
Method in class weka.core. Trie.TrieNode 
 Returns the revision string.
   getRevision()   - 
Method in class weka.core. Utils 
 Returns the revision string.
   getRevision()   - 
Method in class weka.core. Version 
 Returns the revision string.
   getRevision()   - 
Method in class weka.core.xml. KOML 
 Returns the revision string.
   getRevision()   - 
Method in class weka.core.xml. MethodHandler 
 Returns the revision string.
   getRevision()   - 
Method in class weka.core.xml. PropertyHandler 
 Returns the revision string.
   getRevision()   - 
Method in class weka.core.xml. SerialUIDChanger 
 Returns the revision string.
   getRevision()   - 
Method in class weka.core.xml. XMLBasicSerialization 
 Returns the revision string.
   getRevision()   - 
Method in class weka.core.xml. XMLDocument 
 Returns the revision string.
   getRevision()   - 
Method in class weka.core.xml. XMLInstances 
 Returns the revision string.
   getRevision()   - 
Method in class weka.core.xml. XMLOptions 
 Returns the revision string.
   getRevision()   - 
Method in class weka.core.xml. XMLSerialization 
 Returns the revision string.
   getRevision()   - 
Method in class weka.core.xml. XMLSerializationMethodHandler 
 Returns the revision string.
   getRevision()   - 
Method in class weka.core.xml. XStream 
 Returns the revision string.
   getRevision()   - 
Method in class weka.datagenerators.classifiers.classification. Agrawal 
 Returns the revision string.
   getRevision()   - 
Method in class weka.datagenerators.classifiers.classification. BayesNet 
 Returns the revision string.
   getRevision()   - 
Method in class weka.datagenerators.classifiers.classification. LED24 
 Returns the revision string.
   getRevision()   - 
Method in class weka.datagenerators.classifiers.classification. RandomRBF 
 Returns the revision string.
   getRevision()   - 
Method in class weka.datagenerators.classifiers.classification. RDG1 
 Returns the revision string.
   getRevision()   - 
Method in class weka.datagenerators.classifiers.regression. Expression 
 Returns the revision string.
   getRevision()   - 
Method in class weka.datagenerators.classifiers.regression. MexicanHat 
 Returns the revision string.
   getRevision()   - 
Method in class weka.datagenerators.clusterers. BIRCHCluster 
 Returns the revision string.
   getRevision()   - 
Method in class weka.datagenerators.clusterers. SubspaceCluster 
 Returns the revision string.
   getRevision()   - 
Method in class weka.datagenerators.clusterers. SubspaceClusterDefinition 
 Returns the revision string.
   getRevision()   - 
Method in class weka.datagenerators. Test 
 Returns the revision string.
   getRevision()   - 
Method in class weka.estimators. CheckEstimator.AttrTypes 
 Returns the revision string.
   getRevision()   - 
Method in class weka.estimators. CheckEstimator.EstTypes 
 Returns the revision string.
   getRevision()   - 
Method in class weka.estimators. CheckEstimator 
 Returns the revision string.
   getRevision()   - 
Method in class weka.estimators. CheckEstimator.PostProcessor 
 Returns the revision string.
   getRevision()   - 
Method in class weka.estimators. DDConditionalEstimator 
 Returns the revision string.
   getRevision()   - 
Method in class weka.estimators. DiscreteEstimator 
 Returns the revision string.
   getRevision()   - 
Method in class weka.estimators. DKConditionalEstimator 
 Returns the revision string.
   getRevision()   - 
Method in class weka.estimators. DNConditionalEstimator 
 Returns the revision string.
   getRevision()   - 
Method in class weka.estimators. EstimatorUtils 
 Returns the revision string.
   getRevision()   - 
Method in class weka.estimators. KDConditionalEstimator 
 Returns the revision string.
   getRevision()   - 
Method in class weka.estimators. KernelEstimator 
 Returns the revision string.
   getRevision()   - 
Method in class weka.estimators. KKConditionalEstimator 
 Returns the revision string.
   getRevision()   - 
Method in class weka.estimators. MahalanobisEstimator 
 Returns the revision string.
   getRevision()   - 
Method in class weka.estimators. NDConditionalEstimator 
 Returns the revision string.
   getRevision()   - 
Method in class weka.estimators. NNConditionalEstimator 
 Returns the revision string.
   getRevision()   - 
Method in class weka.estimators. NormalEstimator 
 Returns the revision string.
   getRevision()   - 
Method in class weka.estimators. PoissonEstimator 
 Returns the revision string.
   getRevision()   - 
Method in class weka.experiment. AveragingResultProducer 
 Returns the revision string.
   getRevision()   - 
Method in class weka.experiment. ClassifierSplitEvaluator 
 Returns the revision string.
   getRevision()   - 
Method in class weka.experiment. CostSensitiveClassifierSplitEvaluator 
 Returns the revision string.
   getRevision()   - 
Method in class weka.experiment. CrossValidationResultProducer 
 Returns the revision string.
   getRevision()   - 
Method in class weka.experiment. CSVResultListener 
 Returns the revision string.
   getRevision()   - 
Method in class weka.experiment. DatabaseResultListener 
 Returns the revision string.
   getRevision()   - 
Method in class weka.experiment. DatabaseResultProducer 
 Returns the revision string.
   getRevision()   - 
Method in class weka.experiment. DatabaseUtils 
 Returns the revision string.
   getRevision()   - 
Method in class weka.experiment. DensityBasedClustererSplitEvaluator 
 Returns the revision string.
   getRevision()   - 
Method in class weka.experiment. Experiment 
 Returns the revision string.
   getRevision()   - 
Method in class weka.experiment. InstanceQuery 
 Returns the revision string.
   getRevision()   - 
Method in class weka.experiment. InstancesResultListener 
 Returns the revision string.
   getRevision()   - 
Method in class weka.experiment. LearningRateResultProducer 
 Returns the revision string.
   getRevision()   - 
Method in class weka.experiment. OutputZipper 
 Returns the revision string.
   getRevision()   - 
Method in class weka.experiment. PairedCorrectedTTester 
 Returns the revision string.
   getRevision()   - 
Method in class weka.experiment. PairedStats 
 Returns the revision string.
   getRevision()   - 
Method in class weka.experiment. PairedStatsCorrected 
 Returns the revision string.
   getRevision()   - 
Method in class weka.experiment. PairedTTester 
 Returns the revision string.
   getRevision()   - 
Method in class weka.experiment. PropertyNode 
 Returns the revision string.
   getRevision()   - 
Method in class weka.experiment. RandomSplitResultProducer 
 Returns the revision string.
   getRevision()   - 
Method in class weka.experiment. RegressionSplitEvaluator 
 Returns the revision string.
   getRevision()   - 
Method in class weka.experiment. RemoteEngine 
 Returns the revision string.
   getRevision()   - 
Method in class weka.experiment. RemoteExperiment 
 Returns the revision string.
   getRevision()   - 
Method in class weka.experiment. RemoteExperimentSubTask 
 Returns the revision string.
   getRevision()   - 
Method in class weka.experiment. ResultMatrixCSV 
 Returns the revision string.
   getRevision()   - 
Method in class weka.experiment. ResultMatrixGnuPlot 
 Returns the revision string.
   getRevision()   - 
Method in class weka.experiment. ResultMatrixHTML 
 Returns the revision string.
   getRevision()   - 
Method in class weka.experiment. ResultMatrixLatex 
 Returns the revision string.
   getRevision()   - 
Method in class weka.experiment. ResultMatrixPlainText 
 Returns the revision string.
   getRevision()   - 
Method in class weka.experiment. ResultMatrixSignificance 
 Returns the revision string.
   getRevision()   - 
Method in class weka.experiment. Stats 
 Returns the revision string.
   getRevision()   - 
Method in class weka.experiment. TaskStatusInfo 
 Returns the revision string.
   getRevision()   - 
Method in class weka.experiment.xml. XMLExperiment 
 Returns the revision string.
   getRevision()   - 
Method in class weka.filters. AllFilter 
 Returns the revision string.
   getRevision()   - 
Method in class weka.filters. CheckSource 
 Returns the revision string.
   getRevision()   - 
Method in class weka.filters. Filter 
 Returns the revision string.
   getRevision()   - 
Method in class weka.filters. MultiFilter 
 Returns the revision string.
   getRevision()   - 
Method in class weka.filters.supervised.attribute. AddClassification 
 Returns the revision string.
   getRevision()   - 
Method in class weka.filters.supervised.attribute. AttributeSelection 
 Returns the revision string.
   getRevision()   - 
Method in class weka.filters.supervised.attribute. ClassOrder 
 Returns the revision string.
   getRevision()   - 
Method in class weka.filters.supervised.attribute. Discretize 
 Returns the revision string.
   getRevision()   - 
Method in class weka.filters.supervised.attribute. NominalToBinary 
 Returns the revision string.
   getRevision()   - 
Method in class weka.filters.supervised.attribute. PLSFilter 
 Returns the revision string.
   getRevision()   - 
Method in class weka.filters.supervised.instance. Resample 
 Returns the revision string.
   getRevision()   - 
Method in class weka.filters.supervised.instance. SMOTE 
 Returns the revision string.
   getRevision()   - 
Method in class weka.filters.supervised.instance. SpreadSubsample 
 Returns the revision string.
   getRevision()   - 
Method in class weka.filters.supervised.instance. StratifiedRemoveFolds 
 Returns the revision string.
   getRevision()   - 
Method in class weka.filters.unsupervised.attribute. Add 
 Returns the revision string.
   getRevision()   - 
Method in class weka.filters.unsupervised.attribute. AddCluster 
 Returns the revision string.
   getRevision()   - 
Method in class weka.filters.unsupervised.attribute. AddExpression 
 Returns the revision string.
   getRevision()   - 
Method in class weka.filters.unsupervised.attribute. AddID 
 Returns the revision string.
   getRevision()   - 
Method in class weka.filters.unsupervised.attribute. AddNoise 
 Returns the revision string.
   getRevision()   - 
Method in class weka.filters.unsupervised.attribute. AddValues 
 Returns the revision string.
   getRevision()   - 
Method in class weka.filters.unsupervised.attribute. Center 
 Returns the revision string.
   getRevision()   - 
Method in class weka.filters.unsupervised.attribute. ChangeDateFormat 
 Returns the revision string.
   getRevision()   - 
Method in class weka.filters.unsupervised.attribute. ClassAssigner 
 Returns the revision string.
   getRevision()   - 
Method in class weka.filters.unsupervised.attribute. ClusterMembership 
 Returns the revision string.
   getRevision()   - 
Method in class weka.filters.unsupervised.attribute. Copy 
 Returns the revision string.
   getRevision()   - 
Method in class weka.filters.unsupervised.attribute. Discretize 
 Returns the revision string.
   getRevision()   - 
Method in class weka.filters.unsupervised.attribute. FirstOrder 
 Returns the revision string.
   getRevision()   - 
Method in class weka.filters.unsupervised.attribute. InterquartileRange 
 Returns the revision string.
   getRevision()   - 
Method in class weka.filters.unsupervised.attribute. KernelFilter 
 Returns the revision string.
   getRevision()   - 
Method in class weka.filters.unsupervised.attribute. MakeIndicator 
 Returns the revision string.
   getRevision()   - 
Method in class weka.filters.unsupervised.attribute. MathExpression 
 Returns the revision string.
   getRevision()   - 
Method in class weka.filters.unsupervised.attribute. MergeTwoValues 
 Returns the revision string.
   getRevision()   - 
Method in class weka.filters.unsupervised.attribute. MultiInstanceToPropositional 
 Returns the revision string.
   getRevision()   - 
Method in class weka.filters.unsupervised.attribute. NominalToBinary 
 Returns the revision string.
   getRevision()   - 
Method in class weka.filters.unsupervised.attribute. NominalToString 
 Returns the revision string.
   getRevision()   - 
Method in class weka.filters.unsupervised.attribute. Normalize 
 Returns the revision string.
   getRevision()   - 
Method in class weka.filters.unsupervised.attribute. NumericCleaner 
 Returns the revision string.
   getRevision()   - 
Method in class weka.filters.unsupervised.attribute. NumericToBinary 
 Returns the revision string.
   getRevision()   - 
Method in class weka.filters.unsupervised.attribute. NumericToNominal 
 Returns the revision string.
   getRevision()   - 
Method in class weka.filters.unsupervised.attribute. NumericTransform 
 Returns the revision string.
   getRevision()   - 
Method in class weka.filters.unsupervised.attribute. Obfuscate 
 Returns the revision string.
   getRevision()   - 
Method in class weka.filters.unsupervised.attribute. PartitionedMultiFilter 
 Returns the revision string.
   getRevision()   - 
Method in class weka.filters.unsupervised.attribute. PKIDiscretize 
 Returns the revision string.
   getRevision()   - 
Method in class weka.filters.unsupervised.attribute. PrincipalComponents 
 Returns the revision string.
   getRevision()   - 
Method in class weka.filters.unsupervised.attribute. PropositionalToMultiInstance 
 Returns the revision string.
   getRevision()   - 
Method in class weka.filters.unsupervised.attribute. RandomProjection 
 Returns the revision string.
   getRevision()   - 
Method in class weka.filters.unsupervised.attribute. RandomSubset 
 Returns the revision string.
   getRevision()   - 
Method in class weka.filters.unsupervised.attribute. RELAGGS 
 Returns the revision string.
   getRevision()   - 
Method in class weka.filters.unsupervised.attribute. Remove 
 Returns the revision string.
   getRevision()   - 
Method in class weka.filters.unsupervised.attribute. RemoveType 
 Returns the revision string.
   getRevision()   - 
Method in class weka.filters.unsupervised.attribute. RemoveUseless 
 Returns the revision string.
   getRevision()   - 
Method in class weka.filters.unsupervised.attribute. Reorder 
 Returns the revision string.
   getRevision()   - 
Method in class weka.filters.unsupervised.attribute. ReplaceMissingValues 
 Returns the revision string.
   getRevision()   - 
Method in class weka.filters.unsupervised.attribute. Standardize 
 Returns the revision string.
   getRevision()   - 
Method in class weka.filters.unsupervised.attribute. StringToNominal 
 Returns the revision string.
   getRevision()   - 
Method in class weka.filters.unsupervised.attribute. StringToWordVector 
 Returns the revision string.
   getRevision()   - 
Method in class weka.filters.unsupervised.attribute. SwapValues 
 Returns the revision string.
   getRevision()   - 
Method in class weka.filters.unsupervised.attribute. TimeSeriesDelta 
 Returns the revision string.
   getRevision()   - 
Method in class weka.filters.unsupervised.attribute. TimeSeriesTranslate 
 Returns the revision string.
   getRevision()   - 
Method in class weka.filters.unsupervised.attribute. Wavelet 
 Returns the revision string.
   getRevision()   - 
Method in class weka.filters.unsupervised.instance. NonSparseToSparse 
 Returns the revision string.
   getRevision()   - 
Method in class weka.filters.unsupervised.instance. Normalize 
 Returns the revision string.
   getRevision()   - 
Method in class weka.filters.unsupervised.instance. Randomize 
 Returns the revision string.
   getRevision()   - 
Method in class weka.filters.unsupervised.instance. RemoveFolds 
 Returns the revision string.
   getRevision()   - 
Method in class weka.filters.unsupervised.instance. RemoveFrequentValues 
 Returns the revision string.
   getRevision()   - 
Method in class weka.filters.unsupervised.instance. RemoveMisclassified 
 Returns the revision string.
   getRevision()   - 
Method in class weka.filters.unsupervised.instance. RemovePercentage 
 Returns the revision string.
   getRevision()   - 
Method in class weka.filters.unsupervised.instance. RemoveRange 
 Returns the revision string.
   getRevision()   - 
Method in class weka.filters.unsupervised.instance. RemoveWithValues 
 Returns the revision string.
   getRevision()   - 
Method in class weka.filters.unsupervised.instance. Resample 
 Returns the revision string.
   getRevision()   - 
Method in class weka.filters.unsupervised.instance. ReservoirSample 
 Returns the revision string.
   getRevision()   - 
Method in class weka.filters.unsupervised.instance. SparseToNonSparse 
 Returns the revision string.
   getRevision()   - 
Method in class weka.filters.unsupervised.instance. SubsetByExpression 
 Returns the revision string.
   getRevision()   - 
Method in class weka.gui.beans. FlowRunner 
  
   getRevision()   - 
Method in class weka.gui.sql. DbUtils 
 Returns the revision string.
   getRidge()   - 
Method in class weka.classifiers.functions. LinearRegression 
 Get the value of Ridge.
   getRidge()   - 
Method in class weka.classifiers.functions. Logistic 
 Gets the ridge in the log-likelihood.
   getRidge()   - 
Method in class weka.classifiers.functions. RBFNetwork 
 Gets the ridge value.
   getRidge()   - 
Method in class weka.classifiers.mi. MILR 
 Gets the ridge in the log-likelihood.
   getRocAnalysis()   - 
Method in class weka.associations. Tertius 
 Get the value of rocAnalysis.
   getROCArea(Instances)   - 
Static method in class weka.classifiers.evaluation. ThresholdCurve 
 Calculates the area under the ROC curve as the Wilcoxon-Mann-Whitney statistic.
   getROCString()   - 
Method in class weka.gui.visualize. ThresholdVisualizePanel 
 This extracts the ROC area string
   getRoot()   - 
Method in class weka.core. Trie 
 returns the root node of the trie
   getRoot()   - 
Method in class weka.gui.treevisualizer. Node 
 Get the value of root.
   getRootNode()   - 
Method in class weka.core.xml. XMLDocument 
 returns the current root node.
   getRow(int)   - 
Method in class weka.core. Matrix 
  Deprecated.  Gets a row of the matrix and returns it as double array.
   getRow()   - 
Static method in class weka.gui.experiment. ExperimenterDefaults 
 the comma-separated list of attribute names that identify a row
   getRowCount()   - 
Method in class weka.clusterers.forOPTICSAndDBScan.OPTICS_GUI. ResultVectorTableModel 
 Returns the number of rows of this model.
   getRowCount()   - 
Method in class weka.experiment. ResultMatrix 
 returns the number of rows
   getRowCount()   - 
Method in class weka.gui.arffviewer. ArffTableModel 
 returns the number of rows in the model
   getRowCount()   - 
Method in class weka.gui. SortedTableModel 
 Returns the number of rows in the model.
   getRowCount()   - 
Method in class weka.gui.sql. ResultSetHelper 
 returns the number of rows in the resultset.
   getRowCount()   - 
Method in class weka.gui.sql. ResultSetTableModel 
 returns the number of rows in the model.
   getRowDimension()   - 
Method in class weka.core.matrix. Matrix 
 Get row dimension.
   getRowHidden(int)   - 
Method in class weka.experiment. ResultMatrix 
 returns the hidden status of the row, if the index is valid, otherwise
 false
   getRowName(int)   - 
Method in class weka.experiment. ResultMatrix 
 returns the name of the row, if the index is valid, otherwise null.
   getRowNameWidth()   - 
Method in class weka.experiment. ResultMatrix 
 returns the current width for the row names
   getRowOrder()   - 
Method in class weka.experiment. ResultMatrix 
 returns the current order of the rows, null means the default order
   getRowPackedCopy()   - 
Method in class weka.core.matrix. Matrix 
 Make a one-dimensional row packed copy of the internal array.
   getRsource()   - 
Method in class weka.gui.treevisualizer. Edge 
 Get the value of rsource.
   getRtarget()   - 
Method in class weka.gui.treevisualizer. Edge 
 Get the value of rtarget.
   getRuleset()   - 
Method in class weka.classifiers.rules. JRip 
 Get the ruleset generated by Ripper
   getRuleset()   - 
Method in class weka.classifiers.rules. RuleStats 
 Get the ruleset of the stats
   getRulesetSize()   - 
Method in class weka.classifiers.rules. RuleStats 
 Get the size of the ruleset in the stats
   getRulesMustContain()   - 
Method in class weka.associations. FPGrowth 
 Get the comma separated list of items that
 rules must contain in order to be output.
   getRuleStats(int)   - 
Method in class weka.classifiers.rules. JRip 
 Get the statistics of the ruleset in the given position
   getRunColumn()   - 
Method in class weka.experiment. PairedTTester 
 Get the value of RunColumn.
   getRunColumn()   - 
Method in interface weka.experiment. Tester 
 Get the value of RunColumn.
   getRunLower()   - 
Method in class weka.experiment. Experiment 
 Get the lower run number for the experiment.
   getRunNumber()   - 
Method in class weka.gui.beans. BatchClassifierEvent 
 Get the run number.
   getRunNumber()   - 
Method in class weka.gui.beans. TestSetEvent 
 Get the run number that this training set belongs to.
   getRunNumber()   - 
Method in class weka.gui.beans. TrainingSetEvent 
 Get the run number that this training set belongs to.
   getRuns()   - 
Method in class weka.classifiers.bayes.net.search.global. GeneticSearch 
  
   getRuns()   - 
Method in class weka.classifiers.bayes.net.search.global. RepeatedHillClimber 
 Returns the number of runs
   getRuns()   - 
Method in class weka.classifiers.bayes.net.search.global. SimulatedAnnealing 
  
   getRuns()   - 
Method in class weka.classifiers.bayes.net.search.global. TabuSearch 
  
   getRuns()   - 
Method in class weka.classifiers.bayes.net.search.local. GeneticSearch 
  
   getRuns()   - 
Method in class weka.classifiers.bayes.net.search.local. RepeatedHillClimber 
  
   getRuns()   - 
Method in class weka.classifiers.bayes.net.search.local. SimulatedAnnealing 
  
   getRuns()   - 
Method in class weka.classifiers.bayes.net.search.local. TabuSearch 
  
   getRunUpper()   - 
Method in class weka.experiment. Experiment 
 Get the upper run number for the experiment.
   getS()   - 
Method in class weka.core.matrix. SingularValueDecomposition 
 Return the diagonal matrix of singular values
   getSampleSize()   - 
Method in class weka.attributeSelection. ReliefFAttributeEval 
 Get the number of instances used for estimating attributes
   getSampleSize()   - 
Method in class weka.classifiers.functions. LeastMedSq 
 gets number of samples
   getSampleSize()   - 
Method in class weka.filters.unsupervised.instance. ReservoirSample 
 Gets the subsample size.
   getSampleSizePercent()   - 
Method in class weka.classifiers.meta. GridSearch 
 Gets the sample size for the initial grid search.
   getSampleSizePercent()   - 
Method in class weka.filters.supervised.instance. Resample 
 Gets the subsample size as a percentage of the original set.
   getSampleSizePercent()   - 
Method in class weka.filters.unsupervised.instance. Resample 
 Gets the subsample size as a percentage of the original set.
   getSaveDialogTitle()   - 
Method in class weka.gui.visualize. PrintableComponent 
 returns the title for the save dialog.
   getSaveDialogTitle()   - 
Method in interface weka.gui.visualize. PrintableHandler 
 returns the title for the save dialog
   getSaveDialogTitle()   - 
Method in class weka.gui.visualize. PrintablePanel 
 returns the title for the save dialog
   getSaveInstanceData()   - 
Method in class weka.classifiers.trees. ADTree 
 Gets whether the tree is to save instance data.
   getSaveInstanceData()   - 
Method in class weka.classifiers.trees. J48 
 Check whether instance data is to be saved.
   getSaveInstanceData()   - 
Method in class weka.classifiers.trees. J48graft 
 Check whether instance data is to be saved.
   getSaveInstanceData()   - 
Method in class weka.clusterers. Cobweb 
 Get the value of saveInstances.
   getSaveInstances()   - 
Method in class weka.classifiers.trees. M5P 
 Get whether instance data is being save.
   getSaver()   - 
Method in class weka.gui. ConverterFileChooser 
 returns the saver that was chosen by the user, can be null in case the
 user aborted the dialog or the open dialog was shown
   getSaverForExtension(String)   - 
Static method in class weka.core.converters. ConverterUtils 
 tries to determine the saver to use for this kind of extension, returns
 null if none can be found.
   getSaverForFile(String)   - 
Static method in class weka.core.converters. ConverterUtils 
 tries to determine the saver to use for this kind of file, returns
 null if none can be found.
   getSaverForFile(File)   - 
Static method in class weka.core.converters. ConverterUtils 
 tries to determine the saver to use for this kind of file, returns
 null if none can be found.
   getSaverTemplate()   - 
Method in class weka.gui.beans. Saver 
 Get the saver
   getScale()   - 
Method in class weka.filters.unsupervised.attribute. Normalize 
 Get the scaling factor.
   getScalingEnabled()   - 
Method in class weka.gui.visualize. JComponentWriter 
 whether scaling is enabled or ignored
   getScoreType()   - 
Method in class weka.classifiers.bayes.net.search.local. LocalScoreSearchAlgorithm 
 get quality measure to be used in searching for networks.
   getSearch()   - 
Method in class weka.attributeSelection. CheckAttributeSelection 
 Get the current search method
   getSearch()   - 
Method in class weka.classifiers.meta. AttributeSelectedClassifier 
 Gets the search method used
   getSearch()   - 
Method in class weka.classifiers.rules. DecisionTable 
 Gets the current search method
   getSearch()   - 
Method in class weka.classifiers.rules. DTNB 
 Gets the current search method
   getSearch()   - 
Method in class weka.filters.supervised.attribute. AttributeSelection 
 Get the name of the search method
   getSearchAlgorithm()   - 
Method in class weka.classifiers.bayes. BayesNet 
 Get the SearchAlgorithm used as the search algorithm
   getSearchBackwards()   - 
Method in class weka.attributeSelection. GreedyStepwise 
 Get whether to search backwards
   getSearchPath()   - 
Method in class weka.classifiers.trees. ADTree 
 Gets the method of searching the tree for a new insertion.
   getSearchPercent()   - 
Method in class weka.attributeSelection. RandomSearch 
 get the percentage of the search space to consider
   getSearchString()   - 
Method in class weka.gui.arffviewer. ArffTable 
 returns the search string, can be NULL if no search string is set
   getSearchTermination()   - 
Method in class weka.attributeSelection. BestFirst 
 Get the termination criterion (number of non-improving nodes).
   getSearchTermination()   - 
Method in class weka.attributeSelection. LinearForwardSelection 
 Get the termination criterion (number of non-improving nodes).
   getSecondValueIndex()   - 
Method in class weka.filters.unsupervised.attribute. MergeTwoValues 
 Get the index of the second value used.
   getSecondValueIndex()   - 
Method in class weka.filters.unsupervised.attribute. SwapValues 
 Get the index of the second value used.
   getSeed()   - 
Method in class weka.attributeSelection. CostSensitiveASEvaluation 
 Gets the seed for the random number generations.
   getSeed()   - 
Method in class weka.attributeSelection. GeneticSearch 
 get the value of the random number generator's seed
   getSeed()   - 
Method in class weka.attributeSelection. OneRAttributeEval 
 Get the random number seed
   getSeed()   - 
Method in class weka.attributeSelection. ReliefFAttributeEval 
 Get the seed used for randomly sampling instances.
   getSeed()   - 
Method in class weka.attributeSelection. ScatterSearchV1 
 get the value of the random number generator's seed
   getSeed()   - 
Method in class weka.attributeSelection. SubsetSizeForwardSelection 
 Seed for cross validation subset size determination.
   getSeed()   - 
Method in class weka.attributeSelection. WrapperSubsetEval 
 Get the random number seed used for cross validation
   getSeed()   - 
Method in class weka.classifiers.bayes.net.search.global. GeneticSearch 
  
   getSeed()   - 
Method in class weka.classifiers.bayes.net.search.global. RepeatedHillClimber 
 Returns the random seed
   getSeed()   - 
Method in class weka.classifiers.bayes.net.search.global. SimulatedAnnealing 
  
   getSeed()   - 
Method in class weka.classifiers.bayes.net.search.local. GeneticSearch 
  
   getSeed()   - 
Method in class weka.classifiers.bayes.net.search.local. RepeatedHillClimber 
  
   getSeed()   - 
Method in class weka.classifiers.bayes.net.search.local. SimulatedAnnealing 
  
   getSeed()   - 
Method in class weka.classifiers. BVDecompose 
 Gets the random number seed
   getSeed()   - 
Method in class weka.classifiers. BVDecomposeSegCVSub 
 Gets the random number seed
   getSeed()   - 
Method in class weka.classifiers.evaluation. EvaluationUtils 
 Gets the seed for randomization during cross-validation
   getSeed()   - 
Method in class weka.classifiers.functions. MultilayerPerceptron 
  
   getSeed()   - 
Method in class weka.classifiers.functions.supportVector. RegOptimizer 
 Gets the current seed value for the random number generator
   getSeed()   - 
Method in class weka.classifiers.functions. VotedPerceptron 
 Get the value of Seed.
   getSeed()   - 
Method in class weka.classifiers.functions. Winnow 
 Get the value of Seed.
   getSeed()   - 
Method in class weka.classifiers.meta. MultiScheme 
 Gets the random number seed.
   getSeed()   - 
Method in class weka.classifiers. RandomizableClassifier 
 Gets the seed for the random number generations
   getSeed()   - 
Method in class weka.classifiers. RandomizableIteratedSingleClassifierEnhancer 
 Gets the seed for the random number generations
   getSeed()   - 
Method in class weka.classifiers. RandomizableMultipleClassifiersCombiner 
 Gets the seed for the random number generations
   getSeed()   - 
Method in class weka.classifiers. RandomizableSingleClassifierEnhancer 
 Gets the seed for the random number generations
   getSeed()   - 
Method in class weka.classifiers.rules. ConjunctiveRule 
 returns the current seed value for randomizing the data
   getSeed()   - 
Method in class weka.classifiers.rules. JRip 
 Gets the current seed value to use in randomizing the data
   getSeed()   - 
Method in class weka.classifiers.rules. PART 
 Get the value of Seed.
   getSeed()   - 
Method in class weka.classifiers.rules. Ridor 
  
   getSeed()   - 
Method in class weka.classifiers.trees. J48 
 Get the value of Seed.
   getSeed()   - 
Method in class weka.classifiers.trees. RandomForest 
 Gets the seed for the random number generations
   getSeed()   - 
Method in class weka.classifiers.trees. RandomTree 
 Gets the seed for the random number generations
   getSeed()   - 
Method in class weka.classifiers.trees. REPTree 
 Get the value of Seed.
   getSeed()   - 
Method in class weka.clusterers. RandomizableClusterer 
 Gets the seed for the random number generations
   getSeed()   - 
Method in class weka.clusterers. RandomizableDensityBasedClusterer 
 Gets the seed for the random number generations
   getSeed()   - 
Method in class weka.clusterers. RandomizableSingleClustererEnhancer 
 Gets the seed for the random number generations
   getSeed()   - 
Method in class weka.core.neighboursearch.balltrees. MiddleOutConstructor 
 Returns the seed for random number generator.
   getSeed()   - 
Method in interface weka.core. Randomizable 
 Gets the seed for the random number generations
   getSeed()   - 
Method in class weka.core. TestInstances 
 returns the current seed value
   getSeed()   - 
Method in class weka.datagenerators. DataGenerator 
 Gets the random number seed.
   getSeed()   - 
Method in class weka.filters.supervised.attribute. ClassOrder 
 Get the current randomization seed
   getSeed()   - 
Method in class weka.filters.supervised.instance. StratifiedRemoveFolds 
 Gets the random number seed used for shuffling the dataset.
   getSeed()   - 
Method in class weka.filters.unsupervised.attribute. PropositionalToMultiInstance 
 Returns the current seed value for randomizing the order of the generated
 data
   getSeed()   - 
Method in class weka.filters.unsupervised.attribute. RandomSubset 
 Get the seed value for the random number generator.
   getSeed()   - 
Method in class weka.filters.unsupervised.instance. RemoveFolds 
 Gets the random number seed used for shuffling the dataset.
   getSeed()   - 
Method in class weka.gui.beans. CrossValidationFoldMaker 
 Get the currently set seed
   getSeed()   - 
Method in class weka.gui.beans. TrainTestSplitMaker 
 Get the value of the random seed
   getSelectedAttributes()   - 
Method in class weka.gui. AttributeSelectionPanel 
 Gets an array containing the indices of all selected attributes.
   getSelectedBuffer()   - 
Method in class weka.gui. ResultHistoryPanel 
 Gets the buffer associated with the currently
 selected item in the list.
   getSelectedName()   - 
Method in class weka.gui. ResultHistoryPanel 
 Get the name of the currently selected item in the list
   getSelectedObject()   - 
Method in class weka.gui. ResultHistoryPanel 
 Gets the object associated with the currently
 selected item in the list.
   getSelectedRange()   - 
Method in class weka.filters.unsupervised.attribute. RELAGGS 
 Gets the current range selection.
   getSelectedRange()   - 
Method in class weka.filters.unsupervised.attribute. StringToWordVector 
 Get the value of m_SelectedRange.
   getSelectedTag()   - 
Method in class weka.core. SelectedTag 
 Gets the selected Tag.
   getSelection()   - 
Method in class weka.core. Range 
 Gets an array containing all the selected values, in the order
 that they were selected (or ascending order if range inversion is on)
   getSelectionModel()   - 
Method in class weka.gui. AttributeListPanel 
 Gets the selection model used by the table.
   getSelectionModel()   - 
Method in class weka.gui. AttributeSelectionPanel 
 Gets the selection model used by the table.
   getSelectionModel()   - 
Method in class weka.gui. ResultHistoryPanel 
 Gets the selection model used by the results list.
   getSelectionThreshold()   - 
Method in class weka.attributeSelection. RaceSearch 
 Returns the threshold so that the AttributeSelection module can
 discard attributes from the ranking.
   getSeparatingThreshold()   - 
Method in class weka.classifiers.functions.pace. ChisqMixture 
 Gets the separating threshold value.
   getSeparatingThreshold()   - 
Method in class weka.classifiers.functions.pace. NormalMixture 
 Gets the separating threshold value.
   getSeperator()   - 
Method in class weka.gui. HierarchyPropertyParser 
 Get the seperator between levels.
   getSequentialAttIndex(int)   - 
Method in class weka.classifiers.lazy. LBR.Indexes 
 Returns the boolean value at the specified index in the Sequential Attribute Indexes array
   getSequentialInstanceIndex(int)   - 
Method in class weka.classifiers.lazy. LBR.Indexes 
 Returns the boolean value at the specified index in the Sequential Instance Indexes array
   getSequentialNumAttributes()   - 
Method in class weka.classifiers.lazy. LBR.Indexes 
 Returns the number of attributes in the Sequential array
   getSequentialNumInstances()   - 
Method in class weka.classifiers.lazy. LBR.Indexes 
 Returns the number of instances in the Sequential array
   getSerializedClassifierFile()   - 
Method in class weka.filters.supervised.attribute. AddClassification 
 Gets the file pointing to a serialized, trained classifier.
   getSERObject()   - 
Method in class weka.clusterers. OPTICS 
 Returns the internal database
   getSetNumber()   - 
Method in class weka.gui.beans. BatchClassifierEvent 
 Get the set number (ie which fold this is)
   getSetNumber()   - 
Method in class weka.gui.beans. BatchClustererEvent 
 Get the set number (ie which fold this is)
   getSetNumber()   - 
Method in class weka.gui.beans. TestSetEvent 
 Get the test set number (eg.
   getSetNumber()   - 
Method in class weka.gui.beans. TrainingSetEvent 
 Get the set number (eg.
   getShape()   - 
Method in class weka.gui.treevisualizer. Node 
 Get the value of shape.
   getShowAttBars()   - 
Method in class weka.gui.visualize. VisualizePanel 
 Gets whether or not attribute bars are being displayed.
   getShowAverage()   - 
Method in class weka.experiment. ResultMatrix 
 returns whether average per column is displayed or not
   getShowAverage()   - 
Static method in class weka.gui.experiment. ExperimenterDefaults 
 returns whether the Average is shown by default
   getShowAverage()   - 
Method in class weka.gui.experiment. OutputFormatDialog 
 returns whether the average for each column is displayed.
   getShowClassPanel()   - 
Method in class weka.gui.visualize. VisualizePanel 
 Gets whether or not the class panel is being displayed.
   getShowGUI()   - 
Method in class weka.clusterers. OPTICS 
 Returns the flag for showing the OPTICS visualizer GUI.
   getShowStdDev()   - 
Method in class weka.experiment. ResultMatrix 
 returns whether std deviations are displayed or not
   getShowStdDevs()   - 
Method in class weka.experiment. PairedTTester 
 Returns true if standard deviations have been requested.
   getShowStdDevs()   - 
Method in interface weka.experiment. Tester 
 Returns true if standard deviations have been requested.
   getShowStdDevs()   - 
Static method in class weka.gui.experiment. ExperimenterDefaults 
 returns whether StdDevs are shown by default
   getShrinkage()   - 
Method in class weka.classifiers.meta. AdditiveRegression 
 Get the shrinkage rate.
   getShrinkage()   - 
Method in class weka.classifiers.meta. LogitBoost 
 Get the value of Shrinkage.
   getShrinking()   - 
Method in class weka.classifiers.functions. LibSVM 
 whether to use the shrinking heuristics
   getShuffle()   - 
Method in class weka.classifiers.rules. Ridor 
  
   getSigma()   - 
Method in class weka.attributeSelection. ReliefFAttributeEval 
 Get the value of sigma.
   getSigma()   - 
Method in class weka.classifiers. BVDecompose 
 Get the calculated sigma squared
   getSigma()   - 
Method in class weka.classifiers.functions.supportVector. Puk 
 Gets the sigma value.
   getSignificance(int, int)   - 
Method in class weka.experiment. ResultMatrix 
 returns the significance at the given position, if the position is valid,
 otherwise SIGNIFICANCE_ATIE
   getSignificance()   - 
Static method in class weka.gui.experiment. ExperimenterDefaults 
 returns the default significance
   getSignificanceCount(int, int)   - 
Method in class weka.experiment. ResultMatrix 
 counts the occurrences of the given significance type in the given
 column.
   getSignificanceLevel()   - 
Method in class weka.associations. Apriori 
 Get the value of significanceLevel.
   getSignificanceLevel()   - 
Method in class weka.attributeSelection. RaceSearch 
 Get the significance level
   getSignificanceLevel()   - 
Method in class weka.experiment. PairedTTester 
 Get the value of SignificanceLevel.
   getSignificanceLevel()   - 
Method in interface weka.experiment. Tester 
 Get the value of SignificanceLevel.
   getSignificanceWidth()   - 
Method in class weka.experiment. ResultMatrix 
 returns the current width for the significance
   getSilent()   - 
Method in class weka.core. Check 
 Get whether silent mode is turned on
   getSilent()   - 
Method in class weka.core. Javadoc 
 whether output in the console is suppressed
   getSilent()   - 
Method in class weka.estimators. CheckEstimator 
 Get whether silent mode is turned on
   getSimpleStats(int)   - 
Method in class weka.classifiers.rules. RuleStats 
 Get the simple stats of one rule, including 6 parameters:
 0: coverage; 1:uncoverage; 2: true positive; 3: true negatives;
 4: false positives; 5: false negatives
   getSIndex()   - 
Method in class weka.gui.visualize. VisualizePanel 
 Get the index of the shape selected for creating splits.
   getSingleIndex()   - 
Method in class weka.core. SingleIndex 
 Gets the string representing the selected range of values
   getSingleModeFlag()   - 
Method in class weka.datagenerators.classifiers.classification. Agrawal 
 Return if single mode is set for the given data generator
 mode depends on option setting and or generator type.
   getSingleModeFlag()   - 
Method in class weka.datagenerators.classifiers.classification. BayesNet 
 Return if single mode is set for the given data generator
 mode depends on option setting and or generator type.
   getSingleModeFlag()   - 
Method in class weka.datagenerators.classifiers.classification. LED24 
 Return if single mode is set for the given data generator
 mode depends on option setting and or generator type.
   getSingleModeFlag()   - 
Method in class weka.datagenerators.classifiers.classification. RandomRBF 
 Return if single mode is set for the given data generator
 mode depends on option setting and or generator type.
   getSingleModeFlag()   - 
Method in class weka.datagenerators.classifiers.classification. RDG1 
 Gets the single mode flag.
   getSingleModeFlag()   - 
Method in class weka.datagenerators.classifiers.regression. Expression 
 Return if single mode is set for the given data generator
 mode depends on option setting and or generator type.
   getSingleModeFlag()   - 
Method in class weka.datagenerators.classifiers.regression. MexicanHat 
 Return if single mode is set for the given data generator
 mode depends on option setting and or generator type.
   getSingleModeFlag()   - 
Method in class weka.datagenerators.clusterers. BIRCHCluster 
 Gets the single mode flag.
   getSingleModeFlag()   - 
Method in class weka.datagenerators.clusterers. SubspaceCluster 
 Gets the single mode flag.
   getSingleModeFlag()   - 
Method in class weka.datagenerators. DataGenerator 
 Return if single mode is set for the given data generator
 mode depends on option setting and or generator type.
   getSingleton()   - 
Static method in class weka.core.logging. Logger 
 Returns the singleton instance of the logger.
   getSingleton()   - 
Static method in class weka.gui.beans. KnowledgeFlowApp 
 Return the singleton instance of the KnowledgeFlow
   getSingleton()   - 
Static method in class weka.gui. GUIChooser 
 Get the singleton instance of the GUIChooser
   getSingleton()   - 
Static method in class weka.gui. Main 
 Return the singleton instance of the Main GUI.
   getSingularValues()   - 
Method in class weka.core.matrix. SingularValueDecomposition 
 Return the one-dimensional array of singular values
   getSize()   - 
Method in class weka.core. Debug.Log 
 returns the size of the files
   getSizePer()   - 
Method in class weka.classifiers.trees. BFTree 
 Get training set size.
   getSizePer()   - 
Method in class weka.classifiers.trees. SimpleCart 
 Get training set size.
   getSkipIdentical()   - 
Method in class weka.core.neighboursearch. LinearNNSearch 
 Gets whether if identical instances are skipped from the neighbourhood.
   getSlope()   - 
Method in class weka.classifiers.functions. SimpleLinearRegression 
 Returns the slope of the function.
   getSmoothing()   - 
Method in class weka.classifiers.trees.m5. Rule 
 Get whether or not smoothing has been turned on
   getSmoothingParameter()   - 
Method in class weka.classifiers.bayes. ComplementNaiveBayes 
 Gets the smoothing value to be used to avoid zero WordGivenClass
 probabilities.
   getSort()   - 
Method in class weka.filters.unsupervised.attribute. AddValues 
 Gets whether the labels are sorted or not.
   getSortColumn()   - 
Method in class weka.experiment. PairedTTester 
 Returns the column to sort on, -1 means the default sorting.
   getSortColumn()   - 
Method in interface weka.experiment. Tester 
 Returns the column to sort on, -1 means the default sorting.
   getSortColumnName()   - 
Method in class weka.experiment. PairedTTester 
 Returns the name of the column to sort on.
   getSortColumnName()   - 
Method in interface weka.experiment. Tester 
 Returns the name of the column to sort on.
   getSorting()   - 
Static method in class weka.gui.experiment. ExperimenterDefaults 
 returns the default sorting (empty string means none)
   getSource()   - 
Method in class weka.gui.beans. BeanConnection 
 returns the source BeanInstance for this connection
   getSource()   - 
Method in class weka.gui.treevisualizer. Edge 
 Get the value of source.
   getSourceCode()   - 
Method in class weka.classifiers. CheckSource 
 Gets the class to test.
   getSourceCode()   - 
Method in class weka.filters. CheckSource 
 Gets the class to test.
   getSparseData()   - 
Method in class weka.experiment. InstanceQuery 
 Gets whether data is to be returned as a set of sparse instances
   getSplitByDataSet()   - 
Method in class weka.experiment. RemoteExperiment 
 Returns true if sub experiments are to be created on the basis of
 data set..
   getSplitDim()   - 
Method in class weka.core.neighboursearch.kdtrees. KDTreeNode 
 Gets the splitting dimension.
   getSplitEvaluator()   - 
Method in class weka.experiment. CrossValidationResultProducer 
 Get the SplitEvaluator.
   getSplitEvaluator()   - 
Method in class weka.experiment. RandomSplitResultProducer 
 Get the SplitEvaluator.
   getSplitOnResiduals()   - 
Method in class weka.classifiers.trees. LMT 
 Get the value of splitOnResiduals.
   getSplitPoint()   - 
Method in class weka.filters.unsupervised.instance. RemoveWithValues 
 Get the split point used for numeric selection
   getSplitValue()   - 
Method in class weka.core.neighboursearch.kdtrees. KDTreeNode 
 Gets the splitting value.
   getSquaredError()   - 
Method in class weka.clusterers. SimpleKMeans 
 Gets the squared error for all clusters
   getStamp()   - 
Method in class weka.core. Debug.Timestamp 
 returns the associated date/time
   getStandardDeviation(Instance)   - 
Method in class weka.classifiers.functions. GaussianProcesses 
 Gives the variance of the prediction at the given instance
   getStart()   - 
Method in class weka.core. Debug.Clock 
 returns the start time
   getStartMessage()   - 
Method in class weka.gui.beans. Loader 
 Gets a string that describes the start action.
   getStartMessage()   - 
Method in interface weka.gui.beans. Startable 
 Gets a string that describes the start action.
   getStartPoint()   - 
Method in class weka.attributeSelection. RankSearch 
 Get the point at which to start evaluating the ranking
   getStartSequentially()   - 
Method in class weka.gui.beans. FlowRunner 
 Gets whether Startable beans will be launched sequentially
 or all in parallel.
   getStartSet()   - 
Method in class weka.attributeSelection. BestFirst 
 Returns a list of attributes (and or attribute ranges) as a String
   getStartSet()   - 
Method in class weka.attributeSelection. GeneticSearch 
 Returns a list of attributes (and or attribute ranges) as a String
   getStartSet()   - 
Method in class weka.attributeSelection. GreedyStepwise 
 Returns a list of attributes (and or attribute ranges) as a String
   getStartSet()   - 
Method in class weka.attributeSelection. LinearForwardSelection 
 Returns a list of attributes (and or attribute ranges) as a String
   getStartSet()   - 
Method in class weka.attributeSelection. RandomSearch 
 Returns a list of attributes (and or attribute ranges) as a String
   getStartSet()   - 
Method in class weka.attributeSelection. Ranker 
 Returns a list of attributes (and or attribute ranges) as a String
   getStartSet()   - 
Method in interface weka.attributeSelection. StartSetHandler 
 Returns a list of attributes (and or attribute ranges) as a String
   getStaticIcon()   - 
Method in class weka.gui.beans. BeanVisual 
 Returns the static icon
   getStats()   - 
Method in class weka.core.neighboursearch. PerformanceStats 
 Returns a string representation of the statistics.
   getStats()   - 
Method in class weka.core.neighboursearch. TreePerformanceStats 
 Returns a string representation of the statistics.
   getStatus()   - 
Method in class weka.gui.beans. IncrementalClassifierEvent 
 Get the status
   getStatus()   - 
Method in class weka.gui.beans. InstanceEvent 
 Get the status
   getStatusFrequency()   - 
Method in class weka.gui.beans. IncrementalClassifierEvaluator 
 Get how often progress is reported to the status bar.
   getStatusMessage()   - 
Method in class weka.experiment. TaskStatusInfo 
 Get the status message.
   getStatusTable()   - 
Method in class weka.gui.beans. LogPanel 
 The JTable used for the status messages (in case clients
 want to attach listeners etc.)
   getStdDev()   - 
Method in class weka.estimators. KernelEstimator 
 Return the standard deviation of this kernel estimator.
   getStdDev()   - 
Method in class weka.estimators. NormalEstimator 
 Return the value of the standard deviation of this normal estimator.
   getStdDev(int, int)   - 
Method in class weka.experiment. ResultMatrix 
 returns the std deviation at the given position, if the position is valid,
 otherwise 0
   getStdDevCoordsPerPoint()   - 
Method in class weka.core.neighboursearch. PerformanceStats 
 Returns the standard deviation of coords per point.
   getStdDevIntNodesVisited()   - 
Method in class weka.core.neighboursearch. TreePerformanceStats 
 Returns the standard deviation of internal nodes visited.
   getStdDevLeavesVisited()   - 
Method in class weka.core.neighboursearch. TreePerformanceStats 
 Returns the standard deviation of leaves visited.
   getStdDevPointsVisited()   - 
Method in class weka.core.neighboursearch. PerformanceStats 
 Returns the standard deviation of points visited.
   getStdDevPrec()   - 
Method in class weka.experiment. ResultMatrix 
 returns the current standard deviation precision
   getStdDevPrec()   - 
Method in class weka.gui.experiment. OutputFormatDialog 
 Gets the precision used for printing the std.
   getStdDevPrecision()   - 
Static method in class weka.gui.experiment. ExperimenterDefaults 
 returns the default precision for the stddevs
   getStddevValue()   - 
Method in class weka.datagenerators.clusterers. SubspaceClusterDefinition 
  
   getStdDevWidth()   - 
Method in class weka.experiment. ResultMatrix 
 returns the current width for the std dev
   getStemmer()   - 
Method in class weka.core.stemmers. SnowballStemmer 
 returns the name of the current stemmer, null if none is set.
   getStemmer()   - 
Method in class weka.filters.unsupervised.attribute. StringToWordVector 
 Returns the current stemming algorithm, null if none is used.
   getStepSize()   - 
Method in class weka.attributeSelection. RankSearch 
 Get the number of attributes to add from the rankining
 in each iteration
   getStepSize()   - 
Method in class weka.experiment. LearningRateResultProducer 
 Get the value of StepSize.
   getStop()   - 
Method in class weka.core. Debug.Clock 
 returns the stop time or, if still running, the current time
   getStopwords()   - 
Method in class weka.filters.unsupervised.attribute. StringToWordVector 
 returns the file used for obtaining the stopwords, if the file represents
 a directory then the default ones are used.
   getString(String)   - 
Static method in class weka.associations.gsp. Messages 
 getString.
   getString(String, Locale)   - 
Static method in class weka.associations.gsp. Messages 
 getString.
   getString(String)   - 
Static method in class weka.associations. Messages 
 getString.
   getString(String, Locale)   - 
Static method in class weka.associations. Messages 
 getString.
   getString(int[])   - 
Method in class weka.classifiers.meta.nestedDichotomies. ClassBalancedND 
 Returns the list of indices as a string.
   getString(int[])   - 
Method in class weka.classifiers.meta.nestedDichotomies. DataNearBalancedND 
 Returns the list of indices as a string.
   getString()   - 
Method in class weka.core. Trie.TrieNode 
 returns the full string up to the root
   getString(String)   - 
Static method in class weka.gui.arffviewer. Messages 
 getString.
   getString(String, Locale)   - 
Static method in class weka.gui.arffviewer. Messages 
 getString.
   getString(String)   - 
Static method in class weka.gui.beans. Messages 
 getString.
   getString(String, Locale)   - 
Static method in class weka.gui.beans. Messages 
 getString.
   getString(String)   - 
Static method in class weka.gui.beans.xml. Messages 
 getString.
   getString(String, Locale)   - 
Static method in class weka.gui.beans.xml. Messages 
 getString.
   getString(String)   - 
Static method in class weka.gui.boundaryvisualizer. Messages 
 getString.
   getString(String, Locale)   - 
Static method in class weka.gui.boundaryvisualizer. Messages 
 getString.
   getString(String)   - 
Static method in class weka.gui.experiment. Messages 
 getString.
   getString(String, Locale)   - 
Static method in class weka.gui.experiment. Messages 
 getString.
   getString(String)   - 
Static method in class weka.gui.explorer. Messages 
 getString.
   getString(String, Locale)   - 
Static method in class weka.gui.explorer. Messages 
 getString.
   getString(String)   - 
Static method in class weka.gui.graphvisualizer. Messages 
 getString.
   getString(String, Locale)   - 
Static method in class weka.gui.graphvisualizer. Messages 
 getString.
   getString(String)   - 
Static method in class weka.gui.hierarchyvisualizer. Messages 
 getString.
   getString(String, Locale)   - 
Static method in class weka.gui.hierarchyvisualizer. Messages 
 getString.
   getString(String)   - 
Static method in class weka.gui. Messages 
 getString.
   getString(String, Locale)   - 
Static method in class weka.gui. Messages 
 getString.
   getString(String)   - 
Static method in class weka.gui.sql.event. Messages 
 getString.
   getString(String, Locale)   - 
Static method in class weka.gui.sql.event. Messages 
 getString.
   getString(String)   - 
Static method in class weka.gui.sql. Messages 
 getString.
   getString(String, Locale)   - 
Static method in class weka.gui.sql. Messages 
 getString.
   getString(String)   - 
Static method in class weka.gui.streams. Messages 
 getString.
   getString(String, Locale)   - 
Static method in class weka.gui.streams. Messages 
 getString.
   getString(String)   - 
Static method in class weka.gui.treevisualizer. Messages 
 getString.
   getString(String, Locale)   - 
Static method in class weka.gui.treevisualizer. Messages 
 getString.
   getString(String)   - 
Static method in class weka.gui.visualize. Messages 
 getString.
   getString(String, Locale)   - 
Static method in class weka.gui.visualize. Messages 
 getString.
   getStringAttributes()   - 
Method in class weka.core.converters. CSVLoader 
 Returns the current attribute range to be forced to type string.
   getStringSelection()   - 
Method in class weka.gui.arffviewer. ArffTable 
 returns the selected content in a StringSelection that can be copied to
 the clipboard and used in Excel, if nothing is selected the whole table
 is copied to the clipboard
   getStroke()   - 
Method in class weka.gui.visualize. PostscriptGraphics 
  
   getStructure()   - 
Method in class weka.core.converters. AbstractLoader 
  
   getStructure()   - 
Method in class weka.core.converters. ArffLoader.ArffReader 
 Returns the header format
   getStructure()   - 
Method in class weka.core.converters. ArffLoader 
 Determines and returns (if possible) the structure (internally the
 header) of the data set as an empty set of instances.
   getStructure()   - 
Method in class weka.core.converters. C45Loader 
 Determines and returns (if possible) the structure (internally the 
 header) of the data set as an empty set of instances.
   getStructure()   - 
Method in class weka.core.converters. ConverterUtils.DataSource 
 returns the structure of the data.
   getStructure(int)   - 
Method in class weka.core.converters. ConverterUtils.DataSource 
 returns the structure of the data, with the defined class index.
   getStructure()   - 
Method in class weka.core.converters. CSVLoader 
 Determines and returns (if possible) the structure (internally the 
 header) of the data set as an empty set of instances.
   getStructure()   - 
Method in class weka.core.converters. DatabaseLoader 
 Determines and returns (if possible) the structure (internally the 
 header) of the data set as an empty set of instances.
   getStructure()   - 
Method in class weka.core.converters. LibSVMLoader 
 Determines and returns (if possible) the structure (internally the 
 header) of the data set as an empty set of instances.
   getStructure()   - 
Method in interface weka.core.converters. Loader 
 Determines and returns (if possible) the structure (internally the 
 header) of the data set as an empty set of instances.
   getStructure()   - 
Method in class weka.core.converters. SerializedInstancesLoader 
 Determines and returns (if possible) the structure (internally the 
 header) of the data set as an empty set of instances.
   getStructure()   - 
Method in class weka.core.converters. SVMLightLoader 
 Determines and returns (if possible) the structure (internally the 
 header) of the data set as an empty set of instances.
   getStructure()   - 
Method in class weka.core.converters. TextDirectoryLoader 
 Determines and returns (if possible) the structure (internally the 
 header) of the data set as an empty set of instances.
   getStructure()   - 
Method in class weka.core.converters. XRFFLoader 
 Determines and returns (if possible) the structure (internally the 
 header) of the data set as an empty set of instances.
   getStructure(String)   - 
Method in class weka.gui.beans. ClassAssigner 
 Get the structure of the output encapsulated in the named
 event.
   getStructure(String)   - 
Method in class weka.gui.beans. ClassValuePicker 
  
   getStructure()   - 
Method in class weka.gui.beans. IncrementalClassifierEvent 
 Get the instances structure (may be null if this is not
 a NEW_BATCH event)
   getStructure()   - 
Method in class weka.gui.beans. InstanceEvent 
 Get the instances structure (may be null if this is not
 a FORMAT_AVAILABLE event)
   getStructure(String)   - 
Method in class weka.gui.beans. Loader 
 Get the structure of the output encapsulated in the named
 event.
   getStructure(String)   - 
Method in interface weka.gui.beans. StructureProducer 
 Get the structure of the output encapsulated in the named
 event.
   getSubFlow()   - 
Method in class weka.gui.beans. MetaBean 
  
   getSubmenuTitle()   - 
Method in interface weka.gui. MainMenuExtension 
 Returns the name of the submenu.
   getSubsequenceLength()   - 
Method in class weka.classifiers.functions.supportVector. StringKernel 
 Returns the length of the subsequence
   getSubsetEvaluator()   - 
Method in class weka.attributeSelection. FilteredSubsetEval 
 Get the subset evaluator to use
   getSubsetSizeEvaluator()   - 
Method in class weka.attributeSelection. SubsetSizeForwardSelection 
 Get the subset evaluator used for subset size determination.
   getSubSpaceSize()   - 
Method in class weka.classifiers.meta. RandomSubSpace 
 Gets the size of each subSpace, as a percentage of the training set size.
   getSubtreeRaising()   - 
Method in class weka.classifiers.trees. J48 
 Get the value of subtreeRaising.
   getSubtreeRaising()   - 
Method in class weka.classifiers.trees. J48graft 
 Get the value of subtreeRaising.
   getSuccess()   - 
Method in class weka.core. CheckGOE 
 returns the success of the tests
   getSuccess()   - 
Method in class weka.core. CheckOptionHandler 
 returns the success of the tests
   getSuitableTargets(EventSetDescriptor)   - 
Method in class weka.gui.beans. MetaBean 
 Return a list of input beans capable of receiving the 
 supplied event
   getSummary()   - 
Method in class weka.gui. SetInstancesPanel 
 Gets the instances summary panel associated with
 this panel
   getSumOfCounts()   - 
Method in class weka.estimators. DiscreteEstimator 
 Get the sum of all the counts
   getSumOfWeights()   - 
Method in class weka.estimators. NormalEstimator 
 Return the sum of the weights for this normal estimator.
   getSupportedCursorScrollType()   - 
Method in class weka.experiment. DatabaseUtils 
 Returns the type of scrolling that the cursor supports, -1 if not
 supported or not connected.
   getSVMType()   - 
Method in class weka.classifiers.functions. LibLINEAR 
 Gets type of SVM
   getSVMType()   - 
Method in class weka.classifiers.functions. LibSVM 
 Gets type of SVM
   getSymbols()   - 
Method in class weka.core.mathematicalexpression. Parser 
 Returns the current variable - value relation in use.
   getSymbols()   - 
Method in class weka.filters.unsupervised.instance.subsetbyexpression. Parser 
 Returns the current variable - value relation in use.
   getSystemInfo()   - 
Method in class weka.core. SystemInfo 
 returns a copy of the system info.
   getSystemLookAndFeel()   - 
Static method in class weka.gui. LookAndFeel 
 returns the system LnF classname
   getSystemWide()   - 
Static method in class weka.core. Environment 
 Get the singleton system-wide (visible to every
 class in the running VM) set of environment
 variables.
   getTabbedPane()   - 
Method in class weka.gui.arffviewer. ArffViewerMainPanel 
 returns the tabbedpane instance
   getTabbedPane()   - 
Method in class weka.gui.explorer. Explorer 
 returns the tabbed pane of the Explorer
   getTable()   - 
Method in class weka.gui.arffviewer. ArffPanel 
 returns the table component
   getTableCellRendererComponent(JTable, Object, boolean, boolean, int, int)   - 
Method in class weka.gui.arffviewer. ArffTableCellRenderer 
 Returns the default table cell renderer.
   getTableCellRendererComponent(JTable, Object, boolean, boolean, int, int)   - 
Method in class weka.gui.sql. ResultSetTableCellRenderer 
 Returns the default table cell renderer.
   getTableModel()   - 
Method in class weka.gui. AttributeSelectionPanel 
 Get the table model in use (or null if no instances
 have been set yet).
   getTableName()   - 
Method in class weka.core.converters. DatabaseSaver 
 Gets the table's name.
   getTabs()   - 
Static method in class weka.gui.explorer. ExplorerDefaults 
 returns an array with the classnames of all the additional panels to 
 display as tabs in the Explorer.
   getTabTitle()   - 
Method in class weka.gui.explorer. AssociationsPanel 
 Returns the title for the tab in the Explorer
   getTabTitle()   - 
Method in class weka.gui.explorer. AttributeSelectionPanel 
 Returns the title for the tab in the Explorer
   getTabTitle()   - 
Method in class weka.gui.explorer. ClassifierPanel 
 Returns the title for the tab in the Explorer
   getTabTitle()   - 
Method in class weka.gui.explorer. ClustererPanel 
 Returns the title for the tab in the Explorer
   getTabTitle()   - 
Method in interface weka.gui.explorer. Explorer.ExplorerPanel 
 Returns the title for the tab in the Explorer
   getTabTitle()   - 
Method in class weka.gui.explorer. PreprocessPanel 
 Returns the title for the tab in the Explorer
   getTabTitle()   - 
Method in class weka.gui.explorer. VisualizePanel 
 Returns the title for the tab in the Explorer
   getTabTitleToolTip()   - 
Method in class weka.gui.explorer. AssociationsPanel 
 Returns the tooltip for the tab in the Explorer
   getTabTitleToolTip()   - 
Method in class weka.gui.explorer. AttributeSelectionPanel 
 Returns the tooltip for the tab in the Explorer
   getTabTitleToolTip()   - 
Method in class weka.gui.explorer. ClassifierPanel 
 Returns the tooltip for the tab in the Explorer
   getTabTitleToolTip()   - 
Method in class weka.gui.explorer. ClustererPanel 
 Returns the tooltip for the tab in the Explorer
   getTabTitleToolTip()   - 
Method in interface weka.gui.explorer. Explorer.ExplorerPanel 
 Returns the tooltip for the tab in the Explorer
   getTabTitleToolTip()   - 
Method in class weka.gui.explorer. PreprocessPanel 
 Returns the tooltip for the tab in the Explorer
   getTabTitleToolTip()   - 
Method in class weka.gui.explorer. VisualizePanel 
 Returns the tooltip for the tab in the Explorer
   getTabuList()   - 
Method in class weka.classifiers.bayes.net.search.global. TabuSearch 
  
   getTabuList()   - 
Method in class weka.classifiers.bayes.net.search.local. TabuSearch 
  
   getTags()   - 
Method in class weka.core. SelectedTag 
 Gets the set of all valid Tags.
   getTags()   - 
Method in class weka.gui. CostMatrixEditor 
 Some objects can return tags, but a cost matrix cannot.
   getTags()   - 
Method in class weka.gui. GenericArrayEditor 
 Returns null as we don't support getting values as tags.
   getTags()   - 
Method in class weka.gui. GenericObjectEditor 
 Returns null as we don't support getting values as tags.
   getTags()   - 
Method in class weka.gui. SelectedTagEditor 
 Gets the list of tags that can be selected from.
   getTags()   - 
Method in class weka.gui. SimpleDateFormatEditor 
 Some objects can return tags, but a date format cannot.
   getTarget()   - 
Method in class weka.gui.beans. BeanConnection 
 Returns the target BeanInstance for this connection
   getTarget()   - 
Method in class weka.gui.treevisualizer. Edge 
 Get the value of target.
   getTargetClass()   - 
Method in class weka.classifiers.bayes. DMNBtext.DNBBinary 
 Gets the Target Class
   getTargetMetaData()   - 
Method in class weka.core.pmml. MiningSchema 
 Get the Target meta data.
   getTaskResult()   - 
Method in class weka.experiment. TaskStatusInfo 
 Get the returnable result of this task.
   getTaskStatus()   - 
Method in class weka.experiment. RemoteExperimentSubTask 
  
   getTaskStatus()   - 
Method in interface weka.experiment. Task 
 Clients should be able to call this method at any time to obtain
 information on a current task.
   getTaskStatus()   - 
Method in class weka.gui.boundaryvisualizer. RemoteBoundaryVisualizerSubTask 
 Return status information for this sub task
   getTechnicalInformation()   - 
Method in class weka.associations. Apriori 
 Returns an instance of a TechnicalInformation object, containing 
 detailed information about the technical background of this class,
 e.g., paper reference or book this class is based on.
   getTechnicalInformation()   - 
Method in class weka.associations. FPGrowth 
 Returns an instance of a TechnicalInformation object, containing 
 detailed information about the technical background of this class,
 e.g., paper reference or book this class is based on.
   getTechnicalInformation()   - 
Method in class weka.associations. GeneralizedSequentialPatterns 
 Returns TechnicalInformation about the paper related to the algorithm.
   getTechnicalInformation()   - 
Method in class weka.associations. PredictiveApriori 
 Returns an instance of a TechnicalInformation object, containing 
 detailed information about the technical background of this class,
 e.g., paper reference or book this class is based on.
   getTechnicalInformation()   - 
Method in class weka.associations. Tertius 
 Returns an instance of a TechnicalInformation object, containing 
 detailed information about the technical background of this class,
 e.g., paper reference or book this class is based on.
   getTechnicalInformation()   - 
Method in class weka.attributeSelection. CfsSubsetEval 
 Returns an instance of a TechnicalInformation object, containing 
 detailed information about the technical background of this class,
 e.g., paper reference or book this class is based on.
   getTechnicalInformation()   - 
Method in class weka.attributeSelection. ConsistencySubsetEval 
 Returns an instance of a TechnicalInformation object, containing 
 detailed information about the technical background of this class,
 e.g., paper reference or book this class is based on.
   getTechnicalInformation()   - 
Method in class weka.attributeSelection. GeneticSearch 
 Returns an instance of a TechnicalInformation object, containing 
 detailed information about the technical background of this class,
 e.g., paper reference or book this class is based on.
   getTechnicalInformation()   - 
Method in class weka.attributeSelection. LinearForwardSelection 
 Returns an instance of a TechnicalInformation object, containing 
 detailed information about the technical background of this class,
 e.g., paper reference or book this class is based on.
   getTechnicalInformation()   - 
Method in class weka.attributeSelection. RaceSearch 
 Returns an instance of a TechnicalInformation object, containing 
 detailed information about the technical background of this class,
 e.g., paper reference or book this class is based on.
   getTechnicalInformation()   - 
Method in class weka.attributeSelection. RandomSearch 
 Returns an instance of a TechnicalInformation object, containing 
 detailed information about the technical background of this class,
 e.g., paper reference or book this class is based on.
   getTechnicalInformation()   - 
Method in class weka.attributeSelection. RankSearch 
 Returns an instance of a TechnicalInformation object, containing 
 detailed information about the technical background of this class,
 e.g., paper reference or book this class is based on.
   getTechnicalInformation()   - 
Method in class weka.attributeSelection. ReliefFAttributeEval 
 Returns an instance of a TechnicalInformation object, containing 
 detailed information about the technical background of this class,
 e.g., paper reference or book this class is based on.
   getTechnicalInformation()   - 
Method in class weka.attributeSelection. ScatterSearchV1 
 Returns an instance of a TechnicalInformation object, containing
 detailed information about the technical background of this class,
 e.g., paper reference or book this class is based on.
   getTechnicalInformation()   - 
Method in class weka.attributeSelection. SubsetSizeForwardSelection 
 Returns an instance of a TechnicalInformation object, containing 
 detailed information about the technical background of this class,
 e.g., paper reference or book this class is based on.
   getTechnicalInformation()   - 
Method in class weka.attributeSelection. SVMAttributeEval 
 Returns an instance of a TechnicalInformation object, containing 
 detailed information about the technical background of this class,
 e.g., paper reference or book this class is based on.
   getTechnicalInformation()   - 
Method in class weka.attributeSelection. WrapperSubsetEval 
 Returns an instance of a TechnicalInformation object, containing 
 detailed information about the technical background of this class,
 e.g., paper reference or book this class is based on.
   getTechnicalInformation()   - 
Method in class weka.classifiers.bayes. AODE 
 Returns an instance of a TechnicalInformation object, containing 
 detailed information about the technical background of this class,
 e.g., paper reference or book this class is based on.
   getTechnicalInformation()   - 
Method in class weka.classifiers.bayes. AODEsr 
 Returns an instance of a TechnicalInformation object, containing
 detailed information about the technical background of this class,
 e.g., paper reference or book this class is based on.
   getTechnicalInformation()   - 
Method in class weka.classifiers.bayes. BayesianLogisticRegression 
 Returns an instance of a TechnicalInformation object, containing
 detailed information about the technical background of this class,
 e.g., paper reference or book this class is based on.
   getTechnicalInformation()   - 
Method in class weka.classifiers.bayes. ComplementNaiveBayes 
 Returns an instance of a TechnicalInformation object, containing 
 detailed information about the technical background of this class,
 e.g., paper reference or book this class is based on.
   getTechnicalInformation()   - 
Method in class weka.classifiers.bayes. DMNBtext 
 Returns an instance of a TechnicalInformation object, containing
 detailed information about the technical background of this class,
 e.g., paper reference or book this class is based on.
   getTechnicalInformation()   - 
Method in class weka.classifiers.bayes. HNB 
 Returns an instance of a TechnicalInformation object, containing 
 detailed information about the technical background of this class,
 e.g., paper reference or book this class is based on.
   getTechnicalInformation()   - 
Method in class weka.classifiers.bayes. NaiveBayes 
 Returns an instance of a TechnicalInformation object, containing 
 detailed information about the technical background of this class,
 e.g., paper reference or book this class is based on.
   getTechnicalInformation()   - 
Method in class weka.classifiers.bayes. NaiveBayesMultinomial 
 Returns an instance of a TechnicalInformation object, containing 
 detailed information about the technical background of this class,
 e.g., paper reference or book this class is based on.
   getTechnicalInformation()   - 
Method in class weka.classifiers.bayes. NaiveBayesSimple 
 Returns an instance of a TechnicalInformation object, containing 
 detailed information about the technical background of this class,
 e.g., paper reference or book this class is based on.
   getTechnicalInformation()   - 
Method in class weka.classifiers.bayes. NaiveBayesUpdateable 
 Returns an instance of a TechnicalInformation object, containing 
 detailed information about the technical background of this class,
 e.g., paper reference or book this class is based on.
   getTechnicalInformation()   - 
Method in class weka.classifiers.bayes.net. ADNode 
 Returns an instance of a TechnicalInformation object, containing 
 detailed information about the technical background of this class,
 e.g., paper reference or book this class is based on.
   getTechnicalInformation()   - 
Method in class weka.classifiers.bayes.net. BIFReader 
 Returns an instance of a TechnicalInformation object, containing 
 detailed information about the technical background of this class,
 e.g., paper reference or book this class is based on.
   getTechnicalInformation()   - 
Method in class weka.classifiers.bayes.net.search.global. K2 
 Returns an instance of a TechnicalInformation object, containing 
 detailed information about the technical background of this class,
 e.g., paper reference or book this class is based on.
   getTechnicalInformation()   - 
Method in class weka.classifiers.bayes.net.search.global. SimulatedAnnealing 
 Returns an instance of a TechnicalInformation object, containing 
 detailed information about the technical background of this class,
 e.g., paper reference or book this class is based on.
   getTechnicalInformation()   - 
Method in class weka.classifiers.bayes.net.search.global. TabuSearch 
 Returns an instance of a TechnicalInformation object, containing 
 detailed information about the technical background of this class,
 e.g., paper reference or book this class is based on.
   getTechnicalInformation()   - 
Method in class weka.classifiers.bayes.net.search.global. TAN 
 Returns an instance of a TechnicalInformation object, containing 
 detailed information about the technical background of this class,
 e.g., paper reference or book this class is based on.
   getTechnicalInformation()   - 
Method in class weka.classifiers.bayes.net.search.local. K2 
 Returns an instance of a TechnicalInformation object, containing 
 detailed information about the technical background of this class,
 e.g., paper reference or book this class is based on.
   getTechnicalInformation()   - 
Method in class weka.classifiers.bayes.net.search.local. SimulatedAnnealing 
 Returns an instance of a TechnicalInformation object, containing 
 detailed information about the technical background of this class,
 e.g., paper reference or book this class is based on.
   getTechnicalInformation()   - 
Method in class weka.classifiers.bayes.net.search.local. TabuSearch 
 Returns an instance of a TechnicalInformation object, containing 
 detailed information about the technical background of this class,
 e.g., paper reference or book this class is based on.
   getTechnicalInformation()   - 
Method in class weka.classifiers.bayes.net.search.local. TAN 
 Returns an instance of a TechnicalInformation object, containing 
 detailed information about the technical background of this class,
 e.g., paper reference or book this class is based on.
   getTechnicalInformation()   - 
Method in class weka.classifiers.bayes. WAODE 
 Returns an instance of a TechnicalInformation object, containing 
 detailed information about the technical background of this class,
 e.g., paper reference or book this class is based on.
   getTechnicalInformation()   - 
Method in class weka.classifiers. BVDecompose 
 Returns an instance of a TechnicalInformation object, containing 
 detailed information about the technical background of this class,
 e.g., paper reference or book this class is based on.
   getTechnicalInformation()   - 
Method in class weka.classifiers. BVDecomposeSegCVSub 
 Returns an instance of a TechnicalInformation object, containing 
 detailed information about the technical background of this class,
 e.g., paper reference or book this class is based on.
   getTechnicalInformation()   - 
Method in class weka.classifiers.functions. GaussianProcesses 
 Returns an instance of a TechnicalInformation object, containing 
 detailed information about the technical background of this class,
 e.g., paper reference or book this class is based on.
   getTechnicalInformation()   - 
Method in class weka.classifiers.functions. LeastMedSq 
 Returns an instance of a TechnicalInformation object, containing 
 detailed information about the technical background of this class,
 e.g., paper reference or book this class is based on.
   getTechnicalInformation()   - 
Method in class weka.classifiers.functions. LibLINEAR 
 Returns an instance of a TechnicalInformation object, containing
 detailed information about the technical background of this class,
 e.g., paper reference or book this class is based on.
   getTechnicalInformation()   - 
Method in class weka.classifiers.functions. LibSVM 
 Returns an instance of a TechnicalInformation object, containing 
 detailed information about the technical background of this class,
 e.g., paper reference or book this class is based on.
   getTechnicalInformation()   - 
Method in class weka.classifiers.functions. Logistic 
 Returns an instance of a TechnicalInformation object, containing 
 detailed information about the technical background of this class,
 e.g., paper reference or book this class is based on.
   getTechnicalInformation()   - 
Method in class weka.classifiers.functions.pace. MixtureDistribution 
 Returns an instance of a TechnicalInformation object, containing 
 detailed information about the technical background of this class,
 e.g., paper reference or book this class is based on.
   getTechnicalInformation()   - 
Method in class weka.classifiers.functions. PaceRegression 
 Returns an instance of a TechnicalInformation object, containing 
 detailed information about the technical background of this class,
 e.g., paper reference or book this class is based on.
   getTechnicalInformation()   - 
Method in class weka.classifiers.functions. SimpleLogistic 
 Returns an instance of a TechnicalInformation object, containing 
 detailed information about the technical background of this class,
 e.g., paper reference or book this class is based on.
   getTechnicalInformation()   - 
Method in class weka.classifiers.functions. SMO 
 Returns an instance of a TechnicalInformation object, containing 
 detailed information about the technical background of this class,
 e.g., paper reference or book this class is based on.
   getTechnicalInformation()   - 
Method in class weka.classifiers.functions. SMOreg 
 Returns an instance of a TechnicalInformation object, containing 
 detailed information about the technical background of this class,
 e.g., paper reference or book this class is based on.
   getTechnicalInformation()   - 
Method in class weka.classifiers.functions. SPegasos 
 Returns an instance of a TechnicalInformation object, containing 
 detailed information about the technical background of this class,
 e.g., paper reference or book this class is based on.
   getTechnicalInformation()   - 
Method in class weka.classifiers.functions.supportVector. Puk 
 Returns an instance of a TechnicalInformation object, containing 
 detailed information about the technical background of this class,
 e.g., paper reference or book this class is based on.
   getTechnicalInformation()   - 
Method in class weka.classifiers.functions.supportVector. RegSMO 
 Returns an instance of a TechnicalInformation object, containing 
 detailed information about the technical background of this class,
 e.g., paper reference or book this class is based on.
   getTechnicalInformation()   - 
Method in class weka.classifiers.functions.supportVector. RegSMOImproved 
 Returns an instance of a TechnicalInformation object, containing 
 detailed information about the technical background of this class,
 e.g., paper reference or book this class is based on.
   getTechnicalInformation()   - 
Method in class weka.classifiers.functions.supportVector. StringKernel 
 Returns an instance of a TechnicalInformation object, containing 
 detailed information about the technical background of this class,
 e.g., paper reference or book this class is based on.
   getTechnicalInformation()   - 
Method in class weka.classifiers.functions. VotedPerceptron 
 Returns an instance of a TechnicalInformation object, containing 
 detailed information about the technical background of this class,
 e.g., paper reference or book this class is based on.
   getTechnicalInformation()   - 
Method in class weka.classifiers.functions. Winnow 
 Returns an instance of a TechnicalInformation object, containing 
 detailed information about the technical background of this class,
 e.g., paper reference or book this class is based on.
   getTechnicalInformation()   - 
Method in class weka.classifiers.lazy. IB1 
 Returns an instance of a TechnicalInformation object, containing 
 detailed information about the technical background of this class,
 e.g., paper reference or book this class is based on.
   getTechnicalInformation()   - 
Method in class weka.classifiers.lazy. IBk 
 Returns an instance of a TechnicalInformation object, containing 
 detailed information about the technical background of this class,
 e.g., paper reference or book this class is based on.
   getTechnicalInformation()   - 
Method in class weka.classifiers.lazy. KStar 
 Returns an instance of a TechnicalInformation object, containing 
 detailed information about the technical background of this class,
 e.g., paper reference or book this class is based on.
   getTechnicalInformation()   - 
Method in class weka.classifiers.lazy. LBR 
 Returns an instance of a TechnicalInformation object, containing 
 detailed information about the technical background of this class,
 e.g., paper reference or book this class is based on.
   getTechnicalInformation()   - 
Method in class weka.classifiers.lazy. LWL 
 Returns an instance of a TechnicalInformation object, containing 
 detailed information about the technical background of this class,
 e.g., paper reference or book this class is based on.
   getTechnicalInformation()   - 
Method in class weka.classifiers.meta. AdaBoostM1 
 Returns an instance of a TechnicalInformation object, containing 
 detailed information about the technical background of this class,
 e.g., paper reference or book this class is based on.
   getTechnicalInformation()   - 
Method in class weka.classifiers.meta. AdditiveRegression 
 Returns an instance of a TechnicalInformation object, containing 
 detailed information about the technical background of this class,
 e.g., paper reference or book this class is based on.
   getTechnicalInformation()   - 
Method in class weka.classifiers.meta. Bagging 
 Returns an instance of a TechnicalInformation object, containing 
 detailed information about the technical background of this class,
 e.g., paper reference or book this class is based on.
   getTechnicalInformation()   - 
Method in class weka.classifiers.meta. ClassificationViaRegression 
 Returns an instance of a TechnicalInformation object, containing 
 detailed information about the technical background of this class,
 e.g., paper reference or book this class is based on.
   getTechnicalInformation()   - 
Method in class weka.classifiers.meta. CVParameterSelection 
 Returns an instance of a TechnicalInformation object, containing 
 detailed information about the technical background of this class,
 e.g., paper reference or book this class is based on.
   getTechnicalInformation()   - 
Method in class weka.classifiers.meta. Dagging 
 Returns an instance of a TechnicalInformation object, containing 
 detailed information about the technical background of this class,
 e.g., paper reference or book this class is based on.
   getTechnicalInformation()   - 
Method in class weka.classifiers.meta. Decorate 
 Returns an instance of a TechnicalInformation object, containing 
 detailed information about the technical background of this class,
 e.g., paper reference or book this class is based on.
   getTechnicalInformation()   - 
Method in class weka.classifiers.meta. END 
 Returns an instance of a TechnicalInformation object, containing 
 detailed information about the technical background of this class,
 e.g., paper reference or book this class is based on.
   getTechnicalInformation()   - 
Method in class weka.classifiers.meta. Grading 
 Returns an instance of a TechnicalInformation object, containing 
 detailed information about the technical background of this class,
 e.g., paper reference or book this class is based on.
   getTechnicalInformation()   - 
Method in class weka.classifiers.meta. LogitBoost 
 Returns an instance of a TechnicalInformation object, containing 
 detailed information about the technical background of this class,
 e.g., paper reference or book this class is based on.
   getTechnicalInformation()   - 
Method in class weka.classifiers.meta. MetaCost 
 Returns an instance of a TechnicalInformation object, containing 
 detailed information about the technical background of this class,
 e.g., paper reference or book this class is based on.
   getTechnicalInformation()   - 
Method in class weka.classifiers.meta. MultiBoostAB 
 Returns an instance of a TechnicalInformation object, containing 
 detailed information about the technical background of this class,
 e.g., paper reference or book this class is based on.
   getTechnicalInformation()   - 
Method in class weka.classifiers.meta.nestedDichotomies. ClassBalancedND 
 Returns an instance of a TechnicalInformation object, containing 
 detailed information about the technical background of this class,
 e.g., paper reference or book this class is based on.
   getTechnicalInformation()   - 
Method in class weka.classifiers.meta.nestedDichotomies. DataNearBalancedND 
 Returns an instance of a TechnicalInformation object, containing 
 detailed information about the technical background of this class,
 e.g., paper reference or book this class is based on.
   getTechnicalInformation()   - 
Method in class weka.classifiers.meta.nestedDichotomies. ND 
 Returns an instance of a TechnicalInformation object, containing 
 detailed information about the technical background of this class,
 e.g., paper reference or book this class is based on.
   getTechnicalInformation()   - 
Method in class weka.classifiers.meta. OrdinalClassClassifier 
 Returns an instance of a TechnicalInformation object, containing 
 detailed information about the technical background of this class,
 e.g., paper reference or book this class is based on.
   getTechnicalInformation()   - 
Method in class weka.classifiers.meta. RacedIncrementalLogitBoost 
 Returns an instance of a TechnicalInformation object, containing 
 detailed information about the technical background of this class,
 e.g., paper reference or book this class is based on.
   getTechnicalInformation()   - 
Method in class weka.classifiers.meta. RandomSubSpace 
 Returns an instance of a TechnicalInformation object, containing 
 detailed information about the technical background of this class,
 e.g., paper reference or book this class is based on.
   getTechnicalInformation()   - 
Method in class weka.classifiers.meta. RotationForest 
 Returns an instance of a TechnicalInformation object, containing 
 detailed information about the technical background of this class,
 e.g., paper reference or book this class is based on.
   getTechnicalInformation()   - 
Method in class weka.classifiers.meta. Stacking 
 Returns an instance of a TechnicalInformation object, containing
 detailed information about the technical background of this class,
 e.g., paper reference or book this class is based on.
   getTechnicalInformation()   - 
Method in class weka.classifiers.meta. StackingC 
 Returns an instance of a TechnicalInformation object, containing 
 detailed information about the technical background of this class,
 e.g., paper reference or book this class is based on.
   getTechnicalInformation()   - 
Method in class weka.classifiers.meta. Vote 
 Returns an instance of a TechnicalInformation object, containing 
 detailed information about the technical background of this class,
 e.g., paper reference or book this class is based on.
   getTechnicalInformation()   - 
Method in class weka.classifiers.mi. CitationKNN 
 Returns an instance of a TechnicalInformation object, containing 
 detailed information about the technical background of this class,
 e.g., paper reference or book this class is based on.
   getTechnicalInformation()   - 
Method in class weka.classifiers.mi. MDD 
 Returns an instance of a TechnicalInformation object, containing 
 detailed information about the technical background of this class,
 e.g., paper reference or book this class is based on.
   getTechnicalInformation()   - 
Method in class weka.classifiers.mi. MIBoost 
 Returns an instance of a TechnicalInformation object, containing 
 detailed information about the technical background of this class,
 e.g., paper reference or book this class is based on.
   getTechnicalInformation()   - 
Method in class weka.classifiers.mi. MIDD 
 Returns an instance of a TechnicalInformation object, containing 
 detailed information about the technical background of this class,
 e.g., paper reference or book this class is based on.
   getTechnicalInformation()   - 
Method in class weka.classifiers.mi. MIEMDD 
 Returns an instance of a TechnicalInformation object, containing 
 detailed information about the technical background of this class,
 e.g., paper reference or book this class is based on.
   getTechnicalInformation()   - 
Method in class weka.classifiers.mi. MINND 
 Returns an instance of a TechnicalInformation object, containing 
 detailed information about the technical background of this class,
 e.g., paper reference or book this class is based on.
   getTechnicalInformation()   - 
Method in class weka.classifiers.mi. MIOptimalBall 
 Returns an instance of a TechnicalInformation object, containing 
 detailed information about the technical background of this class,
 e.g., paper reference or book this class is based on.
   getTechnicalInformation()   - 
Method in class weka.classifiers.mi. MISMO 
 Returns an instance of a TechnicalInformation object, containing 
 detailed information about the technical background of this class,
 e.g., paper reference or book this class is based on.
   getTechnicalInformation()   - 
Method in class weka.classifiers.mi. MISVM 
 Returns an instance of a TechnicalInformation object, containing 
 detailed information about the technical background of this class,
 e.g., paper reference or book this class is based on.
   getTechnicalInformation()   - 
Method in class weka.classifiers.mi. MIWrapper 
 Returns an instance of a TechnicalInformation object, containing 
 detailed information about the technical background of this class,
 e.g., paper reference or book this class is based on.
   getTechnicalInformation()   - 
Method in class weka.classifiers.misc. VFI 
 Returns an instance of a TechnicalInformation object, containing 
 detailed information about the technical background of this class,
 e.g., paper reference or book this class is based on.
   getTechnicalInformation()   - 
Method in class weka.classifiers.rules. DecisionTable 
 Returns an instance of a TechnicalInformation object, containing 
 detailed information about the technical background of this class,
 e.g., paper reference or book this class is based on.
   getTechnicalInformation()   - 
Method in class weka.classifiers.rules. DTNB 
 Returns an instance of a TechnicalInformation object, containing 
 detailed information about the technical background of this class,
 e.g., paper reference or book this class is based on.
   getTechnicalInformation()   - 
Method in class weka.classifiers.rules. JRip 
 Returns an instance of a TechnicalInformation object, containing 
 detailed information about the technical background of this class,
 e.g., paper reference or book this class is based on.
   getTechnicalInformation()   - 
Method in class weka.classifiers.rules. M5Rules 
 Returns an instance of a TechnicalInformation object, containing 
 detailed information about the technical background of this class,
 e.g., paper reference or book this class is based on.
   getTechnicalInformation()   - 
Method in class weka.classifiers.rules. NNge 
 Returns an instance of a TechnicalInformation object, containing 
 detailed information about the technical background of this class,
 e.g., paper reference or book this class is based on.
   getTechnicalInformation()   - 
Method in class weka.classifiers.rules. OneR 
 Returns an instance of a TechnicalInformation object, containing 
 detailed information about the technical background of this class,
 e.g., paper reference or book this class is based on.
   getTechnicalInformation()   - 
Method in class weka.classifiers.rules. PART 
 Returns an instance of a TechnicalInformation object, containing 
 detailed information about the technical background of this class,
 e.g., paper reference or book this class is based on.
   getTechnicalInformation()   - 
Method in class weka.classifiers.rules. Prism 
 Returns an instance of a TechnicalInformation object, containing 
 detailed information about the technical background of this class,
 e.g., paper reference or book this class is based on.
   getTechnicalInformation()   - 
Method in class weka.classifiers.trees. ADTree 
 Returns an instance of a TechnicalInformation object, containing 
 detailed information about the technical background of this class,
 e.g., paper reference or book this class is based on.
   getTechnicalInformation()   - 
Method in class weka.classifiers.trees. BFTree 
 Returns an instance of a TechnicalInformation object, containing 
 detailed information about the technical background of this class,
 e.g., paper reference or book this class is based on.
   getTechnicalInformation()   - 
Method in class weka.classifiers.trees. FT 
 Returns an instance of a TechnicalInformation object, containing 
 detailed information about the technical background of this class,
 e.g., paper reference or book this class is based on.
   getTechnicalInformation()   - 
Method in class weka.classifiers.trees. Id3 
 Returns an instance of a TechnicalInformation object, containing 
 detailed information about the technical background of this class,
 e.g., paper reference or book this class is based on.
   getTechnicalInformation()   - 
Method in class weka.classifiers.trees. J48 
 Returns an instance of a TechnicalInformation object, containing 
 detailed information about the technical background of this class,
 e.g., paper reference or book this class is based on.
   getTechnicalInformation()   - 
Method in class weka.classifiers.trees. J48graft 
 Returns an instance of a TechnicalInformation object, containing
 detailed information about the technical background of this class,
 e.g., paper reference or book this class is based on.
   getTechnicalInformation()   - 
Method in class weka.classifiers.trees. LADTree 
 Returns an instance of a TechnicalInformation object, containing 
 detailed information about the technical background of this class,
 e.g., paper reference or book this class is based on.
   getTechnicalInformation()   - 
Method in class weka.classifiers.trees. LMT 
 Returns an instance of a TechnicalInformation object, containing 
 detailed information about the technical background of this class,
 e.g., paper reference or book this class is based on.
   getTechnicalInformation()   - 
Method in class weka.classifiers.trees.m5. M5Base 
 Returns an instance of a TechnicalInformation object, containing 
 detailed information about the technical background of this class,
 e.g., paper reference or book this class is based on.
   getTechnicalInformation()   - 
Method in class weka.classifiers.trees. NBTree 
 Returns an instance of a TechnicalInformation object, containing 
 detailed information about the technical background of this class,
 e.g., paper reference or book this class is based on.
   getTechnicalInformation()   - 
Method in class weka.classifiers.trees. RandomForest 
 Returns an instance of a TechnicalInformation object, containing 
 detailed information about the technical background of this class,
 e.g., paper reference or book this class is based on.
   getTechnicalInformation()   - 
Method in class weka.classifiers.trees. SimpleCart 
 Returns an instance of a TechnicalInformation object, containing 
 detailed information about the technical background of this class,
 e.g., paper reference or book this class is based on.
   getTechnicalInformation()   - 
Method in class weka.classifiers.trees. UserClassifier 
 Returns an instance of a TechnicalInformation object, containing 
 detailed information about the technical background of this class,
 e.g., paper reference or book this class is based on.
   getTechnicalInformation()   - 
Method in class weka.clusterers. CLOPE 
 Returns an instance of a TechnicalInformation object, containing
 detailed information about the technical background of this class,
 e.g., paper reference or book this class is based on.
   getTechnicalInformation()   - 
Method in class weka.clusterers. Cobweb 
 Returns an instance of a TechnicalInformation object, containing 
 detailed information about the technical background of this class,
 e.g., paper reference or book this class is based on.
   getTechnicalInformation()   - 
Method in class weka.clusterers. DBScan 
 Returns an instance of a TechnicalInformation object, containing 
 detailed information about the technical background of this class,
 e.g., paper reference or book this class is based on.
   getTechnicalInformation()   - 
Method in class weka.clusterers. FarthestFirst 
 Returns an instance of a TechnicalInformation object, containing 
 detailed information about the technical background of this class,
 e.g., paper reference or book this class is based on.
   getTechnicalInformation()   - 
Method in class weka.clusterers. OPTICS 
 Returns an instance of a TechnicalInformation object, containing 
 detailed information about the technical background of this class,
 e.g., paper reference or book this class is based on.
   getTechnicalInformation()   - 
Method in class weka.clusterers. sIB 
 Returns an instance of a TechnicalInformation object, containing 
 detailed information about the technical background of this class,
 e.g., paper reference or book this class is based on.
   getTechnicalInformation()   - 
Method in class weka.clusterers. XMeans 
 Returns an instance of a TechnicalInformation object, containing 
 detailed information about the technical background of this class,
 e.g., paper reference or book this class is based on.
   getTechnicalInformation()   - 
Method in class weka.core. ChebyshevDistance 
 Returns an instance of a TechnicalInformation object, containing 
 detailed information about the technical background of this class,
 e.g., paper reference or book this class is based on.
   getTechnicalInformation()   - 
Method in class weka.core. EuclideanDistance 
 Returns an instance of a TechnicalInformation object, containing 
 detailed information about the technical background of this class,
 e.g., paper reference or book this class is based on.
   getTechnicalInformation()   - 
Method in class weka.core. ManhattanDistance 
 Returns an instance of a TechnicalInformation object, containing 
 detailed information about the technical background of this class,
 e.g., paper reference or book this class is based on.
   getTechnicalInformation()   - 
Method in class weka.core.neighboursearch. BallTree 
 Returns an instance of a TechnicalInformation object, containing detailed
 information about the technical background of this class, e.g., paper
 reference or book this class is based on.
   getTechnicalInformation()   - 
Method in class weka.core.neighboursearch.balltrees. BottomUpConstructor 
 Returns an instance of a TechnicalInformation object, containing detailed
 information about the technical background of this class, e.g., paper
 reference or book this class is based on.
   getTechnicalInformation()   - 
Method in class weka.core.neighboursearch.balltrees. MedianDistanceFromArbitraryPoint 
 Returns an instance of a TechnicalInformation object, containing detailed
 information about the technical background of this class, e.g., paper
 reference or book this class is based on.
   getTechnicalInformation()   - 
Method in class weka.core.neighboursearch.balltrees. MedianOfWidestDimension 
 Returns an instance of a TechnicalInformation object, containing detailed
 information about the technical background of this class, e.g., paper
 reference or book this class is based on.
   getTechnicalInformation()   - 
Method in class weka.core.neighboursearch.balltrees. MiddleOutConstructor 
 Returns an instance of a TechnicalInformation object, containing detailed
 information about the technical background of this class, e.g., paper
 reference or book this class is based on.
   getTechnicalInformation()   - 
Method in class weka.core.neighboursearch.balltrees. PointsClosestToFurthestChildren 
 Returns an instance of a TechnicalInformation object, containing detailed
 information about the technical background of this class, e.g., paper
 reference or book this class is based on.
   getTechnicalInformation()   - 
Method in class weka.core.neighboursearch.balltrees. TopDownConstructor 
 Returns an instance of a TechnicalInformation object, containing detailed
 information about the technical background of this class, e.g., paper
 reference or book this class is based on.
   getTechnicalInformation()   - 
Method in class weka.core.neighboursearch. CoverTree 
 Returns an instance of a TechnicalInformation object, containing detailed
 information about the technical background of this class, e.g., paper
 reference or book this class is based on.
   getTechnicalInformation()   - 
Method in class weka.core.neighboursearch. KDTree 
 Returns an instance of a TechnicalInformation object, containing detailed
 information about the technical background of this class, e.g., paper
 reference or book this class is based on.
   getTechnicalInformation()   - 
Method in class weka.core.neighboursearch.kdtrees. KMeansInpiredMethod 
 Returns an instance of a TechnicalInformation object, containing detailed
 information about the technical background of this class, e.g., paper
 reference or book this class is based on.
   getTechnicalInformation()   - 
Method in class weka.core.neighboursearch.kdtrees. MedianOfWidestDimension 
 Returns an instance of a TechnicalInformation object, containing detailed
 information about the technical background of this class, e.g., paper
 reference or book this class is based on.
   getTechnicalInformation()   - 
Method in class weka.core.neighboursearch.kdtrees. MidPointOfWidestDimension 
 Returns an instance of a TechnicalInformation object, containing detailed
 information about the technical background of this class, e.g., paper
 reference or book this class is based on.
   getTechnicalInformation()   - 
Method in class weka.core.neighboursearch.kdtrees. SlidingMidPointOfWidestSide 
 Returns an instance of a TechnicalInformation object, containing detailed
 information about the technical background of this class, e.g., paper
 reference or book this class is based on.
   getTechnicalInformation()   - 
Method in class weka.core. Optimization 
 Returns an instance of a TechnicalInformation object, containing 
 detailed information about the technical background of this class,
 e.g., paper reference or book this class is based on.
   getTechnicalInformation()   - 
Method in class weka.core.stemmers. LovinsStemmer 
 Returns an instance of a TechnicalInformation object, containing 
 detailed information about the technical background of this class,
 e.g., paper reference or book this class is based on.
   getTechnicalInformation()   - 
Method in interface weka.core. TechnicalInformationHandler 
 Returns an instance of a TechnicalInformation object, containing 
 detailed information about the technical background of this class,
 e.g., paper reference or book this class is based on.
   getTechnicalInformation()   - 
Method in class weka.datagenerators.classifiers.classification. Agrawal 
 Returns an instance of a TechnicalInformation object, containing 
 detailed information about the technical background of this class,
 e.g., paper reference or book this class is based on.
   getTechnicalInformation()   - 
Method in class weka.datagenerators.classifiers.classification. LED24 
 Returns an instance of a TechnicalInformation object, containing 
 detailed information about the technical background of this class,
 e.g., paper reference or book this class is based on.
   getTechnicalInformation()   - 
Method in class weka.datagenerators.clusterers. BIRCHCluster 
 Returns an instance of a TechnicalInformation object, containing 
 detailed information about the technical background of this class,
 e.g., paper reference or book this class is based on.
   getTechnicalInformation()   - 
Method in class weka.experiment. PairedCorrectedTTester 
 Returns an instance of a TechnicalInformation object, containing 
 detailed information about the technical background of this class,
 e.g., paper reference or book this class is based on.
   getTechnicalInformation()   - 
Method in class weka.filters.supervised.attribute. Discretize 
 Returns an instance of a TechnicalInformation object, containing 
 detailed information about the technical background of this class,
 e.g., paper reference or book this class is based on.
   getTechnicalInformation()   - 
Method in class weka.filters.supervised.attribute. NominalToBinary 
 Returns an instance of a TechnicalInformation object, containing 
 detailed information about the technical background of this class,
 e.g., paper reference or book this class is based on.
   getTechnicalInformation()   - 
Method in class weka.filters.supervised.attribute. PLSFilter 
 Returns an instance of a TechnicalInformation object, containing 
 detailed information about the technical background of this class,
 e.g., paper reference or book this class is based on.
   getTechnicalInformation()   - 
Method in class weka.filters.supervised.instance. SMOTE 
 Returns an instance of a TechnicalInformation object, containing 
 detailed information about the technical background of this class,
 e.g., paper reference or book this class is based on.
   getTechnicalInformation()   - 
Method in class weka.filters.unsupervised.attribute. KernelFilter 
 Returns an instance of a TechnicalInformation object, containing 
 detailed information about the technical background of this class,
 e.g., paper reference or book this class is based on.
   getTechnicalInformation()   - 
Method in class weka.filters.unsupervised.attribute. PKIDiscretize 
 Returns an instance of a TechnicalInformation object, containing 
 detailed information about the technical background of this class,
 e.g., paper reference or book this class is based on.
   getTechnicalInformation()   - 
Method in class weka.filters.unsupervised.attribute. RandomProjection 
 Returns an instance of a TechnicalInformation object, containing 
 detailed information about the technical background of this class,
 e.g., paper reference or book this class is based on.
   getTechnicalInformation()   - 
Method in class weka.filters.unsupervised.attribute. RELAGGS 
 Returns an instance of a TechnicalInformation object, containing 
 detailed information about the technical background of this class,
 e.g., paper reference or book this class is based on.
   getTechnicalInformation()   - 
Method in class weka.filters.unsupervised.attribute. Wavelet 
 Returns an instance of a TechnicalInformation object, containing 
 detailed information about the technical background of this class,
 e.g., paper reference or book this class is based on.
   getTechnicalInformation()   - 
Method in class weka.gui.boundaryvisualizer. BoundaryVisualizer 
 Returns an instance of a TechnicalInformation object, containing 
 detailed information about the technical background of this class,
 e.g., paper reference or book this class is based on.
   getTempDir()   - 
Static method in class weka.core. Debug 
 returns the system temp directory
   getTester()   - 
Static method in class weka.gui.experiment. ExperimenterDefaults 
 returns the display name of the preferred Tester algorithm
   getTestEvaluator()   - 
Method in class weka.attributeSelection. CheckAttributeSelection 
 Gets whether the evaluator is being tested or the search method.
   getTestOrTrain()   - 
Method in class weka.gui.beans. BatchClustererEvent 
 Get whether the set of instances is a test or a training set
   getTestPredictions(Classifier, Instances)   - 
Method in class weka.classifiers.evaluation. EvaluationUtils 
 Generate a bunch of predictions ready for processing, by performing a
 evaluation on a test set assuming the classifier is already trained.
   getTestSet()   - 
Method in class weka.gui.beans. BatchClassifierEvent 
 Get the test set
   getTestSet()   - 
Method in class weka.gui.beans. BatchClustererEvent 
 Get the training/test set
   getTestSet()   - 
Method in class weka.gui.beans. TestSetEvent 
 Get the test set instances
   getText()   - 
Method in class weka.gui.beans. BeanVisual 
 Get the visual's label
   getText()   - 
Method in class weka.gui.beans. TextEvent 
 Describe  getText  method here.
   getTextTitle()   - 
Method in class weka.gui.beans. TextEvent 
 Describe  getTextTitle  method here.
   getTFTransform()   - 
Method in class weka.filters.unsupervised.attribute. StringToWordVector 
 Gets whether if the word frequencies should be transformed into
  log(1+fij) where fij is the frequency of word i in document(instance) j.
   getThreshold()   - 
Method in class weka.attributeSelection. GreedyStepwise 
 Returns the threshold so that the AttributeSelection module can
 discard attributes from the ranking.
   getThreshold()   - 
Method in class weka.attributeSelection. RaceSearch 
 Get the threshold
   getThreshold()   - 
Method in interface weka.attributeSelection. RankedOutputSearch 
 Gets the threshold by which attributes can be discarded.
   getThreshold()   - 
Method in class weka.attributeSelection. Ranker 
 Returns the threshold so that the AttributeSelection module can
 discard attributes from the ranking.
   getThreshold()   - 
Method in class weka.attributeSelection. ScatterSearchV1 
 Get the treshold
   getThreshold()   - 
Method in class weka.attributeSelection. WrapperSubsetEval 
 Get the value of the threshold
   getThreshold()   - 
Method in class weka.classifiers.bayes. BayesianLogisticRegression 
 Return the threshold being used.
   getThreshold()   - 
Method in class weka.classifiers.functions. PaceRegression 
 Gets the threshold for olsc estimator
   getThreshold()   - 
Method in class weka.classifiers.functions. Winnow 
 Get the value of Threshold.
   getThreshold()   - 
Method in class weka.filters.unsupervised.instance. RemoveMisclassified 
 Gets the threshold for the max error when predicting a numeric class.
   getThresholdInstance(Instances, double)   - 
Static method in class weka.classifiers.evaluation. ThresholdCurve 
 Gets the index of the instance with the closest threshold value to the
 desired target
   getTimestamp()   - 
Static method in class weka.experiment. CrossValidationResultProducer 
 Gets a Double representing the current date and time.
   getTimestamp()   - 
Static method in class weka.experiment. RandomSplitResultProducer 
 Gets a Double representing the current date and time.
   getTitle()   - 
Method in class weka.gui.arffviewer. ArffPanel 
 returns the title for the Tab, i.e.
   getToken(StreamTokenizer)   - 
Static method in class weka.core.converters. ConverterUtils 
 Gets token.
   getTokenizer()   - 
Method in class weka.filters.unsupervised.attribute. StringToWordVector 
 Returns the current tokenizer algorithm.
   getTolerance()   - 
Method in class weka.classifiers.bayes. BayesianLogisticRegression 
 Get the tolerance value
   getTolerance()   - 
Method in class weka.classifiers.functions.supportVector. RegSMOImproved 
 returns the current tolerance
   getToleranceParameter()   - 
Method in class weka.attributeSelection. SVMAttributeEval 
 Get the value of T used with SMO
   getToleranceParameter()   - 
Method in class weka.classifiers.functions. SMO 
 Get the value of tolerance parameter.
   getToleranceParameter()   - 
Method in class weka.classifiers.mi. MISMO 
 Get the value of tolerance parameter.
   getToolTipText()   - 
Method in class weka.experiment. PairedCorrectedTTester 
 returns a string that is displayed as tooltip on the "perform test"
 button in the experimenter
   getToolTipText()   - 
Method in class weka.experiment. PairedTTester 
 returns a string that is displayed as tooltip on the "perform test"
 button in the experimenter
   getToolTipText()   - 
Method in interface weka.experiment. Tester 
 returns a string that is displayed as tooltip on the "perform test"
 button in the experimenter
   getToolTipText(MouseEvent)   - 
Method in class weka.gui. AttributeVisualizationPanel 
 Returns "<nominal value> [<nominal value count>]"
 if displaying a bar plot and mouse is on some bar.
   getToolTipText(PrintableComponent)   - 
Static method in class weka.gui.visualize. PrintableComponent 
 Returns a tooltip only if the user wants it.
   getTop()   - 
Method in class weka.gui.treevisualizer. Node 
 Get the value of top.
   getTotalCoordsPerPoint()   - 
Method in class weka.core.neighboursearch. PerformanceStats 
 Returns the total sum of coords per point.
   getTotalCount(Node, int)   - 
Static method in class weka.gui.treevisualizer. Node 
 Recursively finds the total number of nodes there are.
   getTotalGCount(Node, int)   - 
Static method in class weka.gui.treevisualizer. Node 
 Recursively finds the total number of groups of siblings there are.
   getTotalHeight(Node, int)   - 
Static method in class weka.gui.treevisualizer. Node 
 Recursively finds the total number of levels there are.
   getTotalIntNodesVisited()   - 
Method in class weka.core.neighboursearch. TreePerformanceStats 
 Returns the total number of internal nodes visited.
   getTotalLeavesVisited()   - 
Method in class weka.core.neighboursearch. TreePerformanceStats 
 Returns the total number of leaves visited.
   getTotalPointsVisited()   - 
Method in class weka.core.neighboursearch. PerformanceStats 
 Returns the total number of points visited.
   getTotalSupport()   - 
Method in class weka.associations. FPGrowth.AssociationRule 
 Get the total support for this rule (premise + consequence).
   getTotalTransactions()   - 
Method in class weka.associations. FPGrowth.AssociationRule 
 Get the total number of transactions in the data.
   getToYear()   - 
Static method in class weka.core. Copyright 
 returns the end year of the copyright (i.e., current year)
   getTPRate()   - 
Method in class weka.associations.tertius. Rule 
 Get the rate of True Positive instances of this rule.
   getTrainingSet()   - 
Method in class weka.gui.beans. TrainingSetEvent 
 Get the training instances
   getTrainingTime()   - 
Method in class weka.classifiers.functions. MultilayerPerceptron 
  
   getTrainIterations()   - 
Method in class weka.classifiers. BVDecompose 
 Gets the maximum number of boost iterations
   getTrainPercent()   - 
Method in class weka.experiment. RandomSplitResultProducer 
 Get the value of TrainPercent.
   getTrainPercent()   - 
Method in class weka.gui.beans. TrainTestSplitMaker 
 Get the percentage of the data that will be in the training portion of
 the split
   getTrainPercentage()   - 
Static method in class weka.gui.experiment. ExperimenterDefaults 
 returns the training percentage in case of splits
   getTrainPoolSize()   - 
Method in class weka.classifiers. BVDecompose 
 Get the number of instances in the training pool.
   getTrainSet()   - 
Method in class weka.gui.beans. BatchClassifierEvent 
 Get the train set
   getTrainSize()   - 
Method in class weka.classifiers. BVDecomposeSegCVSub 
 Get the training size
   getTrainTestPredictions(Classifier, Instances, Instances)   - 
Method in class weka.classifiers.evaluation. EvaluationUtils 
 Generate a bunch of predictions ready for processing, by performing a
 evaluation on a test set after training on the given training set.
   getTransactionsMustContain()   - 
Method in class weka.associations. FPGrowth 
 Gets the comma separated list of items that
 transactions must contain in order to be considered
 for large item sets and rules.
   getTransform()   - 
Method in class weka.gui.visualize. PostscriptGraphics 
  
   getTransformAllValues()   - 
Method in class weka.filters.supervised.attribute. NominalToBinary 
 Gets if all nominal values are turned into new attributes, not only if
 there are more than 2.
   getTransformAllValues()   - 
Method in class weka.filters.unsupervised.attribute. NominalToBinary 
 Gets if all nominal values are turned into new attributes, not only if
 there are more than 2.
   getTransformationDictionary()   - 
Method in class weka.core.pmml. MiningSchema 
 Get the transformation dictionary .
   getTransformBackToOriginal()   - 
Method in class weka.attributeSelection. PrincipalComponents 
 Gets whether the data is to be transformed back to the original
 space.
   getTransformMethod()   - 
Method in class weka.classifiers.mi. SimpleMI 
 Get the method used in transformation.
   getTranslation()   - 
Method in class weka.filters.unsupervised.attribute. Normalize 
 Get the translation.
   getTraversal()   - 
Method in class weka.classifiers.meta. GridSearch 
 Gets the type of traversal for the grid.
   getTrimingThreshold()   - 
Method in class weka.classifiers.functions.pace. ChisqMixture 
 Gets the triming thresholding value.
   getTrimingThreshold()   - 
Method in class weka.classifiers.functions.pace. NormalMixture 
 Gets the triming thresholding value.
   getTrueNegative()   - 
Method in class weka.classifiers.evaluation. TwoClassStats 
 Gets the number of negative instances predicted as negative
   getTruePositive()   - 
Method in class weka.classifiers.evaluation. TwoClassStats 
 Gets the number of positive instances predicted as positive
   getTruePositiveRate()   - 
Method in class weka.classifiers.evaluation. TwoClassStats 
 Calculate the true positive rate.
   getTStart()   - 
Method in class weka.classifiers.bayes.net.search.global. SimulatedAnnealing 
  
   getTStart()   - 
Method in class weka.classifiers.bayes.net.search.local. SimulatedAnnealing 
  
   getTwoClassStats(int)   - 
Method in class weka.classifiers.evaluation. ConfusionMatrix 
 Gets the performance with respect to one of the classes
 as a TwoClassStats object.
   getType()   - 
Method in class weka.associations.tertius. IndividualLiteral 
  
   getType()   - 
Method in class weka.associations.tertius. LiteralSet 
 Give the type of properties in this set (individual or part properties).
   getType()   - 
Method in class weka.attributeSelection. LinearForwardSelection 
 Get the type
   getType()   - 
Method in class weka.attributeSelection. SubsetSizeForwardSelection 
 Get the type
   getType()   - 
Method in class weka.classifiers.functions.neural. NeuralConnection 
  
   getType()   - 
Method in class weka.core. AttributeLocator 
 returns the type of attribute that is located
   getType(RevisionHandler)   - 
Static method in class weka.core. RevisionUtils 
 Determines the type of a (sanitized) revision string returned by the 
 RevisionHandler.
   getType(String)   - 
Static method in class weka.core. RevisionUtils 
 Determines the type of a (sanitized) revision string.
   getType()   - 
Method in class weka.core. TechnicalInformation 
 returns the type of this technical information
   getType(int)   - 
Method in class weka.gui.arffviewer. ArffSortedTableModel 
 returns the TYPE of the attribute at the given position
   getType(int, int)   - 
Method in class weka.gui.arffviewer. ArffSortedTableModel 
 returns the TYPE of the attribute at the given position
   getType(int)   - 
Method in class weka.gui.arffviewer. ArffTableModel 
 returns the TYPE of the attribute at the given position
   getType(int, int)   - 
Method in class weka.gui.arffviewer. ArffTableModel 
 returns the TYPE of the attribute at the given position
   getType()   - 
Method in class weka.gui.sql.event. ConnectionEvent 
 returns the type of this event, CONNECT or DISCONNECT
   getU()   - 
Method in class weka.core. Matrix 
  Deprecated.  Returns the U part of the matrix.
   getU()   - 
Method in class weka.core.matrix. LUDecomposition 
 Return upper triangular factor
   getU()   - 
Method in class weka.core.matrix. SingularValueDecomposition 
 Return the left singular vectors
   getUID(String)   - 
Static method in class weka.core. SerializationHelper 
 reads or creates the serialVersionUID for the given class.
   getUID(Class)   - 
Static method in class weka.core. SerializationHelper 
 reads or creates the serialVersionUID for the given class.
   getUnpruned()   - 
Method in class weka.classifiers.rules. PART 
 Get the value of unpruned.
   getUnpruned()   - 
Method in class weka.classifiers.trees. J48 
 Get the value of unpruned.
   getUnpruned()   - 
Method in class weka.classifiers.trees. J48graft 
 Get the value of unpruned.
   getUnpruned()   - 
Method in class weka.classifiers.trees.m5. M5Base 
 Get whether unpruned tree/rules are being generated
   getUnpruned()   - 
Method in class weka.classifiers.trees.m5. Rule 
 Get whether unpruned tree/rules are being generated
   getUpdateCount()   - 
Method in class weka.core.converters. DatabaseConnection 
 Dewtermines if the current query retrieves a result set or updates a table
   getUpdateIncrementalClassifier()   - 
Method in class weka.gui.beans. Classifier 
 Get whether an incremental classifier will be updated on the
 incoming instance stream.
   getUpper()   - 
Method in class weka.gui.experiment. RunNumberPanel 
 Gets the current upper run number.
   getUpperBoundMinSupport()   - 
Method in class weka.associations. Apriori 
 Get the value of upperBoundMinSupport.
   getUpperBoundMinSupport()   - 
Method in class weka.associations. FPGrowth 
 Get the value of upperBoundMinSupport.
   getUpperCase()   - 
Method in class weka.core.converters. DatabaseConnection 
 Check if the property checkUpperCaseNames in the DatabaseUtils file is 
 set to true or false.
   getUpperNumericBound()   - 
Method in class weka.core. Attribute 
 Returns the upper bound of a numeric attribute.
   getUpperSize()   - 
Method in class weka.experiment. LearningRateResultProducer 
 Get the value of UpperSize.
   getUrl()   - 
Method in interface weka.core.converters. DatabaseConverter 
  
   getUrl()   - 
Method in class weka.core.converters. DatabaseLoader 
 Gets the URL
   getUrl()   - 
Method in class weka.core.converters. DatabaseSaver 
 Gets the database URL.
   getURL()   - 
Static method in class weka.core. Copyright 
 returns the URL of the owner
   getURL()   - 
Method in class weka.gui. DatabaseConnectionDialog 
 Returns URL from dialog
   getURL(String, String)   - 
Static method in class weka.gui. Loader 
 returns a URL for the given filename, can be NULL if it fails
   getURL(String)   - 
Method in class weka.gui. Loader 
 returns a URL for the given filename, can be NULL if it fails
   getURL()   - 
Method in class weka.gui.sql. ConnectionPanel 
 returns the current URL.
   getURL()   - 
Method in class weka.gui.sql.event. ResultChangedEvent 
 returns the database URL that produced the table model
   getURL()   - 
Method in class weka.gui.sql. ResultSetTable 
 returns the database URL that produced the table model
   getURL()   - 
Method in class weka.gui.sql. SqlViewer 
 returns the database URL from the currently active tab in the ResultPanel,
 otherwise an empty string.
   getURL()   - 
Method in class weka.gui.sql. SqlViewerDialog 
 returns the chosen URL, if any
   getURLFileLoaders()   - 
Static method in class weka.core.converters. ConverterUtils 
 returns a vector with the classnames of all the URL file loaders.
   getURLLoaderForExtension(String)   - 
Static method in class weka.core.converters. ConverterUtils 
 tries to determine the URL loader to use for this kind of extension, returns
 null if none can be found.
   getURLLoaderForFile(String)   - 
Static method in class weka.core.converters. ConverterUtils 
 tries to determine the URL loader to use for this kind of file, returns
 null if none can be found.
   getURLLoaderForFile(File)   - 
Static method in class weka.core.converters. ConverterUtils 
 tries to determine the URL loader to use for this kind of file, returns
 null if none can be found.
   getUsageType()   - 
Method in class weka.core.pmml. MiningFieldMetaInfo 
 Get the usage type of this field.
   getUseADTree()   - 
Method in class weka.classifiers.bayes. BayesNet 
 Method declaration
   getUseAIC()   - 
Method in class weka.classifiers.functions. SimpleLogistic 
 Get the value of useAIC.
   getUseAIC()   - 
Method in class weka.classifiers.trees. FT 
 Get the value of useAIC.
   getUseAIC()   - 
Method in class weka.classifiers.trees. LMT 
 Get the value of useAIC.
   getUseAIC()   - 
Method in class weka.classifiers.trees.lmt. LogisticBase 
 Get the value of useAIC.
   getUseArcReversal()   - 
Method in class weka.classifiers.bayes.net.search.global. HillClimber 
 get use the arc reversal operation
   getUseArcReversal()   - 
Method in class weka.classifiers.bayes.net.search.local. HillClimber 
 get use the arc reversal operation
   getUseBetterEncoding()   - 
Method in class weka.filters.supervised.attribute. Discretize 
 Gets whether better encoding is to be used for MDL.
   getUseClassification()   - 
Static method in class weka.gui.experiment. ExperimenterDefaults 
 whether classification or regression is used
   getUseCpuTime()   - 
Method in class weka.core. Debug.Clock 
 returns whether the use of CPU is time is enabled/disabled (regardless
 whether the system supports it or not)
   getUseCrossOver()   - 
Method in class weka.classifiers.bayes.net.search.global. GeneticSearch 
  
   getUseCrossOver()   - 
Method in class weka.classifiers.bayes.net.search.local. GeneticSearch 
  
   getUseCrossValidation()   - 
Method in class weka.classifiers.functions. SimpleLogistic 
 Get the value of useCrossValidation.
   getUseCustomDimensions()   - 
Method in class weka.gui.visualize. JComponentWriter 
 whether custom dimensions are to used for the size of the image
   getUsedAttributes()   - 
Method in class weka.classifiers.trees.lmt. LogisticBase 
 Returns an array of the indices of the attributes used in the logistic model.
   getUseEqualFrequency()   - 
Method in class weka.classifiers.meta. RegressionByDiscretization 
 Get the value of UseEqualFrequency.
   getUseEqualFrequency()   - 
Method in class weka.filters.unsupervised.attribute. Discretize 
 Get the value of UseEqualFrequency.
   getUseEqualFrequency()   - 
Method in class weka.filters.unsupervised.attribute. PKIDiscretize 
 Get the value of UseEqualFrequency.
   getUseErrorRate()   - 
Method in class weka.classifiers.trees. BFTree 
 Get if use error rate in internal cross-validation.
   getUseGini()   - 
Method in class weka.classifiers.trees. BFTree 
 Get if use Gini index as splitting criterion.
   getUseGUI()   - 
Method in class weka.core. Memory 
 whether to display a dialog in case of a problem (= TRUE) or just print
 on stderr (= FALSE)
   getUseIBk()   - 
Method in class weka.classifiers.rules. DecisionTable 
 Gets whether IBk is being used instead of the majority class
   getUseKDTree()   - 
Method in class weka.clusterers. XMeans 
 Gets whether the KDTree is used or not.
   getUseKernelEstimator()   - 
Method in class weka.classifiers.bayes. NaiveBayes 
 Gets if kernel estimator is being used.
   getUseKononenko()   - 
Method in class weka.filters.supervised.attribute. Discretize 
 Gets whether Kononenko's MDL criterion is to be used.
   getUseLaplace()   - 
Method in class weka.classifiers.bayes. AODEsr 
 Gets if laplace correction is being used.
   getUseLaplace()   - 
Method in class weka.classifiers.trees. J48 
 Get the value of useLaplace.
   getUseLaplace()   - 
Method in class weka.classifiers.trees. J48graft 
 Get the value of useLaplace.
   getUseLeastValues()   - 
Method in class weka.filters.unsupervised.instance. RemoveFrequentValues 
 Gets whether to use values with least or most instances
   getUseLowerOrder()   - 
Method in class weka.classifiers.functions.supportVector. PolyKernel 
 Gets whether lower-order terms are used.
   getUseMEstimates()   - 
Method in class weka.classifiers.bayes. AODE 
 Gets if m-estimaces is being used.
   getUseMissing()   - 
Method in class weka.filters.unsupervised.attribute. AddNoise 
 Gets the flag if missing values are treated as extra values.
   getUseMutation()   - 
Method in class weka.classifiers.bayes.net.search.global. GeneticSearch 
  
   getUseMutation()   - 
Method in class weka.classifiers.bayes.net.search.local. GeneticSearch 
  
   getUseNormalization()   - 
Method in class weka.classifiers.functions.supportVector. StringKernel 
 Returns whether normalization is used.
   getUseOneSE()   - 
Method in class weka.classifiers.trees. BFTree 
 Get if use the 1SE rule to choose final model.
   getUseOneSE()   - 
Method in class weka.classifiers.trees. SimpleCart 
 Get if use the 1SE rule to choose final model.
   getUseORForMustContainList()   - 
Method in class weka.associations. FPGrowth 
 Gets whether OR is to be used rather than AND when
 considering must contain lists.
   getUsePairwiseCoupling()   - 
Method in class weka.classifiers.meta. MultiClassClassifier 
 Gets whether to use pairwise coupling with 1-vs-1 
 classification to improve probability estimates.
   getUseProb()   - 
Method in class weka.classifiers.bayes.net.search.global. GlobalScoreSearchAlgorithm 
  
   getUsePropertyIterator()   - 
Method in class weka.experiment. Experiment 
 Gets whether the custom property iterator should be used.
   getUsePrune()   - 
Method in class weka.classifiers.trees. SimpleCart 
 Get if use minimal cost-complexity pruning.
   getUsePruning()   - 
Method in class weka.classifiers.rules. JRip 
 Gets whether pruning is performed
   getUser()   - 
Method in interface weka.core.converters. DatabaseConverter 
  
   getUser()   - 
Method in class weka.core.converters. DatabaseLoader 
 Gets the user name
   getUser()   - 
Method in class weka.core.converters. DatabaseSaver 
 Gets the database user.
   getUser()   - 
Method in class weka.gui.sql. ConnectionPanel 
 returns the current User.
   getUser()   - 
Method in class weka.gui.sql.event. ResultChangedEvent 
 returns the user that produced the table model
   getUser()   - 
Method in class weka.gui.sql. ResultSetTable 
 returns the user that produced the table model
   getUser()   - 
Method in class weka.gui.sql. SqlViewer 
 returns the user from the currently active tab in the ResultPanel,
 otherwise an empty string.
   getUser()   - 
Method in class weka.gui.sql. SqlViewerDialog 
 returns the chosen user, if any
   getUseRelativePath()   - 
Method in class weka.core.converters. AbstractFileLoader 
 Gets whether relative paths are to be used
   getUseRelativePath()   - 
Method in class weka.core.converters. AbstractFileSaver 
 Gets whether relative paths are to be used
   getUseRelativePath()   - 
Method in interface weka.core.converters. FileSourcedConverter 
 Gets whether relative paths are to be used
   getUseRelativePath()   - 
Method in class weka.gui.beans. SerializedModelSaver 
 Get whether to use relative paths for the directory.
   getUseRelativePaths()   - 
Static method in class weka.gui.experiment. ExperimenterDefaults 
 whether relative paths are used by default
   getUseResampling()   - 
Method in class weka.classifiers.meta. AdaBoostM1 
 Get whether resampling is turned on
   getUseResampling()   - 
Method in class weka.classifiers.meta. LogitBoost 
 Get whether resampling is turned on
   getUseResampling()   - 
Method in class weka.classifiers.meta. RacedIncrementalLogitBoost 
 Get whether resampling is turned on
   getUsername()   - 
Method in class weka.experiment. DatabaseUtils 
 Get the database username.
   getUsername()   - 
Method in class weka.gui. DatabaseConnectionDialog 
 Returns Username from dialog
   getUserOptions()   - 
Method in class weka.classifiers.functions.supportVector. KernelEvaluation 
 returns the options the user supplied for the kernel
   getUserOptions()   - 
Method in class weka.core. CheckOptionHandler 
 Gets the current user-supplied options (creates a copy)
   getUseStars()   - 
Method in class weka.core. Javadoc 
 whether the Javadoc is prefixed with "*"
   getUseStoplist()   - 
Method in class weka.filters.unsupervised.attribute. StringToWordVector 
 Gets whether if the words on the stoplist are to be ignored (The stoplist
  is in weka.core.StopWords).
   getUseSupervisedDiscretization()   - 
Method in class weka.classifiers.bayes. NaiveBayes 
 Get whether supervised discretization is to be used.
   getUseTournamentSelection()   - 
Method in class weka.classifiers.bayes.net.search.global. GeneticSearch 
  
   getUseTournamentSelection()   - 
Method in class weka.classifiers.bayes.net.search.local. GeneticSearch 
  
   getUseTraining()   - 
Method in class weka.attributeSelection. ClassifierSubsetEval 
 Get if training data is to be used instead of hold out/test data
   getUseTree()   - 
Method in class weka.classifiers.trees.m5. Rule 
 get whether an m5 tree is being used rather than rules
   getUseUnsmoothed()   - 
Method in class weka.classifiers.trees.m5. M5Base 
 Get whether or not smoothing is being used
   getV()   - 
Method in class weka.core.matrix. EigenvalueDecomposition 
 Return the eigenvector matrix
   getV()   - 
Method in class weka.core.matrix. SingularValueDecomposition 
 Return the right singular vectors
   getValidating()   - 
Method in class weka.core.xml. XMLDocument 
 returns whether a validating parser is used.
   getValidating()   - 
Method in class weka.core.xml. XMLOptions 
 returns whether a validating parser is used.
   getValidationChunkSize()   - 
Method in class weka.classifiers.meta. RacedIncrementalLogitBoost 
 Get the validation chunk size
   getValidationSetSize()   - 
Method in class weka.classifiers.functions. MultilayerPerceptron 
  
   getValidationThreshold()   - 
Method in class weka.classifiers.functions. MultilayerPerceptron 
  
   getValue()   - 
Method in class weka.classifiers.trees.adtree. PredictionNode 
 Gets the prediction value of the node.
   getValue()   - 
Method in class weka.core.pmml. FieldMetaInfo.Value 
  
   getValue(Object, PropertyPath.Path)   - 
Static method in class weka.core. PropertyPath 
 returns the value specified by the given path from the object
   getValue(Object, String)   - 
Static method in class weka.core. PropertyPath 
 returns the value specified by the given path from the object
   getValue(TechnicalInformation.Field)   - 
Method in class weka.core. TechnicalInformation 
 returns the value associated with the given field, or empty if field is
 not currently stored.
   getValue(Instance, int)   - 
Static method in class weka.filters.unsupervised.instance.subsetbyexpression. Parser 
 Returns either a String object for nominal attributes or a Double for numeric
 ones.
   getValue()   - 
Method in class weka.gui. CostMatrixEditor 
 Gets the cost matrix that is being edited.
   getValue()   - 
Method in class weka.gui. GenericArrayEditor 
 Gets the current object array.
   getValue()   - 
Method in class weka.gui. GenericObjectEditor 
 Gets the current Object.
   getValue()   - 
Method in class weka.gui. HierarchyPropertyParser 
 Get the value of current node
   getValue()   - 
Method in class weka.gui. SimpleDateFormatEditor 
 Gets the date format that is being edited.
   getValue()   - 
Method in class weka.gui. SortedTableModel.SortContainer 
 Returns the value to sort on.
   getValueAt(int, int)   - 
Method in class weka.clusterers.forOPTICSAndDBScan.OPTICS_GUI. ResultVectorTableModel 
 Returns the value for the JTable for a given position.
   getValueAt(int, int)   - 
Method in class weka.gui.arffviewer. ArffTableModel 
 returns the value for the cell at columnindex and rowIndex
   getValueAt(int, int)   - 
Method in class weka.gui. SortedTableModel 
 Returns the value for the cell at columnIndex and rowIndex.
   getValueAt(int, int)   - 
Method in class weka.gui.sql. ResultSetTableModel 
 returns the value for the cell at columnindex and rowIndex.
   getValueIndex()   - 
Method in class weka.associations. FPGrowth.BinaryItem 
 Get the value index for this item.
   getValueIndices()   - 
Method in class weka.filters.unsupervised.attribute. MakeIndicator 
 Get the indices of the indicator values.
   getValueName(int, int)   - 
Method in class weka.classifiers.bayes.net. EditableBayesNet 
 returns value of a node
   getValueRange()   - 
Method in class weka.filters.unsupervised.attribute. MakeIndicator 
 Get the range containing the indicator values.
   getValues(String)   - 
Method in class weka.classifiers.bayes.net. EditableBayesNet 
 returns array of values of a node
   getValues(int)   - 
Method in class weka.classifiers.bayes.net. EditableBayesNet 
 returns array of values of a node
   getValues()   - 
Method in class weka.classifiers.meta. GridSearch 
 returns the parameter pair that was found to work best
   getValues()   - 
Method in class weka.core.pmml. TargetMetaInfo 
 Get the values (discrete case only) for this Target.
   getValues()   - 
Method in class weka.gui.visualize. VisualizePanelEvent 
  
   getValuesList()   - 
Method in class weka.datagenerators.clusterers. SubspaceClusterDefinition 
 returns the range for each attribute as string
   getValuesOutput()   - 
Method in class weka.associations. Tertius 
 Get the value of valuesOutput.
   getVarbValues()   - 
Method in class weka.core. Optimization 
 Get the variable values.
   getVariableNames()   - 
Method in class weka.core. Environment 
 Get the names of the variables (keys) stored in the 
 internal map.
   getVariableValue(String)   - 
Method in class weka.core. Environment 
 Get the value for a particular variable.
   getVariance()   - 
Method in class weka.classifiers. BVDecompose 
 Get the calculated variance
   getVarianceCovered()   - 
Method in class weka.attributeSelection. PrincipalComponents 
 Gets the proportion of total variance to account for when
 retaining principal components
   getVarianceCovered()   - 
Method in class weka.filters.unsupervised.attribute. PrincipalComponents 
 Gets the proportion of total variance to account for when
 retaining principal components.
   getVectorOfAttrTypes()   - 
Method in class weka.estimators. CheckEstimator.AttrTypes 
  
   getVerbose()   - 
Method in class weka.associations. Apriori 
 Gets whether algorithm is run in verbose mode
   getVerbose()   - 
Method in class weka.attributeSelection. ExhaustiveSearch 
 get whether or not output is verbose
   getVerbose()   - 
Method in class weka.attributeSelection. LinearForwardSelection 
 Get whether output is to be verbose
   getVerbose()   - 
Method in class weka.attributeSelection. RandomSearch 
 get whether or not output is verbose
   getVerbose()   - 
Method in class weka.attributeSelection. SubsetSizeForwardSelection 
 Get whether output is to be verbose
   getVerbose()   - 
Method in class weka.classifiers.meta. Dagging 
 Gets the verbose state
   getVersion()   - 
Method in class weka.core.xml. XMLSerialization 
 returns the WEKA version with which the serialized object was created
   getVisible()   - 
Method in class weka.gui.treevisualizer. Node 
 Get the value of visible.
   getVisibleColCount()   - 
Method in class weka.experiment. ResultMatrix 
 returns the number of visible columns
   getVisibleRowCount()   - 
Method in class weka.experiment. ResultMatrix 
 returns the number of visible rows
   getVisual()   - 
Method in class weka.gui.beans. AbstractDataSink 
 Get the visual being used by this data source.
   getVisual()   - 
Method in class weka.gui.beans. AbstractDataSource 
 Get the visual being used by this data source.
   getVisual()   - 
Method in class weka.gui.beans. AbstractEvaluator 
 Get the visual
   getVisual()   - 
Method in class weka.gui.beans. AbstractTestSetProducer 
 Get the visual for this bean
   getVisual()   - 
Method in class weka.gui.beans. AbstractTrainAndTestSetProducer 
 Get the visual for this bean
   getVisual()   - 
Method in class weka.gui.beans. AbstractTrainingSetProducer 
 Get the visual for this bean
   getVisual()   - 
Method in class weka.gui.beans. Associator 
 Gets the visual appearance of this wrapper bean
   getVisual()   - 
Method in class weka.gui.beans. ClassAssigner 
  
   getVisual()   - 
Method in class weka.gui.beans. Classifier 
 Gets the visual appearance of this wrapper bean
   getVisual()   - 
Method in class weka.gui.beans. ClassValuePicker 
  
   getVisual()   - 
Method in class weka.gui.beans. Clusterer 
 Gets the visual appearance of this wrapper bean
   getVisual()   - 
Method in class weka.gui.beans. CostBenefitAnalysis 
  
   getVisual()   - 
Method in class weka.gui.beans. DataVisualizer 
 Return the visual appearance of this bean
   getVisual()   - 
Method in class weka.gui.beans. Filter 
 Get the visual appearance of this bean
   getVisual()   - 
Method in class weka.gui.beans. GraphViewer 
 Get the visual appearance of this bean
   getVisual()   - 
Method in class weka.gui.beans. InstanceStreamToBatchMaker 
 Gets the visual appearance of this wrapper bean
   getVisual()   - 
Method in class weka.gui.beans. MetaBean 
 Gets the visual appearance of this wrapper bean
   getVisual()   - 
Method in class weka.gui.beans. ModelPerformanceChart 
 Return the visual appearance of this bean
   getVisual()   - 
Method in class weka.gui.beans. PredictionAppender 
 Get the visual being used by this data source.
   getVisual()   - 
Method in class weka.gui.beans. SerializedModelSaver 
 Get the visual being used by this data source.
   getVisual()   - 
Method in class weka.gui.beans. StripChart 
 Get the visual appearance of this bean
   getVisual()   - 
Method in class weka.gui.beans. TextViewer 
 Get the visual appearance of this bean
   getVisual()   - 
Method in interface weka.gui.beans. Visible 
 Get the visual representation
   getVisualizeMenuItem(FastVector, Attribute)   - 
Method in interface weka.gui.visualize.plugins. VisualizePlugin 
 Get a JMenu or JMenuItem which contain action listeners
 that perform the visualization, using some but not
 necessarily all of the data.
   getVoteFlag()   - 
Method in class weka.datagenerators.classifiers.classification. RDG1 
 Gets the vote flag.
   getWBias()   - 
Method in class weka.classifiers. BVDecomposeSegCVSub 
 Get the calculated bias according to the Webb definition
   getWeight()   - 
Method in class weka.classifiers.bayes. AODE 
 Gets the weight used in m-estimate
   getWeightByConfidence()   - 
Method in class weka.classifiers.misc. VFI 
 Get whether feature intervals are being weighted by confidence
   getWeightByDistance()   - 
Method in class weka.attributeSelection. ReliefFAttributeEval 
 Get whether nearest neighbours are being weighted by distance
   getWeightingKernel()   - 
Method in class weka.classifiers.lazy. LWL 
 Gets the kernel weighting method to use.
   getWeightMethod()   - 
Method in class weka.classifiers.mi. MIWrapper 
 Returns the current weighting method for instances.
   getWeightMethod()   - 
Method in class weka.filters.unsupervised.attribute. MultiInstanceToPropositional 
 Returns the current weighting method for instances.
   getWeights()   - 
Method in class weka.classifiers.functions. LibLINEAR 
 Gets the parameters C of class i to weight[i]*C (default 1).
   getWeights()   - 
Method in class weka.classifiers.functions. LibSVM 
 Gets the parameters C of class i to weight[i]*C, for C-SVC (default 1).
   getWeights()   - 
Method in class weka.classifiers.functions.neural. NeuralNode 
 call this function to get the weights array.
   getWeights()   - 
Method in class weka.estimators. KernelEstimator 
 Return the weights of the kernels.
   getWeights()   - 
Method in interface weka.gui.boundaryvisualizer. DataGenerator 
 Get weights
   getWeights()   - 
Method in class weka.gui.boundaryvisualizer. KDDataGenerator 
  
   getWeightThreshold()   - 
Method in class weka.classifiers.meta. AdaBoostM1 
 Get the degree of weight thresholding
   getWeightThreshold()   - 
Method in class weka.classifiers.meta. LogitBoost 
 Get the degree of weight thresholding
   getWeightTrimBeta()   - 
Method in class weka.classifiers.functions. SimpleLogistic 
 Get the value of weightTrimBeta.
   getWeightTrimBeta()   - 
Method in class weka.classifiers.trees. FT 
 Get the value of weightTrimBeta.
   getWeightTrimBeta()   - 
Method in class weka.classifiers.trees. LMT 
 Get the value of weightTrimBeta.
   getWeightTrimBeta()   - 
Method in class weka.classifiers.trees.lmt. LogisticBase 
 Get the value of weightTrimBeta.
   getWholeDataErr()   - 
Method in class weka.classifiers.rules. Ridor 
  
   getWidth()   - 
Method in class weka.gui.beans. BeanInstance 
 Gets the width of this bean
   getWindow(Class)   - 
Method in class weka.gui. Main 
 returns the first instance of the given window class, null if none can be 
 found.
   getWindow(String)   - 
Method in class weka.gui. Main 
 returns the first window with the given title, null if none can be 
 found.
   getWindowList()   - 
Method in class weka.gui. Main 
 returns all currently open frames.
   getWindowSize()   - 
Method in class weka.classifiers.lazy. IBk 
 Gets the maximum number of instances allowed in the training
 pool.
   getWithPrefix(String)   - 
Method in class weka.core. Trie 
 returns all stored strings that match the given prefix
   getWords()   - 
Method in class weka.core. CheckScheme 
 returns the words used for assembling strings in a comma-separated list.
   getWords()   - 
Method in class weka.core. TestInstances 
 returns the words used for assembling strings in a comma-separated list.
   getWordSeparators()   - 
Method in class weka.core. CheckScheme 
 returns the word separators (chars) to use for assembling strings.
   getWordSeparators()   - 
Method in class weka.core. TestInstances 
 returns the word separators (chars) to use for assembling strings.
   getWordsToKeep()   - 
Method in class weka.filters.unsupervised.attribute. StringToWordVector 
 Gets the number of words (per class if there is a class attribute
 assigned) to attempt to keep.
   getWrappedAlgorithm()   - 
Method in class weka.gui.beans. Associator 
 Returns the wrapped associator
   getWrappedAlgorithm()   - 
Method in class weka.gui.beans. Classifier 
 Returns the wrapped classifier
   getWrappedAlgorithm()   - 
Method in class weka.gui.beans. Clusterer 
 Returns the wrapped clusterer
   getWrappedAlgorithm()   - 
Method in class weka.gui.beans. Filter 
 Get the filter wrapped by this bean
   getWrappedAlgorithm()   - 
Method in class weka.gui.beans. Loader 
 Get the loader
   getWrappedAlgorithm()   - 
Method in class weka.gui.beans. Saver 
 Get the saver
   getWrappedAlgorithm()   - 
Method in interface weka.gui.beans. WekaWrapper 
 Get the algorithm
   getWriteMode()   - 
Method in class weka.core.converters. AbstractSaver 
 Gets the write mode.
   getWriteMode()   - 
Method in interface weka.core.converters. Saver 
 Gets the write mode
   getWriteOPTICSresults()   - 
Method in class weka.clusterers. OPTICS 
 Returns the flag for writing actions
   getWriter()   - 
Method in class weka.core.converters. AbstractFileSaver 
 Gets the writer
   getWriter(String)   - 
Method in class weka.gui.visualize. PrintableComponent 
 returns the JComponentWriter associated with the given name, is 
  null  if not found.
   getWriter(String)   - 
Method in interface weka.gui.visualize. PrintableHandler 
 returns the JComponentWriter associated with the given name, is 
  null  if not found
   getWriter(String)   - 
Method in class weka.gui.visualize. PrintablePanel 
 returns the JComponentWriter associated with the given name, is 
  null  if not found
   getWriters()   - 
Method in class weka.gui.visualize. PrintableComponent 
 returns a Hashtable with the current available JComponentWriters in the 
 save dialog.
   getWriters()   - 
Method in interface weka.gui.visualize. PrintableHandler 
 returns a Hashtable with the current available JComponentWriters in the 
 save dialog.
   getWriters()   - 
Method in class weka.gui.visualize. PrintablePanel 
 returns a Hashtable with the current available JComponentWriters in the 
 save dialog.
   getWVariance()   - 
Method in class weka.classifiers. BVDecomposeSegCVSub 
 Get the calculated variance according to the Webb definition
   getX()   - 
Method in class weka.classifiers.functions.neural. NeuralConnection 
  
   getX()   - 
Method in class weka.gui.beans. BeanInstance 
 Gets the x coordinate of this bean
   getXBase()   - 
Method in class weka.classifiers.meta. GridSearch 
 Get the value of the base for X.
   getXExpression()   - 
Method in class weka.classifiers.meta. GridSearch 
 Get the expression for the X value.
   getXindex()   - 
Method in class weka.gui.visualize. PlotData2D 
 Get the currently set x index of the data
   getXIndex()   - 
Method in class weka.gui.visualize. VisualizePanel 
 Get the index of the attribute on the x axis
   getXLabelFreq()   - 
Method in class weka.gui.beans. StripChart 
 Get the frequency by which x axis values are printed
   getXMax()   - 
Method in class weka.classifiers.meta. GridSearch 
 Get the value of the Maximum of X.
   getXMin()   - 
Method in class weka.classifiers.meta. GridSearch 
 Get the value of the minimum of X.
   getXMLDocument()   - 
Method in class weka.core.xml. XMLOptions 
 returns the handler of the XML document.
   getXProperty()   - 
Method in class weka.classifiers.meta. GridSearch 
 Get the X property to test (normally the filter).
   getXScale()   - 
Method in class weka.gui.visualize. JComponentWriter 
 returns the scale factor for the x-axis
   getXScale()   - 
Method in class weka.gui.visualize. PrintableComponent 
 returns the scale factor for the x-axis.
   getXScale()   - 
Method in interface weka.gui.visualize. PrintableHandler 
 returns the scale factor for the x-axis
   getXScale()   - 
Method in class weka.gui.visualize. PrintablePanel 
 returns the scale factor for the x-axis
   getXStep()   - 
Method in class weka.classifiers.meta. GridSearch 
 Get the value of the step size for X.
   getY()   - 
Method in class weka.classifiers.functions.neural. NeuralConnection 
  
   getY()   - 
Method in class weka.gui.beans. BeanInstance 
 Gets the y coordinate of this bean
   getYBase()   - 
Method in class weka.classifiers.meta. GridSearch 
 Get the value of the base for Y.
   getYExpression()   - 
Method in class weka.classifiers.meta. GridSearch 
 Get the expression for the Y value.
   getYindex()   - 
Method in class weka.gui.visualize. PlotData2D 
 Get the currently set y index of the data
   getYIndex()   - 
Method in class weka.gui.visualize. VisualizePanel 
 Get the index of the attribute on the y axis
   getYMax()   - 
Method in class weka.classifiers.meta. GridSearch 
 Get the value of the Maximum of Y.
   getYMin()   - 
Method in class weka.classifiers.meta. GridSearch 
 Get the value of the minimum of Y.
   getYProperty()   - 
Method in class weka.classifiers.meta. GridSearch 
 Get the Y property (normally the classifier).
   getYScale()   - 
Method in class weka.gui.visualize. JComponentWriter 
 returns the scale factor for the y-axis
   getYScale()   - 
Method in class weka.gui.visualize. PrintableComponent 
 returns the scale factor for the y-axis.
   getYScale()   - 
Method in interface weka.gui.visualize. PrintableHandler 
 returns the scale factor for the y-axis
   getYScale()   - 
Method in class weka.gui.visualize. PrintablePanel 
 returns the scale factor for the y-axis
   getYStep()   - 
Method in class weka.classifiers.meta. GridSearch 
 Get the value of the step size for Y.
   globalBlendTipText()   - 
Method in class weka.classifiers.lazy. KStar 
 Returns the tip text for this property
   globalInfo()   - 
Method in class weka.associations. Apriori 
 Returns a string describing this associator
   globalInfo()   - 
Method in class weka.associations. FilteredAssociator 
 Returns a string describing this Associator
   globalInfo()   - 
Method in class weka.associations. FPGrowth 
 Returns a string describing this associator
   globalInfo()   - 
Method in class weka.associations. GeneralizedSequentialPatterns 
 Returns global information about the algorithm.
   globalInfo()   - 
Method in class weka.associations. PredictiveApriori 
 Returns a string describing this associator
   globalInfo()   - 
Method in class weka.associations. Tertius 
 Returns a string describing this associator.
   globalInfo()   - 
Method in class weka.attributeSelection. BestFirst 
 Returns a string describing this search method
   globalInfo()   - 
Method in class weka.attributeSelection. CfsSubsetEval 
 Returns a string describing this attribute evaluator
   globalInfo()   - 
Method in class weka.attributeSelection. ChiSquaredAttributeEval 
 Returns a string describing this attribute evaluator
   globalInfo()   - 
Method in class weka.attributeSelection. ClassifierSubsetEval 
 Returns a string describing this attribute evaluator
   globalInfo()   - 
Method in class weka.attributeSelection. ConsistencySubsetEval 
 Returns a string describing this search method
   globalInfo()   - 
Method in class weka.attributeSelection. CostSensitiveASEvaluation 
  
   globalInfo()   - 
Method in class weka.attributeSelection. ExhaustiveSearch 
 Returns a string describing this search method
   globalInfo()   - 
Method in class weka.attributeSelection. FilteredAttributeEval 
  
   globalInfo()   - 
Method in class weka.attributeSelection. FilteredSubsetEval 
  
   globalInfo()   - 
Method in class weka.attributeSelection. GainRatioAttributeEval 
 Returns a string describing this attribute evaluator
   globalInfo()   - 
Method in class weka.attributeSelection. GeneticSearch 
 Returns a string describing this search method
   globalInfo()   - 
Method in class weka.attributeSelection. GreedyStepwise 
 Returns a string describing this search method
   globalInfo()   - 
Method in class weka.attributeSelection. InfoGainAttributeEval 
 Returns a string describing this attribute evaluator
   globalInfo()   - 
Method in class weka.attributeSelection. LatentSemanticAnalysis 
 Returns a string describing this attribute transformer
   globalInfo()   - 
Method in class weka.attributeSelection. LinearForwardSelection 
 Returns a string describing this search method
   globalInfo()   - 
Method in class weka.attributeSelection. OneRAttributeEval 
 Returns a string describing this attribute evaluator
   globalInfo()   - 
Method in class weka.attributeSelection. PrincipalComponents 
 Returns a string describing this attribute transformer
   globalInfo()   - 
Method in class weka.attributeSelection. RaceSearch 
 Returns a string describing this search method
   globalInfo()   - 
Method in class weka.attributeSelection. RandomSearch 
 Returns a string describing this search method
   globalInfo()   - 
Method in class weka.attributeSelection. Ranker 
 Returns a string describing this search method
   globalInfo()   - 
Method in class weka.attributeSelection. RankSearch 
 Returns a string describing this search method
   globalInfo()   - 
Method in class weka.attributeSelection. ReliefFAttributeEval 
 Returns a string describing this attribute evaluator
   globalInfo()   - 
Method in class weka.attributeSelection. ScatterSearchV1 
 Returns a string describing this search method
   globalInfo()   - 
Method in class weka.attributeSelection. SubsetSizeForwardSelection 
 Returns a string describing this search method
   globalInfo()   - 
Method in class weka.attributeSelection. SVMAttributeEval 
 Returns a string describing this attribute evaluator
   globalInfo()   - 
Method in class weka.attributeSelection. SymmetricalUncertAttributeEval 
 Returns a string describing this attribute evaluator
   globalInfo()   - 
Method in class weka.attributeSelection. WrapperSubsetEval 
 Returns a string describing this attribute evaluator
   globalInfo()   - 
Method in class weka.classifiers.bayes. AODE 
 Returns a string describing this classifier
   globalInfo()   - 
Method in class weka.classifiers.bayes. AODEsr 
 Returns a string describing this classifier
   globalInfo()   - 
Method in class weka.classifiers.bayes. BayesianLogisticRegression 
  
   globalInfo()   - 
Method in class weka.classifiers.bayes. BayesNet 
 This will return a string describing the classifier.
   globalInfo()   - 
Method in class weka.classifiers.bayes. ComplementNaiveBayes 
 Returns a string describing this classifier
   globalInfo()   - 
Method in class weka.classifiers.bayes. DMNBtext 
 Returns a string describing this classifier
   globalInfo()   - 
Method in class weka.classifiers.bayes. HNB 
 Returns a string describing this classifier.
   globalInfo()   - 
Method in class weka.classifiers.bayes. NaiveBayes 
 Returns a string describing this classifier
   globalInfo()   - 
Method in class weka.classifiers.bayes. NaiveBayesMultinomial 
 Returns a string describing this classifier
   globalInfo()   - 
Method in class weka.classifiers.bayes. NaiveBayesMultinomialUpdateable 
 Returns a string describing this classifier
   globalInfo()   - 
Method in class weka.classifiers.bayes. NaiveBayesSimple 
 Returns a string describing this classifier
   globalInfo()   - 
Method in class weka.classifiers.bayes. NaiveBayesUpdateable 
 Returns a string describing this classifier
   globalInfo()   - 
Method in class weka.classifiers.bayes.net. BIFReader 
 This will return a string describing the classifier.
   globalInfo()   - 
Method in class weka.classifiers.bayes.net.estimate. BayesNetEstimator 
 This will return a string describing the class.
   globalInfo()   - 
Method in class weka.classifiers.bayes.net.estimate. BMAEstimator 
 Returns a string describing this object
   globalInfo()   - 
Method in class weka.classifiers.bayes.net.estimate. MultiNomialBMAEstimator 
 Returns a string describing this object
   globalInfo()   - 
Method in class weka.classifiers.bayes.net.estimate. SimpleEstimator 
 Returns a string describing this object
   globalInfo()   - 
Method in class weka.classifiers.bayes.net.search.ci. CISearchAlgorithm 
 Returns a string describing this object
   globalInfo()   - 
Method in class weka.classifiers.bayes.net.search.ci. ICSSearchAlgorithm 
 This will return a string describing the search algorithm.
   globalInfo()   - 
Method in class weka.classifiers.bayes.net.search.fixed. FromFile 
 Returns a string describing this object
   globalInfo()   - 
Method in class weka.classifiers.bayes.net.search.fixed. NaiveBayes 
 Returns a string describing this object
   globalInfo()   - 
Method in class weka.classifiers.bayes.net.search.global. GeneticSearch 
 This will return a string describing the classifier.
   globalInfo()   - 
Method in class weka.classifiers.bayes.net.search.global. GlobalScoreSearchAlgorithm 
 This will return a string describing the search algorithm.
   globalInfo()   - 
Method in class weka.classifiers.bayes.net.search.global. HillClimber 
 This will return a string describing the search algorithm.
   globalInfo()   - 
Method in class weka.classifiers.bayes.net.search.global. K2 
 This will return a string describing the search algorithm.
   globalInfo()   - 
Method in class weka.classifiers.bayes.net.search.global. RepeatedHillClimber 
 This will return a string describing the classifier.
   globalInfo()   - 
Method in class weka.classifiers.bayes.net.search.global. SimulatedAnnealing 
 This will return a string describing the classifier.
   globalInfo()   - 
Method in class weka.classifiers.bayes.net.search.global. TabuSearch 
 This will return a string describing the classifier.
   globalInfo()   - 
Method in class weka.classifiers.bayes.net.search.global. TAN 
 This will return a string describing the classifier.
   globalInfo()   - 
Method in class weka.classifiers.bayes.net.search.local. GeneticSearch 
 This will return a string describing the classifier.
   globalInfo()   - 
Method in class weka.classifiers.bayes.net.search.local. HillClimber 
 This will return a string describing the search algorithm.
   globalInfo()   - 
Method in class weka.classifiers.bayes.net.search.local. K2 
 This will return a string describing the search algorithm.
   globalInfo()   - 
Method in class weka.classifiers.bayes.net.search.local. LAGDHillClimber 
 This will return a string describing the search algorithm.
   globalInfo()   - 
Method in class weka.classifiers.bayes.net.search.local. LocalScoreSearchAlgorithm 
 This will return a string describing the search algorithm.
   globalInfo()   - 
Method in class weka.classifiers.bayes.net.search.local. RepeatedHillClimber 
 This will return a string describing the classifier.
   globalInfo()   - 
Method in class weka.classifiers.bayes.net.search.local. SimulatedAnnealing 
 This will return a string describing the classifier.
   globalInfo()   - 
Method in class weka.classifiers.bayes.net.search.local. TabuSearch 
 This will return a string describing the classifier.
   globalInfo()   - 
Method in class weka.classifiers.bayes.net.search.local. TAN 
 This will return a string describing the classifier.
   globalInfo()   - 
Method in class weka.classifiers.bayes. WAODE 
 Returns a string describing this classifier
   globalInfo()   - 
Method in class weka.classifiers. BVDecompose 
 Returns a string describing this object
   globalInfo()   - 
Method in class weka.classifiers. BVDecomposeSegCVSub 
 Returns a string describing this object
   globalInfo()   - 
Method in class weka.classifiers.functions. GaussianProcesses 
 Returns a string describing classifier
   globalInfo()   - 
Method in class weka.classifiers.functions. IsotonicRegression 
 Returns a string describing this classifier
   globalInfo()   - 
Method in class weka.classifiers.functions. LeastMedSq 
 Returns a string describing this classifier
   globalInfo()   - 
Method in class weka.classifiers.functions. LibLINEAR 
 Returns a string describing classifier
   globalInfo()   - 
Method in class weka.classifiers.functions. LibSVM 
 Returns a string describing classifier
   globalInfo()   - 
Method in class weka.classifiers.functions. LinearRegression 
 Returns a string describing this classifier
   globalInfo()   - 
Method in class weka.classifiers.functions. Logistic 
 Returns a string describing this classifier
   globalInfo()   - 
Method in class weka.classifiers.functions. MultilayerPerceptron 
 This will return a string describing the classifier.
   globalInfo()   - 
Method in class weka.classifiers.functions. PaceRegression 
 Returns a string describing this classifier
   globalInfo()   - 
Method in class weka.classifiers.functions. PLSClassifier 
 Returns a string describing classifier
   globalInfo()   - 
Method in class weka.classifiers.functions. RBFNetwork 
 Returns a string describing this classifier
   globalInfo()   - 
Method in class weka.classifiers.functions. SimpleLinearRegression 
 Returns a string describing this classifier
   globalInfo()   - 
Method in class weka.classifiers.functions. SimpleLogistic 
 Returns a string describing classifier
   globalInfo()   - 
Method in class weka.classifiers.functions. SMO 
 Returns a string describing classifier
   globalInfo()   - 
Method in class weka.classifiers.functions. SMOreg 
 Returns a string describing classifier
   globalInfo()   - 
Method in class weka.classifiers.functions. SPegasos 
 Returns a string describing classifier
   globalInfo()   - 
Method in class weka.classifiers.functions.supportVector. Kernel 
 Returns a string describing the kernel
   globalInfo()   - 
Method in class weka.classifiers.functions.supportVector. NormalizedPolyKernel 
 Returns a string describing the kernel
   globalInfo()   - 
Method in class weka.classifiers.functions.supportVector. PolyKernel 
 Returns a string describing the kernel
   globalInfo()   - 
Method in class weka.classifiers.functions.supportVector. PrecomputedKernelMatrixKernel 
 Returns a string describing the kernel
   globalInfo()   - 
Method in class weka.classifiers.functions.supportVector. Puk 
 Returns a string describing the kernel
   globalInfo()   - 
Method in class weka.classifiers.functions.supportVector. RBFKernel 
 Returns a string describing the kernel
   globalInfo()   - 
Method in class weka.classifiers.functions.supportVector. RegSMO 
 Returns a string describing classifier
   globalInfo()   - 
Method in class weka.classifiers.functions.supportVector. RegSMOImproved 
 Returns a string describing the object
   globalInfo()   - 
Method in class weka.classifiers.functions.supportVector. StringKernel 
 Returns a string describing the kernel
   globalInfo()   - 
Method in class weka.classifiers.functions. VotedPerceptron 
 Returns a string describing this classifier
   globalInfo()   - 
Method in class weka.classifiers.functions. Winnow 
 Returns a string describing classifier
   globalInfo()   - 
Method in class weka.classifiers.lazy. IB1 
 Returns a string describing classifier
   globalInfo()   - 
Method in class weka.classifiers.lazy. IBk 
 Returns a string describing classifier.
   globalInfo()   - 
Method in class weka.classifiers.lazy. KStar 
 Returns a string describing classifier
   globalInfo()   - 
Method in class weka.classifiers.lazy. LBR 
  
   globalInfo()   - 
Method in class weka.classifiers.lazy. LWL 
 Returns a string describing classifier.
   globalInfo()   - 
Method in class weka.classifiers.meta. AdaBoostM1 
 Returns a string describing classifier
   globalInfo()   - 
Method in class weka.classifiers.meta. AdditiveRegression 
 Returns a string describing this attribute evaluator
   globalInfo()   - 
Method in class weka.classifiers.meta. AttributeSelectedClassifier 
 Returns a string describing this search method
   globalInfo()   - 
Method in class weka.classifiers.meta. Bagging 
 Returns a string describing classifier
   globalInfo()   - 
Method in class weka.classifiers.meta. ClassificationViaClustering 
 Returns a string describing classifier
   globalInfo()   - 
Method in class weka.classifiers.meta. ClassificationViaRegression 
 Returns a string describing classifier
   globalInfo()   - 
Method in class weka.classifiers.meta. CostSensitiveClassifier 
  
   globalInfo()   - 
Method in class weka.classifiers.meta. CVParameterSelection 
 Returns a string describing this classifier
   globalInfo()   - 
Method in class weka.classifiers.meta. Dagging 
 Returns a string describing classifier
   globalInfo()   - 
Method in class weka.classifiers.meta. Decorate 
 Returns a string describing classifier
   globalInfo()   - 
Method in class weka.classifiers.meta. END 
 Returns a string describing classifier
   globalInfo()   - 
Method in class weka.classifiers.meta. FilteredClassifier 
 Returns a string describing this classifier
   globalInfo()   - 
Method in class weka.classifiers.meta. Grading 
 Returns a string describing classifier
   globalInfo()   - 
Method in class weka.classifiers.meta. GridSearch 
 Returns a string describing classifier
   globalInfo()   - 
Method in class weka.classifiers.meta. LogitBoost 
 Returns a string describing classifier
   globalInfo()   - 
Method in class weka.classifiers.meta. MetaCost 
 Returns a string describing classifier
   globalInfo()   - 
Method in class weka.classifiers.meta. MultiBoostAB 
 Returns a string describing classifier
   globalInfo()   - 
Method in class weka.classifiers.meta. MultiClassClassifier 
  
   globalInfo()   - 
Method in class weka.classifiers.meta. MultiScheme 
 Returns a string describing classifier
   globalInfo()   - 
Method in class weka.classifiers.meta.nestedDichotomies. ClassBalancedND 
  
   globalInfo()   - 
Method in class weka.classifiers.meta.nestedDichotomies. DataNearBalancedND 
  
   globalInfo()   - 
Method in class weka.classifiers.meta.nestedDichotomies. ND 
  
   globalInfo()   - 
Method in class weka.classifiers.meta. OrdinalClassClassifier 
 Returns a string describing this attribute evaluator
   globalInfo()   - 
Method in class weka.classifiers.meta. RacedIncrementalLogitBoost 
  
   globalInfo()   - 
Method in class weka.classifiers.meta. RandomCommittee 
 Returns a string describing classifier
   globalInfo()   - 
Method in class weka.classifiers.meta. RandomSubSpace 
 Returns a string describing classifier
   globalInfo()   - 
Method in class weka.classifiers.meta. RegressionByDiscretization 
 Returns a string describing classifier
   globalInfo()   - 
Method in class weka.classifiers.meta. RotationForest 
 Returns a string describing classifier
   globalInfo()   - 
Method in class weka.classifiers.meta. Stacking 
 Returns a string describing classifier
   globalInfo()   - 
Method in class weka.classifiers.meta. StackingC 
 Returns a string describing classifier
   globalInfo()   - 
Method in class weka.classifiers.meta. ThresholdSelector 
  
   globalInfo()   - 
Method in class weka.classifiers.meta. Vote 
 Returns a string describing classifier
   globalInfo()   - 
Method in class weka.classifiers.mi. CitationKNN 
 Returns a string describing this filter
   globalInfo()   - 
Method in class weka.classifiers.mi. MDD 
 Returns a string describing this filter
   globalInfo()   - 
Method in class weka.classifiers.mi. MIBoost 
 Returns a string describing this filter
   globalInfo()   - 
Method in class weka.classifiers.mi. MIDD 
 Returns a string describing this filter
   globalInfo()   - 
Method in class weka.classifiers.mi. MIEMDD 
 Returns a string describing this filter
   globalInfo()   - 
Method in class weka.classifiers.mi. MILR 
 Returns the tip text for this property
   globalInfo()   - 
Method in class weka.classifiers.mi. MINND 
 Returns a string describing this filter
   globalInfo()   - 
Method in class weka.classifiers.mi. MIOptimalBall 
 Returns a string describing this filter
   globalInfo()   - 
Method in class weka.classifiers.mi. MISMO 
 Returns a string describing classifier
   globalInfo()   - 
Method in class weka.classifiers.mi. MISVM 
 Returns a string describing this filter
   globalInfo()   - 
Method in class weka.classifiers.mi. MIWrapper 
 Returns a string describing this filter
   globalInfo()   - 
Method in class weka.classifiers.mi. SimpleMI 
 Returns a string describing this filter
   globalInfo()   - 
Method in class weka.classifiers.misc. HyperPipes 
 Returns a string describing classifier
   globalInfo()   - 
Method in class weka.classifiers.misc. SerializedClassifier 
 Returns a string describing classifier
   globalInfo()   - 
Method in class weka.classifiers.misc. VFI 
 Returns a string describing this search method
   globalInfo()   - 
Method in class weka.classifiers.rules. ConjunctiveRule 
 Returns a string describing classifier
   globalInfo()   - 
Method in class weka.classifiers.rules. DecisionTable 
 Returns a string describing classifier
   globalInfo()   - 
Method in class weka.classifiers.rules. DTNB 
 Returns a string describing classifier
   globalInfo()   - 
Method in class weka.classifiers.rules. JRip 
 Returns a string describing classifier
   globalInfo()   - 
Method in class weka.classifiers.rules. M5Rules 
 Returns a string describing classifier
   globalInfo()   - 
Method in class weka.classifiers.rules. NNge 
 Returns a string describing classifier
   globalInfo()   - 
Method in class weka.classifiers.rules. OneR 
 Returns a string describing classifier
   globalInfo()   - 
Method in class weka.classifiers.rules. PART 
 Returns a string describing classifier
   globalInfo()   - 
Method in class weka.classifiers.rules. Prism 
 Returns a string describing classifier
   globalInfo()   - 
Method in class weka.classifiers.rules. Ridor 
 Returns a string describing classifier
   globalInfo()   - 
Method in class weka.classifiers.rules. ZeroR 
 Returns a string describing classifier
   globalInfo()   - 
Method in class weka.classifiers.trees. ADTree 
 Returns a string describing classifier
   globalInfo()   - 
Method in class weka.classifiers.trees. BFTree 
 Returns a string describing classifier
   globalInfo()   - 
Method in class weka.classifiers.trees. DecisionStump 
 Returns a string describing classifier
   globalInfo()   - 
Method in class weka.classifiers.trees. FT 
 Returns a string describing classifier
   globalInfo()   - 
Method in class weka.classifiers.trees. Id3 
 Returns a string describing the classifier.
   globalInfo()   - 
Method in class weka.classifiers.trees. J48 
 Returns a string describing classifier
   globalInfo()   - 
Method in class weka.classifiers.trees. J48graft 
 Returns a string describing classifier
   globalInfo()   - 
Method in class weka.classifiers.trees. LADTree 
 Returns a string describing classifier
   globalInfo()   - 
Method in class weka.classifiers.trees. LMT 
 Returns a string describing classifier
   globalInfo()   - 
Method in class weka.classifiers.trees.m5. M5Base 
 returns information about the classifier
   globalInfo()   - 
Method in class weka.classifiers.trees. NBTree 
 Returns a string describing classifier
   globalInfo()   - 
Method in class weka.classifiers.trees. RandomForest 
 Returns a string describing classifier
   globalInfo()   - 
Method in class weka.classifiers.trees. RandomTree 
 Returns a string describing classifier
   globalInfo()   - 
Method in class weka.classifiers.trees. REPTree 
 Returns a string describing classifier
   globalInfo()   - 
Method in class weka.classifiers.trees. SimpleCart 
 Return a description suitable for displaying in the explorer/experimenter.
   globalInfo()   - 
Method in class weka.classifiers.trees. UserClassifier 
 This will return a string describing the classifier.
   globalInfo()   - 
Method in class weka.clusterers. CLOPE 
 Returns a string describing this DataMining-Algorithm
   globalInfo()   - 
Method in class weka.clusterers. Cobweb 
 Returns a string describing this clusterer
   globalInfo()   - 
Method in class weka.clusterers. DBScan 
 Returns a string describing this DataMining-Algorithm
   globalInfo()   - 
Method in class weka.clusterers. EM 
 Returns a string describing this clusterer
   globalInfo()   - 
Method in class weka.clusterers. FarthestFirst 
 Returns a string describing this clusterer
   globalInfo()   - 
Method in class weka.clusterers. FilteredClusterer 
 Returns a string describing this clusterer.
   globalInfo()   - 
Method in class weka.clusterers. HierarchicalClusterer 
 This will return a string describing the clusterer.
   globalInfo()   - 
Method in class weka.clusterers. MakeDensityBasedClusterer 
 Returns a string describing classifier
   globalInfo()   - 
Method in class weka.clusterers. OPTICS 
 Returns a string describing this DataMining-Algorithm
   globalInfo()   - 
Method in class weka.clusterers. sIB 
 Returns a string describing this clusterer
   globalInfo()   - 
Method in class weka.clusterers. SimpleKMeans 
 Returns a string describing this clusterer
   globalInfo()   - 
Method in class weka.clusterers. XMeans 
 Returns a string describing this clusterer.
   globalInfo()   - 
Method in class weka.core. ChebyshevDistance 
 Returns a string describing this object.
   globalInfo()   - 
Method in class weka.core.converters. ArffLoader 
 Returns a string describing this Loader
   globalInfo()   - 
Method in class weka.core.converters. ArffSaver 
 Returns a string describing this Saver
   globalInfo()   - 
Method in class weka.core.converters. C45Loader 
 Returns a string describing this attribute evaluator
   globalInfo()   - 
Method in class weka.core.converters. C45Saver 
 Returns a string describing this Saver
   globalInfo()   - 
Method in class weka.core.converters. CSVLoader 
 Returns a string describing this attribute evaluator.
   globalInfo()   - 
Method in class weka.core.converters. CSVSaver 
 Returns a string describing this Saver
   globalInfo()   - 
Method in class weka.core.converters. DatabaseLoader 
 Returns a string describing this Loader
   globalInfo()   - 
Method in class weka.core.converters. DatabaseSaver 
 Returns a string describing this Saver.
   globalInfo()   - 
Method in class weka.core.converters. LibSVMLoader 
 Returns a string describing this Loader.
   globalInfo()   - 
Method in class weka.core.converters. LibSVMSaver 
 Returns a string describing this Saver
   globalInfo()   - 
Method in class weka.core.converters. SerializedInstancesLoader 
 Returns a string describing this object
   globalInfo()   - 
Method in class weka.core.converters. SerializedInstancesSaver 
 Returns a string describing this Saver.
   globalInfo()   - 
Method in class weka.core.converters. SVMLightLoader 
 Returns a string describing this Loader.
   globalInfo()   - 
Method in class weka.core.converters. SVMLightSaver 
 Returns a string describing this Saver.
   globalInfo()   - 
Method in class weka.core.converters. TextDirectoryLoader 
 Returns a string describing this loader
   globalInfo()   - 
Method in class weka.core.converters. XRFFLoader 
 Returns a string describing this Loader
   globalInfo()   - 
Method in class weka.core.converters. XRFFSaver 
 Returns a string describing this Saver
   globalInfo()   - 
Method in class weka.core. EditDistance 
 Returns a string describing this object.
   globalInfo()   - 
Method in class weka.core. EuclideanDistance 
 Returns a string describing this object.
   globalInfo()   - 
Method in class weka.core. ManhattanDistance 
 Returns a string describing this object.
   globalInfo()   - 
Method in class weka.core.neighboursearch. BallTree 
 Returns a string describing this nearest neighbour search algorithm.
   globalInfo()   - 
Method in class weka.core.neighboursearch.balltrees. BottomUpConstructor 
 Returns a string describing this nearest neighbour search algorithm.
   globalInfo()   - 
Method in class weka.core.neighboursearch.balltrees. MedianDistanceFromArbitraryPoint 
 Returns a string describing this nearest neighbour search algorithm.
   globalInfo()   - 
Method in class weka.core.neighboursearch.balltrees. MedianOfWidestDimension 
 Returns a string describing this nearest neighbour search algorithm.
   globalInfo()   - 
Method in class weka.core.neighboursearch.balltrees. MiddleOutConstructor 
 Returns a string describing this nearest neighbour search algorithm.
   globalInfo()   - 
Method in class weka.core.neighboursearch.balltrees. PointsClosestToFurthestChildren 
 Returns a string describing this object.
   globalInfo()   - 
Method in class weka.core.neighboursearch.balltrees. TopDownConstructor 
 Returns a string describing this nearest neighbour search algorithm.
   globalInfo()   - 
Method in class weka.core.neighboursearch. CoverTree 
 Returns a string describing this nearest neighbour search algorithm.
   globalInfo()   - 
Method in class weka.core.neighboursearch. KDTree 
 Returns a string describing this nearest neighbour search algorithm.
   globalInfo()   - 
Method in class weka.core.neighboursearch.kdtrees. KMeansInpiredMethod 
 Returns a string describing this nearest neighbour search algorithm.
   globalInfo()   - 
Method in class weka.core.neighboursearch.kdtrees. MedianOfWidestDimension 
 Returns a string describing this nearest neighbour search algorithm.
   globalInfo()   - 
Method in class weka.core.neighboursearch.kdtrees. MidPointOfWidestDimension 
 Returns a string describing this nearest neighbour search algorithm.
   globalInfo()   - 
Method in class weka.core.neighboursearch.kdtrees. SlidingMidPointOfWidestSide 
 Returns a string describing this nearest neighbour search algorithm.
   globalInfo()   - 
Method in class weka.core.neighboursearch. LinearNNSearch 
 Returns a string describing this nearest neighbour search algorithm.
   globalInfo()   - 
Method in class weka.core.neighboursearch. NearestNeighbourSearch 
 Returns a string describing this nearest neighbour search algorithm.
   globalInfo()   - 
Method in class weka.core. NormalizableDistance 
 Returns a string describing this object.
   globalInfo()   - 
Method in class weka.core.stemmers. IteratedLovinsStemmer 
 Returns a string describing the stemmer
   globalInfo()   - 
Method in class weka.core.stemmers. LovinsStemmer 
 Returns a string describing the stemmer
   globalInfo()   - 
Method in class weka.core.stemmers. NullStemmer 
 Returns a string describing the stemmer
   globalInfo()   - 
Method in class weka.core.stemmers. SnowballStemmer 
 Returns a string describing the stemmer.
   globalInfo()   - 
Method in class weka.core.tokenizers. AlphabeticTokenizer 
 Returns a string describing the stemmer
   globalInfo()   - 
Method in class weka.core.tokenizers. NGramTokenizer 
 Returns a string describing the stemmer
   globalInfo()   - 
Method in class weka.core.tokenizers. Tokenizer 
 Returns a string describing the stemmer
   globalInfo()   - 
Method in class weka.core.tokenizers. WordTokenizer 
 Returns a string describing the stemmer
   globalInfo()   - 
Method in class weka.datagenerators.classifiers.classification. Agrawal 
 Returns a string describing this data generator.
   globalInfo()   - 
Method in class weka.datagenerators.classifiers.classification. BayesNet 
 Returns a string describing this data generator.
   globalInfo()   - 
Method in class weka.datagenerators.classifiers.classification. LED24 
 Returns a string describing this data generator.
   globalInfo()   - 
Method in class weka.datagenerators.classifiers.classification. RandomRBF 
 Returns a string describing this data generator.
   globalInfo()   - 
Method in class weka.datagenerators.classifiers.classification. RDG1 
 Returns a string describing this data generator.
   globalInfo()   - 
Method in class weka.datagenerators.classifiers.regression. Expression 
 Returns a string describing this data generator.
   globalInfo()   - 
Method in class weka.datagenerators.classifiers.regression. MexicanHat 
 Returns a string describing this data generator.
   globalInfo()   - 
Method in class weka.datagenerators. ClusterDefinition 
 Returns a string describing this data generator.
   globalInfo()   - 
Method in class weka.datagenerators.clusterers. BIRCHCluster 
 Returns a string describing this data generator.
   globalInfo()   - 
Method in class weka.datagenerators.clusterers. SubspaceCluster 
 Returns a string describing this data generator.
   globalInfo()   - 
Method in class weka.datagenerators.clusterers. SubspaceClusterDefinition 
 Returns a string describing this data generator.
   globalInfo()   - 
Method in class weka.experiment. AveragingResultProducer 
 Returns a string describing this result producer
   globalInfo()   - 
Method in class weka.experiment. ClassifierSplitEvaluator 
 Returns a string describing this split evaluator
   globalInfo()   - 
Method in class weka.experiment. CostSensitiveClassifierSplitEvaluator 
 Returns a string describing this split evaluator
   globalInfo()   - 
Method in class weka.experiment. CrossValidationResultProducer 
 Returns a string describing this result producer
   globalInfo()   - 
Method in class weka.experiment. CSVResultListener 
 Returns a string describing this result listener
   globalInfo()   - 
Method in class weka.experiment. DatabaseResultListener 
 Returns a string describing this result listener
   globalInfo()   - 
Method in class weka.experiment. DatabaseResultProducer 
 Returns a string describing this result producer
   globalInfo()   - 
Method in class weka.experiment. DensityBasedClustererSplitEvaluator 
 Returns a string describing this split evaluator
   globalInfo()   - 
Method in class weka.experiment. InstancesResultListener 
 Returns a string describing this result listener
   globalInfo()   - 
Method in class weka.experiment. LearningRateResultProducer 
 Returns a string describing this result producer
   globalInfo()   - 
Method in class weka.experiment. RandomSplitResultProducer 
 Returns a string describing this result producer
   globalInfo()   - 
Method in class weka.experiment. RegressionSplitEvaluator 
 Returns a string describing this split evaluator
   globalInfo()   - 
Method in class weka.filters. AllFilter 
 Returns a string describing this filter
   globalInfo()   - 
Method in class weka.filters. MultiFilter 
 Returns a string describing this filter
   globalInfo()   - 
Method in class weka.filters. SimpleFilter 
 Returns a string describing this classifier.
   globalInfo()   - 
Method in class weka.filters.supervised.attribute. AddClassification 
 Returns a string describing this filter.
   globalInfo()   - 
Method in class weka.filters.supervised.attribute. AttributeSelection 
 Returns a string describing this filter
   globalInfo()   - 
Method in class weka.filters.supervised.attribute. ClassOrder 
 Returns a string describing this filter
   globalInfo()   - 
Method in class weka.filters.supervised.attribute. Discretize 
 Returns a string describing this filter
   globalInfo()   - 
Method in class weka.filters.supervised.attribute. NominalToBinary 
 Returns a string describing this filter
   globalInfo()   - 
Method in class weka.filters.supervised.attribute. PLSFilter 
 Returns a string describing this classifier.
   globalInfo()   - 
Method in class weka.filters.supervised.instance. Resample 
 Returns a string describing this filter.
   globalInfo()   - 
Method in class weka.filters.supervised.instance. SMOTE 
 Returns a string describing this classifier.
   globalInfo()   - 
Method in class weka.filters.supervised.instance. SpreadSubsample 
 Returns a string describing this filter
   globalInfo()   - 
Method in class weka.filters.supervised.instance. StratifiedRemoveFolds 
 Returns a string describing this filter
   globalInfo()   - 
Method in class weka.filters.unsupervised.attribute. Add 
 Returns a string describing this filter.
   globalInfo()   - 
Method in class weka.filters.unsupervised.attribute. AddCluster 
 Returns a string describing this filter
   globalInfo()   - 
Method in class weka.filters.unsupervised.attribute. AddExpression 
 Returns a string describing this filter
   globalInfo()   - 
Method in class weka.filters.unsupervised.attribute. AddID 
 Returns a string describing this filter
   globalInfo()   - 
Method in class weka.filters.unsupervised.attribute. AddNoise 
 Returns a string describing this filter
   globalInfo()   - 
Method in class weka.filters.unsupervised.attribute. AddValues 
 Returns a string describing this filter
   globalInfo()   - 
Method in class weka.filters.unsupervised.attribute. Center 
 Returns a string describing this filter
   globalInfo()   - 
Method in class weka.filters.unsupervised.attribute. ChangeDateFormat 
 Returns a string describing this filter
   globalInfo()   - 
Method in class weka.filters.unsupervised.attribute. ClassAssigner 
 Returns a string describing this classifier.
   globalInfo()   - 
Method in class weka.filters.unsupervised.attribute. ClusterMembership 
 Returns a string describing this filter
   globalInfo()   - 
Method in class weka.filters.unsupervised.attribute. Copy 
 Returns a string describing this filter
   globalInfo()   - 
Method in class weka.filters.unsupervised.attribute. Discretize 
 Returns a string describing this filter
   globalInfo()   - 
Method in class weka.filters.unsupervised.attribute. FirstOrder 
 Returns a string describing this filter
   globalInfo()   - 
Method in class weka.filters.unsupervised.attribute. InterquartileRange 
 Returns a string describing this filter
   globalInfo()   - 
Method in class weka.filters.unsupervised.attribute. KernelFilter 
 Returns a string describing this filter.
   globalInfo()   - 
Method in class weka.filters.unsupervised.attribute. MakeIndicator 
  
   globalInfo()   - 
Method in class weka.filters.unsupervised.attribute. MathExpression 
 Returns a string describing this filter
   globalInfo()   - 
Method in class weka.filters.unsupervised.attribute. MergeTwoValues 
 Returns a string describing this filter
   globalInfo()   - 
Method in class weka.filters.unsupervised.attribute. MultiInstanceToPropositional 
 Returns a string describing this filter
   globalInfo()   - 
Method in class weka.filters.unsupervised.attribute. NominalToBinary 
 Returns a string describing this filter
   globalInfo()   - 
Method in class weka.filters.unsupervised.attribute. NominalToString 
 Returns a string describing this filter
   globalInfo()   - 
Method in class weka.filters.unsupervised.attribute. Normalize 
 Returns a string describing this filter.
   globalInfo()   - 
Method in class weka.filters.unsupervised.attribute. NumericCleaner 
 Returns a string describing this filter.
   globalInfo()   - 
Method in class weka.filters.unsupervised.attribute. NumericToBinary 
 Returns a string describing this filter
   globalInfo()   - 
Method in class weka.filters.unsupervised.attribute. NumericToNominal 
 Returns a string describing this filter
   globalInfo()   - 
Method in class weka.filters.unsupervised.attribute. NumericTransform 
 Returns a string describing this filter
   globalInfo()   - 
Method in class weka.filters.unsupervised.attribute. Obfuscate 
 Returns a string describing this filter
   globalInfo()   - 
Method in class weka.filters.unsupervised.attribute. PartitionedMultiFilter 
 Returns a string describing this filter.
   globalInfo()   - 
Method in class weka.filters.unsupervised.attribute. PKIDiscretize 
 Returns a string describing this filter
   globalInfo()   - 
Method in class weka.filters.unsupervised.attribute. PrincipalComponents 
 Returns a string describing this filter.
   globalInfo()   - 
Method in class weka.filters.unsupervised.attribute. PropositionalToMultiInstance 
 Returns a string describing this filter
   globalInfo()   - 
Method in class weka.filters.unsupervised.attribute. RandomProjection 
 Returns a string describing this filter
   globalInfo()   - 
Method in class weka.filters.unsupervised.attribute. RandomSubset 
 Returns a string describing this filter.
   globalInfo()   - 
Method in class weka.filters.unsupervised.attribute. RELAGGS 
 Returns a string describing this filter
   globalInfo()   - 
Method in class weka.filters.unsupervised.attribute. Remove 
 Returns a string describing this filter
   globalInfo()   - 
Method in class weka.filters.unsupervised.attribute. RemoveType 
 Returns a string describing this filter
   globalInfo()   - 
Method in class weka.filters.unsupervised.attribute. RemoveUseless 
 Returns a string describing this filter
   globalInfo()   - 
Method in class weka.filters.unsupervised.attribute. Reorder 
 Returns a string describing this filter
   globalInfo()   - 
Method in class weka.filters.unsupervised.attribute. ReplaceMissingValues 
 Returns a string describing this filter
   globalInfo()   - 
Method in class weka.filters.unsupervised.attribute. Standardize 
 Returns a string describing this filter
   globalInfo()   - 
Method in class weka.filters.unsupervised.attribute. StringToNominal 
 Returns a string describing this filter
   globalInfo()   - 
Method in class weka.filters.unsupervised.attribute. StringToWordVector 
 Returns a string describing this filter.
   globalInfo()   - 
Method in class weka.filters.unsupervised.attribute. SwapValues 
 Returns a string describing this filter
   globalInfo()   - 
Method in class weka.filters.unsupervised.attribute. TimeSeriesDelta 
 Returns a string describing this classifier
   globalInfo()   - 
Method in class weka.filters.unsupervised.attribute. TimeSeriesTranslate 
 Returns a string describing this classifier
   globalInfo()   - 
Method in class weka.filters.unsupervised.attribute. Wavelet 
 Returns a string describing this classifier.
   globalInfo()   - 
Method in class weka.filters.unsupervised.instance. NonSparseToSparse 
 Returns a string describing this filter
   globalInfo()   - 
Method in class weka.filters.unsupervised.instance. Normalize 
 Returns a string describing this filter
   globalInfo()   - 
Method in class weka.filters.unsupervised.instance. Randomize 
 Returns a string describing this classifier
   globalInfo()   - 
Method in class weka.filters.unsupervised.instance. RemoveFolds 
 Returns a string describing this filter
   globalInfo()   - 
Method in class weka.filters.unsupervised.instance. RemoveFrequentValues 
 Returns a string describing this filter
   globalInfo()   - 
Method in class weka.filters.unsupervised.instance. RemoveMisclassified 
 Returns a string describing this filter
   globalInfo()   - 
Method in class weka.filters.unsupervised.instance. RemovePercentage 
 Returns a string describing this filter
   globalInfo()   - 
Method in class weka.filters.unsupervised.instance. RemoveRange 
 Returns a string describing this filter
   globalInfo()   - 
Method in class weka.filters.unsupervised.instance. RemoveWithValues 
 Returns a string describing this classifier
   globalInfo()   - 
Method in class weka.filters.unsupervised.instance. Resample 
 Returns a string describing this classifier
   globalInfo()   - 
Method in class weka.filters.unsupervised.instance. ReservoirSample 
 Returns a string describing this filter
   globalInfo()   - 
Method in class weka.filters.unsupervised.instance. SparseToNonSparse 
 Returns a string describing this filter
   globalInfo()   - 
Method in class weka.filters.unsupervised.instance. SubsetByExpression 
 Returns a string describing this filter.
   globalInfo()   - 
Method in class weka.gui.beans. Associator 
 Global info (if it exists) for the wrapped classifier
   globalInfo()   - 
Method in class weka.gui.beans. AttributeSummarizer 
 Global info for this bean
   globalInfo()   - 
Method in class weka.gui.beans. ClassAssigner 
 Global info for this bean
   globalInfo()   - 
Method in class weka.gui.beans. Classifier 
 Global info (if it exists) for the wrapped classifier
   globalInfo()   - 
Method in class weka.gui.beans. ClassifierPerformanceEvaluator 
 Global info for this bean
   globalInfo()   - 
Method in class weka.gui.beans. ClassValuePicker 
 Global info for this bean
   globalInfo()   - 
Method in class weka.gui.beans. Clusterer 
 Global info (if it exists) for the wrapped classifier
   globalInfo()   - 
Method in class weka.gui.beans. ClustererPerformanceEvaluator 
 Global info for this bean
   globalInfo()   - 
Method in class weka.gui.beans. CostBenefitAnalysis 
 Global info for this bean
   globalInfo()   - 
Method in class weka.gui.beans. CrossValidationFoldMaker 
 Global info for this bean
   globalInfo()   - 
Method in class weka.gui.beans. DataVisualizer 
 Global info for this bean
   globalInfo()   - 
Method in class weka.gui.beans. Filter 
 Global info (if it exists) for the wrapped filter
   globalInfo()   - 
Method in class weka.gui.beans. GraphViewer 
 Global info for this bean
   globalInfo()   - 
Method in class weka.gui.beans. IncrementalClassifierEvaluator 
 Global info for this bean
   globalInfo()   - 
Method in class weka.gui.beans. Loader 
 Global info (if it exists) for the wrapped loader
   globalInfo()   - 
Method in class weka.gui.beans. ModelPerformanceChart 
 Global info for this bean
   globalInfo()   - 
Method in class weka.gui.beans. PredictionAppender 
 Global description of this bean
   globalInfo()   - 
Method in class weka.gui.beans. Saver 
 Global info (if it exists) for the wrapped loader
   globalInfo()   - 
Method in class weka.gui.beans. ScatterPlotMatrix 
 Global info for this bean
   globalInfo()   - 
Method in class weka.gui.beans. SerializedModelSaver 
 Global info for this bean.
   globalInfo()   - 
Method in class weka.gui.beans. StripChart 
 Global info for this bean
   globalInfo()   - 
Method in class weka.gui.beans. TestSetMaker 
 Global info for this bean
   globalInfo()   - 
Method in class weka.gui.beans. TextViewer 
 Global info for this bean
   globalInfo()   - 
Method in class weka.gui.beans. TrainingSetMaker 
 Global info for this bean
   globalInfo()   - 
Method in class weka.gui.beans. TrainTestSplitMaker 
 Global info for this bean
   globalInfo()   - 
Method in class weka.gui.boundaryvisualizer. BoundaryVisualizer 
 Returns a string describing this tool
   GLOBALINFO_ENDTAG   - 
Static variable in class weka.core. GlobalInfoJavadoc 
 the end comment tag for inserting the generated Javadoc
   GLOBALINFO_METHOD   - 
Static variable in class weka.core. GlobalInfoJavadoc 
 the globalInfo method name
   GLOBALINFO_STARTTAG   - 
Static variable in class weka.core. GlobalInfoJavadoc 
 the start comment tag for inserting the generated Javadoc
   GlobalInfoJavadoc   - Class in  weka.core  Generates Javadoc comments from the class's globalInfo method.   GlobalInfoJavadoc()   - 
Constructor for class weka.core. GlobalInfoJavadoc 
 default constructor
   GlobalScoreSearchAlgorithm   - Class in  weka.classifiers.bayes.net.search.global  This Bayes Network learning algorithm uses cross validation to estimate classification accuracy.   GlobalScoreSearchAlgorithm()   - 
Constructor for class weka.classifiers.bayes.net.search.global. GlobalScoreSearchAlgorithm 
  
   goDown(String)   - 
Method in class weka.gui. HierarchyPropertyParser 
 Go to a certain node of the tree down from the current node 
 according to the specified relative path.
   goTo(String)   - 
Method in class weka.gui. HierarchyPropertyParser 
 Go to a certain node of the tree according to the specified path
 Note that the path must be absolute path from the root.
   goToChild(String)   - 
Method in class weka.gui. HierarchyPropertyParser 
 Go to one child node from the current position in the tree
 according to the given value  
 If the child node with the given value cannot be found it
 returns false, true otherwise.
   goToChild(int)   - 
Method in class weka.gui. HierarchyPropertyParser 
 Go to one child node from the current position in the tree
 according to the given position  
   goToParent()   - 
Method in class weka.gui. HierarchyPropertyParser 
 Go to the parent from the current position in the tree
 If the current position is the root, it stays there and does 
 not move
   goToRoot()   - 
Method in class weka.gui. HierarchyPropertyParser 
 Go to the root of the tree
   gr(double, double)   - 
Static method in class weka.core. Utils 
 Tests if a is greater than b.
   Grading   - Class in  weka.classifiers.meta  Implements Grading.   Grading()   - 
Constructor for class weka.classifiers.meta. Grading 
  
   GraftSplit   - Class in  weka.classifiers.trees.j48  Class implementing a split for nodes added to a tree during grafting.   GraftSplit(int, double, int, double, double)   - 
Constructor for class weka.classifiers.trees.j48. GraftSplit 
 constructor
   GraftSplit(int, double, int, double, double[][])   - 
Constructor for class weka.classifiers.trees.j48. GraftSplit 
 constructor
   graph(FPGrowth.FPTreeRoot)   - 
Method in class weka.associations. FPGrowth 
 Assemble a dot graph representation of the FP-tree.
   graph()   - 
Method in class weka.classifiers.bayes. BayesNet 
 Returns a BayesNet graph in XMLBIF ver 0.3 format.
   graph()   - 
Method in class weka.classifiers.meta. AttributeSelectedClassifier 
 Returns graph describing the classifier (if possible).
   graph()   - 
Method in class weka.classifiers.meta. CostSensitiveClassifier 
 Returns graph describing the classifier (if possible).
   graph()   - 
Method in class weka.classifiers.meta. CVParameterSelection 
 Returns graph describing the classifier (if possible).
   graph()   - 
Method in class weka.classifiers.meta. FilteredClassifier 
 Returns graph describing the classifier (if possible).
   graph()   - 
Method in class weka.classifiers.meta. ThresholdSelector 
 Returns graph describing the classifier (if possible).
   graph()   - 
Method in class weka.classifiers.trees. ADTree 
 Returns graph describing the tree.
   graph()   - 
Method in class weka.classifiers.trees.ft. FTtree 
 Returns graph describing the tree.
   graph()   - 
Method in class weka.classifiers.trees. FT 
 Returns graph describing the tree.
   graph()   - 
Method in class weka.classifiers.trees.j48. ClassifierTree 
 Returns graph describing the tree.
   graph()   - 
Method in class weka.classifiers.trees. J48 
 Returns graph describing the tree.
   graph()   - 
Method in class weka.classifiers.trees.j48. NBTreeClassifierTree 
 Returns graph describing the tree.
   graph()   - 
Method in class weka.classifiers.trees. J48graft 
 Returns graph describing the tree.
   graph()   - 
Method in class weka.classifiers.trees. LADTree 
 Returns graph describing the tree.
   graph()   - 
Method in class weka.classifiers.trees. LMT 
 Returns graph describing the tree.
   graph()   - 
Method in class weka.classifiers.trees.lmt. LMTNode 
 Returns graph describing the tree.
   graph(StringBuffer)   - 
Method in class weka.classifiers.trees.m5. RuleNode 
 Assign a unique identifier to each node in the tree and then
 calls graphTree
   graph()   - 
Method in class weka.classifiers.trees. M5P 
 Return a dot style String describing the tree.
   graph()   - 
Method in class weka.classifiers.trees. NBTree 
 Returns graph describing the tree.
   graph()   - 
Method in class weka.classifiers.trees. RandomTree 
 Returns graph describing the tree.
   graph()   - 
Method in class weka.classifiers.trees. REPTree 
 Outputs the decision tree as a graph
   graph()   - 
Method in class weka.classifiers.trees. UserClassifier 
  
   graph()   - 
Method in class weka.clusterers. Cobweb 
 Generates the graph string of the Cobweb tree
   graph()   - 
Method in class weka.clusterers. HierarchicalClusterer 
  
   graph()   - 
Method in interface weka.core. Drawable 
 Returns a string that describes a graph representing
 the object.
   GraphConstants   - Interface in  weka.gui.graphvisualizer  GraphConstants.java   GraphEdge   - Class in  weka.gui.graphvisualizer  This class represents an edge in the graph   GraphEdge(int, int, int)   - 
Constructor for class weka.gui.graphvisualizer. GraphEdge 
  
   GraphEdge(int, int, int, String, String)   - 
Constructor for class weka.gui.graphvisualizer. GraphEdge 
  
   GraphEvent   - Class in  weka.gui.beans  Event for graphs   GraphEvent(Object, String, String, int)   - 
Constructor for class weka.gui.beans. GraphEvent 
 Creates a new  GraphEvent  instance.
   GraphListener   - Interface in  weka.gui.beans  Describe interface  TextListener  here.   GraphNode   - Class in  weka.gui.graphvisualizer  This class represents a node in the Graph.   GraphNode(String, String)   - 
Constructor for class weka.gui.graphvisualizer. GraphNode 
 Constructor
   GraphNode(String, String, int)   - 
Constructor for class weka.gui.graphvisualizer. GraphNode 
 Constructor
   GraphPanel   - Class in  weka.clusterers.forOPTICSAndDBScan.OPTICS_GUI  
 GraphPanel.java  
 Authors: Rainer Holzmann, Zhanna Melnikova-Albrecht  
 Date: Sep 16, 2004  
 Time: 10:28:19 AM  
 $ Revision 1.4 $     GraphPanel(FastVector, int, boolean, boolean)   - 
Constructor for class weka.clusterers.forOPTICSAndDBScan.OPTICS_GUI. GraphPanel 
  
   graphType()   - 
Method in class weka.classifiers.bayes. BayesNet 
 Returns the type of graph this classifier
  represents.
   graphType()   - 
Method in class weka.classifiers.meta. AttributeSelectedClassifier 
 Returns the type of graph this classifier
  represents.
   graphType()   - 
Method in class weka.classifiers.meta. CostSensitiveClassifier 
 Returns the type of graph this classifier
  represents.
   graphType()   - 
Method in class weka.classifiers.meta. CVParameterSelection 
 Returns the type of graph this classifier
  represents.
   graphType()   - 
Method in class weka.classifiers.meta. FilteredClassifier 
 Returns the type of graph this classifier
 represents.
   graphType()   - 
Method in class weka.classifiers.meta. ThresholdSelector 
 Returns the type of graph this classifier
 represents.
   graphType()   - 
Method in class weka.classifiers.trees. ADTree 
 Returns the type of graph this classifier
  represents.
   graphType()   - 
Method in class weka.classifiers.trees. FT 
 Returns the type of graph this classifier
  represents.
   graphType()   - 
Method in class weka.classifiers.trees.j48. ClassifierTree 
 Returns the type of graph this classifier
  represents.
   graphType()   - 
Method in class weka.classifiers.trees. J48 
 Returns the type of graph this classifier
  represents.
   graphType()   - 
Method in class weka.classifiers.trees. J48graft 
 Returns the type of graph this classifier
  represents.
   graphType()   - 
Method in class weka.classifiers.trees. LADTree 
 Returns the type of graph this classifier
  represents.
   graphType()   - 
Method in class weka.classifiers.trees. LMT 
 Returns the type of graph this classifier
  represents.
   graphType()   - 
Method in class weka.classifiers.trees. M5P 
 Returns the type of graph this classifier
  represents.
   graphType()   - 
Method in class weka.classifiers.trees. NBTree 
 Returns the type of graph this classifier
  represents.
   graphType()   - 
Method in class weka.classifiers.trees. RandomTree 
 Returns the type of graph this classifier represents.
   graphType()   - 
Method in class weka.classifiers.trees. REPTree 
 Returns the type of graph this classifier
  represents.
   graphType()   - 
Method in class weka.classifiers.trees. UserClassifier 
 Returns the type of graph this classifier
  represents.
   graphType()   - 
Method in class weka.clusterers. Cobweb 
 Returns the type of graphs this class
  represents
   graphType()   - 
Method in class weka.clusterers. HierarchicalClusterer 
  
   graphType()   - 
Method in interface weka.core. Drawable 
 Returns the type of graph representing
 the object.
   GraphViewer   - Class in  weka.gui.beans  A bean encapsulating weka.gui.treevisualize.TreeVisualizer   GraphViewer()   - 
Constructor for class weka.gui.beans. GraphViewer 
  
   GraphViewerBeanInfo   - Class in  weka.gui.beans  Bean info class for the graph viewer   GraphViewerBeanInfo()   - 
Constructor for class weka.gui.beans. GraphViewerBeanInfo 
  
   GraphVisualizer   - Class in  weka.gui.graphvisualizer  This class displays the graph we want to visualize.   GraphVisualizer()   - 
Constructor for class weka.gui.graphvisualizer. GraphVisualizer 
 Constructor 
 Sets up the gui and initializes all the other previously
 uninitialized variables.
   GreedyStepwise   - Class in  weka.attributeSelection  GreedyStepwise : 
  
 Performs a greedy forward or backward search through the space of attribute subsets.   GreedyStepwise()   - 
Constructor for class weka.attributeSelection. GreedyStepwise 
 Constructor
   GRID   - 
Static variable in class weka.datagenerators.clusterers. BIRCHCluster 
 Constant set for choice of pattern.
   gridIsExtendableTipText()   - 
Method in class weka.classifiers.meta. GridSearch 
 Returns the tip text for this property
   GridSearch   - Class in  weka.classifiers.meta  Performs a grid search of parameter pairs for the a classifier (Y-axis, default is LinearRegression with the "Ridge" parameter) and the PLSFilter (X-axis, "# of Components") and chooses the best pair found for the actual predicting. 
  
 The initial grid is worked on with 2-fold CV to determine the values of the parameter pairs for the selected type of evaluation (e.g., accuracy).   GridSearch()   - 
Constructor for class weka.classifiers.meta. GridSearch 
 the default constructor
   grOrEq(double, double)   - 
Static method in class weka.core. Utils 
 Tests if a is greater or equal to b.
   grouping(boolean)   - 
Method in class weka.core.matrix. FlexibleDecimalFormat 
  
   grow(Instances)   - 
Method in class weka.classifiers.rules. Rule 
 Build this rule
   GT   - 
Static variable in interface weka.core.mathematicalexpression. sym 
  
   GT   - 
Static variable in interface weka.filters.unsupervised.instance.subsetbyexpression. sym 
  
   GUI   - Class in  weka.classifiers.bayes.net  GUI interface to Bayesian Networks.   GUI()   - 
Constructor for class weka.classifiers.bayes.net. GUI 
 Constructor 
 Sets up the gui and initializes all the other previously uninitialized
 variables.
   GUI_MDI   - 
Static variable in class weka.gui. Main 
 displays the GUI as MDI.
   GUI_SDI   - 
Static variable in class weka.gui. Main 
 displays the GUI as SDI.
   GUIChooser   - Class in  weka.gui  The main class for the Weka GUIChooser.   GUIChooser()   - 
Constructor for class weka.gui. GUIChooser 
 Creates the experiment environment gui with no initial experiment
   GUIChooser.ChildFrameSDI   - Class in  weka.gui  Specialized JFrame class.   GUIChooser.ChildFrameSDI(GUIChooser, String)   - 
Constructor for class weka.gui. GUIChooser.ChildFrameSDI 
 constructs a new internal frame that knows about its parent.
   GUIEDITORS_PROPERTY_FILE   - 
Static variable in class weka.gui. GenericObjectEditor 
 the properties files containing the class/editor mappings.
   GUITipText()   - 
Method in class weka.classifiers.functions. MultilayerPerceptron 
  
 
 
    
 H  
 
   h(double)   - 
Method in class weka.classifiers.functions.pace. ChisqMixture 
 Computes the value of h(x) given the mixture.
   h(DoubleVector)   - 
Method in class weka.classifiers.functions.pace. ChisqMixture 
 Computes the value of h(x) given the mixture, where x is a vector.
   h(double)   - 
Method in class weka.classifiers.functions.pace. NormalMixture 
 Computes the value of h(x) given the mixture.
   h(DoubleVector)   - 
Method in class weka.classifiers.functions.pace. NormalMixture 
 Computes the value of h(x) given the mixture, where x is a vector.
   h1(int, int)   - 
Method in class weka.classifiers.functions.pace. PaceMatrix 
 Constructs single Householder transformation for a column
   h2(int, int, double, PaceMatrix, int)   - 
Method in class weka.classifiers.functions.pace. PaceMatrix 
 Performs single Householder transformation on one column of a matrix
   handles(Capabilities.Capability)   - 
Method in class weka.core. Capabilities 
 returns true if the classifier handler has the specified capability
   handles(Capabilities.Capability)   - 
Method in class weka.core. FindWithCapabilities 
 returns true if the given capability can be handled.
   hasAdditional()   - 
Method in class weka.core. TechnicalInformation 
 returns true if there are further technical informations stored in this
   hasAntds()   - 
Method in class weka.classifiers.rules. ConjunctiveRule 
 Whether this rule has antecedents, i.e.
   hasAntds()   - 
Method in class weka.classifiers.rules. Rule 
 Whether this rule has antecedents, i.e.
   hasClasspathProblems()   - 
Method in class weka.core. CheckScheme 
 returns TRUE if the classifier returned a "not in classpath" Exception
   hasClasspathProblems()   - 
Method in class weka.estimators. CheckEstimator 
 returns TRUE if the estimator returned a "not in classpath" Exception
   hasDependencies()   - 
Method in class weka.core. Capabilities 
 Checks whether there are any dependencies at all
   hasDependency(Capabilities.Capability)   - 
Method in class weka.core. Capabilities 
 returns true if the classifier handler has a dependency for the specified 
 capability
   hasFalseHead()   - 
Method in class weka.associations.tertius. Rule 
 Test if the head of the rule is false.
   hash   - 
Variable in class weka.classifiers.lazy.kstar. KStarCache.TableEntry 
 attribute value hash code
   hashCode()   - 
Method in class weka.associations. FPGrowth.BinaryItem 
  
   hashCode()   - 
Method in class weka.associations. ItemSet 
 Produces a hash code for a item set.
   hashCode()   - 
Method in class weka.attributeSelection. ConsistencySubsetEval.hashKey 
 Calculates a hash code
   hashCode()   - 
Method in class weka.classifiers.rules. DecisionTableHashKey 
 Calculates a hash code
   hashCode()   - 
Method in class weka.core. SerializedObject 
 Returns a hashcode for this object.
   hashCode()   - 
Method in class weka.core. Trie 
 Returns the hash code value for this collection.
   hasIncomingBatchInstances()   - 
Method in class weka.gui.beans. Classifier 
 Returns true if this classifier has an incoming connection that is
 a batch set of instances
   hasIncomingBatchInstances()   - 
Method in class weka.gui.beans. Clusterer 
 Returns true if this clusterer has an incoming connection that is
 a batch set of instances
   hasIncomingStreamInstances()   - 
Method in class weka.gui.beans. Classifier 
 Returns true if this classifier has an incoming connection that is
 an instance stream
   hasIndex()   - 
Method in class weka.core. PropertyPath.PathElement 
 returns whether the property is an index-based one
   hasInterface(String, String)   - 
Static method in class weka.core. ClassDiscovery 
 Checks whether the given class implements the given interface.
   hasInterface(Class, Class)   - 
Static method in class weka.core. ClassDiscovery 
 Checks whether the given class implements the given interface.
   hasMaxCounterInstances()   - 
Method in class weka.associations.tertius. LiteralSet 
 Test if all the intances are counter-instances.
   hasMaxRows()   - 
Method in class weka.gui.sql. ResultSetHelper 
 whether a limit on the rows to retrieve was set.
   hasMissingValue()   - 
Method in class weka.core. Instance 
 Tests whether an instance has a missing value.
   hasModels()   - 
Method in class weka.classifiers.trees.ft. FTtree 
 Returns true if the logistic regression model at this node has changed compared to the
one at the parent node.
   hasModels()   - 
Method in class weka.classifiers.trees.lmt. LMTNode 
 Returns true if the logistic regression model at this node has changed compared to the
one at the parent node.
   hasMoreElements(Instances)   - 
Method in class weka.core.converters. ConverterUtils.DataSource 
 returns whether there are more Instance objects in the data.
   hasMoreElements()   - 
Method in class weka.core. FastVector.FastVectorEnumeration 
 Tests if there are any more elements to enumerate.
   hasMoreElements()   - 
Method in class weka.core.tokenizers. AlphabeticTokenizer 
 returns whether there are more elements still
   hasMoreElements()   - 
Method in class weka.core.tokenizers. NGramTokenizer 
 returns true if there's more elements available
   hasMoreElements()   - 
Method in class weka.core.tokenizers. Tokenizer 
 Tests if this enumeration contains more elements.
   hasMoreElements()   - 
Method in class weka.core.tokenizers. WordTokenizer 
 Tests if this enumeration contains more elements.
   hasMoreIterations()   - 
Method in class weka.experiment. Experiment 
 Returns true if there are more iterations to carry out in the experiment.
   hasNext()   - 
Method in class weka.associations.tertius. SimpleLinkedList.LinkedListIterator 
  
   hasNext()   - 
Method in class weka.clusterers.forOPTICSAndDBScan.Utils. PriorityQueue 
 Tests, if the queue has some more elements left
   hasNext()   - 
Method in class weka.clusterers.forOPTICSAndDBScan.Utils. UpdateQueue 
 Tests, if the queue has some more elements left
   hasNext()   - 
Method in class weka.core. Trie.TrieIterator 
 Returns true if the iteration has more elements.
   hasPrevious()   - 
Method in class weka.associations.tertius. SimpleLinkedList.LinkedListInverseIterator 
  
   hasResult()   - 
Method in class weka.gui.sql.event. QueryExecuteEvent 
 whether a ResultSet was produced, e.g.
   hasTargetMetaData()   - 
Method in class weka.core.pmml. MiningSchema 
 Returns true if there is Target meta data.
   hasTrueBody()   - 
Method in class weka.associations.tertius. Rule 
 Test if the body of the rule is true.
   hasUID(String)   - 
Static method in class weka.core. SerializationHelper 
 checks whether the given class contains a serialVersionUID.
   hasUID(Class)   - 
Static method in class weka.core. SerializationHelper 
 checks whether the given class contains a serialVersionUID.
   hasZeropoint()   - 
Method in class weka.core. Attribute 
 Returns whether the attribute has a zeropoint and may be
 added meaningfully.
   HDRankTipText()   - 
Method in class weka.classifiers.mi. CitationKNN 
 Returns the tip text for this property
   Head   - Class in  weka.associations.tertius  Class representing the head of a rule.   Head()   - 
Constructor for class weka.associations.tertius. Head 
 Constructor without storing the counter-instances.
   Head(Instances)   - 
Constructor for class weka.associations.tertius. Head 
 Constructor storing the counter-instances.
   headContains(Literal)   - 
Method in class weka.associations.tertius. Rule 
 Test if the head of the rule contains a literal.
   header(int)   - 
Method in class weka.experiment. PairedTTester 
 Creates a "header" string describing the current resultsets.
   header(int)   - 
Method in interface weka.experiment. Tester 
 Creates a "header" string describing the current resultsets.
   headerKeys()   - 
Method in class weka.experiment. ResultMatrix 
 returns an enumeration of the header keys
   HEIGHT   - 
Static variable in class weka.gui.arffviewer. ArffViewerMainPanel 
 default height
   HEIGHT   - 
Static variable in class weka.gui.sql. SqlViewer 
 the height property in the history file.
   heuristicStopTipText()   - 
Method in class weka.classifiers.functions. SimpleLogistic 
 Returns the tip text for this property
   heuristicTipText()   - 
Method in class weka.classifiers.trees. BFTree 
 Returns the tip text for this property
   heuristicTipText()   - 
Method in class weka.classifiers.trees. SimpleCart 
 Returns the tip text for this property
   hf(double)   - 
Method in class weka.classifiers.functions.pace. ChisqMixture 
 Computes the value of h(x) / f(x) given the mixture.
   hf(double)   - 
Method in class weka.classifiers.functions.pace. NormalMixture 
 Computes the value of h(x) / f(x) given the mixture.
   hiddenLayersTipText()   - 
Method in class weka.classifiers.functions. MultilayerPerceptron 
  
   HierarchicalBCEngine   - Class in  weka.gui.graphvisualizer  This class lays out the vertices of a graph in a
 hierarchy of vertical levels, with a number of nodes
 in each level.   HierarchicalBCEngine(FastVector, FastVector, int, int)   - 
Constructor for class weka.gui.graphvisualizer. HierarchicalBCEngine 
 Constructor - takes in FastVectors of nodes and edges, and the initial
  width and height of a node
   HierarchicalBCEngine(FastVector, FastVector, int, int, boolean)   - 
Constructor for class weka.gui.graphvisualizer. HierarchicalBCEngine 
 Constructor - takes in FastVectors of nodes and edges, the initial width
 and height of a node, and a boolean value to indicate if the edges
 should be concentrated.
   HierarchicalBCEngine()   - 
Constructor for class weka.gui.graphvisualizer. HierarchicalBCEngine 
 SimpleConstructor
 If we want to instantiate the class first, and if information for
 nodes and edges is not available.
   HierarchicalClusterer   - Class in  weka.clusterers  Hierarchical clustering class.   HierarchicalClusterer()   - 
Constructor for class weka.clusterers. HierarchicalClusterer 
  
   HierarchyPropertyParser   - Class in  weka.gui  This class implements a parser to read properties that have
 a hierarchy(i.e.   HierarchyPropertyParser()   - 
Constructor for class weka.gui. HierarchyPropertyParser 
 Default constructor
   HierarchyPropertyParser(String, String)   - 
Constructor for class weka.gui. HierarchyPropertyParser 
 Constructor that builds a tree from the given property with
 the given delimitor
   HierarchyVisualizer   - Class in  weka.gui.hierarchyvisualizer      HierarchyVisualizer(String)   - 
Constructor for class weka.gui.hierarchyvisualizer. HierarchyVisualizer 
  
   HillClimber   - Class in  weka.classifiers.bayes.net.search.global  This Bayes Network learning algorithm uses a hill climbing algorithm adding, deleting and reversing arcs.   HillClimber()   - 
Constructor for class weka.classifiers.bayes.net.search.global. HillClimber 
  
   HillClimber   - Class in  weka.classifiers.bayes.net.search.local  This Bayes Network learning algorithm uses a hill climbing algorithm adding, deleting and reversing arcs.   HillClimber()   - 
Constructor for class weka.classifiers.bayes.net.search.local. HillClimber 
  
   HISTORY_NAME   - 
Static variable in class weka.gui.sql. ConnectionPanel 
 the name of the history.
   HISTORY_NAME   - 
Static variable in class weka.gui.sql. QueryPanel 
 the name of the history.
   historyChanged(HistoryChangedEvent)   - 
Method in interface weka.gui.sql.event. HistoryChangedListener 
 This method gets called when a history is modified.
   historyChanged(HistoryChangedEvent)   - 
Method in class weka.gui.sql. SqlViewer 
 This method gets called when a history is modified.
   HistoryChangedEvent   - Class in  weka.gui.sql.event  An event that is generated when a history is modified.   HistoryChangedEvent(Object, String, DefaultListModel)   - 
Constructor for class weka.gui.sql.event. HistoryChangedEvent 
 constructs the event
   HistoryChangedListener   - Interface in  weka.gui.sql.event  A listener for changes in a history.   hit(Rectangle, Shape, boolean)   - 
Method in class weka.gui.visualize. PostscriptGraphics 
  
   HLINE   - 
Static variable in class weka.gui.visualize. VisualizePanelEvent 
  
   HNB   - Class in  weka.classifiers.bayes  Contructs Hidden Naive Bayes classification model with high classification accuracy and AUC. 
  
 For more information refer to: 
  
 H.   HNB()   - 
Constructor for class weka.classifiers.bayes. HNB 
  
   holdOutFileTipText()   - 
Method in class weka.attributeSelection. ClassifierSubsetEval 
 Returns the tip text for this property
   HoldOutSubsetEvaluator   - Class in  weka.attributeSelection  Abstract attribute subset evaluator capable of evaluating subsets with
 respect to a data set that is distinct from that used to initialize/
 train the subset evaluator.   HoldOutSubsetEvaluator()   - 
Constructor for class weka.attributeSelection. HoldOutSubsetEvaluator 
  
   hornClausesTipText()   - 
Method in class weka.associations. Tertius 
 Returns the tip text for this property.
   HostListPanel   - Class in  weka.gui.experiment  This panel controls setting a list of hosts for a RemoteExperiment to
 use.   HostListPanel(RemoteExperiment)   - 
Constructor for class weka.gui.experiment. HostListPanel 
 Creates the host list panel with the given experiment.
   HostListPanel()   - 
Constructor for class weka.gui.experiment. HostListPanel 
 Create the host list panel initially disabled.
   HyperparameterRange   - 
Variable in class weka.classifiers.bayes. BayesianLogisticRegression 
 CV Hyperparameter Range
   hyperparameterRangeTipText()   - 
Method in class weka.classifiers.bayes. BayesianLogisticRegression 
 Returns the tip text for this property
   Hyperparameters   - 
Variable in class weka.classifiers.bayes. BayesianLogisticRegression 
 Array to store Hyperparameter values for each feature.
   HyperparameterSelection   - 
Variable in class weka.classifiers.bayes. BayesianLogisticRegression 
 Hyperparameter selection method
   hyperparameterSelectionTipText()   - 
Method in class weka.classifiers.bayes. BayesianLogisticRegression 
 Returns the tip text for this property
   HyperparameterValue   - 
Variable in class weka.classifiers.bayes. BayesianLogisticRegression 
 Best hyperparameter for test phase
   hyperparameterValueTipText()   - 
Method in class weka.classifiers.bayes. BayesianLogisticRegression 
 Returns the tip text for this property
   HyperPipes   - Class in  weka.classifiers.misc  Class implementing a HyperPipe classifier.   HyperPipes()   - 
Constructor for class weka.classifiers.misc. HyperPipes 
  
   hypot(double, double)   - 
Static method in class weka.core.matrix. Maths 
 sqrt(a^2 + b^2) without under/overflow.
 
 
    
 I  
 
   I0   - 
Static variable in class weka.classifiers.functions.supportVector. RegSMOImproved 
  
   I0a   - 
Static variable in class weka.classifiers.functions.supportVector. RegSMOImproved 
  
   I0b   - 
Static variable in class weka.classifiers.functions.supportVector. RegSMOImproved 
  
   I1   - 
Static variable in class weka.classifiers.functions.supportVector. RegSMOImproved 
  
   I2   - 
Static variable in class weka.classifiers.functions.supportVector. RegSMOImproved 
  
   I3   - 
Static variable in class weka.classifiers.functions.supportVector. RegSMOImproved 
  
   IB1   - Class in  weka.classifiers.lazy  Nearest-neighbour classifier.   IB1()   - 
Constructor for class weka.classifiers.lazy. IB1 
  
   IBk   - Class in  weka.classifiers.lazy  K-nearest neighbours classifier.   IBk(int)   - 
Constructor for class weka.classifiers.lazy. IBk 
 IBk classifier.
   IBk()   - 
Constructor for class weka.classifiers.lazy. IBk 
 IB1 classifer.
   ICON_PATH   - 
Static variable in class weka.gui.beans. BeanVisual 
  
   ICSSearchAlgorithm   - Class in  weka.classifiers.bayes.net.search.ci  This Bayes Network learning algorithm uses conditional independence tests to find a skeleton, finds V-nodes and applies a set of rules to find the directions of the remaining arrows.   ICSSearchAlgorithm()   - 
Constructor for class weka.classifiers.bayes.net.search.ci. ICSSearchAlgorithm 
  
   Id3   - Class in  weka.classifiers.trees  Class for constructing an unpruned decision tree based on the ID3 algorithm.   Id3()   - 
Constructor for class weka.classifiers.trees. Id3 
  
   identity(int, int)   - 
Static method in class weka.core.matrix. Matrix 
 Generate identity matrix
   IDFTransformTipText()   - 
Method in class weka.filters.unsupervised.attribute. StringToWordVector 
 Returns the tip text for this property.
   IDIndexTipText()   - 
Method in class weka.filters.unsupervised.attribute. AddID 
 Returns the tip text for this property
   IDLE   - 
Static variable in class weka.gui.beans. BeanInstance 
  
   IFELSE   - 
Static variable in interface weka.core.mathematicalexpression. sym 
  
   ignoreClassTipText()   - 
Method in class weka.filters.unsupervised.attribute. PotentialClassIgnorer 
 Returns the tip text for this property
   ignored()   - 
Method in class weka.core.xml. PropertyHandler 
 returns an enumeration of the stored display names and classes of 
 properties to ignore.  
  NOTE:  String and Class Objects are mixed in this enumeration, depending
 whether it is a global property to ignore or just one for a certain class!
   ignoredAttributeIndicesTipText()   - 
Method in class weka.filters.unsupervised.attribute. AddCluster 
 Returns the tip text for this property
   ignoredAttributeIndicesTipText()   - 
Method in class weka.filters.unsupervised.attribute. ClusterMembership 
 Returns the tip text for this property
   ignoreRangeTipText()   - 
Method in class weka.filters.unsupervised.attribute. MathExpression 
 Returns the tip text for this property
   IMAGES   - 
Static variable in class weka.gui. ComponentHelper 
 the default directories for images
   IMPLICIT   - 
Static variable in class weka.associations. Tertius 
 Way of handling missing values: max counterinstances
   ImproveSolutions()   - 
Method in class weka.attributeSelection. ScatterSearchV1 
 Improve the solutions previously combined by adding the attributes that improve that solution
   Impurity   - Class in  weka.classifiers.trees.m5  Class for handling the impurity values when spliting the instances   Impurity(int, int, Instances, int)   - 
Constructor for class weka.classifiers.trees.m5. Impurity 
 Constructs an Impurity object containing the impurity values of partitioning the instances using an attribute
   includeClassTipText()   - 
Method in class weka.filters.unsupervised.attribute. NumericCleaner 
 Returns the tip text for this property
   incompleteBeta(double, double, double)   - 
Static method in class weka.core. Statistics 
 Returns the Incomplete Beta Function evaluated from zero to  xx .
   incompleteBetaFraction1(double, double, double)   - 
Static method in class weka.core. Statistics 
 Continued fraction expansion #1 for incomplete beta integral.
   incompleteBetaFraction2(double, double, double)   - 
Static method in class weka.core. Statistics 
 Continued fraction expansion #2 for incomplete beta integral.
   incompleteGamma(double, double)   - 
Static method in class weka.core. Statistics 
 Returns the Incomplete Gamma function.
   incompleteGammaComplement(double, double)   - 
Static method in class weka.core. Statistics 
 Returns the Complemented Incomplete Gamma function.
   incorrect()   - 
Method in class weka.classifiers.evaluation. ConfusionMatrix 
 Gets the number of incorrect classifications (that is, for which an
 incorrect prediction was made).
   incorrect()   - 
Method in class weka.classifiers. Evaluation 
 Gets the number of instances incorrectly classified (that is, for
 which an incorrect prediction was made).
   incrCoordCount()   - 
Method in class weka.core.neighboursearch. PerformanceStats 
 Increments the coordinate count
 (number of coordinates/attributes 
 looked at).
   increaseFrequency(int)   - 
Method in class weka.associations. FPGrowth.BinaryItem 
 Increase the frequency of this item.
   increaseFrequency()   - 
Method in class weka.associations. FPGrowth.BinaryItem 
 Increment the frequency of this item.
   incremental(double, int)   - 
Method in class weka.classifiers.trees.m5. Impurity 
 Incrementally computes the impurirty values
   INCREMENTAL   - 
Static variable in interface weka.core.converters. Loader 
  
   INCREMENTAL   - 
Static variable in interface weka.core.converters. Saver 
  
   IncrementalClassifierEvaluator   - Class in  weka.gui.beans  Bean that evaluates incremental classifiers   IncrementalClassifierEvaluator()   - 
Constructor for class weka.gui.beans. IncrementalClassifierEvaluator 
  
   IncrementalClassifierEvaluatorBeanInfo   - Class in  weka.gui.beans  Bean info class for the incremental classifier evaluator bean   IncrementalClassifierEvaluatorBeanInfo()   - 
Constructor for class weka.gui.beans. IncrementalClassifierEvaluatorBeanInfo 
  
   IncrementalClassifierEvaluatorCustomizer   - Class in  weka.gui.beans  GUI Customizer for the incremental classifier evaluator bean   IncrementalClassifierEvaluatorCustomizer()   - 
Constructor for class weka.gui.beans. IncrementalClassifierEvaluatorCustomizer 
  
   IncrementalClassifierEvent   - Class in  weka.gui.beans  Class encapsulating an incrementally built classifier and current instance   IncrementalClassifierEvent(Object, Classifier, Instance, int)   - 
Constructor for class weka.gui.beans. IncrementalClassifierEvent 
 Creates a new  IncrementalClassifierEvent  instance.
   IncrementalClassifierEvent(Object, Classifier, Instances)   - 
Constructor for class weka.gui.beans. IncrementalClassifierEvent 
 Creates a new incremental classifier event that encapsulates
 header information and classifier.
   IncrementalClassifierEvent(Object)   - 
Constructor for class weka.gui.beans. IncrementalClassifierEvent 
  
   IncrementalClassifierListener   - Interface in  weka.gui.beans  Interface to something that can process a IncrementalClassifierEvent   IncrementalConverter   - Interface in  weka.core.converters  Marker interface for a loader/saver that can retrieve instances incrementally   IncrementalEstimator   - Interface in  weka.estimators  Interface for an incremental probability estimators.   incrIntNodeCount()   - 
Method in class weka.core.neighboursearch. TreePerformanceStats 
 Increments the internal node count.
   incrLeafCount()   - 
Method in class weka.core.neighboursearch. TreePerformanceStats 
 Increments the leaf count.
   incrPointCount()   - 
Method in class weka.core.neighboursearch. PerformanceStats 
 Increments the point count 
 (number of datapoints looked at).
   index()   - 
Method in class weka.core. Attribute 
 Returns the index of this attribute.
   index(int)   - 
Method in class weka.core. Instance 
 Returns the index of the attribute stored at the given position.
   index(int)   - 
Method in class weka.core. SparseInstance 
 Returns the index of the attribute stored at the given position.
   INDEX_BEANCONNECTIONS   - 
Static variable in class weka.gui.beans.xml. XMLBeans 
 the index in the Vector, where the BeanConnections are stored
 (Instances and Connections are stored in a Vector and then serialized)
   INDEX_BEANINSTANCES   - 
Static variable in class weka.gui.beans.xml. XMLBeans 
 the index in the Vector, where the BeanInstances are stored 
 (Instances and Connections are stored in a Vector and then serialized)
   indexOf(Literal)   - 
Method in class weka.associations.tertius. Predicate 
  
   indexOf(Object)   - 
Method in class weka.core. FastVector 
 Searches for the first occurence of the given argument, 
 testing for equality using the equals method.
   indexOf(Object)   - 
Method in class weka.gui. CheckBoxList.CheckBoxListModel 
 Searches for the first occurrence of elem.
   indexOf(Object, int)   - 
Method in class weka.gui. CheckBoxList.CheckBoxListModel 
 Searches for the first occurrence of elem, beginning the search at index.
   indexOfMax()   - 
Method in class weka.core.matrix. DoubleVector 
 Returns the index of the maximum.
   indexOfValue(String)   - 
Method in class weka.core. Attribute 
 Returns the index of a given attribute value.
   indexToString(int)   - 
Static method in class weka.core. SingleIndex 
 Creates a string representation of the given index.
   indicesToRangeList(int[])   - 
Static method in class weka.core. Range 
 Creates a string representation of the indices in the supplied array.
   INDIVIDUAL_PROPERTY   - 
Static variable in class weka.associations.tertius. IndividualLiteral 
  
   IndividualInstance   - Class in  weka.associations.tertius      IndividualInstance(Instance, Instances)   - 
Constructor for class weka.associations.tertius. IndividualInstance 
  
   IndividualInstance(IndividualInstance)   - 
Constructor for class weka.associations.tertius. IndividualInstance 
  
   IndividualInstances   - Class in  weka.associations.tertius      IndividualInstances(Instances, Instances)   - 
Constructor for class weka.associations.tertius. IndividualInstances 
  
   IndividualLiteral   - Class in  weka.associations.tertius      IndividualLiteral(Predicate, String, int, int, int, int)   - 
Constructor for class weka.associations.tertius. IndividualLiteral 
  
   individualPredictions(Instance)   - 
Method in class weka.classifiers.meta. MultiClassClassifier 
 Returns the individual predictions of the base classifiers
 for an instance.
   INFO   - 
Static variable in class weka.core. Debug 
 the log level Info
   info(int[])   - 
Static method in class weka.core. Utils 
 Computes entropy for an array of integers.
   infoGain()   - 
Method in class weka.classifiers.trees.j48. BinC45Split 
 Returns (C4.5-type) information gain for the generated split.
   infoGain()   - 
Method in class weka.classifiers.trees.j48. C45Split 
 Returns (C4.5-type) information gain for the generated split.
   InfoGainAttributeEval   - Class in  weka.attributeSelection  InfoGainAttributeEval : 
  
 Evaluates the worth of an attribute by measuring the information gain with respect to the class. 
  
 InfoGain(Class,Attribute) = H(Class) - H(Class | Attribute). 
  
  

  
 Valid options are:     InfoGainAttributeEval()   - 
Constructor for class weka.attributeSelection. InfoGainAttributeEval 
 Constructor
   InfoGainSplitCrit   - Class in  weka.classifiers.trees.j48  Class for computing the information gain for a given distribution.   InfoGainSplitCrit()   - 
Constructor for class weka.classifiers.trees.j48. InfoGainSplitCrit 
  
   InfoPanel   - Class in  weka.gui.sql  A simple panel for displaying information, e.g.   InfoPanel(JFrame)   - 
Constructor for class weka.gui.sql. InfoPanel 
 creates the panel
   InfoPanelCellRenderer   - Class in  weka.gui.sql  A specialized renderer that takes care of JLabels in a JList.   InfoPanelCellRenderer()   - 
Constructor for class weka.gui.sql. InfoPanelCellRenderer 
 the constructor
   Init(int, int)   - 
Method in class weka.classifiers.bayes.net. BayesNetGenerator 
 Init defines a minimal Bayes net with no arcs
   initAsNaiveBayesTipText()   - 
Method in class weka.classifiers.bayes.net.search. SearchAlgorithm 
  
   initClassifier(Instances)   - 
Method in class weka.classifiers.bayes. DMNBtext.DNBBinary 
  
   initClassifier(Instances)   - 
Method in interface weka.classifiers. IterativeClassifier 
 Inits an iterative classifier.
   initClassifier(Instances)   - 
Method in class weka.classifiers.trees. ADTree 
 Sets up the tree ready to be trained, using two-class optimized method.
   initClassifier(Instances)   - 
Method in class weka.classifiers.trees. LADTree 
 Sets up the tree ready to be trained.
   initCPTs()   - 
Method in class weka.classifiers.bayes. BayesNet 
 initializes the conditional probabilities
   initCPTs(BayesNet)   - 
Method in class weka.classifiers.bayes.net.estimate. BayesNetEstimator 
 initCPTs reserves space for CPTs and set all counts to zero
   initCPTs(BayesNet)   - 
Method in class weka.classifiers.bayes.net.estimate. BMAEstimator 
 initCPTs reserves space for CPTs and set all counts to zero
   initCPTs(BayesNet)   - 
Method in class weka.classifiers.bayes.net.estimate. MultiNomialBMAEstimator 
 initCPTs reserves space for CPTs and set all counts to zero
   initCPTs(BayesNet)   - 
Method in class weka.classifiers.bayes.net.estimate. SimpleEstimator 
 initCPTs reserves space for CPTs and set all counts to zero
   initDebugVectorsInput()   - 
Method in class weka.clusterers. XMeans 
 Initialises the debug vector input.
   initFileClassIndexTipText()   - 
Method in class weka.filters.unsupervised.attribute. KernelFilter 
 Returns the tip text for this property
   initFileTipText()   - 
Method in class weka.filters.unsupervised.attribute. KernelFilter 
 Returns the tip text for this property
   initFilter(Instances)   - 
Method in class weka.filters.unsupervised.attribute. KernelFilter 
 initializes the filter with the given dataset, i.e., the kernel gets
 built.
   INITIAL_STEP   - 
Static variable in interface weka.classifiers.lazy.kstar. KStarConstants 
  
   initialAnchorRandomTipText()   - 
Method in class weka.core.neighboursearch.balltrees. MiddleOutConstructor 
 Returns the tip text for this property.
   initialize()   - 
Method in class weka.classifiers.bayes. BayesianLogisticRegression 
 
 (1)Initialize m_Beta[j] to 0.
   initialize()   - 
Method in class weka.classifiers. CostMatrix 
 Initializes the matrix
   initialize()   - 
Method in class weka.classifiers.trees.j48. Distribution 
 Sets all counts to zero.
   initialize(int, int, int)   - 
Method in class weka.classifiers.trees.m5. CorrelationSplitInfo 
 Resets the object of split information
   initialize(int, int, int)   - 
Method in class weka.classifiers.trees.m5. YongSplitInfo 
 Resets the object of split information
   initialize()   - 
Method in class weka.experiment. Experiment 
 Prepares an experiment for running, initializing current iterator
 settings.
   initialize()   - 
Method in class weka.experiment. RemoteExperiment 
 Prepares a remote experiment for running, creates sub experiments
   initialize()   - 
Method in class weka.gui.visualize. BMPWriter 
 further initialization
   initialize()   - 
Method in class weka.gui.visualize. JPEGWriter 
 further initialization.
   initialize()   - 
Method in class weka.gui.visualize. PNGWriter 
 further initialization
   initializeDown(boolean)   - 
Method in class weka.classifiers.bayes.net. MarginCalculator.JunctionTreeNode 
  
   initializeRanges(int)   - 
Method in class weka.core. Debug.DBO 
 Initialize ranges, upper limit must be set
   initializeRanges()   - 
Method in class weka.core. NormalizableDistance 
 Initializes the ranges using all instances of the dataset.
   initializeRanges(int[])   - 
Method in class weka.core. NormalizableDistance 
 Initializes the ranges of a subset of the instances of this dataset.
   initializeRanges(int[], int, int)   - 
Method in class weka.core. NormalizableDistance 
 Initializes the ranges of a subset of the instances of this dataset.
   initializeRangesEmpty(int, double[][])   - 
Method in class weka.core. NormalizableDistance 
 Used to initialize the ranges.
   initializeUp()   - 
Method in class weka.classifiers.bayes.net. MarginCalculator.JunctionTreeNode 
  
   initInternalFields()   - 
Method in class weka.gui.visualize. MatrixPanel 
 Initializes internal data fields, i.e.
   InitPopulation(int)   - 
Method in class weka.attributeSelection. ScatterSearchV1 
 Creating space for introducing the population
   initStructure()   - 
Method in class weka.classifiers.bayes. BayesNet 
 Init structure initializes the structure to an empty graph or a Naive Bayes
 graph (depending on the -N flag).
   innerProduct(DoubleVector)   - 
Method in class weka.core.matrix. DoubleVector 
 Returns the inner product of two DoubleVectors
   INPUT   - 
Static variable in class weka.classifiers.functions.neural. NeuralConnection 
 This unit is an input unit.
   input(Instance)   - 
Method in class weka.filters. AllFilter 
 Input an instance for filtering.
   input(Instance)   - 
Method in class weka.filters. Filter 
 Input an instance for filtering.
   input(Instance)   - 
Method in class weka.filters. SimpleBatchFilter 
 Input an instance for filtering.
   input(Instance)   - 
Method in class weka.filters. SimpleStreamFilter 
 Input an instance for filtering.
   input(Instance)   - 
Method in class weka.filters.supervised.attribute. AttributeSelection 
 Input an instance for filtering.
   input(Instance)   - 
Method in class weka.filters.supervised.attribute. ClassOrder 
 Input an instance for filtering.
   input(Instance)   - 
Method in class weka.filters.supervised.attribute. Discretize 
 Input an instance for filtering.
   input(Instance)   - 
Method in class weka.filters.supervised.attribute. NominalToBinary 
 Input an instance for filtering.
   input(Instance)   - 
Method in class weka.filters.supervised.instance. Resample 
 Input an instance for filtering.
   input(Instance)   - 
Method in class weka.filters.supervised.instance. SMOTE 
 Input an instance for filtering.
   input(Instance)   - 
Method in class weka.filters.supervised.instance. SpreadSubsample 
 Input an instance for filtering.
   input(Instance)   - 
Method in class weka.filters.supervised.instance. StratifiedRemoveFolds 
 Input an instance for filtering.
   input(Instance)   - 
Method in class weka.filters.unsupervised.attribute. AbstractTimeSeries 
 Input an instance for filtering.
   input(Instance)   - 
Method in class weka.filters.unsupervised.attribute. Add 
 Input an instance for filtering.
   input(Instance)   - 
Method in class weka.filters.unsupervised.attribute. AddCluster 
 Input an instance for filtering.
   input(Instance)   - 
Method in class weka.filters.unsupervised.attribute. AddExpression 
 Input an instance for filtering.
   input(Instance)   - 
Method in class weka.filters.unsupervised.attribute. AddID 
 Input an instance for filtering.
   input(Instance)   - 
Method in class weka.filters.unsupervised.attribute. AddNoise 
 Input an instance for filtering.
   input(Instance)   - 
Method in class weka.filters.unsupervised.attribute. AddValues 
 Input an instance for filtering.
   input(Instance)   - 
Method in class weka.filters.unsupervised.attribute. Center 
 Input an instance for filtering.
   input(Instance)   - 
Method in class weka.filters.unsupervised.attribute. ChangeDateFormat 
 Input an instance for filtering.
   input(Instance)   - 
Method in class weka.filters.unsupervised.attribute. ClusterMembership 
 Input an instance for filtering.
   input(Instance)   - 
Method in class weka.filters.unsupervised.attribute. Copy 
 Input an instance for filtering.
   input(Instance)   - 
Method in class weka.filters.unsupervised.attribute. Discretize 
 Input an instance for filtering.
   input(Instance)   - 
Method in class weka.filters.unsupervised.attribute. FirstOrder 
 Input an instance for filtering.
   input(Instance)   - 
Method in class weka.filters.unsupervised.attribute. MakeIndicator 
 Input an instance for filtering.
   input(Instance)   - 
Method in class weka.filters.unsupervised.attribute. MathExpression 
 Input an instance for filtering.
   input(Instance)   - 
Method in class weka.filters.unsupervised.attribute. MergeTwoValues 
 Input an instance for filtering.
   input(Instance)   - 
Method in class weka.filters.unsupervised.attribute. MultiInstanceToPropositional 
 Input an instance for filtering.
   input(Instance)   - 
Method in class weka.filters.unsupervised.attribute. NominalToBinary 
 Input an instance for filtering.
   input(Instance)   - 
Method in class weka.filters.unsupervised.attribute. NominalToString 
 Input an instance for filtering.
   input(Instance)   - 
Method in class weka.filters.unsupervised.attribute. Normalize 
 Input an instance for filtering.
   input(Instance)   - 
Method in class weka.filters.unsupervised.attribute. NumericToBinary 
 Input an instance for filtering.
   input(Instance)   - 
Method in class weka.filters.unsupervised.attribute. NumericTransform 
 Input an instance for filtering.
   input(Instance)   - 
Method in class weka.filters.unsupervised.attribute. Obfuscate 
 Input an instance for filtering.
   input(Instance)   - 
Method in class weka.filters.unsupervised.attribute. PrincipalComponents 
 Input an instance for filtering.
   input(Instance)   - 
Method in class weka.filters.unsupervised.attribute. RandomProjection 
 Input an instance for filtering.
   input(Instance)   - 
Method in class weka.filters.unsupervised.attribute. Remove 
 Input an instance for filtering.
   input(Instance)   - 
Method in class weka.filters.unsupervised.attribute. RemoveType 
 Input an instance for filtering.
   input(Instance)   - 
Method in class weka.filters.unsupervised.attribute. RemoveUseless 
 Input an instance for filtering.
   input(Instance)   - 
Method in class weka.filters.unsupervised.attribute. Reorder 
 Input an instance for filtering.
   input(Instance)   - 
Method in class weka.filters.unsupervised.attribute. ReplaceMissingValues 
 Input an instance for filtering.
   input(Instance)   - 
Method in class weka.filters.unsupervised.attribute. Standardize 
 Input an instance for filtering.
   input(Instance)   - 
Method in class weka.filters.unsupervised.attribute. StringToNominal 
 Input an instance for filtering.
   input(Instance)   - 
Method in class weka.filters.unsupervised.attribute. StringToWordVector 
 Input an instance for filtering.
   input(Instance)   - 
Method in class weka.filters.unsupervised.attribute. SwapValues 
 Input an instance for filtering.
   input(Instance)   - 
Method in class weka.filters.unsupervised.instance. NonSparseToSparse 
 Input an instance for filtering.
   input(Instance)   - 
Method in class weka.filters.unsupervised.instance. Normalize 
 Input an instance for filtering.
   input(Instance)   - 
Method in class weka.filters.unsupervised.instance. Randomize 
 Input an instance for filtering.
   input(Instance)   - 
Method in class weka.filters.unsupervised.instance. RemoveFolds 
 Input an instance for filtering.
   input(Instance)   - 
Method in class weka.filters.unsupervised.instance. RemoveFrequentValues 
 Input an instance for filtering.
   input(Instance)   - 
Method in class weka.filters.unsupervised.instance. RemoveMisclassified 
 Input an instance for filtering.
   input(Instance)   - 
Method in class weka.filters.unsupervised.instance. RemovePercentage 
 Input an instance for filtering.
   input(Instance)   - 
Method in class weka.filters.unsupervised.instance. RemoveRange 
 Input an instance for filtering.
   input(Instance)   - 
Method in class weka.filters.unsupervised.instance. RemoveWithValues 
 Input an instance for filtering.
   input(Instance)   - 
Method in class weka.filters.unsupervised.instance. Resample 
 Input an instance for filtering.
   input(Instance)   - 
Method in class weka.filters.unsupervised.instance. ReservoirSample 
 Input an instance for filtering.
   input(Instance)   - 
Method in class weka.filters.unsupervised.instance. SparseToNonSparse 
 Input an instance for filtering.
   input(Instance)   - 
Method in class weka.filters.unsupervised.instance. SubsetByExpression 
 Input an instance for filtering.
   input(Instance)   - 
Method in class weka.gui.streams. InstanceCounter 
  
   input(Instance)   - 
Method in class weka.gui.streams. InstanceJoiner 
  
   input(Instance)   - 
Method in class weka.gui.streams. InstanceSavePanel 
  
   input(Instance)   - 
Method in class weka.gui.streams. InstanceTable 
  
   input(Instance)   - 
Method in class weka.gui.streams. InstanceViewer 
  
   inputCenterFileTipText()   - 
Method in class weka.clusterers. XMeans 
 Returns the tip text for this property.
   inputFormat(Instances)   - 
Method in class weka.gui.streams. InstanceCounter 
  
   inputFormat(Instances)   - 
Method in class weka.gui.streams. InstanceJoiner 
 Sets the format of the input instances.
   inputFormat(Instances)   - 
Method in class weka.gui.streams. InstanceSavePanel 
  
   inputFormat(Instances)   - 
Method in class weka.gui.streams. InstanceTable 
  
   inputFormat(Instances)   - 
Method in class weka.gui.streams. InstanceViewer 
  
   InputHyperparameterValues   - 
Static variable in class weka.classifiers.bayes. BayesianLogisticRegression 
 Set of values to be used as hyperparameter values during Cross-Validation.
   inputOrderTipText()   - 
Method in class weka.datagenerators.clusterers. BIRCHCluster 
 Returns the tip text for this property
   inputs(Vector)   - 
Static method in class weka.gui.beans. BeanConnection 
 Returns a vector of BeanInstances that can be considered
 as inputs (or the left-hand side of a sub-flow)
   inputsContains(BeanInstance)   - 
Method in class weka.gui.beans. MetaBean 
  
   inRanges(Instance, double[][])   - 
Method in class weka.core. NormalizableDistance 
 Test if an instance is within the given ranges.
   insert(int)   - 
Method in class weka.classifiers.functions.supportVector. SMOset 
 Inserts an element into the set.
   insert(double, double, double)   - 
Method in class weka.classifiers.lazy.kstar. KStarCache.CacheTable 
 Inserts a new entry in the hashtable using the specified key.
   insert(DataObject)   - 
Method in interface weka.clusterers.forOPTICSAndDBScan.Databases. Database 
 Inserts a new dataObject into the database
   insert(DataObject)   - 
Method in class weka.clusterers.forOPTICSAndDBScan.Databases. SequentialDatabase 
 Inserts a new dataObject into the database
   insertAttributeAt(int)   - 
Method in class weka.core. Instance 
 Inserts an attribute at the given position (0 to 
 numAttributes()).
   insertAttributeAt(Attribute, int)   - 
Method in class weka.core. Instances 
 Inserts an attribute at the given position (0 to
 numAttributes()) and sets all values to be missing.
   insertElementAt(Object, int)   - 
Method in class weka.core. FastVector 
 Inserts an element at the given position.
   insertElementAt(Object, int)   - 
Method in class weka.gui. CheckBoxList.CheckBoxListModel 
 Inserts the specified object as a component in this list at the 
 specified index.
   installLinearModels()   - 
Method in class weka.classifiers.trees.m5. RuleNode 
 Traverses the tree and installs linear models at each node.
   installSmoothedModels()   - 
Method in class weka.classifiers.trees.m5. RuleNode 
  
   Instance   - Class in  weka.core  Class for handling an instance.   Instance(Instance)   - 
Constructor for class weka.core. Instance 
 Constructor that copies the attribute values and the weight from
 the given instance.
   Instance(double, double[])   - 
Constructor for class weka.core. Instance 
 Constructor that inititalizes instance variable with given
 values.
   Instance(int)   - 
Constructor for class weka.core. Instance 
 Constructor of an instance that sets weight to one, all values to
 be missing, and the reference to the dataset to null.
   instance(int)   - 
Method in class weka.core. Instances 
 Returns the instance at the given position.
   INSTANCE_AVAILABLE   - 
Static variable in class weka.gui.beans. InstanceEvent 
  
   INSTANCE_AVAILABLE   - 
Static variable in class weka.gui.streams. InstanceEvent 
 Specifies that an instance is available
   InstanceComparator   - Class in  weka.core  A comparator for the Instance class.   InstanceComparator()   - 
Constructor for class weka.core. InstanceComparator 
 initializes the comparator and includes the class in the comparison
   InstanceComparator(boolean)   - 
Constructor for class weka.core. InstanceComparator 
 initializes the comparator
   InstanceCounter   - Class in  weka.gui.streams  A bean that counts instances streamed to it.   InstanceCounter()   - 
Constructor for class weka.gui.streams. InstanceCounter 
  
   InstanceEvent   - Class in  weka.gui.beans  Event that encapsulates a single instance or header information only   InstanceEvent(Object, Instance, int)   - 
Constructor for class weka.gui.beans. InstanceEvent 
 Creates a new  InstanceEvent  instance that encapsulates
 a single instance only.
   InstanceEvent(Object, Instances)   - 
Constructor for class weka.gui.beans. InstanceEvent 
 Creates a new  InstanceEvent  instance which encapsulates
 header information only.
   InstanceEvent(Object)   - 
Constructor for class weka.gui.beans. InstanceEvent 
  
   InstanceEvent   - Class in  weka.gui.streams  An event encapsulating an instance stream event.   InstanceEvent(Object, int)   - 
Constructor for class weka.gui.streams. InstanceEvent 
 Constructs an InstanceEvent with the specified source object and event 
 type
   InstanceJoiner   - Class in  weka.gui.streams  A bean that joins two streams of instances into one.   InstanceJoiner()   - 
Constructor for class weka.gui.streams. InstanceJoiner 
 Setup the initial states of the member variables
   InstanceListener   - Interface in  weka.gui.beans  Interface to something that can accept instance events   InstanceListener   - Interface in  weka.gui.streams  An interface for objects interested in listening to streams of instances.   InstanceLoader   - Class in  weka.gui.streams  A bean that produces a stream of instances from a file.   InstanceLoader()   - 
Constructor for class weka.gui.streams. InstanceLoader 
  
   instanceProduced(InstanceEvent)   - 
Method in class weka.gui.streams. InstanceCounter 
  
   instanceProduced(InstanceEvent)   - 
Method in class weka.gui.streams. InstanceJoiner 
  
   instanceProduced(InstanceEvent)   - 
Method in interface weka.gui.streams. InstanceListener 
  
   instanceProduced(InstanceEvent)   - 
Method in class weka.gui.streams. InstanceSavePanel 
  
   instanceProduced(InstanceEvent)   - 
Method in class weka.gui.streams. InstanceTable 
  
   instanceProduced(InstanceEvent)   - 
Method in class weka.gui.streams. InstanceViewer 
  
   InstanceProducer   - Interface in  weka.gui.streams  An interface for objects capable of producing streams of instances.   InstanceQuery   - Class in  weka.experiment  Convert the results of a database query into instances.   InstanceQuery()   - 
Constructor for class weka.experiment. InstanceQuery 
 Sets up the database drivers
   instanceRangeTipText()   - 
Method in class weka.filters.unsupervised.attribute. AbstractTimeSeries 
 Returns the tip text for this property
   Instances   - Class in  weka.core  Class for handling an ordered set of weighted instances.   Instances(Reader)   - 
Constructor for class weka.core. Instances 
 Reads an ARFF file from a reader, and assigns a weight of
 one to each instance.
   Instances(Reader, int)   - 
Constructor for class weka.core. Instances 
  Deprecated.   instead of using this method in conjunction with the
  readInstance(Reader)  method, one should use the
  ArffLoader  or  DataSource  class instead. 
   Instances(Instances)   - 
Constructor for class weka.core. Instances 
 Constructor copying all instances and references to
 the header information from the given set of instances.
   Instances(Instances, int)   - 
Constructor for class weka.core. Instances 
 Constructor creating an empty set of instances.
   Instances(Instances, int, int)   - 
Constructor for class weka.core. Instances 
 Creates a new set of instances by copying a
 subset of another set.
   Instances(String, FastVector, int)   - 
Constructor for class weka.core. Instances 
 Creates an empty set of instances.
   InstanceSavePanel   - Class in  weka.gui.streams  A bean that saves a stream of instances to a file.   InstanceSavePanel()   - 
Constructor for class weka.gui.streams. InstanceSavePanel 
  
   instancesDownBranch(int, Instances)   - 
Method in class weka.classifiers.trees.adtree. Splitter 
 Gets the subset of instances that apply to a particluar branch of the split.
   instancesDownBranch(int, Instances)   - 
Method in class weka.classifiers.trees.adtree. TwoWayNominalSplit 
 Gets the subset of instances that apply to a particluar branch of the split.
   instancesDownBranch(int, Instances)   - 
Method in class weka.classifiers.trees.adtree. TwoWayNumericSplit 
 Gets the subset of instances that apply to a particluar branch of the split.
   instancesIndicesTipText()   - 
Method in class weka.filters.unsupervised.instance. RemoveRange 
 Returns the tip text for this property
   InstancesResultListener   - Class in  weka.experiment  Outputs the received results in arff format to a Writer.   InstancesResultListener()   - 
Constructor for class weka.experiment. InstancesResultListener 
 Sets temporary file.
   InstancesSummaryPanel   - Class in  weka.gui  This panel just displays relation name, number of instances, and number of
 attributes.   InstancesSummaryPanel()   - 
Constructor for class weka.gui. InstancesSummaryPanel 
 Creates the instances panel with no initial instances.
   InstanceStreamToBatchMaker   - Class in  weka.gui.beans  Bean that converts an instance stream into a (batch) data set.   InstanceStreamToBatchMaker()   - 
Constructor for class weka.gui.beans. InstanceStreamToBatchMaker 
  
   InstanceStreamToBatchMakerBeanInfo   - Class in  weka.gui.beans  BeanInfo class for the InstanceStreamToBatchMaker bean   InstanceStreamToBatchMakerBeanInfo()   - 
Constructor for class weka.gui.beans. InstanceStreamToBatchMakerBeanInfo 
  
   InstanceTable   - Class in  weka.gui.streams  A bean that takes a stream of instances and displays in a table.   InstanceTable()   - 
Constructor for class weka.gui.streams. InstanceTable 
  
   instanceToSchema(Instance, MiningSchema)   - 
Method in class weka.core.pmml. MappingInfo 
 Convert an  Instance  to an array of values that matches the
 format of the mining schema.
   InstanceViewer   - Class in  weka.gui.streams  This is a very simple instance viewer - just displays the dataset as
 text output as it would be written to a file.   InstanceViewer()   - 
Constructor for class weka.gui.streams. InstanceViewer 
  
   intCount   - 
Variable in class weka.core. AttributeStats 
 The number of int-like values
   INTEGER   - 
Static variable in class weka.datagenerators.clusterers. SubspaceCluster 
 cluster subtype: integer
   INTEGER   - 
Static variable in class weka.experiment. DatabaseUtils 
 Type mapping for INTEGER used for reading experiment results.
   intercept()   - 
Method in class weka.classifiers.trees.m5. PreConstructedLinearModel 
 Return the intercept
   internalCacheSizeTipText()   - 
Method in class weka.classifiers.functions.supportVector. StringKernel 
 Returns the tip text for this property
   internalsTipText()   - 
Method in class weka.classifiers.bayes. WAODE 
 Returns the tip text for this property
   InterquartileRange   - Class in  weka.filters.unsupervised.attribute  A filter for detecting outliers and extreme values based on interquartile ranges.   InterquartileRange()   - 
Constructor for class weka.filters.unsupervised.attribute. InterquartileRange 
  
   intersectSubsets(ScatterSearchV1.Subset, ScatterSearchV1.Subset)   - 
Method in class weka.attributeSelection. ScatterSearchV1 
 Intersects two subsets
   IntervalEstimator   - Interface in  weka.classifiers  Interface for classifiers that can output confidence intervals   IntVector   - Class in  weka.core.matrix  A vector specialized on integers.   IntVector()   - 
Constructor for class weka.core.matrix. IntVector 
 Constructs a null vector.
   IntVector(int)   - 
Constructor for class weka.core.matrix. IntVector 
 Constructs an n-vector of zeros.
   IntVector(int, int)   - 
Constructor for class weka.core.matrix. IntVector 
 Constructs an n-vector of a constant
   IntVector(int[])   - 
Constructor for class weka.core.matrix. IntVector 
 Constructs a vector given an int array
   inverse()   - 
Method in class weka.core.matrix. Matrix 
 Matrix inverse or pseudoinverse
   inverseIterator()   - 
Method in class weka.associations.tertius. SimpleLinkedList 
  
   invertSelectionTipText()   - 
Method in class weka.core. NormalizableDistance 
 Returns the tip text for this property.
   invertSelectionTipText()   - 
Method in class weka.filters.supervised.attribute. Discretize 
 Returns the tip text for this property
   invertSelectionTipText()   - 
Method in class weka.filters.supervised.instance. Resample 
 Returns the tip text for this property.
   invertSelectionTipText()   - 
Method in class weka.filters.supervised.instance. StratifiedRemoveFolds 
 Returns the tip text for this property
   invertSelectionTipText()   - 
Method in class weka.filters.unsupervised.attribute. AbstractTimeSeries 
 Returns the tip text for this property
   invertSelectionTipText()   - 
Method in class weka.filters.unsupervised.attribute. Copy 
 Returns the tip text for this property
   invertSelectionTipText()   - 
Method in class weka.filters.unsupervised.attribute. Discretize 
 Returns the tip text for this property
   invertSelectionTipText()   - 
Method in class weka.filters.unsupervised.attribute. MathExpression 
 Returns the tip text for this property
   invertSelectionTipText()   - 
Method in class weka.filters.unsupervised.attribute. NominalToBinary 
 Returns the tip text for this property
   invertSelectionTipText()   - 
Method in class weka.filters.unsupervised.attribute. NumericCleaner 
 Returns the tip text for this property
   invertSelectionTipText()   - 
Method in class weka.filters.unsupervised.attribute. NumericToNominal 
 Returns the tip text for this property
   invertSelectionTipText()   - 
Method in class weka.filters.unsupervised.attribute. NumericTransform 
 Returns the tip text for this property
   invertSelectionTipText()   - 
Method in class weka.filters.unsupervised.attribute. RELAGGS 
 Returns the tip text for this property
   invertSelectionTipText()   - 
Method in class weka.filters.unsupervised.attribute. Remove 
 Returns the tip text for this property
   invertSelectionTipText()   - 
Method in class weka.filters.unsupervised.attribute. RemoveType 
 Returns the tip text for this property
   invertSelectionTipText()   - 
Method in class weka.filters.unsupervised.attribute. StringToWordVector 
 Returns the tip text for this property.
   invertSelectionTipText()   - 
Method in class weka.filters.unsupervised.instance. RemoveFolds 
 Returns the tip text for this property
   invertSelectionTipText()   - 
Method in class weka.filters.unsupervised.instance. RemoveFrequentValues 
 Returns the tip text for this property
   invertSelectionTipText()   - 
Method in class weka.filters.unsupervised.instance. RemovePercentage 
 Returns the tip text for this property
   invertSelectionTipText()   - 
Method in class weka.filters.unsupervised.instance. RemoveRange 
 Returns the tip text for this property
   invertSelectionTipText()   - 
Method in class weka.filters.unsupervised.instance. RemoveWithValues 
 Returns the tip text for this property
   invertSelectionTipText()   - 
Method in class weka.filters.unsupervised.instance. Resample 
 Returns the tip text for this property
   invertTipText()   - 
Method in class weka.filters.unsupervised.instance. RemoveMisclassified 
 Returns the tip text for this property
   invoke(String, Class[], Object[])   - 
Method in class weka.core. Jython 
 executes the specified method on the current interpreter and returns the 
 result, if any
   invoke(Object, String, Class[], Object[])   - 
Static method in class weka.core. Jython 
 executes the specified method and returns the result, if any
   invokeMain(String, String[])   - 
Static method in class weka.gui. SplashWindow 
 Invokes the main method of the provided class name.
   invokeMethod(String, String, String[])   - 
Static method in class weka.gui. SplashWindow 
 Invokes the named method of the provided class name.
   is(String)   - 
Method in class weka.core. Stopwords 
 Returns true if the given string is a stop word.
   IS   - 
Static variable in interface weka.filters.unsupervised.instance.subsetbyexpression. sym 
  
   isALeaf()   - 
Method in class weka.core.neighboursearch.balltrees. BallNode 
 Returns true if the node is a leaf node (if
 both its left and right child are null).
   isALeaf()   - 
Method in class weka.core.neighboursearch. CoverTree.CoverTreeNode 
 Returns whether if the node is a leaf or not.
   isALeaf()   - 
Method in class weka.core.neighboursearch.kdtrees. KDTreeNode 
 Checks if node is a leaf.
   isAllowed(Class, String)   - 
Method in class weka.core.xml. PropertyHandler 
 returns whether the given property (display name) is allowed for the
 given class.
   isAllowed(Object, String)   - 
Method in class weka.core.xml. PropertyHandler 
 returns whether the given property (display name) is allowed for the given
 object .
   isArff(String)   - 
Static method in class weka.core.converters. ConverterUtils.DataSource 
 returns whether the extension of the location is likely to be of ARFF
 format, i.e., ending in ".arff" or ".arff.gz" (case-insensitive).
   isAttribute()   - 
Method in enum weka.core. Capabilities.Capability 
 returns true if the capability is an attribute
   isAttributeCapability()   - 
Method in enum weka.core. Capabilities.Capability 
 returns true if the capability is an attribute capability
   isAveragable()   - 
Method in class weka.core. Attribute 
 Returns whether the attribute can be averaged meaningfully.
   isBoolean(int)   - 
Method in class weka.datagenerators.clusterers. SubspaceCluster 
 Returns true if attribute is boolean
   isBusy()   - 
Method in class weka.gui.beans. Associator 
 Returns true if.
   isBusy()   - 
Method in interface weka.gui.beans. BeanCommon 
 Returns true if.
   isBusy()   - 
Method in class weka.gui.beans. ClassAssigner 
 Returns true if.
   isBusy()   - 
Method in class weka.gui.beans. Classifier 
 Returns true if.
   isBusy()   - 
Method in class weka.gui.beans. ClassifierPerformanceEvaluator 
 Returns true if.
   isBusy()   - 
Method in class weka.gui.beans. ClassValuePicker 
 Returns true if.
   isBusy()   - 
Method in class weka.gui.beans. Clusterer 
 Returns true if.
   isBusy()   - 
Method in class weka.gui.beans. ClustererPerformanceEvaluator 
 Returns true if.
   isBusy()   - 
Method in class weka.gui.beans. CostBenefitAnalysis 
 Returns true if.
   isBusy()   - 
Method in class weka.gui.beans. CrossValidationFoldMaker 
 Returns true if.
   isBusy()   - 
Method in class weka.gui.beans. Filter 
 Returns true if.
   isBusy()   - 
Method in class weka.gui.beans. IncrementalClassifierEvaluator 
 Returns true if.
   isBusy()   - 
Method in class weka.gui.beans. InstanceStreamToBatchMaker 
 Returns true if.
   isBusy()   - 
Method in class weka.gui.beans. Loader 
 Returns true if.
   isBusy()   - 
Method in class weka.gui.beans. MetaBean 
 Returns true if.
   isBusy()   - 
Method in class weka.gui.beans. PredictionAppender 
 Returns true if.
   isBusy()   - 
Method in class weka.gui.beans. Saver 
 Returns true if.
   isBusy()   - 
Method in class weka.gui.beans. SerializedModelSaver 
 Returns true if.
   isBusy()   - 
Method in class weka.gui.beans. StripChart 
 Returns true if.
   isBusy()   - 
Method in class weka.gui.beans. TestSetMaker 
 Returns true if.
   isBusy()   - 
Method in class weka.gui.beans. TextViewer 
 Returns true if.
   isBusy()   - 
Method in class weka.gui.beans. TrainingSetMaker 
 Returns true if.
   isBusy()   - 
Method in class weka.gui.beans. TrainTestSplitMaker 
 Returns true if.
   isCellEditable(int, int)   - 
Method in class weka.gui.arffviewer. ArffTableModel 
 returns true if the cell at rowindex and columnindexis editable
   isCellEditable(int, int)   - 
Method in class weka.gui. SortedTableModel 
 Returns true if the cell at rowIndex and columnIndex is editable.
   isCellEditable(int, int)   - 
Method in class weka.gui.sql. ResultSetTableModel 
 returns true if the cell at rowindex and columnindexis editable.
   isChanged()   - 
Method in class weka.classifiers.bayes.net. EditableBayesNet 
 return true when current state differs from the state the network was last saved
   isChanged()   - 
Method in class weka.gui.arffviewer. ArffPanel 
 returns whether the content of the panel was changed
   isChanged()   - 
Method in class weka.gui. ViewerDialog 
 returns whether the data has been changed
   isClass()   - 
Method in class weka.associations.tertius. Predicate 
  
   isClass()   - 
Method in enum weka.core. Capabilities.Capability 
 returns true if the capability is a class
   isClassCapability()   - 
Method in enum weka.core. Capabilities.Capability 
 returns true if the capability is a other capability
   isClassname(String)   - 
Method in class weka.gui. SimpleCLIPanel.CommandlineCompletion 
 tests whether the given partial string is the name of a class with
 classpath - it basically tests, whether the string consists only
 of alphanumeric literals, underscores and dots.
   isConnected()   - 
Method in class weka.experiment. DatabaseUtils 
 Returns true if a database connection is active.
   isConnected()   - 
Method in class weka.gui.sql.event. ConnectionEvent 
 returns whether the connection is still open.
   isContainedBy(Instance)   - 
Method in class weka.associations.gsp. Element 
 Checks if an Element is contained by a given Instance.
   isContinuous()   - 
Method in class weka.datagenerators.clusterers. SubspaceClusterDefinition 
 checks, whether cluster sub type is continuous
   isCoreFileLoader(String)   - 
Static method in class weka.core.converters. ConverterUtils 
 checks whether the given class is one of the hardcoded core file loaders.
   isCoreFileSaver(String)   - 
Static method in class weka.core.converters. ConverterUtils 
 checks whether the given class is one of the hardcoded core file savers.
   isCover(Instance)   - 
Method in class weka.classifiers.rules. ConjunctiveRule 
 Whether the instance covered by this rule
   isCpuTime()   - 
Method in class weka.core. Debug.Clock 
 whether the measurement is based on the msecs returned from the System
 class or on the more accurate CPU time.
   isCursorScrollable()   - 
Method in class weka.experiment. DatabaseUtils 
 Checks whether cursors are scrollable in general, false otherwise 
 (also if not connected).
   isCursorScrollSensitive()   - 
Method in class weka.experiment. DatabaseUtils 
 Returns whether the cursors only support forward movement or are
 scroll sensitive (with ResultSet.CONCUR_READ_ONLY concurrency).
   isDate()   - 
Method in class weka.core. Attribute 
 Tests if the attribute is a date type.
   isDebug()   - 
Method in class weka.classifiers.bayes. BayesianLogisticRegression 
 Returns true if debug is turned on.
   isEmpty()   - 
Method in class weka.associations.gsp. Element 
 Checks if the Element contains any events.
   isEmpty()   - 
Method in class weka.associations.tertius. LiteralSet 
 Test if this set is empty.
   isEmpty()   - 
Method in class weka.associations.tertius. Rule 
 Test if this rule is empty.
   isEmpty()   - 
Method in class weka.associations.tertius. SimpleLinkedList 
  
   isEmpty()   - 
Method in class weka.classifiers.functions.pace. DiscreteFunction 
 Returns true if it is empty.
   isEmpty()   - 
Method in class weka.classifiers.functions.pace. PaceMatrix 
 Check if the matrix is empty
   isEmpty()   - 
Method in class weka.classifiers.lazy.kstar. KStarCache.CacheTable 
 Tests if this hashtable maps no keys to values.
   isEmpty()   - 
Method in class weka.core.matrix. DoubleVector 
 Checks if it is an empty vector
   isEmpty()   - 
Method in class weka.core.matrix. IntVector 
 Returns true if the vector is empty
   isEmpty()   - 
Method in class weka.core. Trie 
 Returns true if this collection contains no elements.
   isEnabled(Capabilities.Capability)   - 
Method in class weka.core. FindWithCapabilities 
 whether the given capability is enabled.
   isEnabled()   - 
Method in class weka.core. Memory 
 returns whether the memory management is enabled
   isEnabledNot(Capabilities.Capability)   - 
Method in class weka.core. FindWithCapabilities 
 whether the given "not to have" capability is enabled.
   isEqual(ScatterSearchV1.Subset)   - 
Method in class weka.attributeSelection. ScatterSearchV1.Subset 
  
   isFirstBatchDone()   - 
Method in class weka.filters. Filter 
 Returns true if the first batch of instances got processed.
   isFullRank()   - 
Method in class weka.core.matrix. QRDecomposition 
 Is the matrix full rank?
   isGaussian()   - 
Method in class weka.datagenerators.clusterers. SubspaceClusterDefinition 
 checks, whether cluster type is gaussian
   isHidden()   - 
Method in class weka.gui.beans. BeanConnection 
 Returns true if this connection is invisible
   isHierachic(String)   - 
Method in class weka.gui. HierarchyPropertyParser 
 Whether the given string has a hierachy structure with
 the seperators
   isIgnored(String)   - 
Method in class weka.core.xml. PropertyHandler 
 checks whether the given display name is an ignored property
   isIgnored(Class, String)   - 
Method in class weka.core.xml. PropertyHandler 
 checks whether the given display name of a certain class is an ignored 
 property.
   isIgnored(Object, String)   - 
Method in class weka.core.xml. PropertyHandler 
 checks whether the given display name of a given object is an ignored 
 property.
   isIncludedIn(Rule)   - 
Method in class weka.associations.tertius. Body 
 Test if this Body is included in a rule.
   isIncludedIn(Rule)   - 
Method in class weka.associations.tertius. Head 
 Test if this Head is included in a rule.
   isIncludedIn(Rule)   - 
Method in class weka.associations.tertius. LiteralSet 
 Test if this LiteralSet is included in a rule.
   isIncremental()   - 
Method in class weka.core.converters. ConverterUtils.DataSource 
 returns whether the loader is an incremental one.
   isInitialAnchorRandom()   - 
Method in class weka.core.neighboursearch.balltrees. MiddleOutConstructor 
 Gets whether if the initial anchor is chosen randomly.
   isInRange(double)   - 
Method in class weka.core. Attribute 
 Determines whether a value lies within the bounds of the attribute.
   isInRange(int)   - 
Method in class weka.core. Range 
 Gets whether the supplied cardinal number is included in the current
 range.
   isInteger()   - 
Method in class weka.datagenerators.clusterers. SubspaceClusterDefinition 
 checks, whether cluster sub type is integer
   isKeyword(String)   - 
Method in class weka.experiment. DatabaseUtils 
 Checks whether the given string is a reserved keyword.
   isKOML(String)   - 
Static method in class weka.core.xml. SerialUIDChanger 
 checks whether the given filename ends with ".koml"
   isLeaf()   - 
Method in class weka.classifiers.trees.m5. RuleNode 
 Return true if this node is a leaf
   isLeafReached()   - 
Method in class weka.gui. HierarchyPropertyParser 
 Whether the current position is a leaf
   isMissing(int)   - 
Method in class weka.core. Instance 
 Tests if a specific value is "missing".
   isMissing(Attribute)   - 
Method in class weka.core. Instance 
 Tests if a specific value is "missing".
   isMissing(int)   - 
Method in class weka.core. SparseInstance 
 Tests if a specific value is "missing".
   ISMISSING   - 
Static variable in interface weka.filters.unsupervised.instance.subsetbyexpression. sym 
  
   isMissingAt(int, int)   - 
Method in class weka.gui.arffviewer. ArffSortedTableModel 
 checks whether the value at the given position is missing
   isMissingAt(int, int)   - 
Method in class weka.gui.arffviewer. ArffTableModel 
 checks whether the value at the given position is missing
   isMissingSparse(int)   - 
Method in class weka.core. Instance 
 Tests if a specific value is "missing".
   isMissingValue(double)   - 
Static method in class weka.core. Instance 
 Tests if the given value codes "missing".
   isMonitoring()   - 
Method in class weka.gui. MemoryUsagePanel 
 Returns whether the thread is still running.
   isNewBatch()   - 
Method in class weka.filters. Filter 
 Returns true if the a new batch was started, either a new instance of the 
 filter was created or the batchFinished() method got called.
   isNewer(Object)   - 
Method in class weka.core. Version 
 checks whether this version is newer than the one from the given 
 version string
   isNominal()   - 
Method in class weka.core. Attribute 
 Test if the attribute is nominal.
   isNominal(int)   - 
Method in class weka.datagenerators.clusterers. SubspaceCluster 
 Returns true if attribute is nominal
   isNominal()   - 
Method in class weka.filters.unsupervised.instance. RemoveFrequentValues 
 Returns true if selection attribute is nominal.
   isNominal()   - 
Method in class weka.filters.unsupervised.instance. RemoveWithValues 
 Returns true if selection attribute is nominal.
   isNonsingular()   - 
Method in class weka.core.matrix. LUDecomposition 
 Is the matrix nonsingular?
   isNormalizeData()   - 
Method in class weka.classifiers.bayes. BayesianLogisticRegression 
 Returns true if the data is to be normalized first
   isNotificationEnabled()   - 
Method in class weka.gui.arffviewer. ArffSortedTableModel 
 returns whether the notification of changes is enabled
   isNotificationEnabled()   - 
Method in class weka.gui.arffviewer. ArffTableModel 
 returns whether the notification of changes is enabled
   isNullAt(int, int)   - 
Method in class weka.gui.sql. ResultSetTableModel 
 checks whether the value of the cell is NULL.
   isNumeric()   - 
Method in class weka.core. Attribute 
 Tests if the attribute is numeric.
   isNumeric()   - 
Method in class weka.filters.unsupervised.instance. RemoveWithValues 
 Returns true if selection attribute is numeric.
   isNumericAt(int)   - 
Method in class weka.gui.sql. ResultSetTableModel 
 returns whether the column at the given index is numeric.
   isOlder(Object)   - 
Method in class weka.core. Version 
 checks whether this version is older than the one from the given 
 version string
   isOpticsOutputs()   - 
Method in class weka.clusterers.forOPTICSAndDBScan.OPTICS_GUI. SERObject 
 Returns the flag for writing actions
   isOtherCapability()   - 
Method in enum weka.core. Capabilities.Capability 
 returns true if the capability is a class capability
   IsotonicRegression   - Class in  weka.classifiers.functions  Learns an isotonic regression model.   IsotonicRegression()   - 
Constructor for class weka.classifiers.functions. IsotonicRegression 
  
   isOutOfMemory()   - 
Method in class weka.core. Memory 
 checks if there's still enough memory left.
   isOutputFormatDefined()   - 
Method in class weka.filters. Filter 
 Returns whether the output format is ready to be collected
   isOutputFormatDefined()   - 
Method in class weka.filters.unsupervised.attribute. RemoveType 
 Returns whether the output format is ready to be collected
   isPaintable()   - 
Method in class weka.gui. CostMatrixEditor 
 Indicates whether the object can be represented graphically.
   isPaintable()   - 
Method in class weka.gui. FileEditor 
 Returns true since this editor is paintable.
   isPaintable()   - 
Method in class weka.gui. GenericArrayEditor 
 Returns true to indicate that we can paint a representation of the
 string array.
   isPaintable()   - 
Method in class weka.gui. GenericObjectEditor 
 Returns true to indicate that we can paint a representation of the
 Object.
   isPaintable()   - 
Method in class weka.gui. SimpleDateFormatEditor 
 Indicates whether the object can be represented graphically.
   isPanelSelected()   - 
Method in class weka.gui.arffviewer. ArffViewerMainPanel 
 checks whether a panel is currently selected
   isPresent()   - 
Static method in class weka.classifiers.functions. LibLINEAR 
 returns whether the liblinear classes are present or not, i.e.
   isPresent()   - 
Static method in class weka.classifiers.functions. LibSVM 
 returns whether the libsvm classes are present or not, i.e.
   isPresent()   - 
Static method in class weka.core. Jython 
 returns whether the Jython classes are present or not, i.e.
   isPresent()   - 
Static method in class weka.core.stemmers. SnowballStemmer 
 returns whether Snowball is present or not, i.e.
   isPresent()   - 
Static method in class weka.core.xml. KOML 
 returns whether KOML is present or not, i.e.
   isPresent()   - 
Static method in class weka.core.xml. XStream 
 returns whether XStream is present or not, i.e.
   isProcessed()   - 
Method in interface weka.clusterers.forOPTICSAndDBScan.DataObjects. DataObject 
 Gives information about the status of a dataObject
   isProcessed()   - 
Method in class weka.clusterers.forOPTICSAndDBScan.DataObjects. EuclidianDataObject 
 Gives information about the status of a dataObject
   isProcessed()   - 
Method in class weka.clusterers.forOPTICSAndDBScan.DataObjects. ManhattanDataObject 
 Gives information about the status of a dataObject
   isRandom()   - 
Method in class weka.datagenerators.clusterers. SubspaceClusterDefinition 
 checks, whether cluster type is random
   isReadOnly()   - 
Method in class weka.gui.arffviewer. ArffPanel 
 returns whether the model is read-only
   isReadOnly()   - 
Method in class weka.gui.arffviewer. ArffSortedTableModel 
 returns whether the model is read-only
   isReadOnly()   - 
Method in class weka.gui.arffviewer. ArffTable 
 returns whether the model is read-only
   isReadOnly()   - 
Method in class weka.gui.arffviewer. ArffTableModel 
 returns whether the model is read-only
   isRegular()   - 
Method in class weka.core. Attribute 
 Returns whether the attribute values are equally spaced.
   isRelationValued()   - 
Method in class weka.core. Attribute 
 Tests if the attribute is relation valued.
   isResultRequired(ResultProducer, Object[])   - 
Method in class weka.experiment. AveragingResultProducer 
 Determines whether the results for a specified key must be
 generated.
   isResultRequired(ResultProducer, Object[])   - 
Method in class weka.experiment. CSVResultListener 
 Always says a result is required.
   isResultRequired(ResultProducer, Object[])   - 
Method in class weka.experiment. DatabaseResultListener 
 Always says a result is required.
   isResultRequired(ResultProducer, Object[])   - 
Method in class weka.experiment. DatabaseResultProducer 
 Determines whether the results for a specified key must be
 generated.
   isResultRequired(ResultProducer, Object[])   - 
Method in class weka.experiment. LearningRateResultProducer 
 Determines whether the results for a specified key must be
 generated.
   isResultRequired(ResultProducer, Object[])   - 
Method in interface weka.experiment. ResultListener 
 Determines whether the results for a specified key must be
 generated.
   isRootReached()   - 
Method in class weka.gui. HierarchyPropertyParser 
 Whether the current position is the root
   isRunning()   - 
Method in class weka.core. Debug.Clock 
 whether the time is still being clocked
   isSaved()   - 
Method in class weka.classifiers.bayes.net. EditableBayesNet 
 indicate the network state was saved
   isSequentialAttIndexValid()   - 
Method in class weka.classifiers.lazy. LBR.Indexes 
 Returns whether or not the Sequential Attribute Index requires rebuilding due to a change
   isSequentialInstanceIndexValid()   - 
Method in class weka.classifiers.lazy. LBR.Indexes 
 Returns whether or not the Sequential Instance Index requires rebuilding due to a change
   isSerializable(String)   - 
Static method in class weka.core. SerializationHelper 
 checks whether a class is serializable.
   isSerializable(Class)   - 
Static method in class weka.core. SerializationHelper 
 checks whether a class is serializable.
   isShowCoreDistances()   - 
Method in class weka.clusterers.forOPTICSAndDBScan.OPTICS_GUI. GraphPanel 
 Returns the flag for showCoreDistances
   isShowReachabilityDistances()   - 
Method in class weka.clusterers.forOPTICSAndDBScan.OPTICS_GUI. GraphPanel 
 Returns the flag for showReachabilityDistances
   isSorted()   - 
Method in class weka.gui. SortedTableModel 
 returns whether the table was sorted
   isSPD()   - 
Method in class weka.core.matrix. CholeskyDecomposition 
 Is the matrix symmetric and positive definite?
   isSquare()   - 
Method in class weka.core.matrix. Matrix 
 returns whether the matrix is a square matrix or not.
   isStopword(String)   - 
Static method in class weka.core. Stopwords 
 Returns true if the given string is a stop word.
   isStreamableFilter()   - 
Method in class weka.filters. MultiFilter 
 tests whether all the enclosed filters are streamable
   isString()   - 
Method in class weka.core. Attribute 
 Tests if the attribute is a string.
   isStructureOnly()   - 
Method in class weka.gui.beans. DataSetEvent 
 Returns true if the encapsulated instances
 contain just header information
   isStructureOnly()   - 
Method in class weka.gui.beans. TestSetEvent 
 Returns true if the encapsulated instances
 contain just header information
   isStructureOnly()   - 
Method in class weka.gui.beans. TrainingSetEvent 
 Returns true if the encapsulated instances
 contain just header information
   isSubclass(String, String)   - 
Static method in class weka.core. ClassDiscovery 
 Checks whether the "otherclass" is a subclass of the given "superclass".
   isSubclass(Class, Class)   - 
Static method in class weka.core. ClassDiscovery 
 Checks whether the "otherclass" is a subclass of the given "superclass".
   isSymmetric()   - 
Method in class weka.core. Matrix 
  Deprecated.  Returns true if the matrix is symmetric.
   isSymmetric()   - 
Method in class weka.core.matrix. Matrix 
 Returns true if the matrix is symmetric.
   isUndoEnabled()   - 
Method in interface weka.core. Undoable 
 returns whether undo support is enabled
   isUndoEnabled()   - 
Method in class weka.gui.arffviewer. ArffPanel 
 returns whether undo support is enabled
   isUndoEnabled()   - 
Method in class weka.gui.arffviewer. ArffSortedTableModel 
 returns whether undo support is enabled
   isUndoEnabled()   - 
Method in class weka.gui.arffviewer. ArffTableModel 
 returns whether undo support is enabled
   isUniform()   - 
Method in class weka.datagenerators.clusterers. SubspaceClusterDefinition 
 checks, whether cluster type is uniform
   isUseK2Prior()   - 
Method in class weka.classifiers.bayes.net.estimate. BMAEstimator 
 Returns whether K2 prior is used
   isUseK2Prior()   - 
Method in class weka.classifiers.bayes.net.estimate. MultiNomialBMAEstimator 
  
   isUseVariant1()   - 
Method in class weka.classifiers.functions.supportVector. RegSMOImproved 
 Whether variant 1 is used
   itemAt(int)   - 
Method in class weka.associations. ItemSet 
 Gest the index of the value of the specified attribute
   items()   - 
Method in class weka.associations. ItemSet 
 Gest the item set as an int array
   ItemSet   - Class in  weka.associations  Class for storing a set of items.   ItemSet(int)   - 
Constructor for class weka.associations. ItemSet 
 Constructor
   ItemSet(int, int[])   - 
Constructor for class weka.associations. ItemSet 
 Constructor
   ItemSet(int[])   - 
Constructor for class weka.associations. ItemSet 
 Contsructor
   itemStateChanged(ItemEvent)   - 
Method in class weka.gui.treevisualizer. TreeVisualizer 
 Performs the action associated with the ItemEvent.
   IteratedLovinsStemmer   - Class in  weka.core.stemmers  An iterated version of the Lovins stemmer.   IteratedLovinsStemmer()   - 
Constructor for class weka.core.stemmers. IteratedLovinsStemmer 
  
   IteratedSingleClassifierEnhancer   - Class in  weka.classifiers  Abstract utility class for handling settings common to
 meta classifiers that build an ensemble from a single base learner.   IteratedSingleClassifierEnhancer()   - 
Constructor for class weka.classifiers. IteratedSingleClassifierEnhancer 
  
   iterationCounter   - 
Variable in class weka.classifiers.bayes. BayesianLogisticRegression 
 Iteration counter
   IterativeClassifier   - Interface in  weka.classifiers  Interface for classifiers that can induce models of growing
 complexity one step at a time.   iterator()   - 
Method in class weka.associations.tertius. SimpleLinkedList 
  
   iterator()   - 
Method in class weka.core. Trie 
 Returns an iterator over the elements in this collection.
 
 
    
 J  
 
   J48   - Class in  weka.classifiers.trees  Class for generating a pruned or unpruned C4.5 decision tree.   J48()   - 
Constructor for class weka.classifiers.trees. J48 
  
   J48graft   - Class in  weka.classifiers.trees  Class for generating a grafted (pruned or unpruned) C4.5 decision tree.   J48graft()   - 
Constructor for class weka.classifiers.trees. J48graft 
  
   Javadoc   - Class in  weka.core  Abstract superclass for classes that generate Javadoc comments and replace
 the content between certain comment tags.   Javadoc()   - 
Constructor for class weka.core. Javadoc 
  
   JComponentWriter   - Class in  weka.gui.visualize  This class takes any JComponent and outputs it to a file.   JComponentWriter()   - 
Constructor for class weka.gui.visualize. JComponentWriter 
 initializes the object
   JComponentWriter(JComponent)   - 
Constructor for class weka.gui.visualize. JComponentWriter 
 initializes the object with the given Component
   JComponentWriter(JComponent, File)   - 
Constructor for class weka.gui.visualize. JComponentWriter 
 initializes the object with the given Component and filename
   JListHelper   - Class in  weka.gui  A helper class for JList GUI elements with DefaultListModel or 
 derived models.   JListHelper()   - 
Constructor for class weka.gui. JListHelper 
  
   joinOptions(String[])   - 
Static method in class weka.core. Utils 
 Joins all the options in an option array into a single string,
 as might be used on the command line.
   joinSubsets(ScatterSearchV1.Subset, ScatterSearchV1.Subset)   - 
Method in class weka.attributeSelection. ScatterSearchV1 
 Join two subsets
   JPEGWriter   - Class in  weka.gui.visualize  This class takes any JComponent and outputs it to a JPEG-file.   JPEGWriter()   - 
Constructor for class weka.gui.visualize. JPEGWriter 
 initializes the object.
   JPEGWriter(JComponent)   - 
Constructor for class weka.gui.visualize. JPEGWriter 
 initializes the object with the given Component.
   JPEGWriter(JComponent, File)   - 
Constructor for class weka.gui.visualize. JPEGWriter 
 initializes the object with the given Component and filename.
   JRip   - Class in  weka.classifiers.rules  This class implements a propositional rule learner, Repeated Incremental Pruning to Produce Error Reduction (RIPPER), which was proposed by William W.   JRip()   - 
Constructor for class weka.classifiers.rules. JRip 
  
   JTableHelper   - Class in  weka.gui  A helper class for JTable, e.g.   JTableHelper(JTable)   - 
Constructor for class weka.gui. JTableHelper 
 initializes the object
   Jython   - Class in  weka.core  A helper class for  Jython .   Jython()   - 
Constructor for class weka.core. Jython 
 default constructor, tries to instantiate a Python Interpreter
   JythonObject   - Interface in  weka.core  An indicator interface for Jython objects.   JythonSerializableObject   - Interface in  weka.core  An indicator interface for serializable Jython objects. 
 
    
 K  
 
   K2   - Class in  weka.classifiers.bayes.net.search.global  This Bayes Network learning algorithm uses a hill climbing algorithm restricted by an order on the variables. 
  
 For more information see: 
  
 G.F.   K2()   - 
Constructor for class weka.classifiers.bayes.net.search.global. K2 
  
   K2   - Class in  weka.classifiers.bayes.net.search.local  This Bayes Network learning algorithm uses a hill climbing algorithm restricted by an order on the variables. 
  
 For more information see: 
  
 G.F.   K2()   - 
Constructor for class weka.classifiers.bayes.net.search.local. K2 
  
   k_nextNeighbourQuery(int, double, DataObject)   - 
Method in interface weka.clusterers.forOPTICSAndDBScan.Databases. Database 
 Emits the k next-neighbours and performs an epsilon-range-query at the parallel.
   k_nextNeighbourQuery(int, double, DataObject)   - 
Method in class weka.clusterers.forOPTICSAndDBScan.Databases. SequentialDatabase 
 Emits the k next-neighbours and performs an epsilon-range-query at the parallel.
   kappa()   - 
Method in class weka.classifiers. Evaluation 
 Returns value of kappa statistic if class is nominal.
   KBInformation()   - 
Method in class weka.classifiers. Evaluation 
 Return the total Kononenko & Bratko Information score in bits
   KBMeanInformation()   - 
Method in class weka.classifiers. Evaluation 
 Return the Kononenko & Bratko Information score in bits per 
 instance.
   KBRelativeInformation()   - 
Method in class weka.classifiers. Evaluation 
 Return the Kononenko & Bratko Relative Information score
   KDConditionalEstimator   - Class in  weka.estimators  Conditional probability estimator for a numeric domain conditional upon
 a discrete domain (utilises separate kernel estimators for each discrete
 conditioning value).   KDConditionalEstimator(int, double)   - 
Constructor for class weka.estimators. KDConditionalEstimator 
 Constructor
   KDDataGenerator   - Class in  weka.gui.boundaryvisualizer  KDDataGenerator.   KDDataGenerator()   - 
Constructor for class weka.gui.boundaryvisualizer. KDDataGenerator 
  
   KDTree   - Class in  weka.core.neighboursearch  Class implementing the KDTree search algorithm for nearest neighbour search. 
 The connection to dataset is only a reference.   KDTree()   - 
Constructor for class weka.core.neighboursearch. KDTree 
 Creates a new instance of KDTree.
   KDTree(Instances)   - 
Constructor for class weka.core.neighboursearch. KDTree 
 Creates a new instance of KDTree.
   KDTreeNode   - Class in  weka.core.neighboursearch.kdtrees  A class representing a KDTree node.   KDTreeNode()   - 
Constructor for class weka.core.neighboursearch.kdtrees. KDTreeNode 
 Constructor.
   KDTreeNode(int, int, int, double[][])   - 
Constructor for class weka.core.neighboursearch.kdtrees. KDTreeNode 
 Constructor.
   KDTreeNode(int, int, int, double[][], double[][])   - 
Constructor for class weka.core.neighboursearch.kdtrees. KDTreeNode 
  
   KDTreeNodeSplitter   - Class in  weka.core.neighboursearch.kdtrees  Class that splits up a KDTreeNode.   KDTreeNodeSplitter()   - 
Constructor for class weka.core.neighboursearch.kdtrees. KDTreeNodeSplitter 
 default constructor.
   KDTreeNodeSplitter(int[], Instances, EuclideanDistance)   - 
Constructor for class weka.core.neighboursearch.kdtrees. KDTreeNodeSplitter 
 Creates a new instance of KDTreeNodeSplitter.
   KDTreeTipText()   - 
Method in class weka.clusterers. XMeans 
 Returns the tip text for this property.
   Kernel   - Class in  weka.classifiers.functions.supportVector  Abstract kernel.   Kernel()   - 
Constructor for class weka.classifiers.functions.supportVector. Kernel 
  
   KernelEstimator   - Class in  weka.estimators  Simple kernel density estimator.   KernelEstimator(double)   - 
Constructor for class weka.estimators. KernelEstimator 
 Constructor that takes a precision argument.
   KernelEvaluation   - Class in  weka.classifiers.functions.supportVector  Class for evaluating Kernels.   KernelEvaluation()   - 
Constructor for class weka.classifiers.functions.supportVector. KernelEvaluation 
 default constructor
   kernelFactorExpressionTipText()   - 
Method in class weka.filters.unsupervised.attribute. KernelFilter 
 Returns the tip text for this property
   KernelFilter   - Class in  weka.filters.unsupervised.attribute  Converts the given set of predictor variables into a kernel matrix.   KernelFilter()   - 
Constructor for class weka.filters.unsupervised.attribute. KernelFilter 
  
   kernelMatrixFileTipText()   - 
Method in class weka.classifiers.functions.supportVector. PrecomputedKernelMatrixKernel 
 Returns the tip text for this property
   kernelTipText()   - 
Method in class weka.classifiers.functions. GaussianProcesses 
 Returns the tip text for this property
   kernelTipText()   - 
Method in class weka.classifiers.functions. SMO 
 Returns the tip text for this property
   kernelTipText()   - 
Method in class weka.classifiers.functions. SMOreg 
 Returns the tip text for this property
   kernelTipText()   - 
Method in class weka.classifiers.mi. MISMO 
 Returns the tip text for this property
   kernelTipText()   - 
Method in class weka.classifiers.mi. MISVM 
 Returns the tip text for this property
   kernelTipText()   - 
Method in class weka.filters.unsupervised.attribute. KernelFilter 
 Returns the tip text for this property
   KERNELTYPE_LINEAR   - 
Static variable in class weka.classifiers.functions. LibSVM 
 kernel type linear: u'*v
   KERNELTYPE_POLYNOMIAL   - 
Static variable in class weka.classifiers.functions. LibSVM 
 kernel type polynomial: (gamma*u'*v + coef0)^degree
   KERNELTYPE_RBF   - 
Static variable in class weka.classifiers.functions. LibSVM 
 kernel type radial basis function: exp(-gamma*|u-v|^2)
   KERNELTYPE_SIGMOID   - 
Static variable in class weka.classifiers.functions. LibSVM 
 kernel type sigmoid: tanh(gamma*u'*v + coef0)
   kernelTypeTipText()   - 
Method in class weka.classifiers.functions. LibSVM 
 Returns the tip text for this property
   key   - 
Variable in class weka.classifiers.lazy.kstar. KStarCache.TableEntry 
 attribute value
   keyFieldNameTipText()   - 
Method in class weka.experiment. AveragingResultProducer 
 Returns the tip text for this property
   keyIterator()   - 
Method in interface weka.clusterers.forOPTICSAndDBScan.Databases. Database 
 Returns an iterator over all the keys
   keyIterator()   - 
Method in class weka.clusterers.forOPTICSAndDBScan.Databases. SequentialDatabase 
 Returns an iterator over all the keys
   keys()   - 
Method in class weka.core.xml. MethodHandler 
 returns an enumeration over all currently stored custom methods, i.e.
   keysTipText()   - 
Method in class weka.core.converters. DatabaseLoader 
 the tip text for this property
   kFoldCV(BayesNet, int)   - 
Method in class weka.classifiers.bayes.net.search.global. GlobalScoreSearchAlgorithm 
 kFoldCV uses k-fold cross validation to measure the accuracy of a Bayes
 network classifier.
   KKConditionalEstimator   - Class in  weka.estimators  Conditional probability estimator for a numeric domain conditional upon
 a numeric domain.   KKConditionalEstimator(double)   - 
Constructor for class weka.estimators. KKConditionalEstimator 
 Constructor
   KMeansInpiredMethod   - Class in  weka.core.neighboursearch.kdtrees  The class that splits a node into two such that the overall sum of squared distances of points to their centres on both sides of the (axis-parallel) splitting plane is minimum. 
  
 For more information see also: 
  
 Ashraf Masood Kibriya (2007).   KMeansInpiredMethod()   - 
Constructor for class weka.core.neighboursearch.kdtrees. KMeansInpiredMethod 
  
   kNearestNeighbours(Instance, int)   - 
Method in class weka.core.neighboursearch. BallTree 
 Returns k nearest instances in the current neighbourhood to the supplied
 instance.
   kNearestNeighbours(Instance, int)   - 
Method in class weka.core.neighboursearch. CoverTree 
 Returns k-NNs of a given target instance, from among the previously
 supplied training instances (supplied through setInstances method)
 P.S.: May return more than k-NNs if more one instances have
 the same distance to the target as the kth NN.
   kNearestNeighbours(Instance, int)   - 
Method in class weka.core.neighboursearch. KDTree 
 Returns the k nearest neighbours of the supplied instance.
   kNearestNeighbours(Instance, int)   - 
Method in class weka.core.neighboursearch. LinearNNSearch 
 Returns k nearest instances in the current neighbourhood to the supplied
 instance.
   kNearestNeighbours(Instance, int)   - 
Method in class weka.core.neighboursearch. NearestNeighbourSearch 
 Returns k nearest instances in the current neighbourhood to the supplied
 instance.
   KNNTipText()   - 
Method in class weka.classifiers.lazy. IBk 
 Returns the tip text for this property.
   KNNTipText()   - 
Method in class weka.classifiers.lazy. LWL 
 Returns the tip text for this property.
   KnowledgeFlow   - Class in  weka.gui.beans  Startup class for the KnowledgeFlow.   KnowledgeFlow()   - 
Constructor for class weka.gui.beans. KnowledgeFlow 
  
   KnowledgeFlowApp   - Class in  weka.gui.beans  Main GUI class for the KnowledgeFlow.   KnowledgeFlowApp(boolean)   - 
Constructor for class weka.gui.beans. KnowledgeFlowApp 
 Creates a new  KnowledgeFlowApp  instance.
   KOML   - Class in  weka.core.xml  This class is a helper class for XML serialization using  KOML  .   KOML()   - 
Constructor for class weka.core.xml. KOML 
  
   komlToBinary(String, String)   - 
Static method in class weka.core.xml. SerialUIDChanger 
 converts a KOML file into a binary one
   KOMLV   - 
Static variable in class weka.gui.beans. SerializedModelSaver 
  
   KStar   - Class in  weka.classifiers.lazy  K* is an instance-based classifier, that is the class of a test instance is based upon the class of those training instances similar to it, as determined by some similarity function.   KStar()   - 
Constructor for class weka.classifiers.lazy. KStar 
  
   KStarCache   - Class in  weka.classifiers.lazy.kstar  A class representing the caching system used to keep track of each attribute
 value and its corresponding scale factor or stop parameter.   KStarCache()   - 
Constructor for class weka.classifiers.lazy.kstar. KStarCache 
  
   KStarCache.CacheTable   - Class in  weka.classifiers.lazy.kstar  A custom hashtable class to support the caching system.   KStarCache.CacheTable(int, float)   - 
Constructor for class weka.classifiers.lazy.kstar. KStarCache.CacheTable 
 Constructs a new hashtable with a default capacity and load factor.
   KStarCache.CacheTable()   - 
Constructor for class weka.classifiers.lazy.kstar. KStarCache.CacheTable 
 Constructs a new hashtable with a default capacity and load factor.
   KStarCache.TableEntry   - Class in  weka.classifiers.lazy.kstar  Hashtable collision list.   KStarCache.TableEntry(int, double, double, double, KStarCache.TableEntry)   - 
Constructor for class weka.classifiers.lazy.kstar. KStarCache.TableEntry 
 Constructor
   KStarConstants   - Interface in  weka.classifiers.lazy.kstar      KStarNominalAttribute   - Class in  weka.classifiers.lazy.kstar  A custom class which provides the environment for computing the
 transformation probability of a specified test instance nominal
 attribute to a specified train instance nominal attribute.   KStarNominalAttribute(Instance, Instance, int, Instances, int[][], KStarCache)   - 
Constructor for class weka.classifiers.lazy.kstar. KStarNominalAttribute 
 Constructor
   KStarNumericAttribute   - Class in  weka.classifiers.lazy.kstar  A custom class which provides the environment for computing the
 transformation probability of a specified test instance numeric
 attribute to a specified train instance numeric attribute.   KStarNumericAttribute(Instance, Instance, int, Instances, int[][], KStarCache)   - 
Constructor for class weka.classifiers.lazy.kstar. KStarNumericAttribute 
 Constructor
   KStarWrapper   - Class in  weka.classifiers.lazy.kstar      KStarWrapper()   - 
Constructor for class weka.classifiers.lazy.kstar. KStarWrapper 
  
   kthSmallestValue(Attribute, int)   - 
Method in class weka.core. Instances 
 Returns the kth-smallest attribute value of a numeric attribute.
   kthSmallestValue(int, int)   - 
Method in class weka.core. Instances 
 Returns the kth-smallest attribute value of a numeric attribute.
   kthSmallestValue(int[], int)   - 
Static method in class weka.core. Utils 
 Returns the kth-smallest value in the array.
   kthSmallestValue(double[], int)   - 
Static method in class weka.core. Utils 
 Returns the kth-smallest value in the array
   kullback(double[], double[], double[], double[], int)   - 
Method in class weka.classifiers.mi. MINND 
 This function calculates the Kullback Leibler distance between
 two normal distributions.
   KValueTipText()   - 
Method in class weka.classifiers.trees. RandomTree 
 Returns the tip text for this property
 
 
    
 L  
 
   LabeledItemSet   - Class in  weka.associations  Class for storing a set of items together with a  class label.   LabeledItemSet(int, int)   - 
Constructor for class weka.associations. LabeledItemSet 
 Constructor
   labelsTipText()   - 
Method in class weka.filters.unsupervised.attribute. AddValues 
 Returns the tip text for this property
   LADTree   - Class in  weka.classifiers.trees  Class for generating a multi-class alternating decision tree using the LogitBoost strategy.   LADTree()   - 
Constructor for class weka.classifiers.trees. LADTree 
  
   LAGDHillClimber   - Class in  weka.classifiers.bayes.net.search.local  This Bayes Network learning algorithm uses a Look Ahead Hill Climbing algorithm called LAGD Hill Climbing.   LAGDHillClimber()   - 
Constructor for class weka.classifiers.bayes.net.search.local. LAGDHillClimber 
  
   lambdaTipText()   - 
Method in class weka.classifiers.functions. SPegasos 
 Returns the tip text for this property
   lambdaTipText()   - 
Method in class weka.classifiers.functions.supportVector. StringKernel 
 Returns the tip text for this property
   LaplaceEstimate(double, double, double)   - 
Method in class weka.classifiers.bayes. AODEsr 
 Returns the probability estimate, using laplace correction
   laplaceForSubsetOfInterest()   - 
Method in class weka.classifiers.trees.j48. GraftSplit 
  
   LaplacePriorImpl   - Class in  weka.classifiers.bayes.blr  Implementation of the Gaussian Prior update function based on modified
  CLG Algorithm (CLG-Lasso) with a certain Trust Region Update based
 on Laplace Priors.   LaplacePriorImpl()   - 
Constructor for class weka.classifiers.bayes.blr. LaplacePriorImpl 
  
   laplaceProb(int)   - 
Method in class weka.classifiers.trees.j48. Distribution 
 Returns relative frequency of class over all bags with
 Laplace correction.
   laplaceProb(int, int)   - 
Method in class weka.classifiers.trees.j48. Distribution 
 Returns relative frequency of class for given bag.
   laplaceUpdate(int, double)   - 
Method in class weka.classifiers.bayes.blr. LaplacePriorImpl 
 This is the CLG-lasso update function described in the
   LAPLACIAN   - 
Static variable in class weka.classifiers.bayes. BayesianLogisticRegression 
  
   last()   - 
Method in class weka.core.neighboursearch.covertrees. Stack 
 Returns the last element in the stack.
   LAST   - 
Static variable in class weka.filters.unsupervised.attribute. ClassAssigner 
 use the last attribute as class.
   lastActionMsg()   - 
Method in class weka.classifiers.bayes.net. EditableBayesNet 
 get message representing the last action performed on the network
   lastElement()   - 
Method in class weka.core. FastVector 
 Returns the last element of the vector.
   lastElement()   - 
Method in class weka.gui. CheckBoxList.CheckBoxListModel 
 Returns the last component of the list.
   lastIndexOf(Object)   - 
Method in class weka.gui. CheckBoxList.CheckBoxListModel 
 Returns the index of the last occurrence of elem.
   lastIndexOf(Object, int)   - 
Method in class weka.gui. CheckBoxList.CheckBoxListModel 
 Searches backwards for elem, starting from the specified index, 
 and returns an index to it.
   lastInstance()   - 
Method in class weka.core. Instances 
 Returns the last instance in the set.
   LatentSemanticAnalysis   - Class in  weka.attributeSelection  Performs latent semantic analysis and transformation of the data.   LatentSemanticAnalysis()   - 
Constructor for class weka.attributeSelection. LatentSemanticAnalysis 
  
   launchNext(int, int)   - 
Method in class weka.experiment. RemoteExperiment 
 Launch a sub experiment on a remote host
   layoutCompleted(LayoutCompleteEvent)   - 
Method in class weka.classifiers.bayes.net. GUI 
 This method is an implementation for LayoutCompleteEventListener class.
   layoutCompleted(LayoutCompleteEvent)   - 
Method in class weka.gui.graphvisualizer. GraphVisualizer 
 This method is an implementation for LayoutCompleteEventListener
 class.
   layoutCompleted(LayoutCompleteEvent)   - 
Method in interface weka.gui.graphvisualizer. LayoutCompleteEventListener 
  
   LayoutCompleteEvent   - Class in  weka.gui.graphvisualizer  This is an event which is fired by a LayoutEngine once
 a LayoutEngine finishes laying out the graph, so
 that the Visualizer can repaint the screen to show
 the changes.   LayoutCompleteEvent(Object)   - 
Constructor for class weka.gui.graphvisualizer. LayoutCompleteEvent 
  
   LayoutCompleteEventListener   - Interface in  weka.gui.graphvisualizer  This interface should be implemented by any class
 which needs to receive LayoutCompleteEvents from
 the LayoutEngine.   LayoutEngine   - Interface in  weka.gui.graphvisualizer  This interface class has been added to facilitate the addition
 of other layout engines to this package.   layoutGraph(FastVector, FastVector)   - 
Method in class weka.classifiers.bayes.net. EditableBayesNet 
 set positions of all nodes
   layoutGraph()   - 
Method in class weka.gui.graphvisualizer. GraphVisualizer 
 This method lays out the graph by calling the
 LayoutEngine's layoutGraph() method.
   layoutGraph()   - 
Method in class weka.gui.graphvisualizer. HierarchicalBCEngine 
 This method does a complete layout of the graph which includes
 removing cycles, assigning levels to nodes, reducing edge crossings
 and laying out the vertices horizontally for better visibility.
   layoutGraph()   - 
Method in interface weka.gui.graphvisualizer. LayoutEngine 
 This method lays out the graph for better visualization
   LBR   - Class in  weka.classifiers.lazy  Lazy Bayesian Rules Classifier.   LBR()   - 
Constructor for class weka.classifiers.lazy. LBR 
  
   LBR.Indexes   - Class in  weka.classifiers.lazy  Class for handling instances and the associated attributes.   LBR.Indexes(int, int, boolean, int)   - 
Constructor for class weka.classifiers.lazy. LBR.Indexes 
 constructor
   LBR.Indexes(LBR.Indexes)   - 
Constructor for class weka.classifiers.lazy. LBR.Indexes 
 constructor
   LE   - 
Static variable in interface weka.core.mathematicalexpression. sym 
  
   LE   - 
Static variable in interface weka.filters.unsupervised.instance.subsetbyexpression. sym 
  
   LearningRateResultProducer   - Class in  weka.experiment  Tells a sub-ResultProducer to reproduce the current run for varying sized subsamples of the dataset.   LearningRateResultProducer()   - 
Constructor for class weka.experiment. LearningRateResultProducer 
  
   learningRateTipText()   - 
Method in class weka.classifiers.functions. MultilayerPerceptron 
  
   leastExplainingColumn(PaceMatrix, IntVector, int, int)   - 
Method in class weka.classifiers.functions.pace. PaceMatrix 
 Returns the index of the column that has the smallest (squared)
  response, when the column is moved to become the (ks-1)-th
  column.
   LeastMedSq   - Class in  weka.classifiers.functions  Implements a least median sqaured linear regression utilising the existing weka LinearRegression class to form predictions.   LeastMedSq()   - 
Constructor for class weka.classifiers.functions. LeastMedSq 
  
   leaveOneOut(LBR.Indexes, int[][][], int[], boolean[])   - 
Method in class weka.classifiers.lazy. LBR 
 Leave-one-out strategy.
   leaveOneOutCV(BayesNet)   - 
Method in class weka.classifiers.bayes.net.search.global. GlobalScoreSearchAlgorithm 
 LeaveOneOutCV returns the accuracy calculated using Leave One Out
 cross validation.
   LED24   - Class in  weka.datagenerators.classifiers.classification  This generator produces data for a display with 7 LEDs.   LED24()   - 
Constructor for class weka.datagenerators.classifiers.classification. LED24 
 initializes the generator with default values
   LEFT_PARENTHESES   - 
Variable in class weka.experiment. ResultMatrix 
 the left parentheses for enumerating cols/rows
   leftNode()   - 
Method in class weka.classifiers.trees.m5. RuleNode 
 Get the left child of this node
   leftSide(Instances)   - 
Method in class weka.classifiers.trees.j48. BinC45Split 
 Prints left side of condition.
   leftSide(Instances)   - 
Method in class weka.classifiers.trees.j48. C45Split 
 Prints left side of condition..
   leftSide(Instances)   - 
Method in class weka.classifiers.trees.j48. ClassifierSplitModel 
 Prints left side of condition satisfied by instances.
   leftSide(Instances)   - 
Method in class weka.classifiers.trees.j48. GraftSplit 
 Prints left side of condition satisfied by instances.
   leftSide(Instances)   - 
Method in class weka.classifiers.trees.j48. NBTreeNoSplit 
 Does nothing because no condition has to be satisfied.
   leftSide(Instances)   - 
Method in class weka.classifiers.trees.j48. NBTreeSplit 
 Prints left side of condition..
   leftSide(Instances)   - 
Method in class weka.classifiers.trees.j48. NoSplit 
 Does nothing because no condition has to be satisfied.
   leftSide(Instances)   - 
Method in class weka.classifiers.trees.lmt. ResidualSplit 
 Returns name of splitting attribute (left side of condition).
   legend()   - 
Method in class weka.classifiers.trees. ADTree 
 Returns the legend of the tree, describing how results are to be interpreted.
   legend()   - 
Method in class weka.classifiers.trees. LADTree 
 Returns the legend of the tree, describing how results are to be interpreted.
   LegendPanel   - Class in  weka.gui.visualize  This panel displays legends for a list of plots.   LegendPanel()   - 
Constructor for class weka.gui.visualize. LegendPanel 
 Constructor
   length   - 
Variable in class weka.core.neighboursearch.covertrees. Stack 
 The number of elements in the stack.
   leverageForRule(AprioriItemSet, AprioriItemSet, int, int)   - 
Method in class weka.associations. AprioriItemSet 
 Outputs the leverage for a rule.
   LFSMethods   - Class in  weka.attributeSelection      LFSMethods()   - 
Constructor for class weka.attributeSelection. LFSMethods 
 empty constructor

 methods are not static because of access to inner class Link2 and
 LinkedList2
   LFSMethods.Link2   - Class in  weka.attributeSelection  Class for a node in a linked list.   LFSMethods.Link2(Object[], double)   - 
Constructor for class weka.attributeSelection. LFSMethods.Link2 
  
   LFSMethods.LinkedList2   - Class in  weka.attributeSelection  Class for handling a linked list.   LFSMethods.LinkedList2(int)   - 
Constructor for class weka.attributeSelection. LFSMethods.LinkedList2 
  
   LibLINEAR   - Class in  weka.classifiers.functions  A wrapper class for the liblinear tools (the liblinear classes, typically the jar file, need to be in the classpath to use this classifier). 
 Rong-En Fan, Kai-Wei Chang, Cho-Jui Hsieh, Xiang-Rui Wang, Chih-Jen Lin (2008).   LibLINEAR()   - 
Constructor for class weka.classifiers.functions. LibLINEAR 
  
   LibSVM   - Class in  weka.classifiers.functions  A wrapper class for the libsvm tools (the libsvm classes, typically the jar file, need to be in the classpath to use this classifier). 
 LibSVM runs faster than SMO since it uses LibSVM to build the SVM classifier. 
 LibSVM allows users to experiment with One-class SVM, Regressing SVM, and nu-SVM supported by LibSVM tool.   LibSVM()   - 
Constructor for class weka.classifiers.functions. LibSVM 
  
   LibSVMLoader   - Class in  weka.core.converters  Reads a source that is in libsvm format. 
  
 For more information about libsvm see: 
  
 http://www.csie.ntu.edu.tw/~cjlin/libsvm/
  
     LibSVMLoader()   - 
Constructor for class weka.core.converters. LibSVMLoader 
  
   LibSVMSaver   - Class in  weka.core.converters  Writes to a destination that is in libsvm format. 
  
 For more information about libsvm see: 
  
 http://www.csie.ntu.edu.tw/~cjlin/libsvm/
  
  

  
 Valid options are:     LibSVMSaver()   - 
Constructor for class weka.core.converters. LibSVMSaver 
 Constructor
   LIFT_NAME   - 
Static variable in class weka.classifiers.evaluation. ThresholdCurve 
 attribute name: Lift
   liftForRule(AprioriItemSet, AprioriItemSet, int)   - 
Method in class weka.associations. AprioriItemSet 
 Outputs the lift for a rule.
   likelihoodThresholdTipText()   - 
Method in class weka.classifiers.meta. LogitBoost 
 Returns the tip text for this property
   LINE   - 
Static variable in class weka.gui.visualize. VisualizePanelEvent 
  
   LinearForwardSelection   - Class in  weka.attributeSelection  LinearForwardSelection: 
  
 Extension of BestFirst.   LinearForwardSelection()   - 
Constructor for class weka.attributeSelection. LinearForwardSelection 
 Constructor
   LinearNNSearch   - Class in  weka.core.neighboursearch  Class implementing the brute force search algorithm for nearest neighbour search.   LinearNNSearch()   - 
Constructor for class weka.core.neighboursearch. LinearNNSearch 
 Constructor.
   LinearNNSearch(Instances)   - 
Constructor for class weka.core.neighboursearch. LinearNNSearch 
 Constructor that uses the supplied set of 
 instances.
   LinearRegression   - Class in  weka.classifiers.functions  Class for using linear regression for prediction.   LinearRegression()   - 
Constructor for class weka.classifiers.functions. LinearRegression 
  
   LinearRegression   - Class in  weka.core.matrix  Class for performing (ridged) linear regression.   LinearRegression(Matrix, Matrix, double)   - 
Constructor for class weka.core.matrix. LinearRegression 
 Performs a (ridged) linear regression.
   LinearRegression(Matrix, Matrix, double[], double)   - 
Constructor for class weka.core.matrix. LinearRegression 
 Performs a weighted (ridged) linear regression.
   LinearUnit   - Class in  weka.classifiers.functions.neural  This can be used by the 
 neuralnode to perform all it's computations (as a Linear unit).   LinearUnit()   - 
Constructor for class weka.classifiers.functions.neural. LinearUnit 
  
   linkTypeTipText()   - 
Method in class weka.clusterers. HierarchicalClusterer 
  
   LINUX_BROWSERS   - 
Static variable in class weka.gui. BrowserHelper 
 Linux/Unix binaries to look for
   listOptions()   - 
Method in class weka.associations. Apriori 
 Returns an enumeration describing the available options.
   listOptions()   - 
Method in class weka.associations. CheckAssociator 
 Returns an enumeration describing the available options.
   listOptions()   - 
Method in class weka.associations. FilteredAssociator 
 Returns an enumeration describing the available options.
   listOptions()   - 
Method in class weka.associations. FPGrowth 
 Returns an enumeration describing the available options.
   listOptions()   - 
Method in class weka.associations. GeneralizedSequentialPatterns 
 Returns an enumeration of the available options.
   listOptions()   - 
Method in class weka.associations. PredictiveApriori 
 Returns an enumeration describing the available options.
   listOptions()   - 
Method in class weka.associations. SingleAssociatorEnhancer 
 Returns an enumeration describing the available options.
   listOptions()   - 
Method in class weka.associations. Tertius 
 Returns an enumeration describing the available options.
   listOptions()   - 
Method in class weka.attributeSelection. BestFirst 
 Returns an enumeration describing the available options.
   listOptions()   - 
Method in class weka.attributeSelection. CfsSubsetEval 
 Returns an enumeration describing the available options.
   listOptions()   - 
Method in class weka.attributeSelection. CheckAttributeSelection 
 Returns an enumeration describing the available options.
   listOptions()   - 
Method in class weka.attributeSelection. ChiSquaredAttributeEval 
 Returns an enumeration describing the available options
   listOptions()   - 
Method in class weka.attributeSelection. ClassifierSubsetEval 
 Returns an enumeration describing the available options.
   listOptions()   - 
Method in class weka.attributeSelection. CostSensitiveASEvaluation 
 Returns an enumeration describing the available options.
   listOptions()   - 
Method in class weka.attributeSelection. ExhaustiveSearch 
 Returns an enumeration describing the available options.
   listOptions()   - 
Method in class weka.attributeSelection. FilteredAttributeEval 
 Returns an enumeration describing the available options.
   listOptions()   - 
Method in class weka.attributeSelection. FilteredSubsetEval 
 Returns an enumeration describing the available options.
   listOptions()   - 
Method in class weka.attributeSelection. GainRatioAttributeEval 
 Returns an enumeration describing the available options.
   listOptions()   - 
Method in class weka.attributeSelection. GeneticSearch 
 Returns an enumeration describing the available options.
   listOptions()   - 
Method in class weka.attributeSelection. GreedyStepwise 
 Returns an enumeration describing the available options.
   listOptions()   - 
Method in class weka.attributeSelection. InfoGainAttributeEval 
 Returns an enumeration describing the available options.
   listOptions()   - 
Method in class weka.attributeSelection. LatentSemanticAnalysis 
 Returns an enumeration describing the available options.
   listOptions()   - 
Method in class weka.attributeSelection. LinearForwardSelection 
 Returns an enumeration describing the available options.
   listOptions()   - 
Method in class weka.attributeSelection. OneRAttributeEval 
 Returns an enumeration describing the available options.
   listOptions()   - 
Method in class weka.attributeSelection. PrincipalComponents 
 Returns an enumeration describing the available options.
   listOptions()   - 
Method in class weka.attributeSelection. RaceSearch 
 Returns an enumeration describing the available options.
   listOptions()   - 
Method in class weka.attributeSelection. RandomSearch 
 Returns an enumeration describing the available options.
   listOptions()   - 
Method in class weka.attributeSelection. Ranker 
 Returns an enumeration describing the available options.
   listOptions()   - 
Method in class weka.attributeSelection. RankSearch 
 Returns an enumeration describing the available options.
   listOptions()   - 
Method in class weka.attributeSelection. ReliefFAttributeEval 
 Returns an enumeration describing the available options.
   listOptions()   - 
Method in class weka.attributeSelection. ScatterSearchV1 
 Returns an enumeration describing the available options.
   listOptions()   - 
Method in class weka.attributeSelection. SubsetSizeForwardSelection 
 Returns an enumeration describing the available options.
   listOptions()   - 
Method in class weka.attributeSelection. SVMAttributeEval 
 Returns an enumeration describing all the available options
   listOptions()   - 
Method in class weka.attributeSelection. SymmetricalUncertAttributeEval 
 Returns an enumeration describing the available options.
   listOptions()   - 
Method in class weka.attributeSelection. WrapperSubsetEval 
 Returns an enumeration describing the available options.
   listOptions()   - 
Method in class weka.classifiers.bayes. AODE 
 Returns an enumeration describing the available options
   listOptions()   - 
Method in class weka.classifiers.bayes. AODEsr 
 Returns an enumeration describing the available options
   listOptions()   - 
Method in class weka.classifiers.bayes. BayesianLogisticRegression 
 Returns an enumeration describing the available options.
   listOptions()   - 
Method in class weka.classifiers.bayes. BayesNet 
 Returns an enumeration describing the available options
   listOptions()   - 
Method in class weka.classifiers.bayes. ComplementNaiveBayes 
 Returns an enumeration describing the available options.
   listOptions()   - 
Method in class weka.classifiers.bayes. DMNBtext 
 Returns an enumeration describing the available options.
   listOptions()   - 
Method in class weka.classifiers.bayes. NaiveBayes 
 Returns an enumeration describing the available options.
   listOptions()   - 
Method in class weka.classifiers.bayes.net. BayesNetGenerator 
 Returns an enumeration describing the available options
   listOptions()   - 
Method in class weka.classifiers.bayes.net.estimate. BayesNetEstimator 
 Returns an enumeration describing the available options
   listOptions()   - 
Method in class weka.classifiers.bayes.net.estimate. BMAEstimator 
 Returns an enumeration describing the available options
   listOptions()   - 
Method in class weka.classifiers.bayes.net.estimate. MultiNomialBMAEstimator 
 Returns an enumeration describing the available options
   listOptions()   - 
Method in class weka.classifiers.bayes.net.search.ci. ICSSearchAlgorithm 
 Returns an enumeration describing the available options.
   listOptions()   - 
Method in class weka.classifiers.bayes.net.search.fixed. FromFile 
 Returns an enumeration describing the available options.
   listOptions()   - 
Method in class weka.classifiers.bayes.net.search.global. GeneticSearch 
 Returns an enumeration describing the available options.
   listOptions()   - 
Method in class weka.classifiers.bayes.net.search.global. GlobalScoreSearchAlgorithm 
 Returns an enumeration describing the available options
   listOptions()   - 
Method in class weka.classifiers.bayes.net.search.global. HillClimber 
 Returns an enumeration describing the available options.
   listOptions()   - 
Method in class weka.classifiers.bayes.net.search.global. K2 
 Returns an enumeration describing the available options.
   listOptions()   - 
Method in class weka.classifiers.bayes.net.search.global. RepeatedHillClimber 
 Returns an enumeration describing the available options.
   listOptions()   - 
Method in class weka.classifiers.bayes.net.search.global. SimulatedAnnealing 
 Returns an enumeration describing the available options.
   listOptions()   - 
Method in class weka.classifiers.bayes.net.search.global. TabuSearch 
 Returns an enumeration describing the available options.
   listOptions()   - 
Method in class weka.classifiers.bayes.net.search.global. TAN 
 Returns an enumeration describing the available options.
   listOptions()   - 
Method in class weka.classifiers.bayes.net.search.local. GeneticSearch 
 Returns an enumeration describing the available options.
   listOptions()   - 
Method in class weka.classifiers.bayes.net.search.local. HillClimber 
 Returns an enumeration describing the available options.
   listOptions()   - 
Method in class weka.classifiers.bayes.net.search.local. K2 
 Returns an enumeration describing the available options.
   listOptions()   - 
Method in class weka.classifiers.bayes.net.search.local. LAGDHillClimber 
 Returns an enumeration describing the available options.
   listOptions()   - 
Method in class weka.classifiers.bayes.net.search.local. LocalScoreSearchAlgorithm 
 Returns an enumeration describing the available options
   listOptions()   - 
Method in class weka.classifiers.bayes.net.search.local. RepeatedHillClimber 
 Returns an enumeration describing the available options.
   listOptions()   - 
Method in class weka.classifiers.bayes.net.search.local. SimulatedAnnealing 
 Returns an enumeration describing the available options.
   listOptions()   - 
Method in class weka.classifiers.bayes.net.search.local. TabuSearch 
 Returns an enumeration describing the available options.
   listOptions()   - 
Method in class weka.classifiers.bayes.net.search.local. TAN 
 Returns an enumeration describing the available options.
   listOptions()   - 
Method in class weka.classifiers.bayes.net.search. SearchAlgorithm 
 Returns an enumeration describing the available options.
   listOptions()   - 
Method in class weka.classifiers.bayes. WAODE 
 Gets an enumeration describing the available options.
   listOptions()   - 
Method in class weka.classifiers. BVDecompose 
 Returns an enumeration describing the available options.
   listOptions()   - 
Method in class weka.classifiers. BVDecomposeSegCVSub 
 Returns an enumeration describing the available options.
   listOptions()   - 
Method in class weka.classifiers. CheckClassifier 
 Returns an enumeration describing the available options.
   listOptions()   - 
Method in class weka.classifiers. CheckSource 
 Returns an enumeration describing the available options.
   listOptions()   - 
Method in class weka.classifiers. Classifier 
 Returns an enumeration describing the available options.
   listOptions()   - 
Method in class weka.classifiers.functions. GaussianProcesses 
 Returns an enumeration describing the available options.
   listOptions()   - 
Method in class weka.classifiers.functions. LeastMedSq 
 Returns an enumeration of all the available options..
   listOptions()   - 
Method in class weka.classifiers.functions. LibLINEAR 
 Returns an enumeration describing the available options.
   listOptions()   - 
Method in class weka.classifiers.functions. LibSVM 
 Returns an enumeration describing the available options.
   listOptions()   - 
Method in class weka.classifiers.functions. LinearRegression 
 Returns an enumeration describing the available options.
   listOptions()   - 
Method in class weka.classifiers.functions. Logistic 
 Returns an enumeration describing the available options
   listOptions()   - 
Method in class weka.classifiers.functions. MultilayerPerceptron 
 Returns an enumeration describing the available options.
   listOptions()   - 
Method in class weka.classifiers.functions. PaceRegression 
 Returns an enumeration describing the available options.
   listOptions()   - 
Method in class weka.classifiers.functions. PLSClassifier 
 Gets an enumeration describing the available options.
   listOptions()   - 
Method in class weka.classifiers.functions. RBFNetwork 
 Returns an enumeration describing the available options
   listOptions()   - 
Method in class weka.classifiers.functions. SimpleLogistic 
 Returns an enumeration describing the available options.
   listOptions()   - 
Method in class weka.classifiers.functions. SMO 
 Returns an enumeration describing the available options.
   listOptions()   - 
Method in class weka.classifiers.functions. SMOreg 
 Returns an enumeration describing the available options.
   listOptions()   - 
Method in class weka.classifiers.functions. SPegasos 
 Returns an enumeration describing the available options.
   listOptions()   - 
Method in class weka.classifiers.functions.supportVector. CachedKernel 
 Returns an enumeration describing the available options.
   listOptions()   - 
Method in class weka.classifiers.functions.supportVector. CheckKernel 
 Returns an enumeration describing the available options.
   listOptions()   - 
Method in class weka.classifiers.functions.supportVector. Kernel 
 Returns an enumeration describing the available options.
   listOptions()   - 
Method in class weka.classifiers.functions.supportVector. PolyKernel 
 Returns an enumeration describing the available options.
   listOptions()   - 
Method in class weka.classifiers.functions.supportVector. PrecomputedKernelMatrixKernel 
 Returns an enumeration describing the available options.
   listOptions()   - 
Method in class weka.classifiers.functions.supportVector. Puk 
 Returns an enumeration describing the available options.
   listOptions()   - 
Method in class weka.classifiers.functions.supportVector. RBFKernel 
 Returns an enumeration describing the available options.
   listOptions()   - 
Method in class weka.classifiers.functions.supportVector. RegOptimizer 
 Gets an enumeration describing the available options.
   listOptions()   - 
Method in class weka.classifiers.functions.supportVector. RegSMO 
 Returns an enumeration describing the available options
   listOptions()   - 
Method in class weka.classifiers.functions.supportVector. RegSMOImproved 
 Returns an enumeration describing the available options
   listOptions()   - 
Method in class weka.classifiers.functions.supportVector. StringKernel 
 Returns an enumeration describing the available options.
   listOptions()   - 
Method in class weka.classifiers.functions. VotedPerceptron 
 Returns an enumeration describing the available options.
   listOptions()   - 
Method in class weka.classifiers.functions. Winnow 
 Returns an enumeration describing the available options
   listOptions()   - 
Method in class weka.classifiers. IteratedSingleClassifierEnhancer 
 Returns an enumeration describing the available options.
   listOptions()   - 
Method in class weka.classifiers.lazy. IBk 
 Returns an enumeration describing the available options.
   listOptions()   - 
Method in class weka.classifiers.lazy. KStar 
 Returns an enumeration describing the available options.
   listOptions()   - 
Method in class weka.classifiers.lazy. LWL 
 Returns an enumeration describing the available options.
   listOptions()   - 
Method in class weka.classifiers.meta. AdaBoostM1 
 Returns an enumeration describing the available options.
   listOptions()   - 
Method in class weka.classifiers.meta. AdditiveRegression 
 Returns an enumeration describing the available options.
   listOptions()   - 
Method in class weka.classifiers.meta. AttributeSelectedClassifier 
 Returns an enumeration describing the available options.
   listOptions()   - 
Method in class weka.classifiers.meta. Bagging 
 Returns an enumeration describing the available options.
   listOptions()   - 
Method in class weka.classifiers.meta. ClassificationViaClustering 
 Gets an enumeration describing the available options.
   listOptions()   - 
Method in class weka.classifiers.meta. CostSensitiveClassifier 
 Returns an enumeration describing the available options.
   listOptions()   - 
Method in class weka.classifiers.meta. CVParameterSelection 
 Returns an enumeration describing the available options.
   listOptions()   - 
Method in class weka.classifiers.meta. Dagging 
 Returns an enumeration describing the available options.
   listOptions()   - 
Method in class weka.classifiers.meta. Decorate 
 Returns an enumeration describing the available options
   listOptions()   - 
Method in class weka.classifiers.meta. FilteredClassifier 
 Returns an enumeration describing the available options.
   listOptions()   - 
Method in class weka.classifiers.meta. GridSearch 
 Gets an enumeration describing the available options.
   listOptions()   - 
Method in class weka.classifiers.meta. LogitBoost 
 Returns an enumeration describing the available options.
   listOptions()   - 
Method in class weka.classifiers.meta. MetaCost 
 Returns an enumeration describing the available options.
   listOptions()   - 
Method in class weka.classifiers.meta. MultiBoostAB 
 Returns an enumeration describing the available options
   listOptions()   - 
Method in class weka.classifiers.meta. MultiClassClassifier 
 Returns an enumeration describing the available options
   listOptions()   - 
Method in class weka.classifiers.meta. MultiScheme 
 Returns an enumeration describing the available options.
   listOptions()   - 
Method in class weka.classifiers.meta. OrdinalClassClassifier 
 Returns an enumeration describing the available options.
   listOptions()   - 
Method in class weka.classifiers.meta. RacedIncrementalLogitBoost 
 Returns an enumeration describing the available options
   listOptions()   - 
Method in class weka.classifiers.meta. RandomSubSpace 
 Returns an enumeration describing the available options.
   listOptions()   - 
Method in class weka.classifiers.meta. RegressionByDiscretization 
 Returns an enumeration describing the available options.
   listOptions()   - 
Method in class weka.classifiers.meta. RotationForest 
 Returns an enumeration describing the available options.
   listOptions()   - 
Method in class weka.classifiers.meta. Stacking 
 Returns an enumeration describing the available options.
   listOptions()   - 
Method in class weka.classifiers.meta. ThresholdSelector 
 Returns an enumeration describing the available options.
   listOptions()   - 
Method in class weka.classifiers.meta. Vote 
 Returns an enumeration describing the available options.
   listOptions()   - 
Method in class weka.classifiers.mi. CitationKNN 
 Returns an enumeration of all the available options..
   listOptions()   - 
Method in class weka.classifiers.mi. MDD 
 Returns an enumeration describing the available options
   listOptions()   - 
Method in class weka.classifiers.mi. MIBoost 
 Returns an enumeration describing the available options
   listOptions()   - 
Method in class weka.classifiers.mi. MIDD 
 Returns an enumeration describing the available options
   listOptions()   - 
Method in class weka.classifiers.mi. MIEMDD 
 Returns an enumeration describing the available options
   listOptions()   - 
Method in class weka.classifiers.mi. MILR 
 Returns an enumeration describing the available options
   listOptions()   - 
Method in class weka.classifiers.mi. MINND 
 Returns an enumeration describing the available options
   listOptions()   - 
Method in class weka.classifiers.mi. MIOptimalBall 
 Returns an enumeration describing the available options.
   listOptions()   - 
Method in class weka.classifiers.mi. MISMO 
 Returns an enumeration describing the available options.
   listOptions()   - 
Method in class weka.classifiers.mi. MISVM 
 Returns an enumeration describing the available options
   listOptions()   - 
Method in class weka.classifiers.mi. MIWrapper 
 Returns an enumeration describing the available options.
   listOptions()   - 
Method in class weka.classifiers.mi. SimpleMI 
 Returns an enumeration describing the available options.
   listOptions()   - 
Method in class weka.classifiers.misc. SerializedClassifier 
 Gets an enumeration describing the available options.
   listOptions()   - 
Method in class weka.classifiers.misc. VFI 
 Returns an enumeration describing the available options.
   listOptions()   - 
Method in class weka.classifiers. MultipleClassifiersCombiner 
 Returns an enumeration describing the available options
   listOptions()   - 
Method in class weka.classifiers. RandomizableClassifier 
 Returns an enumeration describing the available options.
   listOptions()   - 
Method in class weka.classifiers. RandomizableIteratedSingleClassifierEnhancer 
 Returns an enumeration describing the available options.
   listOptions()   - 
Method in class weka.classifiers. RandomizableMultipleClassifiersCombiner 
 Returns an enumeration describing the available options.
   listOptions()   - 
Method in class weka.classifiers. RandomizableSingleClassifierEnhancer 
 Returns an enumeration describing the available options.
   listOptions()   - 
Method in class weka.classifiers.rules. ConjunctiveRule 
 Returns an enumeration describing the available options
 Valid options are:
   listOptions()   - 
Method in class weka.classifiers.rules. DecisionTable 
 Returns an enumeration describing the available options.
   listOptions()   - 
Method in class weka.classifiers.rules. DTNB 
 Returns an enumeration describing the available options.
   listOptions()   - 
Method in class weka.classifiers.rules. JRip 
 Returns an enumeration describing the available options
 Valid options are:
   listOptions()   - 
Method in class weka.classifiers.rules. NNge 
 Returns an enumeration of all the available options..
   listOptions()   - 
Method in class weka.classifiers.rules. OneR 
 Returns an enumeration describing the available options..
   listOptions()   - 
Method in class weka.classifiers.rules. PART 
 Returns an enumeration describing the available options.
   listOptions()   - 
Method in class weka.classifiers.rules. Ridor 
 Returns an enumeration describing the available options
 Valid options are:
   listOptions()   - 
Method in class weka.classifiers. SingleClassifierEnhancer 
 Returns an enumeration describing the available options.
   listOptions()   - 
Method in class weka.classifiers.trees. ADTree 
 Returns an enumeration describing the available options..
   listOptions()   - 
Method in class weka.classifiers.trees. BFTree 
 Returns an enumeration describing the available options.
   listOptions()   - 
Method in class weka.classifiers.trees. FT 
 Returns an enumeration describing the available options.
   listOptions()   - 
Method in class weka.classifiers.trees. J48 
 Returns an enumeration describing the available options.
   listOptions()   - 
Method in class weka.classifiers.trees. J48graft 
 Returns an enumeration describing the available options.
   listOptions()   - 
Method in class weka.classifiers.trees. LADTree 
 Returns an enumeration describing the available options.
   listOptions()   - 
Method in class weka.classifiers.trees. LMT 
 Returns an enumeration describing the available options.
   listOptions()   - 
Method in class weka.classifiers.trees.m5. M5Base 
 Returns an enumeration describing the available options
   listOptions()   - 
Method in class weka.classifiers.trees. M5P 
 Returns an enumeration describing the available options
   listOptions()   - 
Method in class weka.classifiers.trees. RandomForest 
 Returns an enumeration describing the available options.
   listOptions()   - 
Method in class weka.classifiers.trees. RandomTree 
 Lists the command-line options for this classifier.
   listOptions()   - 
Method in class weka.classifiers.trees. REPTree 
 Lists the command-line options for this classifier.
   listOptions()   - 
Method in class weka.classifiers.trees. SimpleCart 
 Returns an enumeration describing the available options.
   listOptions()   - 
Method in class weka.clusterers. CheckClusterer 
 Returns an enumeration describing the available options.
   listOptions()   - 
Method in class weka.clusterers. CLOPE 
  
   listOptions()   - 
Method in class weka.clusterers. Cobweb 
 Returns an enumeration describing the available options.
   listOptions()   - 
Method in class weka.clusterers. DBScan 
 Returns an enumeration of all the available options..
   listOptions()   - 
Method in class weka.clusterers. EM 
 Returns an enumeration describing the available options.
   listOptions()   - 
Method in class weka.clusterers. FarthestFirst 
 Returns an enumeration describing the available options.
   listOptions()   - 
Method in class weka.clusterers. FilteredClusterer 
 Returns an enumeration describing the available options.
   listOptions()   - 
Method in class weka.clusterers. HierarchicalClusterer 
 Returns an enumeration describing the available options.
   listOptions()   - 
Method in class weka.clusterers. MakeDensityBasedClusterer 
 Returns an enumeration describing the available options..
   listOptions()   - 
Method in class weka.clusterers. OPTICS 
 Returns an enumeration of all the available options.
   listOptions()   - 
Method in class weka.clusterers. RandomizableClusterer 
 Returns an enumeration describing the available options.
   listOptions()   - 
Method in class weka.clusterers. RandomizableDensityBasedClusterer 
 Returns an enumeration describing the available options.
   listOptions()   - 
Method in class weka.clusterers. RandomizableSingleClustererEnhancer 
 Returns an enumeration describing the available options.
   listOptions()   - 
Method in class weka.clusterers. sIB 
 Returns an enumeration describing the available options.
   listOptions()   - 
Method in class weka.clusterers. SimpleKMeans 
 Returns an enumeration describing the available options.
   listOptions()   - 
Method in class weka.clusterers. SingleClustererEnhancer 
 Returns an enumeration describing the available options.
   listOptions()   - 
Method in class weka.clusterers. XMeans 
 Returns an enumeration describing the available options.
   listOptions()   - 
Method in class weka.core. Check 
 Returns an enumeration describing the available options.
   listOptions()   - 
Method in class weka.core. CheckGOE 
 Returns an enumeration describing the available options.
   listOptions()   - 
Method in class weka.core. CheckOptionHandler 
 Returns an enumeration describing the available options.
   listOptions()   - 
Method in class weka.core. CheckScheme 
 Returns an enumeration describing the available options.
   listOptions()   - 
Method in class weka.core.converters. AbstractFileSaver 
 Returns an enumeration describing the available options.
   listOptions()   - 
Method in class weka.core.converters. ArffSaver 
 Returns an enumeration describing the available options.
   listOptions()   - 
Method in class weka.core.converters. C45Saver 
 Returns an enumeration describing the available options.
   listOptions()   - 
Method in class weka.core.converters. CSVLoader 
 Returns an enumeration describing the available options.
   listOptions()   - 
Method in class weka.core.converters. DatabaseLoader 
 Lists the available options
   listOptions()   - 
Method in class weka.core.converters. DatabaseSaver 
 Lists the available options.
   listOptions()   - 
Method in class weka.core.converters. LibSVMSaver 
 Returns an enumeration describing the available options.
   listOptions()   - 
Method in class weka.core.converters. SVMLightSaver 
 Returns an enumeration describing the available options.
   listOptions()   - 
Method in class weka.core.converters. TextDirectoryLoader 
 Lists the available options
   listOptions()   - 
Method in class weka.core.converters. XRFFSaver 
 Returns an enumeration describing the available options.
   listOptions()   - 
Method in class weka.core. FindWithCapabilities 
 Returns an enumeration describing the available options.
   listOptions()   - 
Method in class weka.core. Javadoc 
 Returns an enumeration describing the available options.
   ListOptions   - Class in  weka.core  Lists the options of an OptionHandler   ListOptions()   - 
Constructor for class weka.core. ListOptions 
  
   listOptions()   - 
Method in class weka.core. ListOptions 
 Returns an enumeration describing the available options.
   listOptions()   - 
Method in class weka.core.neighboursearch. BallTree 
 Returns an enumeration describing the available options.
   listOptions()   - 
Method in class weka.core.neighboursearch.balltrees. BallSplitter 
 Returns an enumeration describing the available options.
   listOptions()   - 
Method in class weka.core.neighboursearch.balltrees. BallTreeConstructor 
 Returns an enumeration describing the available options.
   listOptions()   - 
Method in class weka.core.neighboursearch.balltrees. MedianDistanceFromArbitraryPoint 
 Returns an enumeration describing the available options.
   listOptions()   - 
Method in class weka.core.neighboursearch.balltrees. MedianOfWidestDimension 
 Returns an enumeration describing the available options.
   listOptions()   - 
Method in class weka.core.neighboursearch.balltrees. MiddleOutConstructor 
 Returns an enumeration describing the available options.
   listOptions()   - 
Method in class weka.core.neighboursearch.balltrees. TopDownConstructor 
 Returns an enumeration describing the available options.
   listOptions()   - 
Method in class weka.core.neighboursearch. CoverTree 
 Returns an enumeration describing the available options.
   listOptions()   - 
Method in class weka.core.neighboursearch. KDTree 
 Returns an enumeration describing the available options.
   listOptions()   - 
Method in class weka.core.neighboursearch.kdtrees. KDTreeNodeSplitter 
 Returns an enumeration describing the available options.
   listOptions()   - 
Method in class weka.core.neighboursearch. LinearNNSearch 
 Returns an enumeration describing the available options.
   listOptions()   - 
Method in class weka.core.neighboursearch. NearestNeighbourSearch 
 Returns an enumeration describing the available options.
   listOptions()   - 
Method in class weka.core. NormalizableDistance 
 Returns an enumeration describing the available options.
   listOptions()   - 
Method in interface weka.core. OptionHandler 
 Returns an enumeration of all the available options..
   listOptions()   - 
Method in class weka.core. OptionHandlerJavadoc 
 Returns an enumeration describing the available options.
   listOptions()   - 
Method in class weka.core.stemmers. SnowballStemmer 
 Returns an enumeration describing the available options.
   listOptions()   - 
Method in class weka.core. TechnicalInformationHandlerJavadoc 
 Returns an enumeration describing the available options.
   listOptions()   - 
Method in class weka.core. TestInstances 
 Returns an enumeration describing the available options.
   listOptions()   - 
Method in class weka.core.tokenizers. CharacterDelimitedTokenizer 
 Returns an enumeration of all the available options..
   listOptions()   - 
Method in class weka.core.tokenizers. NGramTokenizer 
 Returns an enumeration of all the available options..
   listOptions()   - 
Method in class weka.core.tokenizers. Tokenizer 
 Returns an enumeration of all the available options..
   listOptions()   - 
Method in class weka.datagenerators. ClassificationGenerator 
 Returns an enumeration describing the available options.
   listOptions()   - 
Method in class weka.datagenerators.classifiers.classification. Agrawal 
 Returns an enumeration describing the available options.
   listOptions()   - 
Method in class weka.datagenerators.classifiers.classification. BayesNet 
 Returns an enumeration describing the available options.
   listOptions()   - 
Method in class weka.datagenerators.classifiers.classification. LED24 
 Returns an enumeration describing the available options.
   listOptions()   - 
Method in class weka.datagenerators.classifiers.classification. RandomRBF 
 Returns an enumeration describing the available options.
   listOptions()   - 
Method in class weka.datagenerators.classifiers.classification. RDG1 
 Returns an enumeration describing the available options.
   listOptions()   - 
Method in class weka.datagenerators.classifiers.regression. Expression 
 Returns an enumeration describing the available options.
   listOptions()   - 
Method in class weka.datagenerators.classifiers.regression. MexicanHat 
 Returns an enumeration describing the available options.
   listOptions()   - 
Method in class weka.datagenerators. ClusterDefinition 
 Returns an enumeration describing the available options.
   listOptions()   - 
Method in class weka.datagenerators.clusterers. BIRCHCluster 
 Returns an enumeration describing the available options.
   listOptions()   - 
Method in class weka.datagenerators.clusterers. SubspaceCluster 
 Returns an enumeration describing the available options.
   listOptions()   - 
Method in class weka.datagenerators.clusterers. SubspaceClusterDefinition 
 Returns an enumeration describing the available options.
   listOptions()   - 
Method in class weka.datagenerators. ClusterGenerator 
 Returns an enumeration describing the available options.
   listOptions()   - 
Method in class weka.datagenerators. DataGenerator 
 Returns an enumeration describing the available options.
   listOptions()   - 
Method in class weka.datagenerators. RegressionGenerator 
 Returns an enumeration describing the available options.
   listOptions()   - 
Method in class weka.estimators. CheckEstimator 
 Returns an enumeration describing the available options.
   listOptions()   - 
Method in class weka.estimators. Estimator 
 Returns an enumeration describing the available options.
   listOptions()   - 
Method in class weka.experiment. AveragingResultProducer 
 Returns an enumeration describing the available options..
   listOptions()   - 
Method in class weka.experiment. ClassifierSplitEvaluator 
 Returns an enumeration describing the available options..
   listOptions()   - 
Method in class weka.experiment. CostSensitiveClassifierSplitEvaluator 
 Returns an enumeration describing the available options..
   listOptions()   - 
Method in class weka.experiment. CrossValidationResultProducer 
 Returns an enumeration describing the available options..
   listOptions()   - 
Method in class weka.experiment. CSVResultListener 
 Returns an enumeration describing the available options..
   listOptions()   - 
Method in class weka.experiment. DatabaseResultProducer 
 Returns an enumeration describing the available options..
   listOptions()   - 
Method in class weka.experiment. DensityBasedClustererSplitEvaluator 
 Returns an enumeration describing the available options.
   listOptions()   - 
Method in class weka.experiment. Experiment 
 Returns an enumeration describing the available options..
   listOptions()   - 
Method in class weka.experiment. InstanceQuery 
 Returns an enumeration describing the available options
   listOptions()   - 
Method in class weka.experiment. LearningRateResultProducer 
 Returns an enumeration describing the available options..
   listOptions()   - 
Method in class weka.experiment. PairedTTester 
 Lists options understood by this object.
   listOptions()   - 
Method in class weka.experiment. RandomSplitResultProducer 
 Returns an enumeration describing the available options..
   listOptions()   - 
Method in class weka.experiment. RegressionSplitEvaluator 
 Returns an enumeration describing the available options..
   listOptions()   - 
Method in class weka.filters. CheckSource 
 Returns an enumeration describing the available options.
   listOptions()   - 
Method in class weka.filters. MultiFilter 
 Returns an enumeration describing the available options.
   listOptions()   - 
Method in class weka.filters. SimpleFilter 
 Returns an enumeration describing the available options.
   listOptions()   - 
Method in class weka.filters.supervised.attribute. AddClassification 
 Returns an enumeration describing the available options.
   listOptions()   - 
Method in class weka.filters.supervised.attribute. AttributeSelection 
 Returns an enumeration describing the available options.
   listOptions()   - 
Method in class weka.filters.supervised.attribute. ClassOrder 
 Returns an enumeration describing the available options.
   listOptions()   - 
Method in class weka.filters.supervised.attribute. Discretize 
 Gets an enumeration describing the available options.
   listOptions()   - 
Method in class weka.filters.supervised.attribute. NominalToBinary 
 Returns an enumeration describing the available options.
   listOptions()   - 
Method in class weka.filters.supervised.attribute. PLSFilter 
 Gets an enumeration describing the available options.
   listOptions()   - 
Method in class weka.filters.supervised.instance. Resample 
 Returns an enumeration describing the available options.
   listOptions()   - 
Method in class weka.filters.supervised.instance. SMOTE 
 Returns an enumeration describing the available options.
   listOptions()   - 
Method in class weka.filters.supervised.instance. SpreadSubsample 
 Returns an enumeration describing the available options.
   listOptions()   - 
Method in class weka.filters.supervised.instance. StratifiedRemoveFolds 
 Gets an enumeration describing the available options..
   listOptions()   - 
Method in class weka.filters.unsupervised.attribute. AbstractTimeSeries 
 Returns an enumeration describing the available options.
   listOptions()   - 
Method in class weka.filters.unsupervised.attribute. Add 
 Returns an enumeration describing the available options.
   listOptions()   - 
Method in class weka.filters.unsupervised.attribute. AddCluster 
 Returns an enumeration describing the available options.
   listOptions()   - 
Method in class weka.filters.unsupervised.attribute. AddExpression 
 Returns an enumeration describing the available options.
   listOptions()   - 
Method in class weka.filters.unsupervised.attribute. AddID 
 Returns an enumeration describing the available options.
   listOptions()   - 
Method in class weka.filters.unsupervised.attribute. AddNoise 
 Returns an enumeration describing the available options
   listOptions()   - 
Method in class weka.filters.unsupervised.attribute. AddValues 
 Returns an enumeration describing the available options.
   listOptions()   - 
Method in class weka.filters.unsupervised.attribute. ChangeDateFormat 
 Returns an enumeration describing the available options
   listOptions()   - 
Method in class weka.filters.unsupervised.attribute. ClassAssigner 
 Returns an enumeration describing the available options.
   listOptions()   - 
Method in class weka.filters.unsupervised.attribute. ClusterMembership 
 Returns an enumeration describing the available options.
   listOptions()   - 
Method in class weka.filters.unsupervised.attribute. Copy 
 Returns an enumeration describing the available options.
   listOptions()   - 
Method in class weka.filters.unsupervised.attribute. Discretize 
 Gets an enumeration describing the available options.
   listOptions()   - 
Method in class weka.filters.unsupervised.attribute. FirstOrder 
 Returns an enumeration describing the available options.
   listOptions()   - 
Method in class weka.filters.unsupervised.attribute. InterquartileRange 
 Returns an enumeration describing the available options.
   listOptions()   - 
Method in class weka.filters.unsupervised.attribute. KernelFilter 
 Returns an enumeration describing the available options.
   listOptions()   - 
Method in class weka.filters.unsupervised.attribute. MakeIndicator 
 Returns an enumeration describing the available options.
   listOptions()   - 
Method in class weka.filters.unsupervised.attribute. MathExpression 
 Returns an enumeration describing the available options.
   listOptions()   - 
Method in class weka.filters.unsupervised.attribute. MergeTwoValues 
 Returns an enumeration describing the available options.
   listOptions()   - 
Method in class weka.filters.unsupervised.attribute. MultiInstanceToPropositional 
 Returns an enumeration describing the available options
   listOptions()   - 
Method in class weka.filters.unsupervised.attribute. NominalToBinary 
 Returns an enumeration describing the available options.
   listOptions()   - 
Method in class weka.filters.unsupervised.attribute. NominalToString 
 Returns an enumeration describing the available options.
   listOptions()   - 
Method in class weka.filters.unsupervised.attribute. Normalize 
 Returns an enumeration describing the available options.
   listOptions()   - 
Method in class weka.filters.unsupervised.attribute. NumericCleaner 
 Returns an enumeration describing the available options.
   listOptions()   - 
Method in class weka.filters.unsupervised.attribute. NumericToNominal 
 Gets an enumeration describing the available options.
   listOptions()   - 
Method in class weka.filters.unsupervised.attribute. NumericTransform 
 Returns an enumeration describing the available options.
   listOptions()   - 
Method in class weka.filters.unsupervised.attribute. PartitionedMultiFilter 
 Returns an enumeration describing the available options.
   listOptions()   - 
Method in class weka.filters.unsupervised.attribute. PKIDiscretize 
 Gets an enumeration describing the available options.
   listOptions()   - 
Method in class weka.filters.unsupervised.attribute. PotentialClassIgnorer 
 Returns an enumeration describing the available options.
   listOptions()   - 
Method in class weka.filters.unsupervised.attribute. PrincipalComponents 
 Returns an enumeration describing the available options.
   listOptions()   - 
Method in class weka.filters.unsupervised.attribute. PropositionalToMultiInstance 
 Returns an enumeration describing the available options
   listOptions()   - 
Method in class weka.filters.unsupervised.attribute. RandomProjection 
 Returns an enumeration describing the available options.
   listOptions()   - 
Method in class weka.filters.unsupervised.attribute. RandomSubset 
 Returns an enumeration describing the available options.
   listOptions()   - 
Method in class weka.filters.unsupervised.attribute. RELAGGS 
 Returns an enumeration describing the available options.
   listOptions()   - 
Method in class weka.filters.unsupervised.attribute. Remove 
 Returns an enumeration describing the available options.
   listOptions()   - 
Method in class weka.filters.unsupervised.attribute. RemoveType 
 Returns an enumeration describing the available options.
   listOptions()   - 
Method in class weka.filters.unsupervised.attribute. RemoveUseless 
 Returns an enumeration describing the available options.
   listOptions()   - 
Method in class weka.filters.unsupervised.attribute. Reorder 
 Returns an enumeration describing the available options.
   listOptions()   - 
Method in class weka.filters.unsupervised.attribute. StringToNominal 
 Returns an enumeration describing the available options.
   listOptions()   - 
Method in class weka.filters.unsupervised.attribute. StringToWordVector 
 Returns an enumeration describing the available options.
   listOptions()   - 
Method in class weka.filters.unsupervised.attribute. SwapValues 
 Returns an enumeration describing the available options.
   listOptions()   - 
Method in class weka.filters.unsupervised.attribute. Wavelet 
 Gets an enumeration describing the available options.
   listOptions()   - 
Method in class weka.filters.unsupervised.instance. Normalize 
 Returns an enumeration describing the available options.
   listOptions()   - 
Method in class weka.filters.unsupervised.instance. Randomize 
 Returns an enumeration describing the available options.
   listOptions()   - 
Method in class weka.filters.unsupervised.instance. RemoveFolds 
 Gets an enumeration describing the available options..
   listOptions()   - 
Method in class weka.filters.unsupervised.instance. RemoveFrequentValues 
 Returns an enumeration describing the available options.
   listOptions()   - 
Method in class weka.filters.unsupervised.instance. RemoveMisclassified 
 Returns an enumeration describing the available options.
   listOptions()   - 
Method in class weka.filters.unsupervised.instance. RemovePercentage 
 Gets an enumeration describing the available options..
   listOptions()   - 
Method in class weka.filters.unsupervised.instance. RemoveRange 
 Returns an enumeration describing the available options.
   listOptions()   - 
Method in class weka.filters.unsupervised.instance. RemoveWithValues 
 Returns an enumeration describing the available options.
   listOptions()   - 
Method in class weka.filters.unsupervised.instance. Resample 
 Returns an enumeration describing the available options.
   listOptions()   - 
Method in class weka.filters.unsupervised.instance. ReservoirSample 
 Returns an enumeration describing the available options.
   listOptions()   - 
Method in class weka.filters.unsupervised.instance. SubsetByExpression 
 Returns an enumeration describing the available options.
   listOptions()   - 
Method in class weka.gui. Main 
 Gets an enumeration describing the available options.
   ListSelectorDialog   - Class in  weka.gui  A dialog to present the user with a list of items, that the user can
 make a selection from, or cancel the selection.   ListSelectorDialog(Frame, JList)   - 
Constructor for class weka.gui. ListSelectorDialog 
 Create the list selection dialog.
   listStemmers()   - 
Static method in class weka.core.stemmers. SnowballStemmer 
 returns an enumeration over all currently stored stemmer names.
   Literal   - Class in  weka.associations.tertius      Literal(Predicate, int, int)   - 
Constructor for class weka.associations.tertius. Literal 
  
   LiteralSet   - Class in  weka.associations.tertius  Class representing a set of literals, being either the body or the head
 of a rule.   LiteralSet()   - 
Constructor for class weka.associations.tertius. LiteralSet 
 Constructor for a set that does not store its counter-instances.
   LiteralSet(Instances)   - 
Constructor for class weka.associations.tertius. LiteralSet 
 Constructor initializing the set of counter-instances to all the instances.
   LMT   - Class in  weka.classifiers.trees  Classifier for building 'logistic model trees', which are classification trees with logistic regression functions at the leaves.   LMT()   - 
Constructor for class weka.classifiers.trees. LMT 
 Creates an instance of LMT with standard options
   LMTNode   - Class in  weka.classifiers.trees.lmt  Class for logistic model tree structure.   LMTNode(ModelSelection, int, boolean, boolean, int, double, boolean)   - 
Constructor for class weka.classifiers.trees.lmt. LMTNode 
 Constructor for logistic model tree node.
   lnFactorial(int)   - 
Method in class weka.classifiers.bayes. NaiveBayesMultinomial 
 Fast computation of ln(n!) for non-negative ints

 negative ints are passed on to the general gamma-function
 based version in weka.core.SpecialFunctions

 if the current n value is higher than any previous one,
 the cache is extended and filled to cover it

 the common case is reduced to a simple array lookup
   lnFactorial(double)   - 
Static method in class weka.core. SpecialFunctions 
 Returns natural logarithm of factorial using gamma function.
   lnGamma(double)   - 
Static method in class weka.core. Statistics 
 Returns natural logarithm of gamma function.
   LNormTipText()   - 
Method in class weka.filters.unsupervised.instance. Normalize 
 Returns the tip text for this property
   lnsrch(double[], double[], double[], double, boolean[], double[][], Optimization.DynamicIntArray)   - 
Method in class weka.core. Optimization 
 Find a new point x in the direction p from a point xold at which the
 value of the function has decreased sufficiently, the positive 
 definiteness of B matrix (approximation of the inverse of the Hessian)
 is preserved and no bound constraints are violated.
   load(InputStream)   - 
Method in class weka.core. ProtectedProperties 
 Overrides a method to prevent the properties from being modified.
   load(String)   - 
Method in class weka.gui.beans. FlowRunner 
 Load a serialized KnowledgeFlow (either binary or xml)
   loadBinary(String)   - 
Method in class weka.gui.beans. FlowRunner 
 Load a binary serialized KnowledgeFlow
   Loader   - Interface in  weka.core.converters  Interface to something that can load Instances from an input source in some
 format.   Loader   - Class in  weka.gui.beans  Loads data sets using weka.core.converter classes   Loader()   - 
Constructor for class weka.gui.beans. Loader 
  
   Loader   - Class in  weka.gui  This class is for loading resources from a JAR archive.   Loader(String)   - 
Constructor for class weka.gui. Loader 
 initializes the object
   LOADER_DIALOG   - 
Static variable in class weka.gui. ConverterFileChooser 
 the loader dialog
   LoaderBeanInfo   - Class in  weka.gui.beans  Bean info class for the loader bean   LoaderBeanInfo()   - 
Constructor for class weka.gui.beans. LoaderBeanInfo 
  
   LoaderCustomizer   - Class in  weka.gui.beans  GUI Customizer for the loader bean   LoaderCustomizer()   - 
Constructor for class weka.gui.beans. LoaderCustomizer 
  
   loadFile(String)   - 
Method in class weka.gui.arffviewer. ArffViewerMainPanel 
 loads the specified file
   loadFile()   - 
Method in class weka.gui.arffviewer. ArffViewerMainPanel 
 loads the specified file into the table
   loadFromFile(String)   - 
Static method in class weka.core. Debug 
 deserializes the content of the file and returns it, null if an error
 occurred.
   loadIcons(String, String)   - 
Method in class weka.gui.beans. BeanVisual 
 Loads static and animated versions of a beans icons.
   loadInitialLayout(String)   - 
Method in class weka.gui.beans. KnowledgeFlowApp 
 Loads the specified file at input

 Added by Zerbetto
   loadModel()   - 
Method in class weka.gui.beans. Classifier 
  
   loadModel()   - 
Method in class weka.gui.beans. Clusterer 
  
   loadProperties()   - 
Static method in class weka.gui.beans. KnowledgeFlowApp 
 Loads KnowledgeFlow properties and any plugins (adds jars to
 the classpath)
   loadXML(String)   - 
Method in class weka.gui.beans. FlowRunner 
 Load an XML serialized KnowledgeFlow
   localDistributionForInstance(Instance, LBR.Indexes)   - 
Method in class weka.classifiers.lazy. LBR 
 Calculates the class membership probabilities.
   locallyPredictiveTipText()   - 
Method in class weka.attributeSelection. CfsSubsetEval 
 Returns the tip text for this property
   localNaiveBayes(LBR.Indexes)   - 
Method in class weka.classifiers.lazy. LBR 
 Class for building and using a simple Naive Bayes classifier.
   LocalScoreSearchAlgorithm   - Class in  weka.classifiers.bayes.net.search.local  The ScoreBasedSearchAlgorithm class supports Bayes net structure search algorithms that are based on maximizing scores (as opposed to for example conditional independence based search algorithms).   LocalScoreSearchAlgorithm()   - 
Constructor for class weka.classifiers.bayes.net.search.local. LocalScoreSearchAlgorithm 
 default constructor
   LocalScoreSearchAlgorithm(BayesNet, Instances)   - 
Constructor for class weka.classifiers.bayes.net.search.local. LocalScoreSearchAlgorithm 
 constructor
   locateIndex(int)   - 
Method in class weka.core. SparseInstance 
 Locates the greatest index that is not greater than the
 given index.
   log(String)   - 
Method in class weka.core. Debug 
 prints the given message with level INFO
   log(Level, String)   - 
Method in class weka.core. Debug 
 prints the given message with the specified level and an empty sourceclass
   log(Level, String, String)   - 
Method in class weka.core. Debug 
 prints the given message with the specified level
   log(Level, String, String, String)   - 
Method in class weka.core. Debug 
 prints the given message with the specified level
   log(Level, String)   - 
Method in class weka.core. Debug.Log 
 logs the given message
   log(Level, String, String)   - 
Method in class weka.core. Debug.Log 
 prints the given message with the specified level
   log(Level, String, String, String)   - 
Method in class weka.core. Debug.Log 
 prints the given message with the specified level
   log(String)   - 
Method in class weka.core. Debug.SimpleLog 
 logs the given message to the file
   log(Logger.Level, String)   - 
Static method in class weka.core.logging. Logger 
 Logs the given message under the given level.
   log(Logger.Level, Throwable)   - 
Static method in class weka.core.logging. Logger 
 Logs the given message under the given level.
   LOG   - 
Static variable in interface weka.core.mathematicalexpression. sym 
  
   LOG   - 
Static variable in interface weka.filters.unsupervised.instance.subsetbyexpression. sym 
  
   LOG2   - 
Static variable in interface weka.classifiers.lazy.kstar. KStarConstants 
  
   log2   - 
Static variable in class weka.core. Utils 
 The natural logarithm of 2.
   log2(double)   - 
Static method in class weka.core. Utils 
 Returns the logarithm of a for base 2.
   log2Binomial(double, double)   - 
Static method in class weka.core. SpecialFunctions 
 Returns base 2 logarithm of binomial coefficient using gamma function.
   log2Multinomial(double, double[])   - 
Static method in class weka.core. SpecialFunctions 
 Returns base 2 logarithm of multinomial using gamma function.
   log2MultipleHypergeometric(double[][])   - 
Static method in class weka.core. ContingencyTables 
 Returns negative base 2 logarithm of multiple hypergeometric
 probability for a contingency table.
   logbinomialCoefficient(int, int)   - 
Static method in class weka.associations. PriorEstimation 
 Method that calculates the base 2 logarithm of a binomial coefficient
   logDensityForInstance(Instance)   - 
Method in class weka.clusterers. AbstractDensityBasedClusterer 
 Computes the density for a given instance.
   logDensityForInstance(Instance)   - 
Method in interface weka.clusterers. DensityBasedClusterer 
 Computes the density for a given instance.
   logDensityPerClusterForInstance(Instance)   - 
Method in class weka.clusterers. AbstractDensityBasedClusterer 
 Computes the log of the conditional density (per cluster) for a given instance.
   logDensityPerClusterForInstance(Instance)   - 
Method in interface weka.clusterers. DensityBasedClusterer 
 Computes the log of the conditional density (per cluster) for a given instance.
   logDensityPerClusterForInstance(Instance)   - 
Method in class weka.clusterers. EM 
 Computes the log of the conditional density (per cluster) for a given instance.
   logDensityPerClusterForInstance(Instance)   - 
Method in class weka.clusterers. MakeDensityBasedClusterer 
 Computes the log of the conditional density (per cluster) for a given instance.
   logFileTipText()   - 
Method in class weka.classifiers.meta. GridSearch 
 Returns the tip text for this property
   logFunc(double)   - 
Method in class weka.classifiers.trees.j48. EntropyBasedSplitCrit 
 Help method for computing entropy.
   Logger   - Class in  weka.core.logging  Abstract superclass for all loggers.   Logger()   - 
Constructor for class weka.core.logging. Logger 
 Initializes the logger.
   Logger   - Interface in  weka.gui  Interface for objects that display log (permanent historical) and
 status (transient) messages.   Logger.Level   - Enum in  weka.core.logging  The logging level.   Logistic   - Class in  weka.classifiers.functions  Class for building and using a multinomial logistic regression model with a ridge estimator. 
  
 There are some modifications, however, compared to the paper of leCessie and van Houwelingen(1992):  
  
 If there are k classes for n instances with m attributes, the parameter matrix B to be calculated will be an m*(k-1) matrix. 
  
 The probability for class j with the exception of the last class is 
  
 Pj(Xi) = exp(XiBj)/((sum[j=1..(k-1)]exp(Xi*Bj))+1)  
  
 The last class has probability 
  
 1-(sum[j=1..(k-1)]Pj(Xi))  
        = 1/((sum[j=1..(k-1)]exp(Xi*Bj))+1) 
  
 The (negative) multinomial log-likelihood is thus:  
  
 L = -sum[i=1..n]{ 
        sum[j=1..(k-1)](Yij * ln(Pj(Xi))) 
        +(1 - (sum[j=1..(k-1)]Yij))  
        * ln(1 - sum[j=1..(k-1)]Pj(Xi)) 
        } + ridge * (B^2) 
  
 In order to find the matrix B for which L is minimised, a Quasi-Newton Method is used to search for the optimized values of the m*(k-1) variables.   Logistic()   - 
Constructor for class weka.classifiers.functions. Logistic 
  
   LogisticBase   - Class in  weka.classifiers.trees.lmt  Base/helper class for building logistic regression models with the LogitBoost algorithm.   LogisticBase()   - 
Constructor for class weka.classifiers.trees.lmt. LogisticBase 
 Constructor that creates LogisticBase object with standard options.
   LogisticBase(int, boolean, boolean)   - 
Constructor for class weka.classifiers.trees.lmt. LogisticBase 
 Constructor to create LogisticBase object.
   logisticLinkFunction(double)   - 
Static method in class weka.classifiers.bayes. BayesianLogisticRegression 
 This method computes the values for the logistic link function.
   LogitBoost   - Class in  weka.classifiers.meta  Class for performing additive logistic regression.   LogitBoost()   - 
Constructor for class weka.classifiers.meta. LogitBoost 
 Constructor.
   logJointDensitiesForInstance(Instance)   - 
Method in class weka.clusterers. AbstractDensityBasedClusterer 
 Returns the logs of the joint densities for a given instance.
   logJointDensitiesForInstance(Instance)   - 
Method in interface weka.clusterers. DensityBasedClusterer 
 Returns the logs of the joint densities for a given instance.
   LogLikelihood   - 
Static variable in class weka.classifiers.bayes. BayesianLogisticRegression 
 Log-likelihood values to be used to choose the best hyperparameter.
   logMessage(String)   - 
Method in class weka.gui.beans. FlowRunner.SimpleLogger 
  
   logMessage(String)   - 
Method in class weka.gui.beans. LogPanel 
 Sends the supplied message to the log area.
   logMessage(String)   - 
Method in interface weka.gui. Logger 
 Sends the supplied message to the log area.
   logMessage(String)   - 
Method in class weka.gui. LogPanel 
 Sends the supplied message to the log area.
   logMessage(String)   - 
Method in class weka.gui. SysErrLog 
 Sends the supplied message to the log area.
   LogPanel   - Class in  weka.gui.beans  Class for displaying a status area (made up of a variable number of
 lines) and a log area.   LogPanel()   - 
Constructor for class weka.gui.beans. LogPanel 
  
   LogPanel   - Class in  weka.gui  This panel allows log and status messages to be posted.   LogPanel()   - 
Constructor for class weka.gui. LogPanel 
 Creates the log panel with no task monitor and
 the log always visible.
   LogPanel(WekaTaskMonitor)   - 
Constructor for class weka.gui. LogPanel 
 Creates the log panel with a task monitor,
 where the log is hidden.
   LogPanel(WekaTaskMonitor, boolean)   - 
Constructor for class weka.gui. LogPanel 
 Creates the log panel, possibly with task monitor,
 where the log is optionally hidden.
   LogPanel(WekaTaskMonitor, boolean, boolean, boolean)   - 
Constructor for class weka.gui. LogPanel 
 Creates the log panel, possibly with task monitor,
 where the either the log is optionally hidden or the status
 (having both hidden is not allowed).
   logPSI   - 
Static variable in class weka.core.matrix. Maths 
 The constant - log( sqrt(2 pi) )
   logs2probs(double[])   - 
Static method in class weka.core. Utils 
 Converts an array containing the natural logarithms of
 probabilities stored in a vector back into probabilities.
   logScore(int, int)   - 
Method in class weka.classifiers.bayes.net.estimate. DiscreteEstimatorBayes 
 Gets the log score contribution of this distribution
   logScore(int)   - 
Method in class weka.classifiers.bayes.net.search.local. LocalScoreSearchAlgorithm 
 logScore returns the log of the quality of a network
 (e.g.
   logScore(int, int)   - 
Method in interface weka.classifiers.bayes.net.search.local. Scoreable 
 Returns log-score
   logSystemInfo()   - 
Method in class weka.core. Debug.Log 
 a convenience method for dumping the current system info in the 
 log file
   logSystemInfo()   - 
Method in class weka.core. Debug.SimpleLog 
 a convenience method for dumping the current system info in the 
 log file
   LogWindow   - Class in  weka.gui  Frame that shows the output from stdout and stderr.   LogWindow()   - 
Constructor for class weka.gui. LogWindow 
 creates the frame
   LogWriter   - Interface in  weka.gui.beans  Interface to be implemented by classes that should be able to write their
 own output to the Weka logger.   LONG   - 
Static variable in class weka.experiment. DatabaseUtils 
 Type mapping for LONG used for reading experiment results.
   LookAndFeel   - Class in  weka.gui  A little helper class for setting the Look and Feel of the user interface.   LookAndFeel()   - 
Constructor for class weka.gui. LookAndFeel 
  
   lookupCacheSizeTipText()   - 
Method in class weka.attributeSelection. BestFirst 
 Returns the tip text for this property
   lookupCacheSizeTipText()   - 
Method in class weka.attributeSelection. LinearForwardSelection 
 Returns the tip text for this property
   lookupCacheSizeTipText()   - 
Method in class weka.attributeSelection. SubsetSizeForwardSelection 
 Returns the tip text for this property
   LOSS_STRING   - 
Variable in class weka.experiment. ResultMatrix 
 loss string
   lossFunctionTipText()   - 
Method in class weka.classifiers.functions. SPegasos 
 Returns the tip text for this property
   lossTipText()   - 
Method in class weka.classifiers.functions. LibSVM 
 Returns the tip text for this property
   LovinsStemmer   - Class in  weka.core.stemmers  A stemmer based on the Lovins stemmer, described here: 
  
 Julie Beth Lovins (1968).   LovinsStemmer()   - 
Constructor for class weka.core.stemmers. LovinsStemmer 
  
   lowerBoundMinSupportTipText()   - 
Method in class weka.associations. Apriori 
 Returns the tip text for this property
   lowerBoundMinSupportTipText()   - 
Method in class weka.associations. FPGrowth 
 Returns the tip text for this property
   lowerCaseTokensTipText()   - 
Method in class weka.filters.unsupervised.attribute. StringToWordVector 
 Returns the tip text for this property.
   lowerNumericBoundIsOpen()   - 
Method in class weka.core. Attribute 
 Returns whether the lower numeric bound of the attribute is open.
   lowerSizeTipText()   - 
Method in class weka.experiment. LearningRateResultProducer 
 Returns the tip text for this property
   LPAREN   - 
Static variable in interface weka.core.mathematicalexpression. sym 
  
   LPAREN   - 
Static variable in interface weka.filters.unsupervised.instance.subsetbyexpression. sym 
  
   lsqr(PaceMatrix, IntVector, int)   - 
Method in class weka.classifiers.functions.pace. PaceMatrix 
 QR transformation for a least squares problem 
            A x = b 
 implicitly both A and b are transformed.
   lsqrSelection(PaceMatrix, IntVector, int)   - 
Method in class weka.classifiers.functions.pace. PaceMatrix 
 QR transformation for a least squares problem  
            A x = b  
 implicitly both A and b are transformed.
   LT   - 
Static variable in interface weka.core.mathematicalexpression. sym 
  
   LT   - 
Static variable in interface weka.filters.unsupervised.instance.subsetbyexpression. sym 
  
   lu()   - 
Method in class weka.core.matrix. Matrix 
 LU Decomposition
   LUDecomposition   - Class in  weka.core.matrix  LU Decomposition.   LUDecomposition()   - 
Method in class weka.core. Matrix 
  Deprecated.  Performs a LUDecomposition on the matrix.
   LUDecomposition(Matrix)   - 
Constructor for class weka.core.matrix. LUDecomposition 
 LU Decomposition
   LWL   - Class in  weka.classifiers.lazy  Locally weighted learning.   LWL()   - 
Constructor for class weka.classifiers.lazy. LWL 
 Constructor.
 
 
    
 M  
 
   M5Base   - Class in  weka.classifiers.trees.m5  M5Base.   M5Base()   - 
Constructor for class weka.classifiers.trees.m5. M5Base 
 Constructor
   M5P   - Class in  weka.classifiers.trees  M5Base.   M5P()   - 
Constructor for class weka.classifiers.trees. M5P 
 Creates a new  M5P  instance.
   M5Rules   - Class in  weka.classifiers.rules  Generates a decision list for regression problems using separate-and-conquer.   M5Rules()   - 
Constructor for class weka.classifiers.rules. M5Rules 
 Constructor
   m_ADNodes   - 
Variable in class weka.classifiers.bayes.net. VaryNode 
 list of ADNode children
   m_alpha   - 
Variable in class weka.classifiers.functions.supportVector. RegOptimizer 
 alpha and alpha* arrays containing weights for solving dual problem
   m_alpha   - 
Variable in class weka.classifiers.trees.lmt. LMTNode 
 Alpha-value (for pruning) at the node
   m_alphaStar   - 
Variable in class weka.classifiers.functions.supportVector. RegOptimizer 
  
   m_alwaysDisplayPointsOfThisSize   - 
Variable in class weka.gui.visualize. PlotData2D 
 If the shape size of a point equals this size then always plot
 it (i.e.
   m_AttIndexes   - 
Variable in class weka.classifiers.lazy. LBR.Indexes 
 the array attribute indexes
   M_AVERAGE   - 
Static variable in interface weka.classifiers.lazy.kstar. KStarConstants 
  
   m_children   - 
Variable in class weka.classifiers.bayes.net. MarginCalculator.JunctionTreeNode 
  
   m_ClassIndex   - 
Variable in class weka.classifiers.lazy. LBR.Indexes 
 the Class Index for the data set
   m_col   - 
Variable in class weka.gui.treevisualizer. NamedColor 
 The actual color object
   m_cols   - 
Variable in class weka.gui.treevisualizer. Colors 
 The array with all the colors input
   m_CoordCount   - 
Variable in class weka.core.neighboursearch. PerformanceStats 
 The number of coordinates looked at for
 the current/last query.
   m_CurrDebugFlag   - 
Variable in class weka.clusterers. XMeans 
 Flag: I'm debugging.
   m_customColour   - 
Variable in class weka.gui.visualize. PlotData2D 
  
   m_defaultExpression   - 
Static variable in class weka.filters.unsupervised.attribute. MathExpression 
 The default modification expression
   M_DELETE   - 
Static variable in interface weka.classifiers.lazy.kstar. KStarConstants 
 Missing value handling mode
   m_displayAllPoints   - 
Variable in class weka.gui.visualize. PlotData2D 
 Display all points (ie.
   m_Distributions   - 
Variable in class weka.classifiers.bayes. BayesNet 
 The attribute estimators containing CPTs.
   m_End   - 
Variable in class weka.core.neighboursearch.balltrees. BallNode 
 The end index of the portion of the master index array, 
 which stores indices of the instances/points the node 
 contains.
   m_End   - 
Variable in class weka.core.neighboursearch.kdtrees. KDTreeNode 
 The end index of the portion of the master index array, 
 which stores indices of the instances/points the node 
 contains.
   m_experimentFinished   - 
Variable in class weka.experiment. RemoteExperimentEvent 
 True if a remote experiment has finished
   m_Filter   - 
Variable in class weka.classifiers.bayes. BayesianLogisticRegression 
 Filter interface used to point to weka.filters.unsupervised.attribute.Normalize object
   m_indexVal   - 
Variable in class weka.gui.visualize. AttributePanelEvent 
 The index for the new attribute
   m_iNode   - 
Variable in class weka.classifiers.bayes.net. VaryNode 
 index of the node varied
   m_Instances   - 
Variable in class weka.classifiers.bayes. BayesNet 
 The dataset header for the purposes of printing out a semi-intelligible
 model
   m_Instances   - 
Variable in class weka.classifiers.bayes.net. ADNode 
 list of Instance children (either m_Instances or m_VaryNodes is instantiated)
   m_InstIndexes   - 
Variable in class weka.classifiers.lazy. LBR.Indexes 
 the array instance indexes
   m_Left   - 
Variable in class weka.core.neighboursearch.balltrees. BallNode 
 The left child of the node.
   m_Left   - 
Variable in class weka.core.neighboursearch.kdtrees. KDTreeNode 
 left subtree; contains instances with smaller or equal to split value.
   m_logMessage   - 
Variable in class weka.experiment. RemoteExperimentEvent 
 A log type message
   m_MaxC   - 
Variable in class weka.core.neighboursearch. PerformanceStats 
 The min and max coordinates(attributes) looked
 at per query.
   M_MAXDIFF   - 
Static variable in interface weka.classifiers.lazy.kstar. KStarConstants 
  
   m_MaxP   - 
Variable in class weka.core.neighboursearch. PerformanceStats 
 The min and max data points looked for a query by 
 the NNS algorithm.
   m_messageString   - 
Variable in class weka.experiment. RemoteExperimentEvent 
 The message
   m_MinC   - 
Variable in class weka.core.neighboursearch. PerformanceStats 
 The min and max coordinates(attributes) looked
 at per query.
   m_MinP   - 
Variable in class weka.core.neighboursearch. PerformanceStats 
 The min and max data points looked for a query by 
 the NNS algorithm.
   m_name   - 
Variable in class weka.gui.treevisualizer. NamedColor 
 The name of the color
   m_nCount   - 
Variable in class weka.classifiers.bayes.net. ADNode 
 count
   m_nMCV   - 
Variable in class weka.classifiers.bayes.net. VaryNode 
 most common value
   m_nNodes   - 
Variable in class weka.classifiers.bayes.net. MarginCalculator.JunctionTreeNode 
 nodes of the Bayes net in this junction node
   m_NodeNumber   - 
Variable in class weka.core.neighboursearch.balltrees. BallNode 
 The node number/id.
   m_NodeNumber   - 
Variable in class weka.core.neighboursearch.kdtrees. KDTreeNode 
 node number (only for debug).
   m_NodeRanges   - 
Variable in class weka.core.neighboursearch.kdtrees. KDTreeNode 
 lowest and highest value and width (= high - low) for each
 dimension.
   m_NodesRectBounds   - 
Variable in class weka.core.neighboursearch.kdtrees. KDTreeNode 
 The lo and high bounds of the hyper rectangle described by the
 node.
   M_NORMAL   - 
Static variable in interface weka.classifiers.lazy.kstar. KStarConstants 
  
   m_nStartNode   - 
Variable in class weka.classifiers.bayes.net. ADNode 
 first node in VaryNode array
   m_NumAttsSet   - 
Variable in class weka.classifiers.lazy. LBR.Indexes 
 the number of attributes "in use"  or set to a the original value (true or false)
   m_numIncorrectModel   - 
Variable in class weka.classifiers.trees.lmt. LMTNode 
 Weighted number of training examples currently misclassified by the logistic model at the node
   m_numIncorrectTree   - 
Variable in class weka.classifiers.trees.lmt. LMTNode 
 Weighted number of training examples currently misclassified by the subtree rooted at the node
   m_NumInstances   - 
Variable in class weka.core.neighboursearch.balltrees. BallNode 
 The number of instances/points in the node.
   m_NumInstsSet   - 
Variable in class weka.classifiers.lazy. LBR.Indexes 
 the number of instances "in use"  or set to a the original value (true or false)
   m_numParameters   - 
Variable in class weka.classifiers.trees.m5. RuleNode 
 the number of paramters in the chosen model for this node---either
 the subtree model or the linear model.
   m_NumSeqAttsSet   - 
Variable in class weka.classifiers.lazy. LBR.Indexes 
 the number of sequential attributes "in use"  or set to a the original value (true or false)
   m_NumSeqInstsSet   - 
Variable in class weka.classifiers.lazy. LBR.Indexes 
 the number of sequential instances "in use"  or set to a the original value (true or false)
   m_OutputFormat   - 
Variable in class weka.core. Debug.Clock 
 the format of the output
   m_outputTypes   - 
Variable in class weka.core. Debug.DBO 
 range of outputtyp
   m_PointCount   - 
Variable in class weka.core.neighboursearch. PerformanceStats 
 The number of data points looked at
 for the current/last query.
   m_Right   - 
Variable in class weka.core.neighboursearch.balltrees. BallNode 
 The right child of the node.
   m_Right   - 
Variable in class weka.core.neighboursearch.kdtrees. KDTreeNode 
 right subtree; contains instances with larger than split value.
   m_root   - 
Variable in class weka.classifiers.bayes.net. MarginCalculator 
  
   m_SequentialAttIndexes   - 
Variable in class weka.classifiers.lazy. LBR.Indexes 
 an array of attribute indexes that are set to either true or false
   m_SequentialInstIndexes   - 
Variable in class weka.classifiers.lazy. LBR.Indexes 
 the array of instance indexes that are set to a either true or false
   m_SplitAttrib   - 
Variable in class weka.core.neighboursearch.balltrees. BallNode 
 The attribute that splits this node (not 
 always used).
   m_SplitDim   - 
Variable in class weka.core.neighboursearch.kdtrees. KDTreeNode 
 attribute to split on.
   m_SplitVal   - 
Variable in class weka.core.neighboursearch.balltrees. BallNode 
 The value of m_SpiltAttrib that splits this
 node (not always used).
   m_SplitValue   - 
Variable in class weka.core.neighboursearch.kdtrees. KDTreeNode 
 value to split on.
   m_Start   - 
Variable in class weka.core.neighboursearch.balltrees. BallNode 
 The start index of the portion of the master index array, 
 which stores the indices of the instances/points the node 
 contains.
   m_Start   - 
Variable in class weka.core.neighboursearch.kdtrees. KDTreeNode 
 The start index of the portion of the master index array, 
 which stores the indices of the instances/points the node 
 contains.
   m_statusMessage   - 
Variable in class weka.experiment. RemoteExperimentEvent 
 A status type message
   m_SumC   - 
Variable in class weka.core.neighboursearch. PerformanceStats 
 The sum of coordinates/attributes looked at
 for all the queries.
   m_SumP   - 
Variable in class weka.core.neighboursearch. PerformanceStats 
 The sum of data points looked 
 at for all the queries.
   m_SumSqC   - 
Variable in class weka.core.neighboursearch. PerformanceStats 
 The squared sum of coordinates/attributes looked at
 for all the queries.
   m_SumSqP   - 
Variable in class weka.core.neighboursearch. PerformanceStats 
 The squared sum of data points looked 
 at for all the queries.
   m_useCustomColour   - 
Variable in class weka.gui.visualize. PlotData2D 
 Custom colour for this plot
   m_UseWordwrap   - 
Variable in class weka.gui. LogWindow 
 whether the JTextPane has wordwrap or not
   m_VaryNodes   - 
Variable in class weka.classifiers.bayes.net. ADNode 
 list of VaryNode children
   m_verboseOn   - 
Variable in class weka.core. Debug.DBO 
 enables/disables output of debug information
   m_xChange   - 
Variable in class weka.gui.visualize. AttributePanelEvent 
 True if the x selection changed
   m_yChange   - 
Variable in class weka.gui.visualize. AttributePanelEvent 
 True if the y selection changed
   MahalanobisEstimator   - Class in  weka.estimators  Simple probability estimator that places a single normal distribution
 over the observed values.   MahalanobisEstimator(Matrix, double, double)   - 
Constructor for class weka.estimators. MahalanobisEstimator 
 Constructor
   main(String[])   - 
Static method in class weka.associations. Apriori 
 Main method.
   main(String[])   - 
Static method in class weka.associations. AssociatorEvaluation 
 A test method for this class.
   main(String[])   - 
Static method in class weka.associations. CheckAssociator 
 Test method for this class
   main(String[])   - 
Static method in class weka.associations. FilteredAssociator 
 Main method for running this class.
   main(String[])   - 
Static method in class weka.associations. FPGrowth 
 Main method.
   main(String[])   - 
Static method in class weka.associations. GeneralizedSequentialPatterns 
 Main method.
   main(String[])   - 
Static method in class weka.associations. PredictiveApriori 
 Main method.
   main(String[])   - 
Static method in class weka.associations. Tertius 
 Main method.
   main(String[])   - 
Static method in class weka.attributeSelection. AttributeSelection 
 Main method for testing this class.
   main(String[])   - 
Static method in class weka.attributeSelection. CfsSubsetEval 
 Main method for testing this class.
   main(String[])   - 
Static method in class weka.attributeSelection. CheckAttributeSelection 
 Test method for this class
   main(String[])   - 
Static method in class weka.attributeSelection. ChiSquaredAttributeEval 
 Main method.
   main(String[])   - 
Static method in class weka.attributeSelection. ClassifierSubsetEval 
 Main method for testing this class.
   main(String[])   - 
Static method in class weka.attributeSelection. ConsistencySubsetEval 
 Main method for testing this class.
   main(String[])   - 
Static method in class weka.attributeSelection. CostSensitiveAttributeEval 
 Main method for testing this class.
   main(String[])   - 
Static method in class weka.attributeSelection. CostSensitiveSubsetEval 
 Main method for testing this class.
   main(String[])   - 
Static method in class weka.attributeSelection. FilteredAttributeEval 
 Main method for testing this class.
   main(String[])   - 
Static method in class weka.attributeSelection. FilteredSubsetEval 
 Main method for testing this class.
   main(String[])   - 
Static method in class weka.attributeSelection. GainRatioAttributeEval 
 Main method.
   main(String[])   - 
Static method in class weka.attributeSelection. InfoGainAttributeEval 
 Main method for testing this class.
   main(String[])   - 
Static method in class weka.attributeSelection. LatentSemanticAnalysis 
 Main method for testing this class
   main(String[])   - 
Static method in class weka.attributeSelection. OneRAttributeEval 
 Main method for testing this class.
   main(String[])   - 
Static method in class weka.attributeSelection. PrincipalComponents 
 Main method for testing this class
   main(String[])   - 
Static method in class weka.attributeSelection. ReliefFAttributeEval 
 Main method for testing this class.
   main(String[])   - 
Static method in class weka.attributeSelection. SVMAttributeEval 
 Main method for testing this class.
   main(String[])   - 
Static method in class weka.attributeSelection. SymmetricalUncertAttributeEval 
 Main method for testing this class.
   main(String[])   - 
Static method in class weka.attributeSelection. WrapperSubsetEval 
 Main method for testing this class.
   main(String[])   - 
Static method in class weka.classifiers.bayes. AODE 
 Main method for testing this class.
   main(String[])   - 
Static method in class weka.classifiers.bayes. AODEsr 
 Main method for testing this class.
   main(String[])   - 
Static method in class weka.classifiers.bayes. BayesianLogisticRegression 
 Main method for testing this class.
   main(String[])   - 
Static method in class weka.classifiers.bayes. BayesNet 
 Main method for testing this class.
   main(String[])   - 
Static method in class weka.classifiers.bayes. ComplementNaiveBayes 
 Main method for testing this class.
   main(String[])   - 
Static method in class weka.classifiers.bayes. DMNBtext 
 Main method for testing this class.
   main(String[])   - 
Static method in class weka.classifiers.bayes. HNB 
 Main method for testing this class.
   main(String[])   - 
Static method in class weka.classifiers.bayes. NaiveBayes 
 Main method for testing this class.
   main(String[])   - 
Static method in class weka.classifiers.bayes. NaiveBayesMultinomial 
 Main method for testing this class.
   main(String[])   - 
Static method in class weka.classifiers.bayes. NaiveBayesMultinomialUpdateable 
 Main method for testing this class.
   main(String[])   - 
Static method in class weka.classifiers.bayes. NaiveBayesSimple 
 Main method for testing this class.
   main(String[])   - 
Static method in class weka.classifiers.bayes. NaiveBayesUpdateable 
 Main method for testing this class.
   main(String[])   - 
Static method in class weka.classifiers.bayes.net. ADNode 
 for testing only
   main(String[])   - 
Static method in class weka.classifiers.bayes.net. BayesNetGenerator 
 Main method
   main(String[])   - 
Static method in class weka.classifiers.bayes.net. BIFReader 
 Loads the file specified as first parameter and prints it to stdout.
   main(String[])   - 
Static method in class weka.classifiers.bayes.net. EditableBayesNet 
  
   main(String[])   - 
Static method in class weka.classifiers.bayes.net.estimate. DiscreteEstimatorBayes 
 Main method for testing this class.
   main(String[])   - 
Static method in class weka.classifiers.bayes.net.estimate. DiscreteEstimatorFullBayes 
 Main method for testing this class.
   main(String[])   - 
Static method in class weka.classifiers.bayes.net. GUI 
 Main method.
   main(String[])   - 
Static method in class weka.classifiers.bayes.net. MarginCalculator 
  
   main(String[])   - 
Static method in class weka.classifiers.bayes.net.search.ci. ICSSearchAlgorithm 
 for testing the class
   main(String[])   - 
Static method in class weka.classifiers.bayes. WAODE 
 Main method for testing this class.
   main(String[])   - 
Static method in class weka.classifiers. BVDecompose 
 Test method for this class
   main(String[])   - 
Static method in class weka.classifiers. BVDecomposeSegCVSub 
 Test method for this class
   main(String[])   - 
Static method in class weka.classifiers. CheckClassifier 
 Test method for this class
   main(String[])   - 
Static method in class weka.classifiers. CheckSource 
 Executes the tests, use "-h" to list the commandline options.
   main(String[])   - 
Static method in class weka.classifiers.evaluation. CostCurve 
 Tests the CostCurve generation from the command line.
   main(String[])   - 
Static method in class weka.classifiers. Evaluation 
 A test method for this class.
   main(String[])   - 
Static method in class weka.classifiers.evaluation. MarginCurve 
 Tests the MarginCurve generation from the command line.
   main(String[])   - 
Static method in class weka.classifiers.evaluation. ThresholdCurve 
 Tests the ThresholdCurve generation from the command line.
   main(String[])   - 
Static method in class weka.classifiers.functions. GaussianProcesses 
 Main method for testing this class.
   main(String[])   - 
Static method in class weka.classifiers.functions. IsotonicRegression 
 Main method for testing this class
   main(String[])   - 
Static method in class weka.classifiers.functions. LeastMedSq 
 generate a Linear regression predictor for testing
   main(String[])   - 
Static method in class weka.classifiers.functions. LibLINEAR 
 Main method for testing this class.
   main(String[])   - 
Static method in class weka.classifiers.functions. LibSVM 
 Main method for testing this class.
   main(String[])   - 
Static method in class weka.classifiers.functions. LinearRegression 
 Generates a linear regression function predictor.
   main(String[])   - 
Static method in class weka.classifiers.functions. Logistic 
 Main method for testing this class.
   main(String[])   - 
Static method in class weka.classifiers.functions. MultilayerPerceptron 
 Main method for testing this class.
   main(String[])   - 
Static method in class weka.classifiers.functions.pace. ChisqMixture 
 Method to test this class
   main(String[])   - 
Static method in class weka.classifiers.functions.pace. DiscreteFunction 
  
   main(String[])   - 
Static method in class weka.classifiers.functions.pace. NormalMixture 
 Method to test this class
   main(String[])   - 
Static method in class weka.classifiers.functions.pace. PaceMatrix 
 for testing only
   main(String[])   - 
Static method in class weka.classifiers.functions. PaceRegression 
 Generates a linear regression function predictor.
   main(String[])   - 
Static method in class weka.classifiers.functions. PLSClassifier 
 Main method for running this classifier from commandline.
   main(String[])   - 
Static method in class weka.classifiers.functions. RBFNetwork 
 Main method for testing this class.
   main(String[])   - 
Static method in class weka.classifiers.functions. SimpleLinearRegression 
 Main method for testing this class
   main(String[])   - 
Static method in class weka.classifiers.functions. SimpleLogistic 
 Main method for testing this class
   main(String[])   - 
Static method in class weka.classifiers.functions. SMO 
 Main method for testing this class.
   main(String[])   - 
Static method in class weka.classifiers.functions. SMOreg 
 Main method for running this classifier.
   main(String[])   - 
Static method in class weka.classifiers.functions. SPegasos 
 Main method for testing this class.
   main(String[])   - 
Static method in class weka.classifiers.functions.supportVector. CheckKernel 
 Test method for this class
   main(String[])   - 
Static method in class weka.classifiers.functions.supportVector. KernelEvaluation 
 A test method for this class.
   main(String[])   - 
Static method in class weka.classifiers.functions. VotedPerceptron 
 Main method.
   main(String[])   - 
Static method in class weka.classifiers.functions. Winnow 
 Main method.
   main(String[])   - 
Static method in class weka.classifiers.lazy. IB1 
 Main method for testing this class.
   main(String[])   - 
Static method in class weka.classifiers.lazy. IBk 
 Main method for testing this class.
   main(String[])   - 
Static method in class weka.classifiers.lazy. KStar 
 Main method for testing this class.
   main(String[])   - 
Static method in class weka.classifiers.lazy. LBR 
 Main method for testing this class.
   main(String[])   - 
Static method in class weka.classifiers.lazy. LWL 
 Main method for testing this class.
   main(String[])   - 
Static method in class weka.classifiers.meta. AdaBoostM1 
 Main method for testing this class.
   main(String[])   - 
Static method in class weka.classifiers.meta. AdditiveRegression 
 Main method for testing this class.
   main(String[])   - 
Static method in class weka.classifiers.meta. AttributeSelectedClassifier 
 Main method for testing this class.
   main(String[])   - 
Static method in class weka.classifiers.meta. Bagging 
 Main method for testing this class.
   main(String[])   - 
Static method in class weka.classifiers.meta. ClassificationViaClustering 
 Runs the classifier with the given options
   main(String[])   - 
Static method in class weka.classifiers.meta. ClassificationViaRegression 
 Main method for testing this class.
   main(String[])   - 
Static method in class weka.classifiers.meta. CostSensitiveClassifier 
 Main method for testing this class.
   main(String[])   - 
Static method in class weka.classifiers.meta. CVParameterSelection 
 Main method for testing this class.
   main(String[])   - 
Static method in class weka.classifiers.meta. Dagging 
 Main method for testing this class.
   main(String[])   - 
Static method in class weka.classifiers.meta. Decorate 
 Main method for testing this class.
   main(String[])   - 
Static method in class weka.classifiers.meta. END 
 Main method for testing this class.
   main(String[])   - 
Static method in class weka.classifiers.meta. FilteredClassifier 
 Main method for testing this class.
   main(String[])   - 
Static method in class weka.classifiers.meta. Grading 
 Main method for testing this class.
   main(String[])   - 
Static method in class weka.classifiers.meta. GridSearch 
 Main method for running this classifier from commandline.
   main(String[])   - 
Static method in class weka.classifiers.meta. LogitBoost 
 Main method for testing this class.
   main(String[])   - 
Static method in class weka.classifiers.meta. MetaCost 
 Main method for testing this class.
   main(String[])   - 
Static method in class weka.classifiers.meta. MultiBoostAB 
 Main method for testing this class.
   main(String[])   - 
Static method in class weka.classifiers.meta. MultiClassClassifier 
 Main method for testing this class.
   main(String[])   - 
Static method in class weka.classifiers.meta. MultiScheme 
 Main method for testing this class.
   main(String[])   - 
Static method in class weka.classifiers.meta.nestedDichotomies. ClassBalancedND 
 Main method for testing this class.
   main(String[])   - 
Static method in class weka.classifiers.meta.nestedDichotomies. DataNearBalancedND 
 Main method for testing this class.
   main(String[])   - 
Static method in class weka.classifiers.meta.nestedDichotomies. ND 
 Main method for testing this class.
   main(String[])   - 
Static method in class weka.classifiers.meta. OrdinalClassClassifier 
 Main method for testing this class.
   main(String[])   - 
Static method in class weka.classifiers.meta. RacedIncrementalLogitBoost 
 Main method for this class.
   main(String[])   - 
Static method in class weka.classifiers.meta. RandomCommittee 
 Main method for testing this class.
   main(String[])   - 
Static method in class weka.classifiers.meta. RandomSubSpace 
 Main method for testing this class.
   main(String[])   - 
Static method in class weka.classifiers.meta. RegressionByDiscretization 
 Main method for testing this class.
   main(String[])   - 
Static method in class weka.classifiers.meta. RotationForest 
 Main method for testing this class.
   main(String[])   - 
Static method in class weka.classifiers.meta. Stacking 
 Main method for testing this class.
   main(String[])   - 
Static method in class weka.classifiers.meta. StackingC 
 Main method for testing this class.
   main(String[])   - 
Static method in class weka.classifiers.meta. ThresholdSelector 
 Main method for testing this class.
   main(String[])   - 
Static method in class weka.classifiers.meta. Vote 
 Main method for testing this class.
   main(String[])   - 
Static method in class weka.classifiers.mi. CitationKNN 
 Main method for testing this class.
   main(String[])   - 
Static method in class weka.classifiers.mi. MDD 
 Main method for testing this class.
   main(String[])   - 
Static method in class weka.classifiers.mi. MIBoost 
 Main method for testing this class.
   main(String[])   - 
Static method in class weka.classifiers.mi. MIDD 
 Main method for testing this class.
   main(String[])   - 
Static method in class weka.classifiers.mi. MIEMDD 
 Main method for testing this class.
   main(String[])   - 
Static method in class weka.classifiers.mi. MILR 
 Main method for testing this class.
   main(String[])   - 
Static method in class weka.classifiers.mi. MINND 
 Main method for testing.
   main(String[])   - 
Static method in class weka.classifiers.mi. MIOptimalBall 
 Main method for testing this class.
   main(String[])   - 
Static method in class weka.classifiers.mi. MISMO 
 Main method for testing this class.
   main(String[])   - 
Static method in class weka.classifiers.mi. MISVM 
 Main method for testing this class.
   main(String[])   - 
Static method in class weka.classifiers.mi. MIWrapper 
 Main method for testing this class.
   main(String[])   - 
Static method in class weka.classifiers.mi. SimpleMI 
 Main method for testing this class.
   main(String[])   - 
Static method in class weka.classifiers.misc. HyperPipes 
 Main method for testing this class.
   main(String[])   - 
Static method in class weka.classifiers.misc. SerializedClassifier 
 Runs the classifier with the given options
   main(String[])   - 
Static method in class weka.classifiers.misc. VFI 
 Main method for testing this class.
   main(String[])   - 
Static method in class weka.classifiers.rules. ConjunctiveRule 
 Main method.
   main(String[])   - 
Static method in class weka.classifiers.rules. DecisionTable 
 Main method for testing this class.
   main(String[])   - 
Static method in class weka.classifiers.rules. DTNB 
 Main method for testing this class.
   main(String[])   - 
Static method in class weka.classifiers.rules. JRip 
 Main method.
   main(String[])   - 
Static method in class weka.classifiers.rules. M5Rules 
 Main method by which this class can be tested
   main(String[])   - 
Static method in class weka.classifiers.rules. NNge 
 Main method for testing this class.
   main(String[])   - 
Static method in class weka.classifiers.rules. OneR 
 Main method for testing this class
   main(String[])   - 
Static method in class weka.classifiers.rules. PART 
 Main method for testing this class.
   main(String[])   - 
Static method in class weka.classifiers.rules. Prism 
 Main method for testing this class
   main(String[])   - 
Static method in class weka.classifiers.rules. Ridor 
 Main method.
   main(String[])   - 
Static method in class weka.classifiers.rules. ZeroR 
 Main method for testing this class.
   main(String[])   - 
Static method in class weka.classifiers.trees. ADTree 
 Main method for testing this class.
   main(String[])   - 
Static method in class weka.classifiers.trees. BFTree 
 Main method.
   main(String[])   - 
Static method in class weka.classifiers.trees. DecisionStump 
 Main method for testing this class.
   main(String[])   - 
Static method in class weka.classifiers.trees. FT 
 Main method for testing this class
   main(String[])   - 
Static method in class weka.classifiers.trees. Id3 
 Main method.
   main(String[])   - 
Static method in class weka.classifiers.trees. J48 
 Main method for testing this class
   main(String[])   - 
Static method in class weka.classifiers.trees. J48graft 
 Main method for testing this class
   main(String[])   - 
Static method in class weka.classifiers.trees. LADTree 
 Main method for testing this class.
   main(String[])   - 
Static method in class weka.classifiers.trees. LMT 
 Main method for testing this class
   main(String[])   - 
Static method in class weka.classifiers.trees. M5P 
 Main method by which this class can be tested
   main(String[])   - 
Static method in class weka.classifiers.trees. NBTree 
 Main method for testing this class
   main(String[])   - 
Static method in class weka.classifiers.trees. RandomForest 
 Main method for this class.
   main(String[])   - 
Static method in class weka.classifiers.trees. RandomTree 
 Main method for this class.
   main(String[])   - 
Static method in class weka.classifiers.trees. REPTree 
 Main method for this class.
   main(String[])   - 
Static method in class weka.classifiers.trees. SimpleCart 
 Main method.
   main(String[])   - 
Static method in class weka.classifiers.trees. UserClassifier 
 Main method for testing this class.
   main(String[])   - 
Static method in class weka.clusterers. CheckClusterer 
 Test method for this class
   main(String[])   - 
Static method in class weka.clusterers. CLOPE 
 Main method for testing this class.
   main(String[])   - 
Static method in class weka.clusterers. ClusterEvaluation 
 Main method for testing this class.
   main(String[])   - 
Static method in class weka.clusterers. Cobweb 
 Main method.
   main(String[])   - 
Static method in class weka.clusterers. DBScan 
 Main Method for testing DBScan
   main(String[])   - 
Static method in class weka.clusterers. EM 
 Main method for testing this class.
   main(String[])   - 
Static method in class weka.clusterers. FarthestFirst 
 Main method for testing this class.
   main(String[])   - 
Static method in class weka.clusterers. FilteredClusterer 
 Main method for testing this class.
   main(String[])   - 
Static method in class weka.clusterers.forOPTICSAndDBScan.OPTICS_GUI. OPTICS_Visualizer 
 Displays the GUI.
   main(String[])   - 
Static method in class weka.clusterers. HierarchicalClusterer 
  
   main(String[])   - 
Static method in class weka.clusterers. MakeDensityBasedClusterer 
 Main method for testing this class.
   main(String[])   - 
Static method in class weka.clusterers. OPTICS 
 Main Method for testing OPTICS
   main(String[])   - 
Static method in class weka.clusterers. sIB 
  
   main(String[])   - 
Static method in class weka.clusterers. SimpleKMeans 
 Main method for testing this class.
   main(String[])   - 
Static method in class weka.clusterers. XMeans 
 Main method for testing this class.
   main(String[])   - 
Static method in class weka.core. AlgVector 
 Main method for testing this class, can take an ARFF file as first argument.
   main(String[])   - 
Static method in class weka.core. AllJavadoc 
 Parses the given commandline parameters and generates the Javadoc.
   main(String[])   - 
Static method in class weka.core. Attribute 
 Simple main method for testing this class.
   main(String[])   - 
Static method in class weka.core. BinarySparseInstance 
 Main method for testing this class.
   main(String[])   - 
Static method in class weka.core. Capabilities 
 loads the given dataset and prints the Capabilities necessary to 
 process it.
   main(String[])   - 
Static method in class weka.core. CheckGOE 
 Main method for using the CheckGOE.
   main(String[])   - 
Static method in class weka.core. CheckOptionHandler 
 Main method for using the CheckOptionHandler.
   main(String[])   - 
Static method in class weka.core. ClassDiscovery 
 Possible calls:
 
    
      weka.core.ClassDiscovery <packages> 
      Prints all the packages in the current classpath
    
    
      weka.core.ClassDiscovery <classname> <packagename(s)> 
      Prints the classes it found.
   main(String[])   - 
Static method in class weka.core. ContingencyTables 
 Main method for testing this class.
   main(String[])   - 
Static method in class weka.core.converters. ArffLoader 
 Main method.
   main(String[])   - 
Static method in class weka.core.converters. ArffSaver 
 Main method.
   main(String[])   - 
Static method in class weka.core.converters. C45Loader 
 Main method for testing this class.
   main(String[])   - 
Static method in class weka.core.converters. C45Saver 
 Main method.
   main(String[])   - 
Static method in class weka.core.converters. ConverterUtils.DataSink 
 for testing only - takes a data file as input and a data file for the
 output.
   main(String[])   - 
Static method in class weka.core.converters. ConverterUtils.DataSource 
 for testing only - takes a data file as input.
   main(String[])   - 
Static method in class weka.core.converters. CSVLoader 
 Main method.
   main(String[])   - 
Static method in class weka.core.converters. CSVSaver 
 Main method.
   main(String[])   - 
Static method in class weka.core.converters. DatabaseLoader 
 Main method.
   main(String[])   - 
Static method in class weka.core.converters. DatabaseSaver 
 Main method.
   main(String[])   - 
Static method in class weka.core.converters. LibSVMLoader 
 Main method.
   main(String[])   - 
Static method in class weka.core.converters. LibSVMSaver 
 Main method.
   main(String[])   - 
Static method in class weka.core.converters. SerializedInstancesLoader 
 Main method.
   main(String[])   - 
Static method in class weka.core.converters. SerializedInstancesSaver 
 Main method.
   main(String[])   - 
Static method in class weka.core.converters. SVMLightLoader 
 Main method.
   main(String[])   - 
Static method in class weka.core.converters. SVMLightSaver 
 Main method.
   main(String[])   - 
Static method in class weka.core.converters. TextDirectoryLoader 
 Main method.
   main(String[])   - 
Static method in class weka.core.converters. XRFFLoader 
 Main method.
   main(String[])   - 
Static method in class weka.core.converters. XRFFSaver 
 Main method.
   main(String[])   - 
Static method in class weka.core. Copyright 
 Only for testing
   main(String[])   - 
Static method in class weka.core. Environment 
 Main method for testing this class.
   main(String[])   - 
Static method in class weka.core. FindWithCapabilities 
 Executes the location of classes with parameters from the commandline.
   main(String[])   - 
Static method in class weka.core. GlobalInfoJavadoc 
 Parses the given commandline parameters and generates the Javadoc.
   main(String[])   - 
Static method in class weka.core. Instance 
 Main method for testing this class.
   main(String[])   - 
Static method in class weka.core. InstanceComparator 
 for testing only.
   main(String[])   - 
Static method in class weka.core. Instances 
 Main method for this class.
   main(String[])   - 
Static method in class weka.core. Jython 
 If no arguments are given, it just prints the presence of the Jython
 classes, otherwise it expects a Jython filename to execute.
   main(String[])   - 
Static method in class weka.core. ListOptions 
 runs the javadoc producer with the given commandline options
   main(String[])   - 
Static method in class weka.core.mathematicalexpression. Parser 
 Runs the parser from commandline.
   main(String[])   - 
Static method in class weka.core.matrix. DoubleVector 
  
   main(String[])   - 
Static method in class weka.core.matrix. IntVector 
 Tests the IntVector class
   main(String[])   - 
Static method in class weka.core. Matrix 
  Deprecated.  Main method for testing this class.
   main(String[])   - 
Static method in class weka.core.matrix. Matrix 
 Main method for testing this class.
   main(String[])   - 
Static method in class weka.core. Memory 
 prints only some statistics
   main(String[])   - 
Static method in class weka.core.neighboursearch. CoverTree 
 Method for testing the class from command line.
   main(String[])   - 
Static method in class weka.core. OptionHandlerJavadoc 
 Parses the given commandline parameters and generates the Javadoc.
   main(String[])   - 
Static method in class weka.core.pmml. Constant 
  
   main(String[])   - 
Static method in class weka.core.pmml. PMMLFactory 
  
   main(String[])   - 
Static method in class weka.core. PropertyPath 
 for testing only
   main(String[])   - 
Static method in class weka.core. Queue 
 Main method for testing this class.
   main(String[])   - 
Static method in class weka.core. RandomVariates 
 Main method for testing this class.
   main(String[])   - 
Static method in class weka.core. Range 
 Main method for testing this class.
   main(String[])   - 
Static method in class weka.core. RevisionUtils 
 For testing only.
   main(String[])   - 
Static method in class weka.core. SerializationHelper 
 Outputs information about a class on the commandline, takes class
 name as arguments.
   main(String[])   - 
Static method in class weka.core. SingleIndex 
 Main method for testing this class.
   main(String[])   - 
Static method in class weka.core. SparseInstance 
 Main method for testing this class.
   main(String[])   - 
Static method in class weka.core. SpecialFunctions 
 Main method for testing this class.
   main(String[])   - 
Static method in class weka.core. Statistics 
 Main method for testing this class.
   main(String[])   - 
Static method in class weka.core.stemmers. IteratedLovinsStemmer 
 Runs the stemmer with the given options
   main(String[])   - 
Static method in class weka.core.stemmers. LovinsStemmer 
 Runs the stemmer with the given options
   main(String[])   - 
Static method in class weka.core.stemmers. NullStemmer 
 Runs the stemmer with the given options
   main(String[])   - 
Static method in class weka.core.stemmers. SnowballStemmer 
 Runs the stemmer with the given options.
   main(String[])   - 
Static method in class weka.core. Stopwords 
 Accepts the following parameter:  

 -i file  
 loads the stopwords from the given file  

 -o file  
 saves the stopwords to the given file  

 -p  
 outputs the current stopwords on stdout  

 Any additional parameters are interpreted as words to test as stopwords.
   main(String[])   - 
Static method in class weka.core. SystemInfo 
 for printing the system info to stdout.
   main(String[])   - 
Static method in class weka.core. TechnicalInformation 
 Prints some examples of technical informations if there are no 
 commandline options given.
   main(String[])   - 
Static method in class weka.core. TechnicalInformationHandlerJavadoc 
 Parses the given commandline parameters and generates the Javadoc.
   main(String[])   - 
Static method in class weka.core. TestInstances 
 for running the class from commandline, prints the generated data
 to stdout
   main(String[])   - 
Static method in class weka.core.tokenizers. AlphabeticTokenizer 
 Runs the tokenizer with the given options and strings to tokenize.
   main(String[])   - 
Static method in class weka.core.tokenizers. NGramTokenizer 
 Runs the tokenizer with the given options and strings to tokenize.
   main(String[])   - 
Static method in class weka.core.tokenizers. WordTokenizer 
 Runs the tokenizer with the given options and strings to tokenize.
   main(String[])   - 
Static method in class weka.core. Trie 
 Only for testing (prints the built Trie).
   main(String[])   - 
Static method in class weka.core. Utils 
 Main method for testing this class.
   main(String[])   - 
Static method in class weka.core. Version 
 only for testing
   main(String[])   - 
Static method in class weka.core.xml. SerialUIDChanger 
 exchanges an old UID for a new one.
   main(String[])   - 
Static method in class weka.core.xml. XMLDocument 
 for testing only.
   main(String[])   - 
Static method in class weka.core.xml. XMLInstances 
 takes an XML document as first argument and then outputs the Instances
 statistics
   main(String[])   - 
Static method in class weka.core.xml. XMLOptions 
 for testing only.
   main(String[])   - 
Static method in class weka.core.xml. XMLSerialization 
 for testing only.
   main(String[])   - 
Static method in class weka.datagenerators.classifiers.classification. Agrawal 
 Main method for executing this class.
   main(String[])   - 
Static method in class weka.datagenerators.classifiers.classification. BayesNet 
 Main method for executing this class.
   main(String[])   - 
Static method in class weka.datagenerators.classifiers.classification. LED24 
 Main method for executing this class.
   main(String[])   - 
Static method in class weka.datagenerators.classifiers.classification. RandomRBF 
 Main method for executing this class.
   main(String[])   - 
Static method in class weka.datagenerators.classifiers.classification. RDG1 
 Main method for testing this class.
   main(String[])   - 
Static method in class weka.datagenerators.classifiers.regression. Expression 
 Main method for testing this class.
   main(String[])   - 
Static method in class weka.datagenerators.classifiers.regression. MexicanHat 
 Main method for testing this class.
   main(String[])   - 
Static method in class weka.datagenerators.clusterers. BIRCHCluster 
 Main method for testing this class.
   main(String[])   - 
Static method in class weka.datagenerators.clusterers. SubspaceCluster 
 Main method for testing this class.
   main(String[])   - 
Static method in class weka.estimators. CheckEstimator 
 Test method for this class
   main(String[])   - 
Static method in class weka.estimators. DDConditionalEstimator 
 Main method for testing this class.
   main(String[])   - 
Static method in class weka.estimators. DiscreteEstimator 
 Main method for testing this class.
   main(String[])   - 
Static method in class weka.estimators. DKConditionalEstimator 
 Main method for testing this class.
   main(String[])   - 
Static method in class weka.estimators. DNConditionalEstimator 
 Main method for testing this class.
   main(String[])   - 
Static method in class weka.estimators. KDConditionalEstimator 
 Main method for testing this class.
   main(String[])   - 
Static method in class weka.estimators. KernelEstimator 
 Main method for testing this class.
   main(String[])   - 
Static method in class weka.estimators. KKConditionalEstimator 
 Main method for testing this class.
   main(String[])   - 
Static method in class weka.estimators. MahalanobisEstimator 
 Main method for testing this class.
   main(String[])   - 
Static method in class weka.estimators. NDConditionalEstimator 
 Main method for testing this class.
   main(String[])   - 
Static method in class weka.estimators. NNConditionalEstimator 
 Main method for testing this class.
   main(String[])   - 
Static method in class weka.estimators. NormalEstimator 
 Main method for testing this class.
   main(String[])   - 
Static method in class weka.estimators. PoissonEstimator 
 Main method for testing this class.
   main(String[])   - 
Static method in class weka.experiment. CrossValidationResultProducer 
 Quick test of timestamp
   main(String[])   - 
Static method in class weka.experiment. Experiment 
 Configures/Runs the Experiment from the command line.
   main(String[])   - 
Static method in class weka.experiment. InstanceQuery 
 Test the class from the command line.
   main(String[])   - 
Static method in class weka.experiment. OutputZipper 
 Main method for testing this class
   main(String[])   - 
Static method in class weka.experiment. PairedCorrectedTTester 
 Test the class from the command line.
   main(String[])   - 
Static method in class weka.experiment. PairedStats 
 Tests the paired stats object from the command line.
   main(String[])   - 
Static method in class weka.experiment. PairedTTester 
 Test the class from the command line.
   main(String[])   - 
Static method in class weka.experiment. RemoteEngine 
 Main method.
   main(String[])   - 
Static method in class weka.experiment. RemoteExperiment 
 Configures/Runs the Experiment from the command line.
   main(String[])   - 
Static method in class weka.experiment. ResultMatrixCSV 
 for testing only
   main(String[])   - 
Static method in class weka.experiment. ResultMatrixGnuPlot 
 for testing only
   main(String[])   - 
Static method in class weka.experiment. ResultMatrixHTML 
 for testing only
   main(String[])   - 
Static method in class weka.experiment. ResultMatrixLatex 
 for testing only
   main(String[])   - 
Static method in class weka.experiment. ResultMatrixPlainText 
 for testing only
   main(String[])   - 
Static method in class weka.experiment. ResultMatrixSignificance 
 for testing only
   main(String[])   - 
Static method in class weka.experiment. Stats 
 Tests the paired stats object from the command line.
   main(String[])   - 
Static method in class weka.experiment.xml. XMLExperiment 
 for testing only.
   main(String[])   - 
Static method in class weka.filters. AllFilter 
 Main method for testing this class.
   main(String[])   - 
Static method in class weka.filters. CheckSource 
 Executes the tests, use "-h" to list the commandline options.
   main(String[])   - 
Static method in class weka.filters. Filter 
 Main method for testing this class.
   main(String[])   - 
Static method in class weka.filters. MultiFilter 
 Main method for executing this class.
   main(String[])   - 
Static method in class weka.filters.supervised.attribute. AddClassification 
 runs the filter with the given arguments.
   main(String[])   - 
Static method in class weka.filters.supervised.attribute. AttributeSelection 
 Main method for testing this class.
   main(String[])   - 
Static method in class weka.filters.supervised.attribute. ClassOrder 
 Main method for testing this class.
   main(String[])   - 
Static method in class weka.filters.supervised.attribute. Discretize 
 Main method for testing this class.
   main(String[])   - 
Static method in class weka.filters.supervised.attribute. NominalToBinary 
 Main method for testing this class.
   main(String[])   - 
Static method in class weka.filters.supervised.attribute. PLSFilter 
 runs the filter with the given arguments.
   main(String[])   - 
Static method in class weka.filters.supervised.instance. Resample 
 Main method for testing this class.
   main(String[])   - 
Static method in class weka.filters.supervised.instance. SMOTE 
 Main method for running this filter.
   main(String[])   - 
Static method in class weka.filters.supervised.instance. SpreadSubsample 
 Main method for testing this class.
   main(String[])   - 
Static method in class weka.filters.supervised.instance. StratifiedRemoveFolds 
 Main method for testing this class.
   main(String[])   - 
Static method in class weka.filters.unsupervised.attribute. Add 
 Main method for testing this class.
   main(String[])   - 
Static method in class weka.filters.unsupervised.attribute. AddCluster 
 Main method for testing this class.
   main(String[])   - 
Static method in class weka.filters.unsupervised.attribute. AddExpression 
 Main method for testing this class.
   main(String[])   - 
Static method in class weka.filters.unsupervised.attribute. AddID 
 Main method for testing this class.
   main(String[])   - 
Static method in class weka.filters.unsupervised.attribute. AddNoise 
 Main method for testing this class.
   main(String[])   - 
Static method in class weka.filters.unsupervised.attribute. AddValues 
 Main method for testing and running this class.
   main(String[])   - 
Static method in class weka.filters.unsupervised.attribute. Center 
 Main method for running this filter.
   main(String[])   - 
Static method in class weka.filters.unsupervised.attribute. ChangeDateFormat 
 Main method for testing this class.
   main(String[])   - 
Static method in class weka.filters.unsupervised.attribute. ClassAssigner 
 Main method for executing this class.
   main(String[])   - 
Static method in class weka.filters.unsupervised.attribute. ClusterMembership 
 Main method for testing this class.
   main(String[])   - 
Static method in class weka.filters.unsupervised.attribute. Copy 
 Main method for testing this class.
   main(String[])   - 
Static method in class weka.filters.unsupervised.attribute. Discretize 
 Main method for testing this class.
   main(String[])   - 
Static method in class weka.filters.unsupervised.attribute. FirstOrder 
 Main method for testing this class.
   main(String[])   - 
Static method in class weka.filters.unsupervised.attribute. InterquartileRange 
 Main method for testing this class.
   main(String[])   - 
Static method in class weka.filters.unsupervised.attribute. KernelFilter 
 runs the filter with the given arguments
   main(String[])   - 
Static method in class weka.filters.unsupervised.attribute. MakeIndicator 
 Main method for testing this class.
   main(String[])   - 
Static method in class weka.filters.unsupervised.attribute. MathExpression 
 Main method for testing this class.
   main(String[])   - 
Static method in class weka.filters.unsupervised.attribute. MergeTwoValues 
 Main method for testing this class.
   main(String[])   - 
Static method in class weka.filters.unsupervised.attribute. MultiInstanceToPropositional 
 Main method for running this filter.
   main(String[])   - 
Static method in class weka.filters.unsupervised.attribute. NominalToBinary 
 Main method for testing this class.
   main(String[])   - 
Static method in class weka.filters.unsupervised.attribute. NominalToString 
 Main method for testing this class.
   main(String[])   - 
Static method in class weka.filters.unsupervised.attribute. Normalize 
 Main method for running this filter.
   main(String[])   - 
Static method in class weka.filters.unsupervised.attribute. NumericCleaner 
 Runs the filter from commandline, use "-h" to see all options.
   main(String[])   - 
Static method in class weka.filters.unsupervised.attribute. NumericToBinary 
 Main method for testing this class.
   main(String[])   - 
Static method in class weka.filters.unsupervised.attribute. NumericToNominal 
 Runs the filter with the given parameters.
   main(String[])   - 
Static method in class weka.filters.unsupervised.attribute. NumericTransform 
 Main method for testing this class.
   main(String[])   - 
Static method in class weka.filters.unsupervised.attribute. Obfuscate 
 Main method for testing this class.
   main(String[])   - 
Static method in class weka.filters.unsupervised.attribute. PartitionedMultiFilter 
 Main method for executing this class.
   main(String[])   - 
Static method in class weka.filters.unsupervised.attribute. PKIDiscretize 
 Main method for testing this class.
   main(String[])   - 
Static method in class weka.filters.unsupervised.attribute. PrincipalComponents 
 Main method for running this filter.
   main(String[])   - 
Static method in class weka.filters.unsupervised.attribute. PropositionalToMultiInstance 
 Main method for running this filter.
   main(String[])   - 
Static method in class weka.filters.unsupervised.attribute. RandomProjection 
 Main method for testing this class.
   main(String[])   - 
Static method in class weka.filters.unsupervised.attribute. RandomSubset 
 Runs the filter with the given parameters.
   main(String[])   - 
Static method in class weka.filters.unsupervised.attribute. RELAGGS 
 runs the filter with the given arguments
   main(String[])   - 
Static method in class weka.filters.unsupervised.attribute. Remove 
 Main method for testing this class.
   main(String[])   - 
Static method in class weka.filters.unsupervised.attribute. RemoveType 
 Main method for testing this class.
   main(String[])   - 
Static method in class weka.filters.unsupervised.attribute. RemoveUseless 
 Main method for testing this class.
   main(String[])   - 
Static method in class weka.filters.unsupervised.attribute. Reorder 
 Main method for testing this class.
   main(String[])   - 
Static method in class weka.filters.unsupervised.attribute. ReplaceMissingValues 
 Main method for testing this class.
   main(String[])   - 
Static method in class weka.filters.unsupervised.attribute. Standardize 
 Main method for testing this class.
   main(String[])   - 
Static method in class weka.filters.unsupervised.attribute. StringToNominal 
 Main method for testing this class.
   main(String[])   - 
Static method in class weka.filters.unsupervised.attribute. StringToWordVector 
 Main method for testing this class.
   main(String[])   - 
Static method in class weka.filters.unsupervised.attribute. SwapValues 
 Main method for testing this class.
   main(String[])   - 
Static method in class weka.filters.unsupervised.attribute. TimeSeriesDelta 
 Main method for testing this class.
   main(String[])   - 
Static method in class weka.filters.unsupervised.attribute. TimeSeriesTranslate 
 Main method for testing this class.
   main(String[])   - 
Static method in class weka.filters.unsupervised.attribute. Wavelet 
 runs the filter with the given arguments
   main(String[])   - 
Static method in class weka.filters.unsupervised.instance. NonSparseToSparse 
 Main method for testing this class.
   main(String[])   - 
Static method in class weka.filters.unsupervised.instance. Normalize 
 Main method for testing this class.
   main(String[])   - 
Static method in class weka.filters.unsupervised.instance. Randomize 
 Main method for testing this class.
   main(String[])   - 
Static method in class weka.filters.unsupervised.instance. RemoveFolds 
 Main method for testing this class.
   main(String[])   - 
Static method in class weka.filters.unsupervised.instance. RemoveFrequentValues 
 Main method for testing this class.
   main(String[])   - 
Static method in class weka.filters.unsupervised.instance. RemoveMisclassified 
 Main method for testing this class.
   main(String[])   - 
Static method in class weka.filters.unsupervised.instance. RemovePercentage 
 Main method for testing this class.
   main(String[])   - 
Static method in class weka.filters.unsupervised.instance. RemoveRange 
 Main method for testing this class.
   main(String[])   - 
Static method in class weka.filters.unsupervised.instance. RemoveWithValues 
 Main method for testing this class.
   main(String[])   - 
Static method in class weka.filters.unsupervised.instance. Resample 
 Main method for testing this class.
   main(String[])   - 
Static method in class weka.filters.unsupervised.instance. ReservoirSample 
 Main method for testing this class.
   main(String[])   - 
Static method in class weka.filters.unsupervised.instance. SparseToNonSparse 
 Main method for testing this class.
   main(String[])   - 
Static method in class weka.filters.unsupervised.instance. SubsetByExpression 
 Main method for running this filter.
   main(String[])   - 
Static method in class weka.filters.unsupervised.instance.subsetbyexpression. Parser 
 Runs the parser from commandline.
   main(String[])   - 
Static method in class weka.gui.arffviewer. ArffViewer 
 shows the frame and it tries to load all the arff files that were
 provided as arguments.
   main(String[])   - 
Static method in class weka.gui. AttributeListPanel 
 Tests the attribute list panel from the command line.
   main(String[])   - 
Static method in class weka.gui. AttributeSelectionPanel 
 Tests the attribute selection panel from the command line.
   main(String[])   - 
Static method in class weka.gui. AttributeSummaryPanel 
 Tests out the attribute summary panel from the command line.
   main(String[])   - 
Static method in class weka.gui. AttributeVisualizationPanel 
 Main method to test this class from command line
   main(String[])   - 
Static method in class weka.gui.beans. AttributeSummarizer 
  
   main(String[])   - 
Static method in class weka.gui.beans. CostBenefitAnalysis 
  
   main(String[])   - 
Static method in class weka.gui.beans. DataVisualizer 
  
   main(String[])   - 
Static method in class weka.gui.beans. FlowRunner 
 Main method for testing this class.
   main(String[])   - 
Static method in class weka.gui.beans. KnowledgeFlow 
 Shows the splash screen, launches the application and then disposes
 the splash screen.
   main(String[])   - 
Static method in class weka.gui.beans. KnowledgeFlowApp 
 Main method.
   main(String[])   - 
Static method in class weka.gui.beans. Loader 
  
   main(String[])   - 
Static method in class weka.gui.beans. LogPanel 
 Main method to test this class.
   main(String[])   - 
Static method in class weka.gui.beans. ModelPerformanceChart 
  
   main(String[])   - 
Static method in class weka.gui.beans. Saver 
 The main method for testing
   main(String[])   - 
Static method in class weka.gui.beans. ScatterPlotMatrix 
  
   main(String[])   - 
Static method in class weka.gui.beans. StripChart 
 Tests out the StripChart from the command line
   main(String[])   - 
Static method in class weka.gui.beans. TextViewer 
  
   main(String[])   - 
Static method in class weka.gui.boundaryvisualizer. BoundaryPanel 
 Main method for testing this class
   main(String[])   - 
Static method in class weka.gui.boundaryvisualizer. BoundaryPanelDistributed 
 Main method for testing this class
   main(String[])   - 
Static method in class weka.gui.boundaryvisualizer. BoundaryVisualizer 
 Main method for testing this class
   main(String[])   - 
Static method in class weka.gui. ConverterFileChooser 
 For testing the file chooser
   main(String[])   - 
Static method in class weka.gui. DatabaseConnectionDialog 
 for testing only
   main(String[])   - 
Static method in class weka.gui.experiment. AlgorithmListPanel 
 Tests out the algorithm list panel from the command line.
   main(String[])   - 
Static method in class weka.gui.experiment. DatasetListPanel 
 Tests out the dataset list panel from the command line.
   main(String[])   - 
Static method in class weka.gui.experiment. DistributeExperimentPanel 
 Tests out the panel from the command line.
   main(String[])   - 
Static method in class weka.gui.experiment. Experimenter 
 Tests out the experiment environment.
   main(String[])   - 
Static method in class weka.gui.experiment. ExperimenterDefaults 
 only for testing - prints the content of the props file
   main(String[])   - 
Static method in class weka.gui.experiment. GeneratorPropertyIteratorPanel 
 Tests out the panel from the command line.
   main(String[])   - 
Static method in class weka.gui.experiment. HostListPanel 
 Tests out the host list panel from the command line.
   main(String[])   - 
Static method in class weka.gui.experiment. OutputFormatDialog 
 for testing only.
   main(String[])   - 
Static method in class weka.gui.experiment. ResultsPanel 
 Tests out the results panel from the command line.
   main(String[])   - 
Static method in class weka.gui.experiment. RunNumberPanel 
 Tests out the panel from the command line.
   main(String[])   - 
Static method in class weka.gui.experiment. RunPanel 
 Tests out the run panel from the command line.
   main(String[])   - 
Static method in class weka.gui.experiment. SetupPanel 
 Tests out the experiment setup from the command line.
   main(String[])   - 
Static method in class weka.gui.explorer. AssociationsPanel 
 Tests out the Associator panel from the command line.
   main(String[])   - 
Static method in class weka.gui.explorer. AttributeSelectionPanel 
 Tests out the attribute selection panel from the command line.
   main(String[])   - 
Static method in class weka.gui.explorer. ClassifierPanel 
 Tests out the classifier panel from the command line.
   main(String[])   - 
Static method in class weka.gui.explorer. ClustererPanel 
 Tests out the clusterer panel from the command line.
   main(String[])   - 
Static method in class weka.gui.explorer. Explorer 
 Tests out the explorer environment.
   main(String[])   - 
Static method in class weka.gui.explorer. ExplorerDefaults 
 only for testing - prints the content of the props file.
   main(String[])   - 
Static method in class weka.gui.explorer. PreprocessPanel 
 Tests out the instance-preprocessing panel from the command line.
   main(String[])   - 
Static method in class weka.gui.explorer. VisualizePanel 
 Tests out the visualize panel from the command line.
   main(String[])   - 
Static method in class weka.gui. GenericArrayEditor 
 Tests out the array editor from the command line.
   main(String[])   - 
Static method in class weka.gui. GenericObjectEditor 
 Tests out the Object editor from the command line.
   main(String[])   - 
Static method in class weka.gui. GenericPropertiesCreator 
 for generating props file:
 
   
     no parameter: 
     see default constructor
   
   
     1 parameter (i.e., filename): 
     see default constructor + setOutputFilename(String)
   
   
     2 parameters (i.e, filenames): 
     see constructor with String argument + setOutputFilename(String)
   
 
   main(String[])   - 
Static method in class weka.gui.graphvisualizer. GraphVisualizer 
 Main method to load a text file with the
 description of a graph from the command
 line
   main(String[])   - 
Static method in class weka.gui. GUIChooser 
 Tests out the GUIChooser environment.
   main(String[])   - 
Static method in class weka.gui. HierarchyPropertyParser 
 Tests out the parser.
   main(String[])   - 
Static method in class weka.gui.hierarchyvisualizer. HierarchyVisualizer 
 Main method for testing this class.
   main(String[])   - 
Static method in class weka.gui. InstancesSummaryPanel 
 Tests out the instance summary panel from the command line.
   main(String[])   - 
Static method in class weka.gui. ListSelectorDialog 
 Tests out the list selector from the command line.
   main(String[])   - 
Static method in class weka.gui. LogPanel 
 Tests out the log panel from the command line.
   main(String[])   - 
Static method in class weka.gui. LogWindow 
 for testing only
   main(String[])   - 
Static method in class weka.gui. LookAndFeel 
 prints all the available LnFs to stdout
   Main   - Class in  weka.gui  Menu-based GUI for Weka, replacement for the GUIChooser.   Main()   - 
Constructor for class weka.gui. Main 
 default constructor.
   main(String[])   - 
Static method in class weka.gui. Main 
 starts the application.
   main(String[])   - 
Static method in class weka.gui. PropertySelectorDialog 
 Tests out the property selector from the command line.
   main(String[])   - 
Static method in class weka.gui. ResultHistoryPanel 
 Tests out the result history from the command line.
   main(String[])   - 
Static method in class weka.gui. SaveBuffer 
 Main method for testing this class
   main(String[])   - 
Static method in class weka.gui. SelectedTagEditor 
 Tests out the selectedtag editor from the command line.
   main(String[])   - 
Static method in class weka.gui. SimpleCLI 
 Method to start up the simple cli
   main(String[])   - 
Static method in class weka.gui. SimpleCLIPanel 
 Main method for testing this class.
   main(String[])   - 
Static method in class weka.gui.sql. SqlViewer 
 starts the SQL-Viewer interface.
   main(String[])   - 
Static method in class weka.gui.sql. SqlViewerDialog 
 for testing only
   main(String[])   - 
Static method in class weka.gui.treevisualizer. TreeVisualizer 
 Main method for testing this class.
   main(String[])   - 
Static method in class weka.gui.visualize. AttributePanel 
 Main method for testing this class.
   main(String[])   - 
Static method in class weka.gui.visualize. BMPWriter 
 for testing only
   main(String[])   - 
Static method in class weka.gui.visualize. ClassPanel 
 Main method for testing this class.
   main(String[])   - 
Static method in class weka.gui.visualize. JPEGWriter 
 for testing only.
   main(String[])   - 
Static method in class weka.gui.visualize. LegendPanel 
 Main method for testing this class
   main(String[])   - 
Static method in class weka.gui.visualize. MatrixPanel 
 Main method for testing this class
   main(String[])   - 
Static method in class weka.gui.visualize. Plot2D 
 Main method for testing this class
   main(String[])   - 
Static method in class weka.gui.visualize. PNGWriter 
 for testing only
   main(String[])   - 
Static method in class weka.gui.visualize. PostscriptWriter 
 for testing only
   main(String[])   - 
Static method in class weka.gui.visualize. ThresholdVisualizePanel 
 Starts the ThresholdVisualizationPanel with parameters from the command line.
   main(String[])   - 
Static method in class weka.gui.visualize. VisualizePanel 
 Main method for testing this class
   main(String[])   - 
Static method in class weka.gui. WekaTaskMonitor 
 Main method for testing this class
   Main.BackgroundDesktopPane   - Class in  weka.gui  DesktopPane with background image.   Main.BackgroundDesktopPane(String)   - 
Constructor for class weka.gui. Main.BackgroundDesktopPane 
 intializes the desktop pane.
   Main.ChildFrameMDI   - Class in  weka.gui  Specialized JInternalFrame class.   Main.ChildFrameMDI(Main, String)   - 
Constructor for class weka.gui. Main.ChildFrameMDI 
 constructs a new internal frame that knows about its parent.
   Main.ChildFrameSDI   - Class in  weka.gui  Specialized JFrame class.   Main.ChildFrameSDI(Main, String)   - 
Constructor for class weka.gui. Main.ChildFrameSDI 
 constructs a new internal frame that knows about its parent.
   MainMenuExtension   - Interface in  weka.gui  Classes implementing this interface will be displayed in the "Extensions"
 menu in the main GUI of Weka.   MAJOR   - 
Static variable in class weka.core. Version 
 the major version
   MAJORITY_VOTING_RULE   - 
Static variable in class weka.classifiers.meta. Vote 
 combination rule: Majority Voting (only nominal classes)
   majorityClassTipText()   - 
Method in class weka.classifiers.rules. Ridor 
 Returns the tip text for this property
   makeADTree(int, FastVector, Instances)   - 
Static method in class weka.classifiers.bayes.net. ADNode 
 create sub tree
   makeADTree(Instances)   - 
Static method in class weka.classifiers.bayes.net. ADNode 
 create AD tree from set of instances
   makeBinaryTipText()   - 
Method in class weka.filters.supervised.attribute. Discretize 
 Returns the tip text for this property
   makeBinaryTipText()   - 
Method in class weka.filters.unsupervised.attribute. Discretize 
 Returns the tip text for this property
   makeCopies(Associator, int)   - 
Static method in class weka.associations. AbstractAssociator 
 Creates copies of the current associator.
   makeCopies(ASEvaluation, int)   - 
Static method in class weka.attributeSelection. ASEvaluation 
 Creates copies of the current evaluator.
   makeCopies(ASSearch, int)   - 
Static method in class weka.attributeSelection. ASSearch 
 Creates copies of the current search scheme.
   makeCopies(Classifier, int)   - 
Static method in class weka.classifiers. Classifier 
 Creates a given number of deep copies of the given classifier using serialization.
   makeCopies(Kernel, int)   - 
Static method in class weka.classifiers.functions.supportVector. Kernel 
 Creates a given number of deep copies of the given kernel using 
 serialization.
   makeCopies(Clusterer, int)   - 
Static method in class weka.clusterers. AbstractClusterer 
 Creates copies of the current clusterer.
   makeCopies(DensityBasedClusterer, int)   - 
Static method in class weka.clusterers. AbstractDensityBasedClusterer 
 Creates copies of the current clusterer.
   makeCopies(Estimator, int)   - 
Static method in class weka.estimators. Estimator 
 Creates a given number of deep copies of the given estimator using serialization.
   makeCopies(Filter, int)   - 
Static method in class weka.filters. Filter 
 Creates a given number of deep copies of the given filter using 
 serialization.
   makeCopy(Associator)   - 
Static method in class weka.associations. AbstractAssociator 
 Creates a deep copy of the given associator using serialization.
   makeCopy(Classifier)   - 
Static method in class weka.classifiers. Classifier 
 Creates a deep copy of the given classifier using serialization.
   makeCopy(Kernel)   - 
Static method in class weka.classifiers.functions.supportVector. Kernel 
 Creates a deep copy of the given kernel using serialization.
   makeCopy(Clusterer)   - 
Static method in class weka.clusterers. AbstractClusterer 
 Creates a deep copy of the given clusterer using serialization.
   makeCopy(Estimator)   - 
Static method in class weka.estimators. Estimator 
 Creates a deep copy of the given estimator using serialization.
   makeCopy(Filter)   - 
Static method in class weka.filters. Filter 
 Creates a deep copy of the given filter using serialization.
   makeCopy(Object)   - 
Static method in class weka.gui. GenericArrayEditor 
 Makes a copy of an object using serialization.
   makeCopy(Object)   - 
Static method in class weka.gui. GenericObjectEditor 
 Makes a copy of an object using serialization.
   makeData(DataGenerator, String[])   - 
Static method in class weka.datagenerators. DataGenerator 
 Calls the data generator.
   MakeDecList   - Class in  weka.classifiers.rules.part  Class for handling a decision list.   MakeDecList(ModelSelection, int)   - 
Constructor for class weka.classifiers.rules.part. MakeDecList 
 Constructor for unpruned dec list.
   MakeDecList(ModelSelection, double, int)   - 
Constructor for class weka.classifiers.rules.part. MakeDecList 
 Constructor for dec list pruned using C4.5 pruning.
   MakeDecList(ModelSelection, int, int, int)   - 
Constructor for class weka.classifiers.rules.part. MakeDecList 
 Constructor for dec list pruned using hold-out pruning.
   MakeDensityBasedClusterer   - Class in  weka.clusterers  Class for wrapping a Clusterer to make it return a distribution and density.   MakeDensityBasedClusterer()   - 
Constructor for class weka.clusterers. MakeDensityBasedClusterer 
 Default constructor.
   MakeDensityBasedClusterer(Clusterer)   - 
Constructor for class weka.clusterers. MakeDensityBasedClusterer 
 Contructs a MakeDensityBasedClusterer wrapping a given Clusterer.
   makeDistribution(double, int)   - 
Static method in class weka.classifiers.evaluation. NominalPrediction 
 Convert a single prediction into a probability distribution
 with all zero probabilities except the predicted value which
 has probability 1.0.
   MakeIndicator   - Class in  weka.filters.unsupervised.attribute  A filter that creates a new dataset with a boolean attribute replacing a nominal attribute.   MakeIndicator()   - 
Constructor for class weka.filters.unsupervised.attribute. MakeIndicator 
 Constructor
   makeUniformDistribution(int)   - 
Static method in class weka.classifiers.evaluation. NominalPrediction 
 Creates a uniform probability distribution -- where each of the
 possible classes is assigned equal probability.
   makeVaryNode(int, FastVector, Instances)   - 
Static method in class weka.classifiers.bayes.net. ADNode 
 create sub tree
   makeWeighted(CostMatrix)   - 
Method in class weka.classifiers.evaluation. ConfusionMatrix 
 Makes a copy of this ConfusionMatrix after applying the
 supplied CostMatrix to the cells.
   ManhattanDataObject   - Class in  weka.clusterers.forOPTICSAndDBScan.DataObjects  
 ManhattanDataObject.java  
 Authors: Rainer Holzmann, Zhanna Melnikova-Albrecht, Matthias Schubert  
 Date: Aug 19, 2004  
 Time: 5:50:22 PM  
 $ Revision 1.4 $     ManhattanDataObject(Instance, String, Database)   - 
Constructor for class weka.clusterers.forOPTICSAndDBScan.DataObjects. ManhattanDataObject 
 Constructs a new DataObject.
   ManhattanDistance   - Class in  weka.core  Implements the Manhattan distance (or Taxicab geometry).   ManhattanDistance()   - 
Constructor for class weka.core. ManhattanDistance 
 Constructs an Manhattan Distance object, Instances must be still set.
   ManhattanDistance(Instances)   - 
Constructor for class weka.core. ManhattanDistance 
 Constructs an Manhattan Distance object and automatically initializes the
 ranges.
   manualThresholdValueTipText()   - 
Method in class weka.classifiers.meta. ThresholdSelector 
  
   map(String, String)   - 
Method in class weka.core.matrix. DoubleVector 
 Applies a method to the vector
   mapClasses(int, int, int[][], int[], double[], double[], int)   - 
Static method in class weka.clusterers. ClusterEvaluation 
 Finds the minimum error mapping of classes to clusters.
   MappingInfo   - Class in  weka.core.pmml  Class that maintains the mapping between incoming data set structure
 and that of the mining schema.   MappingInfo(Instances, MiningSchema, Logger)   - 
Constructor for class weka.core.pmml. MappingInfo 
  
   mapToMiningSchema(Instances)   - 
Method in class weka.classifiers.pmml.consumer. PMMLClassifier 
 Map mining schema to incoming instances.
   margin()   - 
Method in class weka.classifiers.evaluation. NominalPrediction 
 Calculates the prediction margin.
   MarginCalculator   - Class in  weka.classifiers.bayes.net      MarginCalculator()   - 
Constructor for class weka.classifiers.bayes.net. MarginCalculator 
  
   MarginCalculator.JunctionTreeNode   - Class in  weka.classifiers.bayes.net      MarginCalculator.JunctionTreeSeparator   - Class in  weka.classifiers.bayes.net      MarginCurve   - Class in  weka.classifiers.evaluation  Generates points illustrating the prediction margin.   MarginCurve()   - 
Constructor for class weka.classifiers.evaluation. MarginCurve 
  
   markovBlanketClassifierTipText()   - 
Method in class weka.classifiers.bayes.net.search.global. GlobalScoreSearchAlgorithm 
  
   markovBlanketClassifierTipText()   - 
Method in class weka.classifiers.bayes.net.search.local. LocalScoreSearchAlgorithm 
  
   maskKeyword(String)   - 
Method in class weka.experiment. DatabaseUtils 
 If the given string is a keyword, then the mask character will be 
 appended and returned.
   Matchable   - Interface in  weka.core  Interface to something that can be matched with tree matching
 algorithms.   matchMissingValuesTipText()   - 
Method in class weka.filters.unsupervised.instance. RemoveWithValues 
 Returns the tip text for this property
   MathematicalExpression   - Class in  weka.core  Class for evaluating a string adhering the following grammar:    MathematicalExpression()   - 
Constructor for class weka.core. MathematicalExpression 
  
   MathExpression   - Class in  weka.filters.unsupervised.attribute  Modify numeric attributes according to a given expression
  
  
 
  
 Valid options are:     MathExpression()   - 
Constructor for class weka.filters.unsupervised.attribute. MathExpression 
 Constructor
   Maths   - Class in  weka.core.matrix  Utility class.   Maths()   - 
Constructor for class weka.core.matrix. Maths 
  
   matrix()   - 
Method in class weka.classifiers.trees.j48. Distribution 
 Returns matrix with distribution of class values.
   Matrix   - Class in  weka.core   Deprecated.   Use  weka.core.matrix.Matrix  instead - only for
 backwards compatibility.    Matrix(int, int)   - 
Constructor for class weka.core. Matrix 
  Deprecated.  Constructs a matrix and initializes it with default values.
   Matrix(double[][])   - 
Constructor for class weka.core. Matrix 
  Deprecated.  Constructs a matrix using a given array.
   Matrix(Reader)   - 
Constructor for class weka.core. Matrix 
  Deprecated.  Reads a matrix from a reader.
   Matrix   - Class in  weka.core.matrix  Jama = Java Matrix class.   Matrix(int, int)   - 
Constructor for class weka.core.matrix. Matrix 
 Construct an m-by-n matrix of zeros.
   Matrix(int, int, double)   - 
Constructor for class weka.core.matrix. Matrix 
 Construct an m-by-n constant matrix.
   Matrix(double[][])   - 
Constructor for class weka.core.matrix. Matrix 
 Construct a matrix from a 2-D array.
   Matrix(double[][], int, int)   - 
Constructor for class weka.core.matrix. Matrix 
 Construct a matrix quickly without checking arguments.
   Matrix(double[], int)   - 
Constructor for class weka.core.matrix. Matrix 
 Construct a matrix from a one-dimensional packed array
   Matrix(Reader)   - 
Constructor for class weka.core.matrix. Matrix 
 Reads a matrix from a reader.
   MATRIX_ON_DEMAND   - 
Static variable in class weka.attributeSelection. CostSensitiveASEvaluation 
 load cost matrix on demand
   MATRIX_ON_DEMAND   - 
Static variable in class weka.classifiers.meta. CostSensitiveClassifier 
 load cost matrix on demand
   MATRIX_ON_DEMAND   - 
Static variable in class weka.classifiers.meta. MetaCost 
 load cost matrix on demand
   MATRIX_SUPPLIED   - 
Static variable in class weka.attributeSelection. CostSensitiveASEvaluation 
 use explicit cost matrix
   MATRIX_SUPPLIED   - 
Static variable in class weka.classifiers.meta. CostSensitiveClassifier 
 use explicit cost matrix
   MATRIX_SUPPLIED   - 
Static variable in class weka.classifiers.meta. MetaCost 
 use explicit matrix
   MatrixPanel   - Class in  weka.gui.visualize  This panel displays a plot matrix of the user selected attributes
 of a given data set.   MatrixPanel()   - 
Constructor for class weka.gui.visualize. MatrixPanel 
 Constructor
   max()   - 
Method in class weka.core.matrix. DoubleVector 
 Returns the maximum value of all elements
   MAX   - 
Static variable in class weka.core.neighboursearch. KDTree 
 The index of MAX value in attributes' range array.
   MAX   - 
Static variable in class weka.core.neighboursearch.kdtrees. KDTreeNodeSplitter 
 Index of max value in an array of attributes' range.
   max   - 
Variable in class weka.experiment. Stats 
 The maximum value seen, or Double.NaN if no values seen
   MAX_DIGITS   - 
Static variable in class weka.core.converters. SVMLightSaver 
 the number of digits after the decimal point.
   MAX_ROWS   - 
Static variable in class weka.gui.sql. QueryPanel 
 the name for the max rows in the history.
   MAX_RULE   - 
Static variable in class weka.classifiers.meta. Vote 
 combination rule: Maximum Probability
   MAX_SHAPES   - 
Static variable in class weka.gui.visualize. Plot2D 
  
   maxAbs()   - 
Method in class weka.classifiers.functions.pace. PaceMatrix 
 Returns the maximum absolute value of all elements
   maxAbs(int, int, int)   - 
Method in class weka.classifiers.functions.pace. PaceMatrix 
 Returns the maximum absolute value of some elements of a column,
      that is, the elements of A[i0:i1][j].
   maxBag()   - 
Method in class weka.classifiers.trees.j48. Distribution 
 Returns index of bag containing maximum number of instances.
   maxBoostingIterationsTipText()   - 
Method in class weka.classifiers.functions. SimpleLogistic 
 Returns the tip text for this property
   maxCardinalityTipText()   - 
Method in class weka.classifiers.bayes.net.search.ci. ICSSearchAlgorithm 
  
   maxCardinalityTipText()   - 
Method in class weka.filters.unsupervised.attribute. RELAGGS 
 Returns the tip text for this property
   maxChunkSizeTipText()   - 
Method in class weka.classifiers.meta. RacedIncrementalLogitBoost 
  
   maxClass()   - 
Method in class weka.classifiers.trees.j48. Distribution 
 Returns class with highest frequency over all bags.
   maxClass(int)   - 
Method in class weka.classifiers.trees.j48. Distribution 
 Returns class with highest frequency for given bag.
   maxClassForSubsetOfInterest()   - 
Method in class weka.classifiers.trees.j48. GraftSplit 
  
   maxCountTipText()   - 
Method in class weka.filters.supervised.instance. SpreadSubsample 
 Returns the tip text for this property
   maxDefaultTipText()   - 
Method in class weka.filters.unsupervised.attribute. NumericCleaner 
 Returns the tip text for this property
   maxDepthTipText()   - 
Method in class weka.classifiers.trees. RandomForest 
 Returns the tip text for this property
   maxDepthTipText()   - 
Method in class weka.classifiers.trees. RandomTree 
 Returns the tip text for this property
   maxDepthTipText()   - 
Method in class weka.classifiers.trees. REPTree 
 Returns the tip text for this property
   maxGenerationsTipText()   - 
Method in class weka.attributeSelection. GeneticSearch 
 Returns the tip text for this property
   maxGridExtensionsTipText()   - 
Method in class weka.classifiers.meta. GridSearch 
 Returns the tip text for this property
   maxGroupTipText()   - 
Method in class weka.classifiers.meta. RotationForest 
 Returns the tip text for this property
   maxImpurity()   - 
Method in class weka.classifiers.trees.m5. CorrelationSplitInfo 
 Returns the impurity of this split
   maxImpurity()   - 
Method in interface weka.classifiers.trees.m5. SplitEvaluate 
 Returns the impurity of this split
   maxImpurity()   - 
Method in class weka.classifiers.trees.m5. YongSplitInfo 
 Returns the impurity of this split
   maximumAttributeNamesTipText()   - 
Method in class weka.attributeSelection. LatentSemanticAnalysis 
 Returns the tip text for this property
   maximumAttributeNamesTipText()   - 
Method in class weka.attributeSelection. PrincipalComponents 
 Returns the tip text for this property
   maximumAttributeNamesTipText()   - 
Method in class weka.filters.unsupervised.attribute. PrincipalComponents 
 Returns the tip text for this property.
   maximumAttributesTipText()   - 
Method in class weka.filters.unsupervised.attribute. PrincipalComponents 
 Returns the tip text for this property.
   maximumVariancePercentageAllowedTipText()   - 
Method in class weka.filters.unsupervised.attribute. RemoveUseless 
 Returns the tip text for this property
   maxIndex(double[])   - 
Static method in class weka.core. Utils 
 Returns index of maximum element in a given
 array of doubles.
   maxIndex(int[])   - 
Static method in class weka.core. Utils 
 Returns index of maximum element in a given
 array of integers.
   maxInstancesInLeafTipText()   - 
Method in class weka.core.neighboursearch.balltrees. BallTreeConstructor 
 Returns the tip text for this property.
   maxInstInLeafTipText()   - 
Method in class weka.core.neighboursearch. KDTree 
 Tip text for this property.
   maxInstNumTipText()   - 
Method in class weka.datagenerators.clusterers. BIRCHCluster 
 Returns the tip text for this property
   maxInstNumTipText()   - 
Method in class weka.datagenerators.clusterers. SubspaceClusterDefinition 
 Returns the tip text for this property
   maxIterations   - 
Variable in class weka.classifiers.bayes. BayesianLogisticRegression 
 Maximum number of iterations
   maxIterationsTipText()   - 
Method in class weka.classifiers.bayes. BayesianLogisticRegression 
 Returns the tip text for this property
   maxIterationsTipText()   - 
Method in class weka.classifiers.mi. MIBoost 
 Returns the tip text for this property
   maxIterationsTipText()   - 
Method in class weka.classifiers.mi. MISVM 
 Returns the tip text for this property
   maxIterationsTipText()   - 
Method in class weka.clusterers. EM 
 Returns the tip text for this property
   maxIterationsTipText()   - 
Method in class weka.clusterers. sIB 
 Returns the tip text for this property.
   maxIterationsTipText()   - 
Method in class weka.clusterers. SimpleKMeans 
 Returns the tip text for this property
   maxIterationsTipText()   - 
Method in class weka.clusterers. XMeans 
 Returns the tip text for this property.
   maxIterationsTipText()   - 
Method in class weka.filters.unsupervised.instance. RemoveMisclassified 
 Returns the tip text for this property
   maxItsTipText()   - 
Method in class weka.classifiers.functions. Logistic 
 Returns the tip text for this property
   maxItsTipText()   - 
Method in class weka.classifiers.functions. RBFNetwork 
 Returns the tip text for this property
   maxKMeansForChildrenTipText()   - 
Method in class weka.clusterers. XMeans 
 Returns the tip text for this property.
   maxKMeansTipText()   - 
Method in class weka.clusterers. XMeans 
 Returns the tip text for this property.
   maxKTipText()   - 
Method in class weka.classifiers.functions. VotedPerceptron 
 Returns the tip text for this property
   maxNrOfParentsTipText()   - 
Method in class weka.classifiers.bayes.net.search. SearchAlgorithm 
  
   maxNumberOfItemsTipText()   - 
Method in class weka.associations. FPGrowth 
 Tip text for this property suitable for displaying
 in the GUI.
   maxNumClustersTipText()   - 
Method in class weka.clusterers. XMeans 
 Returns the tip text for this property.
   maxParentSetSize(int)   - 
Method in class weka.classifiers.bayes.net. ParentSet 
 reserve memory for parent set
   maxRadiusTipText()   - 
Method in class weka.datagenerators.clusterers. BIRCHCluster 
 Returns the tip text for this property
   maxRangeTipText()   - 
Method in class weka.datagenerators.classifiers.regression. MexicanHat 
 Returns the tip text for this property
   maxRelativeLeafRadiusTipText()   - 
Method in class weka.core.neighboursearch.balltrees. BallTreeConstructor 
 Returns the tip text for this property.
   maxRuleSizeTipText()   - 
Method in class weka.datagenerators.classifiers.classification. RDG1 
 Returns the tip text for this property
   maxSubsequenceLengthTipText()   - 
Method in class weka.classifiers.functions.supportVector. StringKernel 
 Returns the tip text for this property
   maxThresholdTipText()   - 
Method in class weka.filters.unsupervised.attribute. NumericCleaner 
 Returns the tip text for this property
   MAYBE_SUPPORT   - 
Static variable in class weka.gui. GenericObjectEditor.GOETreeNode 
 color for "maybe support".
   MDD   - Class in  weka.classifiers.mi  Modified Diverse Density algorithm, with collective assumption. 
  
 More information about DD: 
  
 Oded Maron (1998).   MDD()   - 
Constructor for class weka.classifiers.mi. MDD 
  
   MDL   - 
Static variable in interface weka.classifiers.bayes.net.search.local. Scoreable 
  
   mean(double[])   - 
Static method in class weka.core. Utils 
 Computes the mean for an array of doubles.
   mean   - 
Variable in class weka.experiment. Stats 
 The mean of values at the last calculateDerived() call
   meanAbsoluteError()   - 
Method in class weka.classifiers. Evaluation 
 Returns the mean absolute error.
   meanOrMode(int)   - 
Method in class weka.core. Instances 
 Returns the mean (mode) for a numeric (nominal) attribute as
 a floating-point value.
   meanOrMode(Attribute)   - 
Method in class weka.core. Instances 
 Returns the mean (mode) for a numeric (nominal) attribute as a
 floating-point value.
   meanPriorAbsoluteError()   - 
Method in class weka.classifiers. Evaluation 
 Returns the mean absolute error of the prior.
   meanSquaredTipText()   - 
Method in class weka.classifiers.lazy. IBk 
 Returns the tip text for this property.
   meanStddevTipText()   - 
Method in class weka.datagenerators.clusterers. SubspaceClusterDefinition 
 Returns the tip text for this property
   measureAICScore()   - 
Method in class weka.classifiers.bayes. BayesNet 
  
   measureAttributesUsed()   - 
Method in class weka.classifiers.functions. SimpleLogistic 
 Returns the fraction of all attributes in the data that are used in the 
 logistic model (in percent).
   measureBayesScore()   - 
Method in class weka.classifiers.bayes. BayesNet 
  
   measureBDeuScore()   - 
Method in class weka.classifiers.bayes. BayesNet 
  
   measureDivergence()   - 
Method in class weka.classifiers.bayes. BayesNet 
  
   measureEntropyScore()   - 
Method in class weka.classifiers.bayes. BayesNet 
  
   measureExamplesCounted()   - 
Method in class weka.classifiers.trees. LADTree 
 Returns the number of examples "counted".
   measureExamplesProcessed()   - 
Method in class weka.classifiers.trees. ADTree 
 Returns the number of examples "counted".
   measureExtraArcs()   - 
Method in class weka.classifiers.bayes. BayesNet 
  
   measureMaxDepth()   - 
Method in class weka.core.neighboursearch. BallTree 
 Returns the depth of the tree.
   measureMaxDepth()   - 
Method in class weka.core.neighboursearch. CoverTree 
 Returns the depth of the tree.
   measureMaxDepth()   - 
Method in class weka.core.neighboursearch. KDTree 
 Returns the depth of the tree.
   measureMDLScore()   - 
Method in class weka.classifiers.bayes. BayesNet 
  
   measureMissingArcs()   - 
Method in class weka.classifiers.bayes. BayesNet 
  
   measureNodesExpanded()   - 
Method in class weka.classifiers.trees. ADTree 
 Returns the number of nodes expanded.
   measureNodesExpanded()   - 
Method in class weka.classifiers.trees. LADTree 
 Returns the number of nodes expanded.
   measureNumAttributesSelected()   - 
Method in class weka.classifiers.meta. AttributeSelectedClassifier 
 Additional measure --- number of attributes selected
   measureNumIterations()   - 
Method in class weka.classifiers.meta. AdditiveRegression 
 return the number of iterations (base classifiers) completed
   measureNumLeaves()   - 
Method in class weka.classifiers.trees. ADTree 
 Calls measure function for leaf size - the number of prediction nodes.
   measureNumLeaves()   - 
Method in class weka.classifiers.trees. FT 
 Returns the number of leaves in the tree
   measureNumLeaves()   - 
Method in class weka.classifiers.trees. J48 
 Returns the number of leaves
   measureNumLeaves()   - 
Method in class weka.classifiers.trees. J48graft 
 Returns the number of leaves
   measureNumLeaves()   - 
Method in class weka.classifiers.trees. LADTree 
 Calls measure function for leaf size.
   measureNumLeaves()   - 
Method in class weka.classifiers.trees. LMT 
 Returns the number of leaves in the tree
   measureNumLeaves()   - 
Method in class weka.classifiers.trees. NBTree 
 Returns the number of leaves
   measureNumLeaves()   - 
Method in class weka.core.neighboursearch. BallTree 
 Returns the number of leaves.
   measureNumLeaves()   - 
Method in class weka.core.neighboursearch. CoverTree 
 Returns the number of leaves.
   measureNumLeaves()   - 
Method in class weka.core.neighboursearch. KDTree 
 Returns the number of leaves.
   measureNumPredictionLeaves()   - 
Method in class weka.classifiers.trees. ADTree 
 Calls measure function for prediction leaf size - the number of 
 prediction nodes without children.
   measureNumPredictionLeaves()   - 
Method in class weka.classifiers.trees. LADTree 
 Calls measure function for leaf size.
   measureNumRules()   - 
Method in class weka.classifiers.rules. DecisionTable 
 Returns the number of rules
   measureNumRules()   - 
Method in class weka.classifiers.rules. PART 
 Return the number of rules.
   measureNumRules()   - 
Method in class weka.classifiers.trees. J48 
 Returns the number of rules (same as number of leaves)
   measureNumRules()   - 
Method in class weka.classifiers.trees. J48graft 
 Returns the number of rules (same as number of leaves)
   measureNumRules()   - 
Method in class weka.classifiers.trees.m5. M5Base 
 return the number of rules
   measureNumRules()   - 
Method in class weka.classifiers.trees. NBTree 
 Returns the number of rules (same as number of leaves)
   measureOutOfBagError()   - 
Method in class weka.classifiers.meta. Bagging 
 Gets the out of bag error that was calculated as the classifier
 was built.
   measureOutOfBagError()   - 
Method in class weka.classifiers.trees. RandomForest 
 Gets the out of bag error that was calculated as the classifier was built.
   measurePercentAttsUsedByDT()   - 
Method in class weka.classifiers.rules. DTNB 
 Returns the number of rules
   measurePerformanceTipText()   - 
Method in class weka.core.neighboursearch. NearestNeighbourSearch 
 Returns the tip text for this property.
   measureReversedArcs()   - 
Method in class weka.classifiers.bayes. BayesNet 
  
   measureSelectionTime()   - 
Method in class weka.classifiers.meta. AttributeSelectedClassifier 
 Additional measure --- time taken (milliseconds) to select the attributes
   measureTime()   - 
Method in class weka.classifiers.meta. AttributeSelectedClassifier 
 Additional measure --- time taken (milliseconds) to select attributes
 and build the classifier
   measureTipText()   - 
Method in class weka.classifiers.meta. ThresholdSelector 
 Tooltip for this property.
   measureTreeSize()   - 
Method in class weka.classifiers.trees. ADTree 
 Calls measure function for tree size - the total number of nodes.
   measureTreeSize()   - 
Method in class weka.classifiers.trees. BFTree 
 Return number of tree size.
   measureTreeSize()   - 
Method in class weka.classifiers.trees. FT 
 Returns the size of the tree
   measureTreeSize()   - 
Method in class weka.classifiers.trees. J48 
 Returns the size of the tree
   measureTreeSize()   - 
Method in class weka.classifiers.trees. J48graft 
 Returns the size of the tree
   measureTreeSize()   - 
Method in class weka.classifiers.trees. LADTree 
 Calls measure function for tree size.
   measureTreeSize()   - 
Method in class weka.classifiers.trees. LMT 
 Returns the size of the tree
   measureTreeSize()   - 
Method in class weka.classifiers.trees. NBTree 
 Returns the size of the tree
   measureTreeSize()   - 
Method in class weka.classifiers.trees. SimpleCart 
 Return number of tree size.
   measureTreeSize()   - 
Method in class weka.core.neighboursearch. BallTree 
 Returns the size of the tree.
   measureTreeSize()   - 
Method in class weka.core.neighboursearch. CoverTree 
 Returns the size of the tree.
   measureTreeSize()   - 
Method in class weka.core.neighboursearch. KDTree 
 Returns the size of the tree.
   MEDIAN_RULE   - 
Static variable in class weka.classifiers.meta. Vote 
 combination rule: Median Probability (only numeric class)
   MedianDistanceFromArbitraryPoint   - Class in  weka.core.neighboursearch.balltrees  Class that splits a BallNode of a ball tree using Uhlmann's described method. 
  
 For information see: 
  
 Jeffrey K.   MedianDistanceFromArbitraryPoint()   - 
Constructor for class weka.core.neighboursearch.balltrees. MedianDistanceFromArbitraryPoint 
 Constructor.
   MedianDistanceFromArbitraryPoint(int[], Instances, EuclideanDistance)   - 
Constructor for class weka.core.neighboursearch.balltrees. MedianDistanceFromArbitraryPoint 
 Constructor.
   MedianOfWidestDimension   - Class in  weka.core.neighboursearch.balltrees  Class that splits a BallNode of a ball tree based on the median value of the widest dimension of the points in the ball.   MedianOfWidestDimension()   - 
Constructor for class weka.core.neighboursearch.balltrees. MedianOfWidestDimension 
 Constructor.
   MedianOfWidestDimension(int[], Instances, EuclideanDistance)   - 
Constructor for class weka.core.neighboursearch.balltrees. MedianOfWidestDimension 
 Constructor.
   MedianOfWidestDimension   - Class in  weka.core.neighboursearch.kdtrees  The class that splits a KDTree node based on the median value of a dimension in which the node's points have the widest spread. 
  
 For more information see also: 
  
 Jerome H.   MedianOfWidestDimension()   - 
Constructor for class weka.core.neighboursearch.kdtrees. MedianOfWidestDimension 
  
   Memory   - Class in  weka.core  A little helper class for Memory management.   Memory()   - 
Constructor for class weka.core. Memory 
 initializes the memory management without GUI support
   Memory(boolean)   - 
Constructor for class weka.core. Memory 
 initializes the memory management
   MemoryUsagePanel   - Class in  weka.gui  A panel for displaying the memory usage.   MemoryUsagePanel()   - 
Constructor for class weka.gui. MemoryUsagePanel 
 default constructor.
   merge(Element, Element)   - 
Static method in class weka.associations.gsp. Element 
 Merges two Elements into one.
   merge(SimpleLinkedList, Comparator)   - 
Method in class weka.associations.tertius. SimpleLinkedList 
  
   merge(ADTree)   - 
Method in class weka.classifiers.trees. ADTree 
 Merges two trees together.
   merge(PredictionNode, ADTree)   - 
Method in class weka.classifiers.trees.adtree. PredictionNode 
 Merges this node with another.
   merge(LADTree)   - 
Method in class weka.classifiers.trees. LADTree 
 Merges two trees together.
   mergeAllItemSets(FastVector, int, int)   - 
Static method in class weka.associations. AprioriItemSet 
 Merges all item sets in the set of (k-1)-item sets 
 to create the (k)-item sets and updates the counters.
   mergeAllItemSets(FastVector, int, int)   - 
Static method in class weka.associations. ItemSet 
 Merges all item sets in the set of (k-1)-item sets
 to create the (k)-item sets and updates the counters.
   mergeAllItemSets(FastVector, int, int)   - 
Static method in class weka.associations. LabeledItemSet 
 Merges all item sets in the set of (k-1)-item sets
 to create the (k)-item sets and updates the counters.
   mergeInstance(Instance)   - 
Method in class weka.core. BinarySparseInstance 
 Merges this instance with the given instance and returns
 the result.
   mergeInstance(Instance)   - 
Method in class weka.core. Instance 
 Merges this instance with the given instance and returns
 the result.
   mergeInstance(Instance)   - 
Method in class weka.core. SparseInstance 
 Merges this instance with the given instance and returns
 the result.
   mergeInstances(Instances, Instances)   - 
Static method in class weka.core. Instances 
 Merges two sets of Instances together.
   MergeTwoValues   - Class in  weka.filters.unsupervised.attribute  Merges two values of a nominal attribute into one value.   MergeTwoValues()   - 
Constructor for class weka.filters.unsupervised.attribute. MergeTwoValues 
  
   Messages   - Class in  weka.associations.gsp  Messages.   Messages()   - 
Constructor for class weka.associations.gsp. Messages 
  
   Messages   - Class in  weka.associations  Messages.   Messages()   - 
Constructor for class weka.associations. Messages 
  
   Messages   - Class in  weka.gui.arffviewer  Messages.   Messages()   - 
Constructor for class weka.gui.arffviewer. Messages 
  
   Messages   - Class in  weka.gui.beans  Messages.   Messages()   - 
Constructor for class weka.gui.beans. Messages 
  
   Messages   - Class in  weka.gui.beans.xml  Messages.   Messages()   - 
Constructor for class weka.gui.beans.xml. Messages 
  
   Messages   - Class in  weka.gui.boundaryvisualizer  Messages.   Messages()   - 
Constructor for class weka.gui.boundaryvisualizer. Messages 
  
   Messages   - Class in  weka.gui.experiment  Messages.   Messages()   - 
Constructor for class weka.gui.experiment. Messages 
  
   Messages   - Class in  weka.gui.explorer  Messages.   Messages()   - 
Constructor for class weka.gui.explorer. Messages 
  
   Messages   - Class in  weka.gui.graphvisualizer  Messages.   Messages()   - 
Constructor for class weka.gui.graphvisualizer. Messages 
  
   Messages   - Class in  weka.gui.hierarchyvisualizer  Messages.   Messages()   - 
Constructor for class weka.gui.hierarchyvisualizer. Messages 
  
   Messages   - Class in  weka.gui  Messages.   Messages()   - 
Constructor for class weka.gui. Messages 
  
   Messages   - Class in  weka.gui.sql.event  Messages.   Messages()   - 
Constructor for class weka.gui.sql.event. Messages 
  
   Messages   - Class in  weka.gui.sql  Messages.   Messages()   - 
Constructor for class weka.gui.sql. Messages 
  
   Messages   - Class in  weka.gui.streams  Messages.   Messages()   - 
Constructor for class weka.gui.streams. Messages 
  
   Messages   - Class in  weka.gui.treevisualizer  Messages.   Messages()   - 
Constructor for class weka.gui.treevisualizer. Messages 
  
   Messages   - Class in  weka.gui.visualize  Messages.   Messages()   - 
Constructor for class weka.gui.visualize. Messages 
  
   MEstimate(double, double, double)   - 
Method in class weka.classifiers.bayes. AODEsr 
 Returns the probability estimate, using m-estimate
   mestWeightTipText()   - 
Method in class weka.classifiers.bayes. AODEsr 
 Returns the tip text for this property
   MetaBean   - Class in  weka.gui.beans  A meta bean that encapsulates several other regular beans, useful for 
 grouping large KnowledgeFlows.   MetaBean()   - 
Constructor for class weka.gui.beans. MetaBean 
  
   metaClassifierTipText()   - 
Method in class weka.classifiers.meta. Stacking 
 Returns the tip text for this property
   MetaCost   - Class in  weka.classifiers.meta  This metaclassifier makes its base classifier cost-sensitive using the method specified in 
  
 Pedro Domingos: MetaCost: A general method for making classifiers cost-sensitive.   MetaCost()   - 
Constructor for class weka.classifiers.meta. MetaCost 
  
   METHOD_1_AGAINST_1   - 
Static variable in class weka.classifiers.meta. MultiClassClassifier 
 1-against-1
   METHOD_1_AGAINST_ALL   - 
Static variable in class weka.classifiers.meta. MultiClassClassifier 
 1-against-all
   METHOD_ERROR_EXHAUSTIVE   - 
Static variable in class weka.classifiers.meta. MultiClassClassifier 
 exhaustive correction code
   METHOD_ERROR_RANDOM   - 
Static variable in class weka.classifiers.meta. MultiClassClassifier 
 random correction code
   MethodHandler   - Class in  weka.core.xml  This class handles relationships between display names of properties 
 (or classes) and Methods that are associated with them.   MethodHandler()   - 
Constructor for class weka.core.xml. MethodHandler 
 initializes the handler
   methodNameTipText()   - 
Method in class weka.filters.unsupervised.attribute. NumericTransform 
 Returns the tip text for this property
   methodTipText()   - 
Method in class weka.classifiers.meta. MultiClassClassifier 
  
   methodTipText()   - 
Method in class weka.classifiers.mi. MIWrapper 
 Returns the tip text for this property
   metricString()   - 
Method in class weka.associations. Apriori 
 Returns the metric string for the chosen metric type
   metricString()   - 
Method in interface weka.associations. CARuleMiner 
 Gets name of the scoring metric used for car mining
   metricString()   - 
Method in class weka.associations. PredictiveApriori 
 Returns the metric string for the chosen metric type.
   metricTypeTipText()   - 
Method in class weka.associations. Apriori 
 Returns the tip text for this property
   metricTypeTipText()   - 
Method in class weka.associations. FPGrowth 
 Tip text for this property suitable for displaying
 in the GUI.
   MexicanHat   - Class in  weka.datagenerators.classifiers.regression  A data generator for the simple 'Mexian Hat' function: 
    y = sin|x| / |x| 
 In addition to this simple function, the amplitude can be changed and gaussian noise can be added.   MexicanHat()   - 
Constructor for class weka.datagenerators.classifiers.regression. MexicanHat 
 initializes the generator
   MIBoost   - Class in  weka.classifiers.mi  MI AdaBoost method, considers the geometric mean of posterior of instances inside a bag (arithmatic mean of log-posterior) and the expectation for a bag is taken inside the loss function. 
  
 For more information about Adaboost, see: 
  
 Yoav Freund, Robert E.   MIBoost()   - 
Constructor for class weka.classifiers.mi. MIBoost 
  
   MIDD   - Class in  weka.classifiers.mi  Re-implement the Diverse Density algorithm, changes the testing procedure. 
  
 Oded Maron (1998).   MIDD()   - 
Constructor for class weka.classifiers.mi. MIDD 
  
   MiddleOutConstructor   - Class in  weka.core.neighboursearch.balltrees  The class that builds a BallTree middle out. 
  
 For more information see also: 
  
 Andrew W.   MiddleOutConstructor()   - 
Constructor for class weka.core.neighboursearch.balltrees. MiddleOutConstructor 
 Creates a new instance of MiddleOutConstructor.
   midPoint(double, int)   - 
Method in class weka.associations. PriorEstimation 
 calculates the mid point of an interval
   MidPointOfWidestDimension   - Class in  weka.core.neighboursearch.kdtrees  The class that splits a KDTree node based on the midpoint value of a dimension in which the node's points have the widest spread. 
  
 For more information see also: 
  
 Andrew Moore (1991).   MidPointOfWidestDimension()   - 
Constructor for class weka.core.neighboursearch.kdtrees. MidPointOfWidestDimension 
  
   midPoints()   - 
Method in class weka.associations. PriorEstimation 
 split the interval [0,1] into a predefined number of intervals and calculates their mid points
   MIEMDD   - Class in  weka.classifiers.mi  EMDD model builds heavily upon Dietterich's Diverse Density (DD) algorithm. 
 It is a general framework for MI learning of converting the MI problem to a single-instance setting using EM.   MIEMDD()   - 
Constructor for class weka.classifiers.mi. MIEMDD 
  
   MILR   - Class in  weka.classifiers.mi  Uses either standard or collective multi-instance assumption, but within linear regression.   MILR()   - 
Constructor for class weka.classifiers.mi. MILR 
  
   MIN   - 
Static variable in class weka.core.neighboursearch. KDTree 
 The index of MIN value in attributes' range array.
   MIN   - 
Static variable in class weka.core.neighboursearch.kdtrees. KDTreeNodeSplitter 
 Index of min value in an array of attributes' range.
   min   - 
Variable in class weka.experiment. Stats 
 The minimum value seen, or Double.NaN if no values seen
   MIN_RULE   - 
Static variable in class weka.classifiers.meta. Vote 
 combination rule: Minimum Probability
   minAbs(int, int, int)   - 
Method in class weka.classifiers.functions.pace. PaceMatrix 
 Returns the minimum absolute value of some elements of a column,
      that is, the elements of A[i0:i1][j].
   minBagDistance(Instance, Instance)   - 
Method in class weka.classifiers.mi. MIOptimalBall 
 Calculate the distance from one data point to a bag
   minBoxRelWidthTipText()   - 
Method in class weka.core.neighboursearch. KDTree 
 Tip text for this property.
   minBucketSizeTipText()   - 
Method in class weka.classifiers.rules. OneR 
 Returns the tip text for this property
   minChangeTipText()   - 
Method in class weka.clusterers. sIB 
 Returns the tip text for this property.
   minChunkSizeTipText()   - 
Method in class weka.classifiers.meta. RacedIncrementalLogitBoost 
  
   minDataDLIfDeleted(int, double, boolean)   - 
Method in class weka.classifiers.rules. RuleStats 
 Compute the minimal data description length of the ruleset
 if the rule in the given position is deleted. 
 The min_data_DL_if_deleted = data_DL_if_deleted - potential
   minDataDLIfExists(int, double, boolean)   - 
Method in class weka.classifiers.rules. RuleStats 
 Compute the minimal data description length of the ruleset
 if the rule in the given position is NOT deleted. 
 The min_data_DL_if_n_deleted = data_DL_if_n_deleted - potential
   minDefaultTipText()   - 
Method in class weka.filters.unsupervised.attribute. NumericCleaner 
 Returns the tip text for this property
   mineCARs(Instances)   - 
Method in class weka.associations. Apriori 
 Method that mines all class association rules with minimum support and
 with a minimum confidence.
   mineCARs(Instances)   - 
Method in interface weka.associations. CARuleMiner 
 Method for mining class association rules.
   mineCARs(Instances)   - 
Method in class weka.associations. PredictiveApriori 
 Method that mines the n best class association rules.
   minGroupTipText()   - 
Method in class weka.classifiers.meta. RotationForest 
 Returns the tip text for this property
   minimax(Instances, int)   - 
Static method in class weka.classifiers.mi. SimpleMI 
 Get the minimal and maximal value of a certain attribute in a certain data
   minimaxTipText()   - 
Method in class weka.classifiers.mi. MISMO 
 Returns the tip text for this property
   minimizeExpectedCostTipText()   - 
Method in class weka.classifiers.meta. CostSensitiveClassifier 
  
   minimizeWindows()   - 
Method in class weka.gui. Main 
 minimizes all windows.
   minimumBucketSizeTipText()   - 
Method in class weka.attributeSelection. OneRAttributeEval 
 Returns a string for this option suitable for display in the gui
 as a tip text
   minIndex(int[])   - 
Static method in class weka.core. Utils 
 Returns index of minimum element in a given
 array of integers.
   minIndex(double[])   - 
Static method in class weka.core. Utils 
 Returns index of minimum element in a given
 array of doubles.
   MiningFieldMetaInfo   - Class in  weka.core.pmml  Class encapsulating information about a MiningField.   MiningFieldMetaInfo(Element)   - 
Constructor for class weka.core.pmml. MiningFieldMetaInfo 
 Constructs a new MiningFieldMetaInfo object.
   MiningSchema   - Class in  weka.core.pmml  This class encapsulates the mining schema from
 a PMML xml file.   MiningSchema(Element, Instances, TransformationDictionary)   - 
Constructor for class weka.core.pmml. MiningSchema 
 Constructor for MiningSchema.
   minInstNumTipText()   - 
Method in class weka.datagenerators.clusterers. BIRCHCluster 
 Returns the tip text for this property
   minInstNumTipText()   - 
Method in class weka.datagenerators.clusterers. SubspaceClusterDefinition 
 Returns the tip text for this property
   minMetricTipText()   - 
Method in class weka.associations. Apriori 
 Returns the tip text for this property
   minMetricTipText()   - 
Method in class weka.associations. FPGrowth 
 Returns the tip text for this property
   MINND   - Class in  weka.classifiers.mi  Multiple-Instance Nearest Neighbour with Distribution learner. 
  
 It uses gradient descent to find the weight for each dimension of each exeamplar from the starting point of 1.0.   MINND()   - 
Constructor for class weka.classifiers.mi. MINND 
  
   minNoTipText()   - 
Method in class weka.classifiers.rules. ConjunctiveRule 
 Returns the tip text for this property
   minNoTipText()   - 
Method in class weka.classifiers.rules. JRip 
 Returns the tip text for this property
   minNoTipText()   - 
Method in class weka.classifiers.rules. Ridor 
 Returns the tip text for this property
   minNumClustersTipText()   - 
Method in class weka.clusterers. XMeans 
 Returns the tip text for this property.
   minNumInstancesTipText()   - 
Method in class weka.classifiers.trees. FT 
 Returns the tip text for this property
   minNumInstancesTipText()   - 
Method in class weka.classifiers.trees. LMT 
 Returns the tip text for this property
   minNumInstancesTipText()   - 
Method in class weka.classifiers.trees.m5. M5Base 
 Returns the tip text for this property
   minNumObjTipText()   - 
Method in class weka.classifiers.rules. PART 
 Returns the tip text for this property
   minNumObjTipText()   - 
Method in class weka.classifiers.trees. BFTree 
 Returns the tip text for this property
   minNumObjTipText()   - 
Method in class weka.classifiers.trees. J48 
 Returns the tip text for this property
   minNumObjTipText()   - 
Method in class weka.classifiers.trees. J48graft 
 Returns the tip text for this property
   minNumObjTipText()   - 
Method in class weka.classifiers.trees. SimpleCart 
 Returns the tip text for this property
   minNumTipText()   - 
Method in class weka.classifiers.trees. RandomTree 
 Returns the tip text for this property
   minNumTipText()   - 
Method in class weka.classifiers.trees. REPTree 
 Returns the tip text for this property
   MINOR   - 
Static variable in class weka.core. Version 
 the minor version
   minPointsTipText()   - 
Method in class weka.clusterers. DBScan 
 Returns the tip text for this property
   minPointsTipText()   - 
Method in class weka.clusterers. OPTICS 
 Returns the tip text for this property
   minProb   - 
Variable in class weka.classifiers.lazy.kstar. KStarWrapper 
 used/reused to hold the smallest transformation probability
   minRadiusTipText()   - 
Method in class weka.datagenerators.clusterers. BIRCHCluster 
 Returns the tip text for this property
   minRangeTipText()   - 
Method in class weka.datagenerators.classifiers.regression. MexicanHat 
 Returns the tip text for this property
   minRuleSizeTipText()   - 
Method in class weka.datagenerators.classifiers.classification. RDG1 
 Returns the tip text for this property
   minsAndMaxs(Instances, double[][], int)   - 
Method in class weka.classifiers.trees.j48. C45Split 
 Returns the minsAndMaxs of the index.th subset.
   minStdDevTipText()   - 
Method in class weka.classifiers.functions. RBFNetwork 
 Returns the tip text for this property
   minStdDevTipText()   - 
Method in class weka.clusterers. EM 
 Returns the tip text for this property
   minStdDevTipText()   - 
Method in class weka.clusterers. MakeDensityBasedClusterer 
 Returns the tip text for this property
   minSupportTipText()   - 
Method in class weka.associations. GeneralizedSequentialPatterns 
 Returns the minimum support option tip text for the Weka GUI.
   minTermFreqTipText()   - 
Method in class weka.filters.unsupervised.attribute. StringToWordVector 
 Returns the tip text for this property.
   minThresholdTipText()   - 
Method in class weka.filters.unsupervised.attribute. NumericCleaner 
 Returns the tip text for this property
   MINUS   - 
Static variable in interface weka.core.mathematicalexpression. sym 
  
   minus(double)   - 
Method in class weka.core.matrix. DoubleVector 
 Subtracts a value
   minus(DoubleVector)   - 
Method in class weka.core.matrix. DoubleVector 
 Subtracts another DoubleVector element by element
   minus(Matrix)   - 
Method in class weka.core.matrix. Matrix 
 C = A - B
   MINUS   - 
Static variable in interface weka.filters.unsupervised.instance.subsetbyexpression. sym 
  
   minusEquals(double)   - 
Method in class weka.core.matrix. DoubleVector 
 Subtracts a value in place
   minusEquals(DoubleVector)   - 
Method in class weka.core.matrix. DoubleVector 
 Subtracts another DoubleVector element by element in place
   minusEquals(Matrix)   - 
Method in class weka.core.matrix. Matrix 
 A = A - B
   minVariancePropTipText()   - 
Method in class weka.classifiers.trees. REPTree 
 Returns the tip text for this property
   MIOptimalBall   - Class in  weka.classifiers.mi  This classifier tries to find a suitable ball in the multiple-instance space, with a certain data point in the instance space as a ball center.   MIOptimalBall()   - 
Constructor for class weka.classifiers.mi. MIOptimalBall 
  
   MIPolyKernel   - Class in  weka.classifiers.mi.supportVector  The polynomial kernel : K(x, y) = <x, y>^p or K(x, y) = (<x, y>+1)^p
  
  
 
  
 Valid options are:     MIPolyKernel()   - 
Constructor for class weka.classifiers.mi.supportVector. MIPolyKernel 
 default constructor - does nothing.
   MIPolyKernel(Instances, int, double, boolean)   - 
Constructor for class weka.classifiers.mi.supportVector. MIPolyKernel 
 Creates a new  MIPolyKernel  instance.
   MIRBFKernel   - Class in  weka.classifiers.mi.supportVector  The RBF kernel.   MIRBFKernel()   - 
Constructor for class weka.classifiers.mi.supportVector. MIRBFKernel 
 default constructor - does nothing.
   MIRBFKernel(Instances, int, double)   - 
Constructor for class weka.classifiers.mi.supportVector. MIRBFKernel 
 Constructor.
   MISMO   - Class in  weka.classifiers.mi  Implements John Platt's sequential minimal optimization algorithm for training a support vector classifier. 
  
 This implementation globally replaces all missing values and transforms nominal attributes into binary ones.   MISMO()   - 
Constructor for class weka.classifiers.mi. MISMO 
  
   MISSING_SHAPE   - 
Static variable in class weka.gui.visualize. Plot2D 
  
   MISSING_VALUE   - 
Static variable in interface weka.classifiers.evaluation. Prediction 
 Constant representing a missing value.
   missingArcs(BayesNet)   - 
Method in class weka.classifiers.bayes.net. BIFReader 
 Count nr of arcs missing from other network compared to current network
 Note that an arc is not 'missing' if it is reversed.
   missingCount   - 
Variable in class weka.core. AttributeStats 
 The number of missing values
   missingMergeTipText()   - 
Method in class weka.attributeSelection. ChiSquaredAttributeEval 
 Returns the tip text for this property
   missingMergeTipText()   - 
Method in class weka.attributeSelection. GainRatioAttributeEval 
 Returns the tip text for this property
   missingMergeTipText()   - 
Method in class weka.attributeSelection. InfoGainAttributeEval 
 Returns the tip text for this property
   missingMergeTipText()   - 
Method in class weka.attributeSelection. SymmetricalUncertAttributeEval 
 Returns the tip text for this property
   missingModeTipText()   - 
Method in class weka.classifiers.lazy. KStar 
 Returns the tip text for this property
   missingSeparateTipText()   - 
Method in class weka.attributeSelection. CfsSubsetEval 
 Returns the tip text for this property
   missingValue()   - 
Static method in class weka.core. Instance 
 Returns the double that codes "missing".
   missingValuesTipText()   - 
Method in class weka.associations. Tertius 
 Returns the tip text for this property.
   missingValueTipText()   - 
Method in class weka.core.converters. CSVLoader 
 Returns the tip text for this property.
   MISVM   - Class in  weka.classifiers.mi  Implements Stuart Andrews' mi_SVM (Maximum pattern Margin Formulation of MIL).   MISVM()   - 
Constructor for class weka.classifiers.mi. MISVM 
  
   MIWrapper   - Class in  weka.classifiers.mi  A simple Wrapper method for applying standard propositional learners to multi-instance data. 
  
 For more information see: 
  
 E.   MIWrapper()   - 
Constructor for class weka.classifiers.mi. MIWrapper 
  
   MixtureDistribution   - Class in  weka.classifiers.functions.pace  Abtract class for manipulating mixture distributions.   MixtureDistribution()   - 
Constructor for class weka.classifiers.functions.pace. MixtureDistribution 
  
   MODEL_FILE_EXTENSION   - 
Static variable in class weka.gui.explorer. ClassifierPanel 
 The filename extension that should be used for model files
   MODEL_FILE_EXTENSION   - 
Static variable in class weka.gui.explorer. ClustererPanel 
 The filename extension that should be used for model files
   MODEL_FT   - 
Static variable in class weka.classifiers.trees. FT 
 model types
   MODEL_FTInner   - 
Static variable in class weka.classifiers.trees. FT 
  
   MODEL_FTLeaves   - 
Static variable in class weka.classifiers.trees. FT 
  
   modelBuilt()   - 
Method in class weka.classifiers.functions.supportVector. RegOptimizer 
 flag to indicate whether the model was built yet
   modelDistributionForInstance(Instance)   - 
Method in class weka.classifiers.trees.ft. FTtree 
 Returns the class probabilities for an instance according to the logistic model at the node.
   modelDistributionForInstance(Instance)   - 
Method in class weka.classifiers.trees.lmt. LMTNode 
 Returns the class probabilities for an instance according to the logistic model at the node.
   modelErrors()   - 
Method in class weka.classifiers.trees.lmt. LMTNode 
 Updates the numIncorrectModel field for all nodes.
   modelErrors()   - 
Method in class weka.classifiers.trees. SimpleCart 
 Updates the numIncorrectModel field for all nodes when subtree (to be 
 pruned) is rooted.
   modelFileTipText()   - 
Method in class weka.classifiers.misc. SerializedClassifier 
 Returns the tip text for this property
   ModelPerformanceChart   - Class in  weka.gui.beans  Bean that can be used for displaying threshold curves (e.g.   ModelPerformanceChart()   - 
Constructor for class weka.gui.beans. ModelPerformanceChart 
  
   ModelPerformanceChartBeanInfo   - Class in  weka.gui.beans  Bean info class for the model performance chart   ModelPerformanceChartBeanInfo()   - 
Constructor for class weka.gui.beans. ModelPerformanceChartBeanInfo 
  
   ModelSelection   - Class in  weka.classifiers.trees.j48  Abstract class for model selection criteria.   ModelSelection()   - 
Constructor for class weka.classifiers.trees.j48. ModelSelection 
  
   modelsToString()   - 
Method in class weka.classifiers.trees.ft. FTtree 
 Returns a string describing the logistic regression function at the node.
   modelsToString()   - 
Method in class weka.classifiers.trees.lmt. LMTNode 
 Returns a string describing the logistic regression function at the node.
   modelTypeTipText()   - 
Method in class weka.classifiers.trees. FT 
 Returns the tip text for this property
   modifyHeaderTipText()   - 
Method in class weka.filters.unsupervised.instance. RemoveFrequentValues 
 Returns the tip text for this property
   modifyHeaderTipText()   - 
Method in class weka.filters.unsupervised.instance. RemoveWithValues 
 Returns the tip text for this property
   momentumTipText()   - 
Method in class weka.classifiers.functions. MultilayerPerceptron 
  
   moralize(BayesNet)   - 
Method in class weka.classifiers.bayes.net. MarginCalculator 
 moralize DAG and calculate
 adjacency matrix representation for a Bayes Network, effecively
 converting the directed acyclic graph to an undirected graph.
   mostExplainingColumn(PaceMatrix, IntVector, int)   - 
Method in class weka.classifiers.functions.pace. PaceMatrix 
 Returns the index of the column that has the largest (squared)
  response, when each of columns pvt[ks:] is moved to become the
  ks-th column.
   mouseClicked(MouseEvent)   - 
Method in class weka.gui.arffviewer. ArffPanel 
 Invoked when a mouse button has been pressed and released on a component
   mouseClicked(MouseEvent)   - 
Method in class weka.gui.treevisualizer. TreeVisualizer 
 Does nothing.
   mouseDragged(MouseEvent)   - 
Method in class weka.gui.treevisualizer. TreeVisualizer 
 Performs intermediate updates to what the user wishes to do.
   mouseEntered(MouseEvent)   - 
Method in class weka.gui.arffviewer. ArffPanel 
 Invoked when the mouse enters a component.
   mouseEntered(MouseEvent)   - 
Method in class weka.gui.treevisualizer. TreeVisualizer 
 Does nothing.
   mouseExited(MouseEvent)   - 
Method in class weka.gui.arffviewer. ArffPanel 
 Invoked when the mouse exits a component
   mouseExited(MouseEvent)   - 
Method in class weka.gui.treevisualizer. TreeVisualizer 
 Does nothing.
   mouseMoved(MouseEvent)   - 
Method in class weka.gui.treevisualizer. TreeVisualizer 
 Does nothing.
   mousePressed(MouseEvent)   - 
Method in class weka.gui.arffviewer. ArffPanel 
 Invoked when a mouse button has been pressed on a component
   mousePressed(MouseEvent)   - 
Method in class weka.gui.treevisualizer. TreeVisualizer 
 Determines what action the user wants to perform.
   mouseReleased(MouseEvent)   - 
Method in class weka.gui.arffviewer. ArffPanel 
 Invoked when a mouse button has been released on a component.
   mouseReleased(MouseEvent)   - 
Method in class weka.gui.treevisualizer. TreeVisualizer 
 Performs the final stages of what the user wants to perform.
   MOVE_DOWN   - 
Static variable in class weka.gui. JListHelper 
 moves items down
   MOVE_UP   - 
Static variable in class weka.gui. JListHelper 
 moves items up
   moveBottom(JList)   - 
Static method in class weka.gui. JListHelper 
 moves the selected items to the end
   moveDown(JList)   - 
Static method in class weka.gui. JListHelper 
 moves the selected item down by 1
   MoveInstanceToBestCluster(Instance)   - 
Method in class weka.clusterers. CLOPE 
 Move instance to best cluster
   moveTop(JList)   - 
Static method in class weka.gui. JListHelper 
 moves the selected items to the top
   moveUp(JList)   - 
Static method in class weka.gui. JListHelper 
 moves the selected items up by 1
   MultiBoostAB   - Class in  weka.classifiers.meta  Class for boosting a classifier using the MultiBoosting method. 
  
 MultiBoosting is an extension to the highly successful AdaBoost technique for forming decision committees.   MultiBoostAB()   - 
Constructor for class weka.classifiers.meta. MultiBoostAB 
  
   MultiClassClassifier   - Class in  weka.classifiers.meta  A metaclassifier for handling multi-class datasets with 2-class classifiers.   MultiClassClassifier()   - 
Constructor for class weka.classifiers.meta. MultiClassClassifier 
 Constructor.
   MultiFilter   - Class in  weka.filters  Applies several filters successively.   MultiFilter()   - 
Constructor for class weka.filters. MultiFilter 
  
   MultiInstanceCapabilitiesHandler   - Interface in  weka.core  Multi-Instance classifiers can specify an additional Capabilities object
 for the data in the relational attribute, since the format of multi-instance
 data is fixed to "bag/NOMINAL,data/RELATIONAL,class".   MultiInstanceToPropositional   - Class in  weka.filters.unsupervised.attribute  Converts the multi-instance dataset into single instance dataset so that the Nominalize, Standardize and other type of filters or transformation  can be applied to these data for the further preprocessing. 
 Note: the first attribute of the converted dataset is a nominal attribute and refers to the bagId.   MultiInstanceToPropositional()   - 
Constructor for class weka.filters.unsupervised.attribute. MultiInstanceToPropositional 
  
   MultilayerPerceptron   - Class in  weka.classifiers.functions  A Classifier that uses backpropagation to classify instances. 
 This network can be built by hand, created by an algorithm or both.   MultilayerPerceptron()   - 
Constructor for class weka.classifiers.functions. MultilayerPerceptron 
 The constructor.
   MultiNomialBMAEstimator   - Class in  weka.classifiers.bayes.net.estimate  Multinomial BMA Estimator.   MultiNomialBMAEstimator()   - 
Constructor for class weka.classifiers.bayes.net.estimate. MultiNomialBMAEstimator 
  
   multinomialWordTipText()   - 
Method in class weka.classifiers.bayes. DMNBtext 
 Returns the tip text for this property
   MultipleClassifiersCombiner   - Class in  weka.classifiers  Abstract utility class for handling settings common to
 meta classifiers that build an ensemble from multiple classifiers.   MultipleClassifiersCombiner()   - 
Constructor for class weka.classifiers. MultipleClassifiersCombiner 
  
   multiply(Matrix)   - 
Method in class weka.core. Matrix 
  Deprecated.  Returns the multiplication of two matrices
   multiResultsetFull(int, int)   - 
Method in class weka.experiment. PairedTTester 
 Creates a comparison table where a base resultset is compared to the
 other resultsets.
   multiResultsetFull(int, int)   - 
Method in interface weka.experiment. Tester 
 Creates a comparison table where a base resultset is compared to the
 other resultsets.
   multiResultsetRanking(int)   - 
Method in class weka.experiment. PairedTTester 
 returns a ranking of the resultsets
   multiResultsetRanking(int)   - 
Method in interface weka.experiment. Tester 
  
   multiResultsetSummary(int)   - 
Method in class weka.experiment. PairedTTester 
 Carries out a comparison between all resultsets, counting the number
 of datsets where one resultset outperforms the other.
   multiResultsetSummary(int)   - 
Method in interface weka.experiment. Tester 
 Carries out a comparison between all resultsets, counting the number
 of datsets where one resultset outperforms the other.
   multiResultsetWins(int, int[][])   - 
Method in class weka.experiment. PairedTTester 
 Carries out a comparison between all resultsets, counting the number
 of datsets where one resultset outperforms the other.
   multiResultsetWins(int, int[][])   - 
Method in interface weka.experiment. Tester 
 Carries out a comparison between all resultsets, counting the number
 of datsets where one resultset outperforms the other.
   MultiScheme   - Class in  weka.classifiers.meta  Class for selecting a classifier from among several using cross validation on the training data or the performance on the training data.   MultiScheme()   - 
Constructor for class weka.classifiers.meta. MultiScheme 
  
   mutationProbTipText()   - 
Method in class weka.attributeSelection. GeneticSearch 
 Returns the tip text for this property
 
 
    
 N  
 
   NaiveBayes   - Class in  weka.classifiers.bayes  Class for a Naive Bayes classifier using estimator classes.   NaiveBayes()   - 
Constructor for class weka.classifiers.bayes. NaiveBayes 
  
   NaiveBayes   - Class in  weka.classifiers.bayes.net.search.fixed  The NaiveBayes class generates a fixed Bayes network structure with arrows from the class variable to each of the attribute variables.   NaiveBayes()   - 
Constructor for class weka.classifiers.bayes.net.search.fixed. NaiveBayes 
  
   NaiveBayesMultinomial   - Class in  weka.classifiers.bayes  Class for building and using a multinomial Naive Bayes classifier.   NaiveBayesMultinomial()   - 
Constructor for class weka.classifiers.bayes. NaiveBayesMultinomial 
  
   NaiveBayesMultinomialUpdateable   - Class in  weka.classifiers.bayes  Class for building and using a multinomial Naive Bayes classifier.   NaiveBayesMultinomialUpdateable()   - 
Constructor for class weka.classifiers.bayes. NaiveBayesMultinomialUpdateable 
  
   NaiveBayesSimple   - Class in  weka.classifiers.bayes  Class for building and using a simple Naive Bayes classifier.Numeric attributes are modelled by a normal distribution. 
  
 For more information, see 
  
 Richard Duda, Peter Hart (1973).   NaiveBayesSimple()   - 
Constructor for class weka.classifiers.bayes. NaiveBayesSimple 
  
   NaiveBayesUpdateable   - Class in  weka.classifiers.bayes  Class for a Naive Bayes classifier using estimator classes.   NaiveBayesUpdateable()   - 
Constructor for class weka.classifiers.bayes. NaiveBayesUpdateable 
  
   name()   - 
Method in class weka.core. Attribute 
 Returns the attribute's name.
   name()   - 
Method in class weka.core. Option 
 Returns the option's name.
   NAME_CLASSFIRST   - 
Static variable in class weka.experiment.xml. XMLExperiment 
 the name of the classFirst property
   NAME_PROPERTYNODE_PARENTCLASS   - 
Static variable in class weka.experiment.xml. XMLExperiment 
 PropertyNode member
   NAME_PROPERTYNODE_PROPERTY   - 
Static variable in class weka.experiment.xml. XMLExperiment 
 PropertyNode member
   NAME_PROPERTYNODE_VALUE   - 
Static variable in class weka.experiment.xml. XMLExperiment 
 PropertyNode member
   NamedColor   - Class in  weka.gui.treevisualizer  This class contains a color name and the rgb values of that color   NamedColor(String, int, int, int)   - 
Constructor for class weka.gui.treevisualizer. NamedColor 
  
   nameTipText()   - 
Method in class weka.filters.unsupervised.attribute. AddExpression 
 Returns the tip text for this property
   NBconditionalProb(Instance, int)   - 
Method in class weka.classifiers.bayes. AODE 
 Calculates the probability of the specified class for the given test
 instance, using naive Bayes.
   NBconditionalProb(Instance, int)   - 
Method in class weka.classifiers.bayes. AODEsr 
 Calculates the probability of the specified class for the given test
 instance, using naive Bayes.
   NBTree   - Class in  weka.classifiers.trees  Class for generating a decision tree with naive Bayes classifiers at the leaves. 
  
 For more information, see 
  
 Ron Kohavi: Scaling Up the Accuracy of Naive-Bayes Classifiers: A Decision-Tree Hybrid.   NBTree()   - 
Constructor for class weka.classifiers.trees. NBTree 
  
   NBTreeClassifierTree   - Class in  weka.classifiers.trees.j48  Class for handling a naive bayes tree structure used for
 classification.   NBTreeClassifierTree(ModelSelection)   - 
Constructor for class weka.classifiers.trees.j48. NBTreeClassifierTree 
  
   NBTreeModelSelection   - Class in  weka.classifiers.trees.j48  Class for selecting a NB tree split.   NBTreeModelSelection(int, Instances)   - 
Constructor for class weka.classifiers.trees.j48. NBTreeModelSelection 
 Initializes the split selection method with the given parameters.
   NBTreeNoSplit   - Class in  weka.classifiers.trees.j48  Class implementing a "no-split"-split (leaf node) for naive bayes
 trees.   NBTreeNoSplit()   - 
Constructor for class weka.classifiers.trees.j48. NBTreeNoSplit 
  
   NBTreeSplit   - Class in  weka.classifiers.trees.j48  Class implementing a NBTree split on an attribute.   NBTreeSplit(int, int, double)   - 
Constructor for class weka.classifiers.trees.j48. NBTreeSplit 
 Initializes the split model.
   ND   - Class in  weka.classifiers.meta.nestedDichotomies  A meta classifier for handling multi-class datasets with 2-class classifiers by building a random tree structure. 
  
 For more info, check 
  
 Lin Dong, Eibe Frank, Stefan Kramer: Ensembles of Balanced Nested Dichotomies for Multi-class Problems.   ND()   - 
Constructor for class weka.classifiers.meta.nestedDichotomies. ND 
 Constructor.
   NDConditionalEstimator   - Class in  weka.estimators  Conditional probability estimator for a numeric domain conditional upon
 a discrete domain (utilises separate normal estimators for each discrete
 conditioning value).   NDConditionalEstimator(int, double)   - 
Constructor for class weka.estimators. NDConditionalEstimator 
 Constructor
   nearestNeighborsTipText()   - 
Method in class weka.filters.supervised.instance. SMOTE 
 Returns the tip text for this property.
   nearestNeighbour(Instance)   - 
Method in class weka.core.neighboursearch. BallTree 
 Returns the nearest instance in the current neighbourhood to the supplied
 instance.
   nearestNeighbour(Instance)   - 
Method in class weka.core.neighboursearch. CoverTree 
 Returns the NN instance of a given target instance, from among
 the previously supplied training instances.
   nearestNeighbour(Instance)   - 
Method in class weka.core.neighboursearch. KDTree 
 Returns the nearest neighbour of the supplied target 
 instance.
   nearestNeighbour(Instance)   - 
Method in class weka.core.neighboursearch. LinearNNSearch 
 Returns the nearest instance in the current neighbourhood to the supplied
 instance.
   nearestNeighbour(Instance)   - 
Method in class weka.core.neighboursearch. NearestNeighbourSearch 
 Returns the nearest instance in the current neighbourhood to the supplied
 instance.
   NearestNeighbourSearch   - Class in  weka.core.neighboursearch  Abstract class for nearest neighbour search.   NearestNeighbourSearch()   - 
Constructor for class weka.core.neighboursearch. NearestNeighbourSearch 
 Constructor.
   NearestNeighbourSearch(Instances)   - 
Constructor for class weka.core.neighboursearch. NearestNeighbourSearch 
 Constructor.
   nearestNeighbourSearchAlgorithmTipText()   - 
Method in class weka.classifiers.lazy. IBk 
 Returns the tip text for this property.
   nearestNeighbourSearchAlgorithmTipText()   - 
Method in class weka.classifiers.lazy. LWL 
 Returns the tip text for this property.
   needExponentialFormat(double)   - 
Method in class weka.core.matrix. FlexibleDecimalFormat 
  
   needsUID(String)   - 
Static method in class weka.core. SerializationHelper 
 checks whether a class needs to declare a serialVersionUID, i.e., it
 implements the java.io.Serializable interface but doesn't declare a
 serialVersionUID.
   needsUID(Class)   - 
Static method in class weka.core. SerializationHelper 
 checks whether a class needs to declare a serialVersionUID, i.e., it
 implements the java.io.Serializable interface but doesn't declare a
 serialVersionUID.
   NEG   - 
Static variable in class weka.associations.tertius. Literal 
  
   negationIncludedIn(LiteralSet)   - 
Method in class weka.associations.tertius. LiteralSet 
 Test if the negation of this LiteralSet is included in another LiteralSet.
   negationSatisfies(Instance)   - 
Method in class weka.associations.tertius. AttributeValueLiteral 
  
   negationSatisfies(Instance)   - 
Method in class weka.associations.tertius. Literal 
  
   negationTipText()   - 
Method in class weka.associations. Tertius 
 Returns the tip text for this property.
   negative()   - 
Method in class weka.associations.tertius. Literal 
  
   nestedEstimate(DoubleVector)   - 
Method in class weka.classifiers.functions.pace. NormalMixture 
 Returns the optimal nested model estimate of a vector.
   NeuralConnection   - Class in  weka.classifiers.functions.neural  Abstract unit in a NeuralNetwork.   NeuralConnection(String)   - 
Constructor for class weka.classifiers.functions.neural. NeuralConnection 
 Constructs The unit with the basic connection information prepared for
 use.
   NeuralMethod   - Interface in  weka.classifiers.functions.neural  This is an interface used to create classes that can be used by the 
 neuralnode to perform all it's computations.   NeuralNetwork   - Class in  weka.classifiers.pmml.consumer  Class implementing import of PMML Neural Network model.   NeuralNetwork(Element, Instances, MiningSchema)   - 
Constructor for class weka.classifiers.pmml.consumer. NeuralNetwork 
  
   NeuralNode   - Class in  weka.classifiers.functions.neural  This class is used to represent a node in the neuralnet.   NeuralNode(String, Random, NeuralMethod)   - 
Constructor for class weka.classifiers.functions.neural. NeuralNode 
  
   NEW_BATCH   - 
Static variable in class weka.gui.beans. IncrementalClassifierEvent 
  
   newClock()   - 
Static method in class weka.core. Debug 
 returns a new instance of a clock
   newDataFormat(DataSetEvent)   - 
Method in class weka.gui.beans. ClassAssignerCustomizer 
  
   newDataFormat(DataSetEvent)   - 
Method in interface weka.gui.beans. DataFormatListener 
 Recieve a DataSetEvent that encapsulates a new data format.
   newDocument(String, String)   - 
Method in class weka.core.xml. XMLDocument 
 creates a new Document with the given information.
   newEnt(Distribution)   - 
Method in class weka.classifiers.trees.j48. EntropyBasedSplitCrit 
 Computes entropy of distribution after splitting.
   Newick   - 
Static variable in interface weka.core. Drawable 
  
   newInstance(File, Class)   - 
Static method in class weka.core. Jython 
 loads the module and returns a new instance of it as instance of the
 provided Java class template.
   newInstance(File, Class, File[])   - 
Static method in class weka.core. Jython 
 loads the module and returns a new instance of it as instance of the
 provided Java class template.
   newInterpreter()   - 
Static method in class weka.core. Jython 
 initializes and returns a Python Interpreter
   newLog(String, int, int)   - 
Static method in class weka.core. Debug 
 returns a new Log instance
   newNominalRule(Attribute, Instances, int[])   - 
Method in class weka.classifiers.rules. OneR 
 Create a rule branching on this nominal attribute.
   newNumericRule(Attribute, Instances, int[])   - 
Method in class weka.classifiers.rules. OneR 
 Create a rule branching on this numeric attribute
   newRandom()   - 
Static method in class weka.core. Debug 
 returns a default debug random object, with no particular seed and 
 debugging enabled.
   newRandom(int)   - 
Static method in class weka.core. Debug 
 returns a debug random object with the specified seed and debugging 
 enabled.
   newRule(Attribute, Instances)   - 
Method in class weka.classifiers.rules. OneR 
 Create a rule branching on this attribute.
   newTimestamp()   - 
Static method in class weka.core. Debug 
 returns a default timestamp for the current date/time
   next()   - 
Method in class weka.associations.tertius. SimpleLinkedList.LinkedListIterator 
  
   next(int)   - 
Method in interface weka.classifiers. IterativeClassifier 
 Performs one iteration.
   next   - 
Variable in class weka.classifiers.lazy.kstar. KStarCache.TableEntry 
 next table entry (separate chaining)
   next(int)   - 
Method in class weka.classifiers.trees. ADTree 
 Performs one iteration.
   next(int)   - 
Method in class weka.classifiers.trees. LADTree 
  
   next()   - 
Method in class weka.clusterers.forOPTICSAndDBScan.Utils. PriorityQueue 
 Returns the element with the highest priority
   next()   - 
Method in class weka.clusterers.forOPTICSAndDBScan.Utils. UpdateQueue 
 Returns the element with the lowest priority
   next()   - 
Method in class weka.core. Trie.TrieIterator 
 Returns the next element in the iteration.
   next_token()   - 
Method in class weka.core.mathematicalexpression. Scanner 
 Resumes scanning until the next regular expression is matched,
 the end of input is encountered or an I/O-Error occurs.
   next_token()   - 
Method in class weka.filters.unsupervised.instance.subsetbyexpression. Scanner 
 Resumes scanning until the next regular expression is matched,
 the end of input is encountered or an I/O-Error occurs.
   nextBoolean()   - 
Method in class weka.core. Debug.Random 
 Returns the next pseudorandom, uniformly distributed boolean value from
 this random number generator's sequence.
   nextBytes(byte[])   - 
Method in class weka.core. Debug.Random 
 Generates random bytes and places them into a user-supplied byte array.
   nextDouble()   - 
Method in class weka.core. Debug.Random 
 Returns the next pseudorandom, uniformly distributed double value between
 0.0 and 1.0 from this random number generator's sequence.
   nextElement(Instances)   - 
Method in class weka.core.converters. ConverterUtils.DataSource 
 returns the next element and sets the specified dataset, null if 
 none available.
   nextElement()   - 
Method in class weka.core. FastVector.FastVectorEnumeration 
 Returns the next element.
   nextElement()   - 
Method in class weka.core.tokenizers. AlphabeticTokenizer 
 returns the next element
   nextElement()   - 
Method in class weka.core.tokenizers. NGramTokenizer 
 Returns N-grams and also (N-1)-grams and ....
   nextElement()   - 
Method in class weka.core.tokenizers. Tokenizer 
 Returns the next element of this enumeration if this enumeration object 
 has at least one more element to provide.
   nextElement()   - 
Method in class weka.core.tokenizers. WordTokenizer 
 Returns the next element of this enumeration if this enumeration object 
 has at least one more element to provide.
   nextErlang(int)   - 
Method in class weka.core. RandomVariates 
 Generate a value of a variate following standard Erlang distribution.
   nextExponential()   - 
Method in class weka.core. RandomVariates 
 Generate a value of a variate following standard exponential
 distribution using simple inverse method.
   nextFloat()   - 
Method in class weka.core. Debug.Random 
 Returns the next pseudorandom, uniformly distributed float  value between
 0.0 and 1.0 from this random number generator's sequence.
   nextGamma(double)   - 
Method in class weka.core. RandomVariates 
 Generate a value of a variate following standard Gamma distribution 
 with shape parameter a.
   nextGaussian()   - 
Method in class weka.core. Debug.Random 
 Returns the next pseudorandom, Gaussian ("normally") distributed double
 value with mean 0.0 and standard deviation 1.0 from this random number
 generator's sequence.
   nextInt()   - 
Method in class weka.core. Debug.Random 
 Returns the next pseudorandom, uniformly distributed int  value from this
 random number generator's sequence.
   nextInt(int)   - 
Method in class weka.core. Debug.Random 
 Returns a pseudorandom, uniformly distributed int value between 0
 (inclusive) and the specified value (exclusive), drawn from this random
 number generator's sequence.
   nextIteration()   - 
Method in class weka.experiment. Experiment 
 Carries out the next iteration of the experiment.
   nextIteration()   - 
Method in class weka.experiment. RemoteExperiment 
 Overides the one in Experiment
   nextLong()   - 
Method in class weka.core. Debug.Random 
 Returns the next pseudorandom, uniformly distributed long  value from this
 random number generator's sequence.
   nextSplitAddedOrder()   - 
Method in class weka.classifiers.trees. ADTree 
 Returns the next number in the order that splitter nodes have been added to
 the tree, and records that a new splitter has been added.
   NGramMaxSizeTipText()   - 
Method in class weka.core.tokenizers. NGramTokenizer 
 Returns the tip text for this property.
   NGramMinSizeTipText()   - 
Method in class weka.core.tokenizers. NGramTokenizer 
 Returns the tip text for this property.
   NGramTokenizer   - Class in  weka.core.tokenizers  Splits a string into an n-gram with min and max grams.   NGramTokenizer()   - 
Constructor for class weka.core.tokenizers. NGramTokenizer 
  
   NNConditionalEstimator   - Class in  weka.estimators  Conditional probability estimator for a numeric domain conditional upon
 a numeric domain (using Mahalanobis distance).   NNConditionalEstimator()   - 
Constructor for class weka.estimators. NNConditionalEstimator 
  
   NNge   - Class in  weka.classifiers.rules  Nearest-neighbor-like algorithm using non-nested generalized exemplars (which are hyperrectangles that can be viewed as if-then rules).   NNge()   - 
Constructor for class weka.classifiers.rules. NNge 
  
   nnls(PaceMatrix, IntVector)   - 
Method in class weka.classifiers.functions.pace. PaceMatrix 
 Solves the nonnegative linear squares problem.
   nnlse(PaceMatrix, PaceMatrix, PaceMatrix, IntVector)   - 
Method in class weka.classifiers.functions.pace. PaceMatrix 
 Solves the nonnegative least squares problem with equality
        constraint.
   nnlse1(PaceMatrix, IntVector)   - 
Method in class weka.classifiers.functions.pace. PaceMatrix 
 Solves the nonnegative least squares problem with equality
        constraint.
   NNMMethod   - 
Static variable in class weka.classifiers.functions.pace. MixtureDistribution 
 The nonnegative-measure-based method
   NO_CLASS   - 
Static variable in class weka.associations. CheckAssociator 
 a "dummy" class type
   NO_CLASS   - 
Static variable in class weka.core. TestInstances 
 can be used to avoid generating a class attribute
   NO_COMMAND   - 
Static variable in class weka.gui.treevisualizer. TreeDisplayEvent 
  
   NO_SUPPORT   - 
Static variable in class weka.gui. GenericObjectEditor.GOETreeNode 
 color for "no support".
   Node   - Class in  weka.gui.treevisualizer  This class records all the data about a particular node for displaying.   Node(String, String, int, int, Color, String)   - 
Constructor for class weka.gui.treevisualizer. Node 
 This will setup all the values of the node except for its top and center.
   NodePlace   - Interface in  weka.gui.treevisualizer  This is an interface for classes that wish to take a node structure and 
 arrange them   nodeSplitterTipText()   - 
Method in class weka.core.neighboursearch. KDTree 
 Returns the tip text for this property.
   nodeToString()   - 
Method in class weka.classifiers.trees.m5. RuleNode 
 Returns a description of this node (debugging purposes)
   nodeType   - 
Variable in class weka.gui.graphvisualizer. GraphNode 
 Type of node.
   NOISE   - 
Static variable in interface weka.clusterers.forOPTICSAndDBScan.DataObjects. DataObject 
  
   noisePercentTipText()   - 
Method in class weka.datagenerators.classifiers.classification. LED24 
 Returns the tip text for this property
   noiseRateTipText()   - 
Method in class weka.datagenerators.classifiers.regression. MexicanHat 
 Returns the tip text for this property
   noiseRateTipText()   - 
Method in class weka.datagenerators.clusterers. BIRCHCluster 
 Returns the tip text for this property
   noiseRateTipText()   - 
Method in class weka.datagenerators.clusterers. SubspaceCluster 
 Returns the tip text for this property
   noiseThresholdTipText()   - 
Method in class weka.associations. Tertius 
 Returns the tip text for this property.
   noiseTipText()   - 
Method in class weka.classifiers.functions. GaussianProcesses 
 Returns the tip text for this property
   noiseVarianceTipText()   - 
Method in class weka.datagenerators.classifiers.regression. MexicanHat 
 Returns the tip text for this property
   NOMINAL   - 
Static variable in class weka.core. Attribute 
 Constant set for nominal attributes.
   nominalAttributesTipText()   - 
Method in class weka.core.converters. CSVLoader 
 Returns the tip text for this property.
   nominalColsTipText()   - 
Method in class weka.datagenerators. ClusterGenerator 
 Returns the tip text for this property
   nominalCounts   - 
Variable in class weka.core. AttributeStats 
 Counts of each nominal value
   nominalIndicesTipText()   - 
Method in class weka.filters.unsupervised.instance. RemoveWithValues 
 Returns the tip text for this property
   nominalLabelsTipText()   - 
Method in class weka.filters.unsupervised.attribute. Add 
 Returns the tip text for this property.
   NominalPrediction   - Class in  weka.classifiers.evaluation  Encapsulates an evaluatable nominal prediction: the predicted probability
 distribution plus the actual class value.   NominalPrediction(double, double[])   - 
Constructor for class weka.classifiers.evaluation. NominalPrediction 
 Creates the NominalPrediction object with a default weight of 1.0.
   NominalPrediction(double, double[], double)   - 
Constructor for class weka.classifiers.evaluation. NominalPrediction 
 Creates the NominalPrediction object.
   NominalToBinary   - Class in  weka.filters.supervised.attribute  Converts all nominal attributes into binary numeric attributes.   NominalToBinary()   - 
Constructor for class weka.filters.supervised.attribute. NominalToBinary 
  
   NominalToBinary   - Class in  weka.filters.unsupervised.attribute  Converts all nominal attributes into binary numeric attributes.   NominalToBinary()   - 
Constructor for class weka.filters.unsupervised.attribute. NominalToBinary 
 Constructor - initialises the filter
   nominalToBinaryFilterTipText()   - 
Method in class weka.classifiers.functions. MultilayerPerceptron 
  
   NominalToString   - Class in  weka.filters.unsupervised.attribute  Converts a nominal attribute (i.e.   NominalToString()   - 
Constructor for class weka.filters.unsupervised.attribute. NominalToString 
  
   NON_NUMERIC   - 
Static variable in class weka.filters.unsupervised.attribute. InterquartileRange 
 indicator for non-numeric attributes
   NONE   - 
Static variable in interface weka.core.converters. Loader 
 The retrieval modes
   NONE   - 
Static variable in interface weka.core.converters. Saver 
 The retrieval modes
   NONE   - 
Static variable in class weka.gui.visualize. VisualizePanelEvent 
 No longer used
   NonSparseToSparse   - Class in  weka.filters.unsupervised.instance  An instance filter that converts all incoming instances into sparse format.   NonSparseToSparse()   - 
Constructor for class weka.filters.unsupervised.instance. NonSparseToSparse 
  
   noPruningTipText()   - 
Method in class weka.classifiers.trees. REPTree 
 Returns the tip text for this property
   noReplacementTipText()   - 
Method in class weka.filters.supervised.instance. Resample 
 Returns the tip text for this property.
   noReplacementTipText()   - 
Method in class weka.filters.unsupervised.instance. Resample 
 Returns the tip text for this property
   norm()   - 
Method in class weka.core. AlgVector 
 Returns the norm of the vector
   norm1()   - 
Method in class weka.core.matrix. DoubleVector 
 Returns the L1-norm of the vector
   norm1()   - 
Method in class weka.core.matrix. Matrix 
 One norm
   norm2()   - 
Method in class weka.core.matrix. DoubleVector 
 Returns the L2-norm of the vector
   norm2()   - 
Method in class weka.core.matrix. Matrix 
 Two norm
   norm2()   - 
Method in class weka.core.matrix. SingularValueDecomposition 
 Two norm
   NORM_BASED   - 
Static variable in class weka.classifiers.bayes. BayesianLogisticRegression 
 Methods for selecting the hyperparameter value
   NORM_EXPECTED_COST_NAME   - 
Static variable in class weka.classifiers.evaluation. CostCurve 
 attribute name: Normalized Expected Cost
   NORMAL   - 
Static variable in interface weka.gui.graphvisualizer. GraphConstants 
 NORMAL node - node actually contained in graphs description
   normalDistribution   - 
Static variable in class weka.core.matrix. Maths 
 Distribution type: noraml
   NormalEstimator   - Class in  weka.estimators  Simple probability estimator that places a single normal distribution
 over the observed values.   NormalEstimator(double)   - 
Constructor for class weka.estimators. NormalEstimator 
 Constructor that takes a precision argument.
   normalInverse(double)   - 
Static method in class weka.core. Statistics 
 Returns the value,  x , for which the area under the
 Normal (Gaussian) probability density function (integrated from
 minus infinity to  x ) is equal to the argument  y 
 (assumes mean is zero, variance is one).
   NormalizableDistance   - Class in  weka.core  Represents the abstract ancestor for normalizable distance functions, like
 Euclidean or Manhattan distance.   NormalizableDistance()   - 
Constructor for class weka.core. NormalizableDistance 
 Invalidates the distance function, Instances must be still set.
   NormalizableDistance(Instances)   - 
Constructor for class weka.core. NormalizableDistance 
 Initializes the distance function and automatically initializes the
 ranges.
   normalize()   - 
Method in class weka.classifiers. CostMatrix 
 Normalizes the matrix so that the diagonal contains zeros.
   normalize()   - 
Method in class weka.classifiers.functions.pace. DiscreteFunction 
 Normalizes the function values with L1-norm.
   normalize(double[])   - 
Static method in class weka.core. Utils 
 Normalizes the doubles in the array by their sum.
   normalize(double[], double)   - 
Static method in class weka.core. Utils 
 Normalizes the doubles in the array using the given value.
   Normalize   - Class in  weka.filters.unsupervised.attribute  Normalizes all numeric values in the given dataset (apart from the class attribute, if set).   Normalize()   - 
Constructor for class weka.filters.unsupervised.attribute. Normalize 
  
   Normalize   - Class in  weka.filters.unsupervised.instance  An instance filter that normalize instances considering only numeric attributes and ignoring class index.   Normalize()   - 
Constructor for class weka.filters.unsupervised.instance. Normalize 
  
   normalizeAttributesTipText()   - 
Method in class weka.classifiers.functions. MultilayerPerceptron 
  
   NormalizeData   - 
Variable in class weka.classifiers.bayes. BayesianLogisticRegression 
 Choose whether to normalize data or not
   normalizeDataTipText()   - 
Method in class weka.classifiers.bayes. BayesianLogisticRegression 
 Returns the tip text for this property
   normalizeDimWidthsTipText()   - 
Method in class weka.core.neighboursearch.balltrees. MedianOfWidestDimension 
 Returns the tip text for this property.
   normalizedKernel(char[], char[])   - 
Method in class weka.classifiers.functions.supportVector. StringKernel 
 evaluates the normalized kernel between s and t.
   normalizeDocLengthTipText()   - 
Method in class weka.filters.unsupervised.attribute. StringToWordVector 
 Returns the tip text for this property.
   NormalizedPolyKernel   - Class in  weka.classifiers.functions.supportVector  The normalized polynomial kernel. 
 K(x,y) = <x,y>/sqrt(<x,x><y,y>) where <x,y> = PolyKernel(x,y)
  
  
 
  
 Valid options are:     NormalizedPolyKernel()   - 
Constructor for class weka.classifiers.functions.supportVector. NormalizedPolyKernel 
 default constructor - does nothing
   NormalizedPolyKernel(Instances, int, double, boolean)   - 
Constructor for class weka.classifiers.functions.supportVector. NormalizedPolyKernel 
 Creates a new  NormalizedPolyKernel  instance.
   normalizeNodeWidthTipText()   - 
Method in class weka.core.neighboursearch. KDTree 
 Tip text for this property.
   normalizeNumericClassTipText()   - 
Method in class weka.classifiers.functions. MultilayerPerceptron 
  
   normalizeTipText()   - 
Method in class weka.attributeSelection. LatentSemanticAnalysis 
 Returns the tip text for this property
   normalizeTipText()   - 
Method in class weka.classifiers.functions. LibLINEAR 
 Returns the tip text for this property
   normalizeTipText()   - 
Method in class weka.classifiers.functions. LibSVM 
 Returns the tip text for this property
   normalizeWordWeightsTipText()   - 
Method in class weka.classifiers.bayes. ComplementNaiveBayes 
 Returns the tip text for this property
   NormalMixture   - Class in  weka.classifiers.functions.pace  Class for manipulating normal mixture distributions.   NormalMixture()   - 
Constructor for class weka.classifiers.functions.pace. NormalMixture 
 Contructs an empty NormalMixture
   normalProbability(double)   - 
Static method in class weka.core. Statistics 
 Returns the area under the Normal (Gaussian) probability density
 function, integrated from minus infinity to  x 
 (assumes mean is zero, variance is one).
   normBasedHyperParameter()   - 
Method in class weka.classifiers.bayes. BayesianLogisticRegression 
 This function computes the norm-based hyperparameters
        and stores them in the m_Hyperparameters.
   NormContinuous   - Class in  weka.core.pmml  Class encapsulating a NormContinuous Expression.   NormContinuous(Element, FieldMetaInfo.Optype, ArrayList<Attribute>)   - 
Constructor for class weka.core.pmml. NormContinuous 
  
   NormDiscrete   - Class in  weka.core.pmml  Class encapsulating a NormDiscrete Expression.   NormDiscrete(Element, FieldMetaInfo.Optype, ArrayList<Attribute>)   - 
Constructor for class weka.core.pmml. NormDiscrete 
 Constructor.
   normF()   - 
Method in class weka.core.matrix. Matrix 
 Frobenius norm
   normInf()   - 
Method in class weka.core.matrix. Matrix 
 Infinity norm
   normTipText()   - 
Method in class weka.filters.unsupervised.instance. Normalize 
 Returns the tip text for this property
   normVector()   - 
Method in class weka.core. AlgVector 
 Norms this vector to length 1.0
   NORTH_CONNECTOR   - 
Static variable in class weka.gui.beans. BeanVisual 
  
   NoSplit   - Class in  weka.classifiers.trees.j48  Class implementing a "no-split"-split.   NoSplit(Distribution)   - 
Constructor for class weka.classifiers.trees.j48. NoSplit 
 Creates "no-split"-split for given distribution.
   NoSupportForMissingValuesException   - Exception in  weka.core  Exception that is raised by an object that is unable to process 
 data with missing values.   NoSupportForMissingValuesException()   - 
Constructor for exception weka.core. NoSupportForMissingValuesException 
 Creates a new NoSupportForMissingValuesException with no message.
   NoSupportForMissingValuesException(String)   - 
Constructor for exception weka.core. NoSupportForMissingValuesException 
 Creates a new NoSupportForMissingValuesException.
   NOT   - 
Static variable in interface weka.core.mathematicalexpression. sym 
  
   NOT   - 
Static variable in interface weka.filters.unsupervised.instance.subsetbyexpression. sym 
  
   NOT_DRAWABLE   - 
Static variable in interface weka.core. Drawable 
  
   notCoveredInstances()   - 
Method in class weka.classifiers.trees.m5. Rule 
 Get the instances not covered by this rule
   notifyCapabilitiesFilterListener(Capabilities)   - 
Method in class weka.gui.explorer. Explorer 
 notifies all the listeners of a change
   notifyListener()   - 
Method in class weka.gui.arffviewer. ArffPanel 
 notfies all listener of the change
   notifyListener(TableModelEvent)   - 
Method in class weka.gui.arffviewer. ArffSortedTableModel 
 notfies all listener of the change of the model
   notifyListener(TableModelEvent)   - 
Method in class weka.gui.arffviewer. ArffTableModel 
 notfies all listener of the change of the model
   notUnifyNormTipText()   - 
Method in class weka.clusterers. sIB 
 Returns the tip text for this property.
   nrOfGoodOperationsTipText()   - 
Method in class weka.classifiers.bayes.net.search.local. LAGDHillClimber 
  
   nrOfLookAheadStepsTipText()   - 
Method in class weka.classifiers.bayes.net.search.local. LAGDHillClimber 
  
   NullStemmer   - Class in  weka.core.stemmers  A dummy stemmer that performs no stemming at all.   NullStemmer()   - 
Constructor for class weka.core.stemmers. NullStemmer 
  
   NUM_RAND_COLS   - 
Static variable in interface weka.classifiers.lazy.kstar. KStarConstants 
  
   numAllConditions(Instances)   - 
Static method in class weka.classifiers.rules. RuleStats 
 Compute the number of all possible conditions that could 
 appear in a rule of a given data.
   numAntdsTipText()   - 
Method in class weka.classifiers.rules. ConjunctiveRule 
 Returns the tip text for this property
   numArcsTipText()   - 
Method in class weka.datagenerators.classifiers.classification. BayesNet 
 Returns the tip text for this property
   numArguments()   - 
Method in class weka.core. Option 
 Returns the option's number of arguments.
   numAttemptsOfGeneOptionTipText()   - 
Method in class weka.classifiers.rules. NNge 
 Returns the tip text for this property
   numAttributes()   - 
Method in class weka.core. Instance 
 Returns the number of attributes.
   numAttributes()   - 
Method in class weka.core. Instances 
 Returns the number of attributes.
   numAttributes()   - 
Method in class weka.core. SparseInstance 
 Returns the number of attributes.
   numAttributesTipText()   - 
Method in class weka.datagenerators.classifiers.classification. BayesNet 
 Returns the tip text for this property
   numAttributesTipText()   - 
Method in class weka.datagenerators.classifiers.classification. RandomRBF 
 Returns the tip text for this property
   numAttributesTipText()   - 
Method in class weka.datagenerators.classifiers.classification. RDG1 
 Returns the tip text for this property
   numAttributesTipText()   - 
Method in class weka.datagenerators.clusterers. SubspaceCluster 
 Returns the tip text for this property
   numAttributesTipText()   - 
Method in class weka.datagenerators. ClusterGenerator 
 Returns the tip text for this property
   numAttributesTipText()   - 
Method in class weka.filters.unsupervised.attribute. RandomSubset 
 Returns the tip text for this property.
   numBags()   - 
Method in class weka.classifiers.trees.j48. Distribution 
 Returns number of bags.
   NUMBER   - 
Static variable in interface weka.core.mathematicalexpression. sym 
  
   NUMBER   - 
Static variable in interface weka.filters.unsupervised.instance.subsetbyexpression. sym 
  
   numberAttributesSelected()   - 
Method in class weka.attributeSelection. AttributeSelection 
 Return the number of attributes selected from the most recent
 run of attribute selection
   numberLiteralsTipText()   - 
Method in class weka.associations. Tertius 
 Returns the tip text for this property.
   numberOfAttributesTipText()   - 
Method in class weka.filters.unsupervised.attribute. RandomProjection 
 Returns the tip text for this property
   numberOfClusters()   - 
Method in class weka.clusterers. AbstractClusterer 
 Returns the number of clusters.
   numberOfClusters()   - 
Method in class weka.clusterers. CLOPE 
  
   numberOfClusters()   - 
Method in interface weka.clusterers. Clusterer 
 Returns the number of clusters.
   numberOfClusters()   - 
Method in class weka.clusterers. Cobweb 
 Returns the number of clusters.
   numberOfClusters()   - 
Method in class weka.clusterers. DBScan 
 Returns the number of clusters.
   numberOfClusters()   - 
Method in class weka.clusterers. EM 
 Returns the number of clusters.
   numberOfClusters()   - 
Method in class weka.clusterers. FarthestFirst 
 Returns the number of clusters.
   numberOfClusters()   - 
Method in class weka.clusterers. HierarchicalClusterer 
  
   numberOfClusters()   - 
Method in class weka.clusterers. MakeDensityBasedClusterer 
 Returns the number of clusters.
   numberOfClusters()   - 
Method in class weka.clusterers. OPTICS 
 Returns the number of clusters.
   numberOfClusters()   - 
Method in class weka.clusterers. sIB 
 Get the number of clusters
   numberOfClusters()   - 
Method in class weka.clusterers. SimpleKMeans 
 Returns the number of clusters.
   numberOfClusters()   - 
Method in class weka.clusterers. SingleClustererEnhancer 
 Returns the number of clusters.
   numberOfClusters()   - 
Method in class weka.clusterers. XMeans 
 Returns the number of clusters.
   NumberOfClustersRequestable   - Interface in  weka.clusterers  Interface to a clusterer that can generate a requested number of
 clusters   numberOfGroupsTipText()   - 
Method in class weka.classifiers.meta. RotationForest 
 Returns the tip text for this property
   numberOfLinearModels()   - 
Method in class weka.classifiers.trees.m5. RuleNode 
 Get the number of linear models in the tree
   numBinsTipText()   - 
Method in class weka.classifiers.meta. RegressionByDiscretization 
 Returns the tip text for this property
   numBoostingIterationsTipText()   - 
Method in class weka.classifiers.functions. SimpleLogistic 
 Returns the tip text for this property
   numBoostingIterationsTipText()   - 
Method in class weka.classifiers.trees. FT 
 Returns the tip text for this property
   numBoostingIterationsTipText()   - 
Method in class weka.classifiers.trees. LMT 
 Returns the tip text for this property
   numCacheHits()   - 
Method in class weka.classifiers.functions.supportVector. CachedKernel 
 Returns the number of cache hits on dot products.
   numCacheHits()   - 
Method in class weka.classifiers.functions.supportVector. Kernel 
 Returns the number of dot product cache hits.
   numCacheHits()   - 
Method in class weka.classifiers.functions.supportVector. PrecomputedKernelMatrixKernel 
 Returns the number of dot product cache hits.
   numCacheHits()   - 
Method in class weka.classifiers.functions.supportVector. StringKernel 
 Returns the number of dot product cache hits.
   numCentroidsTipText()   - 
Method in class weka.datagenerators.classifiers.classification. RandomRBF 
 Returns the tip text for this property
   numChildren()   - 
Method in class weka.gui. HierarchyPropertyParser 
 The number of the children nodes.
   numCitersTipText()   - 
Method in class weka.classifiers.mi. CitationKNN 
 Returns the tip text for this property
   numClassAttributeValues()   - 
Method in class weka.classifiers.functions. SMO 
  
   numClassAttributeValues()   - 
Method in class weka.classifiers.mi. MISMO 
 Returns the number of values of the class attribute.
   numClasses()   - 
Method in class weka.classifiers.trees.j48. Distribution 
 Returns number of classes.
   numClasses()   - 
Method in class weka.core. Instance 
 Returns the number of class labels.
   numClasses()   - 
Method in class weka.core. Instances 
 Returns the number of class labels.
   numClassesTipText()   - 
Method in class weka.datagenerators.classifiers.classification. RandomRBF 
 Returns the tip text for this property
   numClassesTipText()   - 
Method in class weka.datagenerators.classifiers.classification. RDG1 
 Returns the tip text for this property
   numClustersTipText()   - 
Method in class weka.classifiers.functions. RBFNetwork 
 Returns the tip text for this property
   numClustersTipText()   - 
Method in class weka.clusterers. EM 
 Returns the tip text for this property
   numClustersTipText()   - 
Method in class weka.clusterers. FarthestFirst 
 Returns the tip text for this property
   numClustersTipText()   - 
Method in class weka.clusterers. HierarchicalClusterer 
  
   numClustersTipText()   - 
Method in class weka.clusterers. sIB 
 Returns the tip text for this property.
   numClustersTipText()   - 
Method in class weka.clusterers. SimpleKMeans 
 Returns the tip text for this property
   numClustersTipText()   - 
Method in class weka.datagenerators.clusterers. BIRCHCluster 
 Returns the tip text for this property
   numColumns()   - 
Method in class weka.classifiers. CostMatrix 
 Same as size
   numColumns()   - 
Method in class weka.core. Matrix 
  Deprecated.  Returns the number of columns in the matrix.
   numComponentsTipText()   - 
Method in class weka.filters.supervised.attribute. PLSFilter 
 Returns the tip text for this property
   numCorrect()   - 
Method in class weka.classifiers.trees.j48. Distribution 
 Returns perClass(maxClass()).
   numCorrect(int)   - 
Method in class weka.classifiers.trees.j48. Distribution 
 Returns perClassPerBag(index,maxClass(index)).
   numCyclesTipText()   - 
Method in class weka.datagenerators.clusterers. BIRCHCluster 
 Returns the tip text for this property
   numDistinctValues(int)   - 
Method in class weka.core. Instances 
 Returns the number of distinct values of a given attribute.
   numDistinctValues(Attribute)   - 
Method in class weka.core. Instances 
 Returns the number of distinct values of a given attribute.
   numElements()   - 
Method in class weka.classifiers.functions.supportVector. SMOset 
 Returns the number of elements in the set.
   numElements()   - 
Method in class weka.core. AlgVector 
 Returns the number of elements in the vector.
   NUMERIC   - 
Static variable in class weka.core. Attribute 
 Constant set for numeric attributes.
   NumericCleaner   - Class in  weka.filters.unsupervised.attribute  A filter that 'cleanses' the numeric data from values that are too small, too big or very close to a certain value (e.g., 0) and sets these values to a pre-defined default.   NumericCleaner()   - 
Constructor for class weka.filters.unsupervised.attribute. NumericCleaner 
  
   NumericPrediction   - Class in  weka.classifiers.evaluation  Encapsulates an evaluatable numeric prediction: the predicted class value
 plus the actual class value.   NumericPrediction(double, double)   - 
Constructor for class weka.classifiers.evaluation. NumericPrediction 
 Creates the NumericPrediction object with a default weight of 1.0.
   NumericPrediction(double, double, double)   - 
Constructor for class weka.classifiers.evaluation. NumericPrediction 
 Creates the NumericPrediction object.
   numericStats   - 
Variable in class weka.core. AttributeStats 
 Stats on numeric value distributions
   numericTipText()   - 
Method in class weka.filters.unsupervised.attribute. MakeIndicator 
  
   NumericToBinary   - Class in  weka.filters.unsupervised.attribute  Converts all numeric attributes into binary attributes (apart from the class attribute, if set): if the value of the numeric attribute is exactly zero, the value of the new attribute will be zero.   NumericToBinary()   - 
Constructor for class weka.filters.unsupervised.attribute. NumericToBinary 
  
   NumericToNominal   - Class in  weka.filters.unsupervised.attribute  A filter for turning numeric attributes into nominal ones.   NumericToNominal()   - 
Constructor for class weka.filters.unsupervised.attribute. NumericToNominal 
  
   NumericTransform   - Class in  weka.filters.unsupervised.attribute  Transforms numeric attributes using a given transformation method.   NumericTransform()   - 
Constructor for class weka.filters.unsupervised.attribute. NumericTransform 
 Default constructor -- sets the default transform method
 to java.lang.Math.abs().
   numEvals()   - 
Method in class weka.classifiers.functions.supportVector. CachedKernel 
 Returns the number of time Eval has been called.
   numEvals()   - 
Method in class weka.classifiers.functions.supportVector. Kernel 
 Returns the number of kernel evaluation performed.
   numEvals()   - 
Method in class weka.classifiers.functions.supportVector. PrecomputedKernelMatrixKernel 
 Returns the number of kernel evaluation performed.
   numEvals()   - 
Method in class weka.classifiers.functions.supportVector. StringKernel 
 Returns the number of kernel evaluation performed.
   numExamplesTipText()   - 
Method in class weka.datagenerators. ClassificationGenerator 
 Returns the tip text for this property
   numExamplesTipText()   - 
Method in class weka.datagenerators. RegressionGenerator 
 Returns the tip text for this property
   numFalseNegatives(int)   - 
Method in class weka.classifiers. Evaluation 
 Calculate number of false negatives with respect to a particular class.
   numFalsePositives(int)   - 
Method in class weka.classifiers. Evaluation 
 Calculate number of false positives with respect to a particular class.
   numFeaturesTipText()   - 
Method in class weka.classifiers.trees. RandomForest 
 Returns the tip text for this property
   numFoldersMIOptionTipText()   - 
Method in class weka.classifiers.rules. NNge 
 Returns the tip text for this property
   NumFolds   - 
Variable in class weka.classifiers.bayes. BayesianLogisticRegression 
 NumFolds for CV based Hyperparameters selection
   numFoldsPruningTipText()   - 
Method in class weka.classifiers.trees. BFTree 
 Returns the tip text for this property
   numFoldsPruningTipText()   - 
Method in class weka.classifiers.trees. SimpleCart 
 Returns the tip text for this property
   numFoldsTipText()   - 
Method in class weka.classifiers.bayes. BayesianLogisticRegression 
 Returns the tip text for this property
   numFoldsTipText()   - 
Method in class weka.classifiers.functions. SMO 
 Returns the tip text for this property
   numFoldsTipText()   - 
Method in class weka.classifiers.meta. CVParameterSelection 
 Returns the tip text for this property
   numFoldsTipText()   - 
Method in class weka.classifiers.meta. Dagging 
 Returns the tip text for this property
   numFoldsTipText()   - 
Method in class weka.classifiers.meta. LogitBoost 
 Returns the tip text for this property
   numFoldsTipText()   - 
Method in class weka.classifiers.meta. MultiScheme 
 Returns the tip text for this property
   numFoldsTipText()   - 
Method in class weka.classifiers.meta. Stacking 
 Returns the tip text for this property
   numFoldsTipText()   - 
Method in class weka.classifiers.mi. MISMO 
 Returns the tip text for this property
   numFoldsTipText()   - 
Method in class weka.classifiers.rules. PART 
 Returns the tip text for this property
   numFoldsTipText()   - 
Method in class weka.classifiers.trees. J48 
 Returns the tip text for this property
   numFoldsTipText()   - 
Method in class weka.classifiers.trees. RandomTree 
 Returns the tip text for this property
   numFoldsTipText()   - 
Method in class weka.classifiers.trees. REPTree 
 Returns the tip text for this property
   numFoldsTipText()   - 
Method in class weka.experiment. CrossValidationResultProducer 
 Returns the tip text for this property
   numFoldsTipText()   - 
Method in class weka.filters.supervised.instance. StratifiedRemoveFolds 
 Returns the tip text for this property
   numFoldsTipText()   - 
Method in class weka.filters.unsupervised.instance. RemoveFolds 
 Returns the tip text for this property
   numFoldsTipText()   - 
Method in class weka.filters.unsupervised.instance. RemoveMisclassified 
 Returns the tip text for this property
   numIncorrect()   - 
Method in class weka.classifiers.trees.j48. Distribution 
 Returns total-numCorrect().
   numIncorrect(int)   - 
Method in class weka.classifiers.trees.j48. Distribution 
 Returns perBag(index)-numCorrect(index).
   numInnerNodes()   - 
Method in class weka.classifiers.trees. SimpleCart 
 Method to count the number of inner nodes in the tree.
   numInstances()   - 
Method in class weka.classifiers. Evaluation 
 Gets the number of test instances that had a known class value
 (actually the sum of the weights of test instances with known 
 class value).
   numInstances()   - 
Method in class weka.core. Instances 
 Returns the number of instances in the dataset.
   numInstances()   - 
Method in class weka.core.neighboursearch.balltrees. BallNode 
 Returns the number of instances in the
 hyper-spherical region of this node.
   numInstances()   - 
Method in class weka.core.neighboursearch.kdtrees. KDTreeNode 
 Returns the number of Instances 
 in the rectangular region defined 
 by this node.
   numIrrelevantTipText()   - 
Method in class weka.datagenerators.classifiers.classification. RDG1 
 Returns the tip text for this property
   numIterationsTipText()   - 
Method in class weka.classifiers.bayes. DMNBtext 
 Returns the tip text for this property
   numIterationsTipText()   - 
Method in class weka.classifiers.functions. VotedPerceptron 
 Returns the tip text for this property
   numIterationsTipText()   - 
Method in class weka.classifiers.functions. Winnow 
 Returns the tip text for this property
   numIterationsTipText()   - 
Method in class weka.classifiers. IteratedSingleClassifierEnhancer 
 Returns the tip text for this property
   numIterationsTipText()   - 
Method in class weka.classifiers.meta. Decorate 
 Returns the tip text for this property
   numIterationsTipText()   - 
Method in class weka.classifiers.meta. MetaCost 
 Returns the tip text for this property
   numLeaves()   - 
Method in class weka.classifiers.trees. BFTree 
 Compute number of leaf nodes.
   numLeaves()   - 
Method in class weka.classifiers.trees.ft. FTtree 
 Returns the number of leaves (normal count).
   numLeaves()   - 
Method in class weka.classifiers.trees.j48. ClassifierTree 
 Returns number of leaves in tree structure.
   numLeaves()   - 
Method in class weka.classifiers.trees.lmt. LMTNode 
 Returns the number of leaves (normal count).
   numLeaves(int)   - 
Method in class weka.classifiers.trees.m5. RuleNode 
 Sets the leaves' numbers
   numLeaves()   - 
Method in class weka.classifiers.trees. SimpleCart 
 Compute number of leaf nodes.
   numLiterals()   - 
Method in class weka.associations.tertius. LiteralSet 
 Give the number of literals in this set.
   numLiterals()   - 
Method in class weka.associations.tertius. Predicate 
  
   numLiterals()   - 
Method in class weka.associations.tertius. Rule 
 Give the number of literals in this rule.
   numNeighboursTipText()   - 
Method in class weka.attributeSelection. ReliefFAttributeEval 
 Returns the tip text for this property
   numNeighboursTipText()   - 
Method in class weka.classifiers.mi. MINND 
 Returns the tip text for this property
   numNodes()   - 
Method in class weka.classifiers.trees. BFTree 
 Compute size of the tree.
   numNodes()   - 
Method in class weka.classifiers.trees.ft. FTtree 
 Returns the number of nodes.
   numNodes()   - 
Method in class weka.classifiers.trees.j48. ClassifierTree 
 Returns number of nodes in tree structure.
   numNodes()   - 
Method in class weka.classifiers.trees.lmt. LMTNode 
 Returns the number of nodes.
   numNodes()   - 
Method in class weka.classifiers.trees. RandomTree 
 Computes size of the tree.
   numNodes()   - 
Method in class weka.classifiers.trees. REPTree 
 Computes size of the tree.
   numNodes()   - 
Method in class weka.classifiers.trees. SimpleCart 
 Compute size of the tree.
   numNumericTipText()   - 
Method in class weka.datagenerators.classifiers.classification. RDG1 
 Returns the tip text for this property
   numOfBoostingIterationsTipText()   - 
Method in class weka.classifiers.trees. ADTree 
  
   numOfBoostingIterationsTipText()   - 
Method in class weka.classifiers.trees. LADTree 
  
   numParameters()   - 
Method in class weka.classifiers.functions. LinearRegression 
 Get the number of coefficients used in the model
   numParameters()   - 
Method in class weka.classifiers.functions. PaceRegression 
 Get the number of coefficients used in the model
   numParameters()   - 
Method in class weka.classifiers.trees.m5. PreConstructedLinearModel 
 Return the number of parameters (coefficients) in the linear model
   numPendingOutput()   - 
Method in class weka.filters. Filter 
 Returns the number of instances pending output
   numPendingOutput()   - 
Method in class weka.filters.unsupervised.attribute. RemoveType 
 Returns the number of instances pending output
   numReferencesTipText()   - 
Method in class weka.classifiers.mi. CitationKNN 
 Returns the tip text for this property
   numRestartsTipText()   - 
Method in class weka.clusterers. sIB 
 Returns the tip text for this property.
   numRows()   - 
Method in class weka.classifiers. CostMatrix 
 Same as size
   numRows()   - 
Method in class weka.core. Matrix 
  Deprecated.  Returns the number of rows in the matrix.
   numRules()   - 
Method in class weka.classifiers.rules.part. MakeDecList 
 Outputs the number of rules in the classifier.
   numRulesTipText()   - 
Method in class weka.associations. Apriori 
 Returns the tip text for this property
   numRulesTipText()   - 
Method in class weka.associations. PredictiveApriori 
 Returns the tip text for this property
   numRulesToFindTipText()   - 
Method in class weka.associations. FPGrowth 
 Tip text for this property suitable for displaying
 in the GUI.
   numRunsTipText()   - 
Method in class weka.classifiers.meta. LogitBoost 
 Returns the tip text for this property
   numSubCmtysTipText()   - 
Method in class weka.classifiers.meta. MultiBoostAB 
 Returns the tip text for this property
   numSubsets()   - 
Method in class weka.classifiers.trees.j48. ClassifierSplitModel 
 Returns the number of created subsets for the split.
   numSubsetSizeCVFoldsTipText()   - 
Method in class weka.attributeSelection. SubsetSizeForwardSelection 
 Returns the tip text for this property
   numTestingNoisesTipText()   - 
Method in class weka.classifiers.mi. MINND 
 Returns the tip text for this property
   numToSelectTipText()   - 
Method in class weka.attributeSelection. GreedyStepwise 
 Returns the tip text for this property
   numToSelectTipText()   - 
Method in class weka.attributeSelection. RaceSearch 
 Returns the tip text for this property
   numToSelectTipText()   - 
Method in class weka.attributeSelection. Ranker 
 Returns the tip text for this property
   numTrainingNoisesTipText()   - 
Method in class weka.classifiers.mi. MINND 
 Returns the tip text for this property
   numTreesTipText()   - 
Method in class weka.classifiers.trees. RandomForest 
 Returns the tip text for this property
   numTrueNegatives(int)   - 
Method in class weka.classifiers. Evaluation 
 Calculate the number of true negatives with respect to a particular class.
   numTruePositives(int)   - 
Method in class weka.classifiers. Evaluation 
 Calculate the number of true positives with respect to a particular class.
   numUsedAttributesTipText()   - 
Method in class weka.attributeSelection. LinearForwardSelection 
 Returns the tip text for this property
   numUsedAttributesTipText()   - 
Method in class weka.attributeSelection. SubsetSizeForwardSelection 
 Returns the tip text for this property
   numValues()   - 
Method in class weka.core. Attribute 
 Returns the number of attribute values.
   numValues()   - 
Method in class weka.core. Instance 
 Returns the number of values present.
   numValues()   - 
Method in class weka.core. SparseInstance 
 Returns the number of values in the sparse vector.
   numValuesTipText()   - 
Method in class weka.filters.unsupervised.instance. RemoveFrequentValues 
 Returns the tip text for this property
   numXValFoldsTipText()   - 
Method in class weka.classifiers.meta. ThresholdSelector 
  
   nuTipText()   - 
Method in class weka.classifiers.functions. LibSVM 
 Returns the tip text for this property
 
 
    
 O  
 
   Obfuscate   - Class in  weka.filters.unsupervised.attribute  A simple instance filter that renames the relation, all attribute names and all nominal (and string) attribute values.   Obfuscate()   - 
Constructor for class weka.filters.unsupervised.attribute. Obfuscate 
  
   observedComparator   - 
Static variable in class weka.associations.tertius. Rule 
 Comparator used to compare two rules according to their observed number
 of counter-instances.
   obtainVotes(Instance)   - 
Method in class weka.classifiers.functions. SMO 
 Returns an array of votes for the given instance.
   OFF   - 
Static variable in interface weka.classifiers.lazy.kstar. KStarConstants 
  
   OFF   - 
Static variable in class weka.core. Debug 
 the log level Off - i.e., no logging
   oldEnt(Distribution)   - 
Method in class weka.classifiers.trees.j48. EntropyBasedSplitCrit 
 Computes entropy of distribution before splitting.
   omegaTipText()   - 
Method in class weka.classifiers.functions.supportVector. Puk 
 Returns the tip text for this property
   ON   - 
Static variable in interface weka.classifiers.lazy.kstar. KStarConstants 
 Some usefull constants
   onDemandDirectoryTipText()   - 
Method in class weka.attributeSelection. CostSensitiveASEvaluation 
  
   onDemandDirectoryTipText()   - 
Method in class weka.classifiers.meta. CostSensitiveClassifier 
  
   onDemandDirectoryTipText()   - 
Method in class weka.classifiers.meta. MetaCost 
 Returns the tip text for this property
   onDemandDirectoryTipText()   - 
Method in class weka.experiment. CostSensitiveClassifierSplitEvaluator 
 Returns the tip text for this property
   oneElementsToSequences(FastVector)   - 
Static method in class weka.associations.gsp. Sequence 
 Converts a set of 1-Elements into a set of 1-Sequences.
   OneR   - Class in  weka.classifiers.rules  Class for building and using a 1R classifier; in other words, uses the minimum-error attribute for prediction, discretizing numeric attributes.   OneR()   - 
Constructor for class weka.classifiers.rules. OneR 
  
   OneRAttributeEval   - Class in  weka.attributeSelection  OneRAttributeEval : 
  
 Evaluates the worth of an attribute by using the OneR classifier. 
  
  

  
 Valid options are:     OneRAttributeEval()   - 
Constructor for class weka.attributeSelection. OneRAttributeEval 
 Constructor
   onUnit(Graphics, int, int, int, int)   - 
Method in class weka.classifiers.functions.neural. NeuralConnection 
 Call this function to determine if the point at x,y is on the unit.
   openFrame(String)   - 
Method in class weka.gui. ResultHistoryPanel 
 Opens the named result in a separate frame.
   openURL(String)   - 
Static method in class weka.gui. BrowserHelper 
 opens the URL in a browser.
   openURL(Component, String)   - 
Static method in class weka.gui. BrowserHelper 
 opens the URL in a browser.
   openURL(Component, String, boolean)   - 
Static method in class weka.gui. BrowserHelper 
 opens the URL in a browser.
   OPTICS   - Class in  weka.clusterers  Mihael Ankerst, Markus M.   OPTICS()   - 
Constructor for class weka.clusterers. OPTICS 
  
   OPTICS_Visualizer   - Class in  weka.clusterers.forOPTICSAndDBScan.OPTICS_GUI  Start the OPTICS Visualizer from command-line:  
  java weka.clusterers.forOPTICSAndDBScan.OPTICS_GUI.OPTICS_Visualizer [file.ser] 
     OPTICS_Visualizer(SERObject, String)   - 
Constructor for class weka.clusterers.forOPTICSAndDBScan.OPTICS_GUI. OPTICS_Visualizer 
  
   optimisticComparator   - 
Static variable in class weka.associations.tertius. Rule 
 Comparator used to compare two rules according to their optimistic estimate.
   optimisticThenObservedComparator   - 
Static variable in class weka.associations.tertius. Rule 
 Comparator used to compare two rules according to their optimistic estimate
 and then their observed number of counter-instances.
   Optimization   - Class in  weka.core  Implementation of Active-sets method with BFGS update to solve optimization
 problem with only bounds constraints in multi-dimensions.   Optimization()   - 
Constructor for class weka.core. Optimization 
  
   optimizationsTipText()   - 
Method in class weka.classifiers.rules. JRip 
 Returns the tip text for this property
   optimize()   - 
Method in class weka.classifiers.functions.supportVector. RegSMO 
 finds alpha and alpha* parameters that optimize the SVM target function
   OPTIMIZE_0   - 
Static variable in class weka.classifiers.meta. ThresholdSelector 
 first class value
   OPTIMIZE_1   - 
Static variable in class weka.classifiers.meta. ThresholdSelector 
 second class value
   OPTIMIZE_LFREQ   - 
Static variable in class weka.classifiers.meta. ThresholdSelector 
 least frequent class value
   OPTIMIZE_MFREQ   - 
Static variable in class weka.classifiers.meta. ThresholdSelector 
 most frequent class value
   OPTIMIZE_POS_NAME   - 
Static variable in class weka.classifiers.meta. ThresholdSelector 
 class value name, either 'yes' or 'pos(itive)'
   Option   - Class in  weka.core  Class to store information about an option.   Option(String, String, int, String)   - 
Constructor for class weka.core. Option 
 Creates new option with the given parameters.
   OptionHandler   - Interface in  weka.core  Interface to something that understands options.   OptionHandlerJavadoc   - Class in  weka.core  Generates Javadoc comments from the OptionHandler's options.   OptionHandlerJavadoc()   - 
Constructor for class weka.core. OptionHandlerJavadoc 
 default constructor
   OPTIONS_ENDTAG   - 
Static variable in class weka.core. OptionHandlerJavadoc 
 the end comment tag for inserting the generated Javadoc
   OPTIONS_STARTTAG   - 
Static variable in class weka.core. OptionHandlerJavadoc 
 the start comment tag for inserting the generated Javadoc
   or(Capabilities)   - 
Method in class weka.core. Capabilities 
 performs an OR conjunction with the capabilities of the given 
 Capabilities object and updates itself
   OR   - 
Static variable in interface weka.core.mathematicalexpression. sym 
  
   OR   - 
Static variable in interface weka.filters.unsupervised.instance.subsetbyexpression. sym 
  
   orderAdded   - 
Variable in class weka.classifiers.trees.adtree. Splitter 
 The number this node was in the order of nodes added to the tree
   ORDERED   - 
Static variable in class weka.datagenerators.clusterers. BIRCHCluster 
 Constant set for input order (option O)
   ordering()   - 
Method in class weka.core. Attribute 
 Returns the ordering of the attribute.
   ORDERING_MODULO   - 
Static variable in class weka.core. Attribute 
 Constant set for modulo-ordered attributes.
   ORDERING_ORDERED   - 
Static variable in class weka.core. Attribute 
 Constant set for ordered attributes.
   ORDERING_SYMBOLIC   - 
Static variable in class weka.core. Attribute 
 Constant set for symbolic attributes.
   OrdinalClassClassifier   - Class in  weka.classifiers.meta  Meta classifier that allows standard classification algorithms to be applied to ordinal class problems. 
  
 For more information see:  
  
 Eibe Frank, Mark Hall: A Simple Approach to Ordinal Classification.   OrdinalClassClassifier()   - 
Constructor for class weka.classifiers.meta. OrdinalClassClassifier 
 Default constructor.
   originalValue(double)   - 
Method in class weka.filters.supervised.attribute. ClassOrder 
 Return the original internal class value given the randomized 
 class value, i.e.
   outlierFactorTipText()   - 
Method in class weka.filters.unsupervised.attribute. InterquartileRange 
 Returns the tip text for this property
   OUTPUT   - 
Static variable in class weka.classifiers.functions.neural. NeuralConnection 
 This unit is an output unit.
   output()   - 
Method in class weka.filters. Filter 
 Output an instance after filtering and remove from the output queue.
   output()   - 
Method in class weka.filters.unsupervised.attribute. RemoveType 
 Output an instance after filtering and remove from the output queue.
   outputCenterFileTipText()   - 
Method in class weka.clusterers. XMeans 
 Returns the tip text for this property.
   outputClassificationTipText()   - 
Method in class weka.filters.supervised.attribute. AddClassification 
 Returns the tip text for this property.
   outputDistributionTipText()   - 
Method in class weka.filters.supervised.attribute. AddClassification 
 Returns the tip text for this property.
   outputErrorFlagTipText()   - 
Method in class weka.filters.supervised.attribute. AddClassification 
 Returns the tip text for this property.
   outputFileName()   - 
Method in class weka.experiment. CSVResultListener 
 Get the value of OutputFileName.
   outputFilenameTipText()   - 
Method in class weka.core.converters. TextDirectoryLoader 
 the tip text for this property
   outputFileTipText()   - 
Method in class weka.experiment. CrossValidationResultProducer 
 Returns the tip text for this property
   outputFileTipText()   - 
Method in class weka.experiment. CSVResultListener 
 Returns the tip text for this property
   outputFileTipText()   - 
Method in class weka.experiment. RandomSplitResultProducer 
 Returns the tip text for this property
   outputFormat()   - 
Method in class weka.gui.streams. InstanceJoiner 
 Gets the format of the output instances.
   outputFormat()   - 
Method in class weka.gui.streams. InstanceLoader 
  
   outputFormat()   - 
Method in interface weka.gui.streams. InstanceProducer 
  
   OutputFormatDialog   - Class in  weka.gui.experiment  A dialog for setting various output format parameters.   OutputFormatDialog(Frame)   - 
Constructor for class weka.gui.experiment. OutputFormatDialog 
 initializes the dialog with the given parent frame.
   outputItemSetsTipText()   - 
Method in class weka.associations. Apriori 
 Returns the tip text for this property
   OutputLogger   - Class in  weka.core.logging  A logger that logs all output on stdout and stderr to a file.   OutputLogger()   - 
Constructor for class weka.core.logging. OutputLogger 
  
   OutputLogger.OutputPrintStream   - Class in  weka.core.logging  A print stream class to capture all data from stdout and stderr.   OutputLogger.OutputPrintStream(OutputLogger, PrintStream)   - 
Constructor for class weka.core.logging. OutputLogger.OutputPrintStream 
 Default constructor.
   outputOffsetMultiplierTipText()   - 
Method in class weka.filters.unsupervised.attribute. InterquartileRange 
 Returns the tip text for this property
   outputPeek()   - 
Method in class weka.filters. Filter 
 Output an instance after filtering but do not remove from the
 output queue.
   outputPeek()   - 
Method in class weka.filters.unsupervised.attribute. RemoveType 
 Output an instance after filtering but do not remove from the
 output queue.
   outputPeek()   - 
Method in class weka.gui.streams. InstanceJoiner 
 Output an instance after filtering but do not remove from the output
 queue.
   outputPeek()   - 
Method in class weka.gui.streams. InstanceLoader 
  
   outputPeek()   - 
Method in interface weka.gui.streams. InstanceProducer 
  
   outputPerClassInfoRetrievalStatsTipText()   - 
Method in class weka.gui.beans. IncrementalClassifierEvaluator 
 Return a tip text string for this property
   outputs(Vector)   - 
Static method in class weka.gui.beans. BeanConnection 
 Returns a vector of BeanInstances that can be considered
 as outputs (or the right-hand side of a sub-flow)
   outputsContains(BeanInstance)   - 
Method in class weka.gui.beans. MetaBean 
  
   outputTipText()   - 
Method in class weka.datagenerators. DataGenerator 
 Returns the tip text for this property
   outputTypeSet(int)   - 
Method in class weka.core. Debug.DBO 
 Return true if the outputtype is set
   outputValue(NeuralNode)   - 
Method in class weka.classifiers.functions.neural. LinearUnit 
 This function calculates what the output value should be.
   outputValue(boolean)   - 
Method in class weka.classifiers.functions.neural. NeuralConnection 
 Call this to get the output value of this unit.
   outputValue(NeuralNode)   - 
Method in interface weka.classifiers.functions.neural. NeuralMethod 
 This function calculates what the output value should be.
   outputValue(boolean)   - 
Method in class weka.classifiers.functions.neural. NeuralNode 
 Call this to get the output value of this unit.
   outputValue(NeuralNode)   - 
Method in class weka.classifiers.functions.neural. SigmoidUnit 
 This function calculates what the output value should be.
   outputWordCountsTipText()   - 
Method in class weka.filters.unsupervised.attribute. StringToWordVector 
 Returns the tip text for this property.
   OutputZipper   - Class in  weka.experiment  OutputZipper writes output to either gzipped files or to a
 multi entry zip file.   OutputZipper(File)   - 
Constructor for class weka.experiment. OutputZipper 
 Constructor.
   OVAL   - 
Static variable in class weka.gui.visualize. VisualizePanelEvent 
  
   overFrequencyThreshold(double)   - 
Method in class weka.associations.tertius. LiteralSet 
 Test if this LiteralSet has more counter-instances than the threshold.
   overFrequencyThreshold(double)   - 
Method in class weka.associations.tertius. Rule 
 Test if this rule is over the frequency threshold.
 
 
    
 P  
 
   p(String)   - 
Static method in class weka.core. Debug.DBO 
 prints out text.
   p()   - 
Method in class weka.core.neighboursearch. CoverTree.CoverTreeNode 
 Returns the instance represented by the node.
   p1evl(double, double[], int)   - 
Static method in class weka.core. Statistics 
 Evaluates the given polynomial of degree  N  at  x .
   pace2(DoubleVector)   - 
Method in class weka.classifiers.functions.pace. ChisqMixture 
 Returns the pace2 estimate of a vector.
   pace4(DoubleVector)   - 
Method in class weka.classifiers.functions.pace. ChisqMixture 
 Returns the pace4 estimate of a vector.
   pace6(double)   - 
Method in class weka.classifiers.functions.pace. ChisqMixture 
 Returns the pace6 estimate of a single value.
   pace6(DoubleVector)   - 
Method in class weka.classifiers.functions.pace. ChisqMixture 
 Returns the pace6 estimate of a vector.
   PaceMatrix   - Class in  weka.classifiers.functions.pace  Class for matrix manipulation used for pace regression.   PaceMatrix(int, int)   - 
Constructor for class weka.classifiers.functions.pace. PaceMatrix 
 Construct an m-by-n PACE matrix of zeros.
   PaceMatrix(int, int, double)   - 
Constructor for class weka.classifiers.functions.pace. PaceMatrix 
 Construct an m-by-n constant PACE matrix.
   PaceMatrix(double[][])   - 
Constructor for class weka.classifiers.functions.pace. PaceMatrix 
 Construct a PACE matrix from a 2-D array.
   PaceMatrix(double[][], int, int)   - 
Constructor for class weka.classifiers.functions.pace. PaceMatrix 
 Construct a PACE matrix quickly without checking arguments.
   PaceMatrix(double[], int)   - 
Constructor for class weka.classifiers.functions.pace. PaceMatrix 
 Construct a PaceMatrix from a one-dimensional packed array
   PaceMatrix(DoubleVector)   - 
Constructor for class weka.classifiers.functions.pace. PaceMatrix 
 Construct a PaceMatrix with a single column from a DoubleVector
   PaceMatrix(Matrix)   - 
Constructor for class weka.classifiers.functions.pace. PaceMatrix 
 Construct a PaceMatrix from a Matrix
   PaceRegression   - Class in  weka.classifiers.functions  Class for building pace regression linear models and using them for prediction.   PaceRegression()   - 
Constructor for class weka.classifiers.functions. PaceRegression 
  
   PACKAGE   - 
Static variable in class weka.core.stemmers. SnowballStemmer 
 the package name for snowball.
   PACKAGE_EXT   - 
Static variable in class weka.core.stemmers. SnowballStemmer 
 the package name where the stemmers are located.
   pad(String, String, int, boolean)   - 
Static method in class weka.core.pmml. PMMLUtils 
 Utility method to left or right pad strings with arbitrary characters.
   PADDING_ZERO   - 
Static variable in class weka.filters.unsupervised.attribute. Wavelet 
 the type of padding: Zero padding
   paddingTipText()   - 
Method in class weka.filters.unsupervised.attribute. Wavelet 
 Returns the tip text for this property
   padLeft(String, int)   - 
Static method in class weka.core. Utils 
 Pads a string to a specified length, inserting spaces on the left
 as required.
   padRight(String, int)   - 
Static method in class weka.core. Utils 
 Pads a string to a specified length, inserting spaces on the right
 as required.
   paint(Graphics)   - 
Method in class weka.gui. SplashWindow 
 Paints the image on the window.
   paintComponent(Graphics)   - 
Method in class weka.gui. AttributeVisualizationPanel 
 Paints this component
   paintComponent(Graphics)   - 
Method in class weka.gui.beans. BeanVisual 
  
   paintComponent(Graphics)   - 
Method in class weka.gui.hierarchyvisualizer. HierarchyVisualizer 
 Updates the screen contents.
   paintComponent(Graphics)   - 
Method in class weka.gui. Main.BackgroundDesktopPane 
 draws the background image.
   paintComponent(Graphics)   - 
Method in class weka.gui. MemoryUsagePanel 
 draws the background image.
   paintComponent(Graphics)   - 
Method in class weka.gui. PropertyPanel 
 Paints the component, using the property editor's paint method.
   paintComponent(Graphics)   - 
Method in class weka.gui.treevisualizer. TreeVisualizer 
 Updates the screen contents.
   paintComponent(Graphics)   - 
Method in class weka.gui.visualize. ClassPanel 
 Renders this component
   paintComponent(Graphics)   - 
Method in class weka.gui.visualize. Plot2D 
 Renders this component
   paintConnections(Graphics)   - 
Static method in class weka.gui.beans. BeanConnection 
 Renders the connections and their names on the supplied graphics
 context
   paintLabels(Graphics)   - 
Static method in class weka.gui.beans. BeanInstance 
 Renders the textual labels for the beans.
   paintValue(Graphics, Rectangle)   - 
Method in class weka.gui. CostMatrixEditor 
 Paints a graphical representation of the object.
   paintValue(Graphics, Rectangle)   - 
Method in class weka.gui. FileEditor 
 Paints a representation of the current Object.
   paintValue(Graphics, Rectangle)   - 
Method in class weka.gui. GenericArrayEditor 
 Paints a representation of the current classifier.
   paintValue(Graphics, Rectangle)   - 
Method in class weka.gui. GenericObjectEditor 
 Paints a representation of the current Object.
   paintValue(Graphics, Rectangle)   - 
Method in class weka.gui. SimpleDateFormatEditor 
 Paints a graphical representation of the object.
   PairedCorrectedTTester   - Class in  weka.experiment  Behaves the same as PairedTTester, only it uses the corrected
 resampled t-test statistic. 

 For more information see: 

  
 Claude Nadeau, Yoshua Bengio (2001).   PairedCorrectedTTester()   - 
Constructor for class weka.experiment. PairedCorrectedTTester 
  
   PairedStats   - Class in  weka.experiment  A class for storing stats on a paired comparison (t-test and correlation)   PairedStats(double)   - 
Constructor for class weka.experiment. PairedStats 
 Creates a new PairedStats object with the supplied significance level.
   PairedStatsCorrected   - Class in  weka.experiment  A class for storing stats on a paired comparison.   PairedStatsCorrected(double, double)   - 
Constructor for class weka.experiment. PairedStatsCorrected 
 Creates a new PairedStatsCorrected object with the supplied
 significance level and train/test ratio.
   PairedTTester   - Class in  weka.experiment  Calculates T-Test statistics on data stored in a set of instances.   PairedTTester()   - 
Constructor for class weka.experiment. PairedTTester 
  
   pairwiseCoupling(double[][], double[][])   - 
Static method in class weka.classifiers.meta. MultiClassClassifier 
 Implements pairwise coupling.
   pairwiseCoupling(double[][], double[][])   - 
Method in class weka.classifiers.mi. MISMO 
 Implements pairwise coupling.
   parentClass   - 
Variable in class weka.experiment. PropertyNode 
 The class of the object with this property
   parentNode()   - 
Method in class weka.classifiers.trees.m5. RuleNode 
 Get the parent of this node
   ParentSet   - Class in  weka.classifiers.bayes.net  Helper class for Bayes Network classifiers.   ParentSet()   - 
Constructor for class weka.classifiers.bayes.net. ParentSet 
 default constructor
   ParentSet(int)   - 
Constructor for class weka.classifiers.bayes.net. ParentSet 
 constructor
   ParentSet(ParentSet)   - 
Constructor for class weka.classifiers.bayes.net. ParentSet 
 copy constructor
   parentTipText()   - 
Method in class weka.datagenerators. ClusterDefinition 
 Returns the tip text for this property
   parentValue()   - 
Method in class weka.gui. HierarchyPropertyParser 
 The value in the parent node.
   parse()   - 
Method in class weka.gui.graphvisualizer. BIFParser 
 This method parses the string or the InputStream that we
 passed in through the constructor and builds up the
 m_nodes and m_edges vectors
   parse()   - 
Method in class weka.gui.graphvisualizer. DotParser 
 This method parses the string or the InputStream that we
 passed in through the constructor and builds up the
 m_nodes and m_edges vectors
   parseDate(String)   - 
Method in class weka.core. Attribute 
 Parses the given String as Date, according to the current format and
 returns the corresponding amount of milliseconds.
   parseMatlab(String)   - 
Static method in class weka.classifiers. CostMatrix 
 creates a matrix from the given Matlab string.
   parseMatlab(String)   - 
Static method in class weka.core.matrix. Matrix 
 creates a matrix from the given Matlab string.
   parseMatlab(String)   - 
Static method in class weka.core. Matrix 
  Deprecated.  creates a matrix from the given Matlab string.
   parsePath(String)   - 
Static method in class weka.core. PropertyPath.Path 
 returns a path object based on the given path string
   Parser   - Class in  weka.core.mathematicalexpression  CUP v0.11a beta 20060608 generated parser.   Parser()   - 
Constructor for class weka.core.mathematicalexpression. Parser 
 Default constructor.
   Parser(Scanner)   - 
Constructor for class weka.core.mathematicalexpression. Parser 
 Constructor which sets the default scanner.
   Parser(Scanner, SymbolFactory)   - 
Constructor for class weka.core.mathematicalexpression. Parser 
 Constructor which sets the default scanner.
   Parser   - Class in  weka.filters.unsupervised.instance.subsetbyexpression  CUP v0.11a beta 20060608 generated parser.   Parser()   - 
Constructor for class weka.filters.unsupervised.instance.subsetbyexpression. Parser 
 Default constructor.
   Parser(Scanner)   - 
Constructor for class weka.filters.unsupervised.instance.subsetbyexpression. Parser 
 Constructor which sets the default scanner.
   Parser(Scanner, SymbolFactory)   - 
Constructor for class weka.filters.unsupervised.instance.subsetbyexpression. Parser 
 Constructor which sets the default scanner.
   PART   - Class in  weka.classifiers.rules  Class for generating a PART decision list.   PART()   - 
Constructor for class weka.classifiers.rules. PART 
  
   PART_PROPERTY   - 
Static variable in class weka.associations.tertius. IndividualLiteral 
  
   partFileTipText()   - 
Method in class weka.associations. Tertius 
 Returns the tip text for this property.
   partition(Instances, int)   - 
Static method in class weka.classifiers.rules. RuleStats 
 Patition the data into 2, first of which has (numFolds-1)/numFolds of
 the data and the second has 1/numFolds of the data
   PartitionedMultiFilter   - Class in  weka.filters.unsupervised.attribute  A filter that applies filters on subsets of attributes and assembles the output into a new dataset.   PartitionedMultiFilter()   - 
Constructor for class weka.filters.unsupervised.attribute. PartitionedMultiFilter 
  
   partitionOptions(String[])   - 
Static method in class weka.classifiers.bayes. BayesNet 
 Returns the secondary set of options (if any) contained in
 the supplied options array.
   partitionOptions(String[])   - 
Static method in class weka.core. Utils 
 Returns the secondary set of options (if any) contained in
 the supplied options array.
   passesTest(Instance)   - 
Method in class weka.datagenerators. Test 
 Determines whether an instance passes the test.
   passwordTipText()   - 
Method in class weka.core.converters. DatabaseLoader 
 the tip text for this property
   passwordTipText()   - 
Method in class weka.core.converters. DatabaseSaver 
 Returns the tip text for this property.
   passwordTipText()   - 
Method in class weka.experiment. DatabaseUtils 
 Returns the tip text for this property.
   paste(String)   - 
Method in class weka.classifiers.bayes.net. EditableBayesNet 
 Apply paste operation with XMLBIF fragment.
   pattern(int, int)   - 
Static method in class weka.core.matrix. FloatingPointFormat 
  
   patternTipText()   - 
Method in class weka.datagenerators.clusterers. BIRCHCluster 
 Returns the tip text for this property
   pchisq(double)   - 
Static method in class weka.core.matrix. Maths 
 Returns the cumulative probability of the Chi-squared distribution
   pchisq(double, double)   - 
Static method in class weka.core.matrix. Maths 
 Returns the cumulative probability of the noncentral Chi-squared
  distribution.
   pchisq(double, DoubleVector)   - 
Static method in class weka.core.matrix. Maths 
 Returns the cumulative probability of a set of noncentral Chi-squared
  distributions.
   pctCorrect()   - 
Method in class weka.classifiers. Evaluation 
 Gets the percentage of instances correctly classified (that is, for
 which a correct prediction was made).
   pctIncorrect()   - 
Method in class weka.classifiers. Evaluation 
 Gets the percentage of instances incorrectly classified (that is, for
 which an incorrect prediction was made).
   pctUnclassified()   - 
Method in class weka.classifiers. Evaluation 
 Gets the percentage of instances not classified (that is, for
 which no prediction was made by the classifier).
   peek()   - 
Method in class weka.core. Queue 
 Gets object from the front of the queue.
   perBag(int)   - 
Method in class weka.classifiers.trees.j48. Distribution 
 Returns number of (possibly fractional) instances in given bag.
   percentageTipText()   - 
Method in class weka.filters.supervised.instance. SMOTE 
 Returns the tip text for this property.
   percentageTipText()   - 
Method in class weka.filters.unsupervised.instance. RemovePercentage 
 Returns the tip text for this property
   percentAttributesUsed()   - 
Method in class weka.classifiers.trees.lmt. LogisticBase 
 Returns the fraction of all attributes in the data that are used in the 
 logistic model (in percent).
   percentThresholdTipText()   - 
Method in class weka.attributeSelection. SVMAttributeEval 
 Returns a tip text for this property suitable for display in the
 GUI
   percentTipText()   - 
Method in class weka.filters.unsupervised.attribute. AddNoise 
 Returns the tip text for this property
   percentTipText()   - 
Method in class weka.filters.unsupervised.attribute. RandomProjection 
 Returns the tip text for this property
   percentToEliminatePerIterationTipText()   - 
Method in class weka.attributeSelection. SVMAttributeEval 
 Returns a tip text for this property suitable for display in the
 GUI
   perClass(int)   - 
Method in class weka.classifiers.trees.j48. Distribution 
 Returns number of (possibly fractional) instances of given class.
   perClassPerBag(int, int)   - 
Method in class weka.classifiers.trees.j48. Distribution 
 Returns number of (possibly fractional) instances of given class in 
 given bag.
   PerformanceStats   - Class in  weka.core.neighboursearch  The class that measures the performance of a nearest
 neighbour search (NNS) algorithm.   PerformanceStats()   - 
Constructor for class weka.core.neighboursearch. PerformanceStats 
 default constructor.
   performPredictionTipText()   - 
Method in class weka.filters.supervised.attribute. PLSFilter 
 Returns the tip text for this property
   performRankingTipText()   - 
Method in class weka.attributeSelection. LinearForwardSelection 
 Returns the tip text for this property
   performRankingTipText()   - 
Method in class weka.attributeSelection. SubsetSizeForwardSelection 
 Returns the tip text for this property
   performRequest(String)   - 
Method in class weka.gui.beans. Associator 
 Perform a particular request
   performRequest(String)   - 
Method in class weka.gui.beans. AttributeSummarizer 
 Perform a named user request
   performRequest(String)   - 
Method in class weka.gui.beans. Classifier 
 Perform a particular request
   performRequest(String)   - 
Method in class weka.gui.beans. ClassifierPerformanceEvaluator 
 Perform the named request
   performRequest(String)   - 
Method in class weka.gui.beans. Clusterer 
 Perform a particular request
   performRequest(String)   - 
Method in class weka.gui.beans. ClustererPerformanceEvaluator 
 Perform the named request
   performRequest(String)   - 
Method in class weka.gui.beans. CostBenefitAnalysis 
  
   performRequest(String)   - 
Method in class weka.gui.beans. CrossValidationFoldMaker 
 Perform the named request
   performRequest(String)   - 
Method in class weka.gui.beans. DataVisualizer 
 Describe  performRequest  method here.
   performRequest(String)   - 
Method in class weka.gui.beans. Filter 
 Perform the named request
   performRequest(String)   - 
Method in class weka.gui.beans. GraphViewer 
 Perform the named request
   performRequest(String)   - 
Method in class weka.gui.beans. MetaBean 
 Perform a particular request
   performRequest(String)   - 
Method in class weka.gui.beans. ModelPerformanceChart 
 Describe  performRequest  method here.
   performRequest(String)   - 
Method in class weka.gui.beans. ScatterPlotMatrix 
 Perform a named user request
   performRequest(String)   - 
Method in class weka.gui.beans. StripChart 
 Describe  performRequest  method here.
   performRequest(String)   - 
Method in class weka.gui.beans. TextViewer 
 Perform the named request
   performRequest(String)   - 
Method in class weka.gui.beans. TrainTestSplitMaker 
 Perform the named request
   performRequest(String)   - 
Method in interface weka.gui.beans. UserRequestAcceptor 
 Perform the named request
   periodicPruningTipText()   - 
Method in class weka.filters.unsupervised.attribute. StringToWordVector 
 Returns the tip text for this property.
   perturbationFractionTipText()   - 
Method in class weka.datagenerators.classifiers.classification. Agrawal 
 Returns the tip text for this property
   phaseIID(int, int[][])   - 
Method in class weka.gui.graphvisualizer. HierarchicalBCEngine 
 See Sugiyama et al.
   phaseIIU(int, int[][])   - 
Method in class weka.gui.graphvisualizer. HierarchicalBCEngine 
 See Sugiyama et al.
   phaseIU(int, int[][])   - 
Method in class weka.gui.graphvisualizer. HierarchicalBCEngine 
 See Sugiyama et al.
   PI   - 
Static variable in class weka.core.xml. XMLDocument 
 the parsing instructions "<?xml version=\"1.0\" encoding=\"utf-8\"?>" 
 (may not show up in Javadoc due to tags!).
   PKIDiscretize   - Class in  weka.filters.unsupervised.attribute  Discretizes numeric attributes using equal frequency binning, where the number of bins is equal to the square root of the number of non-missing values. 
  
 For more information, see: 
  
 Ying Yang, Geoffrey I.   PKIDiscretize()   - 
Constructor for class weka.filters.unsupervised.attribute. PKIDiscretize 
  
   place(Node)   - 
Method in interface weka.gui.treevisualizer. NodePlace 
 The function to call to postion the tree that starts at Node r
   place(Node)   - 
Method in class weka.gui.treevisualizer. PlaceNode1 
 Call this function to have each node in the tree starting at 'r' placed 
 in a visual
 (not logical, they already are) tree position.
   place(Node)   - 
Method in class weka.gui.treevisualizer. PlaceNode2 
 The Funtion to call to have the nodes arranged.
   PlaceNode1   - Class in  weka.gui.treevisualizer  This class will place the Nodes of a tree.   PlaceNode1()   - 
Constructor for class weka.gui.treevisualizer. PlaceNode1 
  
   PlaceNode2   - Class in  weka.gui.treevisualizer  This class will place the Nodes of a tree.   PlaceNode2()   - 
Constructor for class weka.gui.treevisualizer. PlaceNode2 
  
   PLAINTEXT_ENDTAG   - 
Static variable in class weka.core. TechnicalInformationHandlerJavadoc 
 the end comment tag for inserting the generated BibTex
   PLAINTEXT_STARTTAG   - 
Static variable in class weka.core. TechnicalInformationHandlerJavadoc 
 the start comment tag for inserting the generated BibTex
   pln(String)   - 
Static method in class weka.core. Debug.DBO 
 prints out text + endofline.
   Plot2D   - Class in  weka.gui.visualize  This class plots datasets in two dimensions.   Plot2D()   - 
Constructor for class weka.gui.visualize. Plot2D 
 Constructor
   Plot2DCompanion   - Interface in  weka.gui.visualize  Interface for classes that need to draw to the Plot2D panel *before*
 Plot2D renders anything (eg.   PlotData2D   - Class in  weka.gui.visualize  This class is a container for plottable data.   PlotData2D(Instances)   - 
Constructor for class weka.gui.visualize. PlotData2D 
 Construct a new PlotData2D using the supplied instances
   plotTrainingData()   - 
Method in class weka.gui.boundaryvisualizer. BoundaryPanel 
 Render the training points on-screen.
   plotTrainingData()   - 
Method in class weka.gui.boundaryvisualizer. BoundaryVisualizer 
 Plots the training data on-screen.
   PLSClassifier   - Class in  weka.classifiers.functions  A wrapper classifier for the PLSFilter, utilizing the PLSFilter's ability to perform predictions.   PLSClassifier()   - 
Constructor for class weka.classifiers.functions. PLSClassifier 
  
   PLSFilter   - Class in  weka.filters.supervised.attribute  Runs Partial Least Square Regression over the given instances and computes the resulting beta matrix for prediction. 
 By default it replaces missing values and centers the data. 
  
 For more information see: 
  
 Tormod Naes, Tomas Isaksson, Tom Fearn, Tony Davies (2002).   PLSFilter()   - 
Constructor for class weka.filters.supervised.attribute. PLSFilter 
 default constructor
   PLURAL_DUMMY   - 
Static variable in interface weka.gui.graphvisualizer. GraphConstants 
 PLURAL_DUMMY node - node with more than one outgoing edge
 i.e.
   plus(DiscreteFunction)   - 
Method in class weka.classifiers.functions.pace. DiscreteFunction 
 Returns the combined of two discrete functions
   PLUS   - 
Static variable in interface weka.core.mathematicalexpression. sym 
  
   plus(double)   - 
Method in class weka.core.matrix. DoubleVector 
 Adds a value to all the elements
   plus(DoubleVector)   - 
Method in class weka.core.matrix. DoubleVector 
 Adds another vector element by element
   plus(Matrix)   - 
Method in class weka.core.matrix. Matrix 
 C = A + B
   PLUS   - 
Static variable in interface weka.filters.unsupervised.instance.subsetbyexpression. sym 
  
   PLUS_SHAPE   - 
Static variable in class weka.gui.visualize. Plot2D 
  
   plusEquals(DiscreteFunction)   - 
Method in class weka.classifiers.functions.pace. DiscreteFunction 
 Returns the combined of two discrete functions.
   plusEquals(double)   - 
Method in class weka.core.matrix. DoubleVector 
 Adds a value to all the elements in place
   plusEquals(DoubleVector)   - 
Method in class weka.core.matrix. DoubleVector 
 Adds another vector in place element by element
   plusEquals(Matrix)   - 
Method in class weka.core.matrix. Matrix 
 A = A + B
   pmiss   - 
Variable in class weka.classifiers.lazy.kstar. KStarCache.TableEntry 
 transformation probability to missing value
   PMMethod   - 
Static variable in class weka.classifiers.functions.pace. MixtureDistribution 
 The probability-measure-based method
   PMML_FILE_EXTENSION   - 
Static variable in class weka.gui.explorer. ClassifierPanel 
 The filename extension that should be used for PMML xml files
   PMMLClassifier   - Class in  weka.classifiers.pmml.consumer  Abstract base class for all PMML classifiers.   PMMLFactory   - Class in  weka.core.pmml  This class is a factory class for reading/writing PMML models   PMMLFactory()   - 
Constructor for class weka.core.pmml. PMMLFactory 
  
   PMMLModel   - Interface in  weka.core.pmml  Interface for all PMML models   PMMLUtils   - Class in  weka.core.pmml  Utility routines.   PMMLUtils()   - 
Constructor for class weka.core.pmml. PMMLUtils 
  
   PNGWriter   - Class in  weka.gui.visualize  This class takes any JComponent and outputs it to a PNG-file.   PNGWriter()   - 
Constructor for class weka.gui.visualize. PNGWriter 
 initializes the object
   PNGWriter(JComponent)   - 
Constructor for class weka.gui.visualize. PNGWriter 
 initializes the object with the given Component
   PNGWriter(JComponent, File)   - 
Constructor for class weka.gui.visualize. PNGWriter 
 initializes the object with the given Component and filename
   pnorm(double)   - 
Static method in class weka.core.matrix. Maths 
 Returns the cumulative probability of the standard normal.
   pnorm(double, double, double)   - 
Static method in class weka.core.matrix. Maths 
 Returns the cumulative probability of a normal distribution.
   pnorm(double, DoubleVector, double)   - 
Static method in class weka.core.matrix. Maths 
 Returns the cumulative probability of a set of normal distributions
  with different means.
   PointsClosestToFurthestChildren   - Class in  weka.core.neighboursearch.balltrees  Implements the Moore's method to split a node of a ball tree. 
  
 For more information please see section 2 of the 1st and 3.2.3 of the 2nd: 
  
 Andrew W.   PointsClosestToFurthestChildren()   - 
Constructor for class weka.core.neighboursearch.balltrees. PointsClosestToFurthestChildren 
 Constructor.
   PointsClosestToFurthestChildren(int[], Instances, EuclideanDistance)   - 
Constructor for class weka.core.neighboursearch.balltrees. PointsClosestToFurthestChildren 
 Constructor.
   PoissonEstimator   - Class in  weka.estimators  Simple probability estimator that places a single Poisson distribution
 over the observed values.   PoissonEstimator()   - 
Constructor for class weka.estimators. PoissonEstimator 
  
   POLYGON   - 
Static variable in class weka.gui.visualize. VisualizePanelEvent 
  
   PolyKernel   - Class in  weka.classifiers.functions.supportVector  The polynomial kernel : K(x, y) = <x, y>^p or K(x, y) = (<x, y>+1)^p
  
  
 
  
 Valid options are:     PolyKernel()   - 
Constructor for class weka.classifiers.functions.supportVector. PolyKernel 
 default constructor - does nothing.
   PolyKernel(Instances, int, double, boolean)   - 
Constructor for class weka.classifiers.functions.supportVector. PolyKernel 
 Creates a new  PolyKernel  instance.
   pop()   - 
Method in class weka.core.neighboursearch.covertrees. Stack 
 Pops (removes) the first (last added) 
 element in the stack.
   pop()   - 
Method in class weka.core. Queue 
 Pops an object from the front of the queue.
   populationSizeTipText()   - 
Method in class weka.attributeSelection. GeneticSearch 
 Returns the tip text for this property
   populationSizeTipText()   - 
Method in class weka.attributeSelection. ScatterSearchV1 
 Returns the tip text for this property
   populationSizeTipText()   - 
Method in class weka.classifiers.bayes.net.search.global. GeneticSearch 
  
   populationSizeTipText()   - 
Method in class weka.classifiers.bayes.net.search.local. GeneticSearch 
  
   POS   - 
Static variable in class weka.associations.tertius. Literal 
  
   position()   - 
Method in class weka.classifiers.trees.m5. CorrelationSplitInfo 
 Returns the position of the split in the sorted values.
   position()   - 
Method in interface weka.classifiers.trees.m5. SplitEvaluate 
 Returns the position of the split in the sorted values.
   position()   - 
Method in class weka.classifiers.trees.m5. YongSplitInfo 
 Returns the position of the split in the sorted values.
   positive()   - 
Method in class weka.associations.tertius. Literal 
  
   positiveDiagonal(PaceMatrix, IntVector)   - 
Method in class weka.classifiers.functions.pace. PaceMatrix 
 Sets all diagonal elements to be positive (or nonnegative) without
 changing the least squares solution
   positiveIndexTipText()   - 
Method in class weka.associations. FPGrowth 
 Tip text for this property suitable for displaying
 in the GUI.
   positives(int)   - 
Method in class weka.classifiers.trees.j48. GraftSplit 
  
   positivesForSubsetOfInterest()   - 
Method in class weka.classifiers.trees.j48. GraftSplit 
  
   postProcess(int[])   - 
Method in class weka.attributeSelection. ASEvaluation 
 Provides a chance for a attribute evaluator to do any special
 post processing of the selected attribute set.
   postProcess(int[])   - 
Method in class weka.attributeSelection. CfsSubsetEval 
 Calls locallyPredictive in order to include locally predictive
 attributes (if requested).
   postProcess(int[])   - 
Method in class weka.attributeSelection. CostSensitiveASEvaluation 
 Provides a chance for a attribute evaluator to do any special
 post processing of the selected attribute set.
   postProcess(ResultProducer)   - 
Method in class weka.experiment. AveragingResultProducer 
 When this method is called, it indicates that no more results
 will be sent that need to be grouped together in any way.
   postProcess()   - 
Method in class weka.experiment. AveragingResultProducer 
 When this method is called, it indicates that no more requests to
 generate results for the current experiment will be sent.
   postProcess()   - 
Method in class weka.experiment. CrossValidationResultProducer 
 Perform any postprocessing.
   postProcess(ResultProducer)   - 
Method in class weka.experiment. CSVResultListener 
 Perform any postprocessing.
   postProcess(ResultProducer)   - 
Method in class weka.experiment. DatabaseResultListener 
 Perform any postprocessing.
   postProcess(ResultProducer)   - 
Method in class weka.experiment. DatabaseResultProducer 
 When this method is called, it indicates that no more results
 will be sent that need to be grouped together in any way.
   postProcess()   - 
Method in class weka.experiment. DatabaseResultProducer 
 When this method is called, it indicates that no more requests to
 generate results for the current experiment will be sent.
   postProcess()   - 
Method in class weka.experiment. Experiment 
 Signals that the experiment is finished running, so that cleanup
 can be done.
   postProcess(ResultProducer)   - 
Method in class weka.experiment. InstancesResultListener 
 Perform any postprocessing.
   postProcess(ResultProducer)   - 
Method in class weka.experiment. LearningRateResultProducer 
 When this method is called, it indicates that no more results
 will be sent that need to be grouped together in any way.
   postProcess()   - 
Method in class weka.experiment. LearningRateResultProducer 
 When this method is called, it indicates that no more requests to
 generate results for the current experiment will be sent.
   postProcess()   - 
Method in class weka.experiment. RandomSplitResultProducer 
 Perform any postprocessing.
   postProcess()   - 
Method in class weka.experiment. RemoteExperiment 
 overides the one in Experiment
   postProcess(ResultProducer)   - 
Method in interface weka.experiment. ResultListener 
 Perform any postprocessing.
   postProcess()   - 
Method in interface weka.experiment. ResultProducer 
 Perform any postprocessing.
   postProcessDistances(double[])   - 
Method in interface weka.core. DistanceFunction 
 Does post processing of the distances (if necessary) returned by
 distance(distance(Instance first, Instance second, double cutOffValue).
   postProcessDistances(double[])   - 
Method in class weka.core. EuclideanDistance 
 Does post processing of the distances (if necessary) returned by
 distance(distance(Instance first, Instance second, double cutOffValue).
   postProcessDistances(double[])   - 
Method in class weka.core. NormalizableDistance 
 Does nothing, derived classes may override it though.
   PostscriptGraphics   - Class in  weka.gui.visualize  The PostscriptGraphics class extends the Graphics2D class to 
 produce an encapsulated postscript file rather than on-screen display.   PostscriptGraphics(int, int, OutputStream)   - 
Constructor for class weka.gui.visualize. PostscriptGraphics 
 Constructor
 Creates a new PostscriptGraphics object, given dimensions and 
 output file.
   PostscriptWriter   - Class in  weka.gui.visualize  This class takes any Component and outputs it to a Postscript file.   PostscriptWriter()   - 
Constructor for class weka.gui.visualize. PostscriptWriter 
 initializes the object
   PostscriptWriter(JComponent)   - 
Constructor for class weka.gui.visualize. PostscriptWriter 
 initializes the object with the given Component
   PostscriptWriter(JComponent, File)   - 
Constructor for class weka.gui.visualize. PostscriptWriter 
 initializes the object with the given Component and filename
   potential(int, double, double[], double[], boolean)   - 
Method in class weka.classifiers.rules. RuleStats 
 Calculate the potential to decrease DL of the ruleset,
 i.e.
   PotentialClassIgnorer   - Class in  weka.filters.unsupervised.attribute  This filter should be extended by other unsupervised attribute
 filters to allow processing of the class attribute if that's
 required.   PotentialClassIgnorer()   - 
Constructor for class weka.filters.unsupervised.attribute. PotentialClassIgnorer 
  
   POW   - 
Static variable in interface weka.core.mathematicalexpression. sym 
  
   POW   - 
Static variable in interface weka.filters.unsupervised.instance.subsetbyexpression. sym 
  
   precision(int)   - 
Method in class weka.classifiers. Evaluation 
 Calculate the precision with respect to a particular class.
   PRECISION   - 
Static variable in class weka.classifiers.meta. ThresholdSelector 
 precision
   PRECISION_NAME   - 
Static variable in class weka.classifiers.evaluation. ThresholdCurve 
 attribute name: Precision
   PrecomputedKernelMatrixKernel   - Class in  weka.classifiers.functions.supportVector  This kernel is based on a static kernel matrix that is read from a file.   PrecomputedKernelMatrixKernel()   - 
Constructor for class weka.classifiers.functions.supportVector. PrecomputedKernelMatrixKernel 
  
   PreConstructedLinearModel   - Class in  weka.classifiers.trees.m5  This class encapsulates a linear regression function.   PreConstructedLinearModel(double[], double)   - 
Constructor for class weka.classifiers.trees.m5. PreConstructedLinearModel 
 Constructor
   Predicate   - Class in  weka.associations.tertius      Predicate(String, int, boolean)   - 
Constructor for class weka.associations.tertius. Predicate 
  
   predicted()   - 
Method in class weka.classifiers.evaluation. NominalPrediction 
 Gets the predicted class value.
   predicted()   - 
Method in class weka.classifiers.evaluation. NumericPrediction 
 Gets the predicted class value.
   predicted()   - 
Method in interface weka.classifiers.evaluation. Prediction 
 Gets the predicted class value.
   predictInterval(Instance, double)   - 
Method in class weka.classifiers.functions. GaussianProcesses 
 Predicts a confidence interval for the given instance and confidence level.
   predictInterval(Instance, double)   - 
Method in interface weka.classifiers. IntervalEstimator 
 Returns an N*2 array, where N is the number of possible classes, that estimate 
 the boundaries for the confidence interval with a confidence level specified by
 the second parameter.
   Prediction   - Interface in  weka.classifiers.evaluation  Encapsulates a single evaluatable prediction: the predicted value plus the 
 actual class value.   PredictionAppender   - Class in  weka.gui.beans  Bean that can can accept batch or incremental classifier events
 and produce dataset or instance events which contain instances with
 predictions appended.   PredictionAppender()   - 
Constructor for class weka.gui.beans. PredictionAppender 
 Creates a new  PredictionAppender  instance.
   PredictionAppenderBeanInfo   - Class in  weka.gui.beans  Bean info class for PredictionAppender.   PredictionAppenderBeanInfo()   - 
Constructor for class weka.gui.beans. PredictionAppenderBeanInfo 
  
   PredictionAppenderCustomizer   - Class in  weka.gui.beans  GUI Customizer for the prediction appender bean   PredictionAppenderCustomizer()   - 
Constructor for class weka.gui.beans. PredictionAppenderCustomizer 
  
   PredictionNode   - Class in  weka.classifiers.trees.adtree  Class representing a prediction node in an alternating tree.   PredictionNode(double)   - 
Constructor for class weka.classifiers.trees.adtree. PredictionNode 
 Creates a new prediction node.
   predictions()   - 
Method in class weka.classifiers. Evaluation 
 Returns the predictions that have been collected.
   PredictiveApriori   - Class in  weka.associations  Class implementing the predictive apriori algorithm to mine association rules. 
 It searches with an increasing support threshold for the best 'n' rules concerning a support-based corrected confidence value. 
  
 For more information see: 
  
 Tobias Scheffer: Finding Association Rules That Trade Support Optimally against Confidence.   PredictiveApriori()   - 
Constructor for class weka.associations. PredictiveApriori 
 Constructor that allows to sets default values for the 
 minimum confidence and the maximum number of rules
 the minimum confidence.
   predictiveError(Instances)   - 
Method in class weka.classifiers.trees. LADTree 
  
   prefix()   - 
Method in class weka.classifiers.trees.j48. ClassifierTree 
 Returns tree in prefix order.
   prefix()   - 
Method in class weka.classifiers.trees. J48 
 Returns tree in prefix order.
   prefix()   - 
Method in class weka.classifiers.trees. J48graft 
 Returns tree in prefix order.
   prefix()   - 
Method in interface weka.core. Matchable 
 Returns a string that describes a tree representing
 the object in prefix order.
   PREFIX_CLASSIFIER   - 
Static variable in class weka.classifiers.meta. GridSearch 
 the prefix to indicate that the option is for the classifier
   PREFIX_FILTER   - 
Static variable in class weka.classifiers.meta. GridSearch 
 the prefix to indicate that the option is for the filter
   premise()   - 
Method in class weka.associations. RuleItem 
 Gets the premise of a rule
   prePlot(Graphics)   - 
Method in interface weka.gui.visualize. Plot2DCompanion 
 Something to be drawn before the plot itself
   preprocess(Instances, int)   - 
Method in class weka.classifiers.mi. MINND 
 Pre-process the given exemplar according to the other exemplars 
 in the given exemplars.
   preProcess(ResultProducer)   - 
Method in class weka.experiment. AveragingResultProducer 
 Prepare for the results to be received.
   preProcess()   - 
Method in class weka.experiment. AveragingResultProducer 
 Prepare to generate results.
   preProcess()   - 
Method in class weka.experiment. CrossValidationResultProducer 
 Prepare to generate results.
   preProcess(ResultProducer)   - 
Method in class weka.experiment. CSVResultListener 
 Prepare for the results to be received.
   preProcess(ResultProducer)   - 
Method in class weka.experiment. DatabaseResultListener 
 Prepare for the results to be received.
   preProcess(ResultProducer)   - 
Method in class weka.experiment. DatabaseResultProducer 
 Prepare for the results to be received.
   preProcess()   - 
Method in class weka.experiment. DatabaseResultProducer 
 Prepare to generate results.
   preProcess(ResultProducer)   - 
Method in class weka.experiment. InstancesResultListener 
 Prepare for the results to be received.
   preProcess(ResultProducer)   - 
Method in class weka.experiment. LearningRateResultProducer 
 Prepare for the results to be received.
   preProcess()   - 
Method in class weka.experiment. LearningRateResultProducer 
 Prepare to generate results.
   preProcess()   - 
Method in class weka.experiment. RandomSplitResultProducer 
 Prepare to generate results.
   preProcess(ResultProducer)   - 
Method in interface weka.experiment. ResultListener 
 Prepare for the results to be received.
   preProcess()   - 
Method in interface weka.experiment. ResultProducer 
 Prepare to generate results.
   preprocessData()   - 
Method in class weka.classifiers.mi. CitationKNN 
 Calculates the normalization of each attribute.
   PREPROCESSING_CENTER   - 
Static variable in class weka.filters.supervised.attribute. PLSFilter 
 the type of preprocessing: Center
   PREPROCESSING_NONE   - 
Static variable in class weka.filters.supervised.attribute. PLSFilter 
 the type of preprocessing: None
   PREPROCESSING_STANDARDIZE   - 
Static variable in class weka.filters.supervised.attribute. PLSFilter 
 the type of preprocessing: Standardize
   preprocessingTipText()   - 
Method in class weka.filters.supervised.attribute. PLSFilter 
 Returns the tip text for this property
   preprocessingTipText()   - 
Method in class weka.filters.unsupervised.attribute. KernelFilter 
 Returns the tip text for this property
   PreprocessPanel   - Class in  weka.gui.explorer  This panel controls simple preprocessing of instances.   PreprocessPanel()   - 
Constructor for class weka.gui.explorer. PreprocessPanel 
 Creates the instances panel with no initial instances.
   preserveInstancesOrderTipText()   - 
Method in class weka.clusterers. SimpleKMeans 
 Returns the tip text for this property
   previous()   - 
Method in class weka.associations.tertius. SimpleLinkedList.LinkedListInverseIterator 
  
   PrincipalComponents   - Class in  weka.attributeSelection  Performs a principal components analysis and transformation of the data.   PrincipalComponents()   - 
Constructor for class weka.attributeSelection. PrincipalComponents 
  
   PrincipalComponents   - Class in  weka.filters.unsupervised.attribute  Performs a principal components analysis and transformation of the data. 
 Dimensionality reduction is accomplished by choosing enough eigenvectors to account for some percentage of the variance in the original data -- default 0.95 (95%). 
 Based on code of the attribute selection scheme 'PrincipalComponents' by Mark Hall and Gabi Schmidberger.   PrincipalComponents()   - 
Constructor for class weka.filters.unsupervised.attribute. PrincipalComponents 
  
   print()   - 
Method in class weka.classifiers.bayes.net. ADNode 
 print is used for debugging only and shows the ADTree in ASCII graphics
   print(String)   - 
Method in class weka.classifiers.bayes.net. VaryNode 
 print is used for debugging only, called from ADNode
   print(int)   - 
Method in class weka.core.logging. OutputLogger.OutputPrintStream 
 prints the given int to the streams.
   print(boolean)   - 
Method in class weka.core.logging. OutputLogger.OutputPrintStream 
 prints the given boolean to the streams.
   print(String)   - 
Method in class weka.core.logging. OutputLogger.OutputPrintStream 
 prints the given string to the streams.
   print(Object)   - 
Method in class weka.core.logging. OutputLogger.OutputPrintStream 
 prints the given object to the streams.
   print(int, int)   - 
Method in class weka.core.matrix. Matrix 
 Print the matrix to stdout.
   print(PrintWriter, int, int)   - 
Method in class weka.core.matrix. Matrix 
 Print the matrix to the output stream.
   print(NumberFormat, int)   - 
Method in class weka.core.matrix. Matrix 
 Print the matrix to stdout.
   print(PrintWriter, NumberFormat, int)   - 
Method in class weka.core.matrix. Matrix 
 Print the matrix to the output stream.
   print(int)   - 
Method in class weka.core. Tee 
 prints the given int to the streams.
   print(long)   - 
Method in class weka.core. Tee 
 prints the given long to the streams.
   print(float)   - 
Method in class weka.core. Tee 
 prints the given float to the streams.
   print(double)   - 
Method in class weka.core. Tee 
 prints the given double to the streams.
   print(boolean)   - 
Method in class weka.core. Tee 
 prints the given boolean to the streams.
   print(char)   - 
Method in class weka.core. Tee 
 prints the given char to the streams.
   print(char[])   - 
Method in class weka.core. Tee 
 prints the given char array to the streams.
   print(String)   - 
Method in class weka.core. Tee 
 prints the given string to the streams.
   print(Object)   - 
Method in class weka.core. Tee 
 prints the given object to the streams.
   print()   - 
Method in class weka.core.xml. XMLDocument 
 prints the current DOM document to standard out.
   print_hash_code()   - 
Method in class weka.attributeSelection. ConsistencySubsetEval.hashKey 
 Prints the hash code
   print_hash_code()   - 
Method in class weka.classifiers.rules. DecisionTableHashKey 
 Prints the hash code
   PrintableComponent   - Class in  weka.gui.visualize  This class extends the component which is handed over in the constructor
 by a print dialog.   PrintableComponent(JComponent)   - 
Constructor for class weka.gui.visualize. PrintableComponent 
 initializes the panel.
   PrintableHandler   - Interface in  weka.gui.visualize  This interface is for all JComponent classes that provide the ability to 
 print itself to a file.   PrintablePanel   - Class in  weka.gui.visualize  This Panel enables the user to print the panel to various file formats.   PrintablePanel()   - 
Constructor for class weka.gui.visualize. PrintablePanel 
 initializes the panel
   printAllModels()   - 
Method in class weka.classifiers.trees.m5. RuleNode 
 Print all the linear models at the learf (debugging purposes)
   printClassifications(Classifier, Instances, ConverterUtils.DataSource, int, Range, StringBuffer)   - 
Static method in class weka.classifiers. Evaluation 
 Prints the predictions for the given dataset into a String variable.
   printClassifications(Classifier, Instances, ConverterUtils.DataSource, int, Range, boolean, StringBuffer)   - 
Static method in class weka.classifiers. Evaluation 
 Prints the predictions for the given dataset into a supplied StringBuffer
   printElements()   - 
Method in class weka.classifiers.functions.supportVector. SMOset 
 Prints all the current elements in the set.
   printFeatures()   - 
Method in class weka.classifiers.rules. DecisionTable 
 Returns a string description of the features selected
   printInsts(int, int)   - 
Method in class weka.core.neighboursearch.balltrees. MiddleOutConstructor 
 For printing indices in some given portion
 of the master index array.
   printLeafModels()   - 
Method in class weka.classifiers.trees.j48. NBTreeClassifierTree 
 Print the models at the leaves
   printLeafModels()   - 
Method in class weka.classifiers.trees.m5. RuleNode 
 print all leaf models
   printList(MiddleOutConstructor.MyIdxList)   - 
Method in class weka.core.neighboursearch.balltrees. MiddleOutConstructor 
 For printing indices in a given point list.
   println()   - 
Method in class weka.core.logging. OutputLogger.OutputPrintStream 
 prints a new line to the streams.
   println(int)   - 
Method in class weka.core.logging. OutputLogger.OutputPrintStream 
 prints the given int to the streams.
   println(boolean)   - 
Method in class weka.core.logging. OutputLogger.OutputPrintStream 
 prints the given boolean to the streams.
   println(String)   - 
Method in class weka.core.logging. OutputLogger.OutputPrintStream 
 prints the given string to the streams.
   println(Object)   - 
Method in class weka.core.logging. OutputLogger.OutputPrintStream 
 prints the given object to the streams (for Throwables we print the stack
 trace).
   println()   - 
Method in class weka.core. Tee 
 prints a new line to the streams.
   println(int)   - 
Method in class weka.core. Tee 
 prints the given int to the streams.
   println(long)   - 
Method in class weka.core. Tee 
 prints the given long to the streams.
   println(float)   - 
Method in class weka.core. Tee 
 prints the given float to the streams.
   println(double)   - 
Method in class weka.core. Tee 
 prints the given double to the streams.
   println(boolean)   - 
Method in class weka.core. Tee 
 prints the given boolean to the streams.
   println(char)   - 
Method in class weka.core. Tee 
 prints the given char to the streams.
   println(char[])   - 
Method in class weka.core. Tee 
 prints the given char array to the streams.
   println(String)   - 
Method in class weka.core. Tee 
 prints the given string to the streams.
   println(Object)   - 
Method in class weka.core. Tee 
 prints the given object to the streams (for Throwables we print the stack
 trace).
   printNewickTipText()   - 
Method in class weka.clusterers. HierarchicalClusterer 
  
   printNodeLinearModel()   - 
Method in class weka.classifiers.trees.m5. RuleNode 
 print the linear model at this node
   printSetOfSequences(FastVector)   - 
Static method in class weka.associations.gsp. Sequence 
 Prints a set of Sequences as String output.
   printStackTrace()   - 
Method in class weka.core. Debug.Random 
 prints the current stacktrace
   printSubset(ScatterSearchV1.Subset)   - 
Method in class weka.attributeSelection. ScatterSearchV1 
  
   Prior   - Class in  weka.classifiers.bayes.blr  This is an interface to plug various priors into
 the Bayesian Logistic Regression Model.   Prior()   - 
Constructor for class weka.classifiers.bayes.blr. Prior 
  
   PriorClass   - 
Variable in class weka.classifiers.bayes. BayesianLogisticRegression 
 Distribution Prior class
   priorClassTipText()   - 
Method in class weka.classifiers.bayes. BayesianLogisticRegression 
 Returns the tip text for this property
   priorEntropy()   - 
Method in class weka.classifiers. Evaluation 
 Calculate the entropy of the prior distribution
   PriorEstimation   - Class in  weka.associations  Class implementing the prior estimattion of the predictive apriori algorithm 
 for mining association rules.   PriorEstimation(Instances, int, int, boolean)   - 
Constructor for class weka.associations. PriorEstimation 
 Constructor
   PriorityQueue   - Class in  weka.clusterers.forOPTICSAndDBScan.Utils  
 PriorityQueue.java  
 Authors: Rainer Holzmann, Zhanna Melnikova-Albrecht, Matthias Schubert  
 Date: Aug 27, 2004  
 Time: 5:36:35 PM  
 $ Revision 1.4 $     PriorityQueue()   - 
Constructor for class weka.clusterers.forOPTICSAndDBScan.Utils. PriorityQueue 
 Creates a new PriorityQueue backed on a binary heap.
   PriorityQueueElement   - Class in  weka.clusterers.forOPTICSAndDBScan.Utils  
 PriorityQueueElement.java  
 Authors: Rainer Holzmann, Zhanna Melnikova-Albrecht, Matthias Schubert  
 Date: Aug 31, 2004  
 Time: 6:43:18 PM  
 $ Revision 1.4 $     PriorityQueueElement(double, Object)   - 
Constructor for class weka.clusterers.forOPTICSAndDBScan.Utils. PriorityQueueElement 
  
   Prism   - Class in  weka.classifiers.rules  Class for building and using a PRISM rule set for classification.   Prism()   - 
Constructor for class weka.classifiers.rules. Prism 
  
   prob(int)   - 
Method in class weka.classifiers.trees.j48. Distribution 
 Returns relative frequency of class over all bags.
   prob(int, int)   - 
Method in class weka.classifiers.trees.j48. Distribution 
 Returns relative frequency of class for given bag.
   PROB_COST_FUNC_NAME   - 
Static variable in class weka.classifiers.evaluation. CostCurve 
 attribute name: Probability Cost Function
   probabilityEstimatesTipText()   - 
Method in class weka.classifiers.functions. LibLINEAR 
 Returns the tip text for this property
   probabilityEstimatesTipText()   - 
Method in class weka.classifiers.functions. LibSVM 
 Returns the tip text for this property
   probabilityMatrix(DoubleVector, PaceMatrix)   - 
Method in class weka.classifiers.functions.pace. ChisqMixture 
 Contructs the probability matrix for mixture estimation, given a set
  of support points and a set of intervals.
   probabilityMatrix(DoubleVector, PaceMatrix)   - 
Method in class weka.classifiers.functions.pace. MixtureDistribution 
 Contructs the probability matrix for mixture estimation, given a set
  of support points and a set of intervals.
   probabilityMatrix(DoubleVector, PaceMatrix)   - 
Method in class weka.classifiers.functions.pace. NormalMixture 
 Contructs the probability matrix for mixture estimation, given a set
  of support points and a set of intervals.
   probRound(double, Random)   - 
Static method in class weka.core. Utils 
 Rounds a double to the next nearest integer value in a probabilistic
 fashion (e.g.
   probToLogOdds(double)   - 
Static method in class weka.core. Utils 
 Returns the log-odds for a given probabilitiy.
   process(boolean[][], BayesNet)   - 
Method in class weka.classifiers.bayes.net. MarginCalculator 
  
   process(Instances)   - 
Method in class weka.core. CheckScheme.PostProcessor 
 Provides a hook for derived classes to further modify the data.
   processClassifierPrediction(Instance, Classifier, Evaluation, Instances, FastVector, FastVector)   - 
Static method in class weka.gui.explorer. ClassifierPanel 
 Process a classifier's prediction for an instance and update a
 set of plotting instances and additional plotting info.
   processColour(String, Color)   - 
Static method in class weka.gui.visualize. VisualizeUtils 
 Parses a string containing either a named colour or r,g,b values.
   processFile(String)   - 
Method in class weka.classifiers.bayes.net. BIFReader 
 processFile reads a BIFXML file and initializes a Bayes Net
   processFilename(String)   - 
Method in class weka.gui. Loader 
 returns the processed filename, i.e.
   PROCESSING   - 
Static variable in class weka.experiment. TaskStatusInfo 
  
   processKeyString(String)   - 
Static method in class weka.experiment. DatabaseUtils 
 processes the string in such a way that it can be stored in the
 database, i.e., it changes backslashes into slashes and doubles single 
 quotes.
   processString(String)   - 
Method in class weka.classifiers.bayes.net. BIFReader 
  
   PRODUCT_RULE   - 
Static variable in class weka.classifiers.meta. Vote 
 combination rule: Product of Probabilities (only nominal classes)
   production_table()   - 
Method in class weka.core.mathematicalexpression. Parser 
 Access to production table.
   production_table()   - 
Method in class weka.filters.unsupervised.instance.subsetbyexpression. Parser 
 Access to production table.
   projectionFilterTipText()   - 
Method in class weka.classifiers.meta. RotationForest 
 Returns the tip text for this property
   PROPERTIES_FILE   - 
Static variable in class weka.core. Capabilities 
 the properties file for managing the tests
   PROPERTIES_FILE   - 
Static variable in class weka.core.logging. Logger 
 the properties file.
   PROPERTIES_FILE   - 
Static variable in class weka.gui.treevisualizer. TreeVisualizer 
 the props file.
   property   - 
Variable in class weka.experiment. PropertyNode 
 Other info about the property
   PROPERTY_FILE   - 
Static variable in class weka.core. Copyright 
 the copyright file
   PROPERTY_FILE   - 
Static variable in class weka.experiment. DatabaseUtils 
 The name of the properties file.
   PROPERTY_FILE   - 
Static variable in class weka.gui.experiment. ExperimenterDefaults 
 The name of the properties file
   PROPERTY_FILE   - 
Static variable in class weka.gui.explorer. ExplorerDefaults 
 The name of the properties file.
   PROPERTY_FILE   - 
Static variable in class weka.gui. LookAndFeel 
 The name of the properties file
   propertyChange(PropertyChangeEvent)   - 
Method in class weka.gui.beans. KnowledgeFlowApp 
 Accept property change events
   propertyChange(PropertyChangeEvent)   - 
Method in class weka.gui. PropertySheetPanel 
 Updates the property sheet panel with a changed property and also passed
 the event along.
   PropertyDialog   - Class in  weka.gui  Support for PropertyEditors with custom editors: puts the editor into
 a separate frame.   PropertyDialog(PropertyEditor, int, int)   - 
Constructor for class weka.gui. PropertyDialog 
  Deprecated.   instead of this constructor, one should use the constructors
                        with an explicit owner (either derived from
                         java.awt.Dialog  or from
                         java.awt.Frame ) or, if none available,
                        using  (Frame) null  as owner. 
   PropertyDialog(Dialog, PropertyEditor)   - 
Constructor for class weka.gui. PropertyDialog 
 Creates the (screen-centered) editor dialog.
   PropertyDialog(Dialog, PropertyEditor, int, int)   - 
Constructor for class weka.gui. PropertyDialog 
 Creates the editor dialog at the given position.
   PropertyDialog(Frame, PropertyEditor)   - 
Constructor for class weka.gui. PropertyDialog 
 Creates the (screen-centered) editor dialog.
   PropertyDialog(Frame, PropertyEditor, int, int)   - 
Constructor for class weka.gui. PropertyDialog 
 Creates the editor dialog at the given position.
   PropertyHandler   - Class in  weka.core.xml  This class stores information about properties to ignore or properties
 that are allowed for a certain class.   PropertyHandler()   - 
Constructor for class weka.core.xml. PropertyHandler 
 initializes the handling
   PropertyNode   - Class in  weka.experiment  Stores information on a property of an object: the class of the
 object with the property; the property descriptor, and the current
 value.   PropertyNode(Object)   - 
Constructor for class weka.experiment. PropertyNode 
 Creates a mostly empty property.
   PropertyNode(Object, PropertyDescriptor, Class)   - 
Constructor for class weka.experiment. PropertyNode 
 Creates a fully specified property node.
   PropertyPanel   - Class in  weka.gui  Support for drawing a property value in a component.   PropertyPanel(PropertyEditor)   - 
Constructor for class weka.gui. PropertyPanel 
 Create the panel with the supplied property editor.
   PropertyPanel(PropertyEditor, boolean)   - 
Constructor for class weka.gui. PropertyPanel 
 Create the panel with the supplied property editor,
 optionally ignoring any custom panel the editor can provide.
   PropertyPath   - Class in  weka.core  A helper class for accessing properties in nested objects, e.g., accessing
 the "getRidge" method of a LinearRegression classifier part of 
 MultipleClassifierCombiner, e.g., Vote.   PropertyPath()   - 
Constructor for class weka.core. PropertyPath 
  
   PropertyPath.Path   - Class in  weka.core  Contains a (property) path structure   PropertyPath.Path(String)   - 
Constructor for class weka.core. PropertyPath.Path 
 uses the given dot-path
   PropertyPath.Path(Vector)   - 
Constructor for class weka.core. PropertyPath.Path 
 uses the vector with PathElement objects to initialize with
   PropertyPath.Path(String[])   - 
Constructor for class weka.core. PropertyPath.Path 
 uses the given array as elements for the path
   PropertyPath.PathElement   - Class in  weka.core  Represents a single element of a property path   PropertyPath.PathElement(String)   - 
Constructor for class weka.core. PropertyPath.PathElement 
 initializes the path element with the given property
   PropertySelectorDialog   - Class in  weka.gui  Allows the user to select any (supported) property of an object, including
 properties that any of it's property values may have.   PropertySelectorDialog(Frame, Object)   - 
Constructor for class weka.gui. PropertySelectorDialog 
 Create the property selection dialog.
   PropertySheetPanel   - Class in  weka.gui  Displays a property sheet where (supported) properties of the target
 object may be edited.   PropertySheetPanel()   - 
Constructor for class weka.gui. PropertySheetPanel 
 Creates the property sheet panel.
   PropositionalToMultiInstance   - Class in  weka.filters.unsupervised.attribute  Converts the propositional instance dataset into multi-instance dataset (with relational attribute).   PropositionalToMultiInstance()   - 
Constructor for class weka.filters.unsupervised.attribute. PropositionalToMultiInstance 
  
   ProtectedProperties   - Class in  weka.core  Simple class that extends the Properties class so that the properties are
 unable to be modified.   ProtectedProperties(Properties)   - 
Constructor for class weka.core. ProtectedProperties 
 Creates a set of protected properties from a set of normal ones.
   prune()   - 
Method in class weka.classifiers.trees.ft. FTInnerNode 
 Prunes a tree using C4.5 pruning procedure.
   prune()   - 
Method in class weka.classifiers.trees.ft. FTLeavesNode 
 Prunes a tree using C4.5 pruning procedure.
   prune()   - 
Method in class weka.classifiers.trees.ft. FTNode 
 Method for prunning a tree using C4.5 pruning procedure.
   prune()   - 
Method in class weka.classifiers.trees.ft. FTtree 
 Abstract Method that prunes a tree using C4.5 pruning procedure.
   prune()   - 
Method in class weka.classifiers.trees.j48. C45PruneableClassifierTree 
 Prunes a tree using C4.5's pruning procedure.
   prune()   - 
Method in class weka.classifiers.trees.j48. C45PruneableClassifierTreeG 
 Prunes a tree using C4.5's pruning procedure.
   prune()   - 
Method in class weka.classifiers.trees.j48. PruneableClassifierTree 
 Prunes a tree.
   prune(double)   - 
Method in class weka.classifiers.trees.lmt. LMTNode 
 Prunes a logistic model tree using the CART pruning scheme, given a 
 cost-complexity parameter alpha.
   prune(double[], double[], Instances)   - 
Method in class weka.classifiers.trees.lmt. LMTNode 
 Method for performing one fold in the cross-validation of the cost-complexity parameter.
   prune()   - 
Method in class weka.classifiers.trees.m5. RuleNode 
 Recursively prune the tree
   prune(double)   - 
Method in class weka.classifiers.trees. SimpleCart 
 Prunes the original tree using the CART pruning scheme, given a 
 cost-complexity parameter alpha.
   prune(double[], double[], Instances)   - 
Method in class weka.classifiers.trees. SimpleCart 
 Method for performing one fold in the cross-validation of minimal 
 cost-complexity pruning.
   PruneableClassifierTree   - Class in  weka.classifiers.trees.j48  Class for handling a tree structure that can
 be pruned using a pruning set.   PruneableClassifierTree(ModelSelection, boolean, int, boolean, int)   - 
Constructor for class weka.classifiers.trees.j48. PruneableClassifierTree 
 Constructor for pruneable tree structure.
   PruneableDecList   - Class in  weka.classifiers.rules.part  Class for handling a partial tree structure that
 can be pruned using a pruning set.   PruneableDecList(ModelSelection, int)   - 
Constructor for class weka.classifiers.rules.part. PruneableDecList 
 Constructor for pruneable partial tree structure.
   pruneItemSets(FastVector, Hashtable)   - 
Static method in class weka.associations. ItemSet 
 Prunes a set of (k)-item sets using the given (k-1)-item sets.
   pruneItemSets(FastVector, Hashtable)   - 
Static method in class weka.associations. LabeledItemSet 
 Prunes a set of (k)-item sets using the given (k-1)-item sets.
   pruneRules(List<FPGrowth.AssociationRule>, ArrayList<Attribute>, boolean)   - 
Static method in class weka.associations. FPGrowth.AssociationRule 
  
   pruneRules(FastVector[], double)   - 
Static method in class weka.associations. ItemSet 
 Prunes a set of rules.
   pruneToK(Instances, double[], int)   - 
Method in class weka.classifiers.lazy. IBk 
 Prunes the list to contain the k nearest neighbors.
   PRUNETYPE_LOGLIKELIHOOD   - 
Static variable in class weka.classifiers.meta. RacedIncrementalLogitBoost 
 log likelihood pruning
   PRUNETYPE_NONE   - 
Static variable in class weka.classifiers.meta. RacedIncrementalLogitBoost 
 no pruning
   PRUNING_LAMBDA   - 
Static variable in class weka.classifiers.functions.supportVector. StringKernel 
 Pruning method: Lambda See [2] for details.
   PRUNING_NONE   - 
Static variable in class weka.classifiers.functions.supportVector. StringKernel 
 Pruning method: No Pruning
   PRUNING_POSTPRUNING   - 
Static variable in class weka.classifiers.trees. BFTree 
 pruning strategy: post-pruning
   PRUNING_PREPRUNING   - 
Static variable in class weka.classifiers.trees. BFTree 
 pruning strategy: pre-pruning
   PRUNING_UNPRUNED   - 
Static variable in class weka.classifiers.trees. BFTree 
 pruning strategy: un-pruned
   pruningMethodTipText()   - 
Method in class weka.classifiers.functions.supportVector. StringKernel 
 Returns the tip text for this property
   pruningStrategyTipText()   - 
Method in class weka.classifiers.trees. BFTree 
 Returns the tip text for this property
   pruningTypeTipText()   - 
Method in class weka.classifiers.meta. RacedIncrementalLogitBoost 
  
   PSI   - 
Static variable in class weka.core.matrix. Maths 
 The constant 1 / sqrt(2 pi)
   Puk   - Class in  weka.classifiers.functions.supportVector  The Pearson VII function-based universal kernel. 
  
 For more information see: 
  
 B.   Puk()   - 
Constructor for class weka.classifiers.functions.supportVector. Puk 
 default constructor - does nothing.
   Puk(Instances, int, double, double)   - 
Constructor for class weka.classifiers.functions.supportVector. Puk 
 Constructor.
   PURE_INPUT   - 
Static variable in class weka.classifiers.functions.neural. NeuralConnection 
 This unit is a pure input unit.
   PURE_OUTPUT   - 
Static variable in class weka.classifiers.functions.neural. NeuralConnection 
 This unit is a pure output unit.
   push(T)   - 
Method in class weka.core.neighboursearch.covertrees. Stack 
 Pushes the given element to the stack.
   push(Stack<T>, T)   - 
Method in class weka.core.neighboursearch.covertrees. Stack 
 Pushes the given element onto the given 
 stack.
   push(Object)   - 
Method in class weka.core. Queue 
 Appends an object to the back of the queue.
   put(Object, Object)   - 
Method in class weka.core. ProtectedProperties 
 Overrides a method to prevent the properties from being modified.
   putAll(Map)   - 
Method in class weka.core. ProtectedProperties 
 Overrides a method to prevent the properties from being modified.
   putResultInTable(String, ResultProducer, Object[], Object[])   - 
Method in class weka.experiment. DatabaseUtils 
 Executes a database query to insert a result for the supplied key
 into the database.
 
 
    
 Q  
 
   qr()   - 
Method in class weka.core.matrix. Matrix 
 QR Decomposition
   QRDecomposition   - Class in  weka.core.matrix  QR Decomposition.   QRDecomposition(Matrix)   - 
Constructor for class weka.core.matrix. QRDecomposition 
 QR Decomposition, computed by Householder reflections.
   queryExecuted(QueryExecuteEvent)   - 
Method in interface weka.gui.sql.event. QueryExecuteListener 
 This method gets called when a query has been executed.
   queryExecuted(QueryExecuteEvent)   - 
Method in class weka.gui.sql. ResultPanel 
 This method gets called when a query has been executed.
   queryExecuted(QueryExecuteEvent)   - 
Method in class weka.gui.sql. SqlViewer 
 This method gets called when a query has been executed.
   QueryExecuteEvent   - Class in  weka.gui.sql.event  An event that is generated when a query is executed.   QueryExecuteEvent(Object, DbUtils, String, int, ResultSet, Exception)   - 
Constructor for class weka.gui.sql.event. QueryExecuteEvent 
 constructs the event
   QueryExecuteListener   - Interface in  weka.gui.sql.event  A listener for executing queries.   QueryPanel   - Class in  weka.gui.sql  Represents a panel for entering an SQL query.   QueryPanel(JFrame)   - 
Constructor for class weka.gui.sql. QueryPanel 
 initializes the panel.
   queryTipText()   - 
Method in class weka.core.converters. DatabaseLoader 
 the tip text for this property
   queryTipText()   - 
Method in class weka.experiment. InstanceQuery 
 Returns the tip text for this property
   Queue   - Class in  weka.core  Class representing a FIFO queue.   Queue()   - 
Constructor for class weka.core. Queue 
  
   quickSort(double[], double[], int, int)   - 
Static method in class weka.core.neighboursearch. NearestNeighbourSearch 
 performs quicksort.
   quote(String)   - 
Static method in class weka.core. Utils 
 Quotes a string if it contains special characters.
 
 
    
 R  
 
   R   - 
Variable in class weka.classifiers.bayes. BayesianLogisticRegression 
 R(i)= BetaVector X x(i) X y(i).
   R_HIGH   - 
Static variable in class weka.clusterers. XMeans 
 Index in ranges for HIGH.
   R_LOW   - 
Static variable in class weka.clusterers. XMeans 
 Index in ranges for LOW.
   R_MAX   - 
Static variable in class weka.core. NormalizableDistance 
 Index in ranges for MAX.
   R_MIN   - 
Static variable in class weka.core. NormalizableDistance 
 Index in ranges for MIN.
   R_WIDTH   - 
Static variable in class weka.clusterers. XMeans 
 Index in ranges for WIDTH.
   R_WIDTH   - 
Static variable in class weka.core. NormalizableDistance 
 Index in ranges for WIDTH.
   RacedIncrementalLogitBoost   - Class in  weka.classifiers.meta  Classifier for incremental learning of large datasets by way of racing logit-boosted committees. 
  
 For more information see: 
  
 Eibe Frank, Geoffrey Holmes, Richard Kirkby, Mark Hall:  Racing committees for large datasets.   RacedIncrementalLogitBoost()   - 
Constructor for class weka.classifiers.meta. RacedIncrementalLogitBoost 
 Constructor.
   RaceSearch   - Class in  weka.attributeSelection  Races the cross validation error of competing attribute subsets.   RaceSearch()   - 
Constructor for class weka.attributeSelection. RaceSearch 
  
   raceTypeTipText()   - 
Method in class weka.attributeSelection. RaceSearch 
 Returns the tip text for this property
   randEntropy   - 
Variable in class weka.classifiers.lazy.kstar. KStarWrapper 
 used/reused to hold the random entropy
   random(int)   - 
Static method in class weka.core.matrix. DoubleVector 
 Returns a random vector of uniform distribution
   random(int, int)   - 
Static method in class weka.core.matrix. Matrix 
 Generate matrix with random elements
   RANDOM   - 
Static variable in class weka.datagenerators.clusterers. BIRCHCluster 
 Constant set for choice of pattern.
   RANDOM   - 
Static variable in class weka.filters.supervised.attribute. ClassOrder 
 The class values are sorted in random order
   randomCARule(int, int, Random)   - 
Method in class weka.associations. PriorEstimation 
 Constructs an item set of certain length randomly.
   RandomCommittee   - Class in  weka.classifiers.meta  Class for building an ensemble of randomizable base classifiers.   RandomCommittee()   - 
Constructor for class weka.classifiers.meta. RandomCommittee 
 Constructor.
   RandomForest   - Class in  weka.classifiers.trees  Class for constructing a forest of random trees. 
  
 For more information see:  
  
 Leo Breiman (2001).   RandomForest()   - 
Constructor for class weka.classifiers.trees. RandomForest 
  
   Randomizable   - Interface in  weka.core  Interface to something that has random behaviour that is able to be
 seeded with an integer.   RandomizableClassifier   - Class in  weka.classifiers  Abstract utility class for handling settings common to randomizable
 classifiers.   RandomizableClassifier()   - 
Constructor for class weka.classifiers. RandomizableClassifier 
  
   RandomizableClusterer   - Class in  weka.clusterers  Abstract utility class for handling settings common to randomizable
 clusterers.   RandomizableClusterer()   - 
Constructor for class weka.clusterers. RandomizableClusterer 
  
   RandomizableDensityBasedClusterer   - Class in  weka.clusterers  Abstract utility class for handling settings common to randomizable
 clusterers.   RandomizableDensityBasedClusterer()   - 
Constructor for class weka.clusterers. RandomizableDensityBasedClusterer 
  
   RandomizableIteratedSingleClassifierEnhancer   - Class in  weka.classifiers  Abstract utility class for handling settings common to randomizable
 meta classifiers that build an ensemble from a single base learner.   RandomizableIteratedSingleClassifierEnhancer()   - 
Constructor for class weka.classifiers. RandomizableIteratedSingleClassifierEnhancer 
  
   RandomizableMultipleClassifiersCombiner   - Class in  weka.classifiers  Abstract utility class for handling settings common to randomizable
 meta classifiers that build an ensemble from multiple classifiers based
 on a given random number seed.   RandomizableMultipleClassifiersCombiner()   - 
Constructor for class weka.classifiers. RandomizableMultipleClassifiersCombiner 
  
   RandomizableSingleClassifierEnhancer   - Class in  weka.classifiers  Abstract utility class for handling settings common to randomizable
 meta classifiers that build an ensemble from a single base learner.   RandomizableSingleClassifierEnhancer()   - 
Constructor for class weka.classifiers. RandomizableSingleClassifierEnhancer 
  
   RandomizableSingleClustererEnhancer   - Class in  weka.clusterers  Abstract utility class for handling settings common to randomizable
 clusterers.   RandomizableSingleClustererEnhancer()   - 
Constructor for class weka.clusterers. RandomizableSingleClustererEnhancer 
  
   randomize(int[], Random)   - 
Method in class weka.classifiers. BVDecomposeSegCVSub 
 Accepts an array of ints and randomises the values in the array, using the
 random seed.
   randomize(Random)   - 
Method in class weka.core. Instances 
 Shuffles the instances in the set so that they are ordered
 randomly.
   Randomize   - Class in  weka.filters.unsupervised.instance  Randomly shuffles the order of instances passed through it.   Randomize()   - 
Constructor for class weka.filters.unsupervised.instance. Randomize 
  
   RANDOMIZED   - 
Static variable in class weka.datagenerators.clusterers. BIRCHCluster 
 Constant set for input order (default)
   randomizeDataTipText()   - 
Method in class weka.experiment. RandomSplitResultProducer 
 Returns the tip text for this property
   randomizeTipText()   - 
Method in class weka.filters.unsupervised.attribute. PropositionalToMultiInstance 
 Returns the tip text for this property
   randomNormal(int, int)   - 
Static method in class weka.classifiers.functions.pace. PaceMatrix 
 Generate matrix with standard-normally distributed random elements
   randomOrderTipText()   - 
Method in class weka.classifiers.bayes.net.search.global. K2 
  
   randomOrderTipText()   - 
Method in class weka.classifiers.bayes.net.search.local. K2 
  
   RandomProjection   - Class in  weka.filters.unsupervised.attribute  Reduces the dimensionality of the data by projecting it onto a lower dimensional subspace using a random matrix with columns of unit length (i.e.   RandomProjection()   - 
Constructor for class weka.filters.unsupervised.attribute. RandomProjection 
  
   RandomRBF   - Class in  weka.datagenerators.classifiers.classification  RandomRBF data is generated by first creating a random set of centers for each class.   RandomRBF()   - 
Constructor for class weka.datagenerators.classifiers.classification. RandomRBF 
 initializes the generator with default values
   randomRule(int, int, Random)   - 
Method in class weka.associations. PriorEstimation 
 Constructs an item set of certain length randomly.
   RandomSearch   - Class in  weka.attributeSelection  RandomSearch :  
  
 Performs a Random search in the space of attribute subsets.   RandomSearch()   - 
Constructor for class weka.attributeSelection. RandomSearch 
 Constructor
   randomSeedTipText()   - 
Method in class weka.classifiers.functions. LeastMedSq 
 Returns the tip text for this property
   randomSeedTipText()   - 
Method in class weka.classifiers.functions. SMO 
 Returns the tip text for this property
   randomSeedTipText()   - 
Method in class weka.classifiers.mi. MISMO 
 Returns the tip text for this property
   randomSeedTipText()   - 
Method in class weka.classifiers.trees. ADTree 
  
   randomSeedTipText()   - 
Method in class weka.core.neighboursearch.balltrees. MedianDistanceFromArbitraryPoint 
 Returns the tip text for this property.
   randomSeedTipText()   - 
Method in class weka.filters.supervised.instance. Resample 
 Returns the tip text for this property.
   randomSeedTipText()   - 
Method in class weka.filters.supervised.instance. SMOTE 
 Returns the tip text for this property.
   randomSeedTipText()   - 
Method in class weka.filters.supervised.instance. SpreadSubsample 
 Returns the tip text for this property
   randomSeedTipText()   - 
Method in class weka.filters.unsupervised.attribute. AddNoise 
 Returns the tip text for this property
   randomSeedTipText()   - 
Method in class weka.filters.unsupervised.attribute. RandomProjection 
 Returns the tip text for this property
   randomSeedTipText()   - 
Method in class weka.filters.unsupervised.instance. Randomize 
 Returns the tip text for this property
   randomSeedTipText()   - 
Method in class weka.filters.unsupervised.instance. Resample 
 Returns the tip text for this property
   randomSeedTipText()   - 
Method in class weka.filters.unsupervised.instance. ReservoirSample 
 Returns the tip text for this property
   RandomSplitResultProducer   - Class in  weka.experiment  Generates a single train/test split and calls the appropriate SplitEvaluator to generate some results.   RandomSplitResultProducer()   - 
Constructor for class weka.experiment. RandomSplitResultProducer 
  
   RandomSubset   - Class in  weka.filters.unsupervised.attribute  Chooses a random subset of attributes, either an absolute number or a percentage.   RandomSubset()   - 
Constructor for class weka.filters.unsupervised.attribute. RandomSubset 
  
   RandomSubSpace   - Class in  weka.classifiers.meta  This method constructs a decision tree based classifier that maintains highest accuracy on training data and improves on generalization accuracy as it grows in complexity.   RandomSubSpace()   - 
Constructor for class weka.classifiers.meta. RandomSubSpace 
 Constructor.
   randomTipText()   - 
Method in class weka.datagenerators. DataGenerator 
 Returns the tip text for this property
   RandomTree   - Class in  weka.classifiers.trees  Class for constructing a tree that considers K randomly  chosen attributes at each node.   RandomTree()   - 
Constructor for class weka.classifiers.trees. RandomTree 
  
   RandomVariates   - Class in  weka.core  Class implementing some simple random variates generator.   RandomVariates()   - 
Constructor for class weka.core. RandomVariates 
 Simply the constructor of super class
   RandomVariates(long)   - 
Constructor for class weka.core. RandomVariates 
 Simply the constructor of super class
   randomWidthFactorTipText()   - 
Method in class weka.classifiers.meta. MultiClassClassifier 
  
   Range   - Class in  weka.core  Class representing a range of cardinal numbers.   Range()   - 
Constructor for class weka.core. Range 
 Default constructor.
   Range(String)   - 
Constructor for class weka.core. Range 
 Constructor to set initial range.
   RANGE_BOUNDS   - 
Static variable in class weka.classifiers.meta. ThresholdSelector 
 Correct based on min/max observed
   RANGE_NONE   - 
Static variable in class weka.classifiers.meta. ThresholdSelector 
 no range correction
   rangeCorrectionTipText()   - 
Method in class weka.classifiers.meta. ThresholdSelector 
  
   rangesSet()   - 
Method in class weka.core. NormalizableDistance 
 Check if ranges are set.
   rangesTipText()   - 
Method in class weka.filters.unsupervised.attribute. PartitionedMultiFilter 
 Returns the tip text for this property.
   rank()   - 
Method in class weka.core.matrix. Matrix 
 Matrix rank
   rank()   - 
Method in class weka.core.matrix. SingularValueDecomposition 
 Effective numerical matrix rank
   rankAttributes(Instances, SubsetEvaluator, boolean)   - 
Method in class weka.attributeSelection. LFSMethods 
  
   RankEachAttribute()   - 
Method in class weka.attributeSelection. ScatterSearchV1 
 Rank all the attributes individually acording to their merits
   rankedAttributes()   - 
Method in class weka.attributeSelection. AttributeSelection 
 get the final ranking of the attributes.
   rankedAttributes()   - 
Method in class weka.attributeSelection. GreedyStepwise 
 Produces a ranked list of attributes.
   rankedAttributes()   - 
Method in class weka.attributeSelection. RaceSearch 
  
   rankedAttributes()   - 
Method in interface weka.attributeSelection. RankedOutputSearch 
 Returns a X by 2 list of attribute indexes and corresponding
 evaluations from best (highest) to worst.
   rankedAttributes()   - 
Method in class weka.attributeSelection. Ranker 
 Sorts the evaluated attribute list
   RankedOutputSearch   - Interface in  weka.attributeSelection  Interface for search methods capable of producing a
 ranked list of attributes.   Ranker   - Class in  weka.attributeSelection  Ranker :  
  
 Ranks attributes by their individual evaluations.   Ranker()   - 
Constructor for class weka.attributeSelection. Ranker 
 Constructor
   RankSearch   - Class in  weka.attributeSelection  RankSearch :  
  
 Uses an attribute/subset evaluator to rank all attributes.   RankSearch()   - 
Constructor for class weka.attributeSelection. RankSearch 
 Constructor
   rankTipText()   - 
Method in class weka.attributeSelection. LatentSemanticAnalysis 
 Returns the tip text for this property
   rawOutputTipText()   - 
Method in class weka.experiment. CrossValidationResultProducer 
 Returns the tip text for this property
   rawOutputTipText()   - 
Method in class weka.experiment. RandomSplitResultProducer 
 Returns the tip text for this property
   RBFKernel   - Class in  weka.classifiers.functions.supportVector  The RBF kernel.   RBFKernel()   - 
Constructor for class weka.classifiers.functions.supportVector. RBFKernel 
 default constructor - does nothing.
   RBFKernel(Instances, int, double)   - 
Constructor for class weka.classifiers.functions.supportVector. RBFKernel 
 Constructor.
   RBFNetwork   - Class in  weka.classifiers.functions  Class that implements a normalized Gaussian radial basisbasis function network. 
 It uses the k-means clustering algorithm to provide the basis functions and learns either a logistic regression (discrete class problems) or linear regression (numeric class problems) on top of that.   RBFNetwork()   - 
Constructor for class weka.classifiers.functions. RBFNetwork 
  
   rbind(PaceMatrix)   - 
Method in class weka.classifiers.functions.pace. PaceMatrix 
 Returns a new matrix which binds two matrices together with rows.
   rchisq(int, double, Random)   - 
Static method in class weka.core.matrix. Maths 
 Generates a sample of a Chi-square distribution.
   RDG1   - Class in  weka.datagenerators.classifiers.classification  A data generator that produces data randomly by producing a decision list. 
 The decision list consists of rules. 
 Instances are generated randomly one by one.   RDG1()   - 
Constructor for class weka.datagenerators.classifiers.classification. RDG1 
 initializes the generator with default values
   read(String)   - 
Static method in class weka.core.converters. ConverterUtils.DataSource 
 convencience method for loading a dataset in batch mode.
   read(InputStream)   - 
Static method in class weka.core.converters. ConverterUtils.DataSource 
 convencience method for loading a dataset in batch mode from a stream.
   read(Loader)   - 
Static method in class weka.core.converters. ConverterUtils.DataSource 
 convencience method for loading a dataset in batch mode.
   read(BufferedReader)   - 
Static method in class weka.core.matrix. Matrix 
 Read a matrix from a stream.
   read(String)   - 
Static method in class weka.core. SerializationHelper 
 deserializes the given file and returns the object from it.
   read(InputStream)   - 
Static method in class weka.core. SerializationHelper 
 deserializes from the given stream and returns the object from it.
   read(String)   - 
Method in class weka.core. Stopwords 
 Generates a new Stopwords object from the given file
   read(File)   - 
Method in class weka.core. Stopwords 
 Generates a new Stopwords object from the given file
   read(BufferedReader)   - 
Method in class weka.core. Stopwords 
 Generates a new Stopwords object from the reader.
   read(String)   - 
Static method in class weka.core.xml. KOML 
 reads the XML-serialized object from the given file
   read(File)   - 
Static method in class weka.core.xml. KOML 
 reads the XML-serialized object from the given file
   read(InputStream)   - 
Static method in class weka.core.xml. KOML 
 reads the XML-serialized object from a stream
   read(String)   - 
Method in class weka.core.xml. XMLDocument 
 parses the given XML string (can be XML or a filename) and returns a
 DOM Document.
   read(File)   - 
Method in class weka.core.xml. XMLDocument 
 parses the given file and returns a DOM document.
   read(InputStream)   - 
Method in class weka.core.xml. XMLDocument 
 parses the given stream and returns a DOM document.
   read(Reader)   - 
Method in class weka.core.xml. XMLDocument 
 parses the given reader and returns a DOM document.
   read(String)   - 
Method in class weka.core.xml. XMLSerialization 
 parses the given XML string (can be XML or a filename) and returns an
 Object generated from the representation
   read(File)   - 
Method in class weka.core.xml. XMLSerialization 
 parses the given file and returns a DOM document
   read(InputStream)   - 
Method in class weka.core.xml. XMLSerialization 
 parses the given stream and returns a DOM document
   read(Reader)   - 
Method in class weka.core.xml. XMLSerialization 
 parses the given reader and returns a DOM document
   read()   - 
Method in class weka.core.xml. XMLSerializationMethodHandler 
 returns the handler for read methods
   read(String)   - 
Static method in class weka.core.xml. XStream 
 reads the XML-serialized object from the given file
   read(File)   - 
Static method in class weka.core.xml. XStream 
 reads the XML-serialized object from the given file
   read(InputStream)   - 
Static method in class weka.core.xml. XStream 
 reads the XML-serialized object from the given input stream
   read(Reader)   - 
Static method in class weka.core.xml. XStream 
 reads the XML-serialized object from the given Reader
   read(String)   - 
Static method in class weka.experiment. Experiment 
 Loads an experiment from a file.
   readAll(String)   - 
Static method in class weka.core. SerializationHelper 
 deserializes the given file and returns the objects from it.
   readAll(InputStream)   - 
Static method in class weka.core. SerializationHelper 
 deserializes from the given stream and returns the object from it.
   readBeanConnection(Element)   - 
Method in class weka.gui.beans.xml. XMLBeans 
 builds the BeanConnection from the given DOM node.
   readBeanInstance(Element)   - 
Method in class weka.gui.beans.xml. XMLBeans 
 builds the BeanInstance from the given DOM node.
   readBeanVisual(Element)   - 
Method in class weka.gui.beans.xml. XMLBeans 
 builds the BeanVisual from the given DOM node.
   readBIF(String)   - 
Method in class weka.gui.graphvisualizer. GraphVisualizer 
 BIF reader 
  Reads a graph description in XMLBIF03 from a string
   readBIF(InputStream)   - 
Method in class weka.gui.graphvisualizer. GraphVisualizer 
 BIF reader 
  Reads a graph description in XMLBIF03 from an InputStrem
   readBIFFromFile(String)   - 
Method in class weka.classifiers.bayes.net. GUI 
 BIF reader 
 Reads a graph description in XMLBIF03 from an file
 with name sFileName
   readBooleanFromXML(Element)   - 
Method in class weka.core.xml. XMLSerialization 
 builds the primitive from the given DOM node.
   readByteFromXML(Element)   - 
Method in class weka.core.xml. XMLSerialization 
 builds the primitive from the given DOM node.
   readCharFromXML(Element)   - 
Method in class weka.core.xml. XMLSerialization 
 builds the primitive from the given DOM node.
   readCollection(Element)   - 
Method in class weka.core.xml. XMLBasicSerialization 
 builds the Collection from the given DOM node.
   readColor(Element)   - 
Method in class weka.gui.beans.xml. XMLBeans 
 builds the Color from the given DOM node.
   readColorUIResource(Element)   - 
Method in class weka.gui.beans.xml. XMLBeans 
 builds the ColorUIResource from the given DOM node.
   readCostMatrixOld(Element)   - 
Method in class weka.core.xml. XMLBasicSerialization 
 builds the Matrix (old) from the given DOM node.
   readDefaultListModel(Element)   - 
Method in class weka.core.xml. XMLBasicSerialization 
 builds the DefaultListModel from the given DOM node.
   readDimension(Element)   - 
Method in class weka.gui.beans.xml. XMLBeans 
 builds the Dimension from the given DOM node.
   readDOT(Reader)   - 
Method in class weka.gui.graphvisualizer. GraphVisualizer 
 Dot reader 
  Reads a graph description in DOT format from a string
   readDoubleFromXML(Element)   - 
Method in class weka.core.xml. XMLSerialization 
 builds the primitive from the given DOM node.
   readFloatFromXML(Element)   - 
Method in class weka.core.xml. XMLSerialization 
 builds the primitive from the given DOM node.
   readFont(Element)   - 
Method in class weka.gui.beans.xml. XMLBeans 
 builds the Font from the given DOM node.
   readFontUIResource(Element)   - 
Method in class weka.gui.beans.xml. XMLBeans 
 builds the FontUIResource from the given DOM node.
   readFromXML(Object, String, Element)   - 
Method in class weka.core.xml. XMLSerialization 
 adds the specific node to the object via a set method
   readFromXML(Element)   - 
Method in class weka.core.xml. XMLSerialization 
 builds the object from the given DOM node.
   readInstance(Instances)   - 
Method in class weka.core.converters. ArffLoader.ArffReader 
 Reads a single instance using the tokenizer and returns it.
   readInstance(Instances, boolean)   - 
Method in class weka.core.converters. ArffLoader.ArffReader 
 Reads a single instance using the tokenizer and returns it.
   readInstance(Reader)   - 
Method in class weka.core. Instances 
  Deprecated.   instead of using this method in conjunction with the
  readInstance(Reader)  method, one should use the
  ArffLoader  or  DataSource  class instead. 
   readIntFromXML(Element)   - 
Method in class weka.core.xml. XMLSerialization 
 builds the primitive from the given DOM node.
   readLoader(Element)   - 
Method in class weka.gui.beans.xml. XMLBeans 
 builds the Loader from the given DOM node.
   readLongFromXML(Element)   - 
Method in class weka.core.xml. XMLSerialization 
 builds the primitive from the given DOM node.
   readMap(Element)   - 
Method in class weka.core.xml. XMLBasicSerialization 
 builds the Map from the given DOM node.
   readMatrix(Element)   - 
Method in class weka.core.xml. XMLBasicSerialization 
 builds the Matrix from the given DOM node.
   readMatrixOld(Element)   - 
Method in class weka.core.xml. XMLBasicSerialization 
 builds the Matrix (old) from the given DOM node.
   readMetaBean(Element)   - 
Method in class weka.gui.beans.xml. XMLBeans 
 builds the MetaBean from the given DOM node.
   readOldFormat(Reader)   - 
Method in class weka.classifiers. CostMatrix 
 Loads a cost matrix in the old format from a reader.
   readPoint(Element)   - 
Method in class weka.gui.beans.xml. XMLBeans 
 builds the Point from the given DOM node.
   readProperties(String)   - 
Static method in class weka.core. Utils 
 Reads properties that inherit from three locations.
   readPropertyNode(Element)   - 
Method in class weka.experiment.xml. XMLExperiment 
 builds the PropertyNode from the given DOM node.
   readSaver(Element)   - 
Method in class weka.gui.beans.xml. XMLBeans 
 builds the Saver from the given DOM node.
   readShortFromXML(Element)   - 
Method in class weka.core.xml. XMLSerialization 
 builds the primitive from the given DOM node.
   realCount   - 
Variable in class weka.core. AttributeStats 
 The number of real-like values (i.e.
   recall(int)   - 
Method in class weka.classifiers. Evaluation 
 Calculate the recall with respect to a particular class.
   RECALL   - 
Static variable in class weka.classifiers.meta. ThresholdSelector 
 recall
   RECALL_NAME   - 
Static variable in class weka.classifiers.evaluation. ThresholdCurve 
 attribute name: Recall
   RECTANGLE   - 
Static variable in class weka.gui.visualize. VisualizePanelEvent 
  
   redo()   - 
Method in class weka.classifiers.bayes.net. EditableBayesNet 
 redo the last edit action performed on the network.
   reduce_table()   - 
Method in class weka.core.mathematicalexpression. Parser 
 Access to  reduce_goto  table.
   reduce_table()   - 
Method in class weka.filters.unsupervised.instance.subsetbyexpression. Parser 
 Access to  reduce_goto  table.
   reducedErrorPruningTipText()   - 
Method in class weka.classifiers.rules. PART 
 Returns the tip text for this property
   reducedErrorPruningTipText()   - 
Method in class weka.classifiers.trees. J48 
 Returns the tip text for this property
   reduceDimensionality(Instances)   - 
Method in class weka.attributeSelection. AttributeSelection 
 reduce the dimensionality of a set of instances to include only those 
 attributes chosen by the last run of attribute selection.
   reduceDimensionality(Instance)   - 
Method in class weka.attributeSelection. AttributeSelection 
 reduce the dimensionality of a single instance to include only those 
 attributes chosen by the last run of attribute selection.
   reduceDL(double, boolean)   - 
Method in class weka.classifiers.rules. RuleStats 
 Try to reduce the DL of the ruleset by testing removing the rules
 one by one in reverse order and update all the stats
   reduceMatrix(double[][])   - 
Static method in class weka.core. ContingencyTables 
 Reduces a matrix by deleting all zero rows and columns.
   ReferenceInstances   - Class in  weka.classifiers.trees.adtree  Simple class that extends the Instances class making it possible to create
 subsets of instances that reference their source set.   ReferenceInstances(Instances, int)   - 
Constructor for class weka.classifiers.trees.adtree. ReferenceInstances 
 Creates an empty set of instances.
   refine(ArrayList)   - 
Method in class weka.associations.tertius. Rule 
 Refine a rule by adding literal from a set of predictes.
   refresh()   - 
Method in class weka.gui.arffviewer. ArffViewer 
 validates and repaints the frame
   refresh()   - 
Method in class weka.gui.arffviewer. ArffViewerMainPanel 
 validates and repaints the frame
   refreshFreqTipText()   - 
Method in class weka.gui.beans. StripChart 
 GUI Tip text
   register(Object, Class, String)   - 
Method in class weka.core.xml. XMLSerializationMethodHandler 
 adds read and write methods for the given class, i.e., read&;lt;name>
 and write<name> ("name" is prefixed by read and write)
   registerEditors()   - 
Static method in class weka.gui. GenericObjectEditor 
 registers all the editors in Weka.
   RegOptimizer   - Class in  weka.classifiers.functions.supportVector  Base class implementation for learning algorithm of SMOreg
 
  
 Valid options are:     RegOptimizer()   - 
Constructor for class weka.classifiers.functions.supportVector. RegOptimizer 
 the default constructor
   regOptimizerTipText()   - 
Method in class weka.classifiers.functions. SMOreg 
 Returns the tip text for this property
   Regression   - Class in  weka.classifiers.pmml.consumer  Class implementing import of PMML Regression model.   Regression(Element, Instances, MiningSchema)   - 
Constructor for class weka.classifiers.pmml.consumer. Regression 
 Constructs a new PMML Regression.
   regression(Matrix, double)   - 
Method in class weka.core.matrix. Matrix 
 Performs a (ridged) linear regression.
   regression(Matrix, double[], double)   - 
Method in class weka.core.matrix. Matrix 
 Performs a weighted (ridged) linear regression.
   regression(Matrix, double)   - 
Method in class weka.core. Matrix 
  Deprecated.  Performs a (ridged) linear regression.
   regression(Matrix, double[], double)   - 
Method in class weka.core. Matrix 
  Deprecated.  Performs a weighted (ridged) linear regression.
   RegressionByDiscretization   - Class in  weka.classifiers.meta  A regression scheme that employs any classifier on a copy of the data that has the class attribute (equal-width) discretized.   RegressionByDiscretization()   - 
Constructor for class weka.classifiers.meta. RegressionByDiscretization 
 Default constructor.
   RegressionGenerator   - Class in  weka.datagenerators  Abstract class for data generators for regression classifiers.   RegressionGenerator()   - 
Constructor for class weka.datagenerators. RegressionGenerator 
 initializes the generator with default values
   RegressionSplitEvaluator   - Class in  weka.experiment  A SplitEvaluator that produces results for a classification scheme on a numeric class attribute.   RegressionSplitEvaluator()   - 
Constructor for class weka.experiment. RegressionSplitEvaluator 
 No args constructor.
   RegSMO   - Class in  weka.classifiers.functions.supportVector  Implementation of SMO for support vector regression as described in : 
  
 A.J.   RegSMO()   - 
Constructor for class weka.classifiers.functions.supportVector. RegSMO 
 default constructor
   RegSMOImproved   - Class in  weka.classifiers.functions.supportVector  Learn SVM for regression using SMO with Shevade, Keerthi, et al.   RegSMOImproved()   - 
Constructor for class weka.classifiers.functions.supportVector. RegSMOImproved 
  
   relabelTipText()   - 
Method in class weka.classifiers.trees. J48graft 
 Returns the tip text for this property
   RELAGGS   - Class in  weka.filters.unsupervised.attribute  A propositionalization filter inspired by the RELAGGS algorithm. 
 It processes all relational attributes that fall into the user defined range (all others are skipped, i.e., not added to the output).   RELAGGS()   - 
Constructor for class weka.filters.unsupervised.attribute. RELAGGS 
  
   relation()   - 
Method in class weka.core. Attribute 
 Returns the header info for a relation-valued attribute,
 null if the attribute is not relation-valued.
   relation(int)   - 
Method in class weka.core. Attribute 
 Returns a value of a relation-valued attribute.
   RELATION_NAME   - 
Static variable in class weka.classifiers.evaluation. CostCurve 
 The name of the relation used in cost curve datasets
   RELATION_NAME   - 
Static variable in class weka.classifiers.evaluation. ThresholdCurve 
 The name of the relation used in threshold curve datasets
   RELATIONAL   - 
Static variable in class weka.core. Attribute 
 Constant set for relation-valued attributes.
   RelationalLocator   - Class in  weka.core  This class locates and records the indices of relational attributes,   RelationalLocator(Instances)   - 
Constructor for class weka.core. RelationalLocator 
 Initializes the RelationalLocator with the given data.
   RelationalLocator(Instances, int, int)   - 
Constructor for class weka.core. RelationalLocator 
 Initializes the RelationalLocator with the given data.
   RelationalLocator(Instances, int[])   - 
Constructor for class weka.core. RelationalLocator 
 Initializes the RelationalLocator with the given data.
   relationalValue(int)   - 
Method in class weka.core. Instance 
 Returns the relational value of a relational attribute.
   relationalValue(Attribute)   - 
Method in class weka.core. Instance 
 Returns the relational value of a relational attribute.
   relationForTableNameTipText()   - 
Method in class weka.core.converters. DatabaseSaver 
 Returns the tip text fo this property.
   relationName()   - 
Method in class weka.core. Instances 
 Returns the relation's name.
   relationNameTipText()   - 
Method in class weka.datagenerators. DataGenerator 
 Returns the tip text for this property
   relativeAbsoluteError()   - 
Method in class weka.classifiers. Evaluation 
 Returns the relative absolute error.
   relativeDL(int, double, boolean)   - 
Method in class weka.classifiers.rules. RuleStats 
 The description length (DL) of the ruleset relative to if the
 rule in the given position is deleted, which is obtained by:  
 MDL if the rule exists - MDL if the rule does not exist  
 Note the minimal possible DL of the ruleset is calculated(i.e.
   ReliefFAttributeEval   - Class in  weka.attributeSelection  ReliefFAttributeEval : 
  
 Evaluates the worth of an attribute by repeatedly sampling an instance and considering the value of the given attribute for the nearest instance of the same and different class.   ReliefFAttributeEval()   - 
Constructor for class weka.attributeSelection. ReliefFAttributeEval 
 Constructor
   RemoteBoundaryVisualizerSubTask   - Class in  weka.gui.boundaryvisualizer  Class that encapsulates a sub task for distributed boundary
 visualization.   RemoteBoundaryVisualizerSubTask()   - 
Constructor for class weka.gui.boundaryvisualizer. RemoteBoundaryVisualizerSubTask 
  
   RemoteEngine   - Class in  weka.experiment  A general purpose server for executing Task objects sent via RMI.   RemoteEngine(String)   - 
Constructor for class weka.experiment. RemoteEngine 
 Constructor
   RemoteExperiment   - Class in  weka.experiment  Holds all the necessary configuration information for a distributed
 experiment.   RemoteExperiment()   - 
Constructor for class weka.experiment. RemoteExperiment 
 Construct a new RemoteExperiment using an empty Experiment as base 
 Experiment
   RemoteExperiment(Experiment)   - 
Constructor for class weka.experiment. RemoteExperiment 
 Construct a new RemoteExperiment using a base Experiment
   RemoteExperimentEvent   - Class in  weka.experiment  Class encapsulating information on progress of a remote experiment   RemoteExperimentEvent(boolean, boolean, boolean, String)   - 
Constructor for class weka.experiment. RemoteExperimentEvent 
 Constructor
   RemoteExperimentListener   - Interface in  weka.experiment  Interface for classes that want to listen for updates on RemoteExperiment
 progress   remoteExperimentStatus(RemoteExperimentEvent)   - 
Method in interface weka.experiment. RemoteExperimentListener 
 Called when progress has been made in a remote experiment
   RemoteExperimentSubTask   - Class in  weka.experiment  Class to encapsulate an experiment as a task that can be executed on
 a remote host.   RemoteExperimentSubTask()   - 
Constructor for class weka.experiment. RemoteExperimentSubTask 
  
   RemoteResult   - Class in  weka.gui.boundaryvisualizer  Class that encapsulates a result (and progress info) for part
 of a distributed boundary visualization.   RemoteResult(int, int)   - 
Constructor for class weka.gui.boundaryvisualizer. RemoteResult 
 Creates a new  RemoteResult  instance.
   remove()   - 
Method in class weka.associations.tertius. SimpleLinkedList.LinkedListInverseIterator 
  
   remove()   - 
Method in class weka.associations.tertius. SimpleLinkedList.LinkedListIterator 
  
   remove(Object)   - 
Method in class weka.core. ProtectedProperties 
 Overrides a method to prevent the properties from being modified.
   remove(String)   - 
Method in class weka.core. Stopwords 
 removes the word from the stopword list
   remove(PrintStream)   - 
Method in class weka.core. Tee 
 removes the given PrintStream from the list.
   remove(int)   - 
Method in class weka.core. Tee 
 removes the given PrintStream from the list.
   remove(Object)   - 
Method in class weka.core. Trie 
 Removes a single instance of the specified element from this collection, 
 if it is present.
   remove()   - 
Method in class weka.core. Trie.TrieIterator 
 ignored
   remove(String)   - 
Method in class weka.core. Trie.TrieNode 
 Removes a suffix from the trie.
   remove(String)   - 
Method in class weka.core.xml. MethodHandler 
 removes the method for the property specified by the display name
 from its internal list.
   remove(Class)   - 
Method in class weka.core.xml. MethodHandler 
 removes the method for the specified class from its internal list.
   Remove   - Class in  weka.filters.unsupervised.attribute  A filter that removes a range of attributes from the dataset.   Remove()   - 
Constructor for class weka.filters.unsupervised.attribute. Remove 
 Constructor so that we can initialize the Range variable properly.
   remove()   - 
Method in class weka.gui.beans. BeanConnection 
 Remove this connection
   remove(int)   - 
Method in class weka.gui. CheckBoxList.CheckBoxListModel 
 Removes the element at the specified position in this list.
   REMOVE_CHILDREN   - 
Static variable in class weka.gui.treevisualizer. TreeDisplayEvent 
  
   REMOVE_POINT_RADIUS   - 
Static variable in class weka.gui.boundaryvisualizer. BoundaryPanel 
 The distance we can click away from a point in the GUI and still remove it.
   removeActionListener(ActionListener)   - 
Method in class weka.gui.boundaryvisualizer. BoundaryPanel 
 Remove a listener
   removeAll(Collection<?>)   - 
Method in class weka.core. Trie 
 Removes all this collection's elements that are also contained in the 
 specified collection
   removeAllBeansFromContainer(JComponent)   - 
Static method in class weka.gui.beans. BeanInstance 
 Removes all beans from containing component
   removeAllElements()   - 
Method in class weka.core. FastVector 
 Removes all components from this vector and sets its 
 size to zero.
   removeAllElements()   - 
Method in class weka.core. Queue 
 Removes all objects from the queue m_Tail.m_Next.
   removeAllInputs()   - 
Method in class weka.classifiers.functions.neural. NeuralConnection 
 This function will remove all the inputs to this unit.
   removeAllInputs()   - 
Method in class weka.classifiers.functions.neural. NeuralNode 
 This function will remove all the inputs to this unit.
   removeAllInstances()   - 
Method in class weka.gui.boundaryvisualizer. BoundaryPanel 
 Deletes all training instances from our dataset.
   removeAllMissingColsTipText()   - 
Method in class weka.associations. Apriori 
 Returns the tip text for this property
   removeAllOutputs()   - 
Method in class weka.classifiers.functions.neural. NeuralConnection 
 This function will remove all outputs to this unit.
   removeAllowed(Class, String)   - 
Method in class weka.core.xml. PropertyHandler 
 removes the given property (display name) for the specified class from 
 the list of allowed properties.
   removeAllPlots()   - 
Method in class weka.gui.visualize. Plot2D 
 Clears all plots
   removeAllPlots()   - 
Method in class weka.gui.visualize. VisualizePanel 
 Removes all the plots.
   removeBatchClassifierListener(BatchClassifierListener)   - 
Method in class weka.gui.beans. Classifier 
 Remove a batch classifier listener
   removeBatchClustererListener(BatchClustererListener)   - 
Method in class weka.gui.beans. Clusterer 
 Remove a batch clusterer listener
   removeBean(JComponent)   - 
Method in class weka.gui.beans. BeanInstance 
 Remove this bean from the list of beans and from the containing component
   removeCancelListener(ActionListener)   - 
Method in class weka.gui. GenericObjectEditor.GOEPanel 
 This is used to remove an action listener from the cancel button.
   removeCapabilitiesFilter()   - 
Method in class weka.gui. GenericObjectEditor 
 Removes the current Capabilities filter.
   removeCapabilitiesFilterListener(Explorer.CapabilitiesFilterChangeListener)   - 
Method in class weka.gui.explorer. Explorer 
 Removes the specified listener from the set of listeners if it is present.
   removeChangeListener(ChangeListener)   - 
Method in class weka.gui.arffviewer. ArffPanel 
 Removes a ChangeListener from the panel
   removeChangeListener(ChangeListener)   - 
Method in class weka.gui.arffviewer. ArffTable 
 Removes a ChangeListener from the panel
   removeChartListener(ChartListener)   - 
Method in class weka.gui.beans. IncrementalClassifierEvaluator 
 Remove a chart listener
   removeChildFrame(Container)   - 
Method in class weka.gui. GUIChooser 
 tries to remove the child frame, it returns true if it could do such.
   removeChildFrame(Container)   - 
Method in class weka.gui. Main 
 tries to remove the child frame, it returns true if it could do such.
   removeClassColumnTipText()   - 
Method in class weka.experiment. DensityBasedClustererSplitEvaluator 
 Returns the tip text for this property
   removeConnectionListener(ConnectionListener)   - 
Method in class weka.gui.sql. ConnectionPanel 
 removes the given listener from the list of listeners.
   removeConnectionListener(ConnectionListener)   - 
Method in class weka.gui.sql. SqlViewer 
 removes the given listener from the list of listeners.
   removeConnections(BeanInstance)   - 
Static method in class weka.gui.beans. BeanConnection 
 Remove all connections for a bean.
   removeDataFormatListener(DataFormatListener)   - 
Method in class weka.gui.beans. ClassAssigner 
  
   removeDataFormatListener(DataFormatListener)   - 
Method in class weka.gui.beans. ClassValuePicker 
  
   removeDataSourceListener(DataSourceListener)   - 
Method in class weka.gui.beans. AbstractDataSource 
 Remove a listener
   removeDataSourceListener(DataSourceListener)   - 
Method in class weka.gui.beans. ClassAssigner 
  
   removeDataSourceListener(DataSourceListener)   - 
Method in class weka.gui.beans. ClassValuePicker 
  
   removeDataSourceListener(DataSourceListener)   - 
Method in interface weka.gui.beans. DataSource 
 Remove a data source listener
   removeDataSourceListener(DataSourceListener)   - 
Method in class weka.gui.beans. DataVisualizer 
 Remove a listener
   removeDataSourceListener(DataSourceListener)   - 
Method in class weka.gui.beans. Filter 
 Remove a data source listener
   removeDataSourceListener(DataSourceListener)   - 
Method in class weka.gui.beans. InstanceStreamToBatchMaker 
  
   removeDataSourceListener(DataSourceListener)   - 
Method in class weka.gui.beans. Loader 
 Remove a listener
   removeDataSourceListener(DataSourceListener)   - 
Method in class weka.gui.beans. PredictionAppender 
 Remove a datasource listener
   removedPercentageTipText()   - 
Method in class weka.classifiers.meta. RotationForest 
 Returns the tip text for this property
   removeElement(Object)   - 
Method in class weka.gui. CheckBoxList.CheckBoxListModel 
 Removes the first (lowest-indexed) occurrence of the argument from this 
 list.
   removeElementAt(int)   - 
Method in class weka.core. FastVector 
 Deletes an element from this vector.
   removeFirst()   - 
Method in class weka.associations.tertius. SimpleLinkedList 
  
   RemoveFolds   - Class in  weka.filters.unsupervised.instance  This filter takes a dataset and outputs a specified fold for cross validation.   RemoveFolds()   - 
Constructor for class weka.filters.unsupervised.instance. RemoveFolds 
  
   RemoveFrequentValues   - Class in  weka.filters.unsupervised.instance  Determines which values (frequent or infrequent ones) of an (nominal) attribute are retained and filters the instances accordingly.   RemoveFrequentValues()   - 
Constructor for class weka.filters.unsupervised.instance. RemoveFrequentValues 
  
   removeGraphListener(GraphListener)   - 
Method in class weka.gui.beans. Associator 
 Remove a graph listener
   removeGraphListener(GraphListener)   - 
Method in class weka.gui.beans. Classifier 
 Remove a graph listener
   removeGraphListener(GraphListener)   - 
Method in class weka.gui.beans. Clusterer 
 Remove a graph listener
   removeHistoryChangedListener(HistoryChangedListener)   - 
Method in class weka.gui.sql. ConnectionPanel 
 removes the given listener from the list of listeners.
   removeHistoryChangedListener(HistoryChangedListener)   - 
Method in class weka.gui.sql. QueryPanel 
 removes the given listener from the list of listeners.
   removeHistoryChangedListener(HistoryChangedListener)   - 
Method in class weka.gui.sql. SqlViewer 
 removes the given listener from the list of listeners.
   removeIgnored(String)   - 
Method in class weka.core.xml. PropertyHandler 
 removes the given display name from the ignore list.
   removeIgnored(Class, String)   - 
Method in class weka.core.xml. PropertyHandler 
 removes the given display name from the ignore list of the class.
   removeIncrementalClassifierListener(IncrementalClassifierListener)   - 
Method in class weka.gui.beans. Classifier 
 Remove an incremental classifier listener
   removeInstanceListener(InstanceListener)   - 
Method in class weka.gui.beans. AbstractDataSource 
 Remove an instance listener
   removeInstanceListener(InstanceListener)   - 
Method in class weka.gui.beans. ClassAssigner 
  
   removeInstanceListener(InstanceListener)   - 
Method in interface weka.gui.beans. DataSource 
 Remove an instance listener
   removeInstanceListener(InstanceListener)   - 
Method in class weka.gui.beans. Filter 
 Remove an instance listener
   removeInstanceListener(InstanceListener)   - 
Method in class weka.gui.beans. InstanceStreamToBatchMaker 
  
   removeInstanceListener(InstanceListener)   - 
Method in class weka.gui.beans. Loader 
 Remove an instance listener
   removeInstanceListener(InstanceListener)   - 
Method in class weka.gui.beans. PredictionAppender 
 Remove an instance listener
   removeInstanceListener(InstanceListener)   - 
Method in class weka.gui.streams. InstanceJoiner 
  
   removeInstanceListener(InstanceListener)   - 
Method in class weka.gui.streams. InstanceLoader 
  
   removeInstanceListener(InstanceListener)   - 
Method in interface weka.gui.streams. InstanceProducer 
  
   removeLast()   - 
Method in class weka.classifiers.rules. RuleStats 
 Remove the last rule in the ruleset as well as it's stats.
   removeLayoutCompleteEventListener(LayoutCompleteEventListener)   - 
Method in class weka.gui.graphvisualizer. HierarchicalBCEngine 
 Method to remove a LayoutCompleteEventListener.
   removeLayoutCompleteEventListener(LayoutCompleteEventListener)   - 
Method in interface weka.gui.graphvisualizer. LayoutEngine 
 This method removes a LayoutCompleteEventListener from the
 LayoutEngine.
   removeLinkAt(int)   - 
Method in class weka.attributeSelection. BestFirst.LinkedList2 
 removes an element (Link) at a specific index from the list.
   removeLinkAt(int)   - 
Method in class weka.attributeSelection. LFSMethods.LinkedList2 
 removes an element (Link) at a specific index from the list.
   RemoveMisclassified   - Class in  weka.filters.unsupervised.instance  A filter that removes instances which are incorrectly classified.   RemoveMisclassified()   - 
Constructor for class weka.filters.unsupervised.instance. RemoveMisclassified 
  
   removeNotify()   - 
Method in class weka.gui. PropertyPanel 
 Cleans up when the panel is destroyed.
   removeOkListener(ActionListener)   - 
Method in class weka.gui. GenericObjectEditor.GOEPanel 
 This is used to remove an action listener from the ok button.
   removeOldClassTipText()   - 
Method in class weka.filters.supervised.attribute. AddClassification 
 Returns the tip text for this property.
   RemovePercentage   - Class in  weka.filters.unsupervised.instance  A filter that removes a given percentage of a dataset.   RemovePercentage()   - 
Constructor for class weka.filters.unsupervised.instance. RemovePercentage 
  
   removePropertyChangeListener(String, PropertyChangeListener)   - 
Method in class weka.gui.beans. AbstractDataSource 
 Remove a property change listener from this bean
   removePropertyChangeListener(PropertyChangeListener)   - 
Method in class weka.gui.beans. AssociatorCustomizer 
 Remove a property change listener
   removePropertyChangeListener(PropertyChangeListener)   - 
Method in class weka.gui.beans. BeanVisual 
 Remove a property change listener
   removePropertyChangeListener(PropertyChangeListener)   - 
Method in class weka.gui.beans. ClassAssignerCustomizer 
 Remove a property change listener
   removePropertyChangeListener(PropertyChangeListener)   - 
Method in class weka.gui.beans. ClassifierCustomizer 
 Remove a property change listener
   removePropertyChangeListener(PropertyChangeListener)   - 
Method in class weka.gui.beans. ClassValuePickerCustomizer 
 Remove a property change listener
   removePropertyChangeListener(PropertyChangeListener)   - 
Method in class weka.gui.beans. ClustererCustomizer 
 Remove a property change listener
   removePropertyChangeListener(PropertyChangeListener)   - 
Method in class weka.gui.beans. CrossValidationFoldMakerCustomizer 
 Remove a property change listener
   removePropertyChangeListener(String, PropertyChangeListener)   - 
Method in class weka.gui.beans. DataVisualizer 
 Remove a property change listener from this bean
   removePropertyChangeListener(PropertyChangeListener)   - 
Method in class weka.gui.beans. FilterCustomizer 
 Remove a property change listener
   removePropertyChangeListener(PropertyChangeListener)   - 
Method in class weka.gui.beans. IncrementalClassifierEvaluatorCustomizer 
 Remove a property change listener
   removePropertyChangeListener(PropertyChangeListener)   - 
Method in class weka.gui.beans. LoaderCustomizer 
 Remove a property change listener
   removePropertyChangeListener(String, PropertyChangeListener)   - 
Method in class weka.gui.beans. ModelPerformanceChart 
 Remove a property change listener from this bean
   removePropertyChangeListener(PropertyChangeListener)   - 
Method in class weka.gui.beans. PredictionAppenderCustomizer 
 Remove a property change listener
   removePropertyChangeListener(PropertyChangeListener)   - 
Method in class weka.gui.beans. SaverCustomizer 
 Remove a property change listener
   removePropertyChangeListener(PropertyChangeListener)   - 
Method in class weka.gui.beans. SerializedModelSaverCustomizer 
 Remove a property change listener
   removePropertyChangeListener(PropertyChangeListener)   - 
Method in class weka.gui.beans. StripChartCustomizer 
 Remove a property change listener
   removePropertyChangeListener(String, PropertyChangeListener)   - 
Method in class weka.gui.beans. TextViewer 
 Remove a property change listener from this bean
   removePropertyChangeListener(PropertyChangeListener)   - 
Method in class weka.gui.beans. TrainTestSplitMakerCustomizer 
 Remove a property change listener
   removePropertyChangeListener(PropertyChangeListener)   - 
Method in class weka.gui. CostMatrixEditor 
 Removes an object from the list of those that wish to be informed when the
 cost matrix changes.
   removePropertyChangeListener(PropertyChangeListener)   - 
Method in class weka.gui.experiment. SetupPanel 
 Removes a PropertyChangeListener.
   removePropertyChangeListener(PropertyChangeListener)   - 
Method in class weka.gui.experiment. SimpleSetupPanel 
 Removes a PropertyChangeListener.
   removePropertyChangeListener(PropertyChangeListener)   - 
Method in class weka.gui.explorer. PreprocessPanel 
 Removes a PropertyChangeListener.
   removePropertyChangeListener(PropertyChangeListener)   - 
Method in class weka.gui. GenericArrayEditor 
 Removes a PropertyChangeListener.
   removePropertyChangeListener(PropertyChangeListener)   - 
Method in class weka.gui. GenericObjectEditor 
 Removes a PropertyChangeListener.
   removePropertyChangeListener(PropertyChangeListener)   - 
Method in class weka.gui. PropertySheetPanel 
 Removes a PropertyChangeListener.
   removePropertyChangeListener(PropertyChangeListener)   - 
Method in class weka.gui. SetInstancesPanel 
 Removes a PropertyChangeListener.
   removePropertyChangeListener(PropertyChangeListener)   - 
Method in class weka.gui. SimpleDateFormatEditor 
 Removes an object from the list of those that wish to be informed when the
 date format changes.
   removePropertyChangeListenersSubFlow(PropertyChangeListener)   - 
Method in class weka.gui.beans. MetaBean 
  
   removeQueryExecuteListener(QueryExecuteListener)   - 
Method in class weka.gui.sql. QueryPanel 
 removes the given listener from the list of listeners.
   removeQueryExecuteListener(QueryExecuteListener)   - 
Method in class weka.gui.sql. SqlViewer 
 removes the given listener from the list of listeners.
   RemoveRange   - Class in  weka.filters.unsupervised.instance  A filter that removes a given range of instances of a dataset.   RemoveRange()   - 
Constructor for class weka.filters.unsupervised.instance. RemoveRange 
  
   removeRedundant(RuleItem)   - 
Method in class weka.associations. RuleGeneration 
 Method that removes redundant rules out of the list of the best rules.
   removeResult(String)   - 
Method in class weka.gui. ResultHistoryPanel 
 Removes one of the result buffers from the history.
   removeResultChangedListener(ResultChangedListener)   - 
Method in class weka.gui.sql. ResultPanel 
 removes the given listener from the list of listeners
   removeResultChangedListener(ResultChangedListener)   - 
Method in class weka.gui.sql. SqlViewer 
 removes the given listener from the list of listeners.
   removeSubstring(String, String)   - 
Static method in class weka.core. Utils 
 Removes all occurrences of a string from another string.
   removeTableModelListener(TableModelListener)   - 
Method in class weka.gui.arffviewer. ArffSortedTableModel 
 removes a listener from the list that is notified each time a change to
 the data model occurs
   removeTableModelListener(TableModelListener)   - 
Method in class weka.gui.arffviewer. ArffTableModel 
 removes a listener from the list that is notified each time a change to
 the data model occurs
   removeTableModelListener(TableModelListener)   - 
Method in class weka.gui.sql. ResultSetTableModel 
 removes a listener from the list that is notified each time a change to
 the data model occurs.
   removeTestSetListener(TestSetListener)   - 
Method in class weka.gui.beans. AbstractTestSetProducer 
 Remove a listener for test sets
   removeTestSetListener(TestSetListener)   - 
Method in class weka.gui.beans. AbstractTrainAndTestSetProducer 
 Remove a test set listener
   removeTestSetListener(TestSetListener)   - 
Method in class weka.gui.beans. ClassAssigner 
  
   removeTestSetListener(TestSetListener)   - 
Method in class weka.gui.beans. Filter 
 Remove a test set listener
   removeTestSetListener(TestSetListener)   - 
Method in class weka.gui.beans. PredictionAppender 
 Remove a test set listener
   removeTestSetListener(TestSetListener)   - 
Method in interface weka.gui.beans. TestSetProducer 
 Remove a listener for test set events
   removeTextListener(TextListener)   - 
Method in class weka.gui.beans. Associator 
 Remove a text listener
   removeTextListener(TextListener)   - 
Method in class weka.gui.beans. Classifier 
 Remove a text listener
   removeTextListener(TextListener)   - 
Method in class weka.gui.beans. ClassifierPerformanceEvaluator 
 Remove a text listener
   removeTextListener(TextListener)   - 
Method in class weka.gui.beans. Clusterer 
 Remove a text listener
   removeTextListener(TextListener)   - 
Method in class weka.gui.beans. ClustererPerformanceEvaluator 
 Remove a text listener
   removeTextListener(TextListener)   - 
Method in class weka.gui.beans. IncrementalClassifierEvaluator 
 Remove a text listener
   removeTextListener(TextListener)   - 
Method in class weka.gui.beans. TextViewer 
 Remove a text listener
   removeThresholdDataListener(ThresholdDataListener)   - 
Method in class weka.gui.beans. ClassifierPerformanceEvaluator 
 Remove a Threshold data listener
   removeTrainingInstanceFromMouseLocation(int, int)   - 
Method in class weka.gui.boundaryvisualizer. BoundaryPanel 
 Removes a single training instance from our dataset, if there is one that is close enough
      to the specified mouse location.
   removeTrainingSetListener(TrainingSetListener)   - 
Method in class weka.gui.beans. AbstractTrainAndTestSetProducer 
 Remove a training set listener
   removeTrainingSetListener(TrainingSetListener)   - 
Method in class weka.gui.beans. AbstractTrainingSetProducer 
 Remove a training set listener
   removeTrainingSetListener(TrainingSetListener)   - 
Method in class weka.gui.beans. ClassAssigner 
  
   removeTrainingSetListener(TrainingSetListener)   - 
Method in class weka.gui.beans. Filter 
 Remove a training set listener
   removeTrainingSetListener(TrainingSetListener)   - 
Method in class weka.gui.beans. PredictionAppender 
 Remove a training set listener
   removeTrainingSetListener(TrainingSetListener)   - 
Method in interface weka.gui.beans. TrainingSetProducer 
 Remove a training set listener
   RemoveType   - Class in  weka.filters.unsupervised.attribute  Removes attributes of a given type.   RemoveType()   - 
Constructor for class weka.filters.unsupervised.attribute. RemoveType 
  
   removeUnusedTipText()   - 
Method in class weka.filters.unsupervised.attribute. PartitionedMultiFilter 
 Returns the tip text for this property.
   RemoveUseless   - Class in  weka.filters.unsupervised.attribute  This filter removes attributes that do not vary at all or that vary too much.   RemoveUseless()   - 
Constructor for class weka.filters.unsupervised.attribute. RemoveUseless 
  
   removeVariable(String)   - 
Method in class weka.core. Environment 
 Remove a named variable from the map.
   removeVetoableChangeListener(String, VetoableChangeListener)   - 
Method in class weka.gui.beans. AbstractDataSource 
 Remove a vetoable change listener from this bean
   removeVetoableChangeListener(String, VetoableChangeListener)   - 
Method in class weka.gui.beans. CostBenefitAnalysis 
  
   removeVetoableChangeListener(String, VetoableChangeListener)   - 
Method in class weka.gui.beans. DataVisualizer 
 Remove a vetoable change listener from this bean
   removeVetoableChangeListener(String, VetoableChangeListener)   - 
Method in class weka.gui.beans. GraphViewer 
 Remove a vetoable change listener from this bean
   removeVetoableChangeListener(String, VetoableChangeListener)   - 
Method in class weka.gui.beans. ModelPerformanceChart 
 Remove a vetoable change listener from this bean
   removeVetoableChangeListener(String, VetoableChangeListener)   - 
Method in class weka.gui.beans. TextViewer 
 Remove a vetoable change listener from this bean
   removeVisualizableErrorListener(VisualizableErrorListener)   - 
Method in class weka.gui.beans. ClassifierPerformanceEvaluator 
 Remove a visualizable error listener
   RemoveWithValues   - Class in  weka.filters.unsupervised.instance  Filters instances according to the value of an attribute.   RemoveWithValues()   - 
Constructor for class weka.filters.unsupervised.instance. RemoveWithValues 
 Default constructor
   renameAttribute(int, String)   - 
Method in class weka.core. Instances 
 Renames an attribute.
   renameAttribute(Attribute, String)   - 
Method in class weka.core. Instances 
 Renames an attribute.
   renameAttribute()   - 
Method in class weka.gui.arffviewer. ArffPanel 
 renames the current attribute
   renameAttribute()   - 
Method in class weka.gui.arffviewer. ArffViewerMainPanel 
 renames the current selected Attribute
   renameAttributeAt(int, String)   - 
Method in class weka.gui.arffviewer. ArffSortedTableModel 
 renames the attribute at the given col index
   renameAttributeAt(int, String)   - 
Method in class weka.gui.arffviewer. ArffTableModel 
 renames the attribute at the given col index
   renameAttributeValue(int, int, String)   - 
Method in class weka.core. Instances 
 Renames the value of a nominal (or string) attribute value.
   renameAttributeValue(Attribute, String, String)   - 
Method in class weka.core. Instances 
 Renames the value of a nominal (or string) attribute value.
   renameNodeValue(int, String, String)   - 
Method in class weka.classifiers.bayes.net. EditableBayesNet 
 change the name of a value of a node
   Reorder   - Class in  weka.filters.unsupervised.attribute  A filter that generates output with a new order of the attributes.   Reorder()   - 
Constructor for class weka.filters.unsupervised.attribute. Reorder 
  
   RepeatedHillClimber   - Class in  weka.classifiers.bayes.net.search.global  This Bayes Network learning algorithm repeatedly uses hill climbing starting with a randomly generated network structure and return the best structure of the various runs.   RepeatedHillClimber()   - 
Constructor for class weka.classifiers.bayes.net.search.global. RepeatedHillClimber 
  
   RepeatedHillClimber   - Class in  weka.classifiers.bayes.net.search.local  This Bayes Network learning algorithm repeatedly uses hill climbing starting with a randomly generated network structure and return the best structure of the various runs.   RepeatedHillClimber()   - 
Constructor for class weka.classifiers.bayes.net.search.local. RepeatedHillClimber 
  
   repeatLiteralsTipText()   - 
Method in class weka.associations. Tertius 
 Returns the tip text for this property.
   replaceAllBy(Stack<T>)   - 
Method in class weka.core.neighboursearch.covertrees. Stack 
 Replace all elements in the stack with 
 the elements of another given stack.
   replaceMissingTipText()   - 
Method in class weka.filters.supervised.attribute. PLSFilter 
 Returns the tip text for this property
   replaceMissingValues(double[])   - 
Method in class weka.core. BinarySparseInstance 
 Does nothing, since we don't support missing values.
   replaceMissingValues(double[])   - 
Method in class weka.core. Instance 
 Replaces all missing values in the instance with the
 values contained in the given array.
   replaceMissingValues(double[])   - 
Method in class weka.core. SparseInstance 
 Replaces all missing values in the instance with the 
 values contained in the given array.
   ReplaceMissingValues   - Class in  weka.filters.unsupervised.attribute  Replaces all missing values for nominal and numeric attributes in a dataset with the modes and means from the training data.   ReplaceMissingValues()   - 
Constructor for class weka.filters.unsupervised.attribute. ReplaceMissingValues 
  
   replaceMissingValuesTipText()   - 
Method in class weka.filters.unsupervised.attribute. RandomProjection 
 Returns the tip text for this property
   replaceSubstring(String, String, String)   - 
Static method in class weka.core. Utils 
 Replaces with a new string, all occurrences of a string from 
 another string.
   replot()   - 
Method in class weka.gui.boundaryvisualizer. BoundaryPanel 
 Quickly replot the display using cached probability estimates
   reportFrequencyTipText()   - 
Method in class weka.attributeSelection. GeneticSearch 
 Returns the tip text for this property
   REPTree   - Class in  weka.classifiers.trees  Fast decision tree learner.   REPTree()   - 
Constructor for class weka.classifiers.trees. REPTree 
  
   repulsionTipText()   - 
Method in class weka.clusterers. CLOPE 
 Returns the tip text for this property
   resample(Random)   - 
Method in class weka.core. Instances 
 Creates a new dataset of the same size using random sampling
 with replacement.
   Resample   - Class in  weka.filters.supervised.instance  Produces a random subsample of a dataset using either sampling with replacement or without replacement. 
 The original dataset must fit entirely in memory.   Resample()   - 
Constructor for class weka.filters.supervised.instance. Resample 
  
   Resample   - Class in  weka.filters.unsupervised.instance  Produces a random subsample of a dataset using either sampling with replacement or without replacement.   Resample()   - 
Constructor for class weka.filters.unsupervised.instance. Resample 
  
   resampleWithWeights(Instances, Random, boolean[])   - 
Method in class weka.classifiers.meta. Bagging 
 Creates a new dataset of the same size using random sampling
 with replacement according to the given weight vector.
   resampleWithWeights(Random)   - 
Method in class weka.core. Instances 
 Creates a new dataset of the same size using random sampling
 with replacement according to the current instance weights.
   resampleWithWeights(Random, double[])   - 
Method in class weka.core. Instances 
 Creates a new dataset of the same size using random sampling
 with replacement according to the given weight vector.
   ReservoirSample   - Class in  weka.filters.unsupervised.instance  Produces a random subsample of a dataset using the reservoir sampling Algorithm "R" by Vitter.   ReservoirSample()   - 
Constructor for class weka.filters.unsupervised.instance. ReservoirSample 
  
   reset()   - 
Method in class weka.classifiers.functions.neural. NeuralConnection 
 Call this to reset the unit for another run.
   reset()   - 
Method in class weka.classifiers.functions.neural. NeuralNode 
 Call this to reset the value and error for this unit, ready for the next
 run.
   reset()   - 
Method in class weka.classifiers.functions. SPegasos 
 Reset the classifier.
   reset()   - 
Method in class weka.core.converters. AbstractFileLoader 
 Resets the loader ready to read a new data set
   reset()   - 
Method in class weka.core.converters. AbstractLoader 
 Default implementation sets retrieval mode to NONE
   reset()   - 
Method in class weka.core.converters. ArffLoader 
 Resets the Loader ready to read a new data set or the
 same data set again.
   reset()   - 
Method in class weka.core.converters. C45Loader 
 Resets the Loader ready to read a new data set or the
 same data set again.
   reset()   - 
Method in class weka.core.converters. ConverterUtils.DataSource 
 resets the loader.
   reset()   - 
Method in class weka.core.converters. CSVLoader 
 Resets the Loader ready to read a new data set or the
 same data set again.
   reset()   - 
Method in class weka.core.converters. DatabaseLoader 
 Resets the Loader ready to read a new data set
   reset()   - 
Method in class weka.core.converters. LibSVMLoader 
 Resets the Loader ready to read a new data set.
   reset()   - 
Method in interface weka.core.converters. Loader 
 Resets the Loader object ready to begin loading.
   reset()   - 
Method in class weka.core.converters. SerializedInstancesLoader 
 Resets the Loader ready to read a new data set
   reset()   - 
Method in class weka.core.converters. SVMLightLoader 
 Resets the Loader ready to read a new data set.
   reset()   - 
Method in class weka.core.converters. TextDirectoryLoader 
 Resets the loader ready to read a new data set
   reset()   - 
Method in class weka.core.converters. XRFFLoader 
 Resets the Loader ready to read a new data set
   reset()   - 
Method in class weka.core.neighboursearch. PerformanceStats 
 Resets all internal fields/counters.
   reset()   - 
Method in class weka.core.neighboursearch. TreePerformanceStats 
 Resets all internal fields/counters.
   reset()   - 
Static method in class weka.gui.beans. BeanConnection 
 Reset the list of connections
   reset(JComponent)   - 
Static method in class weka.gui.beans. BeanInstance 
 Reset the list of beans
   resetAttIndex(boolean)   - 
Method in class weka.classifiers.lazy. LBR.Indexes 
 Resets the boolean value in AttIndexes array
   resetAttIndexTo(LBR.Indexes)   - 
Method in class weka.classifiers.lazy. LBR.Indexes 
 Resets the boolean value in AttIndexes array based on another set of Indexes
   resetDatasetBasedOn(LBR.Indexes)   - 
Method in class weka.classifiers.lazy. LBR.Indexes 
 Resets the boolean values in Attribute and Instance array to reflect an empty dataset withthe same attributes set as in the incoming Indexes Object
   resetDistribution(Instances)   - 
Method in class weka.classifiers.trees.j48. BinC45Split 
 Sets distribution associated with model.
   resetDistribution(Instances)   - 
Method in class weka.classifiers.trees.j48. C45Split 
 Sets distribution associated with model.
   resetDistribution(Instances)   - 
Method in class weka.classifiers.trees.j48. ClassifierSplitModel 
 Sets distribution associated with model.
   resetInstanceIndex(boolean)   - 
Method in class weka.classifiers.lazy. LBR.Indexes 
 Resets the boolean value in the Instance Indexes array to a specified value
   resetOptions()   - 
Method in class weka.associations. Apriori 
 Resets the options to the default values.
   resetOptions()   - 
Method in class weka.associations. FPGrowth 
 Reset all options to their default values.
   resetOptions()   - 
Method in class weka.associations. PredictiveApriori 
 Resets the options to the default values.
   resetOptions()   - 
Method in class weka.associations. Tertius 
 Resets the options to the default values.
   resetOptions()   - 
Method in class weka.core.converters. AbstractFileSaver 
 resets the options
   resetOptions()   - 
Method in class weka.core.converters. AbstractSaver 
 resets the options
   resetOptions()   - 
Method in class weka.core.converters. ArffSaver 
 Resets the Saver
   resetOptions()   - 
Method in class weka.core.converters. C45Saver 
 Resets the Saver
   resetOptions()   - 
Method in class weka.core.converters. CSVSaver 
 Resets the Saver
   resetOptions()   - 
Method in class weka.core.converters. DatabaseSaver 
 Resets the Saver ready to save a new data set.
   resetOptions()   - 
Method in class weka.core.converters. LibSVMSaver 
 Resets the Saver
   resetOptions()   - 
Method in class weka.core.converters. SerializedInstancesSaver 
 Resets the Saver.
   resetOptions()   - 
Method in class weka.core.converters. SVMLightSaver 
 Resets the Saver.
   resetOptions()   - 
Method in class weka.core.converters. XRFFSaver 
 Resets the Saver
   resetStructure()   - 
Method in class weka.core.converters. AbstractSaver 
 Resets the structure (header information of the instances)
   resetStructure()   - 
Method in class weka.core.converters. DatabaseLoader 
 Resets the structure of instances
   resetTipText()   - 
Method in class weka.classifiers.functions. MultilayerPerceptron 
  
   resetWriter()   - 
Method in class weka.core.converters. AbstractFileSaver 
 Sets the writer to null.
   resetWriter()   - 
Method in class weka.core.converters. SerializedInstancesSaver 
 Resets the writer, setting writer and objectstream to null.
   ResidualModelSelection   - Class in  weka.classifiers.trees.lmt  Helper class for logistic model trees (weka.classifiers.trees.lmt.LMT) to implement the 
 splitting criterion based on residuals.   ResidualModelSelection(int)   - 
Constructor for class weka.classifiers.trees.lmt. ResidualModelSelection 
 Constructor to create ResidualModelSelection object.
   ResidualSplit   - Class in  weka.classifiers.trees.lmt  Helper class for logistic model trees (weka.classifiers.trees.lmt.LMT) to implement the 
 splitting criterion based on residuals of the LogitBoost algorithm.   ResidualSplit(int)   - 
Constructor for class weka.classifiers.trees.lmt. ResidualSplit 
 Creates a split object
   restoreBeans()   - 
Method in class weka.gui.beans. MetaBean 
  
   restoreWeights()   - 
Method in class weka.classifiers.functions.neural. NeuralConnection 
 Call this to have the connection restore from the saved
 weights.
   restoreWeights()   - 
Method in class weka.classifiers.functions.neural. NeuralNode 
 Call this to have the connection restore from the saved
 weights.
   restoreWindows()   - 
Method in class weka.gui. Main 
 restores all windows.
   resultChanged(ResultChangedEvent)   - 
Method in interface weka.gui.sql.event. ResultChangedListener 
 This method gets called when a query has been executed.
   resultChanged(ResultChangedEvent)   - 
Method in class weka.gui.sql. SqlViewer 
 This method gets called when a query has been executed.
   resultChanged(ResultChangedEvent)   - 
Method in class weka.gui.sql. SqlViewerDialog 
 This method gets called when a query has been executed.
   ResultChangedEvent   - Class in  weka.gui.sql.event  An event that is generated when a different Result is activated in the
 ResultPanel.   ResultChangedEvent(Object, String, String, String, String)   - 
Constructor for class weka.gui.sql.event. ResultChangedEvent 
 constructs the event
   ResultChangedListener   - Interface in  weka.gui.sql.event  A listener that is notified if another Result is activated in the
 ResultPanel.   ResultHistoryPanel   - Class in  weka.gui  A component that accepts named stringbuffers and displays the name in a list
 box.   ResultHistoryPanel(JTextComponent)   - 
Constructor for class weka.gui. ResultHistoryPanel 
 Create the result history object
   ResultHistoryPanel.RKeyAdapter   - Class in  weka.gui  Extension of KeyAdapter that implements Serializable.   ResultHistoryPanel.RKeyAdapter()   - 
Constructor for class weka.gui. ResultHistoryPanel.RKeyAdapter 
  
   ResultHistoryPanel.RMouseAdapter   - Class in  weka.gui  Extension of MouseAdapter that implements Serializable.   ResultHistoryPanel.RMouseAdapter()   - 
Constructor for class weka.gui. ResultHistoryPanel.RMouseAdapter 
  
   ResultListener   - Interface in  weka.experiment  Interface for objects able to listen for results obtained
 by a ResultProducer   ResultMatrix   - Class in  weka.experiment  This matrix is a container for the datasets and classifier setups and 
 their statistics.   ResultMatrix()   - 
Constructor for class weka.experiment. ResultMatrix 
 initializes the matrix as 1x1 matrix
   ResultMatrix(int, int)   - 
Constructor for class weka.experiment. ResultMatrix 
 initializes the matrix with the given dimensions
   ResultMatrix(ResultMatrix)   - 
Constructor for class weka.experiment. ResultMatrix 
 initializes the matrix with the values from the given matrix
   ResultMatrixCSV   - Class in  weka.experiment  This matrix is a container for the datasets and classifier setups and 
 their statistics.   ResultMatrixCSV()   - 
Constructor for class weka.experiment. ResultMatrixCSV 
 initializes the matrix as 1x1 matrix
   ResultMatrixCSV(int, int)   - 
Constructor for class weka.experiment. ResultMatrixCSV 
 initializes the matrix with the given dimensions
   ResultMatrixCSV(ResultMatrix)   - 
Constructor for class weka.experiment. ResultMatrixCSV 
 initializes the matrix with the values from the given matrix
   ResultMatrixGnuPlot   - Class in  weka.experiment  This matrix is a container for the datasets and classifier setups and 
 their statistics.   ResultMatrixGnuPlot()   - 
Constructor for class weka.experiment. ResultMatrixGnuPlot 
 initializes the matrix as 1x1 matrix
   ResultMatrixGnuPlot(int, int)   - 
Constructor for class weka.experiment. ResultMatrixGnuPlot 
 initializes the matrix with the given dimensions
   ResultMatrixGnuPlot(ResultMatrix)   - 
Constructor for class weka.experiment. ResultMatrixGnuPlot 
 initializes the matrix with the values from the given matrix
   ResultMatrixHTML   - Class in  weka.experiment  This matrix is a container for the datasets and classifier setups and 
 their statistics.   ResultMatrixHTML()   - 
Constructor for class weka.experiment. ResultMatrixHTML 
 initializes the matrix as 1x1 matrix
   ResultMatrixHTML(int, int)   - 
Constructor for class weka.experiment. ResultMatrixHTML 
 initializes the matrix with the given dimensions
   ResultMatrixHTML(ResultMatrix)   - 
Constructor for class weka.experiment. ResultMatrixHTML 
 initializes the matrix with the values from the given matrix
   ResultMatrixLatex   - Class in  weka.experiment  This matrix is a container for the datasets and classifier setups and 
 their statistics.   ResultMatrixLatex()   - 
Constructor for class weka.experiment. ResultMatrixLatex 
 initializes the matrix as 1x1 matrix
   ResultMatrixLatex(int, int)   - 
Constructor for class weka.experiment. ResultMatrixLatex 
 initializes the matrix with the given dimensions
   ResultMatrixLatex(ResultMatrix)   - 
Constructor for class weka.experiment. ResultMatrixLatex 
 initializes the matrix with the values from the given matrix
   ResultMatrixPlainText   - Class in  weka.experiment  This matrix is a container for the datasets and classifier setups and 
 their statistics.   ResultMatrixPlainText()   - 
Constructor for class weka.experiment. ResultMatrixPlainText 
 initializes the matrix as 1x1 matrix
   ResultMatrixPlainText(int, int)   - 
Constructor for class weka.experiment. ResultMatrixPlainText 
 initializes the matrix with the given dimensions
   ResultMatrixPlainText(ResultMatrix)   - 
Constructor for class weka.experiment. ResultMatrixPlainText 
 initializes the matrix with the values from the given matrix
   ResultMatrixSignificance   - Class in  weka.experiment  This matrix is a container for the datasets and classifier setups and 
 their statistics.   ResultMatrixSignificance()   - 
Constructor for class weka.experiment. ResultMatrixSignificance 
 initializes the matrix as 1x1 matrix
   ResultMatrixSignificance(int, int)   - 
Constructor for class weka.experiment. ResultMatrixSignificance 
 initializes the matrix with the given dimensions
   ResultMatrixSignificance(ResultMatrix)   - 
Constructor for class weka.experiment. ResultMatrixSignificance 
 initializes the matrix with the values from the given matrix
   ResultPanel   - Class in  weka.gui.sql  Represents a panel for displaying the results of a query in table format.   ResultPanel(JFrame)   - 
Constructor for class weka.gui.sql. ResultPanel 
 initializes the panel
   ResultProducer   - Interface in  weka.experiment  This interface defines the methods required for an object 
 that produces results for different randomizations of a dataset.   resultProducerTipText()   - 
Method in class weka.experiment. AveragingResultProducer 
 Returns the tip text for this property
   resultProducerTipText()   - 
Method in class weka.experiment. DatabaseResultProducer 
 Returns the tip text for this property
   resultProducerTipText()   - 
Method in class weka.experiment. LearningRateResultProducer 
 Returns the tip text for this property
   ResultSetHelper   - Class in  weka.gui.sql  Represents an extended JTable, containing a table model based on a ResultSet
 and the corresponding query.   ResultSetHelper(ResultSet)   - 
Constructor for class weka.gui.sql. ResultSetHelper 
 initializes the helper, with unlimited number of rows.
   ResultSetHelper(ResultSet, int)   - 
Constructor for class weka.gui.sql. ResultSetHelper 
 initializes the helper, with the given maximum number of rows (less than
 1 means unlimited).
   resultsetKey()   - 
Method in class weka.experiment. PairedTTester 
 Creates a key that maps resultset numbers to their descriptions.
   resultsetKey()   - 
Method in interface weka.experiment. Tester 
 Creates a key that maps resultset numbers to their descriptions.
   ResultSetTable   - Class in  weka.gui.sql  Represents an extended JTable, containing a table model based on a ResultSet
 and the corresponding query.   ResultSetTable(String, String, String, String, ResultSetTableModel)   - 
Constructor for class weka.gui.sql. ResultSetTable 
 initializes the table
   ResultSetTableCellRenderer   - Class in  weka.gui.sql  Handles the background colors for missing values differently than the
 DefaultTableCellRenderer.   ResultSetTableCellRenderer()   - 
Constructor for class weka.gui.sql. ResultSetTableCellRenderer 
 initializes the Renderer with a standard color
   ResultSetTableCellRenderer(Color, Color)   - 
Constructor for class weka.gui.sql. ResultSetTableCellRenderer 
 initializes the Renderer with the given colors
   ResultSetTableModel   - Class in  weka.gui.sql  The model for an SQL ResultSet.   ResultSetTableModel(ResultSet)   - 
Constructor for class weka.gui.sql. ResultSetTableModel 
 initializes the model, retrieves all rows.
   ResultSetTableModel(ResultSet, int)   - 
Constructor for class weka.gui.sql. ResultSetTableModel 
 initializes the model, retrieves only the given amount of rows (0 means
 all).
   ResultsPanel   - Class in  weka.gui.experiment  This panel controls simple analysis of experimental results.   ResultsPanel()   - 
Constructor for class weka.gui.experiment. ResultsPanel 
 Creates the results panel with no initial experiment.
   ResultVectorTableModel   - Class in  weka.clusterers.forOPTICSAndDBScan.OPTICS_GUI  
 ResultVectorTableModel.java  
 Authors: Rainer Holzmann, Zhanna Melnikova-Albrecht  
 Date: Sep 12, 2004  
 Time: 9:23:31 PM  
 $ Revision 1.4 $     ResultVectorTableModel(FastVector)   - 
Constructor for class weka.clusterers.forOPTICSAndDBScan.OPTICS_GUI. ResultVectorTableModel 
 Constructs a default  DefaultTableModel 
  which is a table of zero columns and zero rows.
   retainAll(Collection<?>)   - 
Method in class weka.core. Trie 
 Retains only the elements in this collection that are contained in 
 the specified collection
   retrieveDir()   - 
Method in class weka.core.converters. AbstractFileSaver 
 Gets the directory
   retrieveDir()   - 
Method in class weka.core.converters. AbstractSaver 
 Default implementation throws an IOException.
   retrieveDir()   - 
Method in interface weka.core.converters. Saver 
 Gets the driectory of the output file
 This method is used in the KnowledgeFlow GUI.
   retrieveFile()   - 
Method in class weka.core.converters. AbstractFileLoader 
 get the File specified as the source
   retrieveFile()   - 
Method in class weka.core.converters. AbstractFileSaver 
 Gets the destination file.
   retrieveFile()   - 
Method in class weka.core.converters. ArffLoader 
 get the File specified as the source
   retrieveFile()   - 
Method in interface weka.core.converters. FileSourcedConverter 
 Return the current source file/ destination file
   retrieveInstances()   - 
Method in class weka.experiment. InstanceQuery 
 Makes a database query using the query set through the -Q option 
 to convert a table into a set of instances
   retrieveInstances(String)   - 
Method in class weka.experiment. InstanceQuery 
 Makes a database query to convert a table into a set of instances
   retrieveURL()   - 
Method in class weka.core.converters. ArffLoader 
 Return the current url
   retrieveURL()   - 
Method in class weka.core.converters. LibSVMLoader 
 Return the current url.
   retrieveURL()   - 
Method in class weka.core.converters. SVMLightLoader 
 Return the current url.
   retrieveURL()   - 
Method in interface weka.core.converters. URLSourcedLoader 
 Return the current url
   retrieveURL()   - 
Method in class weka.core.converters. XRFFLoader 
 Return the current url
   returnLeaves(FastVector[])   - 
Method in class weka.classifiers.trees.m5. RuleNode 
 Return a list containing all the leaves in the tree
   rev()   - 
Method in class weka.core.matrix. DoubleVector 
 Returns the reverse vector
   REVERSED   - 
Static variable in interface weka.gui.graphvisualizer. GraphConstants 
 Types of Edges
   reversedArcs(BayesNet)   - 
Method in class weka.classifiers.bayes.net. BIFReader 
 Count nr of reversed arcs from other network compared to current network
   revertNewLines(String)   - 
Static method in class weka.core. Utils 
 Reverts \r and \n in a string into carriage returns and new lines.
   REVISION   - 
Static variable in class weka.core. Version 
 the revision
   RevisionHandler   - Interface in  weka.core  For classes that should return their source control revision.   RevisionUtils   - Class in  weka.core  Contains utility functions for handling revisions.   RevisionUtils()   - 
Constructor for class weka.core. RevisionUtils 
  
   RevisionUtils.Type   - Enum in  weka.core  Enumeration of source control types.   ridgeTipText()   - 
Method in class weka.classifiers.functions. LinearRegression 
 Returns the tip text for this property
   ridgeTipText()   - 
Method in class weka.classifiers.functions. Logistic 
 Returns the tip text for this property
   ridgeTipText()   - 
Method in class weka.classifiers.functions. RBFNetwork 
 Returns the tip text for this property
   ridgeTipText()   - 
Method in class weka.classifiers.mi. MILR 
 Returns the tip text for this property
   Ridor   - Class in  weka.classifiers.rules  An implementation of a RIpple-DOwn Rule learner. 
  
 It generates a default rule first and then the exceptions for the default rule with the least (weighted) error rate.   Ridor()   - 
Constructor for class weka.classifiers.rules. Ridor 
  
   RIGHT_PARENTHESES   - 
Variable in class weka.experiment. ResultMatrix 
 the right parentheses for enumerating cols/rows
   rightNode()   - 
Method in class weka.classifiers.trees.m5. RuleNode 
 Get the right child of this node
   rightSide(int, Instances)   - 
Method in class weka.classifiers.trees.j48. BinC45Split 
 Prints the condition satisfied by instances in a subset.
   rightSide(int, Instances)   - 
Method in class weka.classifiers.trees.j48. C45Split 
 Prints the condition satisfied by instances in a subset.
   rightSide(int, Instances)   - 
Method in class weka.classifiers.trees.j48. ClassifierSplitModel 
 Prints left side of condition satisfied by instances in subset index.
   rightSide(int, Instances)   - 
Method in class weka.classifiers.trees.j48. GraftSplit 
 Prints condition satisfied by instances in subset index.
   rightSide(int, Instances)   - 
Method in class weka.classifiers.trees.j48. NBTreeNoSplit 
 Does nothing because no condition has to be satisfied.
   rightSide(int, Instances)   - 
Method in class weka.classifiers.trees.j48. NBTreeSplit 
 Prints the condition satisfied by instances in a subset.
   rightSide(int, Instances)   - 
Method in class weka.classifiers.trees.j48. NoSplit 
 Does nothing because no condition has to be satisfied.
   rightSide(int, Instances)   - 
Method in class weka.classifiers.trees.lmt. ResidualSplit 
 Prints the condition satisfied by instances in a subset.
   RINT   - 
Static variable in interface weka.core.mathematicalexpression. sym 
  
   RINT   - 
Static variable in interface weka.filters.unsupervised.instance.subsetbyexpression. sym 
  
   rmCoveredBySuccessives(Instances, FastVector, int)   - 
Static method in class weka.classifiers.rules. RuleStats 
 Static utility function to count the data covered by the 
 rules after the given index in the given rules, and then
 remove them.
   rnorm(int, double, double, Random)   - 
Static method in class weka.core.matrix. Maths 
 Generates a sample of a normal distribution.
   rocAnalysisTipText()   - 
Method in class weka.associations. Tertius 
 Returns the tip text for this property.
   rocToString()   - 
Method in class weka.associations.tertius. Rule 
 Return a String giving the TP-rate and FP-rate of 
 this rule.
   ROOT_FINDER_ACCURACY   - 
Static variable in interface weka.classifiers.lazy.kstar. KStarConstants 
  
   ROOT_FINDER_MAX_ITER   - 
Static variable in interface weka.classifiers.lazy.kstar. KStarConstants 
 How close the root finder for numeric and nominal have to get
   ROOT_NODE   - 
Static variable in class weka.core.xml. XMLOptions 
 the root node.
   ROOT_NODE   - 
Static variable in class weka.core.xml. XMLSerialization 
 the root node of the XML document
   rootMeanPriorSquaredError()   - 
Method in class weka.classifiers. Evaluation 
 Returns the root mean prior squared error.
   rootMeanSquaredError()   - 
Method in class weka.classifiers. Evaluation 
 Returns the root mean squared error.
   rootRelativeSquaredError()   - 
Method in class weka.classifiers. Evaluation 
 Returns the root relative squared error if the class is numeric.
   rotate(double, double, double)   - 
Method in class weka.gui.visualize. PostscriptGraphics 
  
   rotate(double)   - 
Method in class weka.gui.visualize. PostscriptGraphics 
  
   RotationForest   - Class in  weka.classifiers.meta  Class for construction a Rotation Forest.   RotationForest()   - 
Constructor for class weka.classifiers.meta. RotationForest 
 Constructor.
   round(double)   - 
Static method in class weka.core. Utils 
 Rounds a double to the next nearest integer value.
   roundDouble(double, int)   - 
Static method in class weka.core. Utils 
 Rounds a double to the given number of decimal places.
   RPAREN   - 
Static variable in interface weka.core.mathematicalexpression. sym 
  
   RPAREN   - 
Static variable in interface weka.filters.unsupervised.instance.subsetbyexpression. sym 
  
   rsolve(PaceMatrix, IntVector, int)   - 
Method in class weka.classifiers.functions.pace. PaceMatrix 
 Solves upper-triangular equation  
        R x = b  
  On output, the solution is stored in b
   Rule   - Class in  weka.associations.tertius  Class representing a rule with a body and a head.   Rule(boolean, int, boolean, boolean, boolean, boolean)   - 
Constructor for class weka.associations.tertius. Rule 
 Constructor for a rule when the counter-instances are not stored,
 giving all the constraints applied to this rule.
   Rule(Instances, boolean, int, boolean, boolean, boolean, boolean)   - 
Constructor for class weka.associations.tertius. Rule 
 Constructor for a rule when the counter-instances are stored,
 giving all the constraints applied to this rule.
   Rule   - Class in  weka.classifiers.rules  Abstract class of generic rule   Rule()   - 
Constructor for class weka.classifiers.rules. Rule 
  
   Rule   - Class in  weka.classifiers.trees.m5  Generates a single m5 tree or rule   Rule()   - 
Constructor for class weka.classifiers.trees.m5. Rule 
 Constructor declaration
   RuleGeneration   - Class in  weka.associations  Class implementing the rule generation procedure of the predictive apriori algorithm.   RuleGeneration(ItemSet)   - 
Constructor for class weka.associations. RuleGeneration 
 Constructor
   RuleItem   - Class in  weka.associations  Class for storing an (class) association rule.   RuleItem()   - 
Constructor for class weka.associations. RuleItem 
 Constructor for an empty RuleItem
   RuleItem(RuleItem)   - 
Constructor for class weka.associations. RuleItem 
 Constructor that generates a RuleItem out of a given one
   RuleItem(ItemSet, ItemSet, int, int, double[], Hashtable)   - 
Constructor for class weka.associations. RuleItem 
 Constructor
   RuleNode   - Class in  weka.classifiers.trees.m5  Constructs a node for use in an m5 tree or rule   RuleNode(double, double, RuleNode)   - 
Constructor for class weka.classifiers.trees.m5. RuleNode 
 Creates a new  RuleNode  instance.
   rulesMustContainTipText()   - 
Method in class weka.associations. FPGrowth 
 Returns the tip text for this property
   RuleStats   - Class in  weka.classifiers.rules  This class implements the statistics functions used in the 
 propositional rule learner, from the simpler ones like count of
 true/false positive/negatives, filter data based on the ruleset, etc.   RuleStats()   - 
Constructor for class weka.classifiers.rules. RuleStats 
 Default constructor
   RuleStats(Instances, FastVector)   - 
Constructor for class weka.classifiers.rules. RuleStats 
 Constructor that provides ruleset and data
   run()   - 
Method in class weka.associations. Tertius 
 Run the search.
   run()   - 
Method in class weka.gui.beans. FlowRunner 
 Launch all loaded KnowledgeFlow
   RUN_FIELD_NAME   - 
Static variable in class weka.experiment. CrossValidationResultProducer 
 The name of the key field containing the run number
   RUN_FIELD_NAME   - 
Static variable in class weka.experiment. RandomSplitResultProducer 
 The name of the key field containing the run number
   runCommand(String)   - 
Method in class weka.gui. SimpleCLIPanel 
 Executes a simple cli command.
   runExperiment()   - 
Method in class weka.experiment. Experiment 
 Runs all iterations of the experiment, continuing past errors.
   runExperiment()   - 
Method in class weka.experiment. RemoteExperiment 
 Overides runExperiment in Experiment
   runFileLoader(AbstractFileLoader, String[])   - 
Static method in class weka.core.converters. AbstractFileLoader 
 runs the given loader with the provided options
   runFileSaver(AbstractFileSaver, String[])   - 
Static method in class weka.core.converters. AbstractFileSaver 
 runs the given saver with the specified options
   RunNumberPanel   - Class in  weka.gui.experiment  This panel controls configuration of lower and upper run numbers
 in an experiment.   RunNumberPanel()   - 
Constructor for class weka.gui.experiment. RunNumberPanel 
 Creates the panel with no initial experiment.
   RunNumberPanel(Experiment)   - 
Constructor for class weka.gui.experiment. RunNumberPanel 
 Creates the panel with the supplied initial experiment.
   RunPanel   - Class in  weka.gui.experiment  This panel controls the running of an experiment.   RunPanel()   - 
Constructor for class weka.gui.experiment. RunPanel 
 Creates the run panel with no initial experiment.
   RunPanel(Experiment)   - 
Constructor for class weka.gui.experiment. RunPanel 
 Creates the panel with the supplied initial experiment.
   runsTipText()   - 
Method in class weka.classifiers.bayes.net.search.global. GeneticSearch 
  
   runsTipText()   - 
Method in class weka.classifiers.bayes.net.search.global. RepeatedHillClimber 
  
   runsTipText()   - 
Method in class weka.classifiers.bayes.net.search.global. SimulatedAnnealing 
  
   runsTipText()   - 
Method in class weka.classifiers.bayes.net.search.global. TabuSearch 
  
   runsTipText()   - 
Method in class weka.classifiers.bayes.net.search.local. GeneticSearch 
  
   runsTipText()   - 
Method in class weka.classifiers.bayes.net.search.local. RepeatedHillClimber 
  
   runsTipText()   - 
Method in class weka.classifiers.bayes.net.search.local. SimulatedAnnealing 
  
   runsTipText()   - 
Method in class weka.classifiers.bayes.net.search.local. TabuSearch 
  
   runTokenizer(Tokenizer, String[])   - 
Static method in class weka.core.tokenizers. Tokenizer 
 initializes the given tokenizer with the given options and runs the
 tokenizer over all the remaining strings in the options array.
 
 
    
 S  
 
   s_fileFormatsAvailable   - 
Static variable in class weka.gui.beans. SerializedModelSaver 
 Available file formats.
   s_startupListeners   - 
Static variable in class weka.gui.beans. KnowledgeFlowApp 
  
   sameClauseAs(Rule)   - 
Method in class weka.associations.tertius. Rule 
 Test if this rule and another rule correspond to the same clause.
   sameClauseTipText()   - 
Method in class weka.associations. Tertius 
 Returns the tip text for this property.
   SAMPLE_SIZE_NAME   - 
Static variable in class weka.classifiers.evaluation. ThresholdCurve 
 attribute name: Sample Size
   sampleSizePercentTipText()   - 
Method in class weka.classifiers.meta. GridSearch 
 Returns the tip text for this property
   sampleSizePercentTipText()   - 
Method in class weka.filters.supervised.instance. Resample 
 Returns the tip text for this property.
   sampleSizePercentTipText()   - 
Method in class weka.filters.unsupervised.instance. Resample 
 Returns the tip text for this property
   sampleSizeTipText()   - 
Method in class weka.attributeSelection. ReliefFAttributeEval 
 Returns the tip text for this property
   sampleSizeTipText()   - 
Method in class weka.classifiers.functions. LeastMedSq 
 Returns the tip text for this property
   sampleSizeTipText()   - 
Method in class weka.filters.unsupervised.instance. ReservoirSample 
 Returns the tip text for this property
   satisfies(Instance)   - 
Method in class weka.associations.tertius. AttributeValueLiteral 
  
   satisfies(Instance)   - 
Method in class weka.associations.tertius. Literal 
  
   save(StringBuffer)   - 
Method in class weka.gui. SaveBuffer 
 Save a buffer
   saveBatch()   - 
Method in class weka.gui.beans. Saver 
 Saves instances in batch mode
   saveBinary(File, Object, Instances)   - 
Static method in class weka.gui.beans. SerializedModelSaver 
 Save a model in binary form.
   SaveBuffer   - Class in  weka.gui  This class handles the saving of StringBuffers to files.   SaveBuffer(Logger, Component)   - 
Constructor for class weka.gui. SaveBuffer 
 Constructor
   saveComponent()   - 
Method in class weka.gui.visualize. PrintableComponent 
 displays a save dialog for saving the panel to a file.
   saveComponent()   - 
Method in interface weka.gui.visualize. PrintableHandler 
 displays a save dialog for saving the component to a file.
   saveComponent()   - 
Method in class weka.gui.visualize. PrintablePanel 
 displays a save dialog for saving the panel to a file.
   saveFile()   - 
Method in class weka.gui.arffviewer. ArffViewerMainPanel 
 saves the current data into a file
   saveFileAs()   - 
Method in class weka.gui.arffviewer. ArffViewerMainPanel 
 saves the current data into a new file
   saveInstanceDataTipText()   - 
Method in class weka.classifiers.trees. ADTree 
  
   saveInstanceDataTipText()   - 
Method in class weka.classifiers.trees. J48 
 Returns the tip text for this property
   saveInstanceDataTipText()   - 
Method in class weka.classifiers.trees. J48graft 
 Returns the tip text for this property
   saveInstanceDataTipText()   - 
Method in class weka.clusterers. Cobweb 
 Returns the tip text for this property
   saveInstancesTipText()   - 
Method in class weka.classifiers.trees. M5P 
 Returns the tip text for this property
   saveInstancesToFile(AbstractFileSaver, Instances)   - 
Method in class weka.gui.explorer. PreprocessPanel 
 saves the data with the specified saver
   saveKOML(File, Object, Instances)   - 
Static method in class weka.gui.beans. SerializedModelSaver 
 Save a model in KOML deep object serialized XML form.
   saveLayout(OutputStream)   - 
Method in class weka.gui.beans. KnowledgeFlowApp 
 Save the knowledge flow into the OutputStream passed at input.
   saveModel()   - 
Method in class weka.gui.beans. Classifier 
  
   saveModel()   - 
Method in class weka.gui.beans. Clusterer 
  
   Saver   - Interface in  weka.core.converters  Interface to something that can save Instances to an output destination in some
 format.   Saver   - Class in  weka.gui.beans  Saves data sets using weka.core.converter classes   Saver()   - 
Constructor for class weka.gui.beans. Saver 
 Contsructor
   SAVER_DIALOG   - 
Static variable in class weka.gui. ConverterFileChooser 
 the saver dialog
   SaverBeanInfo   - Class in  weka.gui.beans  Bean info class for the saver bean   SaverBeanInfo()   - 
Constructor for class weka.gui.beans. SaverBeanInfo 
  
   SaverCustomizer   - Class in  weka.gui.beans  GUI Customizer for the saver bean   SaverCustomizer()   - 
Constructor for class weka.gui.beans. SaverCustomizer 
 Constructor
   saveSize()   - 
Method in class weka.gui.sql. SqlViewer 
 obtains the size of the panel and saves it in the history.
   saveToFile(String, Object)   - 
Static method in class weka.core. Debug 
 writes the serialized object to the speicified file
   saveWeights()   - 
Method in class weka.classifiers.functions.neural. NeuralConnection 
 Call this to have the connection save the current
 weights.
   saveWeights()   - 
Method in class weka.classifiers.functions.neural. NeuralNode 
 Call this to have the connection save the current
 weights.
   saveWorkingInstancesToFileQ()   - 
Method in class weka.gui.explorer. PreprocessPanel 
 Queries the user for a file to save instances as, then saves the
 instances in a background process.
   saveXStream(File, Object, Instances)   - 
Static method in class weka.gui.beans. SerializedModelSaver 
 Save a model in XStream deep object serialized XML form.
   scalarMultiply(double)   - 
Method in class weka.core. AlgVector 
 Computes the scalar product of this vector with a scalar
   scale(int)   - 
Method in class weka.gui.beans. BeanVisual 
 Reduce this BeanVisual's icon size by the given factor
   scale(double, double)   - 
Method in class weka.gui.visualize. PostscriptGraphics 
  
   scaleTipText()   - 
Method in class weka.filters.unsupervised.attribute. Normalize 
 Returns the tip text for this property.
   Scanner   - Class in  weka.core.mathematicalexpression  A scanner for mathematical expressions.   Scanner(InputStream, SymbolFactory)   - 
Constructor for class weka.core.mathematicalexpression. Scanner 
  
   Scanner(Reader)   - 
Constructor for class weka.core.mathematicalexpression. Scanner 
 Creates a new scanner
 There is also a java.io.InputStream version of this constructor.
   Scanner(InputStream)   - 
Constructor for class weka.core.mathematicalexpression. Scanner 
 Creates a new scanner.
   Scanner   - Class in  weka.filters.unsupervised.instance.subsetbyexpression  A scanner for evaluating whether an Instance is to be included in a subset
 or not.   Scanner(InputStream, SymbolFactory)   - 
Constructor for class weka.filters.unsupervised.instance.subsetbyexpression. Scanner 
  
   Scanner(Reader)   - 
Constructor for class weka.filters.unsupervised.instance.subsetbyexpression. Scanner 
 Creates a new scanner
 There is also a java.io.InputStream version of this constructor.
   Scanner(InputStream)   - 
Constructor for class weka.filters.unsupervised.instance.subsetbyexpression. Scanner 
 Creates a new scanner.
   ScatterPlotMatrix   - Class in  weka.gui.beans  Bean that encapsulates weka.gui.visualize.MatrixPanel for displaying a
 scatter plot matrix.   ScatterPlotMatrix()   - 
Constructor for class weka.gui.beans. ScatterPlotMatrix 
  
   ScatterPlotMatrixBeanInfo   - Class in  weka.gui.beans  Bean info class for the scatter plot matrix bean   ScatterPlotMatrixBeanInfo()   - 
Constructor for class weka.gui.beans. ScatterPlotMatrixBeanInfo 
  
   ScatterSearchV1   - Class in  weka.attributeSelection  Class for performing the Sequential Scatter Search.   ScatterSearchV1()   - 
Constructor for class weka.attributeSelection. ScatterSearchV1 
  
   ScatterSearchV1.Subset   - Class in  weka.attributeSelection      ScatterSearchV1.Subset(BitSet, double)   - 
Constructor for class weka.attributeSelection. ScatterSearchV1.Subset 
  
   Scoreable   - Interface in  weka.classifiers.bayes.net.search.local  Interface for allowing to score a classifier   scoreTypeTipText()   - 
Method in class weka.classifiers.bayes.net.search.local. LocalScoreSearchAlgorithm 
  
   scrollToVisible(int, int)   - 
Method in class weka.gui. JTableHelper 
 Assumes table is contained in a JScrollPane.
   scrollToVisible(JTable, int, int)   - 
Static method in class weka.gui. JTableHelper 
 Assumes table is contained in a JScrollPane.
   search()   - 
Method in class weka.associations. Tertius 
 Search in the space of hypotheses the rules that have the highest 
 confirmation.
   search(ASEvaluation, Instances)   - 
Method in class weka.attributeSelection. ASSearch 
 Searches the attribute subset/ranking space.
   search(ASEvaluation, Instances)   - 
Method in class weka.attributeSelection. BestFirst 
 Searches the attribute subset space by best first search
   search(ASEvaluation, Instances)   - 
Method in class weka.attributeSelection. ExhaustiveSearch 
 Searches the attribute subset space using an exhaustive search.
   search(ASEvaluation, Instances)   - 
Method in class weka.attributeSelection. GeneticSearch 
 Searches the attribute subset space using a genetic algorithm.
   search(ASEvaluation, Instances)   - 
Method in class weka.attributeSelection. GreedyStepwise 
 Searches the attribute subset space by forward selection.
   search(ASEvaluation, Instances)   - 
Method in class weka.attributeSelection. LinearForwardSelection 
 Searches the attribute subset space by linear forward selection
   search(ASEvaluation, Instances)   - 
Method in class weka.attributeSelection. RaceSearch 
 Searches the attribute subset space by racing cross validation
 errors of competing subsets
   search(ASEvaluation, Instances)   - 
Method in class weka.attributeSelection. RandomSearch 
 Searches the attribute subset space randomly.
   search(ASEvaluation, Instances)   - 
Method in class weka.attributeSelection. Ranker 
 Kind of a dummy search algorithm.
   search(ASEvaluation, Instances)   - 
Method in class weka.attributeSelection. RankSearch 
 Ranks attributes using the specified attribute evaluator and then
 searches the ranking using the supplied subset evaluator.
   search(ASEvaluation, Instances)   - 
Method in class weka.attributeSelection. ScatterSearchV1 
 Searches the attribute subset space using Scatter Search.
   search(ASEvaluation, Instances)   - 
Method in class weka.attributeSelection. SubsetSizeForwardSelection 
 Searches the attribute subset space by subset size forward selection
   search(BayesNet, Instances)   - 
Method in class weka.classifiers.bayes.net.search.global. K2 
 search determines the network structure/graph of the network
 with the K2 algorithm, restricted by its initial structure (which can
 be an empty graph, or a Naive Bayes graph.
   search(BayesNet, Instances)   - 
Method in class weka.classifiers.bayes.net.search.global. SimulatedAnnealing 
  
   search(BayesNet, Instances)   - 
Method in class weka.classifiers.bayes.net.search.local. K2 
 search determines the network structure/graph of the network
 with the K2 algorithm, restricted by its initial structure (which can
 be an empty graph, or a Naive Bayes graph.
   search(BayesNet, Instances)   - 
Method in class weka.classifiers.bayes.net.search.local. SimulatedAnnealing 
  
   search()   - 
Method in class weka.gui.arffviewer. ArffPanel 
 searches for a string in the cells
   search()   - 
Method in class weka.gui.arffviewer. ArffViewerMainPanel 
 searches for a string in the cells
   search(Vector, String)   - 
Method in class weka.gui. HierarchyPropertyParser 
 Helper function to search for the given target string in a 
 given vector in which the elements' value may hopefully is equal
 to the target.
   SearchAlgorithm   - Class in  weka.classifiers.bayes.net.search  This is the base class for all search algorithms for learning Bayes networks.   SearchAlgorithm()   - 
Constructor for class weka.classifiers.bayes.net.search. SearchAlgorithm 
 c'tor
   searchAlgorithmTipText()   - 
Method in class weka.classifiers.bayes. BayesNet 
  
   searchBackwardsTipText()   - 
Method in class weka.attributeSelection. GreedyStepwise 
 Returns the tip text for this property
   searchFinish()   - 
Method in class weka.core.neighboursearch. PerformanceStats 
 Signals end of the nearest neighbour search.
   searchFinish()   - 
Method in class weka.core.neighboursearch. TreePerformanceStats 
 Signals end of the nearest neighbour search.
   SEARCHPATH_ALL   - 
Static variable in class weka.classifiers.trees. ADTree 
 search mode: Expand all paths
   SEARCHPATH_HEAVIEST   - 
Static variable in class weka.classifiers.trees. ADTree 
 search mode: Expand the heaviest path
   SEARCHPATH_RANDOM   - 
Static variable in class weka.classifiers.trees. ADTree 
 search mode: Expand a random path
   SEARCHPATH_ZPURE   - 
Static variable in class weka.classifiers.trees. ADTree 
 search mode: Expand the best z-pure path
   searchPathTipText()   - 
Method in class weka.classifiers.trees. ADTree 
  
   searchPercentTipText()   - 
Method in class weka.attributeSelection. RandomSearch 
 Returns the tip text for this property
   searchPoints(int, int, boolean)   - 
Method in class weka.gui.visualize. Plot2D 
 Pops up a window displaying attribute information on any instances
 at a point+-plotting_point_size (in panel coordinates)
   searchStart()   - 
Method in class weka.core.neighboursearch. PerformanceStats 
 Signals start of the nearest neighbour search.
   searchStart()   - 
Method in class weka.core.neighboursearch. TreePerformanceStats 
 Signals start of the nearest neighbour search.
   searchTerminationTipText()   - 
Method in class weka.attributeSelection. BestFirst 
 Returns the tip text for this property
   searchTerminationTipText()   - 
Method in class weka.attributeSelection. LinearForwardSelection 
 Returns the tip text for this property
   searchTipText()   - 
Method in class weka.classifiers.meta. AttributeSelectedClassifier 
 Returns the tip text for this property
   searchTipText()   - 
Method in class weka.classifiers.rules. DecisionTable 
 Returns the tip text for this property
   searchTipText()   - 
Method in class weka.filters.supervised.attribute. AttributeSelection 
 Returns the tip text for this property
   secondInstanceProduced(InstanceEvent)   - 
Method in class weka.gui.streams. InstanceJoiner 
  
   secondInstanceProduced(InstanceEvent)   - 
Method in interface weka.gui.streams. SerialInstanceListener 
  
   secondValueIndexTipText()   - 
Method in class weka.filters.unsupervised.attribute. MergeTwoValues 
  
   secondValueIndexTipText()   - 
Method in class weka.filters.unsupervised.attribute. SwapValues 
  
   seedTipText()   - 
Method in class weka.attributeSelection. CostSensitiveASEvaluation 
 Returns the tip text for this property
   seedTipText()   - 
Method in class weka.attributeSelection. GeneticSearch 
 Returns the tip text for this property
   seedTipText()   - 
Method in class weka.attributeSelection. OneRAttributeEval 
 Returns a string for this option suitable for display in the gui
 as a tip text
   seedTipText()   - 
Method in class weka.attributeSelection. ReliefFAttributeEval 
 Returns the tip text for this property
   seedTipText()   - 
Method in class weka.attributeSelection. ScatterSearchV1 
 Returns the tip text for this property
   seedTipText()   - 
Method in class weka.attributeSelection. SubsetSizeForwardSelection 
 Returns the tip text for this property
   seedTipText()   - 
Method in class weka.attributeSelection. WrapperSubsetEval 
 Returns the tip text for this property
   seedTipText()   - 
Method in class weka.classifiers.bayes.net.search.global. GeneticSearch 
  
   seedTipText()   - 
Method in class weka.classifiers.bayes.net.search.global. RepeatedHillClimber 
  
   seedTipText()   - 
Method in class weka.classifiers.bayes.net.search.global. SimulatedAnnealing 
  
   seedTipText()   - 
Method in class weka.classifiers.bayes.net.search.local. GeneticSearch 
  
   seedTipText()   - 
Method in class weka.classifiers.bayes.net.search.local. RepeatedHillClimber 
  
   seedTipText()   - 
Method in class weka.classifiers.bayes.net.search.local. SimulatedAnnealing 
  
   seedTipText()   - 
Method in class weka.classifiers.functions. MultilayerPerceptron 
  
   seedTipText()   - 
Method in class weka.classifiers.functions.supportVector. RegOptimizer 
 Returns the tip text for this property
   seedTipText()   - 
Method in class weka.classifiers.functions. VotedPerceptron 
 Returns the tip text for this property
   seedTipText()   - 
Method in class weka.classifiers.functions. Winnow 
 Returns the tip text for this property
   seedTipText()   - 
Method in class weka.classifiers.meta. MultiScheme 
 Returns the tip text for this property
   seedTipText()   - 
Method in class weka.classifiers. RandomizableClassifier 
 Returns the tip text for this property
   seedTipText()   - 
Method in class weka.classifiers. RandomizableIteratedSingleClassifierEnhancer 
 Returns the tip text for this property
   seedTipText()   - 
Method in class weka.classifiers. RandomizableMultipleClassifiersCombiner 
 Returns the tip text for this property
   seedTipText()   - 
Method in class weka.classifiers. RandomizableSingleClassifierEnhancer 
 Returns the tip text for this property
   seedTipText()   - 
Method in class weka.classifiers.rules. ConjunctiveRule 
 Returns the tip text for this property
   seedTipText()   - 
Method in class weka.classifiers.rules. JRip 
 Returns the tip text for this property
   seedTipText()   - 
Method in class weka.classifiers.rules. PART 
 Returns the tip text for this property
   seedTipText()   - 
Method in class weka.classifiers.rules. Ridor 
 Returns the tip text for this property
   seedTipText()   - 
Method in class weka.classifiers.trees. J48 
 Returns the tip text for this property
   seedTipText()   - 
Method in class weka.classifiers.trees. RandomForest 
 Returns the tip text for this property
   seedTipText()   - 
Method in class weka.classifiers.trees. RandomTree 
 Returns the tip text for this property
   seedTipText()   - 
Method in class weka.classifiers.trees. REPTree 
 Returns the tip text for this property
   seedTipText()   - 
Method in class weka.clusterers. Cobweb 
 Returns the tip text for this property
   seedTipText()   - 
Method in class weka.clusterers. RandomizableClusterer 
 Returns the tip text for this property
   seedTipText()   - 
Method in class weka.clusterers. RandomizableDensityBasedClusterer 
 Returns the tip text for this property
   seedTipText()   - 
Method in class weka.clusterers. RandomizableSingleClustererEnhancer 
 Returns the tip text for this property
   seedTipText()   - 
Method in class weka.core.neighboursearch.balltrees. MiddleOutConstructor 
 Returns the tip text for this property.
   seedTipText()   - 
Method in class weka.datagenerators. DataGenerator 
 Returns the tip text for this property
   seedTipText()   - 
Method in class weka.filters.supervised.attribute. ClassOrder 
 Returns the tip text for this property
   seedTipText()   - 
Method in class weka.filters.supervised.instance. StratifiedRemoveFolds 
 Returns the tip text for this property
   seedTipText()   - 
Method in class weka.filters.unsupervised.attribute. PropositionalToMultiInstance 
 Returns the tip text for this property
   seedTipText()   - 
Method in class weka.filters.unsupervised.attribute. RandomSubset 
 Returns the tip text for this property.
   seedTipText()   - 
Method in class weka.filters.unsupervised.instance. RemoveFolds 
 Returns the tip text for this property
   seedTipText()   - 
Method in class weka.gui.beans. CrossValidationFoldMaker 
 Tip text for this property
   seedTipText()   - 
Method in class weka.gui.beans. TrainTestSplitMaker 
 Tip text for this property
   select(int, int[], int, int, int)   - 
Method in class weka.core.neighboursearch.balltrees. MedianOfWidestDimension 
 Implements computation of the kth-smallest element according
 to Manber's "Introduction to Algorithms".
   select(int, int[], int, int, int)   - 
Method in class weka.core.neighboursearch.kdtrees. MedianOfWidestDimension 
 Implements computation of the kth-smallest element according
 to Manber's "Introduction to Algorithms".
   select(String)   - 
Method in class weka.experiment. DatabaseUtils 
 Executes a SQL SELECT query that returns a ResultSet.
   SelectAttributes(ASEvaluation, String[])   - 
Static method in class weka.attributeSelection. AttributeSelection 
 Perform attribute selection with a particular evaluator and
 a set of options specifying search method and input file etc.
   SelectAttributes(Instances)   - 
Method in class weka.attributeSelection. AttributeSelection 
 Perform attribute selection on the supplied training instances.
   SelectAttributes(ASEvaluation, String[], Instances)   - 
Static method in class weka.attributeSelection. AttributeSelection 
 Perform attribute selection with a particular evaluator and
 a set of options specifying search method and options for the
 search method and evaluator.
   selectAttributesCVSplit(Instances)   - 
Method in class weka.attributeSelection. AttributeSelection 
 Select attributes for a split of the data.
   selectedAttributes()   - 
Method in class weka.attributeSelection. AttributeSelection 
 get the final selected set of attributes.
   SelectedTag   - Class in  weka.core  Represents a selected value from a finite set of values, where each
 value is a Tag (i.e.   SelectedTag(int, Tag[])   - 
Constructor for class weka.core. SelectedTag 
 Creates a new  SelectedTag  instance.
   SelectedTag(String, Tag[])   - 
Constructor for class weka.core. SelectedTag 
 Creates a new  SelectedTag  instance.
   SelectedTagEditor   - Class in  weka.gui  A PropertyEditor that uses tags, where the tags are obtained from a
 weka.core.SelectedTag object.   SelectedTagEditor()   - 
Constructor for class weka.gui. SelectedTagEditor 
  
   SELECTION_GREEDY   - 
Static variable in class weka.classifiers.functions. LinearRegression 
 Attribute selection method: Greedy method
   SELECTION_M5   - 
Static variable in class weka.classifiers.functions. LinearRegression 
 Attribute selection method: M5 method
   SELECTION_NONE   - 
Static variable in class weka.classifiers.functions. LinearRegression 
 Attribute selection method: No attribute selection
   selectionThresholdTipText()   - 
Method in class weka.attributeSelection. RaceSearch 
 Returns the tip text for this property
   selectModel(Instances)   - 
Method in class weka.classifiers.trees.j48. BinC45ModelSelection 
 Selects C4.5-type split for the given dataset.
   selectModel(Instances, Instances)   - 
Method in class weka.classifiers.trees.j48. BinC45ModelSelection 
 Selects C4.5-type split for the given dataset.
   selectModel(Instances)   - 
Method in class weka.classifiers.trees.j48. C45ModelSelection 
 Selects C4.5-type split for the given dataset.
   selectModel(Instances, Instances)   - 
Method in class weka.classifiers.trees.j48. C45ModelSelection 
 Selects C4.5-type split for the given dataset.
   selectModel(Instances)   - 
Method in class weka.classifiers.trees.j48. ModelSelection 
 Selects a model for the given dataset.
   selectModel(Instances, Instances)   - 
Method in class weka.classifiers.trees.j48. ModelSelection 
 Selects a model for the given train data using the given test data
   selectModel(Instances)   - 
Method in class weka.classifiers.trees.j48. NBTreeModelSelection 
 Selects NBTree-type split for the given dataset.
   selectModel(Instances, Instances)   - 
Method in class weka.classifiers.trees.j48. NBTreeModelSelection 
 Selects NBTree-type split for the given dataset.
   selectModel(Instances, double[][], double[][])   - 
Method in class weka.classifiers.trees.lmt. ResidualModelSelection 
 Selects split based on residuals for the given dataset.
   selectModel(Instances)   - 
Method in class weka.classifiers.trees.lmt. ResidualModelSelection 
 Method not in use
   selectModel(Instances, Instances)   - 
Method in class weka.classifiers.trees.lmt. ResidualModelSelection 
 Method not in use
   SEND_INSTANCES   - 
Static variable in class weka.gui.treevisualizer. TreeDisplayEvent 
 Command to remove instances from this node and send them to the 
 VisualizePanel.
   separable(DoubleVector, int, int, double)   - 
Method in class weka.classifiers.functions.pace. ChisqMixture 
 Return true if a value can be considered for mixture estimation
  separately from the data indexed between i0 and i1
   separable(DoubleVector, int, int, double)   - 
Method in class weka.classifiers.functions.pace. MixtureDistribution 
 Return true if a value can be considered for mixture estimatino
  separately from the data indexed between i0 and i1
   separable(DoubleVector, int, int, double)   - 
Method in class weka.classifiers.functions.pace. NormalMixture 
 Return true if a value can be considered for mixture estimatino
  separately from the data indexed between i0 and i1
   seq(int, int)   - 
Static method in class weka.core.matrix. IntVector 
 Generates an IntVector that stores all integers inclusively between
  two integers.
   Sequence   - Class in  weka.associations.gsp  Class representing a sequence of elements/itemsets.   Sequence()   - 
Constructor for class weka.associations.gsp. Sequence 
 Constructor.
   Sequence(FastVector)   - 
Constructor for class weka.associations.gsp. Sequence 
 Constructor accepting a set of elements as parameter.
   Sequence(int)   - 
Constructor for class weka.associations.gsp. Sequence 
 Constructor accepting an int value as parameter to set the support count.
   SequentialDatabase   - Class in  weka.clusterers.forOPTICSAndDBScan.Databases  
 SequentialDatabase.java  
 Authors: Rainer Holzmann, Zhanna Melnikova-Albrecht, Matthias Schubert  
 Date: Aug 20, 2004  
 Time: 1:23:38 PM  
 $ Revision 1.4 $     SequentialDatabase(Instances)   - 
Constructor for class weka.clusterers.forOPTICSAndDBScan.Databases. SequentialDatabase 
 Constructs a new sequential database and holds the original instances
   SERFileFilter   - Class in  weka.clusterers.forOPTICSAndDBScan.OPTICS_GUI  
 SERFileFilter.java  
 Authors: Rainer Holzmann, Zhanna Melnikova-Albrecht  
 Date: Sep 15, 2004  
 Time: 6:54:56 PM  
 $ Revision 1.4 $     SERFileFilter(String, String)   - 
Constructor for class weka.clusterers.forOPTICSAndDBScan.OPTICS_GUI. SERFileFilter 
  
   SERIAL_VERSION_UID   - 
Static variable in class weka.core. SerializationHelper 
 the field name of serialVersionUID.
   SerialInstanceListener   - Interface in  weka.gui.streams  Defines an interface for objects able to produce two output streams of
 instances.   SerializationHelper   - Class in  weka.core  A helper class for determining serialVersionUIDs and checking whether
 classes contain one and/or need one.   SerializationHelper()   - 
Constructor for class weka.core. SerializationHelper 
  
   serialize(Object)   - 
Static method in class weka.core.xml. XStream 
 Serializes the supplied object xml
   SERIALIZED_OBJ_FILE_EXTENSION   - 
Static variable in class weka.core. Instances 
 The filename extension that should be used for bin.
   SerializedClassifier   - Class in  weka.classifiers.misc  A wrapper around a serialized classifier model.   SerializedClassifier()   - 
Constructor for class weka.classifiers.misc. SerializedClassifier 
  
   serializedClassifierFileTipText()   - 
Method in class weka.filters.supervised.attribute. AddClassification 
 Returns the tip text for this property.
   SerializedInstancesLoader   - Class in  weka.core.converters  Reads a source that contains serialized Instances.   SerializedInstancesLoader()   - 
Constructor for class weka.core.converters. SerializedInstancesLoader 
  
   SerializedInstancesSaver   - Class in  weka.core.converters  Serializes the instances to a file with extension bsi.   SerializedInstancesSaver()   - 
Constructor for class weka.core.converters. SerializedInstancesSaver 
 Constructor.
   SerializedModelSaver   - Class in  weka.gui.beans  A bean that saves serialized models   SerializedModelSaver()   - 
Constructor for class weka.gui.beans. SerializedModelSaver 
 Constructor.
   SerializedModelSaverBeanInfo   - Class in  weka.gui.beans  Bean info class for the serialized model saver bean   SerializedModelSaverBeanInfo()   - 
Constructor for class weka.gui.beans. SerializedModelSaverBeanInfo 
  
   SerializedModelSaverCustomizer   - Class in  weka.gui.beans  GUI Customizer for the SerializedModelSaver bean   SerializedModelSaverCustomizer()   - 
Constructor for class weka.gui.beans. SerializedModelSaverCustomizer 
 Constructor
   SerializedObject   - Class in  weka.core  Class for storing an object in serialized form in memory.   SerializedObject(Object)   - 
Constructor for class weka.core. SerializedObject 
 Creates a new serialized object (without compression).
   SerializedObject(Object, boolean)   - 
Constructor for class weka.core. SerializedObject 
 Creates a new serialized object.
   serializePMMLModel(PMMLModel, String)   - 
Static method in class weka.core.pmml. PMMLFactory 
 Serialize a  PMMLModel  object that encapsulates a PMML model
   serializePMMLModel(PMMLModel, File)   - 
Static method in class weka.core.pmml. PMMLFactory 
 Serialize a  PMMLModel  object that encapsulates a PMML model
   serializePMMLModel(PMMLModel, OutputStream)   - 
Static method in class weka.core.pmml. PMMLFactory 
 Serialize a  PMMLModel  object that encapsulates a PMML model
   SerialUIDChanger   - Class in  weka.core.xml  This class enables one to change the UID of a serialized object and therefore
 not losing the data stored in the binary format.   SerialUIDChanger()   - 
Constructor for class weka.core.xml. SerialUIDChanger 
  
   SERObject   - Class in  weka.clusterers.forOPTICSAndDBScan.OPTICS_GUI  
 SERObject.java  
 Authors: Rainer Holzmann, Zhanna Melnikova-Albrecht  
 Date: Sep 15, 2004  
 Time: 9:43:00 PM  
 $ Revision 1.4 $     SERObject(FastVector, int, int, double, int, boolean, String, String, int, String)   - 
Constructor for class weka.clusterers.forOPTICSAndDBScan.OPTICS_GUI. SERObject 
  
   set(int, double)   - 
Method in class weka.core.matrix. DoubleVector 
 Set a single element.
   set(double)   - 
Method in class weka.core.matrix. DoubleVector 
 Set all elements to a value
   set(int, int, double)   - 
Method in class weka.core.matrix. DoubleVector 
 Set some elements to a value
   set(int, int, double[], int)   - 
Method in class weka.core.matrix. DoubleVector 
 Set some elements using a 2-D array
   set(DoubleVector)   - 
Method in class weka.core.matrix. DoubleVector 
 Set the elements using a DoubleVector
   set(int, int, DoubleVector, int)   - 
Method in class weka.core.matrix. DoubleVector 
 Set some elements using a DoubleVector.
   set(int)   - 
Method in class weka.core.matrix. IntVector 
 Sets the value of an element.
   set(int, int, int[], int)   - 
Method in class weka.core.matrix. IntVector 
 Sets the values of elements from an int array.
   set(int, int, IntVector, int)   - 
Method in class weka.core.matrix. IntVector 
 Sets the values of elements from another IntVector.
   set(IntVector)   - 
Method in class weka.core.matrix. IntVector 
 Sets the values of elements from another IntVector.
   set(int, int)   - 
Method in class weka.core.matrix. IntVector 
 Sets a single element.
   set(int, int, double)   - 
Method in class weka.core.matrix. Matrix 
 Set a single element.
   set(int, T)   - 
Method in class weka.core.neighboursearch.covertrees. Stack 
 Sets the ith element in the stack.
   set(int, Object)   - 
Method in class weka.gui. CheckBoxList.CheckBoxListModel 
 Replaces the element at the specified position in this list with the 
 specified element.
   setAcuity(double)   - 
Method in class weka.clusterers. Cobweb 
 set the acuity.
   setAdditionalMeasures(String[])   - 
Method in class weka.experiment. AveragingResultProducer 
 Set a list of method names for additional measures to look for
 in SplitEvaluators.
   setAdditionalMeasures(String[])   - 
Method in class weka.experiment. ClassifierSplitEvaluator 
 Set a list of method names for additional measures to look for
 in Classifiers.
   setAdditionalMeasures(String[])   - 
Method in class weka.experiment. CrossValidationResultProducer 
 Set a list of method names for additional measures to look for
 in SplitEvaluators.
   setAdditionalMeasures(String[])   - 
Method in class weka.experiment. DatabaseResultProducer 
 Set a list of method names for additional measures to look for
 in SplitEvaluators.
   setAdditionalMeasures(String[])   - 
Method in class weka.experiment. DensityBasedClustererSplitEvaluator 
 Set a list of method names for additional measures to look for
 in Classifiers.
   setAdditionalMeasures(String[])   - 
Method in class weka.experiment. LearningRateResultProducer 
 Set a list of method names for additional measures to look for
 in SplitEvaluators.
   setAdditionalMeasures(String[])   - 
Method in class weka.experiment. RandomSplitResultProducer 
 Set a list of method names for additional measures to look for
 in SplitEvaluators.
   setAdditionalMeasures(String[])   - 
Method in class weka.experiment. RegressionSplitEvaluator 
 Set a list of method names for additional measures to look for
 in Classifiers.
   setAdditionalMeasures(String[])   - 
Method in interface weka.experiment. ResultProducer 
 Sets a list of method names for additional measures to look for
 in SplitEvaluators.
   setAdditionalMeasures(String[])   - 
Method in interface weka.experiment. SplitEvaluator 
 Sets a list of method names for additional measures to look for
 in SplitEvaluators.
   setAdjustWeights(boolean)   - 
Method in class weka.filters.supervised.instance. SpreadSubsample 
 Sets whether the instance weights will be adjusted to maintain
 total weight per class.
   setAdvanceDataSetFirst(boolean)   - 
Method in class weka.experiment. Experiment 
 Set the value of m_AdvanceDataSetFirst.
   setAlgorithm(SelectedTag)   - 
Method in class weka.filters.supervised.attribute. PLSFilter 
 Sets the type of algorithm to use
   setAlgorithm(SelectedTag)   - 
Method in class weka.filters.unsupervised.attribute. Wavelet 
 Sets the type of algorithm to use
   setAlgorithmType(SelectedTag)   - 
Method in class weka.classifiers.mi. MILR 
 Sets the algorithm type.
   setAllowUnclassifiedInstances(boolean)   - 
Method in class weka.classifiers.trees. RandomTree 
 Set the value of AllowUnclassifiedInstances.
   setAlpha(double)   - 
Method in class weka.classifiers.bayes.net.estimate. BayesNetEstimator 
 Set prior used in probability table estimation
   setAlpha(double)   - 
Method in class weka.classifiers.functions. Winnow 
 Set the value of Alpha.
   setAmplitude(double)   - 
Method in class weka.datagenerators.classifiers.regression. MexicanHat 
 Sets the amplitude multiplier.
   setAnimated()   - 
Method in class weka.gui.beans. BeanVisual 
 Set the animated version of the icon
   setAppendPredictedProbabilities(boolean)   - 
Method in class weka.gui.beans. PredictionAppender 
 Set whether to append predicted probabilities rather than
 class value (for discrete class data sets)
   setAppropriateSize()   - 
Method in class weka.classifiers.bayes.net. GUI 
 Sets the preferred size for m_GraphPanel GraphPanel to the minimum size that is
 neccessary to display the graph.
   setArffFile(String)   - 
Method in class weka.gui.streams. InstanceLoader 
  
   setArffFile(String)   - 
Method in class weka.gui.streams. InstanceSavePanel 
  
   setArtificialSize(double)   - 
Method in class weka.classifiers.meta. Decorate 
 Sets factor that determines number of artificial examples to generate.
   setAssociatedConnections(Vector)   - 
Method in class weka.gui.beans. MetaBean 
  
   setAssociator(Associator)   - 
Method in class weka.associations. CheckAssociator 
 Set the associator to test.
   setAssociator(Associator)   - 
Method in class weka.associations. SingleAssociatorEnhancer 
 Set the base associator.
   setAssociator(Associator)   - 
Method in class weka.gui.beans. Associator 
 Set the associator for this wrapper
   setAsText(String)   - 
Method in class weka.gui. CostMatrixEditor 
 Some objects can be represented as text, but a cost matrix cannot.
   setAsText(String)   - 
Method in class weka.gui. GenericArrayEditor 
 Returns null as we don't support getting/setting values as text.
   setAsText(String)   - 
Method in class weka.gui. GenericObjectEditor 
 Returns null as we don't support getting/setting values as text.
   setAsText(String)   - 
Method in class weka.gui. SelectedTagEditor 
 Sets the current property value as text.
   setAsText(String)   - 
Method in class weka.gui. SimpleDateFormatEditor 
 Sets the date format string.
   setAttIndex(int, boolean)   - 
Method in class weka.classifiers.lazy. LBR.Indexes 
 Changes the boolean value at the specified index in the AttIndexes array
   setAttList_Irr(boolean[])   - 
Method in class weka.datagenerators.classifiers.classification. RDG1 
 Sets the array that defines which of the attributes
 are seen to be irrelevant.
   setAttribute(int)   - 
Method in class weka.gui. AttributeSummaryPanel 
 Sets the attribute that statistics will be displayed for.
   setAttribute(int)   - 
Method in class weka.gui. AttributeVisualizationPanel 
 Tells the panel which attribute to visualize.
   setAttributeEvaluator(ASEvaluation)   - 
Method in class weka.attributeSelection. FilteredAttributeEval 
 Set the attribute evaluator to use
   setAttributeEvaluator(ASEvaluation)   - 
Method in class weka.attributeSelection. RaceSearch 
 Set the attribute evaluator to use for generating the ranking.
   setAttributeEvaluator(ASEvaluation)   - 
Method in class weka.attributeSelection. RankSearch 
 Set the attribute evaluator to use for generating the ranking.
   setAttributeID(int)   - 
Method in class weka.experiment. ClassifierSplitEvaluator 
 Set the index of Attibute Identifying the instances
   setAttributeIndex(String)   - 
Method in class weka.filters.unsupervised.attribute. Add 
 Sets index of the attribute used.
   setAttributeIndex(String)   - 
Method in class weka.filters.unsupervised.attribute. AddNoise 
 Sets index of the attribute used.
   setAttributeIndex(String)   - 
Method in class weka.filters.unsupervised.attribute. AddValues 
 Sets index of the attribute used.
   setAttributeIndex(String)   - 
Method in class weka.filters.unsupervised.attribute. ChangeDateFormat 
 Sets the index of the attribute used.
   setAttributeIndex(String)   - 
Method in class weka.filters.unsupervised.attribute. MakeIndicator 
 Sets index of the attribute used.
   setAttributeIndex(String)   - 
Method in class weka.filters.unsupervised.attribute. MergeTwoValues 
 Sets index of the attribute used.
   setAttributeIndex(String)   - 
Method in class weka.filters.unsupervised.attribute. SwapValues 
 Sets index of the attribute used.
   setAttributeIndex(String)   - 
Method in class weka.filters.unsupervised.instance. RemoveFrequentValues 
 Sets index of the attribute used.
   setAttributeIndex(String)   - 
Method in class weka.filters.unsupervised.instance. RemoveWithValues 
 Sets index of the attribute used.
   setAttributeIndexes(String)   - 
Method in class weka.filters.unsupervised.attribute. NominalToString 
 Sets index of the attribute used.
   setAttributeIndices(String)   - 
Method in interface weka.core. DistanceFunction 
 Sets the range of attributes to use in the calculation of the distance.
   setAttributeIndices(String)   - 
Method in class weka.core. NormalizableDistance 
 Sets the range of attributes to use in the calculation of the distance.
   setAttributeIndices(String)   - 
Method in class weka.filters.supervised.attribute. Discretize 
 Sets which attributes are to be Discretized (only numeric
 attributes among the selection will be Discretized).
   setAttributeIndices(String)   - 
Method in class weka.filters.unsupervised.attribute. AbstractTimeSeries 
 Set which attributes are to be copied (or kept if invert is true)
   setAttributeIndices(String)   - 
Method in class weka.filters.unsupervised.attribute. Copy 
 Set which attributes are to be copied (or kept if invert is true)
   setAttributeIndices(String)   - 
Method in class weka.filters.unsupervised.attribute. Discretize 
 Sets which attributes are to be Discretized (only numeric
 attributes among the selection will be Discretized).
   setAttributeIndices(String)   - 
Method in class weka.filters.unsupervised.attribute. FirstOrder 
 Set which attributes are to be deleted (or kept if invert is true)
   setAttributeIndices(String)   - 
Method in class weka.filters.unsupervised.attribute. InterquartileRange 
 Sets which attributes are to be used for interquartile calculations and
 outlier/extreme value detection (only numeric attributes among the 
 selection will be used).
   setAttributeIndices(String)   - 
Method in class weka.filters.unsupervised.attribute. NominalToBinary 
 Sets which attributes are to be acted on.
   setAttributeIndices(String)   - 
Method in class weka.filters.unsupervised.attribute. NumericCleaner 
 Sets the columns to use, e.g., first-last or first-3,5-last
   setAttributeIndices(String)   - 
Method in class weka.filters.unsupervised.attribute. NumericToNominal 
 Sets which attributes are to be "nominalized" (only numeric
 attributes among the selection will be transformed).
   setAttributeIndices(String)   - 
Method in class weka.filters.unsupervised.attribute. NumericTransform 
 Set which attributes are to be transformed (or kept if invert is true).
   setAttributeIndices(String)   - 
Method in class weka.filters.unsupervised.attribute. Remove 
 Set which attributes are to be deleted (or kept if invert is true)
   setAttributeIndices(String)   - 
Method in class weka.filters.unsupervised.attribute. Reorder 
 Set which attributes are to be copied (or kept if invert is true)
   setAttributeIndices(String)   - 
Method in class weka.filters.unsupervised.attribute. StringToWordVector 
 Sets which attributes are to be worked on.
   setAttributeIndicesArray(int[])   - 
Method in class weka.filters.supervised.attribute. Discretize 
 Sets which attributes are to be Discretized (only numeric
 attributes among the selection will be Discretized).
   setAttributeIndicesArray(int[])   - 
Method in class weka.filters.unsupervised.attribute. AbstractTimeSeries 
 Set which attributes are to be copied (or kept if invert is true)
   setAttributeIndicesArray(int[])   - 
Method in class weka.filters.unsupervised.attribute. Copy 
 Set which attributes are to be copied (or kept if invert is true)
   setAttributeIndicesArray(int[])   - 
Method in class weka.filters.unsupervised.attribute. Discretize 
 Sets which attributes are to be Discretized (only numeric
 attributes among the selection will be Discretized).
   setAttributeIndicesArray(int[])   - 
Method in class weka.filters.unsupervised.attribute. FirstOrder 
 Set which attributes are to be deleted (or kept if invert is true)
   setAttributeIndicesArray(int[])   - 
Method in class weka.filters.unsupervised.attribute. InterquartileRange 
 Sets which attributes are to be used for interquartile calculations and
 outlier/extreme value detection (only numeric attributes among the 
 selection will be used).
   setAttributeIndicesArray(int[])   - 
Method in class weka.filters.unsupervised.attribute. NumericToNominal 
 Sets which attributes are to be transoformed to nominal.
   setAttributeIndicesArray(int[])   - 
Method in class weka.filters.unsupervised.attribute. NumericTransform 
 Set which attributes are to be transformed (or kept if invert is true)
   setAttributeIndicesArray(int[])   - 
Method in class weka.filters.unsupervised.attribute. Remove 
 Set which attributes are to be deleted (or kept if invert is true)
   setAttributeIndicesArray(int[])   - 
Method in class weka.filters.unsupervised.attribute. Reorder 
 Set which attributes are to be copied (or kept if invert is true)
   setAttributeIndicesArray(int[])   - 
Method in class weka.filters.unsupervised.attribute. StringToWordVector 
 Sets which attributes are to be processed.
   setAttributeName(String)   - 
Method in class weka.filters.unsupervised.attribute. Add 
 Set the new attribute's name.
   setAttributeName(String)   - 
Method in class weka.filters.unsupervised.attribute. AddID 
 Set the new attribute's name
   setAttributeNamePrefix(String)   - 
Method in class weka.filters.unsupervised.attribute. StringToWordVector 
 Set the attribute name prefix.
   setAttributeRange(String)   - 
Method in class weka.filters.unsupervised.attribute. StringToNominal 
 Sets range of indices of the attributes used.
   setAttributeSelectionMethod(SelectedTag)   - 
Method in class weka.classifiers.functions. LinearRegression 
 Sets the method used to select attributes for use in the
 linear regression.
   setAttributeType(SelectedTag)   - 
Method in class weka.filters.unsupervised.attribute. Add 
 Sets the type of attribute to generate.
   setAttributeType(SelectedTag)   - 
Method in class weka.filters.unsupervised.attribute. RemoveType 
 Sets the attribute type to be deleted by the filter.
   setAttributeTypes(Hashtable)   - 
Method in class weka.filters.unsupervised.instance.subsetbyexpression. Parser 
 Sets the attribute - attribute-type relation to use.
   setAttrIndexRange(String)   - 
Method in class weka.datagenerators.clusterers. SubspaceClusterDefinition 
 Sets which attributes are used in the cluster
 attributes among the selection will be discretized.
   setAtts(int[], boolean)   - 
Method in class weka.classifiers.lazy. LBR.Indexes 
 Changes the boolean value at the specified index in the InstIndexes array
   setAttsToEliminatePerIteration(int)   - 
Method in class weka.attributeSelection. SVMAttributeEval 
 Set the constant rate of attribute elimination per iteration
   setAutoBuild(boolean)   - 
Method in class weka.classifiers.functions. MultilayerPerceptron 
 This will set whether the network is automatically built
 or if it is left up to the user.
   setAutoKeyGeneration(boolean)   - 
Method in class weka.core.converters. DatabaseSaver 
 En/Dis-ables the automatic generation of a primary key.
   setBackground(Color)   - 
Method in class weka.gui.visualize. BMPWriter 
 sets the background color to use in creating the JPEG
   setBackground(Color)   - 
Method in class weka.gui.visualize. JPEGWriter 
 sets the background color to use in creating the JPEG.
   setBackground(Color)   - 
Method in class weka.gui.visualize. PNGWriter 
 sets the background color to use in creating the JPEG
   setBackground(Color)   - 
Method in class weka.gui.visualize. PostscriptGraphics 
  
   setBagSizePercent(int)   - 
Method in class weka.classifiers.meta. Bagging 
 Sets the size of each bag, as a percentage of the training set size.
   setBagSizePercent(int)   - 
Method in class weka.classifiers.meta. MetaCost 
 Sets the size of each bag, as a percentage of the training set size.
   setBalanceClass(boolean)   - 
Method in class weka.datagenerators.classifiers.classification. Agrawal 
 Sets whether the class is balanced.
   setBalanced(boolean)   - 
Method in class weka.classifiers.functions. Winnow 
 Set the value of Balanced.
   setBallSplitter(BallSplitter)   - 
Method in class weka.core.neighboursearch.balltrees. TopDownConstructor 
 Sets the ball splitting algorithm to be used by the 
 TopDown constructor.
   setBallTreeConstructor(BallTreeConstructor)   - 
Method in class weka.core.neighboursearch. BallTree 
 Sets the BallTreeConstructor for building the BallTree 
 (default TopDownConstructor).
   setBase(double)   - 
Method in class weka.core.neighboursearch. CoverTree 
 Sets the base to use for expansion constant.
   setBaseExperiment(Experiment)   - 
Method in class weka.experiment. RemoteExperiment 
 Set the base experiment.
   setBeanContext(BeanContext)   - 
Method in class weka.gui.beans. AbstractDataSource 
 Set a bean context for this bean
   setBeanContext(BeanContext)   - 
Method in class weka.gui.beans. CostBenefitAnalysis 
  
   setBeanContext(BeanContext)   - 
Method in class weka.gui.beans. DataVisualizer 
 Set a bean context for this bean
   setBeanContext(BeanContext)   - 
Method in class weka.gui.beans. GraphViewer 
 Set a bean context for this bean
   setBeanContext(BeanContext)   - 
Method in class weka.gui.beans. Loader 
 Set a bean context for this bean
   setBeanContext(BeanContext)   - 
Method in class weka.gui.beans. ModelPerformanceChart 
 Set a bean context for this bean
   setBeanContext(BeanContext)   - 
Method in class weka.gui.beans. TextViewer 
 Set a bean context for this bean
   setBeanInstances(Vector, JComponent)   - 
Static method in class weka.gui.beans. BeanInstance 
 Describe  setBeanInstances  method here.
   setBeta(double)   - 
Method in class weka.classifiers.functions. Winnow 
 Set the value of Beta.
   setBias(double)   - 
Method in class weka.classifiers.functions. LibLINEAR 
 Sets bias term value (default 1)
 No bias term is added if value < 0
   setBias(double)   - 
Method in class weka.classifiers.misc. VFI 
 Set the value of the exponential bias towards more confident intervals
   setBiasToUniformClass(double)   - 
Method in class weka.filters.supervised.instance. Resample 
 Sets the bias towards a uniform class.
   setBIFFile(String)   - 
Method in class weka.classifiers.bayes. BayesNet 
 Set name of network in BIF file to compare with
   setBIFFile(String)   - 
Method in class weka.classifiers.bayes.net.search.fixed. FromFile 
 Set name of network in BIF file to read structure from
   setBinarizeNumericAttributes(boolean)   - 
Method in class weka.attributeSelection. ChiSquaredAttributeEval 
 Binarize numeric attributes.
   setBinarizeNumericAttributes(boolean)   - 
Method in class weka.attributeSelection. InfoGainAttributeEval 
 Binarize numeric attributes.
   setBinaryAttributesNominal(boolean)   - 
Method in class weka.filters.supervised.attribute. NominalToBinary 
 Sets if binary attributes are to be treates as nominal ones.
   setBinaryAttributesNominal(boolean)   - 
Method in class weka.filters.unsupervised.attribute. NominalToBinary 
 Sets if binary attributes are to be treates as nominal ones.
   setBinarySplits(boolean)   - 
Method in class weka.classifiers.rules. PART 
 Set the value of binarySplits.
   setBinarySplits(boolean)   - 
Method in class weka.classifiers.trees. J48 
 Set the value of binarySplits.
   setBinarySplits(boolean)   - 
Method in class weka.classifiers.trees. J48graft 
 Set the value of binarySplits.
   setBins(int)   - 
Method in class weka.filters.unsupervised.attribute. Discretize 
 Sets the number of bins to divide each selected numeric attribute into
   setBins(int)   - 
Method in class weka.filters.unsupervised.attribute. PKIDiscretize 
 Ignored
   setBinSplit(boolean)   - 
Method in class weka.classifiers.trees. FT 
 Set the value of binarySplits.
   setBinValue(double)   - 
Method in class weka.clusterers. XMeans 
 Sets the distance value between true and false of binary attributes.
   setBlendFactor(int)   - 
Method in class weka.classifiers.lazy.kstar. KStarNumericAttribute 
 Set the blending factor
   setBlendMethod(int)   - 
Method in class weka.classifiers.lazy.kstar. KStarNumericAttribute 
 Set the blending method
   setBooleanCols(Range)   - 
Method in class weka.datagenerators. ClusterGenerator 
 Sets which attributes are boolean.
   setBooleanIndices(String)   - 
Method in class weka.datagenerators. ClusterGenerator 
 Sets which attributes are boolean
   setBuildLogisticModels(boolean)   - 
Method in class weka.classifiers.functions. SMO 
 Set the value of buildLogisticModels.
   setBuildLogisticModels(boolean)   - 
Method in class weka.classifiers.mi. MISMO 
 Set the value of buildLogisticModels.
   setBuildRegressionTree(boolean)   - 
Method in class weka.classifiers.trees.m5. M5Base 
 Set the value of regressionTree.
   setC(double)   - 
Method in class weka.classifiers.functions. SMO 
 Set the value of C.
   setC(double)   - 
Method in class weka.classifiers.functions. SMOreg 
 Set the value of C.
   setC(double)   - 
Method in class weka.classifiers.mi. MISMO 
 Set the value of C.
   setC(double)   - 
Method in class weka.classifiers.mi. MISVM 
 Set the value of C.
   setCacheKeyName(String)   - 
Method in class weka.experiment. DatabaseResultListener 
 Set the value of CacheKeyName.
   setCacheSize(double)   - 
Method in class weka.classifiers.functions. LibSVM 
 Sets cache memory size in MB (default 40)
   setCacheSize(int)   - 
Method in class weka.classifiers.functions.supportVector. CachedKernel 
 Sets the size of the cache to use (a prime number)
   setCacheSize(int)   - 
Method in class weka.classifiers.functions.supportVector. StringKernel 
 Sets the size of the cache to use (a prime number)
   setCalcOutOfBag(boolean)   - 
Method in class weka.classifiers.meta. Bagging 
 Set whether the out of bag error is calculated.
   setCalculateStdDevs(boolean)   - 
Method in class weka.experiment. AveragingResultProducer 
 Set the value of CalculateStdDevs.
   setCanChangeClassInDialog(boolean)   - 
Method in class weka.gui. GenericObjectEditor 
 Sets whether the user can change the class in the dialog.
   setCapabilities(Capabilities)   - 
Method in class weka.core. FindWithCapabilities 
 Uses the given Capabilities for the search.
   setCapabilities(Capabilities)   - 
Method in class weka.gui. GenericObjectEditor.CapabilitiesFilterDialog 
 sets the initial capabilities.
   setCapabilitiesFilter(Capabilities)   - 
Method in class weka.gui. ConverterFileChooser 
 sets the capabilities that the savers must have.
   setCapabilitiesFilter(Capabilities)   - 
Method in class weka.gui. GenericObjectEditor 
 Sets the capabilities to use for filtering.
   setCapacity(int)   - 
Method in class weka.core. FastVector 
 Sets the vector's capacity to the given value.
   setCapacity(int)   - 
Method in class weka.core.matrix. DoubleVector 
 Sets the capacity of the vector
   setCapacity(int)   - 
Method in class weka.core.matrix. IntVector 
 Sets the capacity of the vector
   setCar(boolean)   - 
Method in class weka.associations. Apriori 
 Sets class association rule mining
   setCar(boolean)   - 
Method in class weka.associations. PredictiveApriori 
 Sets class association rule mining
   setCardinality(int)   - 
Method in class weka.datagenerators.classifiers.classification. BayesNet 
 Sets the cardinality of the attributes (incl class attribute)
   setCell(int, int, Object)   - 
Method in class weka.classifiers. CostMatrix 
 Set the value of a particular cell in the matrix
   setCenter(double)   - 
Method in class weka.gui.treevisualizer. Node 
 Set the value of center.
   setCenterData(boolean)   - 
Method in class weka.attributeSelection. PrincipalComponents 
 Set whether to center (rather than standardize)
 the data.
   setCenterData(boolean)   - 
Method in class weka.filters.unsupervised.attribute. PrincipalComponents 
 Set whether to center (rather than standardize)
 the data.
   setCenteredLocation()   - 
Method in class weka.gui.arffviewer. ArffViewer 
 positions the window at the center of the screen
   setChanged(boolean)   - 
Method in class weka.gui.arffviewer. ArffPanel 
 can only reset the changed state to FALSE
   setChar(Character)   - 
Method in class weka.core. Trie.TrieNode 
 sets the character this node represents
   setCharSet(String)   - 
Method in class weka.core.converters. TextDirectoryLoader 
 Set the character set to use when reading text files (an empty string
 indicates that the default character set will be used).
   setChecked(int, boolean)   - 
Method in class weka.gui. CheckBoxList.CheckBoxListModel 
 sets the checked state of the element at the given index
   setChecked(int, boolean)   - 
Method in class weka.gui. CheckBoxList 
 sets the checked state of the element at the given index
   setCheckErrorRate(boolean)   - 
Method in class weka.classifiers.rules. JRip 
 Sets whether to check for error rate is in stopping criterion
   setChecksTurnedOff(boolean)   - 
Method in class weka.classifiers.functions. SMO 
 Disables or enables the checks (which could be time-consuming).
   setChecksTurnedOff(boolean)   - 
Method in class weka.classifiers.functions.supportVector. Kernel 
 Disables or enables the checks (which could be time-consuming).
   setChecksTurnedOff(boolean)   - 
Method in class weka.classifiers.mi. MISMO 
 Disables or enables the checks (which could be time-consuming).
   setChecksTurnedOff(boolean)   - 
Method in class weka.filters.unsupervised.attribute. KernelFilter 
 Disables or enables the checks (which could be time-consuming).
   setChildForBranch(int, PredictionNode)   - 
Method in class weka.classifiers.trees.adtree. Splitter 
 Sets the child for a branch of the split.
   setChildForBranch(int, PredictionNode)   - 
Method in class weka.classifiers.trees.adtree. TwoWayNominalSplit 
 Sets the child for a branch of the split.
   setChildForBranch(int, PredictionNode)   - 
Method in class weka.classifiers.trees.adtree. TwoWayNumericSplit 
 Sets the child for a branch of the split.
   setCindex(int, double, double)   - 
Method in class weka.gui.visualize. AttributePanel 
 Set the index of the attribute by which to colour the data.
   setCindex(int)   - 
Method in class weka.gui.visualize. AttributePanel 
 Set the index of the attribute by which to colour the data.
   setCindex(int)   - 
Method in class weka.gui.visualize. ClassPanel 
 Set the index of the attribute to display coloured labels for
   setCindex(int)   - 
Method in class weka.gui.visualize. Plot2D 
 Set the index of the attribute to use for colouring
   setCindex(int)   - 
Method in class weka.gui.visualize. PlotData2D 
 Set the colouring index of the data
   setClass(Attribute)   - 
Method in class weka.core. Instances 
 Sets the class attribute.
   setClassColumn(String)   - 
Method in class weka.gui.beans. ClassAssigner 
  
   setClassFlag(boolean)   - 
Method in class weka.datagenerators. ClusterGenerator 
 Sets the class flag, if class flag is set, 
 the cluster is listed as class atrribute in an extra attribute.
   setClassForIRStatistics(int)   - 
Method in class weka.experiment. ClassifierSplitEvaluator 
 Set the value of ClassForIRStatistics.
   setClassification(boolean)   - 
Method in class weka.associations. Tertius 
 Set the value of classification.
   setClassifier(Classifier)   - 
Method in class weka.attributeSelection. ClassifierSubsetEval 
 Set the classifier to use for accuracy estimation
   setClassifier(Classifier)   - 
Method in class weka.attributeSelection. WrapperSubsetEval 
 Set the classifier to use for accuracy estimation
   setClassifier(Classifier)   - 
Method in class weka.classifiers. BVDecompose 
 Set the classifiers being analysed
   setClassifier(Classifier)   - 
Method in class weka.classifiers. BVDecomposeSegCVSub 
 Set the classifiers being analysed
   setClassifier(Classifier)   - 
Method in class weka.classifiers. CheckClassifier 
 Set the classifier for boosting.
   setClassifier(Classifier)   - 
Method in class weka.classifiers. CheckSource 
 Sets the classifier to use for the comparison.
   setClassifier(Classifier)   - 
Method in class weka.classifiers.meta. GridSearch 
 Set the base learner.
   setClassifier(Classifier)   - 
Method in class weka.classifiers.meta. RacedIncrementalLogitBoost 
 Set the base learner.
   setClassifier(Classifier)   - 
Method in class weka.classifiers. SingleClassifierEnhancer 
 Set the base learner.
   setClassifier(Classifier)   - 
Method in class weka.experiment. ClassifierSplitEvaluator 
 Sets the classifier.
   setClassifier(Classifier)   - 
Method in class weka.experiment. RegressionSplitEvaluator 
 Sets the classifier.
   setClassifier(Classifier)   - 
Method in class weka.filters.supervised.attribute. AddClassification 
 Sets the classifier to classify instances with.
   setClassifier(Classifier)   - 
Method in class weka.filters.unsupervised.instance. RemoveMisclassified 
 Sets the classifier to classify instances with.
   setClassifier(Classifier)   - 
Method in class weka.gui.beans. BatchClassifierEvent 
 Set the classifier
   setClassifier(Classifier)   - 
Method in class weka.gui.beans. IncrementalClassifierEvent 
  
   setClassifier(Classifier)   - 
Method in class weka.gui.boundaryvisualizer. BoundaryPanel 
 Set the classifier to use.
   setClassifier(Classifier)   - 
Method in class weka.gui.boundaryvisualizer. BoundaryVisualizer 
 Set a classifier to use
   setClassifier(Classifier)   - 
Method in class weka.gui.boundaryvisualizer. RemoteBoundaryVisualizerSubTask 
 Set the classifier to use
   setClassifierName(String)   - 
Method in class weka.experiment. ClassifierSplitEvaluator 
 Set the Classifier to use, given it's class name.
   setClassifierName(String)   - 
Method in class weka.experiment. RegressionSplitEvaluator 
 Set the Classifier to use, given it's class name.
   setClassifiers(Classifier[])   - 
Method in class weka.classifiers.meta. MultiScheme 
 Sets the list of possible classifers to choose from.
   setClassifiers(Classifier[])   - 
Method in class weka.classifiers. MultipleClassifiersCombiner 
 Sets the list of possible classifers to choose from.
   setClassifierTemplate(Classifier)   - 
Method in class weka.gui.beans. Classifier 
 Set the classifier for this wrapper
   setClassifyIterations(int)   - 
Method in class weka.classifiers. BVDecomposeSegCVSub 
 Sets the number of times an instance is classified
   setClassIndex(int)   - 
Method in class weka.associations. Apriori 
 Sets the class index
   setClassIndex(int)   - 
Method in interface weka.associations. CARuleMiner 
 Sets the class index for the class association rule miner
   setClassIndex(int)   - 
Method in class weka.associations. FilteredAssociator 
 Sets the class index
   setClassIndex(int)   - 
Method in class weka.associations. PredictiveApriori 
 Sets the class index
   setClassIndex(int)   - 
Method in class weka.associations. Tertius 
 Set the value of classIndex.
   setClassIndex(int)   - 
Method in class weka.classifiers. BVDecompose 
 Sets index of attribute to discretize on
   setClassIndex(int)   - 
Method in class weka.classifiers. BVDecomposeSegCVSub 
 Sets index of attribute to discretize on
   setClassIndex(int)   - 
Method in class weka.classifiers. CheckSource 
 Sets the class index of the dataset.
   setClassIndex(String)   - 
Method in class weka.core.converters. LibSVMSaver 
 Sets index of the class attribute.
   setClassIndex(String)   - 
Method in class weka.core.converters. SVMLightSaver 
 Sets index of the class attribute.
   setClassIndex(String)   - 
Method in class weka.core.converters. XRFFSaver 
 Sets index of the class attribute.
   setClassIndex(String)   - 
Method in class weka.core. FindWithCapabilities 
 sets the class index, -1 for none, first and last are also valid.
   setClassIndex(int)   - 
Method in class weka.core. Instances 
 Sets the class index of the set.
   setClassIndex(int)   - 
Method in class weka.core. TestInstances 
 sets the class index (0-based)
   setClassIndex(int)   - 
Method in class weka.filters. CheckSource 
 Sets the class index of the dataset.
   setClassIndex(String)   - 
Method in class weka.filters.unsupervised.attribute. ClassAssigner 
 sets the class index.
   setClassIndex(int)   - 
Method in class weka.filters.unsupervised.instance. RemoveMisclassified 
 Sets the attribute on which misclassifications are based.
   setClassMissing()   - 
Method in class weka.core. Instance 
 Sets the class value of an instance to be "missing".
   setClassname(String)   - 
Method in class weka.core. AllJavadoc 
 sets the classname of the class to generate the Javadoc for
   setClassname(String)   - 
Method in class weka.core. Javadoc 
 sets the classname of the class to generate the Javadoc for
   setClassname(String)   - 
Method in class weka.core. ListOptions 
 sets the classname of the class to generate the Javadoc for
   setClassName(String)   - 
Method in class weka.filters.unsupervised.attribute. NumericTransform 
 Sets the class containing the transformation method.
   setClassOrder(int)   - 
Method in class weka.filters.supervised.attribute. ClassOrder 
 Set the wanted class order
   setClassType(int)   - 
Method in class weka.core. TestInstances 
 sets the class attribute type
   setClassType(Class)   - 
Method in class weka.gui. GenericObjectEditor 
 Sets the class of values that can be edited.
   setClassValue(double)   - 
Method in class weka.core. Instance 
 Sets the class value of an instance to the given value (internal
 floating-point format).
   setClassValue(String)   - 
Method in class weka.core. Instance 
 Sets the class value of an instance to the given value.
   setClassValue(String)   - 
Method in class weka.filters.supervised.instance. SMOTE 
 Sets the index of the class value to which SMOTE should be applied.
   setClassValue(String)   - 
Method in class weka.gui.beans. ClassValuePicker 
 Set the class value index considered to be the "positive"
 class value.
   setClearEachDataset(boolean)   - 
Method in class weka.gui.streams. InstanceViewer 
  
   setClip(int, int, int, int)   - 
Method in class weka.gui.visualize. PostscriptGraphics 
 Not implemented
   setClip(Shape)   - 
Method in class weka.gui.visualize. PostscriptGraphics 
 Not implemented
   setCloseTo(double)   - 
Method in class weka.filters.unsupervised.attribute. NumericCleaner 
 Set the "close to" number.
   setCloseToDefault(double)   - 
Method in class weka.filters.unsupervised.attribute. NumericCleaner 
 Set the "close to" default.
   setCloseToTolerance(double)   - 
Method in class weka.filters.unsupervised.attribute. NumericCleaner 
 Set the "close to" Tolerance.
   setClusterDefinitions(ClusterDefinition[])   - 
Method in class weka.datagenerators.clusterers. SubspaceCluster 
 sets the clusters to use
   setClusterer(Clusterer)   - 
Method in class weka.attributeSelection. UnsupervisedSubsetEvaluator 
 Set the clusterer to use
   setClusterer(Clusterer)   - 
Method in class weka.classifiers.meta. ClassificationViaClustering 
 Set the base clusterer.
   setClusterer(Clusterer)   - 
Method in class weka.clusterers. CheckClusterer 
 Set the clusterer for testing.
   setClusterer(Clusterer)   - 
Method in class weka.clusterers. ClusterEvaluation 
 set the clusterer
   setClusterer(Clusterer)   - 
Method in class weka.clusterers. MakeDensityBasedClusterer 
 Sets the clusterer to wrap.
   setClusterer(Clusterer)   - 
Method in class weka.clusterers. SingleClustererEnhancer 
 Set the base clusterer.
   setClusterer(DensityBasedClusterer)   - 
Method in class weka.experiment. DensityBasedClustererSplitEvaluator 
 Sets the clusterer.
   setClusterer(Clusterer)   - 
Method in class weka.filters.unsupervised.attribute. AddCluster 
 Sets the clusterer to assign clusters with.
   setClusterer(Clusterer)   - 
Method in class weka.gui.beans. Clusterer 
 Set the clusterer for this wrapper
   setClustererName(String)   - 
Method in class weka.experiment. DensityBasedClustererSplitEvaluator 
 Set the Clusterer to use, given it's class name.
   setClusteringSeed(int)   - 
Method in class weka.classifiers.functions. RBFNetwork 
 Set the random seed to be passed on to K-means.
   setClusterLabel(int)   - 
Method in interface weka.clusterers.forOPTICSAndDBScan.DataObjects. DataObject 
 Sets the clusterID (cluster), to which this DataObject belongs to
   setClusterLabel(int)   - 
Method in class weka.clusterers.forOPTICSAndDBScan.DataObjects. EuclidianDataObject 
 Sets the clusterID (cluster), to which this DataObject belongs to
   setClusterLabel(int)   - 
Method in class weka.clusterers.forOPTICSAndDBScan.DataObjects. ManhattanDataObject 
 Sets the clusterID (cluster), to which this DataObject belongs to
   setClusterSubType(SelectedTag)   - 
Method in class weka.datagenerators.clusterers. SubspaceClusterDefinition 
 Sets the cluster sub type.
   setClusterType(SelectedTag)   - 
Method in class weka.datagenerators.clusterers. SubspaceClusterDefinition 
 Sets the cluster type.
   setCoef0(double)   - 
Method in class weka.classifiers.functions. LibSVM 
 Sets coef (default 0)
   setColHidden(int, boolean)   - 
Method in class weka.experiment. ResultMatrix 
 sets the hidden status of the column (if the index is valid)
   setColName(int, String)   - 
Method in class weka.experiment. ResultMatrix 
 sets the name of the column (if the index is valid)
   setColNameWidth(int)   - 
Method in class weka.experiment. ResultMatrix 
 sets the width for the column names (0 = optimal)
   setColor(Color)   - 
Method in class weka.gui.treevisualizer. Node 
 Set the value of color.
   setColor(Color)   - 
Method in class weka.gui.visualize. PostscriptGraphics 
 Set current pen color.
   setColOrder(int[])   - 
Method in class weka.experiment. ResultMatrix 
 sets the ordering of the columns, null means default
   setColoringIndex(int)   - 
Method in class weka.gui. AttributeVisualizationPanel 
 Set the coloring (class) index for the plot
   setColoringIndex(int)   - 
Method in class weka.gui.beans. AttributeSummarizer 
 Set the coloring index for the attribute summary plots
   setColors(FastVector)   - 
Method in class weka.gui.boundaryvisualizer. BoundaryPanel 
 Set a vector of Color objects for the classes
   setColourIndex(int)   - 
Method in class weka.gui.visualize. VisualizePanel 
 Sets the index used for colouring.
   setColours(FastVector)   - 
Method in class weka.gui.visualize. AttributePanel 
 Sets a list of colours to use for colouring data points
   setColours(FastVector)   - 
Method in class weka.gui.visualize. ClassPanel 
 Set a list of colours to use for colouring labels
   setColours(FastVector)   - 
Method in class weka.gui.visualize. Plot2D 
 Set a list of colours to use when colouring points according
 to class values or cluster numbers
   setColumn(int, double[])   - 
Method in class weka.core. Matrix 
  Deprecated.  Sets a column of the matrix to the given column.
   setColumnDimension(int)   - 
Method in class weka.classifiers.functions.pace. PaceMatrix 
 Set the column dimenion of the matrix
   setCombination(SelectedTag)   - 
Method in class weka.attributeSelection. ScatterSearchV1 
 Set the kind of combination
   setCombinationRule(SelectedTag)   - 
Method in class weka.classifiers.meta. Vote 
 Sets the combination rule to use.
   setComplexityParameter(double)   - 
Method in class weka.attributeSelection. SVMAttributeEval 
 Set the value of C for SMO
   setComponent(JComponent)   - 
Method in class weka.gui.visualize. JComponentWriter 
 sets the component to print to an output format
   setComposite(Composite)   - 
Method in class weka.gui.visualize. PostscriptGraphics 
  
   setCompressOutput(boolean)   - 
Method in class weka.core.converters. ArffSaver 
 Sets whether to compress the output.
   setCompressOutput(boolean)   - 
Method in class weka.core.converters. XRFFSaver 
 Sets whether to compress the output.
   setConfidenceFactor(float)   - 
Method in class weka.classifiers.rules. PART 
 Set the value of CF.
   setConfidenceFactor(float)   - 
Method in class weka.classifiers.trees. J48 
 Set the value of CF.
   setConfidenceFactor(float)   - 
Method in class weka.classifiers.trees. J48graft 
 Set the value of CF.
   setConfirmationThreshold(double)   - 
Method in class weka.associations. Tertius 
 Set the value of confirmationThreshold.
   setConfirmationValues(int)   - 
Method in class weka.associations. Tertius 
 Set the value of confirmationValues.
   setConfirmExit(boolean)   - 
Method in class weka.gui.arffviewer. ArffViewer 
 whether to present a MessageBox on Exit or not
   setConfirmExit(boolean)   - 
Method in class weka.gui.arffviewer. ArffViewerMainPanel 
 whether to present a MessageBox on Exit or not
   setConnections(Vector)   - 
Static method in class weka.gui.beans. BeanConnection 
 Describe  setConnections  method here.
   setConnectPoints(boolean[])   - 
Method in class weka.gui.visualize. PlotData2D 
 Set whether consecutive points should be connected by lines
   setConnectPoints(FastVector)   - 
Method in class weka.gui.visualize. PlotData2D 
 Set whether consecutive points should be connected by lines
   setConservativeForwardSelection(boolean)   - 
Method in class weka.attributeSelection. GreedyStepwise 
 Set whether attributes should continue to be added during
 a forward search as long as merit does not decrease
   setContainChildBalls(boolean)   - 
Method in class weka.core.neighboursearch.balltrees. BallTreeConstructor 
 Sets whether if a parent ball should completely enclose
 its two child balls.
   setConvertNominal(boolean)   - 
Method in class weka.classifiers.trees. LMT 
 Set the value of convertNominal.
   setConvertNominalToBinary(boolean)   - 
Method in class weka.classifiers.functions. LibLINEAR 
 Whether to turn on conversion of nominal attributes
 to binary.
   setCoreConvertersOnly(boolean)   - 
Method in class weka.gui. ConverterFileChooser 
 Whether to display only the hardocded core converters.
   setCoreDistance(double)   - 
Method in interface weka.clusterers.forOPTICSAndDBScan.DataObjects. DataObject 
 Sets a new coreDistance for this dataObject
   setCoreDistance(double)   - 
Method in class weka.clusterers.forOPTICSAndDBScan.DataObjects. EuclidianDataObject 
 Sets a new coreDistance for this dataObject
   setCoreDistance(double)   - 
Method in class weka.clusterers.forOPTICSAndDBScan.DataObjects. ManhattanDataObject 
 Sets a new coreDistance for this dataObject
   setCoreDistanceColor(Color)   - 
Method in class weka.clusterers.forOPTICSAndDBScan.OPTICS_GUI. GraphPanel 
 Sets a new color for the coreDistance
   setCost(double)   - 
Method in class weka.classifiers.functions. LibLINEAR 
 Sets the cost parameter C (default 1)
   setCost(double)   - 
Method in class weka.classifiers.functions. LibSVM 
 Sets the parameter C of C-SVC, epsilon-SVR, and nu-SVR (default 1)
   setCostMatrix(CostMatrix)   - 
Method in class weka.attributeSelection. CostSensitiveASEvaluation 
 Sets the misclassification cost matrix.
   setCostMatrix(CostMatrix)   - 
Method in class weka.classifiers.meta. CostSensitiveClassifier 
 Sets the misclassification cost matrix.
   setCostMatrix(CostMatrix)   - 
Method in class weka.classifiers.meta. MetaCost 
 Sets the misclassification cost matrix.
   setCostMatrixSource(SelectedTag)   - 
Method in class weka.attributeSelection. CostSensitiveASEvaluation 
 Sets the source location of the cost matrix.
   setCostMatrixSource(SelectedTag)   - 
Method in class weka.classifiers.meta. CostSensitiveClassifier 
 Sets the source location of the cost matrix.
   setCostMatrixSource(SelectedTag)   - 
Method in class weka.classifiers.meta. MetaCost 
 Sets the source location of the cost matrix.
   setCount(int, double)   - 
Method in class weka.experiment. ResultMatrix 
 sets the count for the row (if the index is valid)
   setCounter(int)   - 
Method in class weka.associations. ItemSet 
 Sets the counter
   setCountWidth(int)   - 
Method in class weka.experiment. ResultMatrix 
 sets the width for the counts (0 = optimal)
   setCreatorApplication(Document)   - 
Method in class weka.classifiers.pmml.consumer. PMMLClassifier 
 Set the name of the application (if specified) that created this
 model
   setCreatorApplication(Document)   - 
Method in interface weka.core.pmml. PMMLModel 
 Set the name of the application (if specified) that created this.
   setCriticalValue(int)   - 
Method in class weka.classifiers.bayes. AODEsr 
 Sets the critical value
   setCrossoverProb(double)   - 
Method in class weka.attributeSelection. GeneticSearch 
 set the probability of crossover
   setCrossVal(int)   - 
Method in class weka.classifiers.rules. DecisionTable 
 Sets the number of folds for cross validation (1 = leave one out)
   setCrossValidate(boolean)   - 
Method in class weka.classifiers.lazy. IBk 
 Sets whether hold-one-out cross-validation will be used
 to select the best k value.
   setCurrentFilename(String)   - 
Method in class weka.gui.arffviewer. ArffViewerMainPanel 
 sets the filename of the current tab
   setCurrentInstance(Instance)   - 
Method in class weka.gui.beans. IncrementalClassifierEvent 
 Set the current instance for this event
   setCurveData(PlotData2D, Attribute)   - 
Method in class weka.gui.beans. CostBenefitAnalysis 
 Set the threshold curve data to use.
   setCustomColour(Color)   - 
Method in class weka.gui.visualize. PlotData2D 
 Set a custom colour to use for this plot.
   setCustomHeight(int)   - 
Method in class weka.gui.visualize. JComponentWriter 
 sets the custom height to use
   setCustomName(String)   - 
Method in class weka.gui.beans. Associator 
 Set a custom (descriptive) name for this bean
   setCustomName(String)   - 
Method in interface weka.gui.beans. BeanCommon 
 Set a custom (descriptive) name for this bean
   setCustomName(String)   - 
Method in class weka.gui.beans. ClassAssigner 
 Set a custom (descriptive) name for this bean
   setCustomName(String)   - 
Method in class weka.gui.beans. Classifier 
 Set a custom (descriptive) name for this bean
   setCustomName(String)   - 
Method in class weka.gui.beans. ClassifierPerformanceEvaluator 
 Set a custom (descriptive) name for this bean
   setCustomName(String)   - 
Method in class weka.gui.beans. ClassValuePicker 
 Set a custom (descriptive) name for this bean
   setCustomName(String)   - 
Method in class weka.gui.beans. Clusterer 
 Set a custom (descriptive) name for this bean
   setCustomName(String)   - 
Method in class weka.gui.beans. ClustererPerformanceEvaluator 
 Set a custom (descriptive) name for this bean
   setCustomName(String)   - 
Method in class weka.gui.beans. CostBenefitAnalysis 
 Set a custom (descriptive) name for this bean
   setCustomName(String)   - 
Method in class weka.gui.beans. CrossValidationFoldMaker 
 Set a custom (descriptive) name for this bean
   setCustomName(String)   - 
Method in class weka.gui.beans. Filter 
 Set a custom (descriptive) name for this bean
   setCustomName(String)   - 
Method in class weka.gui.beans. IncrementalClassifierEvaluator 
 Set a custom (descriptive) name for this bean
   setCustomName(String)   - 
Method in class weka.gui.beans. InstanceStreamToBatchMaker 
 Set a custom (descriptive) name for this bean
   setCustomName(String)   - 
Method in class weka.gui.beans. Loader 
 Set a custom (descriptive) name for this bean
   setCustomName(String)   - 
Method in class weka.gui.beans. MetaBean 
 Set a custom (descriptive) name for this bean
   setCustomName(String)   - 
Method in class weka.gui.beans. PredictionAppender 
 Set a custom (descriptive) name for this bean
   setCustomName(String)   - 
Method in class weka.gui.beans. Saver 
 Set a custom (descriptive) name for this bean
   setCustomName(String)   - 
Method in class weka.gui.beans. SerializedModelSaver 
 Set a custom (descriptive) name for this bean
   setCustomName(String)   - 
Method in class weka.gui.beans. StripChart 
 Set a custom (descriptive) name for this bean
   setCustomName(String)   - 
Method in class weka.gui.beans. TestSetMaker 
 Set a custom (descriptive) name for this bean
   setCustomName(String)   - 
Method in class weka.gui.beans. TextViewer 
 Set a custom (descriptive) name for this bean
   setCustomName(String)   - 
Method in class weka.gui.beans. TrainingSetMaker 
 Set a custom (descriptive) name for this bean
   setCustomName(String)   - 
Method in class weka.gui.beans. TrainTestSplitMaker 
 Set a custom (descriptive) name for this bean
   setCustomWidth(int)   - 
Method in class weka.gui.visualize. JComponentWriter 
 sets the custom width to use
   setCutoff(double)   - 
Method in class weka.clusterers. Cobweb 
 set the cutoff
   setCutOffFactor(double)   - 
Method in class weka.clusterers. XMeans 
 Sets a new cutoff factor.
   setCVisible(boolean)   - 
Method in class weka.gui.treevisualizer. Node 
 Sets all the children of this node either to visible or invisible
   setCVParameters(Object[])   - 
Method in class weka.classifiers.meta. CVParameterSelection 
 Set method for CVParameters.
   setCVType(SelectedTag)   - 
Method in class weka.classifiers.bayes.net.search.global. GlobalScoreSearchAlgorithm 
 set cross validation strategy to be used in searching for networks.
   setData(Instances)   - 
Method in class weka.classifiers.bayes.net. EditableBayesNet 
 Assuming a network structure is defined and we want to learn from data,
 the data set must be put if correct order first and possibly discretized/missing
 values filled in before proceeding to CPT learning.
   setData(Instances)   - 
Method in class weka.classifiers.rules. RuleStats 
 Set the data of the stats, overwriting the old one if any
   setDatabase_distanceType(String)   - 
Method in class weka.clusterers. DBScan 
 Sets a new distance-type
   setDatabase_distanceType(String)   - 
Method in class weka.clusterers. OPTICS 
 Sets a new distance-type
   setDatabase_Type(String)   - 
Method in class weka.clusterers. DBScan 
 Sets a new database-type
   setDatabase_Type(String)   - 
Method in class weka.clusterers. OPTICS 
 Sets a new database-type
   setDatabaseOutput(File)   - 
Method in class weka.clusterers. OPTICS 
 Sets the the file to save the generated database to.
   setDatabaseURL(String)   - 
Method in class weka.experiment. DatabaseUtils 
 Set the value of DatabaseURL.
   setDataFileName(String)   - 
Method in class weka.classifiers. BVDecompose 
 Sets the name of the data file used for the decomposition
   setDataFileName(String)   - 
Method in class weka.classifiers. BVDecomposeSegCVSub 
 Sets the name of the dataset file.
   setDataGenerator(DataGenerator)   - 
Method in class weka.gui.boundaryvisualizer. BoundaryPanel 
 Set the data generator to use for generating new instances
   setDataGenerator(DataGenerator)   - 
Method in class weka.gui.boundaryvisualizer. RemoteBoundaryVisualizerSubTask 
 Set the density estimator to use
   setDataPoint(double[])   - 
Method in class weka.gui.beans. ChartEvent 
 Set the data point
   setDataSeqID(int)   - 
Method in class weka.associations. GeneralizedSequentialPatterns 
 Sets the attribute representing the data sequence ID.
   setDataset(File)   - 
Method in class weka.classifiers. CheckSource 
 Sets the dataset to use for testing.
   setDataset(Instances)   - 
Method in class weka.core. Instance 
 Sets the reference to the dataset.
   setDataset(File)   - 
Method in class weka.filters. CheckSource 
 Sets the dataset to use for testing.
   setDatasetFormat(Instances)   - 
Method in class weka.datagenerators. DataGenerator 
 Sets the format of the dataset that is to be generated.
   setDatasetKeyColumns(Range)   - 
Method in class weka.experiment. PairedTTester 
 Set the value of DatasetKeyColumns.
   setDatasetKeyColumns(Range)   - 
Method in interface weka.experiment. Tester 
 Set the value of DatasetKeyColumns.
   setDatasetKeyFromDialog()   - 
Method in class weka.gui.experiment. ResultsPanel 
  
   setDatasets(DefaultListModel)   - 
Method in class weka.experiment. Experiment 
 Set the datasets to use in the experiment
   setDatasets(DefaultListModel)   - 
Method in class weka.experiment. RemoteExperiment 
 Set the datasets to use in the experiment
   setDataType(int)   - 
Method in class weka.gui.beans.xml. XMLBeans 
 sets what kind of data is to be read/written
   setDateAttributes(String)   - 
Method in class weka.core.converters. CSVLoader 
 Set the attribute range to be forced to type date.
   setDateFormat(String)   - 
Method in class weka.core.converters. CSVLoader 
 Set the format to use for parsing date values.
   setDateFormat(String)   - 
Method in class weka.filters.unsupervised.attribute. Add 
 Set the date format, complying to ISO-8601.
   setDateFormat(String)   - 
Method in class weka.filters.unsupervised.attribute. ChangeDateFormat 
 Sets the output date format.
   setDateFormat(SimpleDateFormat)   - 
Method in class weka.filters.unsupervised.attribute. ChangeDateFormat 
 Sets the output date format.
   setDB(boolean)   - 
Method in class weka.gui.beans. Loader 
  
   setDebug(boolean)   - 
Method in class weka.associations. GeneralizedSequentialPatterns 
 Set debugging mode.
   setDebug(boolean)   - 
Method in class weka.attributeSelection. RaceSearch 
 Set whether verbose output should be generated.
   setDebug(boolean)   - 
Method in class weka.attributeSelection. ScatterSearchV1 
 Set whether verbose output should be generated.
   setDebug(boolean)   - 
Method in class weka.classifiers.bayes. BayesianLogisticRegression 
  
   setDebug(boolean)   - 
Method in class weka.classifiers. BVDecompose 
 Sets debugging mode
   setDebug(boolean)   - 
Method in class weka.classifiers. BVDecomposeSegCVSub 
 Sets debugging mode
   setDebug(boolean)   - 
Method in class weka.classifiers. Classifier 
 Set debugging mode.
   setDebug(boolean)   - 
Method in class weka.classifiers.functions. LeastMedSq 
 sets  whether or not debugging output shouild be printed
   setDebug(boolean)   - 
Method in class weka.classifiers.functions. LinearRegression 
 Controls whether debugging output will be printed
   setDebug(boolean)   - 
Method in class weka.classifiers.functions. Logistic 
 Sets whether debugging output will be printed.
   setDebug(boolean)   - 
Method in class weka.classifiers.functions. PaceRegression 
 Controls whether debugging output will be printed
   setDebug(boolean)   - 
Method in class weka.classifiers.functions.supportVector. Kernel 
 Enables or disables the output of debug information (if the derived
 kernel supports that)
   setDebug(boolean)   - 
Method in class weka.classifiers.meta. MultiScheme 
 Set debugging mode
   setDebug(boolean)   - 
Method in class weka.classifiers.rules. JRip 
 Sets whether debug information is output to the console
   setDebug(boolean)   - 
Method in class weka.clusterers. EM 
 Set debug mode - verbose output
   setDebug(boolean)   - 
Method in class weka.clusterers. HierarchicalClusterer 
 Set debugging mode.
   setDebug(boolean)   - 
Method in class weka.clusterers. sIB 
 Set debug mode - verbose output
   setDebug(boolean)   - 
Method in class weka.core. Check 
 Set debugging mode
   setDebug(boolean)   - 
Method in class weka.core.converters. TextDirectoryLoader 
 Sets whether to print some debug information.
   setDebug(boolean)   - 
Method in class weka.core. Debug.Random 
 sets whether to print the generated random values or not
   setDebug(boolean)   - 
Method in class weka.core. Optimization 
 Set whether in debug mode
   setDebug(boolean)   - 
Method in class weka.datagenerators. DataGenerator 
 Sets the debug flag.
   setDebug(boolean)   - 
Method in class weka.estimators. CheckEstimator 
 Set debugging mode
   setDebug(boolean)   - 
Method in class weka.estimators. Estimator 
 Set debugging mode.
   setDebug(boolean)   - 
Method in class weka.experiment. DatabaseUtils 
 Sets whether there should be printed some debugging output to stderr or not.
   setDebug(boolean)   - 
Method in class weka.filters. SimpleFilter 
 Sets the debugging mode
   setDebug(boolean)   - 
Method in class weka.filters.unsupervised.attribute. AddExpression 
 Set debug mode.
   setDebug(boolean)   - 
Method in class weka.gui. SimpleCLIPanel.CommandlineCompletion 
 sets debug mode on/off.
   setDebug(boolean)   - 
Method in class weka.gui.streams. InstanceCounter 
  
   setDebug(boolean)   - 
Method in class weka.gui.streams. InstanceJoiner 
  
   setDebug(boolean)   - 
Method in class weka.gui.streams. InstanceLoader 
  
   setDebug(boolean)   - 
Method in class weka.gui.streams. InstanceSavePanel 
  
   setDebug(boolean)   - 
Method in class weka.gui.streams. InstanceTable 
  
   setDebug(boolean)   - 
Method in class weka.gui.streams. InstanceViewer 
  
   setDebugLevel(int)   - 
Method in class weka.clusterers. XMeans 
 Sets the debug level.
   setDebugVectorsFile(File)   - 
Method in class weka.clusterers. XMeans 
 Sets the file that has the random vectors stored.
   setDecay(boolean)   - 
Method in class weka.classifiers.functions. MultilayerPerceptron 
  
   setDecimals(int)   - 
Method in class weka.filters.unsupervised.attribute. NumericCleaner 
 Set the number of decimals to round to.
   setDefaultValue()   - 
Method in class weka.gui. GenericObjectEditor 
 Sets the current object to be the default, taken as the first item in
 the chooser.
   setDefaultWeight(double)   - 
Method in class weka.classifiers.functions. Winnow 
 Set the value of defaultWeight.
   setDegree(int)   - 
Method in class weka.classifiers.functions. LibSVM 
 Sets the degree of the kernel
   setDegreesOfFreedom(int)   - 
Method in class weka.experiment. PairedStats 
 Sets the degrees of freedom (if calibration is required).
   setDeleteEmptyBins(boolean)   - 
Method in class weka.classifiers.meta. RegressionByDiscretization 
 Sets the number of bins to divide each selected numeric attribute into
   setDelimiters(String)   - 
Method in class weka.core.tokenizers. CharacterDelimitedTokenizer 
 Set the value of delimiters.
   setDelta(double)   - 
Method in class weka.associations. Apriori 
 Set the value of delta.
   setDelta(double)   - 
Method in class weka.associations. FPGrowth 
 Set the value of delta.
   setDelta(double)   - 
Method in class weka.classifiers.bayes.net.search.global. SimulatedAnnealing 
 Sets the m_fDelta.
   setDelta(double)   - 
Method in class weka.classifiers.bayes.net.search.local. SimulatedAnnealing 
 Sets the m_fDelta.
   setDensityBasedClusterer(DensityBasedClusterer)   - 
Method in class weka.filters.unsupervised.attribute. ClusterMembership 
 Set the clusterer for use in filtering
   setDescendantPopulationSize(int)   - 
Method in class weka.classifiers.bayes.net.search.global. GeneticSearch 
  
   setDescendantPopulationSize(int)   - 
Method in class weka.classifiers.bayes.net.search.local. GeneticSearch 
  
   setDescendents(ArrayList, C45PruneableClassifierTreeG)   - 
Method in class weka.classifiers.trees.j48. C45PruneableClassifierTreeG 
 add the grafted nodes at originalLeaf's position in tree.
   setDesign(boolean)   - 
Method in class weka.gui.beans. AttributeSummarizer 
 Set whether the appearance of this bean should be design or
 application
   setDesignatedClass(SelectedTag)   - 
Method in class weka.classifiers.meta. ThresholdSelector 
 Sets the method to determine which class value to optimize.
   setDesiredSize(int)   - 
Method in class weka.classifiers.meta. Decorate 
 Sets the desired size of the committee.
   setDesiredWeightOfInstancesPerInterval(double)   - 
Method in class weka.filters.unsupervised.attribute. Discretize 
 Set the DesiredWeightOfInstancesPerInterval value.
   setDestination(File)   - 
Method in class weka.core.converters. AbstractFileSaver 
 Sets the destination file (and directories if necessary).
   setDestination(OutputStream)   - 
Method in class weka.core.converters. AbstractFileSaver 
 Sets the destination output stream.
   setDestination(File)   - 
Method in class weka.core.converters. AbstractSaver 
 Default implementation throws an IOException.
   setDestination(OutputStream)   - 
Method in class weka.core.converters. AbstractSaver 
 Default implementation throws an IOException.
   setDestination(OutputStream)   - 
Method in class weka.core.converters. ArffSaver 
 Sets the destination output stream.
   setDestination(String, String, String)   - 
Method in class weka.core.converters. DatabaseSaver 
 Sets the database url.
   setDestination(String)   - 
Method in class weka.core.converters. DatabaseSaver 
 Sets the database url.
   setDestination()   - 
Method in class weka.core.converters. DatabaseSaver 
 Sets the database url using the DatabaseUtils file.
   setDestination(File)   - 
Method in interface weka.core.converters. Saver 
 Resets the Saver object and sets the destination to be 
 the supplied File object.
   setDestination(OutputStream)   - 
Method in interface weka.core.converters. Saver 
 Resets the Saver object and sets the destination to be
 the supplied InputStream.
   setDestination(OutputStream)   - 
Method in class weka.core.converters. SerializedInstancesSaver 
 Sets the destination output stream.
   setDestination(OutputStream)   - 
Method in class weka.core.converters. XRFFSaver 
 Sets the destination output stream.
   setDetectionPerAttribute(boolean)   - 
Method in class weka.filters.unsupervised.attribute. InterquartileRange 
 Set whether an Outlier/ExtremeValue attribute pair is generated for 
 each numeric attribute ("true") or just one pair for all numeric 
 attributes together ("false").
   setDir(String)   - 
Method in class weka.core.converters. AbstractFileSaver 
 Sets the directory where the instances should be stored
   setDir(String)   - 
Method in class weka.core.converters. AbstractSaver 
 Default implementation throws an IOException.
   setDir(String)   - 
Method in interface weka.core.converters. Saver 
 Sets the directory of the output file.
   setDir(String)   - 
Method in class weka.core. Javadoc 
 sets the dir containing the file that is to be updated.
   setDirAndPrefix(String, String)   - 
Method in class weka.core.converters. AbstractFileSaver 
 Sets the directory and the file prefix.
   setDirAndPrefix(String, String)   - 
Method in class weka.core.converters. AbstractSaver 
 Default implementation throws an IOException.
   setDirAndPrefix(String, String)   - 
Method in interface weka.core.converters. Saver 
 Sets the file prefix and the directory.
   setDirection(SelectedTag)   - 
Method in class weka.attributeSelection. BestFirst 
 Set the search direction
   setDirectory(File)   - 
Method in class weka.core.converters. TextDirectoryLoader 
 sets the source directory
   setDirectory(File)   - 
Method in class weka.gui.beans. SerializedModelSaver 
 Set the directory that the model(s) will be saved into.
   setDiscretizeBin(int)   - 
Method in class weka.classifiers.mi. MIBoost 
 Set the number of bins in discretization
   setDisplayConnectors(boolean)   - 
Method in class weka.gui.beans. BeanVisual 
 Turn on/off the connector points
   setDisplayConnectors(boolean, Color)   - 
Method in class weka.gui.beans. BeanVisual 
 Turn on/off the connector points
   setDisplayedFromDialog()   - 
Method in class weka.gui.experiment. ResultsPanel 
  
   setDisplayedResultsets(int[])   - 
Method in class weka.experiment. PairedTTester 
 Sets the indicies of the datasets to display ( null  means all).
   setDisplayedResultsets(int[])   - 
Method in interface weka.experiment. Tester 
 Sets the indicies of the datasets to display ( null  means all).
   setDisplayModelInOldFormat(boolean)   - 
Method in class weka.classifiers.bayes. NaiveBayes 
 Set whether to display model output in the old, original
 format.
   setDisplayModelInOldFormat(boolean)   - 
Method in class weka.clusterers. EM 
 Set whether to display model output in the old, original
 format.
   setDisplayRules(boolean)   - 
Method in class weka.classifiers.rules. DecisionTable 
 Sets whether rules are to be printed
   setDisplayStdDevs(boolean)   - 
Method in class weka.clusterers. SimpleKMeans 
 Sets whether standard deviations and nominal count
 Should be displayed in the clustering output
   setDistanceF(DistanceFunction)   - 
Method in class weka.clusterers. XMeans 
 gets the "binary" distance value.
   setDistanceFunction(DistanceFunction)   - 
Method in class weka.clusterers. HierarchicalClusterer 
  
   setDistanceFunction(DistanceFunction)   - 
Method in class weka.clusterers. SimpleKMeans 
 sets the distance function to use for instance comparison.
   setDistanceFunction(DistanceFunction)   - 
Method in class weka.core.neighboursearch. CoverTree 
 Sets the distance function to use for nearest neighbour search.
   setDistanceFunction(DistanceFunction)   - 
Method in class weka.core.neighboursearch. KDTree 
 sets the distance function to use for nearest neighbour search.
   setDistanceFunction(DistanceFunction)   - 
Method in class weka.core.neighboursearch. NearestNeighbourSearch 
 sets the distance function to use for nearest neighbour search.
   setDistanceIsBranchLength(boolean)   - 
Method in class weka.clusterers. HierarchicalClusterer 
  
   setDistanceWeighting(SelectedTag)   - 
Method in class weka.classifiers.lazy. IBk 
 Sets the distance weighting method used.
   setDistMult(double)   - 
Method in class weka.datagenerators.clusterers. BIRCHCluster 
 Sets the distance multiplier.
   setDistribution(String, double[][])   - 
Method in class weka.classifiers.bayes.net. EditableBayesNet 
 specify distribution of a node
   setDistribution(int, double[][])   - 
Method in class weka.classifiers.bayes.net. EditableBayesNet 
 specify distribution of a node
   setDistribution(SelectedTag)   - 
Method in class weka.filters.unsupervised.attribute. RandomProjection 
 Sets the distribution to use for calculating the random matrix
   setDistributionSpread(double)   - 
Method in class weka.filters.supervised.instance. SpreadSubsample 
 Sets the value for the distribution spread
   setDocType(String)   - 
Method in class weka.core.xml. XMLDocument 
 sets the DOCTYPE-String to use in the XML output.
   setDocument(Document)   - 
Method in class weka.core.xml. XMLDocument 
 sets the DOM document to use.
   setDoNotOperateOnPerClassBasis(boolean)   - 
Method in class weka.filters.unsupervised.attribute. StringToWordVector 
 Set the DoNotOperateOnPerClassBasis value.
   setDoNotReplaceMissingValues(boolean)   - 
Method in class weka.classifiers.functions. LibLINEAR 
 Whether to turn off automatic replacement of missing values.
   setDoNotReplaceMissingValues(boolean)   - 
Method in class weka.classifiers.functions. LibSVM 
 Whether to turn off automatic replacement of missing values.
   setDontFilterAfterFirstBatch(boolean)   - 
Method in class weka.filters.unsupervised.instance. RemoveWithValues 
 Set whether to apply the filter to instances that arrive once
 the first (training) batch has been seen.
   setDontNormalize(boolean)   - 
Method in class weka.classifiers.functions. SPegasos 
 Turn normalization off/on.
   setDontNormalize(boolean)   - 
Method in class weka.core. NormalizableDistance 
 Sets whether if the attribute values are to be normalized in distance
 calculation.
   setDontReplaceMissing(boolean)   - 
Method in class weka.classifiers.functions. SPegasos 
 Turn global replacement of missing values off/on.
   setDontReplaceMissingValues(boolean)   - 
Method in class weka.clusterers. SimpleKMeans 
 Sets whether missing values are to be replaced
   setElement(int, int, double)   - 
Method in class weka.classifiers. CostMatrix 
 Set the value of a cell as a double
   setElement(int, double)   - 
Method in class weka.core. AlgVector 
 Sets an element of the matrix to the given value.
   setElement(int, int, double)   - 
Method in class weka.core. Matrix 
  Deprecated.  Sets an element of the matrix to the given value.
   setElementAt(Object, int)   - 
Method in class weka.core. FastVector 
 Sets the element at the given index.
   setElementAt(Object, int)   - 
Method in class weka.gui. CheckBoxList.CheckBoxListModel 
 Sets the component at the specified index of this list to be the 
 specified object.
   setElements(double[])   - 
Method in class weka.core. AlgVector 
 Sets the elements of the vector to values of the given array.
   setEliminateColinearAttributes(boolean)   - 
Method in class weka.classifiers.functions. LinearRegression 
 Set the value of EliminateColinearAttributes.
   setEnabled(boolean)   - 
Method in class weka.core. Debug 
 sets whether the logging is enabled or not
   setEnabled(boolean)   - 
Method in class weka.core. Memory 
 sets whether the memory management is enabled
   setEnabled(boolean)   - 
Method in class weka.gui. GenericObjectEditor 
 Sets whether the editor is "enabled", meaning that the current
 values will be painted.
   setEnabled(boolean)   - 
Method in class weka.gui. PropertyPanel 
 Passes on enabled/disabled status to the custom
 panel (if one is set).
   setEntropicAutoBlend(boolean)   - 
Method in class weka.classifiers.lazy. KStar 
 Set whether entropic blending is to be used.
   setEnumerateColNames(boolean)   - 
Method in class weka.experiment. ResultMatrix 
 sets whether the column names are prefixed with "(x)" where "x" is
 the index
   setEnumerateRowNames(boolean)   - 
Method in class weka.experiment. ResultMatrix 
 sets whether to the row names or numbers instead are enumerateed
   setEnvironment(Environment)   - 
Method in class weka.core.converters. AbstractFileLoader 
 Set the environment variables to use.
   setEnvironment(Environment)   - 
Method in class weka.core.converters. AbstractFileSaver 
 Set the environment variables to use.
   setEnvironment(Environment)   - 
Method in interface weka.core. EnvironmentHandler 
 Set environment variables to use.
   setEnvironment(Environment)   - 
Method in class weka.gui.beans. FlowRunner 
 Set the environment variables to use.
   setEnvironment(Environment)   - 
Method in class weka.gui.beans. KnowledgeFlowApp 
 Set the environment variables to use.
   setEnvironment(Environment)   - 
Method in class weka.gui.beans. Loader 
 Set environment variables to use.
   setEnvironment(Environment)   - 
Method in class weka.gui.beans. Saver 
 Set environment variables to use.
   setEnvironment(Environment)   - 
Method in class weka.gui.beans. SerializedModelSaver 
 Set environment variables to use.
   setEpochs(int)   - 
Method in class weka.classifiers.functions. SPegasos 
 Set the number of epochs to use
   setEps(double)   - 
Method in class weka.classifiers.functions. LibLINEAR 
 Sets tolerance of termination criterion (default 0.001)
   setEps(double)   - 
Method in class weka.classifiers.functions. LibSVM 
 Sets tolerance of termination criterion (default 0.001)
   setEpsilon(double)   - 
Method in class weka.classifiers.functions. SMO 
 Set the value of epsilon.
   setEpsilon(double)   - 
Method in class weka.classifiers.functions.supportVector. RegSMO 
 Set the value of epsilon.
   setEpsilon(double)   - 
Method in class weka.classifiers.mi. MISMO 
 Set the value of epsilon.
   setEpsilon(double)   - 
Method in class weka.clusterers. DBScan 
 Sets a new value for epsilon
   setEpsilon(double)   - 
Method in class weka.clusterers. OPTICS 
 Sets a new value for epsilon
   setEpsilonParameter(double)   - 
Method in class weka.attributeSelection. SVMAttributeEval 
 Set the value of P for SMO
   setEpsilonParameter(double)   - 
Method in class weka.classifiers.functions.supportVector. RegOptimizer 
 Set the value of epsilon parameter of the epsilon insensitive loss function.
   setErrorOnProbabilities(boolean)   - 
Method in class weka.classifiers.functions. SimpleLogistic 
 Set the value of errorOnProbabilities.
   setErrorOnProbabilities(boolean)   - 
Method in class weka.classifiers.trees. FT 
 Set the value of errorOnProbabilities.
   setErrorOnProbabilities(boolean)   - 
Method in class weka.classifiers.trees. LMT 
 Set the value of errorOnProbabilities.
   setEstimator(BayesNetEstimator)   - 
Method in class weka.classifiers.bayes. BayesNet 
 Set the Estimator Algorithm used in calculating the CPTs
   setEstimator(SelectedTag)   - 
Method in class weka.classifiers.functions. PaceRegression 
 Sets the estimator.
   setEstimator(Estimator)   - 
Method in class weka.estimators. CheckEstimator 
 Set the estimator for boosting.
   setEuclideanDistanceFunction(EuclideanDistance)   - 
Method in class weka.core.neighboursearch.balltrees. BallSplitter 
 Sets the distance function used to (or to be used 
 to) build the tree.
   setEuclideanDistanceFunction(EuclideanDistance)   - 
Method in class weka.core.neighboursearch.balltrees. BallTreeConstructor 
 Sets the distance function to use to build the 
 tree.
   setEuclideanDistanceFunction(EuclideanDistance)   - 
Method in class weka.core.neighboursearch.kdtrees. KDTreeNodeSplitter 
 Sets the EuclideanDistance object to use for 
 splitting nodes.
   setEvaluation(SelectedTag)   - 
Method in class weka.classifiers.meta. GridSearch 
 Sets the criterion to use for evaluating the classifier performance.
   setEvaluationMeasure(SelectedTag)   - 
Method in class weka.classifiers.rules. DecisionTable 
 Sets the performance evaluation measure to use for selecting attributes
 for the decision table
   setEvaluationMode(SelectedTag)   - 
Method in class weka.classifiers.meta. ThresholdSelector 
 Sets the evaluation mode used.
   setEvaluator(ASEvaluation)   - 
Method in class weka.attributeSelection. AttributeSelection 
 set the attribute/subset evaluator
   setEvaluator(ASEvaluation)   - 
Method in class weka.attributeSelection. CheckAttributeSelection 
 Set the evaluator to test.
   setEvaluator(ASEvaluation)   - 
Method in class weka.attributeSelection. CostSensitiveASEvaluation 
 Set the base evaluator.
   setEvaluator(ASEvaluation)   - 
Method in class weka.attributeSelection. CostSensitiveAttributeEval 
 Set the base evaluator.
   setEvaluator(ASEvaluation)   - 
Method in class weka.attributeSelection. CostSensitiveSubsetEval 
 Set the base evaluator.
   setEvaluator(ASEvaluation)   - 
Method in class weka.classifiers.meta. AttributeSelectedClassifier 
 Sets the attribute evaluator
   setEvaluator(ASEvaluation)   - 
Method in class weka.filters.supervised.attribute. AttributeSelection 
 set attribute/subset evaluator
   setEvalUsingTrainingData(boolean)   - 
Method in class weka.attributeSelection. OneRAttributeEval 
 Use the training data to evaluate attributes rather than cross validation
   setEvidence(int, int)   - 
Method in class weka.classifiers.bayes.net. EditableBayesNet 
 set evidence state of a node.
   setEvidence(int, int)   - 
Method in class weka.classifiers.bayes.net. MarginCalculator.JunctionTreeNode 
  
   setEvidence(int, int)   - 
Method in class weka.classifiers.bayes.net. MarginCalculator 
  
   setExclusive(boolean)   - 
Method in class weka.classifiers.rules. ConjunctiveRule 
 Sets whether exclusive expressions for nominal attributes splits are 
 considered
   setExecutionSlots(int)   - 
Method in class weka.gui.beans. Classifier 
 Set the number of execution slots (threads) to use to
 train models with.
   setExecutionStatus(int)   - 
Method in class weka.experiment. TaskStatusInfo 
 Set the execution status of this Task.
   setExitIfNoWindowsOpen(boolean)   - 
Static method in class weka.gui.boundaryvisualizer. BoundaryVisualizer 
 Sets whether System.exit gets called when no more windows are open.
   setExitOnClose(boolean)   - 
Method in class weka.gui.arffviewer. ArffViewer 
 whether to do a System.exit(0) on close
   setExitOnClose(boolean)   - 
Method in class weka.gui.arffviewer. ArffViewerMainPanel 
 whether to do a System.exit(0) on close
   setExpectedResultsPerAverage(int)   - 
Method in class weka.experiment. AveragingResultProducer 
 Set the value of ExpectedResultsPerAverage.
   setExperiment(Experiment)   - 
Method in class weka.experiment. RemoteExperimentSubTask 
 Set the experiment for this sub task
   setExperiment(Experiment)   - 
Method in class weka.gui.experiment. AlgorithmListPanel 
 Tells the panel to act on a new experiment.
   setExperiment(Experiment)   - 
Method in class weka.gui.experiment. DatasetListPanel 
 Tells the panel to act on a new experiment.
   setExperiment(Experiment)   - 
Method in class weka.gui.experiment. DistributeExperimentPanel 
 Sets the experiment to be configured.
   setExperiment(Experiment)   - 
Method in class weka.gui.experiment. GeneratorPropertyIteratorPanel 
 Sets the experiment which will have the custom properties edited.
   setExperiment(RemoteExperiment)   - 
Method in class weka.gui.experiment. HostListPanel 
 Tells the panel to act on a new experiment.
   setExperiment(Experiment)   - 
Method in class weka.gui.experiment. ResultsPanel 
 Tells the panel to use a new experiment.
   setExperiment(Experiment)   - 
Method in class weka.gui.experiment. RunNumberPanel 
 Sets the experiment to be configured.
   setExperiment(Experiment)   - 
Method in class weka.gui.experiment. RunPanel 
 Sets the experiment the panel operates on.
   setExperiment(Experiment)   - 
Method in class weka.gui.experiment. SetupPanel 
 Sets the experiment to configure.
   setExperiment(Experiment)   - 
Method in class weka.gui.experiment. SimpleSetupPanel 
 Sets the experiment to configure.
   setExplicitPropsFile(boolean)   - 
Method in class weka.gui. GenericPropertiesCreator 
 if FALSE, the locating of a props-file of the Utils-class is used, 
 otherwise it's tried to load the specified file
   setExplorer(Explorer)   - 
Method in class weka.gui.explorer. AssociationsPanel 
 Sets the Explorer to use as parent frame (used for sending notifications
 about changes in the data)
   setExplorer(Explorer)   - 
Method in class weka.gui.explorer. AttributeSelectionPanel 
 Sets the Explorer to use as parent frame (used for sending notifications
 about changes in the data)
   setExplorer(Explorer)   - 
Method in class weka.gui.explorer. ClassifierPanel 
 Sets the Explorer to use as parent frame (used for sending notifications
 about changes in the data)
   setExplorer(Explorer)   - 
Method in class weka.gui.explorer. ClustererPanel 
 Sets the Explorer to use as parent frame (used for sending notifications
 about changes in the data)
   setExplorer(Explorer)   - 
Method in interface weka.gui.explorer. Explorer.ExplorerPanel 
 Sets the Explorer to use as parent frame (used for sending notifications
 about changes in the data)
   setExplorer(Explorer)   - 
Method in class weka.gui.explorer. PreprocessPanel 
 Sets the Explorer to use as parent frame (used for sending notifications
 about changes in the data)
   setExplorer(Explorer)   - 
Method in class weka.gui.explorer. VisualizePanel 
 Sets the Explorer to use as parent frame (used for sending notifications
 about changes in the data)
   setExponent(double)   - 
Method in class weka.classifiers.functions.supportVector. NormalizedPolyKernel 
 Sets the exponent value (must be different from 1.0).
   setExponent(double)   - 
Method in class weka.classifiers.functions.supportVector. PolyKernel 
 Sets the exponent value.
   setExponent(double)   - 
Method in class weka.classifiers.functions. VotedPerceptron 
 Set the value of exponent.
   setExpression(String)   - 
Method in class weka.datagenerators.classifiers.regression. Expression 
 Sets the mathematical expression to generate y out of x.
   setExpression(String)   - 
Method in class weka.filters.unsupervised.attribute. AddExpression 
 Set the expression to apply
   setExpression(String)   - 
Method in class weka.filters.unsupervised.attribute. MathExpression 
 Set the expression to apply
   setExpression(String)   - 
Method in class weka.filters.unsupervised.instance. SubsetByExpression 
 Sets the expression used for filtering.
   setExtremeValuesAsOutliers(boolean)   - 
Method in class weka.filters.unsupervised.attribute. InterquartileRange 
 Set whether extreme values are also tagged as outliers.
   setExtremeValuesFactor(double)   - 
Method in class weka.filters.unsupervised.attribute. InterquartileRange 
 Sets the factor for determining the thresholds for extreme values.
   setFalseNegative(double)   - 
Method in class weka.classifiers.evaluation. TwoClassStats 
 Sets the number of positive instances predicted as negative
   setFalsePositive(double)   - 
Method in class weka.classifiers.evaluation. TwoClassStats 
 Sets the number of negative instances predicted as positive
   setFastRegression(boolean)   - 
Method in class weka.classifiers.trees. LMT 
 Set the value of fastRegression.
   setFieldDefs(ArrayList<Attribute>)   - 
Method in class weka.core.pmml. DerivedFieldMetaInfo 
 Upadate the field definitions for this derived field
   setFieldDefs(ArrayList<Attribute>)   - 
Method in class weka.core.pmml. Discretize 
 Set the field definitions for this Expression to use
   setFieldDefs(ArrayList<Attribute>)   - 
Method in class weka.core.pmml. Expression 
 Set the field definitions for this Expression to use
   setFieldDefs(ArrayList<Attribute>)   - 
Method in class weka.core.pmml. FieldRef 
  
   setFieldDefs(ArrayList<Attribute>)   - 
Method in class weka.core.pmml. NormContinuous 
 Set the field definitions for this Expression to use
   setFieldDefs(ArrayList<Attribute>)   - 
Method in class weka.core.pmml. NormDiscrete 
 Set the field definitions for this Expression to use
   setFile(File)   - 
Method in class weka.core.converters. AbstractFileLoader 
 sets the source File
   setFile(File)   - 
Method in class weka.core.converters. AbstractFileSaver 
 Sets the destination file.
   setFile(File)   - 
Method in class weka.core.converters. AbstractSaver 
 Default implementation throws an IOException.
   setFile(File)   - 
Method in class weka.core.converters. ArffLoader 
 sets the source File
   setFile(File)   - 
Method in class weka.core.converters. ArffSaver 
 Sets the destination file.
   setFile(File)   - 
Method in interface weka.core.converters. FileSourcedConverter 
 Set the file to load from/ to save in
   setFile(File)   - 
Method in interface weka.core.converters. Saver 
 Sets the output file
   setFile(File)   - 
Method in class weka.core.converters. XRFFSaver 
 Sets the destination file.
   setFile(File)   - 
Method in class weka.gui.visualize. JComponentWriter 
 sets the file to store the output in
   setFileFormat(Tag)   - 
Method in class weka.gui.beans. SerializedModelSaver 
 Set the file format to use for saving.
   setFileMustExist(boolean)   - 
Method in class weka.gui. ConverterFileChooser 
 Whether the selected file must exst (only open dialog).
   setFilename(String)   - 
Method in class weka.core. FindWithCapabilities 
 Sets the dataset filename to base the capabilities on.
   setFilename(String)   - 
Method in class weka.gui.arffviewer. ArffPanel 
 sets the filename
   setFilename(int, String)   - 
Method in class weka.gui.arffviewer. ArffViewerMainPanel 
 sets the filename of the specified panel
   setFilePrefix(String)   - 
Method in class weka.core.converters. AbstractFileSaver 
 Sets the file name prefix
   setFilePrefix(String)   - 
Method in class weka.core.converters. AbstractSaver 
 Default implementation throws an IOException.
   setFilePrefix(String)   - 
Method in interface weka.core.converters. Saver 
 Sets the file prefix.
   setFillWithMissing(boolean)   - 
Method in class weka.filters.unsupervised.attribute. AbstractTimeSeries 
 Sets whether missing values should be used rather than removing instances
 where the translated value is not known (due to border effects).
   setFilter(Filter)   - 
Method in class weka.associations. FilteredAssociator 
 Sets the filter
   setFilter(Filter)   - 
Method in class weka.attributeSelection. FilteredAttributeEval 
 Set the filter to use
   setFilter(Filter)   - 
Method in class weka.attributeSelection. FilteredSubsetEval 
 Set the filter to use
   setFilter(Filter)   - 
Method in class weka.classifiers.functions. PLSClassifier 
 Set the PLS filter (only used for setup).
   setFilter(Filter)   - 
Method in class weka.classifiers.meta. FilteredClassifier 
 Sets the filter
   setFilter(Filter)   - 
Method in class weka.classifiers.meta. GridSearch 
 Set the kernel filter (only used for setup).
   setFilter(Filter)   - 
Method in class weka.clusterers. FilteredClusterer 
 Sets the filter.
   setFilter(Filter)   - 
Method in class weka.filters. CheckSource 
 Sets the filter to use for the comparison.
   setFilter(Filter)   - 
Method in class weka.filters.unsupervised.attribute. Wavelet 
 Set the preprocessing filter (only used for setup).
   setFilter(Filter)   - 
Method in class weka.gui.beans. Filter 
 Set the filter to be wrapped by this bean
   setFilterAfterFirstBatch(boolean)   - 
Method in class weka.filters.unsupervised.instance. SubsetByExpression 
 Set whether to apply the filter to instances that arrive once
 the first (training) batch has been seen.
   setFilterAttributes(String)   - 
Method in class weka.associations. GeneralizedSequentialPatterns 
 Sets the String containing the attributes which are used for output 
 filtering.
   setFilters(Filter[])   - 
Method in class weka.filters. MultiFilter 
 Sets the list of possible filters to choose from.
   setFilters(Filter[])   - 
Method in class weka.filters.unsupervised.attribute. PartitionedMultiFilter 
 Sets the list of possible filters to choose from.
   setFilterType(SelectedTag)   - 
Method in class weka.attributeSelection. SVMAttributeEval 
 The filtering mode to pass to SMO
   setFilterType(SelectedTag)   - 
Method in class weka.classifiers.functions. GaussianProcesses 
 Sets how the training data will be transformed.
   setFilterType(SelectedTag)   - 
Method in class weka.classifiers.functions. SMO 
 Sets how the training data will be transformed.
   setFilterType(SelectedTag)   - 
Method in class weka.classifiers.functions. SMOreg 
 Sets how the training data will be transformed.
   setFilterType(SelectedTag)   - 
Method in class weka.classifiers.mi. MDD 
 Sets how the training data will be transformed.
   setFilterType(SelectedTag)   - 
Method in class weka.classifiers.mi. MIDD 
 Sets how the training data will be transformed.
   setFilterType(SelectedTag)   - 
Method in class weka.classifiers.mi. MIEMDD 
 Sets how the training data will be transformed.
   setFilterType(SelectedTag)   - 
Method in class weka.classifiers.mi. MIOptimalBall 
 Sets how the training data will be transformed.
   setFilterType(SelectedTag)   - 
Method in class weka.classifiers.mi. MISMO 
 Sets how the training data will be transformed.
   setFilterType(SelectedTag)   - 
Method in class weka.classifiers.mi. MISVM 
 Sets how the training data will be transformed.
   setFindAllRulesForSupportLevel(boolean)   - 
Method in class weka.associations. FPGrowth 
 If true then turn off the iterative support reduction method
 of finding x rules that meet the minimum support and metric
 thresholds and just return all the rules that meet the
 lower bound on minimum support and the minimum metric.
   setFindNumBins(boolean)   - 
Method in class weka.filters.unsupervised.attribute. Discretize 
 Set the value of FindNumBins.
   setFindNumBins(boolean)   - 
Method in class weka.filters.unsupervised.attribute. PKIDiscretize 
 Set the value of FindNumBins.
   setFirstValueIndex(String)   - 
Method in class weka.filters.unsupervised.attribute. MergeTwoValues 
 Sets index of the first value used.
   setFirstValueIndex(String)   - 
Method in class weka.filters.unsupervised.attribute. SwapValues 
 Sets index of the first value used.
   setFlow(Vector)   - 
Method in class weka.gui.beans. KnowledgeFlowApp 
 Set the flow for the KnowledgeFlow to edit.
   setFlows(Vector)   - 
Method in class weka.gui.beans. FlowRunner 
 Set the vector holding the flows(s) to run
   setFocus()   - 
Method in class weka.gui.sql. ConnectionPanel 
 sets the focus in a designated control.
   setFocus()   - 
Method in class weka.gui.sql. InfoPanel 
 sets the focus in a designated control
   setFocus()   - 
Method in class weka.gui.sql. QueryPanel 
 sets the focus in a designated control.
   setFocus()   - 
Method in class weka.gui.sql. ResultPanel 
 sets the focus in a designated control
   setFold(int)   - 
Method in class weka.filters.supervised.instance. StratifiedRemoveFolds 
 Selects a fold.
   setFold(int)   - 
Method in class weka.filters.unsupervised.instance. RemoveFolds 
 Selects a fold.
   setFoldColumn(int)   - 
Method in class weka.experiment. PairedTTester 
 Set the value of FoldColumn.
   setFoldColumn(int)   - 
Method in interface weka.experiment. Tester 
 Set the value of FoldColumn.
   setFolds(int)   - 
Method in class weka.attributeSelection. AttributeSelection 
 set the number of folds for cross validation
   setFolds(int)   - 
Method in class weka.attributeSelection. OneRAttributeEval 
 Set the number of folds to use for cross validation
   setFolds(int)   - 
Method in class weka.attributeSelection. WrapperSubsetEval 
 Set the number of folds to use for accuracy estimation
   setFolds(int)   - 
Method in class weka.classifiers.rules. ConjunctiveRule 
 the number of folds to use
   setFolds(int)   - 
Method in class weka.classifiers.rules. JRip 
 Sets the number of folds to use
   setFolds(int)   - 
Method in class weka.classifiers.rules. Ridor 
  
   setFolds(int)   - 
Method in class weka.gui.beans. CrossValidationFoldMaker 
 Set the number of folds for the cross validation
   setFoldsType(SelectedTag)   - 
Method in class weka.attributeSelection. RaceSearch 
 Set the xfold type
   setFont(Font)   - 
Method in class weka.gui.visualize. PostscriptGraphics 
 Set current font.
   setFormat(String)   - 
Method in class weka.core. Debug.Timestamp 
 sets the format for the timestamp
   setForwardSelectionMethod(SelectedTag)   - 
Method in class weka.attributeSelection. LinearForwardSelection 
 Set the search direction
   setFrequencyLimit(int)   - 
Method in class weka.classifiers.bayes. AODE 
 Sets the frequency limit
   setFrequencyLimit(int)   - 
Method in class weka.classifiers.bayes. AODEsr 
 Sets the frequency limit
   setFrequencyThreshold(double)   - 
Method in class weka.associations. Tertius 
 Set the value of frequencyThreshold.
   setFunction(SelectedTag)   - 
Method in class weka.datagenerators.classifiers.classification. Agrawal 
 Sets the function for generating the data.
   setFunctionValue(int, double)   - 
Method in class weka.classifiers.functions.pace. DiscreteFunction 
 Sets a particular function value
   setGamma(double)   - 
Method in class weka.classifiers.functions. LibSVM 
 Sets gamma (default = 1/no of attributes)
   setGamma(double)   - 
Method in class weka.classifiers.functions.supportVector. RBFKernel 
 Sets the gamma value.
   setGenerateRanking(boolean)   - 
Method in class weka.attributeSelection. GreedyStepwise 
 Records whether the user has requested a ranked list of attributes.
   setGenerateRanking(boolean)   - 
Method in class weka.attributeSelection. RaceSearch 
 Records whether the user has requested a ranked list of attributes.
   setGenerateRanking(boolean)   - 
Method in interface weka.attributeSelection. RankedOutputSearch 
 Sets whether or not ranking is to be performed.
   setGenerateRanking(boolean)   - 
Method in class weka.attributeSelection. Ranker 
 This is a dummy set method---Ranker is ONLY capable of producing
 a ranked list of attributes for attribute evaluators.
   setGenerator(DataGenerator)   - 
Method in class weka.gui.explorer. DataGeneratorPanel 
 sets the generator to use initially
   setGeneratorSamplesBase(double)   - 
Method in class weka.gui.boundaryvisualizer. BoundaryPanel 
 Set the base for computing the number of samples to obtain from each
 generator.
   setGeneratorSamplesBase(double)   - 
Method in class weka.gui.boundaryvisualizer. RemoteBoundaryVisualizerSubTask 
 Set the base for computing the number of samples to obtain from each
 generator.
   setGlobalBlend(int)   - 
Method in class weka.classifiers.lazy. KStar 
 Set the global blend parameter
   setGlobalModel(NBTreeNoSplit)   - 
Method in class weka.classifiers.trees.j48. NBTreeSplit 
 Set the global naive bayes model for this node
   setGridIsExtendable(boolean)   - 
Method in class weka.classifiers.meta. GridSearch 
 Set whether the grid can be extended dynamically.
   setGridWidth(int)   - 
Method in class weka.gui.beans. AttributeSummarizer 
 Set the width of the grid of plots
   setGroupIdentifier(long)   - 
Method in class weka.gui.beans. BatchClassifierEvent 
  
   setGUI(boolean)   - 
Method in class weka.classifiers.functions. MultilayerPerceptron 
 This will set whether A GUI is brought up to allow interaction by the user
 with the neural network during training.
   setGUIType(SelectedTag)   - 
Method in class weka.gui. Main 
 Sets the type of GUI to use.
   setHandler(CapabilitiesHandler)   - 
Method in class weka.core. FindWithCapabilities 
 sets the Capabilities handler to generate the data for.
   setHandler(CapabilitiesHandler)   - 
Method in class weka.core. TestInstances 
 sets the Capabilities handler to generate the data for
   setHandleRightClicks(boolean)   - 
Method in class weka.gui. ResultHistoryPanel 
 Set whether the result history list should handle right clicks
 or whether the parent object will handle them.
   setHashtable(Hashtable)   - 
Method in class weka.classifiers.meta.nestedDichotomies. ClassBalancedND 
 Set hashtable from END.
   setHashtable(Hashtable)   - 
Method in class weka.classifiers.meta.nestedDichotomies. DataNearBalancedND 
 Set hashtable from END.
   setHashtable(Hashtable)   - 
Method in class weka.classifiers.meta.nestedDichotomies. ND 
 Set hashtable from END.
   setHDRank(int)   - 
Method in class weka.classifiers.mi. CitationKNN 
 Sets the rank associated to the Hausdorff distance
   setHeuristic(boolean)   - 
Method in class weka.classifiers.trees. BFTree 
 Set if use heuristic search for nominal attributes in multi-class problems.
   setHeuristic(boolean)   - 
Method in class weka.classifiers.trees. SimpleCart 
 Set if use heuristic search for nominal attributes in multi-class problems.
   setHeuristicStop(int)   - 
Method in class weka.classifiers.functions. SimpleLogistic 
 Set the value of heuristicStop.
   setHeuristicStop(int)   - 
Method in class weka.classifiers.trees.lmt. LogisticBase 
 Sets the option "heuristicStop".
   setHidden(boolean)   - 
Method in class weka.gui.beans. BeanConnection 
 Make this connection invisible on the display
   setHiddenLayers(String)   - 
Method in class weka.classifiers.functions. MultilayerPerceptron 
 This will set what the hidden layers are made up of when auto build is
 enabled.
   setHighlight(String)   - 
Method in class weka.gui.treevisualizer. TreeVisualizer 
 Set the highlight for the node with the given id
   setHistory(DefaultListModel)   - 
Method in class weka.gui.sql. ConnectionPanel 
 sets the local history to the given one.
   setHistory(DefaultListModel)   - 
Method in class weka.gui.sql. QueryPanel 
 sets the local history to the given one.
   setHoldOutFile(File)   - 
Method in class weka.attributeSelection. ClassifierSubsetEval 
 Set the file that contains hold out/test instances
   setHornClauses(boolean)   - 
Method in class weka.associations. Tertius 
 Set the value of hornClauses.
   setHyperparameterRange(String)   - 
Method in class weka.classifiers.bayes. BayesianLogisticRegression 
 Set the range of hyperparameter values to consider
 during CV-based selection
   setHyperparameterSelection(SelectedTag)   - 
Method in class weka.classifiers.bayes. BayesianLogisticRegression 
 Set the method used to select the hyperparameter
   setHyperparameterValue(double)   - 
Method in class weka.classifiers.bayes. BayesianLogisticRegression 
 Set the hyperparameter value.
   setID(int)   - 
Method in class weka.core. Tag 
 Sets the numeric ID of the Tag.
   setIDFTransform(boolean)   - 
Method in class weka.filters.unsupervised.attribute. StringToWordVector 
 Sets whether if the word frequencies in a document should be transformed
 into:  
 fij*log(num of Docs/num of Docs with word i)  
      where fij is the frequency of word i in document(instance) j.
   setIDIndex(String)   - 
Method in class weka.filters.unsupervised.attribute. AddID 
 Sets index of the attribute used.
   setIDStr(String)   - 
Method in class weka.core. Tag 
 Sets the string ID of the Tag.
   setIgnoreClass(boolean)   - 
Method in class weka.filters.unsupervised.attribute. PotentialClassIgnorer 
 Set the IgnoreClass value.
   setIgnoredAttributeIndices(String)   - 
Method in class weka.filters.unsupervised.attribute. AddCluster 
 Sets the ranges of attributes to be ignored.
   setIgnoredAttributeIndices(String)   - 
Method in class weka.filters.unsupervised.attribute. ClusterMembership 
 Sets the ranges of attributes to be ignored.
   setIgnoredProperties(String)   - 
Method in class weka.core. CheckGOE 
 Sets the properties to ignore in checkToolTips().
   setIgnoreRange(String)   - 
Method in class weka.filters.unsupervised.attribute. MathExpression 
 Set which attributes are to be ignored
   setIncludeClass(boolean)   - 
Method in class weka.core. InstanceComparator 
 sets whether the class should be included (= TRUE) in the comparison
   setIncludeClass(boolean)   - 
Method in class weka.filters.unsupervised.attribute. NumericCleaner 
 Sets whether the class can be cleaned, too.
   setIndex(int)   - 
Method in class weka.core.pmml. MiningFieldMetaInfo 
 Set the index of this field in the mining schema Instances
   setInitAsNaiveBayes(boolean)   - 
Method in class weka.classifiers.bayes.net.search.global. HillClimber 
 Sets whether to init as naive bayes
   setInitAsNaiveBayes(boolean)   - 
Method in class weka.classifiers.bayes.net.search.global. K2 
 Sets whether to init as naive bayes
   setInitAsNaiveBayes(boolean)   - 
Method in class weka.classifiers.bayes.net.search.local. HillClimber 
 Sets whether to init as naive bayes
   setInitAsNaiveBayes(boolean)   - 
Method in class weka.classifiers.bayes.net.search.local. K2 
 Sets whether to init as naive bayes
   setInitFile(File)   - 
Method in class weka.filters.unsupervised.attribute. KernelFilter 
 Sets the file to initialize the filter with, can be null.
   setInitFileClassIndex(String)   - 
Method in class weka.filters.unsupervised.attribute. KernelFilter 
 Sets class index of the file to initialize the filter with.
   setInitialAnchorRandom(boolean)   - 
Method in class weka.core.neighboursearch.balltrees. MiddleOutConstructor 
 Sets whether if the initial anchor is chosen randomly.
   setInputCenterFile(File)   - 
Method in class weka.clusterers. XMeans 
 Sets the file to read the list of centers from.
   setInputFilename(String)   - 
Method in class weka.gui. GenericPropertiesCreator 
 sets the file to get the information about the packages from.
   setInputFormat(Instances)   - 
Method in class weka.filters. AllFilter 
 Sets the format of the input instances.
   setInputFormat(Instances)   - 
Method in class weka.filters. Filter 
 Sets the format of the input instances.
   setInputFormat(Instances)   - 
Method in class weka.filters. SimpleFilter 
 Sets the format of the input instances.
   setInputFormat(Instances)   - 
Method in class weka.filters.supervised.attribute. ClassOrder 
 Sets the format of the input instances.
   setInputFormat(Instances)   - 
Method in class weka.filters.supervised.attribute. Discretize 
 Sets the format of the input instances.
   setInputFormat(Instances)   - 
Method in class weka.filters.supervised.attribute. NominalToBinary 
 Sets the format of the input instances.
   setInputFormat(Instances)   - 
Method in class weka.filters.supervised.instance. Resample 
 Sets the format of the input instances.
   setInputFormat(Instances)   - 
Method in class weka.filters.supervised.instance. SMOTE 
 Sets the format of the input instances.
   setInputFormat(Instances)   - 
Method in class weka.filters.supervised.instance. SpreadSubsample 
 Sets the format of the input instances.
   setInputFormat(Instances)   - 
Method in class weka.filters.supervised.instance. StratifiedRemoveFolds 
 Sets the format of the input instances.
   setInputFormat(Instances)   - 
Method in class weka.filters.unsupervised.attribute. AbstractTimeSeries 
 Sets the format of the input instances.
   setInputFormat(Instances)   - 
Method in class weka.filters.unsupervised.attribute. Add 
 Sets the format of the input instances.
   setInputFormat(Instances)   - 
Method in class weka.filters.unsupervised.attribute. AddCluster 
 Sets the format of the input instances.
   setInputFormat(Instances)   - 
Method in class weka.filters.unsupervised.attribute. AddExpression 
 Sets the format of the input instances.
   setInputFormat(Instances)   - 
Method in class weka.filters.unsupervised.attribute. AddID 
 Sets the format of the input instances.
   setInputFormat(Instances)   - 
Method in class weka.filters.unsupervised.attribute. AddNoise 
 Sets the format of the input instances.
   setInputFormat(Instances)   - 
Method in class weka.filters.unsupervised.attribute. AddValues 
 Sets the format of the input instances.
   setInputFormat(Instances)   - 
Method in class weka.filters.unsupervised.attribute. Center 
 Sets the format of the input instances.
   setInputFormat(Instances)   - 
Method in class weka.filters.unsupervised.attribute. ChangeDateFormat 
 Sets the format of the input instances.
   setInputFormat(Instances)   - 
Method in class weka.filters.unsupervised.attribute. ClusterMembership 
 Sets the format of the input instances.
   setInputFormat(Instances)   - 
Method in class weka.filters.unsupervised.attribute. Copy 
 Sets the format of the input instances.
   setInputFormat(Instances)   - 
Method in class weka.filters.unsupervised.attribute. Discretize 
 Sets the format of the input instances.
   setInputFormat(Instances)   - 
Method in class weka.filters.unsupervised.attribute. FirstOrder 
 Sets the format of the input instances.
   setInputFormat(Instances)   - 
Method in class weka.filters.unsupervised.attribute. MakeIndicator 
 Sets the format of the input instances.
   setInputFormat(Instances)   - 
Method in class weka.filters.unsupervised.attribute. MathExpression 
 Sets the format of the input instances.
   setInputFormat(Instances)   - 
Method in class weka.filters.unsupervised.attribute. MergeTwoValues 
 Sets the format of the input instances.
   setInputFormat(Instances)   - 
Method in class weka.filters.unsupervised.attribute. MultiInstanceToPropositional 
 Sets the format of the input instances.
   setInputFormat(Instances)   - 
Method in class weka.filters.unsupervised.attribute. NominalToBinary 
 Sets the format of the input instances.
   setInputFormat(Instances)   - 
Method in class weka.filters.unsupervised.attribute. NominalToString 
 Sets the format of the input instances.
   setInputFormat(Instances)   - 
Method in class weka.filters.unsupervised.attribute. Normalize 
 Sets the format of the input instances.
   setInputFormat(Instances)   - 
Method in class weka.filters.unsupervised.attribute. NumericToBinary 
 Sets the format of the input instances.
   setInputFormat(Instances)   - 
Method in class weka.filters.unsupervised.attribute. NumericTransform 
 Sets the format of the input instances.
   setInputFormat(Instances)   - 
Method in class weka.filters.unsupervised.attribute. Obfuscate 
 Sets the format of the input instances.
   setInputFormat(Instances)   - 
Method in class weka.filters.unsupervised.attribute. PKIDiscretize 
 Sets the format of the input instances.
   setInputFormat(Instances)   - 
Method in class weka.filters.unsupervised.attribute. PotentialClassIgnorer 
 Sets the format of the input instances.
   setInputFormat(Instances)   - 
Method in class weka.filters.unsupervised.attribute. PrincipalComponents 
 Sets the format of the input instances.
   setInputFormat(Instances)   - 
Method in class weka.filters.unsupervised.attribute. PropositionalToMultiInstance 
 Sets the format of the input instances.
   setInputFormat(Instances)   - 
Method in class weka.filters.unsupervised.attribute. RandomProjection 
 Sets the format of the input instances.
   setInputFormat(Instances)   - 
Method in class weka.filters.unsupervised.attribute. Remove 
 Sets the format of the input instances.
   setInputFormat(Instances)   - 
Method in class weka.filters.unsupervised.attribute. RemoveType 
 Sets the format of the input instances.
   setInputFormat(Instances)   - 
Method in class weka.filters.unsupervised.attribute. RemoveUseless 
 Sets the format of the input instances.
   setInputFormat(Instances)   - 
Method in class weka.filters.unsupervised.attribute. Reorder 
 Sets the format of the input instances.
   setInputFormat(Instances)   - 
Method in class weka.filters.unsupervised.attribute. ReplaceMissingValues 
 Sets the format of the input instances.
   setInputFormat(Instances)   - 
Method in class weka.filters.unsupervised.attribute. Standardize 
 Sets the format of the input instances.
   setInputFormat(Instances)   - 
Method in class weka.filters.unsupervised.attribute. StringToNominal 
 Sets the format of the input instances.
   setInputFormat(Instances)   - 
Method in class weka.filters.unsupervised.attribute. StringToWordVector 
 Sets the format of the input instances.
   setInputFormat(Instances)   - 
Method in class weka.filters.unsupervised.attribute. SwapValues 
 Sets the format of the input instances.
   setInputFormat(Instances)   - 
Method in class weka.filters.unsupervised.attribute. TimeSeriesDelta 
 Sets the format of the input instances.
   setInputFormat(Instances)   - 
Method in class weka.filters.unsupervised.attribute. TimeSeriesTranslate 
 Sets the format of the input instances.
   setInputFormat(Instances)   - 
Method in class weka.filters.unsupervised.instance. NonSparseToSparse 
 Sets the format of the input instances.
   setInputFormat(Instances)   - 
Method in class weka.filters.unsupervised.instance. Normalize 
 Sets the format of the input instances.
   setInputFormat(Instances)   - 
Method in class weka.filters.unsupervised.instance. Randomize 
 Sets the format of the input instances.
   setInputFormat(Instances)   - 
Method in class weka.filters.unsupervised.instance. RemoveFolds 
 Sets the format of the input instances.
   setInputFormat(Instances)   - 
Method in class weka.filters.unsupervised.instance. RemoveFrequentValues 
 Sets the format of the input instances.
   setInputFormat(Instances)   - 
Method in class weka.filters.unsupervised.instance. RemoveMisclassified 
 Sets the format of the input instances.
   setInputFormat(Instances)   - 
Method in class weka.filters.unsupervised.instance. RemovePercentage 
 Sets the format of the input instances.
   setInputFormat(Instances)   - 
Method in class weka.filters.unsupervised.instance. RemoveRange 
 Sets the format of the input instances.
   setInputFormat(Instances)   - 
Method in class weka.filters.unsupervised.instance. RemoveWithValues 
 Sets the format of the input instances.
   setInputFormat(Instances)   - 
Method in class weka.filters.unsupervised.instance. Resample 
 Sets the format of the input instances.
   setInputFormat(Instances)   - 
Method in class weka.filters.unsupervised.instance. ReservoirSample 
 Sets the format of the input instances.
   setInputFormat(Instances)   - 
Method in class weka.filters.unsupervised.instance. SparseToNonSparse 
 Sets the format of the input instances.
   setInputOrder(SelectedTag)   - 
Method in class weka.datagenerators.clusterers. BIRCHCluster 
 Sets the input order.
   setInputs(Vector)   - 
Method in class weka.gui.beans. MetaBean 
  
   setInstance(Instance)   - 
Method in class weka.gui.beans. InstanceEvent 
 Set the instance
   setInstanceIndex(int, boolean)   - 
Method in class weka.classifiers.lazy. LBR.Indexes 
 Changes the boolean value at the specified index in the InstIndexes array
   setInstanceList(int[])   - 
Method in class weka.core.neighboursearch.balltrees. BallSplitter 
 Sets the master index array containing indices of the
 training instances.
   setInstanceList(int[])   - 
Method in class weka.core.neighboursearch.balltrees. BallTreeConstructor 
 Sets the master index array that points to 
 instances in m_Instances, so that only this array
 is manipulated, and m_Instances is left 
 untouched.
   setInstanceList(int[])   - 
Method in class weka.core.neighboursearch.balltrees. MiddleOutConstructor 
 Sets the master index array that points to 
 instances in m_Instances, so that only this array
 is manipulated, and m_Instances is left 
 untouched.
   setInstanceList(int[])   - 
Method in class weka.core.neighboursearch.kdtrees. KDTreeNodeSplitter 
 Sets the master index array containing indices of the
 training instances.
   setInstanceRange(int)   - 
Method in class weka.filters.unsupervised.attribute. AbstractTimeSeries 
 Sets the number of instances forward to translate values between.
   setInstances(Instances)   - 
Method in class weka.core.converters. AbstractSaver 
 Sets instances that should be stored.
   setInstances(Instances)   - 
Method in class weka.core.converters. LibSVMSaver 
 Sets instances that should be stored.
   setInstances(Instances)   - 
Method in interface weka.core.converters. Saver 
 Sets the instances to be saved
   setInstances(Instances)   - 
Method in class weka.core.converters. SVMLightSaver 
 Sets instances that should be stored.
   setInstances(Instances)   - 
Method in class weka.core.converters. XRFFSaver 
 Sets instances that should be stored.
   setInstances(Instances)   - 
Method in interface weka.core. DistanceFunction 
 Sets the instances.
   setInstances(Instances)   - 
Method in class weka.core.neighboursearch. BallTree 
 Builds the BallTree based on the given set of instances.
   setInstances(Instances)   - 
Method in class weka.core.neighboursearch.balltrees. BallSplitter 
 Sets the training instances on which the tree is 
 (or is to be) built.
   setInstances(Instances)   - 
Method in class weka.core.neighboursearch.balltrees. BallTreeConstructor 
 Sets the instances on which the tree is to be built.
   setInstances(Instances)   - 
Method in class weka.core.neighboursearch.balltrees. MiddleOutConstructor 
 Sets the instances on which the tree is to be built.
   setInstances(Instances)   - 
Method in class weka.core.neighboursearch. CoverTree 
 Builds the Cover Tree on the given set of instances.
   setInstances(Instances)   - 
Method in class weka.core.neighboursearch. KDTree 
 Builds the KDTree on the given set of instances.
   setInstances(Instances)   - 
Method in class weka.core.neighboursearch.kdtrees. KDTreeNodeSplitter 
 Sets the training instances on which the tree is (or is 
 to be) built.
   setInstances(Instances)   - 
Method in class weka.core.neighboursearch. LinearNNSearch 
 Sets the instances comprising the current neighbourhood.
   setInstances(Instances)   - 
Method in class weka.core.neighboursearch. NearestNeighbourSearch 
 Sets the instances.
   setInstances(Instances)   - 
Method in class weka.core. NormalizableDistance 
 Sets the instances.
   setInstances(Instances)   - 
Method in class weka.core.xml. XMLInstances 
 builds up the XML structure based on the given data
   setInstances(Instances)   - 
Method in class weka.experiment. AveragingResultProducer 
 Sets the dataset that results will be obtained for.
   setInstances(Instances)   - 
Method in class weka.experiment. CrossValidationResultProducer 
 Sets the dataset that results will be obtained for.
   setInstances(Instances)   - 
Method in class weka.experiment. DatabaseResultProducer 
 Sets the dataset that results will be obtained for.
   setInstances(Instances)   - 
Method in class weka.experiment. LearningRateResultProducer 
 Sets the dataset that results will be obtained for.
   setInstances(Instances)   - 
Method in class weka.experiment. PairedTTester 
 Set the value of Instances.
   setInstances(Instances)   - 
Method in class weka.experiment. RandomSplitResultProducer 
 Sets the dataset that results will be obtained for.
   setInstances(Instances)   - 
Method in interface weka.experiment. ResultProducer 
 Sets the dataset that results will be obtained for.
   setInstances(Instances)   - 
Method in interface weka.experiment. Tester 
 Set the value of Instances.
   setInstances(Instances)   - 
Method in class weka.gui.arffviewer. ArffPanel 
 displays the given instances, i.e.
   setInstances(Instances)   - 
Method in class weka.gui.arffviewer. ArffSortedTableModel 
 sets the data
   setInstances(Instances)   - 
Method in class weka.gui.arffviewer. ArffTableModel 
 sets the data
   setInstances(Instances)   - 
Method in class weka.gui. AttributeListPanel 
 Sets the instances who's attribute names will be displayed.
   setInstances(Instances)   - 
Method in class weka.gui. AttributeSelectionPanel 
 Sets the instances who's attribute names will be displayed.
   setInstances(Instances)   - 
Method in class weka.gui. AttributeSummaryPanel 
 Tells the panel to use a new set of instances.
   setInstances(Instances)   - 
Method in class weka.gui. AttributeVisualizationPanel 
 Sets the instances for use
   setInstances(Instances)   - 
Method in class weka.gui.beans. AttributeSummarizer 
 Set instances for this bean.
   setInstances(Instances)   - 
Method in class weka.gui.beans. DataVisualizer 
 Set instances for this bean.
   setInstances(Instances)   - 
Method in class weka.gui.beans. ScatterPlotMatrix 
 Set instances for this bean.
   setInstances(Instances)   - 
Method in class weka.gui.boundaryvisualizer. BoundaryVisualizer 
 Set the training instances
   setInstances(Instances)   - 
Method in class weka.gui.boundaryvisualizer. RemoteBoundaryVisualizerSubTask 
 Set the training data
   setInstances(Instances)   - 
Method in class weka.gui.experiment. ResultsPanel 
 Sets up the panel with a new set of instances, attempting
 to guess the correct settings for various columns.
   setInstances(Instances)   - 
Method in class weka.gui.explorer. AssociationsPanel 
 Tells the panel to use a new set of instances.
   setInstances(Instances)   - 
Method in class weka.gui.explorer. AttributeSelectionPanel 
 Tells the panel to use a new set of instances.
   setInstances(Instances)   - 
Method in class weka.gui.explorer. ClassifierPanel 
 Tells the panel to use a new set of instances.
   setInstances(Instances)   - 
Method in class weka.gui.explorer. ClustererPanel 
 Tells the panel to use a new set of instances.
   setInstances(Instances)   - 
Method in interface weka.gui.explorer. Explorer.ExplorerPanel 
 Tells the panel to use a new set of instances.
   setInstances(Instances)   - 
Method in class weka.gui.explorer. PreprocessPanel 
 Tells the panel to use a new base set of instances.
   setInstances(Instances)   - 
Method in class weka.gui. InstancesSummaryPanel 
 Tells the panel to use a new set of instances.
   setInstances(Instances)   - 
Method in class weka.gui. SetInstancesPanel 
 Updates the set of instances that is currently held by the panel
   setInstances(Instances)   - 
Method in class weka.gui. ViewerDialog 
 sets the instances to display
   setInstances(Instances)   - 
Method in class weka.gui.visualize. AttributePanel 
 This sets the instances to be drawn into the attribute panel
   setInstances(Instances)   - 
Method in class weka.gui.visualize. ClassPanel 
 Set the instances.
   setInstances(Instances)   - 
Method in class weka.gui.visualize. MatrixPanel 
 This method changes the Instances object of this class to a new one.
   setInstances(Instances)   - 
Method in class weka.gui.visualize. Plot2D 
 Sets the master plot from a set of instances
   setInstances(Instances)   - 
Method in class weka.gui.visualize. VisualizePanel 
 Tells the panel to use a new set of instances.
   setInstancesFromDB(InstanceQuery)   - 
Method in class weka.gui.explorer. PreprocessPanel 
 Loads instances from a database
   setInstancesFromDBQ(String, String, String, String)   - 
Method in class weka.gui.explorer. PreprocessPanel 
 Loads instances from an SQL query the user provided with the
 SqlViewerDialog, then loads the instances in a background process.
   setInstancesFromFile(AbstractFileLoader)   - 
Method in class weka.gui.explorer. PreprocessPanel 
 Loads results from a set of instances retrieved with the supplied loader.
   setInstancesFromFileQ()   - 
Method in class weka.gui.boundaryvisualizer. BoundaryVisualizer 
 Queries the user for a file to load instances from, then loads the
 instances in a background process.
   setInstancesFromFileQ()   - 
Method in class weka.gui.explorer. PreprocessPanel 
 Queries the user for a file to load instances from, then loads the
 instances in a background process.
   setInstancesFromFileQ()   - 
Method in class weka.gui. SetInstancesPanel 
 Queries the user for a file to load instances from, then loads the
 instances in a background process.
   setInstancesFromURL(URL)   - 
Method in class weka.gui.explorer. PreprocessPanel 
 Loads instances from a URL.
   setInstancesFromURLQ()   - 
Method in class weka.gui.explorer. PreprocessPanel 
 Queries the user for a URL to load instances from, then loads the
 instances in a background process.
   setInstancesFromURLQ()   - 
Method in class weka.gui. SetInstancesPanel 
 Queries the user for a URL to load instances from, then loads the
 instances in a background process.
   setInstancesIndices(String)   - 
Method in class weka.filters.unsupervised.instance. RemoveRange 
 Sets the ranges of instances to be selected.
   SetInstancesPanel   - Class in  weka.gui  A panel that displays an instance summary for a set of instances and
 lets the user open a set of instances from either a file or URL.   SetInstancesPanel()   - 
Constructor for class weka.gui. SetInstancesPanel 
 Create the panel.
   setInsts(int[], boolean)   - 
Method in class weka.classifiers.lazy. LBR.Indexes 
 Changes the boolean value at the specified index in the InstIndexes array
   setInterAnchorDistances(Vector, MiddleOutConstructor.TempNode, Vector)   - 
Method in class weka.core.neighboursearch.balltrees. MiddleOutConstructor 
 Sets the distances of a supplied new
 anchor to all the rest of the 
 previous anchor points.
   setInternalCacheSize(int)   - 
Method in class weka.classifiers.functions.supportVector. StringKernel 
 sets the size of the internal cache for intermediate results.
   setInternals(boolean)   - 
Method in class weka.classifiers.bayes. WAODE 
 Sets whether internals about classifier are printed via toString().
   setInvert(boolean)   - 
Method in class weka.core. Range 
 Sets whether the range sense is inverted, i.e.
   setInvert(boolean)   - 
Method in class weka.filters.unsupervised.instance. RemoveMisclassified 
 Set whether selection is inverted.
   setInvertSelection(boolean)   - 
Method in interface weka.core. DistanceFunction 
 Sets whether the matching sense of attribute indices is inverted or not.
   setInvertSelection(boolean)   - 
Method in class weka.core. NormalizableDistance 
 Sets whether the matching sense of attribute indices is inverted or not.
   setInvertSelection(boolean)   - 
Method in class weka.filters.supervised.attribute. Discretize 
 Sets whether selected columns should be removed or kept.
   setInvertSelection(boolean)   - 
Method in class weka.filters.supervised.instance. Resample 
 Sets whether the selection is inverted (only if instances are drawn WIHTOUT 
 replacement).
   setInvertSelection(boolean)   - 
Method in class weka.filters.supervised.instance. StratifiedRemoveFolds 
 Sets if selection is to be inverted.
   setInvertSelection(boolean)   - 
Method in class weka.filters.unsupervised.attribute. AbstractTimeSeries 
 Set whether selected columns should be removed or kept.
   setInvertSelection(boolean)   - 
Method in class weka.filters.unsupervised.attribute. Copy 
 Set whether selected columns should be removed or kept.
   setInvertSelection(boolean)   - 
Method in class weka.filters.unsupervised.attribute. Discretize 
 Sets whether selected columns should be removed or kept.
   setInvertSelection(boolean)   - 
Method in class weka.filters.unsupervised.attribute. MathExpression 
 Set whether selected columns should be select or unselect.
   setInvertSelection(boolean)   - 
Method in class weka.filters.unsupervised.attribute. NominalToBinary 
 Sets whether selected columns should be removed or kept.
   setInvertSelection(boolean)   - 
Method in class weka.filters.unsupervised.attribute. NumericCleaner 
 Sets whether the selection of the indices is inverted or not
   setInvertSelection(boolean)   - 
Method in class weka.filters.unsupervised.attribute. NumericToNominal 
 Sets whether selected columns should be worked on or all the others apart
 from these.
   setInvertSelection(boolean)   - 
Method in class weka.filters.unsupervised.attribute. NumericTransform 
 Set whether selected columns should be transformed or not.
   setInvertSelection(boolean)   - 
Method in class weka.filters.unsupervised.attribute. RELAGGS 
 Sets whether selected columns should be processed or skipped.
   setInvertSelection(boolean)   - 
Method in class weka.filters.unsupervised.attribute. Remove 
 Set whether selected columns should be removed or kept.
   setInvertSelection(boolean)   - 
Method in class weka.filters.unsupervised.attribute. RemoveType 
 Set whether selected columns should be removed or kept.
   setInvertSelection(boolean)   - 
Method in class weka.filters.unsupervised.attribute. StringToWordVector 
 Sets whether selected columns should be processed or skipped.
   setInvertSelection(boolean)   - 
Method in class weka.filters.unsupervised.instance. RemoveFolds 
 Sets if selection is to be inverted.
   setInvertSelection(boolean)   - 
Method in class weka.filters.unsupervised.instance. RemoveFrequentValues 
 Set whether selected values should be removed or kept.
   setInvertSelection(boolean)   - 
Method in class weka.filters.unsupervised.instance. RemovePercentage 
 Sets if selection is to be inverted.
   setInvertSelection(boolean)   - 
Method in class weka.filters.unsupervised.instance. RemoveRange 
 Sets if selection is to be inverted.
   setInvertSelection(boolean)   - 
Method in class weka.filters.unsupervised.instance. RemoveWithValues 
 Set whether selected values should be removed or kept.
   setInvertSelection(boolean)   - 
Method in class weka.filters.unsupervised.instance. Resample 
 Sets whether the selection is inverted (only if instances are drawn WIHTOUT 
 replacement).
   setItem(int[])   - 
Method in class weka.associations. ItemSet 
 Sets an item sets
   setItemAt(int, int)   - 
Method in class weka.associations. ItemSet 
 Sets the index of an attribute value
   setJitter(int)   - 
Method in class weka.gui.visualize. Plot2D 
 Set level of jitter and repaint the plot using the new jitter value
   setKDTree(KDTree)   - 
Method in class weka.clusterers. XMeans 
 Sets the KDTree class.
   setKernel(Kernel)   - 
Method in class weka.classifiers.functions. GaussianProcesses 
 Sets the kernel to use.
   setKernel(Kernel)   - 
Method in class weka.classifiers.functions. SMO.BinarySMO 
 sets the kernel to use
   setKernel(Kernel)   - 
Method in class weka.classifiers.functions. SMO 
 sets the kernel to use
   setKernel(Kernel)   - 
Method in class weka.classifiers.functions. SMOreg 
 sets the kernel to use
   setKernel(Kernel)   - 
Method in class weka.classifiers.functions.supportVector. CheckKernel 
 Set the lernel to test.
   setKernel(Kernel)   - 
Method in class weka.classifiers.mi. MISMO 
 Sets the kernel to use.
   setKernel(Kernel)   - 
Method in class weka.classifiers.mi. MISVM 
 Sets the kernel to use.
   setKernel(Kernel)   - 
Method in class weka.filters.unsupervised.attribute. KernelFilter 
 Sets the kernel to use.
   setKernelBandwidth(int)   - 
Method in class weka.gui.boundaryvisualizer. KDDataGenerator 
 Set the kernel bandwidth (number of nearest neighbours to cover)
   setKernelFactorExpression(String)   - 
Method in class weka.filters.unsupervised.attribute. KernelFilter 
 Sets the expression for the kernel.
   setKernelMatrixFile(File)   - 
Method in class weka.classifiers.functions.supportVector. PrecomputedKernelMatrixKernel 
 Sets the file holding the kernel matrix
   setKernelType(SelectedTag)   - 
Method in class weka.classifiers.functions. LibSVM 
 Sets type of kernel function (default KERNELTYPE_RBF)
   setKey(String)   - 
Method in interface weka.clusterers.forOPTICSAndDBScan.DataObjects. DataObject 
 Sets the key for this DataObject
   setKey(String)   - 
Method in class weka.clusterers.forOPTICSAndDBScan.DataObjects. EuclidianDataObject 
 Sets the key for this DataObject
   setKey(String)   - 
Method in class weka.clusterers.forOPTICSAndDBScan.DataObjects. ManhattanDataObject 
 Sets the key for this DataObject
   setKeyFieldName(String)   - 
Method in class weka.experiment. AveragingResultProducer 
 Set the value of KeyFieldName.
   setKeys(String)   - 
Method in class weka.core.converters. DatabaseLoader 
 Sets the key columns of a database table
   setKeywords(String)   - 
Method in class weka.experiment. DatabaseUtils 
 Sets the keywords (comma-separated list) to use.
   setKeywordsMaskChar(String)   - 
Method in class weka.experiment. DatabaseUtils 
 Sets the mask character to append to table or attribute names that
 are a reserved keyword.
   setKNN(int)   - 
Method in class weka.classifiers.lazy. IBk 
 Set the number of neighbours the learner is to use.
   setKNN(int)   - 
Method in class weka.classifiers.lazy. LWL 
 Sets the number of neighbours used for kernel bandwidth setting.
   setKValue(int)   - 
Method in class weka.classifiers.trees. RandomTree 
 Set the value of K.
   setLabels(String)   - 
Method in class weka.filters.unsupervised.attribute. AddValues 
 Sets the comma-separated list of labels.
   setLambda(double)   - 
Method in class weka.classifiers.functions. SPegasos 
 Set the value of lambda to use
   setLambda(double)   - 
Method in class weka.classifiers.functions.supportVector. StringKernel 
 Sets the lambda constant used in the string kernel
   setLearningRate(double)   - 
Method in class weka.classifiers.functions. MultilayerPerceptron 
 The learning rate can be set using this command.
   setLegendText(Vector)   - 
Method in class weka.gui.beans. ChartEvent 
 Set the legend text vector
   setLikelihoodThreshold(double)   - 
Method in class weka.classifiers.meta. LogitBoost 
 Set the value of Precision.
   setLinkType(SelectedTag)   - 
Method in class weka.clusterers. HierarchicalClusterer 
  
   setListData(Object[])   - 
Method in class weka.gui. CheckBoxList 
 Constructs a CheckBoxListModel from an array of objects and then applies 
 setModel to it.
   setListData(Vector)   - 
Method in class weka.gui. CheckBoxList 
 Constructs a CheckBoxListModel from a Vector and then applies setModel 
 to it.
   setLNorm(double)   - 
Method in class weka.filters.unsupervised.instance. Normalize 
 Set the L-norm to used
   setLoader(Loader)   - 
Method in class weka.gui.beans. Loader 
 Set the loader to use
   setLocallyPredictive(boolean)   - 
Method in class weka.attributeSelection. CfsSubsetEval 
 Include locally predictive attributes
   setLocationProbs(int, double[])   - 
Method in class weka.gui.boundaryvisualizer. RemoteResult 
 Store the classifier's distribution for a particular pixel in the
 visualization
   setLog(Logger)   - 
Method in class weka.classifiers.pmml.consumer. PMMLClassifier 
 Set a logger to use.
   setLog(Debug.Log)   - 
Method in class weka.core. Debug.Random 
 the log to use, if it is null then stdout is used
   setLog(Logger)   - 
Method in interface weka.core.pmml. PMMLModel 
 Set a logger to use.
   setLog(Logger)   - 
Method in class weka.gui.beans. AbstractDataSink 
 Set a log for this bean
   setLog(Logger)   - 
Method in class weka.gui.beans. AbstractEvaluator 
 Set a logger
   setLog(Logger)   - 
Method in class weka.gui.beans. AbstractTestSetProducer 
 Set a logger
   setLog(Logger)   - 
Method in class weka.gui.beans. AbstractTrainAndTestSetProducer 
 Set a log for this bean
   setLog(Logger)   - 
Method in class weka.gui.beans. AbstractTrainingSetProducer 
 Set a logger
   setLog(Logger)   - 
Method in class weka.gui.beans. Associator 
 Set a logger
   setLog(Logger)   - 
Method in interface weka.gui.beans. BeanCommon 
 Set a logger
   setLog(Logger)   - 
Method in class weka.gui.beans. ClassAssigner 
  
   setLog(Logger)   - 
Method in class weka.gui.beans. Classifier 
 Set a logger
   setLog(Logger)   - 
Method in class weka.gui.beans. ClassValuePicker 
  
   setLog(Logger)   - 
Method in class weka.gui.beans. Clusterer 
 Set a logger
   setLog(Logger)   - 
Method in class weka.gui.beans. CostBenefitAnalysis 
 Set a logger
   setLog(Logger)   - 
Method in class weka.gui.beans. Filter 
 Set a logger
   setLog(Logger)   - 
Method in class weka.gui.beans. FlowRunner 
  
   setLog(Logger)   - 
Method in class weka.gui.beans. InstanceStreamToBatchMaker 
 Set a logger
   setLog(Logger)   - 
Method in class weka.gui.beans. Loader 
 Set a logger
   setLog(Logger)   - 
Method in interface weka.gui.beans. LogWriter 
 Set a logger
   setLog(Logger)   - 
Method in class weka.gui.beans. MetaBean 
 Set a logger
   setLog(Logger)   - 
Method in class weka.gui.beans. PredictionAppender 
 Set a logger
   setLog(Logger)   - 
Method in class weka.gui.beans. SerializedModelSaver 
 Set a log for this bean.
   setLog(Logger)   - 
Method in class weka.gui.beans. StripChart 
 Set a logger
   setLog(Logger)   - 
Method in class weka.gui.beans. TextViewer 
 Set a logger
   setLog(Logger)   - 
Method in class weka.gui.explorer. AssociationsPanel 
 Sets the Logger to receive informational messages
   setLog(Logger)   - 
Method in class weka.gui.explorer. AttributeSelectionPanel 
 Sets the Logger to receive informational messages
   setLog(Logger)   - 
Method in class weka.gui.explorer. ClassifierPanel 
 Sets the Logger to receive informational messages
   setLog(Logger)   - 
Method in class weka.gui.explorer. ClustererPanel 
 Sets the Logger to receive informational messages
   setLog(Logger)   - 
Method in class weka.gui.explorer. DataGeneratorPanel 
 Sets the Logger to receive informational messages
   setLog(Logger)   - 
Method in interface weka.gui.explorer. Explorer.LogHandler 
 Sets the Logger to receive informational messages
   setLog(Logger)   - 
Method in class weka.gui.explorer. PreprocessPanel 
 Sets the Logger to receive informational messages
   setLog(Logger)   - 
Method in class weka.gui.visualize. VisualizePanel 
 Sets the Logger to receive informational messages
   setLogFile(File)   - 
Method in class weka.classifiers.meta. GridSearch 
 Sets the log file to use.
   setLookAndFeel(String)   - 
Static method in class weka.gui. LookAndFeel 
 sets the look and feel to the specified class
   setLookAndFeel()   - 
Static method in class weka.gui. LookAndFeel 
 sets the look and feel to the one in the props-file or if not set the 
 default one of the system
   setLookupCacheSize(int)   - 
Method in class weka.attributeSelection. BestFirst 
 Set the maximum size of the evaluated subset cache (hashtable).
   setLookupCacheSize(int)   - 
Method in class weka.attributeSelection. LinearForwardSelection 
 Set the maximum size of the evaluated subset cache (hashtable).
   setLookupCacheSize(int)   - 
Method in class weka.attributeSelection. SubsetSizeForwardSelection 
 Set the maximum size of the evaluated subset cache (hashtable).
   setLoss(double)   - 
Method in class weka.classifiers.functions. LibSVM 
 Sets the epsilon in loss function of epsilon-SVR (default 0.1)
   setLossFunction(SelectedTag)   - 
Method in class weka.classifiers.functions. SPegasos 
 Set the loss function to use.
   setLowerBoundMinSupport(double)   - 
Method in class weka.associations. Apriori 
 Set the value of lowerBoundMinSupport.
   setLowerBoundMinSupport(double)   - 
Method in class weka.associations. FPGrowth 
 Set the value of lowerBoundMinSupport.
   setLowerCaseTokens(boolean)   - 
Method in class weka.filters.unsupervised.attribute. StringToWordVector 
 Sets whether if the tokens are to be downcased or not.
   setLowerSize(int)   - 
Method in class weka.experiment. LearningRateResultProducer 
 Set the value of LowerSize.
   setMajorityClass(boolean)   - 
Method in class weka.classifiers.rules. Ridor 
  
   setMakeBinary(boolean)   - 
Method in class weka.filters.supervised.attribute. Discretize 
 Sets whether binary attributes should be made for discretized ones.
   setMakeBinary(boolean)   - 
Method in class weka.filters.unsupervised.attribute. Discretize 
 Sets whether binary attributes should be made for discretized ones.
   setManualThresholdValue(double)   - 
Method in class weka.classifiers.meta. ThresholdSelector 
 Sets the value for a manual threshold.
   setMargin(int, double[])   - 
Method in class weka.classifiers.bayes.net. EditableBayesNet 
 set marginal distibution for a node
   setMarkovBlanketClassifier(boolean)   - 
Method in class weka.classifiers.bayes.net.search.global. GlobalScoreSearchAlgorithm 
  
   setMarkovBlanketClassifier(boolean)   - 
Method in class weka.classifiers.bayes.net.search.local. LocalScoreSearchAlgorithm 
  
   setMasterPlot(PlotData2D)   - 
Method in class weka.gui.visualize. Plot2D 
 Set the master plot.
   setMasterPlot(PlotData2D)   - 
Method in class weka.gui.visualize. VisualizePanel 
 Set the master plot for the visualize panel
   setMatchMissingValues(boolean)   - 
Method in class weka.filters.unsupervised.instance. RemoveWithValues 
 Sets whether missing values are counted as a match.
   setMatrix(int, int, int, int, double)   - 
Method in class weka.classifiers.functions.pace. PaceMatrix 
 Set the submatrix A[i0:i1][j0:j1] with a same value
   setMatrix(int, int, int, DoubleVector)   - 
Method in class weka.classifiers.functions.pace. PaceMatrix 
 Set the submatrix A[i0:i1][j] with the values stored in a
  DoubleVector
   setMatrix(double[], boolean)   - 
Method in class weka.classifiers.functions.pace. PaceMatrix 
 Set the whole matrix from a 1-D array
   setMatrix(int, int, int, int, Matrix)   - 
Method in class weka.core.matrix. Matrix 
 Set a submatrix.
   setMatrix(int[], int[], Matrix)   - 
Method in class weka.core.matrix. Matrix 
 Set a submatrix.
   setMatrix(int[], int, int, Matrix)   - 
Method in class weka.core.matrix. Matrix 
 Set a submatrix.
   setMatrix(int, int, int[], Matrix)   - 
Method in class weka.core.matrix. Matrix 
 Set a submatrix.
   setMax(double)   - 
Method in class weka.gui.beans. ChartEvent 
 Set the max y value
   setMaxBoostingIterations(int)   - 
Method in class weka.classifiers.functions. SimpleLogistic 
 Set the value of maxBoostingIterations.
   setMaxCardinality(int)   - 
Method in class weka.classifiers.bayes.net.search.ci. ICSSearchAlgorithm 
 sets the cardinality
   setMaxCardinality(int)   - 
Method in class weka.filters.unsupervised.attribute. RELAGGS 
 Sets the maximum number of values allowed for nominal attributes, before
 they're skipped.
   setMaxChunkSize(int)   - 
Method in class weka.classifiers.meta. RacedIncrementalLogitBoost 
 Set the maximum chunk size
   setMaxCount(double)   - 
Method in class weka.filters.supervised.instance. SpreadSubsample 
 Sets the value for the max count
   setMaxDefault(double)   - 
Method in class weka.filters.unsupervised.attribute. NumericCleaner 
 Set the naximum default.
   setMaxDepth(int)   - 
Method in class weka.classifiers.trees. RandomForest 
 Set the maximum depth of the tree, 0 for unlimited.
   setMaxDepth(int)   - 
Method in class weka.classifiers.trees. RandomTree 
 Set the maximum depth of the tree, 0 for unlimited.
   setMaxDepth(int)   - 
Method in class weka.classifiers.trees. REPTree 
 Set the value of MaxDepth.
   setMaxGenerations(int)   - 
Method in class weka.attributeSelection. GeneticSearch 
 set the number of generations to evaluate
   setMaxGridExtensions(int)   - 
Method in class weka.classifiers.meta. GridSearch 
 Sets the maximum number of grid extensions, -1 for unlimited.
   setMaxGroup(int)   - 
Method in class weka.classifiers.meta. RotationForest 
 Sets the maximum size of a group.
   setMaximumAttributeNames(int)   - 
Method in class weka.attributeSelection. LatentSemanticAnalysis 
 Sets maximum number of attributes to include in
 transformed attribute names.
   setMaximumAttributeNames(int)   - 
Method in class weka.attributeSelection. PrincipalComponents 
 Sets maximum number of attributes to include in
 transformed attribute names.
   setMaximumAttributeNames(int)   - 
Method in class weka.filters.unsupervised.attribute. PrincipalComponents 
 Sets maximum number of attributes to include in
 transformed attribute names.
   setMaximumAttributes(int)   - 
Method in class weka.filters.unsupervised.attribute. PrincipalComponents 
 Sets maximum number of PC attributes to retain.
   setMaximumVariancePercentageAllowed(double)   - 
Method in class weka.filters.unsupervised.attribute. RemoveUseless 
 Sets the maximum variance attributes are allowed to have before they are
 deleted by the filter.
   setMaxInstancesInLeaf(int)   - 
Method in class weka.core.neighboursearch.balltrees. BallTreeConstructor 
 Sets the maximum number of instances allowed in a leaf.
   setMaxInstancesInLeaf(int)   - 
Method in class weka.core.neighboursearch.balltrees. MiddleOutConstructor 
 Sets the maximum number of instances allowed in a leaf.
   setMaxInstInLeaf(int)   - 
Method in class weka.core.neighboursearch. KDTree 
 Sets the maximum number of instances in a leaf.
   setMaxInstNum(int)   - 
Method in class weka.datagenerators.clusterers. BIRCHCluster 
 Sets the upper boundary for instances per cluster.
   setMaxInstNum(int)   - 
Method in class weka.datagenerators.clusterers. SubspaceClusterDefinition 
 Sets the upper boundary for instances per cluster.
   setMaxIteration(int)   - 
Method in class weka.core. Optimization 
 Set the maximal number of iterations in searching (Default 200)
   setMaxIterations(int)   - 
Method in class weka.classifiers.bayes. BayesianLogisticRegression 
 Set the maximum number of iterations to perform
   setMaxIterations(int)   - 
Method in class weka.classifiers.mi. MIBoost 
 Set the maximum number of boost iterations
   setMaxIterations(int)   - 
Method in class weka.classifiers.mi. MISVM 
 Sets the maximum number of iterations.
   setMaxIterations(int)   - 
Method in class weka.classifiers.trees.lmt. LogisticBase 
 Sets the parameter "maxIterations".
   setMaxIterations(int)   - 
Method in class weka.clusterers. EM 
 Set the maximum number of iterations to perform
   setMaxIterations(int)   - 
Method in class weka.clusterers. sIB 
 Set the max number of iterations
   setMaxIterations(int)   - 
Method in class weka.clusterers. SimpleKMeans 
 set the maximum number of iterations to be executed
   setMaxIterations(int)   - 
Method in class weka.clusterers. XMeans 
 Sets the maximum number of iterations to perform.
   setMaxIterations(int)   - 
Method in class weka.filters.unsupervised.instance. RemoveMisclassified 
 Sets the maximum number of cleansing iterations to perform
 - < 1 means go until fully cleansed
   setMaxIts(int)   - 
Method in class weka.classifiers.functions. Logistic 
 Set the value of MaxIts.
   setMaxIts(int)   - 
Method in class weka.classifiers.functions. RBFNetwork 
 Set the value of MaxIts.
   setMaxK(int)   - 
Method in class weka.classifiers.functions. VotedPerceptron 
 Set the value of maxK.
   setMaxKMeans(int)   - 
Method in class weka.clusterers. XMeans 
 Set the maximum number of iterations to perform in KMeans.
   setMaxKMeansForChildren(int)   - 
Method in class weka.clusterers. XMeans 
 Sets the maximum number of iterations KMeans that is performed 
 on the child centers.
   setMaxNrOfParents(int)   - 
Method in class weka.classifiers.bayes.net.search.global. HillClimber 
 Sets the max number of parents
   setMaxNrOfParents(int)   - 
Method in class weka.classifiers.bayes.net.search.global. K2 
 Sets the max number of parents
   setMaxNrOfParents(int)   - 
Method in class weka.classifiers.bayes.net.search.local. HillClimber 
 Sets the max number of parents
   setMaxNrOfParents(int)   - 
Method in class weka.classifiers.bayes.net.search.local. K2 
 Sets the max number of parents
   setMaxNrOfParents(int)   - 
Method in class weka.classifiers.bayes.net.search.local. LAGDHillClimber 
 Sets the max number of parents
   setMaxNumberOfItems(int)   - 
Method in class weka.associations. FPGrowth 
 Set the maximum number of items to include in large items sets.
   setMaxNumClusters(int)   - 
Method in class weka.clusterers. XMeans 
 Sets the maximum number of clusters to generate.
   setMaxPlots(int)   - 
Method in class weka.gui.beans. AttributeSummarizer 
 Set the maximum number of plots to display
   setMaxRadius(double)   - 
Method in class weka.datagenerators.clusterers. BIRCHCluster 
 Sets the upper boundary for the radiuses of the clusters.
   setMaxRange(double)   - 
Method in class weka.datagenerators.classifiers.regression. MexicanHat 
 Sets the upper boundary for the range of x
   setMaxRelativeLeafRadius(double)   - 
Method in class weka.core.neighboursearch.balltrees. BallTreeConstructor 
 Sets the maximum relative radius, allowed for a leaf node.
   setMaxRows(int)   - 
Method in class weka.gui.sql. QueryPanel 
 sets the maximum number of rows to display.
   setMaxRuleSize(int)   - 
Method in class weka.datagenerators.classifiers.classification. RDG1 
 Sets the maximum number of tests in rules.
   setMaxSubsequenceLength(int)   - 
Method in class weka.classifiers.functions.supportVector. StringKernel 
 Sets the maximum length of the subsequence.
   setMaxThreshold(double)   - 
Method in class weka.filters.unsupervised.attribute. NumericCleaner 
 Set the maximum threshold.
   setMDLTheoryWeight(double)   - 
Method in class weka.classifiers.rules. RuleStats 
 Set the weight of theory in MDL calcualtion
   setMean(int, int, double)   - 
Method in class weka.experiment. ResultMatrix 
 sets the mean at the given position (if the position is valid)
   setMeanPrec(int)   - 
Method in class weka.experiment. ResultMatrix 
 sets the precision for the means
   setMeanPrec(int)   - 
Method in class weka.gui.experiment. OutputFormatDialog 
 Sets the precision of the mean output.
   setMeanSquared(boolean)   - 
Method in class weka.classifiers.lazy. IBk 
 Sets whether the mean squared error is used rather than mean
 absolute error when doing cross-validation.
   setMeanStddev(String)   - 
Method in class weka.datagenerators.clusterers. SubspaceClusterDefinition 
 Sets mean and standarddeviation.
   setMeanWidth(int)   - 
Method in class weka.experiment. ResultMatrix 
 sets the width for the mean (0 = optimal)
   setMeasure(SelectedTag)   - 
Method in class weka.classifiers.meta. ThresholdSelector 
 set measure used for determining threshold
   setMeasurePerformance(boolean)   - 
Method in class weka.core.neighboursearch. BallTree 
 Sets whether to calculate the performance statistics or not.
   setMeasurePerformance(boolean)   - 
Method in class weka.core.neighboursearch. KDTree 
 Sets whether to calculate the performance statistics or not.
   setMeasurePerformance(boolean)   - 
Method in class weka.core.neighboursearch. NearestNeighbourSearch 
 Sets whether to calculate the performance statistics or not.
   setMestWeight(double)   - 
Method in class weka.classifiers.bayes. AODEsr 
 Sets the weight for m-estimate
   setMetaClassifier(Classifier)   - 
Method in class weka.classifiers.meta. Stacking 
 Adds meta classifier
   setMethod(NeuralMethod)   - 
Method in class weka.classifiers.functions.neural. NeuralNode 
 Set how this node should operate (note that the neural method has no
 internal state, so the same object can be used by any number of nodes.
   setMethod(SelectedTag)   - 
Method in class weka.classifiers.meta. MultiClassClassifier 
 Sets the method used.
   setMethod(SelectedTag)   - 
Method in class weka.classifiers.mi. MIWrapper 
 Set the method used in testing.
   setMethodName(String)   - 
Method in class weka.filters.unsupervised.attribute. NumericTransform 
 Set the transformation method.
   setMetricType(SelectedTag)   - 
Method in class weka.associations. Apriori 
 Set the metric type for ranking rules
   setMetricType(SelectedTag)   - 
Method in class weka.associations. FPGrowth 
 Set the metric type to use.
   setMin(double)   - 
Method in class weka.gui.beans. ChartEvent 
 Set the min y value
   setMinBoxRelWidth(double)   - 
Method in class weka.core.neighboursearch. KDTree 
 Sets the minimum relative box width.
   setMinBucketSize(int)   - 
Method in class weka.classifiers.rules. OneR 
 Set the value of minBucketSize.
   setMinChange(int)   - 
Method in class weka.clusterers. sIB 
 set the minimum number of changes
   setMinChunkSize(int)   - 
Method in class weka.classifiers.meta. RacedIncrementalLogitBoost 
 Set the minimum chunk size
   setMinDefault(double)   - 
Method in class weka.filters.unsupervised.attribute. NumericCleaner 
 Set the minimum default.
   setMinGroup(int)   - 
Method in class weka.classifiers.meta. RotationForest 
 Sets the minimum size of a group.
   setMinimax(boolean)   - 
Method in class weka.classifiers.mi. MISMO 
 Set if the MIMinimax feature space is to be used.
   setMinimizeExpectedCost(boolean)   - 
Method in class weka.classifiers.meta. CostSensitiveClassifier 
 Set the value of MinimizeExpectedCost.
   setMinimumBucketSize(int)   - 
Method in class weka.attributeSelection. OneRAttributeEval 
 Set the minumum bucket size used by OneR
   setMinimumNumberInstances(int)   - 
Method in class weka.core. Capabilities 
 sets the minimum number of instances that have to be in the dataset
   setMinInstNum(int)   - 
Method in class weka.datagenerators.clusterers. BIRCHCluster 
 Sets the lower boundary for instances per cluster.
   setMinInstNum(int)   - 
Method in class weka.datagenerators.clusterers. SubspaceClusterDefinition 
 Sets the lower boundary for instances per cluster.
   setMinMaxValues()   - 
Method in interface weka.clusterers.forOPTICSAndDBScan.Databases. Database 
 Sets the minimum and maximum values for each attribute in different arrays
 by walking through every DataObject of the database
   setMinMaxValues()   - 
Method in class weka.clusterers.forOPTICSAndDBScan.Databases. SequentialDatabase 
 Sets the minimum and maximum values for each attribute in different arrays
 by walking through every DataObject of the database
   setMinMaxX(double, double)   - 
Method in class weka.gui.boundaryvisualizer. RemoteBoundaryVisualizerSubTask 
 Set the minimum and maximum values of the x axis fixed dimension
   setMinMaxY(double, double)   - 
Method in class weka.gui.boundaryvisualizer. RemoteBoundaryVisualizerSubTask 
 Set the minimum and maximum values of the y axis fixed dimension
   setMinMetric(double)   - 
Method in class weka.associations. Apriori 
 Set the value of minConfidence.
   setMinMetric(double)   - 
Method in class weka.associations. FPGrowth 
 Set the value of minConfidence.
   setMinNo(double)   - 
Method in class weka.classifiers.rules. ConjunctiveRule 
 Sets the minimum total weight of the instances in a rule
   setMinNo(double)   - 
Method in class weka.classifiers.rules. JRip 
 Sets the minimum total weight of the instances in a rule
   setMinNo(double)   - 
Method in class weka.classifiers.rules. Ridor 
  
   setMinNum(double)   - 
Method in class weka.classifiers.trees. RandomTree 
 Set the value of MinNum.
   setMinNum(double)   - 
Method in class weka.classifiers.trees. REPTree 
 Set the value of MinNum.
   setMinNumClusters(int)   - 
Method in class weka.clusterers. XMeans 
 Sets the minimum number of clusters to generate.
   setMinNumInstances(int)   - 
Method in class weka.classifiers.trees. FT 
 Set the value of minNumInstances.
   setMinNumInstances(int)   - 
Method in class weka.classifiers.trees. LMT 
 Set the value of minNumInstances.
   setMinNumInstances(double)   - 
Method in class weka.classifiers.trees.m5. M5Base 
 Set the minimum number of instances to allow at a leaf node
   setMinNumInstances(double)   - 
Method in class weka.classifiers.trees.m5. Rule 
 Set the minumum number of instances to allow at a leaf node
   setMinNumInstances(double)   - 
Method in class weka.classifiers.trees.m5. RuleNode 
 Set the minumum number of instances to allow at a leaf node
   setMinNumObj(int)   - 
Method in class weka.classifiers.rules. PART 
 Set the value of minNumObj.
   setMinNumObj(int)   - 
Method in class weka.classifiers.trees. BFTree 
 Set minimal number of instances at the terminal nodes.
   setMinNumObj(int)   - 
Method in class weka.classifiers.trees. J48 
 Set the value of minNumObj.
   setMinNumObj(int)   - 
Method in class weka.classifiers.trees. J48graft 
 Set the value of minNumObj.
   setMinNumObj(double)   - 
Method in class weka.classifiers.trees. SimpleCart 
 Set minimal number of instances at the terminal nodes.
   setMinPoints(int)   - 
Method in class weka.clusterers. DBScan 
 Sets a new value for minPoints
   setMinPoints(int)   - 
Method in class weka.clusterers. OPTICS 
 Sets a new value for minPoints
   setMinRadius(double)   - 
Method in class weka.datagenerators.clusterers. BIRCHCluster 
 Sets the lower boundary for the radiuses of the clusters.
   setMinRange(double)   - 
Method in class weka.datagenerators.classifiers.regression. MexicanHat 
 Sets the lower boundary for the range of x
   setMinRuleSize(int)   - 
Method in class weka.datagenerators.classifiers.classification. RDG1 
 Sets the minimum number of tests in rules.
   setMinStdDev(double)   - 
Method in class weka.classifiers.functions. RBFNetwork 
 Set the MinStdDev value.
   setMinStdDev(double)   - 
Method in class weka.clusterers. EM 
 Set the minimum value for standard deviation when calculating
 normal density.
   setMinStdDev(double)   - 
Method in class weka.clusterers. MakeDensityBasedClusterer 
 Set the minimum value for standard deviation when calculating
 normal density.
   setMinStdDevPerAtt(double[])   - 
Method in class weka.clusterers. EM 
  
   setMinSupport(double)   - 
Method in class weka.associations. GeneralizedSequentialPatterns 
 Sets the minimum support threshold.
   setMinTermFreq(int)   - 
Method in class weka.filters.unsupervised.attribute. StringToWordVector 
 Set the MinTermFreq value.
   setMinThreshold(double)   - 
Method in class weka.filters.unsupervised.attribute. NumericCleaner 
 Set the minimum threshold.
   setMinVarianceProp(double)   - 
Method in class weka.classifiers.trees. REPTree 
 Set the value of MinVarianceProp.
   setMissing(int)   - 
Method in class weka.core. Instance 
 Sets a specific value to be "missing".
   setMissing(Attribute)   - 
Method in class weka.core. Instance 
 Sets a specific value to be "missing".
   setMissingMerge(boolean)   - 
Method in class weka.attributeSelection. ChiSquaredAttributeEval 
 distribute the counts for missing values across observed values
   setMissingMerge(boolean)   - 
Method in class weka.attributeSelection. GainRatioAttributeEval 
 distribute the counts for missing values across observed values
   setMissingMerge(boolean)   - 
Method in class weka.attributeSelection. InfoGainAttributeEval 
 distribute the counts for missing values across observed values
   setMissingMerge(boolean)   - 
Method in class weka.attributeSelection. SymmetricalUncertAttributeEval 
 distribute the counts for missing values across observed values
   setMissingMode(int)   - 
Method in class weka.classifiers.lazy.kstar. KStarNumericAttribute 
 Set the missing value mode.
   setMissingMode(SelectedTag)   - 
Method in class weka.classifiers.lazy. KStar 
 Sets the method to use for handling missing values.
   setMissingSeparate(boolean)   - 
Method in class weka.attributeSelection. CfsSubsetEval 
 Treat missing as a separate value
   setMissingValue(String)   - 
Method in class weka.core.converters. CSVLoader 
 Sets the placeholder for missing values.
   setMissingValues(SelectedTag)   - 
Method in class weka.associations. Tertius 
 Set the value of missingValues.
   setMixingDistribution(DiscreteFunction)   - 
Method in class weka.classifiers.functions.pace. MixtureDistribution 
 Sets the mixing distribution
   setModel(Classifier)   - 
Method in class weka.classifiers.misc. SerializedClassifier 
 Sets the fully built model to use, if one doesn't want to load a model
 from a file or already deserialized a model from somewhere else.
   setModel(TableModel)   - 
Method in class weka.gui.arffviewer. ArffTable 
 sets the new model
   setModel(ListModel)   - 
Method in class weka.gui. CheckBoxList 
 sets the model - must be an instance of CheckBoxListModel
   setModel(TableModel)   - 
Method in class weka.gui. SortedTableModel 
 sets the model to use
   setModelFile(File)   - 
Method in class weka.classifiers.misc. SerializedClassifier 
 Sets the file containing the serialized model.
   setModelType(SelectedTag)   - 
Method in class weka.classifiers.trees. FT 
 Set the Functional Tree type.
   setModePanel(SetupModePanel)   - 
Method in class weka.gui.experiment. SimpleSetupPanel 
 Sets the panel used to switch between simple and advanced modes.
   setModifyHeader(boolean)   - 
Method in class weka.filters.unsupervised.instance. RemoveFrequentValues 
 Sets whether the header will be modified when selecting on nominal
 attributes.
   setModifyHeader(boolean)   - 
Method in class weka.filters.unsupervised.instance. RemoveWithValues 
 Sets whether the header will be modified when selecting on nominal
 attributes.
   setMomentum(double)   - 
Method in class weka.classifiers.functions. MultilayerPerceptron 
 The momentum can be set using this command.
   setMultiInstance(boolean)   - 
Method in class weka.core. TestInstances 
 sets whether multi-instance data should be generated (with a fixed
 data structure)
   setMultinomialWord(boolean)   - 
Method in class weka.classifiers.bayes. DMNBtext 
 Sets whether use binary text representation
   setMutationProb(double)   - 
Method in class weka.attributeSelection. GeneticSearch 
 set the probability of mutation
   setName(String)   - 
Method in class weka.filters.unsupervised.attribute. AddExpression 
 Set the name for the new attribute.
   setName(String)   - 
Method in class weka.gui.visualize. VisualizePanel 
 Set a name for this plot
   setNearestNeighbors(int)   - 
Method in class weka.filters.supervised.instance. SMOTE 
 Sets the number of nearest neighbors to use.
   setNearestNeighbourSearchAlgorithm(NearestNeighbourSearch)   - 
Method in class weka.classifiers.lazy. IBk 
 Sets the nearestNeighbourSearch algorithm to be used for finding nearest
 neighbour(s).
   setNearestNeighbourSearchAlgorithm(NearestNeighbourSearch)   - 
Method in class weka.classifiers.lazy. LWL 
 Sets the nearestNeighbourSearch algorithm to be used for finding nearest
 neighbour(s).
   setNegation(Literal)   - 
Method in class weka.associations.tertius. Literal 
  
   setNegation(SelectedTag)   - 
Method in class weka.associations. Tertius 
 Set the value of negation.
   setNewToolTip(String)   - 
Method in class weka.clusterers.forOPTICSAndDBScan.OPTICS_GUI. GraphPanel 
 Displays a toolTip for the selected DataObject
   setNGramMaxSize(int)   - 
Method in class weka.core.tokenizers. NGramTokenizer 
 Sets the max size of the Ngram.
   setNGramMinSize(int)   - 
Method in class weka.core.tokenizers. NGramTokenizer 
 Sets the min size of the Ngram.
   setNoClass(boolean)   - 
Method in class weka.core. TestInstances 
 whether to have no class, e.g., for clusterers; otherwise the class
 attribute index is set to last
   setNodeName(int, String)   - 
Method in class weka.classifiers.bayes.net. EditableBayesNet 
 change the name of a node
   setNodesEdges(FastVector, FastVector)   - 
Method in class weka.gui.graphvisualizer. HierarchicalBCEngine 
 Sets the nodes and edges for this LayoutEngine.
   setNodesEdges(FastVector, FastVector)   - 
Method in interface weka.gui.graphvisualizer. LayoutEngine 
 This method sets the nodes and edges vectors of the LayoutEngine
   setNodeSize(int, int)   - 
Method in class weka.gui.graphvisualizer. HierarchicalBCEngine 
 Sets the size of a node.
   setNodeSize(int, int)   - 
Method in interface weka.gui.graphvisualizer. LayoutEngine 
 This method sets the allowed size of the node
   setNodeSplitter(KDTreeNodeSplitter)   - 
Method in class weka.core.neighboursearch. KDTree 
 Sets the splitting method to use to split the nodes of the KDTree.
   setNodeWidthNormalization(boolean)   - 
Method in class weka.core.neighboursearch.kdtrees. KDTreeNodeSplitter 
 Sets whether if a nodes region is normalized 
 or not.
   setNoise(double)   - 
Method in class weka.classifiers.functions. GaussianProcesses 
 Set the level of Gaussian Noise.
   setNoisePercent(double)   - 
Method in class weka.datagenerators.classifiers.classification. LED24 
 Sets the noise percentage.
   setNoiseRate(double)   - 
Method in class weka.datagenerators.classifiers.regression. MexicanHat 
 Sets the gaussian noise rate.
   setNoiseRate(double)   - 
Method in class weka.datagenerators.clusterers. BIRCHCluster 
 Sets the percentage of noise set.
   setNoiseRate(double)   - 
Method in class weka.datagenerators.clusterers. SubspaceCluster 
 Sets the percentage of noise set.
   setNoiseThreshold(double)   - 
Method in class weka.associations. Tertius 
 Set the value of noiseThreshold.
   setNoiseVariance(double)   - 
Method in class weka.datagenerators.classifiers.regression. MexicanHat 
 Sets the noise variance
   setNominalAttributes(String)   - 
Method in class weka.core.converters. CSVLoader 
 Sets the attribute range to be forced to type nominal.
   setNominalCols(Range)   - 
Method in class weka.datagenerators. ClusterGenerator 
 Sets which attributes are nominal.
   setNominalIndices(String)   - 
Method in class weka.datagenerators. ClusterGenerator 
 Sets which attributes are nominal
   setNominalIndices(String)   - 
Method in class weka.filters.unsupervised.instance. RemoveWithValues 
 Set which nominal labels are to be included in the selection.
   setNominalIndicesArr(int[])   - 
Method in class weka.filters.unsupervised.instance. RemoveWithValues 
 Set which values of a nominal attribute are to be used for
 selection.
   setNominalLabels(String)   - 
Method in class weka.filters.unsupervised.attribute. Add 
 Set the labels for nominal attribute creation.
   setNominalToBinaryFilter(boolean)   - 
Method in class weka.classifiers.functions. MultilayerPerceptron 
  
   setNoPruning(boolean)   - 
Method in class weka.classifiers.trees. REPTree 
 Set the value of NoPruning.
   setNoReplacement(boolean)   - 
Method in class weka.filters.supervised.instance. Resample 
 Sets whether instances are drawn with or with out replacement.
   setNoReplacement(boolean)   - 
Method in class weka.filters.unsupervised.instance. Resample 
 Sets whether instances are drawn with or with out replacement.
   setNorm(double)   - 
Method in class weka.filters.unsupervised.instance. Normalize 
 Set the norm of the instances
   setNormalize(boolean)   - 
Method in class weka.attributeSelection. LatentSemanticAnalysis 
 Set whether input data will be normalized.
   setNormalize(boolean)   - 
Method in class weka.classifiers.functions. LibLINEAR 
 whether to normalize input data
   setNormalize(boolean)   - 
Method in class weka.classifiers.functions. LibSVM 
 whether to normalize input data
   setNormalizeAttributes(boolean)   - 
Method in class weka.classifiers.functions. MultilayerPerceptron 
  
   setNormalizeData(boolean)   - 
Method in class weka.classifiers.bayes. BayesianLogisticRegression 
 Set whether to normalize the data or not
   setNormalizeDimWidths(boolean)   - 
Method in class weka.core.neighboursearch.balltrees. MedianOfWidestDimension 
 Should we normalize the widths(ranges) of the dimensions (attributes) 
 before selecting the widest one.
   setNormalizeDocLength(SelectedTag)   - 
Method in class weka.filters.unsupervised.attribute. StringToWordVector 
 Sets whether if the word frequencies for a document (instance) should
  be normalized or not.
   setNormalizeNodeWidth(boolean)   - 
Method in class weka.core.neighboursearch. KDTree 
 Sets the flag for normalizing the widths of a KDTree Node by the width of
 the dimension in the universe.
   setNormalizeNumericClass(boolean)   - 
Method in class weka.classifiers.functions. MultilayerPerceptron 
  
   setNormalizeWordWeights(boolean)   - 
Method in class weka.classifiers.bayes. ComplementNaiveBayes 
 Sets whether if the word weights for each class should be normalized
   setNotCapabilities(Capabilities)   - 
Method in class weka.core. FindWithCapabilities 
 Uses the given "not to have" Capabilities for the search.
   setNotes(String)   - 
Method in class weka.experiment. Experiment 
 Set the user notes.
   setNotes(String)   - 
Method in class weka.experiment. RemoteExperiment 
 Set the user notes.
   setNotificationEnabled(boolean)   - 
Method in class weka.gui.arffviewer. ArffSortedTableModel 
 sets whether the notification of changes is enabled
   setNotificationEnabled(boolean)   - 
Method in class weka.gui.arffviewer. ArffTableModel 
 sets whether the notification of changes is enabled
   setNotUnifyNorm(boolean)   - 
Method in class weka.clusterers. sIB 
 Set whether to normalize instances to unify prior probability 
 before building the clusterer
   setNrOfGoodOperations(int)   - 
Method in class weka.classifiers.bayes.net.search.local. LAGDHillClimber 
 Sets the number of "good operations"
   setNrOfLookAheadSteps(int)   - 
Method in class weka.classifiers.bayes.net.search.local. LAGDHillClimber 
 Sets the number of look-ahead steps
   setNu(double)   - 
Method in class weka.classifiers.functions. LibSVM 
 Sets nu of nu-SVC, one-class SVM, and nu-SVR (default 0.5)
   setNumAllConds(double)   - 
Method in class weka.classifiers.rules. RuleStats 
 Set the number of all conditions that could appear 
 in a rule in this RuleStats object, if the number set
 is smaller than 0 (typically -1), then it calcualtes
 based on the data store
   setNumAntds(int)   - 
Method in class weka.classifiers.rules. ConjunctiveRule 
 Sets the number of antecedants
   setNumArcs(int)   - 
Method in class weka.datagenerators.classifiers.classification. BayesNet 
 Sets the number of arcs for the bayesian net
   setNumAttemptsOfGeneOption(int)   - 
Method in class weka.classifiers.rules. NNge 
 Sets the number of attempts for generalisation.
   setNumAttributes(int)   - 
Method in class weka.datagenerators.classifiers.classification. BayesNet 
 Sets the number of attributes the dataset should have.
   setNumAttributes(int)   - 
Method in class weka.datagenerators.classifiers.classification. RandomRBF 
 Sets the number of attributes the dataset should have.
   setNumAttributes(int)   - 
Method in class weka.datagenerators.classifiers.classification. RDG1 
 Sets the number of attributes the dataset should have.
   setNumAttributes(int)   - 
Method in class weka.datagenerators.clusterers. SubspaceCluster 
 Sets the number of attributes the dataset should have.
   setNumAttributes(int)   - 
Method in class weka.datagenerators. ClusterGenerator 
 Sets the number of attributes the dataset should have.
   setNumAttributes(double)   - 
Method in class weka.filters.unsupervised.attribute. RandomSubset 
 Set the number of attributes.
   setNumberLiterals(int)   - 
Method in class weka.associations. Tertius 
 Set the value of numberLiterals.
   setNumberOfAttributes(int)   - 
Method in class weka.filters.unsupervised.attribute. RandomProjection 
 Sets the number of attributes (dimensions) the data should be reduced to
   setNumberOfGroups(boolean)   - 
Method in class weka.classifiers.meta. RotationForest 
 Set whether minGroup and maxGroup refer to the number of groups or their 
 size
   setNumBins(int)   - 
Method in class weka.classifiers.meta. RegressionByDiscretization 
 Sets the number of bins to divide each selected numeric attribute into
   setNumBoostingIterations(int)   - 
Method in class weka.classifiers.functions. SimpleLogistic 
 Set the value of numBoostingIterations.
   setNumBoostingIterations(int)   - 
Method in class weka.classifiers.trees. FT 
 Set the value of numBoostingIterations.
   setNumBoostingIterations(int)   - 
Method in class weka.classifiers.trees. LMT 
 Set the value of numBoostingIterations.
   setNumCentroids(int)   - 
Method in class weka.datagenerators.classifiers.classification. RandomRBF 
 Sets the number of centroids to use.
   setNumCiters(int)   - 
Method in class weka.classifiers.mi. CitationKNN 
 Sets the number of citers considered to estimate
 the class prediction of tests bags
   setNumClasses(int)   - 
Method in class weka.core. TestInstances 
 sets the number of classes
   setNumClasses(int)   - 
Method in class weka.datagenerators.classifiers.classification. RandomRBF 
 Sets the number of classes the dataset should have.
   setNumClasses(int)   - 
Method in class weka.datagenerators.classifiers.classification. RDG1 
 Sets the number of classes the dataset should have.
   setNumClusters(int)   - 
Method in class weka.classifiers.functions. RBFNetwork 
 Set the number of clusters for K-means to generate.
   setNumClusters(int)   - 
Method in class weka.clusterers. EM 
 Set the number of clusters (-1 to select by CV).
   setNumClusters(int)   - 
Method in class weka.clusterers. FarthestFirst 
 set the number of clusters to generate
   setNumClusters(int)   - 
Method in class weka.clusterers. HierarchicalClusterer 
  
   setNumClusters(int)   - 
Method in class weka.clusterers. MakeDensityBasedClusterer 
 Set the number of clusters to generate.
   setNumClusters(int)   - 
Method in interface weka.clusterers. NumberOfClustersRequestable 
 Set the number of clusters to generate
   setNumClusters(int)   - 
Method in class weka.clusterers. sIB 
 Set the number of clusters
   setNumClusters(int)   - 
Method in class weka.clusterers. SimpleKMeans 
 set the number of clusters to generate
   setNumClusters(int)   - 
Method in class weka.datagenerators.clusterers. BIRCHCluster 
 Sets the number of clusters the dataset should have.
   setNumComponents(int)   - 
Method in class weka.filters.supervised.attribute. PLSFilter 
 sets the maximum number of attributes to use.
   setNumCycles(int)   - 
Method in class weka.datagenerators.clusterers. BIRCHCluster 
 Sets the the number of cycles.
   setNumDate(int)   - 
Method in class weka.core. CheckScheme 
 sets the number of data attributes
   setNumDate(int)   - 
Method in class weka.core. TestInstances 
 sets the number of date attributes
   setNumeric(boolean)   - 
Method in class weka.filters.unsupervised.attribute. MakeIndicator 
 Sets if the new Attribute is to be numeric.
   setNumExamples(int)   - 
Method in class weka.datagenerators. ClassificationGenerator 
 Sets the number of examples, given by option.
   setNumExamples(int)   - 
Method in class weka.datagenerators.classifiers.classification. BayesNet 
 Sets the number of examples, given by option.
   setNumExamples(int)   - 
Method in class weka.datagenerators. RegressionGenerator 
 Sets the number of examples, given by option.
   setNumFeatures(int)   - 
Method in class weka.classifiers.trees. RandomForest 
 Set the number of features to use in random selection.
   setNumFoldersMIOption(int)   - 
Method in class weka.classifiers.rules. NNge 
 Sets the number of folder for mutual information.
   setNumFolds(int)   - 
Method in class weka.classifiers.bayes. BayesianLogisticRegression 
 Set the number of folds to use for CV-based hyperparameter
 selection
   setNumFolds(int)   - 
Method in class weka.classifiers.functions. SMO 
 Set the value of numFolds.
   setNumFolds(int)   - 
Method in class weka.classifiers.meta. CVParameterSelection 
 Sets the number of folds for the cross-validation.
   setNumFolds(int)   - 
Method in class weka.classifiers.meta. Dagging 
 Sets the number of folds to use for splitting the training set.
   setNumFolds(int)   - 
Method in class weka.classifiers.meta. LogitBoost 
 Set the value of NumFolds.
   setNumFolds(int)   - 
Method in class weka.classifiers.meta. MultiScheme 
 Sets the number of folds for cross-validation.
   setNumFolds(int)   - 
Method in class weka.classifiers.meta. Stacking 
 Sets the number of folds for the cross-validation.
   setNumFolds(int)   - 
Method in class weka.classifiers.mi. MISMO 
 Set the value of numFolds.
   setNumFolds(int)   - 
Method in class weka.classifiers.rules. PART 
 Set the value of numFolds.
   setNumFolds(int)   - 
Method in class weka.classifiers.trees. J48 
 Set the value of numFolds.
   setNumFolds(int)   - 
Method in class weka.classifiers.trees. RandomTree 
 Set the value of NumFolds.
   setNumFolds(int)   - 
Method in class weka.classifiers.trees. REPTree 
 Set the value of NumFolds.
   setNumFolds(int)   - 
Method in class weka.experiment. CrossValidationResultProducer 
 Set the value of NumFolds.
   setNumFolds(int)   - 
Method in class weka.filters.supervised.instance. StratifiedRemoveFolds 
 Sets the number of folds the dataset is split into.
   setNumFolds(int)   - 
Method in class weka.filters.unsupervised.instance. RemoveFolds 
 Sets the number of folds the dataset is split into.
   setNumFolds(int)   - 
Method in class weka.filters.unsupervised.instance. RemoveMisclassified 
 Sets the number of cross-validation folds to use
 - < 2 means no cross-validation.
   setNumFoldsPruning(int)   - 
Method in class weka.classifiers.trees. BFTree 
 Set number of folds in internal cross-validation.
   setNumFoldsPruning(int)   - 
Method in class weka.classifiers.trees. SimpleCart 
 Set number of folds in internal cross-validation.
   setNumInstances(int)   - 
Method in class weka.core. CheckScheme 
 Sets the number of instances to use in the datasets (some classifiers
 might require more instances).
   setNumInstances(int)   - 
Method in class weka.core. TestInstances 
 sets the number of instances to produce
   setNumInstances(Random)   - 
Method in class weka.datagenerators.clusterers. SubspaceClusterDefinition 
 Sets the real number of instances for this cluster.
   setNumInstances(int)   - 
Method in class weka.estimators. CheckEstimator 
 Sets the number of instances to use in the datasets (some estimators
 might require more instances).
   setNumInstancesRelational(int)   - 
Method in class weka.core. CheckScheme 
 sets the number of instances in relational/bag attributes to produce
   setNumInstancesRelational(int)   - 
Method in class weka.core. TestInstances 
 sets the number of instances in relational/bag attributes to produce
   setNumIrrelevant(int)   - 
Method in class weka.datagenerators.classifiers.classification. RDG1 
 Sets the number of irrelevant attributes.
   setNumIterations(int)   - 
Method in class weka.classifiers.bayes. DMNBtext 
 Sets the number of iterations to be performed
   setNumIterations(int)   - 
Method in class weka.classifiers.functions. VotedPerceptron 
 Set the value of NumIterations.
   setNumIterations(int)   - 
Method in class weka.classifiers.functions. Winnow 
 Set the value of numIterations.
   setNumIterations(int)   - 
Method in class weka.classifiers. IteratedSingleClassifierEnhancer 
 Sets the number of bagging iterations
   setNumIterations(int)   - 
Method in class weka.classifiers.meta. MetaCost 
 Sets the number of bagging iterations
   setNumNeighbours(int)   - 
Method in class weka.attributeSelection. ReliefFAttributeEval 
 Set the number of nearest neighbours
   setNumNeighbours(int)   - 
Method in class weka.classifiers.mi. MINND 
 Sets the number of nearest neighbours to estimate
 the class prediction of tests bags
   setNumNominal(int)   - 
Method in class weka.core. CheckScheme 
 sets the number of nominal attributes
   setNumNominal(int)   - 
Method in class weka.core. TestInstances 
 sets the number of nominal attributes
   setNumNominalValues(int)   - 
Method in class weka.core. TestInstances 
 sets the number of values for nominal attributes
   setNumNumeric(int)   - 
Method in class weka.core. CheckScheme 
 sets the number of numeric attributes
   setNumNumeric(int)   - 
Method in class weka.core. TestInstances 
 sets the number of numeric attributes
   setNumNumeric(int)   - 
Method in class weka.datagenerators.classifiers.classification. RDG1 
 Sets the number of numerical attributes.
   setNumOfBoostingIterations(int)   - 
Method in class weka.classifiers.trees. ADTree 
 Sets the number of boosting iterations.
   setNumOfBoostingIterations(int)   - 
Method in class weka.classifiers.trees. LADTree 
 Sets the number of boosting iterations.
   setNumReferences(int)   - 
Method in class weka.classifiers.mi. CitationKNN 
 Sets the number of references considered to estimate
 the class prediction of tests bags
   setNumRelational(int)   - 
Method in class weka.core. CheckScheme 
 sets the number of relational attributes
   setNumRelational(int)   - 
Method in class weka.core. TestInstances 
 sets the number of relational attributes
   setNumRelationalDate(int)   - 
Method in class weka.core. TestInstances 
 sets the number of date attributes in a relational attribute
   setNumRelationalNominal(int)   - 
Method in class weka.core. TestInstances 
 sets the number of nominal attributes in a relational attribute
   setNumRelationalNominalValues(int)   - 
Method in class weka.core. TestInstances 
 sets the number of values for nominal attributes in a relational attribute
   setNumRelationalNumeric(int)   - 
Method in class weka.core. TestInstances 
 sets the number of numeric attributes in a relational attribute
   setNumRelationalString(int)   - 
Method in class weka.core. TestInstances 
 sets the number of string attributes in a relational attribute
   setNumRestarts(int)   - 
Method in class weka.clusterers. sIB 
 Set the number of restarts
   setNumRules(int)   - 
Method in class weka.associations. Apriori 
 Set the value of numRules.
   setNumRules(int)   - 
Method in class weka.associations. PredictiveApriori 
 Set the value of required rules.
   setNumRulesToFind(int)   - 
Method in class weka.associations. FPGrowth 
 Set the desired number of rules to find.
   setNumRuns(int)   - 
Method in class weka.classifiers.meta. LogitBoost 
 Set the value of NumRuns.
   setNumSamplesPerRegion(int)   - 
Method in class weka.gui.boundaryvisualizer. BoundaryPanel 
 Set the number of points to uniformly sample from a region (fixed
 dimensions).
   setNumSamplesPerRegion(int)   - 
Method in class weka.gui.boundaryvisualizer. RemoteBoundaryVisualizerSubTask 
 Set the number of points to uniformly sample from a region (fixed
 dimensions).
   setNumString(int)   - 
Method in class weka.core. CheckScheme 
 sets the number of string attributes
   setNumString(int)   - 
Method in class weka.core. TestInstances 
 sets the number of string attributes
   setNumSubCmtys(int)   - 
Method in class weka.classifiers.meta. MultiBoostAB 
 Set the number of sub committees to use
   setNumSubsetSizeCVFolds(int)   - 
Method in class weka.attributeSelection. SubsetSizeForwardSelection 
 Set the number of cross validation folds for subset size determination
 (default = 5).
   setNumTestingNoises(int)   - 
Method in class weka.classifiers.mi. MINND 
 Sets The number of nearest neighbour exemplars in the 
 selection of noises in the test data
   setNumToSelect(int)   - 
Method in class weka.attributeSelection. GreedyStepwise 
 Specify the number of attributes to select from the ranked list
 (if generating a ranking).
   setNumToSelect(int)   - 
Method in class weka.attributeSelection. RaceSearch 
 Specify the number of attributes to select from the ranked list
 (if generating a ranking).
   setNumToSelect(int)   - 
Method in interface weka.attributeSelection. RankedOutputSearch 
 Specify the number of attributes to select from the ranked list.
   setNumToSelect(int)   - 
Method in class weka.attributeSelection. Ranker 
 Specify the number of attributes to select from the ranked list.
   setNumTrainingNoises(int)   - 
Method in class weka.classifiers.mi. MINND 
 Sets the number of nearest neighbour instances in the 
 selection of noises in the training data
   setNumTrees(int)   - 
Method in class weka.classifiers.trees. RandomForest 
 Set the value of numTrees.
   setNumUsedAttributes(int)   - 
Method in class weka.attributeSelection. LinearForwardSelection 
 Set the number of top-ranked attributes that taken into account by the
 search process.
   setNumUsedAttributes(int)   - 
Method in class weka.attributeSelection. SubsetSizeForwardSelection 
 Set the number of top-ranked attributes that taken into account by the
 search process.
   setNumValues(int)   - 
Method in class weka.filters.unsupervised.instance. RemoveFrequentValues 
 Sets how many values are retained
   setNumXValFolds(int)   - 
Method in class weka.classifiers.meta. ThresholdSelector 
 Set the number of folds used for cross-validation.
   setObject(Object)   - 
Method in class weka.core. CheckGOE 
 Set the object to work on..
   setObject(Object)   - 
Method in class weka.gui.beans. AssociatorCustomizer 
 Set the classifier object to be edited
   setObject(Object)   - 
Method in class weka.gui.beans. ClassAssignerCustomizer 
 Set the bean to be edited
   setObject(Object)   - 
Method in class weka.gui.beans. ClassifierCustomizer 
 Set the classifier object to be edited
   setObject(Object)   - 
Method in class weka.gui.beans. ClassValuePickerCustomizer 
 Set the bean to be edited
   setObject(Object)   - 
Method in class weka.gui.beans. ClustererCustomizer 
 Set the Clusterer object to be edited
   setObject(Object)   - 
Method in class weka.gui.beans. CrossValidationFoldMakerCustomizer 
 Set the object to be edited
   setObject(Object)   - 
Method in class weka.gui.beans. FilterCustomizer 
 Set the filter bean to be edited
   setObject(Object)   - 
Method in class weka.gui.beans. IncrementalClassifierEvaluatorCustomizer 
 Set the object to be edited
   setObject(Object)   - 
Method in class weka.gui.beans. LoaderCustomizer 
 Set the loader to be customized
   setObject(Object)   - 
Method in class weka.gui.beans. PredictionAppenderCustomizer 
 Set the object to be edited
   setObject(Object)   - 
Method in class weka.gui.beans. SaverCustomizer 
 Set the saver to be customized
   setObject(Object)   - 
Method in class weka.gui.beans. SerializedModelSaverCustomizer 
 Set the model saver to be customized
   setObject(Object)   - 
Method in class weka.gui.beans. StripChartCustomizer 
 Set the StripChart object to be customized
   setObject(Object)   - 
Method in class weka.gui.beans. TrainTestSplitMakerCustomizer 
 Set the TrainTestSplitMaker to be customized
   setOfSequencesToString(FastVector, Instances, FastVector)   - 
Static method in class weka.associations.gsp. Sequence 
 Returns a String representation of a set of Sequences where the numeric 
 value of each event/item is represented by its respective nominal value.
   setOkButtonText(String)   - 
Method in class weka.gui. GenericObjectEditor.GOEPanel 
 Allows customization of the action label on the dialog.
   setOmega(double)   - 
Method in class weka.classifiers.functions.supportVector. Puk 
 Sets the omega value.
   setOn(boolean)   - 
Method in class weka.gui.visualize. ClassPanel 
 Enables the panel
   setOnDemandDirectory(File)   - 
Method in class weka.attributeSelection. CostSensitiveASEvaluation 
 Sets the directory that will be searched for cost files when
 loading on demand.
   setOnDemandDirectory(File)   - 
Method in class weka.classifiers.meta. CostSensitiveClassifier 
 Sets the directory that will be searched for cost files when
 loading on demand.
   setOnDemandDirectory(File)   - 
Method in class weka.classifiers.meta. MetaCost 
 Sets the directory that will be searched for cost files when
 loading on demand.
   setOnDemandDirectory(File)   - 
Method in class weka.experiment. CostSensitiveClassifierSplitEvaluator 
 Sets the directory that will be searched for cost files when
 loading on demand.
   setOptimalColumnWidth(int)   - 
Method in class weka.gui. JTableHelper 
 sets the optimal column width for the given column
   setOptimalColumnWidth(JTable, int)   - 
Static method in class weka.gui. JTableHelper 
 sets the optimal column width for the given column
   setOptimalColumnWidth()   - 
Method in class weka.gui. JTableHelper 
 sets the optimal column width for all columns
   setOptimalColumnWidth(JTable)   - 
Static method in class weka.gui. JTableHelper 
 sets the optimal column width for alls column if the given table
   setOptimalColWidth()   - 
Method in class weka.gui.arffviewer. ArffPanel 
 calculates the optimal column width for the current column
   setOptimalColWidths()   - 
Method in class weka.gui.arffviewer. ArffPanel 
 calculates the optimal column widths for all columns
   setOptimalColWidths()   - 
Method in class weka.gui.arffviewer. ArffViewerMainPanel 
 sets the optimal column width for all columns
   setOptimalHeaderWidth(int)   - 
Method in class weka.gui. JTableHelper 
 sets the optimal header width for the given column
   setOptimalHeaderWidth(JTable, int)   - 
Static method in class weka.gui. JTableHelper 
 sets the optimal header width for the given column
   setOptimalHeaderWidth()   - 
Method in class weka.gui. JTableHelper 
 sets the optimal header width for all columns
   setOptimalHeaderWidth(JTable)   - 
Static method in class weka.gui. JTableHelper 
 sets the optimal header width for alls column if the given table
   setOptimizations(int)   - 
Method in class weka.classifiers.rules. JRip 
 Sets the number of optimization runs
   setOptionHandler(OptionHandler)   - 
Method in class weka.core. CheckOptionHandler 
 Set the OptionHandler to work on..
   setOptions(String[])   - 
Method in class weka.associations. Apriori 
 Parses a given list of options.
   setOptions(String[])   - 
Method in class weka.associations. CheckAssociator 
 Parses a given list of options.
   setOptions(String[])   - 
Method in class weka.associations. FilteredAssociator 
 Parses a given list of options.
   setOptions(String[])   - 
Method in class weka.associations. FPGrowth 
 Parses a given list of options.
   setOptions(String[])   - 
Method in class weka.associations. GeneralizedSequentialPatterns 
 Parses a given list of options.
   setOptions(String[])   - 
Method in class weka.associations. PredictiveApriori 
 Parses a given list of options.
   setOptions(String[])   - 
Method in class weka.associations. SingleAssociatorEnhancer 
 Parses a given list of options.
   setOptions(String[])   - 
Method in class weka.associations. Tertius 
 Parses a given list of options.
   setOptions(String[])   - 
Method in class weka.attributeSelection. BestFirst 
 Parses a given list of options.
   setOptions(String[])   - 
Method in class weka.attributeSelection. CfsSubsetEval 
 Parses and sets a given list of options.
   setOptions(String[])   - 
Method in class weka.attributeSelection. CheckAttributeSelection 
 Parses a given list of options.
   setOptions(String[])   - 
Method in class weka.attributeSelection. ChiSquaredAttributeEval 
 Parses a given list of options.
   setOptions(String[])   - 
Method in class weka.attributeSelection. ClassifierSubsetEval 
 Parses a given list of options.
   setOptions(String[])   - 
Method in class weka.attributeSelection. CostSensitiveASEvaluation 
 Parses a given list of options.
   setOptions(String[])   - 
Method in class weka.attributeSelection. ExhaustiveSearch 
 Parses a given list of options.
   setOptions(String[])   - 
Method in class weka.attributeSelection. FilteredAttributeEval 
 Parses a given list of options.
   setOptions(String[])   - 
Method in class weka.attributeSelection. FilteredSubsetEval 
 Parses a given list of options.
   setOptions(String[])   - 
Method in class weka.attributeSelection. GainRatioAttributeEval 
 Parses a given list of options.
   setOptions(String[])   - 
Method in class weka.attributeSelection. GeneticSearch 
 Parses a given list of options.
   setOptions(String[])   - 
Method in class weka.attributeSelection. GreedyStepwise 
 Parses a given list of options.
   setOptions(String[])   - 
Method in class weka.attributeSelection. InfoGainAttributeEval 
 Parses a given list of options.
   setOptions(String[])   - 
Method in class weka.attributeSelection. LatentSemanticAnalysis 
 Parses a given list of options.
   setOptions(String[])   - 
Method in class weka.attributeSelection. LinearForwardSelection 
 Parses a given list of options.
   setOptions(String[])   - 
Method in class weka.attributeSelection. OneRAttributeEval 
 Parses a given list of options.
   setOptions(String[])   - 
Method in class weka.attributeSelection. PrincipalComponents 
 Parses a given list of options.
   setOptions(String[])   - 
Method in class weka.attributeSelection. RaceSearch 
 Parses a given list of options.
   setOptions(String[])   - 
Method in class weka.attributeSelection. RandomSearch 
 Parses a given list of options.
   setOptions(String[])   - 
Method in class weka.attributeSelection. Ranker 
 Parses a given list of options.
   setOptions(String[])   - 
Method in class weka.attributeSelection. RankSearch 
 Parses a given list of options.
   setOptions(String[])   - 
Method in class weka.attributeSelection. ReliefFAttributeEval 
 Parses a given list of options.
   setOptions(String[])   - 
Method in class weka.attributeSelection. ScatterSearchV1 
 Parses a given list of options.
   setOptions(String[])   - 
Method in class weka.attributeSelection. SubsetSizeForwardSelection 
 Parses a given list of options.
   setOptions(String[])   - 
Method in class weka.attributeSelection. SVMAttributeEval 
 Parses a given list of options.
   setOptions(String[])   - 
Method in class weka.attributeSelection. SymmetricalUncertAttributeEval 
 Parses a given list of options.
   setOptions(String[])   - 
Method in class weka.attributeSelection. WrapperSubsetEval 
 Parses a given list of options.
   setOptions(String[])   - 
Method in class weka.classifiers.bayes. AODE 
 Parses a given list of options.
   setOptions(String[])   - 
Method in class weka.classifiers.bayes. AODEsr 
 Parses a given list of options.
   setOptions(String[])   - 
Method in class weka.classifiers.bayes. BayesianLogisticRegression 
 Parses a given list of options.
   setOptions(String[])   - 
Method in class weka.classifiers.bayes. BayesNet 
 Parses a given list of options.
   setOptions(String[])   - 
Method in class weka.classifiers.bayes. ComplementNaiveBayes 
 Parses a given list of options.
   setOptions(String[])   - 
Method in class weka.classifiers.bayes. DMNBtext 
  
   setOptions(String[])   - 
Method in class weka.classifiers.bayes. NaiveBayes 
 Parses a given list of options.
   setOptions(String[])   - 
Method in class weka.classifiers.bayes.net. BayesNetGenerator 
 Parses a given list of options.
   setOptions(String[])   - 
Method in class weka.classifiers.bayes.net.estimate. BayesNetEstimator 
 Parses a given list of options.
   setOptions(String[])   - 
Method in class weka.classifiers.bayes.net.estimate. BMAEstimator 
 Parses a given list of options.
   setOptions(String[])   - 
Method in class weka.classifiers.bayes.net.estimate. MultiNomialBMAEstimator 
 Parses a given list of options.
   setOptions(String[])   - 
Method in class weka.classifiers.bayes.net.search.ci. ICSSearchAlgorithm 
 Parses a given list of options.
   setOptions(String[])   - 
Method in class weka.classifiers.bayes.net.search.fixed. FromFile 
 Parses a given list of options.
   setOptions(String[])   - 
Method in class weka.classifiers.bayes.net.search.global. GeneticSearch 
 Parses a given list of options.
   setOptions(String[])   - 
Method in class weka.classifiers.bayes.net.search.global. GlobalScoreSearchAlgorithm 
 Parses a given list of options.
   setOptions(String[])   - 
Method in class weka.classifiers.bayes.net.search.global. HillClimber 
 Parses a given list of options.
   setOptions(String[])   - 
Method in class weka.classifiers.bayes.net.search.global. K2 
 Parses a given list of options.
   setOptions(String[])   - 
Method in class weka.classifiers.bayes.net.search.global. RepeatedHillClimber 
 Parses a given list of options.
   setOptions(String[])   - 
Method in class weka.classifiers.bayes.net.search.global. SimulatedAnnealing 
 Parses a given list of options.
   setOptions(String[])   - 
Method in class weka.classifiers.bayes.net.search.global. TabuSearch 
 Parses a given list of options.
   setOptions(String[])   - 
Method in class weka.classifiers.bayes.net.search.global. TAN 
 Parses a given list of options.
   setOptions(String[])   - 
Method in class weka.classifiers.bayes.net.search.local. GeneticSearch 
 Parses a given list of options.
   setOptions(String[])   - 
Method in class weka.classifiers.bayes.net.search.local. HillClimber 
 Parses a given list of options.
   setOptions(String[])   - 
Method in class weka.classifiers.bayes.net.search.local. K2 
 Parses a given list of options.
   setOptions(String[])   - 
Method in class weka.classifiers.bayes.net.search.local. LAGDHillClimber 
 Parses a given list of options.
   setOptions(String[])   - 
Method in class weka.classifiers.bayes.net.search.local. LocalScoreSearchAlgorithm 
 Parses a given list of options.
   setOptions(String[])   - 
Method in class weka.classifiers.bayes.net.search.local. RepeatedHillClimber 
 Parses a given list of options.
   setOptions(String[])   - 
Method in class weka.classifiers.bayes.net.search.local. SimulatedAnnealing 
 Parses a given list of options.
   setOptions(String[])   - 
Method in class weka.classifiers.bayes.net.search.local. TabuSearch 
 Parses a given list of options.
   setOptions(String[])   - 
Method in class weka.classifiers.bayes.net.search.local. TAN 
 Parses a given list of options.
   setOptions(String[])   - 
Method in class weka.classifiers.bayes.net.search. SearchAlgorithm 
 Parses a given list of options.
   setOptions(String[])   - 
Method in class weka.classifiers.bayes. WAODE 
 Parses a given list of options.
   setOptions(String[])   - 
Method in class weka.classifiers. BVDecompose 
 Parses a given list of options.
   setOptions(String[])   - 
Method in class weka.classifiers. BVDecomposeSegCVSub 
 Sets the OptionHandler's options using the given list.
   setOptions(String[])   - 
Method in class weka.classifiers. CheckClassifier 
 Parses a given list of options.
   setOptions(String[])   - 
Method in class weka.classifiers. CheckSource 
 Parses a given list of options.
   setOptions(String[])   - 
Method in class weka.classifiers. Classifier 
 Parses a given list of options.
   setOptions(String[])   - 
Method in class weka.classifiers.functions. GaussianProcesses 
 Parses a given list of options.
   setOptions(String[])   - 
Method in class weka.classifiers.functions. LeastMedSq 
 Sets the OptionHandler's options using the given list.
   setOptions(String[])   - 
Method in class weka.classifiers.functions. LibLINEAR 
 Sets the classifier options  

    
 Valid options are:  
   setOptions(String[])   - 
Method in class weka.classifiers.functions. LibSVM 
 Sets the classifier options  
 
    
 Valid options are:  
   setOptions(String[])   - 
Method in class weka.classifiers.functions. LinearRegression 
 Parses a given list of options.
   setOptions(String[])   - 
Method in class weka.classifiers.functions. Logistic 
 Parses a given list of options.
   setOptions(String[])   - 
Method in class weka.classifiers.functions. MultilayerPerceptron 
 Parses a given list of options.
   setOptions(String[])   - 
Method in class weka.classifiers.functions. PaceRegression 
 Parses a given list of options.
   setOptions(String[])   - 
Method in class weka.classifiers.functions. PLSClassifier 
 Parses the options for this object.
   setOptions(String[])   - 
Method in class weka.classifiers.functions. RBFNetwork 
 Parses a given list of options.
   setOptions(String[])   - 
Method in class weka.classifiers.functions. SimpleLogistic 
 Parses a given list of options.
   setOptions(String[])   - 
Method in class weka.classifiers.functions. SMO 
 Parses a given list of options.
   setOptions(String[])   - 
Method in class weka.classifiers.functions. SMOreg 
 Parses a given list of options.
   setOptions(String[])   - 
Method in class weka.classifiers.functions. SPegasos 
 Parses a given list of options.
   setOptions(String[])   - 
Method in class weka.classifiers.functions.supportVector. CachedKernel 
 Parses a given list of options.
   setOptions(String[])   - 
Method in class weka.classifiers.functions.supportVector. CheckKernel 
 Parses a given list of options.
   setOptions(String[])   - 
Method in class weka.classifiers.functions.supportVector. Kernel 
 Parses a given list of options.
   setOptions(String[])   - 
Method in class weka.classifiers.functions.supportVector. PolyKernel 
 Parses a given list of options.
   setOptions(String[])   - 
Method in class weka.classifiers.functions.supportVector. PrecomputedKernelMatrixKernel 
 Parses a given list of options.
   setOptions(String[])   - 
Method in class weka.classifiers.functions.supportVector. Puk 
 Parses a given list of options.
   setOptions(String[])   - 
Method in class weka.classifiers.functions.supportVector. RBFKernel 
 Parses a given list of options.
   setOptions(String[])   - 
Method in class weka.classifiers.functions.supportVector. RegOptimizer 
 Parses a given list of options.
   setOptions(String[])   - 
Method in class weka.classifiers.functions.supportVector. RegSMO 
 Parses a given list of options.
   setOptions(String[])   - 
Method in class weka.classifiers.functions.supportVector. RegSMOImproved 
 Parses a given list of options.
   setOptions(String[])   - 
Method in class weka.classifiers.functions.supportVector. StringKernel 
 Parses a given list of options.
   setOptions(String[])   - 
Method in class weka.classifiers.functions. VotedPerceptron 
 Parses a given list of options.
   setOptions(String[])   - 
Method in class weka.classifiers.functions. Winnow 
 Parses a given list of options. 

    
 Valid options are:  
   setOptions(String[])   - 
Method in class weka.classifiers. IteratedSingleClassifierEnhancer 
 Parses a given list of options.
   setOptions(String[])   - 
Method in class weka.classifiers.lazy. IBk 
 Parses a given list of options.
   setOptions(int, int, int)   - 
Method in class weka.classifiers.lazy.kstar. KStarNominalAttribute 
 Sets the options.
   setOptions(int, int, int)   - 
Method in class weka.classifiers.lazy.kstar. KStarNumericAttribute 
 Set options.
   setOptions(String[])   - 
Method in class weka.classifiers.lazy. KStar 
 Parses a given list of options.
   setOptions(String[])   - 
Method in class weka.classifiers.lazy. LWL 
 Parses a given list of options.
   setOptions(String[])   - 
Method in class weka.classifiers.meta. AdaBoostM1 
 Parses a given list of options.
   setOptions(String[])   - 
Method in class weka.classifiers.meta. AdditiveRegression 
 Parses a given list of options.
   setOptions(String[])   - 
Method in class weka.classifiers.meta. AttributeSelectedClassifier 
 Parses a given list of options.
   setOptions(String[])   - 
Method in class weka.classifiers.meta. Bagging 
 Parses a given list of options.
   setOptions(String[])   - 
Method in class weka.classifiers.meta. ClassificationViaClustering 
 Parses the options for this object.
   setOptions(String[])   - 
Method in class weka.classifiers.meta. CostSensitiveClassifier 
 Parses a given list of options.
   setOptions(String[])   - 
Method in class weka.classifiers.meta. CVParameterSelection 
 Parses a given list of options.
   setOptions(String[])   - 
Method in class weka.classifiers.meta. Dagging 
 Parses a given list of options.
   setOptions(String[])   - 
Method in class weka.classifiers.meta. Decorate 
 Parses a given list of options.
   setOptions(String[])   - 
Method in class weka.classifiers.meta. FilteredClassifier 
 Parses a given list of options.
   setOptions(String[])   - 
Method in class weka.classifiers.meta. GridSearch 
 Parses the options for this object.
   setOptions(String[])   - 
Method in class weka.classifiers.meta. LogitBoost 
 Parses a given list of options.
   setOptions(String[])   - 
Method in class weka.classifiers.meta. MetaCost 
 Parses a given list of options.
   setOptions(String[])   - 
Method in class weka.classifiers.meta. MultiBoostAB 
 Parses a given list of options.
   setOptions(String[])   - 
Method in class weka.classifiers.meta. MultiClassClassifier 
 Parses a given list of options.
   setOptions(String[])   - 
Method in class weka.classifiers.meta. MultiScheme 
 Parses a given list of options.
   setOptions(String[])   - 
Method in class weka.classifiers.meta. OrdinalClassClassifier 
 Parses a given list of options.
   setOptions(String[])   - 
Method in class weka.classifiers.meta. RacedIncrementalLogitBoost 
 Parses a given list of options.
   setOptions(String[])   - 
Method in class weka.classifiers.meta. RandomSubSpace 
 Parses a given list of options.
   setOptions(String[])   - 
Method in class weka.classifiers.meta. RegressionByDiscretization 
 Parses a given list of options.
   setOptions(String[])   - 
Method in class weka.classifiers.meta. RotationForest 
 Parses a given list of options.
   setOptions(String[])   - 
Method in class weka.classifiers.meta. Stacking 
 Parses a given list of options.
   setOptions(String[])   - 
Method in class weka.classifiers.meta. ThresholdSelector 
 Parses a given list of options.
   setOptions(String[])   - 
Method in class weka.classifiers.meta. Vote 
 Parses a given list of options.
   setOptions(String[])   - 
Method in class weka.classifiers.mi. CitationKNN 
 Sets the OptionHandler's options using the given list.
   setOptions(String[])   - 
Method in class weka.classifiers.mi. MDD 
 Parses a given list of options.
   setOptions(String[])   - 
Method in class weka.classifiers.mi. MIBoost 
 Parses a given list of options.
   setOptions(String[])   - 
Method in class weka.classifiers.mi. MIDD 
 Parses a given list of options.
   setOptions(String[])   - 
Method in class weka.classifiers.mi. MIEMDD 
 Parses a given list of options.
   setOptions(String[])   - 
Method in class weka.classifiers.mi. MILR 
 Parses a given list of options.
   setOptions(String[])   - 
Method in class weka.classifiers.mi. MINND 
 Parses a given list of options.
   setOptions(String[])   - 
Method in class weka.classifiers.mi. MIOptimalBall 
 Parses a given list of options.
   setOptions(String[])   - 
Method in class weka.classifiers.mi. MISMO 
 Parses a given list of options.
   setOptions(String[])   - 
Method in class weka.classifiers.mi. MISVM 
 Parses a given list of options.
   setOptions(String[])   - 
Method in class weka.classifiers.mi. MIWrapper 
 Parses a given list of options.
   setOptions(String[])   - 
Method in class weka.classifiers.mi. SimpleMI 
 Parses a given list of options.
   setOptions(String[])   - 
Method in class weka.classifiers.misc. SerializedClassifier 
 Parses the options for this object.
   setOptions(String[])   - 
Method in class weka.classifiers.misc. VFI 
 Parses a given list of options.
   setOptions(String[])   - 
Method in class weka.classifiers. MultipleClassifiersCombiner 
 Parses a given list of options.
   setOptions(String[])   - 
Method in class weka.classifiers. RandomizableClassifier 
 Parses a given list of options.
   setOptions(String[])   - 
Method in class weka.classifiers. RandomizableIteratedSingleClassifierEnhancer 
 Parses a given list of options.
   setOptions(String[])   - 
Method in class weka.classifiers. RandomizableMultipleClassifiersCombiner 
 Parses a given list of options.
   setOptions(String[])   - 
Method in class weka.classifiers. RandomizableSingleClassifierEnhancer 
 Parses a given list of options.
   setOptions(String[])   - 
Method in class weka.classifiers.rules. ConjunctiveRule 
 Parses a given list of options.
   setOptions(String[])   - 
Method in class weka.classifiers.rules. DecisionTable 
 Parses the options for this object.
   setOptions(String[])   - 
Method in class weka.classifiers.rules. DTNB 
 Parses the options for this object.
   setOptions(String[])   - 
Method in class weka.classifiers.rules. JRip 
 Parses a given list of options.
   setOptions(String[])   - 
Method in class weka.classifiers.rules. NNge 
 Sets the OptionHandler's options using the given list.
   setOptions(String[])   - 
Method in class weka.classifiers.rules. OneR 
 Parses a given list of options.
   setOptions(String[])   - 
Method in class weka.classifiers.rules. PART 
 Parses a given list of options.
   setOptions(String[])   - 
Method in class weka.classifiers.rules. Ridor 
 Parses a given list of options.
   setOptions(String[])   - 
Method in class weka.classifiers. SingleClassifierEnhancer 
 Parses a given list of options.
   setOptions(String[])   - 
Method in class weka.classifiers.trees. ADTree 
 Parses a given list of options.
   setOptions(String[])   - 
Method in class weka.classifiers.trees. BFTree 
 Parses the options for this object.
   setOptions(String[])   - 
Method in class weka.classifiers.trees. FT 
 Parses a given list of options.
   setOptions(String[])   - 
Method in class weka.classifiers.trees. J48 
 Parses a given list of options.
   setOptions(String[])   - 
Method in class weka.classifiers.trees. J48graft 
 Parses a given list of options.
   setOptions(String[])   - 
Method in class weka.classifiers.trees. LADTree 
 Parses a given list of options.
   setOptions(String[])   - 
Method in class weka.classifiers.trees. LMT 
 Parses a given list of options.
   setOptions(String[])   - 
Method in class weka.classifiers.trees.m5. M5Base 
 Parses a given list of options.
   setOptions(String[])   - 
Method in class weka.classifiers.trees. M5P 
 Parses a given list of options.
   setOptions(String[])   - 
Method in class weka.classifiers.trees. RandomForest 
 Parses a given list of options.
   setOptions(String[])   - 
Method in class weka.classifiers.trees. RandomTree 
 Parses a given list of options.
   setOptions(String[])   - 
Method in class weka.classifiers.trees. REPTree 
 Parses a given list of options.
   setOptions(String[])   - 
Method in class weka.classifiers.trees. SimpleCart 
 Parses a given list of options.
   setOptions(String[])   - 
Method in class weka.clusterers. CheckClusterer 
 Parses a given list of options.
   setOptions(String[])   - 
Method in class weka.clusterers. CLOPE 
 Parses a given list of options.
   setOptions(String[])   - 
Method in class weka.clusterers. Cobweb 
 Parses a given list of options.
   setOptions(String[])   - 
Method in class weka.clusterers. DBScan 
 Sets the OptionHandler's options using the given list.
   setOptions(String[])   - 
Method in class weka.clusterers. EM 
 Parses a given list of options.
   setOptions(String[])   - 
Method in class weka.clusterers. FarthestFirst 
 Parses a given list of options.
   setOptions(String[])   - 
Method in class weka.clusterers. FilteredClusterer 
 Parses a given list of options.
   setOptions(String[])   - 
Method in class weka.clusterers. HierarchicalClusterer 
 Parses a given list of options.
   setOptions(String[])   - 
Method in class weka.clusterers. MakeDensityBasedClusterer 
 Parses a given list of options.
   setOptions(String[])   - 
Method in class weka.clusterers. OPTICS 
 Sets the OptionHandler's options using the given list.
   setOptions(String[])   - 
Method in class weka.clusterers. RandomizableClusterer 
 Parses a given list of options.
   setOptions(String[])   - 
Method in class weka.clusterers. RandomizableDensityBasedClusterer 
 Parses a given list of options.
   setOptions(String[])   - 
Method in class weka.clusterers. RandomizableSingleClustererEnhancer 
 Parses a given list of options.
   setOptions(String[])   - 
Method in class weka.clusterers. sIB 
 Parses a given list of options.
   setOptions(String[])   - 
Method in class weka.clusterers. SimpleKMeans 
 Parses a given list of options.
   setOptions(String[])   - 
Method in class weka.clusterers. SingleClustererEnhancer 
 Parses a given list of options.
   setOptions(String[])   - 
Method in class weka.clusterers. XMeans 
 Parses a given list of options.
   setOptions(String[])   - 
Method in class weka.core. Check 
 Parses a given list of options.
   setOptions(String[])   - 
Method in class weka.core. CheckGOE 
 Parses a given list of options.
   setOptions(String[])   - 
Method in class weka.core. CheckOptionHandler 
 Parses a given list of options.
   setOptions(String[])   - 
Method in class weka.core. CheckScheme 
 Parses a given list of options.
   setOptions(String[])   - 
Method in class weka.core.converters. AbstractFileSaver 
 Parses a given list of options.
   setOptions(String[])   - 
Method in class weka.core.converters. ArffSaver 
 Parses the options for this object.
   setOptions(String[])   - 
Method in class weka.core.converters. C45Saver 
 Parses a given list of options.
   setOptions(String[])   - 
Method in class weka.core.converters. CSVLoader 
 Parses a given list of options.
   setOptions(String[])   - 
Method in class weka.core.converters. DatabaseLoader 
 Sets the options.
   setOptions(String[])   - 
Method in class weka.core.converters. DatabaseSaver 
 Sets the options.
   setOptions(String[])   - 
Method in class weka.core.converters. LibSVMSaver 
 Parses the options for this object.
   setOptions(String[])   - 
Method in class weka.core.converters. SVMLightSaver 
 Parses the options for this object.
   setOptions(String[])   - 
Method in class weka.core.converters. TextDirectoryLoader 
 Parses a given list of options.
   setOptions(String[])   - 
Method in class weka.core.converters. XRFFSaver 
 Parses the options for this object.
   setOptions(String[])   - 
Method in class weka.core. FindWithCapabilities 
 Parses a given list of options.
   setOptions(String[])   - 
Method in class weka.core. Javadoc 
 Parses a given list of options.
   setOptions(String[])   - 
Method in class weka.core. ListOptions 
 Parses a given list of options.
   setOptions(String[])   - 
Method in class weka.core.neighboursearch. BallTree 
 Parses a given list of options.
   setOptions(String[])   - 
Method in class weka.core.neighboursearch.balltrees. BallSplitter 
 Parses a given list of options.
   setOptions(String[])   - 
Method in class weka.core.neighboursearch.balltrees. BallTreeConstructor 
 Parses a given list of options.
   setOptions(String[])   - 
Method in class weka.core.neighboursearch.balltrees. MedianDistanceFromArbitraryPoint 
 Parses a given list of options.
   setOptions(String[])   - 
Method in class weka.core.neighboursearch.balltrees. MedianOfWidestDimension 
 Parses a given list of options.
   setOptions(String[])   - 
Method in class weka.core.neighboursearch.balltrees. MiddleOutConstructor 
 Parses a given list of options.
   setOptions(String[])   - 
Method in class weka.core.neighboursearch.balltrees. TopDownConstructor 
 Parses a given list of options.
   setOptions(String[])   - 
Method in class weka.core.neighboursearch. CoverTree 
 Parses a given list of options.
   setOptions(String[])   - 
Method in class weka.core.neighboursearch. KDTree 
 Parses a given list of options.
   setOptions(String[])   - 
Method in class weka.core.neighboursearch.kdtrees. KDTreeNodeSplitter 
 Parses a given list of options.
   setOptions(String[])   - 
Method in class weka.core.neighboursearch. LinearNNSearch 
 Parses a given list of options.
   setOptions(String[])   - 
Method in class weka.core.neighboursearch. NearestNeighbourSearch 
 Parses a given list of options.
   setOptions(String[])   - 
Method in class weka.core. NormalizableDistance 
 Parses a given list of options.
   setOptions(String[])   - 
Method in interface weka.core. OptionHandler 
 Sets the OptionHandler's options using the given list.
   setOptions(String[])   - 
Method in class weka.core. OptionHandlerJavadoc 
 Parses a given list of options.
   setOptions(String[])   - 
Method in class weka.core.stemmers. SnowballStemmer 
 Parses the options.
   setOptions(String[])   - 
Method in class weka.core. TechnicalInformationHandlerJavadoc 
 Parses a given list of options.
   setOptions(String[])   - 
Method in class weka.core. TestInstances 
 Parses a given list of options.
   setOptions(String[])   - 
Method in class weka.core.tokenizers. CharacterDelimitedTokenizer 
 Sets the OptionHandler's options using the given list.
   setOptions(String[])   - 
Method in class weka.core.tokenizers. NGramTokenizer 
 Parses a given list of options.
   setOptions(String[])   - 
Method in class weka.core.tokenizers. Tokenizer 
 Sets the OptionHandler's options using the given list.
   setOptions(String[])   - 
Method in class weka.datagenerators. ClassificationGenerator 
 Sets the options.
   setOptions(String[])   - 
Method in class weka.datagenerators.classifiers.classification. Agrawal 
 Parses a list of options for this object.
   setOptions(String[])   - 
Method in class weka.datagenerators.classifiers.classification. BayesNet 
 Parses a list of options for this object.
   setOptions(String[])   - 
Method in class weka.datagenerators.classifiers.classification. LED24 
 Parses a list of options for this object.
   setOptions(String[])   - 
Method in class weka.datagenerators.classifiers.classification. RandomRBF 
 Parses a list of options for this object.
   setOptions(String[])   - 
Method in class weka.datagenerators.classifiers.classification. RDG1 
 Parses a list of options for this object.
   setOptions(String[])   - 
Method in class weka.datagenerators.classifiers.regression. Expression 
 Parses a list of options for this object.
   setOptions(String[])   - 
Method in class weka.datagenerators.classifiers.regression. MexicanHat 
 Parses a list of options for this object.
   setOptions(String[])   - 
Method in class weka.datagenerators. ClusterDefinition 
 Parses a list of options for this object.
   setOptions(String[])   - 
Method in class weka.datagenerators.clusterers. BIRCHCluster 
 Parses a list of options for this object.
   setOptions(String[])   - 
Method in class weka.datagenerators.clusterers. SubspaceCluster 
 Parses a list of options for this object.
   setOptions(String[])   - 
Method in class weka.datagenerators.clusterers. SubspaceClusterDefinition 
 Parses a list of options for this object.
   setOptions(String[])   - 
Method in class weka.datagenerators. ClusterGenerator 
 Sets the options.
   setOptions(String[])   - 
Method in class weka.datagenerators. DataGenerator 
 Parses a list of options for this object.
   setOptions(String[])   - 
Method in class weka.datagenerators. RegressionGenerator 
 Sets the options.
   setOptions(String[])   - 
Method in class weka.estimators. CheckEstimator 
 Parses a given list of options.
   setOptions(String[])   - 
Method in class weka.estimators. Estimator 
 Parses a given list of options.
   setOptions(String[])   - 
Method in class weka.experiment. AveragingResultProducer 
 Parses a given list of options.
   setOptions(String[])   - 
Method in class weka.experiment. ClassifierSplitEvaluator 
 Parses a given list of options.
   setOptions(String[])   - 
Method in class weka.experiment. CostSensitiveClassifierSplitEvaluator 
 Parses a given list of options.
   setOptions(String[])   - 
Method in class weka.experiment. CrossValidationResultProducer 
 Parses a given list of options.
   setOptions(String[])   - 
Method in class weka.experiment. CSVResultListener 
 Parses a given list of options.
   setOptions(String[])   - 
Method in class weka.experiment. DatabaseResultProducer 
 Parses a given list of options.
   setOptions(String[])   - 
Method in class weka.experiment. DensityBasedClustererSplitEvaluator 
 Parses a given list of options.
   setOptions(String[])   - 
Method in class weka.experiment. Experiment 
 Parses a given list of options.
   setOptions(String[])   - 
Method in class weka.experiment. InstanceQuery 
 Parses a given list of options.
   setOptions(String[])   - 
Method in class weka.experiment. LearningRateResultProducer 
 Parses a given list of options.
   setOptions(String[])   - 
Method in class weka.experiment. PairedTTester 
 Parses a given list of options.
   setOptions(String[])   - 
Method in class weka.experiment. RandomSplitResultProducer 
 Parses a given list of options.
   setOptions(String[])   - 
Method in class weka.experiment. RegressionSplitEvaluator 
 Parses a given list of options.
   setOptions(String[])   - 
Method in class weka.filters. CheckSource 
 Parses a given list of options.
   setOptions(String[])   - 
Method in class weka.filters. MultiFilter 
 Parses a list of options for this object.
   setOptions(String[])   - 
Method in class weka.filters. SimpleFilter 
 Parses a list of options for this object.
   setOptions(String[])   - 
Method in class weka.filters.supervised.attribute. AddClassification 
 Parses the options for this object.
   setOptions(String[])   - 
Method in class weka.filters.supervised.attribute. AttributeSelection 
 Parses a given list of options.
   setOptions(String[])   - 
Method in class weka.filters.supervised.attribute. ClassOrder 
 Parses a given list of options.
   setOptions(String[])   - 
Method in class weka.filters.supervised.attribute. Discretize 
 Parses a given list of options.
   setOptions(String[])   - 
Method in class weka.filters.supervised.attribute. NominalToBinary 
 Parses a given list of options.
   setOptions(String[])   - 
Method in class weka.filters.supervised.attribute. PLSFilter 
 Parses the options for this object.
   setOptions(String[])   - 
Method in class weka.filters.supervised.instance. Resample 
 Parses a given list of options.
   setOptions(String[])   - 
Method in class weka.filters.supervised.instance. SMOTE 
 Parses a given list of options.
   setOptions(String[])   - 
Method in class weka.filters.supervised.instance. SpreadSubsample 
 Parses a given list of options.
   setOptions(String[])   - 
Method in class weka.filters.supervised.instance. StratifiedRemoveFolds 
 Parses a given list of options.
   setOptions(String[])   - 
Method in class weka.filters.unsupervised.attribute. AbstractTimeSeries 
 Parses a given list of options controlling the behaviour of this object.
   setOptions(String[])   - 
Method in class weka.filters.unsupervised.attribute. Add 
 Parses a given list of options.
   setOptions(String[])   - 
Method in class weka.filters.unsupervised.attribute. AddCluster 
 Parses a given list of options.
   setOptions(String[])   - 
Method in class weka.filters.unsupervised.attribute. AddExpression 
 Parses a given list of options.
   setOptions(String[])   - 
Method in class weka.filters.unsupervised.attribute. AddID 
 Parses a given list of options.
   setOptions(String[])   - 
Method in class weka.filters.unsupervised.attribute. AddNoise 
 Parses a given list of options.
   setOptions(String[])   - 
Method in class weka.filters.unsupervised.attribute. AddValues 
 Parses a given list of options.
   setOptions(String[])   - 
Method in class weka.filters.unsupervised.attribute. ChangeDateFormat 
 Parses a given list of options.
   setOptions(String[])   - 
Method in class weka.filters.unsupervised.attribute. ClassAssigner 
 Parses a list of options for this object.
   setOptions(String[])   - 
Method in class weka.filters.unsupervised.attribute. ClusterMembership 
 Parses a given list of options.
   setOptions(String[])   - 
Method in class weka.filters.unsupervised.attribute. Copy 
 Parses a given list of options.
   setOptions(String[])   - 
Method in class weka.filters.unsupervised.attribute. Discretize 
 Parses a given list of options.
   setOptions(String[])   - 
Method in class weka.filters.unsupervised.attribute. FirstOrder 
 Parses a given list of options.
   setOptions(String[])   - 
Method in class weka.filters.unsupervised.attribute. InterquartileRange 
 Parses a list of options for this object.
   setOptions(String[])   - 
Method in class weka.filters.unsupervised.attribute. KernelFilter 
 Parses a given list of options.
   setOptions(String[])   - 
Method in class weka.filters.unsupervised.attribute. MakeIndicator 
 Parses a given list of options.
   setOptions(String[])   - 
Method in class weka.filters.unsupervised.attribute. MathExpression 
 Parses a given list of options.
   setOptions(String[])   - 
Method in class weka.filters.unsupervised.attribute. MergeTwoValues 
 Parses a given list of options.
   setOptions(String[])   - 
Method in class weka.filters.unsupervised.attribute. MultiInstanceToPropositional 
 Parses a given list of options.
   setOptions(String[])   - 
Method in class weka.filters.unsupervised.attribute. NominalToBinary 
 Parses a given list of options.
   setOptions(String[])   - 
Method in class weka.filters.unsupervised.attribute. NominalToString 
 Parses a given list of options.
   setOptions(String[])   - 
Method in class weka.filters.unsupervised.attribute. Normalize 
 Parses a given list of options.
   setOptions(String[])   - 
Method in class weka.filters.unsupervised.attribute. NumericCleaner 
 Parses a given list of options.
   setOptions(String[])   - 
Method in class weka.filters.unsupervised.attribute. NumericToNominal 
 Parses a given list of options.
   setOptions(String[])   - 
Method in class weka.filters.unsupervised.attribute. NumericTransform 
 Parses a given list of options.
   setOptions(String[])   - 
Method in class weka.filters.unsupervised.attribute. PartitionedMultiFilter 
 Parses a list of options for this object.
   setOptions(String[])   - 
Method in class weka.filters.unsupervised.attribute. PKIDiscretize 
 Parses a given list of options.
   setOptions(String[])   - 
Method in class weka.filters.unsupervised.attribute. PotentialClassIgnorer 
 Parses a list of options for this object.
   setOptions(String[])   - 
Method in class weka.filters.unsupervised.attribute. PrincipalComponents 
 Parses a list of options for this object.
   setOptions(String[])   - 
Method in class weka.filters.unsupervised.attribute. PropositionalToMultiInstance 
 Parses a given list of options.
   setOptions(String[])   - 
Method in class weka.filters.unsupervised.attribute. RandomProjection 
 Parses a given list of options.
   setOptions(String[])   - 
Method in class weka.filters.unsupervised.attribute. RandomSubset 
 Parses a given list of options.
   setOptions(String[])   - 
Method in class weka.filters.unsupervised.attribute. RELAGGS 
 Parses the options for this object.
   setOptions(String[])   - 
Method in class weka.filters.unsupervised.attribute. Remove 
 Parses a given list of options.
   setOptions(String[])   - 
Method in class weka.filters.unsupervised.attribute. RemoveType 
 Parses a given list of options.
   setOptions(String[])   - 
Method in class weka.filters.unsupervised.attribute. RemoveUseless 
 Parses a given list of options.
   setOptions(String[])   - 
Method in class weka.filters.unsupervised.attribute. Reorder 
 Parses a given list of options.
   setOptions(String[])   - 
Method in class weka.filters.unsupervised.attribute. StringToNominal 
 Parses a given list of options.
   setOptions(String[])   - 
Method in class weka.filters.unsupervised.attribute. StringToWordVector 
 Parses a given list of options.
   setOptions(String[])   - 
Method in class weka.filters.unsupervised.attribute. SwapValues 
 Parses a given list of options.
   setOptions(String[])   - 
Method in class weka.filters.unsupervised.attribute. Wavelet 
 Parses the options for this object.
   setOptions(String[])   - 
Method in class weka.filters.unsupervised.instance. Normalize 
 Parses a given list of options.
   setOptions(String[])   - 
Method in class weka.filters.unsupervised.instance. Randomize 
 Parses a given list of options.
   setOptions(String[])   - 
Method in class weka.filters.unsupervised.instance. RemoveFolds 
 Parses a given list of options.
   setOptions(String[])   - 
Method in class weka.filters.unsupervised.instance. RemoveFrequentValues 
 Parses a given list of options.
   setOptions(String[])   - 
Method in class weka.filters.unsupervised.instance. RemoveMisclassified 
 Parses a given list of options.
   setOptions(String[])   - 
Method in class weka.filters.unsupervised.instance. RemovePercentage 
 Parses a given list of options.
   setOptions(String[])   - 
Method in class weka.filters.unsupervised.instance. RemoveRange 
 Parses a given list of options.
   setOptions(String[])   - 
Method in class weka.filters.unsupervised.instance. RemoveWithValues 
 Parses a given list of options.
   setOptions(String[])   - 
Method in class weka.filters.unsupervised.instance. Resample 
 Parses a given list of options.
   setOptions(String[])   - 
Method in class weka.filters.unsupervised.instance. ReservoirSample 
 Parses a given list of options.
   setOptions(String[])   - 
Method in class weka.filters.unsupervised.instance. SubsetByExpression 
 Parses a given list of options.
   setOptions(String[])   - 
Method in class weka.gui. Main 
 Parses the options for this object.
   setOriginalCoords(Vector)   - 
Method in class weka.gui.beans. MetaBean 
 sets the vector containing the original coordinates (instances of class
 Point) for the inputs
   setOutlierFactor(double)   - 
Method in class weka.filters.unsupervised.attribute. InterquartileRange 
 Sets the factor for determining the thresholds for outliers.
   setOutput(PrintWriter)   - 
Method in class weka.datagenerators. DataGenerator 
 Sets the print writer.
   setOutputCenterFile(File)   - 
Method in class weka.clusterers. XMeans 
 Sets file to write the list of centers to.
   setOutputClassification(boolean)   - 
Method in class weka.filters.supervised.attribute. AddClassification 
 Set whether the classification of the classifier is output.
   setOutputDistribution(boolean)   - 
Method in class weka.filters.supervised.attribute. AddClassification 
 Set whether the Distribution of the classifier is output.
   setOutputErrorFlag(boolean)   - 
Method in class weka.filters.supervised.attribute. AddClassification 
 Set whether the classification of the classifier is output.
   setOutputFile(File)   - 
Method in class weka.experiment. CrossValidationResultProducer 
 Set the value of OutputFile.
   setOutputFile(File)   - 
Method in class weka.experiment. CSVResultListener 
 Set the value of OutputFile.
   setOutputFile(File)   - 
Method in class weka.experiment. RandomSplitResultProducer 
 Set the value of OutputFile.
   setOutputFilename(boolean)   - 
Method in class weka.core.converters. TextDirectoryLoader 
 Sets whether the filename will be stored as an extra attribute.
   setOutputFileName(String)   - 
Method in class weka.experiment. CSVResultListener 
 Set the value of OutputFileName.
   setOutputFilename(String)   - 
Method in class weka.gui. GenericPropertiesCreator 
 sets the file to output the properties for the GEO to
   setOutputFormat(int)   - 
Method in class weka.core. Debug.Clock 
 sets the format of the output
   setOutputFormatFromDialog()   - 
Method in class weka.gui.experiment. ResultsPanel 
 displays the Dialog for the output format and sets the chosen settings, 
 if the user approves.
   setOutputItemSets(boolean)   - 
Method in class weka.associations. Apriori 
 Sets whether itemsets are output as well
   setOutputOffsetMultiplier(boolean)   - 
Method in class weka.filters.unsupervised.attribute. InterquartileRange 
 Set whether an additional attribute "Offset" is generated per 
 Outlier/ExtremeValue attribute pair that lists the multiplier the value
 is off the median: value = median + 'multiplier' * IQR.
   setOutputPerClassInfoRetrievalStats(boolean)   - 
Method in class weka.gui.beans. IncrementalClassifierEvaluator 
 Set whether to output per-class information retrieval
 statistics (nominal class only).
   setOutputs(Vector)   - 
Method in class weka.gui.beans. MetaBean 
  
   setOutputTypes(String)   - 
Method in class weka.core. Debug.DBO 
 Switches the outputs on that are requested from the option O
   setOutputWordCounts(boolean)   - 
Method in class weka.filters.unsupervised.attribute. StringToWordVector 
 Sets whether output instances contain 0 or 1 indicating word
 presence, or word counts.
   setOverwriteWarning(boolean)   - 
Method in class weka.gui. ConverterFileChooser 
 Whether a warning is popped up if the file that is to be saved already
 exists (only save dialog).
   setOwner(CapabilitiesHandler)   - 
Method in class weka.core. Capabilities 
 sets the owner of this capabilities object
   setP(double)   - 
Method in class weka.classifiers. BVDecomposeSegCVSub 
 Set the proportion of instances that are common between two training sets
 used to train a classifier.
   setPadding(SelectedTag)   - 
Method in class weka.filters.unsupervised.attribute. Wavelet 
 Sets the type of Padding to use
   setPaint(Paint)   - 
Method in class weka.gui.visualize. PostscriptGraphics 
  
   setPaintMode()   - 
Method in class weka.gui.visualize. PostscriptGraphics 
 Not implemented
   setPanelHeight(int)   - 
Method in class weka.gui.boundaryvisualizer. RemoteBoundaryVisualizerSubTask 
 Set the height of the visualization
   setPanelWidth(int)   - 
Method in class weka.gui.boundaryvisualizer. RemoteBoundaryVisualizerSubTask 
 Set the width of the visualization
   setParameterDefs(ArrayList<Attribute>)   - 
Method in class weka.core.pmml. BuiltInArithmetic 
 Set the structure of the parameters that are expected as input by
 this function.
   setParameterDefs(ArrayList<Attribute>)   - 
Method in class weka.core.pmml. BuiltInMath 
 Set the structure of the parameters that are expected as input by
 this function.
   setParameterDefs(ArrayList<Attribute>)   - 
Method in class weka.core.pmml. BuiltInString 
 Set the structure of the parameters that are expected as input by
 this function.
   setParameterDefs(ArrayList<Attribute>)   - 
Method in class weka.core.pmml. DefineFunction 
 Set the structure of the parameters that are expected as input by
 this function.
   setParameterDefs(ArrayList<Attribute>)   - 
Method in class weka.core.pmml. Function 
 Set the structure of the parameters that are expected as input by
 this function.
   SetParent(int, int)   - 
Method in class weka.classifiers.bayes.net. ParentSet 
 sets index parent of parent specified by index
   setParent(ClusterGenerator)   - 
Method in class weka.datagenerators. ClusterDefinition 
 sets the parent datagenerator this cluster belongs to
   setParent(SubspaceCluster)   - 
Method in class weka.datagenerators.clusterers. SubspaceClusterDefinition 
 sets the parent datagenerator this cluster belongs to
   setParent(Container)   - 
Method in class weka.gui.arffviewer. ArffViewerMainPanel 
 sets the new parent frame
   setParent(Edge)   - 
Method in class weka.gui.treevisualizer. Node 
 Set the value of parent.
   setParentFrame(JFrame)   - 
Method in class weka.gui.beans. AssociatorCustomizer 
  
   setParentFrame(JFrame)   - 
Method in class weka.gui.beans. ClassAssignerCustomizer 
  
   setParentFrame(JFrame)   - 
Method in class weka.gui.beans. ClassifierCustomizer 
  
   setParentFrame(JFrame)   - 
Method in class weka.gui.beans. ClassValuePickerCustomizer 
  
   setParentFrame(JFrame)   - 
Method in class weka.gui.beans. ClustererCustomizer 
  
   setParentFrame(JFrame)   - 
Method in interface weka.gui.beans. CustomizerCloseRequester 
 A reference to the parent is passed in
   setParentFrame(JFrame)   - 
Method in class weka.gui.beans. FilterCustomizer 
  
   setParentFrame(JFrame)   - 
Method in class weka.gui.beans. LoaderCustomizer 
  
   setParentFrame(JFrame)   - 
Method in class weka.gui.beans. SaverCustomizer 
  
   setParentFrame(JFrame)   - 
Method in class weka.gui.beans. SerializedModelSaverCustomizer 
  
   setParentFrame(JFrame)   - 
Method in class weka.gui. SetInstancesPanel 
 Sets the frame, this panel resides in.
   setParentSeparator(MarginCalculator.JunctionTreeSeparator)   - 
Method in class weka.classifiers.bayes.net. MarginCalculator.JunctionTreeNode 
  
   setPassword(String)   - 
Method in interface weka.core.converters. DatabaseConverter 
  
   setPassword(String)   - 
Method in class weka.core.converters. DatabaseLoader 
 Sets user password for the database
   setPassword(String)   - 
Method in class weka.core.converters. DatabaseSaver 
 Sets the database password.
   setPassword(String)   - 
Method in class weka.experiment. DatabaseUtils 
 Set the database password.
   setPassword(String)   - 
Method in class weka.gui.sql. ConnectionPanel 
 sets the Password.
   setPattern(SelectedTag)   - 
Method in class weka.datagenerators.clusterers. BIRCHCluster 
 Sets the pattern type.
   setPercent(int)   - 
Method in class weka.filters.unsupervised.attribute. AddNoise 
 Sets the size of noise data, as a percentage of the original set.
   setPercent(double)   - 
Method in class weka.filters.unsupervised.attribute. RandomProjection 
 Sets the percent the attributes (dimensions) of the data should be reduced to
   setPercent()   - 
Method in class weka.gui.visualize. MatrixPanel 
 Calculates the percentage to resample
   setPercentage(double)   - 
Method in class weka.filters.supervised.instance. SMOTE 
 Sets the percentage of SMOTE instances to create.
   setPercentage(double)   - 
Method in class weka.filters.unsupervised.instance. RemovePercentage 
 Sets the percentage of intances to select.
   setPercentCompleted(int)   - 
Method in class weka.gui.boundaryvisualizer. RemoteResult 
 Set the progress for this row so far
   setPercentThreshold(int)   - 
Method in class weka.attributeSelection. SVMAttributeEval 
 Set the threshold below which percentage elimination reverts to
 constant elimination.
   setPercentToEliminatePerIteration(int)   - 
Method in class weka.attributeSelection. SVMAttributeEval 
 Set the percentage of attributes to eliminate per iteration
   setPerformPrediction(boolean)   - 
Method in class weka.filters.supervised.attribute. PLSFilter 
 Sets whether to update the class attribute with the predicted value.
   setPerformRanking(boolean)   - 
Method in class weka.attributeSelection. LinearForwardSelection 
 Perform initial ranking to select top-ranked attributes.
   setPerformRanking(boolean)   - 
Method in class weka.attributeSelection. SubsetSizeForwardSelection 
 Perform initial ranking to select top-ranked attributes.
   setPeriodicPruning(double)   - 
Method in class weka.filters.unsupervised.attribute. StringToWordVector 
 Sets the rate at which the dictionary is periodically pruned, as a 
 percentage of the dataset size.
   setPerturbationFraction(double)   - 
Method in class weka.datagenerators.classifiers.classification. Agrawal 
 Sets the perturbation fraction.
   setPivot(Instance)   - 
Method in class weka.core.neighboursearch.balltrees. BallNode 
 Sets the pivot/centre of this nodes
 ball.
   setPixHeight(double)   - 
Method in class weka.gui.boundaryvisualizer. RemoteBoundaryVisualizerSubTask 
 Set the height of a pixel
   setPixWidth(double)   - 
Method in class weka.gui.boundaryvisualizer. RemoteBoundaryVisualizerSubTask 
 Set the width of a pixel
   setPlotCompanion(Plot2DCompanion)   - 
Method in class weka.gui.visualize. Plot2D 
 Set a companion class.
   setPlotList(FastVector)   - 
Method in class weka.gui.visualize. LegendPanel 
 Set the list of plots to generate legend entries for
   setPlotName(String)   - 
Method in class weka.gui.visualize. PlotData2D 
 Set the name of this plot
   setPlotNameHTML(String)   - 
Method in class weka.gui.visualize. PlotData2D 
 Set the plot name for use in a tool tip text.
   setPlotTrainingData(boolean)   - 
Method in class weka.gui.boundaryvisualizer. BoundaryPanel 
 Set whether to superimpose the training data
 plot
   setPlus(int, int, double)   - 
Method in class weka.classifiers.functions.pace. PaceMatrix 
 Add a value to an element and reset the element
   setPlus(int, double)   - 
Method in class weka.core.matrix. DoubleVector 
 Adds a value to an element
   setPMMLVersion(Document)   - 
Method in class weka.classifiers.pmml.consumer. PMMLClassifier 
 Set the version of PMML used for this model.
   setPMMLVersion(Document)   - 
Method in interface weka.core.pmml. PMMLModel 
 Set the version of the PMML.
   setPoints(MiddleOutConstructor.TempNode, int, int, int[])   - 
Method in class weka.core.neighboursearch.balltrees. MiddleOutConstructor 
 Sets the points of an anchor node.
   setPointValue(int, double)   - 
Method in class weka.classifiers.functions.pace. DiscreteFunction 
 Sets a particular point value
   setPopulationSize(int)   - 
Method in class weka.attributeSelection. GeneticSearch 
 set the population size
   setPopulationSize(int)   - 
Method in class weka.attributeSelection. ScatterSearchV1 
 Set the population size
   setPopulationSize(int)   - 
Method in class weka.classifiers.bayes.net.search.global. GeneticSearch 
  
   setPopulationSize(int)   - 
Method in class weka.classifiers.bayes.net.search.local. GeneticSearch 
  
   setPopup(JPopupMenu)   - 
Method in class weka.gui. GenericObjectEditor.CapabilitiesFilterDialog 
 sets the JPopupMenu to display again after closing the dialog.
   setPosition(int, int, int)   - 
Method in class weka.classifiers.bayes.net. EditableBayesNet 
 set position of node
   setPosition(int, int, int, FastVector)   - 
Method in class weka.classifiers.bayes.net. EditableBayesNet 
 Set position of node.
   setPositiveIndex(int)   - 
Method in class weka.associations. FPGrowth 
 Set the index of the attribute value to consider as positive
 for binary attributes in normal dense instances.
   setPostProcessor(CheckScheme.PostProcessor)   - 
Method in class weka.core. CheckScheme 
 sets the PostProcessor to use
   setPostProcessor(CheckEstimator.PostProcessor)   - 
Method in class weka.estimators. CheckEstimator 
 sets the PostProcessor to use
   setPredTargetColumn(boolean)   - 
Method in class weka.experiment. ClassifierSplitEvaluator 
 Set the flag for prediction and target output.
   setPreferredScrollableViewportSize(Dimension)   - 
Method in class weka.gui. AttributeSelectionPanel 
  
   setPrefix(String)   - 
Method in class weka.gui.beans. SerializedModelSaver 
 Set the prefix to prepend to the model file names.
   setPreprocessing(SelectedTag)   - 
Method in class weka.filters.supervised.attribute. PLSFilter 
 Sets the type of preprocessing to use
   setPreprocessing(Filter)   - 
Method in class weka.filters.unsupervised.attribute. KernelFilter 
 Sets the filter to use for preprocessing (use the AllFilter for no 
 preprocessing)
   setPreserveInstancesOrder(boolean)   - 
Method in class weka.clusterers. SimpleKMeans 
 Sets whether order of instances must be preserved
   setPrintColNames(boolean)   - 
Method in class weka.experiment. ResultMatrix 
 sets whether the column names or numbers instead are printed.
   setPrintNewick(boolean)   - 
Method in class weka.clusterers. HierarchicalClusterer 
  
   setPrintRowNames(boolean)   - 
Method in class weka.experiment. ResultMatrix 
 sets whether the row names or numbers instead are printed
 deactivating automatically sets m_EnumerateColNames to TRUE.
   setPriorClass(SelectedTag)   - 
Method in class weka.classifiers.bayes. BayesianLogisticRegression 
 Set the type of prior to use.
   setPriors(Instances)   - 
Method in class weka.classifiers. Evaluation 
 Sets the class prior probabilities
   setProbabilityEstimates(boolean)   - 
Method in class weka.classifiers.functions. LibLINEAR 
 Returns whether probability estimates are generated instead of -1/+1 for
 classification problems.
   setProbabilityEstimates(boolean)   - 
Method in class weka.classifiers.functions. LibSVM 
 Returns whether probability estimates are generated instead of -1/+1 for 
 classification problems.
   setProcessed(boolean)   - 
Method in interface weka.clusterers.forOPTICSAndDBScan.DataObjects. DataObject 
 Marks this dataObject as processed
   setProcessed(boolean)   - 
Method in class weka.clusterers.forOPTICSAndDBScan.DataObjects. EuclidianDataObject 
 Marks this dataObject as processed
   setProcessed(boolean)   - 
Method in class weka.clusterers.forOPTICSAndDBScan.DataObjects. ManhattanDataObject 
 Marks this dataObject as processed
   setProjectionFilter(Filter)   - 
Method in class weka.classifiers.meta. RotationForest 
 Sets the filter used to project the data.
   setProlog(boolean)   - 
Method in class weka.core. OptionHandlerJavadoc 
 sets whether to add the "Valid options are..." prolog
   setProlog(boolean)   - 
Method in class weka.core. TechnicalInformationHandlerJavadoc 
 sets whether to add the "Valid options are..." prolog
   setProperty(String, String)   - 
Method in class weka.core. ProtectedProperties 
 Overrides a method to prevent the properties from being modified.
   setPropertyArray(Object)   - 
Method in class weka.experiment. Experiment 
 Sets the array of values to set the custom property to.
   setPropertyArray(Object)   - 
Method in class weka.experiment. RemoteExperiment 
 Sets the array of values to set the custom property to.
   setPropertyPath(PropertyNode[])   - 
Method in class weka.experiment. Experiment 
 Sets the path of properties taken to get to the custom property
 to iterate over.
   setPropertyPath(PropertyNode[])   - 
Method in class weka.experiment. RemoteExperiment 
 Sets the path of properties taken to get to the custom property
 to iterate over.
   setPruningMethod(SelectedTag)   - 
Method in class weka.classifiers.functions.supportVector. StringKernel 
 Sets the method used to for pruning.
   setPruningStrategy(SelectedTag)   - 
Method in class weka.classifiers.trees. BFTree 
 Sets the pruning strategy.
   setPruningType(SelectedTag)   - 
Method in class weka.classifiers.meta. RacedIncrementalLogitBoost 
 Set the pruning type
   setQuality(float)   - 
Method in class weka.gui.visualize. JPEGWriter 
 sets the quality the JPEG is saved in.
   setQuery(String)   - 
Method in class weka.core.converters. DatabaseLoader 
 Sets the query to execute against the database
   setQuery(String)   - 
Method in class weka.experiment. InstanceQuery 
 Set the query to execute against the database
   setQuery(String)   - 
Method in class weka.gui.sql. QueryPanel 
 sets the query in the textarea.
   setQueryPanel(QueryPanel)   - 
Method in class weka.gui.sql. ResultPanel 
 sets the QueryPanel to use for displaying the query
   setRaceType(SelectedTag)   - 
Method in class weka.attributeSelection. RaceSearch 
 Set the race type
   setRadius(double)   - 
Method in class weka.core.neighboursearch.balltrees. BallNode 
 Sets the radius of the node's 
 ball.
   setRandom(Random)   - 
Method in class weka.datagenerators. DataGenerator 
 Sets the random generator.
   setRandomize(boolean)   - 
Method in class weka.filters.unsupervised.attribute. PropositionalToMultiInstance 
 Sets whether the order of the generated data is randomized
   setRandomizeData(boolean)   - 
Method in class weka.experiment. RandomSplitResultProducer 
 Set to true if dataset is to be randomized
   setRandomOrder(boolean)   - 
Method in class weka.classifiers.bayes.net.search.global. K2 
 Set random order flag
   setRandomOrder(boolean)   - 
Method in class weka.classifiers.bayes.net.search.local. K2 
 Set random order flag
   setRandomSeed(long)   - 
Method in class weka.classifiers.functions. LeastMedSq 
 Set the seed for the random number generator
   setRandomSeed(int)   - 
Method in class weka.classifiers.functions. SMO 
 Set the value of randomSeed.
   setRandomSeed(int)   - 
Method in class weka.classifiers.mi. MISMO 
 Set the value of randomSeed.
   setRandomSeed(int)   - 
Method in class weka.classifiers.trees. ADTree 
 Sets random seed for a random walk.
   setRandomSeed(int)   - 
Method in class weka.core.neighboursearch.balltrees. MedianDistanceFromArbitraryPoint 
 Sets the seed for random number generator.
   setRandomSeed(int)   - 
Method in class weka.filters.supervised.instance. Resample 
 Sets the random number seed.
   setRandomSeed(int)   - 
Method in class weka.filters.supervised.instance. SMOTE 
 Sets the random number seed.
   setRandomSeed(int)   - 
Method in class weka.filters.supervised.instance. SpreadSubsample 
 Sets the random number seed.
   setRandomSeed(int)   - 
Method in class weka.filters.unsupervised.attribute. AddNoise 
 Sets the random number seed.
   setRandomSeed(long)   - 
Method in class weka.filters.unsupervised.attribute. RandomProjection 
 Sets the random seed of the random number generator
   setRandomSeed(int)   - 
Method in class weka.filters.unsupervised.instance. Randomize 
 Set the random number generator seed value.
   setRandomSeed(int)   - 
Method in class weka.filters.unsupervised.instance. Resample 
 Sets the random number seed.
   setRandomSeed(int)   - 
Method in class weka.filters.unsupervised.instance. ReservoirSample 
 Sets the random number seed.
   setRandomWidthFactor(double)   - 
Method in class weka.classifiers.meta. MultiClassClassifier 
 Sets the multiplier when generating random codes.
   setRangeCorrection(SelectedTag)   - 
Method in class weka.classifiers.meta. ThresholdSelector 
 Sets the confidence range correction mode used.
   setRanges(String)   - 
Method in class weka.core. Range 
 Sets the ranges from a string representation.
   setRanges(Range[])   - 
Method in class weka.filters.unsupervised.attribute. PartitionedMultiFilter 
 Sets the list of possible Ranges to choose from.
   setRank(double)   - 
Method in class weka.attributeSelection. LatentSemanticAnalysis 
 Sets the desired matrix rank (or coverage proportion) for feature-space reduction
   setRanking(boolean)   - 
Method in class weka.attributeSelection. AttributeSelection 
 produce a ranking (if possible with the set search and evaluator)
   setRanking(int[][])   - 
Method in class weka.experiment. ResultMatrix 
 sets the ranking data based on the wins
   setRawOutput(boolean)   - 
Method in class weka.experiment. CrossValidationResultProducer 
 Set to true if raw split evaluator output is to be saved
   setRawOutput(boolean)   - 
Method in class weka.experiment. RandomSplitResultProducer 
 Set to true if raw split evaluator output is to be saved
   setReachabilityDistance(double)   - 
Method in interface weka.clusterers.forOPTICSAndDBScan.DataObjects. DataObject 
 Sets a new reachability-distance for this dataObject
   setReachabilityDistance(double)   - 
Method in class weka.clusterers.forOPTICSAndDBScan.DataObjects. EuclidianDataObject 
 Sets a new reachability-distance for this dataObject
   setReachabilityDistance(double)   - 
Method in class weka.clusterers.forOPTICSAndDBScan.DataObjects. ManhattanDataObject 
 Sets a new reachability-distance for this dataObject
   setReachabilityDistanceColor(Color)   - 
Method in class weka.clusterers.forOPTICSAndDBScan.OPTICS_GUI. GraphPanel 
 Sets a new color for the reachabilityDistance
   setReadable(String)   - 
Method in class weka.core. Tag 
 Sets the string description of the Tag.
   setReadIncrementally(boolean)   - 
Method in class weka.gui. SetInstancesPanel 
 Sets whether or not instances should be read incrementally
 by the Loader.
   setReadOnly(boolean)   - 
Method in class weka.gui.arffviewer. ArffPanel 
 sets whether the model is read-only
   setReadOnly(boolean)   - 
Method in class weka.gui.arffviewer. ArffSortedTableModel 
 sets whether the model is read-only
   setReadOnly(boolean)   - 
Method in class weka.gui.arffviewer. ArffTable 
 sets whether the model is read-only
   setReadOnly(boolean)   - 
Method in class weka.gui.arffviewer. ArffTableModel 
 sets whether the model is read-only
   setReducedErrorPruning(boolean)   - 
Method in class weka.classifiers.rules. PART 
 Set the value of reducedErrorPruning.
   setReducedErrorPruning(boolean)   - 
Method in class weka.classifiers.trees. J48 
 Set the value of reducedErrorPruning.
   setRefer(String)   - 
Method in class weka.gui.treevisualizer. Node 
 Set the value of refer.
   setRefreshFreq(int)   - 
Method in class weka.gui.beans. StripChart 
 Set how often (in x axis points) to refresh the display
   setRegOptimizer(RegOptimizer)   - 
Method in class weka.classifiers.functions. SMOreg 
 sets the learning algorithm
   setRegressionTree(boolean)   - 
Method in class weka.classifiers.trees.m5. Rule 
 Set the value of regressionTree.
   setRegressionTree(boolean)   - 
Method in class weka.classifiers.trees.m5. RuleNode 
 Set the value of regressionTree.
   setRelabel(boolean)   - 
Method in class weka.classifiers.trees. J48graft 
 Set the value of relabelling.
   setRelation(String)   - 
Method in class weka.core. TestInstances 
 sets the name of the relation
   setRelationalClassFormat(Instances)   - 
Method in class weka.core. TestInstances 
 sets the structure for the relational class attribute
   setRelationalFormat(int, Instances)   - 
Method in class weka.core. TestInstances 
 sets the structure for the bags for the relational attribute
   setRelationForTableName(boolean)   - 
Method in class weka.core.converters. DatabaseSaver 
 En/Dis-ables that the relation name is used for the name of the table (default enabled).
   setRelationName(String)   - 
Method in class weka.core. Instances 
 Sets the relation's name.
   setRelationName(String)   - 
Method in class weka.datagenerators. DataGenerator 
 Sets the relation name the dataset should have.
   setRelationNameForFilename(boolean)   - 
Method in class weka.gui.beans. Saver 
 Set whether to use the relation name as the primary part
 of the filename.
   setRemoteHosts(DefaultListModel)   - 
Method in class weka.experiment. RemoteExperiment 
 Set the list of remote host names
   setRemoteHosts(Vector)   - 
Method in class weka.gui.boundaryvisualizer. BoundaryPanelDistributed 
 Set a list of host names of machines to distribute processing to
   setRemoveAllMissingCols(boolean)   - 
Method in class weka.associations. Apriori 
 Remove columns containing all missing values.
   setRemoveClassColumn(boolean)   - 
Method in class weka.experiment. DensityBasedClustererSplitEvaluator 
 Set whether the class column should be removed from the data.
   setRemovedPercentage(int)   - 
Method in class weka.classifiers.meta. RotationForest 
 Sets the percentage of instance to be removed
   setRemoveFilterName(boolean)   - 
Method in class weka.experiment. ResultMatrix 
 sets whether to remove the filter classname from the dataset name
   setRemoveFilterName(boolean)   - 
Method in class weka.gui.experiment. OutputFormatDialog 
 sets whether to remove the filter classname from the dataset name.
   setRemoveOldClass(boolean)   - 
Method in class weka.filters.supervised.attribute. AddClassification 
 Set whether the old class attribute is removed.
   setRemoveUnused(boolean)   - 
Method in class weka.filters.unsupervised.attribute. PartitionedMultiFilter 
 Sets whether unused attributes (ones that are not covered by any of the
 ranges) are removed from the output.
   setRenderingHint(RenderingHints.Key, Object)   - 
Method in class weka.gui.visualize. PostscriptGraphics 
  
   setRenderingHints(Map)   - 
Method in class weka.gui.visualize. PostscriptGraphics 
  
   setRepeatLiterals(boolean)   - 
Method in class weka.associations. Tertius 
 Set the value of repeatLiterals.
   setReplaceMissing(boolean)   - 
Method in class weka.filters.supervised.attribute. PLSFilter 
 Sets whether to replace missing values.
   setReplaceMissingValues(boolean)   - 
Method in class weka.filters.unsupervised.attribute. RandomProjection 
 Sets either to use replace missing values filter or not
   setReportFrequency(int)   - 
Method in class weka.attributeSelection. GeneticSearch 
 set how often reports are generated
   setRepulsion(double)   - 
Method in class weka.clusterers. CLOPE 
 set the repulsion
   setReset(boolean)   - 
Method in class weka.classifiers.functions. MultilayerPerceptron 
 This sets the network up to be able to reset itself with the current 
 settings and the learning rate at half of what it is currently.
   setReset(boolean)   - 
Method in class weka.gui.beans. ChartEvent 
 Set the reset flag
   setResult(Double)   - 
Method in class weka.core.mathematicalexpression. Parser 
 Sets the result of the evaluation.
   setResult(Boolean)   - 
Method in class weka.filters.unsupervised.instance.subsetbyexpression. Parser 
 Sets the result of the evaluation.
   setResultKeyFromDialog()   - 
Method in class weka.gui.experiment. ResultsPanel 
  
   setResultListener(ResultListener)   - 
Method in class weka.experiment. AveragingResultProducer 
 Sets the object to send results of each run to.
   setResultListener(ResultListener)   - 
Method in class weka.experiment. CrossValidationResultProducer 
 Sets the object to send results of each run to.
   setResultListener(ResultListener)   - 
Method in class weka.experiment. DatabaseResultProducer 
 Sets the object to send results of each run to.
   setResultListener(ResultListener)   - 
Method in class weka.experiment. Experiment 
 Sets the result listener where results will be sent.
   setResultListener(ResultListener)   - 
Method in class weka.experiment. LearningRateResultProducer 
 Sets the object to send results of each run to.
   setResultListener(ResultListener)   - 
Method in class weka.experiment. RandomSplitResultProducer 
 Sets the object to send results of each run to.
   setResultListener(ResultListener)   - 
Method in class weka.experiment. RemoteExperiment 
 Sets the result listener where results will be sent.
   setResultListener(ResultListener)   - 
Method in interface weka.experiment. ResultProducer 
 Sets the object to send results of each run to.
   setResultMatrix(ResultMatrix)   - 
Method in class weka.experiment. PairedTTester 
 Sets the matrix to use to produce the output.
   setResultMatrix(ResultMatrix)   - 
Method in interface weka.experiment. Tester 
 Sets the matrix to use to produce the output.
   setResultMatrix(Class)   - 
Method in class weka.gui.experiment. OutputFormatDialog 
 Sets the matrix to use as initial selected output format.
   setResultProducer(ResultProducer)   - 
Method in class weka.experiment. AveragingResultProducer 
 Set the ResultProducer.
   setResultProducer(ResultProducer)   - 
Method in class weka.experiment. DatabaseResultProducer 
 Set the ResultProducer.
   setResultProducer(ResultProducer)   - 
Method in class weka.experiment. Experiment 
 Set the result producer used for the current experiment.
   setResultProducer(ResultProducer)   - 
Method in class weka.experiment. LearningRateResultProducer 
 Set the ResultProducer.
   setResultProducer(ResultProducer)   - 
Method in class weka.experiment. RemoteExperiment 
 Set the result producer used for the current experiment.
   setResultsetKeyColumns(Range)   - 
Method in class weka.experiment. PairedTTester 
 Set the value of ResultsetKeyColumns.
   setResultsetKeyColumns(Range)   - 
Method in interface weka.experiment. Tester 
 Set the value of ResultsetKeyColumns.
   setResultsPanel(ResultsPanel)   - 
Method in class weka.gui.experiment. RunPanel 
 Sets the pointer to the results panel.
   setResultVector(FastVector)   - 
Method in class weka.clusterers.forOPTICSAndDBScan.OPTICS_GUI. GraphPanel 
 Sets a new resultVector
   setRetrieval(int)   - 
Method in class weka.core.converters. AbstractLoader 
 Sets the retrieval mode.
   setRetrieval(int)   - 
Method in class weka.core.converters. AbstractSaver 
 Sets the retrieval mode.
   setRetrieval(int)   - 
Method in interface weka.core.converters. Loader 
 Sets the retrieval mode.
   setRetrieval(int)   - 
Method in interface weka.core.converters. Saver 
 Sets the retrieval mode
   setRidge(double)   - 
Method in class weka.classifiers.functions. LinearRegression 
 Set the value of Ridge.
   setRidge(double)   - 
Method in class weka.classifiers.functions. Logistic 
 Sets the ridge in the log-likelihood.
   setRidge(double)   - 
Method in class weka.classifiers.functions. RBFNetwork 
 Sets the ridge value for logistic or linear regression.
   setRidge(double)   - 
Method in class weka.classifiers.mi. MILR 
 Sets the ridge in the log-likelihood.
   setRocAnalysis(boolean)   - 
Method in class weka.associations. Tertius 
 Set the value of rocAnalysis.
   setROCString(String)   - 
Method in class weka.gui.visualize. ThresholdVisualizePanel 
 Set the string with ROC area
   setRoot(boolean)   - 
Method in class weka.gui.treevisualizer. Node 
 Set the value of root.
   setRootNode(String)   - 
Method in class weka.core.xml. XMLDocument 
 sets the root node to use in the XML output.
   setRow(int, double[])   - 
Method in class weka.core. Matrix 
  Deprecated.  Sets a row of the matrix to the given row.
   setRowDimension(int)   - 
Method in class weka.classifiers.functions.pace. PaceMatrix 
 Set the row dimenion of the matrix
   setRowHidden(int, boolean)   - 
Method in class weka.experiment. ResultMatrix 
 sets the hidden status of the row (if the index is valid)
   setRowName(int, String)   - 
Method in class weka.experiment. ResultMatrix 
 sets the name of the row (if the index is valid)
   setRowNameWidth(int)   - 
Method in class weka.experiment. ResultMatrix 
 sets the width for the row names (0 = optimal)
   setRowNumber(int)   - 
Method in class weka.gui.boundaryvisualizer. RemoteBoundaryVisualizerSubTask 
 Set the row number for this sub task
   setRowOrder(int[])   - 
Method in class weka.experiment. ResultMatrix 
 sets the ordering of the rows, null means default
   setRsource(String)   - 
Method in class weka.gui.treevisualizer. Edge 
 Set the value of rsource.
   setRtarget(String)   - 
Method in class weka.gui.treevisualizer. Edge 
 Set the value of rtarget.
   setRuleset(FastVector)   - 
Method in class weka.classifiers.rules. RuleStats 
 Set the ruleset of the stats, overwriting the old one if any
   setRulesMustContain(String)   - 
Method in class weka.associations. FPGrowth 
 Set the comma separated list of items that rules
 must contain in order to be output.
   setRunColumn(int)   - 
Method in class weka.experiment. PairedTTester 
 Set the value of RunColumn.
   setRunColumn(int)   - 
Method in interface weka.experiment. Tester 
 Set the value of RunColumn.
   setRunLower(int)   - 
Method in class weka.experiment. Experiment 
 Set the lower run number for the experiment.
   setRunLower(int)   - 
Method in class weka.experiment. RemoteExperiment 
 Set the lower run number for the experiment.
   setRuns(int)   - 
Method in class weka.classifiers.bayes.net.search.global. GeneticSearch 
 Sets the number of runs
   setRuns(int)   - 
Method in class weka.classifiers.bayes.net.search.global. RepeatedHillClimber 
 Sets the number of runs
   setRuns(int)   - 
Method in class weka.classifiers.bayes.net.search.global. SimulatedAnnealing 
 Sets the m_nRuns.
   setRuns(int)   - 
Method in class weka.classifiers.bayes.net.search.global. TabuSearch 
 Sets the number of runs
   setRuns(int)   - 
Method in class weka.classifiers.bayes.net.search.local. GeneticSearch 
 Sets the number of runs
   setRuns(int)   - 
Method in class weka.classifiers.bayes.net.search.local. RepeatedHillClimber 
 Sets the number of runs
   setRuns(int)   - 
Method in class weka.classifiers.bayes.net.search.local. SimulatedAnnealing 
 Sets the m_nRuns.
   setRuns(int)   - 
Method in class weka.classifiers.bayes.net.search.local. TabuSearch 
 Sets the number of runs
   setRunUpper(int)   - 
Method in class weka.experiment. Experiment 
 Set the upper run number for the experiment.
   setRunUpper(int)   - 
Method in class weka.experiment. RemoteExperiment 
 Set the upper run number for the experiment.
   setSampleSize(int)   - 
Method in class weka.attributeSelection. ReliefFAttributeEval 
 Set the number of instances to sample for attribute estimation
   setSampleSize(int)   - 
Method in class weka.classifiers.functions. LeastMedSq 
 sets number of samples
   setSampleSize(int)   - 
Method in class weka.filters.unsupervised.instance. ReservoirSample 
 Sets the size of the subsample.
   setSampleSizePercent(double)   - 
Method in class weka.classifiers.meta. GridSearch 
 Sets the sample size for the initial grid search.
   setSampleSizePercent(double)   - 
Method in class weka.filters.supervised.instance. Resample 
 Sets the size of the subsample, as a percentage of the original set.
   setSampleSizePercent(double)   - 
Method in class weka.filters.unsupervised.instance. Resample 
 Sets the size of the subsample, as a percentage of the original set.
   setSaveDialogTitle(String)   - 
Method in class weka.gui.visualize. PrintableComponent 
 sets the title for the save dialog.
   setSaveDialogTitle(String)   - 
Method in interface weka.gui.visualize. PrintableHandler 
 sets the title for the save dialog
   setSaveDialogTitle(String)   - 
Method in class weka.gui.visualize. PrintablePanel 
 sets the title for the save dialog
   setSaveInstanceData(boolean)   - 
Method in class weka.classifiers.trees. ADTree 
 Sets whether the tree is to save instance data.
   setSaveInstanceData(boolean)   - 
Method in class weka.classifiers.trees. J48 
 Set whether instance data is to be saved.
   setSaveInstanceData(boolean)   - 
Method in class weka.classifiers.trees. J48graft 
 Set whether instance data is to be saved.
   setSaveInstanceData(boolean)   - 
Method in class weka.clusterers. Cobweb 
 Set the value of saveInstances.
   setSaveInstances(boolean)   - 
Method in class weka.classifiers.trees. M5P 
 Set whether to save instance data at each node in the
 tree for visualization purposes
   setSaverTemplate(Saver)   - 
Method in class weka.gui.beans. Saver 
 Set the loader to use
   setScale(double)   - 
Method in class weka.filters.unsupervised.attribute. Normalize 
 Sets the scaling factor.
   setScale(double, double)   - 
Method in class weka.gui.visualize. JComponentWriter 
 sets the scale factor - is ignored since we always create a screenshot!
   setScale(double, double)   - 
Method in class weka.gui.visualize. PrintableComponent 
 sets the scale factor.
   setScale(double, double)   - 
Method in interface weka.gui.visualize. PrintableHandler 
 sets the scale factor
   setScale(double, double)   - 
Method in class weka.gui.visualize. PrintablePanel 
 sets the scale factor
   setScalingEnabled(boolean)   - 
Method in class weka.gui.visualize. JComponentWriter 
 sets whether to enable scaling
   setScoreType(SelectedTag)   - 
Method in class weka.classifiers.bayes.net.search.local. LocalScoreSearchAlgorithm 
 set quality measure to be used in searching for networks.
   setSearch(ASSearch)   - 
Method in class weka.attributeSelection. AttributeSelection 
 set the search method
   setSearch(ASSearch)   - 
Method in class weka.attributeSelection. CheckAttributeSelection 
 Set the search method to test.
   setSearch(ASSearch)   - 
Method in class weka.classifiers.meta. AttributeSelectedClassifier 
 Sets the search method
   setSearch(ASSearch)   - 
Method in class weka.classifiers.rules. DecisionTable 
 Sets the search method to use
   setSearch(ASSearch)   - 
Method in class weka.classifiers.rules. DTNB 
 Sets the search method to use
   setSearch(ASSearch)   - 
Method in class weka.filters.supervised.attribute. AttributeSelection 
 Set search class
   setSearchAlgorithm(SearchAlgorithm)   - 
Method in class weka.classifiers.bayes. BayesNet 
 Set the SearchAlgorithm used in searching for network structures.
   setSearchBackwards(boolean)   - 
Method in class weka.attributeSelection. GreedyStepwise 
 Set whether to search backwards instead of forwards
   setSearchPath(SelectedTag)   - 
Method in class weka.classifiers.trees. ADTree 
 Sets the method of searching the tree for a new insertion.
   setSearchPercent(double)   - 
Method in class weka.attributeSelection. RandomSearch 
 set the percentage of the search space to consider
   setSearchString(String)   - 
Method in class weka.gui.arffviewer. ArffTable 
 sets the search string to look for in the table, NULL or "" disables
 the search
   setSearchTermination(int)   - 
Method in class weka.attributeSelection. BestFirst 
 Set the numnber of non-improving nodes to consider before terminating
 search.
   setSearchTermination(int)   - 
Method in class weka.attributeSelection. LinearForwardSelection 
 Set the numnber of non-improving nodes to consider before terminating
 search.
   setSecondValueIndex(String)   - 
Method in class weka.filters.unsupervised.attribute. MergeTwoValues 
 Sets index of the second value used.
   setSecondValueIndex(String)   - 
Method in class weka.filters.unsupervised.attribute. SwapValues 
 Sets index of the second value used.
   setSeed(int)   - 
Method in class weka.attributeSelection. AttributeSelection 
 set the seed for use in cross validation
   setSeed(int)   - 
Method in class weka.attributeSelection. CostSensitiveASEvaluation 
 Set the seed for random number generation.
   setSeed(int)   - 
Method in class weka.attributeSelection. GeneticSearch 
 set the seed for random number generation
   setSeed(int)   - 
Method in class weka.attributeSelection. OneRAttributeEval 
 Set the random number seed for cross validation
   setSeed(int)   - 
Method in class weka.attributeSelection. ReliefFAttributeEval 
 Set the random number seed for randomly sampling instances.
   setSeed(int)   - 
Method in class weka.attributeSelection. ScatterSearchV1 
 set the seed for random number generation
   setSeed(int)   - 
Method in class weka.attributeSelection. SubsetSizeForwardSelection 
 Seed for cross validation subset size determination.
   setSeed(int)   - 
Method in class weka.attributeSelection. WrapperSubsetEval 
 Set the seed to use for cross validation
   setSeed(int)   - 
Method in class weka.classifiers.bayes.net.search.global. GeneticSearch 
 Sets the random number seed
   setSeed(int)   - 
Method in class weka.classifiers.bayes.net.search.global. RepeatedHillClimber 
 Sets the random number seed
   setSeed(int)   - 
Method in class weka.classifiers.bayes.net.search.global. SimulatedAnnealing 
 Sets the random number seed
   setSeed(int)   - 
Method in class weka.classifiers.bayes.net.search.local. GeneticSearch 
 Sets the random number seed
   setSeed(int)   - 
Method in class weka.classifiers.bayes.net.search.local. RepeatedHillClimber 
 Sets the random number seed
   setSeed(int)   - 
Method in class weka.classifiers.bayes.net.search.local. SimulatedAnnealing 
 Sets the random number seed
   setSeed(int)   - 
Method in class weka.classifiers. BVDecompose 
 Sets the random number seed
   setSeed(int)   - 
Method in class weka.classifiers. BVDecomposeSegCVSub 
 Sets the random number seed
   setSeed(int)   - 
Method in class weka.classifiers.evaluation. EvaluationUtils 
 Sets the seed for randomization during cross-validation
   setSeed(int)   - 
Method in class weka.classifiers.functions. MultilayerPerceptron 
 This seeds the random number generator, that is used when a random
 number is needed for the network.
   setSeed(int)   - 
Method in class weka.classifiers.functions.supportVector. RegOptimizer 
 Sets the seed value for the random number generator
   setSeed(int)   - 
Method in class weka.classifiers.functions. VotedPerceptron 
 Set the value of Seed.
   setSeed(int)   - 
Method in class weka.classifiers.functions. Winnow 
 Set the value of Seed.
   setSeed(int)   - 
Method in class weka.classifiers.meta. MultiScheme 
 Sets the seed for random number generation.
   setSeed(int)   - 
Method in class weka.classifiers. RandomizableClassifier 
 Set the seed for random number generation.
   setSeed(int)   - 
Method in class weka.classifiers. RandomizableIteratedSingleClassifierEnhancer 
 Set the seed for random number generation.
   setSeed(int)   - 
Method in class weka.classifiers. RandomizableMultipleClassifiersCombiner 
 Set the seed for random number generation.
   setSeed(int)   - 
Method in class weka.classifiers. RandomizableSingleClassifierEnhancer 
 Set the seed for random number generation.
   setSeed(long)   - 
Method in class weka.classifiers.rules. ConjunctiveRule 
 sets the seed for randomizing the data
   setSeed(long)   - 
Method in class weka.classifiers.rules. JRip 
 Sets the seed value to use in randomizing the data
   setSeed(int)   - 
Method in class weka.classifiers.rules. PART 
 Set the value of Seed.
   setSeed(int)   - 
Method in class weka.classifiers.rules. Ridor 
  
   setSeed(int)   - 
Method in class weka.classifiers.trees. J48 
 Set the value of Seed.
   setSeed(int)   - 
Method in class weka.classifiers.trees. RandomForest 
 Set the seed for random number generation.
   setSeed(int)   - 
Method in class weka.classifiers.trees. RandomTree 
 Set the seed for random number generation.
   setSeed(int)   - 
Method in class weka.classifiers.trees. REPTree 
 Set the value of Seed.
   setSeed(int)   - 
Method in class weka.clusterers. RandomizableClusterer 
 Set the seed for random number generation.
   setSeed(int)   - 
Method in class weka.clusterers. RandomizableDensityBasedClusterer 
 Set the seed for random number generation.
   setSeed(int)   - 
Method in class weka.clusterers. RandomizableSingleClustererEnhancer 
 Set the seed for random number generation.
   setSeed(long)   - 
Method in class weka.core. Debug.Random 
 Sets the seed of this random number generator using a single long seed.
   setSeed(int)   - 
Method in class weka.core.neighboursearch.balltrees. MiddleOutConstructor 
 Sets the seed for random number generator 
 (that is used for selecting the first anchor 
 point randomly).
   setSeed(int)   - 
Method in interface weka.core. Randomizable 
 Set the seed for random number generation.
   setSeed(int)   - 
Method in class weka.core. TestInstances 
 sets the seed value for the random number generator
   setSeed(int)   - 
Method in class weka.datagenerators. DataGenerator 
 Sets the random number seed.
   setSeed(long)   - 
Method in class weka.filters.supervised.attribute. ClassOrder 
 Set randomization seed
   setSeed(long)   - 
Method in class weka.filters.supervised.instance. StratifiedRemoveFolds 
 Sets the random number seed for shuffling the dataset.
   setSeed(int)   - 
Method in class weka.filters.unsupervised.attribute. PropositionalToMultiInstance 
 Sets the new seed for randomizing the order of the generated data
   setSeed(int)   - 
Method in class weka.filters.unsupervised.attribute. RandomSubset 
 Set the seed value for the random number generator.
   setSeed(long)   - 
Method in class weka.filters.unsupervised.instance. RemoveFolds 
 Sets the random number seed for shuffling the dataset.
   setSeed(int)   - 
Method in class weka.gui.beans. CrossValidationFoldMaker 
 Set the seed
   setSeed(int)   - 
Method in class weka.gui.beans. TrainTestSplitMaker 
 Set the random seed
   setSeed(int)   - 
Method in interface weka.gui.boundaryvisualizer. DataGenerator 
 Set a seed for random number generation (if needed).
   setSeed(int)   - 
Method in class weka.gui.boundaryvisualizer. KDDataGenerator 
 Initializes a new random number generator using the
 supplied seed.
   setSelectedAttributes(boolean[])   - 
Method in class weka.gui. AttributeSelectionPanel 
 Set the selected attributes in the widget.
   setSelectedColumn(int)   - 
Method in class weka.gui.arffviewer. ArffTable 
 sets the selected column
   setSelectedRange(String)   - 
Method in class weka.filters.unsupervised.attribute. RELAGGS 
 Set the range of attributes to process.
   setSelectedRange(String)   - 
Method in class weka.filters.unsupervised.attribute. StringToWordVector 
 Set the value of m_SelectedRange.
   setSelectionThreshold(double)   - 
Method in class weka.attributeSelection. RaceSearch 
 Set the threshold by which the AttributeSelection module can discard
 attributes.
   setSeparatingThreshold(double)   - 
Method in class weka.classifiers.functions.pace. ChisqMixture 
 Sets the separating threshold value
   setSeparatingThreshold(double)   - 
Method in class weka.classifiers.functions.pace. NormalMixture 
 Sets the separating threshold value
   setSeperator(String)   - 
Method in class weka.gui. HierarchyPropertyParser 
 Set the seperator between levels.
   setSequentialAttIndex(boolean)   - 
Method in class weka.classifiers.lazy. LBR.Indexes 
 A Sequential Attribute index is all those Attributes that are set to the specified value placed in a sequential array.
   setSequentialDataset(boolean)   - 
Method in class weka.classifiers.lazy. LBR.Indexes 
 Sets both the Instance and Attribute indexes to a specified value
   setSequentialInstanceIndex(boolean)   - 
Method in class weka.classifiers.lazy. LBR.Indexes 
 A Sequential Instance index is all those Instances that are set to the specified value placed in a sequential array.
   setSerializedClassifierFile(File)   - 
Method in class weka.filters.supervised.attribute. AddClassification 
 Sets the file pointing to a serialized, trained classifier.
   setShape(int)   - 
Method in class weka.gui.treevisualizer. Node 
 Set the value of shape.
   setShapes(FastVector)   - 
Method in class weka.gui.visualize. VisualizePanel 
 This will set the shapes for the instances.
   setShapeSize(int[])   - 
Method in class weka.gui.visualize. PlotData2D 
 Set the shape sizes for the plot data
   setShapeSize(FastVector)   - 
Method in class weka.gui.visualize. PlotData2D 
 Set the shape sizes for the plot data
   setShapeType(int[])   - 
Method in class weka.gui.visualize. PlotData2D 
 Set the shape type for the plot data
   setShapeType(FastVector)   - 
Method in class weka.gui.visualize. PlotData2D 
 Set the shape type for the plot data
   setShowAttBars(boolean)   - 
Method in class weka.gui.visualize. VisualizePanel 
 Set whether the attribute bars should be shown or not.
   setShowAverage(boolean)   - 
Method in class weka.experiment. ResultMatrix 
 sets whether to display the average per column or not
   setShowAverage(boolean)   - 
Method in class weka.gui.experiment. OutputFormatDialog 
 sets whether the average for each column is displayed.
   setShowClassPanel(boolean)   - 
Method in class weka.gui.visualize. VisualizePanel 
 Set whether the class panel should be shown or not.
   setShowCoreDistances(boolean)   - 
Method in class weka.clusterers.forOPTICSAndDBScan.OPTICS_GUI. GraphPanel 
 Sets the flag for showCoreDistances
   setShowGUI(boolean)   - 
Method in class weka.clusterers. OPTICS 
 Sets the flag for displaying the GUI.
   setShowReachabilityDistances(boolean)   - 
Method in class weka.clusterers.forOPTICSAndDBScan.OPTICS_GUI. GraphPanel 
 Sets the flag for showReachabilityDistances
   setShowStdDev(boolean)   - 
Method in class weka.experiment. ResultMatrix 
 sets whether to display the std deviations or not
   setShowStdDev(boolean)   - 
Method in class weka.experiment. ResultMatrixSignificance 
 sets whether to display the std deviations or not - always false!
   setShowStdDevs(boolean)   - 
Method in class weka.experiment. PairedTTester 
 Set whether standard deviations are displayed or not.
   setShowStdDevs(boolean)   - 
Method in interface weka.experiment. Tester 
 Set whether standard deviations are displayed or not.
   setShrinkage(double)   - 
Method in class weka.classifiers.meta. AdditiveRegression 
 Set the shrinkage parameter
   setShrinkage(double)   - 
Method in class weka.classifiers.meta. LogitBoost 
 Set the value of Shrinkage.
   setShrinking(boolean)   - 
Method in class weka.classifiers.functions. LibSVM 
 whether to use the shrinking heuristics
   setShuffle(int)   - 
Method in class weka.classifiers.rules. Ridor 
  
   setSigma(int)   - 
Method in class weka.attributeSelection. ReliefFAttributeEval 
 Sets the sigma value.
   setSigma(double)   - 
Method in class weka.classifiers.functions.supportVector. Puk 
 Sets the sigma value.
   setSignificance(int, int, int)   - 
Method in class weka.experiment. ResultMatrix 
 sets the significance at the given position (if the position is valid)
   setSignificanceLevel(double)   - 
Method in class weka.associations. Apriori 
 Set the value of significanceLevel.
   setSignificanceLevel(double)   - 
Method in class weka.attributeSelection. RaceSearch 
 Sets the significance level to use
   setSignificanceLevel(double)   - 
Method in class weka.experiment. PairedTTester 
 Set the value of SignificanceLevel.
   setSignificanceLevel(double)   - 
Method in interface weka.experiment. Tester 
 Set the value of SignificanceLevel.
   setSignificanceWidth(int)   - 
Method in class weka.experiment. ResultMatrix 
 sets the width for the significance (0 = optimal)
   setSilent(boolean)   - 
Method in class weka.core. AllJavadoc 
 sets whether to suppress output in the console
   setSilent(boolean)   - 
Method in class weka.core. Check 
 Set slient mode, i.e., no output at all to stdout
   setSilent(boolean)   - 
Method in class weka.core. Javadoc 
 sets whether to suppress output in the console
   setSilent(boolean)   - 
Method in class weka.estimators. CheckEstimator 
 Set slient mode, i.e., no output at all to stdout
   setSIndex(int)   - 
Method in class weka.gui.visualize. VisualizePanel 
 Set the shape for creating splits.
   setSingle(String)   - 
Method in class weka.gui. ResultHistoryPanel 
 Sets the single-click display to view the named result.
   setSingleIndex(String)   - 
Method in class weka.core. SingleIndex 
 Sets the index from a string representation.
   setSize(int)   - 
Method in class weka.core.matrix. DoubleVector 
 Sets the size of the vector
   setSize(int)   - 
Method in class weka.core.matrix. IntVector 
 Sets the size of the vector.
   setSize(int, int)   - 
Method in class weka.experiment. ResultMatrix 
 clears the content of the matrix and sets the new size
   setSizePer(double)   - 
Method in class weka.classifiers.trees. BFTree 
 Set training set size.
   setSizePer(double)   - 
Method in class weka.classifiers.trees. SimpleCart 
 Set training set size.
   setSkipIdentical(boolean)   - 
Method in class weka.core.neighboursearch. LinearNNSearch 
 Sets the property to skip identical instances (with distance zero from 
 the target) from the set of neighbours returned.
   setSmoothing(boolean)   - 
Method in class weka.classifiers.trees.m5. Rule 
 Smooth predictions
   setSmoothingParameter(double)   - 
Method in class weka.classifiers.bayes. ComplementNaiveBayes 
 Sets the smoothing value used to avoid zero WordGivenClass probabilities
   setSMOReg(SMOreg)   - 
Method in class weka.classifiers.functions.supportVector. RegOptimizer 
 sets the parent SVM
   setSort(boolean)   - 
Method in class weka.filters.unsupervised.attribute. AddValues 
 Sets whether the labels are sorted.
   setSortColumn(int)   - 
Method in class weka.experiment. PairedTTester 
 Set the column to sort on, -1 means the default sorting.
   setSortColumn(int)   - 
Method in interface weka.experiment. Tester 
 Set the column to sort on, -1 means the default sorting.
   setSource(File)   - 
Method in class weka.core.converters. AbstractFileLoader 
 Resets the Loader object and sets the source of the data set to be 
 the supplied File object.
   setSource(File)   - 
Method in class weka.core.converters. AbstractLoader 
 Default implementation throws an IOException.
   setSource(InputStream)   - 
Method in class weka.core.converters. AbstractLoader 
 Default implementation throws an IOException.
   setSource(URL)   - 
Method in class weka.core.converters. ArffLoader 
 Resets the Loader object and sets the source of the data set to be
 the supplied url.
   setSource(InputStream)   - 
Method in class weka.core.converters. ArffLoader 
 Resets the Loader object and sets the source of the data set to be
 the supplied InputStream.
   setSource(File)   - 
Method in class weka.core.converters. C45Loader 
 Resets the Loader object and sets the source of the data set to be 
 the supplied File object.
   setSource(InputStream)   - 
Method in class weka.core.converters. CSVLoader 
 Resets the Loader object and sets the source of the data set to be 
 the supplied Stream object.
   setSource(File)   - 
Method in class weka.core.converters. CSVLoader 
 Resets the Loader object and sets the source of the data set to be 
 the supplied File object.
   setSource(String, String, String)   - 
Method in class weka.core.converters. DatabaseLoader 
 Sets the database url, user and pw
   setSource(String)   - 
Method in class weka.core.converters. DatabaseLoader 
 Sets the database url
   setSource()   - 
Method in class weka.core.converters. DatabaseLoader 
 Sets the database url using the DatabaseUtils file
   setSource(URL)   - 
Method in class weka.core.converters. LibSVMLoader 
 Resets the Loader object and sets the source of the data set to be 
 the supplied url.
   setSource(InputStream)   - 
Method in class weka.core.converters. LibSVMLoader 
 Resets the Loader object and sets the source of the data set to be 
 the supplied InputStream.
   setSource(File)   - 
Method in interface weka.core.converters. Loader 
 Resets the Loader object and sets the source of the data set to be 
 the supplied File object.
   setSource(InputStream)   - 
Method in interface weka.core.converters. Loader 
 Resets the Loader object and sets the source of the data set to be 
 the supplied InputStream.
   setSource(InputStream)   - 
Method in class weka.core.converters. SerializedInstancesLoader 
 Resets the Loader object and sets the source of the data set to be 
 the supplied InputStream.
   setSource(URL)   - 
Method in class weka.core.converters. SVMLightLoader 
 Resets the Loader object and sets the source of the data set to be 
 the supplied url.
   setSource(InputStream)   - 
Method in class weka.core.converters. SVMLightLoader 
 Resets the Loader object and sets the source of the data set to be 
 the supplied InputStream.
   setSource(File)   - 
Method in class weka.core.converters. TextDirectoryLoader 
 Resets the Loader object and sets the source of the data set to be 
 the supplied File object.
   setSource(File)   - 
Method in class weka.core.converters. XRFFLoader 
 Resets the Loader object and sets the source of the data set to be 
 the supplied File object.
   setSource(URL)   - 
Method in class weka.core.converters. XRFFLoader 
 Resets the Loader object and sets the source of the data set to be 
 the supplied url.
   setSource(InputStream)   - 
Method in class weka.core.converters. XRFFLoader 
 Resets the Loader object and sets the source of the data set to be 
 the supplied InputStream.
   setSource(Node)   - 
Method in class weka.gui.treevisualizer. Edge 
 Set the value of source.
   setSourceCode(Classifier)   - 
Method in class weka.classifiers. CheckSource 
 Sets the class to test.
   setSourceCode(Filter)   - 
Method in class weka.filters. CheckSource 
 Sets the class to test.
   setSparseData(boolean)   - 
Method in class weka.experiment. InstanceQuery 
 Sets whether data should be encoded as sparse instances
   setSplitByDataSet(boolean)   - 
Method in class weka.experiment. RemoteExperiment 
 Set whether sub experiments are to be created on the basis of
 data set.
   setSplitEvaluator(SplitEvaluator)   - 
Method in class weka.experiment. CrossValidationResultProducer 
 Set the SplitEvaluator.
   setSplitEvaluator(SplitEvaluator)   - 
Method in class weka.experiment. RandomSplitResultProducer 
 Set the SplitEvaluator.
   setSplitOnResiduals(boolean)   - 
Method in class weka.classifiers.trees. LMT 
 Set the value of splitOnResiduals.
   setSplitPoint(Instances)   - 
Method in class weka.classifiers.trees.j48. BinC45Split 
 Sets split point to greatest value in given data smaller or equal to
 old split point.
   setSplitPoint(Instances)   - 
Method in class weka.classifiers.trees.j48. C45Split 
 Sets split point to greatest value in given data smaller or equal to
 old split point.
   setSplitPoint(double)   - 
Method in class weka.filters.unsupervised.instance. RemoveWithValues 
 Split point to be used for selection on numeric attribute.
   setStartEndIndices(int, int)   - 
Method in class weka.core.neighboursearch.balltrees. BallNode 
 Sets the the start and end index of the
 portion of the master index array that is
 assigned to this node.
   setStartPoint(int)   - 
Method in class weka.attributeSelection. RankSearch 
 Set the point at which to start evaluating the ranking
   setStartSequentially(boolean)   - 
Method in class weka.gui.beans. FlowRunner 
 Set whether to launch Startable beans one after the other
 or all in parallel.
   setStartSet(String)   - 
Method in class weka.attributeSelection. BestFirst 
 Sets a starting set of attributes for the search.
   setStartSet(String)   - 
Method in class weka.attributeSelection. GeneticSearch 
 Sets a starting set of attributes for the search.
   setStartSet(String)   - 
Method in class weka.attributeSelection. GreedyStepwise 
 Sets a starting set of attributes for the search.
   setStartSet(String)   - 
Method in class weka.attributeSelection. LinearForwardSelection 
 Sets a starting set of attributes for the search.
   setStartSet(String)   - 
Method in class weka.attributeSelection. RandomSearch 
 Sets a starting set of attributes for the search.
   setStartSet(String)   - 
Method in class weka.attributeSelection. Ranker 
 Sets a starting set of attributes for the search.
   setStartSet(String)   - 
Method in interface weka.attributeSelection. StartSetHandler 
 Sets a starting set of attributes for the search.
   setStatic()   - 
Method in class weka.gui.beans. BeanVisual 
 Set the static version of the icon
   setStatus(int)   - 
Method in class weka.gui.beans. IncrementalClassifierEvent 
 Set the status
   setStatus(int)   - 
Method in class weka.gui.beans. InstanceEvent 
 Set the status
   setStatusFrequency(int)   - 
Method in class weka.gui.beans. IncrementalClassifierEvaluator 
 Set how often progress is reported to the status bar.
   setStatusMessage(String)   - 
Method in class weka.experiment. TaskStatusInfo 
 Set the status message.
   setStdDev(int, int, double)   - 
Method in class weka.experiment. ResultMatrix 
 sets the std deviation at the given position (if the position is valid)
   setStdDevPrec(int)   - 
Method in class weka.experiment. ResultMatrix 
 sets the precision for the standard deviation
   setStdDevPrec(int)   - 
Method in class weka.gui.experiment. OutputFormatDialog 
 Sets the precision of the std.
   setStdDevWidth(int)   - 
Method in class weka.experiment. ResultMatrix 
 sets the width for the std dev (0 = optimal)
   setStemmer(String)   - 
Method in class weka.core.stemmers. SnowballStemmer 
 sets the stemmer with the given name, e.g., "porter".
   setStemmer(Stemmer)   - 
Method in class weka.filters.unsupervised.attribute. StringToWordVector 
 the stemming algorithm to use, null means no stemming at all (i.e., the
 NullStemmer is used).
   setStepSize(int)   - 
Method in class weka.attributeSelection. RankSearch 
 Set the number of attributes to add from the rankining
 in each iteration
   setStepSize(int)   - 
Method in class weka.experiment. LearningRateResultProducer 
 Set the value of StepSize.
   setStopwords(File)   - 
Method in class weka.filters.unsupervised.attribute. StringToWordVector 
 sets the file containing the stopwords, null or a directory unset the
 stopwords.
   setStringAttributes(String)   - 
Method in class weka.core.converters. CSVLoader 
 Sets the attribute range to be forced to type string.
   setStroke(Stroke)   - 
Method in class weka.gui.visualize. PostscriptGraphics 
  
   setStructure(Instances)   - 
Method in class weka.core.converters. AbstractSaver 
 Sets the strcuture of the instances for the first step of incremental saving.
   setStructure(Instances)   - 
Method in class weka.gui.beans. IncrementalClassifierEvent 
 Set the instances structure
   setStructure(Instances)   - 
Method in class weka.gui.beans. InstanceEvent 
 Set the instances structure
   setSubFlow(Vector)   - 
Method in class weka.gui.beans. MetaBean 
  
   setSubFlowPreview(ImageIcon)   - 
Method in class weka.gui.beans. MetaBean 
  
   setSubsequenceLength(int)   - 
Method in class weka.classifiers.functions.supportVector. StringKernel 
 Sets the length of the subsequence.
   setSubsetEvaluator(ASEvaluation)   - 
Method in class weka.attributeSelection. FilteredSubsetEval 
 Set the subset evaluator to use
   setSubsetSizeEvaluator(ASEvaluation)   - 
Method in class weka.attributeSelection. SubsetSizeForwardSelection 
 Set the subset evaluator to use for subset size determination.
   setSubSpaceSize(double)   - 
Method in class weka.classifiers.meta. RandomSubSpace 
 Sets the size of each subSpace, as a percentage of the training set size.
   setSubtreeRaising(boolean)   - 
Method in class weka.classifiers.trees. J48 
 Set the value of subtreeRaising.
   setSubtreeRaising(boolean)   - 
Method in class weka.classifiers.trees. J48graft 
 Set the value of subtreeRaising.
   setSummary(int[][], int[][])   - 
Method in class weka.experiment. ResultMatrix 
 sets the non-significant and significant wins of the resultsets
   setSuppressErrorMessage(boolean)   - 
Method in class weka.classifiers.functions. SimpleLinearRegression 
 Turn off the error message that is reported when no useful attribute is found.
   setSVMType(SelectedTag)   - 
Method in class weka.classifiers.functions. LibLINEAR 
 Sets type of SVM (default SVMTYPE_L2)
   setSVMType(SelectedTag)   - 
Method in class weka.classifiers.functions. LibSVM 
 Sets type of SVM (default SVMTYPE_C_SVC)
   setSymbols(HashMap)   - 
Method in class weka.core.mathematicalexpression. Parser 
 Sets the variable - value relation to use.
   setSymbols(HashMap)   - 
Method in class weka.filters.unsupervised.instance.subsetbyexpression. Parser 
 Sets the variable - value relation to use.
   setTableName(String)   - 
Method in class weka.core.converters. DatabaseSaver 
 Sets the table's name.
   setTabuList(int)   - 
Method in class weka.classifiers.bayes.net.search.global. TabuSearch 
 Sets the Tabu List length.
   setTabuList(int)   - 
Method in class weka.classifiers.bayes.net.search.local. TabuSearch 
 Sets the Tabu List length.
   setTarget(Object)   - 
Method in class weka.gui. PropertySheetPanel 
 Sets a new target object for customisation.
   setTarget(Node)   - 
Method in class weka.gui.treevisualizer. Edge 
 Set the value of target.
   setTargetClass(int)   - 
Method in class weka.classifiers.bayes. DMNBtext.DNBBinary 
 Sets the Target Class
   setTaskResult(Object)   - 
Method in class weka.experiment. TaskStatusInfo 
 Set the returnable result for this task..
   setTestBaseFromDialog()   - 
Method in class weka.gui.experiment. ResultsPanel 
  
   setTestEvaluator(boolean)   - 
Method in class weka.attributeSelection. CheckAttributeSelection 
 Sets whether the evaluator or the search method is being tested.
   setTestSet(DataSetEvent)   - 
Method in class weka.gui.beans. BatchClassifierEvent 
 Set the test set
   setText(String)   - 
Method in class weka.gui.beans. BeanVisual 
 Set the label for the visual.
   setTFTransform(boolean)   - 
Method in class weka.filters.unsupervised.attribute. StringToWordVector 
 Sets whether if the word frequencies should be transformed into
  log(1+fij) where fij is the frequency of word i in document(instance) j.
   setThreshold(double)   - 
Method in class weka.attributeSelection. GreedyStepwise 
 Set the threshold by which the AttributeSelection module can discard
 attributes.
   setThreshold(double)   - 
Method in class weka.attributeSelection. RaceSearch 
 Sets the threshold for comparisons
   setThreshold(double)   - 
Method in interface weka.attributeSelection. RankedOutputSearch 
 Sets a threshold by which attributes can be discarded from the
 ranking.
   setThreshold(double)   - 
Method in class weka.attributeSelection. Ranker 
 Set the threshold by which the AttributeSelection module can discard
 attributes.
   setThreshold(double)   - 
Method in class weka.attributeSelection. ScatterSearchV1 
 Set the treshold
   setThreshold(double)   - 
Method in class weka.attributeSelection. WrapperSubsetEval 
 Set the value of the threshold for repeating cross validation
   setThreshold(double)   - 
Method in class weka.classifiers.bayes. BayesianLogisticRegression 
 Set the threshold to use.
   setThreshold(double)   - 
Method in class weka.classifiers.functions. PaceRegression 
 Set threshold for the olsc estimator
   setThreshold(double)   - 
Method in class weka.classifiers.functions. Winnow 
 Set the value of Threshold.
   setThreshold(double)   - 
Method in class weka.filters.unsupervised.instance. RemoveMisclassified 
 Sets the threshold for the max error when predicting a numeric class.
   setTimes(int, int, double)   - 
Method in class weka.classifiers.functions.pace. PaceMatrix 
 Multiply a value with an element and reset the element
   setTimes(int, double)   - 
Method in class weka.core.matrix. DoubleVector 
 Multiplies a value to an element
   setTokenizer(Tokenizer)   - 
Method in class weka.filters.unsupervised.attribute. StringToWordVector 
 the tokenizer algorithm to use.
   setTolerance(double)   - 
Method in class weka.classifiers.bayes. BayesianLogisticRegression 
 Set the tolerance value
   setTolerance(double)   - 
Method in class weka.classifiers.functions.supportVector. RegSMOImproved 
 sets the tolerance
   setToleranceParameter(double)   - 
Method in class weka.attributeSelection. SVMAttributeEval 
 Set the value of T for SMO
   setToleranceParameter(double)   - 
Method in class weka.classifiers.functions. SMO 
 Set the value of tolerance parameter.
   setToleranceParameter(double)   - 
Method in class weka.classifiers.mi. MISMO 
 Set the value of tolerance parameter.
   setTop(double)   - 
Method in class weka.gui.treevisualizer. Node 
 Set the value of top.
   setTrainingData(Instances)   - 
Method in class weka.gui.boundaryvisualizer. BoundaryPanel 
 Set the training data to use
   setTrainingTime(int)   - 
Method in class weka.classifiers.functions. MultilayerPerceptron 
 Set the number of training epochs to perform.
   setTrainIterations(int)   - 
Method in class weka.classifiers. BVDecompose 
 Sets the maximum number of boost iterations
   setTrainPercent(double)   - 
Method in class weka.experiment. RandomSplitResultProducer 
 Set the value of TrainPercent.
   setTrainPercent(double)   - 
Method in class weka.gui.beans. TrainTestSplitMaker 
 Set the percentage of data to be in the training portion of the split
   setTrainPoolSize(int)   - 
Method in class weka.classifiers. BVDecompose 
 Set the number of instances in the training pool.
   setTrainSet(DataSetEvent)   - 
Method in class weka.gui.beans. BatchClassifierEvent 
 Set the training set
   setTrainSize(int)   - 
Method in class weka.classifiers. BVDecomposeSegCVSub 
 Set the training size.
   setTransactionsMustContain(String)   - 
Method in class weka.associations. FPGrowth 
 Set the comma separated list of items that transactions
 must contain in order to be considered for large
 item sets and rules.
   setTransform(AffineTransform)   - 
Method in class weka.gui.visualize. PostscriptGraphics 
  
   setTransformAllValues(boolean)   - 
Method in class weka.filters.supervised.attribute. NominalToBinary 
 Sets whether all nominal values are transformed into new attributes, not
 just if there are more than 2.
   setTransformAllValues(boolean)   - 
Method in class weka.filters.unsupervised.attribute. NominalToBinary 
 Sets whether all nominal values are transformed into new attributes, not
 just if there are more than 2.
   setTransformBackToOriginal(boolean)   - 
Method in class weka.attributeSelection. PrincipalComponents 
 Sets whether the data should be transformed back to the original
 space
   setTransformMethod(SelectedTag)   - 
Method in class weka.classifiers.mi. SimpleMI 
 Set the method used in transformation.
   setTranslation(double)   - 
Method in class weka.filters.unsupervised.attribute. Normalize 
 Sets the translation.
   setTraversal(SelectedTag)   - 
Method in class weka.classifiers.meta. GridSearch 
 Sets the type of traversal for the grid.
   setTrimingThreshold(double)   - 
Method in class weka.classifiers.functions.pace. ChisqMixture 
 Sets the triming thresholding value.
   setTrimingThreshold(double)   - 
Method in class weka.classifiers.functions.pace. NormalMixture 
 Sets the triming thresholding value.
   setTrueNegative(double)   - 
Method in class weka.classifiers.evaluation. TwoClassStats 
 Sets the number of negative instances predicted as negative
   setTruePositive(double)   - 
Method in class weka.classifiers.evaluation. TwoClassStats 
 Sets the number of positive instances predicted as positive
   setTStart(double)   - 
Method in class weka.classifiers.bayes.net.search.global. SimulatedAnnealing 
 Sets the m_fTStart.
   setTStart(double)   - 
Method in class weka.classifiers.bayes.net.search.local. SimulatedAnnealing 
 Sets the m_fTStart.
   setType(SelectedTag)   - 
Method in class weka.attributeSelection. LinearForwardSelection 
 Set the type
   setType(SelectedTag)   - 
Method in class weka.attributeSelection. SubsetSizeForwardSelection 
 Set the type
   setType(int)   - 
Method in class weka.classifiers.functions.neural. NeuralConnection 
  
   setUndoEnabled(boolean)   - 
Method in interface weka.core. Undoable 
 sets whether undo support is enabled
   setUndoEnabled(boolean)   - 
Method in class weka.gui.arffviewer. ArffPanel 
 sets whether undo support is enabled
   setUndoEnabled(boolean)   - 
Method in class weka.gui.arffviewer. ArffSortedTableModel 
 sets whether undo support is enabled
   setUndoEnabled(boolean)   - 
Method in class weka.gui.arffviewer. ArffTableModel 
 sets whether undo support is enabled
   setUnpruned(boolean)   - 
Method in class weka.classifiers.rules. PART 
 Set the value of unpruned.
   setUnpruned(boolean)   - 
Method in class weka.classifiers.trees. J48 
 Set the value of unpruned.
   setUnpruned(boolean)   - 
Method in class weka.classifiers.trees. J48graft 
 Set the value of unpruned.
   setUnpruned(boolean)   - 
Method in class weka.classifiers.trees.m5. M5Base 
 Use unpruned tree/rules
   setUnpruned(boolean)   - 
Method in class weka.classifiers.trees.m5. Rule 
 Use unpruned tree/rules
   setupAttribLists()   - 
Method in class weka.gui.visualize. MatrixPanel 
 Sets up the UI's attributes lists
   setUpBoundaryPanel()   - 
Method in class weka.gui.boundaryvisualizer. BoundaryVisualizer 
 Sets up the BoundaryPanel object so that it is ready for plotting.
   setUpComboBoxes(Instances)   - 
Method in class weka.gui.visualize. ThresholdVisualizePanel 
 This overloads VisualizePanel's setUpComboBoxes to add 
 ActionListeners to watch for when the X/Y Axis comboboxes
 are changed.
   setUpComboBoxes(Instances)   - 
Method in class weka.gui.visualize. VisualizePanel 
 initializes the comboboxes based on the data
   setUpdateIncrementalClassifier(boolean)   - 
Method in class weka.gui.beans. Classifier 
 Set whether an incremental classifier will be updated on the
 incoming instance stream.
   setUpFile()   - 
Method in class weka.gui.beans. LoaderCustomizer 
  
   setUpFile()   - 
Method in class weka.gui.beans. SaverCustomizer 
 Sets up dialog for saving instances in a file
   setUpFile()   - 
Method in class weka.gui.beans. SerializedModelSaverCustomizer 
 Sets up dialog for saving models to a file
   SetupModePanel   - Class in  weka.gui.experiment  This panel switches between simple and advanced experiment setup panels.   SetupModePanel()   - 
Constructor for class weka.gui.experiment. SetupModePanel 
 Creates the setup panel with no initial experiment.
   SetupPanel   - Class in  weka.gui.experiment  This panel controls the configuration of an experiment.   SetupPanel(Experiment)   - 
Constructor for class weka.gui.experiment. SetupPanel 
 Creates the setup panel with the supplied initial experiment.
   SetupPanel()   - 
Constructor for class weka.gui.experiment. SetupPanel 
 Creates the setup panel with no initial experiment.
   setUpper(int)   - 
Method in class weka.core. Range 
 Sets the value of "last".
   setUpper(int)   - 
Method in class weka.core. SingleIndex 
 Sets the value of "last".
   setUpperBoundMinSupport(double)   - 
Method in class weka.associations. Apriori 
 Set the value of upperBoundMinSupport.
   setUpperBoundMinSupport(double)   - 
Method in class weka.associations. FPGrowth 
 Set the value of upperBoundMinSupport.
   setUpperSize(int)   - 
Method in class weka.experiment. LearningRateResultProducer 
 Set the value of UpperSize.
   setUpVisualizableInstances(Instances)   - 
Static method in class weka.gui.explorer. ClassifierPanel 
 Sets up the structure for the visualizable instances.
   setUpVisualizableInstances(Instances, ClusterEvaluation)   - 
Static method in class weka.gui.explorer. ClustererPanel 
 Sets up the structure for the visualizable instances.
   setURL(String)   - 
Method in class weka.core.converters. ArffLoader 
 Set the url to load from
   setUrl(String)   - 
Method in interface weka.core.converters. DatabaseConverter 
  
   setUrl(String)   - 
Method in class weka.core.converters. DatabaseLoader 
 Sets the database URL
   setUrl(String)   - 
Method in class weka.core.converters. DatabaseSaver 
 Sets the database URL.
   setURL(String)   - 
Method in class weka.core.converters. LibSVMLoader 
 Set the url to load from.
   setURL(String)   - 
Method in class weka.core.converters. SVMLightLoader 
 Set the url to load from.
   setURL(String)   - 
Method in interface weka.core.converters. URLSourcedLoader 
 Set the url to load from
   setURL(String)   - 
Method in class weka.core.converters. XRFFLoader 
 Set the url to load from
   setURL(String)   - 
Method in class weka.gui.sql. ConnectionPanel 
 sets the URL.
   setUseADTree(boolean)   - 
Method in class weka.classifiers.bayes. BayesNet 
 Set whether ADTree structure is used or not
   setUseAIC(boolean)   - 
Method in class weka.classifiers.functions. SimpleLogistic 
 Set the value of useAIC.
   setUseAIC(boolean)   - 
Method in class weka.classifiers.trees. FT 
 Set the value of useAIC.
   setUseAIC(boolean)   - 
Method in class weka.classifiers.trees.lmt. LogisticBase 
 Set the value of useAIC.
   setUseAIC(boolean)   - 
Method in class weka.classifiers.trees. LMT 
 Set the value of useAIC.
   setUseArcReversal(boolean)   - 
Method in class weka.classifiers.bayes.net.search.global. HillClimber 
 set use the arc reversal operation
   setUseArcReversal(boolean)   - 
Method in class weka.classifiers.bayes.net.search.local. HillClimber 
 set use the arc reversal operation
   setUseBetterEncoding(boolean)   - 
Method in class weka.filters.supervised.attribute. Discretize 
 Sets whether better encoding is to be used for MDL.
   setUseCpuTime(boolean)   - 
Method in class weka.core. Debug.Clock 
 enables/disables the use of CPU time (if measurement of CPU time is 
 available).
   setUseCrossOver(boolean)   - 
Method in class weka.classifiers.bayes.net.search.global. GeneticSearch 
  
   setUseCrossOver(boolean)   - 
Method in class weka.classifiers.bayes.net.search.local. GeneticSearch 
  
   setUseCrossValidation(boolean)   - 
Method in class weka.classifiers.functions. SimpleLogistic 
 Set the value of useCrossValidation.
   setUseCustomDimensions(boolean)   - 
Method in class weka.gui.visualize. JComponentWriter 
 sets whether to use custom dimensions for the image
   setUseEqualFrequency(boolean)   - 
Method in class weka.classifiers.meta. RegressionByDiscretization 
 Set the value of UseEqualFrequency.
   setUseEqualFrequency(boolean)   - 
Method in class weka.filters.unsupervised.attribute. Discretize 
 Set the value of UseEqualFrequency.
   setUseEqualFrequency(boolean)   - 
Method in class weka.filters.unsupervised.attribute. PKIDiscretize 
 Set the value of UseEqualFrequency.
   setUseErrorRate(boolean)   - 
Method in class weka.classifiers.trees. BFTree 
 Set if use error rate in internal cross-validation.
   setUseGini(boolean)   - 
Method in class weka.classifiers.trees. BFTree 
 Set if use Gini index as splitting criterion.
   setUseIBk(boolean)   - 
Method in class weka.classifiers.rules. DecisionTable 
 Sets whether IBk should be used instead of the majority class
   setUseK2Prior(boolean)   - 
Method in class weka.classifiers.bayes.net.estimate. BMAEstimator 
 Sets the UseK2Prior.
   setUseK2Prior(boolean)   - 
Method in class weka.classifiers.bayes.net.estimate. MultiNomialBMAEstimator 
 Sets the UseK2Prior.
   setUseKDTree(boolean)   - 
Method in class weka.clusterers. XMeans 
 Sets whether to use the KDTree or not.
   setUseKernelEstimator(boolean)   - 
Method in class weka.classifiers.bayes. NaiveBayes 
 Sets if kernel estimator is to be used.
   setUseKononenko(boolean)   - 
Method in class weka.filters.supervised.attribute. Discretize 
 Sets whether Kononenko's MDL criterion is to be used.
   setUseLaplace(boolean)   - 
Method in class weka.classifiers.bayes. AODEsr 
 Sets if laplace correction is to be used.
   setUseLaplace(boolean)   - 
Method in class weka.classifiers.trees. J48 
 Set the value of useLaplace.
   setUseLaplace(boolean)   - 
Method in class weka.classifiers.trees. J48graft 
 Set the value of useLaplace.
   setUseLeastValues(boolean)   - 
Method in class weka.filters.unsupervised.instance. RemoveFrequentValues 
 Sets whether to use values with least or most instances
   setUseLowerOrder(boolean)   - 
Method in class weka.classifiers.functions.supportVector. PolyKernel 
 Sets whether to use lower-order terms.
   setUseMEstimates(boolean)   - 
Method in class weka.classifiers.bayes. AODE 
 Sets if m-estimates is to be used.
   setUseMissing(boolean)   - 
Method in class weka.filters.unsupervised.attribute. AddNoise 
 Sets the flag if missing values are treated as extra values.
   setUseMutation(boolean)   - 
Method in class weka.classifiers.bayes.net.search.global. GeneticSearch 
  
   setUseMutation(boolean)   - 
Method in class weka.classifiers.bayes.net.search.local. GeneticSearch 
  
   setUseNormalization(boolean)   - 
Method in class weka.classifiers.functions.supportVector. StringKernel 
 Sets whether to use normalization.
   setUseOneSE(boolean)   - 
Method in class weka.classifiers.trees. BFTree 
 Set if use the 1SE rule to choose final model.
   setUseOneSE(boolean)   - 
Method in class weka.classifiers.trees. SimpleCart 
 Set if use the 1SE rule to choose final model.
   setUseORForMustContainList(boolean)   - 
Method in class weka.associations. FPGrowth 
 Set whether to use OR rather than AND when considering
 must contain lists.
   setUsePairwiseCoupling(boolean)   - 
Method in class weka.classifiers.meta. MultiClassClassifier 
 Set whether to use pairwise coupling with 1-vs-1 
 classification to improve probability estimates.
   setUseProb(boolean)   - 
Method in class weka.classifiers.bayes.net.search.global. GlobalScoreSearchAlgorithm 
  
   setUsePropertyIterator(boolean)   - 
Method in class weka.experiment. Experiment 
 Sets whether the custom property iterator should be used.
   setUsePropertyIterator(boolean)   - 
Method in class weka.experiment. RemoteExperiment 
 Sets whether the custom property iterator should be used.
   setUsePrune(boolean)   - 
Method in class weka.classifiers.trees. SimpleCart 
 Set if use minimal cost-complexity pruning.
   setUsePruning(boolean)   - 
Method in class weka.classifiers.rules. JRip 
 Sets whether pruning is performed
   setUser(String)   - 
Method in interface weka.core.converters. DatabaseConverter 
  
   setUser(String)   - 
Method in class weka.core.converters. DatabaseLoader 
 Sets the database user
   setUser(String)   - 
Method in class weka.core.converters. DatabaseSaver 
 Sets the database user.
   setUser(String)   - 
Method in class weka.gui.sql. ConnectionPanel 
 sets the User.
   setUseRelativePath(boolean)   - 
Method in class weka.core.converters. AbstractFileLoader 
 Set whether to use relative rather than absolute paths
   setUseRelativePath(boolean)   - 
Method in class weka.core.converters. AbstractFileSaver 
 Set whether to use relative rather than absolute paths
   setUseRelativePath(boolean)   - 
Method in interface weka.core.converters. FileSourcedConverter 
 Set whether to use relative rather than absolute paths
   setUseRelativePath(boolean)   - 
Method in class weka.gui.beans. SerializedModelSaver 
 Set whether to use relative paths for the directory.
   setUseResampling(boolean)   - 
Method in class weka.classifiers.meta. AdaBoostM1 
 Set resampling mode
   setUseResampling(boolean)   - 
Method in class weka.classifiers.meta. LogitBoost 
 Set resampling mode
   setUseResampling(boolean)   - 
Method in class weka.classifiers.meta. RacedIncrementalLogitBoost 
 Set resampling mode
   setUsername(String)   - 
Method in class weka.experiment. DatabaseUtils 
 Set the database username.
   setUserOptions(String[])   - 
Method in class weka.classifiers.functions.supportVector. KernelEvaluation 
 sets the option the user supplied for the kernel
   setUserOptions(String[])   - 
Method in class weka.core. CheckOptionHandler 
 Sets the user-supplied options (creates a copy)
   setUseStars(boolean)   - 
Method in class weka.core. AllJavadoc 
 sets whether to prefix the Javadoc with "*"
   setUseStars(boolean)   - 
Method in class weka.core. Javadoc 
 sets whether to prefix the Javadoc with "*"
   setUseStoplist(boolean)   - 
Method in class weka.filters.unsupervised.attribute. StringToWordVector 
 Sets whether if the words that are on a stoplist are to be ignored (The
 stop list is in weka.core.StopWords).
   setUseSupervisedDiscretization(boolean)   - 
Method in class weka.classifiers.bayes. NaiveBayes 
 Set whether supervised discretization is to be used.
   setUseSupervisedDiscretization(boolean)   - 
Method in class weka.classifiers.bayes. NaiveBayesUpdateable 
 Set whether supervised discretization is to be used.
   setUseTournamentSelection(boolean)   - 
Method in class weka.classifiers.bayes.net.search.global. GeneticSearch 
  
   setUseTournamentSelection(boolean)   - 
Method in class weka.classifiers.bayes.net.search.local. GeneticSearch 
  
   setUseTraining(boolean)   - 
Method in class weka.attributeSelection. ClassifierSubsetEval 
 Set if training data is to be used instead of hold out/test data
   setUseTree(boolean)   - 
Method in class weka.classifiers.trees.m5. Rule 
 Use an m5 tree rather than generate rules
   setUseUnsmoothed(boolean)   - 
Method in class weka.classifiers.trees.m5. M5Base 
 Use unsmoothed predictions
   setUseVariant1(boolean)   - 
Method in class weka.classifiers.functions.supportVector. RegSMOImproved 
 Sets whether to use variant 1
   setValidating(boolean)   - 
Method in class weka.core.xml. XMLDocument 
 sets whether to use a validating parser or not. 
 Note: this does clear the current DOM document!
   setValidating(boolean)   - 
Method in class weka.core.xml. XMLOptions 
 sets whether to use a validating parser or not.
   setValidationChunkSize(int)   - 
Method in class weka.classifiers.meta. RacedIncrementalLogitBoost 
 Set the validation chunk size
   setValidationSetSize(int)   - 
Method in class weka.classifiers.functions. MultilayerPerceptron 
 This will set the size of the validation set.
   setValidationThreshold(int)   - 
Method in class weka.classifiers.functions. MultilayerPerceptron 
 This sets the threshold to use for when validation testing is being done.
   setValue(double)   - 
Method in class weka.classifiers.trees.adtree. PredictionNode 
 Sets the prediction value of the node.
   setValue(int, double)   - 
Method in class weka.core. BinarySparseInstance 
 Sets a specific value in the instance to the given value 
 (internal floating-point format).
   setValue(int, double)   - 
Method in class weka.core. Instance 
 Sets a specific value in the instance to the given value 
 (internal floating-point format).
   setValue(int, String)   - 
Method in class weka.core. Instance 
 Sets a value of a nominal or string attribute to the given
 value.
   setValue(Attribute, double)   - 
Method in class weka.core. Instance 
 Sets a specific value in the instance to the given value
 (internal floating-point format).
   setValue(Attribute, String)   - 
Method in class weka.core. Instance 
 Sets a value of an nominal or string attribute to the given
 value.
   setValue(Object, PropertyPath.Path, Object)   - 
Static method in class weka.core. PropertyPath 
 set the given value specified by the given path in the object
   setValue(Object, String, Object)   - 
Static method in class weka.core. PropertyPath 
 set the given value specified by the given path in the object
   setValue(int, double)   - 
Method in class weka.core. SparseInstance 
 Sets a specific value in the instance to the given value 
 (internal floating-point format).
   setValue(TechnicalInformation.Field, String)   - 
Method in class weka.core. TechnicalInformation 
 sets the value for the given field, overwrites any previously existing one.
   setValue(Object)   - 
Method in class weka.gui. CostMatrixEditor 
 Sets the value of the CostMatrix to be edited.
   setValue(Object)   - 
Method in class weka.gui. GenericArrayEditor 
 Sets the current object array.
   setValue(Object)   - 
Method in class weka.gui. GenericObjectEditor 
 Sets the current Object.
   setValue(Object)   - 
Method in class weka.gui. SimpleDateFormatEditor 
 Sets the value of the date format to be edited.
   setValueAt(Object, int, int)   - 
Method in class weka.gui.arffviewer. ArffTableModel 
 sets the value in the cell at columnIndex and rowIndex to aValue.
   setValueAt(Object, int, int, boolean)   - 
Method in class weka.gui.arffviewer. ArffTableModel 
 sets the value in the cell at columnIndex and rowIndex to aValue.
   setValueAt(Object, int, int)   - 
Method in class weka.gui. SortedTableModel 
 Sets the value in the cell at columnIndex and rowIndex to aValue.
   setValueAt(Object, int, int)   - 
Method in class weka.gui.sql. ResultSetTableModel 
 sets the value in the cell at columnIndex and rowIndex to aValue.
   setValueIndex(int)   - 
Method in class weka.filters.unsupervised.attribute. MakeIndicator 
 Sets index of the indicator value.
   setValueIndices(String)   - 
Method in class weka.filters.unsupervised.attribute. MakeIndicator 
 Sets indices of the indicator values.
   setValueIndicesArray(int[])   - 
Method in class weka.filters.unsupervised.attribute. MakeIndicator 
 Set which attributes are to be deleted (or kept if invert is true)
   setValuesList(String)   - 
Method in class weka.datagenerators.clusterers. SubspaceClusterDefinition 
 Sets the ranges for each attribute.
   setValuesList(String, double[], double[], String)   - 
Method in class weka.datagenerators.clusterers. SubspaceClusterDefinition 
 Sets the ranges for each attribute.
   setValuesOutput(SelectedTag)   - 
Method in class weka.associations. Tertius 
 Set the value of valuesOutput.
   setValueSparse(int, double)   - 
Method in class weka.core. BinarySparseInstance 
 Sets a specific value in the instance to the given value 
 (internal floating-point format).
   setValueSparse(int, double)   - 
Method in class weka.core. Instance 
 Sets a specific value in the instance to the given value 
 (internal floating-point format).
   setValueSparse(int, double)   - 
Method in class weka.core. SparseInstance 
 Sets a specific value in the instance to the given value 
 (internal floating-point format).
   setVarianceCovered(double)   - 
Method in class weka.attributeSelection. PrincipalComponents 
 Sets the amount of variance to account for when retaining
 principal components
   setVarianceCovered(double)   - 
Method in class weka.filters.unsupervised.attribute. PrincipalComponents 
 Sets the amount of variance to account for when retaining
 principal components.
   setVerbose(boolean)   - 
Method in class weka.associations. Apriori 
 Sets verbose mode
   setVerbose(boolean)   - 
Method in class weka.attributeSelection. ExhaustiveSearch 
 set whether or not to output new best subsets as the search proceeds
   setVerbose(boolean)   - 
Method in class weka.attributeSelection. LinearForwardSelection 
 Set whether verbose output should be generated.
   setVerbose(boolean)   - 
Method in class weka.attributeSelection. RandomSearch 
 set whether or not to output new best subsets as the search proceeds
   setVerbose(boolean)   - 
Method in class weka.attributeSelection. SubsetSizeForwardSelection 
 Set whether verbose output should be generated.
   setVerbose(boolean)   - 
Method in class weka.classifiers.meta. Dagging 
 Set the verbose state.
   setVerboseOn()   - 
Method in class weka.core. Debug.DBO 
 Set the verbose on flag on
   setVerticalAdjustment(int)   - 
Method in class weka.clusterers.forOPTICSAndDBScan.OPTICS_GUI. GraphPanel 
 Sets a new value for the vertical verticalAdjustment
   setVisible(boolean)   - 
Method in class weka.gui. Main 
 Shows or hides this component depending on the value of parameter b.
   setVisible(boolean)   - 
Method in class weka.gui.sql. SqlViewerDialog 
 displays the dialog if TRUE
   setVisual(BeanVisual)   - 
Method in class weka.gui.beans. AbstractDataSink 
 Set the visual for this data source
   setVisual(BeanVisual)   - 
Method in class weka.gui.beans. AbstractDataSource 
 Set the visual for this data source
   setVisual(BeanVisual)   - 
Method in class weka.gui.beans. AbstractEvaluator 
 Set the visual
   setVisual(BeanVisual)   - 
Method in class weka.gui.beans. AbstractTestSetProducer 
 Set the visual for this bean
   setVisual(BeanVisual)   - 
Method in class weka.gui.beans. AbstractTrainAndTestSetProducer 
 Set the visual for this bean
   setVisual(BeanVisual)   - 
Method in class weka.gui.beans. AbstractTrainingSetProducer 
 Set the visual for this bean
   setVisual(BeanVisual)   - 
Method in class weka.gui.beans. Associator 
 Sets the visual appearance of this wrapper bean
   setVisual(BeanVisual)   - 
Method in class weka.gui.beans. ClassAssigner 
  
   setVisual(BeanVisual)   - 
Method in class weka.gui.beans. Classifier 
 Sets the visual appearance of this wrapper bean
   setVisual(BeanVisual)   - 
Method in class weka.gui.beans. ClassValuePicker 
  
   setVisual(BeanVisual)   - 
Method in class weka.gui.beans. Clusterer 
 Sets the visual appearance of this wrapper bean
   setVisual(BeanVisual)   - 
Method in class weka.gui.beans. CostBenefitAnalysis 
  
   setVisual(BeanVisual)   - 
Method in class weka.gui.beans. DataVisualizer 
 Set the visual appearance of this bean
   setVisual(BeanVisual)   - 
Method in class weka.gui.beans. Filter 
 Set the visual appearance of this bean
   setVisual(BeanVisual)   - 
Method in class weka.gui.beans. GraphViewer 
 Set the visual appearance of this bean
   setVisual(BeanVisual)   - 
Method in class weka.gui.beans. InstanceStreamToBatchMaker 
 Sets the visual appearance of this wrapper bean
   setVisual(BeanVisual)   - 
Method in class weka.gui.beans. MetaBean 
 Sets the visual appearance of this wrapper bean
   setVisual(BeanVisual)   - 
Method in class weka.gui.beans. ModelPerformanceChart 
 Set the visual appearance of this bean
   setVisual(BeanVisual)   - 
Method in class weka.gui.beans. PredictionAppender 
 Set the visual for this data source
   setVisual(BeanVisual)   - 
Method in class weka.gui.beans. SerializedModelSaver 
 Set the visual for this data source.
   setVisual(BeanVisual)   - 
Method in class weka.gui.beans. StripChart 
 Set the visual appearance of this bean
   setVisual(BeanVisual)   - 
Method in class weka.gui.beans. TextViewer 
 Describe  setVisual  method here.
   setVisual(BeanVisual)   - 
Method in interface weka.gui.beans. Visible 
 Set a new visual representation
   setVoteFlag(boolean)   - 
Method in class weka.datagenerators.classifiers.classification. RDG1 
 Sets the vote flag.
   setWeight(int)   - 
Method in class weka.classifiers.bayes. AODE 
 Sets the weight for m-estimate
   setWeight(double)   - 
Method in class weka.core. Attribute 
 Sets the new attribute's weight
   setWeight(double)   - 
Method in class weka.core. Instance 
 Sets the weight of an instance.
   setWeightByConfidence(boolean)   - 
Method in class weka.classifiers.misc. VFI 
 Set weighting by confidence
   setWeightByDistance(boolean)   - 
Method in class weka.attributeSelection. ReliefFAttributeEval 
 Set the nearest neighbour weighting method
   setWeightingDimensions(boolean[])   - 
Method in interface weka.gui.boundaryvisualizer. DataGenerator 
 Set the dimensions to be used in computing a weight for
 each instance generated
   setWeightingDimensions(boolean[])   - 
Method in class weka.gui.boundaryvisualizer. KDDataGenerator 
 Set which dimensions to use when computing a weight for the next
 instance to generate
   setWeightingKernel(int)   - 
Method in class weka.classifiers.lazy. LWL 
 Sets the kernel weighting method to use.
   setWeightingValues(double[])   - 
Method in interface weka.gui.boundaryvisualizer. DataGenerator 
 Set the values of the dimensions (chosen via setWeightingDimensions)
 to be used when computing instance weights
   setWeightingValues(double[])   - 
Method in class weka.gui.boundaryvisualizer. KDDataGenerator 
 Set the values for the weighting dimensions to be used when computing
 the weight for the next instance to be generated
   setWeightMethod(SelectedTag)   - 
Method in class weka.classifiers.mi. MIWrapper 
 The new method for weighting the instances.
   setWeightMethod(SelectedTag)   - 
Method in class weka.filters.unsupervised.attribute. MultiInstanceToPropositional 
 The new method for weighting the instances.
   setWeights(String)   - 
Method in class weka.classifiers.functions. LibLINEAR 
 Sets the parameters C of class i to weight[i]*C (default 1).
   setWeights(String)   - 
Method in class weka.classifiers.functions. LibSVM 
 Sets the parameters C of class i to weight[i]*C, for C-SVC (default 1).
   setWeightThreshold(int)   - 
Method in class weka.classifiers.meta. AdaBoostM1 
 Set weight threshold
   setWeightThreshold(int)   - 
Method in class weka.classifiers.meta. LogitBoost 
 Set weight thresholding
   setWeightTrimBeta(double)   - 
Method in class weka.classifiers.functions. SimpleLogistic 
 Set the value of weightTrimBeta.
   setWeightTrimBeta(double)   - 
Method in class weka.classifiers.trees. FT 
 Set the value of weightTrimBeta.
   setWeightTrimBeta(double)   - 
Method in class weka.classifiers.trees.lmt. LogisticBase 
 Sets the option "weightTrimBeta".
   setWeightTrimBeta(double)   - 
Method in class weka.classifiers.trees. LMT 
 Set the value of weightTrimBeta.
   setWholeDataErr(boolean)   - 
Method in class weka.classifiers.rules. Ridor 
  
   setWindowSize(int)   - 
Method in class weka.classifiers.lazy. IBk 
 Sets the maximum number of instances allowed in the training
 pool.
   setWords(String)   - 
Method in class weka.core. CheckScheme 
 Sets the comma-separated list of words to use for generating strings.
   setWords(String)   - 
Method in class weka.core. TestInstances 
 Sets the comma-separated list of words to use for generating strings.
   setWordSeparators(String)   - 
Method in class weka.core. CheckScheme 
 sets the word separators (chars) to use for assembling strings.
   setWordSeparators(String)   - 
Method in class weka.core. TestInstances 
 sets the word separators (chars) to use for assembling strings.
   setWordsToKeep(int)   - 
Method in class weka.filters.unsupervised.attribute. StringToWordVector 
 Sets the number of words (per class if there is a class attribute
 assigned) to attempt to keep.
   setWordwrap(boolean)   - 
Method in class weka.gui. LogWindow 
 toggles the wordwrap 
 override wordwrap from: 
 http://forum.java.sun.com/thread.jspa?threadID=498535&messageID=2356174
   setWrappedAlgorithm(Object)   - 
Method in class weka.gui.beans. Associator 
 Sets the algorithm (associator) for this bean
   setWrappedAlgorithm(Object)   - 
Method in class weka.gui.beans. Classifier 
 Sets the algorithm (classifier) for this bean
   setWrappedAlgorithm(Object)   - 
Method in class weka.gui.beans. Clusterer 
 Sets the algorithm (clusterer) for this bean
   setWrappedAlgorithm(Object)   - 
Method in class weka.gui.beans. Filter 
 Set the filter to be wrapped by this bean
   setWrappedAlgorithm(Object)   - 
Method in class weka.gui.beans. Loader 
 Set the loader
   setWrappedAlgorithm(Object)   - 
Method in class weka.gui.beans. Saver 
 Set the saver
   setWrappedAlgorithm(Object)   - 
Method in interface weka.gui.beans. WekaWrapper 
 Set the algorithm.
   setWriteOPTICSresults(boolean)   - 
Method in class weka.clusterers. OPTICS 
 Sets the flag for writing actions
   setX(double)   - 
Method in class weka.classifiers.functions.neural. NeuralConnection 
  
   setX(int)   - 
Method in class weka.gui.beans. BeanInstance 
 Sets the x coordinate of this bean
   setX(int)   - 
Method in class weka.gui.visualize. AttributePanel 
 shows which bar is the current x attribute.
   setXAttribute(int)   - 
Method in class weka.gui.boundaryvisualizer. BoundaryPanel 
 Set the x attribute index
   setXAttribute(int)   - 
Method in class weka.gui.boundaryvisualizer. RemoteBoundaryVisualizerSubTask 
 Set the x axis fixed dimension
   setXBase(double)   - 
Method in class weka.classifiers.meta. GridSearch 
 Set the value of the base for X.
   setXExpression(String)   - 
Method in class weka.classifiers.meta. GridSearch 
 Set the expression for the X value.
   setXindex(int)   - 
Method in class weka.gui.visualize. Plot2D 
 Set the index of the attribute to go on the x axis
   setXindex(int)   - 
Method in class weka.gui.visualize. PlotData2D 
 Set the x index of the data.
   setXIndex(int)   - 
Method in class weka.gui.visualize. VisualizePanel 
 Set the index of the attribute for the x axis
   setXLabelFreq(int)   - 
Method in class weka.gui.beans. StripChart 
 Set the frequency for printing x label values
   setXMax(double)   - 
Method in class weka.classifiers.meta. GridSearch 
 Set the value of the Maximum of X.
   setXMin(double)   - 
Method in class weka.classifiers.meta. GridSearch 
 Set the value of the minimum of X.
   setXML(Reader)   - 
Method in class weka.core.xml. XMLInstances 
 reads the XML structure from the given reader
   setXORMode(Color)   - 
Method in class weka.gui.visualize. PostscriptGraphics 
 Not implemented
   setXProperty(String)   - 
Method in class weka.classifiers.meta. GridSearch 
 Set the X property.
   setXStep(double)   - 
Method in class weka.classifiers.meta. GridSearch 
 Set the value of the step size for X.
   setXval(boolean)   - 
Method in class weka.attributeSelection. AttributeSelection 
 do a cross validation
   setXY(int, int)   - 
Method in class weka.gui.beans. BeanInstance 
 Set the x and y coordinates of this bean
   setY(double)   - 
Method in class weka.classifiers.functions.neural. NeuralConnection 
  
   setY(int)   - 
Method in class weka.gui.beans. BeanInstance 
 Sets the y coordinate of this bean
   setY(int)   - 
Method in class weka.gui.visualize. AttributePanel 
 shows which bar is the current y attribute.
   setYAttribute(int)   - 
Method in class weka.gui.boundaryvisualizer. BoundaryPanel 
 Set the y attribute index
   setYAttribute(int)   - 
Method in class weka.gui.boundaryvisualizer. RemoteBoundaryVisualizerSubTask 
 Set the y axis fixed dimension
   setYBase(double)   - 
Method in class weka.classifiers.meta. GridSearch 
 Set the value of the base for Y.
   setYExpression(String)   - 
Method in class weka.classifiers.meta. GridSearch 
 Set the expression for the Y value.
   setYindex(int)   - 
Method in class weka.gui.visualize. Plot2D 
 Set the index of the attribute to go on the y axis
   setYindex(int)   - 
Method in class weka.gui.visualize. PlotData2D 
 Set the y index of the data
   setYIndex(int)   - 
Method in class weka.gui.visualize. VisualizePanel 
 Set the index of the attribute for the y axis
   setYMax(double)   - 
Method in class weka.classifiers.meta. GridSearch 
 Set the value of the Maximum of Y.
   setYMin(double)   - 
Method in class weka.classifiers.meta. GridSearch 
 Set the value of the minimum of Y.
   setYProperty(String)   - 
Method in class weka.classifiers.meta. GridSearch 
 Set the Y property (normally the classifier).
   setYStep(double)   - 
Method in class weka.classifiers.meta. GridSearch 
 Set the value of the step size for Y.
   SEVERE   - 
Static variable in class weka.core. Debug 
 the log level Severe
   SFEntropyGain()   - 
Method in class weka.classifiers. Evaluation 
 Returns the total SF, which is the null model entropy minus
 the scheme entropy.
   SFMeanEntropyGain()   - 
Method in class weka.classifiers. Evaluation 
 Returns the SF per instance, which is the null model entropy
 minus the scheme entropy, per instance.
   SFMeanPriorEntropy()   - 
Method in class weka.classifiers. Evaluation 
 Returns the entropy per instance for the null model
   SFMeanSchemeEntropy()   - 
Method in class weka.classifiers. Evaluation 
 Returns the entropy per instance for the scheme
   SFPriorEntropy()   - 
Method in class weka.classifiers. Evaluation 
 Returns the total entropy for the null model
   SFSchemeEntropy()   - 
Method in class weka.classifiers. Evaluation 
 Returns the total entropy for the scheme
   sgn(double)   - 
Static method in class weka.classifiers.bayes. BayesianLogisticRegression 
 Sign for a given value.
   shear(double, double)   - 
Method in class weka.gui.visualize. PostscriptGraphics 
  
   shift(int, int, Instance)   - 
Method in class weka.classifiers.trees.j48. Distribution 
 Shifts given instance from one bag to another one.
   shift(int, int)   - 
Method in class weka.core.matrix. IntVector 
 Shifts an element to another position.
   shiftBeans(BeanInstance, boolean)   - 
Method in class weka.gui.beans. MetaBean 
 Move coords of all inputs and outputs of this meta bean
 to the coords of the supplied BeanInstance.
   shiftRange(int, int, Instances, int, int)   - 
Method in class weka.classifiers.trees.j48. Distribution 
 Shifts all instances in given range from one bag to another one.
   shiftToEnd(int)   - 
Method in class weka.core.matrix. IntVector 
 Shifts an element to the end of the vector.
   SHORT   - 
Static variable in class weka.experiment. DatabaseUtils 
 Type mapping for SHORT used for reading experiment results.
   show(Component, int, int)   - 
Method in class weka.gui. GenericObjectEditor.JTreePopupMenu 
 Displays the menu, making sure it will fit on the screen.
   showAttributes()   - 
Method in class weka.gui.arffviewer. ArffViewerMainPanel 
 displays all the attributes, returns the selected item or NULL if canceled
   showChart()   - 
Method in class weka.gui.beans. StripChart 
 Popup the chart panel
   showDialog(Component, String)   - 
Method in class weka.gui. ConverterFileChooser 
 Pops a custom file chooser dialog with a custom approve button.
   showDialog()   - 
Method in class weka.gui.experiment. OutputFormatDialog 
 Pops up the modal dialog and waits for cancel or a selection.
   showDialog()   - 
Method in class weka.gui. ListSelectorDialog 
 Pops up the modal dialog and waits for cancel or a selection.
   showDialog()   - 
Method in class weka.gui. PropertySelectorDialog 
 Pops up the modal dialog and waits for cancel or a selection.
   showDialog()   - 
Method in class weka.gui. ViewerDialog 
 Pops up the modal dialog and waits for Cancel or OK.
   showDialog(Instances)   - 
Method in class weka.gui. ViewerDialog 
 Pops up the modal dialog and waits for Cancel or OK.
   showExplorer(String)   - 
Method in class weka.gui. GUIChooser 
  
   showGUITipText()   - 
Method in class weka.clusterers. OPTICS 
 Returns the tip text for this property.
   showHistory()   - 
Method in class weka.gui.sql. ConnectionPanel 
 displays the query history.
   showHistory()   - 
Method in class weka.gui.sql. QueryPanel 
 displays the query history.
   showInputBox(Component, String, String, Object)   - 
Static method in class weka.gui. ComponentHelper 
 pops up an input dialog
   showKnowledgeFlow(String)   - 
Method in class weka.gui. GUIChooser 
  
   showMessageBox(Component, String, String, int, int)   - 
Static method in class weka.gui. ComponentHelper 
 displays a message box with the given title, message, buttons and icon
 ant the dimension.
   showOpenDialog(Component)   - 
Method in class weka.gui. ConverterFileChooser 
 Pops up an "Open File" file chooser dialog.
   showOutOfMemory()   - 
Method in class weka.core. Memory 
 prints an error message if OutOfMemory (and if GUI is present a dialog),
 otherwise nothing happens.
   showPopup()   - 
Method in class weka.gui. GenericObjectEditor.CapabilitiesFilterDialog 
 if a JPopupMenu is set, it is displayed again.
   showProperties()   - 
Method in class weka.gui.arffviewer. ArffViewerMainPanel 
 displays some properties of the instances
   showPropertyDialog()   - 
Method in class weka.gui. PropertyPanel 
 Displays the property edit dialog for the panel.
   showResults()   - 
Method in class weka.gui.beans. GraphViewer 
 Popup a result list from which the user can select a graph to view
   showResults()   - 
Method in class weka.gui.beans. TextViewer 
 Popup a component to display the selected text
   showSaveDialog(Component)   - 
Method in class weka.gui. ConverterFileChooser 
 Pops up an "Save File" file chooser dialog.
   showTree()   - 
Method in class weka.gui. HierarchyPropertyParser 
 Show the whole tree in text format
   showValues()   - 
Method in class weka.gui.arffviewer. ArffViewerMainPanel 
 displays all the distinct values for an attribute
   showWindow(Container)   - 
Method in class weka.gui. Main 
 brings child frame to the top.
   showWindow(Class)   - 
Method in class weka.gui. Main 
 brings the first frame to the top that is of the specified
 window class.
   shrinkageTipText()   - 
Method in class weka.classifiers.meta. AdditiveRegression 
 Returns the tip text for this property
   shrinkageTipText()   - 
Method in class weka.classifiers.meta. LogitBoost 
 Returns the tip text for this property
   shrinkingTipText()   - 
Method in class weka.classifiers.functions. LibSVM 
 Returns the tip text for this property
   shuffleTipText()   - 
Method in class weka.classifiers.rules. Ridor 
 Returns the tip text for this property
   sIB   - Class in  weka.clusterers  Cluster data using the sequential information bottleneck algorithm. 
  
 Note: only hard clustering scheme is supported.   sIB()   - 
Constructor for class weka.clusterers. sIB 
  
   sigLevel   - 
Variable in class weka.experiment. PairedStats 
 The significance level for comparisons
   sigmaTipText()   - 
Method in class weka.attributeSelection. ReliefFAttributeEval 
 Returns the tip text for this property
   sigmaTipText()   - 
Method in class weka.classifiers.functions.supportVector. Puk 
 Returns the tip text for this property
   SigmoidUnit   - Class in  weka.classifiers.functions.neural  This can be used by the 
 neuralnode to perform all it's computations (as a sigmoid unit).   SigmoidUnit()   - 
Constructor for class weka.classifiers.functions.neural. SigmoidUnit 
  
   sign()   - 
Method in class weka.core.matrix. DoubleVector 
 Returns the signs of all elements in terms of -1, 0 and +1.
   SIGNIFICANCE_LOSS   - 
Static variable in class weka.experiment. ResultMatrix 
 loss
   SIGNIFICANCE_TIE   - 
Static variable in class weka.experiment. ResultMatrix 
 tie
   SIGNIFICANCE_WIN   - 
Static variable in class weka.experiment. ResultMatrix 
 win
   significanceLevelTipText()   - 
Method in class weka.associations. Apriori 
 Returns the tip text for this property
   significanceLevelTipText()   - 
Method in class weka.attributeSelection. RaceSearch 
 Returns the tip text for this property
   SIGNIFICANT   - 
Static variable in class weka.associations. Tertius 
 Way of handling missing values: missing as a particular value
   simetricDif(ScatterSearchV1.Subset, ScatterSearchV1.Subset, int)   - 
Method in class weka.attributeSelection. ScatterSearchV1 
  
   SimetricDiference(ScatterSearchV1.Subset, BitSet)   - 
Method in class weka.attributeSelection. ScatterSearchV1 
 Calculate the Simetric Diference of two subsets
   SimpleBatchFilter   - Class in  weka.filters  This filter is a superclass for simple batch filters.   SimpleBatchFilter()   - 
Constructor for class weka.filters. SimpleBatchFilter 
  
   SimpleCart   - Class in  weka.classifiers.trees  Class implementing minimal cost-complexity pruning. 
 Note when dealing with missing values, use "fractional instances" method instead of surrogate split method. 
  
 For more information, see: 
  
 Leo Breiman, Jerome H.   SimpleCart()   - 
Constructor for class weka.classifiers.trees. SimpleCart 
  
   SimpleCLI   - Class in  weka.gui  Creates a very simple command line for invoking the main method of
 classes.   SimpleCLI()   - 
Constructor for class weka.gui. SimpleCLI 
 Constructor
   SimpleCLIPanel   - Class in  weka.gui  Creates a very simple command line for invoking the main method of
 classes.   SimpleCLIPanel()   - 
Constructor for class weka.gui. SimpleCLIPanel 
 Constructor.
   SimpleCLIPanel.CommandlineCompletion   - Class in  weka.gui  A class for commandline completion of classnames.   SimpleCLIPanel.CommandlineCompletion()   - 
Constructor for class weka.gui. SimpleCLIPanel.CommandlineCompletion 
 default constructor.
   SimpleDateFormatEditor   - Class in  weka.gui  Class for editing SimpleDateFormat strings.   SimpleDateFormatEditor()   - 
Constructor for class weka.gui. SimpleDateFormatEditor 
 Constructs a new SimpleDateFormatEditor.
   SimpleEstimator   - Class in  weka.classifiers.bayes.net.estimate  SimpleEstimator is used for estimating the conditional probability tables of a Bayes network once the structure has been learned.   SimpleEstimator()   - 
Constructor for class weka.classifiers.bayes.net.estimate. SimpleEstimator 
  
   SimpleFilter   - Class in  weka.filters  This filter contains common behavior of the SimpleBatchFilter and the
 SimpleStreamFilter.   SimpleFilter()   - 
Constructor for class weka.filters. SimpleFilter 
  
   SimpleKMeans   - Class in  weka.clusterers  Cluster data using the k means algorithm
  
  

  
 Valid options are:     SimpleKMeans()   - 
Constructor for class weka.clusterers. SimpleKMeans 
 the default constructor
   SimpleLinearRegression   - Class in  weka.classifiers.functions  Learns a simple linear regression model.   SimpleLinearRegression()   - 
Constructor for class weka.classifiers.functions. SimpleLinearRegression 
  
   SimpleLinkedList   - Class in  weka.associations.tertius      SimpleLinkedList()   - 
Constructor for class weka.associations.tertius. SimpleLinkedList 
  
   SimpleLinkedList.LinkedListInverseIterator   - Class in  weka.associations.tertius      SimpleLinkedList.LinkedListInverseIterator()   - 
Constructor for class weka.associations.tertius. SimpleLinkedList.LinkedListInverseIterator 
  
   SimpleLinkedList.LinkedListIterator   - Class in  weka.associations.tertius      SimpleLinkedList.LinkedListIterator()   - 
Constructor for class weka.associations.tertius. SimpleLinkedList.LinkedListIterator 
  
   SimpleLogistic   - Class in  weka.classifiers.functions  Classifier for building linear logistic regression models.   SimpleLogistic()   - 
Constructor for class weka.classifiers.functions. SimpleLogistic 
 Constructor for creating SimpleLogistic object with standard options.
   SimpleLogistic(int, boolean, boolean)   - 
Constructor for class weka.classifiers.functions. SimpleLogistic 
 Constructor for creating SimpleLogistic object.
   SimpleMI   - Class in  weka.classifiers.mi  Reduces MI data into mono-instance data.   SimpleMI()   - 
Constructor for class weka.classifiers.mi. SimpleMI 
  
   SimpleSetupPanel   - Class in  weka.gui.experiment  This panel controls the configuration of an experiment.   SimpleSetupPanel(Experiment)   - 
Constructor for class weka.gui.experiment. SimpleSetupPanel 
 Creates the setup panel with the supplied initial experiment.
   SimpleSetupPanel()   - 
Constructor for class weka.gui.experiment. SimpleSetupPanel 
 Creates the setup panel with no initial experiment.
   SimpleStreamFilter   - Class in  weka.filters  This filter is a superclass for simple stream filters.   SimpleStreamFilter()   - 
Constructor for class weka.filters. SimpleStreamFilter 
  
   SimulatedAnnealing   - Class in  weka.classifiers.bayes.net.search.global  This Bayes Network learning algorithm uses the general purpose search method of simulated annealing to find a well scoring network structure. 
  
 For more information see: 
  
 R.R.   SimulatedAnnealing()   - 
Constructor for class weka.classifiers.bayes.net.search.global. SimulatedAnnealing 
  
   SimulatedAnnealing   - Class in  weka.classifiers.bayes.net.search.local  This Bayes Network learning algorithm uses the general purpose search method of simulated annealing to find a well scoring network structure. 
  
 For more information see: 
  
 R.R.   SimulatedAnnealing()   - 
Constructor for class weka.classifiers.bayes.net.search.local. SimulatedAnnealing 
  
   SIN   - 
Static variable in interface weka.core.mathematicalexpression. sym 
  
   SIN   - 
Static variable in interface weka.filters.unsupervised.instance.subsetbyexpression. sym 
  
   SINE   - 
Static variable in class weka.datagenerators.clusterers. BIRCHCluster 
 Constant set for choice of pattern.
   SingleAssociatorEnhancer   - Class in  weka.associations  Abstract utility class for handling settings common to meta
 associators that use a single base associator.   SingleAssociatorEnhancer()   - 
Constructor for class weka.associations. SingleAssociatorEnhancer 
  
   SingleClassifierEnhancer   - Class in  weka.classifiers  Abstract utility class for handling settings common to meta
 classifiers that use a single base learner.   SingleClassifierEnhancer()   - 
Constructor for class weka.classifiers. SingleClassifierEnhancer 
  
   SingleClustererEnhancer   - Class in  weka.clusterers  Meta-clusterer for enhancing a base clusterer.   SingleClustererEnhancer()   - 
Constructor for class weka.clusterers. SingleClustererEnhancer 
  
   singleConsequence(Instances)   - 
Static method in class weka.associations. CaRuleGeneration 
 generates a consequence of length 1 for a class association rule.
   singleConsequence(Instances, int, FastVector)   - 
Static method in class weka.associations. RuleGeneration 
 generates a consequence of length 1 for an association rule.
   SingleIndex   - Class in  weka.core  Class representing a single cardinal number.   SingleIndex()   - 
Constructor for class weka.core. SingleIndex 
 Default constructor.
   SingleIndex(String)   - 
Constructor for class weka.core. SingleIndex 
 Constructor to set initial index.
   singletons(Instances)   - 
Static method in class weka.associations. AprioriItemSet 
 Converts the header info of the given set of instances into a set 
 of item sets (singletons).
   singletons(Instances)   - 
Static method in class weka.associations. CaRuleGeneration 
 Converts the header info of the given set of instances into a set 
 of item sets (singletons).
   singletons(Instances)   - 
Static method in class weka.associations. ItemSet 
 Converts the header info of the given set of instances into a set 
 of item sets (singletons).
   singletons(Instances, Instances)   - 
Static method in class weka.associations. LabeledItemSet 
 Converts the header info of the given set of instances into a set
 of item sets (singletons).
   SINGULAR_DUMMY   - 
Static variable in interface weka.gui.graphvisualizer. GraphConstants 
 SINGULAR_DUMMY node - node with only one outgoing edge
 i.e.
   SingularValueDecomposition   - Class in  weka.core.matrix  Singular Value Decomposition.   SingularValueDecomposition(Matrix)   - 
Constructor for class weka.core.matrix. SingularValueDecomposition 
 Construct the singular value decomposition
   size()   - 
Method in class weka.associations.tertius. SimpleLinkedList 
  
   size()   - 
Method in class weka.classifiers. CostMatrix 
 The number of rows (and columns)
   size()   - 
Method in class weka.classifiers.evaluation. ConfusionMatrix 
 Gets the number of classes.
   size()   - 
Method in class weka.classifiers.functions.pace. DiscreteFunction 
 Returns the size of the point set.
   size()   - 
Method in class weka.classifiers.lazy.kstar. KStarCache.CacheTable 
 Returns the number of keys in this hashtable.
   size()   - 
Method in class weka.classifiers.rules. Rule 
 The size of the rule.
   size()   - 
Method in interface weka.clusterers.forOPTICSAndDBScan.Databases. Database 
 Returns the size of the database (the number of dataObjects in the database)
   size()   - 
Method in class weka.clusterers.forOPTICSAndDBScan.Databases. SequentialDatabase 
 Returns the size of the database (the number of dataObjects in the database)
   size()   - 
Method in class weka.clusterers.forOPTICSAndDBScan.Utils. PriorityQueue 
 Returns the queue's size
   size()   - 
Method in class weka.clusterers.forOPTICSAndDBScan.Utils. UpdateQueue 
 Returns the queue's size
   size()   - 
Method in class weka.core. FastVector 
 Returns the vector's current size.
   size()   - 
Method in class weka.core.matrix. DoubleVector 
 Gets the size of the vector.
   size()   - 
Method in class weka.core.matrix. IntVector 
 Gets the size of the vector.
   size()   - 
Method in class weka.core. PropertyPath.Path 
 returns the number of path elements of this structure
   size()   - 
Method in class weka.core. Queue 
 Gets queue's size.
   size()   - 
Method in class weka.core. Tee 
 returns the number of streams currently in the list.
   size()   - 
Method in class weka.core. Trie 
 Returns the number of elements in this collection.
   size()   - 
Method in class weka.core. Trie.TrieNode 
 returns the number of stored strings, i.e., leaves
   size()   - 
Method in class weka.core.xml. MethodHandler 
 returns the number of currently stored Methods
   sizePerTipText()   - 
Method in class weka.classifiers.trees. BFTree 
 Returns the tip text for this property
   sizePerTipText()   - 
Method in class weka.classifiers.trees. SimpleCart 
 Returns the tip text for this property
   skipIdenticalTipText()   - 
Method in class weka.core.neighboursearch. LinearNNSearch 
 Returns the tip text for this property.
   SlidingMidPointOfWidestSide   - Class in  weka.core.neighboursearch.kdtrees  The class that splits a node into two based on the midpoint value of the dimension in which the node's rectangle is widest.   SlidingMidPointOfWidestSide()   - 
Constructor for class weka.core.neighboursearch.kdtrees. SlidingMidPointOfWidestSide 
  
   sm(double, double)   - 
Static method in class weka.core. Utils 
 Tests if a is smaller than b.
   SMALL   - 
Static variable in class weka.core. Utils 
 The small deviation allowed in double comparisons.
   SMO   - Class in  weka.classifiers.functions  Implements John Platt's sequential minimal optimization algorithm for training a support vector classifier. 
  
 This implementation globally replaces all missing values and transforms nominal attributes into binary ones.   SMO()   - 
Constructor for class weka.classifiers.functions. SMO 
  
   SMO.BinarySMO   - Class in  weka.classifiers.functions  Class for building a binary support vector machine.   SMO.BinarySMO()   - 
Constructor for class weka.classifiers.functions. SMO.BinarySMO 
  
   smoothingParameterTipText()   - 
Method in class weka.classifiers.bayes. ComplementNaiveBayes 
 Returns the tip text for this property
   SMOreg   - Class in  weka.classifiers.functions  SMOreg implements the support vector machine for regression.   SMOreg()   - 
Constructor for class weka.classifiers.functions. SMOreg 
  
   smOrEq(double, double)   - 
Static method in class weka.core. Utils
[truncated: 234,936 more chars]
